# Evaluating the Effectiveness of Large Language Models in Providing Patient Education for Chinese Patients With Ocular Myasthenia Gravis: Mixed Methods Study

**DOI:** 10.2196/67883

**Published:** 2025-04-10

**Authors:** Bin Wei, Lili Yao, Xin Hu, Yuxiang Hu, Jie Rao, Yu Ji, Zhuoer Dong, Yichong Duan, Xiaorong Wu

**Affiliations:** 1 Jiangxi Medical College The First Affiliated Hospital Nanchang University Nanchang China

**Keywords:** LLM, large language models, ocular myasthenia gravis, patient education, China, effectiveness, deep learning, artificial intelligence, health care, accuracy, applicability, neuromuscular disorder, extraocular muscles, ptosis, diplopia, ophthalmology, ChatGPT, clinical practice, digital health

## Abstract

**Background:**

Ocular myasthenia gravis (OMG) is a neuromuscular disorder primarily affecting the extraocular muscles, leading to ptosis and diplopia. Effective patient education is crucial for disease management; however, in China, limited health care resources often restrict patients’ access to personalized medical guidance. Large language models (LLMs) have emerged as potential tools to bridge this gap by providing instant, AI-driven health information. However, their accuracy and readability in educating patients with OMG remain uncertain.

**Objective:**

The purpose of this study was to systematically evaluate the effectiveness of multiple LLMs in the education of Chinese patients with OMG. Specifically, the validity of these models in answering patients with OMG-related questions was assessed through accuracy, completeness, readability, usefulness, and safety, and patients’ ratings of their usability and readability were analyzed.

**Methods:**

The study was conducted in two phases: 130 choice ophthalmology examination questions were input into 5 different LLMs. Their performance was compared with that of undergraduates, master’s students, and ophthalmology residents. In addition, 23 common patients with OMG-related patient questions were posed to 4 LLMs, and their responses were evaluated by ophthalmologists across 5 domains. In the second phase, 20 patients with OMG interacted with the 2 LLMs from the first phase, each asking 3 questions. Patients assessed the responses for satisfaction and readability, while ophthalmologists evaluated the responses again using the 5 domains.

**Results:**

ChatGPT o1-preview achieved the highest accuracy rate of 73% on 130 ophthalmology examination questions, outperforming other LLMs and professional groups like undergraduates and master’s students. For 23 common patients with OMG-related questions, ChatGPT o1-preview scored highest in correctness (4.44), completeness (4.44), helpfulness (4.47), and safety (4.6). GEMINI (Google DeepMind) provided the easiest-to-understand responses in readability assessments, while GPT-4o had the most complex responses, suitable for readers with higher education levels. In the second phase with 20 patients with OMG, ChatGPT o1-preview received higher satisfaction scores than Ernie 3.5 (Baidu; 4.40 vs 3.89, *P*=.002), although Ernie 3.5’s responses were slightly more readable (4.31 vs 4.03, *P*=.01).

**Conclusions:**

LLMs such as ChatGPT o1-preview may have the potential to enhance patient education. Addressing challenges such as misinformation risk, readability issues, and ethical considerations is crucial for their effective and safe integration into clinical practice.

## Introduction

A large language model (LLM) is a deep learning–based artificial intelligence model specifically designed to process and generate natural language. With massive parameters and complex architectures, LLMs are trained on vast amounts of textual data, enabling them to perform a wide range of natural language processing (NLP) tasks [1,2]. In November 2022, OpenAI introduced the first LLM, ChatGPT, marking a significant advancement in the NLP domain [3]. The latest model, OpenAI o1-Preview, released in September 2024, surpasses its predecessor, ChatGPT (GPT-4o), in handling complex logical tasks, heralding a new era of artificial intelligence (AI)–driven reasoning [4]. As various LLMs emerge, the applications of deep learning and machine learning in fields such as medicine and ophthalmology are rapidly expanding, offering promising opportunities in patient education and health care [5-7].

Ocular myasthenia gravis (OMG) is one of the most common neuromuscular junction disorders, primarily affecting the extraocular muscles and causing symptoms such as ptosis and diplopia. If not adequately controlled, patients with OMG can progress to generalized myasthenia gravis (GMG), impacting respiratory muscles and limb function, and posing significant health risks [8,9]. Effective management of patients with OMG relies on accurate diagnosis, patient education, and continuous medical support. However, in China, due to the large patient population and limited medical resources, personalized education and support present significant challenges, making the internet a crucial source for patients seeking medical information [10,11]. LLMs like ChatGPT offer patients a quick and convenient way to access medical information, demonstrating potential value, particularly in ophthalmology, where AI can assist in providing preliminary information about common eye diseases and supporting health care professionals [12]. However, LLMs are not specifically designed for medical use, as their training data come from a wide range of internet sources rather than specialized medical datasets, which may lead to inaccurate or misleading responses [13-15]. Given the severity of patients with OMG, ensuring that patients receive reliable and accurate medical information is crucial. Therefore, a comprehensive evaluation of chatbots’ reliability and accuracy in addressing medical inquiries is essential to ensure their effective application in managing diseases like OMG [16].

Recent studies have explored the application of LLMs in ophthalmology. Jaskari et al [17] introduced a model named DR-GPT, designed to analyze fundus images, demonstrating that LLMs can be applied to unstructured medical report databases to aid in classifying diabetic retinopathy. Prashant D’s team conducted a randomized, blinded, multicenter study that showed LLMs performed comparably to experts in terms of quality, empathy, and safety metrics, highlighting their potential for use in clinical environments [18]. However, another study revealed that LLMs achieved only a 45% accuracy rate in identifying information for retinal disease patients, indicating significant gaps in their clinical application in ophthalmology [19]. To date, no studies have assessed LLM performance in educating Chinese patients about patients with OMG.

Although several studies have explored the application of LLMs in consultation processes and diagnostic capabilities within Chinese ophthalmology subspecialties, research remains limited. For instance, Ming et al [20] compared GPT-3.5 and GPT-4.0 (OpenAI) in recommending ophthalmology subspecialty registrations, finding that both models demonstrated moderate performance. GPT-4.0 performed comparably to, and even numerically outperformed, residents in differential diagnosis, suggesting the potential of chatbots in facilitating ophthalmic patient triage [20]. Similarly, Liu et al [21] analyzed 316 surgical cases, comparing GPT-3.5 and GPT-4.0, and found that GPT-4.0 exhibited higher diagnostic accuracy. However, further real-world clinical studies are needed to improve LLMs’ ability to identify patient symptoms and interpret laboratory data [21].

Unlike previous research, which primarily focused on English-language medical Q and A, this study is the first to assess the educational effectiveness of LLMs for Chinese patients with OMG. Moreover, while most previous studies evaluated AI-generated responses from an expert perspective, our study uniquely integrates real patient interactions with chatbots, enabling a more practical and patient-centered assessment of LLM effectiveness. By comparing multiple LLMs within a real-world Chinese ophthalmology setting, our findings provide new insights into the strengths and limitations of AI-driven patient education.

## Methods

### Study Design

**Figure 1:**
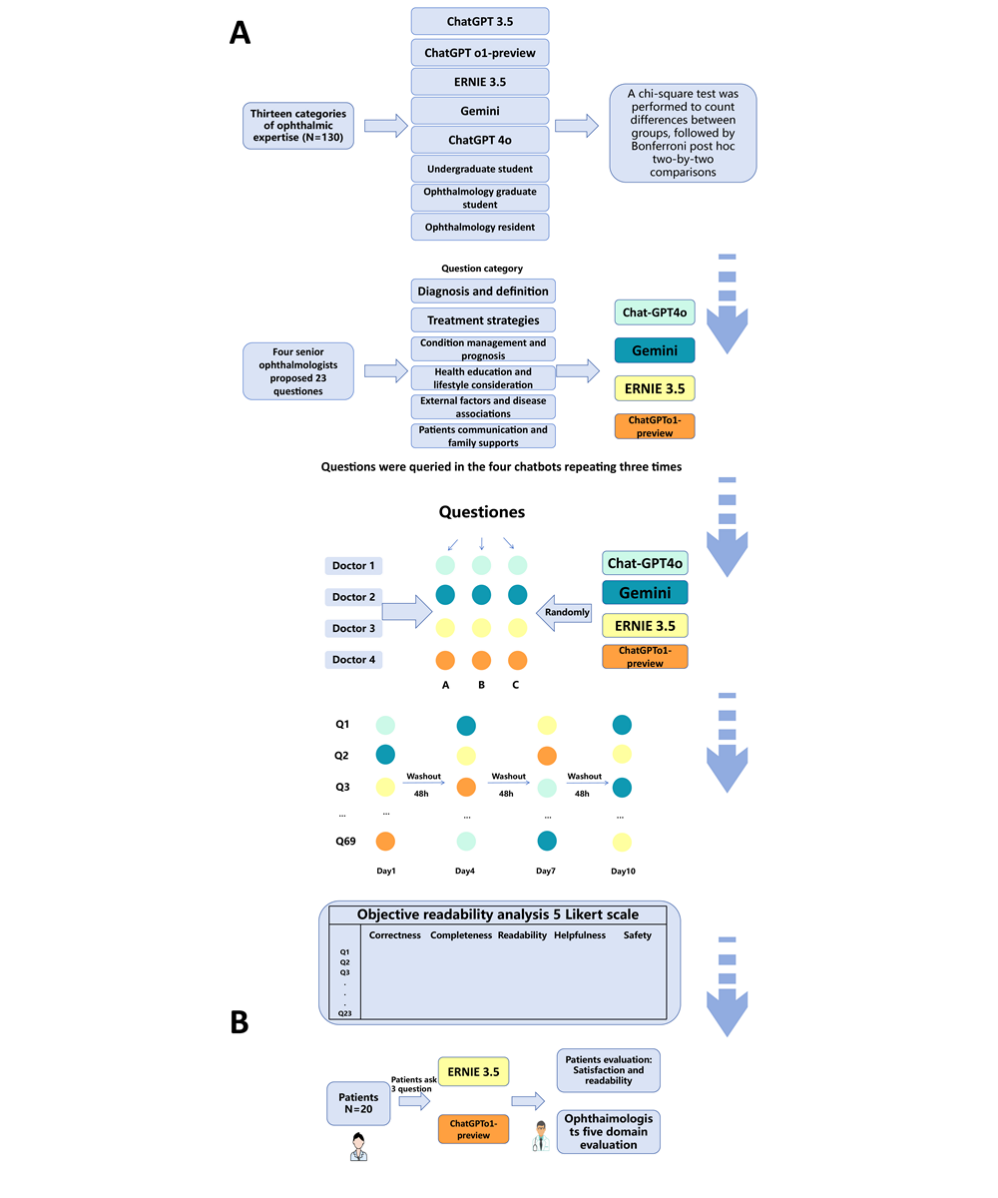
Flowchart of the overall study design. (A) Two tests of the large language model in the first phase and (B) the second phase is real scenario testing.

This study consists of 2 phases: the first phase aims to perform an initial screening and performance evaluation of 5 LLMs (Table 1). The second phase was not influenced by the results of the first phase and was used for the validation of the real-world study to ensure the independence and comprehensiveness of the results (Figure 1).

In the first part of the first phase, 130 choice questions were randomly selected from the Chinese ophthalmology attending physician examination question bank. These questions were input into 5 different LLMs for testing, with each test starting from a reset initialization prompt. The results of the LLMs were then compared with the performance of three groups of professionals: (1) undergraduate students, (2) ophthalmology graduate students, and (3) ophthalmology residents. The participants included 3 randomly selected clinical medicine undergraduates from Nanchang University, 3 ophthalmology master’s students, and 3 attending ophthalmologists from the First Affiliated Hospital of Nanchang University. All participants took a 2-hour closed-book examination.

In the second part of the first phase, 4 senior ophthalmologists provided common patient questions regarding OMG (Multimedia Appendix 1). A total of 23 patients with OMG-related questions were entered into the online interfaces of 4 different LLMs (Multimedia Appendices 2-5), with each question repeated 3 times to observe potential variations in the responses. The questions covered topics such as disease diagnosis and definition, treatment strategies, condition management and prognosis, health education and lifestyle advice, external factors associated with the disease, as well as patient communication and family support. All responses were generated using independent prompts, with the following prompt provided: “I would like you to assume the role of an ophthalmologist and respond to a patient’s inquiry about a specific ophthalmic disease.”

In this study, all generated responses were converted into plain text format to effectively conceal the unique characteristics of each chatbot’s answers. A total number of 4 senior ophthalmologists conducted a blind review of the 276 questions generated by the 4 LLMs. The evaluation process was divided into 4 rounds, each separated by a 48-hour interval to minimize potential effects. In addition, a Chinese readability platform (CRP) was used to objectively assess the reading difficulty of the chatbot-generated responses.

In the second phase, 20 representative patients from the ophthalmology and neurology departments were recruited through convenience sampling to participate in a specialized patient education session. Before the patients interacted with the chatbots, a general prompt was input to establish context: “Please assist the ophthalmologist in providing patient education on ocular myasthenia gravis.” Each patient then asked 3 patients with OMG-related questions to 2 different chatbots and evaluated the responses in terms of satisfaction and readability. Meanwhile, 2 ophthalmologists assessed the chatbot responses across 5 domains (Multimedia Appendix 6).

**Table 1:** Large language models (LLMs) used in this study.

LLMs	Version	Company
GPT-4	4o	OpenAI
GPT-3.5	3.5	OpenAI
GPT-4	o1-preview	OpenAI
Ernie 3.5	3.5	Baidu
Gemini	3.5	Google

### Study Population

The inclusion criteria for ophthalmologists were (1) serving as chief ophthalmologists with at least 10 years of clinical experience, (2) native Mandarin speakers, and (3) with expertise in assessing the quality of patient education materials and health consultation texts.

The inclusion criteria for patients were (1) aged between 18 and 50 years, (2) native Mandarin speakers, and (3) diagnosed patients with OMG within the past year.

Exclusion criteria were (1) patients unable to comply with the study procedures, (2) a history of cognitive impairment, and (3) a history of severe vision impairment.

### Ophthalmic Question Bank

In the first part of the first phase, the choice questions were sourced from the Chinese Ophthalmology Attending Physician Exam Question Bank (Yikaobang), divided into 13 subcategories. A total number of 10 choice questions were randomly selected from each subcategory. For the second part of the first phase, the patients with OMG question bank was compiled and refined by 4 experienced chief ophthalmologists, based on their clinical experience. The questions were standardized and optimized to ensure consistency and clarity in language, with 23 finalized questions used for testing.

### Evaluation of 5 Domains

In the first phase, 4 ophthalmologists, and in the second phase, 2 ophthalmologists, evaluated the LLMs’ responses based on five domains: (1) correctness, (2) completeness, (3) readability, (4) helpfulness, and (5) safety. In the second phase, patients assessed the LLMs’ responses by rating their satisfaction and comprehensibility. All evaluations were conducted using continuous rating scales.

### Objective Readability Analysis

In the first phase of this study, all responses were assessed by medical professionals. Therefore, the Common Questions section was presented in English. In the second phase, when questions were directed at patients, all queries, including multiple-choice questions from a designated question bank, were presented in Chinese and translated using Google’s built-in web page translation tool. Previous research has shown that these chatbots can provide accurate responses in English; however, their answers are often complex, requiring readers to have a higher level of education. To account for differences between Chinese and English, as well as subjective factors such as patients’ educational backgrounds and physicians’ experience, we used a CRP. This platform uses a multiple linear regression model to assess the correlation between 52 linguistic factors and text difficulty. It generates a reading difficulty score, the corresponding educational level, and the recommended reading age. The score is directly proportional to the complexity of understanding the text.

### Statistical Analysis

IBM SPSS (version 29.0.1.0) and Prism10 (GraphPad Software, Inc) were used for statistical analysis and plotting. The accuracy rates of 130 choice questions across 8 groups were compared using the chi-square test, with Bonferroni correction applied to adjust *P* values for detecting intergroup differences. For the 23 common questions, the Friedman test was used to compare each chatbot’s evaluation scores and readability scores, followed by post-hoc pairwise comparisons using the Dunnett test. A two-sample t test was used to compare the mean response scores of GPT o1-Preview and Ernie 3.5. A *P* value of ≤.05 was considered statistically significant.

### Ethical Considerations

This study adheres to ethical guidelines and has been reviewed and approved by the Ethics Committee of The First Affiliated Hospital of Nanchang University (2023CDYFYYLK01-014). Informed consent was obtained from all participants prior to data collection, explicitly permitting secondary analysis. To ensure privacy and confidentiality, all data were anonymized before analysis and do not contain any personally identifiable information. No images or data in this manuscript include identifiable personal details. If any identifiable data were required, explicit consent would be obtained and appropriately documented.

This study was conducted from August 1 to October 1, 2024, at the Departments of Ophthalmology and Neurology, the First Affiliated Hospital of Nanchang University, China. The study was approved by the institutional review board and adhered to the principles of the Declaration of Helsinki. Written informed consent was obtained from all patients.

All data generated or analyzed during this study from patients are included in this published article. All Patients and guardians were provided consent to publish these pictures.

## Results

### Study for Test in the First Phase

**Figure 2:**
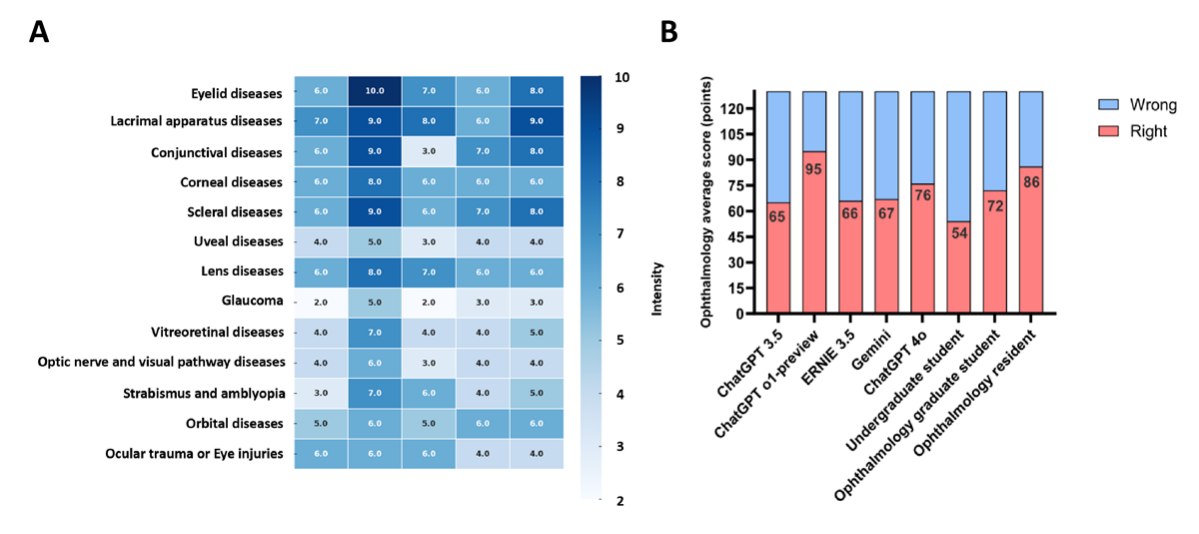
A test with 130 questions for 3 groups of people and 5 large language models.

**Figure 3:**
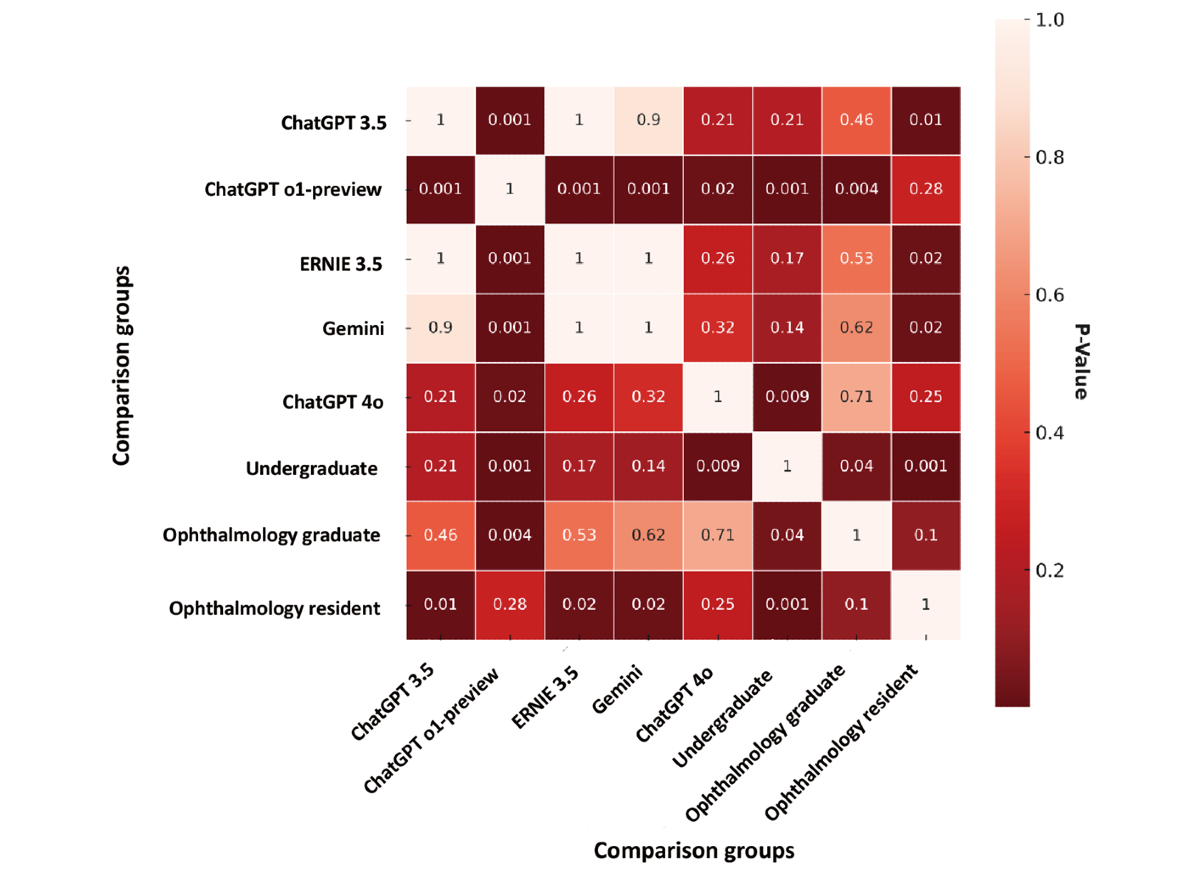
The heatmap presents a *P* value matrix comparing different groups for statistical significance analysis.

Regarding the first phase of the study, as shown in Figure 1, Figure 2 and Table 2 display the number of correct answers in each subcategory and the overall accuracy rates. Among the 130 questions, the accuracy rates for ChatGPT 3.5, ChatGPT o1-preview, Ernie 3.5, Gemini, ChatGPT 4o, undergraduate student, ophthalmology graduate student, and ophthalmology resident were 65/130 (50%), 95/130 (73%), 66/130 (51%), 67/130 (52%), 76/130 (59%), 54/130 (42%), 72/130 (55%), and 86/130 (66%), respectively. The overall results indicated statistically significant differences (*P*<.001). Figure 3 shows statistically significant differences between specific groups (*P*<.05).

Figure 2 shows a heat map of the number of correct answers for 130 questions across 13 categories by 5 LLMs and histograms of the number of questions answered correctly for 5 LLMs and 3 populations.

The color intensity represents the magnitude of the *P* value, with darker shades indicating smaller *P* values and thus more significant differences between groups. The horizontal and vertical axes list the comparison groups, including ChatGPT 3.5, ChatGPT o1-preview, ERNIE 3.5, Gemini, ChatGPT 4o, undergraduate students, ophthalmology graduate students, and ophthalmology residents. A color bar on the right side indicates the *P* value range.

**Table 2:** Number of correct answers and comparison among the 8 groups (*P*<.001).

Variable	Number of correct answers							
	ChatGPT 3.5	ChatGPT o1-preview	ERNIE 3.5	Gemini	ChatGPT 4o	Undergraduate student	Ophthalmology graduate student	Ophthalmology resident
Eyelid diseases (n=10), n	6	10	7	6	8	6	6	8
Lacrimal apparatus diseases (n=10), n	7	9	8	6	9	4	6	7
Conjunctival diseases (n=10), n	6	9	3	7	8	7	6	7
Corneal diseases (n=10), n	6	8	6	6	6	6	5	5
Scleral diseases (n=10), n	6	9	6	7	8	2	4	6
Uveal diseases (n=10), n	4	5	3	4	4	1	7	8
Lens diseases (n=10), n	6	8	7	6	6	6	7	7
Glaucoma (n=10), n	2	5	2	3	3	5	5	8
Vitreoretinal diseases (n=10), n	4	7	4	4	5	4	5	5
Optic nerve and visual pathway diseases (n=10), n	4	6	3	4	4	2	4	5
Strabismus and amblyopia (n=10), n	3	7	6	4	5	3	5	7
Orbital diseases (n=10), n	5	6	5	6	6	3	6	5
Ocular trauma or eye injuries (n=10), n	6	6	6	4	4	5	6	8
Total (N=130), n (%)	65 (50)	95 (73)	66 (51)	67 (52)	76 (59)	54 (42)	72 (55)	86 (66)

**Figure 4:**
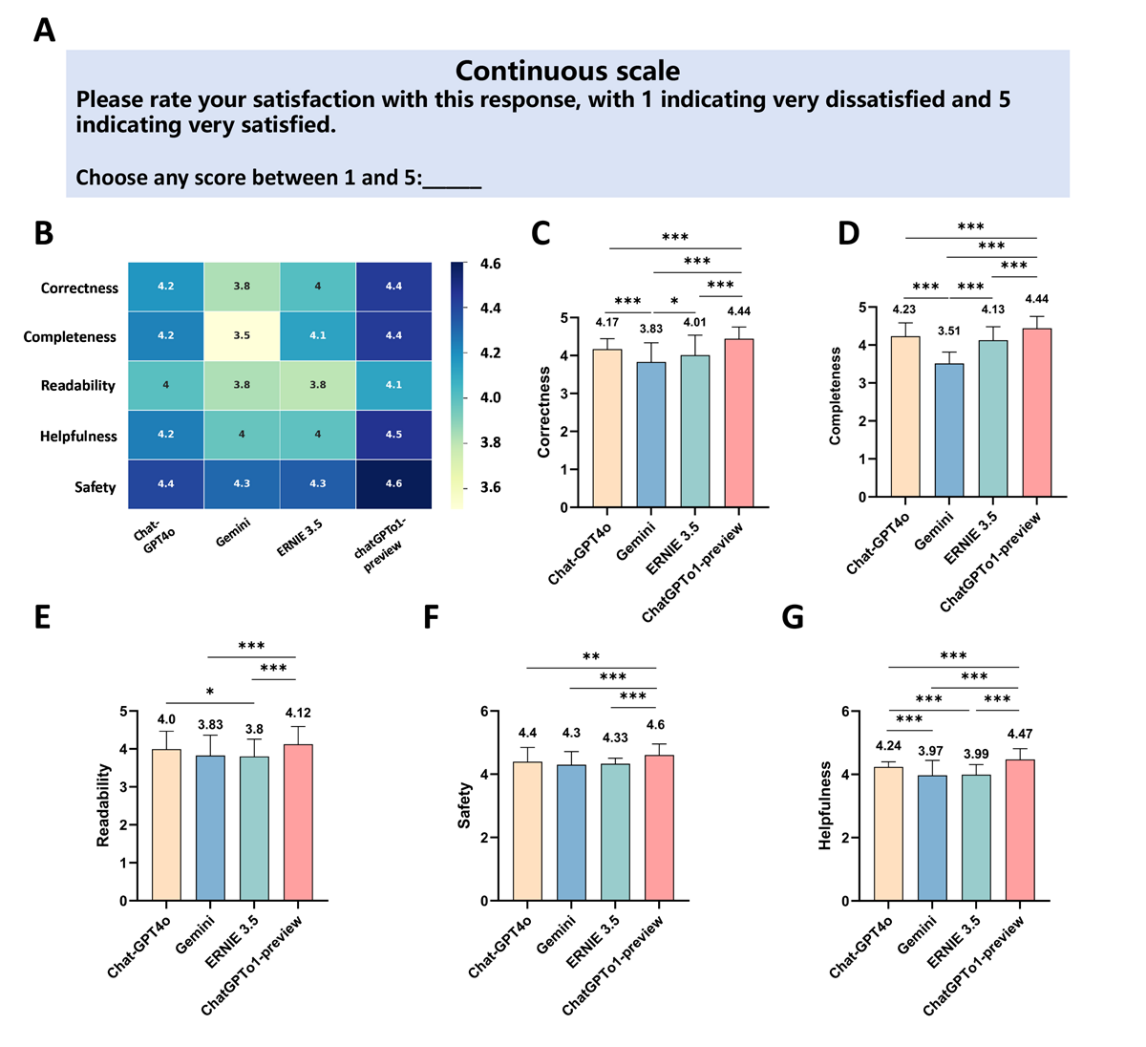
Evaluate the responses of 4 large language models using a continuous scale.

Figure 4 shows the average scores of the 4 LLM chatbots in 5 domains when answering patients with OMG-related questions. Among them, ChatGPT o1-preview performed the best, with the following scores: correctness (4.44), completeness (4.44), readability (4.12), helpfulness (4.47), and safety (4.6), as confirmed by the post hoc Dunnett test (*P*<.001). Multimedia Appendices 2-5 provide the detailed scores for each LLM across the 5 domains for every question. Figure 4A shows a continuous scale for evaluation. Figure 4B shows a heat map of scores for 4 LLMs across 5 domains. Figures 4C-G show average scores for the 5 domains (correctness, completeness, helpfulness, and safety) of the 4 LLM chatbot responses in the first phase of the study. The Friedman test and post hoc Dunnett tests were used to assess the statistical significance of the differences observed. Data are expressed as mean (SD) values.

**Figure 5:**
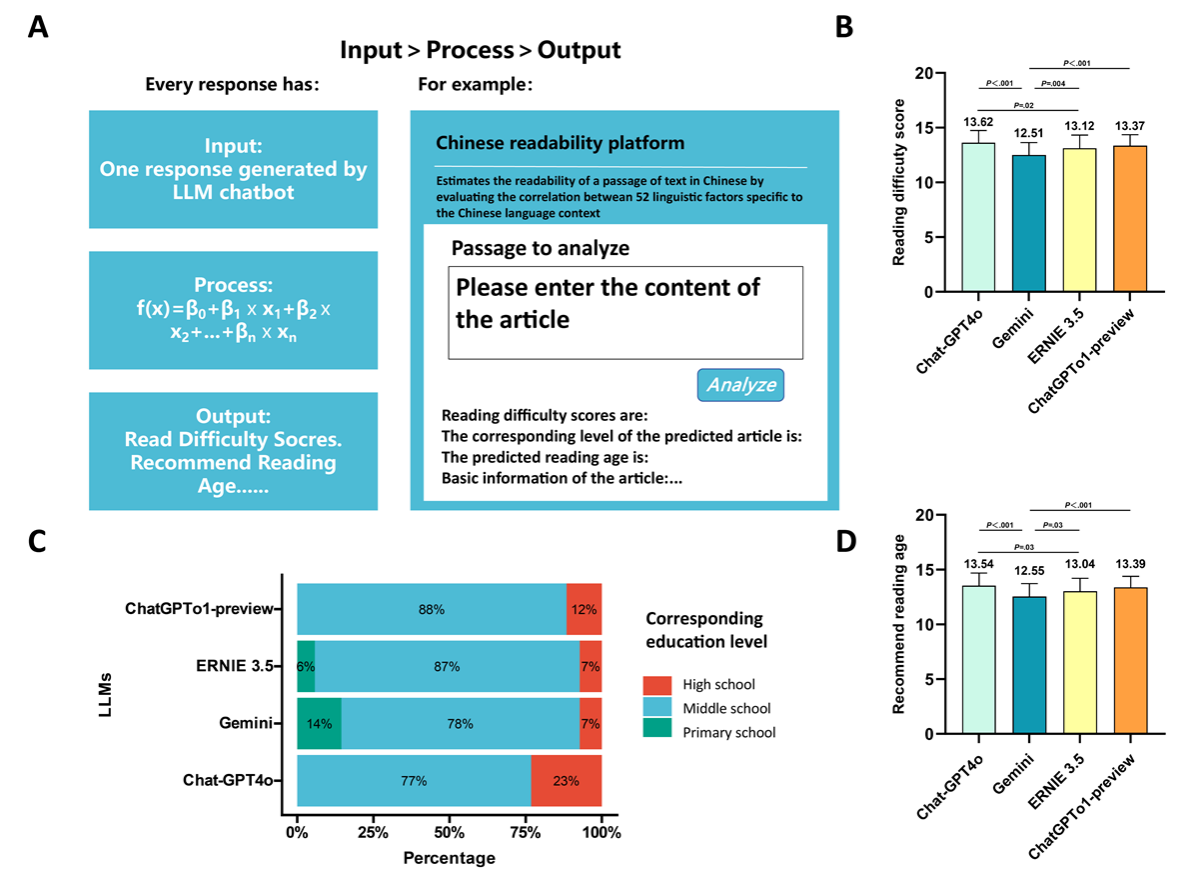
An objective readability analysis of responses from four large language models.

In the objective reading difficulty assessment, the responses of GPT-4o had the highest reading difficulty score (13.62, post hoc Dunnett test, *P*<.001), with a recommended reading age of 13.54 years, and 23% of its responses were deemed suitable for a high school education level (Figures 5B-D). In contrast, GEMINI received the lowest scores, and its responses were the easiest to understand. Multimedia Appendices 2-5 provides the objective scores for each of the LLMs.

Figure 5A shows a Chinese readability analysis platform. Figure 5B shows reading difficulty scores, representing the comprehension difficulty level of responses generated by 4 chatbots. Figure 5C shows the education level corresponding to the responses of 4 LLMs. Figure 5D shows the recommended reading age, indicating the appropriate age group for the text responses of 4 LLMs. The Friedman test and post hoc Dunnett tests were used to assess the statistical significance of the differences observed. Data are expressed as mean (SD) values.

### Real-World Study for Validation in the Second Phase

**Figure 6:**
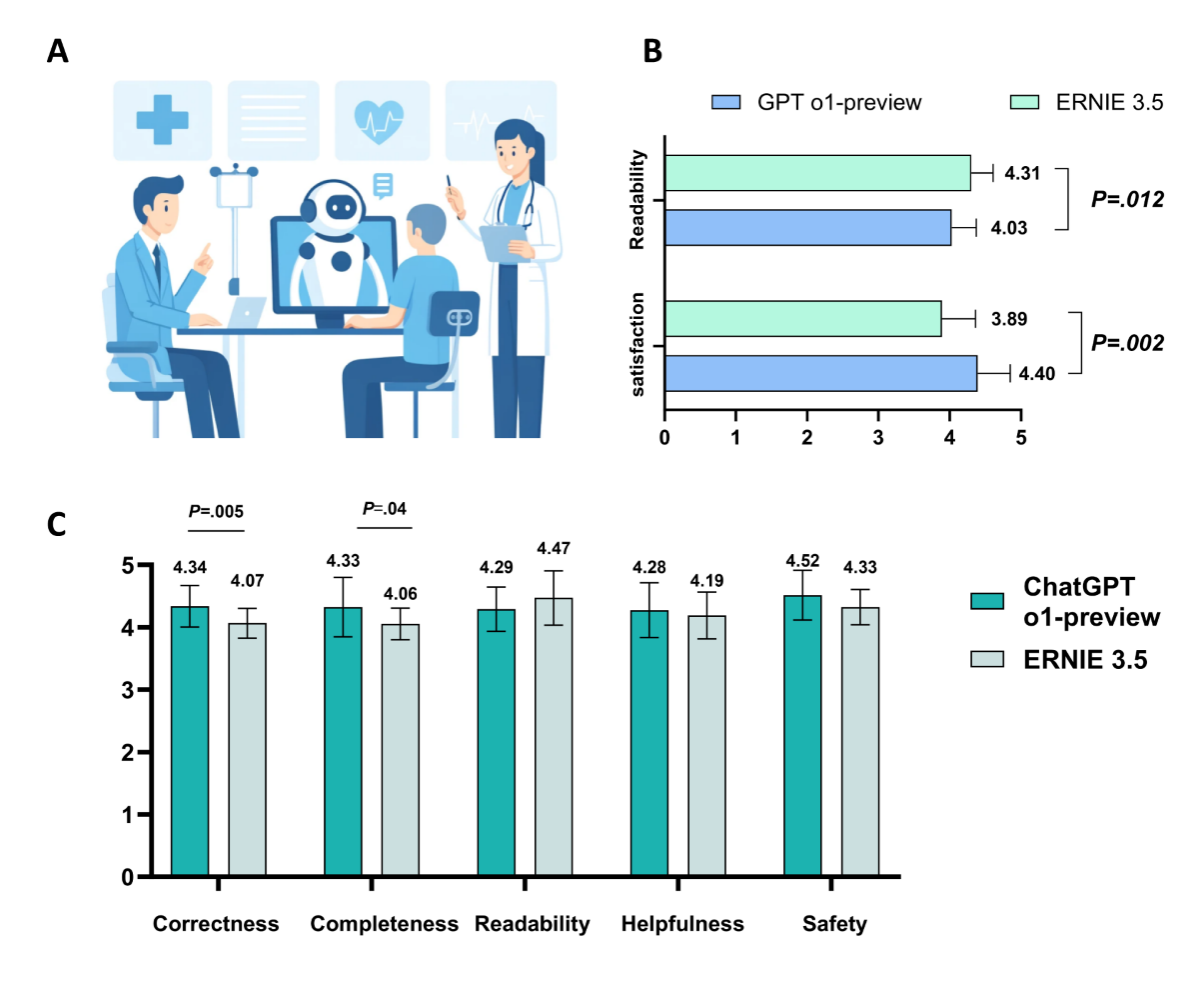
Evaluation of GPT o1-preview and Ernie 3.5 addressing patients with OMG queries in real-world assessment.

In the second phase, 20 patients with OMG asked questions to ChatGPT o1-preview and Ernie 3.5, the 2 LLMs selected for their top performance in the first phase and ease of access. Patient satisfaction and readability scores showed significant differences between the 2 groups (satisfaction: 4.40 vs 3.89, *P*=.002; readability: 4.03 vs 4.31, *P*=.01; Figure 6B). The evaluations by 2 ophthalmologists of ChatGPT o1-preview and Ernie 3.5 across 5 domains were as follows: correctness=4.34 vs 4.07 (*P*=.005), completeness=4.33 vs 4.06 (*P*=.04), readability=4.29 vs 4.47), helpfulness=4.28 vs 4.19), and safety=4.52 vs 4.33) (Figure 6C).

Figure 6A shows the human-chatbot interface. Figure 6B shows patient satisfaction with the responses of the 2 LLM chatbots and readability of the responses of the 2 chatbots in the real-world evaluation. Figure 6C shows average scores for the 5 domains (correctness, completeness, readability, helpfulness, and safety) of the responses of GPT o1-preview and Ernie 3.5 in the real-world evaluation. A 2-tailed *t* test was used to assess the statistical significance of the differences observed. Data are expressed as mean (SD) values.

## Discussion

### Principal Findings

The study evaluated the effectiveness of several LLMs in providing patient education on patients with OMG. The results showed that ChatGPT o1-preview achieved an accuracy rate of 73% on ophthalmology examination questions, surpassing undergraduates and ophthalmology master’s students, and approaching the performance of ophthalmology residents. Among the models tested, ChatGPT o1-preview excelled in answering patient questions (correctness, completeness, readability, helpfulness, and safety), indicating that LLMs have significant potential to enhance patient education, particularly in resource-limited settings, for medical education and consultation tasks.

This study is the first to assess the application of LLMs in educating patients with OMG through real patient chatbot interactions, addressing a critical gap in the current literature. The strong performance of ChatGPT o1-preview can be attributed to its advanced architecture and extensive training data, enabling it to generate more accurate and contextually appropriate responses. This aligns with previous studies that highlighted the capabilities of LLMs in medical education and consultation tasks [22-24]. Despite the encouraging results, our study also underscores several challenges and limitations in using LLMs for patient education. One of the primary concerns is the risk of misinformation due to the “hallucination” phenomenon, where LLMs may produce responses that sound plausible but are factually incorrect or nonsensical [25,26]. For example, although ChatGPT o1-preview generally performed well, in some cases, the information provided was either not entirely accurate or too complex for patients with lower levels of education. Objective readability analysis revealed that GPT-4o’s responses had higher reading difficulty scores, which may hinder comprehension for patients with lower educational backgrounds.

Ethical and legal considerations are also critical when integrating LLMs into clinical practice. Accountability becomes an issue if patients make decisions based on incorrect information provided by AI models. Currently, there is a lack of regulatory frameworks governing the use of AI in health care, making it unclear who would be held responsible in such cases [27]. Moreover, patient privacy and data security are paramount, as interactions with chatbots may involve sharing sensitive personal health information [28,29]. Ensuring that LLMs comply with data protection regulations is essential for maintaining patient trust and confidentiality.

To effectively integrate LLMs into clinical practice, addressing these challenges is essential. One approach is to develop LLMs specifically tailored for medical use and train them on specialized datasets to improve accuracy and reliability [30,31]. Involving health care professionals in the oversight process can further reduce the risk of misinformation, as clinicians can review and verify the information provided by AI. In addition, customizing language and content based on patients’ cultural and educational backgrounds can enhance comprehension and engagement [32]. Future research should focus on expanding sample sizes, including more diverse patient populations, as our current study used a convenience sampling approach, which may limit the generalizability of the findings, and exploring multimodal AI systems capable of handling various types of clinical data.

### Limitations

First, LLMs were not trained specifically for medical use, which could affect response accuracy in complex clinical cases. Second, the sample size was small, limiting generalizability. Third, evaluations focused on satisfaction, readability, and correctness but did not address cultural sensitivity or long-term educational impact. Finally, the controlled study setting may not reflect real-world challenges like internet access and language barriers. Future research should involve larger samples, use specialized medical datasets, and assess the long-term effectiveness of LLM-driven patient education.

### Conclusions

This study provides preliminary findings suggesting that LLMs, especially ChatGPT o1-preview, may be effective in providing patient education on OMG among Chinese patients, outperforming other models and even some professionals. While LLMs have significant potential to enhance patient understanding, challenges like misinformation risks, readability issues for less-educated patients, and ethical concerns about accountability and privacy need careful attention. Addressing these challenges can enable LLMs to become valuable tools in improving patient education and health care outcomes for patients with OMG.

## Appendix

Multimedia Appendix 1 Questions (n=23) frequently asked by patients concerning ocular myasthenia gravis.

Multimedia Appendix 2 Chat-GPT4o answers to 23 questions, raw scores in 5 areas and raw data for objective readability analysis.

Multimedia Appendix 3 Gemini answers to 23 questions, raw scores in 5 areas and raw data for objective readability analysis.

Multimedia Appendix 4 ERNIE 3.5 answers to 23 questions, raw scores in 5 areas and raw data for objective readability analysis.

Multimedia Appendix 5 ChatGPTo1-preview answers to 23 questions, raw scores in 5 areas and raw data for objective readability analysis.

Multimedia Appendix 6 Detailed information of the real-world study.

## Abbreviations

AI: artificial intelligence
CRP: Chinese readability platform
GMG: generalized myasthenia gravis
LLM: large language model
NLP: natural language processing
OMG: ocular myasthenia gravis

## Data Availability

The datasets generated during and/or analyzed during this study are available from the corresponding author on reasonable request.
