# Supplementary material for: Evaluating the Effectiveness of Large Language Models in Providing Patient Education for Chinese Patients With Ocular Myasthenia Gravis: Mixed Methods Study
Source: J Med Internet Res. 2025 Apr 10;27:e67883. doi: 10.2196/67883 (PMC12022522; doi:10.2196/67883)
Supplement: Multimedia Appendix 1 [file jmir_v27i1e67883_app1.docx]

**Table S1: 23 questions frequently asked by patients concerning Ocular Myasthenia Gravis**

| **Question** | **category** |
| --- | --- |
| 1.What is ocular myasthenia gravis (OMG)? | Diagnosis and Definition |
| 2.What are the typical symptoms of ocular myasthenia gravis? |  |
| 3.What is the difference between ocular myasthenia gravis and generalized myasthenia gravis? |  |
| 4.How is ocular myasthenia gravis diagnosed? |  |
| 5.Do ocular myasthenia gravis patients need long-term medication? | Treatment Strategies |
| 6.What medications are commonly used to treat ocular myasthenia gravis? What are their side effects? |  |
| 7.Can ocular myasthenia gravis be treated with surgery? |  |
| 8.Can patients with ocular myasthenia gravis undergo laser eye surgery (such as LASIK)? |  |
| 9.Can dietary changes help alleviate symptoms of ocular myasthenia gravis? |  |
| 10.Will ocular myasthenia gravis eventually progress to generalized myasthenia gravis? | Condition Management and Prognosis |
| 11.Does ocular myasthenia gravis cause permanent vision loss? |  |
| 12.Does the severity of ocular myasthenia gravis increase with age? |  |
| 13.How should physical activities or exercise be planned for patients after their symptoms have stabilized? |  |
| 14.Will prolonged screen time worsen the symptoms of ocular myasthenia gravis? |  |
| 15.Does ocular myasthenia gravis affect a patient’s sleep quality? |  |
| 16.What should patients with ocular myasthenia gravis pay attention to in daily life? | Health Education and Lifestyle Considerations |
| 17.Can ocular myasthenia gravis patients drive or operate machinery safely? |  |
| 18.What daily rehabilitation exercises can help improve eye symptoms in ocular myasthenia gravis patients? |  |
| 19.Is ocular myasthenia gravis associated with other autoimmune diseases? | External Factors and Disease Associations |
| 20.Can weather changes or emotional stress affect the symptoms of ocular myasthenia gravis? |  |
| 21.What should ocular myasthenia gravis patients be aware of when receiving vaccinations? |  |
| 22.How should patients with ocular myasthenia gravis communicate with their doctor about changes in their condition? | Patient Communication and Family Support |
| 23.How can family members provide psychological support and practical help to a patient with ocular myasthenia gravis? |  |
