# Supplementary material for: Evaluating the Effectiveness of Large Language Models in Providing Patient Education for Chinese Patients With Ocular Myasthenia Gravis: Mixed Methods Study
Source: J Med Internet Res. 2025 Apr 10;27:e67883. doi: 10.2196/67883 (PMC12022522; doi:10.2196/67883)
Supplement: Multimedia Appendix 2 [file jmir_v27i1e67883_app2.docx]

| **Table S2: Chat-GPT4o answers to 23 questions, raw scores in 5 areas and raw data for objective readability analysis** | | | |
| --- | --- | --- | --- |
| **Question1** | **What is ocular myasthenia gravis (OMG)?** | | |
|  | Ocular myasthenia gravis (OMG) is a subtype of myasthenia gravis (MG), an autoimmune neuromuscular disorder characterized by weakness and fatigue of the voluntary muscles. In OMG, the disease predominantly affects the muscles that control eye movements, eyelid elevation, and sometimes the muscles responsible for eyelid closure.  Key features of OMG include:   1. Ptosis (drooping of one or both eyelids): One of the most common symptoms, ptosis can be intermittent and may worsen with prolonged activity or at the end of the day. 2. Diplopia (double vision): This occurs due to weakness of the extraocular muscles, leading to misalignment of the eyes. 3. Fluctuating muscle weakness: The symptoms of OMG tend to fluctuate, often worsening with fatigue or stress and improving after rest.   Unlike generalized myasthenia gravis (gMG), which affects other muscle groups in addition to the eyes, OMG is typically confined to ocular muscles. However, about 50% of patients with OMG can progress to generalized MG within two years.  Diagnosis usually involves clinical examination, blood tests to detect antibodies such as acetylcholine receptor (AChR) antibodies or muscle-specific kinase (MuSK) antibodies, electrophysiological tests (e.g., repetitive nerve stimulation), and sometimes imaging to rule out other causes. Treatment may include medications such as cholinesterase inhibitors, immunosuppressive therapies, or, in some cases, corticosteroids. | Ocular myasthenia gravis (OMG) is a localized form of myasthenia gravis (MG), a chronic autoimmune disorder that affects neuromuscular transmission. In OMG, the immune system produces antibodies (often against the acetylcholine receptors or other proteins like muscle-specific kinase) that interfere with communication between nerves and muscles, leading to muscle weakness. Unlike generalized MG, which can affect muscles throughout the body, OMG is limited to the muscles that control eye movements and the eyelids.  Key features of ocular myasthenia gravis include:  Ptosis: Drooping of one or both eyelids, which can vary throughout the day.  Diplopia: Double vision due to weakness in the extraocular muscles, leading to difficulty aligning the eyes properly.  Fluctuating symptoms: Muscle weakness in OMG tends to worsen with activity and improve with rest.  Progression risk: About 50% of patients with OMG may develop generalized myasthenia gravis within two years of symptom onset.  Diagnosis often involves clinical evaluation, blood tests to detect specific antibodies, and electrodiagnostic tests such as repetitive nerve stimulation or single-fiber electromyography. Treatment typically includes medications like acetylcholinesterase inhibitors (e.g., pyridostigmine) and immunosuppressants for more severe cases. | Ocular myasthenia gravis (OMG) is a localized form of myasthenia gravis, an autoimmune disorder that primarily affects the muscles controlling eye and eyelid movement. It occurs when the body produces antibodies that attack the acetylcholine receptors or other proteins involved in neuromuscular transmission at the neuromuscular junction. This disruption impairs communication between nerves and muscles, leading to muscle weakness.  Key characteristics of OMG include:  1. Ptosis: Drooping of one or both eyelids, which may worsen throughout the day.  2. Diplopia: Double vision due to weakness in the muscles that control eye movement.  3.Fatigue: Symptoms often worsen with activity and improve after rest.  In OMG, the muscle weakness is typically restricted to the eyes and eyelids, but about 50% of patients may develop generalized myasthenia gravis (gMG) over time, which affects other muscles in the body, including those involved in speaking, swallowing, and breathing.  Diagnosis is usually confirmed through tests like anti-acetylcholine receptor antibody testing, single-fiber electromyography, or a Tensilon (edrophonium) test. Treatment may include medications such as acetylcholinesterase inhibitors (e.g., pyridostigmine), immunosuppressants, or corticosteroids. |
| 问题1 | **什么是眼肌型重症肌无力？** | | |
|  | 眼部重症肌无力 (OMG) 是重症肌无力 (MG) 的一个亚型，重症肌无力是一种自身免疫性神经肌肉疾病，其特征是随意肌无力和疲劳。在 OMG 中，该疾病主要影响控制眼球运动、眼睑抬高的肌肉，有时还影响负责眼睑闭合的肌肉。  OMG 的主要功能包括：  眼睑下垂（一侧或两侧眼睑下垂）：眼睑下垂是最常见的症状之一，可能是间歇性的，并且可能因长时间活动或一天结束时而恶化。  复视（双重视觉）：这是由于眼外肌无力导致眼睛错位而发生的。  波动性肌无力：OMG 的症状往往波动，通常因疲劳或压力而恶化，休息后会改善。  全身性重症肌无力 (gMG) 除了影响眼睛之外，还会影响其他肌肉群，而 OMG 通常只局限于眼部肌肉。不过，约 50% 的 OMG 患者可在两年内发展为全身性重症肌无力。  诊断通常包括临床检查、血液检测（以检测乙酰胆碱受体 (AChR) 抗体或肌肉特异性激酶 (MuSK) 抗体等抗体）、电生理检查（例如重复神经刺激），有时还需进行影像学检查以排除其他原因。治疗可能包括药物治疗（例如胆碱酯酶抑制剂）、免疫抑制疗法，或在某些情况下使用皮质类固醇。 | 眼部重症肌无力 (OMG) 是重症肌无力 (MG) 的一种局部形式，重症肌无力是一种影响神经肌肉传递的慢性自身免疫性疾病。在眼部重症肌无力中，免疫系统会产生抗体（通常针对乙酰胆碱受体或其他蛋白质，如肌肉特异性激酶），干扰神经和肌肉之间的交流，导致肌肉无力。与会影响全身肌肉的全身性重症肌无力不同，眼部重症肌无力仅限于控制眼球运动和眼睑的肌肉。  眼部重症肌无力的主要特征包括：  眼睑下垂：一只或两只眼睑下垂，一天之内症状可能有所不同。  复视：由于眼外肌无力而导致的复视，导致眼睛难以正确对准。  症状波动：OMG 的肌肉无力往往随着活动而恶化，随着休息而改善。  进展风险：约 50% 的 OMG 患者在症状出现后两年内可能发展为全身性重症肌无力。  诊断通常涉及临床评估、血液检测以检测特定抗体，以及电诊断测试，例如重复神经刺激或单纤维肌电图。治疗通常包括乙酰胆碱酯酶抑制剂（例如吡啶斯的明）等药物，以及针对更严重病例的免疫抑制剂。 | 眼部重症肌无力 (OMG) 是重症肌无力的一种局部形式，重症肌无力是一种自身免疫性疾病，主要影响控制眼球和眼睑运动的肌肉。当身体产生抗体攻击乙酰胆碱受体或神经肌肉接头处参与神经肌肉传递的其他蛋白质时，就会发生这种情况。这种干扰会损害神经和肌肉之间的沟通，导致肌肉无力。  OMG 的主要特点包括：  眼睑下垂：一只或两只眼睑下垂，并且可能在一天内逐渐恶化。  复视：由于控制眼球运动的肌肉无力而导致的复视。  疲劳：症状通常因活动而恶化，休息后有所改善。  在 OMG 中，肌肉无力通常仅限于眼睛和眼睑，但大约 50% 的患者可能会随着时间的推移而发展为全身性重症肌无力 (gMG)，这会影响身体的其他肌肉，包括参与说话、吞咽和呼吸的肌肉。  通常通过抗乙酰胆碱受体抗体检测、单纤维肌电图或腾喜龙（依酚氯铵）检测等测试来确认诊断。治疗可能包括使用乙酰胆碱酯酶抑制剂（例如吡斯的明）、免疫抑制剂或皮质类固醇等药物。 |
| Correctness | 3.9 | 4.4 | 3.9 |
| Completeness | 4.2 | 4.2 | 3.6 |
| Readability | 4 | 4.6 | 4.6 |
| Helpfulness | 4.1 | 4.5 | 4 |
| Safety | 5 | 4.6 | 5 |
| Reading Difficuty Score | 12.33 | 12.41 | 12.78 |
| Corresponding Education Level | Middle School | Middle School | Middle School |
| Recommend Reading Age | 12 | 12 | 13 |
| **Question2** | **What are the typical symptoms of ocular myasthenia gravis?** | | |
|  | The typical symptoms of ocular myasthenia gravis (OMG) primarily affect the muscles that control the eyes and eyelids. These symptoms can fluctuate in severity and often worsen with fatigue or as the day progresses. The key symptoms include:  1. Ptosis (Drooping Eyelids) : One or both eyelids droop, and this drooping can become more pronounced after prolonged use of the eyes, such as reading or driving.  2. Diplopia (Double Vision) : Weakness in the eye muscles can lead to misalignment of the eyes, causing double vision. This may occur in different directions of gaze or may change throughout the day.  3. Fluctuating Symptoms : The weakness tends to fluctuate, worsening with activity and improving after rest.  4. Eye Fatigue : Eyes may tire quickly, particularly during activities that require sustained visual focus, such as reading or using a computer.  These symptoms are typically the earliest and most prominent features of OMG, but in some cases, the condition may progress to generalized myasthenia gravis, involving other muscles of the body. | The typical symptoms of ocular myasthenia gravis (OMG) primarily affect the muscles controlling the eyes and eyelids, and they can vary in severity over time, often worsening with activity and improving with rest. Common symptoms include:  1. Ptosis (drooping of one or both eyelids): This is often one of the first symptoms of OMG and can become more pronounced as the day progresses or after prolonged use of the eyes.    2. Diplopia (double vision): Due to weakness in the extraocular muscles, patients may experience misalignment of the eyes, leading to double vision. The severity of the diplopia can fluctuate throughout the day.  3. Eye fatigue : Prolonged use of the eyes, such as reading or driving, can lead to increased fatigue and worsening of ptosis and diplopia.  4. Difficulty focusing : Some patients may find it hard to focus their vision, especially during tasks requiring sustained visual attention.  5. Blurry vision : Although less common, some patients report intermittent blurred vision, which may be linked to the muscle weakness.  These symptoms tend to fluctuate in intensity and are often worse at the end of the day or after exertion. Importantly, in ocular myasthenia gravis, muscle weakness is limited to the eyes and eyelids, unlike generalized myasthenia gravis, which affects other muscles in the body. However, about half of OMG patients may progress to the generalized form of the disease. | The typical symptoms of ocular myasthenia gravis (OMG) primarily involve the muscles that control eye movement and eyelid function. These symptoms often fluctuate in severity and tend to worsen with fatigue or prolonged use of the affected muscles. Common symptoms include:  1. Ptosis (Drooping Eyelid) : One or both eyelids may droop, and the drooping often becomes more pronounced as the day progresses or with prolonged visual tasks.    2. Diplopia (Double Vision) : Due to weakness in the extraocular muscles, patients may experience double vision that worsens with prolonged visual focus or when looking in specific directions.  3. Ocular Muscle Fatigue : Patients may report that their eye movements feel "weak" or that their vision becomes blurry with extended use, especially later in the day.  4. Difficulty in Eye Movement : Some individuals may find it hard to move their eyes in certain directions, leading to restricted eye movement.  These symptoms are typically asymmetrical, meaning one eye may be more affected than the other. Importantly, unlike generalized myasthenia gravis, OMG does not initially affect muscles outside the eyes and eyelids, though some patients may later develop generalized symptoms. |
| 问题2 | **眼肌型重症肌无力的典型症状是什么？** | | |
|  | 眼部重症肌无力(OMG)的典型症状主要影响控制眼睛和眼睑的肌肉。这些症状的严重程度可以波动，并经常随着疲劳或一天的进展而恶化。主要症状包括:  1. 上睑下垂(眼睑下垂):单侧或双侧眼睑下垂，在长时间使用眼睛后，如阅读或开车，这种下垂会变得更加明显。  2. 复视:眼部肌肉无力会导致眼睛错位，导致复视。这可能发生在凝视的不同方向，也可能在一天中发生变化。  3. 波动症状:虚弱倾向于波动，活动时加重，休息后好转。  4. 眼睛疲劳:眼睛可能会很快疲劳，特别是在需要持续视觉聚焦的活动中，比如阅读或使用电脑。  这些症状通常是OMG最早和最突出的特征，但在某些情况下，病情可能发展为全身重症肌无力，累及身体的其他肌肉。 | 眼部重症肌无力(OMG)的典型症状主要影响控制眼睛和眼睑的肌肉，其严重程度随时间变化而变化，通常随着活动而恶化，随着休息而改善。常见症状包括:  1. 上睑下垂(单眼皮或双眼皮下垂):这通常是OMG的最初症状之一，随着时间的推移或长时间使用眼睛后会变得更加明显。  2. 复视:由于眼外肌无力，患者可能会出现眼睛错位，导致复视。复视的严重程度可以在一天中波动。  3. 眼睛疲劳:长时间使用眼睛，如阅读或驾驶，可导致疲劳增加和恶化的上睑下垂和复视。  4. 难以集中注意力:一些患者可能会发现很难集中他们的视力，特别是在需要持续视觉注意力的任务中。  5. 视力模糊:虽然不太常见，但一些患者报告间歇性视力模糊，这可能与肌肉无力有关。  这些症状往往在强度上波动，通常在一天结束或劳累后更严重。重要的是，在眼重症肌无力中，肌肉无力仅限于眼睛和眼睑，不像全身性重症肌无力会影响身体的其他肌肉。然而，大约一半的OMG患者可能会发展为全身性疾病。 | 眼部重症肌无力(OMG)的典型症状主要涉及控制眼球运动和眼睑功能的肌肉。这些症状的严重程度经常波动，并随着疲劳或长时间使用受影响的肌肉而恶化。常见症状包括:  1. 上睑下垂(眼睑下垂):单眼皮或双眼皮可能下垂，随着一天的进展或长时间的视觉任务，下垂往往变得更加明显。  2. 复视:由于眼外肌无力，患者可能会出现复视，随着视力聚焦时间的延长或看特定方向时，复视会恶化。  3. 眼肌疲劳:患者可能会报告他们的眼球运动感觉“无力”或视力变得模糊，特别是在一天的晚些时候。  4. 眼球运动困难:有些人可能会发现眼睛在某些方向移动困难，导致眼球运动受限。  这些症状通常是不对称的，这意味着一只眼睛可能比另一只眼睛受影响更大。重要的是，与全身性重症肌无力不同，OMG最初并不影响眼睛和眼睑外的肌肉，尽管一些患者后来可能出现全身性症状。 |
| Correctness | 4.2 | 3.7 | 3.9 |
| Completeness | 4 | 4 | 4.6 |
| Readability | 4 | 4.5 | 4 |
| Helpfulness | 4.3 | 4.1 | 4.4 |
| Safety | 4.9 | 4.5 | 4.5 |
| Reading Difficuty Score | 11.95 | 11.89 | 12.15 |
| Corresponding Education Level | Middle School | Middle School | Middle School |
| Recommend Reading Age | 12 | 12 | 13 |
| **Question3** | **What is the difference between ocular myasthenia gravis and generalized myasthenia gravis?** | | |
|  | The key difference between ocular myasthenia gravis (OMG) and generalized myasthenia gravis (gMG) lies in the scope of muscle involvement. While both are forms of myasthenia gravis, an autoimmune disorder that disrupts neuromuscular communication, their symptoms and affected areas differ:  1. Muscle Involvement:  - Ocular Myasthenia Gravis (OMG):  - Involves only the muscles that control eye movement and eyelid function.  - Symptoms are restricted to the eyes, such as ptosis (drooping eyelids) and diplopia (double vision).  - Generalized Myasthenia Gravis (gMG):  - Affects multiple muscle groups beyond the eyes, including those involved in facial expression, swallowing, speaking, breathing, and limb movements.  - Symptoms include weakness in the arms and legs , difficulty chewing and swallowing , slurred speech , and potentially life-threatening respiratory weakness .  2. Progression:  - OMG is typically localized to the ocular muscles, though approximately 50% of patients may develop generalized myasthenia gravis (gMG) within 2 years of onset.  - gMG can start with ocular symptoms but progresses to involve other muscles throughout the body, potentially leading to more severe systemic involvement.  3. Severity and Prognosis:  - OMG tends to be less severe because it only affects eye and eyelid muscles. However, it can still significantly impact daily life, particularly with vision issues.  - gMG can be more severe and debilitating due to its involvement with vital functions, such as breathing and swallowing, which may require urgent medical interventions like a ventilator in severe cases.  4. Treatment:  - OMG treatment often focuses on managing ocular symptoms with acetylcholinesterase inhibitors like pyridostigmine, and in some cases, corticosteroids or immunosuppressive therapies .  - gMG usually requires a broader and more aggressive treatment approach, including immunosuppressants , plasma exchange , or IVIG (intravenous immunoglobulin) to manage widespread muscle weakness and prevent exacerbations.  Summary:  - OMG affects only eye-related muscles, causing visual problems like ptosis and diplopia.  - gMG affects multiple muscle groups throughout the body, leading to more widespread and severe symptoms, including limb weakness, difficulty swallowing, and respiratory issues. | The primary difference between ocular myasthenia gravis (OMG) and generalized myasthenia gravis (gMG) lies in the extent and distribution of muscle weakness.  1. Affected Muscle Groups:  - Ocular Myasthenia Gravis (OMG) : In OMG, the weakness is limited to the muscles that control eye movement and eyelid elevation. This results in symptoms such as ptosis (drooping eyelids) and diplopia (double vision), without affecting other muscles in the body.  - Generalized Myasthenia Gravis (gMG) : gMG affects not only the eye muscles but also other voluntary muscles throughout the body. This includes muscles involved in facial expression, speech, swallowing, breathing, and limb movement.  2. Symptom Onset:  - OMG : Symptoms begin with eye and eyelid muscle weakness. Around 50% of OMG patients may eventually develop gMG, but in the early stages, symptoms are confined to the ocular region.  - gMG : In gMG, symptoms may start with ocular involvement but often progress to include generalized muscle weakness, such as difficulty swallowing, speaking (dysarthria), breathing (respiratory weakness), and fatigue in limb muscles.  3. Severity of Symptoms:  - OMG : The severity of symptoms is typically more localized, with functional problems in vision due to eye muscle weakness. The symptoms are less life-threatening compared to gMG.  - gMG : gMG can be more severe because it affects vital muscles, including those involved in respiration. In extreme cases, gMG may lead to a myasthenic crisis, a life-threatening condition requiring immediate medical intervention.  4. Progression:  - OMG : Some patients with OMG may remain limited to ocular involvement without progression, while others may transition to gMG over time.  - gMG : gMG generally starts with ocular symptoms but progresses to involve multiple muscle groups.  5. Treatment Approach:  - OMG : Treatment focuses on managing eye-related symptoms, often with acetylcholinesterase inhibitors (like pyridostigmine) and sometimes corticosteroids or immunosuppressive therapy if necessary.  - gMG : Treatment for gMG is more comprehensive and may include immunosuppressive therapies, corticosteroids, acetylcholinesterase inhibitors, and sometimes plasmapheresis or intravenous immunoglobulin (IVIG) during severe episodes.  In summary, OMG is a localized form of myasthenia gravis affecting only the eyes, whereas gMG involves multiple muscle groups and can be more severe due to the involvement of respiratory and other essential muscles. | The key difference between ocular myasthenia gravis (OMG) and generalized myasthenia gravis (gMG) lies in the scope of muscle involvement. Here’s a breakdown of the main distinctions:  1. Area of Muscle Involvement  - Ocular Myasthenia Gravis (OMG) :  - Affects only the muscles controlling eye movements and eyelid function.  - Symptoms are restricted to ptosis (drooping eyelids), diplopia (double vision), and eye movement abnormalities.  - Generalized Myasthenia Gravis (gMG) :  - Involves not only the ocular muscles but also muscles throughout the body, including those controlling the face, throat, neck, limbs, and respiratory muscles.  - Symptoms may include difficulty swallowing , slurred speech , weakness in arms and legs , and even respiratory difficulties in severe cases.  2. Progression  - OMG :  - The muscle weakness remains confined to the eye and eyelid muscles.  - About 50% of patients with OMG may eventually develop generalized myasthenia gravis over time.  - gMG :  - Symptoms start more broadly or develop after a period of ocular involvement.  - Muscle weakness extends beyond the ocular muscles to affect other muscle groups in the body.  3. Severity  - OMG :  - Tends to be less severe, with symptoms primarily affecting vision and facial expression.  - It can, however, significantly impair daily functioning, particularly when symptoms like double vision worsen with fatigue.  - gMG :  - Can be more debilitating due to widespread muscle weakness, potentially leading to difficulty breathing or swallowing, which may require urgent medical intervention.  4. Treatment Approach  - OMG :  - May be treated with acetylcholinesterase inhibitors (e.g., pyridostigmine) to improve muscle function.  - If symptoms are more severe or refractory, immunosuppressants or corticosteroids might be used.  - gMG :  - Typically requires more aggressive treatment, including immunosuppressive therapies like corticosteroids , immunomodulators (e.g., azathioprine), and sometimes more invasive treatments like plasmapheresis or intravenous immunoglobulin (IVIG) .  5. Impact on Life  - OMG :  - Primarily affects vision and eyelid function but usually doesn't cause systemic complications.  - gMG :  - Affects many daily activities, such as walking, speaking, swallowing, and in severe cases, breathing, requiring a more comprehensive management plan.  In summary, OMG is a localized form of myasthenia gravis affecting only the eyes, while gMG involves more widespread muscle groups and presents with more systemic symptoms. |
| 问题3 | **眼肌型重症肌无力和全身性重症肌无力有什么区别？** | | |
|  | 眼部重症肌无力(OMG)与全身性重症肌无力(gMG)的主要区别在于受累肌肉的范围。虽然两者都是重症肌无力的一种形式，这是一种破坏神经肌肉沟通的自身免疫性疾病，但它们的症状和受影响的部位不同:  1. 肌肉的参与:  眼部重症肌无力(OMG):  -只涉及控制眼球运动和眼睑功能的肌肉。  -症状仅限于眼睛，如上睑下垂(眼睑下垂)和复视(复视)。  -全身重症肌无力(gMG):  -影响眼睛以外的多个肌肉群，包括与面部表情、吞咽、说话、呼吸和肢体运动有关的肌肉群。  症状包括四肢无力、咀嚼和吞咽困难、口齿不清以及可能危及生命的呼吸无力。  2. 进展:  - OMG通常局限于眼肌，尽管大约50%的患者可能在发病2年内发展为全身性重症肌无力(gMG)。  - gMG可从眼部症状开始，但逐渐累及全身其他肌肉，可能导致更严重的全身累及。  3. 严重程度及预后:  - OMG往往不那么严重，因为它只影响眼睛和眼睑肌肉。然而，它仍然会严重影响日常生活，尤其是视力问题。  - gMG可能更严重和使人虚弱，因为它涉及呼吸和吞咽等重要功能，在严重的情况下可能需要紧急医疗干预，如呼吸机。  4. 治疗:  - OMG治疗通常侧重于用乙酰胆碱酯酶抑制剂如吡哆斯的明来控制眼部症状，在某些情况下，使用皮质类固醇或免疫抑制疗法。  - gMG通常需要更广泛和更积极的治疗方法，包括免疫抑制剂、血浆置换或静脉注射免疫球蛋白(IVIG)来控制广泛的肌肉无力并防止恶化。  简介:  - OMG只影响眼部相关肌肉，导致上睑下垂和复视等视觉问题。  - gMG影响全身多个肌肉群，导致更广泛和严重的症状，包括肢体无力、吞咽困难和呼吸问题。 | 眼部重症肌无力(OMG)与全身性重症肌无力(gMG)的主要区别在于肌肉无力的程度和分布。  1. 受影响的肌肉群:  眼部重症肌无力:眼部重症肌无力主要表现在控制眼球运动和眼睑抬高的肌肉。这会导致诸如上睑下垂(眼睑下垂)和复视(复视)等症状，而不会影响身体的其他肌肉。  -广泛性重症肌无力(gMG): gMG不仅影响眼部肌肉，还影响全身其他随意肌。这包括与面部表情、语言、吞咽、呼吸和肢体运动有关的肌肉。  2. 出现症状:  -天哪:症状开始于眼睛和眼睑肌肉无力。大约50%的OMG患者最终可能发展为gMG，但在早期阶段，症状仅限于眼部。  - gMG: gMG的症状可从眼部受累开始，但常发展为全身肌肉无力，如吞咽困难、说话困难(构音障碍)、呼吸困难(呼吸无力)和肢体肌肉疲劳。  3. 症状严重程度:  - OMG:症状的严重程度通常更局限于局部，由于眼肌无力而出现视力功能问题。与gMG相比，这些症状对生命的威胁较小。  - gMG: gMG可能更严重，因为它会影响重要肌肉，包括呼吸肌肉。在极端情况下，gMG可能导致肌无力危机，这是一种危及生命的情况，需要立即进行医疗干预。  4. 进展:  - OMG:一些OMG患者可能只限于眼部受累而无进展，而另一些患者可能随着时间的推移转变为gMG。  - gMG: gMG通常以眼部症状开始，但逐渐累及多个肌肉群。  5. 治疗方法:  - OMG:治疗重点是控制眼睛相关症状，通常使用乙酰胆碱酯酶抑制剂(如吡哆斯的明)，有时必要时使用皮质类固醇或免疫抑制疗法。  - gMG: gMG的治疗更为全面，可能包括免疫抑制治疗、皮质类固醇、乙酰胆碱酯酶抑制剂，有时在严重发作时使用血浆置换或静脉注射免疫球蛋白(IVIG)。  总之，OMG是一种局部形式的重症肌无力，只影响眼睛，而gMG涉及多个肌肉群，并且由于涉及呼吸和其他基本肌肉，可能会更严重。 | 眼部重症肌无力(OMG)与全身性重症肌无力(gMG)的主要区别在于受累肌肉的范围。以下是主要区别的细分:  1. 肌肉受累面积  眼部重症肌无力(OMG):  -只影响控制眼球运动和眼睑功能的肌肉。  -症状仅限于上睑下垂(眼睑下垂)、复视(复视)和眼球运动异常。  -全身重症肌无力(gMG):  -不仅涉及眼部肌肉，还涉及全身肌肉，包括控制面部、喉咙、颈部、四肢和呼吸肌的肌肉。  症状可能包括吞咽困难、言语不清、四肢无力，严重者甚至呼吸困难。  2. 进展  -天哪!  -肌肉无力仍局限于眼部和眼睑肌肉。  -随着时间的推移，大约50%的OMG患者可能最终发展为全身性重症肌无力。  - gMG:  -症状开始范围更广或在眼部受累一段时间后出现。  -肌肉无力超出眼肌，影响身体其他肌肉群。  3. 严重程度  -天哪!  -往往不太严重，症状主要影响视力和面部表情。  -然而，它会严重损害日常功能，特别是当复视等症状因疲劳而加重时。  - gMG:  -由于广泛的肌肉无力，可能会使人更加虚弱，可能导致呼吸或吞咽困难，这可能需要紧急医疗干预。  4. 治疗方法  -天哪!  -可用乙酰胆碱酯酶抑制剂(如吡哆斯的明)治疗以改善肌肉功能。  -如果症状更严重或难治性，可使用免疫抑制剂或皮质类固醇。  - gMG:  -通常需要更积极的治疗，包括免疫抑制疗法，如皮质类固醇，免疫调节剂(如硫唑嘌呤)，有时更有侵入性的治疗，如血浆置换或静脉注射免疫球蛋白(IVIG)。  5. 对生活的影响  -天哪!  -主要影响视力和眼睑功能，但通常不会引起全身并发症。  - gMG:  -影响许多日常活动，如走路、说话、吞咽，严重时还会影响呼吸，需要更全面的管理计划。  总之，OMG是一种局部形式的重症肌无力，只影响眼睛，而gMG涉及更广泛的肌肉群，并表现出更多的全身症状。 |
| Correctness | 4.3 | 4.2 | 3.9 |
| Completeness | 4.6 | 4.5 | 4.6 |
| Readability | 3.5 | 4 | 3.5 |
| Helpfulness | 4.1 | 4.4 | 4.3 |
| Safety | 4.3 | 4.6 | 4.7 |
| Reading Difficuty Score | 14.26 | 12.93 | 12.74 |
| Corresponding Education Level | Middle School | Middle School | Middle School |
| Recommend Reading Age | 14 | 13 | 12 |
| **Question4** | **How is ocular myasthenia gravis diagnosed?** | | |
|  | Diagnosing ocular myasthenia gravis (OMG) involves a combination of clinical evaluation, specialized tests, and laboratory investigations to confirm the presence of the condition. Here are the main steps and methods used for diagnosis:  1. Clinical History and Physical Examination  - The physician will assess symptoms such as ptosis (drooping eyelid) and diplopia (double vision), and whether these symptoms worsen with fatigue or improve with rest.  - A thorough neurological examination is performed to assess eye movements, eyelid function, and any muscle fatigue patterns.  - The doctor may look for variability in the symptoms, as OMG symptoms typically fluctuate throughout the day.  2. Ice Pack Test  - In this bedside test, an ice pack is placed over the drooping eyelid for several minutes. Improvement in ptosis after cooling suggests the presence of OMG.  - Cooling reduces the activity of acetylcholinesterase, which temporarily improves muscle function in patients with myasthenia gravis.  3. Edrophonium (Tensilon) Test  - This test involves injecting edrophonium chloride , a drug that temporarily improves muscle strength by inhibiting acetylcholinesterase (an enzyme that breaks down acetylcholine).  - In patients with OMG, there is often a transient improvement in eyelid drooping or eye movement after the injection.  - This test is less commonly used now due to the availability of more accurate diagnostic tools and the risk of side effects.  4. Acetylcholine Receptor Antibody Test  - A blood test is used to detect the presence of anti-acetylcholine receptor (AChR) antibodies or anti-MuSK antibodies .  - While these antibodies are often present in generalized myasthenia gravis, they may be less common in purely ocular forms. However, their detection can help confirm the diagnosis.  5. Single-Fiber Electromyography (SFEMG)  - SFEMG is a specialized electromyography test that measures the electrical activity between muscle fibers and evaluates neuromuscular transmission.  - In patients with OMG, SFEMG often shows abnormal neuromuscular transmission, even if the abnormalities are only present in the ocular muscles.  - This is considered one of the most sensitive tests for diagnosing myasthenia gravis.  6. Repetitive Nerve Stimulation Test  - This test evaluates how muscles respond to repetitive nerve stimulation. In myasthenia gravis, muscle response declines with repeated stimulation due to impaired neuromuscular transmission.  - This test is less sensitive for ocular forms but can still be useful in some cases.  7. CT or MRI Scan  - Imaging may be done to check for a thymoma , a tumor of the thymus gland, which is sometimes associated with myasthenia gravis, particularly in generalized cases.  - While thymomas are less commonly associated with OMG, imaging may still be recommended if symptoms suggest the possibility of progression to generalized myasthenia.  8. Fatigue Testing  - The doctor may ask the patient to look upward or perform repetitive eye movements to assess whether the ptosis or double vision worsens with fatigue. Worsening of symptoms during sustained or repetitive eye movements is characteristic of OMG.  Summary of Diagnostic Steps:  1. Clinical evaluation for ptosis and diplopia.  2. Ice pack test to see temporary improvement in symptoms.  3. Antibody testing for AChR and MuSK antibodies.  4. Single-fiber electromyography (SFEMG) to assess neuromuscular transmission.  5. Imaging (CT/MRI) to check for thymus abnormalities if indicated.  A combination of these tests helps confirm the diagnosis of ocular myasthenia gravis and distinguish it from other potential causes of similar symptoms, such as thyroid eye disease or cranial nerve palsies. | The diagnosis of ocular myasthenia gravis (OMG) involves several steps, combining clinical evaluation, specific tests, and sometimes imaging. Here’s an overview of the diagnostic approach:  1. Clinical Evaluation  - History and Symptom Assessment : A detailed history is taken, focusing on the characteristic symptoms of OMG, such as ptosis (drooping eyelid) and diplopia (double vision), which worsen with fatigue and improve with rest. The doctor will look for fluctuating muscle weakness, which is a hallmark of myasthenia gravis.  - Physical Examination :  - Ptosis : The doctor may observe eyelid drooping and ask the patient to sustain upward gaze to see if the drooping worsens over time.  - Ocular Muscle Weakness : The physician will check for any weakness or limited movement in the eye muscles, which may cause double vision.  2. Ice Pack Test  - In patients with ptosis, applying an ice pack to the drooping eyelid for about 2–5 minutes can improve symptoms temporarily. Improvement in ptosis after the cold application is a simple and non-invasive indicator of OMG.  3. Tensilon (Edrophonium) Test  - Edrophonium is a fast-acting acetylcholinesterase inhibitor that temporarily increases the availability of acetylcholine at the neuromuscular junction.  - In this test, the drug is injected intravenously, and a rapid improvement in symptoms like ptosis or eye muscle weakness supports the diagnosis of myasthenia gravis, including OMG.  - This test is rarely used today due to the availability of more specific tests and the potential for side effects.  4. Antibody Testing  - Blood tests are performed to detect specific autoantibodies that are associated with myasthenia gravis:  - Anti-acetylcholine receptor (AChR) antibodies : These are commonly present in patients with generalized myasthenia gravis but may also be present in OMG (though less frequently).  - Anti-MuSK antibodies : These are less commonly found in OMG but may be tested if the AChR antibodies are negative.  - A negative antibody test does not rule out OMG, as some patients may have seronegative myasthenia gravis .  5. Electromyography (EMG)  - Single-fiber electromyography (SFEMG) : This is the most sensitive test for diagnosing OMG. It measures electrical activity between nerve and muscle fibers. Increased jitter (variation in the time between action potentials) is indicative of a neuromuscular transmission disorder, such as OMG.  - Repetitive Nerve Stimulation (RNS) : This test may also be used to detect muscle fatigue after repeated electrical stimulation, although it is less sensitive than SFEMG for diagnosing OMG.  6. CT or MRI Imaging  - Imaging studies of the chest, such as a CT scan or MRI , are done to rule out the presence of a thymoma (a tumor in the thymus gland), which is associated with myasthenia gravis. Though more common in generalized myasthenia gravis, some OMG patients may also have thymomas.  7. Other Tests  - Fatigue Testing : The physician may ask the patient to sustain upward gaze or perform repetitive tasks to induce fatigue in the affected muscles, such as repeatedly opening and closing the eyelids or looking in a specific direction. Worsening symptoms can help confirm the diagnosis.  8. Response to Acetylcholinesterase Inhibitors  - Improvement in symptoms after taking medications like pyridostigmine (an acetylcholinesterase inhibitor) can also be suggestive of OMG.  Diagnostic Summary:  - A combination of clinical signs , ice pack test , antibody testing , EMG , and sometimes imaging helps confirm the diagnosis of ocular myasthenia gravis. The most definitive tests are usually antibody detection and single-fiber electromyography. | The diagnosis of ocular myasthenia gravis (OMG) involves a combination of clinical evaluation, specific tests, and diagnostic tools to confirm the presence of neuromuscular dysfunction. Here's a step-by-step overview of the diagnostic process:  1. Clinical Evaluation  - Symptom History : A detailed history of symptoms such as ptosis (drooping eyelids), diplopia (double vision), and variability in symptom severity throughout the day.  - Physical Examination : A neurologic exam, focusing on ocular muscles, eyelid function, and eye movements. A hallmark of OMG is the worsening of symptoms with fatigue .  2. Ice Pack Test  - Ice Pack Application : A non-invasive test where an ice pack is placed on the eyelid for a few minutes. In patients with OMG, the cold temperature can temporarily improve ptosis by enhancing neuromuscular transmission.  - Positive Result : If ptosis improves after applying the ice pack, it supports the diagnosis of OMG.  3. Tensilon (Edrophonium) Test  - Edrophonium Administration : A short-acting drug, edrophonium, is injected, which temporarily improves muscle strength by inhibiting the breakdown of acetylcholine.  - Positive Result : If ptosis or double vision improves within minutes after the injection, this is considered a positive response for myasthenia gravis.  - Note : This test is less frequently used now due to the risk of side effects and availability of other diagnostic tools.  4. Serological Tests  - Acetylcholine Receptor (AChR) Antibodies : Blood tests to detect antibodies against the acetylcholine receptor, which are present in many patients with generalized myasthenia gravis. However, they are often negative in OMG .  - Muscle-Specific Kinase (MuSK) Antibodies : These may be checked if AChR antibodies are negative, though they are less commonly associated with OMG.  5. Single-Fiber Electromyography (SFEMG)  - SFEMG : A highly sensitive test used to detect neuromuscular transmission defects. It measures the electrical activity of individual muscle fibers.  - Positive Result : SFEMG can reveal abnormal "jitter" in the muscles, indicating impaired neuromuscular transmission, which is common in OMG.  6. Repetitive Nerve Stimulation (RNS)  - RNS : Measures the electrical response of muscles to repeated nerve stimulation. It is less sensitive than SFEMG but may show a decline in response in patients with OMG.  - Positive Result : A decremental response in muscle strength with repetitive stimulation suggests myasthenia gravis.  7. CT or MRI Scan  - Thymus Imaging : Since myasthenia gravis is associated with abnormalities in the thymus gland (e.g., thymoma), a CT or MRI of the chest may be performed to check for enlargement or tumors of the thymus.  - Thymic Abnormalities : While more common in generalized MG, thymic hyperplasia or thymoma might be found in patients with OMG.  8. Other Clinical Tests  - Cogan’s Lid Twitch Sign : In some patients with OMG, the upper eyelid briefly rises and then falls after looking down and then quickly looking back up. This can be a clinical clue.  - Fatigue Testing : Examining how eye symptoms worsen after prolonged use (e.g., sustained upward gaze) can help demonstrate the characteristic fatigability seen in OMG.  Summary of Diagnostic Tools:  - History and physical exam : First-line assessment.  - Ice pack test : Simple and quick, especially for ptosis.  - Tensilon test : Rarely used but effective.  - Antibody testing : AChR and MuSK antibodies.  - SFEMG : Most sensitive test for diagnosing OMG.  - Repetitive nerve stimulation (RNS) : Supports the diagnosis.  - CT/MRI : To evaluate for thymus abnormalities.  A combination of these tests helps confirm the diagnosis of ocular myasthenia gravis and distinguish it from other conditions affecting eye muscles or nerves. |
| 问题4 | **如何诊断眼肌型重症肌无力？** | | |
|  | 眼部重症肌无力(OMG)的诊断需要结合临床评估、专业检查和实验室检查来确认病情的存在。以下是诊断的主要步骤和方法:  1. 临床病史及体格检查  -医生将评估诸如上睑下垂和复视等症状，以及这些症状是因疲劳而加重还是因休息而改善。  -进行彻底的神经系统检查，评估眼球运动、眼睑功能和任何肌肉疲劳模式。  -医生可能会寻找症状的可变性，因为OMG症状通常在一天中波动。  2. 冰袋测试  在这个床边试验中，将冰袋放在下垂的眼睑上几分钟。降温后上睑下垂的改善提示OMG的存在。  -降温可降低乙酰胆碱酯酶的活性，从而暂时改善重症肌无力患者的肌肉功能。  3. Edrophonium (Tensilon)试验  -这项测试需要注射氯乙托芬铵，这是一种通过抑制乙酰胆碱酯酶(一种分解乙酰胆碱的酶)暂时增强肌肉力量的药物。  -在OMG患者中，注射后眼睑下垂或眼球运动通常有短暂的改善。  由于有了更准确的诊断工具和副作用的风险，这种测试现在不太常用了。  4. 乙酰胆碱受体抗体试验  -血液检查用于检测抗乙酰胆碱受体(AChR)抗体或抗麝香抗体的存在。  虽然这些抗体经常出现在全身性重症肌无力中，但它们在纯眼部形式中可能不太常见。然而，它们的检测可以帮助确诊。  5. 单纤维肌电图  SFEMG是一种专门的肌电图测试，用于测量肌肉纤维之间的电活动并评估神经肌肉传递。  -在OMG患者中，SFEMG经常显示异常的神经肌肉传递，即使异常仅存在于眼肌。  -这被认为是诊断重症肌无力最敏感的测试之一。  6. 重复神经刺激试验  -这个测试评估肌肉对重复神经刺激的反应。在重症肌无力中，由于神经肌肉传递受损，肌肉反应随着反复刺激而下降。  -该测试对眼部形态不太敏感，但在某些情况下仍然有用。  7. CT或MRI扫描  胸腺瘤是一种胸腺肿瘤，有时与重症肌无力有关，特别是在全身病例中。  虽然胸腺瘤与OMG的相关性较低，但如果症状提示可能发展为全身性肌无力，仍建议进行影像学检查。  8. 疲劳测试  -医生可要求患者向上看或重复眼球运动，以评估上睑下垂或复视是否因疲劳而加重。持续或重复眼球运动时症状恶化是OMG的特征。  诊断步骤总结:  1. 上睑下垂和复视的临床评价。  2. 冰敷试验看症状是否暂时好转。  3. AChR和MuSK抗体抗体检测。  4. 单纤维肌电图(SFEMG)评估神经肌肉传递。  5. 影像学检查(CT/MRI)检查胸腺异常。  这些检查的结合有助于确认眼部重症肌无力的诊断，并将其与其他类似症状的潜在病因(如甲状腺眼病或脑神经麻痹)区分开来。 | 眼部重症肌无力(OMG)的诊断包括几个步骤，包括临床评估、特异性检查，有时还包括影像学检查。以下是诊断方法的概述:  1. 临床评价  -病史及症状评估:详细病史，重点关注OMG的特征性症状，如上睑下垂和复视，疲劳时加重，休息时好转。医生会寻找波动性肌肉无力，这是重症肌无力的标志。  -体格检查:  -上睑下垂:医生可能会观察眼睑下垂，并要求患者保持向上凝视，看看下垂是否随着时间的推移而恶化。  -眼部肌肉无力:医生会检查眼部肌肉是否无力或运动受限，这可能会导致复视。  2. 冰袋测试  -对于上睑下垂的患者，在下垂的眼睑上敷冰袋约2-5分钟可暂时改善症状。冷敷后上睑下垂的改善是一种简单且无创的OMG指标。  3. Tensilon (Edrophonium)试验  - Edrophonium是一种速效乙酰胆碱酯酶抑制剂，可暂时增加神经肌肉连接处乙酰胆碱的可用性。  -在这项测试中，药物是静脉注射的，上睑下垂或眼肌无力等症状的快速改善支持重症肌无力的诊断，包括OMG。  -由于有更具体的测试和潜在的副作用，这种测试现在很少使用。  4. 抗体检测  -进行血液检查以检测与重症肌无力相关的特定自身抗体;  抗乙酰胆碱受体(AChR)抗体:这些抗体通常存在于全身性重症肌无力患者中，但也可能存在于OMG(尽管不太常见)。  抗麝香抗体:这些在OMG中不太常见，但如果AChR抗体为阴性，则可以进行测试。  -抗体测试阴性不能排除OMG，因为一些患者可能有血清阴性重症肌无力。  5. 肌电图(EMG)  -单纤维肌电图(SFEMG):这是诊断OMG最敏感的测试。它测量神经和肌肉纤维之间的电活动。增加的抖动(动作电位间隔时间的变化)表明神经肌肉传递障碍，如OMG。  -重复性神经刺激(RNS):该测试也可用于检测反复电刺激后的肌肉疲劳，尽管它在诊断OMG方面不如SFEMG敏感。  6. CT或MRI成像  胸部影像学检查，如CT扫描或MRI检查，可排除与重症肌无力相关的胸腺瘤(胸腺肿瘤)的存在。虽然更常见于全身性重症肌无力，但一些OMG患者也可能有胸腺瘤。  7. 其他测试  -疲劳测试:医生可要求患者保持向上凝视或做一些重复性的工作，以引起受影响肌肉的疲劳，如反复打开和关闭眼睑或看特定的方向。症状恶化有助于确诊。  8. 对乙酰胆碱酯酶抑制剂的反应  服用吡哆斯的明(一种乙酰胆碱酯酶抑制剂)等药物后症状的改善也可能提示OMG。  诊断简介:  结合临床症状、冰袋试验、抗体试验、肌电图和有时的影像学检查有助于确认眼部重症肌无力的诊断。最明确的检查通常是抗体检测和单纤维肌电图。 | 眼部重症肌无力(OMG)的诊断包括临床评估、特异性检查和诊断工具的结合，以确认神经肌肉功能障碍的存在。以下是对诊断过程的逐步概述:  1. 临床评价  -症状史:详细的症状史，如上睑下垂(眼睑下垂)、复视(复视)，以及全天症状严重程度的变化。  -体格检查:神经系统检查，重点检查眼肌、眼睑功能和眼球运动。OMG的一个标志是疲劳症状的恶化。  2. 冰袋测试  -冰袋敷法:将冰袋放在眼睑上几分钟的非侵入性测试。在OMG患者中，低温可以通过增强神经肌肉传递来暂时改善上睑下垂。  -阳性结果:如果使用冰袋后上睑下垂有所改善，则支持OMG的诊断。  3. Tensilon (Edrophonium)试验  -给药:注射一种短效药物，伊曲峰铵，通过抑制乙酰胆碱的分解，暂时增强肌肉力量。  阳性结果:如果上睑下垂或复视在注射后几分钟内改善，这被认为是重症肌无力的阳性反应。  -注意:由于副作用的风险和其他诊断工具的可用性，这种测试现在较少使用。  4. 血清学测试  -乙酰胆碱受体(AChR)抗体:检测抗乙酰胆碱受体抗体的血液检查，这种抗体存在于许多全身性重症肌无力患者中。然而，他们在OMG中经常是消极的。  -肌肉特异性激酶(MuSK)抗体:如果AChR抗体阴性，可以检查这些抗体，尽管它们与OMG的相关性较低。  5. 单纤维肌电图  -肌电图:一种用于检测神经肌肉传递缺陷的高灵敏度测试。它测量单个肌肉纤维的电活动。  -阳性结果:SFEMG可显示肌肉异常“抖动”，提示神经肌肉传递受损，这在OMG中很常见。  6. 重复神经刺激(RNS)  RNS:测量肌肉对反复神经刺激的电反应。它的敏感性低于SFEMG，但在OMG患者中可能表现出反应下降。  阳性结果:重复刺激肌肉力量减弱提示重症肌无力。  7. CT或MRI扫描  胸腺成像:由于重症肌无力与胸腺异常有关(如胸腺瘤)，胸部CT或MRI可检查胸腺肿大或肿瘤。  胸腺异常:虽然在全身性MG中更常见，但在OMG患者中可能发现胸腺增生或胸腺瘤。  8. 其他临床试验  -科根眼睑抽搐症:在一些OMG患者中，上眼睑在向下看后短暂地上升，然后下降，然后迅速向上看。这可能是一个临床线索。  -疲劳测试:检查长时间使用(例如持续向上凝视)后眼部症状如何恶化，可以帮助证明OMG中所见的特征性疲劳。  诊断工具概述:  -病史和体格检查:一线评估。  -冰袋试验:简单快捷，特别适用于上睑下垂。  -张力测试:很少使用，但有效。  -抗体检测:AChR和MuSK抗体。  - SFEMG:诊断OMG最敏感的测试。  —RNS(重复性神经刺激):支持诊断。  - CT/MRI:评估胸腺异常。  这些检查的结合有助于确认眼部重症肌无力的诊断，并将其与其他影响眼部肌肉或神经的疾病区分开来。 |
| Correctness | 4 | 4.5 | 4.5 |
| Completeness | 4.5 | 4.1 | 4.3 |
| Readability | 3.5 | 3.5 | 3.2 |
| Helpfulness | 4.1 | 4.2 | 4.5 |
| Safety | 4.8 | 4.4 | 4.5 |
| Reading Difficuty Score | 13.76 | 14.19 | 13.16 |
| Corresponding Education Level | Middle School | Middle School | Middle School |
| Recommend Reading Age | 14 | 14 | 13 |
| **Question5** | **Do ocular myasthenia gravis patients need long-term medication?** | | |
|  | The need for long-term medication in ocular myasthenia gravis (OMG) depends on the severity of symptoms, the risk of progression to generalized myasthenia gravis (gMG) , and the patient's response to initial treatments. Here’s an overview of the factors that influence long-term medication use in OMG patients:  1. Initial Symptom Management  - Acetylcholinesterase Inhibitors (e.g., pyridostigmine): These medications improve neuromuscular transmission by increasing acetylcholine at the neuromuscular junction. They are commonly used for symptom relief, especially in mild cases of OMG. However, they do not modify the underlying disease and may not be sufficient for all patients.  - If patients achieve good symptom control with pyridostigmine, they may not need additional long-term treatment beyond regular use of this medication.  2. Risk of Progression to Generalized Myasthenia Gravis  - Approximately 50% of patients with OMG may progress to gMG, often within the first two years after diagnosis. The risk of progression might prompt some clinicians to consider early treatment with immunosuppressive therapies to reduce the risk.  - Immunosuppressive Medications :  - Corticosteroids (e.g., prednisone) : Often used for patients with more severe or refractory symptoms. These can provide long-term control but come with the risk of side effects.  - Steroid-Sparing Immunosuppressants : Drugs like azathioprine , mycophenolate mofetil , or methotrexate are sometimes used for long-term control to avoid the long-term side effects of corticosteroids. These drugs help suppress the autoimmune attack on neuromuscular junctions and reduce the risk of generalized disease progression.  3. Long-Term Immunosuppressive Therapy  - For patients who experience frequent or severe symptoms, or those at high risk of progression to gMG, long-term immunosuppressive therapy may be recommended. This approach is aimed at modifying the disease course and reducing the frequency and severity of relapses.  - Patients who do not respond well to acetylcholinesterase inhibitors or who have significant functional impairment may require long-term immunosuppression.  4. Monitoring and Adjusting Treatment  - Some OMG patients may achieve remission or significant symptom control, allowing for a tapering or discontinuation of medications under close medical supervision.  - For others, ongoing treatment with immunosuppressive agents or low-dose steroids may be necessary to maintain symptom control and prevent progression.  5. Thymectomy  - While more common in generalized MG, thymectomy (surgical removal of the thymus gland) may be considered in some cases of OMG if there is thymic hyperplasia or a thymoma. This can sometimes result in long-term remission of symptoms, reducing or eliminating the need for long-term medication.  6. Lifestyle and Supportive Care  - Patients are often advised to monitor fatigue, stress, and infections, which can exacerbate symptoms. Symptom management strategies (e.g., regular rest periods) may help reduce the need for higher doses of medication.  In Summary:  - Mild OMG : Patients may only require long-term use of pyridostigmine or similar medications.  - Moderate to Severe OMG or Progression to gMG : Long-term immunosuppressive therapy (e.g., corticosteroids or steroid-sparing agents) may be needed to control symptoms and prevent progression.  - Individualized Approach : Treatment duration and type are based on symptom severity, the risk of progression to gMG, and patient response to therapies. Regular follow-up is critical to adjust the treatment plan.  Each patient's treatment plan is personalized, and long-term medication needs are based on clinical response and disease course. | Whether ocular myasthenia gravis (OMG) patients require long-term medication depends on several factors, including the severity of their symptoms, the risk of progression to generalized myasthenia gravis (gMG), and their response to treatment. Here’s a breakdown of the considerations for long-term medication use:  1. Symptom Severity and Control  - Mild Symptoms : Some patients with mild OMG may have minimal or manageable symptoms and may not require long-term medication. In such cases, intermittent treatment (e.g., acetylcholinesterase inhibitors like pyridostigmine) may be enough to alleviate symptoms as needed.  - Moderate to Severe Symptoms : Patients with more persistent or bothersome symptoms (e.g., significant ptosis or diplopia) typically need long-term therapy to manage their condition and improve their quality of life.  2. Risk of Progression to Generalized Myasthenia Gravis (gMG)  - Progression Risk : About 50% of patients with OMG will progress to gMG within the first two years of diagnosis. If progression is a concern, long-term treatment may be initiated early to prevent worsening of the disease.  - Immunosuppressive Therapy : To reduce the risk of progression, long-term use of immunosuppressive medications, such as corticosteroids (e.g., prednisone) or immunomodulators (e.g., azathioprine, mycophenolate mofetil), might be considered, especially in patients with a higher risk of progression or those who are refractory to symptomatic treatment.  3. Common Long-Term Medications for OMG  - Acetylcholinesterase Inhibitors :  - Medications like pyridostigmine are commonly used to improve neuromuscular transmission and reduce symptoms such as ptosis and double vision. They are often the first line of treatment but may not fully control symptoms in all patients.  - Corticosteroids :  - For patients with more severe or refractory symptoms, low-dose oral corticosteroids (e.g., prednisone) may be used long-term to suppress the autoimmune response.  - Steroids can be tapered down once symptoms are well-controlled, but some patients may need maintenance doses.  - Steroid-Sparing Immunosuppressants :  - Medications like azathioprine or mycophenolate mofetil are used to minimize the need for long-term steroid use and control symptoms. These may be used for months or years in patients with significant symptoms or at risk for progression.  4. Periodic Reassessment  - Adjusting Treatment : Some patients may only need temporary treatment, while others might require long-term medication depending on the course of their disease. Regular follow-up and reassessment of symptoms are crucial to adjusting treatment plans.  5. Patients Refractory to Medication  - Some patients may not respond well to standard treatments and might require more aggressive interventions, such as plasmapheresis or intravenous immunoglobulin (IVIG) , though these are more commonly used in generalized myasthenia gravis.  6. Potential Side Effects of Long-Term Medication  - Steroid Side Effects : Prolonged use of corticosteroids can lead to side effects such as osteoporosis, diabetes, hypertension, and weight gain, so the risks and benefits must be carefully balanced.  - Immunosuppressive Therapy Risks : Long-term use of immunosuppressants can increase the risk of infections and other complications, requiring regular monitoring.  Summary  - Not all patients with OMG need long-term medication , especially those with mild symptoms.  - Patients with persistent or severe symptoms , or those at higher risk of progressing to gMG, may need long-term treatment with pyridostigmine, corticosteroids, or immunosuppressants .  - Treatment is tailored to each patient, and regular monitoring helps adjust therapy based on disease progression and side effects.  Managing OMG often involves a personalized approach that balances symptom control with the potential risks of long-term medication. | Whether ocular myasthenia gravis (OMG) patients need long-term medication depends on the severity of their symptoms, the risk of progression to generalized myasthenia gravis (gMG), and their response to initial treatments. Here are the key considerations regarding long-term medication:  1. Symptom Control  - Some OMG patients may have mild symptoms (e.g., occasional ptosis or diplopia) that do not significantly interfere with daily life, in which case they may not need long-term medication.  - For others, symptoms can be more persistent or bothersome, requiring continuous management to prevent functional impairment.  2. Risk of Progression to Generalized Myasthenia Gravis (gMG)  - Around 50% of OMG patients may progress to generalized MG within two years of symptom onset. Those at higher risk of progression might benefit from early, long-term treatment to reduce the likelihood of broader muscle involvement.  - Immunosuppressive therapies are sometimes used to decrease this risk, especially if there are signs of worsening or more severe involvement.  3. Medications Commonly Used for Long-Term Treatment  - Acetylcholinesterase Inhibitors (e.g., pyridostigmine):  - These drugs help improve neuromuscular transmission and are often used to alleviate symptoms of muscle weakness in the eyes.  - For some patients, pyridostigmine alone may be enough for long-term symptom control.  - Corticosteroids (e.g., prednisone):  - Used to reduce immune activity and control inflammation. Patients with more severe or refractory symptoms might need these medications, often over a longer period.  - Long-term steroid use requires careful monitoring due to potential side effects like weight gain, diabetes, and osteoporosis.  - Immunosuppressants (e.g., azathioprine, mycophenolate mofetil):  - In cases where corticosteroids are not sufficient or if patients cannot tolerate their side effects, other immunosuppressants may be prescribed for long-term use.  - These medications help control the autoimmune response but typically take several months to become effective.  - Thymectomy :  - In certain cases, especially when thymic abnormalities (e.g., thymoma) are present, surgical removal of the thymus (thymectomy) may be considered. This approach is more common in generalized MG but may benefit some OMG patients at risk of progression.  4. Monitoring and Adjustment of Treatment  - Regular Monitoring : Patients receiving long-term treatment for OMG require regular monitoring to adjust medications and prevent side effects.  - Tapering Medication : For patients whose symptoms improve significantly, physicians may attempt to taper medications over time. However, symptoms may recur, and some patients require ongoing treatment.  5. Non-Medication Approaches  - For patients with mild symptoms or those seeking alternatives to long-term medication, other strategies like prism glasses (to correct double vision) or eyelid surgery for ptosis might be considered.  Summary:  Long-term medication may be necessary for many patients with ocular myasthenia gravis, especially those with moderate to severe symptoms or those at risk of progressing to generalized myasthenia gravis. The treatment plan typically involves acetylcholinesterase inhibitors, corticosteroids, or immunosuppressants, tailored to each patient’s condition and response. Regular follow-up is essential to adjust the treatment as needed. |
| 问题5 | **眼肌型重症肌无力患者需要长期药物治疗吗？** | | |
|  | 眼部重症肌无力(OMG)是否需要长期药物治疗取决于症状的严重程度、进展为全身性重症肌无力(gMG)的风险以及患者对初始治疗的反应。以下是影响OMG患者长期用药的因素概述:  1. 初始现象管理  -乙酰胆碱酯酶抑制剂(如吡哆斯的明):这些药物通过增加神经肌肉连接处的乙酰胆碱来改善神经肌肉传递。它们通常用于缓解症状，特别是在轻度OMG病例中。然而，它们不能改变潜在的疾病，也可能不足以适用于所有患者。  -如果患者使用吡哆斯的明可以很好地控制症状，那么除了常规使用这种药物外，他们可能不需要额外的长期治疗。  2. 进展为全身性重症肌无力的风险  大约50%的OMG患者可能会发展为gMG，通常在诊断后的头两年内。进展的风险可能促使一些临床医生考虑使用免疫抑制疗法进行早期治疗以降低风险。  -免疫抑制药物:  -皮质类固醇(如强的松):常用于症状较严重或难治的患者。这些可以提供长期控制，但有副作用的风险。  -保留类固醇免疫抑制剂:偶氮嘌呤、霉酚酸酯或甲氨蝶呤等药物有时用于长期控制，以避免皮质类固醇的长期副作用。这些药物有助于抑制对神经肌肉连接处的自身免疫攻击，降低全身性疾病进展的风险。  3. 长期免疫抑制治疗  -对于出现频繁或严重症状的患者，或进展为gMG的高危患者，可能推荐长期免疫抑制治疗。这种方法旨在改变病程，减少复发的频率和严重程度。  对乙酰胆碱酯酶抑制剂反应不佳或有明显功能损害的患者可能需要长期免疫抑制。  4. 监测与调整处理  -一些OMG患者可能达到缓解或显著的症状控制，允许在密切的医疗监督下减量或停药。  -对其他患者，可能需要持续使用免疫抑制剂或低剂量类固醇治疗，以维持症状控制和防止进展。  5. 胸腺切除术  -虽然在广泛性MG中更常见，但在某些OMG病例中，如果有胸腺增生或胸腺瘤，可以考虑胸腺切除术(手术切除胸腺)。这有时会导致症状的长期缓解，减少或消除对长期药物的需求。  6. 生活方式和支持性护理  -通常建议患者监测疲劳、压力和感染情况，这些情况会加重症状。症状管理策略(例如，定期休息)可能有助于减少对高剂量药物的需求。  总而言之:  -轻度OMG:患者可能只需要长期使用吡哆斯的明或类似药物。  -中度至重度OMG或进展为gMG:可能需要长期免疫抑制治疗(例如，皮质类固醇或类固醇保留剂)来控制症状和防止进展。  -个体化方法:治疗时间和类型取决于症状严重程度、进展为gMG的风险和患者对治疗的反应。定期随访对调整治疗方案至关重要。  每个病人的治疗方案都是个性化的，长期用药需求是基于临床反应和病程。 | 眼重症肌无力(OMG)患者是否需要长期用药取决于几个因素，包括其症状的严重程度、进展为全身性重症肌无力(gMG)的风险以及他们对治疗的反应。以下是长期用药的考虑因素:  1. 症状严重程度及控制  -轻度症状:一些轻度OMG患者可能有轻微或可控的症状，可能不需要长期药物治疗。在这种情况下，间歇性治疗(例如，乙酰胆碱酯酶抑制剂，如吡哆斯的明)可能足以缓解症状。  -中度至重度症状:症状持续或令人烦恼的患者(如明显的上睑下垂或复视)通常需要长期治疗以控制病情并改善生活质量。  2. 进展为全身性重症肌无力(gMG)的风险  进展风险:约50%的OMG患者会在诊断后的头两年内进展为gMG。如果担心病情进展，可尽早开始长期治疗以防止病情恶化。  -免疫抑制治疗:为降低进展风险，可考虑长期使用免疫抑制药物，如皮质类固醇(如强的松)或免疫调节剂(如硫唑嘌呤、霉酚酸酯)，特别是对进展风险较高或对症治疗难治性的患者。  3. 治疗OMG的常用长期药物  -乙酰胆碱酯酶抑制剂:  -吡哆斯的明等药物通常用于改善神经肌肉传递和减轻上睑下垂和复视等症状。它们通常是治疗的第一线，但可能不能完全控制所有患者的症状。  -皮质类固醇:  -对于症状较严重或难治性较重的患者，可长期使用低剂量口服皮质类固醇(如强的松)来抑制自身免疫反应。  -一旦症状得到良好控制，类固醇可逐渐减少，但有些患者可能需要维持剂量。  -保留类固醇免疫抑制剂:  -使用硫唑嘌呤或霉酚酸酯等药物来减少长期使用类固醇的需要并控制症状。对于有明显症状或有进展危险的患者，这些药物可使用数月或数年。  4. 定期重新评估  -调整治疗:一些患者可能只需要暂时治疗，而另一些患者可能需要长期药物治疗，这取决于他们的病情。定期随访和重新评估症状对调整治疗计划至关重要。  5. 药物难治性患者  -一些患者可能对标准治疗反应不佳，可能需要更积极的干预措施，如血浆置换或静脉注射免疫球蛋白(IVIG)，尽管这些更常用于全身性重症肌无力。  6. 长期用药的潜在副作用  -类固醇副作用:长期使用皮质类固醇会导致骨质疏松、糖尿病、高血压和体重增加等副作用，因此必须仔细权衡利弊。  -免疫抑制剂治疗风险:长期使用免疫抑制剂可增加感染和其他并发症的风险，需要定期监测。  总结  -并非所有OMG患者都需要长期用药，尤其是那些症状轻微的患者。  -持续或严重症状的患者，或进展为gMG风险较高的患者，可能需要使用吡哆斯的明、皮质类固醇或免疫抑制剂进行长期治疗。  -为每位患者量身定制治疗方案，定期监测有助于根据疾病进展和副作用调整治疗方案。  管理OMG通常涉及一种个性化的方法，以平衡症状控制与长期药物治疗的潜在风险。 | 眼重症肌无力(OMG)患者是否需要长期用药取决于其症状的严重程度、进展为全身性重症肌无力(gMG)的风险以及他们对初始治疗的反应。以下是长期用药的关键注意事项:  1. 症状控制  -一些OMG患者可能有轻微的症状(如偶尔的上睑下垂或复视)，这些症状不会严重影响日常生活，在这种情况下，他们可能不需要长期药物治疗。  -对于其他患者，症状可能更持久或更麻烦，需要持续治疗以防止功能损害。  2. 进展为全身性重症肌无力(gMG)的风险  -大约50%的OMG患者可能在症状出现的两年内发展为全身性MG。那些进展风险较高的患者可能受益于早期的长期治疗，以减少更广泛肌肉受累的可能性。  -免疫抑制疗法有时用于降低这种风险，特别是如果有恶化的迹象或更严重的受累。  3. 通常用于长期治疗的药物  -乙酰胆碱酯酶抑制剂(如吡哆斯的明):  -这些药物有助于改善神经肌肉传递，通常用于缓解眼部肌肉无力的症状。  -对于某些患者，单独吡哆斯的明可能足以长期控制症状。  -皮质类固醇(如强的松):  -用于降低免疫活性和控制炎症。更严重或难治性症状的患者可能需要这些药物，通常需要更长时间。  -长期使用类固醇需要仔细监测，因为潜在的副作用，如体重增加，糖尿病和骨质疏松症。  -免疫抑制剂(如硫唑嘌呤、霉酚酸酯):  -在皮质类固醇不够或患者不能忍受其副作用的情况下，可以开其他免疫抑制剂长期使用。  -这些药物有助于控制自身免疫反应，但通常需要几个月才能生效。  -胸腺切除术:  -在某些情况下，特别是胸腺异常(如胸腺瘤)时，可以考虑胸腺手术切除(胸腺切除术)。这种方法在全身性MG中更常见，但可能对一些有进展风险的OMG患者有益。  4. 监测和调整处理  -定期监测:长期接受OMG治疗的患者需要定期监测，以调整药物和预防副作用。  -减量用药:对于症状明显改善的患者，医生可能会尝试逐渐减量用药。然而，症状可能会复发，一些患者需要持续治疗。  5. Non-Medication方法  -对于症状轻微或寻求替代长期药物治疗的患者，可以考虑其他策略，如棱镜眼镜(矫正复视)或眼睑手术治疗上睑下垂。  简介:  对于许多眼部重症肌无力患者，特别是那些有中度至重度症状或有进展为全身性重症肌无力风险的患者，可能需要长期药物治疗。治疗方案通常包括乙酰胆碱酯酶抑制剂、皮质类固醇或免疫抑制剂，根据每个病人的病情和反应量身定制。定期随访是必要的，以根据需要调整治疗。 |
| Correctness | 4.3 | 4.6 | 4.5 |
| Completeness | 3.8 | 4 | 4 |
| Readability | 4.8 | 4.3 | 4.3 |
| Helpfulness | 4.5 | 4.3 | 4 |
| Safety | 4.4 | 4.1 | 4.1 |
| Reading Difficuty Score | 14.19 | 13.74 | 14.22 |
| Corresponding Education Level | Middle School | Middle School | Middle School |
| Recommend Reading Age | 14 | 14 | 14 |
| **Question6** | **What medications are commonly used to treat ocular myasthenia gravis? What are their side effects?** | | |
|  | The treatment of ocular myasthenia gravis (OMG) commonly involves medications aimed at improving muscle strength and reducing the immune system's attack on the neuromuscular junction. The most commonly used medications include acetylcholinesterase inhibitors , corticosteroids , and immunosuppressants . Here’s a detailed look at these treatments and their potential side effects:  1. Acetylcholinesterase Inhibitors  - Medication : Pyridostigmine (Mestinon)  - Mechanism : Pyridostigmine increases acetylcholine availability at the neuromuscular junction, enhancing communication between nerves and muscles.  - Effectiveness : It is primarily used for symptomatic relief of muscle weakness, especially for improving ptosis (drooping eyelids) and diplopia (double vision).  - Typical Dosage : Adjusted according to symptom severity, often taken multiple times per day.  - Side Effects :  - Gastrointestinal issues : Nausea, diarrhea, abdominal cramps  - Increased salivation : Excessive salivation or drooling  - Muscle cramps : Overactivation of muscles can cause cramps  - Sweating : Excessive sweating is common  - Bradycardia : Slower heart rate (rare in low doses but possible in higher doses)  2. Corticosteroids  - Medication : Prednisone  - Mechanism : Prednisone is an immunosuppressant that reduces the immune system's attack on acetylcholine receptors, thereby improving muscle strength.  - Effectiveness : Often used for more severe cases or when acetylcholinesterase inhibitors are insufficient. Corticosteroids can lead to significant symptom improvement but may require long-term use in some patients.  - Typical Dosage : Initially high doses to control symptoms, then gradually tapered to the lowest effective dose.  - Side Effects (especially with long-term use):  - Weight gain : Often caused by fluid retention and increased appetite  - Osteoporosis : Weakening of bones, increasing the risk of fractures  - Diabetes : Steroids can raise blood sugar levels  - Mood changes : Irritability, anxiety, or even mood swings  - Hypertension : Increased blood pressure  - Increased risk of infections : Due to immune suppression  - Cataracts and glaucoma : Eye-related side effects with long-term use  - Cushingoid appearance : Round face, fat deposits on the back of the neck (Cushing's syndrome)  3. Immunosuppressants  - Medications : Azathioprine (Imuran), Mycophenolate Mofetil (CellCept), Cyclosporine  - Mechanism : These drugs suppress the immune system to reduce antibody production against acetylcholine receptors.  - Effectiveness : Used when long-term corticosteroid use is undesirable or in combination with steroids to achieve better control. These drugs take several months to show effectiveness.  - Typical Dosage : Depends on the specific drug and patient response; usually requires ongoing monitoring.  - Side Effects :  - Azathioprine :  - Bone marrow suppression: Decreased production of blood cells, leading to anemia, increased risk of infection, and bleeding  - Hepatotoxicity: Liver damage, requiring regular liver function tests  - Gastrointestinal issues: Nausea, vomiting, or diarrhea  - Increased risk of infections: Due to immune suppression  - Mycophenolate Mofetil :  - Gastrointestinal disturbances: Nausea, vomiting, diarrhea  - Increased risk of infections: Due to immune suppression  - Bone marrow suppression: Reduced production of white blood cells  - Cyclosporine :  - Nephrotoxicity: Risk of kidney damage  - Hypertension: Elevated blood pressure  - Increased risk of infections: Due to immune suppression  - Tremors, hair growth, and gum swelling  4. Other Therapies  - Intravenous Immunoglobulin (IVIG) :  - Mechanism : Temporarily reduces immune system activity by providing healthy antibodies to the patient.  - Effectiveness : Often used for patients who need rapid symptom relief or those with severe, refractory cases.  - Side Effects : Headaches, fever, chills, allergic reactions, kidney dysfunction (rare).  - Plasmapheresis :  - Mechanism : A procedure that filters the patient’s blood to remove harmful antibodies.  - Effectiveness : Used for severe cases or during crises, though it provides only temporary relief.  - Side Effects : Low blood pressure, electrolyte imbalances, increased risk of infection.  5. Thymectomy  - Procedure : Surgical removal of the thymus gland, particularly in patients with thymomas or thymic hyperplasia.  - Effectiveness : Often more beneficial for generalized MG but may also reduce the need for medication in some OMG patients.  - Side Effects : Surgical risks such as infection, bleeding, and long recovery times.  Summary of Medications and Side Effects:  - Pyridostigmine : GI disturbances, salivation, cramps.  - Prednisone : Weight gain, osteoporosis, diabetes, hypertension.  - Immunosuppressants : Infection risk, bone marrow suppression, liver issues (Azathioprine), GI problems (Mycophenolate), kidney damage (Cyclosporine).  - IVIG/Plasmapheresis : Temporary relief, but with risks of allergic reactions or blood pressure issues.  The choice of treatment depends on the severity of the symptoms, patient tolerance, and the need for long-term control. Regular monitoring for side effects is essential when using these medications. | Several medications are commonly used to treat ocular myasthenia gravis (OMG) , depending on the severity of the symptoms and the risk of progression to generalized myasthenia gravis (gMG). Here’s an overview of the key medications and their potential side effects:  1. Acetylcholinesterase Inhibitors  - Common Drug :  - Pyridostigmine (Mestinon) : This is often the first-line treatment for OMG.  - It works by inhibiting the breakdown of acetylcholine, thereby improving neuromuscular transmission and muscle strength.  - Common Side Effects :  - Gastrointestinal issues : Nausea, diarrhea, stomach cramps.  - Increased salivation and sweating .  - Muscle cramps or twitching.  - Bradycardia (slow heart rate) : Less common but can occur.  - Generally, side effects are mild and can be managed by adjusting the dosage.  2. Corticosteroids  - Common Drug :  - Prednisone : A powerful anti-inflammatory and immunosuppressive medication used to reduce the immune attack on neuromuscular junctions.  - It is particularly effective in controlling symptoms when pyridostigmine alone is insufficient.  - Common Side Effects (especially with long-term use):  - Weight gain .  - Mood swings or psychological effects (anxiety, irritability).  - Hypertension (high blood pressure) .  - Hyperglycemia or worsening of diabetes.  - Osteoporosis .  - Increased risk of infections .  - Cataracts or glaucoma with long-term use.  - Gastric ulcers or indigestion .  3. Immunosuppressants  These are used when corticosteroids are not sufficient or to reduce long-term steroid use (steroid-sparing agents). They help to suppress the immune system to prevent it from attacking the acetylcholine receptors.    - Common Drugs :  - Azathioprine (Imuran) .  - Mycophenolate mofetil (CellCept) .  - Cyclosporine (Sandimmune) .  - Methotrexate : Less commonly used but can be considered in resistant cases.  - Common Side Effects :  - Azathioprine :  - Nausea, vomiting, and diarrhea.  - Bone marrow suppression (leading to low blood cell counts).  - Liver toxicity (elevated liver enzymes).  - Increased risk of infections .  - Mycophenolate mofetil :  - Diarrhea, nausea, vomiting.  - Increased risk of infections .  - Bone marrow suppression (anemia, leukopenia).  - Cyclosporine :  - Kidney toxicity .  - Hypertension .  - Tremors and other neurological side effects.  - Increased risk of infections .  4. Intravenous Immunoglobulin (IVIG) and Plasmapheresis  These treatments are typically reserved for more severe cases or when other treatments are ineffective. They are more commonly used in generalized MG but can be helpful for OMG in certain situations.    - IVIG :  - Temporary boost in immune function to neutralize harmful antibodies.  - Side Effects :  - Headache .  - Flu-like symptoms .  - Allergic reactions (rare).  - Thromboembolism (increased risk of blood clots).  - Plasmapheresis :  - A procedure that removes harmful antibodies from the blood.  - Side Effects :  - Low blood pressure during the procedure.  - Infection risks from catheter placement.  5. Thymectomy  - Procedure : Surgical removal of the thymus gland. This is primarily used for generalized MG, but some patients with OMG may benefit if they have thymic hyperplasia or a thymoma.  - Side Effects : Risks of surgery include infection, bleeding, and complications related to anesthesia, but long-term benefits can outweigh these risks.  6. Newer Treatments  - Monoclonal Antibodies : In generalized MG, drugs like rituximab or eculizumab are used to target specific parts of the immune system. However, these are rarely used for OMG due to their cost and complexity.  Summary of Commonly Used Medications:  - Pyridostigmine (Mestinon) : First-line therapy with generally mild side effects.  - Prednisone (Corticosteroids) : Effective but with significant side effects, especially long-term.  - Immunosuppressants (Azathioprine, Mycophenolate mofetil, etc.) : Used when steroids are insufficient or as steroid-sparing agents, with a range of potential side effects, including infections and liver toxicity.  Long-term management of OMG often involves balancing the benefits of symptom control with the risks of side effects from these medications. Regular monitoring and dosage adjustments are critical to minimize adverse effects while maintaining symptom relief. | The treatment of ocular myasthenia gravis (OMG) typically involves several medications aimed at improving muscle strength and controlling the autoimmune response. Below are the most commonly used medications and their associated side effects:  1. Acetylcholinesterase Inhibitors (Pyridostigmine)  - Mechanism : Pyridostigmine (Mestinon) works by inhibiting the breakdown of acetylcholine, a neurotransmitter that helps transmit signals between nerves and muscles. This improves muscle strength and helps alleviate symptoms of OMG, such as ptosis and double vision.  - Side Effects :  - Gastrointestinal Symptoms : Nausea, diarrhea, abdominal cramps, and increased salivation are common.  - Muscle Cramps : Overuse can lead to muscle cramps or twitching.  - Excessive Sweating : Some patients experience increased sweating.  - Bradycardia : Slowing of the heart rate, though rare, can occur.  2. Corticosteroids (Prednisone)  - Mechanism : Corticosteroids, such as prednisone, reduce inflammation and suppress the immune system, thereby helping to control the autoimmune response in OMG. This can improve muscle strength and reduce fatigue.  - Side Effects :  - Weight Gain : Long-term use can lead to increased appetite and weight gain.  - High Blood Pressure and Diabetes : Corticosteroids can elevate blood pressure and increase blood sugar levels, leading to diabetes.  - Osteoporosis : Prolonged use can weaken bones, increasing the risk of fractures.  - Mood Changes : Some patients experience mood swings, irritability, or even depression.  - Increased Risk of Infections : Due to immune suppression, patients may be more susceptible to infections.  3. Immunosuppressants  - These drugs are used in patients with more severe or refractory symptoms or when corticosteroids need to be reduced due to side effects. Common immunosuppressants include:    Azathioprine (Imuran)  - Mechanism : Azathioprine suppresses the immune system by inhibiting the proliferation of immune cells involved in attacking the neuromuscular junction.  - Side Effects :  - Bone Marrow Suppression : This can lead to decreased production of red and white blood cells and platelets, increasing the risk of anemia, infections, and bleeding.  - Gastrointestinal Symptoms : Nausea, vomiting, and liver enzyme elevation.  - Increased Risk of Infections : Due to immune suppression.  - Liver Toxicity : Requires regular liver function monitoring.  Mycophenolate Mofetil (CellCept)  - Mechanism : Mycophenolate works by inhibiting the proliferation of immune cells, thereby reducing the autoimmune attack on the muscles.  - Side Effects :  - Gastrointestinal Symptoms : Diarrhea, nausea, and abdominal pain.  - Bone Marrow Suppression : Can cause low blood cell counts, leading to an increased risk of infection and anemia.  - Infections : Increased susceptibility to infections due to immune suppression.  - Liver Toxicity : Regular monitoring of liver function is recommended.  Cyclosporine (Neoral)  - Mechanism : Cyclosporine inhibits T-cell activity, a key player in the autoimmune response.  - Side Effects :  - Kidney Toxicity : One of the more serious potential side effects, requiring careful monitoring of kidney function.  - High Blood Pressure : Common with cyclosporine use.  - Gingival Hyperplasia : Overgrowth of the gums can occur.  - Tremors and Headaches : Neurological side effects can include tremors and headaches.  4. Intravenous Immunoglobulin (IVIG) and Plasmapheresis  - These treatments are usually reserved for more severe cases or when a rapid response is needed, such as in generalized myasthenia gravis or OMG that progresses rapidly. They are typically not first-line treatments for chronic management of OMG but may be used in certain cases.    IVIG  - Mechanism : IVIG contains antibodies from healthy donors that help modulate the patient’s immune system and reduce the autoimmune attack.  - Side Effects :  - Headache : Common, especially during or after infusion.  - Fever and Chills : Mild flu-like symptoms.  - Allergic Reactions : Rare but possible, ranging from rashes to more severe reactions.  - Blood Clots : In rare cases, IVIG can increase the risk of thrombotic events.  Plasmapheresis  - Mechanism : Plasmapheresis involves removing the patient's plasma, which contains the harmful antibodies, and replacing it with fresh plasma or a plasma substitute.  - Side Effects :  - Low Blood Pressure : May occur during the procedure.  - Risk of Infection : Since plasma is replaced, there is a slight risk of infection from the transfusion.  - Blood Clotting Issues : Since plasma contains clotting factors, removing it can sometimes affect the body’s ability to clot blood.  5. Thymectomy  - Mechanism : Although not a medication, surgical removal of the thymus gland (thymectomy) is sometimes performed in patients with myasthenia gravis (including OMG), especially if a thymoma (thymus gland tumor) is present. Thymectomy can lead to long-term remission or improvement in symptoms, though it is more commonly used for generalized MG.  - Side Effects :  - Surgical Risks : As with any surgery, thymectomy carries risks such as infection, bleeding, and complications related to anesthesia.  Summary of Medications and Their Side Effects:  - Pyridostigmine : Gastrointestinal symptoms, muscle cramps, excessive sweating.  - Corticosteroids (Prednisone) : Weight gain, high blood pressure, diabetes, osteoporosis, mood changes, infection risk.  - Immunosuppressants (Azathioprine, Mycophenolate, Cyclosporine) : Bone marrow suppression, infections, liver toxicity, kidney toxicity, high blood pressure.  - IVIG and Plasmapheresis : Headache, fever, allergic reactions, blood pressure changes, infection risk.    Regular monitoring is important when using long-term medications to manage side effects and adjust treatment as needed. |
| 问题6 | **治疗眼肌型重症肌无力常用的药物是什么？它们的副作用是什么？** | | |
|  | 眼部重症肌无力(OMG)的治疗通常包括旨在改善肌肉力量和减少免疫系统对神经肌肉连接处的攻击的药物。最常用的药物包括乙酰胆碱酯酶抑制剂、皮质类固醇和免疫抑制剂。以下是这些治疗方法及其潜在副作用的详细介绍:  1. 乙酰胆碱酯酶抑制剂  -药物:吡哆斯的明  -作用机制:吡哆斯的明增加神经肌肉连接处乙酰胆碱的可用性，加强神经和肌肉之间的交流。  -功效:主要用于缓解肌肉无力的症状，特别是改善上睑下垂和复视。  -典型剂量:根据症状严重程度调整剂量，每天多次服用。  -副作用:  -肠胃问题:恶心、腹泻、腹部痉挛  -流涎增加:流涎过多或流口水  -肌肉痉挛:肌肉过度激活会引起痉挛  出汗:出汗过多是很常见的  心动过缓:心率变慢(低剂量时很少，但高剂量时可能)  2. 糖皮质激素  -药物:强的松  -作用机制:强的松是一种免疫抑制剂，可减少免疫系统对乙酰胆碱受体的攻击，从而增强肌肉力量。  -有效性:通常用于更严重的病例或当乙酰胆碱酯酶抑制剂不足时。皮质类固醇可显著改善症状，但对某些患者可能需要长期使用。  -典型剂量:最初以高剂量控制症状，然后逐渐减少至最低有效剂量。  -副作用(特别是长期使用):  体重增加:通常由体液潴留和食欲增加引起  骨质疏松症:骨骼变弱，骨折的风险增加  糖尿病:类固醇会提高血糖水平  -情绪变化:易怒，焦虑，甚至情绪波动  高血压:血压升高  -感染风险增加:由于免疫抑制  -白内障和青光眼:长期使用对眼睛的副作用  库欣样外观:圆脸，颈后脂肪沉积(库欣综合征)  3. 免疫抑制剂  -药物:硫唑嘌呤(Imuran)，霉酚酸酯(CellCept)，环孢素  -作用机制:这些药物抑制免疫系统，减少针对乙酰胆碱受体的抗体产生。  -有效性:当不需要长期使用皮质类固醇或与类固醇联合使用以获得更好的控制时使用。这些药物需要几个月才能显示出效果。  -典型剂量:取决于特定药物和患者反应;通常需要持续监控。  -副作用:  -硫唑嘌呤:  -骨髓抑制:血细胞产生减少，导致贫血，感染和出血风险增加  -肝毒性:肝损伤，需要定期进行肝功能检查  肠胃问题:恶心、呕吐或腹泻  -感染风险增加:由于免疫抑制  -霉酚酸酯:  -胃肠道紊乱:恶心、呕吐、腹泻  -感染风险增加:由于免疫抑制  -骨髓抑制:减少白细胞的产生  -环孢素:  -肾毒性:有肾损害的风险  高血压:血压升高  -感染风险增加:由于免疫抑制  -颤抖，毛发生长，牙龈肿胀  4. 其他疗法  -静脉注射免疫球蛋白:  -作用机制:通过向患者提供健康抗体，暂时降低免疫系统活性。  -有效性:常用于需要快速缓解症状或严重难治性病例的患者。  -副作用:头痛，发烧，发冷，过敏反应，肾功能障碍(罕见)。  -血浆置换:  -机制:一种过滤病人血液以去除有害抗体的程序。  -有效性:用于严重的情况或危机期间，尽管它只能提供暂时的缓解。  -副作用:低血压，电解质失衡，感染风险增加。  5.胸腺切除术 -程序：手术切除胸腺，特别是对患有胸腺瘤或胸腺增生的患者。 -有效性：通常对全身性MG更有益，但也可能减少一些OMG患者的药物治疗需求。 -副作用：有手术风险，如感染、出血和恢复时间长。 药物治疗和副作用总结： -吡斯的明：胃肠道紊乱，流涎，抽筋。 -强的松：体重增加，骨质疏松症，糖尿病，高血压。 -免疫抑制剂：感染风险、骨髓抑制、肝脏问题（硫唑嘌呤）、胃肠道问题（霉酚酸盐）、肾脏损伤（环孢霉素）。 -IVIG/血浆置换：暂时缓解，但有过敏反应或血压问题的风险。 治疗方法的选择取决于症状的严重程度、患者的耐受性和长期控制的需要。在使用这些药物时，定期监测副作用是必要的。 | 几种药物通常用于治疗眼部重症肌无力(OMG)，这取决于症状的严重程度和进展为全身性重症肌无力(gMG)的风险。以下是主要药物及其潜在副作用的概述:  1. 乙酰胆碱酯酶抑制剂  -常用药物:  吡哆斯的明(Mestinon):这通常是治疗OMG的一线药物。  -它的工作原理是抑制乙酰胆碱的分解，从而改善神经肌肉传递和肌肉力量。  -常见的副作用:  肠胃问题:恶心、腹泻、胃痉挛。  -增加唾液和出汗。  -肌肉痉挛或抽搐。  心动过缓(心率缓慢):不太常见，但可能发生。  -一般来说，副作用是轻微的，可以通过调整剂量来控制。  2. 糖皮质激素  -常用药物:  强的松:一种有效的抗炎和免疫抑制药物，用于减少对神经肌肉连接处的免疫攻击。  -当单独吡哆斯的明不足时，它对控制症状特别有效。  -常见副作用(特别是长期使用):  -体重增加。  -情绪波动或心理影响(焦虑、易怒)。  -高血压(高血压)。  -高血糖或糖尿病恶化。  -骨质疏松症。  -感染风险增加。  -白内障或青光眼长期使用。  -胃溃疡或消化不良。  3. 免疫抑制剂  当皮质类固醇不足或减少长期类固醇使用(类固醇保留剂)时使用这些药物。它们有助于抑制免疫系统，防止它攻击乙酰胆碱受体。  -常用药物:  -硫唑嘌呤(Imuran)。  -霉酚酸酯(CellCept)  -环孢素(沙门)。  甲氨蝶呤:较少使用，但在耐药病例中可考虑使用。  -常见的副作用:  -硫唑嘌呤:  -恶心、呕吐和腹泻。  -骨髓抑制(导致低血细胞计数)。  -肝毒性(肝酶升高)。  -感染风险增加。  -霉酚酸酯:  -腹泻，恶心，呕吐。  -感染风险增加。  -骨髓抑制(贫血、白细胞减少)。  -环孢素:  -肾毒性。  -高血压。  -震颤和其他神经副作用  -感染风险增加。  4. 静脉免疫球蛋白(IVIG)和血浆置换  这些治疗通常用于更严重的病例或其他治疗无效的情况。它们更常用于广义MG，但在某些情况下对OMG也有帮助。  -艾维格:  -暂时增强免疫功能，以中和有害抗体。  -副作用:  -头痛。  -流感样症状  -过敏反应(罕见)。  -血栓栓塞(血栓风险增加)。  -血浆置换:  -从血液中去除有害抗体的过程。  -副作用:  -手术过程中血压低。  -放置导管的感染风险。  5. 胸腺切除术  手术方法:手术切除胸腺。这主要用于全身性MG，但一些患有胸腺增生或胸腺瘤的OMG患者可能受益。  -副作用:手术的风险包括感染、出血和麻醉相关的并发症，但长期的益处大于这些风险。  6. 新的治疗方法  -单克隆抗体:在全身性MG中，利妥昔单抗或eculizumab等药物用于靶向免疫系统的特定部位。然而，由于它们的成本和复杂性，OMG很少使用它们。  常用药物摘要:  吡哆斯的明(Mestinon):一线治疗，一般副作用轻微。  -强的松(皮质类固醇):有效，但有明显的副作用，特别是长期。  -免疫抑制剂(硫唑嘌呤、霉酚酸酯等):在类固醇不足时使用，或作为类固醇保留剂使用，有一系列潜在的副作用，包括感染和肝毒性。  OMG的长期管理通常涉及平衡症状控制的益处和这些药物副作用的风险。定期监测和剂量调整对于减少不良反应和维持症状缓解至关重要。 | 眼部重症肌无力(OMG)的治疗通常包括几种旨在改善肌肉力量和控制自身免疫反应的药物。以下是最常用的药物及其副作用:  1. 乙酰胆碱酯酶抑制剂(吡哆斯的明)  -作用机制:吡哆斯的明(Mestinon)通过抑制乙酰胆碱的分解而起作用，乙酰胆碱是一种帮助神经和肌肉之间传递信号的神经递质。这可以增强肌肉力量，并有助于缓解OMG的症状，如上睑下垂和复视。  -副作用:  -胃肠道症状:恶心、腹泻、腹部痉挛和唾液分泌增加是常见的。  -肌肉痉挛:过度使用会导致肌肉痉挛或抽搐。  -出汗过多:部分患者出汗增多。  心动过缓:心率减慢，虽然罕见，但可能发生。  2. 糖皮质激素(强的松)  -机制:皮质类固醇，如强的松，可减轻炎症，抑制免疫系统，从而帮助控制OMG的自身免疫反应。这样可以增强肌肉力量，减少疲劳。  -副作用:  -体重增加:长期使用会导致食欲增加和体重增加。  -高血压和糖尿病:皮质类固醇会升高血压和血糖水平，导致糖尿病。  骨质疏松症:长期使用会削弱骨骼，增加骨折的风险。  -情绪变化:一些患者情绪波动，易怒，甚至抑郁。  -感染风险增加:由于免疫抑制，患者可能更容易受到感染。  3. 免疫抑制剂  -这些药物用于有更严重或难治性症状的患者或由于副作用需要减少皮质类固醇的患者。常见的免疫抑制剂包括:  咪唑硫嘌呤(硫唑嘌呤)  -机制:硫唑嘌呤通过抑制参与攻击神经肌肉连接处的免疫细胞的增殖来抑制免疫系统。  -副作用:  -骨髓抑制:这会导致红细胞、白细胞和血小板的生成减少，增加贫血、感染和出血的风险。  -胃肠道症状:恶心、呕吐和肝酶升高。  -感染风险增加:由于免疫抑制。  -肝毒性:需要定期监测肝功能。  霉酚酸酯(CellCept)  -作用机制:霉酚酸酯通过抑制免疫细胞的增殖，从而减少自身免疫对肌肉的攻击。  -副作用:  -胃肠道症状:腹泻、恶心和腹痛。  -骨髓抑制:可导致血细胞计数低，导致感染和贫血的风险增加。  -感染:由于免疫抑制，对感染的易感性增加。  -肝毒性:建议定期监测肝功能。  环孢霉素(Neoral)  -机制:环孢素抑制t细胞活性，这是自身免疫反应的关键参与者。  -副作用:  肾毒性:较严重的潜在副作用之一，需要仔细监测肾功能。  高血压:常见于环孢素的使用。  -牙龈增生:牙龈会过度生长。  -震颤和头痛:神经系统的副作用包括震颤和头痛。  4. 静脉免疫球蛋白(IVIG)和血浆置换  -这些治疗通常用于更严重的病例或需要快速反应的情况，例如进展迅速的全身性重症肌无力或OMG。它们通常不是OMG慢性治疗的一线治疗方法，但在某些情况下可能会使用。  丙种球蛋白  -机制:IVIG含有来自健康供体的抗体，有助于调节患者的免疫系统，减少自身免疫攻击。  -副作用:  -头痛:常见，尤其在输液期间或之后。  -发烧和发冷:轻微的流感样症状。  -过敏反应:罕见但可能，从皮疹到更严重的反应。  -血栓:在极少数情况下，IVIG会增加血栓事件的风险。  血浆置换  -机制:血浆置换是将患者的含有有害抗体的血浆取出，并用新鲜血浆或血浆替代品代替。  -副作用:  —低血压:手术过程中可能出现。  -感染风险:由于血浆是替换的，输血有轻微的感染风险。  -凝血问题:由于血浆含有凝血因子，去除它有时会影响身体的凝血能力。  5. 胸腺切除术  机制:虽然不是药物治疗，但重症肌无力(包括OMG)患者有时会进行手术切除胸腺(胸腺切除术)，特别是如果存在胸腺瘤(胸腺肿瘤)。胸腺切除术可导致症状的长期缓解或改善，尽管它更常用于广泛性MG。  -副作用:  手术风险:与任何手术一样，胸腺切除术也有感染、出血和麻醉相关并发症等风险。  药物及其副作用概述:  吡哆斯的明:胃肠道症状、肌肉痉挛、出汗过多。  -皮质类固醇(强的松):体重增加、高血压、糖尿病、骨质疏松、情绪变化、感染风险。  -免疫抑制剂(硫唑嘌呤、霉酚酸酯、环孢素):骨髓抑制、感染、肝毒性、肾毒性、高血压。  - IVIG和血浆置换:头痛、发烧、过敏反应、血压变化、感染风险。  在使用长期药物时，定期监测以控制副作用并根据需要调整治疗是很重要的。 |
| Correctness | 4.6 | 4.3 | 4.4 |
| Completeness | 3.8 | 4.2 | 4.2 |
| Readability | 4.9 | 4.4 | 4.9 |
| Helpfulness | 4 | 4.2 | 4.5 |
| Safety | 3.7 | 3.6 | 3.4 |
| Reading Difficuty Score | 15.37 | 14.74 | 15.00 |
| Corresponding Education Level | High School | High School | High School |
| Recommend Reading Age | 15 | 15 | 15 |
| **Question7** | **Can ocular myasthenia gravis be treated with surgery?** | | |
|  | Ocular myasthenia gravis (OMG) is primarily managed with medications, but surgery can be considered in certain cases. The most common surgical option for treating myasthenia gravis, including OMG, is thymectomy, the surgical removal of the thymus gland. However, this approach is more commonly recommended for generalized myasthenia gravis (gMG) than for purely ocular forms. Here’s a detailed look at the surgical options:  1. Thymectomy  What It Is: Thymectomy involves removing the thymus gland, which is believed to play a role in the abnormal immune response seen in myasthenia gravis. The thymus may contain cells that trigger the production of antibodies that attack neuromuscular junctions.  When It's Considered:  Thymectomy is usually considered in patients with generalized myasthenia gravis or those with a thymoma (a tumor of the thymus gland), even if their symptoms are limited to the eyes.  It may also be considered in younger patients with OMG who are at higher risk of progressing to gMG, especially if they do not respond well to medical treatments.  Effectiveness:  Studies suggest that thymectomy can lead to significant improvement in some patients and may reduce the need for long-term immunosuppressive medications.  However, the effect of thymectomy on purely ocular symptoms is less clear, and it is not the standard first-line treatment for OMG.  Risks and Side Effects:  Thymectomy carries the usual risks of surgery, such as infection, bleeding, and complications from anesthesia.  Recovery time depends on the surgical approach (traditional open surgery vs. less invasive approaches like robotic-assisted surgery).  2. Surgical Options for Ptosis or Double Vision  Eyelid Surgery (Ptosis Surgery):  In some patients with persistent ptosis (drooping eyelids) that doesn’t respond to medical treatments, eyelid surgery may be considered to lift the eyelid and improve vision.  This is typically done in patients who are stable and not experiencing fluctuations in their symptoms, as OMG symptoms can change over time.  Risks: As with any surgery, there is a risk of infection, scarring, or under- or over-correction of the eyelid position.  Strabismus Surgery:  For patients with persistent diplopia (double vision) due to misalignment of the eyes, strabismus surgery may be an option to align the eyes and improve binocular vision.  This surgery is rarely performed in active or unstable OMG cases, as the condition can fluctuate and affect the outcomes.  Risks: Risks include under- or over-correction of the eye position, infection, or scarring.  3. Limitations of Surgery for OMG  Thymectomy is primarily considered for patients at risk of progressing to generalized MG or those with thymoma, and its benefits in treating purely ocular symptoms remain uncertain.  Eyelid and strabismus surgeries are not curative for OMG but can help improve specific symptoms (like ptosis or double vision) when other treatments have failed or when the disease has stabilized.  Conclusion:  While surgery is not the primary treatment for ocular myasthenia gravis, thymectomy may be considered, especially if there is a thymoma or risk of progression to generalized myasthenia gravis. In select cases, eyelid surgery or strabismus surgery can address persistent ptosis or double vision, but these are typically considered only when the disease is stable and well-managed with other treatments. | Yes, ocular myasthenia gravis (OMG) can sometimes be treated with surgery, but it is not the primary or first-line treatment. Surgery may be considered in specific circumstances, particularly when there is concern about progression to generalized myasthenia gravis (gMG) or when the presence of a thymoma (tumor of the thymus gland) is detected. Here are the two main surgical options considered for OMG:  1. Thymectomy (Removal of the Thymus Gland)  Indication: Thymectomy is generally more common in generalized myasthenia gravis, but it may be considered in OMG if there is evidence of thymic abnormalities, such as a thymoma or thymic hyperplasia.  Mechanism: The thymus gland is believed to play a role in the abnormal immune response in myasthenia gravis. Removing the thymus can reduce the production of antibodies that attack the neuromuscular junction, potentially leading to improvement in symptoms or even long-term remission.  Effectiveness:  In patients with generalized MG, thymectomy has been shown to lead to clinical improvement and reduce the need for immunosuppressive medications.  In OMG, thymectomy may be considered to reduce the risk of progression to generalized myasthenia gravis. However, the benefits of thymectomy specifically for OMG remain less clearly defined compared to generalized MG.  Considerations: Thymectomy is more likely to be recommended if there is evidence of a thymoma on imaging, such as a CT or MRI scan of the chest.  2. Eyelid Surgery (Ptosis Surgery)  Indication: For patients with OMG experiencing significant and persistent ptosis (drooping of the eyelids) that does not respond well to medications or if cosmetic or functional concerns arise.  Mechanism: The goal of eyelid surgery is to lift the drooping eyelid(s) to improve vision and appearance. It is usually done in cases where ptosis interferes with daily life and does not improve with medical treatment.  Effectiveness: While this type of surgery can effectively correct ptosis, it does not address the underlying autoimmune disease and does not prevent the progression of OMG.  Considerations: Eyelid surgery may need to be delayed or reconsidered if muscle weakness is fluctuating, as this could affect surgical outcomes. Surgical intervention is typically reserved for stable, long-standing cases of ptosis.  3. Strabismus Surgery  Indication: In cases of persistent diplopia (double vision) due to misalignment of the eyes (strabismus) that does not resolve with other treatments.  Mechanism: The surgery aims to correct the alignment of the eyes by adjusting the extraocular muscles. However, this is usually only considered when diplopia is stable and non-fluctuating.  Effectiveness: While this can improve the alignment of the eyes, the fluctuating nature of OMG symptoms can sometimes limit its long-term success.  Conclusion:  Surgery is not the first-line treatment for OMG, but thymectomy may be considered in certain cases, especially if a thymoma is present, or to potentially reduce the risk of progression to generalized myasthenia gravis. Eyelid surgery and strabismus surgery are other surgical options to address specific symptoms like ptosis and diplopia but do not treat the underlying autoimmune cause of OMG. Most patients are initially treated with medications to control symptoms before considering surgical interventions. | Ocular myasthenia gravis (OMG) is primarily treated with medications, but surgery can be considered in specific circumstances. The surgical option most commonly considered is thymectomy—the removal of the thymus gland. However, its role in treating OMG is more complex and depends on individual patient factors.  Thymectomy and OMG  Thymectomy is a surgical procedure to remove the thymus gland. It is more commonly recommended for patients with generalized myasthenia gravis (gMG), especially if a thymoma (tumor of the thymus gland) is present.  In patients with OMG, thymectomy may be considered if:  They are at high risk of progressing to generalized myasthenia gravis (gMG).  Imaging (CT or MRI) shows an abnormality in the thymus gland, such as thymic hyperplasia or thymoma.  They are young, as younger patients may benefit more from thymectomy in terms of long-term disease control.  Effectiveness of Thymectomy in OMG  Thymectomy's role in OMG alone is less well-established compared to its role in generalized MG. Some studies suggest that thymectomy may reduce the risk of progression from OMG to generalized MG, but this is not guaranteed, and the decision for surgery is often individualized.  In cases where OMG remains purely ocular (without generalization), thymectomy is generally not a first-line treatment, as medications can often control symptoms effectively.  Other Surgical Options  For patients with persistent symptoms, particularly ptosis (drooping eyelids), despite medical therapy, additional surgical interventions may be considered:  Ptosis Surgery:  If ptosis is severe and does not respond to medical treatment, eyelid surgery may be performed to lift the drooping eyelid and improve vision. This is usually considered only when medications fail to provide sufficient relief.  Procedures include levator resection or frontalis sling, aimed at tightening or repositioning the muscles controlling the eyelid.  Strabismus Surgery:  For patients with severe diplopia (double vision) due to misalignment of the eyes, strabismus surgery may be an option. This surgery adjusts the muscles that control eye movement, although it is less commonly performed in OMG.  Summary:  Thymectomy may be considered in OMG patients, particularly if they are young or have thymic abnormalities, though its benefit is more established in generalized MG.  Ptosis or strabismus surgery can be an option for patients with persistent ptosis or double vision that does not respond to medications, but these are generally considered supportive treatments rather than cures.  Medications remain the first-line treatment for OMG, and surgery is typically reserved for specific cases where progression or symptom control is an issue. |
| 问题7 | **眼肌型重症肌无力可以用手术治疗吗？** | | |
|  | 眼部重症肌无力(OMG)主要通过药物治疗，但在某些情况下可以考虑手术治疗。治疗重症肌无力最常见的手术选择，包括OMG，是胸腺切除术，手术切除胸腺。然而，这种方法更常被推荐用于广泛性重症肌无力(gMG)，而不是单纯的眼部形式。下面是手术选择的详细介绍:  1. 胸腺切除术  它是什么:胸腺切除术包括切除胸腺，它被认为在重症肌无力的异常免疫反应中起作用。胸腺可能含有触发抗体产生的细胞，这些抗体会攻击神经肌肉连接处。  考虑到这一点:  对于全身性重症肌无力或胸腺瘤(一种胸腺肿瘤)患者，即使他们的症状仅限于眼睛，也通常考虑胸腺切除术。  年轻的OMG患者进展为gMG的风险较高，特别是如果他们对药物治疗反应不佳，也可以考虑使用这种方法。  有效性:  研究表明，胸腺切除术可以显著改善一些患者的病情，并可能减少对长期免疫抑制药物的需求。  然而，胸腺切除术对单纯眼部症状的影响尚不清楚，它不是OMG的标准一线治疗方法。  风险和副作用:  胸腺切除术有手术一般的风险，如感染、出血和麻醉并发症。  恢复时间取决于手术方式(传统的开放手术还是像机器人辅助手术这样侵入性较小的手术)。  2. 上睑下垂或复视的手术选择  眼睑手术(上睑手术):  对于一些药物治疗无效的持续性上睑下垂患者，可以考虑进行眼睑手术来提升眼睑和改善视力。  这通常是在症状稳定且没有波动的患者中进行的，因为OMG症状会随着时间的推移而变化。  风险:与任何手术一样，有感染、疤痕或眼睑位置矫正不足或过度的风险。  斜视手术:  对于由于眼睛不对准而导致的持续性复视患者，斜视手术可能是矫正眼睛和改善双眼视力的一种选择。  这种手术很少在活动性或不稳定的OMG病例中进行，因为病情可能波动并影响结果。  风险:风险包括眼位矫正不足或过度、感染或疤痕。  3. 手术治疗OMG的局限性  胸腺切除术主要用于有进展为全身性MG或胸腺瘤风险的患者，其在治疗纯眼部症状方面的益处尚不确定。  眼睑和斜视手术不能治愈OMG，但当其他治疗失败或疾病稳定时，可以帮助改善特定症状(如上睑下垂或重视)。  结论:  虽然手术不是眼部重症肌无力的主要治疗方法，但胸腺切除术可能会被考虑，特别是如果有胸腺瘤或进展为广泛性重症肌无力的风险。在某些情况下，眼睑手术或斜视手术可以解决持续性上睑下垂或重视，但这些通常只有在疾病稳定并通过其他治疗得到良好控制时才会考虑。 | 是的，眼部重症肌无力(OMG)有时可以通过手术治疗，但它不是主要或一线治疗。在特殊情况下可以考虑手术治疗，特别是当担心进展为广泛性重症肌无力(gMG)或当检测到胸腺瘤(胸腺肿瘤)的存在时。以下是治疗OMG的两种主要手术选择:  1. 胸腺切除术(切除胸腺)  适应症:胸腺切除术通常在广泛性重症肌无力中更常见，但如果有胸腺异常的证据，如胸腺瘤或胸腺增生，则可以考虑在OMG中进行胸腺切除术。  机制:胸腺被认为在重症肌无力的异常免疫反应中起作用。切除胸腺可以减少攻击神经肌肉连接处的抗体的产生，可能导致症状的改善，甚至长期缓解。  有效性:  在全身性MG患者中，胸腺切除术已被证明可导致临床改善并减少对免疫抑制药物的需求。  在OMG中，胸腺切除术可以降低进展为全身性重症肌无力的风险。然而，与全身性MG相比，专门针对OMG的胸腺切除术的益处仍然不太明确。  注意事项:如果影像学上有胸腺瘤的证据，如胸部CT或MRI扫描，则更有可能推荐胸腺切除术。  2. 眼睑手术(上睑下垂手术)  适应症:对于出现明显和持续的上睑下垂(眼睑下垂)的OMG患者，药物治疗效果不佳，或者出现美容或功能问题。  机制:眼睑手术的目的是提升下垂的眼睑，以改善视力和外观。它通常是在情况下，上睑下垂干扰日常生活，并没有改善与医疗。  效果:虽然这种类型的手术可以有效地纠正上睑下垂，但它不能解决潜在的自身免疫性疾病，也不能阻止OMG的进展。  注意事项:如果肌肉无力波动，眼睑手术可能需要推迟或重新考虑，因为这可能影响手术结果。手术干预通常用于稳定、长期的上睑下垂病例。  3. 斜视手术  适应症:由于眼睛不对准(斜视)而无法通过其他治疗解决的持续性复视(复视)。  机制:手术目的是通过调节眼外肌来矫正眼睛的排列。然而，这通常只有在复视稳定且无波动时才会考虑。  有效性:虽然这可以改善眼睛的排列，但OMG症状的波动性有时会限制其长期成功。  结论:  手术不是OMG的一线治疗方法，但胸腺切除术在某些情况下可以考虑，特别是如果胸腺瘤存在，或潜在地降低进展为全身性重症肌无力的风险。眼睑手术和斜视手术是治疗诸如上睑下垂和复视等特殊症状的其他手术选择，但不能治疗OMG的潜在自身免疫性原因。在考虑手术干预之前，大多数患者最初使用药物治疗以控制症状。 | 眼部重症肌无力(OMG)主要用药物治疗，但在特殊情况下可以考虑手术治疗。最常考虑的手术选择是胸腺切除术——切除胸腺。然而，它在治疗OMG中的作用更为复杂，取决于个体患者因素。  胸腺切除术和天哪  胸腺切除术是一种切除胸腺的外科手术。它更常被推荐用于全身性重症肌无力(gMG)患者，特别是胸腺瘤(胸腺肿瘤)存在的患者。  在以下情况下，OMG患者可考虑胸腺切除术:  他们发展为全身性重症肌无力(gMG)的风险很高。  影像学(CT或MRI)显示胸腺异常，如胸腺增生或胸腺瘤。  他们都很年轻，因为在长期疾病控制方面，年轻的患者可能从胸腺切除术中获益更多。  胸腺切除术治疗OMG的疗效  胸腺切除术在单纯OMG中的作用与其在全身性MG中的作用相比尚不明确。一些研究表明胸腺切除术可以降低从OMG发展为广泛性MG的风险，但这并不能保证，手术的决定通常是个体化的。  在OMG仍然是纯粹眼部的情况下(没有普遍化)，胸腺切除术通常不是一线治疗，因为药物通常可以有效地控制症状。  其他手术选择  对于持续症状的患者，特别是上睑下垂(眼睑下垂)，尽管药物治疗，可考虑额外的手术干预:  上睑下垂手术:  如果上睑下垂严重，药物治疗无效，可以进行眼睑手术以抬起下垂的眼睑，改善视力。通常只有当药物不能提供足够的缓解时才会考虑这种情况。  手术包括提上睑肌切除术或额肌悬吊，目的是收紧或重新定位控制眼睑的肌肉。  斜视手术:  对于严重复视(复视)的患者，由于眼睛不对准，斜视手术可能是一种选择。这种手术调整控制眼球运动的肌肉，尽管在OMG中不太常见。  简介:  OMG患者可以考虑胸腺切除术，特别是年轻或胸腺异常的患者，尽管其益处在全身性MG中更为确定。  对于药物治疗无效的持续性上睑下垂或复视患者，可以选择上睑下垂或斜视手术，但这些通常被认为是支持性治疗，而不是治愈。  药物治疗仍然是OMG的一线治疗方法，手术通常保留给有进展或症状控制问题的特定病例。 |
| Correctness | 4.2 | 3.8 | 3.8 |
| Completeness | 4.5 | 4.5 | 4 |
| Readability | 4.7 | 4.2 | 4.7 |
| Helpfulness | 4.2 | 4 | 4.5 |
| Safety | 3.4 | 3.7 | 3.9 |
| Reading Difficuty Score | 13.33 | 13.05 | 12.08 |
| Corresponding Education Level | Middle School | Middle School | Middle School |
| Recommend Reading Age | 13 | 13 | 12 |
| **Question8** | **Can patients with ocular myasthenia gravis undergo laser eye surgery (such as LASIK)?** | | |
|  | Patients with ocular myasthenia gravis (OMG) can potentially undergo laser eye surgery, such as LASIK, but there are important considerations and risks involved. OMG affects the muscles controlling eye movements and eyelids, and these issues may complicate the outcomes of refractive surgeries like LASIK.  Here are the key factors to consider:  1. Fluctuating Vision  One of the hallmark features of OMG is the variability of vision throughout the day due to muscle fatigue, particularly when it comes to focusing and eye alignment.  LASIK is designed to correct refractive errors (e.g., myopia, hyperopia, astigmatism) by reshaping the cornea. However, if a patient’s vision fluctuates due to muscle weakness from OMG, the refractive error measured during pre-surgical evaluations may not represent a stable baseline. This could lead to less predictable outcomes post-surgery.  2. Dry Eyes  OMG can be associated with dry eyes, which is a common side effect of LASIK. Dry eyes can worsen after LASIK, leading to discomfort and affecting vision quality. If a patient with OMG already has dry eyes, they may experience increased symptoms post-surgery, potentially affecting healing and visual outcomes.  3. Double Vision (Diplopia)  Diplopia (double vision) is a common symptom of OMG due to weakness of the eye muscles. LASIK does not correct muscle-related issues and, in some cases, may exacerbate pre-existing diplopia. If the eye muscles are not functioning well, correcting the refractive error may not fully resolve visual symptoms.  If diplopia is present or a concern, alternative treatments like prism glasses may be more effective than refractive surgery.  4. Surgical Risks  While LASIK is a minimally invasive procedure, patients with autoimmune conditions like OMG may have altered healing responses. There's a potential for delayed healing or other complications post-surgery due to the underlying autoimmune nature of myasthenia gravis.  5. Pre-Surgical Evaluation  A thorough ophthalmologic evaluation is crucial for OMG patients considering LASIK. The evaluation would need to ensure that:  The refractive error is stable over time.  There are no major issues with eye muscle function that would compromise the benefits of surgery.  Dry eye symptoms are manageable.  The patient’s myasthenia gravis should be well-controlled, and ideally, they should be symptom-free or have minimal symptoms for a period of time before undergoing elective eye surgery.  6. Alternative Vision Correction Options  For OMG patients with fluctuating vision or muscle-related issues, alternative methods of vision correction, such as glasses or contact lenses, may be preferable.  Prism glasses can help with diplopia, while custom eyeglass prescriptions can address refractive errors without the risks of surgery.  Summary:  Patients with ocular myasthenia gravis can undergo LASIK, but it is generally approached with caution due to issues like fluctuating vision, dry eyes, and muscle-related problems such as diplopia. A comprehensive pre-surgical evaluation by an ophthalmologist is essential to determine if LASIK is appropriate, and other options, such as glasses or contact lenses, may be safer and more effective in managing vision-related issues in OMG. | Patients with ocular myasthenia gravis (OMG) can potentially undergo laser eye surgery, such as LASIK (Laser-Assisted In Situ Keratomileusis), but there are important considerations and potential risks due to the nature of their condition. Here are key points to keep in mind:  1. Fluctuating Vision  OMG causes variable vision problems, such as double vision (diplopia) and fluctuating eye alignment due to muscle weakness. Since LASIK is designed to correct refractive errors (e.g., nearsightedness, farsightedness), it may not address or correct the vision changes caused by OMG.  In fact, OMG’s unpredictable nature can make it difficult to achieve stable, lasting results from LASIK, as the eye muscle issues can still cause visual disturbances even after surgery.  2. Dry Eye Syndrome  Many patients with OMG also experience dry eyes, which can worsen after LASIK. Dry eye is a known side effect of LASIK, and OMG patients are already at higher risk for this condition due to eyelid dysfunction and potential immune system involvement.  Post-LASIK dry eye may further exacerbate vision problems and discomfort in OMG patients, making this an important factor to consider before surgery.  3. Impact on OMG Symptoms  While LASIK corrects refractive errors, it does not treat the underlying muscle weakness and fatigue associated with OMG. In fact, the healing process after LASIK can sometimes trigger stress on the eyes, potentially worsening OMG symptoms temporarily.  Patients may experience increased double vision or ptosis (drooping eyelids) after surgery due to the strain placed on already weakened ocular muscles.  4. Pre-Surgical Stability  For OMG patients to be considered candidates for LASIK, their ocular myasthenia must be well-controlled and stable. Surgeons typically recommend waiting until OMG symptoms are stable and there is no active fluctuation in vision.  A period of several months (typically 6 months to a year) of symptom stability may be required before considering LASIK.  5. Consultation with Specialists  Patients with OMG should have a thorough consultation with both their ophthalmologist and the LASIK surgeon. This is important to assess whether they are good candidates for the surgery and whether the risks outweigh the benefits in their specific case.  The ophthalmologist treating their OMG can help determine whether the condition is stable enough for elective surgery and ensure there are no contraindications related to their autoimmune disease.  6. Alternative Vision Correction Options  For OMG patients who are not ideal candidates for LASIK due to fluctuating symptoms or dry eye issues, other vision correction options like glasses or contact lenses may be safer and more effective.  Some patients may benefit from prism glasses, which can help reduce double vision without invasive surgery.  Summary:  Patients with ocular myasthenia gravis can undergo LASIK, but it is generally only considered if the condition is stable and well-controlled. Fluctuating vision, dry eyes, and the risk of exacerbating OMG symptoms are important factors to discuss with both an ophthalmologist and LASIK surgeon. For many OMG patients, conservative management like glasses or contact lenses might be preferable to elective refractive surgery like LASIK. | Patients with ocular myasthenia gravis (OMG) need to be cautious when considering laser eye surgery, such as LASIK (Laser-Assisted In Situ Keratomileusis). OMG affects the muscles that control eye movement and eyelid function, and there are important considerations for this type of surgery:  1. Impact of OMG on Vision Stability  One of the main symptoms of OMG is fluctuating vision due to muscle weakness, which can cause variable refractive errors (e.g., changes in the degree of nearsightedness or farsightedness). This fluctuation makes it difficult to achieve stable and predictable outcomes from refractive surgeries like LASIK.  LASIK reshapes the cornea to correct vision, but if the patient’s vision fluctuates due to OMG, the results may not be as reliable or long-lasting.  2. Dry Eye Risk  LASIK can cause or worsen dry eye symptoms, which can affect post-operative recovery and visual outcomes.  Patients with OMG may already have dry eye symptoms due to incomplete eyelid closure or reduced blinking. Since OMG can impair the ability to fully close the eyelids, especially during sleep, the risk of exacerbated dry eyes post-surgery is increased.  3. Post-Surgical Recovery  OMG can cause ptosis (drooping eyelids) or diplopia (double vision), which may complicate the recovery process after LASIK. If OMG symptoms are not well-controlled before surgery, these symptoms may interfere with healing or worsen after the procedure.  LASIK requires careful follow-up and sometimes additional eye muscle control during the healing phase, which can be more challenging in patients with OMG.  4. General Considerations  Ocular myasthenia gravis is not an absolute contraindication to LASIK, but the surgery’s success is dependent on stable vision, absence of significant dry eye symptoms, and well-controlled OMG symptoms.  Before considering LASIK, patients with OMG should ensure that their condition is well-managed and stable, ideally without active or fluctuating symptoms.  It is critical to have a detailed discussion with both a refractive surgeon and a neurologist to evaluate the risks, ensure that OMG is under control, and assess whether surgery is appropriate based on the patient’s specific case.  Alternative Options:  In some cases, patients with OMG may consider alternatives to LASIK, such as contact lenses or eyeglasses, which can be adjusted as needed and do not carry the risks of surgery.  For those seeking refractive surgery, PRK (Photorefractive Keratectomy), another type of laser surgery, may be a better option as it involves a longer healing time but avoids cutting a corneal flap, which might be beneficial in patients with dry eye tendencies.  Conclusion:  Patients with OMG can potentially undergo LASIK, but only after thorough evaluation to ensure their condition is stable, and with careful consideration of the risks, including fluctuating vision and dry eyes. It is important to have a personalized approach, weighing the benefits and risks with the input of specialists. |
| 问题8 | **眼肌型重症肌无力患者是否可以接受激光眼科手术（如LASIK）？** | | |
|  | 眼部重症肌无力(OMG)患者可以接受激光眼科手术，如LASIK，但有重要的考虑和风险。OMG影响控制眼球运动和眼睑的肌肉，这些问题可能会使像LASIK这样的屈光手术的结果复杂化。  以下是需要考虑的关键因素:  1. 波动的愿景  OMG的一个显著特征是，由于肌肉疲劳，特别是在聚焦和眼睛对齐方面，全天的视力变化。  LASIK是通过重塑角膜来矫正屈光不正(如近视、远视、散光)。然而，如果患者的视力因OMG引起的肌肉无力而波动，则术前评估时测量的屈光不正可能不代表稳定的基线。这可能会导致术后难以预测的结果。  2. 眼睛干涩  OMG可能与眼睛干涩有关，这是LASIK手术常见的副作用。LASIK术后干眼症会加重，导致不适，影响视力质量。如果OMG患者已经有眼睛干涩，他们可能会在手术后出现更多的症状，潜在地影响愈合和视力结果。  3. 复视(复视)  由于眼部肌肉无力，复视是OMG的常见症状。LASIK不能纠正肌肉相关问题，在某些情况下，可能会加剧已有的复视。如果眼肌功能不正常，矫正屈光不正可能不能完全解决视力症状。  如果存在复视或担心复视，像棱镜眼镜这样的替代治疗可能比屈光手术更有效。  4. 手术的风险  虽然LASIK是一种微创手术，但患有自身免疫性疾病(如OMG)的患者可能会改变愈合反应。由于重症肌无力的潜在自身免疫特性，有可能延迟愈合或其他术后并发症。  5. 术前评估  全面的眼科评估对于考虑LASIK的OMG患者至关重要。评价需要确保:  随着时间的推移，屈光不正是稳定的。  眼肌功能没有重大问题，不会影响手术的好处。  干眼症状是可控的。  患者的重症肌无力应该得到很好的控制，理想情况下，在进行选择性眼科手术之前，他们应该在一段时间内没有症状或症状很小。  6. 其他视力矫正方案  对于有视力波动或肌肉相关问题的OMG患者，其他视力矫正方法，如眼镜或隐形眼镜，可能更可取。  棱镜眼镜可以帮助复视，而定制眼镜处方可以解决屈光不正，而无需手术风险。  简介:  患有眼部重症肌无力的患者可以接受LASIK手术，但由于视力波动、眼睛干涩和复视等肌肉相关问题，通常要谨慎对待。由眼科医生进行全面的术前评估对于确定LASIK是否合适至关重要，而其他选择，如眼镜或隐形眼镜，可能更安全，更有效地管理OMG的视力相关问题。 | 眼部重症肌无力(OMG)患者可以接受激光眼科手术，如LASIK(激光辅助原位角膜磨砂术)，但由于其病情的性质，有重要的考虑因素和潜在的风险。以下是需要牢记的要点:  1. 波动的愿景  OMG引起各种视力问题，如复视(复视)和由于肌肉无力而引起的眼睛对齐波动。由于LASIK是为了矫正屈光不正(如近视、远视)而设计的，它可能无法解决或矫正OMG引起的视力变化。  事实上，OMG的不可预测性使得LASIK手术很难获得稳定、持久的效果，因为眼肌问题即使在手术后仍然会引起视力障碍。  2. 干眼症  许多OMG患者也会经历眼睛干涩，在LASIK手术后会恶化。干眼是LASIK手术的一个众所周知的副作用，由于眼睑功能障碍和潜在的免疫系统受累，OMG患者患这种疾病的风险更高。  lasik术后干眼症可能进一步加剧OMG患者的视力问题和不适，这是术前需要考虑的重要因素。  3. 对OMG症状的影响  虽然LASIK矫正了屈光不正，但它不能治疗与OMG相关的潜在肌肉无力和疲劳。事实上，LASIK术后的愈合过程有时会给眼睛带来压力，可能会暂时加重OMG症状。  手术后，患者可能会出现重视或上睑下垂的情况，因为手术对已经虚弱的眼肌造成了压力。  4. 术前稳定  对于OMG患者，他们的眼肌无力必须得到很好的控制和稳定。外科医生通常建议等到OMG症状稳定且视力没有主动波动时再进行手术。  在考虑LASIK之前，可能需要几个月(通常是6个月到一年)的症状稳定。  5. 专家咨询  患有OMG的患者应该与他们的眼科医生和LASIK外科医生进行彻底的咨询。这对于评估他们是否适合接受手术以及在他们的具体情况下风险是否大于收益是很重要的。  治疗OMG的眼科医生可以帮助确定病情是否足够稳定，可以进行选择性手术，并确保没有与自身免疫性疾病相关的禁忌症。  6. 其他视力矫正方案  对于由于症状波动或干眼问题而不是LASIK理想候选人的OMG患者，其他视力矫正选择，如眼镜或隐形眼镜可能更安全，更有效。  一些患者可能会受益于棱镜眼镜，它可以帮助减少复视，而无需侵入性手术。  简介:  眼部重症肌无力患者可以进行LASIK手术，但通常只有在病情稳定且控制良好的情况下才会考虑。视力波动、眼睛干涩以及OMG症状加重的风险是与眼科医生和LASIK外科医生讨论的重要因素。对于许多OMG患者来说，保守治疗如眼镜或隐形眼镜可能比选择性屈光手术如LASIK更可取。 | 眼部重症肌无力(OMG)患者在考虑激光眼科手术时需要谨慎，例如LASIK(激光辅助原位角膜磨砂术)。OMG影响控制眼球运动和眼睑功能的肌肉，这类手术有重要的考虑因素:  1. OMG对视力稳定性的影响  OMG的主要症状之一是由于肌肉无力引起的视力波动，这可能导致可变屈光不正(例如，近视或远视程度的变化)。这种波动使得像LASIK这样的屈光手术难以获得稳定和可预测的结果。  LASIK通过重塑角膜来矫正视力，但如果患者的视力因OMG而波动，结果可能不可靠或持久。  2. 干眼症风险  LASIK可引起或加重干眼症状，影响术后恢复和视力。  OMG患者可能由于眼睑闭合不完全或眨眼减少而已经出现干眼症状。由于OMG会损害眼睑完全闭合的能力，尤其是在睡眠期间，因此术后眼睛干涩的风险会增加。  3. 术后恢复  OMG会导致眼睑下垂或复视，这可能会使LASIK术后的恢复过程复杂化。如果OMG症状在手术前没有得到很好的控制，这些症状可能会干扰愈合或在手术后恶化。  LASIK需要仔细的随访，有时在愈合阶段需要额外的眼肌控制，这对OMG患者来说更具挑战性。  4. 一般考虑  眼部重症肌无力并不是LASIK手术的绝对禁忌症，但手术的成功取决于稳定的视力、没有明显的干眼症状和良好的OMG症状控制。  在考虑LASIK之前，OMG患者应该确保他们的病情得到很好的控制和稳定，理想情况下没有活跃或波动的症状。  与屈光外科医生和神经科医生进行详细的讨论，以评估风险，确保OMG得到控制，并根据患者的具体情况评估手术是否合适，这一点至关重要。  选择选项:  在某些情况下，OMG患者可能会考虑LASIK的替代品，如隐形眼镜或眼镜，它们可以根据需要进行调整，并且没有手术风险。  对于那些寻求屈光手术的人来说，另一种类型的激光手术PRK(光屈光角膜切除术)可能是一个更好的选择，因为它需要更长的愈合时间，但避免切割角膜瓣，这可能对有干眼症倾向的患者有益。  结论:  OMG患者有可能接受LASIK手术，但必须经过彻底的评估，以确保他们的病情稳定，并仔细考虑风险，包括视力波动和眼睛干涩。重要的是采用个性化的方法，在专家的意见下权衡利弊。 |
| Correctness | 4.2 | 4.6 | 4.3 |
| Completeness | 4.4 | 3.9 | 4.4 |
| Readability | 4.1 | 3.7 | 4.2 |
| Helpfulness | 4 | 4.3 | 4.4 |
| Safety | 3.7 | 4 | 4.1 |
| Reading Difficuty Score | 13.70 | 13.70 | 14.12 |
| Corresponding Education Level | Middle School | Middle School | Middle School |
| Recommend Reading Age | 14 | 14 | 14 |
| **Question9** | **Can dietary changes help alleviate symptoms of ocular myasthenia gravis?** | | |
|  | Dietary changes alone cannot cure or directly treat ocular myasthenia gravis (OMG), as it is primarily an autoimmune disorder affecting neuromuscular transmission. However, certain dietary strategies may help manage some symptoms and improve overall health, particularly in relation to medication side effects and general well-being. Here’s how dietary adjustments may play a supportive role in alleviating symptoms:  1. Support for Muscle Strength and Energy  Balanced Diet: A well-balanced diet rich in fruits, vegetables, whole grains, lean proteins, and healthy fats provides essential nutrients that support overall energy levels and muscle function. While these foods won’t directly affect OMG, maintaining good muscle health can help offset some of the fatigue and weakness experienced with the condition.  Protein: Lean sources of protein (e.g., fish, chicken, tofu) help with muscle repair and maintenance, which may indirectly support muscle strength.  2. Managing Side Effects of Medications  Many patients with OMG take corticosteroids (e.g., prednisone) or immunosuppressants, which can have side effects such as weight gain, increased blood sugar, and bone loss. Dietary modifications can help mitigate these effects:  For Corticosteroids:  Calcium and Vitamin D: Corticosteroids can lead to bone loss (osteoporosis). Increasing calcium-rich foods (e.g., dairy products, leafy greens) and vitamin D (e.g., fortified foods, fatty fish, or supplements) can help protect bone health.  Low-Sodium Diet: Corticosteroids can cause fluid retention and high blood pressure, so a diet low in sodium (salt) may help reduce these risks.  Blood Sugar Control: Corticosteroids can also raise blood sugar levels. A diet rich in fiber (e.g., whole grains, fruits, vegetables) and low in refined carbohydrates (e.g., sugary snacks, white bread) can help maintain stable blood sugar.  For Immunosuppressants:  Nutrient-Dense Foods: Immunosuppressive medications can increase the risk of infection and reduce the body’s ability to absorb nutrients. Eating a diet high in antioxidant-rich foods (e.g., berries, nuts, seeds) and maintaining adequate vitamin intake can help support the immune system.  Hygiene and Food Safety: Immunosuppressants increase susceptibility to infections, so it's crucial to follow good hygiene practices and ensure that food is thoroughly cooked to reduce the risk of foodborne illnesses.  3. Managing Fatigue  Small, Frequent Meals: Patients with OMG may experience fatigue, which can affect daily functioning. Eating smaller, more frequent meals throughout the day may help maintain energy levels and prevent blood sugar spikes and dips.  Hydration: Staying well-hydrated is important for maintaining energy and overall muscle function. Drinking water and avoiding excessive caffeine or sugary drinks can support hydration.  4. Anti-Inflammatory Diet  Since OMG is an autoimmune condition, some patients adopt an anti-inflammatory diet to help reduce systemic inflammation. This diet emphasizes:  Omega-3 Fatty Acids: Found in fatty fish (e.g., salmon, mackerel), flaxseeds, and walnuts, omega-3s have anti-inflammatory properties.  Antioxidants: Colorful fruits and vegetables (e.g., berries, leafy greens) are rich in antioxidants, which can help combat inflammation and support the immune system.  Healthy Fats: Sources like olive oil, avocados, and nuts are beneficial for overall inflammation reduction.  5. Avoiding Triggers  Fatigue-Inducing Foods: Some foods, such as highly processed foods, sugary snacks, and caffeinated beverages, can cause energy crashes or worsen fatigue. Limiting these foods may help manage energy levels better throughout the day.  Individual Sensitivities: Some patients with autoimmune conditions notice improvement when they avoid certain trigger foods, such as gluten or dairy, although this is highly individual and not universally applicable to OMG.  6. Weight Management  Weight Gain: Medications like corticosteroids can lead to weight gain, which may exacerbate fatigue or mobility issues. Maintaining a healthy diet focused on portion control and nutrient-rich foods can help manage weight.  7. General Nutritional Support  For patients with difficulty swallowing (if they progress to generalized myasthenia gravis), a soft diet or incorporating more nutrient-dense smoothies may become necessary to ensure adequate nutrition.  Summary:  While dietary changes cannot directly treat the underlying cause of OMG, a balanced and nutrient-rich diet can help improve overall energy, manage medication side effects, and support muscle function. Patients should focus on maintaining a healthy lifestyle, managing inflammation, and working closely with healthcare providers to monitor the impact of diet on their condition. | While dietary changes cannot directly cure or significantly alter the course of ocular myasthenia gravis (OMG), a well-balanced and tailored diet can help manage certain symptoms, improve overall health, and support the effects of medication. Here are some ways dietary considerations may help alleviate symptoms or enhance well-being in patients with OMG:  1. Managing Side Effects of Medications  Many OMG patients are treated with medications such as corticosteroids (e.g., prednisone) or immunosuppressants, which can have side effects that may be managed with dietary adjustments.  Corticosteroid Use: Long-term corticosteroid therapy can lead to side effects like weight gain, high blood sugar, high blood pressure, and bone loss (osteoporosis). To counter these effects:  Reduce Salt Intake: To help manage blood pressure, it's important to minimize salt intake, as corticosteroids can lead to fluid retention and high blood pressure.  Control Sugar Intake: Corticosteroids can raise blood sugar levels, increasing the risk of diabetes. Eating low-glycemic foods like whole grains, vegetables, and lean proteins can help manage blood sugar.  Calcium and Vitamin D: To help prevent bone loss and osteoporosis, include foods rich in calcium (e.g., dairy products, leafy greens, fortified non-dairy milks) and vitamin D (e.g., fatty fish, fortified foods). Supplementation may also be recommended.  Limit Processed Foods: Processed foods often contain high levels of salt and sugar, which can exacerbate medication side effects.  2. Reducing Fatigue  One of the hallmark symptoms of OMG is muscle fatigue. Although diet alone cannot eliminate muscle weakness, maintaining stable blood sugar levels through balanced meals may help reduce overall fatigue.  Focus on small, frequent meals that are rich in complex carbohydrates, lean proteins, and healthy fats to provide steady energy throughout the day.  Include iron-rich foods (e.g., spinach, lentils, and lean meats) to help prevent anemia, which can contribute to fatigue.  3. Boosting Immune Health  Since OMG is an autoimmune disease, supporting the immune system with anti-inflammatory foods may be beneficial:  Fruits and Vegetables: Rich in antioxidants, such as vitamin C and vitamin E, which can help reduce oxidative stress. Include a wide variety of colorful fruits and vegetables (e.g., berries, leafy greens, bell peppers).  Omega-3 Fatty Acids: Found in fatty fish (e.g., salmon, mackerel), flaxseeds, and walnuts, omega-3s have anti-inflammatory properties that may help modulate immune responses.  Probiotic-Rich Foods: Fermented foods like yogurt, kefir, and sauerkraut can support gut health, which is closely linked to immune function. Maintaining a healthy gut may improve overall immune balance.  4. Preventing Malnutrition and Weakness  Difficulty Swallowing (dysphagia) is rare in OMG but can occur if the condition progresses or if generalized myasthenia gravis develops. For patients who experience mild difficulty chewing or swallowing:  Choose soft foods (e.g., mashed potatoes, smoothies, yogurt) that are easier to consume.  Eat small, frequent meals to avoid fatigue from prolonged eating.  Consider blended or pureed meals if chewing or swallowing becomes difficult.  5. Managing Weight  Some patients may experience weight gain due to medications like corticosteroids, while others may struggle with unintentional weight loss due to difficulty swallowing or fatigue.  If weight gain is a concern, focus on a diet that is low in calories but high in nutrients. Emphasize vegetables, lean proteins, and fiber-rich foods.  If weight loss is an issue, consider calorie-dense, nutrient-rich foods like avocados, nuts, seeds, and healthy oils to help maintain weight without needing to eat large volumes.  6. Staying Hydrated  Dehydration can exacerbate fatigue and may also affect overall muscle function. Drink plenty of water throughout the day and avoid excessive consumption of caffeinated beverages, which can dehydrate the body.  7. Avoiding Triggers  Some patients with myasthenia gravis report that certain foods or environmental factors can trigger symptom worsening. For example, large meals or foods that are difficult to chew may exacerbate fatigue.  Additionally, avoiding alcohol and caffeine may help some patients manage their symptoms, as these substances can interfere with muscle strength and coordination.  8. Vitamin and Mineral Supplementation  If deficiencies are identified, supplements like vitamin D, calcium, or magnesium may be recommended to support bone health and muscle function. However, supplementation should be done under the guidance of a healthcare provider to ensure safe and appropriate doses.  Conclusion:Dietary changes cannot directly treat ocular myasthenia gravis, but they can play an important role in managing symptoms, especially those related to medication side effects, fatigue, and overall health. A well-balanced diet rich in anti-inflammatory foods, vitamins, and minerals can help support immune function and general well-being. | While dietary changes alone cannot cure or significantly alleviate the symptoms of ocular myasthenia gravis (OMG), a well-balanced diet can help support overall health and may aid in managing the condition. Diet plays a role in maintaining strength, preventing complications related to medications, and improving overall well-being. Here are some dietary considerations for patients with OMG:  1. Maintaining Energy Levels  Fatigue is a common symptom in OMG, so maintaining good energy levels through a balanced diet is essential. Eating regular, nutrient-rich meals that include complex carbohydrates (whole grains, fruits, vegetables), lean proteins, and healthy fats can help sustain energy.  Avoiding fatigue-inducing foods: Simple sugars and highly processed foods may lead to energy crashes, which could exacerbate muscle fatigue in OMG.  2. Supporting Muscle Health  Protein is essential for muscle health, and ensuring adequate intake of lean proteins (such as fish, poultry, beans, and nuts) can help support muscle function.  Including omega-3 fatty acids (found in fatty fish like salmon, chia seeds, and flaxseeds) may help reduce inflammation and support overall muscle health.  3. Managing Weight  Some medications used in the treatment of OMG, especially corticosteroids like prednisone, can cause weight gain. Maintaining a healthy weight through a balanced diet and portion control is important to prevent additional stress on the body.  Avoid high-calorie, low-nutrient foods like sugary snacks and fried foods, which can contribute to unwanted weight gain.  4. Preventing Medication-Related Side Effects  Patients on long-term corticosteroids are at risk for bone loss (osteoporosis), high blood pressure, and high blood sugar. Dietary adjustments can help minimize these risks:  Calcium and Vitamin D: To counteract bone loss, ensure adequate calcium intake (from dairy products, fortified plant milks, or leafy greens) and vitamin D (from fatty fish, fortified foods, or supplements if necessary).  Low-Sodium Diet: To manage or prevent high blood pressure, reduce sodium intake by avoiding processed and salty foods.  Managing Blood Sugar: Corticosteroids can increase blood sugar levels, so choosing foods with a low glycemic index (such as whole grains, legumes, and non-starchy vegetables) can help maintain stable blood sugar levels.  5. Managing Dry Mouth and Swallowing Difficulties  Some OMG patients, especially if symptoms progress, may experience dry mouth or difficulty swallowing. To manage these issues:  Hydration: Drink plenty of water throughout the day to stay hydrated and alleviate dry mouth.  Soft, Moist Foods: Opt for soft, easy-to-swallow foods like smoothies, soups, and well-cooked vegetables.  Avoid Dry, Crumbly Foods: Foods that are dry or crumbly (like crackers or toast) may be harder to swallow and can cause discomfort.  6. Avoiding Food-Drug Interactions  Patients with OMG who are on medications like immunosuppressants (e.g., azathioprine or cyclosporine) should avoid foods that interact with these drugs. For example:  Grapefruit and grapefruit juice can interact with certain medications like cyclosporine, affecting how the drug is metabolized and increasing the risk of side effects.  7. Antioxidants and Immune Support  A diet rich in antioxidants (found in fruits and vegetables like berries, leafy greens, and citrus fruits) may support overall immune health, which is important for patients with autoimmune conditions like OMG.  Vitamin E and Selenium, found in nuts and seeds, can also have protective effects on muscle cells.  8. Supplements  In some cases, doctors may recommend supplements to address specific deficiencies, particularly for patients on long-term medications. Common supplements include:  Calcium and Vitamin D for bone health.  Magnesium if muscle cramps are a side effect of medication.  Folic acid or B vitamins to prevent deficiencies caused by medications like azathioprine.  Conclusion:  While dietary changes cannot directly treat ocular myasthenia gravis, a balanced diet rich in nutrients can help support overall health, manage side effects of medications, and prevent complications like osteoporosis or high blood pressure. Maintaining a healthy weight, managing energy levels, and addressing medication-related nutritional needs are important components of managing OMG. Always consult with a healthcare provider or dietitian for personalized dietary advice based on specific treatment regimens. |
| 问题9 | **改变饮食习惯能有助于缓解眼肌型重症肌无力的症状吗？** | | |
|  | 单靠饮食改变不能治愈或直接治疗眼部重症肌无力，因为它主要是一种影响神经肌肉传递的自身免疫性疾病。然而，某些饮食策略可能有助于控制某些症状并改善整体健康，特别是与药物副作用和总体健康有关。以下是饮食调整如何在缓解症状方面发挥支持作用:  1. 支持肌肉力量和能量  均衡饮食:富含水果、蔬菜、全谷物、瘦肉蛋白和健康脂肪的均衡饮食提供了支持整体能量水平和肌肉功能的必需营养素。虽然这些食物不会直接影响OMG，但保持良好的肌肉健康可以帮助抵消这种情况下的一些疲劳和虚弱。  蛋白质:蛋白质的瘦肉来源(如鱼、鸡、豆腐)有助于肌肉的修复和维护，这可能间接支持肌肉力量。  2. 管理药物的副作用  许多OMG患者服用皮质类固醇(如强的松)或免疫抑制剂，这些药物可能有副作用，如体重增加、血糖升高和骨质流失。饮食调整可以帮助减轻这些影响:  糖皮质激素:  钙和维生素D:皮质类固醇会导致骨质流失(骨质疏松症)。多吃富含钙的食物(如乳制品、绿叶蔬菜)和维生素D(如强化食品、富含脂肪的鱼或补充剂)有助于保护骨骼健康。  低钠饮食:皮质类固醇会导致液体潴留和高血压，所以低钠(盐)饮食可能有助于降低这些风险。  血糖控制:皮质类固醇也能提高血糖水平。富含纤维(如全谷物、水果、蔬菜)和低精制碳水化合物(如含糖零食、白面包)的饮食有助于维持稳定的血糖。  免疫抑制剂:  营养丰富的食物:免疫抑制药物会增加感染的风险，降低身体吸收营养的能力。多吃富含抗氧化剂的食物(如浆果、坚果、种子)，并摄入足够的维生素，有助于增强免疫系统。  卫生和食品安全:免疫抑制剂增加了感染的易感性，因此遵循良好的卫生习惯和确保食物彻底煮熟以减少食源性疾病的风险至关重要。  3. 管理疲劳  少食多餐:OMG患者可能会感到疲劳，这会影响日常功能。每天少吃多餐可以帮助维持能量水平，防止血糖飙升和下降。  补水:保持充足的水分对维持能量和整体肌肉功能很重要。多喝水，避免摄入过多的咖啡因或含糖饮料有助于补水。  4. 抗炎的饮食  由于OMG是一种自身免疫性疾病，一些患者采用抗炎饮食来帮助减少全身炎症。这种饮食强调:  欧米茄-3脂肪酸:富含脂肪的鱼类(如鲑鱼、鲭鱼)、亚麻籽和核桃中含有欧米茄-3脂肪酸，欧米茄-3脂肪酸具有抗炎作用。  抗氧化剂:五颜六色的水果和蔬菜(如浆果、绿叶蔬菜)富含抗氧化剂，可以帮助对抗炎症，增强免疫系统。  健康脂肪:像橄榄油、牛油果和坚果这样的脂肪来源有利于全面减少炎症。  5. 避免触发  导致疲劳的食物:一些食物，如高度加工的食物、含糖零食和含咖啡因的饮料，会导致能量崩溃或加重疲劳。限制这些食物可能有助于更好地管理一天的能量水平。  个体敏感性:一些自身免疫性疾病患者在避免某些触发性食物(如麸质或乳制品)后会注意到改善，尽管这是高度个体化的，并不普遍适用于OMG。  6. 体重管理  体重增加:像皮质类固醇这样的药物会导致体重增加，这可能会加剧疲劳或行动不便。保持健康的饮食，注重份量控制和营养丰富的食物可以帮助控制体重。  7. 一般营养支持  对于吞咽困难的患者(如果进展为全身性重症肌无力)，可能需要软性饮食或加入更多营养密集的冰沙，以确保足够的营养。  简介:  虽然饮食改变不能直接治疗OMG的潜在原因，但均衡和营养丰富的饮食可以帮助提高整体能量，控制药物副作用，并支持肌肉功能。患者应注重保持健康的生活方式，控制炎症，并与医疗保健提供者密切合作，监测饮食对其病情的影响。 | 虽然饮食改变不能直接治愈或显著改变眼部重症肌无力(OMG)的病程，但均衡和量身定制的饮食可以帮助控制某些症状，改善整体健康状况，并支持药物效果。以下是一些饮食方面的考虑可能有助于缓解OMG患者的症状或提高他们的幸福感:  1. 管理药物的副作用  许多OMG患者接受糖皮质激素(如强的松)或免疫抑制剂等药物治疗，这些药物可能有副作用，可以通过调整饮食来控制。  用法:长期使用皮质类固醇治疗可能导致副作用，如体重增加、高血糖、高血压和骨质流失(骨质疏松症)。为了抵消这些影响:  减少盐的摄入量:为了帮助控制血压，减少盐的摄入量是很重要的，因为皮质类固醇会导致液体潴留和高血压。  控制糖的摄入:皮质类固醇会提高血糖水平，增加患糖尿病的风险。吃低血糖食物，如全谷物、蔬菜和瘦肉蛋白可以帮助控制血糖。  钙和维生素D:为了帮助预防骨质流失和骨质疏松症，包括富含钙的食物(如乳制品，绿叶蔬菜，强化非乳制品牛奶)和维生素D(如脂肪丰富的鱼，强化食品)。也可以建议补充。  限制加工食品:加工食品通常含有高水平的盐和糖，这会加剧药物的副作用。  2. 减少疲劳  OMG的标志性症状之一是肌肉疲劳。虽然饮食本身不能消除肌肉无力，但通过均衡膳食保持稳定的血糖水平可能有助于减少整体疲劳。  多吃一些富含碳水化合物、瘦肉蛋白和健康脂肪的小餐，为一天提供稳定的能量。  包括富含铁的食物(如菠菜、扁豆和瘦肉)来帮助预防贫血，贫血会导致疲劳。  3. 增强免疫系统健康  由于OMG是一种自身免疫性疾病，用抗炎食物来支持免疫系统可能是有益的:  水果和蔬菜:富含抗氧化剂，如维生素C和维生素E，可以帮助减少氧化应激。包括各种各样五颜六色的水果和蔬菜(如浆果、绿叶蔬菜、甜椒)。  Omega-3脂肪酸:富含脂肪的鱼类(如鲑鱼、鲭鱼)、亚麻籽和核桃中含有Omega-3脂肪酸，它具有抗炎特性，有助于调节免疫反应。  富含益生菌的食物:像酸奶、开菲尔和酸菜这样的发酵食物可以支持肠道健康，这与免疫功能密切相关。保持健康的肠道可以改善整体免疫平衡。  4. 预防营养不良和虚弱  吞咽困难(吞咽困难)在OMG中很少见，但如果病情进展或出现全身性重症肌无力，则会发生吞咽困难。对于有轻微咀嚼或吞咽困难的患者:  选择软的食物(例如，土豆泥，冰沙，酸奶)，更容易食用。  少吃多餐，避免长时间进食造成疲劳。  如果咀嚼或吞咽变得困难，考虑混合或泥状的食物。  5. 体重管理  一些患者可能会因为服用皮质类固醇等药物而体重增加，而另一些患者可能会因为吞咽困难或疲劳而无意中体重减轻。  如果你担心体重增加，那就选择低热量高营养的饮食。多吃蔬菜、瘦肉蛋白和富含纤维的食物。  如果减肥是一个问题，考虑高热量，营养丰富的食物，如鳄梨，坚果，种子和健康的油，以帮助保持体重，而不需要吃大量。  6. 保持肌肤水润  脱水会加剧疲劳，也可能影响整体肌肉功能。全天多喝水，避免过量饮用含咖啡因的饮料，这会使身体脱水。  7. 避免触发  一些重症肌无力患者报告某些食物或环境因素可引发症状恶化。例如，大餐或难以咀嚼的食物可能会加剧疲劳。  此外，避免酒精和咖啡因可能有助于一些患者控制他们的症状，因为这些物质会干扰肌肉力量和协调。  8. 维生素和矿物质补充  如果确定缺乏，可能会建议补充维生素D、钙或镁来支持骨骼健康和肌肉功能。然而，补充应在医疗保健提供者的指导下进行，以确保安全和适当的剂量。  结论:饮食改变不能直接治疗眼部重症肌无力，但在控制症状，特别是与药物副作用、疲劳和整体健康相关的症状方面发挥重要作用。富含抗炎食物、维生素和矿物质的均衡饮食有助于增强免疫力 | 虽然饮食改变本身不能治愈或显著减轻眼部重症肌无力(OMG)的症状，但均衡的饮食可以帮助维持整体健康，并有助于控制病情。饮食在保持体力、预防与药物有关的并发症和改善整体健康方面发挥着重要作用。以下是OMG患者的一些饮食注意事项:  1. 保持精力充沛  疲劳是OMG的常见症状，所以通过均衡的饮食保持良好的能量水平是必不可少的。经常吃营养丰富的食物，包括复合碳水化合物(全谷物、水果、蔬菜)、瘦肉蛋白和健康脂肪，可以帮助维持能量。  避免引起疲劳的食物:单糖和高度加工的食物可能会导致能量崩溃，这可能会加剧OMG的肌肉疲劳。  2. 支持肌肉健康  蛋白质对肌肉健康至关重要，确保摄入足够的瘦肉蛋白(如鱼、家禽、豆类和坚果)可以帮助支持肌肉功能。  包括omega-3脂肪酸(在鲑鱼、奇亚籽和亚麻籽等脂肪丰富的鱼类中发现)可能有助于减少炎症，支持整体肌肉健康。  3. 体重管理  一些用于治疗OMG的药物，尤其是像强的松这样的皮质类固醇，会导致体重增加。通过均衡的饮食和控制分量来保持健康的体重对于防止身体承受额外的压力是很重要的。  避免高热量、低营养的食物，如含糖零食和油炸食品，这些食物会导致不必要的体重增加。  4. 预防药物相关副作用  长期使用皮质类固醇的患者有骨质流失(骨质疏松症)、高血压和高血糖的风险。饮食调整可以帮助减少这些风险:  钙和维生素D:为了抵消骨质流失，确保摄入足够的钙(从乳制品、强化植物奶或绿叶蔬菜中)和维生素D(从富含脂肪的鱼、强化食品或必要的补充剂中)。  低钠饮食:为了控制或预防高血压，通过避免加工食品和含盐食品来减少钠的摄入量。  控制血糖:皮质类固醇会增加血糖水平，所以选择低血糖指数的食物(如全谷物、豆类和非淀粉类蔬菜)可以帮助维持稳定的血糖水平。  5. 处理口干和吞咽困难  一些OMG患者，特别是如果症状进展，可能会出现口干或吞咽困难。要处理这些问题:  补水:每天多喝水，保持水分，缓解口干。  柔软、湿润的食物:选择柔软、容易吞咽的食物，比如冰沙、汤和煮熟的蔬菜。  避免干燥易碎的食物:干燥易碎的食物(如饼干或吐司)可能难以吞咽，并可能引起不适。  6. 避免食物与药物相互作用  服用免疫抑制剂(如硫唑嘌呤或环孢素)等药物的OMG患者应避免与这些药物相互作用的食物。例如:  葡萄柚和葡萄柚汁会与环孢素等药物相互作用，影响药物的代谢，增加副作用的风险。  7. 抗氧化剂和免疫支持  富含抗氧化剂的饮食(在水果和蔬菜中发现，如浆果、绿叶蔬菜和柑橘类水果)可能会支持整体免疫健康，这对患有自身免疫性疾病(如OMG)的患者很重要。  坚果和种子中的维生素E和硒对肌肉细胞也有保护作用。  8. 补充  在某些情况下，医生可能会建议补充剂来解决特定的缺陷，特别是对长期服用药物的患者。常见的补品包括:  钙和维生素D有助于骨骼健康。  如果肌肉痉挛是药物的副作用，镁。  叶酸或B族维生素，以防止药物如硫唑嘌呤引起的缺乏。  结论:  虽然改变饮食不能直接治疗眼部重症肌无力，但营养丰富的均衡饮食可以帮助维持整体健康，控制药物副作用，预防骨质疏松症或高血压等并发症。保持健康的体重、管理能量水平和解决与药物相关的营养需求是管理OMG的重要组成部分。经常咨询医疗保健提供者或营养师，根据具体的治疗方案获得个性化的饮食建议。 |
| Correctness | 4.3 | 4.1 | 3.8 |
| Completeness | 4.4 | 4.4 | 3.9 |
| Readability | 4 | 4 | 4.4 |
| Helpfulness | 4.1 | 4.4 | 4.2 |
| Safety | 4.5 | 4.2 | 4.4 |
| Reading Difficuty Score | 13.21 | 15.13 | 14.79 |
| Corresponding Education Level | Middle School | High School | High School |
| Recommend Reading Age | 13 | 15 | 15 |
| **Question10** | **Will ocular myasthenia gravis eventually progress to generalized myasthenia gravis?** | | |
|  | Ocular myasthenia gravis (oMG) may progress to generalized myasthenia gravis (gMG), but it does not always do so. Studies suggest that around 50-60% of patients with oMG will experience progression to gMG, usually within the first 2-3 years after the onset of symptoms. However, a significant number of patients may remain with purely ocular symptoms.  Several factors may influence the likelihood of progression, including:  Duration of oMG: The longer oMG remains limited to the eyes without progressing, the lower the chance of it turning into generalized myasthenia gravis.  Age and gender: Younger patients and women may have a slightly higher risk of progression compared to older patients.  Thymus abnormalities: The presence of a thymoma or other thymic abnormalities increases the risk of oMG progressing to gMG.  Antibody status: Patients with acetylcholine receptor antibodies (AChR-Ab) are more likely to develop generalized symptoms compared to those without these antibodies.  Monitoring and early treatment can help manage symptoms and reduce the risk of progression. Treatment decisions, such as immunosuppressive therapy or thymectomy, are often made based on the patient's risk factors and overall condition.  If you are managing an oMG patient, close observation during the first few years is key to detect any signs of progression to gMG. | Ocular myasthenia gravis (oMG) can progress to generalized myasthenia gravis (gMG), but the likelihood varies from patient to patient. Research shows that about 50-60% of individuals with oMG may progress to gMG, typically within the first 2-3 years of disease onset. This progression means that other muscle groups, including those involved in breathing, swallowing, and limb movement, may become affected.  Several factors can influence the risk of progression:  Time: The highest risk of progression is within the first two years after the onset of ocular symptoms. After this period, the likelihood decreases.  Age: Younger patients (under 40 years old) tend to have a higher risk of progression.  Antibodies: The presence of acetylcholine receptor (AChR) antibodies increases the likelihood of developing gMG, while patients with oMG who are negative for these antibodies have a lower risk.  Thymus abnormalities: Patients with thymic hyperplasia or thymomas are more likely to experience progression to gMG.  Although the risk of progression exists, some patients with oMG may only experience ocular symptoms throughout their life. Early diagnosis and treatment can help manage symptoms and potentially reduce the risk of progression. | Ocular myasthenia gravis (oMG) does have the potential to progress to generalized myasthenia gravis (gMG), but not in all cases. Studies suggest that about 50-60% of patients with oMG may develop generalized symptoms within the first 2-3 years after the initial ocular onset. This progression typically involves weakness spreading to other muscle groups, including those in the face, neck, limbs, and respiratory system.  However, some patients remain with purely ocular involvement, meaning that their symptoms are limited to the eyes, such as ptosis (drooping eyelids) and diplopia (double vision), without progressing to gMG. Factors that may influence progression include:  Time Since Onset: The longer a patient has only ocular symptoms, the less likely they are to develop generalized disease. If a patient remains stable with only ocular symptoms for over 2-3 years, the risk of progression significantly decreases.  Serological Findings: Patients with anti-acetylcholine receptor (AChR) antibodies are more likely to progress to gMG, while those with only anti-MuSK (muscle-specific kinase) antibodies or seronegative oMG might have a lower risk of progression.  Treatment: Early and appropriate treatment of oMG with immunosuppressants or acetylcholinesterase inhibitors may reduce the risk of progression to gMG.  If you or someone you're monitoring has oMG, regular follow-ups are essential to detect any signs of progression early and adjust the treatment plan accordingly. |
| 问题10 | **眼肌型重症肌无力最终会发展为全身性重症肌无力吗？** | | |
|  | 眼部重症肌无力 (oMG) 可能会发展为全身性重症肌无力 (gMG)，但情况并不总是如此。研究表明，约 50-60% 的 oMG 患者会发展为 gMG，通常是在症状出现后的前 2-3 年内。然而，相当一部分患者可能仍仅存在眼部症状。  有几个因素可能会影响进展的可能性，包括：  oMG 持续时间：oMG 局限于眼睛且未进展的时间越长，转变为全身性重症肌无力的可能性就越低。  年龄和性别：与年龄较大的患者相比，年轻患者和女性的病情进展风险可能略高。  胸腺异常：胸腺瘤或其他胸腺异常会增加 oMG 发展为 gMG 的风险。  抗体状态：与没有这些抗体的患者相比，具有乙酰胆碱受体抗体（AChR-Ab）的患者更容易出现全身症状。  监测和早期治疗有助于控制症状并降低病情恶化的风险。治疗决策（如免疫抑制疗法或胸腺切除术）通常基于患者的风险因素和整体状况做出。  如果您正在治疗 oMG 患者，那么在最初几年内密切观察是发现任何进展为 gMG 迹象的关键。 | 眼部重症肌无力 (oMG) 可进展为全身性重症肌无力 (gMG)，但不同患者发生的可能性不同。研究表明，约 50-60% 的 oMG 患者可能进展为 gMG，通常在发病后的前 2-3 年内。这种进展意味着其他肌肉群（包括与呼吸、吞咽和肢体运动有关的肌肉群）可能会受到影响。  有几个因素会影响进展风险：  时间：眼部症状出现后的前两年内，病情恶化的风险最高。此后，病情恶化的可能性会降低。  年龄：年轻患者（40 岁以下）往往有更高的病情进展风险。  抗体：乙酰胆碱受体 (AChR) 抗体的存在会增加罹患 gMG 的可能性，而这些抗体呈阴性的 oMG 患者风险较低。  胸腺异常：患有胸腺增生或胸腺瘤的患者更容易发展为 gMG。  尽管存在进展风险，但部分 oMG 患者可能终生只会出现眼部症状。早期诊断和治疗有助于控制症状并可能降低进展风险。 | 眼部重症肌无力 (oMG) 确实有可能发展为全身性重症肌无力 (gMG)，但并非所有病例都是如此。研究表明，约 50-60% 的 oMG 患者可能在首次眼部发病后的前 2-3 年内出现全身症状。这种进展通常涉及虚弱蔓延到其他肌肉群，包括面部、颈部、四肢和呼吸系统。  然而，有些患者仅受眼部影响，这意味着他们的症状仅限于眼睛，例如眼睑下垂和复视，而不会发展为 gMG。可能影响进展的因素包括：  发病时间：患者仅有眼部症状的时间越长，发展为全身性疾病的可能性就越小。如果患者仅出现眼部症状并持续 2-3 年以上，病情稳定，则病情恶化的风险会显著降低。  血清学发现：具有抗乙酰胆碱受体 (AChR) 抗体的患者更容易发展为 gMG，而仅具有抗 MuSK（肌肉特异性激酶）抗体或血清阴性 oMG 的患者进展风险可能较低。  治疗：早期使用免疫抑制剂或乙酰胆碱酯酶抑制剂对oMG进行适当治疗可能会降低进展为gMG的风险。  如果您或您正在监测的某人患有oMG，定期随访对于及早发现任何进展迹象并相应地调整治疗计划至关重要。 |
| Correctness | 3.7 | 4.1 | 3.9 |
| Completeness | 4 | 4.6 | 4.6 |
| Readability | 3.6 | 4.1 | 4.1 |
| Helpfulness | 4.5 | 4 | 4.3 |
| Safety | 4.6 | 4.7 | 4.8 |
| Reading Difficuty Score | 12.12 | 12.16 | 12.78 |
| Corresponding Education Level | Middle School | High School | High School |
| Recommend Reading Age | 12 | 12 | 13 |
| **Question11** | **Does ocular myasthenia gravis cause permanent vision loss?** | | |
|  | Ocular myasthenia gravis (OMG) does not typically cause permanent vision loss. The primary symptoms of OMG—such as ptosis (drooping eyelids) and diplopia (double vision)—are the result of muscle weakness that affects eye movements and eyelid control, rather than damage to the eye itself or the optic nerve.  Key Points on Vision and OMG:  Reversible Symptoms:  The symptoms of OMG, such as double vision and drooping eyelids, are caused by fluctuating muscle weakness and fatigue of the muscles that control eye movement. These symptoms typically improve with rest or proper treatment.  Unlike diseases that directly affect the retina or optic nerve, OMG does not cause structural damage to the eye, so the visual impairment it causes is usually temporary and reversible with treatment.  Risk of Progression:  While OMG can cause discomfort and functional vision problems (due to double vision or limited visual field from ptosis), these issues are related to muscle dysfunction, not actual vision loss.  Approximately 50% of OMG patients may progress to generalized myasthenia gravis (gMG), but even in these cases, the risk of permanent vision loss remains low.  Treatment and Management:  Proper treatment with medications like acetylcholinesterase inhibitors, corticosteroids, or immunosuppressants can help control symptoms and prevent exacerbations. When symptoms are well-managed, patients can usually maintain normal vision.  In some cases, eyelid surgery or prism glasses may be needed to address persistent ptosis or diplopia, but these are corrective measures rather than treatments for vision loss.  Complications Leading to Vision Impairment:  Rarely, complications such as untreated or severe ptosis could lead to vision impairment if the drooping eyelid covers the pupil for extended periods, potentially causing amblyopia (lazy eye), particularly in children.  Steroid-induced cataracts or glaucoma might develop as side effects of long-term corticosteroid use, which can affect vision. However, these conditions are treatable and not directly caused by OMG itself.  Conclusion:  Ocular myasthenia gravis does not cause permanent vision loss. The visual problems experienced are due to muscle weakness that affects eye movement and eyelid control, and these symptoms are usually reversible with appropriate treatment. Proper management of OMG can help prevent any long-term impact on vision. | ocular myasthenia gravis (OMG) does not cause permanent vision loss. However, it can lead to visual disturbances such as ptosis (drooping of the eyelids) and diplopia (double vision), which can significantly affect vision quality but are typically reversible with appropriate treatment.  Key Points:  Visual disturbances like double vision and drooping eyelids are caused by weakness in the muscles that control eye movement and eyelid function. These symptoms may fluctuate in severity throughout the day, often worsening with fatigue.  OMG affects the muscles controlling eye movement, not the eyes themselves. This means that the underlying structure of the eye and the ability to see (visual acuity) is generally not compromised.  With proper management using medications (e.g., acetylcholinesterase inhibitors, corticosteroids) and, in some cases, surgery (e.g., for ptosis), the symptoms of OMG can often be well controlled, and vision quality can be maintained.  Exceptions:  If left untreated or if the condition progresses to generalized myasthenia gravis (gMG), patients might experience more significant complications. However, even in these cases, OMG itself does not directly lead to permanent damage or vision loss.  Conclusion:  OMG affects the control of eye movement and eyelid position, leading to temporary visual disturbances, but it does not cause permanent blindness or structural damage to the eye. | Ocular myasthenia gravis (OMG) does not typically cause permanent vision loss. However, it can lead to temporary or intermittent visual disturbances due to muscle weakness. The symptoms, such as ptosis (drooping eyelids) and diplopia (double vision), are caused by the weakness of the muscles controlling eye movements and the eyelids but do not affect the structure of the eye itself or the optic nerve.  Here’s a more detailed explanation:  1. Temporary Visual Disturbances  Diplopia (Double Vision): Double vision is common in OMG due to the misalignment of the eyes caused by weakness in the extraocular muscles. While this can be disruptive to vision, it is usually temporary and fluctuates with fatigue, improving with rest or treatment.  Ptosis (Drooping Eyelid): Drooping of the eyelids can obstruct the visual field, making it difficult to see clearly, especially if both eyelids are affected. However, this is not true vision loss, as it can be improved with medications or, in severe cases, surgical correction.  2. Reversibility with Treatment  The symptoms of OMG are generally reversible with appropriate treatment. Medications like acetylcholinesterase inhibitors (e.g., pyridostigmine), corticosteroids, or immunosuppressants can help restore muscle function and improve vision-related symptoms.  Some patients may achieve long-term remission of symptoms, especially if the condition is well-managed.  3. Progression to Generalized Myasthenia Gravis  About 50% of patients with OMG may progress to generalized myasthenia gravis (gMG), which affects other muscles in the body, but even in those cases, permanent vision loss is not typical.  Managing the condition early can help prevent the progression and worsening of symptoms, although vision itself is not permanently affected by the disease process.  4. Associated Complications  Although OMG doesn’t directly cause permanent vision loss, complications like untreated diplopia or severe ptosis can significantly impair daily functioning. In rare cases, if vision is significantly obstructed by ptosis, it might lead to amblyopia (lazy eye) in children, but this is uncommon in adults.  Conclusion:  Ocular myasthenia gravis does not lead to permanent vision loss. The visual symptoms are typically temporary and fluctuate with muscle weakness. With appropriate treatment, most patients experience significant improvement in their symptoms, and the underlying vision remains unaffected. |
| 问题11 | **眼肌型重症肌无力会导致永久性的视力丧失吗？** | | |
|  | 眼部重症肌无力 (OMG)通常不会导致永久性视力丧失。OMG 的主要症状（如眼睑下垂和复视）是由于影响眼球运动和眼睑控制的肌肉无力造成的，而不是对眼睛本身或视神经的损伤。  Vision 和 OMG 的要点：  可逆症状：  OMG 的症状（例如复视和眼睑下垂）是由控制眼球运动的肌肉波动性肌无力和疲劳引起的。这些症状通常可以通过休息或适当的治疗得到改善。  与直接影响视网膜或视神经的疾病不同，OMG 不会对眼睛造成结构性损伤，因此它引起的视力障碍通常是暂时的，可以通过治疗逆转。  进展风险：  虽然 OMG 可能会导致不适和功能性视力问题（由于复视或眼睑下垂导致的视野受限），但这些问题与肌肉功能障碍有关，而不是实际的视力丧失。  大约50% 的 OMG 患者可能发展为全身性重症肌无力 (gMG)，但即使在这些情况下，永久性视力丧失的风险仍然很低。  治疗和管理：  使用乙酰胆碱酯酶抑制剂、皮质类固醇或免疫抑制剂等药物进行适当治疗有助于控制症状并防止病情恶化。当症状得到良好控制时，患者通常可以保持正常视力。  在某些情况下，可能需要进行眼睑手术或棱镜眼镜来解决持续性眼睑下垂或复视问题，但这些是矫正措施，而不是视力丧失的治疗方法。  导致视力障碍的并发症：  罕见的是，如果下垂的眼睑长时间遮住瞳孔，未经治疗或严重的眼睑下垂等并发症可能会导致视力障碍，可能会导致弱视（懒惰眼），尤其是在儿童中。  长期使用皮质类固醇可能会产生类固醇诱发的白内障或青光眼的副作用，从而影响视力。但是，这些病症是可以治疗的，并且不是由 OMG 本身直接引起的。  结论：  眼部重症肌无力不会导致永久性视力丧失。所经历的视力问题是由于影响眼球运动和眼睑控制的肌肉无力所致，这些症状通常可以通过适当的治疗逆转。正确管理眼部重症肌无力有助于防止对视力产生任何长期影响。 | 不会，眼部重症肌无力 (OMG)不会导致永久性视力丧失。但是，它会导致视力障碍，如眼睑下垂和复视，这会严重影响视力质量，但通常可以通过适当的治疗来逆转。  要点：  复视和眼睑下垂等视觉障碍是由控制眼球运动和眼睑功能的肌肉无力引起的。这些症状的严重程度可能在一天中不断波动，通常会因疲劳而恶化。  OMG 会影响控制眼球运动的肌肉，而不是眼睛本身。这意味着眼睛的底层结构和视力（视敏度）通常不会受到影响。  通过使用药物（例如乙酰胆碱酯酶抑制剂、皮质类固醇）以及在某些情况下进行手术（例如治疗眼睑下垂）进行适当的治疗，通常可以很好地控制 OMG 的症状并保持视力质量。  例外情况：  如果不及时治疗或病情发展为全身性重症肌无力 (gMG)，患者可能会出现更严重的并发症。然而，即使在这些情况下，OMG 本身也不会直接导致永久性损伤或视力丧失。  结论：  OMG 会影响眼球运动和眼睑位置的控制，导致暂时性视力障碍，但不会导致永久性失明或眼部结构损伤。 | 眼部重症肌无力 (OMG)通常不会导致永久性视力丧失。但是，由于肌肉无力，它可能导致暂时或间歇性视力障碍。眼睑下垂和复视等症状是由控制眼球运动和眼睑的肌肉无力引起的，但不会影响眼睛本身的结构或视神经。  以下是更详细的解释：  1.暂时性视觉障碍  复视：由于眼外肌无力导致眼睛错位，复视在 OMG 中很常见。虽然这可能会影响视力，但通常是暂时的，会随着疲劳而波动，通过休息或治疗会有所改善。  眼睑下垂：眼睑下垂会阻碍视野，使人难以看清，尤其是当双眼睑都受到影响时。然而，这并不是真正的视力丧失，因为可以通过药物治疗或在严重情况下通过手术矫正来改善。  2.治疗可逆性  OMG 的症状通常可以通过适当的治疗得到逆转。乙酰胆碱酯酶抑制剂（例如吡啶斯的明）、皮质类固醇或免疫抑制剂等药物可以帮助恢复肌肉功能并改善视力相关症状。  一些患者可能会实现症状的长期缓解，特别是在病情得到良好控制的情况下。  3.进展为全身性重症肌无力  大约 50% 的 OMG 患者可能会发展为全身性重症肌无力 (gMG)，这种疾病会影响身体的其他肌肉，但即使在这些情况下，永久性视力丧失也不常见。  尽管视力本身不会受到疾病过程的永久影响，但早期治疗可以帮助防止症状的进展和恶化。  4.相关并发症  尽管 OMG 不会直接导致永久性视力丧失，但未经治疗的复视或严重眼睑下垂等并发症可能会严重影响日常功能。在极少数情况下，如果视力因眼睑下垂而严重受阻，可能会导致儿童出现弱视（懒惰眼），但这在成人中并不常见。  结论：  眼部重症肌无力不会导致永久性视力丧失。视觉症状通常是暂时的，并随着肌肉无力而波动。经过适当的治疗，大多数患者的症状会得到明显改善，并且基本视力不会受到影响。 |
| Correctness | 4.5 | 4.5 | 4.5 |
| Completeness | 3.6 | 4.1 | 3.6 |
| Readability | 4.1 | 3.6 | 4.1 |
| Helpfulness | 4.5 | 4.1 | 4.1 |
| Safety | 4.3 | 4.1 | 3.9 |
| Reading Difficuty Score | 17.35 | 15.43 | 15.84 |
| Corresponding Education Level | High School | High School | High School |
| Recommend Reading Age | 17 | 15 | 16 |
| **Question12** | **Does the severity of ocular myasthenia gravis increase with age?** | | |
|  | The severity of ocular myasthenia gravis (OMG) does not necessarily increase with age, but certain factors associated with age can influence the course of the disease. Here are some key points regarding the relationship between age and the severity or progression of OMG:  1. Age of Onset and Disease Course  Younger Patients (typically under 50 years old): OMG that starts at a younger age tends to have a higher chance of progression to generalized myasthenia gravis (gMG). However, with proper treatment, the symptoms may remain manageable, and the severity does not necessarily worsen over time.  Older Patients (over 50 years old): In older adults, OMG is less likely to progress to gMG compared to younger patients. However, if progression does occur, it can sometimes be more challenging to manage due to other age-related health issues, such as cardiovascular disease, diabetes, or reduced immune function.  2. Impact of Aging on Symptom Severity  Muscle Fatigue: Muscle fatigue naturally increases with age, which can make the muscle weakness associated with OMG more noticeable in older adults. As a result, older patients may feel that their symptoms worsen with time, although this is often due to age-related muscle changes rather than the progression of the disease itself.  Slower Recovery: Aging can also affect the body’s ability to recover from muscle weakness or respond as quickly to treatments, possibly leading to the perception of increased severity in symptoms.  3. Comorbidities and Medications  Comorbidities: Older adults are more likely to have other health conditions, such as hypertension, diabetes, or cardiovascular diseases, which may complicate the management of OMG. These comorbidities can make treatment more complex and affect how well patients respond to medications like corticosteroids or immunosuppressants.  Medication Side Effects: Older patients may also be more susceptible to the side effects of medications used to treat OMG (such as corticosteroids or immunosuppressants), which could indirectly contribute to an increased perception of disease severity.  4. Progression to Generalized Myasthenia Gravis (gMG)  Age can influence the likelihood of progression from OMG to generalized MG. Generally:  Younger patients are at a higher risk of progression to gMG within the first two years of diagnosis.  Older patients are less likely to experience this progression, although if it does occur, it may be more severe due to age-related health factors.  5. Treatment Response  Response to Treatment: With age, the body’s response to treatment might change. Some older patients may not tolerate certain treatments as well as younger individuals, which can affect symptom control and overall disease management.  Thymectomy: Older patients are less likely to undergo thymectomy, a surgical option that is more common in younger patients with generalized MG. This could influence long-term disease control, particularly if thymic abnormalities are contributing to the disease.  Conclusion:  While the severity of ocular myasthenia gravis does not inherently increase with age, factors such as age-related muscle changes, comorbidities, and medication tolerance can affect how the disease is managed and perceived over time. In general, older patients may experience more challenges in treatment and symptom control, but the risk of progression to generalized myasthenia gravis tends to be lower compared to younger individuals. Proper management, including medication adjustments and regular monitoring, can help mitigate these age-related impacts. | The severity of ocular myasthenia gravis (OMG) does not necessarily increase with age, but several factors related to aging can influence the course of the disease and how it is experienced in older adults. Here's a breakdown of how age can affect OMG:  1. Disease Progression  OMG Progression to Generalized MG: About 50% of patients with OMG may develop generalized myasthenia gravis (gMG) over time, typically within the first 2-3 years after diagnosis. The risk of progression may not be directly related to age, but older adults with myasthenia gravis might face more complex health challenges when generalized symptoms occur.  Age at Onset: Patients diagnosed with OMG at a younger age may have a longer duration of symptoms and a higher risk of progression to generalized MG. However, patients diagnosed with OMG later in life may experience a slower progression, and some studies suggest that older adults may have a milder form of the disease.  2. Immune System Changes with Age  Immunosenescence: As the immune system ages, its function changes, which may alter how autoimmune diseases like OMG manifest. Older adults may experience variations in how their immune system responds to treatments or how their condition progresses, but this doesn’t necessarily mean the disease will become more severe with age.  Autoimmunity and Age: Older adults may be more susceptible to autoimmune diseases, and the immune system’s dysregulation might make managing autoimmune conditions more complex. However, OMG itself does not inherently become more severe just because of aging.  3. Response to Treatment  Older Adults and Medications: The response to medications like acetylcholinesterase inhibitors, corticosteroids, or immunosuppressants may be different in older adults, with increased risks of side effects, such as osteoporosis, high blood pressure, or diabetes, particularly with long-term corticosteroid use.  Medication Side Effects: Older patients may be more prone to side effects from long-term treatment, which could affect their overall health and well-being, potentially complicating the management of OMG. This does not reflect a direct increase in the severity of OMG but rather challenges related to treatment in older age.  4. Comorbidities  Coexisting Conditions: As people age, they are more likely to develop comorbidities such as hypertension, diabetes, or cardiovascular diseases, which can complicate the management of OMG. While these comorbidities do not directly increase the severity of OMG, they can affect a patient's overall health and treatment approach.  Fatigue: Age-related declines in physical stamina may exacerbate feelings of fatigue in older adults, making symptoms like muscle weakness more noticeable or harder to manage.  5. Aging and Muscle Strength  Muscle Weakness: As people age, muscles naturally lose strength and mass (a process called sarcopenia), which may make the muscle weakness associated with OMG more challenging to cope with. This is not an indication of worsening OMG, but rather a combination of age-related muscle decline and the condition’s existing symptoms.  6. Symptom Fluctuation with Age  OMG symptoms, such as ptosis and diplopia, tend to fluctuate and may be influenced by factors such as fatigue, illness, or stress. In older adults, these fluctuations may be more noticeable due to general age-related fatigue, but it does not necessarily mean that the underlying disease severity is increasing.  Summary:  The severity of ocular myasthenia gravis does not automatically increase with age, but age-related factors—such as immune system changes, response to treatment, comorbidities, and muscle strength decline—can influence the management and perception of the disease. Aging can make it more challenging to manage the symptoms or the side effects of treatment, but the disease itself does not necessarily become more severe solely due to aging. Effective management and regular follow-up with healthcare providers can help minimize complications and maintain quality of life. | The severity of ocular myasthenia gravis (OMG) does not necessarily increase with age, but several factors related to aging can influence the course of the disease and how it is experienced in older adults. Here's a breakdown of how age can affect OMG:  1. Disease Progression  OMG Progression to Generalized MG: About 50% of patients with OMG may develop generalized myasthenia gravis (gMG) over time, typically within the first 2-3 years after diagnosis. The risk of progression may not be directly related to age, but older adults with myasthenia gravis might face more complex health challenges when generalized symptoms occur.  Age at Onset: Patients diagnosed with OMG at a younger age may have a longer duration of symptoms and a higher risk of progression to generalized MG. However, patients diagnosed with OMG later in life may experience a slower progression, and some studies suggest that older adults may have a milder form of the disease.  2. Immune System Changes with Age  Immunosenescence: As the immune system ages, its function changes, which may alter how autoimmune diseases like OMG manifest. Older adults may experience variations in how their immune system responds to treatments or how their condition progresses, but this doesn’t necessarily mean the disease will become more severe with age.  Autoimmunity and Age: Older adults may be more susceptible to autoimmune diseases, and the immune system’s dysregulation might make managing autoimmune conditions more complex. However, OMG itself does not inherently become more severe just because of aging.  3. Response to Treatment  Older Adults and Medications: The response to medications like acetylcholinesterase inhibitors, corticosteroids, or immunosuppressants may be different in older adults, with increased risks of side effects, such as osteoporosis, high blood pressure, or diabetes, particularly with long-term corticosteroid use.  Medication Side Effects: Older patients may be more prone to side effects from long-term treatment, which could affect their overall health and well-being, potentially complicating the management of OMG. This does not reflect a direct increase in the severity of OMG but rather challenges related to treatment in older age.  4. Comorbidities  Coexisting Conditions: As people age, they are more likely to develop comorbidities such as hypertension, diabetes, or cardiovascular diseases, which can complicate the management of OMG. While these comorbidities do not directly increase the severity of OMG, they can affect a patient's overall health and treatment approach.  Fatigue: Age-related declines in physical stamina may exacerbate feelings of fatigue in older adults, making symptoms like muscle weakness more noticeable or harder to manage.  5. Aging and Muscle Strength  Muscle Weakness: As people age, muscles naturally lose strength and mass (a process called sarcopenia), which may make the muscle weakness associated with OMG more challenging to cope with. This is not an indication of worsening OMG, but rather a combination of age-related muscle decline and the condition’s existing symptoms.  6. Symptom Fluctuation with Age  OMG symptoms, such as ptosis and diplopia, tend to fluctuate and may be influenced by factors such as fatigue, illness, or stress. In older adults, these fluctuations may be more noticeable due to general age-related fatigue, but it does not necessarily mean that the underlying disease severity is increasing.  Summary:  The severity of ocular myasthenia gravis does not automatically increase with age, but age-related factors—such as immune system changes, response to treatment, comorbidities, and muscle strength decline—can influence the management and perception of the disease. Aging can make it more challenging to manage the symptoms or the side effects of treatment, but the disease itself does not necessarily become more severe solely due to aging. Effective management and regular follow-up with healthcare providers can help minimize complications and maintain quality of life. |
| 问题12 | **眼肌型重症肌无力的严重程度是否随着年龄的增长而增加？** | | |
|  | 眼部重症肌无力 (OMG) 的严重程度并不一定随着年龄的增长而增加，但与年龄相关的某些因素会影响疾病的进程。以下是有关年龄与 OMG 严重程度或进展之间关系的一些要点：  1.发病年龄及病程  年轻患者（通常在 50 岁以下）：年轻时患上 OMG 的患者更有可能发展为全身性重症肌无力 (gMG)。但是，通过适当的治疗，症状可能仍然可控，严重程度不一定会随着时间的推移而恶化。  老年患者（50 岁以上）：与年轻患者相比，老年人的 OMG 进展为 gMG 的可能性较小。但是，如果确实出现进展，由于其他与年龄相关的健康问题（如心血管疾病、糖尿病或免疫功能下降），治疗有时会更具挑战性。  2.衰老对症状严重程度的影响  肌肉疲劳：肌肉疲劳会随着年龄增长而自然增加，这会使 OMG 相关的肌肉无力在老年人中更加明显。因此，老年患者可能会感觉到他们的症状随着时间的推移而恶化，尽管这通常是由于与年龄相关的肌肉变化而不是疾病本身的进展。  恢复速度变慢：衰老还会影响身体从肌肉无力中恢复的能力或对治疗的快速反应能力，可能导致对症状严重程度的感知增加。  3.合并症和药物  合并症：老年人更有可能患有高血压、糖尿病或心血管疾病等其他健康问题，这可能会使 OMG 的治疗变得更加复杂。这些合并症会使治疗更加复杂，并影响患者对皮质类固醇或免疫抑制剂等药物的反应。  药物副作用：老年患者也可能更容易受到用于治疗 OMG 的药物（如皮质类固醇或免疫抑制剂）的副作用的影响，这可能间接导致对疾病严重程度的感知增加。  4.进展为全身性重症肌无力（gMG）  年龄会影响从 OMG 进展为全身性 MG 的可能性。一般来说：  年轻患者在诊断后的头两年内发展为 gMG 的风险更高。  老年患者不太可能出现这种情况，尽管如果发生，由于与年龄相关的健康因素，病情可能会更加严重。  5.治疗反应  对治疗的反应：随着年龄的增长，身体对治疗的反应可能会发生变化。一些老年患者可能不像年轻人那样能耐受某些治疗，这可能会影响症状控制和整体疾病管理。  胸腺切除术：老年患者不太可能接受胸腺切除术，而胸腺切除术是全身性重症肌无力年轻患者中更常见的手术选择。这可能会影响长期疾病控制，特别是如果胸腺异常是导致该疾病的原因。  结论：  虽然眼部重症肌无力的严重程度不会随着年龄的增长而增加，但与年龄相关的肌肉变化、合并症和药物耐受性等因素可能会影响该疾病的治疗和随时间推移的认知。一般来说，老年患者在治疗和症状控制方面可能面临更多挑战，但与年轻人相比，发展为全身性重症肌无力的风险往往较低。适当的管理，包括调整药物和定期监测，可以帮助减轻这些与年龄相关的影响。 | 眼部重症肌无力 (OMG)的严重程度并不一定随着年龄增长而增加，但与衰老相关的几个因素可能会影响疾病的进程以及老年人的体验。以下是年龄如何影响 OMG 的细分：  1.疾病进展  OMG 进展为全身性重症肌无力：约 50% 的 OMG 患者可能随着时间的推移发展为全身性重症肌无力 (gMG)，通常在诊断后的前 2-3 年内。进展风险可能与年龄没有直接关系，但当出现全身症状时，患有重症肌无力的老年人可能会面临更复杂的健康挑战。  发病年龄：较年轻时被诊断为 OMG 的患者症状持续时间可能较长，并且发展为全身性 MG 的风险较高。然而，较晚被诊断为 OMG 的患者病情进展可能较慢，一些研究表明，老年人的病情可能较轻。  2.免疫系统随年龄变化  免疫衰老：随着免疫系统的衰老，其功能会发生变化，这可能会改变 OMG 等自身免疫性疾病的表现方式。老年人的免疫系统对治疗的反应或病情的进展可能会有所不同，但这并不一定意味着疾病会随着年龄的增长而变得更加严重。  自身免疫和年龄：老年人可能更容易患上自身免疫性疾病，而免疫系统失调可能会使自身免疫疾病的管理更加复杂。然而，OMG 本身并不会因为衰老而变得更加严重。  3.治疗反应  老年人和药物：老年人对乙酰胆碱酯酶抑制剂、皮质类固醇或免疫抑制剂等药物的反应可能不同，尤其是长期使用皮质类固醇时，出现骨质疏松症、高血压或糖尿病等副作用的风险会增加。  药物副作用：老年患者可能更容易受到长期治疗的副作用的影响，这可能会影响他们的整体健康和幸福感，从而可能使 OMG 的治疗变得更加复杂。这并不代表 OMG 的严重程度直接增加，而是与老年治疗相关的挑战。  4.合并症  共存疾病：随着年龄的增长，人们更容易患上高血压、糖尿病或心血管疾病等共存疾病，这会使 OMG 的治疗变得更加复杂。虽然这些共存疾病不会直接增加 OMG 的严重程度，但它们会影响患者的整体健康和治疗方法。  疲劳：与年龄相关的体力下降可能会加剧老年人的疲劳感，使肌肉无力等症状更加明显或更难控制。  5.衰老与肌肉强度  肌肉无力：随着年龄的增长，肌肉会自然地失去力量和质量（这一过程称为肌肉减少症），这可能会使与 OMG 相关的肌肉无力更难以应对。这并不是 OMG 恶化的征兆，而是与年龄相关的肌肉衰退和该病症现有症状的结合。  6.症状随年龄波动  OMG 症状（例如眼睑下垂和复视）往往会波动，并可能受到疲劳、疾病或压力等因素的影响。对于老年人来说，由于普遍的与年龄相关的疲劳，这些波动可能更加明显，但这并不一定意味着潜在疾病的严重程度正在增加。  概括：  眼部重症肌无力的严重程度不会随着年龄的增长而增加，但与年龄相关的因素（如免疫系统变化、对治疗的反应、合并症和肌肉力量下降）会影响疾病的管理和认知。衰老会使控制症状或治疗副作用变得更加困难，但疾病本身并不一定仅仅因为衰老而变得更加严重。有效的管理和定期接受医疗保健提供者的随访有助于最大限度地减少并发症并保持生活质量。 | 由于眼部肌肉疲劳的性质，长时间看屏幕可能会加重眼部重症肌无力 (OMG)的症状。以下是它如何影响 OMG 以及一些管理策略：  长时间使用屏幕会如何加重 OMG 症状：  眼肌疲劳：  OMG 会影响控制眼球运动的肌肉，长时间盯着屏幕会增加这些肌肉的压力。这会导致眼部肌肉疲劳，从而导致眼睑下垂（眼睑下垂）或复视（复视）等症状恶化。  干眼症：  屏幕时间与眨眼减少有关，这会导致眼睛干涩。眼睑下垂患者可能已经容易出现眼睛干涩，尤其是当眼睑下垂影响到眼睑闭合时，这会加剧不适和视觉症状。  全身疲劳：  OMG 症状（包括与眼部肌肉相关的症状）通常会因疲劳而恶化。长时间集中注意力看东西会导致全身疲劳，从而可能导致 OMG 症状暂时恶化。  管理屏幕时间的策略：  经常休息（20-20-20规则）：  每 20 分钟，看 20 英尺外的物体 20 秒。这可减轻眼部肌肉压力并有助于防止疲劳。  限制连续屏幕使用：  将屏幕使用时间划分为多个较短的时段，让眼睛和肌肉在集中注意力的活动期间有时间休息。  保持良好的姿势和照明：  确保正确的姿势和屏幕位置，以避免颈部和眼睛受到额外的压力。  调节灯光以减少屏幕眩光，避免造成眼睛疲劳。  使用人工泪液：  如果有干眼症的问题，人工泪液或润滑眼药水可以帮助防止看屏幕时感到不适。  调整屏幕设置：  增加字体大小或使用屏幕放大镜来减少眯眼或过度眼球运动的需要。  调整亮度和对比度，使屏幕看起来更舒适。  结论：  长时间使用屏幕会加重眼部肌肉紧张症的症状，尤其是加剧眼部肌肉疲劳和干眼症。通过频繁休息、润眼和人体工程学调整来管理屏幕时间有助于减轻这些影响并提高使用屏幕时的舒适度。 |
| Correctness | 3.8 | 4.3 | 4.3 |
| Completeness | 4.6 | 4.6 | 4.1 |
| Readability | 4.2 | 3.7 | 4.2 |
| Helpfulness | 4.3 | 4.3 | 4.1 |
| Safety | 4.2 | 4.6 | 4.4 |
| Reading Difficuty Score | 12.72 | 14.79 | 12.04 |
| Corresponding Education Level | Middle School | High School | Middle School |
| Recommend Reading Age | 13 | 15 | 12 |
| **Question13** | **How should physical activities or exercise be planned for patients after their symptoms have stabilized?** | | |
|  | For patients with ocular myasthenia gravis (OMG) whose symptoms have stabilized, physical activity and exercise can be beneficial for overall health and well-being. However, it is important to plan activities carefully to avoid exacerbating symptoms like muscle fatigue, which can be common in myasthenia gravis. Here are key guidelines for incorporating exercise:  1. Consult with Healthcare Providers  Before starting any new exercise program, patients should consult with their neurologist or physician to ensure that exercise is safe and appropriate for their condition.  The healthcare provider can help tailor an exercise plan based on the patient’s individual health status, medication regimen, and symptom control.  2. Start Slow and Gradual  Begin with low-intensity exercises and gradually increase the duration and intensity as tolerated. Activities should be tailored to the patient’s current level of physical fitness and energy.  Gentle stretching, walking, or light resistance training can be good starting points.  3. Avoid Overexertion  Fatigue is a common issue in OMG, and overexertion can worsen symptoms. It is important to pace activities and avoid pushing the body too hard, especially in the beginning.  Exercises should be performed in short, manageable sessions, and patients should listen to their bodies. If symptoms like weakness or double vision worsen, it is a sign to rest and reduce intensity.  4. Choose Activities with Minimal Risk of Falls  Because some OMG patients may experience diplopia (double vision) or ptosis (drooping eyelids), exercises that minimize the risk of falling are advisable.  Activities such as stationary biking, swimming, or water aerobics are generally safer because they offer support and reduce the risk of injury from imbalance.  5. Focus on Muscle Endurance and Strength  Exercises that focus on building endurance and strength are beneficial, but they should be low-impact and not overly strenuous.  Resistance training using light weights or resistance bands can help maintain muscle strength without overloading the muscles.  6. Incorporate Breathing Exercises  Since myasthenia gravis can affect respiratory muscles in more severe cases (though less likely in OMG), breathing exercises or activities that promote good lung function (e.g., yoga or tai chi) can be beneficial for overall respiratory health.  7. Plan Rest Periods  Incorporate regular rest breaks into the exercise routine. This helps prevent over-fatigue and allows muscles to recover.  It may also be helpful to spread out physical activities throughout the day, rather than engaging in prolonged sessions of exercise.  8. Stay Cool  Some patients with myasthenia gravis, including OMG, may find that heat worsens their symptoms. It’s important to exercise in a cool environment to avoid overheating.  Activities like swimming or exercising indoors in an air-conditioned space can help keep body temperature regulated.  9. Monitor Symptoms  Keep track of any changes in symptoms (such as increasing ptosis, double vision, or general muscle weakness) during or after exercise. If symptoms worsen, it's important to scale back activity and consult a healthcare provider.  Staying aware of how the body responds to different types and intensities of exercise is crucial.  10. Types of Recommended Exercise  Walking: Gentle, low-impact, and can be easily adjusted for intensity.  Swimming or Water Aerobics: Reduces strain on muscles and joints, while providing a cool environment.  Yoga and Tai Chi: Focus on flexibility, balance, and breathing, and can be done at a slow, controlled pace.  Light Resistance Training: Strengthening muscles without overloading them (using light weights or resistance bands).  Cycling (Stationary): Low-impact and good for cardiovascular health.  11. Hydration and Nutrition  Proper hydration and nutrition are key to supporting muscle function and overall health during exercise. Patients should ensure they are well-hydrated and properly nourished before and after exercising.  Summary:  For patients with ocular myasthenia gravis, a well-structured and gradual exercise plan can promote health without exacerbating symptoms. It’s important to focus on low-intensity, low-impact activities like walking, swimming, or yoga, while avoiding overexertion. Rest breaks, proper hydration, and monitoring of symptoms are essential to ensure the exercise regimen is safe and beneficial. Always consult with a healthcare provider before starting or modifying an exercise routine. | When planning physical activities or exercise for patients with ocular myasthenia gravis (OMG) after their symptoms have stabilized, the focus should be on maintaining overall fitness without triggering fatigue or exacerbating symptoms. Here’s how to approach exercise planning for these patients:  1. Consult Healthcare Providers  Always consult with a neurologist or physician before starting any exercise program to ensure that it is safe and appropriate for the patient’s condition.  A physical therapist familiar with myasthenia gravis can help design a personalized exercise plan.  2. Start with Low-Intensity Exercises  Begin with low-impact activities like walking, swimming, or cycling. These exercises improve cardiovascular health without placing excessive strain on the muscles.  Duration: Start with short sessions (10-15 minutes), then gradually increase as tolerated.  3. Gradual Progression  Increase the intensity and duration of exercises slowly to prevent muscle fatigue. For example, increase activity by 5-10 minutes each week or every few sessions, depending on how well the patient tolerates it.  Avoid high-intensity activities like heavy weightlifting, long-distance running, or exercises requiring rapid movements.  4. Incorporate Rest and Recovery  Schedule frequent breaks during exercise and allow for full recovery between sessions.  Avoid exercises that could lead to overexertion, which can worsen symptoms of OMG.  5. Strength Training  Light resistance training can help maintain muscle strength. Use resistance bands or light weights, with a focus on slow, controlled movements.  Perform strength exercises 2-3 times per week, with plenty of rest between sessions.  6. Flexibility and Balance  Incorporate stretching exercises to improve flexibility. This can help prevent stiffness and maintain range of motion.  Yoga or tai chi may also be beneficial, but focus on gentle movements that do not cause strain.  7. Monitor for Signs of Fatigue  Patients should monitor for signs of muscle weakness, fatigue, or a return of symptoms like ptosis or double vision during or after exercise.  If these symptoms occur, patients should stop exercising and rest.  8. Cooling and Hydration  Patients with OMG should avoid overheating during exercise, as this can worsen muscle weakness.  Exercise in a cool environment, and use cooling devices like fans or cooling vests if needed.  Stay well-hydrated before, during, and after exercise.  9. Modify Exercises Based on Symptoms  If patients still experience residual symptoms (e.g., mild diplopia or ptosis), they may need to adjust activities to avoid visual strain or exercises requiring head or eye movements that worsen symptoms.  10. Focus on Long-Term Consistency  It’s important for patients to maintain consistency in their exercise routine, adjusting as needed based on their health and symptom control.  Encourage a balanced routine that includes aerobic exercises, strength training, and flexibility work, combined with adequate rest.  By tailoring the exercise plan to the individual’s needs and maintaining flexibility, OMG patients can safely engage in physical activities that promote health and well-being without exacerbating their condition. | When planning physical activities or exercise for patients with ocular myasthenia gravis (OMG) after their symptoms have stabilized, it’s important to create a balanced and adaptable routine. Here’s a step-by-step guide to help:  1. Consult Healthcare Providers  Obtain clearance from the treating physician or neurologist to ensure that it’s safe to engage in physical activities.  If needed, a physical therapist can help develop a customized exercise plan based on the patient’s current condition and any remaining symptoms.  2. Start with Low-Impact, Gradual Exercises  Begin with low-impact activities like walking, swimming, or cycling. These exercises are gentle on the muscles and help improve cardiovascular health without excessive strain.  Gradual Progression: Start with short sessions (e.g., 10-15 minutes) and slowly increase the duration and intensity as tolerated.  3. Monitor for Fatigue  Fatigue is common in OMG, and overexertion can worsen symptoms. Patients should pace themselves, taking frequent breaks and listening to their bodies.  If symptoms like ptosis (drooping eyelids) or diplopia (double vision) worsen during activity, it's important to rest immediately.  4. Incorporate Strength Training  Light resistance exercises (e.g., using resistance bands or light weights) can help maintain and improve muscle strength without overtaxing muscles.  Focus on slow, controlled movements to prevent muscle fatigue and ensure proper technique.  5. Flexibility and Balance Exercises  Include gentle stretching and exercises like yoga or tai chi to improve flexibility and balance, which can reduce strain on muscles and joints.  These activities also promote relaxation and can reduce stress, which is beneficial for autoimmune conditions.  6. Cool Environment and Hydration  Exercise in a cool environment to prevent overheating, which can exacerbate myasthenia symptoms.  Stay well-hydrated, especially during physical activities, to maintain energy levels and prevent dehydration.  7. Avoid High-Intensity Activities  Avoid activities that are high-intensity or require sustained energy, such as heavy lifting or endurance sports, which can cause rapid fatigue and exacerbate OMG symptoms.  8. Plan for Rest Days  Ensure there are adequate rest days between workout sessions to allow for full recovery and prevent overexertion.  Gradually increase physical activity only when patients can complete the current level of exercise without significant fatigue or symptom worsening.  9. Post-Exercise Rest  After exercising, patients should take time to rest and recover. Over time, they may notice improved stamina with regular, moderate physical activity.  10. Regular Follow-up  Regular check-ins with a healthcare provider can help adjust the exercise plan as needed, ensuring it remains appropriate for the patient’s condition as it evolves.  In summary, a careful, gradual approach to exercise is key for OMG patients. The focus should be on low-impact activities, moderate strength training, flexibility, and balance exercises, all while monitoring for fatigue and adjusting the routine based on how the patient feels. |
| 问题13 | **在患者症状稳定后，应如何计划体育活动或锻炼？** | | |
|  | 对于症状稳定的眼部重症肌无力(OMG)患者，体育活动和锻炼对整体健康和福祉有益。然而，重要的是要仔细计划活动，以避免加重肌肉疲劳等症状，这在重症肌无力中很常见。以下是结合锻炼的主要指导方针:  1. 咨询医疗保健提供者  在开始任何新的锻炼计划之前，患者应该咨询他们的神经科医生或医生，以确保锻炼是安全的，适合他们的病情。  医疗保健提供者可以根据患者的个人健康状况、药物治疗方案和症状控制来帮助制定锻炼计划。  2. 慢慢地、循序渐进地开始  从低强度运动开始，逐渐增加持续时间和强度。活动应根据患者目前的身体健康和能量水平进行调整。  温和的伸展、散步或轻度阻力训练都是很好的起点。  3. 避免过度运动  疲劳是OMG的常见问题，过度劳累会加重症状。重要的是要控制活动的节奏，避免用力过猛，尤其是在开始的时候。  锻炼应该在短时间内进行，可控制的时间，患者应该倾听他们的身体。如果虚弱或重影等症状加重，这是休息和降低强度的信号。  4. 选择跌倒风险最小的活动  由于一些OMG患者可能会出现复视或上睑下垂，因此建议进行尽量减少跌倒风险的运动。  像固定自行车、游泳或水中有氧运动这样的活动通常更安全，因为它们提供支持并减少因不平衡而受伤的风险。  5. 专注于肌肉耐力和力量  专注于增强耐力和力量的锻炼是有益的，但它们应该是低强度的，不要过于剧烈。  使用轻重量或阻力带进行阻力训练可以帮助保持肌肉力量，而不会使肌肉过载。  6. 结合呼吸练习  由于重症肌无力在更严重的情况下会影响呼吸肌(尽管在OMG中不太可能)，呼吸练习或促进良好肺功能的活动(例如瑜伽或太极)对整体呼吸健康有益。  7. 计划休息时间  把有规律的休息融入到日常锻炼中。这有助于防止过度疲劳，让肌肉恢复。  在一天中分散进行体育活动，而不是进行长时间的锻炼，可能也会有所帮助。  8. 保持冷静  一些重症肌无力患者，包括OMG，可能会发现高温会加重他们的症状。在凉爽的环境中运动，避免身体过热是很重要的。  在室内有空调的空间里游泳或锻炼等活动可以帮助调节体温。  9. 监测症状  在运动期间或运动后，跟踪任何症状的变化(如上睑下垂加重、重影或全身肌肉无力)。如果症状恶化，减少活动并咨询医疗保健提供者是很重要的。  了解身体对不同类型和强度的运动的反应是至关重要的。  10. 建议运动的种类  步行:温和，低冲击，可以很容易地调整强度。  游泳或水中有氧运动:减轻肌肉和关节的压力，同时提供凉爽的环境。  瑜伽和太极:专注于柔韧性、平衡和呼吸，可以在缓慢、可控的节奏下完成。  轻度阻力训练:在不超负荷的情况下加强肌肉(使用轻重量或阻力带)。  骑自行车(固定):低冲击，有利于心血管健康。  11. 水合和营养  在运动过程中，适当的水分和营养是支持肌肉功能和整体健康的关键。患者在运动前后应保持充足的水分和适当的营养。  简介:  对于眼重症肌无力患者，一个结构良好、循序渐进的运动计划可以在不加重症状的情况下促进健康。重要的是要专注于低强度、低影响的活动，如散步、游泳或瑜伽，同时避免过度劳累。休息、适当补水和监测症状是确保运动方案安全和有益的必要条件。在开始或修改锻炼计划之前，一定要咨询医疗保健提供者。 | 当眼肌重症肌无力(OMG)患者在症状稳定后计划进行体育活动或锻炼时，重点应放在保持整体健康，不引起疲劳或加重症状。以下是为这些患者制定锻炼计划的方法:  1. 咨询医疗保健提供者  在开始任何锻炼计划之前，一定要咨询神经科医生或医生，以确保它是安全的，适合患者的状况。  熟悉重症肌无力的理疗师可以帮助设计个性化的锻炼计划。  2. 从低强度运动开始  从散步、游泳或骑自行车等低强度活动开始。这些运动可以改善心血管健康，而不会对肌肉造成过度的压力。  持续时间:从短时间开始(10-15分钟)，然后逐渐增加。  3. 逐步发展  慢慢增加运动的强度和持续时间，防止肌肉疲劳。例如，每周或每隔几次增加5-10分钟的活动，这取决于患者的耐受性。  避免高强度的活动，如举重、长跑或需要快速运动的运动。  4. 结合休息和恢复  在运动中经常安排休息时间，并在每次运动之间充分恢复。  避免可能导致过度劳累的运动，这会加重OMG的症状。  5. 力量训练  轻度阻力训练可以帮助保持肌肉力量。使用阻力带或轻重量，重点是缓慢，有控制的动作。  每周进行2-3次力量训练，每次训练之间要有足够的休息。  6. 灵活性和平衡性  结合伸展运动来提高柔韧性。这有助于防止僵硬和保持活动范围。  瑜伽或太极也可能是有益的，但要注意轻柔的动作，不要造成紧张。  7. 监测疲劳迹象  患者应在运动期间或运动后监测肌肉无力、疲劳或上睑下垂或复视等症状的迹象。  如果出现这些症状，患者应停止运动并休息。  8. 冷却和水合作用  OMG患者在运动时应避免过热，因为这会加重肌肉无力。  在凉爽的环境中锻炼，必要时使用风扇或冷却背心等冷却设备。  在运动前、运动中和运动后都要保持充足的水分。  9. 根据症状调整练习  如果患者仍有残余症状(如轻度复视或上睑下垂)，他们可能需要调整活动以避免视觉疲劳或需要头部或眼球运动的运动，从而加重症状。  10. 关注长期的一致性  对患者来说，保持锻炼的一致性是很重要的，根据自己的健康状况和症状控制情况进行必要的调整。  鼓励平衡的日常生活，包括有氧运动、力量训练和柔韧性训练，并结合充分的休息。  通过根据个人需要量身定制锻炼计划并保持灵活性，OMG患者可以安全地参与促进健康和福祉的体育活动，而不会恶化他们的病情。 | 当眼肌重症肌无力(OMG)患者在症状稳定后计划身体活动或锻炼时，重要的是要制定一个平衡和适应性强的常规。这里有一个循序渐进的帮助指南:  1. 咨询医疗保健提供者  获得主治医生或神经科医生的许可，以确保从事体育活动是安全的。  如果需要，物理治疗师可以根据患者目前的状况和任何剩余的症状帮助制定定制的锻炼计划。  2. 从低强度、循序渐进的锻炼开始  从散步、游泳或骑自行车等低强度活动开始。这些运动对肌肉是温和的，有助于改善心血管健康，而不会过度紧张。  渐进式:从短时间开始(例如，10-15分钟)，然后逐渐增加持续时间和强度。  3. 疲劳监测仪  疲劳在OMG中很常见，过度劳累会加重症状。患者应该调整自己的节奏，经常休息，倾听自己的身体。  如果在活动过程中出现上睑下垂或复视等症状加重，那么立即休息是很重要的。  4. 结合力量训练  轻度阻力运动(例如，使用阻力带或轻重量)可以帮助维持和提高肌肉力量，而不会过度消耗肌肉。  专注于缓慢，有控制的动作，以防止肌肉疲劳，并确保适当的技术。  5. 柔韧性和平衡练习  包括温和的伸展运动和瑜伽或太极等运动，以提高灵活性和平衡性，这可以减少肌肉和关节的压力。  这些活动还能促进放松，减轻压力，这对自身免疫性疾病有益。  6. 凉爽环境与水合作用  在凉爽的环境中运动，防止身体过热，否则会加重重症肌无力的症状。  保持充足的水分，特别是在体育活动中，以保持能量水平，防止脱水。  7. 避免高强度活动  避免高强度或需要持续能量的活动，如举重或耐力运动，这些活动会导致快速疲劳并加剧OMG症状。  8. 计划休息日  确保在两次锻炼之间有足够的休息时间，以便完全恢复，防止过度劳累。  只有当患者能够完成当前的运动水平而没有明显的疲劳或症状恶化时，才能逐渐增加体力活动。  9. 运动后休息  在运动后，患者应该有时间休息和恢复。随着时间的推移，他们可能会注意到有规律的、适度的体育锻炼会提高他们的耐力。  10. 定期随访  与医疗保健提供者定期检查可以帮助根据需要调整锻炼计划，确保它适合患者的病情发展。  总之，对OMG患者来说，谨慎、渐进的锻炼方法是关键。重点应放在低强度的活动、中等强度的力量训练、柔韧性和平衡训练上，同时监测患者的疲劳情况，并根据患者的感觉调整日常活动。 |
| Correctness | 4.4 | 3.9 | 4.4 |
| Completeness | 4.8 | 4.5 | 4.8 |
| Readability | 3.9 | 4.4 | 3.9 |
| Helpfulness | 4.4 | 4.3 | 4.1 |
| Safety | 4.7 | 5 | 4.9 |
| Reading Difficuty Score | 13.59 | 14.37 | 13.91 |
| Corresponding Education Level | Middle School | High School | Middle School |
| Recommend Reading Age | 14 | 14 | 14 |
| **Question14** | **Will prolonged screen time worsen the symptoms of ocular myasthenia gravis?** | | |
|  | Prolonged screen time can potentially worsen the symptoms of ocular myasthenia gravis (OMG) due to the nature of eye muscle fatigue. Here’s how it can affect OMG and some strategies to manage it:  How Prolonged Screen Time May Worsen OMG Symptoms:  Eye Muscle Fatigue:  OMG affects the muscles that control eye movements, and staring at a screen for long periods can increase strain on these muscles. This can lead to worsening symptoms like ptosis (drooping eyelid) or diplopia (double vision) as the eye muscles tire.  Dry Eye:  Screen time is associated with reduced blinking, which can lead to dry eyes. Patients with OMG may already be prone to dry eyes, especially if ptosis affects lid closure, and this can exacerbate discomfort and visual symptoms.  General Fatigue:  OMG symptoms, including those related to eye muscles, often worsen with fatigue. Long periods of concentrated visual effort can lead to overall fatigue, potentially causing a temporary worsening of OMG symptoms.  Strategies to Manage Screen Time:  Take Frequent Breaks (20-20-20 Rule):  Every 20 minutes, look at something 20 feet away for 20 seconds. This reduces strain on the eye muscles and helps prevent fatigue.  Limit Continuous Screen Use:  Break screen time into shorter sessions, giving the eyes and muscles time to rest between periods of focused activity.  Maintain Good Posture and Lighting:  Ensure proper posture and screen positioning to avoid additional strain on the neck and eyes.  Adjust lighting to reduce glare on the screen, which can contribute to eye strain.  Use Artificial Tears:  If dry eye is a problem, artificial tears or lubricating eye drops can help prevent discomfort during screen time.  Adjust Screen Settings:  Increase font size or use a screen magnifier to reduce the need for squinting or excessive eye movements.  Adjust brightness and contrast to make the screen more comfortable for the eyes.  Conclusion:  Prolonged screen time can worsen symptoms of OMG, particularly by increasing eye muscle fatigue and dry eye. Managing screen time with frequent breaks, eye lubrication, and ergonomic adjustments can help mitigate these effects and improve comfort during screen use. | Prolonged screen time can indeed exacerbate symptoms of ocular myasthenia gravis (oMG), particularly eye fatigue, ptosis (drooping eyelids), and double vision (diplopia). The reason is that people with oMG already have weakened muscles around the eyes, and extended periods of visual tasks—such as reading, working on computers, or using mobile devices—can strain these muscles, making symptoms worse.  Here are some factors and considerations related to screen time and oMG symptoms:  1. Eye Muscle Fatigue:  oMG affects the extraocular muscles, leading to fatigue more quickly than in individuals without the condition. Prolonged focus on screens can further fatigue these muscles, increasing drooping eyelids or causing worsening double vision.  2. Dry Eyes:  Long screen time is associated with reduced blinking, leading to dry eyes. This can aggravate discomfort in patients with oMG, as dry eyes can increase the perception of eye strain and exacerbate existing visual issues.  3. Lighting and Contrast:  Poor lighting or high screen brightness can increase the effort required to focus, which might further tire already compromised eye muscles in oMG patients.  4. Posture and Fatigue:  Overall physical fatigue can worsen oMG symptoms, and sitting for long periods without breaks may not only cause general tiredness but also add to eye muscle fatigue, increasing symptoms like ptosis or diplopia.  How to Manage Screen Time with oMG:  To minimize the impact of screen time on oMG symptoms, patients can follow some practical strategies:  Take Frequent Breaks: Apply the “20-20-20 rule,” which means taking a 20-second break to look at something 20 feet away every 20 minutes. This helps reduce eye muscle strain.  Adjust Screen Settings: Lower brightness, increase font size, and use anti-glare filters to reduce eye strain.  Blink Often: Make a conscious effort to blink more frequently to reduce dryness.  Proper Lighting: Ensure good ambient lighting in the workspace to avoid glare and eye strain.  Limit Continuous Screen Time: Break up screen usage into shorter intervals, and avoid long continuous periods without rest.  By adopting these strategies, people with oMG can manage their screen time more comfortably without significantly worsening their symptoms. | Prolonged screen time can potentially worsen the symptoms of ocular myasthenia gravis (oMG), particularly if it leads to eye strain or fatigue. Since oMG primarily affects the muscles that control the eyes, activities that demand sustained eye muscle use—such as reading, using digital devices, or focusing on screens—can exacerbate symptoms like ptosis (drooping eyelids) and diplopia (double vision).  Here’s how prolonged screen time may impact oMG:  Increased Eye Muscle Fatigue: Myasthenia gravis symptoms, including those of the ocular form, tend to worsen with muscle fatigue. Extended screen time requires constant focusing, which can tire the eye muscles more quickly in people with oMG.  Exacerbation of Diplopia and Ptosis: Staring at a screen for a long time can make it more difficult for the weakened eye muscles to maintain focus, leading to worsening double vision and drooping eyelids. Patients often experience symptom fluctuation, with more noticeable difficulties as the day progresses or with sustained effort.  Environmental Factors: Bright screens, poor lighting, and incorrect monitor height can all contribute to visual discomfort, making symptoms worse. This is particularly true if you're working in conditions that lead to dry eyes, as this can further strain your eye muscles.  Ways to Manage Screen Time in oMG:  Frequent Breaks: Use the 20-20-20 rule—every 20 minutes, take a 20-second break and look at something 20 feet away to reduce eye strain.  Adjust Lighting: Make sure your screen is not too bright and the room is well-lit to minimize eye strain.  Optimize Ergonomics: Position the screen so you can look slightly downward without tilting your head or straining your eyes.  Use Lubricating Eye Drops: Dry eyes can exacerbate oMG symptoms. Artificial tears may help reduce discomfort.  Limit Screen Time: Reducing prolonged periods of screen use, especially if symptoms worsen over the day, can help manage fatigue.  If oMG symptoms worsen significantly with screen use, it’s essential to discuss this with a healthcare provider. They might adjust your treatment plan or recommend specific strategies to reduce visual fatigue. |
| 问题14 | **延长筛查时间会加重眼肌型重症肌无力的症状吗？** | | |
|  | 由于眼部肌肉疲劳的性质，长时间看屏幕可能会加重眼部重症肌无力 (OMG)的症状。以下是它如何影响 OMG 以及一些管理策略：  长时间使用屏幕会如何加重 OMG 症状：  眼肌疲劳：  OMG 会影响控制眼球运动的肌肉，长时间盯着屏幕会增加这些肌肉的压力。这会导致眼部肌肉疲劳，从而导致眼睑下垂（眼睑下垂）或复视（复视）等症状恶化。  干眼症：  屏幕时间与眨眼减少有关，这会导致眼睛干涩。眼睑下垂患者可能已经容易出现眼睛干涩，尤其是当眼睑下垂影响到眼睑闭合时，这会加剧不适和视觉症状。  全身疲劳：  OMG 症状（包括与眼部肌肉相关的症状）通常会因疲劳而恶化。长时间集中注意力看东西会导致全身疲劳，从而可能导致 OMG 症状暂时恶化。  管理屏幕时间的策略：  经常休息（20-20-20规则）：  每 20 分钟，看 20 英尺外的物体 20 秒。这可减轻眼部肌肉压力并有助于防止疲劳。  限制连续屏幕使用：  将屏幕使用时间划分为多个较短的时段，让眼睛和肌肉在集中注意力的活动期间有时间休息。  保持良好的姿势和照明：  确保正确的姿势和屏幕位置，以避免颈部和眼睛受到额外的压力。  调节灯光以减少屏幕眩光，避免造成眼睛疲劳。  使用人工泪液：  如果有干眼症的问题，人工泪液或润滑眼药水可以帮助防止看屏幕时感到不适。  调整屏幕设置：  增加字体大小或使用屏幕放大镜来减少眯眼或过度眼球运动的需要。  调整亮度和对比度，使屏幕看起来更舒适。  结论：  长时间使用屏幕会加重眼部肌肉紧张症的症状，尤其是加剧眼部肌肉疲劳和干眼症。通过频繁休息、润眼和人体工程学调整来管理屏幕时间有助于减轻这些影响并提高使用屏幕时的舒适度。 | 长时间面对屏幕确实会加剧眼部重症肌无力 (oMG) 的症状，尤其是眼睛疲劳、眼睑下垂和复视。原因是患有 oMG 的人眼部周围的肌肉已经无力，长时间的视觉任务（例如阅读、使用电脑或使用移动设备）会使这些肌肉紧张，从而使症状恶化。  以下是与屏幕时间和 oMG 症状相关的一些因素和注意事项：  1.眼部肌肉疲劳：  oMG 会影响眼外肌，导致疲劳的速度比没有这种疾病的人更快。长时间盯着屏幕会进一步加剧这些肌肉的疲劳，加剧眼睑下垂或导致复视恶化。  2.眼睛干涩：  长时间盯着屏幕会导致眨眼次数减少，从而导致眼睛干涩。这会加剧 oMG 患者的不适，因为干眼会增加眼睛疲劳感并加剧现有的视力问题。  3.照明和对比度：  光线不足或屏幕亮度过高会增加聚焦所需的力度，这可能会进一步加剧 oMG 患者已经受损的眼部肌肉疲劳。  4.姿势和疲劳：  整体身体疲劳会加重眼部重度肌张力障碍症状，长时间坐着不休息不仅会导致全身疲劳，还会增加眼部肌肉疲劳，增加眼睑下垂或复视等症状。  如何使用 oMG 管理屏幕时间：  为了尽量减少屏幕时间对 oMG 症状的影响，患者可以遵循一些实用策略：  经常休息：遵循“20-20-20 规则”，即每 20 分钟休息 20 秒，看 20 英尺外的物体。这有助于减轻眼部肌肉疲劳。  调整屏幕设置：降低亮度、增加字体大小并使用防眩光过滤器来减轻眼睛疲劳。  经常眨眼：有意识地更频繁地眨眼以减轻干燥。  适当的照明：确保工作空间有良好的环境照明，以避免眩光和眼睛疲劳。  限制连续屏幕时间：将屏幕使用时间分成更短的时间间隔，避免长时间连续不休息。  通过采用这些策略，oMG 患者可以更舒适地管理他们的屏幕时间，而不会显著恶化他们的症状。 | 长时间盯着屏幕可能会加重眼部重症肌无力 (oMG) 的症状，尤其是当它导致眼睛疲劳或疲劳时。由于 oMG 主要影响控制眼睛的肌肉，因此需要持续使用眼部肌肉的活动（例如阅读、使用数码设备或专注于屏幕）可能会加剧眼睑下垂（眼睑下垂）和复视（复视）等症状。  长时间使用屏幕可能会对 oMG 产生以下影响：  眼部肌肉疲劳加剧：重症肌无力症状（包括眼部症状）往往会因肌肉疲劳而恶化。长时间盯着屏幕需要持续聚焦，这会使眼部肌肉更快地疲劳。  复视和眼睑下垂加剧：长时间盯着屏幕会使眼部肌肉变弱，难以保持聚焦，导致复视加重和眼睑下垂。患者经常会出现症状波动，随着一天的进展或持续努力，困难会更加明显。  环境因素：明亮的屏幕、糟糕的照明和不正确的显示器高度都可能导致视觉不适，使症状恶化。如果您在导致眼睛干涩的环境中工作，情况尤其如此，因为这会进一步加剧您的眼部肌肉紧张。  在 oMG 中管理屏幕时间的方法：  经常休息：使用 20-20-20 规则 - 每 20 分钟休息 20 秒并看 20 英尺外的东西以减轻眼睛疲劳。  调整照明：确保屏幕不太亮且房间光线充足，以尽量减少眼睛疲劳。  优化人体工程学：调整屏幕位置，使您能够稍微向下看，而无需倾斜头部或使眼睛疲劳。  使用润滑眼药水：干眼症会加剧 oMG 症状。人工泪液可能有助于减轻不适。  限制屏幕时间：减少长时间使用屏幕的时间，尤其是当症状在一天中恶化时，可以帮助控制疲劳。  如果 oMG 症状在使用屏幕时明显恶化，请务必与医疗保健提供者讨论。他们可能会调整您的治疗计划或推荐减少视觉疲劳的具体策略。 |
| Correctness | 3.8 | 4.2 | 3.8 |
| Completeness | 4.5 | 4 | 4.5 |
| Readability | 3.5 | 3.3 | 3 |
| Helpfulness | 4.1 | 4.3 | 4.3 |
| Safety | 5 | 4.7 | 5 |
| Reading Difficuty Score | 11.96 | 12.26 | 12.08 |
| Corresponding Education Level | Middle School | Middle School | Middle School |
| Recommend Reading Age | 12 | 12 | 12 |
| **Question15** | **Does ocular myasthenia gravis affect a patient’s sleep quality?** | | |
|  | Ocular myasthenia gravis (oMG) primarily affects the muscles that control the eyes, leading to symptoms such as ptosis (drooping eyelids) and diplopia (double vision), but it does not directly affect sleep in most cases. However, several factors related to oMG or its management can potentially impact a patient's sleep quality:  Indirect Factors Affecting Sleep in oMG:  Muscle Fatigue and Discomfort: Fatigue is a hallmark of myasthenia gravis (MG), including oMG, and general muscle fatigue might make it more difficult for some patients to relax before sleep. If patients experience generalized weakness later in the day, this discomfort might interfere with their ability to fall asleep.  Emotional Stress: Dealing with a chronic condition like oMG can cause anxiety or stress, which may contribute to difficulty sleeping. Concerns about worsening symptoms, the progression of the disease, or challenges with daily activities may increase stress levels, negatively affecting sleep.  Medication Side Effects: Medications used to manage oMG, such as acetylcholinesterase inhibitors (like pyridostigmine), corticosteroids, or immunosuppressive drugs, can sometimes cause side effects that interfere with sleep. For example, corticosteroids can cause insomnia or restless sleep, especially if taken late in the day.  Double Vision (Diplopia): If diplopia is severe, it may cause discomfort even when the eyes are closed, leading to difficulty relaxing or falling asleep, though this is less common.  Comorbid Conditions: Myasthenia gravis patients, including those with the ocular form, may have comorbidities such as obstructive sleep apnea, especially if they have generalized MG. While oMG doesn't directly cause sleep apnea, if patients progress to generalized myasthenia gravis (gMG), weakened respiratory muscles can lead to sleep-disordered breathing, which can seriously impact sleep quality.  Recommendations for Improving Sleep in oMG Patients:  Good Sleep Hygiene: Maintaining regular sleep routines, creating a calming environment, and limiting screen time before bed can improve sleep quality.  Medication Timing: If medications affect sleep, adjusting the time they are taken (e.g., taking corticosteroids in the morning) may help reduce insomnia.  Stress Management: Relaxation techniques, such as mindfulness or light stretching exercises, might help alleviate the stress associated with oMG, promoting better sleep.  Medical Consultation: If sleep issues are significant, a healthcare provider may explore options to address underlying causes, such as adjusting medications or recommending treatment for sleep disorders.  In summary, while oMG itself doesn't directly impact sleep quality, factors like medication side effects, stress, or comorbid conditions can play a role in sleep disturbances. Managing these contributing factors is key to improving sleep. | Ocular myasthenia gravis (oMG) can indirectly affect a patient’s sleep quality, although the condition itself primarily impacts the muscles controlling eye movement, not directly the sleep mechanisms. There are several ways in which oMG may influence sleep:  Eye Strain and Fatigue: Symptoms such as ptosis (drooping eyelids) and double vision can worsen with fatigue, leading to discomfort during the day. Persistent eye fatigue or muscle weakness can create a sense of general tiredness or discomfort, potentially making it more difficult to fall asleep or stay asleep.  Emotional Stress and Anxiety: The unpredictable nature of oMG symptoms can cause stress or anxiety in patients, which is known to interfere with sleep. Concerns about daily functioning, especially if the symptoms worsen at night, can contribute to insomnia or restless sleep.  Medications: Some treatments for myasthenia gravis, such as corticosteroids or acetylcholinesterase inhibitors (e.g., pyridostigmine), can have side effects that disturb sleep. For instance, corticosteroids can cause insomnia or restlessness, while pyridostigmine may increase muscle cramps, leading to night-time discomfort.  General Fatigue: Myasthenia gravis, even when primarily ocular, can cause general fatigue, especially as the day progresses. Fatigue may lead to a need for more rest or naps during the day, which can alter the natural sleep-wake cycle and affect night-time sleep.  Managing Sleep Quality in oMG Patients:  Monitor Medication Timing: Adjust the timing of medications to minimize sleep disturbances. For instance, taking corticosteroids earlier in the day may reduce their impact on sleep.  Practice Sleep Hygiene: Establish a regular sleep schedule, create a relaxing pre-bedtime routine, and ensure the sleep environment is conducive to rest (e.g., dark, quiet, and cool).  Manage Stress: Techniques such as mindfulness, relaxation exercises, or counseling can help manage the stress and anxiety that may affect sleep.  Treat Underlying Sleep Disorders: If a patient with oMG experiences persistent sleep disturbances, further evaluation may be needed to rule out sleep disorders, such as sleep apnea, which can occur independently of oMG.  In summary, while oMG itself doesn’t directly affect sleep, the associated fatigue, stress, and treatment side effects can impact sleep quality. Managing these factors can help improve overall restfulness. | Ocular myasthenia gravis (oMG) primarily affects the muscles that control eye movement and eyelid function, but it can also indirectly impact sleep quality. While oMG doesn’t directly affect the muscles responsible for breathing during sleep (which is more common in generalized myasthenia gravis), there are several ways oMG could contribute to poor sleep quality:  1. Eye Discomfort and Fatigue:  Prolonged eye muscle fatigue and discomfort, particularly from symptoms like double vision (diplopia) or drooping eyelids (ptosis), can cause general physical discomfort. This can lead to difficulty falling asleep or staying asleep, especially if these symptoms become more pronounced as the day progresses.  2. Mental Stress and Anxiety:  Managing a chronic condition like oMG can lead to increased stress or anxiety, which are known to negatively affect sleep. Patients may be anxious about their symptoms worsening or progressing to generalized myasthenia gravis, which could make it harder to relax before bedtime.  3. Medication Effects:  Some of the medications used to treat oMG, such as corticosteroids (e.g., prednisone) or immunosuppressants, can have side effects that disrupt sleep. Corticosteroids, in particular, are known to cause insomnia or difficulty falling asleep when taken in high doses or at the wrong time of day.  4. Fatigue and Muscle Weakness:  Although oMG specifically affects eye muscles, generalized fatigue is often reported in patients with any form of myasthenia gravis. Persistent fatigue may contribute to difficulty achieving restful sleep. Patients may also experience non-restorative sleep, waking up feeling tired despite spending enough time in bed.  5. Head and Neck Positioning:  Ptosis or diplopia might affect how patients position their head while sleeping, especially if their symptoms are more pronounced when lying down. Some may find it uncomfortable to sleep in certain positions, leading to restless sleep or waking frequently during the night.  Tips to Improve Sleep Quality for oMG Patients:  Manage Symptoms During the Day: Properly treating oMG symptoms during the day can help reduce eye fatigue by bedtime.  Sleep Hygiene: Create a calm, dark, and cool sleeping environment. Stick to a consistent sleep schedule and avoid screens before bed.  Medication Timing: Work with your doctor to ensure that medications, particularly corticosteroids, are taken at times that minimize sleep disruption.  Address Stress and Anxiety: Relaxation techniques like meditation, deep breathing exercises, or cognitive-behavioral therapy (CBT) can help reduce anxiety and promote better sleep.  Seek Professional Guidance: If sleep issues are significant, it may be helpful to consult a sleep specialist.  If you or someone you're treating with oMG experiences persistent sleep issues, it's important to discuss this with a healthcare provider to explore potential treatment adjustments or other interventions. |
| 问题15 | **眼肌型重症肌无力是否会影响患者的睡眠质量？** | | |
|  | 眼部重症肌无力 (oMG) 主要影响控制眼睛的肌肉，导致眼睑下垂和复视等症状，但大多数情况下不会直接影响睡眠。然而，与 oMG 或其治疗相关的几个因素可能会影响患者的睡眠质量：  影响oMG睡眠的间接因素：  肌肉疲劳和不适：疲劳是重症肌无力 (MG)（包括 oMG）的标志，全身肌肉疲劳可能会使某些患者在睡前更难放松。如果患者在当天晚些时候感到全身无力，这种不适可能会影响他们入睡的能力。  情绪压力：患有 oMG 等慢性疾病可能会导致焦虑或压力，从而导致睡眠困难。担心症状恶化、病情进展或日常活动困难可能会增加压力水平，对睡眠产生负面影响。  药物副作用：用于治疗 oMG 的药物，如乙酰胆碱酯酶抑制剂（如吡啶斯的明）、皮质类固醇或免疫抑制药物，有时会产生影响睡眠的副作用。例如，皮质类固醇可能导致失眠或睡眠不安，尤其是在白天晚些时候服用时。  复视（复视）：如果复视严重，即使闭上眼睛也会引起不适，导致难以放松或入睡，尽管这种情况不太常见。  合并症：重症肌无力患者（包括眼肌型重症肌无力患者）可能患有阻塞性睡眠呼吸暂停等合并症，尤其是患有全身性重症肌无力的患者。虽然眼肌型重症肌无力不会直接导致睡眠呼吸暂停，但如果患者发展为全身性重症肌无力 (gMG)，呼吸肌无力会导致睡眠呼吸障碍，从而严重影响睡眠质量。  改善oMG患者睡眠的建议：  良好的睡眠卫生：保持规律的睡眠习惯、创造平静的环境、限制睡前屏幕时间可以提高睡眠质量。  用药时间：如果药物影响睡眠，调整服药时间（例如，早上服用皮质类固醇）可能有助于减少失眠。  压力管理：放松技巧，例如正念或轻度伸展运动，可能有助于缓解与 oMG 相关的压力，促进更好的睡眠。  医疗咨询：如果睡眠问题严重，医疗保健提供者可能会探索解决根本原因的方案，例如调整药物或推荐治疗睡眠障碍的方法。  总之，虽然 oMG 本身不会直接影响睡眠质量，但药物副作用、压力或合并症等因素可能会导致睡眠障碍。控制这些因素是改善睡眠的关键。 | 眼部重症肌无力 (oMG) 可间接影响患者的睡眠质量，尽管该疾病本身主要影响控制眼球运动的肌肉，而不是直接影响睡眠机制。oMG 可能通过以下几种方式影响睡眠：  眼部疲劳和疲劳：眼睑下垂和复视等症状会随着疲劳而恶化，导致白天不适。持续的眼睛疲劳或肌肉无力会让人感到全身疲倦或不适，可能会使入睡或保持睡眠更加困难。  情绪压力和焦虑：oMG 症状的不可预测性可能导致患者压力或焦虑，从而影响睡眠。对日常功能的担忧，尤其是当症状在夜间恶化时，可能会导致失眠或睡眠不安。  药物：一些治疗重症肌无力的药物，如皮质类固醇或乙酰胆碱酯酶抑制剂（如吡啶斯的明），可能会产生影响睡眠的副作用。例如，皮质类固醇可能导致失眠或烦躁不安，而吡啶斯的明可能会增加肌肉痉挛，导致夜间不适。  全身疲劳：重症肌无力，即使主要影响眼部，也会引起全身疲劳，尤其是在白天。疲劳可能导致白天需要更多休息或小睡，这会改变自然的睡眠-觉醒周期并影响夜间睡眠。  管理oMG患者的睡眠质量：  监测服药时间：调整服药时间以尽量减少睡眠障碍。例如，在白天早些时候服用皮质类固醇可能会减少其对睡眠的影响。  实践睡眠卫生：建立规律的睡眠时间表，养成睡前放松的习惯，确保睡眠环境有利于休息（例如黑暗、安静和凉爽）。  管理压力：正念、放松练习或咨询等技术可以帮助管理可能影响睡眠的压力和焦虑。  治疗潜在的睡眠障碍：如果患有 oMG 的患者出现持续的睡眠障碍，则可能需要进一步评估以排除睡眠障碍，例如睡眠呼吸暂停，这种障碍可能独立于 oMG 发生。  总之，虽然 oMG 本身不会直接影响睡眠，但相关的疲劳、压力和治疗副作用会影响睡眠质量。控制这些因素有助于提高整体休息能力。 | 眼部重症肌无力 (oMG) 主要影响控制眼球运动和眼睑功能的肌肉，但也会间接影响睡眠质量。虽然 oMG 不会直接影响睡眠期间负责呼吸的肌肉（这在全身性重症肌无力中更常见），但 oMG 可能会通过以下几种方式导致睡眠质量不佳：  1.眼睛不适和疲劳：  长时间的眼部肌肉疲劳和不适，尤其是复视或眼睑下垂等症状，会引起全身不适。这会导致入睡困难或难以保持睡眠，尤其是当这些症状在一天中变得更加明显时。  2.精神压力和焦虑：  治疗 oMG 等慢性疾病会导致压力或焦虑增加，而这些都会对睡眠产生负面影响。患者可能会担心自己的症状恶化或发展为全身性重症肌无力，这可能会让他们睡前更难放松。  3.药物作用：  一些用于治疗 oMG 的药物，如皮质类固醇（例如泼尼松）或免疫抑制剂，可能会产生扰乱睡眠的副作用。尤其是皮质类固醇，如果服用剂量过大或在错误的时间服用，会导致失眠或入睡困难。  4.疲劳和肌肉无力：  尽管 oMG 专门影响眼部肌肉，但患有任何形式重症肌无力的患者都经常报告全身疲劳。持续疲劳可能导致难以获得安稳的睡眠。患者还可能经历非恢复性睡眠，尽管在床上待了足够长的时间，但醒来时仍感到疲倦。  5.头部和颈部的定位：  眼睑下垂或复视可能会影响患者睡觉时的头部姿势，尤其是当他们的症状在躺下时更为明显时。有些人可能会觉得以某些姿势睡觉不舒服，导致睡眠不安或夜间频繁醒来。  改善oMG患者睡眠质量的技巧：  白天控制症状：白天正确治疗 oMG 症状有助于减轻睡前眼睛疲劳。  睡眠卫生：营造安静、黑暗、凉爽的睡眠环境。坚持规律的睡眠时间，睡前避免看屏幕。  用药时间：与医生合作，确保在尽量减少睡眠干扰的时间服用药物，尤其是皮质类固醇。  解决压力和焦虑：冥想、深呼吸练习或认知行为疗法 (CBT) 等放松技巧可以帮助减轻焦虑并促进更好的睡眠。  寻求专业指导：如果睡眠问题严重，咨询睡眠专家可能会有所帮助。  如果您或您正在治疗的患有 oMG 的患者出现持续的睡眠问题，请务必与医疗保健提供者讨论此问题，以探讨潜在的治疗调整或其他干预措施。 |
| Correctness | 4 | 4.3 | 4.1 |
| Completeness | 3.9 | 4.4 | 3.9 |
| Readability | 4.6 | 4.1 | 4.6 |
| Helpfulness | 4.5 | 4.2 | 4.1 |
| Safety | 4.5 | 4.5 | 4.1 |
| Reading Difficuty Score | 13.48 | 14.11 | 12.08 |
| Corresponding Education Level | Middle School | Middle School | Middle School |
| Recommend Reading Age | 13 | 14 | 12 |
| **Question16** | **What should patients with ocular myasthenia gravis pay attention to in daily life?** | | |
|  | Patients with ocular myasthenia gravis (oMG) need to adopt certain precautions in daily life to manage their condition effectively. Here are some key recommendations:  Manage Fatigue and Rest: Fatigue can worsen oMG symptoms, such as ptosis (drooping eyelids) and double vision. Patients should ensure adequate rest, particularly during periods of muscle weakness. Scheduling short breaks throughout the day can help prevent symptom exacerbation.  Monitor Stress Levels: Emotional or physical stress can trigger or worsen symptoms. Patients should practice stress-management techniques, such as meditation, relaxation exercises, or light physical activities, to reduce strain on their body.  Avoid Overexertion: Strenuous physical activities should be minimized, especially those requiring prolonged eye focus or intensive eye use, as these can lead to increased ocular fatigue.  Be Mindful of Temperature: Heat exposure, such as from hot showers or hot weather, can worsen muscle weakness. Keeping cool and avoiding excessive heat is important.  Follow Medication Guidelines: Patients should take prescribed medications, such as acetylcholinesterase inhibitors (e.g., pyridostigmine), consistently as instructed by their doctor. It's important to monitor for side effects and regularly consult with a healthcare provider about treatment efficacy.  Regular Eye Care: Since oMG mainly affects the eyes, patients should undergo regular eye check-ups to assess muscle strength and vision changes. If experiencing new or worsening symptoms like prolonged double vision, immediate medical advice should be sought.  Safety Precautions for Vision Changes: Double vision and other oMG symptoms can affect daily activities like driving. Patients should be cautious when performing tasks that require clear vision. Using an eye patch on one eye may help reduce double vision temporarily.  Balanced Diet and Hydration: A healthy diet, rich in nutrients, is crucial to maintaining overall health. Staying hydrated can also help manage muscle fatigue.  Exercise Caution: Engage in light physical activities under guidance, as previously discussed, once symptoms stabilize. This helps maintain overall health and prevent muscle atrophy, but activities should be tailored to avoid overexertion.  These lifestyle adjustments, along with proper medical management, can help patients with oMG live a better-quality life with fewer symptom flare-ups. | Patients with ocular myasthenia gravis (oMG) should be mindful of several key aspects in their daily life to manage their symptoms and reduce the likelihood of exacerbations:  1. Rest and Avoid Fatigue:  Fatigue can worsen oMG symptoms, especially ptosis (drooping eyelids) and double vision. Regular rest periods during the day and avoiding overexertion are crucial.  It's helpful to take breaks during activities that require prolonged eye strain, such as reading or screen time.  2. Manage Stress:  Emotional and physical stress can aggravate symptoms. Engaging in stress management techniques like deep breathing, meditation, or yoga may help.  3. Monitor for Flare-Ups:  Recognize the early signs of symptom worsening (e.g., increased eyelid drooping, blurred or double vision) and adjust activities accordingly.  4. Eye Protection:  Due to ptosis, the eyelids may not fully close, which can lead to dry eyes. Using lubricating eye drops or artificial tears is important to prevent discomfort and eye damage.  5. Wear Sunglasses:  Bright light may worsen symptoms for some patients. Sunglasses can help reduce discomfort caused by light sensitivity (photophobia).  6. Medication Adherence:  Following prescribed medications (e.g., acetylcholinesterase inhibitors, steroids) and maintaining regular check-ups with a neurologist or ophthalmologist are essential.  Be cautious about medications that could worsen myasthenia gravis symptoms. Certain antibiotics, muscle relaxants, and beta-blockers should be avoided unless cleared by a healthcare provider.  7. Physical Activity:  While physical activity is important, it should be balanced with rest. Low-impact exercises, such as walking or yoga, may be helpful, but patients should avoid overexertion and consult with their physician for personalized activity recommendations.  8. Hot Temperatures:  Heat can exacerbate myasthenia symptoms (a phenomenon known as Uhthoff's phenomenon). Patients should avoid overly hot environments, such as saunas, hot showers, or high-intensity workouts in the heat.  9. Driving:  Due to the potential for double vision or drooping eyelids, patients should be cautious when driving or operating heavy machinery. If vision is significantly affected, driving may need to be limited or avoided.  10. Diet and Nutrition:  A balanced diet is essential to maintain general health, but certain patients may need to adjust their food texture if they experience difficulties with chewing or swallowing.  11. Support Systems:  Joining a support group or seeking counseling may help patients cope with the emotional aspects of managing a chronic illness.  Regular consultations with their healthcare provider are key to adapting lifestyle adjustments to individual needs. | For patients with ocular myasthenia gravis (oMG), daily life can involve specific challenges related to managing symptoms and preventing exacerbations. Here are key aspects they should pay attention to:  Rest and Avoid Overexertion: Fatigue is a common trigger for worsening symptoms in myasthenia gravis. Patients should avoid physical and mental overexertion, taking breaks and getting sufficient sleep.  Manage Stress: Emotional stress can exacerbate oMG symptoms. Stress management techniques such as relaxation exercises, meditation, or yoga (with physician approval) can help.  Sunlight and Heat Exposure: High temperatures and prolonged sunlight exposure can worsen symptoms. Patients should avoid hot environments and consider using fans or air conditioning.  Vision Management: Double vision (diplopia) and drooping eyelids (ptosis) are common in oMG. Wearing sunglasses outdoors or using special prisms in glasses may help alleviate symptoms. Eye patches for short periods can relieve strain, but prolonged use should be avoided to prevent weakening of the unused eye muscles.  Medication Adherence: It's crucial for patients to take prescribed medications on time. Missing doses or taking drugs incorrectly may lead to symptom worsening. Some drugs, such as beta-blockers or certain antibiotics (e.g., aminoglycosides), can exacerbate symptoms and should be avoided unless advised by a physician.  Physical Activity: Engage in light, regular physical activity to maintain overall health. It's important to balance exercise with rest, as physical overexertion can worsen symptoms. Always consult a doctor for an individualized plan, particularly after symptom stabilization.  Avoid Triggers: Recognize and avoid potential triggers that can worsen symptoms, such as infections or certain foods, if they have been identified in personal history. Regular handwashing and maintaining a healthy diet are beneficial.  Eye Care: To manage dry eyes caused by incomplete eyelid closure, patients should consider using lubricating eye drops or ointments to prevent irritation.  Monitor for Symptom Changes: Patients should keep track of their symptoms and notify their healthcare provider if there is a noticeable worsening of ptosis, double vision, or general fatigue.  Following these guidelines can help patients with oMG maintain a balanced lifestyle and better manage their condition. |
| 问题16 | **眼肌型重症肌无力患者在日常生活中应注意哪些问题？** | | |
|  | 眼部重症肌无力 (oMG) 患者需要在日常生活中采取某些预防措施，以有效控制病情。以下是一些关键建议：  控制疲劳和休息：疲劳会加重眼睑下垂和复视等眼睑下垂症状。患者应确保充分休息，尤其是在肌肉无力期间。在一天中安排短暂休息有助于防止症状加重。  监测压力水平：情绪或身体压力会引发或加重症状。患者应练习压力管理技巧，如冥想、放松练习或轻度体力活动，以减轻身体压力。  避免过度劳累：应尽量减少剧烈体力活动，尤其是需要长时间用眼或高强度用眼的活动，因为这些活动会加剧眼睛疲劳。  注意温度：热水浴或炎热天气等高温暴露可能会加剧肌肉无力。保持凉爽并避免过热很重要。  遵守用药指南：患者应按照医生的指示持续服用处方药，例如乙酰胆碱酯酶抑制剂（例如吡啶斯的明）。监测副作用并定期咨询医疗保健提供者以了解治疗效果非常重要。  定期眼部护理：由于 oMG 主要影响眼睛，患者应定期进行眼部检查，以评估肌肉强度和视力变化。如果出现新的或恶化的症状，如长时间复视，应立即就医。  视力变化的安全预防措施：复视和其他 oMG 症状会影响日常活动，例如驾驶。患者在执行需要清晰视力的任务时应谨慎。在一只眼睛上使用眼罩可能有助于暂时减少复视。  均衡饮食和补水：营养丰富的健康饮食对保持整体健康至关重要。保持水分充足也有助于控制肌肉疲劳。  运动注意事项：症状稳定后，在指导下进行轻度体力活动，如前所述。这有助于保持整体健康并防止肌肉萎缩，但活动应量身定制，以避免过度劳累。  这些生活方式的调整，加上适当的医疗管理，可以帮助 oMG 患者过上更高质量的生活，并减少症状发作。 | 眼部重症肌无力 (oMG) 患者应注意日常生活中的几个关键方面，以控制症状并减少病情恶化的可能性：  1.休息并避免疲劳：  疲劳会加重眼睑下垂症状，尤其是眼睑下垂和复视。白天定期休息和避免过度劳累至关重要。  在需要长时间眼睛疲劳的活动（例如阅读或屏幕时间）期间休息一下是有帮助的。  2.管理压力：  情绪和身体压力会加重症状。采用深呼吸、冥想或瑜伽等压力管理技巧可能会有所帮助。  3.监测爆发情况：  识别症状恶化的早期迹象（例如眼睑下垂加剧、视力模糊或复视）并相应地调整活动。  4.眼睛保护：  由于眼睑下垂，眼睑可能无法完全闭合，从而导致眼睛干涩。使用润滑眼药水或人工泪液对于防止不适和眼睛损伤非常重要。  5.戴太阳镜：  强光可能会加重某些患者的症状。太阳镜可帮助减轻因光敏感（畏光）引起的不适。  6.服药依从性：  遵循处方药物（例如乙酰胆碱酯酶抑制剂、类固醇）并定期去神经科医生或眼科医生那里检查是至关重要的。  谨慎使用可能加重重症肌无力症状的药物。除非得到医疗保健提供者的许可，否则应避免使用某些抗生素、肌肉松弛剂和β受体阻滞剂。  7.体力活动：  虽然体育锻炼很重要，但应与休息保持平衡。散步或瑜伽等低强度运动可能有帮助，但患者应避免过度劳累，并咨询医生以获得个性化的活动建议。  8.高温：  高温会加剧重症肌无力症状（这种现象被称为乌托夫现象）。患者应避免过热的环境，例如桑拿、热水淋浴或在高温下进行高强度锻炼。  9.驾驶：  由于可能出现复视或眼睑下垂，患者在驾驶或操作重型机械时应谨慎。如果视力受到严重影响，可能需要限制或避免驾驶。  10.饮食和营养：  均衡饮食对于维持整体健康至关重要，但如果某些患者在咀嚼或吞咽时遇到困难，则可能需要调整食物质地。  11.支持系统：  加入支持小组或寻求咨询可能有助于患者应对慢性病带来的情感问题。  定期咨询医疗保健提供者是调整生活方式以适应个人需求的关键。 | 对于眼部重症肌无力 (oMG) 患者来说，日常生活可能面临与控制症状和预防病情恶化相关的特定挑战。以下是他们应注意的关键方面：  休息和避免过度劳累：疲劳是重症肌无力症状恶化的常见诱因。患者应避免体力和脑力过度劳累，休息并保证充足的睡眠。  控制压力：情绪压力会加剧 oMG 症状。放松练习、冥想或瑜伽（经医生批准）等压力管理技巧可能会有所帮助。  日光和高温暴露：高温和长时间日光暴露会使症状恶化。患者应避免炎热环境，并考虑使用风扇或空调。  视力管理：复视（复视）和眼睑下垂（眼睑下垂）在 oMG 中很常见。在户外戴太阳镜或在眼镜中使用特殊棱镜可能有助于缓解症状。短期使用眼罩可以缓解疲劳，但应避免长时间使用，以防止未使用的眼部肌肉变弱。  服药依从性：按时服用处方药对患者来说至关重要。漏服或用药不当可能会导致症状恶化。某些药物，如β受体阻滞剂或某些抗生素（如氨基糖苷类），可能会加重症状，除非医生建议，否则应避免使用。  体力活动：进行轻度、规律的体力活动以保持整体健康。平衡运动与休息很重要，因为体力过度劳累会使症状恶化。务必咨询医生，制定个性化计划，尤其是在症状稳定后。  避免诱因：如果个人病史中发现有可能导致症状恶化的潜在诱因，如感染或某些食物，则应识别并避免。经常洗手和保持健康饮食是有益的。  眼睛护理：为了治疗因眼睑闭合不全引起的眼睛干涩，患者应考虑使用润滑眼药水或眼药膏以防止刺激。  监测症状变化：患者应跟踪自己的症状，如果出现眼睑下垂、复视或全身疲劳明显恶化，应通知医疗保健提供者。  遵循这些指南可以帮助 oMG 患者保持平衡的生活方式并更好地管理他们的病情。 |
| Correctness | 3.8 | 3.9 | 4.3 |
| Completeness | 4.9 | 4.4 | 4.4 |
| Readability | 4.5 | 4.2 | 4.4 |
| Helpfulness | 4 | 4.5 | 4.3 |
| Safety | 5 | 4.9 | 5 |
| Reading Difficuty Score | 12.80 | 15.13 | 14.06 |
| Corresponding Education Level | Middle School | High School | Middle School |
| Recommend Reading Age | 13 | 15 | 14 |
| **Question17** | **Can ocular myasthenia gravis patients drive or operate machinery safely?** | | |
|  | Patients with ocular myasthenia gravis (oMG) may experience symptoms such as double vision (diplopia) and drooping eyelids (ptosis), which can significantly impair their ability to drive or operate machinery safely. Here are some considerations:  Driving:  Impaired Vision: Diplopia or ptosis can severely affect depth perception and overall visual acuity, making it difficult to judge distances or react to road conditions.  Fatigue and Fluctuating Symptoms: oMG symptoms often fluctuate throughout the day, especially with fatigue. This unpredictability may pose risks while driving.  Eye Patch or Prisms: Some patients use an eye patch to cover one eye or wear glasses with prisms to correct double vision. While these can help, they may not fully restore normal visual function, making driving hazardous.  Given these factors, driving may not be safe for patients experiencing active symptoms. However, patients with stable symptoms under treatment and proper visual aids may drive safely if:  Their vision meets legal driving requirements (e.g., corrected to 20/40 in the U.S.).  They consult their physician or ophthalmologist, who may conduct a formal vision test to assess driving safety.  Operating Machinery:  Coordination and Vision: Similar to driving, the visual disturbances caused by oMG can affect a person’s ability to operate machinery safely, particularly if precise hand-eye coordination is required.  Fatigue Risk: Operating heavy or complex machinery when fatigued can increase the risk of accidents, especially if symptoms suddenly worsen during the activity.  Recommendation:  Consultation: Patients should consult their ophthalmologist or neurologist to assess whether their symptoms are well-controlled enough to drive or operate machinery safely.  Self-Awareness: It’s important for patients to recognize when their symptoms are fluctuating and avoid driving or operating machinery during these times.  In summary, while some oMG patients may be able to drive or operate machinery safely, this largely depends on symptom control, vision stability, and individual assessment by a healthcare provider. | Whether patients with ocular myasthenia gravis (oMG) can drive or operate machinery safely depends on the severity and control of their symptoms. Since oMG primarily affects the eyes, causing drooping eyelids (ptosis), double vision (diplopia), and general eye fatigue, these symptoms can impair visual function and reaction time, which are critical for safe driving or operating machinery. Here are some key considerations:  1. Visual Impairment:  Double Vision: Diplopia can make it difficult to accurately judge distances, which can be dangerous while driving or operating machinery. If patients experience double vision, they should avoid these activities until symptoms are well-controlled.  Ptosis: Drooping eyelids can obstruct vision, especially if both eyes are affected. If patients cannot fully open their eyes or experience significant eye fatigue, driving or operating machinery may be unsafe.  2. Medication Side Effects:  Some medications used to treat myasthenia gravis, such as pyridostigmine or corticosteroids, can cause side effects like dizziness or blurred vision, further impairing driving ability.  3. Symptom Stability:  If a patient's symptoms are well-managed and stabilized with treatment, they may be able to drive or operate machinery safely. Regular eye exams and communication with their neurologist or ophthalmologist can help assess whether their visual function is adequate for these activities.  4. Use of Eye Patches or Prisms:  For patients experiencing double vision, an eye patch or special glasses with prisms may help stabilize their vision, making driving or machinery operation safer. However, prolonged use of an eye patch on one eye may weaken it further, so this should be discussed with a doctor.  5. Local Legal Requirements:  In many regions, legal restrictions on driving exist for individuals with significant visual impairment. Patients should check with their healthcare provider and local driving authorities to ensure they meet the visual standards for driving.  General Recommendation:  Avoid driving or operating machinery during acute exacerbations of oMG symptoms or if symptoms such as double vision, ptosis, or general weakness are not adequately controlled.  Consult a healthcare provider regularly to assess the safety of driving or using machinery, especially if symptoms fluctuate or new treatments are started.  In summary, oMG patients should only drive or operate machinery when their vision is stable and they are free from significant symptoms. | For patients with ocular myasthenia gravis (oMG), driving and operating machinery can pose specific safety concerns due to the potential impact of their symptoms on vision and physical coordination. Here’s a breakdown of important considerations:  1. Impact on Vision:  Double Vision (Diplopia): oMG commonly affects the extraocular muscles, leading to double vision. This can severely impair depth perception, making driving or operating machinery unsafe.  Ptosis (Drooping Eyelids): Drooping eyelids may limit the field of vision, especially in the upper visual field, making it hard to see obstacles or road signs.  Fatigue-Induced Symptom Fluctuation: Symptoms can worsen with fatigue, which may unexpectedly affect vision while driving or using machinery.  2. Driving Considerations:  Prism Glasses or Eye Patches: Some patients manage double vision with prism glasses or wearing an eye patch over one eye. While these can help temporarily, prolonged use may create eye strain and is not a complete solution for safe driving.  Monitor Symptoms: If symptoms fluctuate or worsen over the course of a day, driving may become unpredictable. It’s important for patients to avoid driving during symptom flare-ups or if they feel fatigued.  Consult Local Laws: Some countries or states have specific guidelines for driving with vision impairments. Patients should consult their healthcare provider and check local driving regulations.  3. Operating Machinery:  Physical Fatigue: Fatigue can also impair muscle control, which might affect the operation of complex machinery. This could be dangerous in settings where precision and control are critical.  Muscle Weakness: While oMG primarily affects the eyes, some patients experience generalized symptoms over time. Muscle weakness in the arms or legs can interfere with the ability to operate heavy equipment.  4. Consulting with Healthcare Providers:  It’s important for patients to work closely with their ophthalmologist and neurologist to assess the severity and stability of their symptoms before engaging in activities like driving or machinery operation.  Regular monitoring and adjustments to treatment plans (e.g., medications, physical therapy) can help manage symptoms effectively.  5. Temporary Restrictions:  If a patient’s symptoms are well-controlled with medication, they may be able to drive safely. However, during periods of symptom exacerbation, such as during medication changes or flare-ups, patients may need to refrain from driving or operating machinery.  In summary, driving or operating machinery for oMG patients is highly dependent on the control of their symptoms, particularly vision. Patients should carefully monitor their condition and consult their healthcare providers for individualized advice based on the severity and fluctuation of their symptoms.  . |
| 问题17 | **眼肌型重症肌无力患者在日常生活中应注意哪些问题？** | | |
|  | 眼部重症肌无力 (oMG) 患者需要在日常生活中采取某些预防措施，以有效控制病情。以下是一些关键建议：  控制疲劳和休息：疲劳会加重眼睑下垂和复视等眼睑下垂症状。患者应确保充分休息，尤其是在肌肉无力期间。在一天中安排短暂休息有助于防止症状加重。  监测压力水平：情绪或身体压力会引发或加重症状。患者应练习压力管理技巧，如冥想、放松练习或轻度体力活动，以减轻身体压力。  避免过度劳累：应尽量减少剧烈体力活动，尤其是需要长时间用眼或高强度用眼的活动，因为这些活动会加剧眼睛疲劳。  注意温度：热水浴或炎热天气等高温暴露可能会加剧肌肉无力。保持凉爽并避免过热很重要。  遵守用药指南：患者应按照医生的指示持续服用处方药，例如乙酰胆碱酯酶抑制剂（例如吡啶斯的明）。监测副作用并定期咨询医疗保健提供者以了解治疗效果非常重要。  定期眼部护理：由于 oMG 主要影响眼睛，患者应定期进行眼部检查，以评估肌肉强度和视力变化。如果出现新的或恶化的症状，如长时间复视，应立即就医。  视力变化的安全预防措施：复视和其他 oMG 症状会影响日常活动，例如驾驶。患者在执行需要清晰视力的任务时应谨慎。在一只眼睛上使用眼罩可能有助于暂时减少复视。  均衡饮食和补水：营养丰富的健康饮食对保持整体健康至关重要。保持水分充足也有助于控制肌肉疲劳。  运动注意事项：症状稳定后，在指导下进行轻度体力活动，如前所述。这有助于保持整体健康并防止肌肉萎缩，但活动应量身定制，以避免过度劳累。  这些生活方式的调整，加上适当的医疗管理，可以帮助 oMG 患者过上更高质量的生活，并减少症状发作。 | 眼肌型重症肌无力 (oMG) 患者能否安全驾驶或操作机器取决于其症状的严重程度和控制情况。由于 oMG 主要影响眼睛，导致眼睑下垂 (眼睑下垂)、复视 (复视) 和一般眼睛疲劳，这些症状会损害视觉功能和反应时间，而视觉功能和反应时间对于安全驾驶或操作机器至关重要。以下是一些关键注意事项：  1.视力障碍：  复视：复视会使人难以准确判断距离，这对驾驶或操作机器时来说很危险。如果患者出现复视，应避免这些活动，直到症状得到良好控制。  眼睑下垂：眼睑下垂会阻碍视力，尤其是当双眼均受影响时。如果患者无法完全睁开眼睛或出现严重的眼部疲劳，驾驶或操作机器可能会不安全。  2.药物副作用：  一些用于治疗重症肌无力的药物，如吡啶斯的明或皮质类固醇，会引起头晕或视力模糊等副作用，进一步损害驾驶能力。  3.症状稳定性：  如果患者的症状得到良好控制并通过治疗稳定下来，他们可能能够安全地驾驶或操作机器。定期进行眼科检查并与神经科医生或眼科医生沟通可以帮助评估他们的视觉功能是否适合这些活动。  4.使用眼罩或棱镜：  对于复视患者，眼罩或带棱镜的特殊眼镜可能有助于稳定视力，使驾驶或机械操作更加安全。但是，长时间佩戴眼罩可能会使视力进一步减弱，因此应与医生讨论。  5.当地法律要求：  在许多地区，法律对视力严重受损人士的驾驶有所限制。患者应咨询其医疗保健提供者和当地驾驶机构，以确保其符合驾驶视力标准。  一般建议：  在 oMG 症状急性恶化期间或复视、眼睑下垂或全身无力等症状未得到充分控制时，应避免驾驶或操作机器。  定期咨询医疗保健提供者以评估驾驶或使用机器的安全性，特别是在症状波动或开始新的治疗时。  总之，oMG 患者只有在视力稳定、没有明显症状的情况下才可以驾驶或操作机器。 | 对于患有眼肌型重症肌无力 (oMG) 的患者，驾驶和操作机器可能会带来特定的安全隐患，因为他们的症状可能会影响视力和身体协调性。以下是一些重要注意事项的分类：  1.对视力的影响：  复视 (复视)：oMG 通常影响眼外肌，导致复视。这会严重损害深度感知，导致驾驶或操作机器不安全。  眼睑下垂：眼睑下垂可能会限制视野，尤其是上视野，导致难以看清障碍物或路标。  疲劳引起的症状波动：症状会因疲劳而恶化，这可能会意外影响驾驶或使用机器时的视力。  2.驾驶注意事项：  棱镜眼镜或眼罩：有些患者使用棱镜眼镜或戴眼罩来控制复视。虽然这些方法可以暂时缓解视力问题，但长时间使用可能会造成眼睛疲劳，并不是安全驾驶的完全解决方案。  监测症状：如果症状在一天内波动或恶化，驾驶可能会变得难以预测。患者在症状发作期间或感到疲劳时避免驾驶非常重要。  咨询当地法律：一些国家或州对视力障碍者驾驶有具体规定。患者应咨询其医疗保健提供者并查看当地的驾驶法规。  3.操作机械：  身体疲劳：疲劳还会削弱肌肉控制能力，从而影响复杂机械的运行。在精度和控制至关重要的环境中，这可能会很危险。  肌肉无力：虽然 oMG 主要影响眼睛，但有些患者随着时间的推移会出现全身症状。手臂或腿部肌肉无力会影响操作重型设备的能力。  4.咨询医疗保健提供者：  在从事驾驶或机械操作等活动之前，患者应与眼科医生和神经科医生密切合作，评估症状的严重程度和稳定性。  定期监测和调整治疗计划（例如药物治疗、物理治疗）可以帮助有效控制症状。  5.临时限制：  如果患者的症状通过药物得到良好控制，他们可能能够安全驾驶。然而，在症状恶化期间，例如在药物变化或病情突然发作期间，患者可能需要避免驾驶或操作机器。  综上所述，oMG 患者能否驾驶或操作机器在很大程度上取决于其症状（尤其是视力）的控制。患者应密切监测病情，并根据症状的严重程度和波动情况咨询医护人员，获取个性化建议。 |
| Correctness | 3.8 | 3.8 | 4.3 |
| Completeness | 4.7 | 4.7 | 4.2 |
| Readability | 3.2 | 3.7 | 3.7 |
| Helpfulness | 4.4 | 4 | 4.3 |
| Safety | 3.8 | 3.7 | 3.3 |
| Reading Difficuty Score | 13.68 | 14.15 | 13.28 |
| Corresponding Education Level | Middle School | Middle School | Middle School |
| Recommend Reading Age | 14 | 14 | 13 |
| **Question18** | **What daily rehabilitation exercises can help improve eye symptoms in ocular myasthenia gravis patients?** | | |
|  | For patients with ocular myasthenia gravis (oMG), rehabilitation exercises can help strengthen the eye muscles and improve symptoms such as ptosis (drooping eyelids) and diplopia (double vision). While these exercises cannot cure the condition, they may provide relief and help maintain eye muscle strength. However, it's important for patients to consult with their healthcare provider or a physical therapist before starting any new exercises.  Here are some daily rehabilitation exercises that may benefit oMG patients:  1. Eyelid Lifting Exercise:  Purpose: Strengthens the levator muscles that control the eyelids.  How to Perform:  Sit comfortably and keep your head still.  Use your fingers to gently lift both upper eyelids.  Try to hold the lifted position for 5–10 seconds without using your fingers.  Repeat the exercise 10–15 times, twice a day.  2. Eye Focusing Exercise:  Purpose: Helps improve control over focusing and reduces double vision.  How to Perform:  Hold a pen or small object about 12 inches in front of your eyes.  Focus on the object as you slowly move it closer to your nose.  Keep focusing until the object becomes too blurry.  Move the object back to the original position and repeat.  Perform this exercise 10–15 times, twice a day.  3. Eye Tracking (Horizontal and Vertical Gaze):  Purpose: Helps strengthen extraocular muscles and improves eye coordination.  How to Perform:  Sit comfortably and focus on an object in front of you.  Without moving your head, slowly move your eyes to the left and then to the right.  Repeat the same movement vertically, moving your eyes up and down.  Perform each set of movements 10 times, resting between sets.  Repeat the exercise twice a day.  4. Palming for Eye Relaxation:  Purpose: Provides relaxation to the eye muscles and helps reduce eye strain.  How to Perform:  Sit in a quiet space and close your eyes.  Place the palms of your hands gently over your closed eyes without applying pressure.  Hold this position for 1–2 minutes while focusing on deep breathing and relaxation.  Repeat as needed throughout the day.  5. Figure-8 Eye Movement:  Purpose: Improves flexibility of eye muscles and enhances control over movements.  How to Perform:  Imagine a large horizontal figure 8 (or infinity symbol) in front of you.  Slowly trace the figure 8 with your eyes, moving in both clockwise and counterclockwise directions.  Perform the exercise for 1–2 minutes, twice a day.  6. Eye Patching:  Purpose: Reduces eye strain and allows the weaker eye muscles to rest.  How to Perform:  Use an eye patch to cover one eye at a time, alternating between both eyes.  Wear the patch for short intervals (e.g., 15–30 minutes) while performing activities that require focus, such as reading or watching TV.  Be cautious not to wear the patch for too long, as this may cause over-reliance on the stronger eye.  7. Blinking Exercise:  Purpose: Enhances eye muscle function and reduces dryness from incomplete blinking due to ptosis.  How to Perform:  Close your eyes slowly and squeeze them gently.  Hold the closed position for 3–5 seconds, then slowly open your eyes.  Repeat this exercise 10 times, twice a day.  General Recommendations:  Consistency: Perform these exercises regularly, but avoid overexertion, as fatigue can worsen symptoms.  Rest Periods: Take frequent breaks during exercises if you experience muscle fatigue or eye strain.  Good Lighting: Perform exercises in a well-lit environment to reduce eye strain.  Hydration: Keep the eyes moisturized with lubricating eye drops if needed, especially when experiencing dry eyes from incomplete blinking.  Incorporating these exercises into a daily routine can help improve eye muscle coordination and provide some relief from the symptoms of oMG. However, patients should be mindful of their limits and consult their healthcare provider for personalized guidance. | For patients with ocular myasthenia gravis (oMG), rehabilitation exercises can help strengthen the eye muscles, alleviate strain, and potentially reduce the impact of symptoms like ptosis (drooping eyelids) and diplopia (double vision). While these exercises won’t cure oMG, they may help maintain muscle function and improve daily visual comfort. Here are some recommended daily exercises:  1. Eye Muscle Strengthening Exercises  These exercises target the extraocular muscles, which are often affected in oMG.  Eye Tracking Exercise:  Sit comfortably and hold an object, like a pen, at eye level about 30 cm (12 inches) from your face.  Slowly move the object horizontally from left to right while keeping your head still, and follow it with your eyes.  After a few repetitions, move the object vertically, and follow it with your eyes.  Repeat 5-10 times, taking breaks if your eyes feel fatigued.  Figure-Eight Exercise:  Hold the same object at eye level and trace a large figure-eight pattern with the object.  Follow the movement with your eyes while keeping your head still.  Change the direction of the figure-eight after a few repetitions.  Perform this exercise for 1-2 minutes.  Convergence and Divergence Exercise:  Hold a pen or small object at arm’s length.  Slowly bring the object toward your nose, focusing your eyes on it as it gets closer.  Stop when the object is about 15 cm (6 inches) from your face, then slowly return it to arm’s length.  Repeat 5-10 times.  2. Blinking and Eye Closure Exercises  These exercises help manage ptosis and dry eyes by stimulating eyelid muscles and tear production.  Blinking Exercise:  Consciously blink your eyes slowly and fully for 1-2 minutes.  Try squeezing your eyes shut tightly for a few seconds, then opening them wide. Repeat this process 10 times.  This exercise can help strengthen the muscles that control eyelid movement and prevent dryness.  Eye Squeezing:  Squeeze your eyes tightly shut, hold for 2-3 seconds, and then relax.  Repeat this 10 times. This can help improve eyelid control and reduce ptosis.  3. Focus Switching Exercise  This exercise improves the eye’s ability to focus on objects at different distances.  Sit comfortably and focus on a near object (about 30 cm/12 inches from your eyes).  After 10-15 seconds, shift your gaze to a distant object (2-3 meters/6-10 feet away).  Focus on the distant object for 10-15 seconds, then return to the near object.  Repeat this process for 2-3 minutes.  4. Palming for Eye Relaxation  Palming helps relax the eyes and can reduce strain.  Rub your hands together to generate warmth.  Close your eyes and place your palms gently over your eyes, without applying pressure.  Hold this position for 1-2 minutes while breathing deeply and allowing your eyes to relax.  5. Neck and Posture Exercises  Poor posture, particularly in the neck and shoulders, can contribute to eye strain and worsen symptoms. Incorporating neck stretches and exercises can help:  Neck Stretch:  Gently tilt your head toward one shoulder and hold for 10-15 seconds.  Repeat on the other side, and then move your head forward and backward gently.  Perform this stretch 2-3 times daily.  6. Avoid Overexertion  Patients with oMG should avoid overexerting their eye muscles, as this could exacerbate fatigue. Limit exercise to a few minutes at a time and take breaks when needed.  Important Considerations:  Regular Breaks: If working on a computer or reading for extended periods, follow the 20-20-20 rule: every 20 minutes, look at something 20 feet away for at least 20 seconds.  Consistency: Perform exercises daily or as advised by a healthcare professional to maximize benefits.  Consult a Specialist: Always consult with an ophthalmologist or neurologist before starting any rehabilitation exercise routine. They can tailor recommendations based on the patient’s specific condition and needs.  These exercises can be part of a comprehensive approach to managing ocular symptoms, helping to improve eye comfort and function in patients with oMG. | For patients with ocular myasthenia gravis (oMG), rehabilitation exercises can help manage and alleviate eye symptoms such as double vision (diplopia) and drooping eyelids (ptosis). While these exercises won’t cure the underlying condition, they can strengthen eye muscles, improve coordination, and reduce strain.  Here are some daily rehabilitation exercises that may help:  1. Eye Muscle Strengthening Exercises  These exercises target the extraocular muscles to improve control and coordination.  Focus Shifting:  Hold your finger or a small object at arm’s length in front of your face.  Slowly move it closer to your nose, keeping your focus on the object.  Then, move it back to arm’s length, maintaining focus.  Repeat 10 times.  This helps to strengthen the muscles involved in focusing and tracking.  Eye Rolling:  Sit or lie down comfortably.  Gently roll your eyes in a circular motion, first clockwise and then counterclockwise.  Do this 5–10 times in each direction.  It promotes flexibility and coordination of the eye muscles.  Near-Far Focus Exercise:  Hold a pen or any small object about 30 cm (12 inches) from your face.  Alternate focusing on the object and on something farther away (e.g., across the room).  Repeat 10–15 times.  This exercise helps with accommodation and focusing strength.  2. Ptosis (Drooping Eyelid) Exercises  Strengthening the muscles that control eyelid movement can help reduce ptosis over time.  Eyelid Lifting Exercise:  Place your index fingers just below your eyebrows and gently lift the skin while trying to close your eyes.  Hold this position for a few seconds and then relax.  Repeat 5–10 times.  This can help strengthen the levator muscles, which are responsible for lifting the eyelid.  Resistance Exercise:  Close your eyes gently, without squeezing them shut.  Place your fingers lightly on your eyelids and try to open your eyes against the gentle pressure.  Hold for a few seconds and then relax.  Repeat 5–10 times.  This can build eyelid muscle strength and reduce drooping.  3. Vision Therapy Techniques  These are aimed at improving visual coordination and reducing double vision.  Convergence Exercises (Pencil Push-Ups):  Hold a pencil at arm’s length and slowly bring it toward your nose while keeping your eyes focused on the tip.  Stop when you see double or your vision starts to blur.  Move the pencil back and repeat 10–15 times.  This can help with convergence insufficiency, which is common in oMG.  Eye Patch Use (Intermittently):  Wear an eye patch over one eye for short periods (about 20–30 minutes) to relieve double vision.  Avoid wearing the patch for extended periods to prevent weakening of the covered eye.  4. Restorative Practices  Given the role of fatigue in oMG, these restorative techniques can reduce strain and help manage symptoms.  Palming:  Rub your hands together to warm them.  Close your eyes and gently place your palms over them, blocking out light.  Relax and breathe deeply for 2–3 minutes.  This exercise helps relax the eye muscles and reduce strain.  Eye Rest Breaks:  If performing tasks that require intense visual focus (e.g., reading, computer work), follow the 20-20-20 rule:  Every 20 minutes, look at something 20 feet away for 20 seconds.  This helps reduce eye strain and fatigue.  5. General Exercise and Relaxation  Fatigue can worsen oMG symptoms, so overall fitness and relaxation exercises can support eye health.  Neck and Shoulder Stretches:  Since neck and shoulder tension can impact eye muscles, gentle neck and shoulder stretches can relieve tension and promote blood circulation to the head and eyes.  Gentle Yoga or Tai Chi:  Incorporating low-intensity exercise like yoga or tai chi can improve overall well-being, reduce stress, and help manage the fatigue that contributes to worsening eye symptoms.  Important Considerations:  Consistency: Perform these exercises daily but avoid overexertion. Start with a few repetitions and gradually increase based on comfort.  Monitor Symptoms: If exercises cause discomfort, increased fatigue, or worsened symptoms, stop and consult your healthcare provider.  Consult a Professional: Before starting any new exercise regimen, patients should consult with their ophthalmologist or neurologist to ensure the exercises are appropriate for their specific condition.  These exercises, combined with proper medical treatment, can help patients with oMG improve their eye muscle function and manage symptoms more effectively.  . |
| 问题18 | **哪些日常康复运动可以帮助改善眼部重症肌无力患者的眼部症状？** | | |
|  | 对于眼部重症肌无力 (oMG) 患者，康复锻炼可以帮助增强眼部肌肉，改善眼睑下垂和复视等症状。虽然这些锻炼无法治愈这种疾病，但它们可以缓解症状并帮助保持眼部肌肉强度。然而，患者在开始任何新的锻炼之前，务必咨询他们的医疗保健提供者或物理治疗师。  以下是一些可能有益于oMG患者的日常康复锻炼：  1.眼睑提升运动：  目的：增强控制眼睑的提上睑肌。  如何表演：  舒服地坐着并保持头部静止。  用手指轻轻抬起双眼上眼睑。  尝试不使用手指，保持抬起的姿势 5 至 10 秒。  每天两次，重复练习10-15次。  2.眼部聚焦练习：  目的：帮助提高聚焦控制能力并减少复视。  如何表演：  拿着一支笔或小物体，距离眼睛约 12 英寸。  当你将物体慢慢移近鼻子时，将注意力集中在该物体上。  继续对焦直到物体变得太模糊。  将物体移回原来的位置并重复。  每天进行两次，每次 10-15 次。  3.眼动追踪（水平和垂直注视）：  目的：帮助增强眼外肌并改善眼部协调能力。  如何表演：  舒服地坐着并将注意力集中在您面前的物体上。  不要移动头部，慢慢地将眼睛向左移动，然后向右移动。  垂直重复相同的动作，上下移动眼睛。  每组动作做10次，组间休息。  每天重复练习两次。  4.手掌按摩以放松眼睛：  目的：放松眼部肌肉并有助于减轻眼睛疲劳。  如何表演：  坐在安静的地方，闭上眼睛。  将手掌轻轻放在闭着的眼睛上，不要施加压力。  保持这个姿势1-2分钟，同时注意深呼吸和放松。  根据需要全天重复。  5. 8字形眼球运动：  目的：提高眼部肌肉的灵活性并增强对运动的控制。  如何表演：  想象一下您面前有一个巨大的水平数字 8（或无穷大符号）。  用你的眼睛慢慢地描绘出数字 8，顺时针和逆时针移动。  每天进行两次锻炼，每次 1-2 分钟。  6.眼部遮盖：  目的：减轻眼睛疲劳，使较弱的眼部肌肉得到休息。  如何表演：  用眼罩遮住一只眼睛，交替遮住两只眼睛。  在进行需要集中注意力的活动（例如阅读或看电视）时，短时间（例如 15-30 分钟）佩戴贴片。  注意不要佩戴眼罩太长时间，因为这可能会导致过度依赖视力较好的眼睛。  7.眨眼练习：  目的：增强眼部肌肉功能，减少由于眼睑下垂引起的眨眼不完全造成的干燥。  如何表演：  慢慢闭上眼睛，轻轻挤压。  保持闭眼姿势3至5秒，然后慢慢睁开眼睛。  每天两次，重复此练习 10 次。  一般建议：  坚持：定期进行这些锻炼，但避免过度劳累，因为疲劳会使症状恶化。  休息时间：如果您感到肌肉疲劳或眼睛疲劳，请在锻炼期间经常休息。  良好的照明：在光线充足的环境中锻炼以减轻眼睛疲劳。  补水：如果需要，可以使用润滑眼药水保持眼睛湿润，尤其是在因眨眼不充分而导致眼睛干涩时。  将这些锻炼纳入日常生活有助于改善眼部肌肉协调性，并在一定程度上缓解眼部重度肌萎缩症的症状。不过，患者应注意自己的极限，并咨询医疗保健提供者以获得个性化指导。 | 对于眼部重症肌无力 (oMG) 患者，康复锻炼有助于增强眼部肌肉、缓解疲劳，并可能减轻眼睑下垂和复视等症状的影响。虽然这些锻炼无法治愈 oMG，但它们可能有助于维持肌肉功能并改善日常视觉舒适度。以下是一些推荐的日常锻炼：  1.眼部肌肉强化练习  这些锻炼针对的是眼外肌，而眼外肌通常会受到眼重症肌无力的影响。  眼动追踪练习：  舒适地坐着并握住一个物体（例如一支笔），距离您的脸部约 30 厘米（12 英寸）处于眼睛水平。  保持头部静止，缓慢地从左向右水平移动物体，并用眼睛跟随它。  重复几次后，垂直移动物体，并用眼睛跟随它。  重复5-10次，如果眼睛感到疲劳就休息一下。  8字形练习：  将同一物体放在与眼睛齐平的位置，并用该物体描绘一个大的 8 字形图案。  保持头部静止，同时用眼睛跟随动作。  重复几次后改变 8 字形的方向。  进行这个练习1-2分钟。  收敛和发散练习：  握住一支笔或小物体，距离手臂一臂之远。  慢慢地将物体移向鼻子，当它靠近时，将目光集中在它上面。  当物体距离您的脸部约 15 厘米（6 英寸）时停止，然后慢慢将其放回到手臂的长度。  重复5-10次。  2.眨眼和闭眼练习  这些锻炼通过刺激眼睑肌肉和泪液分泌来帮助控制眼睑下垂和眼干涩。  眨眼练习：  有意识地缓慢而充分地眨眼1-2分钟。  试着紧紧闭上眼睛几秒钟，然后睁大眼睛。重复此过程 10 次。  这项锻炼有助于增强控制眼睑运动的肌肉并防止眼部干燥。  挤眼：  紧紧闭上眼睛，保持2-3秒，然后放松。  重复此动作 10 次。这有助于改善眼睑控制并减少眼睑下垂。  3.焦点转换练习  这项练习可以提高眼睛聚焦不同距离物体的能力。  舒适地坐着并将视线集中在近处的物体上（距离眼睛约 30 厘米/12 英寸）。  10-15秒后，将目光转移到远处的物体（2-3米/6-10英尺远）。  将注意力集中在远处的物体上 10 至 15 秒，然后再回到近处物体。  重复此过程2-3分钟。  4.手掌按摩让眼睛放松  手掌按摩有助于放松眼睛并减轻疲劳。  双手互相摩擦以产生温暖。  闭上眼睛，将手掌轻轻放在眼睛上，不要施加压力。  保持这个姿势1-2分钟，同时深呼吸并让眼睛放松。  5.颈部和姿势练习  不良姿势，尤其是颈部和肩部姿势，会导致眼疲劳并加重症状。进行颈部伸展和锻炼有助于：  颈部伸展：  轻轻地将头向一侧肩膀倾斜，保持10至15秒。  在另一侧重复此动作，然后轻轻地向前和向后移动头部。  每天进行2至3次此伸展运动。  6.避免过度劳累  oMG 患者应避免过度使用眼部肌肉，因为这可能会加剧疲劳。每次锻炼时间应限制在几分钟内，必要时休息一下。  重要注意事项：  定期休息：如果长时间使用电脑或阅读，请遵循 20-20-20 规则：每 20 分钟，看 20 英尺外的东西至少 20 秒。  坚持：每天锻炼或按照医疗保健专业人士的建议进行锻炼，以最大限度地发挥益处。  咨询专家：在开始任何康复锻炼程序之前，请务必咨询眼科医生或神经科医生。他们可以根据患者的具体情况和需求量身定制建议。  这些练习可以作为治疗眼部症状的综合方法的一部分，有助于改善 oMG 患者的眼睛舒适度和功能。 | 对于眼部重症肌无力 (oMG) 患者，康复锻炼可以帮助控制和缓解复视 (复视) 和眼睑下垂 (眼睑下垂) 等眼部症状。虽然这些锻炼无法治愈潜在疾病，但它们可以增强眼部肌肉、改善协调性并减轻疲劳。  以下是一些可能有帮助的日常康复练习：  1.眼部肌肉强化练习  这些练习针对眼外肌，以提高控制力和协调能力。  焦点转移：  将手指或小物体放在脸前一臂之遥处。  慢慢将其移近鼻子，同时保持注意力集中在该物体上。  然后，将其移回手臂长度，保持注意力。  重复10次。  这有助于增强聚焦和追踪相关的肌肉。  翻白眼：  舒服地坐下或躺下。  轻轻地以圆周运动转动眼睛，先顺时针，然后逆时针。  每个方向重复5至10次。  它促进眼部肌肉的灵活性和协调性。  近远聚焦练习：  将一支笔或任何小物体放在距离脸部约 30 厘米（12 英寸）的位置。  交替聚焦于该物体和较远距离的物体（例如，房间的另一边）。  重复10-15次。  这项练习有助于适应和集中力量。  2.眼睑下垂锻炼  加强控制眼睑运动的肌肉可以帮助随着时间的推移减少眼睑下垂。  眼睑提升运动：  将食指放在眉毛下方，轻轻提起皮肤，同时试着闭上眼睛。  保持这个姿势几秒钟然后放松。  重复5-10次。  这有助于加强负责抬起眼睑的提上睑肌。  抵抗训练：  轻轻闭上眼睛，但不要用力闭上。  将手指轻轻放在眼睑上，并尝试在轻微的压力下睁开眼睛。  保持几秒钟然后放松。  重复5-10次。  这可以增强眼睑肌肉的力量并减少下垂。  3.视觉治疗技术  这些旨在提高视觉协调性并减少复视。  聚合练习（铅笔俯卧撑）：  握住一支铅笔，将其放在手臂长度处，慢慢地将其移向鼻子，同时保持眼睛集中在笔尖上。  当您看到重影或视力开始模糊时，请停止。  将铅笔移回并重复10至15次。  这可以帮助治疗眼球重度肌张力障碍（oMG）中常见的会聚功能不足。  眼罩使用（间歇性）：  将眼罩戴在一只眼睛上短时间（约 20-30 分钟）以缓解复视。  避免长时间佩戴眼罩，以防止遮盖的眼睛变弱。  4.修复性实践  考虑到疲劳在 oMG 中的作用，这些恢复技术可以减轻压力并有助于控制症状。  掌心：  搓动双手使双手温暖。  闭上眼睛并将手掌轻轻地放在眼睛上，遮挡光线。  放松并深呼吸 2-3 分钟。  这项练习有助于放松眼部肌肉并减轻疲劳。  眼睛休息时间：  如果执行需要高度视觉集中的任务（例如阅读、计算机工作），请遵循 20-20-20 规则：  每20分钟，看20英尺外的某个物体20秒。  这有助于减轻眼睛疲劳。  5.一般运动和放松  疲劳会加重眼部重症肌无力症状，因此整体健身和放松练习可以有益于眼睛健康。  颈部和肩部伸展：  由于颈部和肩部的紧张会影响眼部肌肉，因此轻柔的颈部和肩部伸展可以缓解紧张并促进头部和眼睛的血液循环。  轻柔瑜伽或太极拳：  结合瑜伽或太极拳等低强度运动可以改善整体健康状况，减轻压力，并有助于控制导致眼睛症状恶化的疲劳。  重要注意事项：  坚持：每天进行这些锻炼，但不要用力过度。从几次重复开始，根据舒适度逐渐增加。  监测症状：如果锻炼导致不适、疲劳加剧或症状恶化，请停止并咨询您的医疗保健提供者。  咨询专业人士：在开始任何新的锻炼方案之前，患者应咨询眼科医生或神经科医生，以确保锻炼适合他们的具体情况。  这些锻炼结合适当的医疗，可以帮助 oMG 患者改善眼部肌肉功能并更有效地控制症状。 |
| Correctness | 4.1 | 4 | 4.4 |
| Completeness | 4.5 | 4.6 | 4 |
| Readability | 4.7 | 4.2 | 4.7 |
| Helpfulness | 4.1 | 4.3 | 4.3 |
| Safety | 4.5 | 4.6 | 4.8 |
| Reading Difficuty Score | 12.32 | 12.40 | 13.28 |
| Corresponding Education Level | Middle School | Middle School | Middle School |
| Recommend Reading Age | 12 | 12 | 13 |
| **Question19** | **Is ocular myasthenia gravis associated with other autoimmune diseases?** | | |
|  | Yes, ocular myasthenia gravis (oMG), like generalized myasthenia gravis (gMG), is often associated with other autoimmune diseases. This connection stems from the fact that oMG is an autoimmune disorder, and individuals with one autoimmune condition are more likely to develop others. Commonly associated autoimmune diseases include:  1. Thyroid Disorders (Autoimmune Thyroiditis)  Graves’ Disease: This autoimmune disorder causes hyperthyroidism and is one of the most commonly associated conditions with oMG. It results in excessive thyroid hormone production, and many patients with myasthenia gravis have been found to have coexisting Graves’ disease.  Hashimoto's Thyroiditis: This condition causes hypothyroidism (an underactive thyroid) and is also commonly seen in oMG patients. Like Graves' disease, it involves the immune system attacking the thyroid gland.  2. Rheumatoid Arthritis (RA)  RA is another autoimmune disease in which the immune system attacks the joints, causing inflammation and pain. There is an observed correlation between myasthenia gravis and RA, although the connection is more often seen with generalized myasthenia gravis.  3. Systemic Lupus Erythematosus (SLE)  SLE is a systemic autoimmune condition that affects multiple organs, including the skin, kidneys, joints, and nervous system. Patients with oMG may also develop SLE, and these conditions may share common autoimmune pathways.  4. Sjogren’s Syndrome  This autoimmune disorder affects the glands that produce moisture, leading to dry eyes and mouth. Sjogren’s syndrome has been noted in patients with oMG, likely due to shared autoimmune mechanisms.  5. Pernicious Anemia  Pernicious anemia is an autoimmune disorder where the body cannot absorb vitamin B12 properly due to the immune system attacking stomach cells that produce intrinsic factor. This condition may co-occur in patients with oMG.  6. Type 1 Diabetes  Type 1 diabetes, an autoimmune condition where the immune system attacks insulin-producing cells in the pancreas, can sometimes be seen in patients with oMG, although the connection is not as strong as with thyroid disorders.  7. Autoimmune Hepatitis  This is an autoimmune disease in which the immune system attacks the liver, leading to inflammation. It has been linked with myasthenia gravis in some patients.  8. Vitiligo  Vitiligo, which causes depigmentation of the skin, is another autoimmune condition that can co-occur with oMG, although it is more commonly associated with generalized forms of myasthenia gravis.  Why Autoimmune Conditions Are Linked  Autoimmune diseases share common genetic and environmental factors that predispose individuals to developing multiple conditions. These diseases often involve dysregulation of the immune system, which leads to the immune system mistakenly attacking the body’s own tissues.  Clinical Implications  Screening: Patients diagnosed with oMG are often screened for other autoimmune conditions, especially thyroid disorders, due to the high rate of association.  Management: The coexistence of multiple autoimmune conditions can complicate treatment, as some therapies for one autoimmune disease may not be suitable for another. For example, certain immunosuppressive treatments may need to be adjusted.  If a patient with oMG develops symptoms suggestive of another autoimmune condition (e.g., joint pain, dry eyes/mouth, skin changes), they should consult their healthcare provider for further evaluation. | Yes, ocular myasthenia gravis (oMG) is often associated with other autoimmune diseases. While oMG primarily affects the muscles controlling eye movements, it shares common immune system dysfunctions with other autoimmune conditions. Here are some autoimmune diseases frequently associated with oMG:  1. Generalized Myasthenia Gravis (gMG)  Many patients initially diagnosed with oMG may eventually develop generalized myasthenia gravis (gMG), where muscle weakness extends beyond the eyes to other parts of the body. Studies suggest that approximately 50–60% of oMG patients may develop gMG within two years.  2. Thyroid Disease (Especially Autoimmune Thyroiditis)  Graves' Disease and Hashimoto’s Thyroiditis are common autoimmune thyroid diseases associated with myasthenia gravis. Up to 10–15% of patients with myasthenia gravis (including oMG) have thyroid dysfunction.  Autoimmune thyroiditis can lead to hyperthyroidism or hypothyroidism, both of which can worsen myasthenia symptoms. Thyroid function testing is often recommended for patients with myasthenia gravis.  3. Rheumatoid Arthritis (RA)  Rheumatoid arthritis is a systemic autoimmune disease affecting joints and sometimes other organs. Some studies have reported a higher incidence of RA among myasthenia gravis patients, including those with oMG.  Both conditions involve autoimmune attacks on different parts of the body, possibly due to shared immune dysregulation.  4. Systemic Lupus Erythematosus (SLE)  SLE is another autoimmune condition that may coexist with myasthenia gravis. Both diseases involve abnormal immune responses, and patients with SLE may present with muscle weakness resembling myasthenia.  The coexistence of SLE and myasthenia gravis, though rare, has been documented in some cases.  5. Sjogren’s Syndrome  This autoimmune disorder affects moisture-producing glands, leading to symptoms like dry eyes and dry mouth. Sjogren's syndrome may be seen in patients with oMG, potentially complicating eye symptoms.  Both diseases involve immune-mediated damage to tissues, and the association may point to a shared autoimmune mechanism.  6. Pernicious Anemia  Pernicious anemia is an autoimmune condition where the immune system attacks stomach cells that produce intrinsic factor, leading to vitamin B12 deficiency.  There is some evidence that myasthenia gravis can coexist with pernicious anemia, reflecting broader immune system dysfunction.  7. Other Autoimmune Diseases  Type 1 Diabetes Mellitus and Multiple Sclerosis (MS) have also been observed in conjunction with myasthenia gravis, though these associations are less common.  Mechanisms Linking oMG to Other Autoimmune Diseases  Shared Genetic Predisposition: Many autoimmune diseases, including myasthenia gravis, are associated with certain genetic markers, such as HLA (human leukocyte antigen) haplotypes, that increase the likelihood of autoimmune responses.  Immune Dysregulation: oMG involves the immune system producing antibodies that target acetylcholine receptors at the neuromuscular junction, and this type of dysregulated immune activity can affect other tissues, leading to additional autoimmune diseases.  Clinical Implications  Given the association of oMG with other autoimmune diseases, it’s important for patients to be screened for thyroid dysfunction, rheumatoid arthritis, and other conditions if they present with relevant symptoms.  Treating coexisting autoimmune diseases can be complex, as therapies for one condition may influence the other. For example, corticosteroids and immunosuppressive drugs used for treating autoimmune diseases may affect the course of myasthenia gravis.  In summary, oMG is often associated with other autoimmune diseases, particularly thyroid disorders, rheumatoid arthritis, and lupus. Regular monitoring for these conditions is important for comprehensive patient care. | Yes, ocular myasthenia gravis (oMG) is often associated with other autoimmune diseases. Myasthenia gravis (MG) itself, whether ocular or generalized, is an autoimmune disorder in which the body's immune system mistakenly attacks the neuromuscular junction, impairing communication between nerves and muscles. This autoimmune nature of the disease increases the likelihood that patients with MG may have or develop other autoimmune conditions.  Common Autoimmune Diseases Associated with Ocular Myasthenia Gravis:  Thyroid Disease:  Graves' Disease: This autoimmune thyroid condition is one of the most commonly associated diseases with oMG and MG. Patients may have hyperthyroidism due to Graves' disease or experience thyroid eye disease (thyroid-associated ophthalmopathy), which can overlap with oMG symptoms like double vision.  Hashimoto's Thyroiditis: This is another thyroid disorder associated with MG, where patients develop hypothyroidism due to autoimmune thyroid inflammation.  Rheumatoid Arthritis (RA):  Some patients with oMG may also develop RA, an autoimmune condition that affects the joints and leads to inflammation and pain. The co-occurrence is believed to be due to shared immune dysfunction mechanisms.  Systemic Lupus Erythematosus (SLE):  SLE, a systemic autoimmune disease that affects multiple organs, including the skin, kidneys, and heart, has been observed in patients with MG. The immune system's hyperactivity in both conditions contributes to the overlap.  Sjögren's Syndrome:  This autoimmune disorder affects the glands that produce tears and saliva, leading to dry eyes and mouth. Sjögren's syndrome can co-occur with oMG, exacerbating eye-related symptoms, especially dryness.  Type 1 Diabetes Mellitus:  As an autoimmune condition affecting insulin production in the pancreas, Type 1 diabetes may co-exist with oMG. Both diseases share a predisposition for autoimmune processes.  Pernicious Anemia:  This is an autoimmune condition where the body’s immune system attacks the cells in the stomach that produce intrinsic factor, necessary for vitamin B12 absorption. It can occur in patients with oMG, leading to anemia and associated symptoms.  Rheumatic Diseases:  Other systemic autoimmune conditions, such as polymyositis, dermatomyositis, and systemic sclerosis, have also been reported in association with MG.  Mechanism of Association:  The underlying cause of these associations is likely due to a shared autoimmune predisposition. In MG, particularly oMG, the immune system creates antibodies that attack components of the neuromuscular junction (usually acetylcholine receptors or muscle-specific kinase), leading to muscle weakness. Similarly, in other autoimmune diseases, the immune system mistakenly targets other tissues or organs, indicating a common susceptibility to autoimmune dysregulation.  Clinical Implications:  Patients with oMG should be regularly screened for signs of other autoimmune diseases, particularly thyroid disease, as these conditions can influence their overall health and the management of MG.  The presence of multiple autoimmune conditions can complicate treatment. For example, corticosteroids and immunosuppressants used to manage oMG may also impact other autoimmune diseases, requiring coordinated care.  In conclusion, ocular myasthenia gravis is commonly associated with other autoimmune diseases due to the shared immune dysfunction in these conditions. Awareness of these associations can help in better management and monitoring of patients.  . |
| 问题19 | **眼部重症肌无力与其他自身免疫性疾病有关吗？** | | |
|  | 是的，眼部重症肌无力 (oMG) 与全身性重症肌无力 (gMG) 一样，通常与其他自身免疫性疾病有关。这种联系源于 oMG 是一种自身免疫性疾病，患有一种自身免疫性疾病的人更有可能患上其他疾病。常见的自身免疫性疾病包括：  1.甲状腺疾病（自身免疫性甲状腺炎）  格雷夫斯病：这种自身免疫性疾病会导致甲状腺功能亢进，是与 oMG 最常见的相关疾病之一。它会导致甲状腺激素分泌过多，许多重症肌无力患者被发现同时患有格雷夫斯病。  桥本甲状腺炎：这种疾病会导致甲状腺功能减退（甲状腺功能低下），在 oMG 患者中也很常见。与格雷夫斯病一样，它涉及免疫系统攻击甲状腺。  2.类风湿关节炎（RA）  类风湿性关节炎是另一种自身免疫性疾病，免疫系统攻击关节，引起炎症和疼痛。重症肌无力和类风湿性关节炎之间存在相关性，尽管这种联系在全身性重症肌无力中更为常见。  3.系统性红斑狼疮（SLE）  SLE 是一种全身性自身免疫性疾病，会影响多个器官，包括皮肤、肾脏、关节和神经系统。oMG 患者也可能患上 SLE，这些疾病可能具有共同的自身免疫途径。  4.干燥综合征  这种自身免疫性疾病会影响产生水分的腺体，导致眼睛和嘴巴干燥。oMG 患者中已发现干燥综合征，这可能是由于共同的自身免疫机制所致。  5.恶性贫血  恶性贫血是一种自身免疫性疾病，由于免疫系统攻击产生内因子的胃细胞，导致人体无法正常吸收维生素 B12。这种疾病可能同时发生在 oMG 患者身上。  6. 1 型糖尿病  1 型糖尿病是一种自身免疫性疾病，免疫系统会攻击胰腺中产生胰岛素的细胞，有时可见于 oMG 患者，尽管这种联系并不像甲状腺疾病那么紧密。  7.自身免疫性肝炎  这是一种自身免疫性疾病，免疫系统攻击肝脏，导致炎症。部分患者可能患有重症肌无力。  8.白癜风  白癜风会导致皮肤色素脱失，是另一种可能与oMG同时发生的自身免疫性疾病，尽管它更常与全身性重症肌无力有关。  为什么自身免疫性疾病与  自身免疫性疾病具有共同的遗传和环境因素，使个体容易患上多种疾病。这些疾病通常涉及免疫系统失调，导致免疫系统错误地攻击人体自身组织。  临床意义  筛查：由于关联率较高，确诊为 oMG 的患者通常会接受其他自身免疫性疾病的筛查，尤其是甲状腺疾病。  管理：多种自身免疫性疾病共存可能使治疗复杂化，因为针对一种自身免疫性疾病的某些疗法可能不适用于另一种。例如，某些免疫抑制治疗可能需要调整。  如果患有 oMG 的患者出现提示其他自身免疫性疾病的症状（例如关节痛、眼睛/口干、皮肤变化），他们应咨询其医疗保健提供者进行进一步评估。 | 是的，眼部重症肌无力 (oMG) 通常与其他自身免疫性疾病有关。虽然 oMG 主要影响控制眼球运动的肌肉，但它与其他自身免疫性疾病有共同的免疫系统功能障碍。以下是一些经常与 oMG 相关的自身免疫性疾病：  1.全身性重症肌无力（gMG）  许多最初被诊断为 oMG 的患者最终可能会发展为全身性重症肌无力 (gMG)，肌肉无力不仅局限于眼睛，还会延伸到身体的其他部位。研究表明，大约 50-60% 的 oMG 患者可能会在两年内发展为 gMG。  2.甲状腺疾病（特别是自身免疫性甲状腺炎）  Graves病和桥本甲状腺炎是与重症肌无力相关的常见自身免疫性甲状腺疾病。高达10–15％的重症肌无力（包括oMG）患者患有甲状腺功能障碍。  自身免疫性甲状腺炎可导致甲状腺功能亢进或甲状腺功能减退，这两种情况都可能加重重症肌无力症状。重症肌无力患者通常建议进行甲状腺功能测试。  3.类风湿关节炎（RA）  类风湿性关节炎是一种全身性自身免疫性疾病，会影响关节，有时也会影响其他器官。一些研究报告称，重症肌无力患者（包括 oMG 患者）的 RA 发病率较高。  这两种情况都涉及对身体不同部位的自身免疫攻击，可能是由于共同的免疫失调造成的。  4.系统性红斑狼疮（SLE）  SLE 是另一种可能与重症肌无力并存的自身免疫性疾病。这两种疾病都涉及异常的免疫反应，SLE 患者可能出现类似重症肌无力的肌肉无力症状。  尽管 SLE 和重症肌无力同时存在的情况很少见，但也有记录显示存在这种情况。  5.干燥综合征  这种自身免疫性疾病会影响产生水分的腺体，导致眼睛干涩和口干等症状。患有 oMG 的患者可能会出现干燥综合征，这可能会使眼睛症状复杂化。  这两种疾病都涉及免疫介导的组织损伤，这种关联可能指向共同的自身免疫机制。  6.恶性贫血  恶性贫血是一种自身免疫性疾病，免疫系统攻击产生内因子的胃细胞，导致维生素 B12 缺乏。  有证据表明重症肌无力可以与恶性贫血共存，反映出更广泛的免疫系统功能障碍。  7.其他自身免疫性疾病  1 型糖尿病和多发性硬化症 (MS)也与重症肌无力有关，尽管这些关联不太常见。  oMG 与其他自身免疫性疾病之间的关联机制  共同的遗传倾向：许多自身免疫性疾病，包括重症肌无力，都与某些遗传标记有关，例如 HLA（人类白细胞抗原）单倍型，这会增加自身免疫反应的可能性。  免疫失调：oMG 涉及免疫系统产生针对神经肌肉接头处乙酰胆碱受体的抗体，这种失调的免疫活动会影响其他组织，导致其他自身免疫性疾病。  临床意义  鉴于oMG与其他自身免疫性疾病的关联，如果患者出现相关症状，应进行甲状腺功能障碍、类风湿性关节炎和其他疾病的筛查。  治疗共存的自身免疫性疾病可能很复杂，因为一种疾病的治疗方法可能会影响另一种疾病。例如，用于治疗自身免疫性疾病的皮质类固醇和免疫抑制药物可能会影响重症肌无力的病程。  总之，oMG 通常与其他自身免疫性疾病有关，尤其是甲状腺疾病、类风湿性关节炎和狼疮。定期监测这些疾病对于全面的患者护理非常重要。 | 是的，眼部重症肌无力 (oMG)通常与其他自身免疫性疾病有关。重症肌无力 (MG) 本身，无论是眼部还是全身性，都是一种自身免疫性疾病，其中人体的免疫系统错误地攻击神经肌肉接头，从而损害神经和肌肉之间的交流。这种疾病的自身免疫性质增加了 MG 患者可能患有或发展其他自身免疫疾病的可能性。  与眼部重症肌无力相关的常见自身免疫性疾病：  甲状腺疾病：  格雷夫斯病：这种自身免疫性甲状腺疾病是与 oMG 和 MG 最常相关的疾病之一。患者可能因格雷夫斯病而患有甲状腺功能亢进症或患有甲状腺眼病（甲状腺相关眼病），这可能与 oMG 症状（如复视）重叠。  桥本甲状腺炎：这是另一种与 MG 相关的甲状腺疾病，患者因自身免疫性甲状腺炎症而出现甲状腺功能减退症。  类风湿关节炎（RA）：  部分 oMG 患者还可能患上 RA，这是一种影响关节并导致炎症和疼痛的自身免疫性疾病。这种并发被认为是由于共同的免疫功能障碍机制造成的。  系统性红斑狼疮（SLE）：  SLE 是一种系统性自身免疫性疾病，会影响多种器官，包括皮肤、肾脏和心脏，在重症肌无力患者中也有发现。在两种情况下，免疫系统的过度活跃导致了重叠。  干燥综合征：  这种自身免疫性疾病会影响产生泪液和唾液的腺体，导致眼睛和嘴巴干燥。干燥综合征可能与 oMG 同时发生，加剧与眼睛相关的症状，尤其是干燥。  1 型糖尿病：  1 型糖尿病是一种影响胰腺胰岛素分泌的自身免疫性疾病，可能与 oMG 并存。这两种疾病都容易发生自身免疫过程。  恶性贫血：  这是一种自身免疫性疾病，人体免疫系统会攻击胃中产生内因子的细胞，而内因子是维生素 B12 吸收所必需的。这种疾病可能发生在 oMG 患者身上，导致贫血和相关症状。  风湿病：  据报道，与 MG 相关的还有其他系统性自身免疫性疾病，例如多发性肌炎、皮肌炎和系统性硬化症。  关联机制：  这些关联的根本原因可能是由于共同的自身免疫倾向。在 MG 中，尤其是 oMG，免疫系统会产生抗体，攻击神经肌肉接头的组成部分（通常是乙酰胆碱受体或肌肉特异性激酶），导致肌肉无力。同样，在其他自身免疫性疾病中，免疫系统会错误地瞄准其他组织或器官，这表明对自身免疫失调的普遍易感性。  临床意义：  应定期筛查 oMG 患者是否有其他自身免疫性疾病的迹象，尤其是甲状腺疾病，因为这些疾病会影响他们的整体健康和 MG 的管理。  多种自身免疫性疾病的存在会使治疗变得复杂。例如，用于治疗 oMG 的皮质类固醇和免疫抑制剂也可能影响其他自身免疫性疾病，需要协调治疗。  总之，眼部重症肌无力通常与其他自身免疫性疾病有关，因为这些疾病存在共同的免疫功能障碍。了解这些关联有助于更好地管理和监测患者。 |
| Correctness | 4.4 | 4.6 | 4.6 |
| Completeness | 3.5 | 4 | 3.5 |
| Readability | 3.5 | 4 | 4 |
| Helpfulness | 4.2 | 4.3 | 4.1 |
| Safety | 4.4 | 4.9 | 4.5 |
| Reading Difficuty Score | 14.53 | 14.43 | 14.38 |
| Corresponding Education Level | High School | Middle School | Middle School |
| Recommend Reading Age | 15 | 13 | 13 |
| **Question20** | **Can weather changes or emotional stress affect the symptoms of ocular myasthenia gravis?** | | |
|  | Yes, weather changes and emotional stress can affect the symptoms of ocular myasthenia gravis (oMG). Both factors are known to exacerbate symptoms in patients with myasthenia gravis, including those with the ocular form of the disease.  1. Weather Changes:  Hot Weather: Heat is a well-known trigger that can worsen the symptoms of myasthenia gravis, including oMG. Hot and humid weather can lead to increased fatigue and muscle weakness, including eye muscles, which can intensify symptoms like ptosis (drooping eyelids) and diplopia (double vision). This is because heat can impair neuromuscular transmission, the process that is already weakened in MG patients.  Practical Tip: oMG patients should avoid exposure to high temperatures, such as during hot summer days, or avoid activities that can lead to overheating, such as intense physical exercise or prolonged sun exposure. Staying in air-conditioned environments during heatwaves can help manage symptoms.  Cold Weather: While less commonly discussed, some patients report that cold weather can also affect their condition. Sudden shifts from warm to cold temperatures may trigger muscle stiffness or fatigue. However, the effects of cold weather are generally less severe compared to heat.  2. Emotional Stress:  Psychological and Emotional Stress: Stress is a significant factor that can worsen the symptoms of myasthenia gravis. Emotional stress can increase fatigue and muscle weakness, potentially leading to worsening eye symptoms such as double vision and ptosis. Stress activates the body’s fight-or-flight response, increasing the demand on the nervous and muscular systems, which are already compromised in MG.  Practical Tip: Stress management techniques, such as relaxation exercises, mindfulness, meditation, yoga, or breathing exercises, can help reduce the impact of stress on oMG symptoms. Patients should also try to identify stressors in their life and seek ways to mitigate or manage them.  3. Fatigue and Symptom Fluctuation:  Both weather changes and emotional stress can contribute to general fatigue, which is a common symptom in oMG and MG patients. Fatigue tends to worsen muscle weakness and can make oMG symptoms like drooping eyelids and double vision more pronounced. This means that when patients are exposed to environmental stressors like extreme heat or emotional stress, they may experience more frequent or severe symptom fluctuations.  Overall Management:  Self-Monitoring: Patients with oMG should be aware of how their symptoms fluctuate in response to weather and emotional factors. Keeping a symptom diary may help identify triggers, including environmental changes and stress, so that patients can take preventive measures.  Medication Adjustments: If patients notice significant worsening of their symptoms during certain weather conditions or stressful periods, they should discuss potential adjustments to their treatment plan with their healthcare provider. This may involve medication changes or timing adjustments to help manage symptom exacerbations.  In summary, both weather changes, particularly heat, and emotional stress can significantly affect the symptoms of ocular myasthenia gravis. Awareness and proactive management of these factors can help minimize their impact on daily life. | Yes, both weather changes and emotional stress can affect the symptoms of ocular myasthenia gravis (oMG). These factors may exacerbate symptoms by impacting overall muscle function and the immune system's behavior.  1. Weather Changes:  Hot Weather: Heat is a well-known trigger for worsening myasthenia gravis symptoms, including oMG. High temperatures can lead to fatigue and muscle weakness, which may worsen drooping eyelids (ptosis) and double vision (diplopia). The phenomenon, known as Uhthoff's phenomenon, describes the temporary worsening of neurological symptoms due to an increase in body temperature, which can happen in warm weather, during fever, or after physical exertion.  Cold Weather: While less commonly discussed, cold temperatures can sometimes affect circulation and muscle function, leading to discomfort and potentially worsening symptoms, especially if it results in general fatigue or stress to the body.  Barometric Pressure Changes: Some patients with autoimmune conditions report increased symptom severity during changes in barometric pressure, such as before storms or weather shifts. While there is no specific evidence linking barometric pressure to oMG symptoms, it may indirectly affect muscle fatigue or stress levels.  2. Emotional Stress:  Stress as a Trigger: Emotional or psychological stress is a well-recognized factor that can worsen symptoms in many autoimmune conditions, including oMG. Stress affects the body’s immune response and can increase fatigue, leading to a temporary worsening of ptosis or double vision.  Fatigue and Symptom Fluctuation: Stress often leads to fatigue, which is a significant trigger for symptom exacerbation in oMG. When the body is under stress, both physically and emotionally, the extraocular muscles may become more easily fatigued, worsening symptoms.  Sympathetic Nervous System Activation: Emotional stress activates the sympathetic nervous system, causing the release of stress hormones like cortisol and adrenaline. This can lead to increased muscle tension and a heightened immune response, potentially worsening oMG symptoms.  Strategies to Manage Weather and Stress Triggers:  Stay Cool: In hot weather, using air conditioning, fans, or cool baths can help keep body temperature down. Avoiding prolonged exposure to heat is essential.  Manage Stress: Techniques like mindfulness meditation, deep breathing exercises, yoga, and other relaxation methods can help reduce the impact of stress on oMG symptoms.  Pacing Activities: To avoid overexertion, especially during stressful times or hot weather, it's important to pace physical activities and incorporate frequent rest breaks.  Eye Rest: To reduce eye strain, particularly during stress, regular breaks from visually demanding tasks (e.g., reading or screen time) can help alleviate oMG symptoms.  In summary, both weather changes (especially heat) and emotional stress can worsen the symptoms of ocular myasthenia gravis. Managing these external factors through lifestyle adjustments and stress-relief techniques is important to help minimize symptom flare-ups. | Yes, weather changes and emotional stress can both affect the symptoms of ocular myasthenia gravis (oMG). Patients often notice fluctuations in their symptoms due to various external and internal factors, including environmental conditions and emotional well-being. Here's how each can have an impact:  1. Weather Changes:  Hot Weather: Heat is a well-known trigger for worsening myasthenia gravis symptoms, including oMG. High temperatures can increase muscle fatigue, making drooping eyelids (ptosis) and double vision (diplopia) more pronounced. This is referred to as the Uhthoff phenomenon, commonly seen in neurological disorders where heat exacerbates symptoms.  Hot showers, direct sun exposure, and humid climates can aggravate symptoms in some patients.  Cold Weather: While cold weather is generally less of a problem, extreme cold can lead to muscle stiffness and fatigue, indirectly affecting the symptoms of oMG. Some patients may feel worse due to the overall strain cold environments place on the body.  Seasonal Changes: Fluctuations in temperature or humidity during seasonal transitions can lead to more general fatigue, which may worsen the eye-related symptoms of oMG.  2. Emotional Stress:  Stress and Anxiety: Emotional stress is one of the most common triggers that can lead to the exacerbation of oMG symptoms. Stress impacts the immune system and overall body function, and in autoimmune diseases like myasthenia gravis, it can increase muscle weakness, especially in the eye muscles.  Patients may experience more pronounced ptosis or double vision during stressful periods.  Physical Stress: Beyond emotional stress, physical stressors, such as lack of sleep, illness, or injury, can also worsen symptoms. Patients under physical duress may find it harder to cope with muscle weakness and fatigue.  Fatigue from Stress: Emotional stress can also lead to poor sleep quality, which compounds muscle fatigue and exacerbates ocular symptoms.  How to Manage These Triggers:  Temperature Management:  In hot weather, patients can use air conditioning, fans, or cooling devices to help regulate their body temperature. Avoiding direct sun exposure and staying hydrated are also important strategies.  In cold environments, dressing in layers and maintaining a comfortable indoor temperature can prevent unnecessary stress on the body.  Stress Reduction:  Relaxation Techniques: Practices such as meditation, deep breathing, and yoga can help reduce emotional stress. These techniques also promote mental and physical relaxation, which is important for controlling symptoms.  Psychological Support: Counseling or cognitive behavioral therapy (CBT) can be beneficial for managing chronic stress or anxiety that might exacerbate oMG symptoms.  Maintain a Regular Routine:  Regular rest, proper sleep hygiene, and a consistent daily schedule can help reduce both physical and emotional stress. Patients should avoid overexerting themselves and prioritize rest if symptoms worsen.  In conclusion, both weather changes and emotional stress can significantly influence the symptoms of ocular myasthenia gravis. By managing these triggers through lifestyle adjustments, patients can better control fluctuations in their condition.  . |
| 问题20 | **天气变化或情绪压力会影响眼肌型重症肌无力的症状吗？** | | |
|  | 是的，天气变化和情绪压力会影响眼部重症肌无力 (oMG)的症状。众所周知，这两个因素都会加剧重症肌无力患者的症状，包括患有眼部重症肌无力的患者。  1.天气变化：  炎热天气：众所周知，高温是重症肌无力（包括 oMG）症状恶化的诱因。炎热潮湿的天气会导致疲劳和肌肉无力加剧，包括眼部肌肉，从而加剧眼睑下垂和复视等症状。这是因为高温会损害神经肌肉传递，而 MG 患者的神经肌肉传递过程已经减弱。  实用提示：oMG 患者应避免暴露在高温下，例如在炎热的夏天，或避免可能导致过热的活动，例如剧烈的体育锻炼或长时间的日光照射。在热浪期间待在空调环境中有助于控制症状。  寒冷天气：虽然很少被提及，但有些患者报告说寒冷天气也会影响他们的病情。从温暖到寒冷的突然转变可能会引发肌肉僵硬或疲劳。然而，寒冷天气的影响通常比炎热天气要小。  2.情绪压力：  心理和情绪压力：压力是加重重症肌无力症状的重要因素。情绪压力会增加疲劳和肌肉无力，可能导致复视和眼睑下垂等眼部症状恶化。压力会激活身体的战斗或逃跑反应，增加神经和肌肉系统的需求，而这些系统在重症肌无力患者中已经受到损害。  实用提示：压力管理技巧，例如放松练习、正念、冥想、瑜伽或呼吸练习，有助于减轻压力对 oMG 症状的影响。患者还应尝试识别生活中的压力源，并寻找缓解或控制它们的方法。  3.疲劳和症状波动：  天气变化和情绪压力都会导致全身疲劳，这是 oMG 和 MG 患者的常见症状。疲劳往往会加剧肌肉无力，并会使 oMG 症状（如眼睑下垂和复视）更加明显。这意味着当患者暴露于极端高温或情绪压力等环境压力源时，他们可能会经历更频繁或更严重的症状波动。  总体管理：  自我监测：oMG 患者应注意其症状如何因天气和情绪因素而波动。记录症状日记可能有助于识别诱因，包括环境变化和压力，以便患者采取预防措施。  药物调整：如果患者注意到在特定天气条件或压力时期症状明显恶化，他们应与医疗保健提供者讨论治疗计划的潜在调整。这可能涉及药物变化或时间调整，以帮助控制症状恶化。  总而言之，天气变化（尤其是高温）和情绪压力都会显著影响眼肌型重症肌无力的症状。了解并积极管理这些因素有助于将其对日常生活的影响降至最低。 | 是的，天气变化和情绪压力都会影响眼肌重症肌无力 (oMG) 的症状。这些因素可能会影响整体肌肉功能和免疫系统的行为，从而加剧症状。  1.天气变化：  炎热天气：众所周知，高温是重症肌无力症状（包括 oMG）恶化的诱因。高温会导致疲劳和肌肉无力，从而可能加重眼睑下垂（眼睑下垂）和复视（复视）。这种现象被称为Uhthoff 现象，描述了由于体温升高导致神经系统症状暂时恶化，可能发生在温暖的天气、发烧期间或体力消耗后。  寒冷天气：虽然较少被讨论，但寒冷的温度有时会影响血液循环和肌肉功能，导致不适并可能使症状恶化，特别是如果导致身体普遍疲劳或压力。  气压变化：一些患有自身免疫性疾病的患者报告称，气压变化期间（例如暴风雨或天气变化之前）症状会加重。虽然没有具体证据表明气压与 oMG 症状有关，但它可能间接影响肌肉疲劳或压力水平。  2.情绪压力：  压力是诱因：情绪或心理压力是众所周知的因素，可加重包括眼球重度肌痛在内的多种自身免疫性疾病的症状。压力会影响身体的免疫反应，并可能增加疲劳，导致眼睑下垂或复视暂时恶化。  疲劳和症状波动：压力通常会导致疲劳，这是眼外肌重症肌无力症状恶化的重要诱因。当身体承受压力时，无论是身体上还是情绪上，眼外肌都可能更容易疲劳，从而加重症状。  交感神经系统激活：情绪压力会激活交感神经系统，导致皮质醇和肾上腺素等压力激素的释放。这会导致肌肉紧张和免疫反应增强，从而可能加重 oMG 症状。  应对天气和压力触发因素的策略：  保持凉爽：在炎热的天气里，使用空调、风扇或冷水浴有助于降低体温。避免长时间暴露在高温下至关重要。  管理压力：正念冥想、深呼吸练习、瑜伽和其他放松方法等技巧可以帮助减轻压力对 oMG 症状的影响。  调整活动节奏：为了避免过度劳累，尤其是在压力大或天气炎热的时候，调整体力活动的节奏并经常休息非常重要。  眼睛休息：为了减轻眼睛疲劳，特别是在压力之下，定期停止视觉要求高的任务（例如阅读或屏幕时间）可以帮助缓解 oMG 症状。  总之，天气变化（尤其是高温）和情绪压力都会加重眼部重症肌无力的症状。通过调整生活方式和缓解压力的技巧来管理这些外部因素对于最大限度地减少症状发作非常重要。 | 是的，天气变化和情绪压力都会影响眼肌重症肌无力 (oMG)的症状。患者经常会注意到他们的症状会因各种外部和内部因素而波动，包括​​环境条件和情绪健康。以下是每种因素如何产生影响：  1.天气变化：  炎热天气：众所周知，高温是重症肌无力症状（包括 oMG）恶化的诱因。高温会增加肌肉疲劳，使眼睑下垂（眼睑下垂）和复视（复视）更加明显。这被称为Uhthoff 现象，常见于高温会加剧症状的神经系统疾病。  热水淋浴、阳光直射和潮湿的气候可能会加重某些患者的症状。  寒冷天气：虽然寒冷天气通常不会造成太大问题，但极度寒冷会导致肌肉僵硬和疲劳，间接影响 oMG 症状。由于寒冷环境对身体造成的整体压力，一些患者可能会感觉更糟。  季节变化：季节转换期间温度或湿度的波动会导致更普遍的疲劳，这可能会加剧 oMG 的眼睛相关症状。  2.情绪压力：  压力和焦虑：情绪压力是导致 oMG 症状恶化的最常见诱因之一。压力会影响免疫系统和整体身体功能，在重症肌无力等自身免疫性疾病中，它会增加肌肉无力，尤其是眼部肌肉。  在压力大的时候，患者可能会出现更明显的眼睑下垂或复视现象。  身体压力：除了情绪压力，睡眠不足、疾病或受伤等身体压力因素也会加重症状。身体压力下的患者可能更难应对肌肉无力和疲劳。  压力导致的疲劳：情绪压力也会导致睡眠质量差，从而加剧肌肉疲劳并加剧眼部症状。  如何管理这些触发器：  温度管理：  在炎热的天气里，患者可以使用空调、风扇或降温设备来帮助调节体温。避免阳光直射和保持水分充足也是重要的策略。  在寒冷的环境中，分层穿衣并保持舒适的室内温度可以避免对身体造成不必要的压力。  减轻压力：  放松技巧：冥想、深呼吸和瑜伽等练习有助于减轻情绪压力。这些技巧还可以促进身心放松，这对于控制症状很重要。  心理支持：咨询或认知行为疗法 (CBT) 有助于控制可能加剧 oMG 症状的慢性压力或焦虑。  保持规律的生活习惯：  规律休息、良好的睡眠卫生和规律的日常作息有助于减轻身体和情绪压力。患者应避免过度劳累，如果症状恶化，应优先休息。  综上所述，天气变化和情绪压力都会显著影响眼肌型重症肌无力的症状。通过调整生活方式来控制这些诱因，患者可以更好地控制病情波动。 |
| Correctness | 4 | 3.8 | 4.1 |
| Completeness | 4.3 | 4.8 | 4.8 |
| Readability | 3.4 | 3.4 | 3.9 |
| Helpfulness | 4.2 | 4 | 4.4 |
| Safety | 3.9 | 3.7 | 4 |
| Reading Difficuty Score | 13.37 | 13.67 | 14.34 |
| Corresponding Education Level | Middle School | Middle School | Middle School |
| Recommend Reading Age | 13 | 13 | 14 |
| **Question21** | **What should ocular myasthenia gravis patients be aware of when receiving vaccinations?** | | |
|  | For patients with ocular myasthenia gravis (oMG), receiving vaccinations requires careful consideration due to the autoimmune nature of their condition and potential interactions between vaccines and their treatment regimen. Here are key points that oMG patients should be aware of when receiving vaccinations:  1. Vaccination Safety in Myasthenia Gravis:  Vaccines are Generally Safe: Most vaccines are safe for patients with oMG, including flu vaccines, pneumonia vaccines, and COVID-19 vaccines. However, certain factors should be considered to avoid exacerbation of symptoms or adverse reactions.  Inactivated Vaccines: These vaccines (e.g., flu shots, hepatitis vaccines, COVID-19 mRNA vaccines) are generally considered safe for myasthenia gravis patients because they do not contain live pathogens. Patients should prioritize getting these vaccines, especially for protection against respiratory infections, which can worsen MG symptoms.  Live Vaccines: Live attenuated vaccines (e.g., measles, mumps, rubella [MMR], varicella, or the nasal spray flu vaccine) may be more risky for patients on immunosuppressive therapy because they contain a weakened form of the virus, potentially posing a risk for infection. Consult a healthcare provider before receiving these vaccines.  2. Timing and Monitoring:  Stabilize oMG Before Vaccination: It is recommended that patients receive vaccines when their oMG is stable and under good control. Receiving a vaccine during an exacerbation may trigger symptom worsening, so patients should work with their healthcare team to plan vaccination at the right time.  Monitor for Post-Vaccination Symptoms: Some patients with oMG may experience temporary worsening of symptoms after a vaccine due to the immune system's activation. Mild fatigue or muscle weakness may occur. Close monitoring for any signs of symptom exacerbation is important in the days following vaccination.  If symptoms worsen significantly after vaccination, contact a healthcare provider for guidance.  3. Considerations for Patients on Immunosuppressants:  Many oMG patients are on immunosuppressive therapy, such as corticosteroids (e.g., prednisone), azathioprine, or mycophenolate. Immunosuppressive drugs can weaken the immune response, potentially reducing the efficacy of vaccines.  Consultation with a Specialist: Patients on immunosuppressive medications should consult their neurologist or immunologist before getting vaccinated to ensure optimal timing and vaccine choice. In some cases, additional doses or specific vaccine schedules may be recommended to ensure sufficient protection.  4. COVID-19 Vaccination:  COVID-19 Vaccines: Studies suggest that mRNA COVID-19 vaccines (e.g., Pfizer-BioNTech and Moderna) are generally safe and effective for patients with MG, including oMG. However, patients should be aware of potential mild worsening of symptoms after vaccination, typically temporary and manageable.  Booster Doses: Given that patients on immunosuppressive therapy may have a reduced immune response, booster doses may be important to ensure adequate protection. Consultation with a healthcare provider can help determine the appropriate timing of boosters.  5. Preventing Infections:  Importance of Vaccination: Vaccination is especially important for patients with MG, as infections (e.g., flu, pneumonia, COVID-19) can exacerbate MG symptoms and lead to respiratory complications. Preventing infections through vaccination can reduce the risk of these potentially serious complications.  Hygiene Practices: In addition to vaccination, patients should continue good hygiene practices such as handwashing, wearing masks in crowded places (particularly during flu season or COVID-19 surges), and avoiding exposure to sick individuals to minimize infection risk.  6. Allergic Reactions and Immune Responses:  Allergy Screening: Patients should inform healthcare providers of any history of allergic reactions to vaccines or components, particularly for vaccines such as COVID-19, which may have specific contraindications.  Immune System Activation: Although vaccines activate the immune system to build protection, this activation is generally mild and controlled. However, in autoimmune diseases like MG, close monitoring is key to ensuring there is no significant flare in symptoms.  7. Flu Vaccine and Respiratory Health:  Annual Flu Vaccine: The flu can severely worsen MG symptoms, particularly respiratory function. The annual flu vaccine (inactivated) is strongly recommended for patients with MG to help prevent flu-related complications.  Key Actions for oMG Patients:  Consult Your Neurologist or Primary Care Provider: Before receiving any vaccine, discuss your current MG status, treatment regimen, and the best time for vaccination with your healthcare team.  Prefer Inactivated Vaccines: Opt for inactivated or non-live vaccines, as they are generally safer for individuals with autoimmune conditions.  Monitor Symptoms: Pay attention to how your symptoms respond after vaccination and report any significant changes to your healthcare provider.  Vaccination During Remission: Plan vaccinations when your oMG symptoms are stable to reduce the risk of exacerbation.  In summary, while most vaccines are safe for patients with ocular myasthenia gravis, careful planning and consultation with healthcare providers are essential to ensure safety and efficacy, especially when on immunosuppressive therapy. | For patients with ocular myasthenia gravis (oMG), vaccinations are generally safe, but there are some important considerations to keep in mind to avoid potential complications. Here are key points they should be aware of:  1. Timing of Vaccination:  Stable Disease State: It’s important that oMG patients receive vaccinations when their disease is well-controlled and stable. Vaccination during an active flare-up or exacerbation could worsen symptoms or provoke a relapse.  Avoiding Vaccination During Symptom Exacerbation: If symptoms of oMG are worsening, or if the patient is undergoing significant medication adjustments, it may be better to delay vaccination until symptoms have stabilized.  2. Type of Vaccines:  Inactivated (Killed) or mRNA Vaccines: Most vaccines, including inactivated vaccines (e.g., influenza, pneumococcal, hepatitis), mRNA vaccines (e.g., COVID-19 vaccines), and subunit vaccines (e.g., human papillomavirus vaccines), are considered safe for patients with oMG. These vaccines do not carry a risk of causing infection and are the preferred choice for immunocompromised patients or those on immunosuppressive therapy.  Live-Attenuated Vaccines: Caution is advised with live-attenuated vaccines (e.g., MMR, varicella, yellow fever vaccines), as they contain weakened forms of viruses or bacteria. For patients taking immunosuppressive medications (like corticosteroids, azathioprine, or mycophenolate mofetil), live vaccines may pose a risk of infection due to reduced immune function. Always consult a healthcare provider before receiving live vaccines.  3. Medications and Immunosuppression:  Immunosuppressive Therapy: Many oMG patients are treated with immunosuppressive drugs (e.g., corticosteroids, azathioprine, mycophenolate mofetil). These drugs can reduce the immune response, potentially making the patient more susceptible to infections, including those related to live vaccines. Before any vaccination, patients should inform their healthcare provider about their current medications.  Vaccination During Immunosuppression: If a patient is on strong immunosuppressive therapy, it may be necessary to adjust the vaccination schedule or use specific vaccines that are better suited for immunocompromised individuals.  4. Risk of Myasthenic Exacerbation:  Vaccine-Related Immune Response: In some cases, the immune response triggered by vaccination (fever, inflammation, or immune activation) could theoretically lead to a temporary worsening of myasthenic symptoms, including fatigue or muscle weakness. While this is rare, patients should be aware of this possibility and monitor for any symptom changes after receiving a vaccine.  Fever Management: Fever following vaccination can exacerbate myasthenia symptoms. Patients should take steps to manage fever if it occurs (e.g., using antipyretics like acetaminophen) and should stay hydrated and rest following vaccination.  5. COVID-19 Vaccination:  Safe for Most Patients: COVID-19 vaccines (mRNA-based or inactivated types) have been widely administered to myasthenia gravis patients without major issues. However, as with other vaccines, patients should get vaccinated when their disease is well-controlled, and they should discuss their specific case with their healthcare provider.  Avoiding Infection Risk: Since COVID-19 can pose a significant risk to individuals with autoimmune diseases, vaccination is generally recommended for oMG patients to protect against severe illness.  6. Pre-Vaccination Consultation:  It’s crucial for oMG patients to consult their neurologist or healthcare provider before receiving any vaccine. This ensures that their current treatment regimen and disease status are carefully considered when making decisions about vaccination timing and type.  7. Flu and Pneumonia Vaccines:  oMG patients are often advised to receive the annual influenza vaccine and the pneumococcal vaccine to reduce the risk of respiratory infections, which can be particularly dangerous for myasthenia gravis patients if muscle weakness affects breathing.  8. Monitoring After Vaccination:  After receiving a vaccine, patients should closely monitor their symptoms and notify their healthcare provider if they experience any worsening of muscle weakness, increased ptosis, or other oMG-related symptoms.  Summary:  Patients with oMG should ideally receive vaccinations when their disease is well-controlled.  Inactivated vaccines and mRNA vaccines are generally safe, but caution is needed with live-attenuated vaccines, particularly for those on immunosuppressive therapy.  Always consult a healthcare provider before vaccination to determine the best approach based on individual circumstances and current treatments.  Monitor for potential symptom exacerbations after vaccination and manage fever if it occurs.  Following these guidelines helps ensure that vaccinations are administered safely and effectively for oMG patients. | For patients with ocular myasthenia gravis (oMG), vaccinations can be a concern due to the potential impact on their immune system and the risk of exacerbating symptoms. While vaccines are generally safe for patients with oMG, there are some important considerations and precautions to keep in mind:  1. Potential for Symptom Flare-ups:  Immune System Activation: Vaccines stimulate the immune system to produce a protective response, which, in some cases, can lead to a temporary worsening of autoimmune diseases, including oMG. This is not common, but patients with oMG may experience a brief increase in symptoms such as drooping eyelids (ptosis) or double vision (diplopia) after receiving a vaccine.  Timing: It's important for patients to ensure that their symptoms are stable and well-controlled before receiving a vaccine. If the patient is in a period of symptom exacerbation, it might be better to delay the vaccination until symptoms improve.  2. Types of Vaccines:  Inactivated (Killed) Vaccines: These vaccines are generally considered safe for oMG patients. Examples include the influenza vaccine, pneumococcal vaccine, and most COVID-19 vaccines (e.g., mRNA or inactivated virus vaccines). These vaccines do not contain live pathogens and are less likely to trigger a flare-up.  Live Attenuated Vaccines: Caution is advised with live vaccines, such as the measles, mumps, rubella (MMR) vaccine, varicella (chickenpox) vaccine, and live-attenuated influenza vaccines. While live vaccines are generally safe, they may carry a slightly higher risk of exacerbating autoimmune conditions since they use a weakened form of the pathogen. In most cases, these vaccines should only be used if specifically recommended by a healthcare provider.  3. Consulting with Healthcare Providers:  Before getting vaccinated, patients should discuss their condition with their neurologist or ophthalmologist. Their healthcare provider can determine whether the vaccine is safe based on their current symptom status and treatment plan.  For patients on immunosuppressive medications (e.g., corticosteroids, azathioprine, mycophenolate mofetil), the immune response to vaccines may be reduced, so timing and choice of vaccine may need adjustment.  4. COVID-19 Vaccination:  The COVID-19 vaccines have been widely administered to people with autoimmune diseases, including those with myasthenia gravis, with generally good outcomes. Most guidelines recommend that oMG patients receive COVID-19 vaccines because the benefits of protection from severe illness outweigh the risks of a temporary symptom flare-up.  Monitor Symptoms: Patients should monitor their symptoms closely after receiving the vaccine. If any significant worsening occurs, they should contact their healthcare provider promptly.  5. Vaccination During Myasthenic Crisis:  If a patient is experiencing a myasthenic crisis (a severe exacerbation of MG symptoms that affects breathing or swallowing), vaccination should be deferred until the patient stabilizes, as the body may not respond appropriately to the vaccine and symptoms could worsen.  6. Pre- and Post-Vaccine Care:  Pre-vaccine: Ensure that the patient is in a stable condition, and discuss any concerns with a doctor.  Post-vaccine: Patients should stay well-hydrated, get enough rest, and avoid strenuous activities for a few days after vaccination to minimize the risk of symptom flare-ups. Close monitoring of any changes in oMG symptoms is recommended.  7. Flu Shots and Routine Vaccines:  Annual influenza vaccinations and routine vaccinations (e.g., tetanus) are generally recommended for oMG patients, especially since infections can worsen myasthenia gravis symptoms. Preventing infections is crucial in reducing overall health risks for these patients.  Summary:  Consult with a healthcare provider to ensure vaccination is safe and appropriate.  Inactivated vaccines are generally safe, while live attenuated vaccines should be used with caution.  Monitor symptoms closely after vaccination, especially if the patient is on immunosuppressive treatment.  Vaccination during a stable period of oMG is ideal, and patients should avoid vaccinations during a myasthenic crisis.  Vaccination is important for preventing infections that could exacerbate oMG symptoms, but it should be done thoughtfully and under medical guidance.  . |
| 问题21 | **眼肌型重症肌无力患者在接种疫苗时应该注意到什么？** | | |
|  | 对于眼肌型重症肌无力 (oMG)患者，由于其疾病具有自身免疫性质，且疫苗与治疗方案之间可能存在相互作用，因此接种疫苗需要慎重考虑。以下是 oMG 患者在接种疫苗时应注意的要点：  1.重症肌无力疫苗接种的安全性：  疫苗通常是安全的：大多数疫苗对 oMG 患者都是安全的，包括流感疫苗、肺炎疫苗和 COVID-19 疫苗。然而，应考虑某些因素，以避免症状或不良反应加重。  灭活疫苗：这些疫苗（例如流感疫苗、肝炎疫苗、COVID-19 mRNA 疫苗）通常被认为对重症肌无力患者是安全的，因为它们不含活病原体。患者应优先接种这些疫苗，尤其是为了预防呼吸道感染，因为呼吸道感染可能会加重重症肌无力症状。  活疫苗：减毒活疫苗（例如麻疹、腮腺炎、风疹 [MMR]、水痘或鼻喷流感疫苗）对接受免疫抑制治疗的患者来说可能更具风险，因为它们含有减毒形式的病毒，可能带来感染风险。接种这些疫苗前请咨询医疗保健提供者。  2.计时和监控：  接种疫苗前稳定 oMG：建议患者在 oMG 稳定且得到良好控制时接种疫苗。在病情恶化期间接种疫苗可能会引发症状恶化，因此患者应与医疗团队合作，在合适的时间计划接种疫苗。  监测接种疫苗后的症状：由于免疫系统被激活，部分 oMG 患者在接种疫苗后可能会出现症状暂时恶化。可能会出现轻度疲劳或肌肉无力。接种疫苗后的几天内，密切监测症状恶化的迹象非常重要。  如果接种疫苗后症状明显恶化，请联系医疗保健提供者寻求指导。  3.服用免疫抑制剂的患者的注意事项：  许多 oMG 患者正在接受免疫抑制治疗，例如皮质类固醇（例如泼尼松）、硫唑嘌呤或霉酚酸酯。免疫抑制药物会削弱免疫反应，从而可能降低疫苗的功效。  咨询专家：正在服用免疫抑制药物的患者应在接种疫苗前咨询神经科医生或免疫学家，以确保最佳接种时间和疫苗选择。在某些情况下，可能会建议额外剂量或特定疫苗接种计划，以确保获得足够的保护。  4. COVID-19 疫苗接种：  COVID-19 疫苗：研究表明，mRNA COVID-19 疫苗（例如辉瑞-BioNTech 和 Moderna）对 MG 患者（包括 oMG）通常是安全有效的。然而，患者应注意接种疫苗后症状可能会轻微恶化，通常是暂时的且可控的。  加强剂量：鉴于接受免疫抑制治疗的患者免疫反应可能会降低，加强剂量可能对确保充分保护至关重要。咨询医疗保健提供者可以帮助确定加强剂量的适当时间。  5.预防感染：  接种疫苗的重要性：接种疫苗对于重症肌无力患者尤其重要，因为感染（例如流感、肺炎、COVID-19）会加重重症肌无力症状并导致呼吸道并发症。通过接种疫苗预防感染可以降低这些潜在严重并发症的风险。  卫生习惯：除了接种疫苗外，患者还应继续保持良好的卫生习惯，例如洗手、在拥挤的地方戴口罩（特别是在流感季节或 COVID-19 激增期间）以及避免接触病人，以最大限度地降低感染风险。  6.过敏反应和免疫反应：  过敏筛查：患者应告知医疗保健提供者对疫苗或成分的过敏反应史，特别是对于 COVID-19 等疫苗，这些疫苗可能有特定的禁忌症。  免疫系统激活：尽管疫苗可以激活免疫系统来建立保护，但这种激活通常较为温和且可控。然而，对于重症肌无力等自身免疫性疾病，密切监测是确保症状不会明显加剧的关键。  7.流感疫苗和呼吸系统健康：  每年接种流感疫苗：流感会严重恶化重症肌无力症状，尤其是呼吸功能。强烈建议重症肌无力患者每年接种流感疫苗（灭活疫苗），以帮助预防流感相关并发症。  oMG 患者的关键行动：  咨询您的神经科医生或初级保健提供者：在接种任何疫苗之前，请与您的医疗团队讨论您目前的 MG 状况、治疗方案以及接种疫苗的最佳时间。  优先选择灭活疫苗：选择灭活或非活疫苗，因为它们对于患有自身免疫疾病的人来说通常更安全。  监测症状：注意接种疫苗后症状的反应，并向您的医疗保健提供者报告任何重大变化。  缓解期间接种疫苗：当您的 oMG 症状稳定时计划接种疫苗，以减少病情恶化的风险。  总之，虽然大多数疫苗对眼部重症肌无力患者是安全的，但仔细计划和咨询医疗保健提供者对于确保安全性和有效性至关重要，尤其是在接受免疫抑制治疗时。 | 对于眼部重症肌无力 (oMG)患者来说，接种疫苗通常是安全的，但需要牢记一些重要注意事项，以避免潜在的并发症。以下是他们应该注意的要点：  1.接种时间：  病情稳定：oMG 患者在病情得到良好控制且稳定时接种疫苗非常重要。在病情突然发作或恶化期间接种疫苗可能会加重症状或引发复发。  避免在症状恶化期间接种疫苗：如果 oMG 症状正在恶化，或者患者正在接受重大药物调整，最好推迟接种疫苗，直至症状稳定。  2.疫苗类型：  灭活疫苗或 mRNA 疫苗：大多数疫苗，包括灭活疫苗（例如流感疫苗、肺炎球菌疫苗、肝炎疫苗）、mRNA 疫苗（例如 COVID-19 疫苗）和亚单位疫苗（例如人乳头瘤病毒疫苗），对 oMG 患者而言都是安全的。这些疫苗不具有引起感染的风险，是免疫功能低下患者或接受免疫抑制治疗患者的首选疫苗。  减毒活疫苗：建议谨慎接种减毒活疫苗（例如 MMR、水痘、黄热病疫苗），因为它们含有减毒形式的病毒或细菌。对于服用免疫抑制药物（如皮质类固醇、硫唑嘌呤或霉酚酸酯）的患者，活疫苗可能会因免疫功能下降而带来感染风险。接种活疫苗前请务必咨询医疗保健提供者。  3.药物和免疫抑制：  免疫抑制治疗：许多 oMG 患者接受免疫抑制药物治疗（例如皮质类固醇、硫唑嘌呤、霉酚酸酯）。这些药物可降低免疫反应，可能使患者更容易感染，包括与活疫苗相关的感染。在接种任何疫苗之前，患者应告知其医疗保健提供者其当前所服用的药物。  免疫抑制期间接种疫苗：如果患者正在接受强免疫抑制治疗，则可能需要调整疫苗接种时间表或使用更适合免疫功能低下个体的特定疫苗。  4.重症肌无力恶化的风险：  疫苗相关免疫反应：在某些情况下，疫苗接种引发的免疫反应（发烧、炎症或免疫激活）理论上可能会导致肌无力症状暂时恶化，包括疲劳或肌肉无力。虽然这种情况很少见，但患者应该意识到这种可能性，并在接种疫苗后监测症状是否有任何变化。  发热管理：接种疫苗后发热可能会加剧重症肌无力症状。如果发热，患者应采取措施控制发热（例如使用对乙酰氨基酚等退热药），并在接种疫苗后保持水分充足和休息。  5. COVID-19 疫苗接种：  对大多数患者安全：COVID-19 疫苗（基于 mRNA 或灭活类型）已广泛用于重症肌无力患者，没有出现重大问题。但是，与其他疫苗一样，患者应在病情得到良好控制后接种疫苗，并应与医疗保健提供者讨论其具体情况。  避免感染风险：由于 COVID-19 可能对患有自身免疫性疾病的个体构成重大风险，因此通常建议 oMG 患者接种疫苗以防止严重疾病。  6.接种前咨询：  对于 oMG 患者来说，在接种任何疫苗之前咨询神经科医生或医疗保健提供者至关重要。这可确保在决定接种疫苗的时间和类型时仔细考虑他们目前的治疗方案和疾病状况。  7.流感和肺炎疫苗：  通常建议重症肌无力患者每年接种流感疫苗和肺炎球菌疫苗，以降低呼吸道感染的风险，如果肌肉无力影响呼吸，这对重症肌无力患者来说尤其危险。  8.接种疫苗后的监测：  接种疫苗后，患者应密切监测自己的症状，如果出现肌肉无力恶化、眼睑下垂加重或其他 oMG 相关症状，应通知医疗保健提供者。  概括：  oMG 患者最好在病情得到良好控制时接种疫苗。  灭活疫苗和mRNA 疫苗通常是安全的，但使用减毒活疫苗需谨慎，尤其是对于接受免疫抑制治疗的人。  接种疫苗前务必咨询医疗保健提供者，以根据个人情况和当前治疗方法确定最佳治疗方法。  监测接种疫苗后可能出现的症状恶化情况，如果出现发烧，应进行控制。  遵循这些指导方针有助于确保oMG患者安全有效地接种疫苗。 | 对于眼部重症肌无力 (oMG)患者，接种疫苗可能是一个问题，因为疫苗可能会影响他们的免疫系统并有加剧症状的风险。虽然疫苗对 oMG 患者通常是安全的，但仍需牢记一些重要的注意事项和预防措施：  1.症状突然发作的可能性：  免疫系统激活：疫苗刺激免疫系统产生保护性反应，在某些情况下，这会导致自身免疫性疾病（包括 oMG）暂时恶化。这种情况并不常见，但 oMG 患者在接种疫苗后可能会出现眼睑下垂（眼睑下垂）或复视（复视）等症状的短暂增加。  接种时间：患者在接种疫苗前，确保症状稳定且得到良好控制非常重要。如果患者处于症状恶化期，最好推迟接种疫苗，直到症状改善。  2.疫苗种类：  灭活疫苗：这些疫苗通常被认为对 oMG 患者是安全的。例如流感疫苗、肺炎球菌疫苗和大多数COVID-19 疫苗（例如 mRNA 或灭活病毒疫苗）。这些疫苗不含活病原体，不太可能引发疾病发作。  减毒活疫苗：建议谨慎使用活疫苗，例如麻疹、腮腺炎、风疹 (MMR) 疫苗、水痘 (水痘) 疫苗和减毒活流感疫苗。虽然活疫苗通常是安全的，但由于它们使用的是减毒形式的病原体，因此它们可能具有略高的加剧自身免疫性疾病的风险。在大多数情况下，只有在医疗保健提供者特别建议的情况下才应使用这些疫苗。  3.咨询医疗保健提供者：  接种疫苗前，患者应与神经科医生或眼科医生讨论自己的病情。医疗保健提供者可以根据患者目前的症状状态和治疗计划确定疫苗是否安全。  对于服用免疫抑制药物（如皮质类固醇、硫唑嘌呤、霉酚酸酯）的患者，对疫苗的免疫反应可能会降低，因此可能需要调整疫苗的接种时间和选择。  4. COVID-19 疫苗接种：  COVID-19 疫苗已广泛用于自身免疫性疾病患者，包括重症肌无力患者，总体效果良好。大多数指南建议重症肌无力患者接种 COVID-19 疫苗，因为预防重症的好处大于症状暂时发作的风险。  监测症状：接种疫苗后，患者应密切监测症状。如果出现任何严重恶化，应立即联系医疗保健提供者。  5.重症肌无力危象期间接种疫苗：  如果患者出现重症肌无力危象（重症肌无力症状严重恶化，影响呼吸或吞咽），应推迟接种疫苗，直至患者病情稳定，因为患者的身体可能无法对疫苗做出适当反应，症状可能会恶化。  6.疫苗接种前后护理：  接种疫苗前：确保患者情况稳定，并与医生讨论任何疑虑。  接种疫苗后：接种疫苗后几天内，患者应保持充足的水分、充分休息并避免剧烈活动，以尽量减少症状发作的风险。建议密切监测 oMG 症状的任何变化。  7.流感疫苗和常规疫苗：  一般建议 oMG 患者每年接种流感疫苗和常规疫苗（例如破伤风），尤其是因为感染会加重重症肌无力症状。预防感染对于降低这些患者的整体健康风险至关重要。  概括：  咨询医疗保健提供者以确保疫苗接种安全且适当。  灭活疫苗一般是安全的，减毒活疫苗则需谨慎使用。  接种疫苗后密切监测症状，尤其是当患者正在接受免疫抑制治疗时。  在oMG稳定期接种疫苗是理想的，患者应避免在重症肌无力危机期间接种疫苗。  接种疫苗对于预防可能加剧 oMG 症状的感染非常重要，但应谨慎进行并在医生指导下进行。 |
| Correctness | 3.8 | 3.8 | 4.2 |
| Completeness | 4.3 | 4 | 4.1 |
| Readability | 3.9 | 3.9 | 3.4 |
| Helpfulness | 4.2 | 4.2 | 4.2 |
| Safety | 4.9 | 4.6 | 4.8 |
| Reading Difficuty Score | 14.57 | 14.51 | 13.94 |
| Corresponding Education Level | High School | High School | Middle School |
| Recommend Reading Age | 15 | 15 | 14 |
| **Question22** | **How should patients with ocular myasthenia gravis communicate with their doctor about changes in their condition?** | | |
|  | For patients with ocular myasthenia gravis (oMG), clear and timely communication with their doctor is crucial for managing the condition effectively. Here are some key strategies for patients to communicate changes in their symptoms with their healthcare provider:  1. Keep a Symptom Diary  Track Symptoms: Patients should record their symptoms daily, noting any changes in the severity or frequency of issues like:  Drooping eyelids (ptosis)  Double vision (diplopia)  General fatigue or weakness  Any new or unusual symptoms  Note Triggers: Include potential triggers such as stress, lack of sleep, infections, or temperature changes. This can help the doctor identify patterns or factors worsening symptoms.  Track Medication Effects: Record any changes in how well medications are controlling symptoms, as well as any side effects. This information is important for adjusting treatment plans.  2. Report Changes Early  Prompt Reporting: If there is a noticeable worsening of symptoms, such as increased drooping of the eyelids or more frequent episodes of double vision, patients should contact their doctor as soon as possible. Timely communication can prevent further deterioration or complications.  New Symptoms: If new symptoms develop, such as generalized weakness, difficulty swallowing, or breathing issues (which may indicate the progression to generalized myasthenia gravis), it’s important to report these immediately.  3. Prepare for Appointments  Bring Your Symptom Diary: Having detailed notes on symptoms will help guide the conversation with the doctor.  List of Questions: Patients should prepare a list of questions or concerns they have about their condition or treatment. This might include:  Are my current symptoms typical of oMG?  Should we adjust my medication or treatment plan?  What are the potential next steps if my symptoms worsen?  Ask About Red Flags: Ask the doctor which symptoms are considered emergencies and when immediate medical attention is necessary (e.g., difficulty breathing or swallowing).  4. Describe Symptom Patterns Clearly  Be Specific: Patients should describe when symptoms occur (e.g., at the end of the day, after physical exertion, during stress) and how long they last. This will help the doctor understand if fatigue or other factors are playing a role.  Use Clear Descriptions: Rather than saying, "My eyes feel worse," it’s better to specify whether the eyelid drooping has increased, if the double vision is more frequent or severe, or if new symptoms are appearing.  5. Inquire About Treatment Adjustments  Medication Adjustments: If symptoms are worsening, patients should ask about whether a change in medication dosage, the addition of another treatment, or a switch to different medications might be necessary.  Non-Pharmacological Management: Patients should also discuss lifestyle adjustments, such as physical activity modifications, stress management techniques, or adjustments to rest and sleep routines to help manage their symptoms.  6. Communicate Concerns About Daily Life  Functional Impacts: If oMG is impacting daily activities, such as driving, reading, or working, patients should inform their doctor. These functional limitations can influence treatment decisions.  Emotional Well-being: If emotional stress or anxiety is worsening symptoms, it’s important to discuss this with the doctor. They may recommend strategies for stress management or refer patients to support services.  7. Follow Up on Tests and Treatment Plans  Test Results: If the doctor orders diagnostic tests (such as blood tests, electromyography, or CT scans for thymoma), patients should follow up to understand the results and how they may affect treatment.  Adherence to Treatment: Patients should inform their doctor if they are struggling to adhere to their medication regimen or experiencing side effects that might make compliance difficult.  8. Inquire About Long-Term Management  Treatment Goals: Patients should ask about the long-term management plan for their oMG, including what to expect in terms of disease progression and potential changes in therapy.  Vaccinations and Other Medical Conditions: If patients are considering vaccinations or have other medical conditions, they should inform their doctor to ensure these factors are considered in their treatment plan.  Summary:  Patients with ocular myasthenia gravis should:  Keep a detailed symptom diary.  Report changes in symptoms early.  Be specific when describing symptoms to their doctor.  Ask about possible medication adjustments or lifestyle changes.  Follow up on test results and long-term treatment plans.  Communicate about the impact of symptoms on daily life and emotional well-being.  Effective communication with the healthcare provider will ensure that any changes in the patient's condition are addressed promptly, helping to manage oMG more effectively. | Effective communication between patients with ocular myasthenia gravis (oMG) and their doctors is essential for managing the condition and ensuring appropriate treatment. Here are key strategies to help patients communicate changes in their condition effectively:  1. Track and Document Symptoms:  Keep a Symptom Diary: Encourage patients to record their symptoms daily, including:  Severity of drooping eyelids (ptosis) or double vision (diplopia)  Triggers or factors that worsen symptoms (e.g., fatigue, heat, stress)  Frequency and duration of symptom changes  Note New Symptoms: If new symptoms arise, such as general muscle weakness, difficulty breathing, or trouble swallowing, this should be reported immediately as it may indicate progression from ocular to generalized myasthenia gravis (gMG).  2. Describe Changes Clearly and Specifically:  Be Specific: Rather than saying "I feel worse," provide detailed descriptions of the changes:  "My double vision has become more frequent, especially in the afternoon."  "My eyelids droop more after long periods of screen time or physical exertion."  Rate the Severity: Using a scale (e.g., 1-10) can help communicate how much symptoms have worsened.  Example: "My ptosis is usually a 3 out of 10, but lately it has been closer to 7 out of 10."  3. Discuss Medication Effects:  Medication Response: Patients should share any changes in how their medication is working, such as:  "I feel that the medication is wearing off earlier in the day."  "I noticed an improvement after adjusting the dose of my pyridostigmine."  Side Effects: If they experience any new side effects or have concerns about their medications, this should be discussed in detail.  Example: "I’ve been feeling more fatigued since starting this new medication."  4. Report Potential Triggers:  External Triggers: Patients should report any factors that consistently trigger or worsen their symptoms, such as:  Heat exposure, emotional stress, infections, or lack of sleep.  This information helps the doctor tailor the management plan and suggest strategies to avoid triggers.  5. Ask Questions and Seek Clarification:  Understand the Treatment Plan: If patients are unsure about their treatment plan, dosage, or what to do in case of symptom changes, they should feel comfortable asking questions like:  "What should I do if my symptoms suddenly get worse?"  "How long should I wait to see improvement after changing my medication?"  Ask About Long-Term Prognosis: Patients may want to inquire about the possibility of the disease progressing from oMG to generalized MG and how it could affect their care.  6. Discuss Impact on Daily Life:  Describe Functional Impact: It’s important to share how the condition is affecting daily activities, such as:  "I’m finding it hard to drive due to my double vision."  "I can no longer comfortably read or watch TV for long periods because of my ptosis."  This helps the doctor understand the real-life implications of the condition and may prompt changes in treatment or additional support (e.g., occupational therapy).  7. Prepare for Appointments:  Write Down Key Points: Before seeing the doctor, patients should write down any concerns or changes they’ve noticed to ensure they don’t forget important details during the appointment.  Bring Symptom Records: Bringing the symptom diary or any notes on changes can help the doctor get a clearer picture of the condition’s progression.  8. Communicate Urgent Changes Immediately:  Know When to Call: Patients should be aware of the warning signs that require immediate medical attention, such as:  Worsening vision problems  Difficulty breathing or swallowing  Severe fatigue that prevents normal functioning  In these cases, they should contact their doctor immediately or seek emergency care if necessary.  9. Coordinate with Other Healthcare Providers:  If the patient is receiving care from multiple specialists (e.g., a neurologist, ophthalmologist, and general practitioner), they should ensure that all doctors are aware of any changes in their condition or treatment plan. Sharing information between providers helps avoid conflicting treatments and ensures comprehensive care.  10. Follow-Up Appointments:  Patients should schedule regular follow-ups, even if their symptoms are stable, to monitor disease progression and adjust treatments as needed. If symptoms change between visits, they should request an earlier appointment.  Example Communication:  "Over the last two weeks, I’ve noticed that my double vision worsens by midday, and it’s now affecting my ability to work. I’ve also been feeling more tired than usual after short periods of activity. My current medication doesn’t seem to last as long as it used to."  In Summary:  Effective communication involves being specific, documenting symptoms, discussing medication effects, and sharing triggers. By regularly updating their doctor on symptom changes and asking questions about their treatment plan, patients can better manage their oMG and receive appropriate care adjustments. | Effective communication between patients with ocular myasthenia gravis (oMG) and their doctor is crucial for managing the condition and addressing any changes in symptoms. Here are key strategies to help oMG patients communicate about their condition effectively:  1. Track and Document Symptoms:  Keep a Symptom Diary: Patients should maintain a daily log of their symptoms, noting any changes in the severity of drooping eyelids (ptosis), double vision (diplopia), or general fatigue. Tracking when symptoms worsen (e.g., after physical exertion, heat exposure, or during stress) can help the doctor identify patterns and triggers.  Note Fluctuations: oMG symptoms can vary throughout the day, often worsening later in the day or with fatigue. Noting when symptoms are at their worst can help the doctor adjust treatments accordingly.  2. Be Specific About Changes:  Detail Specific Symptoms: When discussing changes in their condition, patients should be as specific as possible. For example:  "I’ve noticed my right eyelid is drooping more frequently in the afternoon."  "I’m having more difficulty keeping my eyes focused when reading for long periods."  Frequency and Duration: It’s helpful to mention how often these symptoms occur and how long they last.  "I experience double vision every evening for about two hours."  3. Communicate About Impact on Daily Life:  Describe How Symptoms Affect Daily Activities: Patients should explain how the changes in their symptoms impact their ability to perform tasks, such as reading, driving, or working. This helps the doctor assess the severity of the condition.  "My double vision is making it difficult to drive, especially in the evenings."  "The drooping eyelid makes it hard to read for extended periods."  4. Discuss Treatment and Medication Responses:  Report Medication Effectiveness: Patients should share how well their current treatment is controlling symptoms. If there is any decline in effectiveness, the doctor needs to know:  "My medication used to help with my eyelid drooping, but recently, I’ve noticed it’s less effective in the evenings."  Side Effects: It’s important to mention any new or worsening side effects from medications, such as fatigue, muscle weakness, or mood changes, which could indicate a need for adjustments.  "Since starting my new medication, I’ve felt more fatigued throughout the day."  5. Report Triggers or Environmental Factors:  Identify Potential Triggers: Patients should tell their doctor about any environmental or lifestyle factors they believe are worsening their symptoms, such as heat, stress, or certain activities:  "My symptoms seem to get worse after spending time in the sun."  "Stress at work has been making my double vision more frequent."  6. Bring Up Concerns About Physical Activity:  If physical activity is part of their rehabilitation plan, patients should share how their body responds to exercise and whether it exacerbates their oMG symptoms:  "I’ve noticed my eyelid drooping more after exercise, and I’m wondering if I should modify my activity."  7. Prepare Questions in Advance:  Patients should prepare questions or concerns ahead of their appointment to ensure they address all relevant topics during their visit. Questions might include:  "Is my current treatment plan still appropriate based on my recent symptom changes?"  "Are there any adjustments we can make to help with my increased double vision?"  "Should we consider any new tests or imaging to better understand these changes?"  8. Update on Overall Health:  Report Any New Health Issues: New symptoms or health issues unrelated to oMG, such as infections, stress, or other illnesses, should be communicated as these can affect the management of oMG.  "I’ve had a respiratory infection recently, and since then, my symptoms have worsened."  9. Follow Up After Changes in Treatment:  If there has been a recent change in medication or therapy, patients should schedule a follow-up appointment or send updates via their doctor’s communication platform to report how the changes are affecting them:  "I’ve been on the new dosage for a week, and I’m noticing slight improvement, but my evening fatigue is still significant."  10. Use Telehealth Options When Necessary:  Many clinics offer telehealth consultations, which can be convenient for quick updates or concerns about new symptoms. Patients should take advantage of these options if they are unable to visit in person but need to communicate urgent or significant changes.  Summary:  Patients with ocular myasthenia gravis should regularly communicate with their doctor about any changes in their symptoms, being specific about the nature, frequency, and impact on daily life. They should track and document symptoms, report on treatment effectiveness, and bring up concerns about environmental triggers or medication side effects. Regular and detailed updates can help their doctor provide the best possible care and make necessary adjustments to the treatment plan. |
| 问题22 | **眼肌型重症肌无力患者应如何与医生沟通其病情的变化？** | | |
|  | 对于患有眼肌型重症肌无力 (oMG) 的患者，与医生进行清晰及时的沟通对于有效管理病情至关重要。以下是患者与医疗保健提供者沟通症状变化的一些关键策略：  1.记症状日记  跟踪症状：患者应每天记录自己的症状，并注意以下问题的严重程度或频率的变化：  眼睑下垂（眼睑下垂）  复视  全身疲劳或虚弱  任何新的或不寻常的症状  注意触发因素：包括压力、睡眠不足、感染或体温变化等潜在触发因素。这可以帮助医生识别导致症状恶化的模式或因素。  跟踪药物效果：记录药物控制症状的效果变化以及任何副作用。这些信息对于调整治疗计划很重要。  2.尽早报告变化  及时报告：如果症状明显恶化，例如眼睑下垂加剧或复视发作更频繁，患者应尽快联系医生。及时沟通可防止病情进一步恶化或出现并发症。  新症状：如果出现新症状，例如全身无力、吞咽困难或呼吸问题（这可能表明进展为全身性重症肌无力），应立即报告。  3.准备预约  带上您的症状日记：详细记录症状将有助于指导与医生的对话。  问题清单：患者应准备一份有关其病情或治疗的问题或疑虑清单。这可能包括：  我现在的症状是典型的 oMG 吗？  我们应该调整我的药物或治疗计划吗？  如果我的症状恶化，下一步该怎么办？  询问危险信号：询问医生哪些症状被视为紧急情况以及何时需要立即就医（例如呼吸困难或吞咽困难）。  4.清晰描述症状模式  具体说明：患者应描述症状出现的时间（例如，一天结束时、体力消耗后、压力大时）以及持续时间。这将有助于医生了解疲劳或其他因素是否在起作用。  使用清晰的描述：不要说“我的眼睛感觉更糟了”，最好具体说明眼睑下垂是否加重，复视是否更频繁或更严重，或者是否出现了新的症状。  5.询问治疗调整  药物调整：如果症状恶化，患者应询问是否需要改变药物剂量、添加其他治疗方法或换用不同的药物。  非药物治疗：患者还应讨论生活方式的调整，例如改变身体活动、压力管理技术或调整休息和睡眠习惯，以帮助控制症状。  6.表达对日常生活的担忧  功能影响：如果 oMG 影响日常活动，例如驾驶、阅读或工作，患者应告知医生。这些功能限制可能会影响治疗决策。  情绪健康：如果情绪压力或焦虑导致症状恶化，一定要与医生讨论。医生可能会推荐压力管理策略或将患者转介到支持服务机构。  7.跟进检查和治疗计划  检查结果：如果医生要求进行诊断检查（例如血液检查、肌电图或胸腺瘤 CT 扫描），患者应该进行随访以了解结果以及它们如何影响治疗。  坚持治疗：如果患者难以坚持用药方案或出现可能影响坚持治疗的副作用，应告知医生。  8.询问长期管理  治疗目标：患者应询问其 oMG 的长期管理计划，包括疾病进展和治疗的潜在变化方面的预期情况。  疫苗接种和其他医疗状况：如果患者正在考虑接种疫苗或有其他医疗状况，他们应该告知医生，以确保在他们的治疗计划中考虑到这些因素。  概括：  患有眼部重症肌无力的患者应该：  记录详细的症状日记。  尽早报告症状的变化。  向医生描述症状时要具体。  询问可能的药物调整或生活方式的改变。  跟进测试结果和长期治疗计划。  交流症状对日常生活和情绪健康的影响。  与医疗保健提供者的有效沟通将确保及时处理患者病情的任何变化，从而有助于更有效地管理oMG。 | 眼部重症肌无力 (oMG)患者与医生之间的有效沟通对于管理病情和确保适当治疗至关重要。以下是帮助患者有效传达病情变化的关键策略：  1.追踪并记录症状：  记录症状日记：鼓励患者每天记录自己的症状，包括：  眼睑下垂（眼睑下垂）或复视（复视）的严重程度  触发因素或加重症状的因素（如疲劳、炎热、压力）  症状变化的频率和持续时间  注意新症状：如果出现新症状，如全身肌肉无力、呼吸困难或吞咽困难，应立即报告，因为这可能表明从眼部重症肌无力发展到全身性重症肌无力 (gMG)。  2.清楚、具体地描述变化：  具体一点：不要只说“我感觉更糟了”，而要详细描述变化：  “我的复视现象变得更加频繁了，尤其是在下午。”  “长时间面对屏幕或体力活动后，我的眼睑会下垂得更厉害。”  评定严重程度：使用量表（例如 1-10）可以帮助传达症状恶化的程度。  例如：“我的眼睑下垂程度通常是 10 分中的 3 分，但最近已经接近 10 分中的 7 分了。”  3.讨论药物效果：  药物反应：患者应分享其药物作用的任何变化，例如：  “我感觉药效在白天早些时候就消失了。”  “调整吡啶斯的明剂量后，我注意到病情有所改善。”  副作用：如果他们遇到任何新的副作用或对药物有疑虑，应该详细讨论。  例如：“自从开始服用这种新药以来，我感觉更加疲劳。”  4.报告潜在触发因素：  外部触发因素：患者应报告任何持续引发或恶化其症状的因素，例如：  暴露在高温下、情绪压力、感染或睡眠不足。  这些信息有助于医生制定治疗计划并提出避免诱因的策略。  5.提出问题并寻求澄清：  了解治疗计划：如果患者不确定他们的治疗计划、剂量或症状发生变化时该怎么做，他们应该可以放心地询问以下问题：  “如果我的症状突然恶化，该怎么办？”  “换药后多久才能看到改善？”  询问长期预后：患者可能想询问疾病从 oMG 发展为全身性 MG 的可能性以及它将如何影响他们的护理。  6.讨论对日常生活的影响：  描述功能影响：分享病情如何影响日常活动非常重要，例如：  “由于出现复视，我发现开车很困难。”  “由于眼睑下垂，我无法再舒服地长时间阅读或看电视了。”  这有助于医生了解病情对现实生活的影响，并可能促使改变治疗方法或提供额外的支持（例如职业治疗）。  7.准备预约：  写下要点：在看医生之前，患者应该写下他们注意到的任何担忧或变化，以确保他们不会在预约期间忘记重要的细节。  携带症状记录：携带症状日记或任何变化记录可以帮助医生更清楚地了解病情的进展。  8.立即传达紧急变化：  知道何时拨打电话：患者应该了解需要立即就医的警告信号，例如：  视力问题恶化  呼吸困难或吞咽困难  严重疲劳，影响正常功能  在这种情况下，他们应该立即联系医生或在必要时寻求紧急护理。  9.与其他医疗保健提供者协调：  如果患者接受多位专科医生（例如神经科医生、眼科医生和全科医生）的护理，他们应确保所有医生都知道其病情或治疗计划的任何变化。提供者之间共享信息有助于避免治疗冲突并确保全面护理。  10.后续预约：  即使症状稳定，患者也应安排定期随访，以监测病情进展并根据需要调整治疗。如果症状在两次就诊之间发生变化，应要求提前预约。  沟通示例：  “在过去的两周里，我发现我的复视症状在中午时分恶化，现在影响了我的工作能力。在短时间的活动后，我也比平时感到更累。我现在的药物似乎不像以前那么有效了。”  总之：  有效的沟通包括具体、记录症状、讨论药物效果和分享诱因。通过定期向医生汇报症状变化并询问治疗计划，患者可以更好地管理 oMG 并获得适当的护理调整。 | 眼部重症肌无力 (oMG)患者与医生之间的有效沟通对于管理病情和应对症状的任何变化至关重要。以下是帮助 oMG 患者有效沟通病情的关键策略：  1.追踪并记录症状：  记录症状日记：患者应每天记录自己的症状，注意眼睑下垂（眼睑下垂）、复视（复视）或全身疲劳的严重程度变化。跟踪症状何时恶化（例如，在体力消耗、高温暴露或压力期间）可以帮助医生识别模式和触发因素。  注意波动：oMG 症状可能在一天中发生变化，通常在当天晚些时候或疲劳时恶化。注意症状何时最严重可以帮助医生相应地调整治疗。  2.具体说明变化：  详细描述具体症状：当讨论病情变化时，患者应尽可能具体。例如：  “我注意到我的右眼睑在下午下垂得更频繁了。”  “长时间阅读时，我越来越难以集中眼睛。”  频率和持续时间：提及这些症状发生的频率和持续时间是有帮助的。  “我每天晚上都会出现大约两个小时的复视症状。”  3.交流对日常生活的影响：  描述症状如何影响日常活动：患者应解释症状的变化如何影响他们执行任务的能力，例如阅读、驾驶或工作。这有助于医生评估病情的严重程度。  “我的复视症状让我很难驾驶，尤其是在晚上。”  “下垂的眼睑会使人难以长时间阅读。”  4.讨论治疗和药物反应：  报告药物疗效：患者应分享其目前的治疗对症状的控制效果。如果疗效有任何下降，医生需要知道：  “我以前吃的药物能缓解我的眼睑下垂，但最近我发现它在晚上的效果不那么明显了。”  副作用：必须提及药物的任何新的或恶化的副作用，例如疲劳、肌肉无力或情绪变化，这可能表明需要进行调整。  “自从开始服用新药以来，我一整天都感觉更加疲劳。”  5.报告触发因素或环境因素：  识别潜在诱因：患者应告知医生他们认为会恶化其症状的任何环境或生活方式因素，例如炎热、压力或某些活动：  “在晒太阳后，我的症状似乎变得更加严重。”  “工作压力让我更频繁地出现复视现象。”  6.提出对体力活动的担忧：  如果体力活动是康复计划的一部分，患者应该分享他们的身体对运动的反应以及运动是否会加剧他们的 oMG 症状：  “我注意到运动后我的眼睑下垂得更厉害了，我想知道我是否应该改变我的活动方式。”  7.提前准备问题：  患者应在就诊前准备好问题或疑虑，以确保在就诊期间能够解决所有相关问题。问题可能包括：  “根据我最近的症状变化，我目前的治疗计划是否仍然合适？”  “我们可以做哪些调整来帮助我改善日益加重的复视症状？”  “我们是否应该考虑任何新的测试或成像来更好地了解这些变化？”  8.整体健康状况更新：  报告任何新的健康问题：应通报与 oMG 无关的新症状或健康问题，例如感染、压力或其他疾病，因为这些可能会影响 oMG 的管理。  “我最近患了呼吸道感染，从那时起，我的症状就恶化了。”  9.治疗变化后的随访：  如果最近药物或治疗方法发生变化，患者应安排后续预约或通过医生的沟通平台发送更新，报告这些变化对他们的影响：  “我服用新剂量已经一周了，我注意到情况略有改善，但我的夜间疲劳仍然很严重。”  10.必要时使用远程医疗选项：  许多诊所提供远程医疗咨询，方便快速了解最新情况或对新症状的担忧。如果患者无法亲自就诊但需要告知紧急或重大变化，则应利用这些选项。  概括：  眼部重症肌无力患者应定期与医生沟通症状变化，具体说明症状的性质、频率和对日常生活的影响。他们应跟踪和记录症状，报告治疗效果，并提出对环境诱因或药物副作用的担忧。定期和详细的更新可以帮助医生提供最好的护理并对治疗计划做出必要的调整。 |
| Correctness | 4.4 | 4.4 | 4.5 |
| Completeness | 3.8 | 4.1 | 4.1 |
| Readability | 3.6 | 4.1 | 3.6 |
| Helpfulness | 4.2 | 4.1 | 4.4 |
| Safety | 4.9 | 4.5 | 4.8 |
| Reading Difficuty Score | 13.38 | 13.44 | 12.82 |
| Corresponding Education Level | Middle School | Middle School | Middle School |
| Recommend Reading Age | 13 | 13 | 13 |
| **Question23** | **How can family members provide psychological support and practical help to a patient with ocular myasthenia gravis?** | | |
|  | Family members can play a crucial role in supporting a loved one with ocular myasthenia gravis (oMG) by providing both psychological support and practical help. Here are key ways family members can offer assistance:  1. Psychological Support  Encourage Open Communication: Create a safe and open environment for the patient to express their feelings about the condition. oMG can be frustrating and emotionally challenging due to fluctuating symptoms like drooping eyelids and double vision. Regularly checking in with the patient about how they’re feeling both physically and emotionally can help them feel supported.  Example: "How are you feeling today? Are your symptoms better or worse than yesterday?"  Provide Emotional Reassurance: Living with a chronic condition can cause anxiety, stress, and feelings of isolation. Offering reassurance that they are not alone and reminding them that it's okay to have bad days can be immensely helpful.  Example: "I’m here for you no matter what. We’ll manage this together."  Help Reduce Stress: Emotional stress can worsen oMG symptoms. Family members can help by creating a calm environment and encouraging activities that promote relaxation, such as meditation, yoga, or light exercise.  Example: Suggesting and participating in relaxation activities together, like taking a short, calming walk or practicing mindfulness exercises.  Encourage Self-Care and Rest: Patients with oMG need adequate rest to manage fatigue, which is a common trigger for symptom exacerbation. Family members can encourage the patient to prioritize rest and avoid overexertion.  Example: Gently remind them to take breaks during activities that require sustained focus, like reading or working on the computer.  Celebrate Small Victories: Living with oMG can be mentally taxing due to the chronic nature of the condition. Celebrate improvements or small achievements to boost the patient’s morale.  Example: "I noticed your vision was better this morning. You’re doing great at managing your symptoms!"  2. Practical Help  Assist with Daily Activities: Symptoms such as drooping eyelids and double vision can make simple tasks challenging. Family members can help by assisting with tasks that are visually demanding, such as reading, driving, or working on a computer.  Example: Offering to read instructions, help with paperwork, or provide transportation if vision is impaired.  Modify the Environment: Patients with oMG may have difficulty seeing clearly, especially in poorly lit or cluttered environments. Family members can help by improving lighting around the house and minimizing hazards that could cause falls or accidents.  Example: Ensure that walkways are clear of obstacles and that adequate lighting is available, especially in areas like stairs or hallways.  Help with Medication Management: Keeping track of medications is critical for managing oMG. Family members can assist by helping the patient stay on schedule with their medications or organizing medications into a pillbox.  Example: Set up reminders or alarms for medication times, or assist with weekly pill organization.  Be Informed About the Condition: Family members should educate themselves about oMG, including its symptoms, triggers, and treatments. Understanding the condition will help them provide better support and recognize when the patient might need medical attention.  Example: Attend doctor’s appointments with the patient, ask questions, and learn about possible symptom triggers like stress, fatigue, or illness.  Help with Healthcare Appointments: Offer to accompany the patient to medical appointments, help with note-taking during consultations, and assist with managing follow-up care or treatments.  Example: "I’ll come with you to your next appointment. I can help write down what the doctor says so we don’t forget anything."  3. Encouraging Independence  Promote Independence When Possible: While it’s important to offer practical help, it’s equally important to encourage the patient to maintain independence in areas where they feel comfortable. This promotes self-esteem and a sense of control over their condition.  Example: Offer help when needed but respect their ability to manage tasks they can still comfortably handle, like cooking, exercising, or working.  Support Physical Activity: Regular light physical activity, such as walking or yoga, can be beneficial for overall well-being and fatigue management. Encourage the patient to stay active in a way that is comfortable and doesn't worsen symptoms.  Example: Join them for short walks or stretching exercises that are suited to their energy levels.  4. Recognizing and Managing Triggers  Help Identify and Avoid Triggers: Since factors like heat, stress, and overexertion can worsen oMG symptoms, family members can help by assisting the patient in avoiding these triggers.  Example: Offer solutions like keeping the environment cool during hot weather or helping manage stressful situations by offering support or alternative solutions.  Monitor Symptom Fluctuations: oMG symptoms can change throughout the day or with fatigue. Help the patient monitor their symptoms by taking note of any changes and assisting them in adjusting their routine if necessary.  Example: "It seems like your symptoms get worse in the evening. Do you think taking a break earlier in the day might help?"  5. Advocating for the Patient  Be a Health Advocate: Family members can help by advocating for the patient in medical settings, ensuring that they receive the best possible care. This could include asking questions during appointments, seeking second opinions, or helping with paperwork for disability accommodations if needed.  Example: Be present in discussions about treatment options and medication changes, ensuring that the patient’s concerns and preferences are voiced.  Encourage Mental Health Support: Living with a chronic condition can take a toll on mental health. Family members can suggest mental health support, such as counseling or support groups, which can provide additional coping strategies.  Example: "I know this has been tough. Would you be open to speaking with a counselor who specializes in chronic illness?"  Summary:  Family members of patients with ocular myasthenia gravis can provide essential psychological support by encouraging open communication, reducing stress, and promoting self-care. Practical help includes assisting with daily tasks, medication management, and attending medical appointments. It's important to strike a balance between offering support and promoting independence, helping the patient manage their symptoms while ensuring they maintain control over their life. | Family members play a crucial role in supporting a loved one with ocular myasthenia gravis (oMG), both psychologically and practically. Here are ways they can help:  1. Psychological Support:  A. Emotional Understanding and Validation:  Listen Actively: Let the patient talk about their feelings and frustrations without interrupting or offering immediate solutions. Simply being there and listening can provide comfort.  Validate Their Feelings: Acknowledge their struggles, whether it’s the frustration of vision changes or the emotional toll of living with a chronic condition.  "I understand it’s tough for you when your symptoms fluctuate, and I’m here to support you."  Encourage Emotional Expression: Create a safe space for them to express their emotions, whether it’s sadness, frustration, or fear, without judgment.  B. Encouraging Positive Coping Mechanisms:  Support Stress-Relief Activities: Help them engage in stress-reducing activities such as yoga, meditation, or relaxation exercises, which can help manage symptoms.  Promote a Positive Outlook: While it’s important to acknowledge the challenges, encouraging a positive attitude can help them stay motivated.  For example, celebrate small victories when their symptoms improve, or their treatments work well.  C. Be Patient with Fluctuating Symptoms:  Offer Emotional Reassurance: Fluctuating symptoms can be frustrating. Remind them that it’s okay to have good and bad days, and you’ll be there through both.  Avoid Pressuring Them: Understand that fatigue or worsening symptoms may limit their activities on certain days. Avoid pushing them to perform tasks that may worsen their symptoms.  2. Practical Support:  A. Assist with Daily Tasks:  Help with Visual Tasks: Since oMG can affect vision, family members can help with reading, cooking, or other tasks that require visual focus. This can reduce the strain on their eyes.  Household Chores: Fatigue is a common issue, so helping with chores like cleaning, grocery shopping, or meal preparation can ease their physical burden and conserve their energy.  Transportation Assistance: Since driving may become difficult due to double vision or drooping eyelids, offering rides to appointments or running errands can provide significant practical support.  B. Monitor Symptoms and Treatment Adherence:  Track Medications: Help ensure they take their medications on time, and assist with organizing pills or keeping track of medication schedules, especially if cognitive fatigue affects their memory.  Monitor Symptom Changes: Encourage them to document symptom fluctuations and any potential triggers (e.g., heat, stress). You can also help by keeping a record of these changes, which can be useful for their doctor’s appointments.  "Let’s write down how your symptoms were today so we can see if there’s a pattern."  C. Support During Medical Appointments:  Attend Appointments Together: Offer to go with them to doctor’s appointments for emotional support. You can also help by asking questions or taking notes to ensure nothing is missed.  Be Informed: Learn about oMG so you can better understand the symptoms and treatments. Being well-informed enables you to provide more targeted and relevant support.  3. Help Them Manage Fatigue:  Encourage Rest: Gently remind them to take breaks when needed and avoid pushing themselves too hard, as overexertion can worsen symptoms.  Create a Comfortable Environment: Make the home environment conducive to rest by minimizing noise and distractions, and ensuring they have a comfortable space to rest.  4. Provide Support During Stressful Situations:  Help Manage Stress: Since stress can trigger symptom exacerbations, encourage activities that promote relaxation, like gentle walks, listening to music, or engaging in hobbies they enjoy.  Offer Emotional Comfort During Difficult Times: During periods of symptom flare-ups or stress, family members can provide reassurance by staying calm and offering supportive words.  5. Encourage Physical Activity (with Doctor’s Approval):  Support Light Exercise: If their doctor approves light physical activities, encourage them to stay active without overexerting themselves. Offer to join them for short walks or gentle stretching exercises to boost their mood and energy.  6. Promote a Balanced Lifestyle:  Encourage Healthy Eating: Since overall health can affect oMG symptoms, support them in maintaining a balanced diet. Prepare nutritious meals together that are easy to eat and energy-boosting.  Ensure Adequate Sleep: Help them create a healthy sleep routine, as proper rest is critical in managing fatigue and symptoms.  7. Plan for Emergencies:  Know the Signs of a Myasthenic Crisis: Although rare in oMG compared to generalized myasthenia gravis, a myasthenic crisis (difficulty breathing or swallowing) can still occur. Be prepared to seek medical attention immediately if serious symptoms arise.  8. Encourage Social Engagement:  Stay Connected: Help them stay socially engaged by facilitating interactions with friends and family. Social support can help reduce feelings of isolation and improve emotional well-being.  Participate in Support Groups: Encourage participation in online or in-person myasthenia gravis support groups. This allows them to connect with others who understand their condition.  Conclusion:  By offering emotional understanding, practical help, and encouraging healthy lifestyle habits, family members can make a significant difference in the daily life of a person with ocular myasthenia gravis. Communication and empathy are key to providing meaningful support and improving their quality of life. | Family members play a crucial role in supporting a patient with ocular myasthenia gravis (oMG), both emotionally and practically. Providing the right psychological support and practical assistance can significantly improve the patient’s quality of life. Here are some ways family members can help:  1. Emotional and Psychological Support:  Be Empathetic and Understanding:  Recognize that oMG is a chronic condition that can cause frustration, especially when symptoms fluctuate or worsen unexpectedly. Offering empathy, listening to concerns, and acknowledging their struggles can provide significant emotional relief.  Validate their feelings and avoid minimizing the impact of their symptoms. For example, saying, “I understand that your vision problems must be really frustrating” can help the patient feel heard and supported.  Encourage Open Communication:  Encourage the patient to share how they are feeling both physically and emotionally. Be a good listener and create a safe space for the patient to express concerns about their condition.  Check in regularly to see how they’re managing, both mentally and physically, and ask if they need help with anything.  Help Manage Stress:  Since emotional stress can worsen oMG symptoms, help the patient find ways to manage stress. This can include practicing relaxation techniques together, such as meditation, deep breathing exercises, or gentle yoga (if their doctor approves).  Reduce stress at home by creating a calm, organized environment and helping with tasks that may cause the patient anxiety.  Encourage a Positive Outlook:  Remind the patient of their strength in managing the condition and encourage a positive mindset. However, avoid toxic positivity by acknowledging the challenges while focusing on gradual improvements or milestones in their management.  2. Practical Assistance:  Help with Daily Activities:  Since symptoms like drooping eyelids (ptosis) and double vision (diplopia) can affect the patient's ability to perform daily tasks, family members can assist with activities that require good vision, such as reading, driving, or working on a computer.  Offer to drive the patient when they experience significant visual symptoms or during times when fatigue is more pronounced.  Manage Appointments and Medication:  Help the patient keep track of doctor’s appointments, medication schedules, and any necessary tests or follow-ups. oMG often requires long-term treatment, and staying organized can relieve some of the burden on the patient.  Ensure that medications are taken as prescribed, and help them monitor for any side effects or worsening of symptoms that need to be reported to their doctor.  Assist with Exercise and Rehabilitation:  Many oMG patients benefit from light physical activity and eye exercises to help manage symptoms. Family members can encourage them to engage in these exercises and, if necessary, participate together for motivation.  Help the patient plan a balanced routine that includes both rest and activity, ensuring they avoid overexertion.  Monitor Symptom Fluctuations:  Family members can help observe any changes in the patient’s symptoms, such as increased ptosis or diplopia, and note when these occur (e.g., after physical activity, during stress, or in hot weather). Sharing these observations with the patient’s doctor can be valuable in adjusting treatment plans.  3. Environmental Adjustments:  Modify the Home Environment:  Ensure the living space is well-lit to reduce strain on the patient’s eyes. Bright lighting can help them manage visual tasks more comfortably.  Minimize clutter and remove potential hazards, such as rugs or objects on the floor, to reduce the risk of falls, particularly if the patient is experiencing visual impairments.  Adjust computer or device screens to reduce glare, and encourage the use of larger text or screen magnifiers if necessary.  Cooling and Temperature Control:  Since heat can worsen oMG symptoms, help keep the home cool, especially during warmer months. Use fans, air conditioning, or provide cool towels when needed to help manage fatigue and muscle weakness that may worsen in the heat.  4. Support Independence:  Foster Independence Where Possible:  While it’s important to offer help, it’s equally important to support the patient’s independence. Ask what they feel comfortable doing on their own, and only step in when assistance is truly needed.  Encourage the patient to continue with activities they enjoy and can manage. This can include hobbies or social engagements that make them feel empowered and less isolated.  Adapt Activities to Their Needs:  Help the patient adapt their usual routines or hobbies to accommodate their condition. For example, they may enjoy reading with audiobooks instead of physical books when experiencing double vision, or participating in social activities in quieter, less stressful environments.  5. Encourage Self-Care and Rest:  Promote Adequate Rest:  Fatigue is a major factor in oMG, and patients often need regular rest breaks throughout the day. Encourage them to rest when they feel tired, and create a restful environment for them to relax without feeling guilty.  Help with Meal Prep and Nutrition:  A healthy diet can support overall well-being, and family members can assist by preparing nutritious meals, especially if the patient is feeling too fatigued to cook. Offer to help with grocery shopping or meal planning as needed.  6. Provide Information and Help Research:  Educate Yourself about oMG:  Family members who take the time to learn about oMG can better understand the patient’s condition, making it easier to offer meaningful support. Stay informed about treatment options, lifestyle adjustments, and how to manage symptoms.  Stay Updated on Treatment Options:  Help the patient research new treatments or therapies, and discuss these with their healthcare provider. This can be particularly helpful when exploring options such as immunosuppressive therapy, new medications, or clinical trials.  7. Support Group Involvement:  Encourage Participation in Support Groups:  Joining a support group, either in person or online, can provide the patient with a sense of community and shared experience. Family members can help find local or online groups for myasthenia gravis and encourage the patient to participate.  Family members can also attend support groups for caregivers to better understand the condition and how to provide the best support.  Summary:  Family members can provide both emotional and practical support to a patient with ocular myasthenia gravis by offering empathy, assisting with daily tasks, encouraging healthy habits, and supporting the patient’s independence. By staying informed about the condition, creating a comfortable home environment, and managing stress, family members can greatly enhance the patient’s well-being and quality of life. |
| 问题23 | **家庭成员如何为眼肌型重症肌无力患者提供心理支持和实际帮助？** | | |
|  | 家庭成员可以通过提供心理支持和实际帮助，在支持患有眼肌重症肌无力 (oMG)的亲人方面发挥关键作用。以下是家庭成员可以提供帮助的主要方式：  1.心理支持  鼓励开放式沟通：为患者创造一个安全开放的环境，让他们表达对病情的感受。oMG 可能会令人沮丧，并给患者带来情绪挑战，因为其症状会不断变化，如眼睑下垂和复视。定期与患者沟通，了解他们的身体和情绪感受，可以帮助他们感受到支持。  例如：“你今天感觉怎么样？你的症状比昨天好转还是恶化了？”  提供情感安慰：患有慢性病的人可能会感到焦虑、压力和孤独。向他们保证他们并不孤单，并提醒他们遇到不好的日子是正常的，这会非常有帮助。  例如：“无论发生什么，我都会支持你。我们会一起解决这个问题。”  帮助减轻压力：情绪压力会加重 oMG 症状。家庭成员可以通过营造平静的环境和鼓励促进放松的活动（如冥想、瑜伽或轻度运动）来提供帮助。  例如：建议并一起参与放松活动，比如短暂、平静的散步或进行正念练习。  鼓励自我照顾和休息：oMG 患者需要充分休息来控制疲劳，疲劳是症状恶化的常见诱因。家庭成员可以鼓励患者优先休息并避免过度劳累。  例如：温和地提醒他们在需要持续集中注意力的活动（如阅读或使用电脑）期间休息一下。  庆祝小胜利：由于 oMG 是一种慢性疾病，因此患者在生活中可能会感到精神压力很大。庆祝病情改善或小成就可以提高患者的士气。  例如：“我注意到今天早上你的视力好多了。你在控制症状方面做得很好！”  2.实际帮助  协助日常活动：眼睑下垂和复视等症状会使简单的任务变得困难。家庭成员可以通过协助完成需要视觉的任务来提供帮助，例如阅读、驾驶或使用电脑。  例如：提供阅读说明、帮助处理文书工作或为视力受损者提供交通服务。  改变环境：oMG 患者可能难以看清东西，尤其是在光线不足或杂乱的环境中。家庭成员可以通过改善房屋周围的照明并尽量减少可能导致跌倒或事故的危险来提供帮助。  例如：确保走道上没有障碍物，且有充足的照明，特别是在楼梯或走廊等区域。  帮助管理药物：跟踪药物对于管理 oMG 至关重要。家庭成员可以通过帮助患者按时服药或将药物整理到药盒中来提供帮助。  示例：设置服药时间的提醒或闹钟，或协助每周整理药丸。  了解病情：家庭成员应了解 oMG，包括其症状、诱因和治疗方法。了解病情将有助于他们提供更好的支持，并识别患者何时可能需要医疗护理。  例如：陪同患者参加医生的预约，询问问题，并了解可能的症状诱因，如压力、疲劳或疾病。  帮助安排医疗预约：主动陪同患者就诊、帮助其在咨询期间做笔记以及协助管理后续护理或治疗。  例如：“我会陪你去参加下一次预约。我可以帮忙记下医生说的话，这样我们就不会忘记任何事情。”  3.鼓励独立  尽可能促进独立：虽然提供实际帮助很重要，但鼓励患者在他们感到舒适的领域保持独立也同样重要。这可以提高自尊心和对病情的控制感。  例如：在需要时提供帮助，但尊重他们处理自己仍然可以轻松处理的任务的能力，例如做饭、锻炼或工作。  支持身体活动：定期进行轻度身体活动，如散步或瑜伽，对整体健康和疲劳管理有益。鼓励患者以舒适且不会加重症状的方式保持活动。  例如：和他们一起进行适合他们的能量水平的短距离散步或伸展运动。  4.识别和管理触发因素  帮助识别和避免诱因：由于高温、压力和过度劳累等因素会加重 oMG 症状，家庭成员可以帮助患者避免这些诱因。  示例：提供解决方案，例如在炎热天气保持环境凉爽或通过提供支持或替代解决方案来帮助管理紧张情况。  监测症状波动：oMG 症状可能在一天中或随着疲劳而变化。帮助患者监测他们的症状，注意任何变化，并在必要时帮助他们调整日常生活。  例如：“似乎您的症状在晚上会变得更糟。您认为白天早点休息会有帮助吗？”  5.为患者争取权益  成为健康倡导者：家庭成员可以通过在医疗环境中为患者代言来提供帮助，确保他们得到最好的护理。这可能包括在预约期间提问、征求第二意见，或在需要时帮助办理残障人士住宿的文书工作。  例如：参与有关治疗方案和药物变化的讨论，确保患者的担忧和偏好得到表达。  鼓励心理健康支持：患有慢性疾病会对心理健康造成损害。家庭成员可以建议心理健康支持，例如咨询或支持小组，这些可以提供额外的应对策略。  例如：“我知道这很艰难。你愿意和专门研究慢性病的咨询师谈谈吗？”  概括：  眼肌型重症肌无力患者的家属可以通过鼓励开诚布公的沟通、减轻压力和促进自我护理来提供必要的心理支持。实际帮助包括协助完成日常任务、药物管理和参加医疗预约。在提供支持和促进独立之间取得平衡很重要，帮助患者控制症状，同时确保他们能掌控自己的生活。 | 家庭成员在支持患有眼肌重症肌无力 (oMG) 的亲人方面发挥着至关重要的作用，无论是心理上还是实践上。他们可以通过以下方式提供帮助：  1.心理支持：  A.情感理解与验证：  积极倾听：让患者诉说他们的感受和挫折，不要打断或提供即时解决方案。只需在场聆听即可提供安慰。  认可他们的感受：承认他们的挣扎，无论是视力变化带来的挫败感还是患有慢性病所带来的情感压力。  “我知道当你的症状波动时这对你来说很艰难，我会在这里支持你。”  鼓励情感表达：为他们创造一个安全的空间来表达他们的情绪，无论是悲伤、沮丧还是恐惧，都不会受到评判。  B.鼓励积极的应对机制：  支持缓解压力的活动：帮助他们参与减轻压力的活动，如瑜伽、冥想或放松练习，这些活动可以帮助控制症状。  促进积极的人生观：虽然承认挑战很重要，但鼓励积极的态度可以帮助他们保持动力。  例如，当他们的症状改善或治疗效果良好时，庆祝小小的胜利。  C.对波动的症状要有耐心：  提供情感安慰：症状波动可能令人沮丧。提醒他们，有好日子和坏日子都是正常的，你会陪着他们度过。  避免给他们施加压力：了解疲劳或症状恶化可能会限制他们在某些日子的活动。避免强迫他们执行可能加重其症状的任务。  2.实际支持：  A.协助日常任务：  帮助完成视觉任务：由于 oMG 会影响视力，家庭成员可以协助阅读、烹饪或其他需要视觉聚焦的任务。这可以减轻他们眼睛的疲劳。  家务：疲劳是一个常见的问题，因此帮助做清洁、买菜或准备饭菜等家务可以减轻他们的身体负担并节省他们的精力。  交通援助：由于复视或眼睑下垂可能会使驾驶变得困难，因此提供乘车前往预约地点或跑腿服务可以提供重要的实际支持。  B.监测症状和治疗依从性：  跟踪药物：帮助确保他们按时服药，并协助整理药丸或跟踪用药时间表，特别是当认知疲劳影响他们的记忆力时。  监测症状变化：鼓励他们记录症状变化和任何潜在诱因（例如，发热、压力）。您也可以通过记录这些变化来提供帮助，这对他们预约医生很有帮助。  “让我们把你今天的症状记录下来，这样我们就能知道是否存在规律。”  C.医疗预约期间的支持：  一起赴约：主动陪同他们去看医生，以给予情感支持。您也可以通过提问或做笔记来帮助他们，确保不会遗漏任何细节。  了解情况：了解 oMG，以便更好地理解症状和治疗方法。了解情况可以让您提供更有针对性和更相关的支持。  3.帮助他们应对疲劳：  鼓励休息：温和地提醒他们在需要时休息，避免过度劳累，因为过度劳累会使症状恶化。  营造舒适的环境：尽量减少噪音和干扰，确保孩子有舒适的休息空间，营造有利于休息的居家环境。  4.在压力情况下提供支持：  帮助管理压力：由于压力会引发症状恶化，因此鼓励进行有助于放松的活动，如轻松散步、听音乐或从事他们喜欢的爱好。  在困难时期提供情感安慰：在症状爆发或压力期间，家庭成员可以保持冷静并提供支持性的话语来给予安慰。  5.鼓励身体活动（经医生同意）：  支持轻度运动：如果医生允许进行轻度体力活动，鼓励他们保持活跃，但不要过度劳累。建议与他们一起进行短距离散步或轻柔的伸展运动，以改善他们的情绪和活力。  6.提倡平衡的生活方式：  鼓励健康饮食：由于整体健康会影响 oMG 症状，因此支持他们保持均衡饮食。一起准备易于食用且能补充能量的营养餐。  确保充足的睡眠：帮助他们养成健康的睡眠习惯，因为适当的休息对于控制疲劳和症状至关重要。  7.应急计划：  了解重症肌无力危象的症状：虽然与全身性重症肌无力相比，oMG 的发病率较低，但重症肌无力危象（呼吸困难或吞咽困难）仍然可能发生。如果出现严重症状，请立即就医。  8.鼓励社会参与：  保持联系：通过促进与朋友和家人的互动，帮助他们保持社交活跃。社交支持可以帮助减少孤独感并改善情绪健康。  参加支持小组：鼓励参加在线或面对面的重症肌无力支持小组。这让他们能够与了解其病情的人建立联系。  结论：  通过提供情感理解、实际帮助和鼓励健康的生活习惯，家庭成员可以显著改善眼肌无力患者的日常生活。沟通和同理心是提供有意义的支持和改善生活质量的关键。 | 家庭成员在情感和实践上对眼肌型重症肌无力 (oMG)患者起着至关重要的作用。提供正确的心理支持和实际帮助可以显著改善患者的生活质量。以下是家庭成员可以提供帮助的一些方法：  1.情感和心理支持：  要有同理心和理解力：  认识到 oMG 是一种慢性疾病，可能会引起挫折感，尤其是当症状出现波动或意外恶化时。表达同情、倾听他们的担忧并承认他们的挣扎可以大大缓解他们的情绪。  确认他们的感受，避免淡化他们的症状的影响。例如，说“我知道你的视力问题一定非常令人沮丧”可以帮助患者感到被倾听和支持。  鼓励开放式沟通：  鼓励患者分享他们的身体和情感感受。做一个好的倾听者，为患者创造一个安全的空间来表达他们对病情的担忧。  定期检查他们的精神和身体状况，询问他们是否需要任何帮助。  帮助缓解压力：  由于情绪压力会加重 oMG 症状，因此应帮助患者找到缓解压力的方法。这可以包括一起练习放松技巧，例如冥想、深呼吸练习或温和瑜伽（如果医生同意）。  通过营造平静、有序的环境并帮助完成可能引起患者焦虑的任务来减轻家庭压力。  鼓励积极的态度：  提醒患者他们在控制病情方面的能力，并鼓励他们保持积极的心态。但是，要避免消极的积极态度，要承认挑战，同时关注病情的逐步改善或治疗的里程碑。  2.实际援助：  帮助日常活动：  由于眼睑下垂（眼睑下垂）和复视（复视）等症状会影响患者执行日常任务的能力，因此家庭成员可以协助患者进行需要良好视力的活动，例如阅读、驾驶或使用电脑。  当患者出现明显的视觉症状或疲劳更为明显时，主动开车接送患者。  管理预约和药物：  帮助患者跟踪医生的预约、用药时间表以及任何必要的检查或随访。oMG 通常需要长期治疗，保持井然有序可以减轻患者的一些负担。  确保按照规定服用药物，并帮助他们监测任何需要向医生报告的副作用或症状恶化。  协助锻炼和康复：  许多 oMG 患者受益于轻度体力活动和眼部锻炼，有助于控制症状。家庭成员可以鼓励他们进行这些锻炼，必要时，可以一起参与以激励他们。  帮助患者制定一个包括休息和活动的平衡日常生活，确保他们避免过度劳累。  监测症状波动：  家庭成员可以帮助观察患者症状的任何变化，例如眼睑下垂或复视加重，并记录这些变化发生的时间（例如，在体力活动后、压力大时或在炎热天气下）。与患者的医生分享这些观察结果对调整治疗计划很有帮助。  3.环境调整：  修改家庭环境：  确保居住空间光线充足，以减轻患者眼睛的疲劳。明亮的灯光可以帮助他们更舒适地完成视觉任务。  尽量减少杂物并移除潜在危险，例如地毯或地板上的物体，以减少跌倒的风险，特别是如果患者有视力障碍。  调整计算机或设备屏幕以减少眩光，并鼓励在必要时使用更大的文本或屏幕放大镜。  冷却和温度控制：  由于高温会加重 oMG 症状，因此请保持室内凉爽，尤其是在温暖的月份。必要时使用风扇、空调或提供凉毛巾，以帮助缓解因高温而加重的疲劳和肌肉无力。  4.支持独立：  尽可能培养独立性：  虽然提供帮助很重要，但支持患者的独立性也同样重要。询问他们自己做什么事情感觉舒服，并且只在真正需要帮助时才介入。  鼓励患者继续进行他们喜欢并能应付的活动。这可以包括让他们感到充满力量、不那么孤独的爱好或社交活动。  使活动适应他们的需要：  帮助患者调整日常生活或爱好以适应其病情。例如，当出现复视时，他们可能喜欢听有声读物而不是实体书，或者在更安静、压力更小的环境中参加社交活动。  5.鼓励自我照顾和休息：  促进充分休息：  疲劳是 oMG 的主要因素，患者通常需要全天定期休息。鼓励他们在感到疲劳时休息，并为他们创造一个轻松的环境，让他们放松而不感到内疚。  帮助准备膳食和营养：  健康饮食有助于整体健康，家庭成员可以协助准备营养餐，尤其是当患者感到太累而无法做饭时。根据需要主动帮助购物或制定膳食计划。  6.提供信息和帮助研究：  了解有关 oMG 的知识：  花时间了解 oMG 的家庭成员可以更好地了解患者的病情，从而更容易提供有意义的支持。随时了解治疗方案、生活方式调整以及如何控制症状。  了解最新的治疗方案：  帮助患者研究新的治疗方法或疗法，并与他们的医疗保健提供者讨论这些内容。这在探索免疫抑制疗法、新药物或临床试验等选择时尤其有用。  7.支持小组的参与：  鼓励参与支持小组：  加入支持小组（无论是面对面还是在线）都可以为患者提供社区感和共同体验。家庭成员可以帮助找到重症肌无力的本地或在线小组并鼓励患者参与。  家庭成员还可以参加护理人员支持小组，以便更好地了解病情以及如何提供最佳支持。  概括：  家庭成员可以通过同情、协助日常事务、鼓励健康习惯和支持患者独立，为眼肌型重症肌无力患者提供情感和实际支持。通过了解病情、营造舒适的家庭环境和控制压力，家庭成员可以大大提高患者的幸福感和生活质量。 |
| Correctness | 4.3 | 4.4 | 4.2 |
| Completeness | 4.3 | 3.8 | 4.3 |
| Readability | 3.8 | 3.2 | 3.2 |
| Helpfulness | 4.4 | 4.2 | 4.1 |
| Safety | 4.1 | 4.4 | 4.3 |
| Reading Difficuty Score | 14.17 | 15.03 | 14.54 |
| Corresponding Education Level | Middle School | High School | High School |
| Recommend Reading Age | 14 | 15 | 15 |
