# Supplementary material for: Evaluating the Effectiveness of Large Language Models in Providing Patient Education for Chinese Patients With Ocular Myasthenia Gravis: Mixed Methods Study
Source: J Med Internet Res. 2025 Apr 10;27:e67883. doi: 10.2196/67883 (PMC12022522; doi:10.2196/67883)
Supplement: Multimedia Appendix 3 [file jmir_v27i1e67883_app3.docx]

| **Table S3: Gemini answers to 23 questions, raw scores in 5 areas and raw data for objective readability analysis** | | | |
| --- | --- | --- | --- |
| **Question1** | **What is ocular myasthenia gravis (OMG)?** | | |
|  | Ocular myasthenia gravis (OMG) is a type of autoimmune disorder that primarily affects the muscles of the eyes. It is a specific subtype of myasthenia gravis (MG), a neuromuscular disease characterized by muscle weakness that worsens with activity and improves with rest.  In OMG, the body's immune system mistakenly attacks the acetylcholine receptors at the neuromuscular junction, which are responsible for transmitting nerve signals to the muscles. This leads to muscle weakness in the eyes, often resulting in:  Double vision (diplopia)  Drooping eyelids (ptosis)  Difficulty focusing  While OMG usually only affects the eye muscles, it can sometimes progress to involve other muscles in the body, such as those involved in swallowing or breathing. However, this is less common than in generalized MG.  If you suspect you may have OMG or any other eye condition, it's important to consult with an ophthalmologist for proper diagnosis and treatment. | Ocular Myasthenia Gravis (OMG) is a type of myasthenia gravis that primarily affects the muscles that control the movement of your eyes and eyelids. It's an autoimmune disorder, meaning your body's immune system mistakenly attacks its own healthy tissues.  Symptoms of OMG:  Double vision (diplopia)  Drooping eyelids (ptosis)  Difficulty focusing  Blurred vision  Sensitivity to light  Causes:  OMG occurs when the immune system produces antibodies that block the nerve signals to the muscles. This causes the muscles to become weak and easily fatigued.  Diagnosis:  Diagnosis is typically made through:  Physical examination: Checking for weakness in the eye muscles.  Blood tests: To detect specific antibodies.  Electromyography (EMG): To measure the electrical activity in the muscles.  Treatment:  There's no cure for OMG, but treatments can help manage symptoms. These may include:  Medications: To suppress the immune system or improve nerve-muscle communication.  Plasmapheresis: To remove harmful antibodies from the blood.  Thymectomy: Removal of the thymus gland, which plays a role in the immune system.  If you're experiencing any of the symptoms listed above, it's important to consult with a healthcare professional for a proper diagnosis and treatment plan.  Would you like to know more about a specific aspect of OMG, such as its impact on daily life, or the latest research in this area? | Ocular Myasthenia Gravis (OMG)  Ocular myasthenia gravis (OMG) is a type of myasthenia gravis that primarily affects the muscles around the eyes and eyelids. It is an autoimmune disorder, meaning your immune system mistakenly attacks and damages healthy tissues.  Symptoms of OMG include:  Double vision: Seeing two images of an object instead of one.  Drooping eyelids: Ptosis, where the upper eyelid sags.  Difficulty focusing: Trouble keeping your eyes in focus.  While OMG often begins with only eye muscle involvement, it can sometimes progress to a more generalized form of myasthenia gravis, affecting other muscles in the body.  If you experience any of these symptoms, it's important to consult with a healthcare professional for a diagnosis. Early detection and treatment can help manage the condition and improve quality of life. |
| 问题1 | **什么是眼部重症肌无力？** | | |
|  | 眼部重症肌无力 (OMG)是一种自身免疫性疾病，主要影响眼部肌肉。它是重症肌无力 (MG) 的一个特定亚型，重症肌无力是一种神经肌肉疾病，其特征是肌肉无力，活动时加重，休息时好转。  在 OMG 中，人体的免疫系统错误地攻击神经肌肉接头处的乙酰胆碱受体，该受体负责向肌肉传递神经信号。这会导致眼部肌肉无力，通常会导致：  复视  眼睑下垂（眼睑下垂）  难以集中注意力  虽然 OMG 通常只影响眼部肌肉，但有时也会发展到影响身体的其他肌肉，例如吞咽或呼吸肌肉。不过，这种情况比全身性 MG 少见。  如果您怀疑自己可能患有 OMG 或任何其他眼部疾病，请务必咨询眼科医生以获得正确的诊断和治疗。 | 眼部重症肌无力 (OMG)是一种重症肌无力，主要影响控制眼睛和眼睑运动的肌肉。这是一种自身免疫性疾病，意味着身体的免疫系统会错误地攻击自身的健康组织。  OMG 的症状：  复视  眼睑下垂（眼睑下垂）  难以集中注意力  视力模糊  对光敏感  原因：  当免疫系统产生抗体，阻断神经信号传送至肌肉时，就会发生肌肉萎缩性肌强直 (OMG)。这会导致肌肉变得虚弱，容易疲劳。  诊断：  诊断通常通过以下方式进行：  体格检查：检查眼部肌肉是否无力。  血液检查：检测特定抗体。  肌电图 (EMG)：测量肌肉中的电活动。  治疗：  OMG 无法治愈，但治疗可以帮助控制症状。这些可能包括：  药物：抑制免疫系统或改善神经肌肉交流。  血浆置换：从血液中去除有害抗体。  胸腺切除术：切除在免疫系统中发挥作用的胸腺。  如果您遇到上述任何症状，请务必咨询医疗保健专业人员，以获得正确的诊断和治疗方案。  您想了解有关 OMG 的某个特定方面的更多信息，例如它对日常生活的影响，或该领域的最新研究吗？ | 眼肌型重症肌无力（OMG） 是一种主要影响眼部肌肉的自身免疫性疾病。简单来说，就是你的身体免疫系统错误地攻击了控制眼部肌肉的神经连接，导致眼部肌肉无力。  OMG的主要症状包括：  复视: 看东西时出现重影。  眼睑下垂: 上眼皮无力下垂。  眼球运动困难: 难以聚焦或保持眼球固定。  虽然OMG通常只影响眼部肌肉，但有时也会发展成全身性的重症肌无力，影响身体的其他肌肉。  如果出现上述症状，建议及时就医，进行专业的诊断和治疗。  总结一下，OMG是一种眼部疾病，主要表现为眼部肌肉无力，影响视力。 |
| Correctness | 4.7 | 4.5 | 4.9 |
| Completeness | 3.6 | 4.1 | 4.1 |
| Readability | 3 | 3.4 | 2.9 |
| Helpfulness | 3.7 | 3.4 | 3.2 |
| Safety | 4.9 | 4.9 | 4.5 |
| Reading Difficuty Score | 10.45 | 9.94 | 10.00 |
| Corresponding Education Level | Primary school | Primary school | Primary school |
| Recommend Reading Age | 10 | 10 | 10 |
| **Question2** | **What are the typical symptoms of ocular myasthenia gravis?** | | |
|  | Ocular myasthenia gravis primarily affects the muscles that control your eyes and eyelids. Here are the typical symptoms:  Drooping eyelids (ptosis): This is often the first noticeable symptom.  Double vision (diplopia): This happens when your eyes can't align properly due to weak eye muscles.  Difficulty focusing: Your eyes may struggle to focus on objects.  These symptoms often:  Fluctuate: They may get better or worse throughout the day.  Worsen with use: Symptoms often become more noticeable after prolonged eye use.  If you experience any of these symptoms, it's important to see a healthcare professional. Early diagnosis and treatment can help manage the condition. | Ocular myasthenia gravis primarily affects the muscles that control the eyes and eyelids. Common symptoms include:  Drooping eyelids (ptosis): This is often the first symptom, causing one or both eyelids to sag.  Double vision (diplopia): This occurs when the eyes are misaligned, making it difficult to see clearly.  Difficulty focusing: The eyes may have trouble focusing on objects, especially at close range.  Eye pain or fatigue: Some people may experience discomfort or fatigue in their eyes.  Note: These symptoms can fluctuate throughout the day, often worsening with activity and improving with rest.  If you are experiencing these symptoms, it's important to consult a healthcare professional for a diagnosis. Early detection and treatment can help manage the condition and prevent complications. | Ocular myasthenia gravis primarily affects the muscles that control the eyes and eyelids. The most common symptoms include:  Double vision: Seeing two images of an object instead of one.  Drooping eyelids: Ptosis, a condition where one or both eyelids sag.  Difficulty focusing: The eyes may have trouble focusing on objects.  These symptoms often fluctuate throughout the day, becoming worse with activity and improving with rest. It's important to note that while ocular myasthenia gravis primarily affects the eyes, it can sometimes progress to a generalized form, involving other muscle groups.  If you're experiencing these symptoms, it's recommended to consult a healthcare professional for a proper diagnosis and treatment plan. |
| 问题2 | **眼肌型重症肌无力的典型症状是什么？** | | |
|  | 眼部重症肌无力主要影响控制眼睛和眼睑的肌肉。以下是典型症状：   - 眼睑下垂（眼睑下垂）：这通常是第一个明显的症状。 - 复视（复视）：由于眼部肌肉无力，双眼无法正确对准，就会发生这种情况。 - 聚焦困难：您的眼睛可能难以聚焦在物体上。   这些症状通常：   - 波动：一天内病情可能会变好，也可能会变坏。 - 使用时症状恶化：长时间用眼后症状通常会变得更加明显。   如果您出现任何这些症状，请务必去看医疗保健专业人员。早期诊断和治疗可以帮助控制病情。 | 眼部重症肌无力主要影响控制眼睛和眼睑的肌肉。常见症状包括：  眼睑下垂（眼睑下垂）：这通常是第一个症状，导致一只或两只眼睑下垂。  复视（复视）：当眼睛错位时就会发生这种情况，导致难以看清楚。  聚焦困难： 眼睛可能难以聚焦物体，尤其是在近距离的物体上。  眼睛疼痛或疲劳： 有些人可能会感到眼睛不适或疲劳。  注意： 这些症状可能全天波动，通常因活动而恶化，因休息而改善。  如果您出现这些症状，请务必咨询医疗保健专业人员进行诊断。 早期发现和治疗有助于控制病情并预防并发症 | 眼部重症肌无力主要影响控制眼睛和眼睑的肌肉。最常见的症状包括：  复视：看到一个物体的两个图像，而不是一个。  眼睑下垂：眼睑下垂，即一只或两只眼睑下垂的状况。  聚焦困难： 眼睛可能难以聚焦于物体。  这些症状通常全天波动，因活动而恶化，因休息而改善。 值得注意的是，虽然眼重症肌无力主要影响眼睛，但有时会发展为全身性症状，涉及其他肌肉群。  如果您出现这些症状，建议咨询医疗保健专业人员，以获得正确的诊断和治疗方案。 |
| Correctness | 4.4 | 4.7 | 4.3 |
| Completeness | 3.8 | 3.8 | 3.3 |
| Readability | 3.5 | 3.2 | 3.5 |
| Helpfulness | 3.6 | 3.6 | 3.9 |
| Safety | 3.6 | 3.6 | 3.9 |
| Reading Difficuty Score | 8.84 | 11.37 | 11.57 |
| Corresponding Education Level | Primary school | Primary school | Middle School |
| Recommend Reading Age | 9 | 11 | 12 |
| **Question3** | **What is the difference between ocular myasthenia gravis and generalized myasthenia gravis?** | | |
|  | As we've already established, both ocular and generalized myasthenia gravis are autoimmune disorders affecting the neuromuscular junction. The key difference lies in the muscles they primarily impact:  Ocular myasthenia gravis: Primarily affects the muscles controlling eye movement and eyelid position.  Generalized myasthenia gravis: Affects muscles throughout the body, including those in the arms, legs, and even respiratory system.  Beyond the Basics  While the distinction between the two is clear-cut in terms of the muscles involved, there are other nuances to consider:  Progression:  Ocular myasthenia gravis can sometimes progress to generalized myasthenia gravis over time.  The rate of progression varies widely from person to person.  Associated Conditions:  Both types can be associated with other autoimmune disorders like thyroid disease or lupus.  Treatment:  Treatment options are similar for both types and often involve immunosuppressants, cholinesterase inhibitors, and in some cases, thymectomy.  Quality of Life:  While both can significantly impact quality of life, the severity of symptoms and the muscles affected can vary greatly between individuals.  Visual Comparison  [Image: A table comparing ocular and generalized myasthenia gravis, highlighting the differences in muscles affected, common symptoms, and potential complications.]  Key Points to Remember  Early diagnosis is crucial for effective management and to prevent complications.  Symptoms can fluctuate over time, even with treatment.  Treatment is individualized based on the severity of symptoms and the patient's overall health.  Research is ongoing to better understand the causes and develop more effective treatments for myasthenia gravis. | Ocular vs. Generalized Myasthenia Gravis  Myasthenia gravis is an autoimmune disorder that results in muscle weakness. This occurs when the body's immune system mistakenly attacks the acetylcholine receptors that are crucial for muscle contractions.  The primary distinction between ocular and generalized myasthenia gravis lies in the specific muscles affected:  Ocular myasthenia gravis: This type primarily affects the muscles that control eye movement and eyelid position. Common symptoms include double vision, drooping eyelids, and difficulty focusing.  Generalized myasthenia gravis: This form impacts muscles throughout the entire body. Symptoms can manifest in various ways, such as weakness in the arms, legs, neck, and even the muscles involved in swallowing and breathing.  Key Differences Summarized:  Scope of Affected Muscles: Ocular myasthenia gravis is confined to the eye muscles, while generalized myasthenia gravis affects muscles throughout the body.  Severity of Symptoms: Ocular myasthenia gravis typically presents with milder symptoms primarily affecting vision, whereas generalized myasthenia gravis can lead to more severe, widespread weakness and potentially life-threatening complications.  It's important to note that:  While ocular myasthenia gravis often begins with eye-related symptoms, it can progress to a more generalized form over time.  Both types of myasthenia gravis can fluctuate in severity and may be accompanied by other symptoms like fatigue, difficulty swallowing, or slurred speech. | Myasthenia gravis is an autoimmune disorder that results in muscle weakness. This occurs when the body's immune system mistakenly attacks the acetylcholine receptors that are crucial for muscle contractions.  The primary distinction between ocular and generalized myasthenia gravis lies in the specific muscles affected:  Ocular myasthenia gravis: This type primarily affects the muscles that control eye movement and eyelid position. Common symptoms include double vision, drooping eyelids, and difficulty focusing.  Generalized myasthenia gravis: This form impacts muscles throughout the entire body. Symptoms can manifest in various ways, such as weakness in the arms, legs, neck, and even the muscles involved in swallowing and breathing.  Key Differences Summarized:  Scope of Affected Muscles: Ocular myasthenia gravis is confined to the eye muscles, while generalized myasthenia gravis affects muscles throughout the body.  Severity of Symptoms: Ocular myasthenia gravis typically presents with milder symptoms primarily affecting vision, whereas generalized myasthenia gravis can lead to more severe, widespread weakness and potentially life-threatening complications.  It's important to note that:  While ocular myasthenia gravis often begins with eye-related symptoms, it can progress to a more generalized form over time.  Both types of myasthenia gravis can fluctuate in severity and may be accompanied by other symptoms like fatigue, difficulty swallowing, or slurred speech. |
| 问题3 | **眼肌型重症肌无力和全身性重症肌无力有什么区别？** | | |
|  | 正如我们已经确定的，眼部重症肌无力和全身性重症肌无力都是影响神经肌肉接头的自身免疫性疾病。关键区别在于它们主要影响的肌肉：  眼部重症肌无力：主要影响控制眼球运动和眼睑位置的肌肉。  全身性重症肌无力：影响全身肌肉，包括手臂、腿部甚至呼吸系统的肌肉。  超越基础  虽然从所涉及的肌肉来看，两者的区别很明显，但还有其他细微差别需要考虑：  进展：  眼部重症肌无力有时会随着时间的推移发展为全身性重症肌无力。  每个人的进展速度存在很大差异。  相关条件：  这两种类型都可能与其他自身免疫性疾病有关，如甲状腺疾病或狼疮。  治疗：  两种类型的治疗方案相似，通常涉及免疫抑制剂、胆碱酯酶抑制剂，在某些情况下还包括胸腺切除术。  生活质量：  虽然两者都会严重影响生活质量，但症状的严重程度和受影响的肌肉在个体之间可能存在很大差异。  视觉比较  [图片：比较眼部重症肌无力和全身重症肌无力的表格，突出显示受影响肌肉、常见症状和潜在并发症的差异。]  要记住的要点  早期诊断对于有效治疗和预防并发症至关重要。  即使经过治疗，症状也会随着时间的推移而波动。  根据症状的严重程度和患者的整体健康状况采取个性化治疗。  目前正在进行研究，以更好地了解重症肌无力的病因并开发更有效的治疗方法。 | 重症肌无力是一种导致肌肉无力的自身免疫性疾病。当人体免疫系统错误地攻击对肌肉收缩至关重要的乙酰胆碱受体时，就会发生这种情况。  眼部重症肌无力和全身性重症肌无力的主要区别在于受影响的具体肌肉：  眼部重症肌无力：这种类型主要影响控制眼球运动和眼睑位置的肌肉。常见症状包括复视、眼睑下垂和聚焦困难。  全身性重症肌无力：这种类型会影响全身肌肉。症状表现多种多样，例如手臂、腿部、颈部甚至吞咽和呼吸肌肉无力。  主要差异总结：  受累肌肉范围：眼型重症肌无力仅局限于眼部肌肉，全身型重症肌无力则影响全身肌肉。  症状的严重程度：眼部重症肌无力通常表现为较轻的症状，主要影响视力，而全身性重症肌无力可能导致更严重、更广泛的虚弱和可能危及生命的并发症。  值得注意的是：  虽然眼部重症肌无力通常以眼睛相关症状开始，但随着时间的推移，它会发展为更普遍的形式。  两种类型的重症肌无力的严重程度都会有波动，并可能伴有疲劳、吞咽困难或言语不清等其他症状。 | 当人体免疫系统错误地攻击对肌肉收缩至关重要的乙酰胆碱受体时，就会发生这种情况。  眼部重症肌无力和全身性重症肌无力的主要区别在于受影响的具体肌肉：  眼部重症肌无力：这种类型主要影响控制眼球运动和眼睑位置的肌肉。常见症状包括复视、眼睑下垂和聚焦困难。  全身性重症肌无力：这种类型会影响全身肌肉。症状表现多种多样，例如手臂、腿部、颈部甚至吞咽和呼吸肌肉无力。  主要差异总结：  受累肌肉范围：眼型重症肌无力仅局限于眼部肌肉，全身型重症肌无力则影响全身肌肉。  症状的严重程度：眼部重症肌无力通常表现为较轻的症状，主要影响视力，而全身性重症肌无力可能导致更严重、更广泛的虚弱和可能危及生命的并发症。  值得注意的是：  虽然眼部重症肌无力通常以眼睛相关症状开始，但随着时间的推移，它会发展为更普遍的形式。  两种类型的重症肌无力的严重程度都会有波动，并可能伴有疲劳、吞咽困难或言语不清等其他症状。 |
| Correctness | 3.8 | 4.1 | 4 |
| Completeness | 3.8 | 3.8 | 3.4 |
| Readability | 4 | 4.1 | 3.6 |
| Helpfulness | 3.3 | 3.4 | 3.2 |
| Safety | 4.8 | 4.9 | 5 |
| Reading Difficuty Score | 12.66 | 11.27 | 11.28 |
| Corresponding Education Level | Middle School | Primary school | Primary school |
| Recommend Reading Age | 13 | 11 | 11 |
| **Question4** | **How is ocular myasthenia gravis diagnosed?** | | |
|  | Diagnosing Ocular Myasthenia Gravis  Diagnosing ocular myasthenia gravis typically involves a combination of clinical evaluation, physical examination, and specific tests. While there's no single definitive test, healthcare providers use a variety of methods to confirm the diagnosis.  Common diagnostic procedures include:  Medical history and physical examination: Doctors will inquire about your symptoms, such as double vision, drooping eyelids, and fatigue. They will also conduct a thorough physical examination to assess muscle strength and reflexes.  Ice pack test: Placing an ice pack on the affected eyelid can temporarily improve drooping in some cases of myasthenia gravis.  Tensilon test: This involves injecting a medication called edrophonium. If you have myasthenia gravis, your muscle strength may temporarily improve after the injection.  Blood tests: Blood tests can detect the presence of antibodies that attack the acetylcholine receptors, which are characteristic of myasthenia gravis.  Electromyography (EMG): This test measures the electrical activity of your muscles and can help determine if there is a problem with the nerve-muscle connection.  Repetitive nerve stimulation: This test involves stimulating a nerve with a small electrical impulse and measuring the muscle response. People with myasthenia gravis may have a decreased response to repeated stimulation.  It's important to note that:  The diagnosis of myasthenia gravis can be challenging, as symptoms can vary widely from person to person and may mimic other conditions.  A neurologist is typically the most qualified healthcare provider to diagnose and treat myasthenia gravis.  Key points to remember:  Early diagnosis is crucial for effective management and to prevent complications.  A combination of clinical evaluation and diagnostic tests is usually necessary to confirm the diagnosis.  While there's no cure for myasthenia gravis, there are treatments available to manage symptoms and improve quality of life. | Diagnosing Ocular Myasthenia Gravis  Diagnosing ocular myasthenia gravis typically involves a combination of clinical evaluation, physical examination, and specific tests. While there's no single definitive test, healthcare providers use a variety of methods to confirm the diagnosis.  Common diagnostic procedures include:  Medical history and physical examination: Doctors will inquire about your symptoms, such as double vision, drooping eyelids, and fatigue. They will also conduct a thorough physical examination to assess muscle strength and reflexes.  Ice pack test: Placing an ice pack on the affected eyelid can temporarily improve drooping in some cases of myasthenia gravis.  Tensilon test: This involves injecting a medication called edrophonium. If you have myasthenia gravis, your muscle strength may temporarily improve after the injection.  Blood tests: Blood tests can detect the presence of antibodies that attack the acetylcholine receptors, which are characteristic of myasthenia gravis.  Electromyography (EMG): This test measures the electrical activity of your muscles and can help determine if there is a problem with the nerve-muscle connection.  Repetitive nerve stimulation: This test involves stimulating a nerve with a small electrical impulse and measuring the muscle response. People with myasthenia gravis may have a decreased response to repeated stimulation.  It's important to note that:  The diagnosis of myasthenia gravis can be challenging, as symptoms can vary widely from person to person and may mimic other conditions.  A neurologist is typically the most qualified healthcare provider to diagnose and treat myasthenia gravis.  Key points to remember:  Early diagnosis is crucial for effective management and to prevent complications.  A combination of clinical evaluation and diagnostic tests is usually necessary to confirm the diagnosis.  While there's no cure for myasthenia gravis, there are treatments available to manage symptoms and improve quality of life. | Diagnosing Ocular Myasthenia Gravis  Diagnosing ocular myasthenia gravis typically involves a combination of clinical evaluation, physical examination, and specific tests. While there's no single definitive test, healthcare providers use a variety of methods to confirm the diagnosis.  Common diagnostic procedures include:  Medical history and physical examination: Doctors will inquire about your symptoms, such as double vision, drooping eyelids, and fatigue. They will also conduct a thorough physical examination to assess muscle strength and reflexes.  Ice pack test: Placing an ice pack on the affected eyelid can temporarily improve drooping in some cases of myasthenia gravis.  Tensilon test: This involves injecting a medication called edrophonium. If you have myasthenia gravis, your muscle strength may temporarily improve after the injection.  Blood tests: Blood tests can detect the presence of antibodies that attack the acetylcholine receptors, which are characteristic of myasthenia gravis.  Electromyography (EMG): This test measures the electrical activity of your muscles and can help determine if there is a problem with the nerve-muscle connection.  Repetitive nerve stimulation: This test involves stimulating a nerve with a small electrical impulse and measuring the muscle response. People with myasthenia gravis may have a decreased response to repeated stimulation.  Single fiber electromyography (SFEMG): This test is highly sensitive for ocular myasthenia and can provide a more definitive diagnosis.  It's important to note that:  The diagnosis of myasthenia gravis can be challenging, as symptoms can vary widely from person to person and may mimic other conditions.  A neurologist is typically the most qualified healthcare provider to diagnose and treat myasthenia gravis.  Key points to remember:  Early diagnosis is crucial for effective management and to prevent complications.  A combination of clinical evaluation and diagnostic tests is usually necessary to confirm the diagnosis.  While there's no cure for myasthenia gravis, there are treatments available to manage symptoms and improve quality of life. |
| 问题4 | **如何诊断眼肌型重症肌无力？** | | |
|  | 诊断眼部重症肌无力通常需要结合临床评估、身体检查和特定测试。虽然没有单一的确定性测试，但医疗保健提供者会使用多种方法来确认诊断。  常见的诊断程序包括：   - 病史和体格检查：医生会询问您的症状，例如复视、眼睑下垂和疲劳。他们还会进行全面的体格检查，以评估肌肉力量和反射能力。 - 冰袋测试：在某些重症肌无力病例中，将冰袋放在患眼睑上可以暂时改善眼睑下垂。 - Tensilon 测试：该测试涉及注射一种名为 Edrophonium 的药物。如果您患有重症肌无力，注射后您的肌肉力量可能会暂时改善。 - 血液检查：血液检查可以检测出攻击乙酰胆碱受体的抗体的存在，这是重症肌无力的特征。 - 肌电图 (EMG)：该测试测量您的肌肉的电活动，并有助于确定神经肌肉连接是否存在问题。 - 重复神经刺激：该测试涉及用小电脉冲刺激神经并测量肌肉反应。重症肌无力患者对重复刺激的反应可能会降低。   值得注意的是：   - 重症肌无力的诊断可能很困难，因为症状在人与人之间可能有很大差异，并且可能与其他疾病相似。 - 神经科医生通常是诊断和治疗重症肌无力最有资格的医疗保健提供者。   要记住的要点：   - 早期诊断对于有效治疗和预防并发症至关重要。 - 通常需要结合临床评估和诊断测试来确认诊断。 - 虽然重症肌无力无法治愈，但可以通过治疗来控制症状并改善生活质量。 | 诊断眼部重症肌无力通常需要结合临床评估、身体检查和特定测试。虽然没有单一的确定性测试，但医疗保健提供者会使用多种方法来确认诊断。  常见的诊断程序包括：   - 病史和体格检查：医生会询问您的症状，例如复视、眼睑下垂和疲劳。他们还会进行全面的体格检查，以评估肌肉力量和反射能力。 - 冰袋测试：在某些重症肌无力病例中，将冰袋放在患眼睑上可以暂时改善眼睑下垂。 - Tensilon 测试：该测试涉及注射一种名为 Edrophonium 的药物。如果您患有重症肌无力，注射后您的肌肉力量可能会暂时改善。 - 血液检查：血液检查可以检测出攻击乙酰胆碱受体的抗体的存在，这是重症肌无力的特征。 - 肌电图 (EMG)：该测试测量您的肌肉的电活动，并有助于确定神经肌肉连接是否存在问题。 - 重复神经刺激：该测试涉及用小电脉冲刺激神经并测量肌肉反应。重症肌无力患者对重复刺激的反应可能会降低。   值得注意的是：   - 重症肌无力的诊断可能很困难，因为症状在人与人之间可能有很大差异，并且可能与其他疾病相似。 - 神经科医生通常是诊断和治疗重症肌无力最有资格的医疗保健提供者。   要记住的要点：   - 早期诊断对于有效治疗和预防并发症至关重要。 - 通常需要结合临床评估和诊断测试来确认诊断。 - 虽然重症肌无力无法治愈，但可以通过治疗来控制症状并改善生活质量。 | 诊断眼部重症肌无力通常需要结合临床评估、身体检查和特定测试。虽然没有单一的确定性测试，但医疗保健提供者会使用多种方法来确认诊断。  常见的诊断程序包括：   - 病史和体格检查：医生会询问您的症状，例如复视、眼睑下垂和疲劳。他们还会进行全面的体格检查，以评估肌肉力量和反射能力。 - 冰袋测试：在某些重症肌无力病例中，将冰袋放在患眼睑上可以暂时改善眼睑下垂。 - Tensilon 测试：该测试涉及注射一种名为 Edrophonium 的药物。如果您患有重症肌无力，注射后您的肌肉力量可能会暂时改善。 - 血液检查：血液检查可以检测出攻击乙酰胆碱受体的抗体的存在，这是重症肌无力的特征。 - 肌电图 (EMG)：该测试测量您的肌肉的电活动，并有助于确定神经肌肉连接是否存在问题。 - 重复神经刺激：该测试涉及用小电脉冲刺激神经并测量肌肉反应。重症肌无力患者对重复刺激的反应可能会降低。 - 单纤维肌电图（SFEMG）：该测试对眼肌无力具有高度敏感性，可以提供更明确的诊断。   值得注意的是：   - 重症肌无力的诊断可能很困难，因为症状在人与人之间可能有很大差异，并且可能与其他疾病相似。 - 神经科医生通常是诊断和治疗重症肌无力最有资格的医疗保健提供者。   要记住的要点：   - 早期诊断对于有效治疗和预防并发症至关重要。 - 通常需要结合临床评估和诊断测试来确认诊断。 - 虽然重症肌无力无法治愈，但可以通过治疗来控制症状并改善生活质量。 |
| Correctness | 3.8 | 4 | 4 |
| Completeness | 3.2 | 3.2 | 3.7 |
| Readability | 3.2 | 3.1 | 3.5 |
| Helpfulness | 3.2 | 3.3 | 3.7 |
| Safety | 3.9 | 4 | 4.4 |
| Reading Difficuty Score | 14.04 | 14.04 | 14.36 |
| Corresponding Education Level | Middle School | Middle School | Middle School |
| Recommend Reading Age | 14 | 14 | 14 |
| **Question5** | **Do ocular myasthenia gravis patients need long-term medication?** | | |
|  | - Nature of the Disease: OMG is a chronic autoimmune disorder that causes fluctuating weakness of the eye muscles. While medications can effectively manage the symptoms, there's no known cure for the underlying autoimmune process. - Symptom Management: Medications help to improve muscle strength and reduce symptoms like drooping eyelids (ptosis) and double vision (diplopia). - Disease Progression: While OMG often remains confined to the eye muscles, it can sometimes progress to involve other muscle groups. Long-term medication helps to prevent or delay this progression.   Commonly prescribed medications for OMG include:   - Pyridostigmine: A cholinesterase inhibitor that improves neuromuscular transmission. - Immunosuppressants: Medications like corticosteroids or azathioprine, which help to suppress the overactive immune system. - Intravenous immunoglobulin (IVIG): A concentrated form of antibodies that can temporarily improve muscle strength.   It's important to note:   - Individualized Treatment: The specific medications and dosage will vary depending on the severity of the condition and the individual patient's response to treatment. - Regular Monitoring: Patients with OMG require regular follow-up appointments with their neurologist or ophthalmologist to monitor disease activity and adjust medication as needed. - Potential Side Effects: Medications used to treat OMG can have side effects, and it's important to discuss these with your healthcare provider.   In conclusion, long-term medication is a cornerstone of managing ocular myasthenia gravis. By working closely with your healthcare team, you can effectively control your symptoms and maintain a good quality of life. | - Nature of the disease: Myasthenia gravis is an autoimmune disorder, meaning the body's immune system mistakenly attacks its own healthy tissues. In OMG, the antibodies target the neuromuscular junction, interfering with the transmission of nerve impulses to the muscles. - Fluctuating symptoms: Symptoms of OMG, such as drooping eyelids (ptosis) and double vision (diplopia), can vary in severity over time. Medications help to stabilize these symptoms and prevent exacerbations. - No cure: While there's no cure for myasthenia gravis, medications can significantly improve quality of life for most patients. - Common medications used to treat OMG include: - Pyridostigmine: This is often the first-line treatment for OMG, as it helps to increase the amount of acetylcholine available at the neuromuscular junction. - Immunosuppressants: These drugs, such as prednisone or azathioprine, work by suppressing the immune system to reduce the production of antibodies. - Intravenous immunoglobulin (IVIg): IVIg is a concentrated form of antibodies that can help to temporarily suppress the immune system and improve muscle strength. - Plasmapheresis: This procedure involves removing blood from the body, removing harmful antibodies, and then returning the blood to the body. - It's important to note that: - Individualized treatment: The specific medications and dosages used to treat OMG will vary from person to person, depending on the severity of symptoms and individual response to treatment. - Regular monitoring: Patients with OMG require regular monitoring by a healthcare professional to adjust medication dosages as needed and to monitor for side effects. - Lifestyle modifications: In addition to medication, lifestyle modifications such as getting enough rest, avoiding stress, and eating a healthy diet can also help to manage symptoms. | - Absolutely! Patients with ocular myasthenia gravis (OMG) typically require long-term medication management. While the specific medications and dosages will vary depending on the individual patient and the severity of their symptoms, here's a general overview: - Yes, ocular myasthenia gravis patients often need long-term medication. - The goal of medication is to manage symptoms and improve quality of life. - Common medications include: - Pyridostigmine: This is often the first-line treatment for OMG. It helps to increase the amount of acetylcholine available at the neuromuscular junction. - Immunosuppressants: For more severe cases, immunosuppressants like corticosteroids or azathioprine may be used to suppress the immune system and reduce the production of antibodies that attack the neuromuscular junction. - Other medications: Depending on the specific needs of the patient, other medications such as plasmapheresis or intravenous immunoglobulin may be used. - It's important to note that: - Medication management is individualized. - Regular monitoring by a healthcare provider is essential. - Treatment plans may need to be adjusted over time. |
| 问题5 | **眼肌型重症肌无力患者需要长期药物治疗吗？** | | |
|  | 疾病性质：OMG 是一种慢性自身免疫性疾病，会导致眼部肌肉出现波动性无力。虽然药物可以有效控制症状，但目前尚无治愈潜在自身免疫过程的方法。  症状管理：药物有助于提高肌肉力量并减轻眼睑下垂（眼睑下垂）和复视（复视）等症状。  疾病进展：虽然 OMG 通常局限于眼部肌肉，但有时会进展到其他肌肉群。长期用药有助于预防或延缓这种进展。  治疗 OMG 的常用药物包括：  吡啶斯的明：一种改善神经肌肉传递的胆碱酯酶抑制剂。  免疫抑制剂：皮质类固醇或硫唑嘌呤等药物，有助于抑制过度活跃的免疫系统。  静脉注射免疫球蛋白（IVIG）：一种浓缩形式的抗体，可以暂时提高肌肉力量。  需要注意的是：  个性化治疗：具体药物和剂量将根据病情的严重程度和个体患者对治疗的反应而有所不同。  定期监测：OMG 患者需要定期与神经科医生或眼科医生进行随访预约，以监测病情活动并根据需要调整药物。  潜在副作用：用于治疗 OMG 的药物可能会有副作用，因此与您的医疗保健提供者讨论这些问题非常重要。  总之，长期服药是治疗眼部重症肌无力的基石。通过与医疗团队密切合作，您可以有效控制症状并保持良好的生活质量。 | 疾病性质：重症肌无力是一种自身免疫性疾病，即人体免疫系统错误地攻击自身健康组织。在重症肌无力中，抗体会攻击神经肌肉接头，干扰神经冲动向肌肉的传递。  症状波动：眼睑下垂（眼睑下垂）和复视（复视）等OMG 症状的严重程度会随时间而变化。药物有助于稳定这些症状并防止病情恶化。  无法治愈：虽然重症肌无力无法治愈，但药物可以显著改善大多数患者的生活质量。  治疗 OMG 的常用药物包括：  吡啶斯的明：这通常是 OMG 的一线治疗方法，因为它有助于增加神经肌肉接头处可用的乙酰胆碱的量。  免疫抑制剂：这些药物，如泼尼松或硫唑嘌呤，通过抑制免疫系统来减少抗体的产生。  静脉注射免疫球蛋白 (IVIg)：IVIg 是一种浓缩形式的抗体，可以帮助暂时抑制免疫系统并提高肌肉力量。  血浆置换：该过程包括从体内抽取血液，去除有害抗体，然后将血液输回体内。  值得注意的是：  个性化治疗：治疗 OMG 的具体药物和剂量因人而异，取决于症状的严重程度和个体对治疗的反应。  定期监测：OMG 患者需要医疗保健专业人员定期监测，以根据需要调整药物剂量并监测副作用。  改变生活方式：除了药物治疗外，充足的休息、避免压力、健康饮食等改变生活方式也有助于控制症状。 | 当然！眼部重症肌无力 (OMG) 患者通常需要长期药物治疗。虽然具体药物和剂量会因患者个体及其症状的严重程度而异，但以下是一般概述：  是的，眼肌型重症肌无力患者往往需要长期服药。  药物治疗的目的是控制症状和改善生活质量。  常见药物包括：  吡啶斯的明：这通常是 OMG 的一线治疗药物。它有助于增加神经肌肉接头处可用的乙酰胆碱量。  免疫抑制剂：对于更严重的病例，可以使用皮质类固醇或硫唑嘌呤等免疫抑制剂来抑制免疫系统并减少攻击神经肌肉接头的抗体的产生。  其他药物：根据患者的具体需要，可以使用其他药物，如血浆置换或静脉注射免疫球蛋白。  值得注意的是：  药物管理是个性化的。  医疗保健提供者的定期监测至关重要。  治疗计划可能需要随着时间的推移进行调整。 |
| Correctness | 4.4 | 4 | 4.1 |
| Completeness | 3.6 | 3.6 | 3.1 |
| Readability | 3 | 3.3 | 3.2 |
| Helpfulness | 4.3 | 4.3 | 4.1 |
| Safety | 4.6 | 4.4 | 4.4 |
| Reading Difficuty Score | 13.57 | 11.97 | 12.18 |
| Corresponding Education Level | Middle School | Middle School | Middle School |
| Recommend Reading Age | 14 | 12 | 12 |
| **Question6** | **What medications are commonly used to treat ocular myasthenia gravis? What are their side effects?** | | |
|  | Absolutely! Here's a comprehensive response to your query:  Common Medications for Ocular Myasthenia Gravis:  Patients with ocular myasthenia gravis (OMG) often require long-term medication to manage their symptoms. The specific medication and dosage will depend on the severity of the condition and the individual patient. Here are some commonly prescribed medications:  Pyridostigmine: This is often the first-line treatment for OMG. It works by increasing the amount of acetylcholine, a neurotransmitter that helps transmit signals between nerves and muscles.  Corticosteroids: These medications suppress the immune system, which can help reduce inflammation and muscle weakness. Examples include prednisone and methylprednisolone.  Immunosuppressants: Medications like azathioprine and mycophenolate mofetil can also be used to suppress the immune system and reduce the production of antibodies that attack the neuromuscular junction.  Plasmapheresis: This procedure involves removing blood from the body, removing harmful antibodies, and then returning the blood to the body.  Intravenous immunoglobulin (IVIG): This involves infusing healthy antibodies into the bloodstream to help block the harmful antibodies causing muscle weakness.  Common Side Effects:  It's important to note that every individual may experience side effects differently. Some common side effects associated with these medications include:  Pyridostigmine: Nausea, vomiting, diarrhea, abdominal cramps, muscle cramps, excessive sweating, and increased salivation.  Corticosteroids: Weight gain, mood swings, increased appetite, high blood pressure, increased risk of infections, and thinning of the bones (osteoporosis).  Immunosuppressants: Increased risk of infections, nausea, vomiting, hair loss, and liver damage.  Plasmapheresis: Low blood pressure, headache, muscle aches, and temporary numbness or tingling.  IVIG: Headache, fever, chills, nausea, and muscle aches.  It's crucial to remember:  Individualized Treatment: The best medication and dosage for each person will vary.  Regular Monitoring: Close monitoring by a healthcare provider is essential to manage side effects and adjust treatment as needed.  Long-Term Management: OMG often requires long-term medication management.  If you have any concerns about your medications or experience any new or worsening symptoms, please consult with your healthcare provider.  Disclaimer: This information is intended for general knowledge and informational purposes only, and does not constitute medical advice. Always seek the advice of your physician or other qualified healthcare provider with any questions you may have regarding a medical condition. | Medications for Ocular Myasthenia Gravis  Ocular myasthenia gravis is a condition characterized by weakness of the eye muscles. The treatment typically involves medications that help improve neuromuscular transmission. Here are some commonly used medications and their potential side effects:  Cholinesterase Inhibitors  These drugs prevent the breakdown of acetylcholine, a neurotransmitter that helps muscles contract.  Pyridostigmine: This is often the first-line treatment.  Side effects: Nausea, vomiting, abdominal cramps, diarrhea, increased salivation, and excessive sweating.  Neostigmine: Similar to pyridostigmine.  Side effects: Similar to pyridostigmine.  Immunosuppressants  These medications suppress the immune system to reduce the production of antibodies that attack the neuromuscular junction.  Prednisone: A corticosteroid that can reduce inflammation and suppress the immune system.  Side effects: Weight gain, moon face, high blood pressure, increased risk of infections, and mood changes.  Azathioprine: An antimetabolite that suppresses the immune system.  Side effects: Nausea, vomiting, diarrhea, increased risk of infections, and liver damage.  Mycophenolate mofetil: Another immunosuppressant.  Side effects: Nausea, vomiting, diarrhea, headache, and increased risk of infections.  Other Medications  Intravenous Immunoglobulin (IVIG): This is a concentrated form of antibodies that can help suppress the immune system.  Side effects: Headache, fever, chills, muscle aches, and nausea.  Plasmapheresis: This procedure removes harmful antibodies from the blood.  Side effects: Low blood pressure, muscle cramps, and dizziness.  Please note: This is not an exhaustive list of medications or potential side effects. The specific medications and dosages used will depend on the individual patient and the severity of their symptoms. It is important to discuss the potential risks and benefits of any medication with your doctor.  Disclaimer: I am an AI chatbot and cannot provide medical advice. Always consult with a healthcare professional for any health concerns. | Medications for Ocular Myasthenia Gravis  Ocular myasthenia gravis (OMG) is a chronic autoimmune disorder that primarily affects the muscles that control eye movement. The treatment often involves medications to help manage symptoms and improve quality of life.  Commonly Used Medications  Pyridostigmine: This is the first-line treatment for OMG. It works by increasing the levels of acetylcholine, a neurotransmitter that helps nerve impulses travel to the muscles.  Side effects: Nausea, vomiting, diarrhea, abdominal cramps, increased salivation, and muscle cramps.  Prednisone: This is a corticosteroid that helps to reduce inflammation. It can be used to manage severe symptoms or when pyridostigmine is not effective alone.  Side effects: Weight gain, increased appetite, mood swings, high blood pressure, and increased risk of infection.  Immunosuppressants: These drugs help to suppress the immune system and reduce the production of antibodies that attack the neuromuscular junction. Examples include:  Azathioprine: Can cause nausea, vomiting, hair loss, and increased risk of infection.  Cyclosporine: Can cause kidney problems, high blood pressure, and increased risk of infection.  Mycophenolate mofetil: Can cause nausea, vomiting, diarrhea, and increased risk of infection.  Other Treatments  Intravenous Immunoglobulin (IVIG): This therapy involves receiving a concentrated dose of healthy antibodies to help suppress the immune system.  Plasmapheresis: This procedure removes harmful antibodies from the blood.  Important Considerations  Individualized Treatment: The choice of medication and dosage will depend on the severity of symptoms, overall health, and response to treatment.  Regular Monitoring: Patients with OMG require regular follow-up appointments with their healthcare provider to monitor disease activity and adjust medication as needed.  Side Effects: All medications have potential side effects. It's important to discuss these with your healthcare provider and weigh the benefits against the risks.  Note: This information is intended for general knowledge and informational purposes only, and does not constitute medical advice. Always consult with a qualified healthcare professional for any health concerns or before making any decisions related to your health or treatment. |
| 问题6 | **治疗眼肌型重症肌无力常用的药物是什么？它们的副作用是什么？** | | |
|  | 眼部重症肌无力的常用药物：  眼部重症肌无力 (OMG) 患者通常需要长期服药来控制症状。具体药物和剂量取决于病情严重程度和患者个人情况。以下是一些常用处方药：  吡啶斯的明：这通常是 OMG 的一线治疗药物。它的作用是增加乙酰胆碱的含量，乙酰胆碱是一种神经递质，有助于在神经和肌肉之间传递信号。  皮质类固醇：这些药物可抑制免疫系统，有助于减轻炎症和肌肉无力。例如泼尼松和甲基泼尼松龙。  免疫抑制剂：硫唑嘌呤和霉酚酸酯等药物也可用于抑制免疫系统并减少攻击神经肌肉接头的抗体的产生。  血浆置换：该过程包括从体内抽取血液，去除有害抗体，然后将血液输回体内。  静脉注射免疫球蛋白 (IVIG)：这涉及将健康抗体注入血液以帮助阻断导致肌肉无力的有害抗体。  常见副作用：  值得注意的是，每个人经历的副作用可能不同。这些药物的一些常见副作用包括：  吡啶斯的明：恶心、呕吐、腹泻、腹部痉挛、肌肉痉挛、出汗过多、唾液分泌增多。  皮质类固醇：体重增加、情绪波动、食欲增加、高血压、感染风险增加以及骨质变薄（骨质疏松症）。  免疫抑制剂：增加感染、恶心、呕吐、脱发和肝损伤的风险。  血浆置换：低血压、头痛、肌肉疼痛和暂时麻木或刺痛。  IVIG：头痛、发烧、发冷、恶心和肌肉疼痛。  请务必记住：  个性化治疗：每个人的最佳药物和剂量会有所不同。  定期监测：医疗保健提供者的密切监测对于控制副作用和根据需要调整治疗至关重要。  长期管理： OMG 通常需要长期药物管理。  如果您对所服用的药物有任何疑虑，或者出现任何新的或恶化的症状，请咨询您的医疗保健提供者。  免责声明：本信息仅供一般知识和参考之用，不构成医疗建议。如果您对医疗状况有任何疑问，请务必咨询您的医生或其他合格的医疗保健提供者。 | 眼部重症肌无力是一种以眼部肌肉无力为特征的疾病。治疗通常涉及有助于改善神经肌肉传递的药物。以下是一些常用药物及其潜在副作用：  胆碱酯酶抑制剂  这些药物可以防止乙酰胆碱（一种帮助肌肉收缩的神经递质）的分解。  吡啶斯的明：这通常是一线治疗方法。  副作用：恶心、呕吐、腹部绞痛、腹泻、唾液分泌增多、出汗过多。  新斯的明：与吡斯的明类似。  副作用：与吡斯的明相似。  免疫抑制剂  这些药物抑制免疫系统，减少攻击神经肌肉接头的抗体的产生。  泼尼松：一种可以减轻炎症和抑制免疫系统的皮质类固醇。  副作用：体重增加、月亮脸、高血压、感染风险增加和情绪变化。  硫唑嘌呤：一种抑制免疫系统的抗代谢物。  副作用：恶心、呕吐、腹泻、感染风险增加以及肝损伤。  霉酚酸酯：另一种免疫抑制剂。  副作用：恶心、呕吐、腹泻、头痛、感染风险增加。  其他药物  静脉注射免疫球蛋白 (IVIG)：这是一种浓缩形式的抗体，可以帮助抑制免疫系统。  副作用：头痛、发烧、发冷、肌肉酸痛和恶心。  血浆置换：该过程可去除血液中的有害抗体。  副作用：低血压、肌肉痉挛、头晕。  请注意：这不是药物或潜在副作用的详尽列表。所用的具体药物和剂量取决于患者个人及其症状的严重程度。与医生讨论任何药物的潜在风险和益处非常重要。  免责声明：我是 AI 聊天机器人，无法提供医疗建议。如有任何健康问题，请务必咨询医疗保健专业人士。 | 眼部重症肌无力的药物治疗  眼部重症肌无力(OMG)是一种慢性自身免疫性疾病，主要影响控制眼球运动的肌肉。治疗通常包括药物治疗，以帮助控制症状和改善生活质量。  常用药物  吡哆斯的明:这是治疗OMG的一线药物。它的工作原理是增加乙酰胆碱的水平，乙酰胆碱是一种神经递质，可以帮助神经冲动传递到肌肉。  副作用:恶心，呕吐，腹泻，腹部痉挛，唾液分泌增加，肌肉痉挛。  强的松:这是一种皮质类固醇，有助于减少炎症。它可用于治疗严重症状或当吡哆斯的明单独无效时。  副作用:体重增加、食欲增加、情绪波动、高血压和感染风险增加。  免疫抑制剂:这些药物有助于抑制免疫系统，减少攻击神经肌肉连接处的抗体的产生。例子包括:  硫唑嘌呤:可引起恶心、呕吐、脱发和感染风险增加。  环孢素:可引起肾脏问题，高血压，并增加感染的风险。  霉酚酸酯:可引起恶心，呕吐，腹泻，并增加感染的风险。  其他的治疗方法  静脉注射免疫球蛋白(IVIG):这种疗法包括接受浓缩剂量的健康抗体，以帮助抑制免疫系统。  血浆置换:这一过程去除血液中的有害抗体。  重要的注意事项  个体化治疗:药物和剂量的选择取决于症状的严重程度、整体健康状况和对治疗的反应。  定期监测:OMG患者需要定期与他们的医疗保健提供者进行随访，以监测疾病活动并根据需要调整药物。  副作用:所有药物都有潜在的副作用。与您的医疗保健提供者讨论这些问题并权衡利弊是很重要的。  注:此信息仅用于一般知识和信息目的，不构成医疗建议。在做出与您的健康或治疗有关的任何决定之前，请务必咨询合格的医疗保健专业人员。 |
| Correctness | 4.1 | 4.4 | 4.4 |
| Completeness | 3.2 | 3.2 | 3.8 |
| Readability | 4.4 | 4 | 4.1 |
| Helpfulness | 4.5 | 4.3 | 4.6 |
| Safety | 4.2 | 4.3 | 4.7 |
| Reading Difficuty Score | 13.72 | 13.20 | 11.99 |
| Corresponding Education Level | High School | High School | High School |
| Recommend Reading Age | 14 | 13 | 12 |
| **Question7** | **Can ocular myasthenia gravis be treated with surgery?** | | |
|  | While surgery is not the primary treatment for ocular myasthenia gravis (OMG), it can be a helpful adjunct in certain cases.  Here's a breakdown of how surgery can be used in conjunction with medication:  Thymectomy: In some cases, the thymus gland may be involved in the production of antibodies that cause OMG. Removing the thymus (thymectomy) can help to reduce the production of these antibodies.  Eye muscle surgery: For patients with severe ptosis (drooping eyelid) or strabismus (crossed eyes) that significantly impacts vision and quality of life, eye muscle surgery may be considered to improve alignment and function.  Lid crutches: These are external devices that can be used to support a droopy eyelid, providing temporary relief.  It's important to note that:  Surgery is not a cure for OMG. It can help to improve symptoms, but it will not eliminate the underlying autoimmune disorder.  The decision to undergo surgery is made on an individual basis and depends on the severity of symptoms, the patient's overall health, and the potential risks and benefits of surgery.  Surgery is often combined with medical treatments, such as medications to suppress the immune system, to achieve the best possible outcomes.  Medical treatments, including:  Pyridostigmine: To increase the levels of acetylcholine.  Corticosteroids: To reduce inflammation.  Immunosuppressants: To suppress the immune system.  Intravenous immunoglobulin (IVIG): To provide a concentrated dose of healthy antibodies.  Plasmapheresis: To remove harmful antibodies from the blood.  are typically the first line of treatment for OMG. Surgery is often considered when medical treatments have not been effective or when the patient's quality of life is significantly impacted by their eye symptoms.  If you have OMG, it's important to discuss all treatment options with your healthcare provider to determine the most appropriate course of action for your individual situation. | Thymectomy: If the thymus gland is involved in producing the antibodies that cause OMG, removing it (thymectomy) can help reduce these antibodies.  Eye muscle surgery: For patients with severe ptosis (drooping eyelids) or strabismus (crossed eyes) that significantly affects vision and quality of life, eye muscle surgery can improve alignment and function.  Lid crutches: These external devices can support a droopy eyelid, providing temporary relief.  Important to note:  Surgery isn't a cure. It can improve symptoms but won't eliminate the underlying autoimmune disorder.  The decision is individualized. Factors like symptom severity, overall health, and potential risks/benefits are considered.  Surgery is often combined with medications for optimal results.  Medical treatments, such as:  Pyridostigmine: To increase acetylcholine levels.  Corticosteroids: To reduce inflammation.  Immunosuppressants: To suppress the immune system.  Intravenous immunoglobulin (IVIG): To provide healthy antibodies.  Plasmapheresis: To remove harmful antibodies.  are usually the first line of treatment. Surgery is often considered when medical treatments aren't effective or when quality of life is significantly impacted.  Always consult with your healthcare provider to discuss all treatment options and determine the best course of action for your specific situation. | Ocular myasthenia gravis can indeed be managed with various treatments, including surgery. However, the decision to proceed with surgery is a complex one, typically made on a case-by-case basis and in consultation with a neurologist or ophthalmologist.  Here are some key points to consider:  Surgery is often considered a last resort after other treatments, such as medications, have been tried without success.  The specific surgical procedure will depend on the severity and nature of the symptoms. Common surgical options for OMG include:  Thymectomy: This involves the removal of the thymus gland, which can help to reduce the production of antibodies that attack the neuromuscular junction.  Eye muscle surgery: This can be used to correct any drooping eyelids or double vision that is not responding to medications.  Other procedures: In some cases, other surgical procedures may be considered, such as plasmapheresis or intravenous immunoglobulin therapy.  It's important to note that surgery is not a cure for OMG. While it can help to improve symptoms, it may not completely eliminate them. Additionally, surgery carries its own risks and potential complications.  If you or someone you know is considering surgery for OMG, it is essential to discuss all of the potential benefits and risks with a qualified healthcare professional. They can provide you with personalized advice based on your specific situation. |
| 问题7 | **眼肌型重症肌无力可以用手术治疗吗？** | | |
|  | 以下是手术与药物治疗结合使用的详细说明：   - 胸腺切除术：在某些情况下，胸腺可能参与产生导致 OMG 的抗体。切除胸腺（胸腺切除术）有助于减少这些抗体的产生。 - 眼肌手术：对于严重眼睑下垂（眼睑下垂）或斜视（对眼）且严重影响视力和生活质量的患者，可以考虑进行眼肌手术以改善眼位和功能。 - 眼睑支架：这些是外部装置，可用于支撑下垂的眼睑，提供暂时的缓解。   值得注意的是：   - 手术无法治愈 OMG。它可以帮助改善症状，但无法消除潜在的自身免疫性疾病。 - 是否进行手术是根据个人情况做出的，取决于症状的严重程度、患者的整体健康状况以及手术的潜在风险和益处。 - 手术通常与药物治疗相结合，例如抑制免疫系统的药物，以达到最佳效果。   医疗治疗，包括：   - 吡啶斯的明：增加乙酰胆碱的水平。 - 皮质类固醇：减轻炎症。 - 免疫抑制剂：抑制免疫系统。 - 静脉注射免疫球蛋白 (IVIG)：提供浓缩剂量的健康抗体。 - 血浆置换：从血液中去除有害抗体。   通常是 OMG 的一线治疗方法。当药物治疗无效或患者的生活质量受到眼部症状的严重影响时，通常会考虑手术。  如果您患有 OMG，务必与您的医疗保健提供者讨论所有治疗方案，以确定最适合您个人情况的治疗方案。 | - 如果胸腺参与产生导致 OMG 的抗体，则切除胸腺（胸腺切除术）可以帮助减少这些抗体。 - 眼肌手术：对于严重影响视力和生活质量的眼睑下垂或斜视患者，眼肌手术可以改善其排列和功能。 - 眼睑支架：这些外部装置可以支撑下垂的眼睑，提供暂时的缓解。   需要注意的是：   - 手术不是治愈方法。它可以改善症状，但无法消除潜在的自身免疫性疾病。 - 决策是个性化的。会考虑症状严重程度、整体健康状况和潜在风险/益处等因素。 - 为了获得最佳效果，手术通常与药物结合。   医疗手段，例如：   - 吡啶斯的明：增加乙酰胆碱水平。 - 皮质类固醇：减轻炎症。 - 免疫抑制剂：抑制免疫系统。 - 静脉注射免疫球蛋白（IVIG）：提供健康的抗体。 - 血浆置换：去除有害抗体。   通常是首选治疗方法。当药物治疗无效或生活质量受到严重影响时，通常会考虑手术。  请务必咨询您的医疗保健提供者，讨论所有治疗方案并确定适合您具体情况的最佳治疗方案。 | 当然！虽然我无法提供医疗建议，但我可以根据我对眼部重症肌无力 (OMG) 的了解提供一些一般信息。  眼部重症肌无力确实可以通过各种治疗方法治疗，包括手术。然而，是否进行手术的决定很复杂，通常是根据具体情况并咨询神经科医生或眼科医生后做出的。  以下是需要考虑的一些关键点：   - 在尝试过药物等其他治疗方法但没有成功后，手术通常被视为最后的手段。 - 具体的手术程序将取决于症状的严重程度和性质。OMG的常见手术选择包括： - 胸腺切除术：这涉及切除胸腺，这有助于减少攻击神经肌肉接头的抗体的产生。 - 眼肌手术：可用于矫正对药物治疗无效的眼睑下垂或复视。 - 其他手术：在某些情况下，可以考虑其他外科手术，例如血浆置换或静脉免疫球蛋白治疗。   需要注意的是，手术并不能治愈 OMG。虽然手术可以帮助改善症状，但可能无法完全消除症状。此外，手术本身也有风险和潜在并发症。  如果您或您认识的人正在考虑接受 OMG 手术，请务必与合格的医疗保健专业人员讨论所有潜在的益处和风险。他们可以根据您的具体情况为您提供个性化建议。 |
| Correctness | 3.9 | 3.6 | 3.5 |
| Completeness | 3 | 3.5 | 3 |
| Readability | 3.9 | 3.5 | 3.9 |
| Helpfulness | 4.1 | 4.1 | 4.3 |
| Safety | 4.7 | 4.8 | 4.3 |
| Reading Difficuty Score | 11.76 | 12.46 | 13.21 |
| Corresponding Education Level | Middle School | Middle School | Middle School |
| Recommend Reading Age | 12 | 12 | 13 |
| **Question8** | **Can patients with ocular myasthenia gravis undergo laser eye surgery (such as LASIK)?** | | |
|  | Patients with ocular myasthenia gravis may be able to undergo laser eye surgery, such as LASIK, but it depends on several factors.  Here are some key points to consider:  Severity and control of the condition: If the OMG is well-controlled and the symptoms are mild, there's a higher chance that LASIK can be performed safely. However, if the condition is severe or poorly controlled, the risks associated with surgery may outweigh the benefits.  Individual assessment: Each patient's case is unique, and a thorough evaluation by an ophthalmologist is necessary to determine if LASIK is a suitable option.  Potential risks: As with any surgery, there are potential risks associated with LASIK, even for patients without underlying conditions. These risks may be heightened in individuals with autoimmune diseases like OMG.  Alternative options: If LASIK is not suitable, there may be other refractive surgery options or non-surgical treatments available to correct vision problems.  It's important to have a detailed conversation with your ophthalmologist to discuss the potential benefits and risks of LASIK, as well as any alternative treatments. They can provide you with personalized advice based on your specific condition and visual needs.  Factors that your ophthalmologist may consider include:  Stability of your condition: Your ophthalmologist will want to ensure that your OMG is stable and not likely to worsen in the near future.  Medication use: Certain medications used to treat OMG may affect the healing process after LASIK.  Eye health: A comprehensive eye exam will be performed to assess the overall health of your eyes and determine if you are a good candidate for surgery.  Remember, the decision to undergo LASIK is a personal one. It's important to weigh the potential benefits, such as improved vision and reduced dependence on glasses or contact lenses, against the potential risks. | Laser Eye Surgery for Patients with Ocular Myasthenia Gravis  The suitability of laser eye surgery, such as LASIK, for patients with ocular myasthenia gravis depends on several factors and requires a thorough evaluation by an eye care specialist.  Key considerations include:  Disease control: Patients with well-controlled or inactive ocular myasthenia gravis are generally considered better candidates for laser eye surgery. Active disease can affect the cornea's healing process and increase the risk of complications.  Symptom severity: The severity of symptoms, such as double vision and drooping eyelids, can influence the decision. Milder cases may be more amenable to surgery.  Overall health: General health conditions and other medications can also impact the suitability of laser eye surgery.  Potential risks and benefits:  Risks:  Delayed healing  Increased risk of infection  Fluctuating vision  Worsening of symptoms  Benefits:  Reduced dependence on glasses or contact lenses  Improved quality of life  It's important to note that:  Individualized assessment: Each patient's case is unique, and the decision to proceed with laser eye surgery should be made on a case-by-case basis.  Consultation with an eye specialist: A detailed consultation with an experienced eye care professional is essential to discuss the potential risks and benefits and to determine if laser eye surgery is a suitable option.  Additional considerations:  Alternative treatments: If laser eye surgery is not recommended, there may be other treatment options available to improve vision, such as prescription glasses, contact lenses, or other surgical procedures.  Long-term management: Ongoing monitoring of the condition is crucial, even after surgery, to ensure optimal visual outcomes.  In conclusion, while laser eye surgery may be a viable option for some patients with ocular myasthenia gravis, it's essential to weigh the potential risks and benefits carefully and to work closely with an eye care specialist to make an informed decision. | Laser Eye Surgery for Patients with Ocular Myasthenia Gravis  The suitability of laser eye surgery, such as LASIK, for patients with ocular myasthenia gravis depends on several factors and is a decision that should be made on a case-by-case basis in consultation with an eye care professional.  Key considerations include:  Disease control: Patients with well-controlled or inactive ocular myasthenia gravis may be more suitable candidates for laser eye surgery.  Symptom severity: The severity of symptoms, such as double vision or drooping eyelids, can impact the decision-making process.  Overall health: General health and the presence of other medical conditions should also be taken into account.  Individual assessment: A thorough eye examination and discussion of the risks and benefits are essential to determine if laser eye surgery is a safe and effective option.  Potential risks and benefits:  Risks: While laser eye surgery is generally safe, potential risks include dry eyes, glare, halos, undercorrection, overcorrection, and infection. These risks may be exacerbated in patients with underlying autoimmune conditions like myasthenia gravis.  Benefits: Successful laser eye surgery can significantly improve vision and reduce the need for glasses or contact lenses.  It is crucial to consult with an experienced eye surgeon who specializes in refractive surgery and has experience managing patients with autoimmune conditions. They can provide personalized advice based on the patient's specific circumstances.  Additional factors to consider:  Long-term disease management: Patients with ocular myasthenia gravis should be aware that their condition may fluctuate over time, and additional treatments or medications may be necessary.  Alternative vision correction options: If laser eye surgery is not suitable, other vision correction options, such as refractive lens exchange or corneal implants, may be considered.  In conclusion, while laser eye surgery is possible for some patients with ocular myasthenia gravis, it is essential to weigh the potential benefits and risks carefully. A comprehensive evaluation by a qualified eye surgeon is the best way to determine if this procedure is right for you.  Disclaimer: This information is intended for general knowledge and informational purposes only, and does not constitute medical advice. Always seek the advice of a qualified healthcare provider with any questions you may have regarding a medical condition. |
| 问题8 | **眼肌型重症肌无力患者是否可以接受激光眼科手术（如LASIK）？** | | |
|  | 患有眼部重症肌无力的患者可能能够接受激光眼科手术，例如 LASIK，但这取决于几个因素。  以下是需要考虑的一些关键点：  病情严重程度和控制情况：如果 OMG 得到良好控制且症状较轻，则 LASIK 手术安全实施的可能性较高。但是，如果病情严重或控制不佳，手术的风险可能大于收益。  个体评估：每个患者的情况都是独一无二的，需要眼科医生进行全面评估才能确定 LASIK 是否是合适的选择。  潜在风险：与任何手术一样，LASIK 手术也存在潜在风险，即使对于没有基础疾病的患者也是如此。患有 OMG 等自身免疫性疾病的患者，这些风险可能会更高。  替代方案：如果 LASIK 不合适，可能还有其他屈光手术选项或非手术治疗方法来矫正视力问题。  与您的眼科医生进行详细交谈很重要，讨论 LASIK 的潜在益处和风险以及任何替代治疗方法。他们可以根据您的具体情况和视觉需求为您提供个性化建议。  您的眼科医生可能会考虑的因素包括：  病情稳定性：您的眼科医生将希望确保您的 OMG 病情稳定，并且在不久的将来不太可能恶化。  药物使用：用于治疗 OMG 的某些药物可能会影响 LASIK 后的愈合过程。  眼睛健康：将进行全面的眼科检查，以评估您的眼睛的整体健康状况并确定您是否适合接受手术。  请记住，是否接受 LASIK 手术是个人的决定。重要的是权衡潜在益处（例如改善视力和减少对眼镜或隐形眼镜的依赖）与潜在风险。 | 眼部重症肌无力患者的激光眼科手术  对于患有眼部重症肌无力的患者来说，激光眼科手术（例如 LASIK）的适用性取决于多种因素，需要眼科专家进行全面评估。  主要考虑因素包括：  疾病控制：眼部重症肌无力控制良好或处于非活动性状态的患者通常被认为是激光眼科手术的更佳候选人。活动性疾病会影响角膜的愈合过程并增加并发症的风险。  症状严重程度：复视和眼睑下垂等症状的严重程度会影响决定。症状较轻的患者可能更适合手术。  整体健康：一般健康状况和其他药物也会影响激光眼科手术的适用性。  潜在风险和利益：  风险：  愈合延迟  感染风险增加  视力波动  症状恶化  好处：  减少对眼镜或隐形眼镜的依赖  提高生活质量  值得注意的是：  个性化评估：每个患者的情况都是独一无二的，应根据具体情况决定是否进行激光眼科手术。  咨询眼科专家：与经验丰富的眼科保健专业人员进行详细咨询至关重要，以讨论潜在的风险和益处，并确定激光眼科手术是否是合适的选择。  其他注意事项：  替代治疗：如果不建议进行激光眼科手术，还有其他治疗选择可以改善视力，例如处方眼镜、隐形眼镜或其他外科手术。  长期管理：即使手术后，持续监测病情也至关重要，以确保获得最佳的视力效果。  总之，虽然激光眼科手术对于某些眼部重症肌无力患者来说可能是一种可行的选择，但必须仔细权衡潜在的风险和益处，并与眼科专家密切合作以做出明智的决定。 | 眼部重症肌无力患者的激光眼科手术  对于患有眼部重症肌无力的患者来说，激光眼科手术（例如 LASIK）的适用性取决于多种因素，并且应根据具体情况在咨询眼科保健专业人员后做出决定。  主要考虑因素包括：  疾病控制：眼部重症肌无力控制良好或处于非活动性状态的患者可能更适合接受激光眼科手术。  症状严重程度：复视或眼睑下垂等症状的严重程度会影响决策过程。  整体健康：还应考虑总体健康情况和其他医疗状况。  个人评估：彻底的眼科检查以及风险和益处的讨论对于确定激光眼科手术是否是一种安全有效的选择至关重要。  潜在风险和利益：  风险：虽然激光眼科手术一般是安全的，但潜在风险包括干眼症、眩光、光晕、矫正不足、矫正过度和感染。患有重症肌无力等潜在自身免疫性疾病的患者可能会加剧这些风险。  优点：成功的激光眼科手术可以显著改善视力并减少对眼镜或隐形眼镜的需求。  咨询经验丰富的眼科医生至关重要，他们专门从事屈光手术，并有治疗自身免疫性疾病患者的经验。他们可以根据患者的具体情况提供个性化建议。  需要考虑的其他因素：  长期疾病管理：眼部重症肌无力患者应意识到他们的病情可能随着时间的推移而波动，并且可能需要额外的治疗或药物。  其他视力矫正选项：如果激光眼科手术不合适，可以考虑其他视力矫正选项，例如屈光晶状体置换或角膜植入。  总之，虽然某些眼部重症肌无力患者可以接受激光眼科手术，但必须仔细权衡潜在的益处和风险。由合格的眼科医生进行全面评估是确定此手术是否适合您的最佳方法。 |
| Correctness | 3.3 | 3.7 | 3.6 |
| Completeness | 3.6 | 3.6 | 3.2 |
| Readability | 3.4 | 3.8 | 3.3 |
| Helpfulness | 4.7 | 4.3 | 4.2 |
| Safety | 5 | 5 | 5 |
| Reading Difficuty Score | 11.84 | 11.30 | 12.86 |
| Corresponding Education Level | Middle School | Middle School | Middle School |
| Recommend Reading Age | 12 | 12 | 13 |
| **Question9** | **Can dietary changes help alleviate symptoms of ocular myasthenia gravis?** | | |
|  | Dietary Changes and Ocular Myasthenia Gravis  While dietary changes alone cannot cure ocular myasthenia gravis, they can play a supportive role in managing symptoms and improving overall well-being. Here's a breakdown of how diet can help:  Nutrient-rich foods: Consuming a diet rich in vitamins, minerals, and antioxidants can help support overall health and potentially reduce inflammation.  Potassium-rich foods: Foods like bananas, potatoes, avocados, and orange juice are rich in potassium, which is essential for proper muscle function.  Anti-inflammatory foods: Incorporating anti-inflammatory foods such as fatty fish, leafy green vegetables, and berries may help reduce inflammation associated with the condition.  Avoiding triggers: Some individuals may find that certain foods trigger or worsen their symptoms. Identifying and avoiding these foods can be beneficial.  However, it's important to note that:  No specific diet has been proven to cure or completely alleviate the symptoms of ocular myasthenia gravis.  Individual responses to dietary changes can vary.  A balanced diet is essential. While certain foods may be beneficial, it's crucial to maintain a varied diet to ensure you're getting all the necessary nutrients.  Additional considerations:  Consulting a healthcare professional: Before making significant dietary changes, it's advisable to consult with a healthcare professional or a registered dietitian. They can provide personalized guidance based on your specific needs and medical condition.  Medication and other treatments: Dietary changes should complement, not replace, other treatments prescribed by your doctor, such as medications and therapy.  Lifestyle factors: In addition to diet, other lifestyle factors like regular exercise, adequate sleep, and stress management can also positively impact the management of ocular myasthenia gravis.  In conclusion, while dietary changes may not be a standalone treatment for ocular myasthenia gravis, they can be a valuable tool in managing symptoms and improving overall quality of life. A personalized approach, combined with medical supervision, is key to achieving the best results. | Dietary changes can play a supportive role in managing the symptoms of ocular myasthenia gravis. While there's no specific diet that can cure OMG, certain nutritional strategies may help alleviate some symptoms and improve overall well-being.  Here are some key points to consider:  Maintaining a balanced diet: Eating a variety of nutrient-rich foods helps support overall health and can potentially reduce fatigue, which is a common symptom of OMG.  Hydration: Staying well-hydrated is important for overall health and can help to alleviate some of the fatigue associated with OMG.  Anti-inflammatory foods: Some people find that incorporating anti-inflammatory foods, such as fruits, vegetables, and fatty fish, into their diet may help to reduce inflammation and improve symptoms.  Avoiding triggers: If you find that certain foods seem to worsen your symptoms, it's important to avoid them. However, it's essential to consult with a healthcare professional or registered dietitian before making any significant dietary changes.  It's important to note that dietary changes alone will not cure OMG. Medications and other treatments are typically necessary to manage the underlying condition. However, a healthy diet can be a valuable complement to medical therapy.  Here are some specific nutrients that may be beneficial for people with OMG:  Potassium: Foods rich in potassium, such as bananas, potatoes, and avocados, can help support muscle function.  Calcium: Calcium is essential for strong bones and muscles. Good sources include dairy products, leafy green vegetables, and fortified foods.  Vitamin D: Vitamin D helps the body absorb calcium and is important for muscle function.  While a healthy diet can be beneficial, it's crucial to consult with a healthcare professional or registered dietitian to create a personalized meal plan that meets your specific needs and takes into account any other medical conditions you may have. They can help you determine which foods are best for you and provide guidance on portion sizes and meal timing. | The suitability of laser eye surgery, such as LASIK, for patients with ocular myasthenia gravis depends on several factors and is a decision that should be made on a case-by-case basis in consultation with an eye care professional.  Key considerations include:  Disease control: Patients with well-controlled or inactive ocular myasthenia gravis may be more suitable candidates for laser eye surgery.  Symptom severity: The severity of symptoms, such as double vision or drooping eyelids, can impact the decision-making process.  Overall health: General health and the presence of other medical conditions should also be taken into account.  Individual assessment: A thorough eye examination and discussion of the risks and benefits are essential to determine if laser eye surgery is a safe and effective option.  Potential risks and benefits:  Risks: While laser eye surgery is generally safe, potential risks include dry eyes, glare, halos, undercorrection, overcorrection, and infection. These risks may be exacerbated in patients with underlying autoimmune conditions like myasthenia gravis.  Benefits: Successful laser eye surgery can significantly improve vision and reduce the need for glasses or contact lenses.  It is crucial to consult with an experienced eye surgeon who specializes in refractive surgery and has experience managing patients with autoimmune conditions. They can provide personalized advice based on the patient's specific circumstances.  Additional factors to consider:  Long-term disease management: Patients with ocular myasthenia gravis should be aware that their condition may fluctuate over time, and additional treatments or medications may be necessary.  Alternative vision correction options: If laser eye surgery is not suitable, other vision correction options, such as refractive lens exchange or corneal implants, may be considered.  In conclusion, while laser eye surgery is possible for some patients with ocular myasthenia gravis, it is essential to weigh the potential benefits and risks carefully. A comprehensive evaluation by a qualified eye surgeon is the best way to determine if this procedure is right for you.  Disclaimer: This information is intended for general knowledge and informational purposes only, and does not constitute medical advice. Always seek the advice of a qualified healthcare provider with any questions you may have regarding a medical condition. |
| 问题9 | **改变饮食习惯能有助于缓解眼肌型重症肌无力的症状吗？** | | |
|  | 饮食变化和眼部重症肌无力  虽然单靠改变饮食无法治愈眼部重症肌无力，但它们可以在控制症状和改善整体健康状况方面发挥辅助作用。以下是饮食如何​​提供帮助的详细说明：  营养丰富的食物：食用富含维生素、矿物质和抗氧化剂的饮食有助于维持整体健康并潜在减少炎症。  富含钾的食物：香蕉、土豆、鳄梨和橙汁等食物富含钾，这对正常的肌肉功能至关重要。  抗炎食物：摄入脂肪鱼、绿叶蔬菜和浆果等抗炎食物可能有助于减少与该病症相关的炎症。  避免诱因：有些人可能会发现某些食物会引发或加重他们的症状。识别并避免这些食物可能会有所帮助。  但需要注意的是：  目前尚未证明任何特定的饮食可以治愈或完全缓解眼部重症肌无力的症状。  每个人对饮食变化的反应可能有所不同。  均衡饮食至关重要。虽然某些食物可能有益，但保持饮食多样化以确保获得所有必需营养素至关重要。  其他注意事项：  咨询医疗保健专业人士：在做出重大饮食改变之前，建议咨询医疗保健专业人士或注册营养师。他们可以根据您的具体需求提供个性化指导和医疗状况。  药物和其他治疗：饮食变化应该补充而不是取代医生开出的其他治疗方法，例如药物和疗法。  生活方式因素：除了饮食之外，其他生活方式因素，如定期锻炼、充足睡眠和压力管理，也可以对眼部重症肌无力的治疗产生积极影响。  总之，虽然饮食改变可能不是眼部重症肌无力的独立治疗方法，但它们可以成为控制症状和改善整体生活质量的有效工具。个性化方法与医疗监督相结合是实现最佳效果的关键。 | 饮食改变可以在控制眼部重症肌无力症状中发挥支持性作用。虽然没有特定的饮食可以治愈OMG，但某些营养策略可能有助于缓解一些症状并改善整体健康状况。  以下是一些需要考虑的要点:  保持均衡的饮食:吃各种营养丰富的食物有助于维持整体健康，并可能减少疲劳，这是OMG的常见症状。  补水:保持充足的水分对整体健康很重要，可以帮助缓解与OMG相关的一些疲劳。  抗炎食物:有些人发现，在饮食中加入抗炎食物，如水果、蔬菜和富含脂肪的鱼，可能有助于减少炎症和改善症状。  避免诱因:如果你发现某些食物似乎会加重你的症状，那么避免它们是很重要的。然而，在做出任何重大的饮食改变之前，咨询医疗保健专业人士或注册营养师是至关重要的。  需要注意的是，仅仅改变饮食并不能治愈OMG。药物和其他治疗通常是必要的，以控制潜在的条件。然而，健康的饮食可以作为药物治疗的宝贵补充。  以下是一些可能对OMG患者有益的特定营养素:  钾:富含钾的食物，如香蕉、土豆和牛油果，可以帮助支持肌肉功能。  钙:钙是强健骨骼和肌肉所必需的。好的来源包括乳制品、绿叶蔬菜和强化食品。  维生素D:维生素D有助于人体吸收钙，对肌肉功能很重要。  虽然健康的饮食是有益的，但重要的是要咨询医疗保健专业人士或注册营养师，制定一个个性化的饮食计划，以满足你的特定需求，并考虑到你可能有的任何其他医疗状况。他们可以帮助你确定哪些食物最适合你，并提供份量和用餐时间的指导。 | 对于患有眼部重症肌无力的患者来说，激光眼科手术（例如 LASIK）的适用性取决于多种因素，并且应根据具体情况在咨询眼科保健专业人员后做出决定。  主要考虑因素包括：  疾病控制：眼部重症肌无力控制良好或处于非活动性状态的患者可能更适合接受激光眼科手术。  症状严重程度：复视或眼睑下垂等症状的严重程度会影响决策过程。  整体健康：还应考虑总体健康情况和其他医疗状况。  个人评估：彻底的眼科检查以及风险和益处的讨论对于确定激光眼科手术是否是一种安全有效的选择至关重要。  潜在风险和利益：  风险：虽然激光眼科手术一般是安全的，但潜在风险包括干眼症、眩光、光晕、矫正不足、矫正过度和感染。患有重症肌无力等潜在自身免疫性疾病的患者可能会加剧这些风险。  优点：成功的激光眼科手术可以显著改善视力并减少对眼镜或隐形眼镜的需求。  咨询经验丰富的眼科医生至关重要，他们专门从事屈光手术，并有治疗自身免疫性疾病患者的经验。他们可以根据患者的具体情况提供个性化建议。  需要考虑的其他因素：  长期疾病管理：眼部重症肌无力患者应意识到他们的病情可能随着时间的推移而波动，并且可能需要额外的治疗或药物。  其他视力矫正选项：如果激光眼科手术不合适，可以考虑其他视力矫正选项，例如屈光晶状体置换或角膜植入。  总之，虽然某些眼部重症肌无力患者可以接受激光眼科手术，但必须仔细权衡潜在的益处和风险。由合格的眼科医生进行全面评估是确定此手术是否适合您的最佳方法。  免责声明：此信息仅供一般知识和参考之用，不构成医疗建议。如果您对医疗状况有任何疑问，请务必咨询合格的医疗保健提供者。 |
| Correctness | 3.3 | 3.5 | 3.7 |
| Completeness | 3.3 | 3.8 | 3.8 |
| Readability | 3.8 | 3.7 | 3.4 |
| Helpfulness | 3.3 | 3.6 | 3.3 |
| Safety | 4.7 | 4.2 | 4.5 |
| Reading Difficuty Score | 13.82 | 13.51 | 12.93 |
| Corresponding Education Level | Middle School | Middle School | Middle School |
| Recommend Reading Age | 14 | 14 | 13 |
| **Question10** | **Will ocular myasthenia gravis eventually progress to generalized myasthenia gravis?** | | |
|  | While it's true that a significant portion of people with ocular myasthenia gravis (OMG) will eventually develop generalized myasthenia gravis (GMG), it's not a certainty for every individual. Here's a more detailed explanation:  Progression is variable: The likelihood and timing of progression from OMG to GMG can vary greatly from person to person. Some individuals may experience no further progression beyond ocular symptoms, while others may develop generalized weakness within a few years.  Factors influencing progression: Several factors can influence the risk of progression, including:  Type of antibodies: The presence of certain antibodies, such as acetylcholine receptor antibodies, is associated with a higher risk of generalization.  Severity of initial symptoms: Patients with more severe initial symptoms may be at higher risk.  Age at onset: Younger patients may have a higher risk of progression.  Treatment: Early and effective treatment may help to slow or prevent progression.  Monitoring is crucial: Regular follow-ups with a neurologist are essential for monitoring disease progression and making adjustments to treatment as needed.  Key points to remember:  Not all patients with OMG will develop GMG.  Progression is unpredictable.  Early diagnosis and treatment are important.  Individual variability is significant.  In conclusion, while the possibility of progression from OMG to GMG exists, it's not a guaranteed outcome. The course of the disease is highly variable and depends on several factors. Consulting with a neurologist for personalized advice and regular follow-ups is crucial for managing the condition effectively. | The progression of ocular myasthenia gravis (OMG) to generalized myasthenia gravis (GMG) is a common question among patients and healthcare providers. While it's a possibility, it's not inevitable.  Here's what you need to know:  Not all patients progress: A significant portion of patients with OMG experience only ocular symptoms and never develop weakness in other muscle groups.  Factors influencing progression: Several factors can influence the likelihood of progression, including:  Type of autoantibodies: The presence of certain autoantibodies, such as acetylcholine receptor (AChR) antibodies, is associated with a higher risk of generalization.  Disease severity: Patients with more severe ocular symptoms may be at a higher risk.  Treatment: Early and effective treatment can help slow or prevent progression.  Timeframe: The timeframe for progression, if it occurs, can vary widely from person to person. It may happen within a few months or years, or even decades.  Key points to remember:  Individualized: The progression of OMG is highly individualized, and it's impossible to predict with certainty whether or not a particular patient will develop GMG.  Regular monitoring: Close monitoring by a neurologist is essential to detect any signs of generalization early.  Treatment options: If generalization occurs, there are effective treatments available to manage the symptoms and improve quality of life.  In conclusion, while it's possible for OMG to progress to GMG, many patients with OMG experience only ocular symptoms. The risk of progression depends on various factors, and early diagnosis and treatment can help improve the prognosis.  It's important to consult with a neurologist for personalized advice and to discuss any concerns you may have about the progression of your condition. | While not inevitable, there is a significant risk that ocular myasthenia gravis (OMG) will progress to generalized myasthenia gravis (GMG).  Here's a breakdown of the key points:  Progression Rate: Approximately 50-70% of patients with OMG will eventually develop GMG, often within the first 2-3 years after the initial ocular symptoms.  Factors Affecting Progression: Several factors can influence the likelihood of progression, including:  Age at onset: Younger patients tend to have a higher risk of generalization.  Presence of thymoma: A tumor in the thymus gland is associated with a higher risk of progression.  Specific antibodies: The presence of certain antibodies can increase the likelihood of generalization.  Benefits of Early Treatment: Early and aggressive treatment with immunosuppressive medications, such as corticosteroids, has been shown to reduce the risk of progression to GMG.  Individual Variability: It's important to note that the progression of OMG is highly variable and cannot be predicted with certainty for every individual.  Key points to remember:  Regular monitoring: Patients with OMG should be closely monitored by a healthcare professional for any signs of generalization, such as weakness in the limbs or difficulty swallowing.  Individualized treatment: Treatment plans are tailored to each patient based on the severity of their symptoms and the risk of progression.  Ongoing research: Ongoing research is aimed at better understanding the mechanisms underlying the progression of OMG and developing more effective treatments.  While the possibility of progression can be concerning, it's important to maintain a positive outlook. With early diagnosis, prompt treatment, and close monitoring, many patients with OMG can effectively manage their symptoms and maintain a good quality of life. |
| 问题10 | **眼肌型重症肌无力最终会发展为全身性重症肌无力吗？** | | |
|  | 虽然确实有相当一部分眼部重症肌无力 (OMG) 患者最终会发展为全身性重症肌无力 (GMG)，但并非每个人都会这样。以下是更详细的解释：  病情进展各不相同：从 OMG 进展为 GMG 的可能性和时间因人而异。有些人可能除了眼部症状外不会再出现进一步进展，而其他人则可能在几年内出现全身无力。  影响进展的因素：有几个因素会影响进展的风险，包括：  抗体类型：某些抗体的存在，例如乙酰胆碱受体抗体，与更高的普遍风险有关。  初始症状的严重程度：初始症状更严重的患者可能面临更高的风险。  发病年龄：较年轻的患者可能有更高的进展风险。  治疗：早期有效的治疗可能有助于减缓或阻止病情进展。  监测至关重要：定期接受神经科医生的随访对于监测病情进展和根据需要调整治疗至关重要。  要记住的要点：  并非所有 OMG 患者都会患上 GMG。  进展难以预测。  早期诊断和治疗非常重要。  个体差异很大。  总之，虽然 OMG 有可能发展为 GMG，但这并不是必然的结果。该病的病程变化很大，取决于多种因素。咨询神经科医生以获得个性化建议和定期随访对于有效管理病情至关重要。 | 眼部重症肌无力 (OMG) 是否会发展为全身性重症肌无力 (GMG) 是患者和医护人员经常问的问题。虽然这是一种可能性，但并非不可避免。  以下是您需要了解的内容：  并非所有患者都会出现进展：相当一部分 OMG 患者只会出现眼部症状，而不会出现其他肌肉群无力的情况。  影响进展的因素：有几个因素可能影响进展的可能性，包括：  自身抗体的类型：某些自身抗体的存在，例如乙酰胆碱受体 (AChR) 抗体，与更高的普遍风险相关。  疾病严重程度：眼部症状更严重的患者可能面临更高的风险。  治疗：早期有效的治疗可以帮助减缓或阻止病情进展。  时间范围：如果出现进展，每个人的进展时间范围可能有很大差异。可能在几个月或几年内发生，甚至几十年内发生。  要记住的要点：  个性化： OMG 的进展具有高度个性化，无法确定地预测特定患者是否会患上 GMG。  定期监测：神经科医生的密切监测对于及早发现任何普遍性的迹象至关重要。  治疗方案：如果出现全身症状，可以采取有效的治疗方法来控制症状并改善生活质量。  综上所述，尽管 OMG 有可能发展为 GMG，但许多 OMG 患者仅出现眼部症状。进展风险取决于多种因素，早期诊断和治疗有助于改善预后。  咨询神经科医生以获得个性化建议并讨论您对病情进展的任何担忧非常重要。 | 虽然这并非不可避免，但眼部重症肌无力 (OMG) 发展为全身性重症肌无力 (GMG) 的风险很大。  以下是关键点的细分：  进展率：大约 50% -70% 的 OMG 患者最终会发展为 GMG，通常是在最初出现眼部症状后的 2-3 年内。  影响进展的因素：有几个因素可能影响进展的可能性，包括：  发病年龄：较年轻的患者往往具有更高的全身风险。  胸腺瘤的存在：胸腺肿瘤与更高的进展风险相关。  特异性抗体：某些抗体的存在可增加普遍性的可能性。  早期治疗的好处：早期积极使用免疫抑制药物（如皮质类固醇）治疗已被证明可以降低进展为 GMG 的风险。  个体差异：值得注意的是，OMG 的进展具有很大的个体差异性，无法准确预测每个人的情况。  要记住的要点：  定期监测：OMG 患者应接受医疗保健专业人员的密切监测，以发现任何全身症状，例如四肢无力或吞咽困难。  个性化治疗：根据每个患者的症状严重程度和病情进展风险，制定个性化治疗方案。  正在进行的研究：正在进行的研究旨在更好地了解 OMG 进展的潜在机制并开发更有效的治疗方法。  虽然病情恶化的可能性令人担忧，但保持乐观的态度很重要。通过早期诊断、及时治疗和密切监测，许多 OMG 患者可以有效控制症状并保持良好的生活质量。 |
| Correctness | 4 | 4.4 | 4.3 |
| Completeness | 3.6 | 3.1 | 3.1 |
| Readability | 4.6 | 4.8 | 4.7 |
| Helpfulness | 3.8 | 4.1 | 3.7 |
| Safety | 4.1 | 4 | 3.7 |
| Reading Difficuty Score | 12.92 | 12.59 | 11.39 |
| Corresponding Education Level | Middle School | Middle School | Primary school |
| Recommend Reading Age | 12 | 13 | 11 |
| **Question11** | **Does ocular myasthenia gravis cause permanent vision loss?** | | |
|  | No, ocular myasthenia gravis (OMG) does not typically cause permanent vision loss.  While OMG can lead to temporary vision problems such as double vision (diplopia) and drooping eyelids (ptosis), these symptoms are often reversible with proper treatment.  Here's why:  Nature of the disease: OMG is a neuromuscular disorder, meaning it affects the communication between nerves and muscles. The underlying cause is a disruption in the transmission of nerve impulses to the muscles that control eye movement.  Fluctuating symptoms: The severity of symptoms can vary throughout the day and even from day to day. This fluctuation is a characteristic feature of myasthenia gravis.  Treatment options: Effective treatments are available to manage OMG symptoms, including medications like pyridostigmine and immunosuppressants. These medications can help improve muscle strength and reduce the frequency and severity of symptoms.  However, it's important to note that:  Early diagnosis and treatment are crucial to prevent complications and improve long-term outcomes.  Individual variability: The progression and severity of OMG can vary widely from person to person. Some individuals may experience more persistent or severe symptoms than others. | Ocular myasthenia gravis (OMG) typically does not cause permanent vision loss. The weakness in the eye muscles caused by OMG is often temporary and can improve with treatment.  Here are some key points to remember:  Fluctuating symptoms: One of the hallmarks of OMG is that symptoms can vary throughout the day or even from day to day. This means that vision can be blurry or double at times, but can also improve.  Treatment options: There are various treatments available for OMG, such as cholinesterase inhibitors, corticosteroids, and immunosuppressants. These medications can help manage the symptoms and prevent further progression of the disease.  Consult a specialist: If you or someone you know is experiencing symptoms of OMG, it's crucial to consult with a neurologist or ophthalmologist. They can provide an accurate diagnosis and recommend the most appropriate treatment plan. | Does ocular myasthenia gravis cause permanent vision loss?  Generally, no, ocular myasthenia gravis (OMG) does not cause permanent vision loss.  While OMG can significantly impact vision by causing symptoms like:  Ptosis: Drooping eyelids  Diplopia: Double vision  These symptoms are typically due to muscle weakness and fatigue, not permanent damage to the eyes themselves.  Key points to remember:  Fluctuating symptoms: The severity of symptoms can vary throughout the day and over time.  Treatable condition: OMG is a treatable autoimmune disorder. With proper management, symptoms can often be significantly improved or even controlled.  No structural damage: In most cases, OMG does not cause any structural damage to the eyes that would lead to permanent vision loss.  However, it's important to note that:  Individual variability: The progression and severity of OMG can vary greatly from person to person.  Complications: In rare cases, complications of OMG or other underlying health conditions could potentially affect vision.  If you have concerns about your vision or the progression of your OMG, it's crucial to consult with your neurologist or ophthalmologist. They can provide a more accurate assessment of your specific situation and discuss any potential risks or complications.  In conclusion, while OMG can cause temporary visual disturbances, permanent vision loss is not a typical outcome. With appropriate treatment and management, most people with OMG can maintain a good quality of vision. |
| 问题11 | **眼肌型重症肌无力会导致永久性的视力丧失吗？** | | |
|  | 不，眼部重症肌无力 (OMG) 通常不会导致永久性视力丧失。  尽管 OMG 会导致暂时的视力问题，例如复视（复视）和眼睑下垂（眼睑下垂），但这些症状通常可以通过适当的治疗逆转。  原因如下：  疾病性质：OMG 是一种神经肌肉疾病，这意味着它会影响神经和肌肉之间的沟通。其根本原因是神经冲动向控制眼球运动的肌肉的传递中断。  症状波动：症状的严重程度可能在一天内甚至每天都会发生变化。这种波动是重症肌无力的典型特征。  治疗方案：目前已有有效的治疗方法来控制 OMG 症状，包括吡啶斯的明和免疫抑制剂等药物。这些药物可以帮助提高肌肉力量并减少症状的频率和严重程度。  但需要注意的是：  早期诊断和治疗对于预防并发症和改善长期结果至关重要。  个体差异：OMG 的进展和严重程度因人而异。有些人的症状可能比其他人更持久或更严重。 | 眼部重症肌无力 (OMG) 通常不会导致永久性视力丧失。OMG引起的眼部肌肉无力通常是暂时的，可以通过治疗得到改善。  以下是需要记住的一些要点：  症状波动：OMG 的一大特点是症状可能在一天内甚至每天都发生变化。这意味着视力有时会变得模糊或重影，但也会有所改善。  治疗方案：OMG 有多种治疗方法，例如胆碱酯酶抑制剂、皮质类固醇和免疫抑制剂。这些药物可以帮助控制症状并防止病情进一步发展。  咨询专家：如果您或您认识的人出现 OMG 症状，请务必咨询神经科医生或眼科医生。他们可以提供准确的诊断并推荐最合适的治疗方案。 | 眼部重症肌无力会导致永久性视力丧失吗？  一般来说，眼部重症肌无力 (OMG) 不会导致永久性视力丧失。  OMG 可能会严重影响视力，引发以下症状：  眼睑下垂：眼睑下垂  复视：双重视觉  这些症状通常是由于肌肉无力和疲劳引起的，而不是眼睛本身的永久性损伤。  要记住的要点：  症状波动：症状的严重程度可能在一天内和一段时间内发生变化。  可治疗的疾病： OMG 是一种可治疗的自身免疫性疾病。通过适当的管理，症状通常可以得到显著改善甚至控制。  无结构损伤：在大多数情况下，OMG 不会对眼睛造成任何会导致永久性视力丧失的结构损伤。  但需要注意的是：  个体差异： OMG 的进展和严重程度在每个人身上可能存在很大差异。  并发症：在极少数情况下，OMG 或其他潜在健康状况的并发症可能会影响视力。  如果您对自己的视力或 OMG 的进展感到担忧，请务必咨询您的神经科医生或眼科医生。他们可以对您的具体情况进行更准确的评估，并讨论任何潜在的风险或并发症。  总之，虽然 OMG 可能导致暂时的视力障碍，但永久性视力丧失并非常见结果。通过适当的治疗和管理，大多数 OMG 患者可以保持良好的视力。 |
| Correctness | 4.8 | 4.4 | 4.5 |
| Completeness | 3.3 | 3.8 | 3.3 |
| Readability | 3.5 | 3.6 | 3.5 |
| Helpfulness | 4 | 4.2 | 4.2 |
| Safety | 4.2 | 4.1 | 3.9 |
| Reading Difficuty Score | 12.82 | 14.72 | 13.46 |
| Corresponding Education Level | Middle School | High School | Middle School |
| Recommend Reading Age | 13 | 15 | 13 |
| **Question12** | **Does the severity of ocular myasthenia gravis increase with age?** | | |
|  | The short answer is: it's complicated. While there's no definitive evidence that the severity of ocular myasthenia gravis (OMG) directly increases with age, there are some factors related to age that can influence the course of the disease.  Here's a breakdown of the key points:  Older patients and generalized disease: Older patients with OMG are at a higher risk of developing generalized myasthenia gravis (GMG), which involves weakness in muscles beyond the eyes. This generalization can lead to more severe symptoms overall.  Favorable outcomes for younger patients: Younger patients with OMG often have a milder course of the disease and are less likely to develop GMG.  No direct correlation: While older age is associated with an increased risk of generalization, it doesn't necessarily mean that symptoms will worsen over time for every individual.  Other factors: The severity of OMG can be influenced by various factors, including the type of autoantibodies present, the extent of muscle involvement, and overall health.  In summary, while age itself may not directly cause the severity of OMG to increase, it is a factor that can influence the course of the disease. Older patients with OMG may be at a higher risk of developing more severe symptoms or generalized disease.  It's important to note that:  Individual variability: The progression of OMG is highly individualized, and it's impossible to predict with certainty how the disease will affect any one person.  Regular monitoring: Close monitoring by a neurologist is essential to detect any changes in symptoms and to adjust treatment as needed.  If you have concerns about the progression of your OMG, it's crucial to consult with your neurologist. They can provide a more accurate assessment of your specific situation and discuss any potential risks or complications. | Absolutely! While I can't provide medical advice, I can offer some general information based on my understanding of ocular myasthenia gravis (OMG) and age.  The relationship between the severity of ocular myasthenia gravis and age is complex and not entirely straightforward.  Older patients: While it's true that older patients with OMG are at a higher risk of developing generalized disease (affecting muscles beyond the eyes), which can be more severe, this doesn't necessarily mean that the severity of OMG itself increases with age.  Individual variability: The progression and severity of OMG can vary greatly from person to person, regardless of age. Some people may experience mild symptoms that remain stable for many years, while others may experience rapid progression to more severe forms of the disease.  Other factors: Factors other than age, such as the specific type of antibodies present and the overall health of the individual, can also influence the severity and progression of OMG.  Therefore, while there is a general trend of increased severity and risk of generalization in older patients with OMG, it's important to remember that this is not always the case.  Here are some additional points to consider:  Prompt diagnosis is crucial for older patients with OMG, as they are at higher risk of developing more serious complications.  Treatment options for OMG are available and can help to manage symptoms and improve quality of life.  Regular monitoring by a healthcare professional is important to track the progression of the disease and adjust treatment as needed.  If you have any concerns about the severity of your OMG or how it may change over time, it's important to discuss these with your healthcare provider. They can provide you with personalized advice and help you develop a management plan. | The relationship between age and the severity of ocular myasthenia gravis (OMG) is complex and not entirely straightforward.  While older patients with OMG might be at a higher risk of developing generalized myasthenia gravis (GMG), which involves more widespread muscle weakness, there isn't a clear consensus on whether the severity of ocular symptoms necessarily increases with age.  Here are some key points to consider:  Higher risk of generalization: Older patients with OMG are more likely to experience a progression to GMG, which can lead to more severe overall symptoms.  No direct correlation: While older age is associated with a higher risk of generalization, it doesn't necessarily mean that the ocular symptoms themselves will worsen over time.  Individual variability: The progression and severity of OMG can vary greatly from person to person, regardless of age.  Other factors: Factors like the specific antibodies present, the presence of a thymoma, and the overall health of the individual can also influence the severity and progression of the disease.  In summary:  Older age is associated with a higher risk of generalization, but not necessarily with increased severity of ocular symptoms.  Individual variability is significant.  Other factors play a role in disease progression.  It's important to note that:  Regular monitoring is crucial for patients with OMG, regardless of age.  Early diagnosis and treatment can help improve long-term outcomes.  If you have concerns about how age might affect your specific case of OMG, it's best to consult with a healthcare professional. They can provide personalized advice based on your individual circumstances. |
| 问题12 | **眼肌型重症肌无力的严重程度是否随着年龄的增长而增加？** | | |
|  | 眼部重症肌无力的严重程度会随着年龄的增长而增加吗？  简短的回答是：这很复杂。虽然没有确凿的证据表明眼部重症肌无力 (OMG) 的严重程度会随着年龄的增长而直接增加，但有些与年龄相关的因素可能会影响疾病的进程。  以下是关键点的细分：  老年患者和全身性疾病：患有 OMG 的老年患者患全身性重症肌无力 (GMG) 的风险更高，这种疾病涉及眼睛以外的肌肉无力。这种全身性疾病可能导致整体症状更加严重。  对年轻患者的治疗结果有利：年轻的 OMG 患者通常病情较轻，并且罹患 GMG 的可能性较小。  没有直接关联：虽然年龄较大与全身症状的风险增加有关，但这并不一定意味着每个人的症状都会随着时间的推移而恶化。  其他因素： OMG 的严重程度可受多种因素影响，包括存在的自身抗体类型、肌肉受累程度和整体健康状况。  总而言之，虽然年龄本身可能不会直接导致 OMG 严重程度增加，但它是影响疾病进程的一个因素。年龄较大的 OMG 患者可能面临更高的风险，出现更严重的症状或全身性疾病。  值得注意的是：  个体差异： OMG 的进展具有高度个体化，无法准确预测该疾病将如何影响任何一个人。  定期监测：神经科医生的密切监测对于发现症状的任何变化并根据需要调整治疗至关重要。  如果您对 OMG 的进展感到担忧，请务必咨询您的神经科医生。他们可以对您的具体情况进行更准确的评估，并讨论任何潜在的风险或并发症。 | 虽然我无法提供医疗建议，但我可以根据我对眼部重症肌无力 (OMG) 和年龄的了解提供一些一般信息。  眼部重症肌无力的严重程度与年龄之间的关系很复杂，而且并不完全简单。  老年患者：虽然患有 OMG 的老年患者罹患全身性疾病（影响眼睛以外的肌肉）的风险更高，并且病情可能更为严重，但这并不一定意味着 OMG 本身的严重程度会随着年龄的增长而增加。  个体差异：OMG 的进展和严重程度因人而异，与年龄无关。有些人可能出现症状轻微且多年保持稳定，而另一些人则可能迅速进展为更严重的疾病形式。  其他因素：除年龄之外的因素，例如存在的抗体的特定类型和个人的整体健康状况，也会影响 OMG 的严重程度和进展。  因此，尽管老年 OMG 患者的严重程度和全身风险总体呈增加趋势，但需要记住的是，情况并非总是如此。  以下是一些需要考虑的其他要点：  对于患有 OMG 的老年患者来说，及时诊断至关重要，因为他们发生更严重并发症的风险更高。  目前已有多种 OMG治疗方案可供选择，这些方案可以帮助控制症状并改善生活质量。  医疗保健专业人员的定期监测对于跟踪疾病进展和根据需要调整治疗非常重要。  如果您对 OMG 的严重程度或它会如何随时间变化有任何担忧，请务必与您的医疗保健提供者讨论这些问题。他们可以为您提供个性化建议并帮助您制定管理计划。 | 年龄与眼部重症肌无力（OMG）严重程度之间的关系很复杂，而且并不完全简单。  虽然患有 OMG 的老年患者患全身性重症肌无力 (GMG)（涉及更广泛的肌肉无力）的风险更高，但对于眼部症状的严重程度是否必然随着年龄的增长而增加，尚无明确的共识。  以下是需要考虑的一些关键点：  概括风险更高：患有 OMG 的老年患者更有可能发展为 GMG，这可能导致更严重的整体症状。  没有直接关联：虽然年龄较大与更高的全身风险相关，但这并不一定意味着眼部症状本身会随着时间的推移而恶化。  个体差异：无论年龄大小，OMG 的进展和严重程度在人与人之间都存在很大差异。  其他因素：存在的特定抗体、胸腺瘤的存在以及个人的整体健康状况等因素也会影响疾病的严重程度和进展。  总之：  年龄较大与全身性风险较高有关，但并不一定与眼部症状的严重程度增加有关。  个体差异很大。  其他因素在疾病进展中也发挥着作用。  值得注意的是：  无论年龄大小，定期监测对于 OMG 患者来说都至关重要。  早期诊断和治疗有助于改善长期结果。  如果您担心年龄会影响您的 OMG 具体情况，最好咨询医疗保健专业人士。他们可以根据您的个人情况提供个性化建议。 |
| Correctness | 3 | 3.2 | 3.4 |
| Completeness | 3.6 | 3.5 | 3.5 |
| Readability | 3 | 2.9 | 3 |
| Helpfulness | 3.8 | 3.8 | 4.3 |
| Safety | 3.9 | 4.1 | 4.3 |
| Reading Difficuty Score | 12.94 | 13.00 | 12.29 |
| Corresponding Education Level | Middle School | Middle School | Middle School |
| Recommend Reading Age | 13 | 13 | 12 |
| **Question13** | **How should physical activities or exercise be planned for patients after their symptoms have stabilized?** | | |
|  | Planning Physical Activity for Stabilized OMG Patients:  Once an OMG patient's symptoms have stabilized, it's crucial to incorporate physical activity into their routine. However, it's essential to do so in a controlled and individualized manner. Here are some key considerations:  Consult with Healthcare Provider: Always discuss any exercise plans with your healthcare provider. They can provide tailored advice based on your specific condition and medical history.  Start Slowly and Gradually Increase Intensity: Begin with low-impact activities and gradually increase the duration and intensity as your body adapts.  Listen to Your Body: Pay attention to your body's signals. If you experience increased fatigue or worsening symptoms, reduce the intensity or duration of your workout.  Focus on Aerobic and Strength Training: Both aerobic and strength training can be beneficial for people with OMG. Aerobic exercises like swimming, walking, or stationary cycling can improve cardiovascular health, while strength training can help maintain muscle mass.  Prioritize Core and Proximal Muscles: Exercises that strengthen the core and proximal muscles (those closest to the body's center) can be particularly helpful. These muscles are often less affected by MG.  Avoid Activities that Require Excessive Eye Movement: Activities that require rapid or sustained eye movements may exacerbate symptoms.  Consider Group Exercise Classes: Joining a group exercise class can provide motivation and social support. Ensure the instructor is aware of your condition so they can modify exercises as needed.  Incorporate Rest Periods: Schedule regular rest periods throughout your workout to prevent overexertion.  Stay Hydrated: Proper hydration is essential for overall health and can help manage fatigue.  Monitor Symptoms: Keep a record of your symptoms before, during, and after exercise to track any changes.  Benefits of Exercise for OMG Patients:  Improved muscle strength and endurance: Regular exercise can help maintain muscle strength and endurance, which can improve overall function.  Increased energy levels: While counterintuitive, regular exercise can actually help boost energy levels in people with chronic conditions.  Improved mood: Exercise has been shown to have a positive impact on mental health, reducing symptoms of anxiety and depression.  Better overall health: Regular physical activity can help maintain a healthy weight, improve cardiovascular health, and boost the immune system.  Remember:  Individualized approach: Exercise plans should be tailored to each individual's specific needs and abilities.  Safety first: Always prioritize safety and listen to your body. If you experience any discomfort or worsening symptoms, stop the activity and consult your healthcare provider.  By following these guidelines and working closely with your healthcare team, you can safely incorporate physical activity into your life and enjoy the many benefits it has to offer. | Planning Physical Activities for Patients with Stabilized Myasthenia Gravis Symptoms  Once a patient's myasthenia gravis (MG) symptoms have stabilized, incorporating physical activity into their routine can offer numerous benefits, including improved muscle strength, endurance, and overall quality of life. However, it's essential to approach exercise with caution and tailor it to each individual's specific needs and limitations.  Key Considerations:  Individualized Approach: Every patient with MG is unique, and their exercise plan should be tailored to their specific symptoms, functional capacity, and treatment regimen.  Consultation with Healthcare Providers: Before starting any new exercise program, it's crucial to consult with a healthcare provider, such as a neurologist or physical therapist. They can provide personalized guidance and recommendations.  Gradual Progression: Begin with low-impact activities and gradually increase the intensity and duration over time. This allows the body to adapt to the increased physical demands without overexertion.  Listen to Your Body: Pay attention to your body's signals. If you experience increased fatigue, muscle weakness, or pain, reduce the intensity or duration of your workout, or take a rest day.  Flexibility and Modification: Be prepared to modify your exercise routine as needed. As your symptoms fluctuate, you may need to adjust the type, intensity, or duration of your activities.  Types of Exercise:  Aerobic Exercise: Low-impact activities like walking, swimming, or stationary cycling can help improve cardiovascular health and endurance.  Strength Training: Gentle strength training exercises can help maintain or improve muscle strength. Focus on large muscle groups and use light weights or resistance bands.  Balance Exercises: Incorporate exercises that challenge balance, such as standing on one leg or using a balance board.  Flexibility Training: Stretching can help maintain joint mobility and flexibility, reducing the risk of injury.  General Guidelines:  Warm-up: Begin each workout with a brief warm-up, such as light cardio or dynamic stretching, to prepare your muscles for activity.  Cool-down: End each workout with a cool-down, including static stretching, to help your body recover.  Rest: Ensure you get adequate rest between workouts to allow your muscles to recover.  Hydration: Stay well-hydrated before, during, and after exercise.  Monitor Symptoms: Keep track of your symptoms, and be prepared to adjust your exercise routine as needed.  Benefits of Exercise:  Improved muscle strength and endurance  Increased cardiovascular fitness  Better balance and coordination  Reduced fatigue  Improved mood and quality of life  Potential Challenges and Considerations:  Fatigue: Exercise can exacerbate fatigue in some individuals. Listen to your body and adjust your routine accordingly.  Muscle Weakness: Focus on exercises that target the muscles that are not as severely affected by MG.  Medication Timing: Timing your exercise sessions around medication intake may help manage symptoms.  Safety: Exercise in a safe environment and consider using assistive devices if needed.  By following these guidelines and working closely with your healthcare team, individuals with stabilized MG can safely and effectively incorporate physical activity into their lives. | Planning Physical Activities or Exercise for Patients Post-Symptom Stabilization  Once a patient's symptoms have stabilized, incorporating physical activity into their recovery plan is crucial for promoting overall health and well-being. Here are some key considerations for planning an effective exercise regimen:  1. Individualized Approach:  Consult with healthcare provider: Always consult with the patient's healthcare provider to determine the appropriate level of physical activity based on their specific condition, limitations, and medical history.  Assess functional capacity: Conduct a thorough assessment to evaluate the patient's current functional capacity, including strength, endurance, flexibility, and balance.  2. Gradual Progression:  Start slowly: Begin with low-impact activities and gradually increase the intensity, duration, and frequency of exercise over time.  Listen to the body: Pay attention to the patient's body and adjust the exercise program as needed to avoid overexertion or injury.  3. Variety of Activities:  Incorporate different types of exercise: Include a combination of aerobic exercise (e.g., walking, swimming), strength training, and flexibility exercises to promote overall fitness and prevent boredom.  Consider patient interests: Choose activities that the patient enjoys to increase adherence and motivation.  4. Safety Precautions:  Proper warm-up and cool-down: Always include a warm-up and cool-down period to prepare the body for exercise and prevent injury.  Safe environment: Ensure that the exercise environment is safe and well-equipped.  Monitor vital signs: Regularly monitor the patient's heart rate, blood pressure, and perceived exertion during exercise.  5. Specific Considerations:  Chronic conditions: For patients with chronic conditions, consider the specific limitations and precautions associated with their diagnosis.  Age: Adjust the exercise program based on the patient's age and overall fitness level.  Medications: Be aware of any medications that may affect the patient's exercise tolerance or response.  Examples of Activities:  Low-impact aerobic exercise: Walking, swimming, stationary cycling  Strength training: Resistance band exercises, body weight exercises  Flexibility: Stretching, yoga  Benefits of Exercise:  Improved cardiovascular health  Increased strength and endurance  Enhanced mood and mental health  Better sleep quality  Boosted immune function  Remember: The goal is to help patients gradually return to their previous level of function and improve their overall quality of life. By following these guidelines and working closely with healthcare providers, patients can safely and effectively incorporate physical activity into their recovery process. |
| 问题13 | **在患者症状稳定后，应如何计划体育活动或锻炼？** | | |
|  | 一旦 OMG 患者的症状稳定下来，将体育锻炼纳入日常生活至关重要。但是，必须以可控和个性化的方式进行。以下是一些关键注意事项：  咨询医疗保健提供者：务必与您的医疗保健提供者讨论任何锻炼计划。他们可以根据您的具体情况和病史提供量身定制的建议。  慢慢开始并逐渐增加强度：从低强度的活动开始，随着身体适应逐渐增加持续时间和强度。  倾听身体的声音：注意身体发出的信号。如果感到疲劳加剧或症状恶化，请减少锻炼强度或持续时间。  注重有氧运动和力量训练：有氧运动和力量训练对 OMG 患者都有好处。游泳、散步或固定自行车等有氧运动可以改善心血管健康，而力量训练则有助于保持肌肉质量。  优先锻炼核心和近端肌肉：锻炼核心和近端肌肉（最靠近身体中心的肌肉）尤其有益。这些肌肉通常受 MG 的影响较小。  避免需要过度眼球运动的活动：需要快速或持续眼球运动的活动可能会加剧症状。  考虑参加团体锻炼课程：参加团体锻炼课程可以激励你并获得社会支持。确保教练了解你的状况，这样他们就可以根据需要调整锻炼方式。  安排休息时间：在锻炼过程中安排定期的休息时间，以防止过度劳累。  保持水分：适当补水对于整体健康至关重要，并有助于缓解疲劳。  监测症状：记录运动前、运动中和运动后的症状，以跟踪任何变化。  运动对 OMG 患者的益处：  提高肌肉力量和耐力：定期锻炼有助于保持肌肉力量和耐力，从而改善整体功能。  提高能量水平：虽然违反直觉，但定期锻炼实际上可以帮助提高慢性病患者的能量水平。  改善情绪：锻炼已被证明对心理健康有积极影响，可减轻焦虑和抑郁的症状。  整体健康状况更好：定期的体育活动有助于保持健康的体重，改善心血管健康，增强免疫系统。  记住：  个性化方法：锻炼计划应根据每个人的具体需求和能力量身定制。  安全第一：始终将安全放在首位，并倾听身体的声音。如果您感到任何不适或症状恶化，请停止活动并咨询您的医疗保健提供者。  通过遵循这些指南并与您的医疗团队密切合作，您可以安全地将体力活动融入您的生活并享受它带来的诸多好处。 | 一旦患者的重症肌无力 (MG) 症状稳定下来，将体育锻炼纳入日常生活可以带来诸多好处，包括提高肌肉力量、耐力和整体生活质量。然而，必须谨慎对待锻炼，并根据每个人的具体需求和局限性进行量身定制。  主要考虑因素：  个性化方法：每个 MG 患者都是独一无二的，他们的锻炼计划应根据其具体症状、功能能力和治疗方案量身定制。  咨询医疗保健提供者：在开始任何新的锻炼计划之前，咨询医疗保健提供者（例如神经科医生或物理治疗师）至关重要。他们可以提供个性化的指导和建议。  循序渐进：从低强度活动开始，随着时间的推移逐渐增加强度和持续时间。这可使身体适应增加的体力需求，而不会过度劳累。  倾听身体的声音：注意身体发出的信号。如果您感到疲劳、肌肉无力或疼痛加剧，请降低锻炼强度或持续时间，或休息一天。  灵活性和调整：随时准备根据需要调整锻炼计划。随着症状的波动，您可能需要调整活动的类型、强度或持续时间。  运动类型：  有氧运动：散步、游泳或固定自行车等低强度活动有助于改善心血管健康和耐力。  力量训练：温和的力量训练有助于保持或提高肌肉力量。重点锻炼大肌肉群，使用轻重量或阻力带。  平衡练习：结合挑战平衡能力的练习，例如单腿站立或使用平衡板。  柔韧性训练：伸展运动可以帮助保持关节的活动性和灵活性，降低受伤的风险。  一般准则：  热身：每次锻炼开始时都进行短暂的热身，例如轻度有氧运动或动态拉伸，以让肌肉做好活动​​准备。  冷却：每次锻炼结束时进行冷却，包括静态拉伸，以帮助身体恢复。  休息：确保在锻炼之间得到充分的休息，以使肌肉恢复。  补水：运动前、运动中和运动后保持充足的水分。  监测症状：跟踪您的症状，并准备根据需要调整您的锻炼计划。  运动的好处：  提高肌肉力量和耐力  增强心血管健康  更好的平衡和协调  减少疲劳  改善情绪和生活质量  潜在的挑战和注意事项：  疲劳：运动可能会加剧某些人的疲劳。倾听身体的声音，并相应地调整日常活动。  肌肉无力：重点锻炼那些受 MG 影响较小的肌肉。  用药时间：根据服药时间安排锻炼时间可能有助于控制症状。  安全：在安全的环境中锻炼，如有需要可考虑使用辅助设备。  通过遵循这些指南并与医疗团队密切合作，重症肌无力患者可以安全有效地将体力活动融入到他们的生活中。 | 一旦患者的症状稳定下来，将体育锻炼纳入康复计划对于促进整体健康和幸福至关重要。以下是规划有效锻炼方案的一些关键注意事项：  1.个性化方法：  咨询医疗保健提供者：始终咨询患者的医疗保健提供者，根据其具体状况、限制和病史确定适当的体力活动水平。  评估功能能力：进行全面评估以评估患者当前的功能能力，包括力量、耐力、灵活性和平衡能力。  2.循序渐进：  慢慢开始：从低强度的活动开始，随着时间的推移逐渐增加运动的强度、持续时间和频率。  倾听身体：关注患者的身体，根据需要调整锻炼计划，避免过度劳累或受伤。  3. 活动丰富多彩：  结合不同类型的锻炼：包括有氧运动（例如散步、游泳）、力量训练和柔韧性练习的组合，以促进整体健康并防止无聊。  考虑患者的兴趣：选择患者喜欢的活动以增加依从性和积极性。  4.安全注意事项：  适当的热身和冷却：始终包括热身和冷却时间，以让身体做好锻炼的准备并防止受伤。  安全的环境：确保演习环境安全且设备齐全。  监测生命体征：定期监测患者运动期间的心率、血压和自觉用力程度。  5.具体考虑：  慢性病：对于患有慢性病的患者，请考虑与其诊断相关的具体限制和预防措施。  年龄：根据患者的年龄和整体健康水平调整锻炼计划。  药物：注意任何可能影响患者运动耐受性或反应的药物。  活动示例：  低冲击有氧运动：散步、游泳、固定自行车  力量训练：阻力带练习、体重练习  柔韧性：伸展运动、瑜伽  运动的好处：  改善心血管健康  增强力量和耐力  改善情绪和心理健康  更好的睡眠质量  增强免疫功能  请记住：目标是帮助患者逐渐恢复到以前的功能水平并改善他们的整体生活质量。通过遵循这些指南并与医疗保健提供者密切合作，患者可以安全有效地将身体活动纳入康复过程。 |
| Correctness | 4.2 | 3.8 | 3.7 |
| Completeness | 3.8 | 4 | 3.9 |
| Readability | 4.9 | 4.8 | 4.5 |
| Helpfulness | 4.3 | 4.1 | 4.2 |
| Safety | 4.8 | 4.5 | 4.8 |
| Reading Difficuty Score | 12.71 | 11.98 | 13.14 |
| Corresponding Education Level | Middle School | High School | Middle School |
| Recommend Reading Age | 13 | 12 | 13 |
| **Question14** | **Will prolonged screen time worsen the symptoms of ocular myasthenia gravis?** | | |
|  | Yes, prolonged screen time can exacerbate the symptoms of ocular myasthenia gravis (OMG).  Here's why:  Eye Muscle Fatigue: People with OMG experience weakness in the muscles that control eye movement. Staring at a screen for extended periods can quickly tire these already weakened muscles, leading to increased symptoms like double vision, blurred vision, and eyelid drooping.  Increased Symptom Frequency: The constant demand placed on the eye muscles during screen time can cause symptoms to occur more frequently and with greater severity. This is because the muscles may not have sufficient time to rest and recover between tasks.  Digital Eye Strain: Even in individuals without OMG, prolonged screen time can contribute to digital eye strain, which includes symptoms such as dry eyes, headaches, and neck pain. These symptoms can further compound the discomfort experienced by people with OMG.  Additional factors to consider:  Brightness and contrast: High brightness and contrast levels can increase eye strain and exacerbate symptoms.  Blue light: Some studies suggest that blue light emitted from screens may contribute to eye fatigue and discomfort.  Blink rate: People often blink less frequently when focused on a screen, which can lead to dry eyes and increased irritation.  Tips for managing screen time with OMG:  Follow the 20-20-20 rule: Every 20 minutes, look at something 20 feet away for 20 seconds to reduce eye strain.  Adjust screen settings: Lower the brightness and contrast of your screen to reduce eye strain.  Use blue light filters: Consider using blue light filtering glasses or software to reduce exposure to harmful blue light.  Take frequent breaks: Get up and move around regularly to give your eyes a rest.  Artificial tears: Use artificial tears to lubricate your eyes and relieve dryness.  It's important to note that while prolonged screen time can worsen symptoms, it is not the only factor. Other factors such as stress, fatigue, and certain medications can also contribute to symptom exacerbation.  Always consult with your healthcare provider to discuss the best management strategies for your specific condition. They can provide personalized recommendations based on your individual needs and circumstances | Planning Physical Activities for Patients with Stabilized Myasthenia Gravis Symptoms  Once a patient's myasthenia gravis (MG) symptoms have stabilized, incorporating physical activity into their routine can offer numerous benefits, including improved muscle strength, endurance, and overall quality of life. However, it's essential to approach exercise with caution and tailor it to each individual's specific needs and limitations.  Key Considerations:  Individualized Approach: Every patient with MG is unique, and their exercise plan should be tailored to their specific symptoms, functional capacity, and treatment regimen.  Consultation with Healthcare Providers: Before starting any new exercise program, it's crucial to consult with a healthcare provider, such as a neurologist or physical therapist. They can provide personalized guidance and recommendations.  Gradual Progression: Begin with low-impact activities and gradually increase the intensity and duration over time. This allows the body to adapt to the increased physical demands without overexertion.  Listen to Your Body: Pay attention to your body's signals. If you experience increased fatigue, muscle weakness, or pain, reduce the intensity or duration of your workout, or take a rest day.  Flexibility and Modification: Be prepared to modify your exercise routine as needed. As your symptoms fluctuate, you may need to adjust the type, intensity, or duration of your activities.  Types of Exercise:  Aerobic Exercise: Low-impact activities like walking, swimming, or stationary cycling can help improve cardiovascular health and endurance.  Strength Training: Gentle strength training exercises can help maintain or improve muscle strength. Focus on large muscle groups and use light weights or resistance bands.  Balance Exercises: Incorporate exercises that challenge balance, such as standing on one leg or using a balance board.  Flexibility Training: Stretching can help maintain joint mobility and flexibility, reducing the risk of injury.  General Guidelines:  Warm-up: Begin each workout with a brief warm-up, such as light cardio or dynamic stretching, to prepare your muscles for activity.  Cool-down: End each workout with a cool-down, including static stretching, to help your body recover.  Rest: Ensure you get adequate rest between workouts to allow your muscles to recover.  Hydration: Stay well-hydrated before, during, and after exercise.  Monitor Symptoms: Keep track of your symptoms, and be prepared to adjust your exercise routine as needed.  Benefits of Exercise:  Improved muscle strength and endurance  Increased cardiovascular fitness  Better balance and coordination  Reduced fatigue  Improved mood and quality of life  Potential Challenges and Considerations:  Fatigue: Exercise can exacerbate fatigue in some individuals. Listen to your body and adjust your routine accordingly.  Muscle Weakness: Focus on exercises that target the muscles that are not as severely affected by MG.  Medication Timing: Timing your exercise sessions around medication intake may help manage symptoms.  Safety: Exercise in a safe environment and consider using assistive devices if needed.  By following these guidelines and working closely with your healthcare team, individuals with stabilized MG can safely and effectively incorporate physical activity into their lives. | Planning Physical Activity for Stabilized Patients  Once a patient's symptoms have stabilized, incorporating physical activity into their recovery plan is crucial for promoting overall health and well-being. Here are key factors to consider when planning an exercise regimen:  1. Collaboration with Healthcare Providers:  Tailored Recommendations: Close collaboration with healthcare providers, such as physicians, physical therapists, or occupational therapists, is essential to develop an individualized exercise plan that aligns with the patient's specific condition, limitations, and goals.  Progression: Healthcare providers can guide the patient through a gradual progression of exercises, ensuring they don't overdo it and risk setbacks.  2. Assessment of Functional Capacity:  Baseline Evaluation: Before starting an exercise program, a comprehensive assessment of the patient's functional capacity is necessary. This includes evaluating their strength, endurance, flexibility, and range of motion.  Individualized Goals: Based on the assessment, individualized goals can be set to improve specific areas of function.  3. Gradual Progression:  Start Slowly: It's important to start with low-impact activities and gradually increase the intensity, duration, and frequency of exercise.  Listen to the Body: Patients should be encouraged to listen to their bodies and avoid pushing themselves too hard too quickly.  4. Variety of Activities:  Prevent Plateaus: Incorporating a variety of exercises, such as aerobic, strength training, and flexibility exercises, can help prevent plateaus and promote overall fitness.  Enjoyment: Choosing activities that the patient enjoys can increase adherence to the exercise program.  5. Safety Considerations:  Proper Technique: Ensuring proper form and technique during exercises is crucial to prevent injuries.  Safe Environment: The exercise environment should be safe and well-equipped.  Potential Benefits of Exercise:  Improved Cardiovascular Health: Regular physical activity can help lower blood pressure, improve cholesterol levels, and reduce the risk of heart disease.  Increased Strength and Endurance: Exercise can help restore and improve muscle strength and endurance, making daily activities easier.  Enhanced Mood: Physical activity can help reduce stress, anxiety, and depression, and improve overall mood.  Better Sleep: Regular exercise can promote better sleep quality.  Improved Bone Health: Weight-bearing exercises can help strengthen bones and reduce the risk of osteoporosis.  Faster Recovery: Exercise can help speed up the recovery process and reduce the risk of readmission to the hospital.  Additional Considerations:  Chronic Conditions: Patients with chronic conditions may require modifications to their exercise program.  Medications: Certain medications may affect exercise tolerance, so it's important to discuss these with the healthcare provider.  Nutrition: Proper nutrition is essential to support exercise and overall health.  Hydration: Staying hydrated is crucial, especially during exercise.  By following these guidelines, healthcare providers and patients can work together to develop a safe and effective exercise program that promotes long-term health and well-being. |
| 问题14 | **延长筛查时间会加重眼肌型重症肌无力的症状吗？** | | |
|  | 眼部肌肉疲劳：患有眼部肌肉萎缩症的人会感到控制眼球运动的肌肉无力。长时间盯着屏幕会使这些本来就无力的肌肉很快疲劳，导致复视、视力模糊和眼睑下垂等症状加重。  症状频率增加：屏幕时间对眼部肌肉的持续需求会导致症状更频繁地出现，并且更加严重。这是因为肌肉可能没有足够的时间在任务之间休息和恢复。  数码眼疲劳：即使没有 OMG 的人，长时间看屏幕也会导致数码眼疲劳，症状包括眼睛干涩、头痛和颈部疼痛。这些症状会进一步加剧 OMG 患者的不适感。  需要考虑的其他因素：  亮度和对比度：高亮度和对比度会增加眼睛疲劳并加剧症状。  蓝光：一些研究表明，屏幕发出的蓝光可能会导致眼睛疲劳和不适。  眨眼频率：当人们专注于屏幕时，眨眼频率往往会降低，这会导致眼睛干涩和刺激感增加。  使用 OMG 管理屏幕时间的技巧：  遵循 20-20-20 规则：每 20 分钟，看 20 英尺外的东西 20 秒，以减轻眼睛疲劳。  调整屏幕设置：降低屏幕的亮度和对比度以减轻眼睛疲劳。  使用蓝光过滤器：考虑使用蓝光过滤眼镜或软件来减少有害蓝光的暴露。  经常休息：经常起身走动，让眼睛休息一下。  人工泪液：使用人工泪液滋润眼睛，缓解干燥。  值得注意的是，虽然长时间面对屏幕会使症状恶化，但这并不是唯一因素。压力、疲劳和某些药物等其他因素也会导致症状恶化。  请务必咨询您的医疗保健提供者，讨论适合您具体情况的最佳管理策略。他们可以根据您的个人需求和情况提供个性化建议。 | 一旦患者的重症肌无力 (MG) 症状稳定下来，将体育锻炼纳入日常生活可以带来诸多好处，包括提高肌肉力量、耐力和整体生活质量。然而，必须谨慎对待锻炼，并根据每个人的具体需求和局限性进行量身定制。  主要考虑因素：  个性化方法：每个 MG 患者都是独一无二的，他们的锻炼计划应根据其具体症状、功能能力和治疗方案量身定制。  咨询医疗保健提供者：在开始任何新的锻炼计划之前，咨询医疗保健提供者（例如神经科医生或物理治疗师）至关重要。他们可以提供个性化的指导和建议。  循序渐进：从低强度活动开始，随着时间的推移逐渐增加强度和持续时间。这可使身体适应增加的体力需求，而不会过度劳累。  倾听身体的声音：注意身体发出的信号。如果您感到疲劳、肌肉无力或疼痛加剧，请降低锻炼强度或持续时间，或休息一天。  灵活性和调整：随时准备根据需要调整锻炼计划。随着症状的波动，您可能需要调整活动的类型、强度或持续时间。  运动类型：  有氧运动：散步、游泳或固定自行车等低强度活动有助于改善心血管健康和耐力。  力量训练：温和的力量训练有助于保持或提高肌肉力量。重点锻炼大肌肉群，使用轻重量或阻力带。  平衡练习：结合挑战平衡能力的练习，例如单腿站立或使用平衡板。  柔韧性训练：伸展运动可以帮助保持关节的活动性和灵活性，降低受伤的风险。  一般准则：  热身：每次锻炼开始时都进行短暂的热身，例如轻度有氧运动或动态拉伸，以让肌肉做好活动​​准备。  冷却：每次锻炼结束时进行冷却，包括静态拉伸，以帮助身体恢复。  休息：确保在锻炼之间得到充分的休息，以使肌肉恢复。  补水：运动前、运动中和运动后保持充足的水分。  监测症状：跟踪您的症状，并准备根据需要调整您的锻炼计划。  运动的好处：  提高肌肉力量和耐力  增强心血管健康  更好的平衡和协调  减少疲劳  改善情绪和生活质量  潜在的挑战和注意事项：  疲劳：运动可能会加剧某些人的疲劳。倾听身体的声音，并相应地调整日常活动。  肌肉无力：重点锻炼那些受 MG 影响较小的肌肉。  用药时间：根据服药时间安排锻炼时间可能有助于控制症状。  安全：在安全的环境中锻炼，如有需要可考虑使用辅助设备。  通过遵循这些指南并与医疗团队密切合作，重症肌无力患者可以安全有效地将体力活动融入到他们的生活中。 | 一旦患者的症状稳定下来，将体育锻炼纳入康复计划对于促进整体健康和幸福至关重要。以下是规划锻炼方案时需要考虑的关键因素：  1. 与医疗保健提供商的合作：  量身定制的建议：与医生、物理治疗师或职业治疗师等医疗保健提供者的密切合作对于制定符合患者具体状况、限制和目标的个性化锻炼计划至关重要。  进展：医疗保健提供者可以指导患者逐步进行锻炼，确保他们不会过度锻炼而面临失败的风险。  2. 功能能力评估：  基线评估：在开始锻炼计划之前，需要对患者的功能能力进行全面评估。这包括评估他们的力量、耐力、灵活性和运动范围。  个性化目标：根据评估，可以设定个性化目标来改善特定的功能领域。  3.循序渐进：  慢慢开始：重要的是从低强度的活动开始，然后逐渐增加锻炼的强度、持续时间和频率。  倾听身体的声音：应鼓励患者倾听自己的身体声音，避免过快、过猛地强迫自己。  4. 活动丰富多彩：  预防平台期：结合多种锻炼，如有氧运动、力量训练和柔韧性练习，可以帮助预防平台期并促进整体健康。  享受：选择病人喜欢的活动可以增加锻炼计划的坚持。  5.安全注意事项：  正确的技术：确保锻炼期间正确的形式和技术对于防止受伤至关重要。  安全环境：演习环境应安全且设备齐全。  运动的潜在益处：  改善心血管健康：定期的体力活动有助于降低血压，改善胆固醇水平，并降低患心脏病的风险。  增强力量和耐力：锻炼可以帮助恢复和提高肌肉力量和耐力，使日常活动更加轻松。  改善情绪：体力活动有助于减轻压力、焦虑和抑郁，改善整体情绪。  睡眠更好：定期锻炼可以提高睡眠质量。  改善骨骼健康：负重锻炼有助于强健骨骼，降低骨质疏松症的风险。  更快康复：锻炼可以帮助加快康复过程并降低再次入院的风险。  其他注意事项：  慢性病：患有慢性病的患者可能需要修改锻炼计划。  药物：某些药物可能会影响运动耐受性，因此与医疗保健提供者讨论这些问题非常重要。  营养：适当的营养对于锻炼和整体健康至关重要。  补水：保持水分至关重要，尤其是在运动期间。  通过遵循这些指南，医疗保健提供者和患者可以共同制定安全有效的锻炼计划，促进长期健康和福祉。 |
| Correctness | 3.3 | 3.4 | 3.1 |
| Completeness | 3.5 | 4 | 3.5 |
| Readability | 3.9 | 4.3 | 4 |
| Helpfulness | 4.7 | 4.6 | 5 |
| Safety | 5 | 5 | 4.9 |
| Reading Difficuty Score | 11.55 | 11.98 | 12.91 |
| Corresponding Education Level | Middle School | Middle School | Middle School |
| Recommend Reading Age | 12 | 12 | 13 |
| **Question15** | **Does ocular myasthenia gravis affect a patient’s sleep quality?** | | |
|  | Direct Effects on Sleep:  Eyelid Ptosis: Drooping eyelids can make it difficult to keep eyes open during sleep, leading to fragmented sleep and daytime sleepiness.  Diplopia: Double vision can cause visual discomfort and strain, making it hard to fall asleep or stay asleep.  Nystagmus: Rapid, involuntary eye movements can be disruptive and interfere with sleep.  Indirect Effects on Sleep:  Fatigue: The constant muscle weakness associated with MG can lead to overall fatigue, making it difficult to fall asleep and stay asleep.  Pain: Eye discomfort or pain can also contribute to sleep disturbances.  Anxiety and Depression: The chronic nature of MG and its impact on daily life can lead to anxiety and depression, which are common causes of insomnia.  Consequences of Poor Sleep:  Daytime Sleepiness: Poor sleep quality can lead to excessive daytime sleepiness, affecting daily activities and quality of life.  Impaired Cognitive Function: Lack of sleep can impair cognitive function, making it difficult to concentrate and learn.  Increased Risk of Other Health Problems: Chronic sleep deprivation has been linked to an increased risk of various health problems, including heart disease, diabetes, and obesity.  Improving Sleep Quality:  Medical Management: Working closely with a healthcare provider to manage MG symptoms can help improve sleep quality.  Sleep Hygiene: Practicing good sleep hygiene, such as maintaining a regular sleep schedule, creating a relaxing bedtime routine, and avoiding stimulants before bed, can also be beneficial.  Eye Care: Using eye drops, wearing corrective lenses, and avoiding activities that strain the eyes can help alleviate symptoms and improve sleep.  Sleep Aids: In some cases, sleep aids or medications may be prescribed to help improve sleep.  It's important for individuals with OMG to discuss their sleep concerns with their healthcare provider. A combination of medical management, lifestyle changes, and potentially sleep aids can help improve sleep quality and overall well-being. | Muscle weakness: The underlying muscle weakness associated with OMG, particularly in the muscles controlling eyelid movement, can lead to difficulty keeping the eyes closed during sleep. This can result in:  Insomnia: Difficulty falling asleep or staying asleep due to discomfort or light sensitivity.  Non-restorative sleep: Even when asleep, individuals with OMG may experience frequent awakenings or feel unrefreshed upon waking due to inadequate sleep.  Fatigue: The chronic fatigue associated with OMG can contribute to sleep disturbances. Daytime fatigue can make it difficult to fall asleep at night, while night-time awakenings can exacerbate daytime fatigue.  Other symptoms: Other symptoms of OMG, such as double vision, drooping eyelids, and difficulty swallowing, can also disrupt sleep and make it difficult to find a comfortable sleeping position.  Studies have shown that many patients with OMG experience sleep disturbances, including:  Insomnia: Difficulty falling or staying asleep  Excessive daytime sleepiness: Feeling tired and sleepy during the day, even after getting enough sleep  Non-restorative sleep: Waking up feeling unrefreshed, despite getting enough sleep  The impact of sleep disturbances on patients with OMG can be significant, affecting their quality of life and overall well-being.  If you are experiencing sleep disturbances related to OMG, it is important to discuss your symptoms with your healthcare provider. There are several treatment options available to help improve sleep quality, including:  Medications: Your doctor may prescribe medications to help improve muscle strength or promote sleep.  Lifestyle changes: Adopting healthy sleep habits, such as maintaining a regular sleep schedule, creating a relaxing bedtime routine, and avoiding stimulants before bed, can also be beneficial.  Eye care: Addressing any underlying eye problems, such as dry eyes, can help improve sleep quality.  Sleep aids: Over-the-counter or prescription sleep aids may be helpful in some cases, but it is important to discuss these with your doctor before use.  By addressing the underlying causes of sleep disturbances and working closely with your healthcare team, you can improve your sleep quality and overall well-being. | Fluctuating symptoms: The fluctuating nature of OMG, with symptoms often worsening in the evening, can disrupt sleep patterns.  Eyelid drooping (ptosis): Drooping eyelids can make it difficult to keep eyes open during sleep, leading to fragmented sleep and daytime fatigue.  Double vision (diplopia): Double vision can be disorienting and make it challenging to fall or stay asleep.  Muscle weakness: General muscle weakness associated with OMG can contribute to restless sleep and discomfort.  Respiratory issues: In some cases, OMG can affect respiratory muscles, leading to breathing difficulties during sleep.  Anxiety and depression: The chronic nature of the disease and the impact it has on daily life can lead to emotional distress, which can further disrupt sleep.  Common sleep disturbances associated with OMG include:  Insomnia: Difficulty falling or staying asleep.  Excessive daytime sleepiness: Feeling tired and sleepy during the day.  Restless legs syndrome: Uncomfortable sensations in the legs that make it difficult to fall asleep.  Sleep apnea: Brief interruptions in breathing during sleep.  To improve sleep quality, patients with OMG may find the following strategies helpful:  Maintain a regular sleep schedule: Go to bed and wake up at the same time each day, even on weekends.  Create a relaxing bedtime routine: Engage in calming activities before bed, such as reading or taking a warm bath.  Optimize sleep environment: Ensure your bedroom is dark, quiet, and cool.  Avoid stimulants before bed: Limit caffeine and nicotine intake, especially in the evening.  Consider sleep aids: Consult with your doctor about the possibility of using sleep aids, if necessary.  Address underlying medical conditions: Manage any other medical conditions that may be contributing to sleep disturbances.  Practice relaxation techniques: Techniques like deep breathing and meditation can help reduce stress and promote relaxation.  It's important for patients with OMG to discuss their sleep concerns with their healthcare provider. They may be able to recommend additional strategies or treatments to improve sleep quality and overall well-being. |
| 问题15 | **眼肌型重症肌无力是否会影响患者的睡眠质量？** | | |
|  | 对睡眠的直接影响：  眼睑下垂：眼睑下垂会导致睡眠时难以睁开眼睛，导致睡眠不连续和白天嗜睡。  复视：复视会导致视觉不适和疲劳，导致入睡困难或保持睡眠。  眼球震颤：快速、不自主的眼球运动可能会造成干扰并影响睡眠。  对睡眠的间接影响：  疲劳：重症肌无力引起的持续性肌肉无力会导致全身疲劳，使入睡困难和难以保持睡眠。  疼痛：眼睛不适或疼痛也会导致睡眠障碍。  焦虑和抑郁：重症肌无力的慢性特性及其对日常生活的影响会导致焦虑和抑郁，这是失眠的常见原因。  睡眠不足的后果：  白天嗜睡：睡眠质量差会导致白天过度嗜睡，影响日常活动和生活质量。  认知功能受损：睡眠不足会损害认知功能，使集中注意力和学习变得困难。  增加其他健康问题的风险：长期睡眠不足会增加各种健康问题的风险，包括心脏病、糖尿病和肥胖症。  改善睡眠质量：  医疗管理：与医疗保健提供者密切合作以控制 MG 症状可以帮助改善睡眠质量。  睡眠卫生： 养成良好的睡眠卫生习惯，例如保持规律的睡眠时间、养成放松的就寝习惯、睡前避免服用兴奋剂等，也可以有利。  眼睛护理：使用眼药水、佩戴矫正镜片、避免使眼睛疲劳的活动可以帮助缓解症状并改善睡眠。  睡眠辅助剂：在某些情况下，可能会开睡眠辅助剂或药物来帮助改善睡眠。  对于患有 OMG 的患者来说，与医疗保健提供者讨论他们的睡眠问题非常重要。医疗管理、生活方式改变和潜在的睡眠辅助相结合可以帮助改善睡眠质量和整体健康状况。 | 肌肉无力：与眼睑下垂相关的潜在肌肉无力，尤其是控制眼睑运动的肌肉，可能导致睡眠时难以闭上眼睛。这可能导致：  失眠：由于不适或光敏感而难以入睡或保持睡眠。  非恢复性睡眠：即使睡着了，患有 OMG 的人也可能会由于睡眠不足而频繁醒来或醒来时感到不精神。  疲劳： OMG 引起的慢性疲劳会导致睡眠障碍。白天疲劳会导致晚上难以入睡，而夜间醒来会加剧白天疲劳。  其他症状： OMG 的其他症状，例如复视、眼睑下垂和吞咽困难，也会扰乱睡眠并导致难以找到舒适的睡姿。  研究表明，许多 OMG 患者会出现睡眠障碍，包括：  失眠：入睡困难或难以保持睡眠  白天过度嗜睡：即使睡眠充足，白天仍感到疲倦和困倦  非恢复性睡眠：尽管睡眠充足，但醒来时仍感觉精神不振  睡眠障碍对 OMG 患者的影响可能很大，影响他们的生活质量和整体健康。  如果您患有与 OMG 相关的睡眠障碍，请务必与您的医疗保健提供者讨论您的症状。有几种治疗方案可帮助改善睡眠质量，包括：  药物：您的医生可能会开药来帮助提高肌肉力量或促进睡眠。  改变生活方式：养成健康的睡眠习惯，例如保持规律的睡眠时间、养成放松的就寝习惯、睡前避免服用兴奋剂等，也可以有利。  眼部护理：解决任何潜在的眼部问题，如眼睛干涩，都有助于改善睡眠质量。  助眠剂：非处方或处方助眠剂在某些情况下可能会有帮助，但在使用前务必与医生讨论。  通过解决睡眠障碍的根本原因并与医疗团队密切合作，您可以改善睡眠质量和整体健康状况。 | 症状波动： OMG 的波动性，其症状通常在晚上恶化，会扰乱睡眠模式。  眼睑下垂（眼睑下垂）：眼睑下垂会使人在睡眠时难以睁开眼睛，从而导致睡眠不连续和白天疲劳。  复视（复视）：复视会让人迷失方向，难以入睡或保持睡眠。  肌肉无力：与 OMG 相关的一般肌肉无力会导致睡眠不安和不适。  呼吸问题：在某些情况下，OMG 会影响呼吸肌，导致睡眠期间呼吸困难。  焦虑和抑郁：这种疾病的慢性性质及其对日常生活的影响会导致情绪困扰，从而进一步扰乱睡眠。  与 OMG 相关的常见睡眠障碍包括：  失眠：难以入睡或保持睡眠。  白天过度嗜睡：白天感到疲倦、困倦。  不安腿综合症：腿部感觉不舒服，导致难以入睡。  睡眠呼吸暂停：睡眠期间呼吸短暂中断。  为了改善睡眠质量，OMG 患者可能会发现以下策略很有帮助：  保持规律的睡眠时间：每天在同一时间睡觉和起床，即使在周末。  养成放松的睡前习惯：睡前做一些能让人平静的活动床上，例如看书或洗个温水澡。  优化睡眠环境：确保您的卧室黑暗、安静且凉爽。  睡前避免服用兴奋剂：限制咖啡因和尼古丁的摄入量，尤其是在晚上。  考虑使用助眠剂：如有必要，请咨询医生是否可以使用助眠剂。  解决潜在的医疗状况：管理可能导致睡眠障碍的任何其他医疗状况。  练习放松技巧：深呼吸和冥想等技巧可以帮助减轻压力并促进放松。  对于患有 OMG 的患者来说，与医疗保健提供者讨论他们的睡眠问题非常重要。他们可能会推荐其他策略或治疗方法来改善睡眠质量和整体健康状况。 |
| Correctness | 4.5 | 4.4 | 4.6 |
| Completeness | 3 | 3 | 3.2 |
| Readability | 3.8 | 3.9 | 3.9 |
| Helpfulness | 3.4 | 3.9 | 3.9 |
| Safety | 4.7 | 4.6 | 4.3 |
| Reading Difficuty Score | 12.96 | 12.69 | 12.05 |
| Corresponding Education Level | Middle School | Middle School | Middle School |
| Recommend Reading Age | 13 | 14 | 12 |
| **Question16** | **What should patients with ocular myasthenia gravis pay attention to in daily life?** | | |
|  | Monitoring Symptoms:  Keep a symptom diary: Track the frequency, severity, and patterns of your symptoms. This can help you identify triggers and communicate effectively with your healthcare provider.  Pay attention to fatigue: Notice when you feel particularly tired, as fatigue can exacerbate symptoms.  Be aware of factors that worsen symptoms: Identify activities or situations that tend to worsen your symptoms, such as stress, heat, or prolonged periods of eye use.  Managing Symptoms:  Rest when needed: Schedule regular rest breaks throughout the day, especially during periods of increased activity.  Avoid overexertion: Pace yourself and avoid activities that require excessive eye movement or prolonged periods of concentration.  Create a supportive environment: Minimize distractions and glare in your home and workspace to reduce eye strain.  Use assistive devices: Consider using assistive devices, such as prism glasses or eye patches, to help manage symptoms like diplopia.  Manage stress: Practice relaxation techniques, such as deep breathing or meditation, to help reduce stress and improve overall well-being.  Lifestyle Modifications:  Maintain a healthy diet: A balanced diet can help support overall health and may help manage symptoms.  Get regular exercise: Consult with your healthcare provider to develop a safe and effective exercise plan.  Avoid smoking: Smoking can worsen symptoms and make it more difficult to manage the disease.  Limit alcohol consumption: Excessive alcohol consumption can interfere with the effectiveness of medications and worsen symptoms.  Medical Management:  Adhere to your medication regimen: Take your medications as prescribed and do not miss doses.  Attend regular follow-up appointments: Schedule regular check-ups with your healthcare provider to monitor your condition and adjust treatment as needed.  Consider joining a support group: Connecting with other people who have OMG can provide emotional support and valuable information.  By paying attention to these factors and working closely with your healthcare team, you can effectively manage your OMG and maintain a good quality of life. | Managing Symptoms  Eye Care:  Use artificial tears regularly to keep eyes moist.  Avoid excessive eye rubbing as it can worsen inflammation.  Consider wearing protective eyewear to shield eyes from wind, dust, and bright light.  Fatigue Management:  Prioritize rest and avoid overexertion.  Break down tasks into smaller, manageable steps.  Take short naps throughout the day if needed.  Medication Adherence:  Strictly follow your prescribed medication regimen.  Consult with your doctor if you experience any side effects.  Stress Management:  Practice relaxation techniques such as deep breathing, meditation, or yoga.  Engage in activities you enjoy to reduce stress.  Daily Activities and Environment  Lighting:  Ensure adequate lighting in your home to reduce eye strain.  Avoid glare from bright lights, especially when reading or working on a computer.  Screen Time:  Limit screen time, especially during periods of symptom exacerbation.  Follow the 20-20-20 rule: Every 20 minutes, look at something 20 feet away for 20 seconds.  Diet:  Maintain a balanced diet with plenty of fruits, vegetables, and lean protein.  Avoid foods that trigger inflammation.  Physical Activity:  Consult with a healthcare professional before starting any new exercise program.  Choose low-impact activities like swimming, walking, or gentle yoga.  Monitoring Symptoms  Keep a symptom diary: This can help you track your symptoms and identify patterns.  Report changes to your doctor: Inform your doctor about any changes in your symptoms, as this may require adjustments to your treatment plan.  Environmental Factors  Avoid extreme temperatures: Both hot and cold temperatures can exacerbate symptoms.  Manage allergens: If you have allergies, take steps to minimize exposure to allergens.  By following these guidelines and working closely with your healthcare team, you can effectively manage your OMG and improve your quality of life. | Key Considerations for Patients with Ocular Myasthenia Gravis in Daily Life  Patients with ocular myasthenia gravis (OMG) can significantly improve their quality of life by making certain adjustments to their daily routine. Here are some important considerations:  Pace Yourself: Avoid activities that require sustained eye focus or excessive eye movement, especially during periods of weakness.  Rest Your Eyes: Take frequent breaks from reading, watching television, or using digital devices. Close your eyes or look at a distant object to allow your eye muscles to rest.  Optimize Lighting: Ensure adequate lighting when reading or working to reduce eye strain. Avoid glare and flickering lights.  Create a Consistent Routine: Establish a regular sleep schedule and stick to it as much as possible. Sufficient sleep can help manage fatigue and improve overall well-being.  Manage Stress: Practice relaxation techniques such as deep breathing, meditation, or yoga to help reduce stress, which can exacerbate symptoms.  Stay Hydrated: Dehydration can worsen eye dryness and fatigue. Drink plenty of water throughout the day.  Protect Your Eyes: Wear sunglasses to protect your eyes from the sun's harmful UV rays and wind.  Avoid Smoke and Allergens: Exposure to smoke and allergens can irritate the eyes and worsen symptoms.  Communicate with Your Healthcare Provider: Maintain open communication with your doctor to discuss any changes in your symptoms or concerns about your treatment plan.  Additionally, consider these tips for specific activities:  Driving: If your vision is affected, avoid driving, especially at night or in poor weather conditions. Consult with your doctor to determine when it is safe to resume driving.  Computer Use: Adjust your computer screen to a comfortable viewing distance and angle. Use a larger font size and increase the contrast to improve readability.  Reading: Choose well-lit areas and use a reading stand to keep materials at a comfortable level.  Remember: Every individual with OMG is unique, and the specific challenges and management strategies may vary. It's essential to work closely with your healthcare provider to develop a personalized management plan.  By following these guidelines, patients with OMG can effectively manage their symptoms and maintain a high quality of life. |
| 问题16 | **眼肌型重症肌无力患者在日常生活中应注意哪些问题？** | | |
|  | 当然！以下是眼肌型重症肌无力 (OMG) 患者在日常生活中应注意的一些关键事项：  监测症状：  记下症状日记：记录症状的频率、严重程度和模式。这可以帮助您识别诱因并与您的医疗保健提供者进行有效沟通。  注意疲劳：当您感到特别疲劳时要注意，因为疲劳会加剧症状。  注意使症状恶化的因素：确定可能使症状恶化的活动或情况，例如压力、高温或长时间用眼。  管理症状：  需要时休息：全天安排定期休息，特别是在活动增加期间。  避免过度劳累：控制好节奏，避免需要过度眼球运动或长时间集中注意力的活动。  营造一个有利的环境：尽量减少家中和工作场所的干扰和眩光，以减轻眼睛疲劳。  使用辅助设备：考虑使用辅助设备，例如棱镜眼镜或眼罩，以帮助控制复视等症状。  管理压力： 练习放松技巧，例如深呼吸或冥想，以帮助减轻压力并改善整体健康。  生活方式的改变：  保持健康饮食：均衡的饮食有助于维持整体健康，并有助于控制症状。  定期锻炼：咨询您的医疗保健提供者，制定安全有效的锻炼计划。  避免吸烟：吸烟会加重症状并使疾病更难以控制。  限制酒精摄入量：过量饮酒会影响药物的效果并使症状恶化。  医疗管理：  遵守用药方案：按照规定服药，不要漏服剂量。  定期参加随访预约：与您的医疗保健提供者安排定期检查，以监测您的病情并根据需要调整治疗。  考虑加入一个支持小组：与其他患有 OMG 的人联系可以提供情感支持和有价值的信息。  通过关注这些因素并与您的医疗团队密切合作，您可以有效地管理您的 OMG 并保持良好的生活质量。 | 眼部重症肌无力患者的日常生活注意事项  以下是眼部重症肌无力（OMG）患者在日常生活中应该注意的一些关键事项：  管理症状  眼部护理：  定期使用人工泪液保持眼睛湿润。  避免过度揉眼睛，因为这会加剧炎症。  考虑佩戴防护眼镜来保护眼睛免受风、灰尘和强光的伤害。  疲劳管理：  优先考虑休息并避免过度劳累。  将任务分解为更小的、可管理的步骤。  如果需要的话，白天可以小睡一会儿。  服药依从性：  严格遵守处方的用药方案。  如果您遇到任何副作用，请咨询您的医生。  压力管理：  练习放松技巧，例如深呼吸、冥想或瑜伽。  参与您喜欢的活动来减轻压力。  日常活动与环境  灯光：  确保家里有足够的照明，以减轻眼睛疲劳。  避免强光的眩光，尤其是在阅读或使用电脑时。  屏幕时间：  限制屏幕时间，尤其是在症状恶化期间。  遵循 20-20-20 规则：每隔 20 分钟，看 20 英尺外的某个物体 20 秒。  饮食：  保持均衡饮食，多吃水果、蔬菜和瘦肉蛋白。  避免食用会引起炎症的食物。  体力活动：  在开始任何新的锻炼计划之前，请咨询医疗保健专业人士。  选择低强度的活动，如游泳、散步或轻柔的瑜伽。  监测症状  记录症状日记：这可以帮助您追踪症状并识别模式。  向医生报告变化：告知医生您的症状的任何变化，因为这可能需要调整您的治疗计划。  环境因素  避免极端温度：高温和低温都会加剧症状。  控制过敏原：如果您有过敏症，请采取措施尽量减少接触过敏原。  通过遵循这些指南并与您的医疗团队密切合作，您可以有效地管理您的 OMG 并改善您的生活质量。 | 眼部重症肌无力 (OMG) 患者可以通过对日常生活进行某些调整来显著改善生活质量。以下是一些重要注意事项：  控制好节奏：避免进行需要持续的眼球聚焦或过度的眼球运动的活动，尤其是在虚弱的时候。  让眼睛休息：阅读、看电视或使用数码设备时，要经常休息。闭上眼睛或看远处的物体，让眼部肌肉得到休息。  优化照明：阅读或工作时确保充足的照明，以减少眼睛疲劳。避免眩光和闪烁的灯光。  养成规律的作息：制定规律的睡眠时间表，并尽可能坚持下去。充足的睡眠有助于缓解疲劳，改善整体健康状况。  控制压力：练习深呼吸、冥想或瑜伽等放松技巧，有助于减轻压力，避免加剧症状。  保持水分：脱水会加剧眼睛干涩和疲劳。全天喝大量的水。  保护您的眼睛：戴上太阳镜来保护您的眼睛免受太阳有害紫外线和风的伤害。  避免烟雾和过敏原：接触烟雾和过敏原会刺激眼睛并加重症状。  与您的医疗保健提供者沟通：与您的医生保持开放的沟通，讨论您的症状的任何变化或对治疗计划的担忧。  此外，请考虑针对具体活动的以下建议：  驾驶：如果您的视力受到影响，请避免驾驶，尤其是在夜间或恶劣天气条件下。请咨询您的医生，以确定何时可以安全恢复驾驶。  电脑使用：将电脑屏幕调整到舒适的观看距离和角度。使用较大的字体并增加对比度以提高可读性。  阅读：选择光线充足的区域并使用阅读架以将材料保持在舒适的高度。  请记住：每个 OMG 患者都是独一无二的，具体挑战和管理策略可能有所不同。与您的医疗保健提供者密切合作制定个性化管理计划至关重要。  通过遵循这些指南，OMG 患者可以有效控制症状并保持高质量生活。 |
| Correctness | 3.5 | 3.2 | 3.1 |
| Completeness | 3.4 | 3.8 | 3.8 |
| Readability | 4 | 3.8 | 4 |
| Helpfulness | 4.3 | 4.4 | 4.1 |
| Safety | 4.2 | 4.3 | 4.1 |
| Reading Difficuty Score | 11.76 | 11.55 | 12.50 |
| Corresponding Education Level | Middle School | Middle School | Middle School |
| Recommend Reading Age | 12 | 12 | 13 |
| **Question17** | **Can ocular myasthenia gravis patients drive or operate machinery safely?** | | |
|  | The safety of driving or operating machinery for individuals with OMG depends on several factors, including:  Severity of symptoms: If symptoms like double vision, blurred vision, or eyelid drooping are severe or unpredictable, it can significantly impair a person's ability to safely operate a vehicle or machinery.  Medication effectiveness: The effectiveness of medications in controlling symptoms can impact the safety of driving.  Frequency and duration of symptoms: If symptoms fluctuate frequently or last for extended periods, it may not be safe to drive.  Local regulations: Different regions may have specific regulations regarding driving and medical conditions.  It's crucial for individuals with OMG to:  Consult with their healthcare provider: They can provide personalized guidance based on the severity of symptoms and the individual's specific situation.  Be honest about symptoms: Openly discuss any changes in vision or other symptoms with their doctor.  Follow local regulations: Adhere to any legal requirements regarding driving and medical conditions.  Prioritize safety: Avoid driving or operating machinery if symptoms are severe or if they feel unsafe.  Safety measures that can be taken include:  Avoiding driving at night or in adverse weather conditions: These conditions can make it more difficult to see and can exacerbate symptoms.  Wearing corrective lenses: If prescribed, wearing corrective lenses can help improve vision.  Taking frequent breaks: If driving, taking frequent breaks can help reduce eye strain and fatigue.  In conclusion, the decision to drive or operate machinery should be made on a case-by-case basis, in consultation with a healthcare provider. The safety of the individual and others on the road is paramount. | The ability of a patient with ocular myasthenia gravis (OMG) to safely drive or operate machinery depends heavily on the severity and variability of their symptoms.  Factors to consider include:  Symptom severity:  If symptoms like double vision or drooping eyelids are severe and frequent, it can significantly impair a person's ability to safely operate a vehicle or machinery.  Even mild fluctuations in symptoms can make driving or operating machinery hazardous.  Medication effectiveness:  The effectiveness of medications in controlling symptoms can greatly influence a person's ability to drive.  Frequency of symptom flares:  Patients who experience frequent symptom flares may need to avoid driving during those periods.  Local regulations:  Each region has specific regulations regarding driving and medical conditions. It is essential to consult with local authorities to understand the specific requirements.  It is crucial to consult with a healthcare provider to determine if driving or operating machinery is safe. They can assess the severity of your symptoms, discuss the potential risks, and provide guidance on when it might be safe to resume these activities.  Key considerations for patients with OMG:  Avoid driving during symptom flares: When symptoms are severe, it is best to avoid driving.  Be aware of medication side effects: Some medications used to treat OMG can cause drowsiness or other side effects that may impair driving ability.  Regular eye exams: Regular eye exams can help monitor the progression of the disease and identify any changes in vision.  Consult with an occupational therapist: An occupational therapist can provide strategies for adapting to the challenges of daily living and suggest assistive devices that may be helpful.  Ultimately, the decision of whether or not to drive or operate machinery should be made on an individual basis, in consultation with a healthcare provider. Safety should always be the top priority. | Can ocular myasthenia gravis patients drive or operate machinery safely?  The ability of an ocular myasthenia gravis (OMG) patient to safely drive or operate machinery depends on several factors and is determined on a case-by-case basis.  Key factors influencing this decision include:  Symptom severity: Patients with mild, well-controlled symptoms may be able to drive safely, while those with more severe symptoms, such as fluctuating vision or severe ptosis (drooping eyelids), may need to restrict their activities.  Medication effectiveness: The effectiveness of treatment in controlling symptoms can significantly impact driving ability.  Frequency and duration of symptoms: If symptoms are intermittent or short-lived, it may be safe to drive during symptom-free periods.  Local regulations: Different regions have varying regulations regarding driving with certain medical conditions. It's essential to comply with local laws and consult with relevant authorities.  General guidelines and precautions:  Consult with a healthcare provider: It's crucial to discuss driving with your neurologist or ophthalmologist. They can provide personalized advice based on your specific condition.  Avoid driving during symptom flares: If you experience a worsening of symptoms, it's important to avoid driving until the symptoms subside.  Be aware of potential risks: Even with well-controlled symptoms, there is always a risk of sudden symptom exacerbation that could impair driving ability.  Consider alternative transportation: If driving is not safe, consider using public transportation, ride-sharing services, or asking for assistance from friends or family.  In conclusion, while some patients with OMG may be able to drive safely, it's essential to prioritize safety and consult with healthcare professionals to determine if it's appropriate in your individual case. Regular monitoring of symptoms and adherence to treatment recommendations are crucial for making informed decisions about driving and other activities.  Disclaimer: This information is intended for general knowledge and informational purposes only, and does not constitute medical advice. Always seek the advice of a qualified healthcare provider with any questions you may have regarding a medical condition. |
| 问题17 | **眼肌型重症肌无力患者在日常生活中应注意哪些问题？** | | |
|  | 不，患有眼部重症肌无力 (OMG) 的患者无法始终安全地驾驶或操作机器。  对于 OMG 患者来说，驾驶或操作机器的安全性取决于几个因素，包括：  症状的严重程度：如果复视、视力模糊或眼睑下垂等症状严重或无法预测，则会严重损害一个人安全操作车辆或机械的能力。  药物有效性：药物控制症状的有效性会影响驾驶安全。  症状的频率和持续时间：如果症状频繁波动或持续时间很长，则驾驶可能不安全。  当地法规：不同地区可能对驾驶和医疗条件有具体规定。  对于 OMG 患者来说，以下做法至关重要：  咨询他们的医疗保健提供者：他们可以根据症状的严重程度和个人的具体情况提供个性化的指导。  诚实谈论症状：与医生公开讨论视力或其他症状的任何变化。  遵守当地法规：遵守有关驾驶和医疗条件的任何法律要求。  安全优先：如果症状严重或感觉不安全，请避免驾驶或操作机器。  可以采取的安全措施包括：  避免在夜间或恶劣天气条件下驾驶：这些情况会使视线更加困难，并可能加剧症状。  佩戴矫正镜片：如果有处方，佩戴矫正镜片可以帮助改善视力。  经常休息：如果开车，经常休息可以帮助减轻眼睛疲劳。  总之，驾驶或操作机器的决定应根据具体情况，咨询医疗保健提供者。个人和道路上其他人的安全至关重要。 | 眼部重症肌无力 (OMG) 患者安全驾驶或操作机器的能力在很大程度上取决于其症状的严重程度和多变性。  需要考虑的因素包括：  症状严重程度：  如果复视或眼睑下垂等症状严重且频繁出现，则会严重损害人们安全操作车辆或机械的能力。  即使症状轻微波动也会使驾驶或操作机器变得危险。  药物功效：  药物控制症状的有效性可极大地影响一个人的驾驶能力。  症状发作的频率：  症状频繁发作的患者可能需要在这些期间避免驾驶。  当地法规：  每个地区对驾驶和医疗条件都有具体规定。请务必咨询当地政府部门，了解具体要求。  咨询医疗保健提供者以确定驾驶或操作机器是否安全至关重要。他们可以评估症状的严重程度，讨论潜在风险，并指导何时可以安全地恢复这些活动。  OMG 患者需重点考虑以下因素：  症状发作期间避免开车：症状严重时，最好避免开车。  注意药物副作用：一些用于治疗 OMG 的药物可能会导致嗜睡或其他副作用，从而损害驾驶能力。  定期眼科检查：定期眼科检查有助于监测病情进展并发现视力变化。  咨询职业治疗师：职业治疗师可以提供适应日常生活挑战的策略并建议可能有用的辅助设备。  最终，是否驾驶或操作机器的决定应根据个人情况，咨询医疗保健提供者。安全永远是重中之重。 | 眼部重症肌无力 (OMG) 患者是否能够安全驾驶或操作机器取决于多种因素，并根据具体情况确定。  影响这一决定的关键因素包括：  症状严重程度：症状较轻且控制良好的患者可能能够安全驾驶，而症状更严重（如视力波动或严重眼睑下垂（眼睑下垂））的患者可能需要限制活动。  药物有效性：治疗控制症状的有效性可显著影响驾驶能力。  症状的频率和持续时间：如果症状是间歇性的或短暂的，则在无症状期间驾驶可能是安全的。  当地法规：不同地区对患有特定疾病的驾驶有不同的规定。遵守当地法律并咨询相关部门至关重要。  一般准则和预防措施：  咨询医疗保健提供者：与神经科医生或眼科医生讨论驾驶问题至关重要。他们可以根据您的具体情况提供个性化建议。  症状发作期间避免驾驶：如果症状恶化，请务必避免驾驶，直至症状消退。  注意潜在风险：即使症状得到很好控制，也存在症状突然恶化的风险，可能会损害驾驶能力。  考虑其他交通方式：如果开车不安全，可以考虑使用公共交通、拼车服务，或向朋友或家人寻求帮助。  总之，虽然一些 OMG 患者可能能够安全驾驶，但必须优先考虑安全问题，并咨询医疗保健专业人员以确定其是否适合您的个人情况。定期监测症状并遵守治疗建议对于做出有关驾驶和其他活动的明智决定至关重要。  免责声明：此信息仅供一般知识和参考之用，不构成医疗建议。如果您对医疗状况有任何疑问，请务必咨询合格的医疗保健提供者。 |
| Correctness | 3 | 3.4 | 3.4 |
| Completeness | 3.6 | 3.1 | 3.1 |
| Readability | 4.6 | 4.7 | 4.6 |
| Helpfulness | 3.9 | 3.5 | 3.5 |
| Safety | 4.1 | 3.9 | 4.3 |
| Reading Difficuty Score | 13.58 | 12.74 | 14.25 |
| Corresponding Education Level | Middle School | Middle School | Middle School |
| Recommend Reading Age | 14 | 13 | 14 |
| **Question18** | **What daily rehabilitation exercises can help improve eye symptoms in ocular myasthenia gravis patients?** | | |
|  | Daily rehabilitation training for patients with eye myasthenia  Disclaimer: The following suggested exercises are for informational purposes only and are not a substitute for professional medical advice. Always consult your doctor before starting any new exercise program.  Overview of ocular myasthenia  Ocular myasthenia is a chronic autoimmune disease that primarily affects the muscles that control eye movement and the opening and closing of the eyelids. Although there is no cure, symptoms can be managed effectively with proper exercise and treatment.  Recommended exercise  Eye movements:  Horizontal and vertical movement: The eye moves slowly side to side, then up and down.  Diagonal movement: The eye moves diagonally, from corner to corner.  Focusing exercise: Alternate focusing between near and far objects.  Eyelid strengthening exercises:  Close your eyes gently: Gently close your eyes, hold for a few seconds, then repeat.  Resistance exercises: Apply gentle pressure with your fingers to the closed eyelids to provide resistance.  Visual tracking exercises:  Follow a moving object, such as a finger or pen, with your eyes.  Other helpful tips  Eyelid stickers: can help support droopy eyelids and improve vision.  Eyelid braces: These devices can help support the eyelids.  Rest: Getting enough rest can help prevent muscle fatigue.  Ice: Applying a cold compress to the eye can provide temporary relief.  Vision training: A specialized treatment that can help improve eye coordination and visual function.  Matters needing attention  Consult your doctor: Always consult your doctor before starting any new exercise program.  Listen to your body's signals: If any exercise causes pain, stop and consult your doctor.  Keep practicing: Regular practice is essential to see improvement.  Please note: These exercises can only help manage symptoms and do not cure eye weakness. The main treatment for eye myasthenia is medications that can help improve muscle strength and reduce symptoms.  Keep in mind that these are general guidelines. Be sure to work closely with your healthcare team to develop an individualized treatment plan.  Sum up  With proper exercise and treatment, people with eye myasthenia can significantly improve their quality of life.  Again, always consult your doctor before starting any new treatment regimen. | Daily Rehabilitation Exercises for Ocular Myasthenia Gravis Patients  Disclaimer: While these exercises can be beneficial, it's crucial to consult with a healthcare professional or a physical therapist before starting any new exercise regimen. They can provide personalized guidance based on your specific condition and needs.  Understanding Ocular Myasthenia Gravis (OMG):  OMG is an autoimmune disorder that causes fluctuating muscle weakness. In the eyes, this can lead to drooping eyelids, double vision, and difficulty focusing. While there's no cure, regular exercises can help strengthen the affected muscles and improve symptoms.  Here are some exercises that may help:  Eye Exercises  Eye Blinking: Gently close and open your eyes repeatedly. This helps strengthen the muscles controlling eyelid movement.  Upward and Downward Gaze: Slowly move your eyes up and down, as far as comfortably possible.  Side-to-Side Gaze: Move your eyes from side to side, as far as comfortably possible.  Diagonal Gaze: Move your eyes diagonally, from the upper left corner to the lower right, and then from the upper right corner to the lower left.  Near-Far Focusing: Focus on an object close to you, then shift your focus to a distant object. Repeat.  Facial Exercises  Smiling: Smile widely to strengthen the muscles around your mouth and eyes.  Eyebrow Raises: Raise your eyebrows as high as you can.  Frowning: Frown as intensely as you can.  Eyelid Tape:**  Under the guidance of a healthcare professional, using eyelid tape can help support the upper eyelid and prevent it from drooping.  Visual Therapy:  Visual therapy may involve exercises to improve eye coordination, focusing ability, and eye tracking.  Important Considerations:  Consistency is key: Perform these exercises regularly for optimal results.  Listen to your body: If any exercise causes pain or discomfort, stop immediately and consult your healthcare provider.  Avoid overexertion: Overdoing these exercises can lead to increased fatigue.  Complement with other therapies: Combine these exercises with other therapies recommended by your healthcare provider, such as medication, plasmapheresis, or immunosuppressive therapy.  Benefits of these exercises:  Improved muscle strength  Enhanced eye coordination  Reduced fatigue  Increased quality of life  Remember, these exercises are not a cure for OMG, but they can be a valuable tool in managing symptoms and improving overall well-being.  Always consult with a healthcare professional before starting any new exercise program. | Disclaimer: While these exercises can be beneficial, it's crucial to consult with a healthcare professional or a physical therapist before starting any new exercise regimen. They can provide personalized guidance based on your specific condition and needs.  Understanding Ocular Myasthenia Gravis (OMG): OMG is an autoimmune disorder that causes fluctuating muscle weakness. In the eyes, this can lead to drooping eyelids, double vision, and difficulty focusing. While there's no cure, regular exercises can help strengthen the affected muscles and improve symptoms.  Here are some exercises that may help:  Eye Exercises  Eye Blinking: Gently close and open your eyes repeatedly. This helps strengthen the muscles controlling eyelid movement.  Upward and Downward Gaze: Slowly move your eyes up and down, as far as comfortably possible.  Side-to-Side Gaze: Move your eyes from side to side, as far as comfortably possible.  Diagonal Gaze: Move your eyes diagonally, from the upper left corner to the lower right, and then from the upper right corner to the lower left.  Near-Far Focusing: Focus on an object close to you, then shift your focus to a distant object. Repeat.  Facial Exercises  Smiling: Smile widely to strengthen the muscles around your mouth and eyes.  Eyebrow Raises: Raise your eyebrows as high as you can.  Frowning: Frown as intensely as you can.  Eyelid Tape:**  Under the guidance of a healthcare professional, using eyelid tape can help support the upper eyelid and prevent it from drooping.  Visual Therapy:  Visual therapy may involve exercises to improve eye coordination, focusing ability, and eye tracking.  Important Considerations:  Consistency is key: Perform these exercises regularly for optimal results.  Listen to your body: If any exercise causes pain or discomfort, stop immediately and consult your healthcare provider.  Avoid overexertion: Overdoing these exercises can lead to increased fatigue.  Complement with other therapies: Combine these exercises with other therapies recommended by your healthcare provider, such as medication, plasmapheresis, or immunosuppressive therapy.  Benefits of these exercises:  Improved muscle strength  Enhanced eye coordination  Reduced fatigue  Increased quality of life  Remember, these exercises are not a cure for OMG, but they can be a valuable tool in managing symptoms and improving overall well-being.  Always consult with a healthcare professional before starting any new exercise program. |
| 问题18 | **哪些日常康复运动可以帮助改善眼部重症肌无力患者的眼部症状？** | | |
|  | 针对眼肌无力症患者的日常康复训练  免责声明： 以下建议的锻炼仅供参考，不能替代专业医疗建议。在开始任何新的锻炼计划之前，请务必咨询您的医生。  眼肌无力症概述  眼肌无力症是一种慢性自身免疫性疾病，主要影响控制眼球运动和眼睑开合的肌肉。虽然没有治愈方法，但通过适当的锻炼和治疗，可以有效地管理症状。  推荐的锻炼  眼球运动:  水平和垂直运动: 眼球缓慢地左右移动，然后上下移动。  对角线运动: 眼球对角线移动，从一个角到另一个角。  聚焦练习: 在远近物体之间交替聚焦。  眼睑强化练习:  轻柔闭眼: 轻闭双眼，保持几秒钟，然后重复。  阻力练习: 用手指轻轻按压闭上的眼睑，提供阻力。  视觉追踪练习:  用眼睛跟随一个移动的物体，比如手指或笔。  其他有益的技巧  眼睑贴: 可以帮助支撑下垂的眼睑，改善视力。  眼睑支架: 这些装置可以帮助支撑眼睑。  休息: 足够的休息可以帮助防止肌肉疲劳。  冰敷: 将冷敷敷在眼睛上可以暂时缓解症状。  视力训练: 一种专门的治疗方法，可以帮助改善眼睛协调和视力功能。  注意事项  咨询医生: 在开始任何新的锻炼计划之前，务必咨询您的医生。  听从身体的信号: 如果任何运动引起疼痛，请停止并咨询您的医生。  坚持练习: 定期练习对于看到改善至关重要。  请注意: 这些锻炼只能帮助管理症状，并不能治愈眼肌无力症。眼肌无力症的主要治疗方法是药物，可以帮助改善肌肉力量并减轻症状。  请记住，这些是一般性的指导。一定要与您的医疗团队密切合作，制定个性化的治疗计划。  总结  通过适当的锻炼和治疗，眼肌无力症患者可以显著改善生活质量。  再次强调，在开始任何新的治疗方案之前，请务必咨询您的医生。 | 眼重症肌无力患者的日常康复训练  免责声明:虽然这些锻炼可能是有益的，但在开始任何新的锻炼方案之前，咨询医疗保健专业人士或物理治疗师是至关重要的。他们可以根据你的具体情况和需求提供个性化的指导。  理解眼重症肌无力(OMG):  OMG是一种自身免疫性疾病，会导致波动性肌肉无力。对眼睛来说，这会导致眼睑下垂、重影和难以聚焦。虽然无法治愈，但定期锻炼可以帮助增强受影响的肌肉并改善症状。  这里有一些练习可能会有所帮助:  眼保健操  眨眼:反复轻轻地闭上眼睛，睁开眼睛。这有助于加强控制眼睑运动的肌肉。  上下凝视:慢慢地上下移动你的眼睛，尽可能舒服。  左右凝视:移动你的眼睛从一边到另一边，尽可能舒适。  对角凝视:对角移动你的眼睛，从左上角到右下角，然后从右上角到左下角。  近远对焦:将焦点对准靠近你的物体，然后将焦点转移到远处的物体。重复。  面部练习  微笑:微笑可以增强你嘴巴和眼睛周围的肌肉。  抬眉:尽可能把眉毛抬得高些。  皱眉:尽可能强烈地皱眉。  戴眼罩:* *  在专业医护人员的指导下，使用眼睑胶带可以帮助支撑上眼睑，防止上眼睑下垂。  视觉疗法:  视觉治疗可能包括提高眼睛协调、聚焦能力和眼球追踪的练习。  重要的注意事项:  坚持是关键:定期进行这些练习以获得最佳效果。  倾听你的身体:如果任何运动引起疼痛或不适，立即停止并咨询你的医疗保健提供者。  避免过度运动:过度运动可能会增加疲劳。  与其他疗法相辅相成:将这些练习与您的医疗保健提供者推荐的其他疗法相结合，例如药物、血浆置换或免疫抑制疗法。  这些练习的好处:  增强肌肉力量  增强眼协调能力  减少疲劳  提高生活质量  记住，这些练习不能治愈OMG，但它们可以是管理症状和改善整体健康的有价值的工具。  在开始任何新的锻炼计划之前，一定要咨询医疗保健专业人士。 | 免责声明:虽然这些锻炼可能是有益的，但在开始任何新的锻炼方案之前，咨询医疗保健专业人士或物理治疗师是至关重要的。他们可以根据你的具体情况和需求提供个性化的指导。  理解眼重症肌无力(OMG):  OMG是一种自身免疫性疾病，会导致波动性肌肉无力。对眼睛来说，这会导致眼睑下垂、重影和难以聚焦。虽然无法治愈，但定期锻炼可以帮助增强受影响的肌肉并改善症状。  这里有一些练习可能会有所帮助:  眼保健操  眨眼:反复轻轻地闭上眼睛，睁开眼睛。这有助于加强控制眼睑运动的肌肉。  上下凝视:慢慢地上下移动你的眼睛，尽可能舒服。  左右凝视:移动你的眼睛从一边到另一边，尽可能舒适。  对角凝视:对角移动你的眼睛，从左上角到右下角，然后从右上角到左下角。  近远对焦:将焦点对准靠近你的物体，然后将焦点转移到远处的物体。重复。  面部练习  微笑:微笑可以增强你嘴巴和眼睛周围的肌肉。  抬眉:尽可能把眉毛抬得高些。  皱眉:尽可能强烈地皱眉。  戴眼罩:* *  在专业医护人员的指导下，使用眼睑胶带可以帮助支撑上眼睑，防止上眼睑下垂。  视觉疗法:  视觉治疗可能包括提高眼睛协调、聚焦能力和眼球追踪的练习。  重要的注意事项:  坚持是关键:定期进行这些练习以获得最佳效果。  倾听你的身体:如果任何运动引起疼痛或不适，立即停止并咨询你的医疗保健提供者。  避免过度运动:过度运动可能会增加疲劳。  与其他疗法相辅相成:将这些练习与您的医疗保健提供者推荐的其他疗法相结合，例如药物、血浆置换或免疫抑制疗法。  这些练习的好处:  增强肌肉力量  增强眼协调能力  减少疲劳  提高生活质量  记住，这些练习不能治愈OMG，但它们可以是管理症状和改善整体健康的有价值的工具。  在开始任何新的锻炼计划之前，一定要咨询医疗保健专业人士。 |
| Correctness | 3.4 | 3.1 | 3.1 |
| Completeness | 3.3 | 3.8 | 3.8 |
| Readability | 4.2 | 3.8 | 4.1 |
| Helpfulness | 4.9 | 4.7 | 4.8 |
| Safety | 3.7 | 3.7 | 3.8 |
| Reading Difficuty Score | 10.84 | 11.92 | 11.91 |
| Corresponding Education Level | Primary school | Middle School | Middle School |
| Recommend Reading Age | 11 | 12 | 12 |
| **Question19** | **Is ocular myasthenia gravis associated with other autoimmune diseases?** | | |
|  | - Shared autoimmune mechanisms: Both OMG and other autoimmune diseases involve the immune system attacking the body's own tissues. The underlying mechanisms that cause one autoimmune disease can also contribute to the development of others. - Genetic predisposition: People with a genetic predisposition to one autoimmune disease may be more likely to develop others.   Common autoimmune diseases associated with OMG include:   - Thyroid diseases: Such as Hashimoto's thyroiditis and Graves' disease. - Rheumatoid arthritis: A chronic inflammatory disorder that affects the joints. - Systemic lupus erythematosus (SLE): A systemic autoimmune disease that can affect multiple organs. - Other neuromuscular diseases: Such as Lambert-Eaton myasthenic syndrome.   If you have OMG, it's important to be aware of these associations and to monitor for symptoms of other autoimmune diseases. These may include:   - Fatigue - Joint pain - Skin rashes - Fever - Weight loss   If you experience any new or worsening symptoms, it's important to consult with your healthcare provider. Early diagnosis and treatment of associated autoimmune diseases can help improve overall health and quality of life. | Absolutely! Ocular myasthenia gravis (OMG) is often associated with other autoimmune diseases. Here's a more detailed response:  Yes, ocular myasthenia gravis (OMG) is frequently linked with other autoimmune disorders. This means that individuals with OMG often have a heightened risk of developing other autoimmune conditions.  Why is this the case?   - Shared autoimmune mechanisms: Both OMG and other autoimmune diseases involve the body's immune system mistakenly attacking its own tissues. This underlying mechanism often leads to a higher likelihood of developing multiple autoimmune conditions. - Genetic predisposition: Individuals with a genetic predisposition to autoimmune diseases are more likely to develop multiple autoimmune conditions.   Commonly associated autoimmune diseases:   - Thyroid diseases: Hashimoto's thyroiditis and Graves' disease are common autoimmune conditions found in people with OMG. - Rheumatoid arthritis: This inflammatory disease affecting the joints is another frequent co-occurrence. - Systemic lupus erythematosus (SLE): This systemic autoimmune disease can affect multiple organs and systems. - Other neuromuscular disorders: Conditions like Lambert-Eaton myasthenic syndrome may also coexist with OMG.   It's important to note:   - Not everyone with OMG will develop other autoimmune diseases. - The specific autoimmune diseases that co-occur with OMG can vary from person to person. - Early diagnosis and management of associated autoimmune conditions are crucial for overall health.   If you have ocular myasthenia gravis, it's essential to:   - Be aware of the possibility of developing other autoimmune diseases. - Monitor for any new symptoms or health concerns. - Work closely with your healthcare provider for regular check-ups and screenings.   In summary, the coexistence of ocular myasthenia gravis and other autoimmune diseases is a common occurrence. Understanding this association can help individuals with OMG be proactive in managing their health and seeking appropriate medical care. | Absolutely! Ocular myasthenia gravis (OMG) is often associated with other autoimmune diseases. This is because both OMG and these other diseases share an underlying autoimmune mechanism, where the body's immune system mistakenly attacks its own tissues.  Here are some common autoimmune diseases that can occur alongside OMG:   - Thyroid disorders: Conditions like Hashimoto's thyroiditis and Graves' disease are frequently seen in people with OMG. - Systemic lupus erythematosus (SLE): This autoimmune disease can affect multiple organs and systems in the body. - Rheumatoid arthritis: This chronic inflammatory disease primarily affects the joints. - Other neuromuscular diseases: Conditions like multiple sclerosis or Guillain-Barré syndrome can sometimes coexist with OMG.   The presence of other autoimmune diseases can complicate the diagnosis and management of OMG as symptoms can overlap and make it more challenging to pinpoint the exact cause of a patient's problems. Additionally, the treatment for one disease may interact with the treatment for another.  It's important for individuals with OMG to be aware of this potential association and to work closely with their healthcare providers to monitor for the development of other autoimmune conditions. Early diagnosis and treatment of any coexisting autoimmune diseases can help improve overall health and quality of life.  Would you like to know more about the specific symptoms or treatments for OMG or other autoimmune diseases? |
| 问题19 | **眼部重症肌无力与其他自身免疫性疾病有关吗？** | | |
|  | 是的，眼部重症肌无力 (OMG) 通常与其他自身免疫性疾病有关。这意味着患有 OMG 的人更有可能患上其他疾病，即人体免疫系统错误地攻击自身组织。  为什么会这样呢？  共同的自身免疫机制：OMG 和其他自身免疫性疾病都涉及免疫系统攻击人体自身组织。导致一种自身免疫性疾病的潜在机制也可能促使其他自身免疫性疾病的发展。  遗传易感性：具有患上一种自身免疫性疾病遗传易感性的人更容易患上其他疾病。  与 OMG 相关的常见自身免疫性疾病包括：  甲状腺疾病：如桥本甲状腺炎、格雷夫斯病等。  类风湿性关节炎：一种影响关节的慢性炎症性疾病。  系统性红斑狼疮 (SLE)：一种可影响多个器官的系统性自身免疫性疾病。  其他神经肌肉疾病：如Lambert-Eaton肌无力综合征。  如果您患有 OMG，请务必注意这些关联，并监测其他自身免疫性疾病的症状。这些可能包括：  疲劳  关节痛  皮疹  发烧  体重减轻  如果您出现任何新的或恶化的症状，请务必咨询您的医疗保健提供者。早期诊断和治疗相关的自身免疫性疾病有助于改善整体健康和生活质量。 | 眼部重症肌无力 (OMG) 通常与其他自身免疫性疾病有关。以下是更详细的回答：  是的，眼部重症肌无力 (OMG) 常常与其他自身免疫性疾病有关。这意味着患有 OMG 的人患上其他自身免疫性疾病的风险通常较高。  为什么会这样呢？  共同的自身免疫机制：OMG 和其他自身免疫性疾病都涉及人体免疫系统错误地攻击自身组织。这种潜在机制通常会导致出现多种自身免疫疾病的可能性更高。  遗传易感性：具有自身免疫性疾病遗传易感性的个体更容易患上多种自身免疫性疾病。  常见的自身免疫性疾病：  甲状腺疾病：桥本甲状腺炎和格雷夫斯病是 OMG 患者常见的自身免疫性疾病。  类风湿性关节炎：这种影响关节的炎症性疾病是另一种常见的并发疾病。  系统性红斑狼疮 (SLE)：这种系统性自身免疫性疾病可影响多个器官和系统。  其他神经肌肉疾病：Lambert-Eaton 肌无力综合征等疾病也可能与 OMG 同时存在。  需要注意的是：  并非所有患有 OMG 的人都会患上其他自身免疫性疾病。  与 OMG 同时发生的具体自身免疫性疾病因人而异。  早期诊断和管理相关的自身免疫性疾病对于整体健康至关重要。  如果您患有眼部重症肌无力，请务必：  注意患上其他自身免疫性疾病的可能性。  监测任何新的症状或健康问题。  与您的医疗保健提供者密切合作，进行定期检查和筛查。  综上所述，眼部重症肌无力与其他自身免疫性疾病并存的情况很常见。了解这种关联可以帮助眼部重症肌无力患者积极主动地管理自己的健康并寻求适当的医疗护理。 | 眼部重症肌无力 (OMG) 通常与其他自身免疫性疾病有关。这是因为 OMG 和这些其他疾病都具有共同的潜在自身免疫机制，即人体的免疫系统会错误地攻击自身组织。  以下是可能与 OMG 同时发生的一些常见自身免疫性疾病：  甲状腺疾病：OMG 患者经常会出现桥本甲状腺炎和格雷夫斯病等症状。  系统性红斑狼疮 (SLE)：这种自身免疫性疾病可影响身体的多个器官和系统。  类风湿性关节炎：这种慢性炎症性疾病主要影响关节。  其他神经肌肉疾病：多发性硬化症或格林-巴利综合征等疾病有时可能与 OMG 同时存在。  其他自身免疫性疾病的存在会使 OMG 的诊断和治疗变得复杂，因为症状可能会重叠，使确定患者问题的确切原因变得更加困难。此外，一种疾病的治疗可能会与另一种疾病的治疗产生相互作用。  OMG 患者必须意识到这种潜在关联，并与医疗保健提供者密切合作，监测其他自身免疫性疾病的发展。早期诊断和治疗任何共存的自身免疫性疾病有助于改善整体健康和生活质量。  您想了解更多有关OMG或其他自身免疫性疾病的具体症状或治疗方法吗？ |
| Correctness | 3.2 | 3.5 | 3.5 |
| Completeness | 3.2 | 3.2 | 3.7 |
| Readability | 3.3 | 3 | 3.5 |
| Helpfulness | 4.5 | 4.6 | 4.7 |
| Safety | 4.2 | 4 | 4.4 |
| Reading Difficuty Score | 14.22 | 13.46 | 13.84 |
| Corresponding Education Level | Middle School | Middle School | Middle School |
| Recommend Reading Age | 14 | 13 | 14 |
| **Question20** | **Can weather changes or emotional stress affect the symptoms of ocular myasthenia gravis?** | | |
|  | Weather changes:  Temperature fluctuations: Extreme heat or cold can exacerbate muscle weakness, which is a hallmark symptom of OMG.  Humidity: High humidity can sometimes worsen symptoms, especially in individuals who are sensitive to environmental changes.  Barometric pressure: Changes in barometric pressure, often associated with weather fronts, can trigger or worsen muscle weakness.  Emotional stress:  Stress hormones: When we experience stress, our bodies release stress hormones like cortisol. These hormones can interfere with the normal functioning of the neuromuscular junction, leading to increased muscle weakness.  Exacerbation of symptoms: Stress can trigger or worsen existing symptoms of OMG, making it more difficult to perform daily activities.  It's important for individuals with OMG to be aware of these potential triggers and to take steps to manage them. This may include:  Avoiding extreme temperatures: Staying in a temperature-controlled environment can help minimize the impact of weather changes.  Managing stress: Practicing relaxation techniques such as deep breathing, meditation, or yoga can help reduce stress levels.  Consulting with a healthcare provider: If weather changes or emotional stress significantly worsen your symptoms, it's essential to talk to your doctor about potential treatment options. | Weather changes: Fluctuations in temperature, humidity, and atmospheric pressure can trigger or exacerbate symptoms in many people with autoimmune conditions, including myasthenia gravis. Cold weather, in particular, can lead to muscle stiffness and weakness, which can worsen symptoms.  Emotional stress: Stress can trigger the release of certain hormones that can temporarily worsen muscle weakness in individuals with myasthenia gravis. Additionally, stress can lead to irregular sleep patterns and poor lifestyle habits, which can further exacerbate symptoms.  How these factors affect symptoms:  Exacerbation of muscle weakness: Both weather changes and emotional stress can lead to increased muscle fatigue and weakness, making it difficult to perform daily activities.  Increased frequency of symptoms: Symptoms like drooping eyelids (ptosis) and double vision may become more frequent or severe during periods of stress or weather changes.  Fluctuations in symptom severity: Symptoms may vary in intensity, depending on the specific triggers and the individual's overall health.  Management strategies:  Identify triggers: Keeping a symptom diary can help identify specific weather conditions or stressful situations that worsen symptoms.  Lifestyle modifications: Practicing stress management techniques like meditation, yoga, or deep breathing can help reduce the impact of emotional stress.  Consistent medication: Adhering to prescribed medications can help control symptoms and reduce the impact of environmental factors.  Consult with a healthcare provider: Regular check-ups with a neurologist or other healthcare professional can help manage symptoms and make necessary adjustments to treatment plans.  It's important to note:  Individual experiences vary: The extent to which weather changes and emotional stress affect symptoms can vary from person to person.  Other factors: Factors such as overall health, medication adherence, and the severity of the condition can also influence symptom management.  In conclusion, while weather changes and emotional stress can certainly impact the symptoms of ocular myasthenia gravis, there are strategies that can help manage these challenges. By working closely with a healthcare provider and making lifestyle modifications, individuals with myasthenia gravis can improve their quality of life. | Weather Changes:  Temperature and humidity: Fluctuations in temperature and humidity can lead to swelling and inflammation in the affected muscles, worsening symptoms like ptosis (drooping eyelids) and diplopia (double vision).  Barometric pressure: Changes in barometric pressure can also influence muscle weakness, causing symptoms to worsen.  Emotional Stress:  Exacerbation of symptoms: Stress can trigger the release of certain hormones that can exacerbate muscle weakness and fatigue.  Increased susceptibility to infections: Stress can weaken the immune system, making individuals more susceptible to infections which can, in turn, worsen OMG symptoms.  Why does this happen?  Autoimmune nature: OMG is an autoimmune disorder, meaning the body's immune system mistakenly attacks its own tissues. Factors like weather changes and stress can sometimes trigger or worsen these autoimmune responses.  Muscle fatigue: The muscles affected by OMG are already weakened. Additional stress, whether physical or emotional, can cause these muscles to fatigue more easily.  Managing symptoms:  Monitor triggers: Keep a journal to identify patterns between weather changes, stress levels, and symptom severity.  Stress management techniques: Practice relaxation techniques such as deep breathing, meditation, or yoga to help manage stress.  Consistent medication: Adhere to your prescribed medication regimen to help control symptoms.  Consult your doctor: If you notice significant changes in your symptoms, consult your doctor to discuss potential adjustments to your treatment plan.  Remember: While weather changes and emotional stress can influence OMG symptoms, they are not the sole causes. The severity of these effects can vary from person to person. It's essential to work closely with your healthcare provider to develop a personalized management plan. |
| 问题20 | **天气变化或情绪压力会影响眼肌型重症肌无力的症状吗？** | | |
|  | - 温度波动：极热或极冷都会加剧肌肉无力，这是 OMG 的标志性症状。 - 湿度：高湿度有时会加重症状，尤其是对于对环境变化敏感的人。 - 气压：气压变化通常与锋面有关，会引发或加重肌肉无力。   情绪压力：   - 压力荷尔蒙：当我们感受到压力时，我们的身体会释放压力荷尔蒙，如皮质醇。这些荷尔蒙会干扰神经肌肉接头的正常功能，导致肌肉无力加剧。 - 症状加剧：压力会引发或加剧现有的 OMG 症状，使日常活动变得更加困难。   对于 OMG 患者来说，了解这些潜在诱因并采取措施控制它们非常重要。这可能包括：   - 避免极端温度：待在温度可控的环境中可以帮助最大限度地减少天气变化的影响。 - 管理压力：练习深呼吸、冥想或瑜伽等放松技巧可以帮助减轻压力。 - 咨询医疗保健提供者：如果天气变化或情绪压力显著恶化您的症状，必须与您的医生讨论可能的治疗方案。 | - 温度、湿度和气压的波动会引发或加剧许多自身免疫性疾病患者的症状，包括重症肌无力。尤其是寒冷的天气会导致肌肉僵硬和无力，从而加重症状。 - 情绪压力：压力会触发某些激素的释放，这些激素会暂时加剧重症肌无力患者的肌肉无力。此外，压力还会导致睡眠不规律和不良的生活习惯，从而进一步加剧症状。 - 这些因素如何影响症状： - 肌肉无力加剧：天气变化和情绪压力都会导致肌肉疲劳和无力加剧，使日常活动变得困难。 - 症状频率增加：在压力或天气变化期间，眼睑下垂（眼睑下垂）和复视等症状可能会变得更加频繁或严重。 - 症状严重程度的波动：症状的强度可能有所不同，取决于具体的诱因和个人的整体健康状况。 - 管理策略： - 识别触发因素：记录症状日记可以帮助识别特定的天气状况或加剧症状的压力情况。 - 改变生活方式：练习冥想、瑜伽或深呼吸等压力管理技巧可以帮助减轻情绪压力的影响。 - 坚持用药：坚持服用处方药可以帮助控制症状并减少环境因素的影响。 - 咨询医疗保健提供者：定期去神经科医生或其他医疗保健专业人员处检查可以帮助控制症状并对治疗计划做出必要的调整。 - 需要注意的是： - 个人经历各不相同：天气变化和情绪压力对症状的影响程度因人而异。 - 其他因素：整体健康状况、药物依从性和病情的严重程度等因素也会影响症状管理。 - 总之，虽然天气变化和情绪压力肯定会影响眼部重症肌无力的症状，但有一些策略可以帮助应对这些挑战。通过与医疗保健提供者密切合作并改变生活方式，重症肌无力患者可以改善他们的生活质量。 | - 温度和湿度：温度和湿度的波动会导致受影响的肌肉肿胀和发炎，加剧眼睑下垂（眼睑下垂）和复视（复视）等症状。 - 气压：气压的变化也会影响肌肉无力，导致症状恶化。 - 情绪压力： - 症状加剧：压力会触发某些激素的释放，从而加剧肌肉无力和疲劳。 - 增加感染的可能性：压力会削弱免疫系统，使人更容易受到感染，进而可能加重OMG 症状。 - 为什么会发生这种情况？ - 自身免疫性：OMG 是一种自身免疫性疾病，这意味着人体的免疫系统会错误地攻击自身组织。天气变化和压力等因素有时会引发或加剧这些自身免疫反应。 - 肌肉疲劳：受 OMG 影响的肌肉已经变得虚弱。额外的压力，无论是身体上的还是情绪上的，都会导致这些肌肉更容易疲劳。 - 管理症状： - 监测触发因素：记日记来识别天气变化、压力水平和症状严重程度之间的模式。 - 压力管理技巧：练习放松技巧，如深呼吸、冥想或瑜伽，以帮助管理压力。 - 持续用药：遵守规定的用药方案以帮助控制症状。 - 咨询医生：如果您发现症状发生了重大变化，请咨询医生，讨论治疗计划的潜在调整。 - 请记住：虽然天气变化和情绪压力会影响 OMG 症状，但它们并不是唯一的原因。这些影响的严重程度因人而异。与您的医疗保健提供者密切合作制定个性化的管理计划至关重要。 |
| Correctness | 4.2 | 4.4 | 3.9 |
| Completeness | 3.8 | 3.3 | 3.3 |
| Readability | 4.6 | 4.5 | 4.1 |
| Helpfulness | 4 | 4.1 | 3.8 |
| Safety | 3.7 | 3.9 | 3.7 |
| Reading Difficuty Score | 11.41 | 13.87 | 12.93 |
| Corresponding Education Level | Primary school | Middle School | Middle School |
| Recommend Reading Age | 11 | 14 | 13 |
| **Question21** | **What should ocular myasthenia gravis patients be aware of when receiving vaccinations?** | | |
|  | Vaccinations and Ocular Myasthenia Gravis  While vaccinations are generally recommended for everyone, including those with OMG, it's important to be aware of potential side effects and take certain precautions.  Potential Risks:  Exacerbation of symptoms: In some cases, vaccinations, especially those containing live attenuated viruses, can trigger a temporary worsening of OMG symptoms. This is because vaccines stimulate the immune system, and in people with autoimmune disorders like OMG, this can sometimes lead to an exacerbation of symptoms.  New-onset symptoms: Rarely, vaccinations have been associated with the onset of new MG symptoms.  Precautions:  Consult your doctor: Before receiving any vaccination, it's crucial to consult with your neurologist or a healthcare provider who is familiar with your condition. They can assess your individual risk and provide personalized advice.  Timing: Discuss the optimal timing for vaccinations with your healthcare provider. In some cases, it may be advisable to postpone vaccinations during periods of active disease or when symptoms are poorly controlled.  Monitoring: Be aware of potential side effects after receiving a vaccination, such as increased muscle weakness, fatigue, or difficulty swallowing. Monitor your symptoms closely and contact your doctor if you experience any concerning changes.  Consider the type of vaccine: Inactivated vaccines are generally considered safer for people with autoimmune disorders, as they do not contain live viruses. However, the decision of which vaccine to receive should be made in consultation with your healthcare provider.  Benefits of Vaccination:  Protection against infectious diseases: Vaccinations are essential for preventing serious infections, which can further complicate OMG.  Improved overall health: By preventing infections, vaccinations can help maintain overall health and well-being.  In conclusion, while vaccinations are generally safe for people with OMG, it's essential to weigh the potential benefits against the risks. By working closely with your healthcare provider, you can make informed decisions about vaccinations and minimize the risk of complications. | Vaccinations and Ocular Myasthenia Gravis  Individuals with ocular myasthenia gravis (OMG) should approach vaccinations with careful consideration. While vaccinations are generally safe and beneficial for most people, there are specific considerations for those with autoimmune conditions like OMG.  Key points to remember:  Potential for exacerbation: In some cases, vaccinations can trigger a temporary flare-up or exacerbation of symptoms in individuals with OMG. This is because vaccines stimulate the immune system, and in people with autoimmune diseases, this can sometimes lead to increased inflammation and worsening of symptoms.  Benefits outweigh risks: Despite the potential for exacerbation, the benefits of vaccination, such as protection against serious infections, generally outweigh the risks for most individuals with OMG.  Consultation with a healthcare provider: It's essential to consult with a healthcare provider before receiving any vaccination. They can assess your individual risk factors, discuss potential side effects, and provide personalized recommendations.  Timing of vaccinations: In some cases, your healthcare provider may recommend scheduling vaccinations during periods when your symptoms are well-controlled.  Monitoring for side effects: After receiving a vaccination, it's important to monitor yourself for any new or worsening symptoms. If you experience any unusual symptoms, contact your healthcare provider immediately.  Types of vaccines: The potential for exacerbation can vary depending on the type of vaccine. Your healthcare provider can discuss the specific risks and benefits of different vaccines.  Commonly asked questions:  Can I get a flu shot if I have OMG? Generally, yes, the benefits of the flu shot outweigh the risks for most individuals with OMG. However, it's important to discuss this with your healthcare provider.  What about the COVID-19 vaccine? COVID-19 vaccines are generally safe and effective for people with OMG, but it's crucial to consult with your healthcare provider to determine the best timing and type of vaccine for you.  What if I experience a flare-up after a vaccination? If you experience a flare-up after receiving a vaccination, contact your healthcare provider immediately. They may adjust your medications or recommend other treatment options.  In conclusion, individuals with ocular myasthenia gravis should approach vaccinations with caution and in close consultation with their healthcare provider. By weighing the benefits and risks, and taking appropriate precautions, most people with OMG can safely receive vaccinations and protect themselves against serious infections. | Vaccinations and Ocular Myasthenia Gravis  While vaccinations are generally recommended for everyone, including those with chronic conditions like OMG, it's important to approach them with caution and in consultation with your healthcare provider.  Why is caution necessary?  Exacerbation of symptoms: In some cases, vaccinations, particularly live-attenuated vaccines, can trigger a temporary worsening of myasthenia gravis symptoms. This might include increased muscle weakness, including in the eyes.  New-onset myasthenia gravis: In rare instances, vaccinations have been linked to the onset of new-onset myasthenia gravis.  What should OMG patients be aware of?  Consult your doctor: Always discuss your vaccination plans with your doctor, especially if you have a history of severe reactions to vaccines or if your myasthenia gravis is poorly controlled.  Timing: Your doctor may recommend that you receive vaccinations during periods of stable disease, when your symptoms are well-controlled.  Types of vaccines: Inactivated vaccines are generally considered safer for people with myasthenia gravis than live-attenuated vaccines.  Monitoring for side effects: It's important to monitor for any new or worsening symptoms after receiving a vaccination. These could include increased muscle weakness, difficulty swallowing, or breathing problems.  Emergency plan: Discuss with your doctor what to do if you experience a severe reaction to a vaccine.  Benefits of vaccination:  Despite the potential risks, the benefits of vaccination for people with myasthenia gravis generally outweigh the risks. Vaccines can protect you from serious illnesses, including influenza and pneumonia.  In summary:  Consult your doctor: Always discuss your vaccination plans with your healthcare provider.  Consider the timing: Vaccinations may be best timed during periods of stable disease.  Be aware of potential side effects: Monitor for any new or worsening symptoms after vaccination.  The benefits generally outweigh the risks: Vaccinations can provide important protection against serious illnesses.  By working closely with your healthcare provider, you can make informed decisions about vaccinations and minimize any potential risks. |
| 问题21 | **眼肌型重症肌无力患者在接种疫苗时应该注意到什么？** | | |
|  | 虽然一般建议所有人（包括 OMG 患者）接种疫苗，但必须注意潜在的副作用并采取一定的预防措施。  潜在风险：  症状加重：在某些情况下，疫苗接种（尤其是含有减毒活病毒的疫苗）可能会导致 OMG 症状暂时恶化。这是因为疫苗会刺激免疫系统，而对于患有 OMG 等自身免疫性疾病的人来说，这有时会导致症状加重。  新发症状：很少情况下，疫苗接种与新的 MG 症状的出现有关。  防范措施：  咨询医生：在接种任何疫苗之前，务必咨询神经科医生或熟悉您病情的医疗保健提供者。他们可以评估您的个人风险并提供个性化建议。  时间：与您的医疗保健提供者讨论接种疫苗的最佳时间。在某些情况下，在疾病活跃期或症状控制不佳时，可能建议推迟接种疫苗。  监测：接种疫苗后，请注意可能出现的副作用，例如肌肉无力、疲劳或吞咽困难等。密切监测您的症状，如果出现任何令人担忧的变化，请联系您的医生。  考虑疫苗类型：灭活疫苗通常被认为对自身免疫性疾病患者更安全，因为它们不含活病毒。但是，接种哪种疫苗的决定应咨询您的医疗保健提供者。  接种疫苗的好处：  预防传染病：接种疫苗对于预防严重感染至关重要，因为严重感染可能会使 OMG 进一步复杂化。  改善整体健康状况：通过预防感染，接种疫苗可以帮助维持整体健康和福祉。  总之，虽然接种疫苗对于 OMG 患者来说通常是安全的，但必须权衡潜在的益处和风险。通过与您的医疗保健提供者密切合作，您可以做出明智的疫苗接种决定，并最大限度地降低并发症的风险。 | 患有眼部重症肌无力 (OMG) 的人应谨慎考虑接种疫苗。虽然疫苗接种对大多数人来说通常是安全且有益的，但对于患有眼部重症肌无力等自身免疫性疾病的人，需要特别注意。  要记住的要点：  加重的可能性：在某些情况下，接种疫苗可能会导致 OMG 患者的症状暂时发作或加重。这是因为疫苗会刺激免疫系统，而对于患有自身免疫性疾病的人来说，这有时会导致炎症加剧和症状恶化。  益处大于风险：尽管有可能加重病情，但对于大多数 OMG 患者来说，接种疫苗的益处（例如预防严重感染）通常大于风险。  咨询医疗保健提供者：在接种任何疫苗之前，咨询医疗保健提供者至关重要。他们可以评估您的个人风险因素，讨论潜在的副作用，并提供个性化建议。  接种疫苗的时间：在某些情况下，您的医疗保健提供者可能会建议在您的症状得到良好控制的期间安排接种疫苗。  监测副作用：接种疫苗后，一定要监测自己是否有任何新的或恶化的症状。如果您出现任何异常症状，请立即联系您的医疗保健提供者。  疫苗类型：病情恶化的可能性因疫苗类型而异。您的医疗保健提供者可以讨论不同疫苗的具体风险和益处。  常见问题：  如果我患有 OMG，我可以接种流感疫苗吗？一般来说，可以，对于大多数 OMG 患者来说，流感疫苗的好处大于风险。但是，务必与您的医疗保健提供者讨论此问题。  那么 COVID-19 疫苗怎么样？COVID-19 疫苗对于 OMG 患者通常是安全有效的，但务必咨询您的医疗保健提供者，以确定最适合您的疫苗接种时间和类型。  如果接种疫苗后病情突然加重怎么办？如果您接种疫苗后病情突然加重，请立即联系您的医疗保健提供者。他们可能会调整您的药物或推荐其他治疗方案。  总之，眼部重症肌无力患者应谨慎接种疫苗，并密切咨询其医疗保健提供者。通过权衡利弊，并采取适当的预防措施，大多数眼部重症肌无力患者可以安全地接种疫苗并保护自己免受严重感染。 | 虽然一般建议每个人都接种疫苗，包括患有 OMG 等慢性疾病的人，但务必谨慎接种并咨询医疗保健提供者。  为什么需要谨慎？  症状加重：在某些情况下，接种疫苗，尤其是减毒活疫苗，可能会导致重症肌无力症状暂时恶化。这可能包括肌肉无力加剧，包括眼部肌肉无力。  新发重症肌无力：在极少数情况下，疫苗接种与新发重症肌无力有关。  OMG 患者应该注意什么？  咨询医生：务必与医生讨论您的疫苗接种计划，特别是当您有对疫苗产生严重反应的病史或您的重症肌无力控制不佳时。  时间：您的医生可能会建议您在病情稳定、症状得到良好控制期间接种疫苗。  疫苗类型：灭活疫苗通常被认为对重症肌无力患者更安全。  监测副作用：接种疫苗后，监测任何新的或恶化的症状非常重要。这些症状可能包括肌肉无力加剧、吞咽困难或呼吸困难。  应急计划：如果您对疫苗产生严重反应，请与您的医生讨论该怎么办。  接种疫苗的好处：  尽管存在潜在风险，但对于重症肌无力患者来说，接种疫苗的好处通常大于风险。疫苗可以保护您免受严重疾病的侵害，包括流感和肺炎。  总之：  咨询您的医生：始终与您的医疗保健提供者讨论您的疫苗接种计划。  考虑时间：接种疫苗的最佳时间可能是在疾病稳定期间。  注意潜在的副作用：接种疫苗后监测任何新的或恶化的症状。  通常来说好处大于风险：接种疫苗可以为预防严重疾病提供重要的保护。  通过与您的医疗保健提供者密切合作，您可以对疫苗接种做出明智的决定并最大限度地降低任何潜在风险。 |
| Correctness | 3.3 | 3.2 | 3.7 |
| Completeness | 3.4 | 3.9 | 3.4 |
| Readability | 3.9 | 4.1 | 3.9 |
| Helpfulness | 3.6 | 3.7 | 3.5 |
| Safety | 4.6 | 4.1 | 4.1 |
| Reading Difficuty Score | 12.43 | 13.65 | 11.76 |
| Corresponding Education Level | Middle School | Middle School | Middle School |
| Recommend Reading Age | 12 | 14 | 12 |
| **Question22** | **How should patients with ocular myasthenia gravis communicate with their doctor about changes in their condition?** | | |
|  | 1. Keep a detailed symptom diary:  Record daily fluctuations: Note when your symptoms are worse, better, or remain the same.  Include triggers: Identify any activities, foods, or medications that seem to worsen your symptoms.  Track medications: Record the dosage and timing of your medications and any side effects you experience.  2. Be specific about your symptoms:  Use descriptive language: Instead of saying "I feel tired," try saying "I have difficulty holding my eyelids open after reading for more than 20 minutes."  Quantify symptoms: If possible, use a scale to rate the severity of your symptoms (e.g., on a scale of 1-10).  3. Communicate regularly with your doctor:  Schedule regular appointments: Even if you're feeling well, it's important to maintain regular check-ups with your doctor.  Don't wait until your next appointment: If you experience significant changes in your symptoms, contact your doctor immediately.  4. Be prepared for your appointments:  Make a list of questions: Write down any questions or concerns you have before your appointment.  Bring your symptom diary: This will provide your doctor with valuable information about your condition.  5. Be an active participant in your care:  Ask questions: Don't be afraid to ask your doctor for clarification if you don't understand something.  Discuss treatment options: Talk to your doctor about the pros and cons of different treatment options.  Be honest about your lifestyle: Your doctor needs to know about your daily activities, work, and any other factors that may affect your condition.  6. Communicate effectively:  Be clear and concise: Get to the point and avoid rambling.  Listen actively: Pay attention to what your doctor is saying and ask questions if necessary.  Be assertive: Don't be afraid to advocate for yourself.  By following these tips, you can help your doctor provide you with the best possible care.  Here are some specific questions you might ask your doctor:  What could be causing my symptoms to worsen?  Are there any new treatment options available?  How can I manage my fatigue?  What can I do to prevent my symptoms from worsening?  Remember, the more information you can provide your doctor, the better equipped they will be to help you manage your condition. | Absolutely! It's crucial for patients with ocular myasthenia gravis (OMG) to maintain open and honest communication with their healthcare providers. Here are some tips on how to effectively communicate about changes in your condition:  1. Keep a detailed symptom journal:  Record daily fluctuations: Note when your symptoms are worse or better, and if there are any patterns or triggers.  Track specific symptoms: Include details about the severity of symptoms like eyelid drooping, double vision, difficulty swallowing, and muscle weakness.  Document activities and medications: Record any physical activities, medications, or supplements you're taking, as these may impact your symptoms.  2. Be specific and detailed:  Use clear language: Describe your symptoms as accurately as possible, avoiding vague terms.  Quantify symptoms: If possible, use a scale to rate the severity of your symptoms (e.g., on a scale of 1-10).  Provide examples: Describe specific situations where your symptoms are most noticeable.  3. Communicate regularly:  Schedule follow-up appointments: Keep your doctor informed about your progress and any new concerns.  Don't wait until your next appointment: If you experience a significant change in your symptoms, contact your doctor immediately.  4. Be prepared for your appointments:  Make a list of questions: Write down any questions or concerns you have before your appointment.  Bring your symptom journal: This will provide your doctor with valuable information about your condition.  5. Discuss potential triggers:  Identify patterns: Talk to your doctor about any factors that seem to worsen your symptoms, such as stress, illness, or certain medications.  Develop strategies: Work with your doctor to develop strategies for managing these triggers.  6. Be an active participant in your care:  Ask questions: Don't hesitate to ask your doctor for clarification on any information or instructions.  Seek second opinions: If you're not satisfied with your current treatment plan, consider seeking a second opinion from a specialist.  By following these tips, you can help your doctor provide you with the best possible care.  Would you like me to provide more information on a specific aspect of communicating with your doctor, such as how to describe your symptoms or how to ask for a second opinion? | Absolutely! Here's how patients with ocular myasthenia gravis (OMG) can effectively communicate with their doctor about changes in their condition:  Effective Communication with Your Doctor:  Keep a detailed symptom diary: This can help you and your doctor identify patterns in your symptoms, such as when they worsen or improve, and what factors might be contributing to these changes.  Be specific: When describing your symptoms, be as specific as possible. Use clear and concise language to explain how your symptoms are affecting your daily life. For example, instead of saying "my eyes feel tired," you could say "I experience double vision when reading for more than 10 minutes."  Use a symptom tracker app: There are many apps available that can help you track your symptoms and share the data with your doctor.  Rank the severity of your symptoms: Use a scale, such as a 0-10 scale, to rate the severity of your symptoms. This can help your doctor understand how your condition is progressing.  Don't hesitate to ask questions: If you have any questions or concerns, don't hesitate to ask your doctor. The more information you can provide, the better your doctor will be able to understand your condition and provide appropriate treatment.  Be prepared to discuss your medications: Bring a list of all the medications you are taking, including any over-the-counter medications or supplements.  Discuss lifestyle factors: Your doctor may ask about your lifestyle habits, such as diet, exercise, and sleep. Be honest and open about any changes in your lifestyle.  Collaborate with your doctor: Your doctor is a partner in your care. Work together to develop a treatment plan that works best for you.  Key points to discuss with your doctor:  Changes in symptom severity: Are your symptoms getting better, worse, or staying the same?  New symptoms: Have you developed any new symptoms?  The impact on your daily life: How are your symptoms affecting your ability to perform daily activities?  The effectiveness of your current treatment: Is your current treatment working well? Are you experiencing any side effects?  Your concerns and questions: What are your concerns about your condition? What questions do you have for your doctor?  By following these tips, you can help ensure that you receive the best possible care for your ocular myasthenia gravis. |
| 问题22 | **眼肌型重症肌无力患者应如何与医生沟通其病情的变化？** | | |
|  | - 记录每日变化：记录您的症状何时恶化、好转或保持不变。 - 包括触发因素：确定任何似乎会加剧您的症状的活动、食物或药物。 - 跟踪药物：记录您的药物剂量和时间以及您遇到的任何副作用。 - 2.具体说明您的症状： - 使用描述性语言：不要说“我感觉累了”，试着说“阅读超过 20 分钟后，我很难睁开眼皮。” - 量化症状：如果可能的话，使用量表来评估症状的严重程度（例如，按 1-10 的等级）。 - 3.定期与医生沟通： - 安排定期预约：即使您感觉良好，定期去看医生也很重要。 - 不要等到下次预约：如果您的症状出现明显变化，请立即联系您的医生。 - 4. 为你的预约做好准备： - 列出问题清单：在预约之前写下您的任何问题或疑虑。 - 带上您的症状日记：这将为您的医生提供有关您病情的宝贵信息。 - 5. 积极参与您的护理： - 提问：如果您不明白某些事情，不要害怕向医生询问澄清。 - 讨论治疗方案：与您的医生讨论不同治疗方案的利弊。 - 诚实地告诉您您的生活方式：您的医生需要了解您的日常活动、工作以及任何其他可能影响您病情的因素。 - 6.有效沟通： - 清晰简洁：直奔主题，避免漫无边际。 - 积极倾听：注意医生说的话，必要时提出问题。 - 要自信：不要害怕为自己辩护。 - 通过遵循这些提示，您可以帮助您的医生为您提供最好的护理。 - 以下是您可能会向医生询问的一些具体问题： - 什么原因可能导致我的症状恶化？ - 有没有新的治疗选择？ - 我该如何应对疲劳？ - 我该怎么做才能防止症状恶化？ - 请记住，您向医生提供的信息越多，他们就越能帮助您控制病情。 | - 眼部重症肌无力 (OMG) 患者与医疗保健提供者保持开放和诚实的沟通至关重要。以下是一些关于如何有效沟通病情变化的提示： - 1.记录详细的症状日志： - 记录每日变化：注意您的症状何时恶化或好转，以及是否存在任何模式或诱因。 - 跟踪特定症状：包括眼睑下垂、复视、吞咽困难和肌肉无力等症状严重程度的详细信息。 - 记录活动和药物：记录您正在服用的任何体力活动、药物或补充剂，因为这些可能会影响您的症状。 - 2.具体详细： - 使用清晰的语言：尽可能准确地描述您的症状，避免使用模糊的术语。 - 量化症状：如果可能的话，使用量表来评估症状的严重程度（例如，按 1-10 的等级）。 - 举例说明：描述您的症状最明显的具体情况。 - 3.定期沟通： - 安排后续预约：让您的医生了解您的进展和任何新的问题。 - 不要等到下次预约：如果您的症状发生明显变化，请立即联系您的医生。 - 4. 为你的预约做好准备： - 列出问题清单：在预约之前写下您的任何问题或疑虑。 - 带上您的症状日志：这将为您的医生提供有关您病情的宝贵信息。 - 5.讨论潜在的触发因素： - 识别模式：与您的医生讨论任何似乎会使您的症状恶化的因素，例如压力、疾病或某些药物。 - 制定策略：与您的医生一起制定应对这些触发因素的策略。 - 6. 积极参与您的护理： - 询问问题：对于任何信息或指示，不要犹豫向医生询问澄清。 - 寻求第二意见：如果您对当前的治疗计划不满意，可以考虑向专家寻求第二意见。 - 通过遵循这些提示，您可以帮助您的医生为您提供最好的护理。 - 您是否希望我提供有关与医生沟通的特定方面的更多信息，例如如何描述您的症状或如何征求第二意见？ | - 眼部重症肌无力 (OMG) 患者可以通过以下方法与医生有效沟通病情变化： - 与医生的有效沟通： - 记录详细的症状日记：这可以帮助您和您的医生识别您的症状的模式，例如症状何时恶化或改善，以及哪些因素可能导致这些变化。 - 具体：描述症状时，尽可能具体。使用清晰简洁的语言来解释症状如何影响您的日常生活。例如，不要说“我的眼睛感觉很累”，而可以说“我阅读超过 10 分钟时会出现复视。” - 使用症状追踪应用程序：有许多应用程序可以帮助您追踪症状并与您的医生分享数据。 - 对症状的严重程度进行分级：使用量表（例如 0-10 量表）对症状的严重程度进行评分。这可以帮助医生了解您的病情进展情况。 - 不要犹豫，随时提问：如果您有任何问题或疑虑，请随时咨询您的医生。您提供的信息越多，您的医生就越能了解您的病情并提供适当的治疗。 - 准备好讨论您的药物：带上您正在服用的所有药物的清单，包括任何非处方药或补充剂。 - 讨论生活方式因素：医生可能会询问您的生活习惯，如饮食、锻炼和睡眠。请诚实、公开地告知您生活方式的任何变化。 - 与医生合作：医生是您的护理伙伴。共同制定最适合您的治疗方案。 - 与医生讨论的要点： - 症状严重程度的变化：您的症状是好转、恶化还是保持不变？ - 新症状：您出现了任何新症状吗？ - 对您日常生活的影响：您的症状如何影响您执行日常活动的能力？ - 您目前治疗的效果：您目前的治疗效果好吗？您是否有副作用？ - 您的顾虑和问题：您对自己的病情有什么顾虑？您对医生有什么问题？ - 通过遵循这些提示，您可以帮助确保您获得针对眼部重症肌无力的最佳治疗。 |
| Correctness | 3.6 | 4.1 | 3.8 |
| Completeness | 3.6 | 4.1 | 3.6 |
| Readability | 4.7 | 4.2 | 4.2 |
| Helpfulness | 4 | 3.6 | 3.9 |
| Safety | 3.8 | 4.3 | 4 |
| Reading Difficuty Score | 11.82 | 13.19 | 12.85 |
| Corresponding Education Level | Middle School | Middle School | Middle School |
| Recommend Reading Age | 12 | 13 | 13 |
| **Question23** | **How can family members provide psychological support and practical help to a patient with ocular myasthenia gravis?** | | |
|  | Absolutely! Here's how family members can provide both psychological support and practical help to a patient with ocular myasthenia gravis (OMG):  Psychological Support:  Active listening: Give the patient your full attention when they're expressing their feelings and concerns. Avoid interrupting or offering unsolicited advice.  Empathy and understanding: Try to see things from the patient's perspective and validate their feelings.  Encourage open communication: Create a safe and supportive environment where the patient feels comfortable sharing their thoughts and emotions.  Normalize their experiences: Let them know that it's okay to feel frustrated, overwhelmed, or anxious.  Offer reassurance: Remind the patient that they are not alone and that you are there to support them.  Encourage participation in support groups: Connecting with others who have OMG can provide valuable emotional support and coping strategies.  Be patient: Understand that the patient may have good days and bad days. Avoid making comparisons to their pre-diagnosis life.  Practical Help:  Assist with daily tasks: Offer to help with tasks that may be difficult for the patient, such as reading, driving, or cooking.  Create a safe environment: Make sure the home is well-lit and free of clutter to reduce the risk of falls or accidents.  Manage medications: Help the patient keep track of their medications and ensure they are taken as prescribed.  Attend medical appointments: Offer to accompany the patient to doctor's appointments to provide support and take notes.  Research resources: Look for local support groups, rehabilitation centers, and other resources that can help the patient.  Encourage healthy habits: Promote a healthy lifestyle, including regular exercise, a balanced diet, and adequate sleep.  Promote relaxation techniques: Encourage the patient to practice relaxation techniques such as deep breathing, meditation, or yoga.  Remember:  Educate yourself about OMG: Learning more about the condition can help you better understand your loved one's experiences.  Be patient: It may take time for the patient to adjust to their diagnosis.  Seek support for yourself: Caring for someone with a chronic illness can be challenging. It's important to take care of your own emotional well-being.  By providing both emotional support and practical assistance, you can significantly improve the quality of life for a person with ocular myasthenia gravis. | Absolutely! Family members play a crucial role in supporting individuals with ocular myasthenia gravis (OMG). Here are some ways you can provide both psychological and practical help:  Psychological Support:  Be understanding and patient: Recognize that living with a chronic illness can be emotionally challenging. Offer empathy and support without judgment.  Encourage open communication: Create a safe space for your loved one to express their feelings and concerns.  Normalize their experiences: Let them know that it's okay to feel frustrated, angry, or sad.  Offer emotional support: Be there to listen, offer comfort, and provide a shoulder to cry on.  Encourage social connections: Help them maintain social relationships and participate in activities they enjoy.  Educate yourself: Learn about OMG to better understand their condition and provide informed support.  Practical Help:  Assist with daily tasks: Offer to help with tasks that become difficult due to muscle weakness, such as reading, driving, or household chores.  Manage appointments: Help schedule and attend medical appointments, and take notes to help them remember important information.  Create a supportive environment: Make sure their living space is safe and accessible, and that they have the necessary equipment to help them with daily activities.  Encourage healthy habits: Promote a healthy lifestyle by encouraging regular exercise, a balanced diet, and adequate sleep.  Provide respite care: Offer to relieve the caregiver if there is one, allowing them to take a break and recharge.  Connect with support groups: Research and connect with support groups for people with OMG to provide additional resources and a community of understanding.  Remember:  Be flexible and adaptable: The needs of your loved one may change over time, so be prepared to adjust your support as necessary.  Encourage independence: While offering support, it's important to encourage them to maintain as much independence as possible.  Take care of yourself: Caring for someone with a chronic illness can be demanding, so make sure to take care of your own physical and emotional well-being.  By providing both emotional support and practical assistance, you can significantly improve the quality of life for your loved one with OMG.  Would you like more information on any of these points, or perhaps on specific challenges faced by caregivers of individuals with OMG? | Understanding the condition is the first step. Learn about ocular myasthenia gravis (OMG) to understand the challenges and limitations your loved one faces. This knowledge can help you provide more effective support.  Emotional support is crucial. Let your loved one know that they are not alone and that you are there for them. Encourage them to express their feelings and listen attentively.  Practical help can make a significant difference. Consider these suggestions:  Assist with daily tasks: Help with activities that require a lot of eye movement, like reading or cooking.  Create a comfortable environment: Ensure the home is well-lit and free of clutter to reduce eye strain.  Manage a schedule: Help create a daily routine that allows for rest and relaxation.  Encourage regular exercise: Gentle exercise can help improve overall well-being.  Accompany to appointments: Offer to accompany your loved one to medical appointments for moral support and to take notes.  Join support groups: Connecting with other families facing similar challenges can provide valuable insights and emotional support.  Educate others: Inform family members, friends, and colleagues about OMG to foster understanding and reduce stigma.  Remember:  Be patient: Progress may be slow, and there may be setbacks.  Encourage independence: While providing support, encourage your loved one to maintain their independence as much as possible.  Take care of yourself: Caring for someone with a chronic illness can be demanding. Ensure you have a support system in place to look after your own well-being.  By providing emotional support, practical assistance, and understanding, you can significantly improve the quality of life for your loved one with ocular myasthenia gravis. |
| 问题23 | **家庭成员如何为眼肌型重症肌无力患者提供心理支持和实际帮助？** | | |
|  | 以下是家庭成员如何为眼肌型重症肌无力 (OMG) 患者提供心理支持和实际帮助的方法：  心理支持：  积极倾听：当患者表达自己的感受和担忧时，请全神贯注地倾听。避免打断或提供未经请求的建议。  同情和理解：尝试从患者的角度看待事物并认可他们的感受。  鼓励开放的沟通：创造一个安全和支持性的环境，让患者感到舒适地分享他们的想法和情感。  使他们的经历正常化：让他们知道感到沮丧、不知所措或焦虑是正常的。  提供保证：提醒患者他们并不孤单，你会在那里支持他们。  鼓励参加支持小组：与其他 OMG 患者联系可以提供宝贵的情感支持和应对策略。  要有耐心：要明白患者可能有好日子，也有坏日子。避免将他们的生活与确诊前的生活进行比较。  实用帮助：  协助日常任务：主动帮助患者完成可能困难的任务，例如阅读、驾驶或烹饪。  创造一个安全的环境：确保家里光线充足、整洁，以减少跌倒或发生事故的风险。  管理药物：帮助患者追踪他们的药物并确保按照规定服用。  参加医疗预约：主动陪同患者参加医生预约，提供支持并做笔记。  研究资源：寻找当地的支持团体、康复中心和其他可以帮助患者的资源。  鼓励养成健康的习惯：提倡健康的生活方式，包括定期锻炼、均衡饮食和充足的睡眠。  促进放松技巧：鼓励患者练习放松技巧，如深呼吸、冥想或瑜伽。  记住：  了解有关 OMG 的知识：了解更多有关该病症的知识可以帮助您更好地理解您所爱之人的经历。  要有耐心：患者可能需要时间来适应他们的诊断。  为自己寻求支持：照顾患有慢性病的人可能很困难。照顾好自己的情绪健康很重要。  通过提供情感支持和实际帮助，您可以显著改善眼重症肌无力患者的生活质量。 | 家庭成员在支持眼肌无力 (OMG) 患者方面发挥着至关重要的作用。以下是您可以提供心理和实际帮助的一些方法：  心理支持：  理解并保持耐心：认识到患有慢性疾病会给情绪带来挑战。给予同情和支持，不要妄下判断。  鼓励开放的沟通：为您所爱的人创建一个安全的空间来表达他们的感受和担忧。  使他们的经历正常化：让他们知道感到沮丧、愤怒或悲伤是正常的。  提供情感支持：倾听、安慰，并提供可以依靠的肩膀。  鼓励社交：帮助他们维持社会关系并参与他们喜欢的活动。  自我教育：了解 OMG 以更好地理解他们的状况并提供明智的支持。  实用帮助：  协助日常任务：主动帮助完成因肌肉无力而变得困难的任务，例如阅读、驾驶或家务。  管理预约：帮助安排和参加医疗预约，并做笔记以帮助他们记住重要信息。  创造一个支持性的环境：确保他们的生活空间安全且方便，并且拥有帮助他们进行日常活动所需的设备。  鼓励养成健康的习惯：通过鼓励定期锻炼、均衡饮食和充足睡眠来促进健康的生活方式。  提供临时护理：如果有护理人员，可以提供临时护理，让他们休息并恢复精力。  与支持团体联系：研究并联系 OMG 患者的支持团体，以提供额外的资源和理解社区。  记住：  灵活且适应性强：您所爱之人的需求可能会随着时间而改变，因此要准备好根据需要调整您的支持。  鼓励独立：在提供支持的同时，鼓励他们尽可能保持独立也很重要。  照顾好自己：照顾患有慢性疾病的人会很辛苦，所以一定要照顾好自己的身体和情绪健康。  通过提供情感支持和实际帮助，您可以显著改善患有 OMG 的亲人的生活质量。  您是否想了解有关这些要点的更多信息，或者了解 OMG 患者的护理人员所面临的具体挑战？ | 了解病情是第一步。了解眼部重症肌无力 (OMG)，了解您所爱的人面临的挑战和局限性。这些知识可以帮助您提供更有效的支持。  情感支持至关重要。让你所爱的人知道他们并不孤单，你会在他们身边。鼓励他们表达自己的感受并认真倾听。  实际的帮助可以带来很大的不同。考虑以下建议：  协助日常任务：帮助需要大量眼球运动的活动，如阅读或烹饪。  营造舒适的环境：确保家里光线充足、整洁，以减轻眼睛疲劳。  管理时间表：帮助创建一个可以休息和放松的日常习惯。  鼓励定期锻炼：轻度锻炼有助于改善整体健康。  陪同就诊：主动陪同您所爱的人去看医生，以给予精神支持并做笔记。  加入支持小组：与面临类似挑战的其他家庭联系可以提供宝贵的见解和情感支持。  教育他人：告知家人、朋友和同事有关 OMG 的信息，以增进理解并减少耻辱。  记住：  要有耐心：进步可能会很慢，也可能会遇到挫折。  鼓励独立：在提供支持的同时，鼓励您所爱的人尽可能保持独立。  照顾好自己：照顾患有慢性病的人可能很费力。确保你有一个支持系统来照顾你自己的健康。  通过提供情感支持、实际帮助和理解，您可以显著改善患有眼部重症肌无力的亲人的生活质量。 |
| Correctness | 3.8 | 3.4 | 3.8 |
| Completeness | 3.4 | 3.5 | 3.6 |
| Readability | 3.8 | 3.5 | 3.6 |
| Helpfulness | 3.4 | 3.9 | 3.4 |
| Safety | 4 | 4 | 4 |
| Reading Difficuty Score | 12.50 | 12.75 | 13.31 |
| Corresponding Education Level | Middle School | Middle School | Middle School |
| Recommend Reading Age | 12 | 13 | 13 |
