# Supplementary material for: Evaluating the Effectiveness of Large Language Models in Providing Patient Education for Chinese Patients With Ocular Myasthenia Gravis: Mixed Methods Study
Source: J Med Internet Res. 2025 Apr 10;27:e67883. doi: 10.2196/67883 (PMC12022522; doi:10.2196/67883)
Supplement: Multimedia Appendix 4 [file jmir_v27i1e67883_app4.docx]

| **Table S3: ERNIE 3.5 answers to 23 questions, raw scores in 5 areas and raw data for objective readability analysis** | | | |
| --- | --- | --- | --- |
| **Question1** | **What is ocular myasthenia gravis (OMG)?** | | |
|  | Ocular myasthenia gravis (OMG) is a specific type of myasthenia gravis (MG) that affects only the extraocular muscles, the levator palpebrae superioris muscle, and the orbicularis oculi muscle. MG, in general, is a chronic autoimmune disease characterized by the production of autoantibodies that target the neuromuscular junction, disrupting normal neuromuscular transmission and resulting in muscle weakness.  Here are some key points about OMG:  Prevalence and Presentation:  OMG accounts for approximately half of all MG cases.  It is often the initial manifestation of MG, with approximately 75% of patients experiencing ocular symptoms as their first symptom.  The most common symptoms include ptosis (drooping of the upper eyelid), diplopia (double vision or blurred vision due to eye misalignment), and ophthalmoplegia (paralysis of the eye muscles).  Diagnosis:  The diagnosis of OMG is primarily based on clinical presentation, often requiring a thorough neurological examination.  Ice test, rest test, Cogan eyelid twitch, and Bienfang test are some of the diagnostic tools that may aid in the diagnosis, but they have varying degrees of sensitivity and specificity.  Serological tests, such as detecting antibodies against the acetylcholine receptor (AChR), muscle-specific tyrosine kinase (MuSK), or low-density lipoprotein receptor-related protein 4 (LRP4), can be helpful but may not be sensitive in OMG patients.  Treatment:  The primary goal of treatment for OMG is to alleviate ocular symptoms and prevent or delay the progression to generalized myasthenia gravis (GMG).  Cholinesterase inhibitors, such as pyridostigmine, are commonly used to improve symptoms by increasing the availability of acetylcholine at the neuromuscular junction.  Corticosteroids, such as prednisone, are often prescribed in cases where cholinesterase inhibitors are ineffective or inadequate.  Immunosuppressive agents, such as azathioprine, mycophenolate mofetil, and rituximab, may be considered for patients who do not respond to corticosteroids or have significant side effects.  Thymectomy, the surgical removal of the thymus gland, may be beneficial for OMG patients with thymomas or those who do not respond to other treatments.  Challenges and Considerations:  OMG can be challenging to diagnose due to its similarity to other ocular conditions, such as thyroid-related eye disease or oculomotor nerve palsy.  Early and accurate diagnosis is crucial to improving patient quality of life and preventing the progression to GMG.  Close monitoring and individualized treatment plans are necessary to optimize outcomes and minimize adverse effects.  In summary, OMG is a specific subtype of MG that affects the eye muscles, requiring careful diagnosis and tailored treatment plans to manage symptoms and prevent disease progression. | Ocular Myasthenia Gravis (OMG) is a type of myasthenia gravis that primarily affects the muscles that control the movement of your eyes and eyelids. It's an autoimmune disorder, meaning your body's immune system mistakenly attacks its own healthy tissues.  Symptoms of OMG:  Double vision (diplopia)  Drooping eyelids (ptosis)  Difficulty focusing  Blurred vision  Sensitivity to light  Causes:  OMG occurs when the immune system produces antibodies that block the nerve signals to the muscles. This causes the muscles to become weak and easily fatigued.  Diagnosis:  Diagnosis is typically made through:  Physical examination: Checking for weakness in the eye muscles.  Blood tests: To detect specific antibodies.  Electromyography (EMG): To measure the electrical activity in the muscles.  Treatment:  There's no cure for OMG, but treatments can help manage symptoms. These may include:  Medications: To suppress the immune system or improve nerve-muscle communication.  Plasmapheresis: To remove harmful antibodies from the blood.  Thymectomy: Removal of the thymus gland, which plays a role in the immune system.  If you're experiencing any of the symptoms listed above, it's important to consult with a healthcare professional for a proper diagnosis and treatment plan.  Would you like to know more about a specific aspect of OMG, such as its impact on daily life, or the latest research in this area? | Ocular Myasthenia Gravis (OMG)  Ocular myasthenia gravis (OMG) is a type of myasthenia gravis that primarily affects the muscles around the eyes and eyelids. It is an autoimmune disorder, meaning your immune system mistakenly attacks and damages healthy tissues.  Symptoms of OMG include:  Double vision: Seeing two images of an object instead of one.  Drooping eyelids: Ptosis, where the upper eyelid sags.  Difficulty focusing: Trouble keeping your eyes in focus.  While OMG often begins with only eye muscle involvement, it can sometimes progress to a more generalized form of myasthenia gravis, affecting other muscles in the body.  If you experience any of these symptoms, it's important to consult with a healthcare professional for a diagnosis. Early detection and treatment can help manage the condition and improve quality of life. |
| 问题1 | **什么是眼部重症肌无力？** | | |
|  | 眼重症肌无力 （OMG） 是一种特殊类型的重症肌无力 （MG），仅影响眼外肌、上睑提肌和眼轮匝肌。一般来说，MG 是一种慢性自身免疫性疾病，其特征是产生靶向神经肌肉接头的自身抗体，破坏正常的神经肌肉传递并导致肌肉无力。  以下是有关 OMG 的一些关键点：  患病率和表现：  OMG 约占所有 MG 病例的一半。  它通常是 MG 的初始表现，大约 75% 的患者出现眼部症状作为他们的首发症状。  最常见的症状包括上睑下垂（上眼睑下垂）、复视（由于眼睛错位而导致的复视或视力模糊）和眼肌麻痹（眼肌麻痹）。  诊断：  OMG 的诊断主要基于临床表现，通常需要进行全面的神经系统检查。  冰试验、休息试验、Cogan 眼睑抽搐和边方试验是一些可能有助于诊断的诊断工具，但它们具有不同程度的敏感性和特异性。  血清学检测，例如检测乙酰胆碱受体 （AChR）、肌肉特异性酪氨酸激酶 （MuSK） 或低密度脂蛋白受体相关蛋白 4 （LRP4） 抗体，可能有帮助，但对 OMG 患者可能不敏感。  治疗：  OMG 治疗的主要目标是缓解眼部症状并预防或延缓进展为全身性重症肌无力 （GMG）。  胆碱酯酶抑制剂，如吡斯的明，通常用于通过增加神经肌肉接头处乙酰胆碱的可用性来改善症状。  皮质类固醇，如泼尼松，通常用于胆碱酯酶抑制剂无效或不足的情况下。  免疫抑制剂，例如硫唑嘌呤、吗替麦考酚酯和利妥昔单抗，可考虑用于对皮质类固醇无反应或有明显副作用的患者。  胸腺切除术，即手术切除胸腺，可能对患有胸腺瘤的 OMG 患者或对其他治疗没有反应的患者有益。  挑战和注意事项：  由于 OMG 与其他眼部疾病（例如甲状腺相关眼病或动眼神经麻痹）相似，因此可能难以诊断。  早期和准确的诊断对于改善患者的生活质量和防止进展为 GMG 至关重要。  密切监测和个体化治疗计划对于优化结果和最大限度地减少不良反应是必要的。  总之，OMG 是影响眼部肌肉的 MG 的一种特定亚型，需要仔细诊断和量身定制的治疗计划来控制症状并防止疾病进展。 | 眼部重症肌无力 (OMG)是一种重症肌无力，主要影响控制眼睛和眼睑运动的肌肉。这是一种自身免疫性疾病，意味着身体的免疫系统会错误地攻击自身的健康组织。  OMG 的症状：  复视  眼睑下垂（眼睑下垂）  难以集中注意力  视力模糊  对光敏感  原因：  当免疫系统产生抗体，阻断神经信号传送至肌肉时，就会发生肌肉萎缩性肌强直 (OMG)。这会导致肌肉变得虚弱，容易疲劳。  诊断：  诊断通常通过以下方式进行：  体格检查：检查眼部肌肉是否无力。  血液检查：检测特定抗体。  肌电图 (EMG)：测量肌肉中的电活动。  治疗：  OMG 无法治愈，但治疗可以帮助控制症状。这些可能包括：  药物：抑制免疫系统或改善神经肌肉交流。  血浆置换：从血液中去除有害抗体。  胸腺切除术：切除在免疫系统中发挥作用的胸腺。  如果您遇到上述任何症状，请务必咨询医疗保健专业人员，以获得正确的诊断和治疗方案。  您想了解有关 OMG 的某个特定方面的更多信息，例如它对日常生活的影响，或该领域的最新研究吗？ | 眼肌型重症肌无力（OMG） 是一种主要影响眼部肌肉的自身免疫性疾病。简单来说，就是你的身体免疫系统错误地攻击了控制眼部肌肉的神经连接，导致眼部肌肉无力。  OMG的主要症状包括：  复视: 看东西时出现重影。  眼睑下垂: 上眼皮无力下垂。  眼球运动困难: 难以聚焦或保持眼球固定。  虽然OMG通常只影响眼部肌肉，但有时也会发展成全身性的重症肌无力，影响身体的其他肌肉。  如果出现上述症状，建议及时就医，进行专业的诊断和治疗。  总结一下，OMG是一种眼部疾病，主要表现为眼部肌肉无力，影响视力。 |
| Correctness | 4.4 | 4.1 | 3.9 |
| Completeness | 3.7 | 4.2 | 4.2 |
| Readability | 4.2 | 4.2 | 3.7 |
| Helpfulness | 4.3 | 4.4 | 4.4 |
| Safety | 4.2 | 4.6 | 4.1 |
| Reading Difficuty Score | 15.28 | 9.94 | 10.00 |
| Corresponding Education Level | High school | Primary school | Primary school |
| Recommend Reading Age | 15 | 10 | 10 |
| **Question2** | **What are the typical symptoms of ocular myasthenia gravis?** | | |
|  | Ocular myasthenia gravis primarily affects the muscles that control your eyes and eyelids. Here are the typical symptoms:  Drooping eyelids (ptosis): This is often the first noticeable symptom.  Double vision (diplopia): This happens when your eyes can't align properly due to weak eye muscles.  Difficulty focusing: Your eyes may struggle to focus on objects.  These symptoms often:  Fluctuate: They may get better or worse throughout the day.  Worsen with use: Symptoms often become more noticeable after prolonged eye use.  If you experience any of these symptoms, it's important to see a healthcare professional. Early diagnosis and treatment can help manage the condition. | Ocular myasthenia gravis primarily affects the muscles that control the eyes and eyelids. Common symptoms include:  Drooping eyelids (ptosis): This is often the first symptom, causing one or both eyelids to sag.  Double vision (diplopia): This occurs when the eyes are misaligned, making it difficult to see clearly.  Difficulty focusing: The eyes may have trouble focusing on objects, especially at close range.  Eye pain or fatigue: Some people may experience discomfort or fatigue in their eyes.  Note: These symptoms can fluctuate throughout the day, often worsening with activity and improving with rest.  If you are experiencing these symptoms, it's important to consult a healthcare professional for a diagnosis. Early detection and treatment can help manage the condition and prevent complications. | Ocular myasthenia gravis primarily affects the muscles that control the eyes and eyelids. The most common symptoms include:  Double vision: Seeing two images of an object instead of one.  Drooping eyelids: Ptosis, a condition where one or both eyelids sag.  Difficulty focusing: The eyes may have trouble focusing on objects.  These symptoms often fluctuate throughout the day, becoming worse with activity and improving with rest. It's important to note that while ocular myasthenia gravis primarily affects the eyes, it can sometimes progress to a generalized form, involving other muscle groups.  If you're experiencing these symptoms, it's recommended to consult a healthcare professional for a proper diagnosis and treatment plan. |
| 问题2 | **眼肌型重症肌无力的典型症状是什么？** | | |
|  | 眼部重症肌无力主要影响控制眼睛和眼睑的肌肉。以下是典型症状：   - 眼睑下垂（眼睑下垂）：这通常是第一个明显的症状。 - 复视（复视）：由于眼部肌肉无力，双眼无法正确对准，就会发生这种情况。 - 聚焦困难：您的眼睛可能难以聚焦在物体上。   这些症状通常：   - 波动：一天内病情可能会变好，也可能会变坏。 - 使用时症状恶化：长时间用眼后症状通常会变得更加明显。   如果您出现任何这些症状，请务必去看医疗保健专业人员。早期诊断和治疗可以帮助控制病情。 | 眼部重症肌无力主要影响控制眼睛和眼睑的肌肉。常见症状包括：  眼睑下垂（眼睑下垂）：这通常是第一个症状，导致一只或两只眼睑下垂。  复视（复视）：当眼睛错位时就会发生这种情况，导致难以看清楚。  聚焦困难： 眼睛可能难以聚焦物体，尤其是在近距离的物体上。  眼睛疼痛或疲劳： 有些人可能会感到眼睛不适或疲劳。  注意： 这些症状可能全天波动，通常因活动而恶化，因休息而改善。  如果您出现这些症状，请务必咨询医疗保健专业人员进行诊断。 早期发现和治疗有助于控制病情并预防并发症 | 眼部重症肌无力主要影响控制眼睛和眼睑的肌肉。最常见的症状包括：  复视：看到一个物体的两个图像，而不是一个。  眼睑下垂：眼睑下垂，即一只或两只眼睑下垂的状况。  聚焦困难： 眼睛可能难以聚焦于物体。  这些症状通常全天波动，因活动而恶化，因休息而改善。 值得注意的是，虽然眼重症肌无力主要影响眼睛，但有时会发展为全身性症状，涉及其他肌肉群。  如果您出现这些症状，建议咨询医疗保健专业人员，以获得正确的诊断和治疗方案。 |
| Correctness | 3.3 | 3.7 | 3.3 |
| Completeness | 4.3 | 4 | 4.4 |
| Readability | 4.5 | 4 | 4 |
| Helpfulness | 3.6 | 3.6 | 3.6 |
| Safety | 4.4 | 4.1 | 4.5 |
| Reading Difficuty Score | 8.84 | 11.37 | 11.57 |
| Corresponding Education Level | Primary school | Primary school | Middle School |
| Recommend Reading Age | 9 | 11 | 12 |
| **Question3** | **What is the difference between ocular myasthenia gravis and generalized myasthenia gravis?** | | |
|  | Ocular myasthenia gravis and generalized myasthenia gravis are two distinct forms of myasthenia gravis, a chronic autoimmune disorder that affects the communication between nerve and muscle, particularly at the neuromuscular junction. Here are the key differences between the two:  Involvement of Muscles:  Ocular myasthenia gravis: As the name suggests, this form primarily affects the muscles around the eyes, particularly the extraocular muscles that control eye movements and eyelid position. Patients with ocular myasthenia gravis may experience drooping eyelids (ptosis), double vision (diplopia), and difficulty in moving the eyes up, down, or sideways.  Generalized myasthenia gravis: This form involves weakness of skeletal muscles throughout the body, not just the ocular muscles. It typically starts with ocular symptoms but gradually progresses to affect other muscles, including those in the face, neck, limbs, and even respiratory muscles. The muscle weakness is characterized by fatigue and worsening with activity, followed by improvement with rest.  Extent of Involvement:  Ocular myasthenia gravis: The involvement is limited to the ocular muscles, and patients may not experience systemic symptoms initially. However, in some cases, it can progress to generalized myasthenia gravis over time.  Generalized myasthenia gravis: The involvement is more widespread, affecting multiple muscle groups in the body. It has a greater potential for causing severe disability and even life-threatening complications, such as respiratory failure.  Treatment:  Both forms of myasthenia gravis are typically treated with medications that enhance neuromuscular transmission, such as anticholinesterase drugs (e.g., pyridostigmine). However, the management of generalized myasthenia gravis may require more aggressive treatment, including immunosuppressive therapies and, in some cases, surgery (thymectomy) to remove the thymus gland, which is often involved in the autoimmune process.  Prognosis:  Ocular myasthenia gravis: The prognosis is generally better for patients with ocular myasthenia gravis, as the involvement is limited and the disease progression may be slower. Many patients can manage their symptoms with medication and maintain good quality of life.  Generalized myasthenia gravis: The prognosis for generalized myasthenia gravis varies depending on the severity and extent of muscle involvement. Early diagnosis and treatment are crucial to prevent complications and improve long-term outcomes.  It's important to note that the distinction between ocular and generalized myasthenia gravis is not absolute, as some patients may experience a gradual progression from ocular to generalized symptoms. Additionally, the diagnosis of myasthenia gravis is often confirmed through a combination of clinical assessment, electrophysiological studies (e.g., nerve conduction studies, repetitive nerve stimulation tests), and laboratory tests (e.g., detection of autoantibodies). | The difference between ocular myasthenia gravis (OMG) and generalized myasthenia gravis (GMG) lies primarily in the extent and nature of muscle involvement. Here's a detailed comparison:  Muscle Involvement:  Ocular Myasthenia Gravis (OMG): As the name suggests, OMG is primarily limited to the ocular muscles, particularly the extraocular muscles that control eye movements and eyelid position. Patients with OMG may experience symptoms such as drooping eyelids (ptosis), double vision (diplopia), and difficulty moving the eyes in various directions.  Generalized Myasthenia Gravis (GMG): GMG involves skeletal muscle weakness throughout the body, not just the ocular muscles. It typically starts with ocular symptoms but progresses to affect other muscle groups, including facial, neck, limb, and even respiratory muscles. The muscle weakness is characterized by fatigue and worsening with activity, followed by improvement with rest.  Progression:  OMG: In some cases, OMG may remain localized to the ocular muscles and not progress to generalized involvement. However, about 80% of OMG patients eventually develop generalized symptoms within two years.  GMG: GMG involves a more widespread and progressive muscle weakness that can significantly impact daily functioning and quality of life. It requires more aggressive management to prevent complications and maintain adequate muscle strength.  Diagnosis:  Both OMG and GMG are diagnosed based on a combination of clinical assessment, electrophysiological studies (e.g., repetitive nerve stimulation, single-fiber electromyography), and laboratory tests (e.g., detection of autoantibodies such as acetylcholine receptor antibodies).  The presence of generalized muscle weakness and its characteristic pattern of fatigue and improvement with rest are key diagnostic features of GMG.  Treatment:  OMG and GMG: Both forms of myasthenia gravis are typically treated with medications that enhance neuromuscular transmission, such as anticholinesterase drugs (e.g., pyridostigmine).  GMG: In addition to anticholinesterase drugs, GMG patients may require immunosuppressive therapies (e.g., corticosteroids, immunosuppressants) to control the autoimmune process. In some cases, thymectomy (removal of the thymus gland) may be considered.  Prognosis:  OMG: The prognosis for OMG can be favorable if the disease remains localized to the ocular muscles. However, the risk of progression to GMG must be monitored.  GMG: The prognosis for GMG varies depending on the severity and extent of muscle involvement. Early diagnosis and aggressive management are crucial to prevent complications and improve long-term outcomes.  In summary, the key difference between OMG and GMG lies in the extent and nature of muscle involvement, with OMG being primarily limited to the ocular muscles and GMG involving skeletal muscle weakness throughout the body. The treatment and prognosis also differ, with GMG requiring more aggressive management to prevent serious complications. | The difference between ocular myasthenia gravis (OMG) and generalized myasthenia gravis (GMG) can be summarized as follows:  Affected Muscle Groups:  Ocular Myasthenia Gravis (OMG): As the name implies, OMG primarily affects the muscles around the eyes, including the extraocular muscles that control eye movements and the eyelid muscles. Symptoms such as ptosis (drooping eyelids), diplopia (double vision), and difficulty in eye movements are common in OMG.  Generalized Myasthenia Gravis (GMG): GMG involves skeletal muscle weakness throughout the body, including not just the ocular muscles but also facial, neck, limb, and even respiratory muscles. The muscle weakness is characterized by fatigue and worsening with activity, followed by improvement with rest.  Extent of Involvement:  OMG: OMG is localized to the ocular muscles and may remain so in some cases. However, approximately 80% of OMG patients eventually develop generalized symptoms within two years, progressing to GMG.  GMG: GMG involves a more widespread and progressive muscle weakness that can significantly impact daily functioning and quality of life.  Symptoms:  OMG: Symptoms are primarily ocular, such as ptosis, diplopia, and abnormal eye movements.  GMG: Symptoms include generalized skeletal muscle weakness, with fatigue and worsening symptoms after exertion. The muscle weakness can affect multiple muscle groups, including those involved in breathing, chewing, and swallowing.  Prognosis and Management:  OMG: The prognosis for OMG can be favorable if the disease remains localized to the ocular muscles. However, close monitoring is necessary for early detection of progression to GMG. Treatment typically involves anticholinesterase medications.  GMG: The prognosis for GMG varies depending on the severity and extent of muscle involvement. Early diagnosis and aggressive management, including the use of anticholinesterase medications and immunosuppressive therapies, are crucial to prevent complications and improve long-term outcomes. In some cases, thymectomy may also be considered.  Diagnostic Approach:  Both OMG and GMG are diagnosed based on a combination of clinical presentation, electrophysiological studies (e.g., repetitive nerve stimulation, single-fiber electromyography), and laboratory tests (e.g., autoantibody detection). However, the presence of generalized muscle weakness and its characteristic pattern of fatigue and improvement with rest are key diagnostic features of GMG.  In summary, the primary distinction between OMG and GMG lies in the extent and nature of muscle involvement, with OMG being primarily ocular and potentially progressing to GMG, while GMG involves generalized skeletal muscle weakness throughout the body. The management and prognosis of these two conditions also differ accordingly. |
| 问题3 | **眼肌型重症肌无力和全身性重症肌无力有什么区别？** | | |
|  | 眼重症肌无力和全身性重症肌无力是重症肌无力的两种不同形式，重症肌无力是一种慢性自身免疫性疾病，影响神经和肌肉之间的通讯，尤其是在神经肌肉接头处。以下是两者之间的主要区别：   1. 肌肉受累：    1. 重症眼肌无力：顾名思义，这种形式主要影响眼睛周围的肌肉，特别是控制眼球运动和眼睑位置的眼外肌。重症眼肌无力患者可能会出现眼睑下垂（上睑下垂）、复视（复视）以及眼睛难以上下或侧向移动。    2. 全身性重症肌无力：这种形式涉及全身骨骼肌无力，而不仅仅是眼肌。它通常从眼部症状开始，但逐渐发展到影响其他肌肉，包括面部、颈部、四肢甚至呼吸肌的肌肉。肌无力的特点是疲劳和活动时恶化，休息后改善。 2. 参与程度：    1. 重症眼肌无力：受累仅限于眼部肌肉，患者最初可能不会出现全身症状。然而，在某些情况下，随着时间的推移，它会发展为全身性重症肌无力。    2. 全身性重症肌无力：受累范围更广，影响身体的多个肌肉群。它更有可能导致严重残疾甚至危及生命的并发症，例如呼吸衰竭。 3. 治疗：    1. 两种形式的重症肌无力通常使用增强神经肌肉传递的药物治疗，例如抗胆碱酯酶药物（例如吡斯的明）。然而，全身性重症肌无力的治疗可能需要更积极的治疗，包括免疫抑制疗法，在某些情况下，还需要手术（胸腺切除术）来切除胸腺，胸腺通常参与自身免疫过程。 4. 预后：    1. 重症眼肌无力：重症眼肌无力患者的预后通常较好，因为受累有限且疾病进展可能较慢。许多患者可以通过药物控制症状并保持良好的生活质量。    2. 全身性重症肌无力： 全身性重症肌无力的预后取决于肌肉受累的严重程度和程度。早期诊断和治疗对于预防并发症和改善长期结果至关重要。   需要注意的是，眼部肌无力和全身性重症肌无力之间的区别并不是绝对的，因为一些患者可能会经历从眼部症状逐渐发展为全身症状。此外，重症肌无力的诊断通常通过临床评估、电生理学检查（例如神经传导检查、重复神经刺激试验）和实验室检查（例如自身抗体检测）相结合来确诊。 | 重症眼肌无力 （OMG） 和全身性重症肌无力 （GMG） 之间的区别主要在于肌肉受累的范围和性质。这是一个详细的比较：   1. 肌肉受累：    1. 重症眼肌无力 （OMG）：顾名思义，OMG 主要局限于眼肌，尤其是控制眼球运动和眼睑位置的眼外肌。OMG 患者可能会出现眼睑下垂（上睑下垂）、复视（复视）和眼睛难以向各个方向移动等症状。    2. 全身性重症肌无力 （GMG）：GMG 涉及全身骨骼肌无力，而不仅仅是眼肌。它通常从眼部症状开始，但会发展到影响其他肌肉群，包括面部、颈部、肢体，甚至呼吸肌。肌无力的特点是疲劳和活动时恶化，休息后改善。 2. 进展：    1. OMG：在某些情况下，OMG 可能仍然局限于眼部肌肉，不会发展为全身受累。然而，大约 80% 的 OMG 患者最终会在两年内出现全身症状。    2. GMG：GMG 涉及更广泛和进行性的肌肉无力，这会显着影响日常功能和生活质量。它需要更积极的管理，以防止并发症并保持足够的肌肉力量。 3. 诊断：    1. OMG 和 GMG 的诊断均基于临床评估、电生理学研究（例如，重复神经刺激、单纤维肌电图）和实验室检查（例如，检测乙酰胆碱受体抗体等自身抗体）的组合。    2. 全身性肌无力的存在及其特征性的疲劳和休息后改善是 GMG 的关键诊断特征。 4. 治疗：    1. OMG 和 GMG：两种形式的重症肌无力通常使用增强神经肌肉传递的药物治疗，例如抗胆碱酯酶药物（例如吡斯的明）。    2. GMG：除了抗胆碱酯酶药物外，GMG 患者可能需要免疫抑制疗法（例如皮质类固醇、免疫抑制剂）来控制自身免疫过程。在某些情况下，可以考虑胸腺切除术（切除胸腺）。 5. 预后：    1. OMG：如果疾病仍然局限于眼部肌肉，则 OMG 的预后可能是有利的。然而，必须监测进展为 GMG 的风险。    2. GMG：GMG 的预后取决于肌肉受累的严重程度和程度。早期诊断和积极管理对于预防并发症和改善长期结局至关重要。   总之，OMG 和 GMG 之间的主要区别在于肌肉受累的范围和性质，OMG 主要限于眼部肌肉，而 GMG 涉及全身骨骼肌无力。治疗和预后也不同，GMG 需要更积极的管理以防止严重的并发症。 | 眼重症肌无力 （OMG） 和全身性重症肌无力 （GMG） 之间的区别可以总结如下：   1. 受影响的肌肉群：    1. 重症眼肌无力 （OMG）：顾名思义，OMG 主要影响眼睛周围的肌肉，包括控制眼球运动的眼外肌和眼睑肌肉。上睑下垂（眼睑下垂）、复视（复视）和眼球运动困难等症状在 OMG 中很常见。    2. 全身性重症肌无力 （GMG）：GMG 涉及全身骨骼肌无力，不仅包括眼部肌肉，还包括面部、颈部、四肢甚至呼吸肌。肌无力的特点是疲劳和活动时恶化，休息后改善。 2. 参与程度：    1. OMG：OMG 位于眼肌，在某些情况下可能会保持如此。然而，大约 80% 的 OMG 患者最终在两年内出现全身症状，进展为 GMG。    2. GMG：GMG 涉及更广泛和进行性的肌肉无力，这会显着影响日常功能和生活质量。 3. 症状：    1. OMG：症状主要是眼部的，例如上睑下垂、复视和异常眼球运动。    2. GMG：症状包括全身骨骼肌无力、疲劳和劳累后症状恶化。肌无力会影响多个肌肉群，包括参与呼吸、咀嚼和吞咽的肌肉群。 4. 预后和管理：    1. OMG：如果疾病仍然局限于眼部肌肉，则 OMG 的预后可能是有利的。然而，密切监测对于早期发现进展为 GMG 是必要的。治疗通常包括抗胆碱酯酶药物。    2. GMG：GMG 的预后取决于肌肉受累的严重程度和程度。早期诊断和积极管理，包括使用抗胆碱酯酶药物和免疫抑制疗法，对于预防并发症和改善长期结局至关重要。在某些情况下，也可以考虑胸腺切除术。 5. 诊断方法：    1. OMG 和 GMG 的诊断均基于临床表现、电生理学检查（例如重复神经刺激、单纤维肌电图）和实验室检查（例如自身抗体检测）的组合。然而，全身性肌无力的存在及其特征性的疲劳和休息后改善是 GMG 的关键诊断特征。   总之，OMG 和 GMG 之间的主要区别在于肌肉受累的范围和性质，OMG 主要是眼部，并可能发展为 GMG，而 GMG 涉及全身全身骨骼肌无力。这两种情况的治疗和预后也相应地不同。 |
| Correctness | 3.3 | 3.3 | 3.7 |
| Completeness | 4.2 | 3.6 | 3.6 |
| Readability | 3.5 | 4 | 4 |
| Helpfulness | 4 | 3.9 | 3.9 |
| Safety | 4.1 | 4.5 | 4.4 |
| Reading Difficuty Score | 11.73 | 12.33 | 13.26 |
| Corresponding Education Level | Middle School | Middle School | Middle School |
| Recommend Reading Age | 12 | 12 | 13 |
| **Question4** | **How is ocular myasthenia gravis diagnosed?** | | |
|  | Ocular myasthenia gravis (OMG) is diagnosed through a combination of clinical examination, laboratory tests, and specialized diagnostic procedures. The diagnosis process typically involves the following steps:  Clinical Examination:  Symptom Assessment: The doctor will inquire about specific symptoms such as ptosis (drooping eyelid), diplopia (double vision), and eye movement abnormalities.  Physical Examination: The examination may include tests like the ice pack test, fatigue test, and Cogan's lid twitch sign, which help evaluate muscle weakness and its fluctuation. The doctor will observe for signs of muscle fatigue during sustained eye movements or upon repeated eyelid closure.  Specialized Diagnostic Tests:  Electrophysiology Tests: Repetitive nerve stimulation (RNS) and single-fiber electromyography (SFEMG) are used to evaluate neuromuscular transmission at the neuromuscular junction. RNS measures the decrement in muscle action potential amplitude after repeated nerve stimulation, which is characteristic of OMG. SFEMG provides a more sensitive measure of neuromuscular transmission abnormalities.  Autoantibody Testing: Blood tests are performed to detect serum antibodies specific to OMG, such as anti-acetylcholine receptor (AChR) antibodies and anti-muscle-specific tyrosine kinase (MuSK) antibodies. The presence of these antibodies supports the diagnosis of OMG.  Imaging Studies: Imaging techniques like computed tomography (CT) or magnetic resonance imaging (MRI) of the chest may be used to identify thymoma or thymic hyperplasia, which can be associated with OMG.  Pharmacological Testing:  Edrophonium (Tensilon) Test: This test involves the intravenous administration of edrophonium, a short-acting acetylcholinesterase inhibitor. If the patient's symptoms improve significantly after the injection, it suggests a positive diagnosis of OMG.  Differential Diagnosis:  Other conditions that can present with similar symptoms, such as thyroid eye disease, orbital myositis, and cranial nerve palsies, need to be ruled out.  In summary, the diagnosis of ocular myasthenia gravis is a comprehensive process that combines clinical examination, specialized diagnostic tests, autoantibody testing, imaging studies, and pharmacological testing. The goal is to confirm the diagnosis and differentiate it from other conditions that may mimic OMG. | Ocular myasthenia gravis (OMG) is diagnosed through a multi-step approach that involves clinical assessment, specialized diagnostic tests, and laboratory analysis. Here's a detailed breakdown of the diagnostic process:  Clinical Assessment:  The doctor will begin by taking a detailed history of the patient's symptoms, focusing on the presence of ptosis (drooping eyelids), diplopia (double vision), and difficulty with eye movements.  A physical examination is conducted to assess the strength of the eye muscles and observe for any signs of muscle fatigue during prolonged or repeated movements.  Tests like the ice pack test and the fatigue test may be performed to assess the variability in muscle strength over time, which is characteristic of OMG.  Specialized Diagnostic Tests:  Electrodiagnostic Tests:  Repetitive nerve stimulation (RNS): This test involves stimulating a nerve repeatedly and measuring the muscle response. In OMG, there is often a decremental response, indicating impaired neuromuscular transmission.  Single-fiber electromyography (SFEMG): This test is more sensitive than RNS and can detect subtle abnormalities in neuromuscular transmission.  Pharmacological Testing:  Edrophonium (Tensilon) test: An intravenous injection of edrophonium, a short-acting acetylcholinesterase inhibitor, is given. If symptoms improve significantly after the injection, it supports the diagnosis of OMG.  Laboratory Analysis:  Blood tests are performed to detect the presence of autoantibodies against the acetylcholine receptor (AChR) or muscle-specific tyrosine kinase (MuSK). These autoantibodies are frequently found in patients with OMG.  Imaging Studies:  Chest imaging (CT scan or MRI) may be performed to identify any thymic abnormalities, such as thymoma or thymic hyperplasia, which can be associated with OMG.  Differential Diagnosis:  Other conditions that can present with similar symptoms, such as thyroid eye disease, orbital myositis, cranial nerve palsies, or other neuromuscular disorders, must be ruled out.  In summary, the diagnosis of ocular myasthenia gravis is based on a comprehensive evaluation that includes clinical assessment, specialized diagnostic tests, laboratory analysis, imaging studies, and careful consideration of the differential diagnosis. The goal is to confirm the presence of OMG and exclude other potential causes of the patient's symptoms. | Ocular myasthenia gravis (OMG) is diagnosed through a combination of clinical assessment, specialized tests, and laboratory analysis. Here's a step-by-step outline of the diagnostic process:  Clinical Assessment:  Symptom Review: The healthcare provider will begin by reviewing the patient's symptoms, particularly the presence of ptosis (drooping eyelids), diplopia (double vision), and difficulties with eye movements. These are the hallmark symptoms of OMG.  Physical Examination: A thorough physical examination will be conducted, focusing on the eyelids and eye movements. The healthcare provider will test the muscles when they are fatigued and when they have rested to observe any changes in strength.  Specialized Tests:  Edrophonium (Tensilon) Test: This test involves administering a short-acting acetylcholinesterase inhibitor intravenously. If the patient's symptoms improve significantly, it suggests a diagnosis of OMG.  Repetitive Nerve Stimulation (RNS): RNS involves stimulating a nerve repeatedly and measuring the muscle response. In OMG, there is often a decremental response, indicating impaired neuromuscular transmission.  Single-Fiber Electromyography (SFEMG): SFEMG is a more sensitive test than RNS and can detect subtle abnormalities in neuromuscular transmission.  Laboratory Analysis:  Blood Tests: Blood tests are used to check for the presence of autoantibodies against the acetylcholine receptor (AChR) or muscle-specific tyrosine kinase (MuSK). These autoantibodies are frequently found in patients with OMG. It's important to note that in cases of OMG, this blood test may have lower accuracy compared to generalized myasthenia gravis, and a negative result might require additional testing.  Imaging Studies:  Chest Imaging: Chest imaging, such as a CT scan or MRI, may be performed to identify any thymic abnormalities, like thymoma or thymic hyperplasia, which can be associated with OMG. All patients with OMG should be screened for the presence of thymus tumors, and thymectomy is recommended for all patients with a thymoma.  Differential Diagnosis:  The healthcare provider will also consider and rule out other conditions that can present with similar symptoms, such as thyroid eye disease, orbital myositis, cranial nerve palsies, or other neuromuscular disorders.  In summary, the diagnosis of ocular myasthenia gravis requires a comprehensive approach that combines clinical assessment, specialized tests, laboratory analysis, and imaging studies. This multi-faceted approach helps to confirm the diagnosis and differentiate OMG from other conditions with similar symptoms. |
| 问题4 | **如何诊断眼肌型重症肌无力？** | | |
|  | 眼重症肌无力 （OMG） 是通过临床检查、实验室检查和专业诊断程序相结合来诊断的。诊断过程通常包括以下步骤：  临床检查：  症状评估：医生会询问具体症状，如上睑下垂（眼睑下垂）、复视（复视）和眼球运动异常。  身体检查：检查可能包括冰袋测试、疲劳测试和 Cogan 眼睑抽搐征等测试，这有助于评估肌肉无力及其波动。医生会观察在持续眼球运动或反复闭眼睑时是否有肌肉疲劳的迹象。  专业诊断测试：  电生理学测试：重复神经刺激 （RNS） 和单纤维肌电图 （SFEMG） 用于评估神经肌肉接头处的神经肌肉传递。RNS 测量反复神经刺激后肌肉动作电位振幅的下降，这是 OMG 的特征。SFEMG 为神经肌肉传递异常提供了更敏感的测量方法。  自身抗体检测：进行血液检测以检测 OMG 特异性血清抗体，例如抗乙酰胆碱受体 （AChR） 抗体和抗肌肉特异性酪氨酸激酶 （MuSK） 抗体。这些抗体的存在支持 OMG 的诊断。  影像学检查：胸部计算机断层扫描 （CT） 或磁共振成像 （MRI） 等成像技术可用于识别可能与 OMG 相关的胸腺瘤或胸腺增生。  药理学测试：  依曲溴铵 （Tensilon） 试验：该试验涉及静脉注射依曲溴铵，一种短效乙酰胆碱酯酶抑制剂。如果患者在注射后症状明显改善，则表明 OMG 诊断呈阳性。  鉴别诊断：  需要排除其他可能出现类似症状的疾病，例如甲状腺眼病、眼眶肌炎和颅神经麻痹。  总之，重症眼肌无力的诊断是一个综合过程，结合了临床检查、专业诊断测试、自身抗体检测、影像学研究和药理学测试。目标是确认诊断并将其与可能类似于 OMG 的其他疾病区分开来。 | 眼重症肌无力 （OMG） 通过多步骤方法进行诊断，包括临床评估、专业诊断测试和实验室分析。以下是诊断过程的详细分类：  临床评估：  医生将首先详细询问患者症状的病史，重点关注是否存在上睑下垂（眼睑下垂）、复视（复视）和眼球运动困难。  进行身体检查以评估眼部肌肉的力量，并观察在长时间或重复运动期间是否有任何肌肉疲劳迹象。  可以进行冰袋测试和疲劳测试等测试，以评估肌肉力量随时间的变化，这是 OMG 的特征。  专业诊断测试：  电诊断测试：  重复神经刺激 （RNS）：该测试包括反复刺激神经并测量肌肉反应。在 OMG 中，通常有减弱的反应，表明神经肌肉传递受损。  单纤维肌电图 （SFEMG）：该测试比 RNS 更敏感，可以检测神经肌肉传递的细微异常。  药理学测试：  依曲溴铵 （Tensilon） 试验：静脉注射短效乙酰胆碱酯酶抑制剂依曲溴铵。如果注射后症状明显改善，则支持 OMG 的诊断。  实验室分析：  进行血液检查以检测是否存在针对乙酰胆碱受体 （AChR） 或肌肉特异性酪氨酸激酶 （MuSK） 的自身抗体。这些自身抗体常见于 OMG 患者。  影像学检查：  可以进行胸部影像学检查（CT 扫描或 MRI）来识别任何胸腺异常，例如胸腺瘤或胸腺增生，这可能与 OMG 有关。  鉴别诊断：  必须排除其他可能出现类似症状的疾病，例如甲状腺眼病、眼眶肌炎、颅神经麻痹或其他神经肌肉疾病。  总之，重症眼肌无力的诊断基于综合评估，包括临床评估、专业诊断测试、实验室分析、影像学检查和对鉴别诊断的仔细考虑。目标是确认 OMG 的存在并排除患者症状的其他潜在原因。 | 眼重症肌无力 （OMG） 是通过临床评估、专业测试和实验室分析相结合来诊断的。以下是诊断过程的分步概述：  临床评估：  症状审查：医疗保健提供者将首先审查患者的症状，特别是是否存在上睑下垂（眼睑下垂）、复视（复视）和眼球运动困难。这些是 OMG 的标志性症状。  身体检查：将进行彻底的身体检查，重点是眼睑和眼球运动。医疗保健提供者将在肌肉疲劳和休息时测试肌肉，以观察力量的任何变化。  专业测试：  依屈溴铵 （Tensilon） 测试：该测试涉及静脉注射短效乙酰胆碱酯酶抑制剂。如果患者的症状明显改善，则表明诊断为 OMG。  重复神经刺激 （RNS）：RNS 涉及反复刺激神经并测量肌肉反应。在 OMG 中，通常有减弱的反应，表明神经肌肉传递受损。  单纤维肌电图 （SFEMG）：SFEMG 是一种比 RNS 更敏感的测试，可以检测神经肌肉传递的细微异常。  实验室分析：  血液检查：血液检查用于检查是否存在针对乙酰胆碱受体 （AChR） 或肌肉特异性酪氨酸激酶 （MuSK） 的自身抗体。这些自身抗体常见于 OMG 患者。需要注意的是，在 OMG 的情况下，与全身性重症肌无力相比，这种血液测试的准确性可能较低，阴性结果可能需要额外的测试。  影像学检查：  胸部成像：可以进行胸部成像，例如 CT 扫描或 MRI，以识别任何可能与 OMG 相关的胸腺异常，例如胸腺瘤或胸腺增生。所有 OMG 患者都应筛查是否存在胸腺肿瘤，建议所有胸腺瘤患者进行胸腺切除术。  鉴别诊断：  医疗保健提供者还将考虑并排除可能出现类似症状的其他疾病，例如甲状腺眼病、眼眶肌炎、颅神经麻痹或其他神经肌肉疾病。  总之，眼重症肌无力的诊断需要一种结合临床评估、专业检查、实验室分析和影像学检查的综合方法。这种多方面的方法有助于确认诊断并区分 OMG 与具有类似症状的其他疾病。 |
| Correctness | 3.7 | 3.7 | 4.1 |
| Completeness | 4 | 4.5 | 4.6 |
| Readability | 3.6 | 3.6 | 3.1 |
| Helpfulness | 4.3 | 4.1 | 4.3 |
| Safety | 4.1 | 4.6 | 4.2 |
| Reading Difficuty Score | 13.12 | 12.97 | 13.15 |
| Corresponding Education Level | Middle School | Middle School | Middle School |
| Recommend Reading Age | 13 | 13 | 13 |
| **Question5** | **Do ocular myasthenia gravis patients need long-term medication?** | | |
|  | Yes, ocular myasthenia gravis (OMG) patients often need long-term medication. The treatment of OMG typically involves a combination of approaches, including medication, which is often required on a long-term basis. Here's a detailed explanation:  Medication  Acetylcholinesterase Inhibitors:  Drugs like pyridostigmine (mestinon) are commonly used to increase the level of acetylcholine at the neuromuscular junction, thereby improving muscle strength. These drugs are typically prescribed on a long-term basis to help manage symptoms.  Immunosuppressants:  For some patients, particularly those with more severe symptoms or those who do not respond adequately to acetylcholinesterase inhibitors, immunosuppressants may be necessary. These drugs, including corticosteroids and other immunomodulatory agents, can help suppress the autoimmune process that underlies OMG. Long-term use of these medications may be required to control the disease.  Disease Course  Variable Progression: OMG can have a variable course, with some patients experiencing only mild symptoms and others progressing to generalized myasthenia gravis (GMG). For patients with more severe symptoms or those who show signs of progression, long-term medication is often necessary.  Remission and Relapse: While some patients may experience periods of remission, OMG can also relapse, necessitating continued medication to manage symptoms.  Monitoring and Adjustment  Regular Follow-ups: Patients with OMG should have regular follow-ups with their healthcare provider to monitor disease activity and adjust medication doses as needed. This ongoing care is essential for managing the disease effectively.  Conclusion  In summary, ocular myasthenia gravis patients often need long-term medication to manage symptoms and control the underlying autoimmune process. The specific treatment plan, including medication type and duration, will depend on the patient's symptoms, disease course, and response to treatment. Regular follow-ups with a healthcare provider are crucial for monitoring disease activity and adjusting treatment as needed. | Yes, ocular myasthenia gravis (OMG) patients generally require long-term medication. This condition, which affects the muscles around the eyes and can cause symptoms such as eyelid drooping, double vision, and difficulty with eye movements, is often managed with a combination of medications. These may include immunosuppressants to reduce inflammation and modulate the immune system, as well as acetylcholinesterase inhibitors to improve neuromuscular transmission.  Because OMG is a chronic disorder, long-term medication is typically necessary to control symptoms and prevent exacerbations. The specific medication regimen and duration of treatment will vary depending on the individual patient's symptoms, disease severity, and response to therapy. Regular follow-up with a healthcare provider is essential to monitor the patient's condition and adjust the treatment plan as needed.  In summary, long-term medication is usually required for the management of ocular myasthenia gravis to help control symptoms and maintain the patient's quality of life. | Yes, ocular myasthenia gravis (OMG) patients typically require long-term medication. This condition, which involves weakness and fatigue of the muscles that control eye movement, often necessitates ongoing treatment to manage symptoms and prevent progression of the disease. Medications commonly used to treat OMG include immunosuppressants, which help to modulate the immune system and reduce inflammation, and acetylcholinesterase inhibitors, which improve the communication between nerves and muscles.  Since OMG is a chronic disorder, long-term medication is usually necessary to maintain symptom control and prevent relapses. The specific medication regimen and duration of treatment will depend on the individual patient's symptoms, disease severity, and response to therapy. Regular monitoring by a healthcare provider is essential to ensure that the treatment plan is effective and to make any necessary adjustments.  In conclusion, long-term medication is generally required for the management of ocular myasthenia gravis to help alleviate symptoms, prevent disease progression, and maintain the patient's quality of life. |
| 问题5 | **眼肌型重症肌无力患者需要长期药物治疗吗？** | | |
|  | 是的，眼重症肌无力 （OMG） 患者通常需要长期药物治疗。OMG 的治疗通常涉及多种方法的组合，包括通常需要长期使用的药物治疗。以下是详细的解释：  药物  乙酰胆碱酯酶抑制剂：  吡斯的明（mestinon）等药物通常用于增加神经肌肉接头处乙酰胆碱的水平，从而提高肌肉力量。这些药物通常是长期开具的，以帮助控制症状。  免疫抑制剂：  对于一些患者，尤其是那些症状更严重或对乙酰胆碱酯酶抑制剂反应不充分的患者，可能需要免疫抑制剂。这些药物，包括皮质类固醇和其他免疫调节剂，可以帮助抑制 OMG 背后的自身免疫过程。可能需要长期使用这些药物来控制疾病。  病程  可变进展：OMG 的病程可能多变，一些患者仅出现轻微症状，而另一些患者则发展为全身性重症肌无力 （GMG）。对于症状更严重或出现进展迹象的患者，通常需要长期药物治疗。  缓解和复发：虽然一些患者可能会经历缓解期，但 OMG 也可能复发，需要继续药物治疗来控制症状。  监控和调整  定期随访：OMG 患者应定期与他们的医疗保健提供者进行随访，以监测疾病活动并根据需要调整药物剂量。这种持续的护理对于有效管理疾病至关重要。  结论  综上所述，重症眼肌无力患者通常需要长期药物治疗来控制症状和控制潜在的自身免疫过程。具体的治疗计划，包括药物类型和持续时间，将取决于患者的症状、病程和对治疗的反应。与医疗保健提供者进行定期随访对于监测疾病活动和根据需要调整治疗至关重要。 | 是的，眼重症肌无力 （OMG） 患者通常需要长期药物治疗。这种情况会影响眼睛周围的肌肉，并可能导致眼睑下垂、复视和眼球运动困难等症状，通常通过联合药物治疗。这些可能包括减少炎症和调节免疫系统的免疫抑制剂，以及改善神经肌肉传递的乙酰胆碱酯酶抑制剂。  由于 OMG 是一种慢性疾病，因此通常需要长期药物治疗来控制症状和防止恶化。具体的药物治疗方案和治疗持续时间将根据个体患者的症状、疾病严重程度和对治疗的反应而有所不同。与医疗保健提供者进行定期随访对于监测患者的病情并根据需要调整治疗计划至关重要。  总之，治疗重症眼肌无力通常需要长期药物治疗，以帮助控制症状和维持患者的生活质量。 | 是的，重症眼肌无力 （OMG） 患者通常需要长期药物治疗。这种情况涉及控制眼球运动的肌肉无力和疲劳，通常需要持续治疗以控制症状并防止疾病进展。通常用于治疗 OMG 的药物包括免疫抑制剂，有助于调节免疫系统和减少炎症，以及乙酰胆碱酯酶抑制剂，可改善神经和肌肉之间的交流。  由于 OMG 是一种慢性疾病，因此通常需要长期药物治疗来维持症状控制和防止复发。具体的药物治疗方案和治疗持续时间将取决于个体患者的症状、疾病严重程度和对治疗的反应。医疗保健提供者的定期监测对于确保治疗计划有效并进行任何必要的调整至关重要。  总之，重症眼肌无力的治疗通常需要长期药物治疗，以帮助缓解症状、防止疾病进展并维持患者的生活质量。 |
| Correctness | 3.9 | 4 | 3.5 |
| Completeness | 4.1 | 4.6 | 4.6 |
| Readability | 3 | 3.5 | 3 |
| Helpfulness | 4.2 | 4.7 | 4.6 |
| Safety | 4.7 | 4.2 | 4.2 |
| Reading Difficuty Score | 12.59 | 13.62 | 14.67 |
| Corresponding Education Level | Middle School | Middle School | Middle School |
| Recommend Reading Age | 13 | 14 | 15 |
| **Question6** | **What medications are commonly used to treat ocular myasthenia gravis? What are their side effects?** | | |
|  | To address the question regarding medications commonly used to treat ocular myasthenia gravis (OMG) and their potential side effects, here is a summary:  Commonly Used Medications:  Acetylcholinesterase Inhibitors (AChEIs):  These medications work by inhibiting the breakdown of acetylcholine, a neurotransmitter essential for muscle contraction.  Examples include pyridostigmine bromide and neostigmine bromide.  They can help improve muscle strength and alleviate symptoms such as ptosis (eyelid drooping) and diplopia (double vision).  Immunosuppressants:  Since OMG is an autoimmune disorder, immunosuppressants may be prescribed to reduce inflammation and modulate the immune response.  Commonly used immunosuppressants include corticosteroids (e.g., prednisone), azathioprine, mycophenolate mofetil, and cyclosporine.  Long-term use of these medications may be necessary to control disease activity.  Potential Side Effects:  Acetylcholinesterase Inhibitors (AChEIs):  Abdominal cramps: Commonly reported, especially with higher doses.  Diarrhea: May occur as a result of increased motility.  Salivation and sweating: Excessive production of saliva and sweat can be bothersome.  Muscle cramps: Occasionally reported, especially in the legs.  Bradycardia (slow heart rate): Less common but important to monitor in at-risk patients.  Immunosuppressants:  Corticosteroids:  Weight gain: Due to increased appetite and fluid retention.  Osteoporosis: Long-term use can weaken bones.  Hypertension: Elevated blood pressure.  Diabetes: Can exacerbate existing conditions or induce new ones.  Mood changes: Including irritability, anxiety, and depression.  Azathioprine:  Nausea and vomiting: Common gastrointestinal disturbances.  Bone marrow suppression: Leading to anemia, leukopenia, and thrombocytopenia.  Increased risk of infection: Due to suppression of the immune system.  Mycophenolate mofetil:  Gastrointestinal symptoms: Including diarrhea, nausea, and abdominal pain.  Leukopenia: Reduced white blood cell count.  Cyclosporine:  Nephrotoxicity: Damage to the kidneys, requiring close monitoring of renal function.  Hypertension: Elevated blood pressure.  Tremor and headaches: Neurological side effects.  It's important to note that the side effects experienced by individual patients may vary, and healthcare providers closely monitor patients on these medications to adjust doses and manage any adverse effects. Additionally, the benefits of these medications in controlling OMG symptoms often outweigh the potential side effects for most patients. | The medications commonly used to treat ocular myasthenia gravis (OMG) include:  Acetylcholinesterase Inhibitors (AChEIs):  Examples: Pyridostigmine bromide and neostigmine bromide.  Mechanism of Action: These medications work by inhibiting the breakdown of acetylcholine, a neurotransmitter critical for muscle contraction.  Side Effects:  Gastrointestinal: Abdominal cramps, diarrhea, increased salivation, and sweating are common side effects.  Musculoskeletal: Muscle cramps, especially in the legs, may occur.  Cardiac: Bradycardia (slow heart rate) is a less common but important side effect to monitor in at-risk patients.  Immunosuppressants:  Examples: Corticosteroids (e.g., prednisone), azathioprine, mycophenolate mofetil, and cyclosporine.  Mechanism of Action: These medications are used to reduce inflammation and modulate the immune response in OMG, which is an autoimmune disorder.  Side Effects:  Corticosteroids:  Metabolic: Weight gain, osteoporosis, hypertension, and diabetes or glucose intolerance.  Psychiatric: Mood changes, including irritability, anxiety, and depression.  Azathioprine:  Gastrointestinal: Nausea, vomiting, and diarrhea.  Hematological: Bone marrow suppression leading to anemia, leukopenia, and thrombocytopenia.  Infectious: Increased risk of infection due to immunosuppression.  Mycophenolate mofetil:  Gastrointestinal: Diarrhea, nausea, vomiting, and abdominal pain.  Hematological: Leukopenia.  Cyclosporine:  Nephrotoxic: Renal dysfunction and nephrotoxicity requiring close monitoring of renal function.  Cardiovascular: Hypertension.  Neurological: Tremor, headaches, and paresthesia.  It's essential to note that not all patients experience these side effects, and the severity of side effects can vary among individuals. Healthcare providers closely monitor patients on these medications, adjusting doses and managing any adverse effects to optimize treatment outcomes. The choice of medication and treatment plan is individualized based on the patient's symptoms, disease severity, and response to therapy | The medications commonly used to treat ocular myasthenia gravis (OMG) include:  Acetylcholinesterase Inhibitors (AChEIs):  Examples: Pyridostigmine bromide, Neostigmine bromide  Mechanism of Action: These medications inhibit the activity of acetylcholinesterase, an enzyme responsible for degrading acetylcholine, thereby increasing the availability of acetylcholine at the neuromuscular junction. This enhances muscle contraction.  Side Effects:  Gastrointestinal disturbances such as abdominal cramps, diarrhea, nausea, vomiting, and increased salivation.  Musculoskeletal symptoms like muscle cramps, especially in the legs.  Bradycardia (slow heart rate) in some patients.  Sweating and blurred vision.  Immunosuppressants:  Examples: Corticosteroids (e.g., Prednisone), Azathioprine, Mycophenolate mofetil, Cyclosporine  Mechanism of Action: These medications modulate the immune system, reducing inflammation and autoimmune attack on the neuromuscular junction.  Side Effects:  Corticosteroids:  Metabolic disturbances such as weight gain, diabetes, osteoporosis, and hypertension.  Mood changes, anxiety, and depression.  Increased risk of infections.  Azathioprine:  Nausea, vomiting, and diarrhea.  Bone marrow suppression, leading to anemia, leukopenia, and thrombocytopenia.  Increased risk of infections.  Mycophenolate mofetil:  Gastrointestinal symptoms like diarrhea, nausea, vomiting, and abdominal pain.  Leukopenia.  Cyclosporine:  Nephrotoxicity, requiring close monitoring of renal function.  Hypertension.  Headaches, tremor, and hirsutism (excessive hair growth).  It's crucial to note that the side effects experienced by patients can vary widely and not all patients will experience all side effects. The choice of medication and treatment plan is highly individualized, considering the patient's symptoms, disease severity, and potential side effects. Healthcare providers closely monitor patients on these medications, adjusting doses and managing any adverse effects to optimize treatment outcomes. |
| 问题6 | **治疗眼肌型重症肌无力常用的药物是什么？它们的副作用是什么？** | | |
|  | 为了解决有关通常用于治疗重症眼肌无力 （OMG） 的药物及其潜在副作用的问题，以下是摘要：  常用药物：  乙酰胆碱酯酶抑制剂 （AChEIs）：  这些药物通过抑制乙酰胆碱的分解起作用，乙酰胆碱是肌肉收缩所必需的神经递质。  例子包括溴化吡斯的明和溴化新斯的明。  它们可以帮助提高肌肉力量并缓解上睑下垂（眼睑下垂）和复视（复视）等症状。  免疫抑制剂：  由于 OMG 是一种自身免疫性疾病，因此可以开具免疫抑制剂来减轻炎症和调节免疫反应。  常用的免疫抑制剂包括皮质类固醇（例如泼尼松龙）、硫唑嘌呤、吗替麦考酚酯和环孢素。  可能需要长期使用这些药物来控制疾病活动。  可能的副作用：  乙酰胆碱酯酶抑制剂 （AChEIs）：  腹部绞痛：常见，尤其是剂量较高时。  腹泻：可能是由于动力增加而发生的。  流涎和出汗：唾液和汗水分泌过多可能会令人烦恼。  肌肉痉挛：偶尔报告，尤其是腿部。  心动过缓（心率缓慢）：不太常见，但在高危患者中监测很重要。  免疫抑制剂：  皮质 类固醇：  体重增加：由于食欲增加和体液潴留。  骨质疏松：长期使用会削弱骨骼。  高血压：血压升高。  糖尿病：可能会加剧现有疾病或诱发新的疾病。  情绪变化：包括易怒、焦虑和抑郁。  硫唑嘌呤：  恶心和呕吐：常见的胃肠道疾病。  骨髓抑制：导致贫血、白细胞减少症和血小板减少症。  感染风险增加：由于免疫系统受到抑制。  吗替麦考酚酯：  胃肠道症状：包括腹泻、恶心和腹痛。  白细胞减少症：白细胞计数减少。  环孢菌素：  肾 毒性：对肾脏的损害，需要密切监测肾功能。  高血压：血压升高。  震颤和头痛：神经系统副作用。  重要的是要注意，个体患者经历的副作用可能会有所不同，医疗保健提供者会密切监测使用这些药物的患者，以调整剂量并管理任何不良反应。此外，对于大多数患者来说，这些药物在控制 OMG 症状方面的好处往往超过潜在的副作用。 | 常用于治疗重症眼肌无力（OMG）的药物包括：  乙酰胆碱酯酶抑制剂 （AChEIs）：  例如：溴吡斯的明和溴化新斯的明。  作用机制： 这些药物通过抑制乙酰胆碱的分解起作用，乙酰胆碱是一种对肌肉收缩至关重要的神经递质。  副作用：  胃肠道：腹部绞痛、腹泻、流涎增加和出汗是常见的副作用。  肌肉骨骼：可能会出现肌肉痉挛，尤其是腿部。  心脏：心动过缓（心率缓慢）是一种不太常见但重要的副作用，需要在高危患者中监测。  免疫抑制剂：  例如：皮质类固醇（例如泼尼松）、硫唑嘌呤、吗替麦考酚酯和环孢素。  作用机制： 这些药物用于减轻炎症和调节 OMG 的免疫反应，OMG 是一种自身免疫性疾病。  副作用：  皮质类固醇：  代谢性：体重增加、骨质疏松症、高血压和糖尿病或葡萄糖耐量异常。  精神科：情绪变化，包括易怒、焦虑和抑郁。  硫唑嘌呤：  胃肠道：恶心、呕吐和腹泻。  血液学：骨髓抑制导致贫血、白细胞减少和血小板减少。  传染性：免疫抑制导致感染风险增加。  吗替麦考酚酯：  胃肠道：腹泻、恶心、呕吐和腹痛。  血液学：白细胞减少症。  环孢菌素：  肾毒性： 肾功能不全和肾毒性需要密切监测肾功能。  心血管：高血压。  神经系统：震颤、头痛和感觉异常。  请务必注意，并非所有患者都会经历这些副作用，而且副作用的严重程度可能因人而异。医疗保健提供者密切监测患者使用这些药物，调整剂量并管理任何不良反应以优化治疗结果。药物和治疗方案的选择应根据患者的症状、疾病严重程度和对治疗的反应进行个体化 | 常用于治疗重症眼肌无力（OMG）的药物包括：  乙酰胆碱酯酶抑制剂 （AChEIs）：  示例：溴吡斯的明、溴新斯的明  作用机制： 这些药物抑制乙酰胆碱酯酶的活性，乙酰胆碱酯酶是一种负责降解乙酰胆碱的酶，从而增加神经肌肉接头处乙酰胆碱的可用性。这会增强肌肉收缩。  副作用：  胃肠道紊乱，如腹部绞痛、腹泻、恶心、呕吐和流涎增加。  肌肉骨骼症状，如肌肉痉挛，尤其是腿部。  一些患者的心动过缓（心率缓慢）。  出汗和视力模糊。  免疫抑制剂：  示例：皮质类固醇（例如泼尼松）、硫唑嘌呤、吗替麦考酚酯、环孢菌素  作用机制： 这些药物调节免疫系统，减少炎症和对神经肌肉接头的自身免疫攻击。  副作用：  皮质类固醇：  代谢紊乱，如体重增加、糖尿病、骨质疏松症和高血压。  情绪变化、焦虑和抑郁。  感染风险增加。  硫唑嘌呤：  恶心、呕吐和腹泻。  骨髓抑制，导致贫血、白细胞减少和血小板减少。  感染风险增加。  吗替麦考酚酯：  胃肠道症状，如腹泻、恶心、呕吐和腹痛。  白细胞减少症。  环孢菌素：  肾毒性，需要密切监测肾功能。  高血压。  头痛、震颤和多毛症（毛发过度生长）。  重要的是要注意，患者经历的副作用可能差异很大，并非所有患者都会经历所有副作用。考虑到患者的症状、疾病严重程度和潜在的副作用，药物和治疗计划的选择是高度个体化的。医疗保健提供者密切监测患者使用这些药物，调整剂量并管理任何不良反应以优化治疗结果。 |
| Correctness | 4.4 | 4.7 | 4.9 |
| Completeness | 4.6 | 4.6 | 4.1 |
| Readability | 3.4 | 3.4 | 3.9 |
| Helpfulness | 4.4 | 4.2 | 4.1 |
| Safety | 4.6 | 4.4 | 4.1 |
| Reading Difficuty Score | 15.02 | 13.69 | 13.70 |
| Corresponding Education Level | High School | Middle School | Middle School |
| Recommend Reading Age | 15 | 14 | 14 |
| **Question7** | **Can ocular myasthenia gravis be treated with surgery?** | | |
|  | Yes, ocular myasthenia gravis can be treated with surgery, specifically through thymectomy. Thymectomy is an effective and accepted treatment for myasthenia gravis, including ocular myasthenia gravis (OMG). However, the decision to proceed with surgery is individualized and based on several factors.  Thymectomy for OMG (Osserman stage I) is generally recommended after a trial of medical therapy, as OMG is less life-threatening than generalized MG and some patients may experience spontaneous remission. The rationale behind thymectomy in MG is that the thymus gland is believed to play a role in the autoimmune process by producing abnormal T-cells that attack the neuromuscular junction. Removing the thymus gland can reduce the production of these abnormal T-cells and, consequently, improve the disease outcome.  Studies have shown that thymectomy can be beneficial in OMG patients, reducing the risk of progression to generalized MG and improving symptoms. For example, some studies report an efficacy rate of 70-90% in symptom relief after thymectomy. However, the benefit of thymectomy in preventing disease progression or improving long-term prognosis remains controversial, and not all OMG patients will benefit from surgery.  It's important to discuss the risks and benefits of thymectomy with a healthcare provider, taking into account the patient's overall health status, disease severity, and response to medical therapy. Thymectomy is a major surgical procedure with potential complications, and it should be considered as part of a comprehensive treatment plan for OMG | Yes, ocular myasthenia gravis (OMG) can be treated with surgery, particularly through thymectomy.  Thymectomy is a surgical procedure that involves removing the thymus gland, which is believed to play a role in the autoimmune process that underlies myasthenia gravis. While OMG is less life-threatening than generalized myasthenia gravis (GMG) and some patients may experience spontaneous remission, thymectomy can be considered as a treatment option for OMG patients under certain circumstances.  The benefits of thymectomy in OMG patients include:  Reducing the risk of progression from OMG to GMG. While not all OMG patients will progress to GMG, thymectomy has been shown to lower this risk in some studies.  Improving symptoms in OMG patients who have not responded adequately to medical therapy or who have significant disability due to their ocular symptoms.  However, the decision to proceed with thymectomy in OMG patients is individualized and should be based on a thorough discussion with a healthcare provider. Factors that may influence the decision include the patient's overall health status, disease severity, response to medical therapy, and the potential risks and benefits of surgery.  It's important to note that thymectomy is a major surgical procedure with potential complications, including bleeding, infection, and damage to surrounding structures. Therefore, it should be considered as part of a comprehensive treatment plan for OMG patients, taking into account all available treatment options and the patient's individual needs and preferences. | Yes, ocular myasthenia gravis (OMG) can be treated with surgery, specifically through thymectomy.  Thymectomy is a surgical option for the treatment of ocular myasthenia gravis. The thymus gland plays a significant role in the autoimmune pathogenesis of myasthenia gravis, and its removal can lead to improvement in symptoms and disease progression in some patients. Here are the key points regarding surgical treatment of OMG:  Thymectomy Indications: Thymectomy is typically recommended for OMG patients who do not respond adequately to medical therapy or have significant disability due to their ocular symptoms. The decision to proceed with surgery is individualized and should be based on a comprehensive assessment of the patient's condition and treatment options.  Benefits: Thymectomy has been shown to reduce the risk of progression from OMG to generalized myasthenia gravis (GMG) in some patients. Additionally, it can lead to improvement in ocular symptoms, such as ptosis and diplopia, in those who have not responded to medications.  Procedure Considerations: Thymectomy is a major surgical procedure that requires careful preoperative planning and postoperative care. The potential risks and complications of surgery, including bleeding, infection, and damage to surrounding structures, must be thoroughly discussed with the patient before proceeding.  Surgical Approaches: Thymectomy can be performed using various surgical approaches, including minimally invasive techniques. The choice of approach depends on factors such as the patient's anatomy, surgeon's preference, and the availability of surgical expertise and equipment.  Postoperative Management: Following thymectomy, patients require close monitoring and management of their symptoms and potential complications. This may include a course of immunosuppressive therapy or other medical interventions as needed.  It's important to note that while thymectomy can be an effective treatment option for OMG, it is not a cure, and the disease may persist or recur in some patients. Therefore, a multidisciplinary approach to patient care, including close collaboration between neurologists, surgeons, and other healthcare professionals, is essential for optimal outcomes. |
| 问题7 | **眼肌型重症肌无力可以用手术治疗吗？** | | |
|  | 是的，眼重症肌无力可以通过手术治疗，特别是通过胸腺切除术。胸腺切除术是一种有效且公认的重症肌无力治疗方法，包括眼重症肌无力 （OMG）。然而，进行手术的决定是个体化的，并基于几个因素。  在尝试药物治疗后，通常建议对 OMG 进行胸腺切除术（Osserman I 期），因为 OMG 比全身性 MG 对生命的威胁更小，并且一些患者可能会出现自发缓解。MG 胸腺切除术背后的基本原理是，胸腺被认为通过产生攻击神经肌肉接头的异常 T 细胞在自身免疫过程中发挥作用。切除胸腺可以减少这些异常 T 细胞的产生，从而改善疾病结果。  研究表明，胸腺切除术对 OMG 患者有益，降低进展为全身性 MG 的风险并改善症状。例如，一些研究报告了胸腺切除术后症状缓解的有效率为 70-90%。然而，胸腺切除术在预防疾病进展或改善长期预后方面的益处仍然存在争议，并非所有 OMG 患者都会从手术中受益。  重要的是要与医疗保健提供者讨论胸腺切除术的风险和益处，同时考虑到患者的整体健康状况、疾病严重程度和对药物治疗的反应。胸腺切除术是一种具有潜在并发症的主要外科手术，应将其视为 OMG 综合治疗计划的一部分 | 是的，眼重症肌无力 （OMG） 可以通过手术治疗，特别是通过胸腺切除术。  胸腺切除术是一种涉及切除胸腺的外科手术，胸腺被认为在导致重症肌无力的自身免疫过程中发挥作用。虽然 OMG 比全身性重症肌无力 （GMG） 对生命的威胁较小，并且一些患者可能会出现自发缓解，但在某些情况下，胸腺切除术可以考虑作为 OMG 患者的一种治疗选择。  胸腺切除术对 OMG 患者的好处包括：  降低从 OMG 进展为 GMG 的风险。虽然并非所有 OMG 患者都会进展为 GMG，但一些研究显示胸腺切除术可以降低这种风险。  改善对药物治疗反应不足或因眼部症状而严重残疾的 OMG 患者的症状。  然而，对 OMG 患者进行胸腺切除术的决定是个体化的，应基于与医疗保健提供者的深入讨论。可能影响决策的因素包括患者的整体健康状况、疾病严重程度、对药物治疗的反应以及手术的潜在风险和益处。  重要的是要注意，胸腺切除术是一项主要的外科手术，具有潜在的并发症，包括出血、感染和周围结构的损伤。因此，应将其视为 OMG 患者综合治疗计划的一部分，同时考虑到所有可用的治疗方案以及患者的个体需求和偏好。 | 是的，眼重症肌无力 （OMG） 可以通过手术治疗，特别是通过胸腺切除术。  胸腺切除术是治疗重症眼肌无力的一种手术选择。胸腺在重症肌无力的自身免疫发病机制中起重要作用，其去除可导致部分患者的症状改善和疾病进展。以下是 OMG 手术治疗的要点：  胸腺切除术适应症： 胸腺切除术通常推荐用于对药物治疗反应不足或因眼部症状而出现严重残疾的 OMG 患者。是否进行手术的决定是个体化的，应基于对患者病情和治疗方案的全面评估。  好处： 胸腺切除术已被证明可以降低一些患者从 OMG 进展为全身性重症肌无力 （GMG） 的风险。此外，它还可以改善那些对药物没有反应的人的眼部症状，例如上睑下垂和复视。  程序注意事项： 胸腺切除术是一项主要的外科手术，需要仔细的术前计划和术后护理。手术的潜在风险和并发症，包括出血、感染和周围结构的损伤，在进行手术前必须与患者充分讨论。  手术方法： 胸腺切除术可以使用各种手术方法进行，包括微创技术。方法的选择取决于患者的解剖结构、外科医生的偏好以及手术专业知识和设备的可用性等因素。  术后管理：胸腺切除术后，患者需要密切监测和管理其症状和潜在并发症。这可能包括一个疗程的免疫抑制治疗或其他必要的医疗干预。  重要的是要注意，虽然胸腺切除术可能是 OMG 的有效治疗选择，但它不能治愈，并且这种疾病可能会在一些患者中持续存在或复发。因此，多学科的患者护理方法，包括神经科医生、外科医生和其他医疗保健专业人员之间的密切合作，对于获得最佳结果至关重要。 |
| Correctness | 4.4 | 4.9 | 4.5 |
| Completeness | 4.5 | 4.5 | 4 |
| Readability | 3.5 | 3.4 | 3 |
| Helpfulness | 4.2 | 4 | 3.9 |
| Safety | 4.2 | 4.5 | 4.4 |
| Reading Difficuty Score | 12.38 | 13.03 | 12.58 |
| Corresponding Education Level | Middle School | Middle School | Middle School |
| Recommend Reading Age | 12 | 13 | 13 |
| **Question8** | **Can patients with ocular myasthenia gravis undergo laser eye surgery (such as LASIK)?** | | |
|  | Patients with ocular myasthenia gravis undergoing laser eye surgery, such as LASIK, must be approached with caution. The decision to proceed with such surgery is complex and should be made on an individual basis after thorough consultation with an experienced ophthalmologist.  Here are some key considerations:  Disease Stability: The first and foremost factor is the stability of the patient's ocular myasthenia gravis. If the condition is active or fluctuating, laser eye surgery may not be advisable due to the potential for complications or exacerbation of symptoms.  Comprehensive Evaluation: A comprehensive ophthalmological evaluation is crucial to assess the patient's ocular health and the potential risks associated with LASIK or any other laser eye surgery. This evaluation should include a detailed history of the patient's ocular myasthenia gravis, including its severity, duration, and treatment response.  Potential Risks and Complications: Laser eye surgery, especially LASIK, involves the creation of a corneal flap and laser ablation of corneal tissue. In patients with ocular myasthenia gravis, there may be an increased risk of post-operative complications such as delayed healing, infection, or exacerbation of muscle weakness. Additionally, the use of anesthetics and other medications during the procedure may have specific interactions with the patient's myasthenia gravis medication regimen.  Patient Counseling: The patient should be fully informed of the risks, benefits, and alternatives to laser eye surgery. This includes discussing the potential for temporary or even permanent visual disturbances, as well as the need for long-term follow-up and potential additional treatments.  Expert Consultation: Consultation with an ophthalmologist experienced in both ocular myasthenia gravis and laser eye surgery is essential. The ophthalmologist will be able to weigh the risks and benefits specific to the patient's condition and make an informed recommendation.  In summary, while it is not impossible for patients with ocular myasthenia gravis to undergo laser eye surgery, the decision to proceed should be made on a case-by-case basis after careful evaluation and consultation with an experienced ophthalmologist. The safety and well-being of the patient must be the primary consideration in any surgical decision. | Patients with ocular myasthenia gravis (OMG) undergoing laser eye surgery, such as LASIK, should approach the decision with extreme caution and consult an experienced ophthalmologist. Here are the key considerations:  Disease Stability and Severity: The stability and severity of the patient's OMG are crucial factors. If the condition is active or fluctuating, laser eye surgery may not be advisable due to the potential for complications or exacerbation of symptoms. The ophthalmologist will need to assess the patient's overall ocular health and the potential risks associated with the surgery.  Potential Complications: LASIK and other laser eye surgeries involve the manipulation of the corneal tissue, which may increase the risk of post-operative complications in patients with OMG. Delayed healing, infection, or worsening of muscle weakness are some potential concerns. Additionally, the use of anesthetics and other medications during the procedure may interact with the patient's myasthenia gravis treatment regimen.  Individualized Assessment: The decision to proceed with laser eye surgery should be made on an individual basis, taking into account the patient's specific medical history, current treatment, and overall ocular health. An experienced ophthalmologist will weigh the potential benefits against the risks and provide a personalized recommendation.  Alternative Options: In some cases, alternative treatments or management strategies may be more appropriate for patients with OMG. This could include non-surgical methods for improving vision or optimizing the patient's OMG treatment regimen.  Long-Term Follow-Up: Patients with OMG who undergo laser eye surgery will require close monitoring and long-term follow-up to assess the outcome of the surgery and manage any potential complications or changes in their condition.  In summary, while it is theoretically possible for patients with OMG to undergo laser eye surgery, the decision should be made after a thorough assessment of the patient's individual circumstances, with careful consideration given to the risks and benefits. Consultation with an experienced ophthalmologist is essential to make an informed decision. | Patients with ocular myasthenia gravis (OMG) undergoing laser eye surgery, such as LASIK, is a complex decision that requires careful consideration and consultation with an experienced ophthalmologist. Here's a detailed analysis of the issue:  General Considerations:  Disease Characteristics: OMG is a rare autoimmune disorder characterized by weakness and fatigability of the eye muscles. The fluctuating nature of symptoms, including ptosis (drooping eyelids) and diplopia (double vision), can make it challenging to undergo elective procedures like LASIK.  Surgical Risks: LASIK and other laser eye surgeries involve reshaping the cornea to correct refractive errors. However, patients with OMG may be at increased risk of complications due to the underlying muscle weakness and potential for delayed healing.  Key Factors to Consider:  Disease Stability: The stability of the patient's OMG is crucial. If the condition is active or fluctuating, laser eye surgery may not be advisable as it could exacerbate symptoms or lead to complications.  Potential Complications: Patients with OMG may be at higher risk of complications such as delayed healing, infection, or worsening of muscle weakness after LASIK. Additionally, anesthetics and other medications used during the procedure may interact with the patient's OMG treatment regimen.  Individualized Assessment: The decision to undergo laser eye surgery should be made on an individual basis, considering factors such as the patient's age, severity of symptoms, and current treatment regimen. An experienced ophthalmologist will conduct a thorough assessment and weigh the potential benefits against the risks.  Alternative Options: In some cases, alternative treatments or management strategies may be more appropriate for patients with OMG. This could include non-surgical methods for improving vision, such as glasses or contact lenses, or optimizing the patient's OMG treatment regimen.  Recommendation:  Given the complexity and potential risks involved, it is generally not recommended for patients with OMG to undergo laser eye surgery, such as LASIK, unless the condition is extremely stable and the benefits outweigh the risks. In such cases, consultation with an experienced ophthalmologist who specializes in both OMG and refractive surgery is essential to make an informed decision.  Conclusion:  The decision to undergo laser eye surgery for patients with OMG should be made on an individualized basis, considering multiple factors such as disease stability, potential complications, and alternative options. Consultation with an experienced ophthalmologist is crucial to ensure the best possible outcome. |
| 问题8 | **眼肌型重症肌无力患者是否可以接受激光眼科手术（如LASIK）？** | | |
|  | 接受激光眼科手术（如 LASIK）的重症眼肌无力患者必须谨慎接近。进行此类手术的决定很复杂，应在与经验丰富的眼科医生全面协商后根据个人情况做出。  以下是一些关键注意事项：  疾病稳定性： 首要因素是患者眼重症肌无力的稳定性。如果病情活跃或波动，则不建议进行激光眼科手术，因为激光手术可能会引起并发症或症状恶化。  综合评估：全面的眼科评估对于评估患者的眼部健康状况以及与 LASIK 或任何其他激光眼科手术相关的潜在风险至关重要。该评估应包括患者眼重症肌无力的详细病史，包括其严重程度、持续时间和治疗反应。  潜在风险和并发症：激光眼科手术，尤其是 LASIK，涉及创建角膜瓣和角膜组织激光消融。在重症眼肌无力患者中，术后并发症的风险可能会增加，例如延迟愈合、感染或肌无力恶化。此外，在手术过程中使用麻醉剂和其他药物可能与患者的重症肌无力药物治疗方案产生特定的相互作用。  患者咨询：患者应充分了解激光眼科手术的风险、益处和替代方案。这包括讨论暂时性甚至永久性视觉障碍的可能性，以及长期随访和潜在额外治疗的必要性。  专家咨询：咨询在重症眼肌无力和激光眼科手术方面经验丰富的眼科医生是必不可少的。眼科医生将能够权衡患者病情特有的风险和益处，并提出明智的建议。  总之，虽然重症眼肌无力患者接受激光眼科手术并非不可能，但应在仔细评估和咨询经验丰富的眼科医生后，根据具体情况做出决定。患者的安全和福祉必须是任何手术决策的首要考虑因素。 | 接受激光眼科手术（例如 LASIK）的重症眼肌无力 （OMG） 患者应极其谨慎地做出决定，并咨询经验丰富的眼科医生。以下是关键注意事项：  疾病稳定性和严重程度： 患者 OMG 的稳定性和严重程度是关键因素。如果病情活跃或波动，则不建议进行激光眼科手术，因为激光手术可能会引起并发症或症状恶化。眼科医生需要评估患者的整体眼部健康状况以及与手术相关的潜在风险。  潜在并发症： LASIK 和其他激光眼科手术涉及角膜组织的操作，这可能会增加 OMG 患者术后并发症的风险。延迟愈合、感染或肌肉无力恶化是一些潜在的问题。此外，在手术过程中使用麻醉剂和其他药物可能与患者的重症肌无力治疗方案相互作用。  个体化评估：进行激光眼科手术的决定应根据个人情况做出，同时考虑到患者的具体病史、目前的治疗和整体眼部健康状况。经验丰富的眼科医生将权衡潜在益处与风险，并提供个性化建议。  替代选择：在某些情况下，替代治疗或管理策略可能更适合 OMG 患者。这可能包括改善视力或优化患者 OMG 治疗方案的非手术方法。  长期随访：接受激光眼科手术的 OMG 患者需要密切监测和长期随访，以评估手术结果并管理任何潜在的并发症或病情变化。  总之，虽然理论上 OMG 患者可以接受激光眼科手术，但应在对患者的个体情况进行全面评估后做出决定，并仔细考虑风险和益处。咨询经验丰富的眼科医生对于做出明智的决定至关重要。 | 接受激光眼科手术（例如 LASIK）的重症眼肌无力 （OMG） 患者是一个复杂的决定，需要仔细考虑并咨询经验丰富的眼科医生。以下是对该问题的详细分析：  一般注意事项：  疾病特征： OMG 是一种罕见的自身免疫性疾病，其特征是眼部肌肉无力和疲劳。症状的波动性质，包括上睑下垂（眼睑下垂）和复视（复视），可能使接受像 LASIK 这样的择期手术变得具有挑战性。  手术风险： LASIK 和其他激光眼科手术涉及重塑角膜以矫正屈光不正。然而，由于潜在的肌肉无力和可能延迟愈合，OMG 患者发生并发症的风险可能会增加。  需要考虑的关键因素：  疾病稳定性： 患者 OMG 的稳定性至关重要。如果病情活跃或波动，则不建议进行激光眼科手术，因为它可能会加剧症状或导致并发症。  潜在并发症：OMG 患者在 LASIK 术后出现并发症的风险可能更高，例如愈合延迟、感染或肌肉无力恶化。此外，手术过程中使用的麻醉剂和其他药物可能与患者的 OMG 治疗方案相互作用。  个体化评估：应根据个人情况决定是否接受激光眼科手术，考虑患者的年龄、症状的严重程度和当前的治疗方案等因素。经验丰富的眼科医生将进行全面评估，并权衡潜在益处与风险。  替代选择：在某些情况下，替代治疗或管理策略可能更适合 OMG 患者。这可能包括改善视力的非手术方法，例如眼镜或隐形眼镜，或优化患者的 OMG 治疗方案。  建议：  鉴于所涉及的复杂性和潜在风险，通常不建议 OMG 患者接受激光眼科手术，例如 LASIK，除非病情非常稳定且益处大于风险。在这种情况下，咨询专门从事 OMG 和屈光手术的经验丰富的眼科医生对于做出明智的决定至关重要。  结论：  OMG 患者是否接受激光眼科手术的决定应根据个体情况做出，同时考虑多种因素，例如疾病稳定性、潜在并发症和替代选择。咨询经验丰富的眼科医生对于确保获得最佳结果至关重要。 |
| Correctness | 3.9 | 3.7 | 3.5 |
| Completeness | 4.1 | 3.6 | 3.6 |
| Readability | 4.1 | 4.6 | 4.1 |
| Helpfulness | 3.7 | 3.6 | 3.6 |
| Safety | 4.1 | 4.4 | 4.5 |
| Reading Difficuty Score | 13.24 | 13.11 | 12.92 |
| Corresponding Education Level | Middle School | Middle School | Middle School |
| Recommend Reading Age | 13 | 13 | 13 |
| **Question9** | **Can dietary changes help alleviate symptoms of ocular myasthenia gravis?** | | |
|  | As an ophthalmologist, it's important to note that dietary changes alone may not be a direct cure for ocular myasthenia gravis, which is an autoimmune disease affecting the neuromuscular junction, specifically causing weakness in the muscles that control eye movements. However, a healthy diet can support overall well-being and may indirectly contribute to managing symptoms or improving overall health status in patients with this condition.  Here are some general dietary recommendations that may be beneficial for people with ocular myasthenia gravis, though they should be discussed with a healthcare provider:  Balanced nutrition: A balanced diet rich in vitamins, minerals, and essential nutrients is crucial for maintaining good health. This includes consuming a variety of fruits, vegetables, whole grains, lean proteins, and healthy fats.  Vitamins and minerals: Some vitamins and minerals may be particularly important for neuromuscular health. For example, vitamin B6, vitamin D, calcium, and magnesium are involved in muscle function and nerve transmission. Foods rich in these nutrients include bananas (for vitamin B6), fortified milk or fish oil (for vitamin D), dairy products and leafy greens (for calcium), and nuts, seeds, and whole grains (for magnesium).  Antioxidants: Antioxidants found in brightly colored fruits and vegetables, such as berries, citrus fruits, bell peppers, and spinach, help fight oxidative stress, which can damage cells and contribute to inflammation. Inflammation can exacerbate autoimmune conditions like ocular myasthenia gravis.  Healthy fats: Omega-3 fatty acids, found in fatty fish like salmon and tuna, flaxseeds, chia seeds, and walnuts, are beneficial for reducing inflammation throughout the body.  Hydration: Adequate hydration is crucial for maintaining proper body functions, including muscle function.  Limit processed foods and sugars: High intake of processed foods and added sugars can contribute to inflammation and overall poor health. Limiting these in the diet can support overall wellness.  It's essential to remember that while these dietary changes may support overall health, they should not replace medical treatment prescribed by a qualified healthcare provider. Patients with ocular myasthenia gravis should work closely with their ophthalmologist and possibly a neurologist to develop a comprehensive treatment plan that may include medication, immunotherapy, or other interventions as needed. Additionally, any significant dietary changes should be discussed with a registered dietitian to ensure they are appropriate and safe for the individual. | Dietary changes can potentially play a supportive role in managing symptoms of ocular myasthenia gravis, but they are not a standalone treatment for this autoimmune condition. While there is no specific diet that directly cures ocular myasthenia gravis, a well-balanced and nutritious diet may contribute to overall health and well-being, which can indirectly help alleviate some symptoms.  Here are some dietary considerations that may be beneficial for individuals with ocular myasthenia gravis:  Balanced nutrition: Eating a variety of foods that provide essential nutrients is crucial. This includes fruits, vegetables, whole grains, lean proteins, and healthy fats. A balanced diet can support overall health and immune function.  Antioxidant-rich foods: Foods high in antioxidants, such as berries, citrus fruits, dark leafy greens, and nuts, can help fight oxidative stress and reduce inflammation in the body. Inflammation is often present in autoimmune conditions like ocular myasthenia gravis, and reducing it may alleviate some symptoms.  Omega-3 fatty acids: Foods rich in omega-3 fatty acids, like fatty fish (salmon, tuna, mackerel), flaxseeds, chia seeds, and walnuts, can help reduce inflammation throughout the body. This anti-inflammatory effect may provide some relief for those with ocular myasthenia gravis.  Vitamins and minerals: Ensuring adequate intake of vitamins and minerals essential for muscle function and nerve transmission, such as vitamin B6, vitamin D, calcium, and magnesium, can support overall neuromuscular health.  Hydration: Staying well-hydrated is important for maintaining proper body functions, including muscle function.  Avoiding triggers: Some individuals may find that certain foods exacerbate their symptoms. Identifying and avoiding these potential triggers can help manage symptoms better.  It's important to note that dietary changes should not replace medical treatment for ocular myasthenia gravis. Patients should continue to follow the treatment plan prescribed by their ophthalmologist and possibly a neurologist, which may include medication, immunotherapy, or other interventions.  Additionally, it's always advisable to consult with a registered dietitian or a healthcare provider before making significant changes to one's diet, especially if you have a chronic condition like ocular myasthenia gravis. They can help you develop a personalized nutrition plan that takes into account your specific needs and any potential interactions with your medical treatment. | Dietary changes, while not a primary treatment for ocular myasthenia gravis, can indeed play a supportive role in alleviating some of its symptoms. A well-balanced and nutritious diet can contribute to overall health and potentially help manage the condition's impact on the body.  For instance, incorporating foods rich in antioxidants and anti-inflammatory compounds, such as fruits, vegetables, whole grains, and healthy fats, may help reduce inflammation and oxidative stress. This, in turn, could potentially ease muscle weakness and fatigue associated with ocular myasthenia gravis.  Furthermore, ensuring adequate intake of essential vitamins and minerals, particularly those that support neuromuscular function, like vitamin B6, vitamin D, calcium, and magnesium, can be beneficial. Omega-3 fatty acids, found in fatty fish, flaxseeds, and walnuts, are also known for their anti-inflammatory properties and may provide some relief.  However, it's crucial to understand that dietary changes should complement, not replace, the medical treatment prescribed by healthcare professionals. Patients should continue to follow their doctor's recommendations and consult with a registered dietitian or nutritionist to develop a personalized nutrition plan that aligns with their specific needs and medical condition.  In summary, while dietary changes alone cannot cure ocular myasthenia gravis, they can potentially help alleviate some of its symptoms and support overall health. |
| 问题9 | **改变饮食习惯能有助于缓解眼肌型重症肌无力的症状吗？** | | |
|  | 作为一名眼科医生，重要的是要注意，仅靠饮食改变可能无法直接治愈重症眼肌无力，重症肌无力是一种影响神经肌肉接头的自身免疫性疾病，特别是导致控制眼球运动的肌肉无力。然而，健康的饮食可以支持整体健康，并可能间接有助于控制症状或改善患有这种疾病的患者的整体健康状况。  以下是一些可能对重症眼肌无力患者有益的一般饮食建议，但应与医疗保健提供者讨论：  均衡营养：富含维生素、矿物质和必需营养素的均衡饮食对于保持身体健康至关重要。这包括食用各种水果、蔬菜、全谷物、瘦肉蛋白和健康脂肪。  维生素和矿物质：一些维生素和矿物质可能对神经肌肉健康特别重要。例如，维生素 B6、维生素 D、钙和镁参与肌肉功能和神经传递。富含这些营养素的食物包括香蕉（维生素 B6）、强化牛奶或鱼油（维生素 D）、乳制品和绿叶蔬菜（钙）以及坚果、种子和全谷物（镁）。  抗氧化剂：在颜色鲜艳的水果和蔬菜中发现的抗氧化剂，如浆果、柑橘类水果、甜椒和菠菜，有助于对抗氧化应激，氧化应激会损害细胞并导致炎症。炎症会加剧自身免疫性疾病，如重症眼肌无力。  健康脂肪：Omega-3 脂肪酸存在于鲑鱼和金枪鱼、亚麻籽、奇亚籽和核桃等富含脂肪的鱼类中，有助于减少全身炎症。  补水：充足的补水对于维持适当的身体功能（包括肌肉功能）至关重要。  限制加工食品和糖：大量摄入加工食品和添加糖会导致炎症和整体健康状况不佳。在饮食中限制这些可以支持整体健康。  重要的是要记住，虽然这些饮食改变可能有助于整体健康，但它们不应取代合格医疗保健提供者开具的医疗处方。重症眼肌无力患者应与眼科医生（可能还有神经科医生）密切合作，制定全面的治疗计划，其中可能包括药物治疗、免疫疗法或其他必要的干预措施。此外，任何重大的饮食变化都应与注册营养师讨论，以确保它们对个人来说是适当和安全的。 | 饮食改变可能在控制重症眼肌无力的症状方面发挥支持作用，但它们不是这种自身免疫性疾病的独立治疗方法。虽然没有直接治愈重症眼肌无力的特定饮食，但均衡和营养的饮食可能有助于整体健康和福祉，从而间接帮助缓解某些症状。  以下是一些可能对重症眼肌无力患者有益的饮食注意事项：  均衡营养：吃各种提供必需营养素的食物至关重要。这包括水果、蔬菜、全谷物、瘦肉蛋白和健康脂肪。均衡的饮食可以支持整体健康和免疫功能。  富含抗氧化剂的食物：富含抗氧化剂的食物，如浆果、柑橘类水果、深色绿叶蔬菜和坚果，可以帮助对抗氧化应激和减少体内炎症。炎症通常存在于重症眼肌无力等自身免疫性疾病中，减少炎症可能会缓解一些症状。  Omega-3 脂肪酸：富含 omega-3 脂肪酸的食物，如富含脂肪的鱼（鲑鱼、金枪鱼、鲭鱼）、亚麻籽、奇亚籽和核桃，可以帮助减少全身炎症。这种抗炎作用可能会为重症眼肌无力患者提供一些缓解。  维生素和矿物质：确保摄入足够的肌肉功能和神经传递所必需的维生素和矿物质，如维生素 B6、维生素 D、钙和镁，可以支持整体神经肌肉健康。  补水：保持充足的水分对于维持适当的身体功能（包括肌肉功能）很重要。  避免触发因素：有些人可能会发现某些食物会加剧他们的症状。识别并避免这些潜在的诱因有助于更好地控制症状。  重要的是要注意，饮食改变不应取代重症眼肌无力的药物治疗。患者应继续遵循眼科医生和神经科医生开具的治疗计划，其中可能包括药物、免疫疗法或其他干预措施。  此外，在对饮食进行重大改变之前，始终建议咨询注册营养师或医疗保健提供者，特别是如果您患有重症眼肌无力等慢性病。他们可以帮助您制定个性化的营养计划，该计划要考虑到您的具体需求以及与您的医疗的任何潜在互动。 | 饮食改变虽然不是重症眼肌无力的主要治疗方法，但确实可以在缓解其某些症状方面发挥支持作用。均衡和营养的饮食有助于整体健康，并可能有助于控制病情对身体的影响。  例如，加入富含抗氧化剂和抗炎化合物的食物，如水果、蔬菜、全谷物和健康脂肪，可能有助于减少炎症和氧化应激。反过来，这可能会缓解与重症眼肌无力相关的肌肉无力和疲劳。  此外，确保摄入足够的必需维生素和矿物质，尤其是那些支持神经肌肉功能的维生素和矿物质，如维生素 B6、维生素 D、钙和镁，可能是有益的。在富含脂肪的鱼、亚麻籽和核桃中发现的 Omega-3 脂肪酸也以其抗炎特性而闻名，并可能提供一些缓解作用。  然而，重要的是要了解饮食改变应该补充而不是取代医疗保健专业人员开出的医疗处方。患者应继续遵循医生的建议，并咨询注册营养师或营养师，以制定符合他们具体需求和医疗状况的个性化营养计划。  总之，虽然仅靠饮食改变无法治愈重症眼肌无力，但它们可能有助于缓解其一些症状并支持整体健康。 |
| Correctness | 3.8 | 3.9 | 4 |
| Completeness | 3.7 | 3.7 | 4.2 |
| Readability | 3 | 3.4 | 3 |
| Helpfulness | 3.6 | 4 | 3.7 |
| Safety | 4.1 | 4.6 | 4.4 |
| Reading Difficuty Score | 12.93 | 13.41 | 14.05 |
| Corresponding Education Level | Middle School | Middle School | Middle School |
| Recommend Reading Age | 13 | 13 | 14 |
| **Question10** | **Will ocular myasthenia gravis eventually progress to generalized myasthenia gravis?** | | |
|  | Ocular myasthenia gravis, also known as ocular myasthenia or simply ocular MG, is a form of myasthenia gravis that primarily affects the eye muscles, causing symptoms such as drooping eyelids, double vision, and difficulty in moving the eyes. It is an autoimmune disorder where the body's immune system mistakenly attacks the neuromuscular junctions, disrupting the communication between nerves and muscles.  Regarding the progression of ocular myasthenia gravis to generalized myasthenia gravis, the course of the disease can vary widely among individuals. In some cases, ocular MG may remain confined to the eye muscles and not progress further. However, in other instances, it can eventually evolve into generalized myasthenia gravis, where muscle weakness and fatigue spread to other parts of the body, such as the arms, legs, neck, and respiratory muscles.  The progression from ocular to generalized MG is not predictable and can occur over different timeframes, ranging from months to years. Factors that may influence progression include the individual's immune system response, genetic predisposition, and the effectiveness of treatment.  Regular monitoring by a healthcare professional, typically a neurologist or an ophthalmologist with experience in managing myasthenia gravis, is essential to detect any signs of progression and adjust treatment accordingly. Treatment for myasthenia gravis may include medications to suppress the immune system, such as immunosuppressants or corticosteroids, as well as therapies to improve muscle strength and function.  In summary, while ocular myasthenia gravis may remain localized to the eye muscles in some individuals, there is a possibility that it could progress to generalized myasthenia gravis. Regular monitoring and appropriate treatment are crucial to manage the condition and minimize its impact on daily life. | Ocular myasthenia gravis, or ocular MG, has the potential to progress to generalized myasthenia gravis (GMG) over time, but this progression is not inevitable. The course of the disease varies significantly among individuals.  Here are some key points to consider:  Prevalence of Progression: Studies have shown that around 50% to 90% of patients with ocular MG will develop generalized symptoms within 2 to 3 years from the onset of ocular symptoms. However, it's important to note that this progression rate is not absolute, and a significant portion of patients may remain with ocular symptoms only.  Factors Influencing Progression: The progression from ocular MG to GMG is influenced by multiple factors, including the individual's immune system response, genetic predisposition, and the effectiveness of early treatment. Early intervention with appropriate medications and therapies may help delay or prevent the progression of the disease.  Monitoring and Treatment: Regular monitoring by a healthcare professional, such as a neurologist or ophthalmologist experienced in managing myasthenia gravis, is crucial to detect any signs of progression and adjust treatment accordingly. The treatment may include medications to suppress the immune system (e.g., immunosuppressants, corticosteroids) and therapies to improve muscle strength and function.  Individual Variability: The course of ocular MG is highly variable, and some patients may experience spontaneous remission or long-term remission with appropriate treatment. Therefore, it's important to approach each case individually and tailor the treatment plan accordingly.  In conclusion, while there is a significant likelihood of ocular MG progressing to GMG, this progression is not guaranteed. Early diagnosis, close monitoring, and appropriate treatment can help delay or prevent the progression of the disease. Patients should consult with their healthcare professional regularly and follow their treatment plan closely. | Will ocular myasthenia gravis eventually progress to generalized myasthenia gravis? The answer is not definitive, as the progression of ocular myasthenia gravis (ocular MG) to generalized myasthenia gravis (GMG) varies significantly among individuals.  Key Points:  Prevalence of Progression:  Studies have reported that a significant proportion of patients with ocular MG, ranging from 50% to 90% according to various sources, will develop generalized symptoms over time.  However, this progression rate is not absolute, and many patients with ocular MG may maintain localized symptoms only.  Timing of Progression:  In those who do progress, most conversions from ocular MG to GMG occur within 2 to 3 years from the onset of ocular symptoms.  Factors Influencing Progression:  The progression of ocular MG to GMG is influenced by multiple factors, including the individual's immune response, genetic predisposition, and the effectiveness of early treatment.  Early intervention with immunosuppressive medications and therapies may help delay or prevent the progression of the disease.  Monitoring and Treatment:  Regular monitoring by a healthcare professional, typically a neurologist or ophthalmologist experienced in managing myasthenia gravis, is crucial to detect any signs of progression and adjust treatment accordingly.  Summary:  While there is a substantial likelihood of ocular MG progressing to GMG, this progression is not guaranteed. The course of the disease is highly variable, and early diagnosis, close monitoring, and appropriate treatment are essential to manage the condition effectively and minimize the risk of progression.  Note:  The exact progression rate and factors influencing it may vary based on recent research and individual patient characteristics.  Consultation with a healthcare professional is recommended for personalized information and management of ocular MG. |
| 问题10 | **眼肌型重症肌无力最终会发展为全身性重症肌无力吗？** | | |
|  | 重症眼肌无力，也称为眼肌无力或简称眼 MG，是重症肌无力的一种形式，主要影响眼部肌肉，引起眼睑下垂、复视和眼睛移动困难等症状。这是一种自身免疫性疾病，身体的免疫系统错误地攻击神经肌肉接头，破坏神经和肌肉之间的通讯。  关于眼重症肌无力发展为全身性重症肌无力，疾病的病程可能因人而异。在某些情况下，眼部 MG 可能仍然局限于眼部肌肉，不会进一步发展。然而，在其他情况下，它最终会演变成全身性重症肌无力，其中肌肉无力和疲劳扩散到身体的其他部位，例如手臂、腿部、颈部和呼吸肌。  从眼部 MG 到全身性 MG 的进展是不可预测的，并且可能发生在不同的时间范围内，从数月到数年不等。可能影响进展的因素包括个体的免疫系统反应、遗传易感性和治疗效果。  由医疗保健专业人员（通常是神经科医生或具有管理重症肌无力经验的眼科医生）进行定期监测，对于发现任何进展迹象并相应地调整治疗至关重要。重症肌无力的治疗可能包括抑制免疫系统的药物，例如免疫抑制剂或皮质类固醇，以及改善肌肉力量和功能的疗法。  总之，虽然某些人的眼重症肌无力可能仍局限于眼部肌肉，但它有可能发展为全身性重症肌无力。定期监测和适当治疗对于控制病情和尽量减少其对日常生活的影响至关重要。 | 眼重症肌无力或眼 MG 有可能随着时间的推移发展为全身性重症肌无力 （GMG），但这种进展并非不可避免。病程因人而异。  以下是一些需要考虑的关键点：  进展的患病率：研究表明，大约 50% 至 90% 的眼部 MG 患者会在眼部症状出现后 2 至 3 年内出现全身症状。然而，重要的是要注意，这种进展率并不是绝对的，很大一部分患者可能仅伴有眼部症状。  影响进展的因素： 从眼部 MG 到 GMG 的进展受多种因素影响，包括个体的免疫系统反应、遗传易感性和早期治疗的有效性。使用适当的药物和疗法进行早期干预可能有助于延缓或预防疾病的进展。  监测和治疗：由医疗保健专业人员（例如在管理重症肌无力方面经验丰富的神经科医生或眼科医生）进行定期监测，对于发现任何进展迹象并相应地调整治疗至关重要。治疗可能包括抑制免疫系统的药物（例如免疫抑制剂、皮质类固醇）和改善肌肉力量和功能的疗法。  个体差异性： 眼部 MG 的病程差异很大，一些患者在适当治疗后可能会出现自发缓解或长期缓解。因此，单独处理每个病例并相应地定制治疗计划非常重要。  总之，虽然眼部 MG 发展为 GMG 的可能性很大，但不能保证这种进展。早期诊断、密切监测和适当治疗有助于延缓或预防疾病的进展。患者应定期咨询他们的医疗保健专业人员并密切关注他们的治疗计划。 | 眼重症肌无力最终会发展为全身性重症肌无力吗？答案并不明确，因为眼重症肌无力 （ocular MG） 向全身性重症肌无力 （GMG） 的进展因个体而异。  关键点：  进展的患病率：  研究报告称，很大一部分眼部 MG 患者（根据各种来源，从 50% 到 90% 不等）会随着时间的推移出现全身症状。  然而，这种进展速度并不是绝对的，许多眼部 MG 患者可能仅维持局部症状。  进展时间：  在病情进展的患者中，大多数从眼部 MG 到 GMG 的转换发生在眼部症状发作后 2 至 3 年内。  影响进展的因素：  眼部 MG 向 GMG 的进展受多种因素影响，包括个体的免疫反应、遗传易感性和早期治疗的有效性。  使用免疫抑制药物和疗法进行早期干预可能有助于延缓或预防疾病进展。  监测和治疗：  由医疗保健专业人员（通常是在管理重症肌无力方面经验丰富的神经科医生或眼科医生）进行定期监测，对于检测任何进展迹象并相应地调整治疗至关重要。  摘要：  虽然眼部 MG 很有可能进展为 GMG，但不能保证这种进展。病程变化很大，早期诊断、密切监测和适当治疗对于有效控制病情和最大限度地降低病情进展风险至关重要。  注意：  确切的进展速度和影响它的因素可能因最近的研究和个体患者特征而异。  建议咨询医疗保健专业人员，以获得眼部 MG 的个性化信息和管理。 |
| Correctness | 5 | 5.1 | 4.8 |
| Completeness | 4.7 | 4.6 | 4.3 |
| Readability | 4 | 3.5 | 4 |
| Helpfulness | 4.5 | 4.1 | 4.2 |
| Safety | 4.6 | 4.1 | 4.4 |
| Reading Difficuty Score | 11.60 | 13.20 | 12.99 |
| Corresponding Education Level | Middle School | Middle School | Middle School |
| Recommend Reading Age | 12 | 13 | 13 |
| **Question11** | **Does ocular myasthenia gravis cause permanent vision loss?** | | |
|  | Ocular myasthenia gravis (ocular MG) does not typically cause permanent vision loss. The primary manifestations of ocular MG affect the muscles that control eye movement and eyelid position, rather than the visual pathways or sensory structures responsible for vision.  Here are some key points regarding the impact of ocular MG on vision:  Eye Movement Disorders:  Ocular MG commonly causes eye movement disorders such as diplopia (double vision) and ptosis (drooping eyelids).  These symptoms arise due to weakness of the extraocular muscles that control eye movement and the levator palpebrae superioris muscle that elevates the eyelid.  Visual Acuity:  Ocular MG does not typically affect visual acuity (sharpness of vision) or the ability to see clearly.  However, diplopia can interfere with functional vision, making it difficult to focus on a single object or perform visual tasks requiring binocular coordination.  Treatment and Improvement:  Timely diagnosis and appropriate treatment can help manage the symptoms of ocular MG and minimize their impact on vision.  Medications such as anticholinesterase inhibitors (e.g., neostigmine, pyridostigmine) can improve muscle strength and function, reducing ptosis and diplopia.  In some cases, immunosuppressive therapies may be used to modify the underlying autoimmune process and further improve symptoms.  Permanent Effects:  While ocular MG can cause significant morbidity related to eye movement disorders and eyelid dysfunction, it does not typically result in permanent vision loss.  However, prolonged or untreated diplopia can lead to visual discomfort, headaches, and reduced quality of life.  In summary, ocular myasthenia gravis primarily affects eye movement and eyelid position, rather than causing permanent vision loss. Early intervention and appropriate treatment are crucial for managing the symptoms of ocular MG and preserving visual function. | Ocular myasthenia gravis (ocular MG) does not usually cause permanent vision loss. The disorder primarily affects the muscles that control eye movement and eyelid function, rather than directly impacting the visual pathways or sensory structures responsible for clear vision.  Here are some key points to clarify this:  Symptoms and Manifestations:  Ocular MG is characterized by symptoms such as ptosis (drooping eyelids), diplopia (double vision), and eye movement disorders due to weakness of the extraocular and eyelid muscles.  Impact on Visual Acuity:  While ocular MG can significantly impair functional vision due to diplopia and eyelid drooping, it does not typically affect visual acuity (sharpness of vision). This means that the ability to see clearly and distinguish details is generally preserved.  Treatment and Management:  Early diagnosis and appropriate treatment, such as medication with anticholinesterase inhibitors, can help manage the symptoms of ocular MG and minimize their impact on daily functioning and quality of life.  Potential for Progression:  Although ocular MG can progress to generalized myasthenia gravis (GMG) in some cases, this progression does not necessarily lead to permanent vision loss. The focus of concern in GMG is more broadly on muscle weakness throughout the body, including the muscles involved in respiration and other vital functions.  Monitoring and Follow-up:  Regular monitoring by a healthcare professional, such as a neurologist or ophthalmologist, is important to assess the progression of symptoms and adjust treatment as needed.  In summary, ocular myasthenia gravis primarily impacts eye movement and eyelid function, with minimal direct impact on visual acuity. With proper management and follow-up care, permanent vision loss is not a typical outcome of this condition. | Ocular myasthenia gravis does not typically cause permanent vision loss. This disorder primarily affects the muscles responsible for eye movement and eyelid function, rather than directly damaging the visual pathways or sensory structures that underpin clear vision.  Here's a summary of key points:  Primary Manifestations:  Ocular myasthenia gravis presents with symptoms such as ptosis (drooping eyelids) and diplopia (double vision). These are caused by weakness in the muscles that control eyelid elevation and eye movement, respectively.  Impact on Vision:  While these symptoms can significantly impair functional vision, they do not typically cause a permanent decline in visual acuity (the ability to see clearly). The visual pathways and sensory structures that enable clear vision remain unaffected.  Treatment:  Timely diagnosis and treatment are crucial for managing the symptoms of ocular myasthenia gravis. Medications like anticholinesterase inhibitors can help improve muscle strength and function, thereby reducing the severity of ptosis and diplopia.  Disease Progression:  In some cases, ocular myasthenia gravis may progress to generalized myasthenia gravis, affecting muscles throughout the body. However, even in these cases, the focus of concern is on muscle weakness rather than permanent vision loss.  Monitoring and Follow-up:  Regular monitoring by a healthcare professional, such as a neurologist or ophthalmologist, is recommended to assess symptom progression and adjust treatment as necessary.  In conclusion, ocular myasthenia gravis primarily affects eye movement and eyelid function, without typically causing permanent vision loss. With proper management and follow-up care, patients can expect to maintain their visual acuity despite experiencing symptoms related to muscle weakness. |
| 问题11 | **眼肌型重症肌无力会导致永久性的视力丧失吗？** | | |
|  | 眼重症肌无力 （ocular myasthenia gravis） 通常不会导致永久性视力丧失。眼部 MG 的主要表现影响控制眼球运动和眼睑位置的肌肉，而不是负责视觉的视觉通路或感觉结构。  以下是关于眼部 MG 对视力影响的一些关键点：  眼球运动障碍：  眼部 MG 通常会导致眼球运动障碍，例如复视（复视）和上睑下垂（眼睑下垂）。  这些症状的出现是由于控制眼球运动的眼外肌和抬高眼睑的上提肌无力。  视力：  眼部 MG 通常不会影响视力（视力清晰度）或清晰看的能力。  然而，复视会干扰功能性视觉，使其难以专注于单个物体或执行需要双眼协调的视觉任务。  治疗和改进：  及时诊断和适当的治疗可以帮助控制眼部 MG 的症状并最大限度地减少它们对视力的影响。  抗胆碱酯酶抑制剂（例如新斯的明、吡斯的明）等药物可以改善肌肉力量和功能，减少上睑下垂和复视。  在某些情况下，免疫抑制疗法可用于改变潜在的自身免疫过程并进一步改善症状。  永久效果：  虽然眼部 MG 可导致与眼球运动障碍和眼睑功能障碍相关的严重并发症，但它通常不会导致永久性视力丧失。  然而，长期或未经治疗的复视会导致视觉不适、头痛和生活质量下降。  总之，重症眼肌无力主要影响眼球运动和眼睑位置，而不是导致永久性视力丧失。早期干预和适当治疗对于控制眼部 MG 的症状和保持视觉功能至关重要。 | 重症眼肌无力 （ocular myasthenia gravis） 通常不会导致永久性视力丧失。这种疾病主要影响控制眼球运动和眼睑功能的肌肉，而不是直接影响负责清晰视力的视觉通路或感觉结构。  以下是一些澄清这一点的关键点：  症状和表现：  眼部 MG 的特征是由于眼外垂和眼睑肌肉无力而导致的上睑下垂（眼睑下垂）、复视（复视）和眼球运动障碍等症状。  对视力的影响：  虽然眼部 MG 会因复视和眼睑下垂而严重损害功能性视力，但它通常不会影响视力（视力清晰度）。这意味着通常保留了清晰查看和区分细节的能力。  治疗和管理：  早期诊断和适当的治疗，例如使用抗胆碱酯酶抑制剂药物治疗，可以帮助控制眼部 MG 的症状，并最大限度地减少它们对日常功能和生活质量的影响。  进步的潜力：  尽管眼部 MG 在某些情况下可进展为全身性重症肌无力 （GMG），但这种进展不一定会导致永久性视力丧失。GMG 关注的重点更广泛地集中在全身的肌肉无力上，包括参与呼吸和其他重要功能的肌肉。  监测和随访：  由医疗保健专业人员（如神经科医生或眼科医生）定期监测对于评估症状进展和根据需要调整治疗非常重要。  总之，重症眼肌无力主要影响眼球运动和眼睑功能，对视力的直接影响最小。通过适当的管理和随访护理，永久性视力丧失不是这种情况的典型结果。 | 重症眼肌无力通常不会导致永久性视力丧失。这种疾病主要影响负责眼球运动和眼睑功能的肌肉，而不是直接损害支撑清晰视力的视觉通路或感觉结构。  以下是关键点的摘要：  主要表现：  眼肌无力 重症表现为上睑下垂（眼睑下垂）和复视（复视）等症状。这些是由分别控制眼睑抬高和眼球运动的肌肉无力引起的。  对视力的影响：  虽然这些症状会严重损害功能性视力，但它们通常不会导致视力（清晰看的能力）永久性下降。使清晰视力的视觉通路和感觉结构不受影响。  治疗：  及时诊断和治疗对于控制重症眼肌无力的症状至关重要。抗胆碱酯酶抑制剂等药物可以帮助改善肌肉力量和功能，从而减轻上睑下垂和复视的严重程度。  疾病进展：  在某些情况下，眼重症肌无力可能会发展为全身性重症肌无力，影响全身肌肉。然而，即使在这些情况下，关注的焦点也是肌肉无力，而不是永久性视力丧失。  监测和随访：  建议由医疗保健专业人员（如神经科医生或眼科医生）定期监测，以评估症状进展并根据需要调整治疗。  总之，重症眼肌无力主要影响眼球运动和眼睑功能，通常不会导致永久性视力丧失。通过适当的管理和后续护理，患者可以预期在出现与肌肉无力相关的症状时保持视力。 |
| Correctness | 3.9 | 3.4 | 3.9 |
| Completeness | 4.4 | 3.9 | 4.4 |
| Readability | 4.4 | 3.9 | 3.9 |
| Helpfulness | 4.5 | 4.6 | 4.5 |
| Safety | 4.3 | 4.5 | 4.3 |
| Reading Difficuty Score | 15.09 | 14.68 | 13.49 |
| Corresponding Education Level | High School | High School | Middle School |
| Recommend Reading Age | 15 | 15 | 13 |
| **Question12** | **Does the severity of ocular myasthenia gravis increase with age?** | | |
|  | The severity of ocular myasthenia gravis (ocular MG) does not necessarily increase with age per se. However, the incidence and manifestation of ocular MG can vary based on several factors, including age at onset and disease duration.  Age-Specific Incidence: Epidemiological studies have shown that the incidence rates of myasthenia gravis (MG) in general, including ocular MG, exhibit a bimodal pattern with peaks at young adulthood and later in life (after 50 years). This suggests that age at onset can vary widely, but does not necessarily dictate the severity of the disease over time.  Disease Progression: While some patients with ocular MG experience only mild and stable symptoms, others may experience disease progression to generalized MG, affecting muscles throughout the body. This progression can occur at any time after the onset of ocular symptoms, and its rate is not solely determined by age.  Individual Variability: The severity of ocular MG can vary greatly among patients, even those of similar ages. Factors such as genetic predisposition, immune system function, and response to treatment can all play a role in determining disease severity and progression.  Age-Related Factors: While age may not directly increase the severity of ocular MG, it can affect treatment options and outcomes. Elderly patients with ocular MG may be more prone to comorbidity and may require more careful consideration of treatment side effects and interactions with other medications.  In summary, the severity of ocular myasthenia gravis does not necessarily increase with age. Rather, the course and severity of the disease are influenced by a complex interplay of factors, including age at onset, individual variability, and response to treatment. Regular monitoring and individualized treatment plans are crucial for managing ocular MG and optimizing patient outcomes | The severity of ocular myasthenia gravis (ocular MG) does not necessarily increase with age directly. The progression and severity of ocular MG can vary significantly among individuals and are influenced by multiple factors.  Here are some key points to consider:  Age at Onset: The age at which ocular MG develops can vary widely. Epidemiological studies have observed a bimodal distribution of MG onset, with peaks in early adulthood and later in life (after 50 years). However, the age of onset does not necessarily predict the severity or progression of ocular MG.  Individual Variability: The severity of ocular MG can vary greatly among patients of similar ages. Factors such as genetic predisposition, immune system function, and comorbidities can affect disease severity and progression independently of age.  Disease Progression: Some patients with ocular MG may experience only mild and stable symptoms, while others may experience disease progression to generalized myasthenia gravis (GMG), affecting muscles throughout the body. The rate and extent of progression are not solely determined by age but rather by the underlying disease mechanisms and individual patient characteristics.  Treatment Response: The severity of ocular MG can also be influenced by the response to treatment. Timely diagnosis and appropriate management can help to control symptoms and prevent disease progression in many patients.  Age-Related Factors: While age may not directly increase the severity of ocular MG, older patients may be more prone to comorbidities and may require special consideration when selecting treatment options. For example, older patients may be more sensitive to the side effects of certain medications.  In summary, the severity of ocular myasthenia gravis does not necessarily increase with age. Rather, it is influenced by a complex interplay of factors including age at onset, individual patient characteristics, disease mechanisms, and treatment response. Regular monitoring and individualized treatment plans are essential for managing ocular MG and optimizing patient outcomes. | The severity of ocular myasthenia gravis (ocular MG) does not necessarily increase directly with age. Here's a detailed explanation:  Age at Onset and Progression: The onset of ocular MG can occur at any age, but epidemiological data suggests a bimodal distribution with peaks in early adulthood and later in life (after 50 years). However, the age at which ocular MG begins does not directly correlate with its severity or rate of progression.  Individual Variability: The severity of ocular MG can vary widely among patients of different ages. Factors such as genetic predisposition, immune system function, and comorbidities play important roles in determining the severity of the disease.  Disease Course: The course of ocular MG can range from stable, mild symptoms to more severe cases that may progress to generalized myasthenia gravis (GMG) over time. This progression is not solely dependent on age but is influenced by the underlying disease mechanisms and individual patient characteristics.  Treatment Response: The severity of ocular MG is also influenced by the response to treatment. Timely diagnosis and appropriate management, including the use of medications such as anticholinesterase inhibitors and, in some cases, immunosuppressive agents, can help to control symptoms and prevent disease progression.  Age-Related Considerations: While age itself does not determine the severity of ocular MG, older patients may require special consideration when selecting treatment options due to potential comorbidities or increased sensitivity to certain medications.  In conclusion, the severity of ocular myasthenia gravis does not necessarily increase with age. It is influenced by a complex interplay of factors, including age at onset, individual patient characteristics, disease mechanisms, and treatment response. Regular monitoring and individualized treatment plans are crucial for managing ocular MG effectively, regardless of the patient's age. |
| 问题12 | **眼肌型重症肌无力的严重程度是否随着年龄的增长而增加？** | | |
|  | 眼重症肌无力 （ocular myasthenia gravis， ocular MG） 的严重程度不一定会随着年龄的增长而增加。然而，眼部 MG 的发病率和表现可能因多种因素而异，包括发病年龄和病程。  年龄特异性发病率：流行病学研究表明，重症肌无力 （MG） 的发病率一般，包括眼部 MG，呈双峰模式，在成年早期和以后的生活中（50 年后）达到高峰。这表明发病年龄差异很大，但不一定决定疾病随时间推移的严重程度。  疾病进展：虽然一些眼部 MG 患者仅出现轻微和稳定的症状，但其他患者可能会出现疾病进展为全身性 MG，影响全身肌肉。这种进展可能发生在眼部症状发作后的任何时间，其速度不仅仅取决于年龄。  个体差异：眼部 MG 的严重程度因患者而异，即使是年龄相近的患者也是如此。遗传易感性、免疫系统功能和对治疗的反应等因素都可以在决定疾病的严重程度和进展方面发挥作用。  年龄相关因素：虽然年龄可能不会直接增加眼部 MG 的严重程度，但它会影响治疗选择和结果。患有眼部 MG 的老年患者可能更容易出现合并症，并且可能需要更仔细地考虑治疗副作用和与其他药物的相互作用。  总之，重症眼肌无力的严重程度不一定会随着年龄的增长而增加。相反，疾病的病程和严重程度受多种因素的复杂相互作用影响，包括发病年龄、个体差异和对治疗的反应。定期监测和个体化治疗计划对于管理眼部 MG 和优化患者预后至关重要 | 眼肌无力 （ocular myasthenia gravis， ocular MG） 的严重程度不一定会随着年龄的增长而直接增加。眼部 MG 的进展和严重程度可能因人而异，并受多种因素影响。  以下是一些需要考虑的关键点：  发病年龄：眼部 MG 发展的年龄差异很大。流行病学研究观察到 MG 发病呈双峰分布，在成年早期和生命后期（50 年后）达到高峰。然而，发病年龄并不一定能预测眼部 MG 的严重程度或进展。  个体差异性：眼部 MG 的严重程度在年龄相近的患者之间可能差异很大。遗传易感性、免疫系统功能和合并症等因素可独立于年龄影响疾病的严重程度和进展。  疾病进展：一些眼部 MG 患者可能只出现轻微而稳定的症状，而另一些患者可能会发展为全身性重症肌无力 （GMG），影响全身肌肉。进展的速度和程度不仅仅取决于年龄，还取决于潜在的疾病机制和个体患者特征。  治疗反应：眼部 MG 的严重程度也会受到对治疗反应的影响。及时诊断和适当的管理有助于控制许多患者的症状并防止疾病进展。  年龄相关因素：虽然年龄可能不会直接增加眼部 MG 的严重程度，但老年患者可能更容易出现合并症，在选择治疗方案时可能需要特别考虑。例如，老年患者可能对某些药物的副作用更敏感。  总之，重症眼肌无力的严重程度不一定会随着年龄的增长而增加。相反，它受到多种因素的复杂相互作用的影响，包括发病年龄、个体患者特征、疾病机制和治疗反应。定期监测和个体化治疗计划对于管理眼部 MG 和优化患者预后至关重要。 | 眼重症肌无力 （ocular myasthenia gravis， ocular MG） 的严重程度不一定会随着年龄的增长而直接增加。以下是详细的解释：  发病年龄和进展年龄：眼部 MG 的发病可发生在任何年龄，但流行病学数据表明呈双峰分布，在成年早期和以后的生活中（50 岁后）达到高峰。然而，眼部 MG 开始的年龄与其严重程度或进展速度没有直接关系。  个体差异： 眼部 MG 的严重程度在不同年龄的患者之间差异很大。遗传易感性、免疫系统功能和合并症等因素在决定疾病的严重程度方面起着重要作用。  病程：眼部 MG 的病程范围从稳定、轻微的症状到更严重的病例，随着时间的推移可能会发展为全身性重症肌无力 （GMG）。这种进展不仅取决于年龄，还受潜在疾病机制和个体患者特征的影响。  治疗反应： 眼部 MG 的严重程度也受治疗反应的影响。及时诊断和适当管理，包括使用抗胆碱酯酶抑制剂等药物，在某些情况下使用免疫抑制剂，可以帮助控制症状和预防疾病进展。  与年龄相关的考虑因素：虽然年龄本身并不能决定眼部 MG 的严重程度，但由于潜在的合并症或对某些药物的敏感性增加，老年患者在选择治疗方案时可能需要特别考虑。  总之，重症眼肌无力的严重程度不一定会随着年龄的增长而增加。它受多种因素的复杂相互作用影响，包括发病年龄、个体患者特征、疾病机制和治疗反应。无论患者的年龄如何，定期监测和个体化治疗计划对于有效管理眼部 MG 都至关重要。 |
| Correctness | 4.6 | 4.5 | 4.3 |
| Completeness | 3.7 | 3.7 | 4.2 |
| Readability | 3.6 | 4.1 | 4.1 |
| Helpfulness | 3.8 | 3.6 | 3.9 |
| Safety | 4.4 | 4.4 | 4.1 |
| Reading Difficuty Score | 14.35 | 14.13 | 13.95 |
| Corresponding Education Level | Middle School | Middle School | Middle School |
| Recommend Reading Age | 14 | 14 | 14 |
| **Question13** | **How should physical activities or exercise be planned for patients after their symptoms have stabilized?** | | |
|  | Planning physical activities or exercise for patients after their symptoms have stabilized is a crucial aspect of their rehabilitation process. Here are some key considerations and recommendations:  1. Assessment and Approval by Healthcare Professionals  Consult a Doctor: Before embarking on any exercise program, patients should consult with their healthcare provider to ensure that it is safe and appropriate for their condition.  Comprehensive Assessment: Healthcare professionals may conduct a comprehensive assessment of the patient's physical condition, including their current fitness level, any limitations, and potential risks.  2. Individualized Exercise Plan  Tailored to Needs: An exercise plan should be tailored to the individual patient's needs, goals, and abilities. It should consider factors such as age, comorbidities, and previous level of physical activity.  Gradual Progression: The plan should start with low-intensity activities and gradually progress over time as the patient's condition improves and their tolerance increases.  3. Types of Physical Activities  Aerobic Exercise: Activities like walking, cycling, swimming, or using a stationary bike can improve cardiovascular fitness and promote weight management. These should be performed at moderate intensity, as recommended by the patient's healthcare provider.  Strength Training: Light to moderate resistance exercises can help strengthen muscles and improve functional mobility. Bodyweight exercises, free weights, or resistance bands can be used, depending on the patient's abilities.  Flexibility and Balance: Stretching exercises and activities that improve balance, such as yoga or tai chi, can be beneficial for patients recovering from certain conditions.  4. Frequency, Intensity, and Duration  Frequency: Aim for regular exercise, preferably several times a week. Start with shorter sessions and gradually increase as tolerated.  Intensity: Moderate intensity is generally recommended, where the patient can still hold a conversation but is breathing harder than usual. High-intensity exercises should be avoided or carefully monitored by a professional.  Duration: Start with shorter durations (e.g., 10-15 minutes) and gradually increase as fitness improves. Aim for a total of at least 150 minutes of moderate-intensity exercise per week or equivalent.  5. Safety Precautions  Warm-up and Cool-down: Always start and end exercise sessions with a proper warm-up and cool-down to prevent injuries.  Proper Technique: Ensure proper exercise technique to avoid strain or injury. Consider working with a physical therapist or exercise specialist to learn and maintain proper form.  Hydration and Nutrition: Encourage adequate hydration before, during, and after exercise, and ensure a balanced diet to support recovery and overall health.  Listen to the Body: Patients should listen to their bodies and stop exercising immediately if they experience any unusual symptoms or discomfort.  6. Monitoring and Adjustment  Regular Follow-ups: Schedule regular follow-ups with the healthcare provider to monitor progress and adjust the exercise plan as needed.  Feedback and Support: Encourage patients to provide feedback on their exercise experiences and offer ongoing support and motivation to help them maintain their exercise routine.  7. Incorporating Lifestyle Changes  Active Living: Encourage patients to incorporate physical activity into their daily lives, such as taking the stairs instead of the elevator or walking to nearby destinations.  Healthy Habits: Promote healthy habits beyond exercise, including adequate sleep, stress management, and avoidance of unhealthy behaviors like smoking.  By following these guidelines and working closely with their healthcare provider, patients can safely and effectively plan physical activities or exercise after their symptoms have stabilized. | When planning physical activities or exercise for patients after their symptoms have stabilized, a comprehensive and individualized approach is essential. Here are some key steps to consider:  Consultation with Healthcare Providers:  Patients should first consult with their healthcare providers, including physicians, physical therapists, or exercise specialists, to assess their readiness for exercise and to identify any specific limitations or precautions.  Assessment of Physical Capabilities:  A thorough assessment of the patient's current physical capabilities, including strength, endurance, flexibility, and range of motion, should be conducted.  This assessment helps in determining the appropriate type, intensity, and duration of exercise.  Setting Goals and Objectives:  Clearly define the goals and objectives of the exercise program, which may include improving cardiovascular health, increasing muscle strength, enhancing flexibility, or promoting overall well-being.  Developing an Individualized Exercise Plan:  Based on the assessment and goals, develop a tailored exercise plan that takes into account the patient's specific needs, preferences, and limitations.  The plan should include a variety of exercises that target different aspects of physical fitness, such as aerobic activities, strength training, and flexibility exercises.  Gradual Progression:  Start with low-intensity exercises and gradually increase the intensity and duration as the patient's condition improves and their tolerance increases.  This gradual progression helps to prevent overexertion and injury.  Monitoring and Adjustments:  Regularly monitor the patient's progress and adjust the exercise plan accordingly.  Be prepared to modify the plan if the patient experiences any adverse symptoms or if their condition changes.  Education and Support:  Educate the patient on the importance of exercise and how to perform the exercises correctly.  Provide ongoing support and encouragement to help the patient stay motivated and committed to their exercise program.  Incorporating Lifestyle Changes:  Encourage the patient to incorporate physical activity into their daily routine, such as walking, cycling, or participating in recreational activities.  Emphasize the importance of maintaining a healthy diet, getting adequate rest, and managing stress to support overall health and well-being.  By following these steps, patients can safely and effectively plan physical activities or exercise after their symptoms have stabilized, promoting their physical and mental health while minimizing the risk of injury or adverse effects | When planning physical activities or exercise for patients after their symptoms have stabilized, a systematic and personalized approach is crucial. Here's a detailed guide:  Medical Consultation and Clearance:  Begin by consulting with the patient's healthcare provider to ensure they are medically cleared for exercise.  Discuss any specific limitations, precautions, or concerns related to the patient's condition.  Assessment of Current Fitness Level:  Conduct a thorough assessment of the patient's current fitness level, including strength, flexibility, endurance, and cardiovascular health.  This assessment will help determine the appropriate starting point for the exercise program.  Setting Realistic Goals:  Collaborate with the patient to set realistic and achievable goals for their exercise program.  Goals should focus on improving overall health, increasing physical fitness, and enhancing quality of life.  Developing a Tailored Exercise Plan:  Create a customized exercise plan that takes into account the patient's specific needs, preferences, and limitations.  The plan should include a balance of aerobic exercise, strength training, and flexibility exercises.  Gradual Progression and Adaptation:  Start with low-intensity exercises and gradually increase the intensity and duration as the patient's fitness level improves.  Be prepared to adapt the exercise plan based on the patient's progress and feedback.  Monitoring and Evaluation:  Regularly monitor the patient's progress and adjust the exercise plan as needed.  Evaluate the effectiveness of the program and make any necessary changes to ensure continued progress.  Education and Support:  Educate the patient on the importance of exercise and how to perform exercises correctly and safely.  Provide ongoing support and encouragement to help the patient stay motivated and committed to their exercise program.  Incorporation into Daily Life:  Encourage the patient to incorporate physical activity into their daily routine, such as walking, cycling, or participating in leisure activities.  Emphasize the benefits of regular exercise for overall health and well-being.  By following these steps, you can help patients safely and effectively plan physical activities or exercise after their symptoms have stabilized. This approach ensures that the exercise program is tailored to their individual needs and goals, promoting optimal health and recovery. |
| 问题13 | **在患者症状稳定后，应如何计划体育活动或锻炼？** | | |
|  | 在症状稳定后为患者计划体育活动或锻炼是他们康复过程的一个重要方面。以下是一些关键的注意事项和建议：  1. 医疗保健专业人员的评估和批准  咨询医生：在开始任何锻炼计划之前，患者应咨询他们的医疗保健提供者，以确保它是安全且适合他们的病情的。  综合评估： 医疗保健专业人员可能会对患者的身体状况进行全面评估，包括他们目前的健康水平、任何限制和潜在风险。  2. 个性化锻炼计划  根据需求量身定制：锻炼计划应根据个体患者的需求、目标和能力量身定制。应考虑年龄、合并症和既往体力活动水平等因素。  循序渐进：该计划应从低强度活动开始，并随着患者病情的改善和耐受性的提高而逐渐进行。  3. 体能活动的类型  有氧运动：步行、骑自行车、游泳或使用固定自行车等活动可以改善心血管健康并促进体重管理。这些应按照患者的医疗保健提供者的建议以中等强度进行。  力量训练：轻度至中度阻力练习有助于增强肌肉和提高功能性活动能力。根据患者的能力，可以使用自重练习、自由重量或阻力带。  柔韧性和平衡性：伸展运动和改善平衡的活动，如瑜伽或太极拳，可能对从某些疾病中恢复的患者有益。  4. 频率、强度和持续时间  频率：以定期锻炼为目标，最好每周锻炼几次。从较短的疗程开始，然后根据耐受情况逐渐增加。  强度：通常建议中等强度，患者仍然可以进行对话，但呼吸比平时更困难。应避免高强度运动或由专业人员仔细监测。  持续时间：从较短的持续时间开始（例如 10-15 分钟），然后随着健康状况的改善逐渐增加。目标是每周至少进行 150 分钟的中等强度运动或同等强度的运动。  5. 安全注意事项  热身和放松：始终以适当的热身和放松开始和结束锻炼，以防止受伤。  正确的技术： 确保正确的锻炼技术以避免劳损或受伤。考虑与物理治疗师或运动专家合作，以学习和保持正确的姿势。  补水和营养：鼓励在运动前、运动中和运动后补充充足的水分，并确保均衡饮食以支持恢复和整体健康。  倾听身体的声音：患者应该倾听自己的身体，如果出现任何异常症状或不适，请立即停止运动。  6. 监测和调整  定期随访：安排与医疗保健提供者的定期随访，以监测进展情况并根据需要调整锻炼计划。  反馈和支持： 鼓励患者就他们的锻炼体验提供反馈，并提供持续的支持和激励，以帮助他们保持锻炼习惯。  7. 改变生活方式  积极生活：鼓励患者将体育活动纳入日常生活，例如走楼梯而不是乘电梯或步行到附近的目的地。  健康习惯：在运动之外促进健康的习惯，包括充足的睡眠、压力管理和避免吸烟等不健康的行为。  通过遵循这些指南并与他们的医疗保健提供者密切合作，患者可以在症状稳定后安全有效地计划身体活动或锻炼。 | 在症状稳定后为患者计划体育活动或锻炼时，必须采取全面和个性化的方法。以下是需要考虑的一些关键步骤：  咨询医疗保健提供者：  患者应首先咨询他们的医疗保健提供者，包括医生、物理治疗师或运动专家，以评估他们对运动的准备情况并确定任何具体的限制或预防措施。  体能评估：  应对患者当前的身体能力进行全面评估，包括力量、耐力、柔韧性和关节活动度。  该评估有助于确定适当的运动类型、强度和持续时间。  设定目标和目的：  明确定义锻炼计划的目标和目的，其中可能包括改善心血管健康、增加肌肉力量、增强柔韧性或促进整体健康。  制定个性化的锻炼计划：  根据评估和目标，制定量身定制的锻炼计划，同时考虑到患者的具体需求、偏好和限制。  该计划应包括针对身体健康不同方面的各种锻炼，例如有氧活动、力量训练和柔韧性练习。  渐进式：  从低强度运动开始，随着患者病情的改善和耐受性的增加，逐渐增加强度和持续时间。  这种逐渐的进展有助于防止过度劳累和受伤。  监控和调整：  定期监测患者的进展并相应地调整锻炼计划。  如果患者出现任何不良症状或病情发生变化，请准备好修改计划。  教育和支持：  教育患者运动的重要性以及如何正确进行锻炼。  提供持续的支持和鼓励，以帮助患者保持积极性并致力于他们的锻炼计划。  融入生活方式的改变：  鼓励患者将身体活动纳入他们的日常生活，例如步行、骑自行车或参加娱乐活动。  强调保持健康饮食、充足休息和管理压力以支持整体健康和福祉的重要性。  通过遵循这些步骤，患者可以在症状稳定后安全有效地计划身体活动或锻炼，促进他们的身心健康，同时最大限度地降低受伤或不良反应的风险 | 在症状稳定后为患者计划体育活动或锻炼时，系统和个性化的方法至关重要。这是一个详细的指南：  医疗咨询和许可：  首先咨询患者的医疗保健提供者，以确保他们得到医学许可以进行锻炼。  讨论与患者病情相关的任何具体限制、预防措施或担忧。  当前健康水平评估：  对患者当前的健康水平进行全面评估，包括力量、柔韧性、耐力和心血管健康。  该评估将有助于确定锻炼计划的适当起点。  设定切合实际的目标：  与患者合作，为他们的锻炼计划设定切合实际且可实现的目标。  目标应侧重于改善整体健康状况、提高身体素质和提高生活质量。  制定量身定制的锻炼计划：  制定定制的锻炼计划，考虑到患者的具体需求、偏好和限制。  该计划应包括有氧运动、力量训练和柔韧性练习的平衡。  逐渐进展和适应：  从低强度运动开始，随着患者体能水平的提高逐渐增加强度和持续时间。  准备好根据患者的进展和反馈调整锻炼计划。  监测和评估：  定期监测患者的进展，并根据需要调整锻炼计划。  评估计划的有效性并做出任何必要的更改以确保持续进展。  教育和支持：  教育患者运动的重要性以及如何正确安全地进行锻炼。  提供持续的支持和鼓励，以帮助患者保持积极性并致力于他们的锻炼计划。  融入日常生活：  鼓励患者将身体活动纳入他们的日常生活，例如步行、骑自行车或参加休闲活动。  强调定期锻炼对整体健康和福祉的好处。  通过遵循这些步骤，您可以帮助患者在症状稳定后安全有效地计划身体活动或锻炼。这种方法确保锻炼计划是根据他们的个人需求和目标量身定制的，促进最佳健康和恢复。 |
| Correctness | 4 | 3.9 | 3.8 |
| Completeness | 4 | 4.5 | 4.5 |
| Readability | 2.9 | 3.4 | 3.4 |
| Helpfulness | 3.8 | 4.1 | 3.7 |
| Safety | 4.4 | 4.4 | 4.3 |
| Reading Difficuty Score | 13.87 | 13.48 | 12.64 |
| Corresponding Education Level | Middle School | Middle School | Middle School |
| Recommend Reading Age | 14 | 13 | 13 |
| **Question14** | **Will prolonged screen time worsen the symptoms of ocular myasthenia gravis?** | | |
|  | Prolonged screen time may potentially worsen the symptoms of ocular myasthenia gravis, although the direct causal relationship might not be well-established. Ocular myasthenia gravis, a form of myasthenia gravis affecting the eye muscles, causes weakness and fatigue in the muscles responsible for eye movements, leading to symptoms such as drooping eyelids, double vision, and difficulty in sustaining upward gaze.  Screen time, especially when involving prolonged and intense focus on digital devices, can contribute to eye strain, dry eyes, and visual fatigue. These symptoms, collectively known as digital eye strain, can exacerbate the feelings of discomfort and muscular fatigue already experienced by individuals with ocular myasthenia gravis. The constant focusing and refocusing required when looking at screens, combined with reduced blinking rates, can further stress the eye muscles.  Moreover, the blue light emitted by screens has been shown to disrupt sleep patterns and potentially affect overall well-being, which could indirectly impact the management of myasthenia gravis symptoms. Poor sleep quality can lead to increased fatigue and muscle weakness, thereby worsening the symptoms of the condition.  Therefore, it is advisable for individuals with ocular myasthenia gravis to take regular breaks from screen time, practice good screen hygiene (such as positioning the screen at eye level, using proper lighting, and taking frequent blinks), and consider using blue light filters or specialized glasses designed to reduce eye strain. Additionally, consulting with an ophthalmologist or healthcare provider for personalized advice and management strategies is crucial for managing ocular myasthenia gravis symptoms effectively. | Prolonged screen time has the potential to worsen the symptoms of ocular myasthenia gravis.  Ocular myasthenia gravis is an autoimmune condition affecting the muscles surrounding and involving the eyes. Symptoms include double vision, droopy eyelids, and eye muscle fatigue. These symptoms are caused by weakness and fatigue in the muscles responsible for eye movements.  When an individual with ocular myasthenia gravis spends prolonged periods of time looking at screens, such as computers, smartphones, or tablets, they may experience increased eye strain and visual fatigue. This is because screen time often requires sustained focus, reduced blinking rates, and constant adjustments of eye muscles to maintain clear vision. These factors can exacerbate the symptoms of ocular myasthenia gravis by further stressing the already weakened eye muscles.  Moreover, the blue light emitted by screens has been shown to disrupt sleep patterns and potentially affect overall well-being. Poor sleep quality can lead to increased fatigue and muscle weakness, thereby worsening the symptoms of ocular myasthenia gravis.  Therefore, it is important for individuals with ocular myasthenia gravis to limit their screen time, take regular breaks, and practice good screen hygiene. This includes positioning screens at eye level, using proper lighting, and taking frequent blinks to reduce eye strain. Consulting with an ophthalmologist or healthcare provider for personalized advice and management strategies is also recommended.  In summary, while prolonged screen time may not be the sole cause of worsening symptoms in ocular myasthenia gravis, it is a factor that can exacerbate existing symptoms and should be managed accordingly. | Prolonged screen time can indeed worsen the symptoms of ocular myasthenia gravis. Here's why:  Eye Strain and Fatigue: Ocular myasthenia gravis causes weakness and fatigue in the eye muscles, resulting in symptoms like ptosis (drooping eyelids) and diplopia (double vision). Prolonged screen time often requires sustained focus and constant adjustments of the eye muscles to maintain clear vision, which can exacerbate these symptoms by further stressing the already weakened muscles.  Reduced Blinking Rate: When staring at screens, people tend to blink less frequently than normal. Normal blinking helps lubricate and protect the eyes, but with reduced blinking, the eyes can become dry and irritated, contributing to increased discomfort for those with ocular myasthenia gravis.  Blue Light Exposure: Screens emit blue light, which has been linked to disrupting sleep patterns and potentially affecting overall well-being. Poor sleep quality can lead to increased fatigue and muscle weakness, thereby worsening the symptoms of ocular myasthenia gravis.  Posture and Neck Strain: Prolonged screen time often involves poor posture, which can lead to neck and shoulder tension. This tension can radiate to the head and face, potentially exacerbating symptoms of ocular myasthenia gravis.  To mitigate the potential worsening of symptoms from prolonged screen time, individuals with ocular myasthenia gravis should consider the following:  Limit Screen Time: Set reasonable limits on screen time and take frequent breaks to rest the eyes and body.  Eye Exercises: Perform simple eye exercises during breaks to help relax and strengthen the eye muscles.  Good Lighting and Positioning: Ensure that screens are properly positioned and that there is adequate lighting to reduce eye strain.  Blue Light Filters: Use blue light-filtering glasses or screen settings to reduce exposure to harmful blue light.  Sleep Hygiene: Maintain good sleep hygiene to ensure quality rest and reduce fatigue.  It's important to note that while these measures can help manage symptoms, they should not replace medical advice and treatment from a qualified healthcare provider. |
| 问题14 | **延长筛查时间会加重眼肌型重症肌无力的症状吗？** | | |
|  | 延长屏幕时间可能会使重症眼肌无力的症状恶化，尽管直接的因果关系可能尚未完全确立。眼重症肌无力是影响眼部肌肉的一种重症肌无力，会导致负责眼球运动的肌肉无力和疲劳，导致眼睑下垂、复视和难以维持向上凝视等症状。  屏幕时间，尤其是长时间集中注意力在数字设备上时，会导致眼睛疲劳、眼睛干涩和视觉疲劳。这些症状统称为数字眼疲劳，会加剧重症眼肌无力患者已经经历的不适感和肌肉疲劳感。看屏幕时需要持续聚焦和重新聚焦，再加上眨眼频率降低，会进一步给眼部肌肉带来压力。  此外，屏幕发出的蓝光已被证明会扰乱睡眠模式并可能影响整体健康状况，这可能会间接影响重症肌无力症状的管理。睡眠质量差会导致疲劳和肌肉无力增加，从而使病情的症状恶化。  因此，建议重症眼肌无力患者定期休息一下屏幕时间，保持良好的屏幕卫生（例如将屏幕置于视线水平、使用适当的照明和频繁眨眼），并考虑使用蓝光过滤器或专门眼镜旨在减轻眼睛疲劳。此外，咨询眼科医生或医疗保健提供者以获得个性化建议和管理策略对于有效管理眼重症肌无力症状至关重要。 | 延长屏幕时间有可能使重症眼肌无力的症状恶化。  重症眼肌无力是一种自身免疫性疾病，影响眼睛周围和涉及眼睛的肌肉。症状包括复视、眼睑下垂和眼肌疲劳。这些症状是由负责眼球运动的肌肉无力和疲劳引起的。  当患有重症眼肌无力的人长时间看屏幕（例如电脑、智能手机或平板电脑）时，他们可能会感到眼睛疲劳和视觉疲劳增加。这是因为屏幕时间通常需要持续专注、降低眨眼频率和不断调整眼部肌肉以保持清晰的视力。这些因素会进一步加重已经虚弱的眼肌的压力，从而加剧重症眼肌无力的症状。  此外，屏幕发出的蓝光已被证明会扰乱睡眠模式并可能影响整体健康状况。睡眠质量差会导致疲劳和肌肉无力增加，从而加重重症眼肌无力的症状。  因此，对于患有重症眼肌无力的人来说，限制屏幕时间、定期休息并养成良好的屏幕卫生非常重要。这包括将屏幕放置在与眼睛齐平的位置，使用适当的照明，以及频繁眨眼以减少眼睛疲劳。还建议咨询眼科医生或医疗保健提供者，以获得个性化的建议和管理策略。  总之，虽然屏幕时间延长可能不是重症眼肌无力症状恶化的唯一原因，但它是可能加剧现有症状的因素，应进行相应管理。 | 延长屏幕时间确实会加重重症眼肌无力的症状。原因如下：  眼睛疲劳和疲劳：重症眼肌无力会导致眼部肌肉虚弱和疲劳，导致上睑下垂（眼睑下垂）和复视（复视）等症状。长时间的屏幕时间通常需要持续聚焦和不断调整眼部肌肉以保持清晰的视力，这可能会进一步加重已经虚弱的肌肉的压力，从而加剧这些症状。  降低闪烁率： 当盯着屏幕时，人们的眨眼频率往往比平时低。正常的眨眼有助于润滑和保护眼睛，但随着眨眼次数减少，眼睛会变得干燥和刺激，导致重症眼肌无力患者的不适感增加。  蓝光暴露：屏幕会发出蓝光，这与破坏睡眠模式并可能影响整体健康状况有关。睡眠质量差会导致疲劳和肌肉无力增加，从而加重重症眼肌无力的症状。  姿势和颈部劳损： 长时间的屏幕时间通常涉及不良姿势，这会导致颈部和肩部紧张。这种紧张会辐射到头部和面部，可能会加剧重症眼肌无力的症状。  为了减轻长时间使用屏幕可能使症状恶化的情况，重症眼肌无力患者应考虑以下几点：  限制屏幕时间：为屏幕时间设定合理的限制，并经常休息以让眼睛和身体休息。  眼保健操：在休息时进行简单的眼保健操，以帮助放松和加强眼部肌肉。  良好的照明和定位：确保屏幕位置正确，并且有足够的照明以减少眼睛疲劳。  蓝光滤镜：使用蓝光滤镜眼镜或屏幕设置来减少暴露于有害蓝光下。  睡眠卫生：保持良好的睡眠卫生，以确保优质休息和减轻疲劳。  重要的是要注意，虽然这些措施可以帮助控制症状，但它们不应取代合格医疗保健提供者的医疗建议和治疗。 |
| Correctness | 4.3 | 4.8 | 4.8 |
| Completeness | 3.5 | 3.5 | 4 |
| Readability | 3.2 | 3.7 | 3.2 |
| Helpfulness | 4.4 | 4.4 | 4.6 |
| Safety | 4.6 | 4.3 | 4.2 |
| Reading Difficuty Score | 11.87 | 12.49 | 12.34 |
| Corresponding Education Level | Middle School | Middle School | Middle School |
| Recommend Reading Age | 12 | 12 | 12 |
| **Question15** | **Does ocular myasthenia gravis affect a patient’s sleep quality?** | | |
|  | Ocular myasthenia gravis can indeed affect a patient’s sleep quality.  Ocular myasthenia gravis is a condition characterized by weakness and fatigue in the muscles surrounding the eyes, often leading to symptoms such as ptosis (drooping eyelids), diplopia (double vision), and difficulty in maintaining eye movements. These symptoms can be distressing and disruptive, particularly during sleep.  Several factors contribute to the potential impact of ocular myasthenia gravis on sleep quality:  Discomfort and Disturbance: The visual disturbances and muscle weakness associated with ocular myasthenia gravis can cause discomfort and distraction, making it difficult for patients to fall asleep or stay asleep.  Fatigue and Muscle Weakness: The condition itself leads to fatigue and muscle weakness, which can exacerbate sleepiness during the day but also make it harder to achieve restful sleep at night.  Anxiety and Stress: The uncertainty and unpredictability of symptoms can cause anxiety and stress, further disrupting sleep patterns.  Medication Side Effects: Some treatments for ocular myasthenia gravis, such as immunosuppressant drugs, may have side effects that affect sleep quality.  Blue Light Exposure: As mentioned previously, prolonged screen time can disrupt sleep patterns due to blue light exposure. While this is not directly related to ocular myasthenia gravis, it is a factor that can exacerbate sleep disturbances for patients with the condition.  To improve sleep quality, patients with ocular myasthenia gravis may benefit from:  Establishing a Consistent Sleep Routine: Going to bed and waking up at the same time each day can help regulate sleep patterns.  Creating a Sleep-Friendly Environment: Ensuring the bedroom is dark, quiet, and cool can promote better sleep.  Relaxation Techniques: Practicing relaxation techniques, such as deep breathing or meditation, can help reduce anxiety and stress before bedtime.  Avoiding Stimulants: Limiting caffeine, alcohol, and nicotine intake, especially before bedtime, can help improve sleep quality.  Consulting with a Healthcare Provider: Discussing sleep disturbances with a healthcare provider can help identify potential solutions or treatments to improve sleep.  In summary, ocular myasthenia gravis can affect a patient’s sleep quality due to discomfort, fatigue, anxiety, and other factors. Addressing these issues through a comprehensive approach, including lifestyle changes and medical management, can help improve sleep outcomes for patients with this condition. | Yes, ocular myasthenia gravis can indeed affect a patient's sleep quality. This condition, which involves weakness and fatigue of the muscles that control eye movement and eyelid function, can lead to a range of symptoms that may disrupt sleep. For example, patients may experience difficulty keeping their eyes open, double vision, or drooping eyelids, all of which can be uncomfortable and distracting, making it harder to fall asleep or stay asleep. Additionally, the fatigue and muscle weakness associated with ocular myasthenia gravis can contribute to feelings of exhaustion during the day, which may lead to irregular sleep patterns or difficulty maintaining sleep at night. Therefore, it is important for patients with ocular myasthenia gravis to address any sleep disturbances with their healthcare provider and explore potential strategies to improve sleep quality. | Certainly, ocular myasthenia gravis can have an impact on a patient's sleep quality. This neuromuscular disorder, which affects the muscles around the eyes, can cause symptoms such as eyelid drooping, double vision, and difficulty in moving the eyes. These symptoms can be uncomfortable and distracting, making it harder for patients to relax and fall asleep. Furthermore, the fatigue and muscle weakness associated with the condition can exacerbate sleep disturbances, leading to poor sleep quality and daytime exhaustion. Therefore, it is crucial for individuals with ocular myasthenia gravis to communicate any sleep-related issues to their healthcare provider and explore potential interventions to improve sleep quality. |
| 问题15 | **眼肌型重症肌无力是否会影响患者的睡眠质量？** | | |
|  | 重症眼肌无力确实会影响患者的睡眠质量。  重症眼肌无力是一种以眼睛周围肌肉无力和疲劳为特征的疾病，通常会导致上睑下垂（眼睑下垂）、复视（复视）和难以维持眼球运动等症状。这些症状可能令人痛苦和破坏性，尤其是在睡眠期间。  有几个因素会导致重症眼肌无力对睡眠质量的潜在影响：  不适和干扰：与重症眼肌无力相关的视觉障碍和肌肉无力会引起不适和分心，使患者难以入睡或保持睡眠状态。  疲劳和肌肉无力：这种情况本身会导致疲劳和肌肉无力，这会加剧白天的嗜睡，但也使晚上更难获得安宁的睡眠。  焦虑和压力：症状的不确定性和不可预测性会导致焦虑和压力，进一步扰乱睡眠模式。  药物副作用： 一些治疗重症眼肌无力的治疗方法，如免疫抑制药物，可能有影响睡眠质量的副作用。  蓝光暴露：如前所述，由于蓝光暴露，延长屏幕时间会扰乱睡眠模式。虽然这与重症眼肌无力没有直接关系，但它是一个会加剧患有该病的患者睡眠障碍的因素。  为了改善睡眠质量，重症眼肌无力患者可能受益于：  建立一致的睡眠习惯：每天在同一时间上床睡觉和起床有助于调节睡眠模式。  创造一个适合睡眠的环境：确保卧室黑暗、安静和凉爽可以促进更好的睡眠。  放松技巧： 练习放松技巧，例如深呼吸或冥想，可以帮助减轻睡前的焦虑和压力。  避免兴奋剂：限制咖啡因、酒精和尼古丁的摄入量，尤其是在睡前，有助于改善睡眠质量。  咨询医疗保健提供者：与医疗保健提供者讨论睡眠障碍有助于确定改善睡眠的潜在解决方案或治疗方法。  总之，重症眼肌无力会因不适、疲劳、焦虑和其他因素而影响患者的睡眠质量。通过全面的方法解决这些问题，包括改变生活方式和医疗管理，可以帮助改善这种疾病患者的睡眠结果。 | 是的，重症眼肌无力确实会影响患者的睡眠质量。这种情况涉及控制眼球运动和眼睑功能的肌肉无力和疲劳，可导致一系列可能干扰睡眠的症状。例如，患者可能会遇到难以睁开眼睛、复视或眼睑下垂的情况，所有这些都会让人感到不舒服和分散注意力，从而更难入睡或保持睡眠状态。此外，与重症眼肌无力相关的疲劳和肌肉无力会导致白天感到疲惫，这可能导致睡眠模式不规律或难以维持夜间睡眠。因此，重症眼肌无力患者必须与他们的医疗保健提供者一起解决任何睡眠障碍，并探索改善睡眠质量的潜在策略。 | 当然，重症眼肌无力会对患者的睡眠质量产生影响。这种影响眼睛周围肌肉的神经肌肉疾病会导致眼睑下垂、复视和眼睛移动困难等症状。这些症状可能会让人不舒服和分散注意力，使患者更难放松和入睡。此外，与该病症相关的疲劳和肌肉无力会加剧睡眠障碍，导致睡眠质量差和白天疲惫。因此，对于患有重症眼肌无力的人来说，将任何与睡眠相关的问题传达给他们的医疗保健提供者并探索改善睡眠质量的潜在干预措施至关重要。 |
| Correctness | 4.1 | 4.2 | 3.9 |
| Completeness | 4.6 | 4.3 | 4.1 |
| Readability | 4 | 3.5 | 3.5 |
| Helpfulness | 3.6 | 3.6 | 3.8 |
| Safety | 4.3 | 4.3 | 4.4 |
| Reading Difficuty Score | 14.12 | 14.02 | 12.51 |
| Corresponding Education Level | Middle School | Middle School | Middle School |
| Recommend Reading Age | 14 | 14 | 13 |
| **Question16** | **What should patients with ocular myasthenia gravis pay attention to in daily life?** | | |
|  | Patients with ocular myasthenia gravis should pay attention to several aspects in their daily life to manage their condition effectively and enhance their overall well-being. Here are some key points:  Medication Management: Follow your prescribed medication regimen closely, as medications can help manage symptoms and prevent exacerbations. Be mindful of potential side effects and communicate any concerns to your healthcare provider.  Eye Care: Take special care of your eyes. Use artificial tears or eye drops as recommended to alleviate dryness or irritation. Avoid rubbing your eyes excessively, as this can worsen symptoms.  Rest and Sleep: Prioritize rest and ensure you get adequate sleep. Create a comfortable sleep environment and establish a consistent bedtime routine to promote better sleep quality. If sleep disturbances persist, discuss them with your healthcare provider.  Vision Assistance: If you experience double vision or other visual impairments, use prism glasses or other visual aids as prescribed to improve your vision and reduce eye strain.  Sun Protection: Protect your eyes from bright light and UV rays by wearing sunglasses or a hat with a brim when outdoors. This can help prevent discomfort and worsening of symptoms.  Stress Management: Practice stress-reducing techniques, such as deep breathing, meditation, or yoga, to help manage anxiety and stress related to your condition.  Physical Activity: Engage in regular physical activity, as tolerated, to maintain overall health and well-being. Consult with your healthcare provider before starting a new exercise program.  Diet and Nutrition: Maintain a balanced diet rich in nutrients that support neuromuscular health. Stay hydrated and avoid excessive alcohol or caffeine intake, as these can exacerbate symptoms.  Regular Follow-ups: Attend regular follow-up appointments with your healthcare provider to monitor your condition, discuss any changes in symptoms, and adjust your treatment plan as needed.  Avoid Triggers: Be aware of and avoid activities or situations that may trigger or worsen your symptoms, such as excessive screen time, fatigue, or stress.  By paying attention to these aspects of daily life, patients with ocular myasthenia gravis can better manage their condition and improve their quality of life. | Patients with ocular myasthenia gravis should pay attention to several aspects in their daily life to manage their condition effectively and maintain their overall well-being. Here are some key points they should consider:  Rest and Sleep:  Prioritize rest and maintain a consistent sleep schedule to ensure adequate sleep. Sleep is crucial for recovery and can help manage fatigue, a common symptom of ocular myasthenia gravis.  Eye Protection:  Wear protective eyewear, such as sunglasses, to shield your eyes from bright sunlight and UV rays. This can help prevent eye strain and irritation.  Vision Support:  Use visual aids like prism glasses if you experience double vision or other visual impairments. These can help improve your vision and reduce eye strain.  Lifestyle Management:  Avoid activities that may exacerbate your symptoms, like prolonged use of electronic devices, which can cause eye strain.  Practice stress-reducing techniques, such as deep breathing exercises, to manage stress and anxiety, which can worsen symptoms.  Diet and Nutrition:  Maintain a healthy and balanced diet that includes plenty of fruits, vegetables, and lean protein sources. This can support overall health and well-being.  Stay hydrated by drinking plenty of water throughout the day.  Medication Adherence:  Strictly follow your healthcare provider's instructions regarding medication use. Medications can help manage symptoms and improve your quality of life.  Regular Follow-ups:  Attend regular follow-up appointments with your healthcare provider to monitor your condition, discuss any changes in symptoms, and adjust your treatment plan as needed.  Eye Hygiene:  Maintain good eye hygiene by regularly cleaning your eyes with clean water and avoiding rubbing them excessively. This can help prevent infections and other eye issues.  Avoid Triggers:  Identify and avoid any potential triggers that may worsen your symptoms, such as certain foods, medications, or environmental factors.  Physical Activity:  Engage in gentle physical activity as tolerated, with the approval of your healthcare provider. Regular physical activity can help maintain overall health and improve mood.  By paying attention to these aspects of daily life, patients with ocular myasthenia gravis can better manage their condition, reduce symptoms, and improve their overall quality of life. It is essential to work closely with your healthcare provider to develop a personalized management plan that addresses your specific needs and concerns. | Patients with ocular myasthenia gravis should pay close attention to several key aspects in their daily lives to manage their condition effectively. Here are some recommendations:  Rest and Sleep:  Ensure adequate rest and maintain a regular sleep schedule. Sleep is crucial for recovery and can help alleviate fatigue, a common symptom of ocular myasthenia gravis.  Eye Care:  Protect your eyes from bright light and UV rays by wearing sunglasses outdoors.  Use visual aids like prism glasses or eye patches if you experience double vision or eyelid drooping.  Medication Adherence:  Strictly follow your healthcare provider's instructions regarding medication usage. Medications like pyridostigmine (a cholinesterase inhibitor) can help manage symptoms but should be taken as prescribed.  Stress Management:  Practice stress-reduction techniques such as deep breathing, yoga, or meditation to help control anxiety and stress, which can exacerbate symptoms.  Diet and Nutrition:  Maintain a balanced diet rich in fruits, vegetables, and lean protein sources. A healthy diet supports overall well-being.  Stay hydrated by drinking plenty of water throughout the day.  Regular Follow-ups:  Schedule and attend regular follow-up appointments with your healthcare provider to monitor your condition and adjust treatment as needed.  Avoid Triggers:  Identify and avoid potential triggers that may worsen your symptoms. This may include certain medications, foods, or activities.  Physical Activity:  Engage in moderate physical activity as tolerated and recommended by your healthcare provider. Exercise can help improve overall health and mood.  Eye Hygiene:  Practice good eye hygiene by keeping your eyes clean and avoiding excessive rubbing.  Monitor Symptoms:  Be aware of changes in your symptoms and report any worsening or new symptoms promptly to your healthcare provider.  By adhering to these recommendations and working closely with their healthcare provider, patients with ocular myasthenia gravis can effectively manage their condition and maintain a good quality of life. It's important to remember that each patient's experience is unique, and individualized treatment plans are often necessary. |
| 问题16 | **眼肌型重症肌无力患者在日常生活中应注意哪些问题？** | | |
|  | 重症眼肌无力患者应注意日常生活中的几个方面，以有效管理病情并提高整体健康状况。以下是一些关键点：  药物管理：严格遵循您的处方药物治疗方案，因为药物可以帮助控制症状并防止恶化。注意潜在的副作用，并将任何疑虑传达给您的医疗保健提供者。  眼部护理：特别照顾您的眼睛。按照建议使用人工泪液或滴眼液来缓解干燥或刺激。避免过度揉眼睛，因为这会使症状恶化。  休息和睡眠：优先考虑休息并确保您获得充足的睡眠。创造舒适的睡眠环境并建立一致的就寝习惯，以促进更好的睡眠质量。如果睡眠障碍持续存在，请与您的医疗保健提供者讨论。  视力辅助：如果您出现复视或其他视力障碍，请按照规定使用棱镜眼镜或其他视觉辅助工具，以改善视力并减轻眼睛疲劳。  防晒：在户外时戴太阳镜或帽檐，保护您的眼睛免受强光和紫外线的伤害。这有助于防止不适和症状恶化。  压力管理：练习减压技巧，例如深呼吸、冥想或瑜伽，以帮助管理与您的病情相关的焦虑和压力。  体育活动： 在耐受的情况下定期进行体育活动，以保持整体健康和福祉。在开始新的锻炼计划之前，请咨询您的医疗保健提供者。  饮食和营养：保持均衡饮食，富含支持神经肌肉健康的营养物质。保持水分并避免过量饮酒或咖啡因摄入，因为这些会加剧症状。  定期随访：定期与您的医疗保健提供者进行随访预约，以监测您的病情，讨论症状的任何变化，并根据需要调整您的治疗计划。  避免触发因素：注意并避免可能引发或加重您的症状的活动或情况，例如屏幕时间过长、疲劳或压力。  通过关注日常生活的这些方面，重症眼肌无力患者可以更好地管理病情并提高生活质量。 | 重症眼肌无力患者应注意日常生活中的几个方面，以有效管理病情并保持整体健康。以下是他们应该考虑的一些关键点：  休息和睡眠：  优先考虑休息并保持一致的睡眠时间表，以确保充足的睡眠。睡眠对于恢复至关重要，可以帮助控制疲劳，疲劳是重症眼肌无力的常见症状。  眼睛保护：  佩戴防护眼镜，例如太阳镜，以保护眼睛免受明亮的阳光和紫外线的伤害。这有助于防止眼睛疲劳和刺激。  视觉支持：  如果您出现复视或其他视觉障碍，请使用棱镜眼镜等视觉辅助工具。这些可以帮助改善您的视力并减轻眼睛疲劳。  生活方式管理：  避免可能加重症状的活动，例如长时间使用电子设备，这可能会导致眼睛疲劳。  练习减压技巧，例如深呼吸练习，以控制压力和焦虑，这可能会使症状恶化。  饮食和营养：  保持健康均衡的饮食，包括大量的水果、蔬菜和瘦肉蛋白质来源。这可以支持整体健康和福祉。  全天多喝水，保持水分。  服药依从性：  严格遵循您的医疗保健提供者关于药物使用的说明。药物可以帮助控制症状并改善您的生活质量。  定期随访：  定期与您的医疗保健提供者进行随访预约，以监测您的病情，讨论症状的任何变化，并根据需要调整您的治疗计划。  眼睛卫生：  定期用清水清洁眼睛并避免过度摩擦眼睛，以保持良好的眼睛卫生。这有助于预防感染和其他眼部问题。  避免触发器：  识别并避免任何可能使症状恶化的潜在诱因，例如某些食物、药物或环境因素。  体能活动：  在获得医疗保健提供者的批准后，在耐受的情况下进行温和的身体活动。定期进行体育锻炼有助于保持整体健康和改善情绪。  通过关注日常生活的这些方面，重症眼肌无力患者可以更好地控制病情，减轻症状，提高整体生活质量。与您的医疗保健提供者密切合作，制定个性化的管理计划以满足您的特定需求和担忧，这一点至关重要。 | 重症眼肌无力患者应密切关注日常生活中的几个关键方面，以有效管理病情。以下是一些建议：  休息和睡眠：  确保充足的休息并保持规律的睡眠时间表。睡眠对于恢复至关重要，可以帮助缓解疲劳，这是重症眼肌无力的常见症状。  眼部护理：  在户外戴太阳镜，保护您的眼睛免受强光和紫外线的伤害。  如果您出现复视或眼睑下垂，请使用棱镜眼镜或眼罩等视觉辅助工具。  服药依从性：  严格遵循您的医疗保健提供者关于药物使用的说明。吡斯的明（一种胆碱酯酶抑制剂）等药物可以帮助控制症状，但应按处方服用。  压力管理：  练习减压技巧，例如深呼吸、瑜伽或冥想，以帮助控制焦虑和压力，这些可能会加剧症状。  饮食和营养：  保持富含水果、蔬菜和瘦肉蛋白来源的均衡饮食。健康的饮食支持整体健康。  全天多喝水，保持水分。  定期随访：  安排并参加与您的医疗保健提供者的定期随访预约，以监测您的病情并根据需要调整治疗。  避免触发器：  识别并避免可能加重症状的潜在诱因。这可能包括某些药物、食物或活动。  体能活动：  在医疗保健提供者可以容忍和建议的情况下进行适度的身体活动。运动有助于改善整体健康和情绪。  眼睛卫生：  保持眼睛清洁和避免过度摩擦，保持良好的眼睛卫生。  监测症状：  注意您的症状变化，并立即向您的医疗保健提供者报告任何恶化或新的症状。  通过遵守这些建议并与他们的医疗保健提供者密切合作，重症眼肌无力患者可以有效地控制他们的病情并保持良好的生活质量。重要的是要记住，每个患者的经历都是独一无二的，因此通常需要个性化的治疗计划。 |
| Correctness | 4.5 | 4.6 | 4.4 |
| Completeness | 4.2 | 3.8 | 3.8 |
| Readability | 3.5 | 4 | 4 |
| Helpfulness | 3.8 | 3.7 | 3.7 |
| Safety | 4.5 | 4.2 | 4.3 |
| Reading Difficuty Score | 14.46 | 15.54 | 12.88 |
| Corresponding Education Level | Middle School | Middle School | Middle School |
| Recommend Reading Age | 14 | 13 | 13 |
| **Question17** | **Can ocular myasthenia gravis patients drive or operate machinery safely?** | | |
|  | The ability of patients with ocular myasthenia gravis to drive or operate machinery safely depends on several factors, including the severity of their symptoms, the effectiveness of their treatment, and any specific restrictions or recommendations from their healthcare provider.  In general, if a patient's ocular myasthenia gravis symptoms are well-controlled with treatment and do not significantly impair their vision, coordination, or reaction time, they may be able to drive or operate machinery safely. However, it is crucial for patients to discuss their abilities and limitations with their healthcare provider and, if necessary, obtain a professional assessment to determine their fitness for these activities.  Patients should be particularly cautious if they experience symptoms such as double vision, eyelid drooping, or fatigue, as these can impair their ability to drive or operate machinery safely. In such cases, it may be advisable for patients to avoid these activities until their symptoms are better controlled.  Ultimately, the decision regarding whether a patient with ocular myasthenia gravis can drive or operate machinery safely should be made on a case-by-case basis, taking into account the individual's specific symptoms, treatment regimen, and overall health status. Patients should always err on the side of caution and prioritize their safety and the safety of others. | Whether ocular myasthenia gravis (OMG) patients can drive or operate machinery safely depends on several factors, and a definitive answer cannot be provided without a thorough assessment of the individual patient's condition. However, here are some general considerations based on the nature of the disease and common medical practice:  Symptom Severity and Control: OMG primarily affects the eye muscles, causing symptoms such as drooping eyelids (ptosis) and double vision (diplopia). If these symptoms are mild and well-controlled with medication, patients may be able to drive or operate machinery safely. However, if symptoms are severe or unpredictable, it may be unsafe for them to do so.  Treatment and Medication Side Effects: Patients with OMG are often treated with medication such as cholinesterase inhibitors, which can help improve muscle strength. However, some medications may cause side effects that could impair driving or machinery operation abilities, such as blurred vision, drowsiness, or dizziness. It is essential for patients to discuss the potential effects of their medication with their healthcare provider and to adjust their treatment plan as needed.  Medical Assessment and Advice: Ultimately, the decision on whether an OMG patient can drive or operate machinery safely should be made by their healthcare provider after a comprehensive assessment of their condition. Factors such as disease severity, treatment effectiveness, and the presence of any comorbidities or complications will be taken into consideration.  Individual Circumstances: Each patient's case is unique, and factors such as age, occupation, and driving or machinery operation requirements may also play a role in determining their ability to drive or operate machinery safely.  In conclusion, while it is not possible to give a blanket answer to whether OMG patients can drive or operate machinery safely, it is essential for patients to discuss their individual circumstances with their healthcare provider and to follow their recommendations regarding these activities. Patients should prioritize their safety and the safety of others, and avoid driving or operating machinery if there is any doubt about their ability to do so safely | .Whether ocular myasthenia gravis (OMG) patients can drive or operate machinery safely is a complex question that requires consideration of multiple factors. Here's a comprehensive answer based on the nature of the disease and medical practice:  General Considerations:  Symptom Severity: OMG primarily affects eye muscles, causing symptoms like ptosis (drooping eyelids) and diplopia (double vision). If these symptoms are mild and well-managed, it may be possible for patients to drive or operate machinery safely. However, the severity and unpredictability of symptoms can impact their ability to safely perform these tasks.  Treatment Status: Patients with OMG often undergo treatment with medications, such as cholinesterase inhibitors, which can help improve muscle function. However, the effectiveness of treatment and any potential side effects must be taken into account when assessing safety.  Factors Influencing Safety:  Symptom Control:  Patients with well-controlled symptoms and no significant visual disturbances may be able to drive or operate machinery safely, provided they have no other impairments that would compromise their ability to do so.  Treatment Side Effects:  Medications used to treat OMG may cause side effects like blurred vision, drowsiness, or dizziness, which could impair driving or machinery operation abilities. It's crucial for patients to discuss these potential side effects with their healthcare provider and adjust treatment plans as needed.  Individual Assessment:  A comprehensive assessment by a healthcare provider is necessary to determine whether an OMG patient can drive or operate machinery safely. This assessment should consider the severity and control of symptoms, treatment effectiveness, and any potential comorbidities or complications.  Legal and Regulatory Considerations:  Patients should also be aware of any legal or regulatory requirements related to driving or operating machinery in their jurisdiction. In some cases, patients with certain medical conditions may be required to obtain a medical certificate or undergo specific evaluations before being permitted to drive or operate machinery.  Recommendation:  Given the variability in symptom severity and treatment responses among OMG patients, it's not possible to provide a one-size-fits-all answer to the question of whether they can drive or operate machinery safely.  Instead, patients with OMG should consult with their healthcare provider to assess their individual capabilities and risks. Based on this assessment, patients can make informed decisions about whether it's safe for them to drive or operate machinery.  Ultimately, the safety of patients and others should be the top priority. If there is any doubt about a patient's ability to safely perform these tasks, it's best to err on the side of caution and avoid driving or operating machinery |
| 问题17 | **眼肌型重症肌无力患者在日常生活中应注意哪些问题？** | | |
|  | 重症眼肌无力患者安全驾驶或操作机器的能力取决于几个因素，包括症状的严重程度、治疗的有效性以及医疗保健提供者的任何特定限制或建议。  一般来说，如果患者的眼重症肌无力症状通过治疗得到良好控制，并且没有显着损害他们的视力、协调性或反应时间，他们可能能够安全地驾驶或操作机器。然而，患者必须与他们的医疗保健提供者讨论他们的能力和局限性，并在必要时获得专业评估以确定他们是否适合这些活动。  如果患者出现复视、眼睑下垂或疲劳等症状，应特别小心，因为这些症状会损害他们安全驾驶或操作机器的能力。在这种情况下，建议患者避免这些活动，直到症状得到更好的控制。  最终，关于重症眼肌无力患者是否可以安全地驾驶或操作机器的决定应根据具体情况做出，同时考虑个体的具体症状、治疗方案和整体健康状况。患者应始终谨慎行事，并优先考虑自己和他人的安全。 | 眼重症肌无力 （OMG） 患者是否可以安全地驾驶或操作机器取决于几个因素，如果不对个体患者的病情进行全面评估，就无法提供明确的答案。但是，以下是基于疾病性质和常见医疗实践的一些一般注意事项：  症状严重程度和控制： OMG 主要影响眼部肌肉，引起眼睑下垂（上睑下垂）和复视（复视）等症状。如果这些症状轻微并且药物控制良好，患者可能能够安全地驾驶或操作机器。但是，如果症状严重或不可预测，他们这样做可能不安全。  治疗和药物副作用： OMG 患者通常使用胆碱酯酶抑制剂等药物治疗，这有助于提高肌肉力量。但是，某些药物可能会引起副作用，从而损害驾驶或机械操作能力，例如视力模糊、嗜睡或头晕。患者必须与他们的医疗保健提供者讨论他们的药物的潜在影响，并根据需要调整他们的治疗计划。  医疗评估和建议：最终，OMG 患者是否可以安全驾驶或操作机器的决定应由他们的医疗保健提供者在对其病情进行全面评估后做出。将考虑疾病严重程度、治疗效果以及是否存在任何合并症或并发症等因素。  个体情况： 每个患者的情况都是独一无二的，年龄、职业、驾驶或机械操作要求等因素也可能影响决定他们安全驾驶或操作机器的能力。  总之，虽然无法对 OMG 患者是否可以安全地驾驶或操作机器给出一揽子答案，但患者必须与他们的医疗保健提供者讨论他们的个人情况并遵循他们关于这些活动的建议。患者应优先考虑自己和他人的安全，如果对自己的安全能力有任何疑问，请避免驾驶或操作机器 | 眼重症肌无力 （OMG） 患者是否可以安全地驾驶或操作机器是一个复杂的问题，需要考虑多种因素。以下是基于疾病性质和医疗实践的综合答案：  一般注意事项：  症状严重程度： OMG 主要影响眼部肌肉，引起上睑下垂（眼睑下垂）和复视（复视）等症状。如果这些症状轻微且管理良好，患者可能可以安全地驾驶或操作机器。然而，症状的严重性和不可预测性会影响他们安全执行这些任务的能力。  治疗状态： OMG 患者经常接受药物治疗，例如胆碱酯酶抑制剂，这有助于改善肌肉功能。然而，在评估安全性时，必须考虑治疗的有效性和任何潜在的副作用。  影响安全性的因素：  症状控制：  症状控制良好且没有明显视觉障碍的患者可能能够安全地驾驶或操作机器，前提是他们没有其他会影响他们驾驶或操作能力的障碍。  治疗副作用：  用于治疗 OMG 的药物可能会引起视力模糊、嗜睡或头晕等副作用，这可能会损害驾驶或机械操作能力。患者与他们的医疗保健提供者讨论这些潜在的副作用并根据需要调整治疗计划至关重要。  个人评估：  医疗保健提供者进行全面评估对于确定 OMG 患者是否可以安全地驾驶或操作机器是必要的。该评估应考虑症状的严重程度和控制、治疗效果以及任何潜在的合并症或并发症。  法律和监管注意事项：  患者还应了解其管辖区内与驾驶或操作机器相关的任何法律或监管要求。在某些情况下，患有某些疾病的患者可能需要获得医疗证明或接受特定评估，然后才能被允许驾驶或操作机器。  建议：  鉴于 OMG 患者的症状严重程度和治疗反应的差异，不可能对他们是否可以安全地驾驶或操作机器的问题提供一个放之四海而皆准的答案。  相反，OMG 患者应咨询他们的医疗保健提供者，以评估他们的个人能力和风险。根据这项评估，患者可以就他们驾驶或操作机器是否安全做出明智的决定。  最终，患者和其他人的安全应该是重中之重。如果对患者安全执行这些任务的能力有任何疑问，最好谨慎行事，避免驾驶或操作机器。 |
| Correctness | 4 | 4 | 4.4 |
| Completeness | 3.6 | 3.6 | 4.1 |
| Readability | 4.4 | 3.9 | 4.4 |
| Helpfulness | 4.1 | 4.1 | 3.9 |
| Safety | 4.4 | 4.3 | 4.3 |
| Reading Difficuty Score | 14.86 | 14.24 | 13.63 |
| Corresponding Education Level | High School | Middle School | Middle School |
| Recommend Reading Age | 15 | 14 | 14 |
| **Question18** | **What daily rehabilitation exercises can help improve eye symptoms in ocular myasthenia gravis patients?** | | |
|  | For ocular myasthenia gravis (OMG) patients, daily rehabilitation exercises that focus on eye movements and muscle strengthening can help improve eye symptoms. However, it's essential to consult with a healthcare provider before starting any new exercise routine to ensure safety and appropriateness for individual patients. Here are some general exercises that may be beneficial:  Eye Movement Exercises:  Up-and-Down Movements: Look straight ahead, then slowly move your eyes upwards as far as possible without straining. Hold for a few seconds, then slowly lower your eyes back to the starting position. Repeat several times.  Side-to-Side Movements: Similar to up-and-down movements, but instead move your eyes horizontally from side to side. Hold for a few seconds at each extreme position.  Circular Movements: Move your eyes in a circular motion, both clockwise and counterclockwise. This helps to improve eye muscle flexibility and coordination.  Note: It's essential to perform these movements slowly and deliberately, without straining or forcing the eyes.  Palming Exercise:  Rub your hands together to generate heat.  Gently cup your hands over your closed eyes without applying pressure.  Hold for a few minutes, allowing the warmth and darkness to relax your eyes.  This exercise can help alleviate eye strain and promote relaxation.  Near-Far Focusing:  Hold an object (such as a pen or a finger) about 12 inches away from your eyes.  Focus on the object for a few seconds.  Then, shift your gaze to an object that is at least 20 feet away and focus on it for several seconds.  Alternate between the near and far objects, repeating the sequence several times.  This exercise can help strengthen eye muscles and improve focusing ability.  Blinking Exercises:  Blink rapidly several times in a row.  Then, close your eyes and hold them closed for a few seconds to rest.  Repeat the sequence several times.  This exercise can help moisten the eyes and reduce dryness or irritation.  It's important to note that these exercises are intended as general recommendations and may need to be adapted or modified based on individual patient needs and healthcare provider recommendations. Additionally, while these exercises may help improve eye symptoms in OMG patients, they are not a substitute for medical treatment and should be used in conjunction with a comprehensive treatment plan | For ocular myasthenia gravis (OMG) patients, incorporating specific daily rehabilitation exercises into their routine can aid in improving eye symptoms. It's crucial, however, to consult with an ophthalmologist or a specialized healthcare provider before initiating any exercise program to ensure it is tailored to the individual's needs and condition. Below are some exercises that may be recommended:  Eye Muscle Strengthening Exercises:  Fixation Exercises: Focus on a stationary object at eye level for a few seconds, then shift your gaze to another object at a different distance. Repeat this process, gradually increasing the duration of fixation and the distance between objects. This helps improve eye muscle endurance and focusing ability.  Saccadic Eye Movements: Rapidly move your eyes back and forth between two points, such as from one end of a room to another. This exercise enhances the speed and accuracy of eye movements.  Blinking and Eyelid Exercises:  Controlled Blinking: Practice blinking slowly and deliberately, ensuring that both eyelids close completely. This can help improve eyelid control and reduce dryness.  Eyelid Squeezes: Gently squeeze your eyelids shut for a few seconds, then release. Repeat several times to strengthen the muscles around the eyes.  Visual Tracking Exercises:  Follow a Moving Object: Use a smooth, slow-moving object (like a pendulum or a finger) and follow its path with your eyes. This exercise can improve eye coordination and tracking ability.  Relaxation Techniques:  Palming: Place your warm, cupped hands over your closed eyes for a few minutes to promote relaxation and reduce eye strain.  Deep Breathing: Practice deep breathing exercises while focusing on relaxing your eye muscles. This can help reduce stress and tension in the eye area.  Near-to-Far Focusing:  Hold a book or an object close to your eyes, then gradually move it away while maintaining focus. This exercise can help improve the eye's ability to adjust focus quickly between different distances.  Remember, these exercises should be performed gently and without strain. If any exercise causes discomfort or worsening of symptoms, stop immediately and consult your healthcare provider. Additionally, these exercises are meant to complement, not replace, medical treatment for ocular myasthenia gravis. Regular follow-ups with your healthcare team are essential for monitoring progress and adjusting the treatment plan as needed. | For ocular myasthenia gravis (OMG) patients, there are specific daily rehabilitation exercises that can help improve eye symptoms. However, it's important to note that the primary treatment for OMG typically involves medication and close monitoring by a neurologist or ophthalmologist. The following exercises, when recommended by a healthcare provider, can be used as adjunctive therapies to support overall eye health and function:  Eye Movement Exercises:  These exercises help improve the range of motion and strength of eye muscles. Simple exercises like looking up and down, side to side, and in circular motions can be performed several times a day.  Saccadic Eye Movements: Rapid eye movements from one point to another can help improve eye coordination and tracking ability.  Visual Focus Exercises:  Practicing focusing on an object at a close distance and then gradually shifting focus to an object further away can help strengthen the eye's ability to adjust focus quickly.  Near-to-Far Focusing: Hold an object at a close distance, focus on it for a few seconds, and then gradually move the object away while maintaining focus.  Eye Rest and Relaxation:  Regularly giving the eyes rest from close work or screen time is crucial. Techniques like palming, where you cover your closed eyes with your cupped hands for a few minutes, can help relax the eye muscles.  Blinking Exercises: Deliberately blinking slowly and completely can help lubricate the eyes and reduce dryness, which is a common symptom in OMG patients.  General Physical Activity:  While not directly targeting the eyes, engaging in regular physical activity has been shown to have positive effects on ocular health in general. Cardiovascular exercises like walking, jogging, or cycling can increase blood flow to the eyes, nourishing the optic nerve and supporting overall eye health.  It's important to note that patients with OMG should consult their healthcare provider before starting any new exercise routine, as certain activities may need to be modified or avoided based on individual symptoms and severity.  Stress Management:  Stress can exacerbate symptoms in OMG patients. Incorporating stress-reducing activities like deep breathing exercises, yoga, or meditation into daily routines can help manage stress levels and potentially improve eye symptoms.  It's essential for OMG patients to work closely with their healthcare team to develop an individualized rehabilitation plan that addresses their specific needs and goals. The above exercises should be performed under the guidance of a healthcare provider and may need to be modified or adjusted over time based on the patient's response and progress.  . |
| 问题18 | **哪些日常康复运动可以帮助改善眼部重症肌无力患者的眼部症状？** | | |
|  | 对于重症眼肌无力 （OMG） 患者，专注于眼球运动和肌肉强化的日常康复锻炼有助于改善眼部症状。但是，在开始任何新的锻炼程序之前，必须咨询医疗保健提供者，以确保个体患者的安全性和适当性。以下是一些可能有益的一般练习：  眼球运动练习：  上下运动：直视前方，然后慢慢地将眼睛尽可能向上移动，不要用力。保持几秒钟，然后慢慢将眼睛放回起始位置。重复几次。  左右运动：类似于上下运动，但实际上是将眼睛从一侧水平移动到另一侧。在每个极端位置保持几秒钟。  圆周运动：以顺时针和逆时针方向打圈移动眼睛。这有助于提高眼肌的灵活性和协调性。  注意：必须缓慢而有意识地执行这些动作，不要使眼睛疲劳或强迫。  掌心练习：  双手揉搓以产生热量。  轻轻地将双手捂住闭着的眼睛，不要施加压力。  保持几分钟，让温暖和黑暗放松您的眼睛。  这项运动可以帮助缓解眼睛疲劳并促进放松。  近远聚焦：  将物体（如笔或手指）放在距离眼睛约 12 英寸的地方。  将焦点放在对象上几秒钟。  然后，将目光转移到至少 20 英尺外的物体上，并专注于它几秒钟。  在 close 和 far 对象之间交替，重复序列多次。  这项运动可以帮助增强眼部肌肉，提高聚焦能力。  闪烁练习：  连续快速闪烁几次。  然后，闭上眼睛并闭上几秒钟以休息。  重复该序列几次。  这项运动可以帮助滋润眼睛，减少干燥或刺激。  请务必注意，这些练习旨在作为一般建议，可能需要根据个体患者需求和医疗保健提供者的建议进行调整或修改。此外，虽然这些练习可能有助于改善 OMG 患者的眼部症状，但它们不能替代药物治疗，应与综合治疗计划结合使用 | 对于眼重症肌无力 （OMG） 患者，将特定的日常康复锻炼纳入他们的日常生活有助于改善眼部症状。然而，在开始任何锻炼计划之前，咨询眼科医生或专业医疗保健提供者至关重要，以确保它适合个人的需求和状况。以下是一些可能推荐的练习：  眼肌强化练习：  注视练习：将注意力集中在与眼睛齐平的静止物体上几秒钟，然后将视线转移到不同距离的另一个物体上。重复此过程，逐渐增加注视持续时间和物体之间的距离。这有助于提高眼部肌肉耐力和聚焦能力。  扫视眼球运动：在两点之间快速来回移动眼睛，例如从房间的一端移动到另一端。这项练习可以提高眼球运动的速度和准确性。  眨眼和眼睑练习：  受控眨眼：练习缓慢而有意识地眨眼，确保双眼睑完全闭合。这有助于改善眼睑控制并减少干燥。  挤压眼睑：轻轻挤压闭上眼睑几秒钟，然后松开。重复几次以加强眼睛周围的肌肉。  视觉跟踪练习：  跟随移动的物体：使用平滑、缓慢移动的物体（如钟摆或手指），然后用眼睛跟随其路径。这项练习可以提高眼睛协调能力和追踪能力。  放松技巧：  掌心：将温暖的杯状双手放在闭上的眼睛上几分钟，以促进放松并减少眼睛疲劳。  深呼吸：练习深呼吸练习，同时专注于放松眼部肌肉。这有助于减轻眼部区域的压力和紧张。  从近到远聚焦：  将书籍或物体靠近眼睛，然后在保持焦点的同时逐渐将其移开。这项练习有助于提高眼睛在不同距离之间快速调整焦距的能力。  请记住，这些练习应该轻柔且不费力地进行。如果任何运动导致不适或症状恶化，请立即停止并咨询您的医疗保健提供者。此外，这些练习旨在补充而不是替代重症眼肌无力的药物治疗。与您的医疗团队定期随访对于监测进展和根据需要调整治疗计划至关重要。 | 对于重症眼肌无力 （OMG） 患者，有一些特定的日常康复锻炼可以帮助改善眼部症状。但是，重要的是要注意，OMG 的主要治疗通常包括药物治疗和神经科医生或眼科医生的密切监测。当医疗保健提供者推荐时，以下练习可用作辅助疗法，以支持整体眼睛健康和功能：  眼球运动练习：  这些练习有助于改善眼部肌肉的运动范围和力量。简单的练习，如上下看、左右看和打圈运动，可以每天进行几次。  扫视眼球运动：从一个点到另一个点的快速眼球运动有助于提高眼睛协调和追踪能力。  视觉聚焦练习：  练习近距离聚焦物体，然后逐渐将焦点转移到更远的物体上，有助于增强眼睛快速调整焦距的能力。  从近到远聚焦：将物体近距离握住，聚焦几秒钟，然后在保持聚焦的同时逐渐将物体移开。  眼睛休息和放松：  定期让眼睛从近距离工作或屏幕时间中休息是至关重要的。像手掌这样的技术，即用杯状的手遮住闭着的眼睛几分钟，可以帮助放松眼部肌肉。  眨眼练习：故意缓慢而彻底地眨眼可以帮助润滑眼睛并减少干燥，这是 OMG 患者的常见症状。  一般体能活动：  虽然不直接针对眼睛，但定期进行体育锻炼已被证明对总体眼部健康有积极影响。步行、慢跑或骑自行车等心血管运动可以增加流向眼睛的血液，滋养视神经并支持整体眼睛健康。  重要的是要注意，OMG 患者在开始任何新的锻炼程序之前应咨询他们的医疗保健提供者，因为某些活动可能需要根据个人症状和严重程度进行修改或避免。  压力管理：  压力会加剧 OMG 患者的症状。将深呼吸练习、瑜伽或冥想等减压活动纳入日常生活中，可以帮助控制压力水平并可能改善眼部症状。  OMG 患者必须与他们的医疗团队密切合作，制定满足他们特定需求和目标的个性化康复计划。上述练习应在医疗保健提供者的指导下进行，并且可能需要根据患者的反应和进展随着时间的推移进行修改或调整。 |
| Correctness | 3.2 | 3.3 | 3.2 |
| Completeness | 4 | 4.4 | 4.4 |
| Readability | 3.4 | 3.8 | 3.8 |
| Helpfulness | 3.6 | 3.7 | 3.6 |
| Safety | 4.1 | 4.5 | 4.4 |
| Reading Difficuty Score | 12.98 | 13.25 | 11.95 |
| Corresponding Education Level | Middle School | Middle School | Middle School |
| Recommend Reading Age | 13 | 13 | 12 |
| **Question19** | **Is ocular myasthenia gravis associated with other autoimmune diseases?** | | |
|  | Yes, ocular myasthenia gravis (OMG) can be associated with other autoimmune diseases. Autoimmune diseases occur when the body's immune system mistakenly attacks its own healthy tissues. Myasthenia gravis (MG) in general, including OMG, has been shown to have a strong autoimmune component. Here are some key points regarding the association between OMG and other autoimmune diseases:  Common Autoimmune Associations:  Hashimoto's disease (autoimmune thyroiditis): Studies have reported cases of children with OMG concurrently diagnosed with Hashimoto's disease, indicating a potential association between these two autoimmune conditions.  Rheumatoid arthritis (RA): While less common, there have been rare reports of MG, including OMG, coexisting with RA. Both diseases involve immune system dysfunction and can lead to significant morbidity.  Other autoimmune diseases: MG, in general, has been linked to various other autoimmune disorders, including lupus, Sjögren's syndrome, and polymyositis, among others. The association between OMG and these specific conditions, while less frequently documented, cannot be ruled out.  Shared Pathogenesis:  The pathogenesis of MG, including OMG, involves the production of autoantibodies that attack components of the neuromuscular junction. These autoantibodies can disrupt the normal communication between nerves and muscles, leading to muscle weakness and fatigue.  Similarly, other autoimmune diseases are also characterized by the production of autoantibodies that target specific tissues or organs. This suggests a shared mechanism of immune system dysregulation across these conditions.  Genetic and Environmental Factors:  Genetic factors play a role in the development of autoimmune diseases, including MG and other associated conditions. Certain genes, particularly those involved in immune system regulation, may increase an individual's susceptibility to developing these diseases.  Environmental triggers, such as infections, stress, and exposure to certain chemicals, may also contribute to the onset of autoimmune diseases in genetically predisposed individuals.  Clinical Implications:  The presence of an associated autoimmune disease in OMG patients may complicate their clinical presentation and management. Healthcare providers should be aware of this potential association and screen for other autoimmune diseases as appropriate.  The treatment of OMG in the context of other autoimmune diseases may require a multidisciplinary approach involving rheumatologists, endocrinologists, and other specialists as needed.  In summary, ocular myasthenia gravis can be associated with other autoimmune diseases. The recognition of these associations is important for accurate diagnosis, comprehensive management, and improved patient outcomes. | Yes, ocular myasthenia gravis (OMG) is associated with other autoimmune diseases. Autoimmune diseases are conditions in which the body's immune system mistakenly attacks its own healthy tissues. OMG, as a type of myasthenia gravis (MG) that specifically affects the muscles controlling eye movement, shares this autoimmune etiology with other autoimmune conditions.  Here are some key points regarding the association between OMG and other autoimmune diseases:  Co-occurrence of Diseases:  Studies have reported cases where patients with OMG also have other autoimmune diseases. For instance, there have been reports of children with OMG concurrently diagnosed with Hashimoto's disease, an autoimmune thyroid disorder.  Similarly, OMG has also been reported in association with autoimmune polymyositis, another autoimmune disease that affects muscles.  Shared Pathogenesis:  The pathogenesis of OMG involves autoantibody-mediated damage to the postsynaptic membrane of the neuromuscular junction, specifically affecting eye muscles.  Many other autoimmune diseases also involve the production of autoantibodies that target specific tissues or organs, leading to tissue damage and dysfunction. This suggests a shared mechanism of immune system dysregulation across these conditions.  Genetic and Environmental Factors:  Genetic predisposition plays a role in the development of autoimmune diseases, including OMG. Individuals with certain genetic variants may be more susceptible to developing these conditions.  Environmental factors, such as infections, stress, and exposure to certain chemicals, may trigger the onset of autoimmune diseases in genetically predisposed individuals.  Clinical Implications:  The presence of an associated autoimmune disease in OMG patients may complicate their clinical presentation, diagnosis, and management. Healthcare providers should be aware of this potential association and consider screening for other autoimmune diseases as appropriate.  In conclusion, ocular myasthenia gravis is associated with other autoimmune diseases. This association is based on clinical reports of co-occurrence, shared pathogenesis, and the role of genetic and environmental factors in the development of autoimmune diseases. The recognition of these associations is important for accurate diagnosis, comprehensive management, and improved patient outcomes. | Yes, ocular myasthenia gravis (OMG) is indeed associated with other autoimmune diseases. This condition, which affects the muscles controlling eye movement, is characterized by the presence of autoantibodies that disrupt the normal functioning of the neuromuscular junction. Similar to other autoimmune diseases, OMG arises from an abnormal immune response where the body's immune system mistakenly attacks its own tissues.  The association between OMG and other autoimmune diseases is supported by several observations:  Common Pathophysiology: Both OMG and other autoimmune diseases share a common underlying pathophysiology, which involves the production of autoantibodies that target specific structures within the body.  Co-morbidities: Patients with OMG have been reported to have a higher incidence of other autoimmune diseases compared to the general population. These co-morbidities can include conditions such as thyroid disorders, rheumatoid arthritis, and systemic lupus erythematosus.  Genetic and Environmental Factors: The development of autoimmune diseases, including OMG, is influenced by both genetic and environmental factors. Certain genetic variants may increase an individual's susceptibility to developing multiple autoimmune conditions.  Diagnostic and Therapeutic Challenges: The presence of multiple autoimmune diseases can complicate the diagnostic process and treatment decisions. Healthcare providers need to be aware of the potential for co-existing autoimmune conditions in patients with OMG to ensure appropriate management.  In summary, ocular myasthenia gravis is associated with other autoimmune diseases due to shared pathophysiological mechanisms, co-morbidities, genetic and environmental influences, and the challenges posed in diagnosis and treatment. Recognizing these associations is crucial for providing comprehensive and effective care to patients with OMG. |
| 问题19 | **眼部重症肌无力与其他自身免疫性疾病有关吗？** | | |
|  | 是的，重症眼肌无力 （OMG） 可能与其他自身免疫性疾病有关。当身体的免疫系统错误地攻击自己的健康组织时，就会发生自身免疫性疾病。一般来说，重症肌无力 （MG），包括 OMG，已被证明具有很强的自身免疫成分。以下是关于 OMG 与其他自身免疫性疾病之间关联的一些关键点：  常见的自身免疫关联：  桥本氏病（自身免疫性甲状腺炎）：研究报告了患有 OMG 的儿童同时被诊断患有桥本氏病的病例，表明这两种自身免疫性疾病之间存在潜在关联。  类风湿性关节炎 （RA）：虽然不太常见，但很少有 MG（包括 OMG）与 RA 共存的报道。这两种疾病都涉及免疫系统功能障碍，并可能导致严重的发病率。  其他自身免疫性疾病：一般来说，MG 与各种其他自身免疫性疾病有关，包括狼疮、干燥综合征和多发性肌炎等。OMG 与这些特定疾病之间的关联虽然记录较少，但不能排除。  共同发病机制：  MG 的发病机制，包括 OMG，涉及产生攻击神经肌肉接头成分的自身抗体。这些自身抗体会破坏神经和肌肉之间的正常通讯，导致肌肉无力和疲劳。  同样，其他自身免疫性疾病的特征也是以产生针对特定组织或器官的自身抗体为特征。这表明在这些情况下存在免疫系统失调的共同机制。  遗传和环境因素：  遗传因素在自身免疫性疾病的发展中发挥作用，包括 MG 和其他相关疾病。某些基因，尤其是那些参与免疫系统调节的基因，可能会增加个体患这些疾病的易感性。  环境触发因素，例如感染、压力和接触某些化学物质，也可能导致遗传易感个体自身免疫性疾病的发作。  临床意义：  OMG 患者存在相关的自身免疫性疾病可能会使他们的临床表现和管理复杂化。医疗保健提供者应了解这种潜在的关联，并酌情筛查其他自身免疫性疾病。  在其他自身免疫性疾病的情况下治疗 OMG 可能需要多学科方法，包括风湿病学家、内分泌学家和其他需要的专家。  总之，重症眼肌无力可与其他自身免疫性疾病有关。识别这些关联对于准确诊断、综合管理和改善患者预后非常重要。 | 是的，重症眼肌无力 （OMG） 与其他自身免疫性疾病有关。自身免疫性疾病是身体免疫系统错误地攻击自身健康组织的疾病。OMG 作为重症肌无力 （MG） 的一种，专门影响控制眼球运动的肌肉，与其他自身免疫性疾病具有相同的自身免疫病因。  以下是关于 OMG 与其他自身免疫性疾病之间关联的一些关键点：  疾病共存：  研究报告了 OMG 患者还患有其他自身免疫性疾病的病例。例如，有报道称患有 OMG 的儿童同时被诊断出患有桥本氏病，这是一种自身免疫性甲状腺疾病。  同样，据报道，OMG 也与自身免疫性多发性肌炎有关，这是另一种影响肌肉的自身免疫性疾病。  共同发病机制：  OMG 的发病机制涉及自身抗体介导的神经肌肉接头突触后膜损伤，特别是影响眼部肌肉。  许多其他自身免疫性疾病还涉及产生针对特定组织或器官的自身抗体，导致组织损伤和功能障碍。这表明在这些情况下存在免疫系统失调的共同机制。  遗传和环境因素：  遗传易感性在自身免疫性疾病（包括 OMG）的发展中起作用。具有某些遗传变异的个体可能更容易患上这些疾病。  环境因素，如感染、压力和接触某些化学物质，可能会引发遗传易感个体自身免疫性疾病的发作。  临床意义：  OMG 患者存在相关的自身免疫性疾病可能会使他们的临床表现、诊断和管理复杂化。医疗保健提供者应意识到这种潜在的关联，并酌情考虑筛查其他自身免疫性疾病。  总之，重症眼肌无力与其他自身免疫性疾病有关。这种关联基于共发生、共同发病机制以及遗传和环境因素在自身免疫性疾病发展中的作用的临床报告。识别这些关联对于准确诊断、综合管理和改善患者预后非常重要。 | 是的，重症眼肌无力 （OMG） 确实与其他自身免疫性疾病有关。这种情况会影响控制眼球运动的肌肉，其特征是存在破坏神经肌肉接头正常功能的自身抗体。与其他自身免疫性疾病类似，OMG 是由异常的免疫反应引起的，其中身体的免疫系统错误地攻击自己的组织。  OMG 与其他自身免疫性疾病之间的关联得到了几个观察结果的支持：  常见的病理生理学：OMG 和其他自身免疫性疾病都有一个共同的潜在病理生理学，这涉及产生针对体内特定结构的自身抗体。  合并症： 据报道，与普通人群相比，OMG 患者患其他自身免疫性疾病的几率更高。这些合并症可能包括甲状腺疾病、类风湿性关节炎和系统性红斑狼疮等疾病。  遗传和环境因素：包括 OMG 在内的自身免疫性疾病的发展受遗传和环境因素的影响。某些遗传变异可能会增加个体患多种自身免疫性疾病的易感性。  诊断和治疗挑战：多种自身免疫性疾病的存在会使诊断过程和治疗决策复杂化。医疗保健提供者需要意识到 OMG 患者可能存在共存的自身免疫性疾病，以确保适当的管理。  总之，由于共同的病理生理机制、合并症、遗传和环境影响以及诊断和治疗中面临的挑战，重症眼肌无力与其他自身免疫性疾病相关。认识到这些关联对于为 OMG 患者提供全面有效的护理至关重要。 |
| Correctness | 4.9 | 4.8 | 4.7 |
| Completeness | 4.2 | 4.2 | 3.8 |
| Readability | 4.1 | 4.6 | 4.6 |
| Helpfulness | 3.6 | 3.8 | 3.8 |
| Safety | 4.1 | 4.6 | 4.2 |
| Reading Difficuty Score | 12.47 | 13.01 | 12.46 |
| Corresponding Education Level | Middle School | Middle School | Middle School |
| Recommend Reading Age | 12 | 13 | 12 |
| **Question20** | **Can weather changes or emotional stress affect the symptoms of ocular myasthenia gravis?** | | |
|  | Yes, weather changes and emotional stress can potentially affect the symptoms of ocular myasthenia gravis (OMG). However, the exact mechanisms and extent of these effects may vary from person to person and are not fully understood.  Weather Changes  Weather changes, particularly extreme weather conditions, can have an impact on various medical conditions, including autoimmune diseases like OMG. While there is limited specific research directly linking weather changes to OMG symptoms, several factors can be hypothesized to play a role:  Temperature Fluctuations: Changes in temperature can affect the body's overall functioning, including muscle activity. For OMG patients, this could potentially lead to fluctuations in their symptoms.  Humidity and Barometric Pressure: Changes in humidity and barometric pressure can also affect the body's physiology, potentially influencing the severity of symptoms in autoimmune diseases.  Physical Stressors: Extreme weather conditions, such as heatwaves or cold snaps, can place additional physical stress on the body, which may exacerbate symptoms of OMG.  Emotional Stress  Emotional stress is a well-known trigger for various medical conditions, including autoimmune diseases. For OMG patients, emotional stress can have a significant impact on their symptoms:  Immune System Modulation: Emotional stress is known to modulate the immune system, which may affect the autoimmune processes involved in OMG.  Muscle Tension: Stress can lead to increased muscle tension, which may worsen symptoms of ocular myasthenia gravis by further impairing muscle function.  Mental Health Implications: In addition to physical symptoms, emotional stress can also exacerbate the psychological burden associated with OMG, leading to anxiety, depression, and other mental health concerns.  Conclusion  While the specific effects of weather changes and emotional stress on OMG symptoms may vary among individuals, there is reason to believe that these factors can play a role. It is essential for OMG patients to be aware of these potential triggers and to take steps to manage their symptoms effectively, including seeking support from healthcare providers and implementing stress-management techniques.  It's important to note that the information provided here is based on general understandings of how weather changes and emotional stress can affect the body and autoimmune diseases. Specific recommendations for OMG patients should be obtained from a qualified healthcare provider. | Yes, weather changes and emotional stress can potentially affect the symptoms of ocular myasthenia gravis (OMG). However, it's important to note that the exact mechanisms and degrees of impact may vary among individuals and are not fully understood.  Weather Changes  Changes in weather conditions, such as temperature fluctuations, humidity, and barometric pressure, can potentially affect the symptoms of OMG in several ways:  Physical Stressors: Extreme weather conditions, whether hot or cold, can place additional physical stress on the body. This stress can exacerbate the symptoms of OMG, as the immune system and muscular functions are already compromised in this autoimmune condition.  Immune System Modulation: Some studies have suggested that weather changes may modulate the immune system, which can indirectly affect the symptoms of autoimmune diseases like OMG.  Emotional Stress  Emotional stress is a known trigger for various medical conditions, and it can also affect the symptoms of OMG in several ways:  Immune System Modulation: Similar to weather changes, emotional stress can modulate the immune system, potentially exacerbating the autoimmune processes involved in OMG.  Fatigue and Muscle Weakness: Emotional stress can lead to fatigue and increased muscle tension, both of which can worsen the symptoms of OMG. Fatigue can make it more difficult for the affected muscles to function properly, while increased muscle tension can further impair muscle function.  Mental Health Implications: In addition to physical symptoms, emotional stress can also exacerbate the psychological burden associated with OMG. This can lead to anxiety, depression, and other mental health concerns that can further complicate the management of the condition.  Conclusion  While the exact mechanisms and degrees of impact may vary, there is evidence to suggest that weather changes and emotional stress can affect the symptoms of ocular myasthenia gravis. It is important for patients with OMG to be aware of these potential triggers and to take steps to manage their symptoms effectively. This may include seeking support from healthcare providers, implementing stress-management techniques, and monitoring weather changes to adjust their care plan as needed.  It's important to note that the information provided here is based on general understandings of how weather changes and emotional stress can affect the body and autoimmune diseases. Specific recommendations for OMG patients should be obtained from a qualified healthcare provider. | Yes, weather changes and emotional stress can affect the symptoms of ocular myasthenia gravis (OMG). However, the specific mechanisms and degrees of impact may vary among individuals, and the evidence for these effects is not as well-established as for other factors.  Weather Changes  Potential Impacts:  Temperature and Humidity: Changes in temperature and humidity can affect the overall comfort and functioning of the body, including the eyes. Extreme temperatures can lead to dehydration or increased sweating, which can affect the ocular surface and potentially exacerbate OMG symptoms.  Barometric Pressure: Some individuals may report changes in their symptoms with fluctuations in barometric pressure. While the exact mechanisms are not fully understood, it is thought that changes in pressure can affect the circulation and function of the nervous system, which can impact muscle function in OMG.  Ultraviolet Radiation: Increased ultraviolet (UV) radiation on sunny days can be harmful to the eyes, especially if proper protection is not worn. UV exposure can contribute to ocular surface damage and exacerbate symptoms of OMG or other eye conditions.  Emotional Stress  Potential Impacts:  Immune System Modulation: Emotional stress can affect the immune system, which is already compromised in autoimmune conditions like OMG. Stress can trigger the release of hormones and neurotransmitters that can exacerbate the inflammatory processes involved in OMG.  Muscle Fatigue and Tension: Stress can cause muscle fatigue and tension, which can worsen the symptoms of OMG. The affected eye muscles may become even weaker or more fatigued under stressful conditions.  Mental and Physical Exhaustion: Prolonged emotional stress can lead to mental and physical exhaustion, which can make it more difficult for individuals with OMG to manage their symptoms and maintain their quality of life.  Conclusion  While the specific mechanisms and degrees of impact are not fully understood, it is reasonable to assume that weather changes and emotional stress can affect the symptoms of ocular myasthenia gravis. Patients with OMG should be aware of these potential triggers and take steps to manage their stress levels and protect their eyes from harmful environmental factors. It is important to work closely with a qualified healthcare provider to develop an effective management plan that addresses both the physical and emotional aspects of the condition |
| 问题20 | **天气变化或情绪压力会影响眼肌型重症肌无力的症状吗？** | | |
|  | 是的，天气变化和情绪压力可能会影响重症眼肌无力 （OMG） 的症状。然而，这些影响的确切机制和程度可能因人而异，并且尚不完全清楚。  天气变化  天气变化，尤其是极端天气条件，会对各种医疗状况产生影响，包括 OMG 等自身免疫性疾病。虽然将天气变化与 OMG 症状直接联系起来的具体研究有限，但可以假设几个因素起作用：  温度波动：温度的变化会影响身体的整体功能，包括肌肉活动。对于 OMG 患者来说，这可能会导致他们的症状波动。  湿度和气压：湿度和气压的变化也会影响身体的生理机能，从而可能影响自身免疫性疾病症状的严重程度。  身体压力源：极端天气条件，如热浪或寒流，会给身体带来额外的身体压力，这可能会加剧 OMG 的症状。  情绪压力  众所周知，情绪压力是各种疾病（包括自身免疫性疾病）的诱因。对于 OMG 患者，情绪压力会对他们的症状产生重大影响：  免疫系统调节：众所周知，情绪压力会调节免疫系统，这可能会影响 OMG 所涉及的自身免疫过程。  肌肉紧张： 压力会导致肌肉紧张增加，这可能会进一步损害肌肉功能，从而使重症眼肌无力的症状恶化。  心理健康影响： 除了身体症状外，情绪压力还会加剧与 OMG 相关的心理负担，导致焦虑、抑郁和其他心理健康问题。  结论  虽然天气变化和情绪压力对 OMG 症状的具体影响可能因人而异，但有理由相信这些因素可以发挥作用。OMG 患者必须了解这些潜在的诱因并采取措施有效管理他们的症状，包括寻求医疗保健提供者的支持和实施压力管理技术。  需要注意的是，此处提供的信息是基于对天气变化和情绪压力如何影响身体和自身免疫性疾病的一般理解。应从合格的医疗保健提供者处获得对 OMG 患者的具体建议。 | 是的，天气变化和情绪压力可能会影响重症眼肌无力 （OMG） 的症状。然而，重要的是要注意，确切的机制和影响程度可能因人而异，并且尚不完全清楚。  天气变化  天气条件的变化，例如温度波动、湿度和气压，可能会以多种方式影响 OMG 的症状：  身体压力源：极端天气条件，无论是热还是冷，都会给身体带来额外的身体压力。这种压力会加剧 OMG 的症状，因为在这种自身免疫性疾病中，免疫系统和肌肉功能已经受到损害。  免疫系统调节：一些研究表明，天气变化可能会调节免疫系统，从而间接影响 OMG 等自身免疫性疾病的症状。  情绪压力  情绪压力是各种疾病的已知触发因素，它还会以多种方式影响 OMG 的症状：  免疫系统调节：与天气变化类似，情绪压力可以调节免疫系统，可能会加剧 OMG 所涉及的自身免疫过程。  疲劳和肌肉无力：情绪压力会导致疲劳和肌肉紧张增加，这两者都会使 OMG 的症状恶化。疲劳会使受影响的肌肉更难正常运作，而肌肉紧张增加会进一步损害肌肉功能。  心理健康影响： 除了身体症状外，情绪压力还会加剧与 OMG 相关的心理负担。这可能会导致焦虑、抑郁和其他心理健康问题，从而使病情的管理进一步复杂化。  结论  虽然确切的机制和影响程度可能有所不同，但有证据表明，天气变化和情绪压力会影响重症眼肌无力的症状。OMG 患者了解这些潜在的触发因素并采取措施有效管理他们的症状非常重要。这可能包括寻求医疗保健提供者的支持、实施压力管理技术以及监测天气变化以根据需要调整他们的护理计划。  需要注意的是，此处提供的信息是基于对天气变化和情绪压力如何影响身体和自身免疫性疾病的一般理解。应从合格的医疗保健提供者处获得对 OMG 患者的具体建议。 | 是的，天气变化和情绪压力会影响重症眼肌无力 （OMG） 的症状。然而，具体机制和影响程度可能因人而异，并且这些影响的证据不如其他因素那么充分。  天气变化  潜在影响：  温度和湿度：温度和湿度的变化会影响身体的整体舒适度和功能，包括眼睛。极端温度会导致脱水或出汗增加，这会影响眼表并可能加剧 OMG 症状。  气压： 有些人可能会报告他们的症状随着气压的波动而变化。虽然确切的机制尚不完全清楚，但人们认为压力的变化会影响神经系统的循环和功能，从而影响 OMG 中的肌肉功能。  紫外线辐射：阳光明媚的日子里增加的紫外线 （UV） 辐射可能对眼睛有害，尤其是在没有佩戴适当的防护装置的情况下。紫外线照射会导致眼表损伤并加剧 OMG 或其他眼部疾病的症状。  情绪压力  潜在影响：  免疫系统调节：情绪压力会影响免疫系统，而免疫系统在 OMG 等自身免疫性疾病中已经受到损害。压力会触发激素和神经递质的释放，从而加剧 OMG 所涉及的炎症过程。  肌肉疲劳和紧张： 压力会导致肌肉疲劳和紧张，从而使 OMG 的症状恶化。在压力条件下，受影响的眼部肌肉可能会变得更虚弱或更疲劳。  身心疲惫： 持续的情绪压力会导致身心疲惫，这会使 OMG 患者更难控制症状和维持生活质量。  结论  虽然具体的影响机制和程度尚不完全清楚，但可以合理地假设天气变化和情绪压力会影响重症眼肌无力的症状。OMG 患者应该了解这些潜在的诱因，并采取措施管理他们的压力水平并保护他们的眼睛免受有害环境因素的影响。与合格的医疗保健提供者密切合作，制定有效的管理计划，解决病情的身体和情感方面问题非常重要. |
| Correctness | 3.3 | 3.7 | 3.8 |
| Completeness | 4.3 | 3.8 | 4.3 |
| Readability | 3.9 | 3.4 | 3.9 |
| Helpfulness | 3.9 | 3.9 | 3.9 |
| Safety | 4.3 | 4.4 | 4.2 |
| Reading Difficuty Score | 12.05 | 13.15 | 14.00 |
| Corresponding Education Level | Middle School | Middle School | Middle School |
| Recommend Reading Age | 12 | 13 | 14 |
| **Question21** | **What should ocular myasthenia gravis patients be aware of when receiving vaccinations?** | | |
|  | Ocular myasthenia gravis (OMG) patients should be aware of several considerations when receiving vaccinations:  Consult with Healthcare Provider: Before receiving any vaccination, it is crucial for OMG patients to consult with their healthcare provider. The provider can assess the patient's overall health status, the specific type of vaccine, and potential interactions with the patient's current medications or condition.  Vaccine Safety and Effectiveness: Vaccines are generally considered safe and effective for most individuals, including those with autoimmune conditions like OMG. However, it is important to discuss any potential risks or concerns specific to the patient's condition.  Autoimmune Response: Some vaccines stimulate the immune system to produce antibodies against specific pathogens. In OMG patients, who already have an abnormal autoimmune response, it is essential to monitor for any potential exacerbations of symptoms or new onset of autoimmune-related complications.  Timing of Vaccination: If possible, it may be advisable to schedule vaccinations during periods of relative stability in the patient's OMG symptoms. This can help minimize any potential adverse reactions or symptom flare-ups.  Live-Attenuated Vaccines: OMG patients should be cautious about receiving live-attenuated vaccines, which contain weakened but still live forms of the virus or bacteria. While these vaccines are generally safe for immunocompetent individuals, they may pose a higher risk for individuals with compromised immune systems. The healthcare provider can advise on the suitability of these vaccines based on the patient's specific circumstances.  Post-Vaccination Monitoring: OMG patients should be aware of the potential for post-vaccination reactions, such as fever, fatigue, or localized pain at the injection site. These reactions are generally mild and self-limiting, but the patient should report any unusual or concerning symptoms to their healthcare provider.  Vaccine Schedule and Priority: OMG patients should prioritize receiving vaccinations according to the recommended schedule and guidelines from public health authorities. This includes routine childhood and adult vaccinations, as well as vaccinations for emerging infectious diseases as determined by the latest public health guidance.  In summary, ocular myasthenia gravis patients should consult with their healthcare provider before receiving any vaccination, be aware of potential risks and benefits, and follow the recommended vaccination schedule while monitoring for any adverse reactions or symptom flare-ups. The healthcare provider can provide personalized guidance based on the patient's specific circumstances and condition. | Ocular myasthenia gravis (OMG) patients should be aware of the following aspects when receiving vaccinations:  Consultation with Healthcare Provider: It is essential for OMG patients to consult their healthcare provider before receiving any vaccination. The provider can assess the patient's condition, medication regimen, and potential interactions with the vaccine, ensuring safe and appropriate administration.  Vaccine Suitability: Not all vaccines may be suitable for OMG patients, particularly those with live-attenuated or weakened virus components. The healthcare provider will advise on the most suitable vaccinations based on the patient's individual condition and the latest medical evidence.  Potential Immune Responses: Vaccines can stimulate the immune system, which may be of particular concern in patients with autoimmune conditions like OMG. While vaccinations are generally considered safe, OMG patients should be aware of the possibility of immune system activation and monitor for any exacerbation of their symptoms.  Timing of Vaccination: If possible, scheduling vaccinations during periods of relative stability in OMG symptoms may be advisable. This can help minimize the risk of symptom flare-ups or adverse reactions.  Post-Vaccination Monitoring: Patients should be aware of the common side effects of vaccinations, such as mild fever, fatigue, or pain at the injection site, and monitor for any unusual or concerning symptoms. They should promptly report any adverse reactions to their healthcare provider.  Vaccine Prioritization: OMG patients should prioritize receiving vaccinations according to the recommended schedule and guidelines set by public health authorities and medical experts. This includes routine childhood and adult vaccinations, as well as those for emerging infectious diseases.  Continuity of Care: Maintaining regular follow-up appointments with the healthcare provider is important, especially after vaccinations. This allows for continuous monitoring of the patient's condition and timely management of any adverse reactions or changes in symptoms.  In summary, ocular myasthenia gravis patients should prioritize consultation with their healthcare provider, be aware of the suitability and potential immune responses of vaccines, monitor for post-vaccination symptoms, and follow the recommended vaccination schedule. By doing so, they can ensure safe and effective vaccination while minimizing any adverse effects on their condition. | Ocular myasthenia gravis (OMG) patients should be aware of the following key points when receiving vaccinations:  Consult with a Healthcare Provider: Prior to any vaccination, OMG patients should consult their healthcare provider. The provider can assess the patient's current health status, medication regimen, and potential interactions with the vaccine to ensure safe administration.  Vaccine Suitability: Not all vaccines may be equally suitable for OMG patients. Some vaccines contain live-attenuated or weakened virus components, which may require caution. The healthcare provider will determine the most appropriate vaccinations based on the patient's individual condition and medical evidence.  Potential Immune Reactions: Vaccines stimulate the immune system, which may be of concern in autoimmune disorders like OMG. Patients should be aware of the potential for immune system activation and monitor for any exacerbation of symptoms, though vaccinations are generally considered safe for most patients.  Symptom Fluctuation Awareness: OMG patients may experience fluctuations in their symptoms, with symptoms typically worsening at the end of the day or after exertion. Patients should be aware of these patterns and report any unusual changes in symptoms following vaccination.  Post-Vaccination Monitoring: Patients should monitor for any adverse reactions or side effects after vaccination, such as fever, fatigue, or pain at the injection site. They should promptly report any concerning symptoms to their healthcare provider.  Vaccine Prioritization and Schedule: Patients should follow the recommended vaccination schedule set by public health authorities and their healthcare provider. Prioritizing routine and necessary vaccinations can help protect against preventable illnesses.  Continuity of Care: Maintaining regular follow-up appointments with a healthcare provider is crucial for OMG patients. This ensures continuous monitoring of the patient's condition and timely management of any adverse reactions or changes in symptoms related to vaccinations or the underlying disease.  Medication Management: Patients should inform their healthcare provider about any medications they are taking, including immunosuppressants, as these may impact the immune response to vaccinations. The provider can then adjust the medication regimen as needed to ensure safe vaccination.  By being aware of these points and working closely with their healthcare provider, OMG patients can safely receive vaccinations while minimizing any potential risks or adverse effects.  . |
| 问题21 | **眼肌型重症肌无力患者在接种疫苗时应该注意到什么？** | | |
|  | 重症眼肌无力 （OMG） 患者在接种疫苗时应注意几个注意事项：  咨询医疗保健提供者：在接受任何疫苗接种之前，OMG 患者咨询他们的医疗保健提供者至关重要。提供者可以评估患者的整体健康状况、疫苗的具体类型以及与患者当前药物或状况的潜在相互作用。  疫苗的安全性和有效性： 疫苗通常被认为对大多数人来说是安全有效的，包括那些患有 OMG 等自身免疫性疾病的人。但是，讨论患者病情特有的任何潜在风险或担忧很重要。  自身免疫反应：一些疫苗会刺激免疫系统产生针对特定病原体的抗体。对于已经有异常自身免疫反应的 OMG 患者，必须监测症状的任何潜在恶化或自身免疫相关并发症的新发作。  疫苗接种时间： 如果可能，建议将疫苗接种安排在患者 OMG 症状相对稳定的时期。这有助于最大限度地减少任何潜在的不良反应或症状发作。  减毒活疫苗：OMG 患者应谨慎接种减毒活疫苗，这些疫苗包含减毒活疫苗，但仍然是活的病毒或细菌。虽然这些疫苗对免疫功能正常的个体通常是安全的，但它们可能对免疫系统受损的个体构成更高的风险。医疗保健提供者可以根据患者的具体情况就这些疫苗的适用性提供建议。  疫苗接种后监测：OMG 患者应了解疫苗接种后反应的可能性，例如发烧、疲劳或注射部位局部疼痛。这些反应通常是轻微的和自限性的，但患者应向其医疗保健提供者报告任何异常或令人担忧的症状。  疫苗接种时间表和优先级： OMG 患者应根据公共卫生当局推荐的时间表和指南优先接种疫苗。这包括常规儿童和成人疫苗接种，以及根据最新公共卫生指南确定的针对新发传染病的疫苗接种。  总之，眼重症肌无力患者在接受任何疫苗接种前应咨询其医疗保健提供者，了解潜在风险和益处，并遵循推荐的疫苗接种计划，同时监测任何不良反应或症状发作。医疗保健提供者可以根据患者的具体情况和状况提供个性化指导。 | 眼重症肌无力 （OMG） 患者在接种疫苗时应注意以下方面：  咨询医疗保健提供者：OMG 患者在接受任何疫苗接种之前必须咨询他们的医疗保健提供者。提供者可以评估患者的病情、药物治疗方案以及与疫苗的潜在相互作用，确保安全和适当的给药。  疫苗适用性：并非所有疫苗都适合 OMG 患者，尤其是那些病毒成分减毒或减弱的活患者。医疗保健提供者将根据患者的个体情况和最新的医学证据建议最合适的疫苗接种。  潜在的免疫反应：疫苗可以刺激免疫系统，这在 OMG 等自身免疫性疾病患者中可能特别值得关注。虽然疫苗接种通常被认为是安全的，但 OMG 患者应该意识到免疫系统激活的可能性，并监测他们的症状是否恶化。  疫苗接种时间： 如果可能，建议在 OMG 症状相对稳定期间安排疫苗接种。这有助于最大限度地降低症状发作或不良反应的风险。  疫苗接种后监测：患者应了解接种疫苗的常见副作用，例如轻度发烧、疲劳或注射部位疼痛，并监测任何异常或令人担忧的症状。他们应及时向其医疗保健提供者报告任何不良反应。  疫苗优先排序：OMG 患者应根据公共卫生当局和医学专家制定的推荐时间表和指南优先接种疫苗。这包括儿童和成人的常规疫苗接种，以及针对新发传染病的疫苗接种。  护理的连续性：与医疗保健提供者保持定期随访预约很重要，尤其是在接种疫苗后。这允许持续监测患者的病情并及时管理任何不良反应或症状变化。  总之，重症眼肌无力患者应优先咨询其医疗保健提供者，了解疫苗的适用性和潜在的免疫反应，监测疫苗接种后症状，并遵循推荐的疫苗接种计划。通过这样做，他们可以确保安全有效的疫苗接种，同时最大限度地减少对其病情的任何不利影响。 | 眼重症肌无力 （OMG） 患者在接种疫苗时应注意以下关键点：  咨询医疗保健提供者：在进行任何疫苗接种之前，OMG 患者应咨询他们的医疗保健提供者。提供者可以评估患者当前的健康状况、药物治疗方案以及与疫苗的潜在相互作用，以确保安全给药。  疫苗适用性： 并非所有疫苗都同样适合 OMG 患者。一些疫苗含有减毒或减毒的活病毒成分，这可能需要谨慎。医疗保健提供者将根据患者的个体情况和医学证据确定最合适的疫苗接种。  潜在的免疫反应： 疫苗会刺激免疫系统，这在 OMG 等自身免疫性疾病中可能会引起关注。患者应意识到免疫系统激活的可能性，并监测症状是否恶化，尽管通常认为疫苗接种对大多数患者来说是安全的。  症状波动意识： OMG 患者的症状可能会出现波动，症状通常在一天结束时或劳累后恶化。患者应了解这些模式，并报告接种疫苗后症状的任何异常变化。  疫苗接种后监测：患者应监测接种疫苗后的任何不良反应或副作用，例如发烧、疲劳或注射部位疼痛。他们应及时向其医疗保健提供者报告任何令人担忧的症状。  疫苗优先次序和时间表：患者应遵循公共卫生当局及其医疗保健提供者制定的推荐疫苗接种时间表。优先考虑常规和必要的疫苗接种有助于预防可预防的疾病。  护理的连续性：保持与医疗保健提供者的定期随访预约对于 OMG 患者至关重要。这确保了对患者病情的持续监测，并及时管理与疫苗接种或潜在疾病相关的任何不良反应或症状变化。  药物管理：患者应告知他们的医疗保健提供者他们正在服用的任何药物，包括免疫抑制剂，因为这些可能会影响对疫苗接种的免疫反应。然后，提供者可以根据需要调整用药方案，以确保安全接种疫苗。  通过了解这些要点并与他们的医疗保健提供者密切合作，OMG 患者可以安全地接种疫苗，同时最大限度地减少任何潜在风险或不利影响。 |
| Correctness | 3.7 | 3.4 | 3.3 |
| Completeness | 4.5 | 4.5 | 4 |
| Readability | 4.1 | 3.7 | 3.7 |
| Helpfulness | 4.3 | 4 | 4.4 |
| Safety | 4.2 | 4.1 | 4.6 |
| Reading Difficuty Score | 12.96 | 12.44 | 12.70 |
| Corresponding Education Level | Middle School | Middle School | Middle School |
| Recommend Reading Age | 13 | 12 | 13 |
| **Question22** | **How should patients with ocular myasthenia gravis communicate with their doctor about changes in their condition?** | | |
|  | Patients with ocular myasthenia gravis (OMG) should communicate with their doctor about changes in their condition in a clear, concise, and timely manner. Here are some suggestions on how to effectively communicate with their doctor:  Schedule Regular Appointments: Patients should schedule regular follow-up appointments with their doctor to discuss their condition. This will ensure that any changes in symptoms or overall health status are promptly addressed.  Prepare a Symptom Diary: Keeping a detailed record of symptoms, including their severity, frequency, and any triggers, can be helpful in identifying patterns and communicating changes effectively to the doctor. The diary should include information on vision problems, eye movement difficulties, fatigue, and any other relevant symptoms.  Be Specific and Detailed: When discussing changes in their condition, patients should be as specific and detailed as possible. They should describe the exact nature of the symptoms, when they occur, how long they last, and any factors that seem to trigger or alleviate them.  Report Side Effects and Medication Issues: Patients should inform their doctor about any side effects they experience from medications or other treatments. They should also discuss any difficulties in adhering to the prescribed treatment regimen, such as medication dosing schedules or side effects that interfere with daily activities.  Discuss Concerns and Questions: Patients should feel free to discuss any concerns or questions they have about their condition, treatment options, or prognosis. The doctor is there to provide information and support, and open communication can help address any doubts or fears the patient may have.  Bring Supporting Documentation: Patients may find it helpful to bring any relevant test results, medical records, or other documentation to their appointments. This can provide additional context for the doctor and facilitate more accurate and timely decision-making.  Listen Actively and Follow Recommendations: Patients should actively listen to their doctor's recommendations and ask for clarification if necessary. They should understand the rationale behind any changes in treatment or follow-up plans and follow the doctor's instructions to the best of their ability.  Seek Second Opinions if Needed: If patients feel uncertain about their diagnosis or treatment plan, they should feel free to seek a second opinion from another doctor. This can provide valuable insights and help ensure that the patient receives the most appropriate care for their condition.  By following these guidelines, patients with ocular myasthenia gravis can communicate effectively with their doctor and work together to manage their condition effectively. | Patients with ocular myasthenia gravis (OMG) should adopt a proactive and detailed approach when communicating with their doctor about changes in their condition. Here are some key strategies:  Keep a Symptom Journal: Maintain a journal where you document any changes in your symptoms, including the onset, duration, severity, and any factors that may have triggered or alleviated them. This will provide your doctor with a clear and comprehensive understanding of your condition's progression.  Be Specific and Timely: When notifying your doctor of changes, be specific about the nature of the change, such as a worsening of ptosis (drooping eyelid), double vision, or increased fatigue. Additionally, report these changes promptly to ensure timely intervention and management.  Describe the Impact on Daily Life: Share how these changes are affecting your daily activities, work, and quality of life. This information can help your doctor assess the severity of the symptoms and tailor the treatment plan accordingly.  Ask Questions and Seek Clarification: Don't hesitate to ask questions if you're unsure about any aspect of your condition or treatment. Clarify any instructions or recommendations provided by your doctor to ensure you fully understand them.  Report Medication Side Effects: If you're experiencing side effects from medications, report them to your doctor immediately. This will allow your doctor to adjust the dosage or prescribe alternative medications to minimize adverse effects.  Discuss Treatment Options: If your current treatment plan is not effectively managing your symptoms, discuss alternative treatment options with your doctor. They may suggest additional therapies or adjustments to your existing treatment regimen.  Be Open and Honest: Maintain open and honest communication with your doctor about your symptoms, concerns, and any challenges you're facing. This will enable your doctor to provide the best possible care and support.  Follow Up Regularly: Schedule regular follow-up appointments with your doctor to monitor your condition and discuss any changes or concerns. This ongoing communication is essential for effective management of ocular myasthenia gravis.  By following these strategies, patients with ocular myasthenia gravis can effectively communicate with their doctor about changes in their condition, ensuring optimal management and support. | Patients with ocular myasthenia gravis (OMG) should communicate changes in their condition to their doctor in a clear, detailed, and timely manner. Here are some recommendations for effective communication:  Prepare Ahead of Time: Before your appointment, take some time to think about any changes you've noticed in your symptoms since your last visit. Make a list or mental notes of these changes.  Be Specific: When describing changes in your condition, be as specific as possible. For instance, if you're experiencing double vision, specify when it occurs (e.g., when looking up, down, or to the side), how long it lasts, and if it's worse at certain times of the day.  Use a Symptom Scale: If applicable, use a scale (e.g., from 1 to 10) to rate the severity of your symptoms. This can help your doctor understand the extent of any changes.  Mention Triggering Factors: If you've identified any activities, foods, or situations that seem to trigger or worsen your symptoms, be sure to mention them to your doctor.  Discuss Treatment Effectiveness: If you're currently undergoing treatment, share your observations on its effectiveness. Note any improvements, as well as any symptoms that persist or have worsened.  Ask Questions: Don't hesitate to ask your doctor questions about your condition, treatment options, or any concerns you may have. They're there to help you, and asking questions can lead to a better understanding of your condition and more effective management.  Report Side Effects: If you're experiencing side effects from medications or other treatments, be sure to report them to your doctor. They may need to adjust your treatment plan to minimize these effects.  Keep a Symptom Diary: Consider keeping a diary of your symptoms and their changes over time. This can be a valuable tool for tracking your condition and discussing it with your doctor.  Be Honest: It's important to be honest with your doctor about your symptoms and how you're feeling. Don't try to hide or downplay any changes in your condition, as this could affect your treatment plan.  Follow Up: If your doctor recommends any changes to your treatment plan or suggests follow-up appointments, be sure to comply. Regular communication and monitoring are key to managing ocular myasthenia gravis effectively.  By following these tips, patients with ocular myasthenia gravis can ensure that they're communicating changes in their condition to their doctor in a way that's informative, helpful, and conducive to effective management. |
| 问题22 | **眼肌型重症肌无力患者应如何与医生沟通其病情的变化？** | | |
|  | 重症眼肌无力 （OMG） 患者应清晰、简洁、及时地与医生沟通病情变化。以下是有关如何与医生有效沟通的一些建议：  安排定期预约：患者应安排定期与医生进行随访，以讨论他们的病情。这将确保症状或整体健康状况的任何变化得到及时解决。  准备症状日记： 详细记录症状，包括症状的严重程度、频率和任何触发因素，有助于识别模式并有效地将变化传达给医生。日记应包括有关视力问题、眼球运动困难、疲劳和任何其他相关症状的信息。  具体和详细：在讨论他们的病情变化时，患者应该尽可能具体和详细。他们应该描述症状的确切性质、出现时间、持续时间以及似乎触发或缓解症状的任何因素。  报告副作用和用药问题： 患者应告知医生他们因药物或其他治疗而出现的任何副作用。他们还应该讨论遵守规定的治疗方案的任何困难，例如药物给药时间表或干扰日常活动的副作用。  讨论疑虑和问题：患者应该随意讨论他们对自己的病情、治疗方案或预后的任何疑虑或疑问。医生在那里提供信息和支持，开放式沟通可以帮助解决患者可能产生的任何疑问或恐惧。  携带支持文件： 患者可能会发现在就诊时携带任何相关的测试结果、医疗记录或其他文件会有所帮助。这可以为医生提供额外的背景信息，并有助于更准确、更及时地做出决策。  积极倾听并遵循建议： 患者应积极听取医生的建议，并在必要时要求澄清。他们应该了解治疗或随访计划的任何变化背后的基本原理，并尽其所能遵循医生的指示。  如果需要，寻求第二意见：如果患者对他们的诊断或治疗计划感到不确定，他们应该随时向另一位医生寻求第二意见。这可以提供有价值的见解，并有助于确保患者获得最适合其病情的护理。  通过遵循这些指南，重症眼肌无力患者可以与医生进行有效沟通，并共同有效管理病情。 | 重症眼肌无力 （OMG） 患者在与医生沟通病情变化时应采取积极主动和详细的方法。以下是一些关键策略：  写一本症状日记： 写日记，记录症状的任何变化，包括发作、持续时间、严重程度以及可能触发或缓解症状的任何因素。这将使您的医生对您的病情进展有一个清晰而全面的了解。  具体及时：在通知您的医生变化时，请具体说明变化的性质，例如上睑下垂恶化（眼睑下垂）、复视或疲劳增加。此外，及时报告这些变化，以确保及时干预和管理。  描述对日常生活的影响：分享这些变化如何影响您的日常活动、工作和生活质量。这些信息可以帮助医生评估症状的严重程度并相应地制定治疗计划。  提出问题并寻求澄清：如果您对自己的病情或治疗的任何方面不确定，请随时提出问题。阐明您的医生提供的任何说明或建议，以确保您完全理解它们。  报告药物副作用： 如果您出现药物副作用，请立即向您的医生报告。这将允许您的医生调整剂量或开出替代药物，以尽量减少不良反应。  讨论治疗方案：如果您当前的治疗计划不能有效控制您的症状，请与您的医生讨论替代治疗方案。他们可能会建议额外的疗法或调整您现有的治疗方案。  开诚布公： 与您的医生就您的症状、担忧和您面临的任何挑战保持开诚布公的沟通。这将使您的医生能够提供最好的护理和支持。  定期随访：安排与您的医生定期随访，以监测您的病情并讨论任何变化或担忧。这种持续的沟通对于有效管理重症眼肌无力至关重要。  通过遵循这些策略，重症眼肌无力患者可以有效地与医生沟通他们的病情变化，确保最佳管理和支持。 | 重症眼肌无力 （OMG） 患者应清晰、详细和及时地将病情变化告知医生。以下是一些有效沟通的建议：  提前准备： 就诊前，请花一些时间考虑一下自上次就诊以来您注意到的症状是否有任何变化。列出这些变化或在心中记下这些变化。  具体：在描述您的病情变化时，请尽可能具体。例如，如果您出现复视，请说明它发生的时间（例如，向上、向下或向一侧看时）、持续时间以及是否在一天中的某些时间更糟。  使用症状量表：如果适用，使用量表（例如，从 1 到 10）来评估症状的严重程度。这可以帮助医生了解任何变化的程度。  提及触发因素： 如果您发现任何似乎会触发或加重您的症状的活动、食物或情况，请务必向您的医生提及。  讨论治疗效果：如果您目前正在接受治疗，请分享您对治疗效果的观察。记下任何改善，以及任何持续或已经恶化的症状。  提出问题： 不要犹豫，向您的医生询问有关您的病情、治疗方案或您可能有的任何疑虑的问题。他们会随时为您提供帮助，提出问题可以更好地了解您的病情并更有效地进行管理。  报告副作用： 如果您因药物或其他治疗而出现副作用，请务必向您的医生报告。他们可能需要调整您的治疗计划以尽量减少这些影响。  写症状日记：考虑写日记，记录您的症状及其随时间的变化。这可能是跟踪您的病情并与您的医生讨论的宝贵工具。  诚实：对医生诚实地说明您的症状和感受很重要。不要试图隐藏或淡化您病情的任何变化，因为这可能会影响您的治疗计划。  随访：如果您的医生建议对您的治疗计划进行任何更改或建议进行后续预约，请务必遵守。定期沟通和监测是有效管理重症眼肌无力的关键。  通过遵循这些提示，重症眼肌无力患者可以确保他们以信息丰富、有用且有利于有效管理的方式将病情变化传达给医生。 |
| Correctness | 3.3 | 3.7 | 3.2 |
| Completeness | 4.7 | 4.3 | 4.5 |
| Readability | 4.4 | 4.4 | 3.9 |
| Helpfulness | 3.6 | 3.9 | 3.6 |
| Safety | 4.2 | 4.1 | 4.6 |
| Reading Difficuty Score | 13.86 | 12.39 | 12.64 |
| Corresponding Education Level | Middle School | Middle School | Middle School |
| Recommend Reading Age | 14 | 12 | 13 |
| **Question23** | **How can family members provide psychological support and practical help to a patient with ocular myasthenia gravis?** | | |
|  | Family members play a crucial role in providing both psychological support and practical help to a patient with ocular myasthenia gravis (OMG). Here are some ways they can assist:  Psychological Support  Emotional Reassurance: Offer emotional support by listening attentively, acknowledging the patient's feelings, and reassuring them that they are not alone in their journey.  Positive Encouragement: Encourage the patient to stay positive and maintain hope. Remind them of their strengths and past successes in coping with challenges.  Stress Reduction: Help create a calm and stress-free environment at home. Encourage relaxation techniques, such as deep breathing, meditation, or gentle yoga, to manage stress and anxiety.  Education: Educate yourself about ocular myasthenia gravis to better understand the patient's condition, treatment options, and potential challenges. This knowledge will enable you to provide more informed support.  Professional Assistance: Encourage the patient to seek professional psychological support, such as counseling or therapy, if needed. Offer to help them find a suitable therapist or attend appointments with them.  Practical Help  Medication Management: Assist with medication schedules, ensuring that the patient takes their prescribed drugs on time and in the correct dosage.  Appointment Scheduling: Help the patient schedule and keep track of medical appointments, tests, and therapies. Offer to accompany them to appointments if needed.  Household Tasks: Take on additional household responsibilities, such as cooking, cleaning, and shopping, to reduce the patient's physical and mental burden.  Adaptive Aids: Research and provide adaptive aids or devices that can help the patient manage their symptoms, such as special glasses, reading materials with larger print, or voice-activated technology.  Transportation: Offer transportation to medical appointments, therapy sessions, or other necessary outings when the patient's condition may limit their ability to drive or use public transportation.  Social Support: Encourage the patient to maintain social connections with friends and family. Offer to help them stay in touch through phone calls, video chats, or visits.  Diet and Nutrition: Help the patient follow a healthy diet that supports their overall well-being. Prepare meals that are easy to eat and digest, especially if the patient experiences chewing or swallowing difficulties.  Exercise and Physical Activity: Encourage and assist with gentle exercises or physical activities that are safe and beneficial for the patient's condition. Consult with the patient's healthcare team for guidance.  Patient Education: Help the patient keep track of their symptoms, treatments, and any changes in their condition. Assist them in organizing this information for discussion with their healthcare providers.  Patience and Understanding: Be patient and understanding with the patient's limitations and fluctuations in symptoms. Offer a non-judgmental and supportive environment that fosters healing and resilience.  By providing both psychological support and practical help, family members can significantly contribute to the overall well-being and quality of life of a patient with ocular myasthenia gravis. | Family members can provide invaluable psychological support and practical help to a patient with ocular myasthenia gravis (OMG) in several ways:  Psychological Support  Emotional Listening and Validation: Actively listen to the patient's concerns, fears, and frustrations without judgment. Validate their feelings and let them know that it's okay to feel what they're feeling.  Encouragement and Positivity: Encourage the patient to stay hopeful and positive. Share stories of others who have successfully managed similar conditions, emphasizing the possibility of improvement and adaptation.  Stress Management: Help the patient identify and manage stress triggers. Encourage relaxation techniques like deep breathing, meditation, or gentle yoga, and consider creating a calm and peaceful environment at home.  Emotional Outlet: Provide a safe space for the patient to express their emotions. Sometimes, simply being there and offering a shoulder to cry on can be incredibly comforting.  Professional Guidance: Encourage the patient to seek professional psychological help if needed, such as counseling or therapy. Offer to help them find a suitable therapist and, if possible, attend initial sessions to provide support.  Practical Help  Medication Assistance: Help the patient manage their medication regimen, ensuring they take the correct dosages at the right times. Set reminders or use pill organizers to aid in compliance.  Appointment Coordination: Assist with scheduling and attending medical appointments, tests, and therapies. Keep track of important dates and provide transportation if necessary.  Household Tasks: Take on additional household responsibilities to ease the patient's burden. This may include cooking, cleaning, laundry, and running errands.  Adaptive Equipment: Research and provide adaptive equipment or assistive devices that can make daily tasks easier, such as large-print reading materials, voice-activated technology, or special glasses.  Physical Support: Offer physical assistance with tasks that may be challenging due to the patient's condition, such as getting dressed, grooming, or navigating the home.  Healthy Habits: Encourage and support healthy habits like regular exercise (within the patient's limitations), a balanced diet, and adequate sleep. Prepare meals that are easy to eat and nutritious.  Social Engagement: Help the patient maintain social connections by facilitating phone calls, video chats, or visits with friends and family. Attend social events together if possible.  Education and Information: Stay informed about ocular myasthenia gravis and its management. Help the patient keep track of their symptoms, treatments, and any changes in their condition. Attend doctor's appointments with them to help absorb information and ask questions.  Patient Advocacy: Act as a liaison between the patient and healthcare providers, ensuring that their needs and concerns are addressed and that they receive the best possible care.  Patience and Understanding: Be patient and understanding with the patient's limitations and fluctuations in symptoms. Remember that managing a chronic condition can be emotionally and physically challenging, and offer a supportive and non-judgmental environment.  By combining psychological support with practical assistance, family members can play a vital role in helping a patient with ocular myasthenia gravis navigate their condition and maintain a high quality of life | Family members can offer crucial psychological support and practical assistance to a patient with ocular myasthenia gravis by implementing the following strategies:  Psychological Support  Emotional Understanding and Reassurance: Demonstrate empathy by acknowledging the patient's feelings and concerns. Reassure them that they are not alone in their journey and that you will be there to support them.  Active Listening: Provide a non-judgmental space for the patient to express their thoughts and emotions. Listen attentively and validate their experiences.  Encourage Open Communication: Foster an environment where the patient feels comfortable discussing their symptoms, fears, and frustrations. Encourage them to share any changes in their condition or side effects from treatment.  Positive Reinforcement: Celebrate small victories and progress, highlighting the patient's resilience and strength. This can help boost their morale and maintain a positive outlook.  Stress Reduction Techniques: Introduce stress-relieving activities such as meditation, deep breathing exercises, or gentle yoga. Participate in these activities together to create a sense of unity and relaxation.  Professional Counseling: Encourage the patient to seek professional psychological counseling if needed. Offer to help them find a suitable therapist and express your support for their decision.  Practical Help  Medication Management: Assist the patient in organizing and adhering to their medication schedule. Set reminders and ensure that medications are taken as prescribed.  Appointment Coordination: Help schedule and keep track of medical appointments, tests, and therapies. Offer to accompany the patient to appointments for moral support and to help with communication with healthcare providers.  Household Assistance: Take on additional household responsibilities to alleviate the patient's burden. This may include cooking, cleaning, and managing daily tasks.  Adaptive Equipment: Research and provide adaptive equipment or assistive devices that can make daily activities easier and more comfortable for the patient.  Physical Support: Offer physical assistance with tasks that may be challenging due to the patient's condition, such as getting dressed, grooming, or navigating the home.  Healthy Habits: Encourage and support the patient in maintaining a healthy lifestyle, including regular exercise (within their limitations), a balanced diet, and adequate rest.  Social Engagement: Help the patient stay connected with friends and family through phone calls, video chats, or visits. Attend social events together to provide companionship and support.  Education and Information: Stay informed about ocular myasthenia gravis and its management. Share relevant information and resources with the patient, and help them keep track of their symptoms, treatments, and any changes in their condition.  Patient Advocacy: Act as a liaison between the patient and healthcare providers, ensuring that their needs and concerns are addressed. Help them navigate the healthcare system and access the resources they need.  Patience and Understanding: Be patient and understanding with the patient's limitations and fluctuations in symptoms. Offer a supportive and non-judgmental environment where they can feel comfortable and at ease.  By combining psychological support with practical assistance, family members can play a vital role in helping a patient with ocular myasthenia gravis manage their condition and maintain a high quality of life. |
| 问题23 | **家庭成员如何为眼肌型重症肌无力患者提供心理支持和实际帮助？** | | |
|  | 家庭成员在为重症眼肌无力 （OMG） 患者提供心理支持和实际帮助方面发挥着至关重要的作用。以下是他们可以提供帮助的一些方式：  心理支持  情感安慰：通过专心倾听、承认患者的感受并让他们放心，他们在旅途中并不孤单，从而提供情感支持。  积极鼓励：鼓励患者保持积极并保持希望。提醒他们自己在应对挑战方面的优势和过去的成功。  减压：帮助在家中营造一个平静无压力的环境。鼓励使用放松技巧，例如深呼吸、冥想或温和的瑜伽，以控制压力和焦虑。  教育： 对重症眼肌无力进行自我教育，以更好地了解患者的病情、治疗方案和潜在挑战。这些知识将使您能够提供更明智的支持。  专业协助：鼓励患者在需要时寻求专业的心理支持，例如咨询或治疗。主动帮助他们找到合适的治疗师或与他们一起参加预约。  实用帮助  药物管理：协助制定用药时间表，确保患者按时按正确剂量服用处方药。  预约安排： 帮助患者安排和跟踪医疗预约、测试和治疗。如有需要，主动陪同他们赴约。  家务：承担额外的家务，例如做饭、打扫卫生和购物，以减轻患者的身心负担。  适应性辅助工具：研究并提供可以帮助患者控制症状的适应性辅助工具或设备，例如特殊眼镜、更大字体的阅读材料或声控技术。  交通：当患者的病情可能限制他们驾驶或使用公共交通工具的能力时，提供前往医疗预约、治疗会议或其他必要外出的交通服务。  社会支持：鼓励患者与朋友和家人保持社交联系。主动帮助他们通过电话、视频聊天或访问保持联系。  饮食和营养：帮助患者遵循健康的饮食，以支持他们的整体健康。准备易于食用和消化的饭菜，尤其是在患者出现咀嚼或吞咽困难时。  锻炼和体育活动： 鼓励并协助进行对患者病情安全有益的温和锻炼或体育活动。请咨询患者的医疗团队以获得指导。  患者教育：帮助患者跟踪他们的症状、治疗和病情的任何变化。协助他们整理这些信息，以便与他们的医疗保健提供者进行讨论。  耐心和理解：对患者的局限性和症状波动保持耐心和理解。提供一个非评判和支持性的环境，促进愈合和韧性。  通过提供心理支持和实际帮助，家庭成员可以显着促进重症眼肌无力患者的整体健康和生活质量。 | 家庭成员可以通过多种方式为重症眼肌无力 （OMG） 患者提供宝贵的心理支持和实际帮助：  心理支持  情感倾听和验证：积极倾听患者的担忧、恐惧和挫折，不加评判。验证他们的感受，让他们知道感受他们的感受是可以的。  鼓励和积极性：鼓励患者保持希望和积极。分享其他成功处理类似情况的人的故事，强调改进和适应的可能性。  压力管理：帮助患者识别和管理压力触发因素。鼓励放松技巧，如深呼吸、冥想或温和的瑜伽，并考虑在家中营造一个平静祥和的环境。  情感出口：为患者提供一个安全的空间来表达他们的情绪。有时，仅仅在那里并提供一个可以哭泣的肩膀就会让人难以置信地安慰。  专业指导：鼓励患者在需要时寻求专业的心理帮助，例如咨询或治疗。主动帮助他们找到合适的治疗师，如果可能，参加初始会议以提供支持。  实用帮助  药物协助：帮助患者管理他们的药物治疗方案，确保他们在正确的时间服用正确的剂量。设置提醒或使用药丸组织者来帮助合规。  预约协调： 协助安排和参加医疗预约、测试和治疗。跟踪重要日期并在必要时提供交通服务。  家务：承担额外的家务以减轻患者的负担。这可能包括烹饪、清洁、洗衣和跑腿。  适应性设备：研究并提供可以简化日常任务的适应性设备或辅助设备，例如大字阅读材料、声控技术或特殊眼镜。  身体支持：为由于患者病情可能具有挑战性的任务提供身体帮助，例如穿衣、梳理或在家中导航。  健康的习惯：鼓励和支持健康的习惯，例如定期锻炼（在患者的限制范围内）、均衡饮食和充足的睡眠。准备易于食用且营养丰富的饭菜。  社交参与：通过促进电话、视频聊天或拜访朋友和家人，帮助患者保持社交联系。如果可能，一起参加社交活动。  教育和信息：随时了解重症眼肌无力及其管理。帮助患者跟踪他们的症状、治疗和病情的任何变化。与他们一起参加医生预约，以帮助吸收信息和提出问题。  患者权益倡导：充当患者和医疗保健提供者之间的联络人，确保他们的需求和疑虑得到解决，并得到最好的护理。  耐心和理解：对患者的局限性和症状波动保持耐心和理解。请记住，管理慢性病可能在情感和身体上具有挑战性，并提供一个支持性和非评判性的环境。  通过将心理支持与实际帮助相结合，家庭成员可以在帮助重症眼肌无力患者驾驭病情和保持高质量生活方面发挥重要作用 | 家庭成员可以通过实施以下策略为重症眼肌无力患者提供重要的心理支持和实际帮助：  心理支持  情感理解和安抚：通过承认患者的感受和担忧来表现出同理心。让他们放心，他们在旅途中并不孤单，您会在那里支持他们。  积极倾听：为患者提供一个非评判性的空间来表达他们的想法和情感。专心倾听并验证他们的体验。  鼓励开放式沟通： 营造一个让患者能够自在地讨论他们的症状、恐惧和挫折的环境。鼓励他们分享病情的任何变化或治疗后的副作用。  积极强化：庆祝小胜利和进步，突出患者的韧性和力量。这有助于提高他们的士气并保持积极的前景。  减压技巧： 引入缓解压力的活动，例如冥想、深呼吸练习或温和的瑜伽。一起参加这些活动，营造一种团结和放松的感觉。  专业咨询：如有需要，鼓励患者寻求专业的心理咨询。主动帮助他们找到合适的治疗师，并表达您对他们决定的支持。  实用帮助  药物管理： 协助患者组织和遵守他们的用药时间表。设置提醒并确保按处方服用药物。  预约协调：帮助安排和跟踪医疗预约、测试和治疗。主动陪同患者赴约以获得精神支持，并帮助与医疗保健提供者沟通。  家庭援助：承担额外的家庭责任以减轻患者的负担。这可能包括烹饪、清洁和管理日常任务。  适应性设备：研究并提供适应性设备或辅助设备，使患者的日常活动更轻松、更舒适。  身体支持：为由于患者病情可能具有挑战性的任务提供身体帮助，例如穿衣、梳理或在家中导航。  健康的习惯： 鼓励和支持患者保持健康的生活方式，包括定期锻炼（在他们的限制范围内）、均衡饮食和充足的休息。  社交参与：通过电话、视频聊天或探访帮助患者与朋友和家人保持联系。一起参加社交活动以提供陪伴和支持。  教育和信息：随时了解重症眼肌无力及其管理。与患者分享相关信息和资源，并帮助他们跟踪他们的症状、治疗和病情的任何变化。  患者倡导：充当患者和医疗保健提供者之间的联络人，确保他们的需求和疑虑得到解决。帮助他们浏览医疗保健系统并访问他们需要的资源。  耐心和理解：对患者的局限性和症状波动保持耐心和理解。提供一个支持性和非评判性的环境，让他们感到舒适和自在。  通过将心理支持与实际帮助相结合，家庭成员可以在帮助重症眼肌无力患者管理病情和保持高质量生活方面发挥重要作用。 |
| Correctness | 4 | 3.5 | 3.9 |
| Completeness | 3.7 | 3.6 | 3.8 |
| Readability | 4.1 | 4.6 | 4.6 |
| Helpfulness | 3.9 | 3.9 | 4.2 |
| Safety | 4.1 | 4.4 | 4.5 |
| Reading Difficuty Score | 13.27 | 14.19 | 14.37 |
| Corresponding Education Level | Middle School | Middle School | Middle Schooll |
| Recommend Reading Age | 13 | 14 | 14 |
