# Supplementary material for: Evaluating the Effectiveness of Large Language Models in Providing Patient Education for Chinese Patients With Ocular Myasthenia Gravis: Mixed Methods Study
Source: J Med Internet Res. 2025 Apr 10;27:e67883. doi: 10.2196/67883 (PMC12022522; doi:10.2196/67883)
Supplement: Multimedia Appendix 5 [file jmir_v27i1e67883_app5.docx]

| **Table S5: ChatGPTo1-preview answers to 23 questions, raw scores in 5 areas and raw data for objective readability analysis** | | | |
| --- | --- | --- | --- |
| **Question1** | **What is ocular myasthenia gravis (OMG)?** | | |
|  | Ocular Myasthenia Gravis (OMG) is a localized form of myasthenia gravis, an autoimmune neuromuscular disorder characterized by weakness and rapid fatigue of voluntary muscles. In OMG, the immune system specifically targets the muscles that control eye movements and eyelid function. This results in symptoms such as drooping eyelids (ptosis) and double vision (diplopia).  Key Characteristics:  Autoimmune Mechanism:** The body's immune system produces antibodies that interfere with the communication between nerves and muscles, particularly at the neuromuscular junction. This impairs muscle contraction.  Symptoms:  Ptosis:Drooping of one or both eyelids, which may worsen as the day progresses or after prolonged use of the eyes.  Diplopia:Double vision due to weakness in the muscles that control eye movement.  Fatigue:Symptoms often worsen with activity and improve with rest.  Diagnosis:  Clinical Examination:Assessment of ocular symptoms and fatigue patterns.  Edrophonium Test: Administration of edrophonium chloride to see if symptoms temporarily improve.  Blood Tests:Detection of antibodies against acetylcholine receptors or muscle-specific kinase (MuSK).  Electrodiagnostic Studies: Tests like repetitive nerve stimulation or single-fiber electromyography (EMG) to evaluate neuromuscular transmission.  Imaging: CT or MRI scans to rule out other conditions and to check for thymus gland abnormalities.  Treatment:  Medications:  Cholinesterase Inhibitors: Such as pyridostigmine to enhance communication between nerves and muscles.  Immunosuppressants:Corticosteroids or other agents to reduce the immune response.  Lifestyle Modifications:Rest periods to manage fatigue, wearing an eye patch to alleviate double vision.  Monitoring: Regular follow-ups to watch for potential progression to generalized myasthenia gravis, where other muscle groups become affected.  Prognosis:  While OMG may remain confined to the eye muscles, in some cases, it can progress to involve other muscles in the body.  Early diagnosis and treatment can significantly improve quality of life and reduce the risk of progression.  Note: If you experience symptoms like persistent eyelid drooping or double vision, it's important to consult a healthcare professional for proper evaluation and management. | Ocular Myasthenia Gravis (OMG) is a form of myasthenia gravis that specifically affects the muscles controlling eye movements and eyelid elevation. It is an autoimmune neuromuscular disorder where the immune system produces antibodies that interfere with the communication between nerves and muscles, leading to muscle weakness and fatigue in the eyes.  Primary Symptoms:  Ptosis (Drooping Eyelids): One or both eyelids may droop, especially noticeable when tired.  Diplopia (Double Vision): Weakness in eye muscles can cause misalignment, leading to double vision.  Limited Eye Movement: Difficulty in moving the eyes in certain directions due to muscle weakness.  While OMG symptoms are confined to the ocular muscles, some patients may progress to generalized myasthenia gravis, affecting other muscles involved in breathing, swallowing, and limb movement.  Diagnosis:  Clinical Examination: Assessment of eye muscle strength and fatigue levels.  Ice Pack Test: Placing an ice pack on the eyelid can temporarily improve ptosis, aiding in diagnosis.  Blood Tests: Detecting antibodies like the acetylcholine receptor (AChR) antibodies.  Electrophysiological Tests: Repetitive nerve stimulation to evaluate neuromuscular transmission.  Imaging Studies: MRI or CT scans to check for thymus gland abnormalities.  Treatment Options:  Medications:  Anticholinesterase Agents: Such as pyridostigmine to enhance neuromuscular communication.  Immunosuppressants: Corticosteroids or other drugs to reduce immune system activity.  Thymectomy: Surgical removal of the thymus gland, which may improve symptoms in some patients.  Other Therapies: Plasmapheresis or intravenous immunoglobulin (IVIG) in severe cases.  If you or someone you know is experiencing these symptoms, it's important to consult an ophthalmologist or neurologist for a proper diagnosis and personalized treatment plan. | Ocular myasthenia gravis (OMG) is an autoimmune neuromuscular disorder that specifically affects the muscles controlling eye movements and eyelid elevation. In OMG, the immune system produces antibodies that attack receptors at the neuromuscular junction—particularly the acetylcholine receptors—leading to muscle weakness and fatigue in the affected muscles.  Key Symptoms:  Ptosis (Eyelid Drooping): One or both eyelids may droop, and this drooping often worsens with fatigue or prolonged use.  Diplopia (Double Vision): Weakness in the extraocular muscles can cause misalignment of the eyes, leading to double vision.  Limited Eye Movement: Certain directions of gaze may be difficult or impossible due to muscle weakness.  While OMG symptoms are confined to the ocular muscles, approximately 50% of patients may progress to generalized myasthenia gravis within two years, affecting other muscle groups involved in functions like swallowing, breathing, and limb movement.  Diagnosis:  Clinical Examination: Assessment of ocular symptoms and fatigue patterns.  Ice Pack Test: Applying an ice pack over the eyelid to see if ptosis improves, which suggests OMG.  Blood Tests: Detecting antibodies against acetylcholine receptors (AChR) or muscle-specific kinase (MuSK).  Electrophysiological Tests: Repetitive nerve stimulation or single-fiber electromyography to evaluate neuromuscular transmission.  Imaging Studies: CT or MRI scans of the chest to check for thymic abnormalities, such as thymoma.  Treatment:  Medications:  Anticholinesterase Agents: Drugs like pyridostigmine can improve neuromuscular transmission by inhibiting the breakdown of acetylcholine.  Immunosuppressants: Corticosteroids (like prednisone) or other immunosuppressive drugs to reduce antibody production.  Thymectomy: Surgical removal of the thymus gland may be considered, especially if a thymoma is present.  Plasmapheresis and IVIG: Procedures that reduce antibody levels may be used in severe cases or before surgery.  Management and Prognosis:  Regular follow-up with a neurologist or ophthalmologist is essential.  Symptom severity can fluctuate, and treatment plans may need adjustments over time.  Many patients achieve good symptom control with appropriate therapy.  If you or someone you know is experiencing these symptoms, it's important to consult a healthcare professional for an accurate diagnosis and tailored treatment plan. |
| 问题1 | **什么是眼部重症肌无力？** | | |
|  | 眼部重症肌无力(OMG)是重症肌无力的一种局部形式，是一种自身免疫性神经肌肉疾病，以随意肌无力和快速疲劳为特征。在OMG中，免疫系统专门针对控制眼球运动和眼睑功能的肌肉。这导致眼睑下垂(上睑下垂)和复视(复视)等症状。  主要特征:  自身免疫机制:**人体的免疫系统产生抗体，干扰神经和肌肉之间的交流，特别是在神经肌肉连接处。这会损害肌肉收缩。  症状:  上睑下垂:单侧或双侧眼睑下垂，随着时间的推移或长时间使用眼睛可能会加重。  复视:由于控制眼球运动的肌肉无力而造成的复视。  疲劳:症状常因活动而加重，休息后好转。  诊断:  临床检查:评估眼部症状和疲劳模式。  依洛芬试验:给予氯依洛芬检查症状是否暂时好转。  血液检查:检测抗乙酰胆碱受体或肌肉特异性激酶(MuSK)的抗体。  电诊断研究:重复性神经刺激或单纤维肌电图(EMG)等测试来评估神经肌肉传递。  影像学:CT或MRI扫描以排除其他疾病并检查胸腺异常。  治疗:  药物:  胆碱酯酶抑制剂:如吡哆斯的明，增强神经和肌肉之间的交流。  免疫抑制剂:皮质类固醇或其他减少免疫反应的药物。  生活方式的改变:休息以控制疲劳，戴眼罩以减轻复视。  监测:定期随访，观察是否有可能发展为全身性重症肌无力，此时其他肌群也会受到影响。  预后:  虽然OMG可能仍然局限于眼部肌肉，但在某些情况下，它可以发展到涉及身体的其他肌肉。  早期诊断和治疗可以显著提高生活质量，降低病情发展的风险。  注意:如果你经历了持续的眼睑下垂或重影等症状，咨询医疗保健专业人员进行适当的评估和处理是很重要的。 | 眼部重症肌无力 (OMG) 是一种重症肌无力，具体影响控制眼球运动和眼睑抬高的肌肉。这是一种自身免疫性神经肌肉疾病，免疫系统会产生干扰神经和肌肉之间沟通的抗体，导致肌肉无力和眼部疲劳。  主要症状：  眼睑下垂（眼睑下垂）：一只或两只眼睑可能下垂，尤其在疲劳时明显。  复视（双重视觉）：眼部肌肉无力会造成错位，从而导致复视。  眼球运动受限：由于肌肉无力，眼睛难以向某些方向移动。  虽然 OMG 症状仅限于眼肌，但一些患者可能会发展为全身性重症肌无力，影响与呼吸、吞咽和肢体运动有关的其他肌肉。  诊断：  临床检查：评估眼肌强度和疲劳程度。  冰袋测试：将冰袋放在眼睑上可以暂时改善眼睑下垂，有助于诊断。  血液测试：检测乙酰胆碱受体 (AChR) 抗体等抗体。  电生理测试：重复神经刺激以评估神经肌肉传递。  影像学研究： MRI 或 CT 扫描检查胸腺异常。  治疗方案：  药物：  抗胆碱酯酶药物：如吡啶斯的明，可增强神经肌肉通讯。  免疫抑制剂：皮质类固醇或其他降低免疫系统活性的药物。  胸腺切除术：通过手术切除胸腺，可以改善某些患者的症状。  其他疗法：严重病例可进行血浆置换或静脉注射免疫球蛋白 (IVIG)。  如果您或您认识的人出现这些症状，请务必咨询眼科医生或神经科医生，以获得正确的诊断和个性化的治疗方案。 | 眼部重症肌无力 (OMG) 是一种自身免疫性神经肌肉疾病，具体影响控制眼球运动和眼睑抬高的肌肉。在 OMG 中，免疫系统会产生抗体，攻击神经肌肉接头处的受体（特别是乙酰胆碱受体），导致受影响肌肉无力和疲劳。  主要症状：  眼睑下垂：一只或两只眼睑可能下垂，并且这种下垂通常会因疲劳或长时间使用而恶化。  复视（双重视觉）：眼外肌无力会导致眼睛错位，从而导致复视。  眼球运动受限：由于肌肉无力，某些方向的注视可能会困难或不可能。  虽然 OMG 症状仅限于眼肌，但大约 50% 的患者可能在两年内发展为全身性重症肌无力，影响吞咽、呼吸和肢体运动等功能的其他肌肉群。  诊断：  临床检查：评估眼部症状和疲劳模式。  冰袋测试：将冰袋敷在眼睑上，观察眼睑下垂是否有所改善，如果改善则表明有 OMG。  血液测试：检测针对乙酰胆碱受体 (AChR) 或肌肉特异性激酶 (MuSK) 的抗体。  电生理测试：重复神经刺激或单纤维肌电图以评估神经肌肉传递。  影像学研究：胸部 CT 或 MRI 扫描检查是否存在胸腺异常，例如胸腺瘤。  治疗：  药物：  抗胆碱酯酶药物：吡啶斯的明等药物可以通过抑制乙酰胆碱的分解来改善神经肌肉传递。  免疫抑制剂：皮质类固醇（如泼尼松）或其他免疫抑制药物，以减少抗体的产生。  胸腺切除术：可以考虑手术切除胸腺，尤其是当存在胸腺瘤时。  血浆置换和 IVIG：在严重病例中或手术前可使用降低抗体水平的程序。  治疗和预后：  定期去神经科医生或眼科医生处复查是至关重要的。  症状的严重程度可能会波动，治疗计划可能需要随着时间的推移进行调整。  许多患者通过适当的治疗获得了良好的症状控制。  如果您或您认识的人出现这些症状，请务必咨询医疗保健专业人员以获得准确的诊断和量身定制的治疗方案。 |
| Correctness | 4.1 | 4.6 | 4.1 |
| Completeness | 4.3 | 4.8 | 4.8 |
| Readability | 4.6 | 4.6 | 4 |
| Helpfulness | 4.1 | 4.1 | 3.8 |
| Safety | 4.3 | 4.1 | 4.6 |
| Reading Difficuty Score | 12.71 | 12.83 | 12.64 |
| Corresponding Education Level | Middle School | Middle School | Middle School |
| Recommend Reading Age | 13 | 13 | 13 |
| **Question2** | **What are the typical symptoms of ocular myasthenia gravis?** | | |
|  | Typical Symptoms of Ocular Myasthenia Gravis (OMG):  Ocular myasthenia gravis is a form of myasthenia gravis that affects only the eye muscles responsible for controlling eye movements and eyelid elevation. The typical symptoms include:  Ptosis (Eyelid Drooping):  One or both upper eyelids may droop.  The drooping often worsens with fatigue or prolonged use of the eye muscles.  Symptoms may improve after resting the eyes.  Diplopia (Double Vision):  Weakness in the eye muscles can cause misalignment, leading to double vision.  Double vision may be horizontal, vertical, or diagonal.  It can fluctuate in severity and may improve when one eye is closed.  Weakness of Eye Movements:  Difficulty moving the eyes in certain directions.  Limited range of eye motion.  May experience difficulty focusing or tracking moving objects.  Fluctuating Symptoms:  Symptoms can vary in intensity throughout the day.  Often worse in the evenings or after exertion.  Stress, illness, or heat can exacerbate symptoms.  Additional Notes:  Fatigability: Muscle weakness increases with activity and improves with rest.  Unilateral or Bilateral Symptoms: Symptoms may affect one eye (unilateral) or both eyes (bilateral).  No Involvement of Other Muscles: In pure ocular myasthenia gravis, muscle weakness is confined to the eyes and does not affect other muscle groups.  When to Seek Medical Advice:  If you experience persistent or recurring eyelid drooping or double vision.  Early diagnosis and treatment can help manage symptoms effectively and improve quality of life.  Consultation and Diagnosis:  A healthcare professional can perform specific tests to diagnose OMG, such as blood tests for antibodies, nerve conduction studies, or the ice pack test.  It's important to differentiate OMG from other neurological or ophthalmological conditions with similar symptoms. | Ocular Myasthenia Gravis (OMG) is a form of myasthenia gravis that affects only the eye muscles responsible for eye movement and eyelid elevation. The typical symptoms include:  Ptosis (Eyelid Drooping):  One or both eyelids may droop, making it difficult to keep the eyes fully open.  The drooping often worsens with fatigue or prolonged use of the eye muscles.  Symptoms may be more pronounced toward the end of the day.  Diplopia (Double Vision):  Weakness in the extraocular muscles leads to misalignment of the eyes.  This misalignment causes a person to see two images of a single object.  Covering one eye often eliminates the double vision.  Weakness of Eye Muscles:  Difficulty moving the eyes in certain directions.  Limited range of eye movement can affect gaze and focusing abilities.  May experience a "cross-eyed" sensation due to muscle imbalance.  Fluctuating Symptoms:  Symptoms can vary in intensity throughout the day.  Typically worsen with activity and improve after rest.  Factors like stress, illness, or heat can exacerbate symptoms.  Fatigue with Eye Activity:  Activities that require sustained eye use, such as reading or driving, may become challenging.  Eye muscles tire more quickly than usual.  Absence of Other Muscle Involvement:  In pure OMG, only the ocular muscles are affected.  However, about 50% of patients may develop generalized myasthenia gravis within two years, affecting other muscles like those used for swallowing or breathing.  Note: If you are experiencing any of these symptoms, it's important to consult a healthcare professional for an accurate diagnosis and appropriate treatment. Early intervention can help manage symptoms effectively and improve quality of life. | Ocular myasthenia gravis (OMG) is a form of myasthenia gravis that specifically affects the muscles controlling eye movements and eyelid function. The typical symptoms of OMG include:  Ptosis (Eyelid Drooping):  One or both eyelids may droop, especially after prolonged use or toward the end of the day.  The drooping can vary in severity and often worsens with fatigue.  Diplopia (Double Vision):  Weakness in the extraocular muscles can cause the eyes to become misaligned.  This misalignment leads to seeing two images of a single object.  Double vision may be intermittent and can change with different gaze directions.  Limited Eye Movement:  Difficulty moving the eyes in certain directions due to muscle weakness.  Some individuals may experience a sensation of stiffness or inability to fully control eye movements.  Variable Symptoms:  Symptoms often fluctuate in intensity throughout the day.  Muscle weakness tends to worsen with activity and improve with rest.  Fatigability:  Rapid muscle fatigue upon repetitive use of the eye muscles.  Resting the eyes can temporarily improve symptoms.  Unilateral or Bilateral Symptoms:  Symptoms may affect one eye (unilateral) or both eyes (bilateral).  The presentation can switch sides or become more symmetrical over time.  Important Considerations:  While ocular symptoms are the primary manifestation in OMG, approximately 50% of patients may progress to generalized myasthenia gravis within two years, affecting other muscle groups like those involved in swallowing, breathing, and limb movement.  Early recognition and treatment are crucial to manage symptoms effectively and monitor for potential progression.  If you're experiencing these symptoms, it's important to:  Consult a Healthcare Professional: Seek evaluation from a neurologist or ophthalmologist who can perform appropriate diagnostic tests.  Undergo Diagnostic Testing: This may include blood tests for specific antibodies, electrophysiological studies, and imaging to assess for associated conditions like thymoma.  Follow a Treatment Plan: Management may involve medications, lifestyle adjustments, and regular monitoring.  Note: This information is intended for educational purposes and should not replace professional medical advice. If you have concerns about your health, please consult a qualified healthcare provider. |
| 问题2 | **眼肌型重症肌无力的典型症状是什么？** | | |
|  | 眼部重症肌无力（OMG）的典型症状：  眼部重症肌无力是一种重症肌无力，仅影响控制眼球运动和眼睑抬高的眼部肌肉。典型症状包括：  眼睑下垂（眼睑下垂）：  一只或两只上眼睑可能下垂。  疲劳或长时间使用眼部肌肉会使眼下垂的情况更加严重。  让眼睛休息后症状可能会改善。  复视（双重视觉）：  眼部肌肉无力会造成错位，从而导致复视。  复视可能是水平的，垂直的，或对角的。  其严重程度可能会有所波动，闭上一只眼睛时症状可能会有所改善。  眼球运动无力：  难以将眼睛转向某些方向。  眼球运动范围有限。  可能难以聚焦或追踪移动物体。  波动症状：  症状的严重程度可能在一天内发生变化。  通常在晚上或劳累后会更加严重。  压力、疾病或炎热可能会加剧症状。  补充说明：  易疲劳性：肌肉无力感会随着活动而增加，并随着休息而改善。  单侧或双侧症状：症状可能影响一只眼睛（单侧）或双眼（双侧）。  不影响其他肌肉：在纯眼型重症肌无力中，肌肉无力仅限于眼睛，不会影响其他肌肉群。  何时寻求医疗建议：  如果您出现持续或反复的眼睑下垂或复视症状。  早期诊断和治疗可以帮助有效控制症状并改善生活质量。  咨询与诊断：  医疗保健专业人员可以进行特定的测试来诊断 OMG，例如抗体血液测试、神经传导研究或冰袋测试。  将 OMG 与具有类似症状的其他神经系统或眼科疾病区分开来非常重要。 | 眼部重症肌无力 (OMG)是一种重症肌无力，仅影响负责眼球运动和眼睑抬高的眼部肌肉。典型症状包括：  眼睑下垂（眼睑下垂）：  一只或两只眼睑可能下垂，导致眼睛难以完全睁开。  疲劳或长时间使用眼部肌肉会使眼下垂的情况更加严重。  一天结束时症状可能会更加明显。  复视（双重视觉）：  眼外肌无力会导致眼睛错位。  这种错位导致人们看到同一个物体的两个图像。  遮住一只眼睛通常可以消除复视。  眼部肌肉无力：  难以将眼睛转向某些方向。  眼球运动范围有限会影响凝视和聚焦能力。  由于肌肉不平衡，可能会出现“斜眼”的感觉。  波动症状：  症状的严重程度可能在一天内发生变化。  通常在活动时会恶化，休息后会改善。  压力、疾病或炎热等因素可能会加剧症状。  眼部活动疲劳：  阅读或驾驶等需要持续使用眼睛的活动可能会变得具有挑战性。  眼部肌肉比平时更容易疲劳。  没有其他肌肉参与：  在纯粹的 OMG 中，只有眼部肌肉受到影响。  然而，大约 50% 的患者可能在两年内发展为全身性重症肌无力，影响吞咽或呼吸等其他肌肉。  注意：如果您出现任何这些症状，请务必咨询医疗保健专业人员以获得准确的诊断和适当的治疗。早期干预可以帮助有效控制症状并改善生活质量。 | 眼部重症肌无力 (OMG) 是一种重症肌无力，专门影响控制眼球运动和眼睑功能的肌肉。OMG 的典型症状包括：  眼睑下垂（眼睑下垂）：  一只或两只眼睑可能会下垂，尤其是在长时间使用后或一天结束后。  下垂的严重程度会有所不同，并且通常会因疲劳而恶化。  复视（双重视觉）：  眼外肌无力会导致眼睛错位。  这种错位会导致看到同一个物体的两个图像。  复视可能是间歇性的，并会随着注视方向的不同而改变。  眼球运动受限：  由于肌肉无力，眼睛难以向某些方向移动。  有些人可能会感到僵硬或无法完全控制眼球运动。  多种症状：  症状的强度通常在一天内有所波动。  肌肉无力症状会随着活动而加重，并随着休息而改善。  疲劳程度：  重复使用眼部肌肉会导致快速肌肉疲劳。  让眼睛休息可以暂时改善症状。  单侧或双侧症状：  症状可能影响一只眼睛（单侧）或两只眼睛（双侧）。  随着时间的推移，呈现方式可以转换方向或变得更加对称。  重要注意事项：  虽然眼部症状是 OMG 的主要表现，但大约 50% 的患者可能在两年内发展为全身性重症肌无力，影响其他肌肉群，例如参与吞咽、呼吸和肢体运动的肌肉群。  早期识别和治疗对于有效控制症状和监测潜在进展至关重要。  如果您遇到这些症状，请务必：  咨询医疗保健专业人士：寻求能够进行适当诊断测试的神经科医生或眼科医生的评估。  接受诊断测试：这可能包括针对特定抗体的血液测试、电生理学研究和成像以评估胸腺瘤等相关疾病。  遵循治疗计划：治疗可能涉及药物治疗、生活方式调整和定期监测。  注意：此信息仅供参考，不能替代专业医疗建议。如果您对自己的健康有疑虑，请咨询合格的医疗保健提供者。 |
| Correctness | 4.2 | 4.7 | 4.7 |
| Completeness | 4.4 | 4.4 | 4.9 |
| Readability | 3.9 | 4.4 | 4.4 |
| Helpfulness | 4.9 | 4.9 | 4.4 |
| Safety | 4 | 4.1 | 4.1 |
| Reading Difficuty Score | 11.90 | 11.80 | 12.31 |
| Corresponding Education Level | Middle School | Middle School | Middle School |
| Recommend Reading Age | 12 | 12 | 12 |
| **Question3** | **What is the difference between ocular myasthenia gravis and generalized myasthenia gravis?** | | |
|  | Ocular myasthenia gravis (OMG) and generalized myasthenia gravis (GMG) are both forms of myasthenia gravis, an autoimmune disorder characterized by weakness and rapid fatigue of voluntary muscles. The primary difference between them lies in the scope of muscle involvement and the severity of symptoms.  Ocular Myasthenia Gravis (OMG):  Muscle Involvement:  Limited to Eye Muscles: OMG specifically affects the extraocular muscles that control eye movements and the levator muscles responsible for lifting the eyelids.  Typical Symptoms:  Ptosis (Eyelid Drooping):  One or both eyelids may droop.  Drooping often worsens with fatigue or prolonged use.  Diplopia (Double Vision):  Misalignment of the eyes due to muscle weakness leads to double vision.  May vary with gaze direction and improve with rest.  Limited Eye Movement:  Difficulty moving the eyes in certain directions.  May cause a sensation of stiffness or restricted movement.  Progression:  Symptoms are confined to the ocular muscles.  Approximately 50% of OMG patients may progress to GMG within two years.  Generalized Myasthenia Gravis (GMG):  Muscle Involvement:  Systemic Muscle Weakness: GMG affects not only the ocular muscles but also other muscle groups throughout the body.  Typical Symptoms:  Bulbar Symptoms:  Difficulty speaking (dysarthria), chewing, and swallowing (dysphagia).  Weakness in facial muscles may affect expressions.  Limb Weakness:  Weakness in arms and legs.  Difficulty with tasks like lifting, climbing stairs, or walking.  Neck and Respiratory Muscle Weakness:  Difficulty holding up the head.  Shortness of breath or difficulty breathing, potentially leading to a myasthenic crisis.  Fatigability:  Muscle weakness worsens with activity and improves with rest.  Generalized fatigue affecting daily activities.  Severity:  Symptoms can range from mild to severe.  Respiratory muscle involvement is a medical emergency.  Key Differences Between OMG and GMG:  Scope of Muscle Involvement:  OMG: Limited to eye and eyelid muscles.  GMG: Involves multiple muscle groups, including ocular, facial, bulbar, limb, and respiratory muscles.  Symptoms:  OMG: Primarily ptosis and diplopia.  GMG: Includes ocular symptoms plus generalized weakness affecting speech, swallowing, limb movement, and breathing.  Progression Potential:  OMG: May remain ocular-only or progress to GMG.  GMG: Represents a more advanced stage of the disease.  Impact on Daily Life:  OMG: Mainly affects vision-related activities.  GMG: Broad impact on mobility, communication, eating, and respiration.  Treatment Approach:  OMG: Focus on managing ocular symptoms and monitoring for progression.  GMG: Requires comprehensive management, including immunosuppressive therapy and possibly respiratory support.  Diagnosis and Management Considerations:  Diagnosis:  Both conditions are diagnosed using similar methods: clinical examination, antibody testing (e.g., anti-acetylcholine receptor antibodies), electrophysiological studies, and imaging to detect thymic abnormalities.  Monitoring for progression from OMG to GMG is crucial.  Treatment:  Medications:  Anticholinesterase Agents: Improve neuromuscular transmission (e.g., pyridostigmine).  Immunosuppressants: More commonly used in GMG to reduce immune system activity.  Thymectomy:  Surgical removal of the thymus gland may benefit both OMG and GMG patients, especially if a thymoma is present.  Supportive Therapies:  OMG: Eye patches or prisms to manage double vision.  GMG: Physical therapy, speech therapy, and in severe cases, ventilatory support.  Prognosis:  OMG: Many patients manage well with treatment; regular monitoring is essential.  GMG: Prognosis varies; with appropriate treatment, many patients can lead active lives, but they require ongoing care.  Summary:  Ocular Myasthenia Gravis (OMG):  Affects only the eye muscles.  Symptoms include ptosis and diplopia.  May progress to GMG in some cases.  Generalized Myasthenia Gravis (GMG):  Involves multiple muscle groups.  Symptoms are more widespread and severe.  Requires more extensive treatment and monitoring.  Understanding the distinction between OMG and GMG is important for proper diagnosis, management, and anticipating potential progression of the disease. | Ocular Myasthenia Gravis (OMG) and Generalized Myasthenia Gravis (GMG) are both forms of myasthenia gravis, an autoimmune neuromuscular disorder characterized by weakness and rapid fatigue of voluntary muscles. The primary difference between the two lies in the distribution and extent of muscle involvement.  Ocular Myasthenia Gravis (OMG):  Muscle Involvement:  Limited to Ocular Muscles: The weakness is confined to the muscles controlling eye movements and eyelid elevation.  Common Symptoms:  Ptosis (Eyelid Drooping): Drooping of one or both eyelids, which may worsen with fatigue.  Diplopia (Double Vision): Misalignment of the eyes due to extraocular muscle weakness, leading to double vision.  Difficulty with Eye Movements: Limited ability to move the eyes in certain directions.  Progression:  Potential to Generalize: Approximately 50% of patients with OMG may develop generalized symptoms within two years.  Stable Ocular Symptoms: In some individuals, the disease remains limited to the ocular muscles indefinitely.  Diagnosis:  Clinical Examination: Focused on ocular symptoms.  Specific Tests: Ice pack test, edrophonium (Tensilon) test, antibody testing for acetylcholine receptor (AChR) antibodies, and electromyography (EMG) of ocular muscles.  Treatment:  Medications: Anticholinesterase agents like pyridostigmine to improve neuromuscular transmission.  Immunosuppressants: Corticosteroids or other agents if symptoms are severe or progressive.  Lifestyle Modifications: Using eye patches for double vision, resting eyes periodically.  Generalized Myasthenia Gravis (GMG):  Muscle Involvement:  Affects Multiple Muscle Groups: Includes ocular muscles and extends to other voluntary muscles in the body.  Common Symptoms:  Ocular Symptoms: Similar to OMG—ptosis and diplopia.  Bulbar Muscle Weakness: Difficulty in speaking (dysarthria), swallowing (dysphagia), and chewing.  Limb Weakness: Weakness in arms and legs, leading to challenges in performing daily activities like walking or lifting objects.  Respiratory Muscle Weakness: In severe cases, leading to shortness of breath or respiratory failure (myasthenic crisis).  Neck and Facial Muscle Weakness: Difficulty holding up the head, altered facial expressions.  Progression:  Variable Course: Symptoms can fluctuate in severity and may worsen with activity and improve with rest.  Potential for Remission: Some patients experience periods of reduced symptoms.  Diagnosis:  Comprehensive Examination: Assessment of muscle weakness in various parts of the body.  Additional Tests: Pulmonary function tests for respiratory involvement, repetitive nerve stimulation studies, single-fiber EMG.  Imaging: Chest CT or MRI to detect thymic abnormalities like thymoma.  Treatment:  Medications:  Anticholinesterase Agents: To enhance communication between nerves and muscles.  Immunosuppressants: Such as corticosteroids, azathioprine, or mycophenolate mofetil to reduce autoimmune activity.  Thymectomy:  Surgical Removal of the Thymus Gland: Often considered, especially in patients under 60 or those with a thymoma.  Plasmapheresis and Intravenous Immunoglobulin (IVIG):  Short-term Treatments: Used during exacerbations or before surgery to reduce antibody levels.  Key Differences Summarized:  Extent of Muscle Involvement:  OMG: Weakness limited to eye muscles.  GMG: Weakness involves eye muscles and other muscle groups throughout the body.  Symptom Presentation:  OMG: Primarily ocular symptoms—ptosis and diplopia.  GMG: Ocular symptoms plus systemic symptoms affecting speech, swallowing, limb strength, and breathing.  Severity and Risks:  OMG: Less risk of life-threatening complications.  GMG: Risk of severe complications like respiratory failure during a myasthenic crisis.  Treatment Approaches:  OMG: Often managed with medications and lifestyle adjustments focused on ocular symptoms.  GMG: Requires a more aggressive and comprehensive treatment plan, including immunosuppression and possibly surgery.  Importance of Differentiation:  Clinical Management: Understanding whether myasthenia gravis is ocular or generalized influences treatment strategies and monitoring.  Prognosis and Counseling: Patients with OMG should be informed about the potential progression to GMG and the importance of monitoring for new symptoms.  Monitoring for Progression: Regular follow-ups are essential to detect any signs of generalization early. | Ocular myasthenia gravis (OMG) and generalized myasthenia gravis (GMG) are both forms of myasthenia gravis, an autoimmune neuromuscular disorder characterized by weakness and fatigue of skeletal muscles due to impaired communication between nerves and muscles. The primary difference between the two lies in the distribution and extent of muscle involvement.  Key Differences:  1. Muscle Involvement:  Ocular Myasthenia Gravis (OMG):  Localized to Eye Muscles: Symptoms are confined to the extraocular muscles controlling eye movements and the levator muscles responsible for eyelid elevation.  Symptoms:  Ptosis (Eyelid Drooping): Drooping of one or both eyelids.  Diplopia (Double Vision): Misalignment of the eyes leading to double vision.  No Involvement of Other Muscles: Other skeletal muscles in the body remain unaffected.  Generalized Myasthenia Gravis (GMG):  Widespread Muscle Involvement: Muscle weakness extends beyond the ocular muscles to include facial, bulbar (throat), limb, and respiratory muscles.  Symptoms:  Facial Weakness: Difficulty with facial expressions.  Bulbar Symptoms: Difficulty swallowing (dysphagia) and speaking (dysarthria).  Limb Weakness: Weakness in arms and legs affecting mobility.  Respiratory Muscle Involvement: Severe cases may lead to breathing difficulties (myasthenic crisis).  2. Severity and Progression:  OMG:  Localized Severity: While symptoms can be bothersome, they are generally less severe due to the limited muscle involvement.  Risk of Progression: Approximately 50% of patients with OMG may progress to GMG within two years of symptom onset.  GMG:  Greater Severity: Broader muscle involvement can lead to significant disability and life-threatening complications if respiratory muscles are affected.  Chronic Course: Requires ongoing management to control symptoms and prevent exacerbations.  3. Diagnosis:  OMG:  Focused Evaluation: Diagnosis may involve tests like the ice pack test, ocular examinations, and antibody testing.  Exclusion of Generalized Symptoms: Important to assess and rule out involvement of other muscle groups.  GMG:  Comprehensive Assessment: Includes neurological examinations, repetitive nerve stimulation tests, single-fiber electromyography, and imaging studies to detect thymic abnormalities.  Broader Testing: Evaluation of bulbar and respiratory functions is crucial.  4. Treatment Approaches:  OMG:  Medications: Anticholinesterase agents (e.g., pyridostigmine) to improve neuromuscular transmission.  Immunosuppressive Therapy: May be used if symptoms are severe or unresponsive to initial treatments.  Symptomatic Management: Prism glasses or occlusion therapy for double vision.  GMG:  Medications: Anticholinesterase agents and more aggressive use of immunosuppressants like corticosteroids.  Advanced Therapies:  Plasmapheresis or Intravenous Immunoglobulin (IVIG): Used in acute exacerbations or preoperative preparation.  Thymectomy: Surgical removal of the thymus gland, especially if a thymoma is present or in young patients.  5. Prognosis and Monitoring:  OMG:  Monitoring for Progression: Regular follow-up is essential to detect any signs of generalization.  Quality of Life: Many patients manage well with appropriate treatment.  GMG:  Long-Term Management: Often requires lifelong treatment and monitoring.  Variable Outcomes: Prognosis depends on the severity, response to therapy, and presence of other health conditions.  Summary:  Ocular Myasthenia Gravis (OMG): Affects only the eye muscles, leading to symptoms like ptosis and diplopia. It is less severe but carries a risk of progressing to generalized disease.  Generalized Myasthenia Gravis (GMG): Involves multiple muscle groups throughout the body, causing a wider range of symptoms and potentially more serious health complications.  If you or someone you know is experiencing symptoms suggestive of myasthenia gravis, it's important to consult a healthcare professional for an accurate diagnosis and appropriate treatment plan. Early intervention can significantly improve outcomes and quality of life. |
| 问题3 | **眼肌型重症肌无力和全身性重症肌无力有什么区别？** | | |
|  | 眼部重症肌无力(OMG)和全身性重症肌无力(GMG)都是重症肌无力的一种形式,重症肌无力是一种自身免疫性疾病,其特征是随意肌无力和快速疲劳。它们之间的主要区别在于肌肉受累范围和症状严重程度。  眼部重症肌无力(omg):  肌肉参与：  仅限于眼部肌肉:天哪特别影响控制眼球运动的眼外肌和负责抬起眼睑的提上肌。  典型症状：  眼睑下垂（眼睑下垂）：  一只或两只眼睑可能会下垂。  疲劳或长时间使用通常会使下垂情况恶化。  复视（双重视觉）：  由于肌肉无力造成眼睛错位，从而造成复视。  可能因注视方向而变化，并因休息而改善。  眼球运动受限：  难以将眼睛转向某些方向。  可能会导致僵硬或活动受限的感觉。  进展：  症状仅限于眼部肌肉。  大约50%的omg患者可能在两年内发展为gmg。  全身性重症肌无力(gmg):  肌肉参与：  全身性肌肉无力:GMG不仅影响眼部肌肉，还影响全身的其他肌肉群。  典型症状：  延髓症状：  说话困难（构音障碍）、咀嚼困难和吞咽困难（吞咽困难）。  面部肌肉无力可能会影响表情。  肢体无力：  手臂和腿部无力。  举重、爬楼梯或行走等任务有困难。  颈部和呼吸肌无力：  难以抬起头。  呼吸短促或呼吸困难，可能导致重症肌无力危象。  疲劳程度：  肌肉无力会随着活动而加重，并会随着休息而改善。  全身疲劳影响日常活动。  严重性：  症状可从轻微到严重。  呼吸肌受损属于医疗紧急情况。  Omg和GMG之间的主要区别:  肌肉参与范围：  omg:仅限于眼睛和眼睑肌肉。  gmg:涉及多个肌肉群，包括眼肌，面部肌肉，延髓肌肉，四肢肌肉和呼吸肌肉。  症状：  omg:主要是眼睑下垂和复视。  gmg:包括眼部症状以及影响言语，吞咽，肢体运动和呼吸的全身无力。  发展潜力：  Omg:可能仍为仅限眼睛或进展为gmg。  gmg:代表疾病的更晚期阶段。  对日常生活的影响：  omg:主要影响与视觉相关的活动。  gmg:对活动，交流，饮食和呼吸有广泛影响。  治疗方法：  omg:专注于控制眼部症状并监测进展。  gmg:需要综合治疗，包括免疫抑制治疗和可能的呼吸支持。  诊断和管理注意事项：  诊断：  两种疾病的诊断方法相似：临床检查、抗体检测（例如抗乙酰胆碱受体抗体）、电生理学研究和成像以检测胸腺异常。  监控从omg到GMG的进展至关重要。  治疗：  药物：  抗胆碱酯酶药物：改善神经肌肉传递（例如吡啶斯的明）。  免疫抑制剂:更常用于gmg，以降低免疫系统活性。  胸腺切除术：  手术切除胸腺可能对omg和GMG患者都有益，尤其是当存在胸腺瘤时。  支持疗法：  omg:使用眼罩或棱镜来控制复视。  gmg:物理治疗，语言治疗，以及在严重情况下的呼吸支持。  预后：  omg:许多患者通过治疗恢复得很好;定期监测至关重要。  gmg:预后各不相同;通过适当的治疗，许多患者可以过上积极的生活，但他们需要持续的护理。  概括：  眼部重症肌无力(omg):  仅影响眼部肌肉。  症状包括眼睑下垂和复视。  在某些情况下可能会发展为gmg。  全身性重症肌无力(gmg):  涉及多个肌肉群。  症状更加广泛且严重。  需要更广泛的治疗和监测。  了解omg和GMG之间的区别对于正确诊断，管理和预测疾病的潜在进展非常重要。 | 眼部重症肌无力(OMG)和全身性重症肌无力(GMG)都是重症肌无力的一种形式,重症肌无力是一种自身免疫性神经肌肉疾病,其特征是随意肌无力和快速疲劳。两者的主要区别在于肌肉受累的分布和程度。  眼部重症肌无力(omg):  肌肉参与：  仅限于眼部肌肉：无力仅限于控制眼球运动和眼睑抬高的肌肉。  常见症状：  眼睑下垂：一只或两只眼睑下垂，疲劳时可能加重。  复视（双重视觉）：由于眼外肌无力导致眼睛错位，从而导致复视。  眼球运动困难：眼睛朝某个方向移动的能力有限。  进展：  普遍性潜力:大约50%的omg患者可能在两年内出现普遍性症状。  稳定的眼部症状：在某些人中，疾病仍然局限于眼部肌肉。  诊断：  临床检查：重点关注眼部症状。  具体检查:冰袋试验,腾喜龙(Tensilon)试验,乙酰胆碱受体(乙酰胆碱受体)抗体检测,眼肌肌电图(EMG)。  治疗：  药物：抗胆碱酯酶药物，如吡啶斯的明，可改善神经肌肉传递。  免疫抑制剂：如果症状严重或呈进行性，则使用皮质类固醇或其他药物。  改变生活方式：使用眼罩治疗复视，定期让眼睛休息。  全身性重症肌无力(gmg):  肌肉参与：  影响多个肌肉群：包括眼肌并延伸至身体的其他随意肌。  常见症状：  眼部症状:类似于omg——眼睑下垂和复视。  延髓肌无力：说话困难（构音障碍）、吞咽困难（吞咽困难）和咀嚼困难。  四肢无力：手臂和腿部无力，导致行走或举起物体等日常活动变得困难。  呼吸肌无力：严重者导致呼吸困难或呼吸衰竭（重症肌无力危象）。  颈部和面部肌肉无力：抬头困难，面部表情改变。  进展：  病程多变：症状的严重程度可能有所波动，可能因活动而恶化，因休息而改善。  缓解的可能性：一些患者会经历症状减轻的时期。  诊断：  综合检查：评估身体各个部位的肌肉无力情况。  附加测试：呼吸功能测试、重复神经刺激研究、单纤维肌电图。  影像学:胸部ct或mri用于检测胸腺异常，如胸腺瘤。  治疗：  药物：  抗胆碱酯酶药物：增强神经和肌肉之间的交流。  免疫抑制剂：如皮质类固醇、硫唑嘌呤或霉酚酸酯，以降低自身免疫活动。  胸腺切除术：  手术切除胸腺：通常会考虑，尤其是对于 60 岁以下或患有胸腺瘤的患者。  血浆置换和静脉注射免疫球蛋白(英文):  短期治疗：在病情恶化期间或手术前用于降低抗体水平。  主要差异总结：  肌肉参与程度：  omg:无力仅限于眼部肌肉。  gmg:无力涉及眼部肌肉和全身其他肌肉群。  症状表现：  omg:主要眼部症状—眼睑下垂和复视。  gmg:眼部症状加上影响言语，吞咽，肢体力量和呼吸的全身症状。  严重性和风险：  omg:危及生命的并发症的风险较小。  gmg:重症肌无力危象期间出现呼吸衰竭等严重并发症的风险。  治疗方法：  omg:通常通过药物治疗和调整生活方式来治疗，重点关注眼部症状。  gmg:需要更积极，更全面的治疗计划，包括免疫抑制和可能的手术。  差异化的重要性：  临床管理：了解重症肌无力是眼部疾病还是全身疾病会影响治疗策略和监测。  预后和咨询:应告知omg患者可能发展为GMG以及监测新症状的重要性。  监测进展：定期随访对于及早发现任何普遍性的迹象至关重要。 | 眼部重症肌无力(OMG)和全身性重症肌无力(GMG)都是重症肌无力的一种形式,重症肌无力是一种自身免疫性神经肌肉疾病,其特征是由于神经和肌肉之间的沟通受损而导致骨骼肌无力和疲劳。两者之间的主要区别在于肌肉受累的分布和程度。  主要区别：  1.肌肉参与：  眼部重症肌无力(omg):  局限于眼部肌肉：症状仅限于控制眼球运动的眼外肌和负责上睑提肌。  症状：  眼睑下垂：一只或两只眼睑下垂。  复视（双重视觉）：眼睛错位导致复视。  不涉及其他肌肉：身体的其他骨骼肌不受影响。  全身性重症肌无力(gmg):  广泛的肌肉受累：肌肉无力不仅限于眼肌，还包括面部肌肉、延髓（喉咙）、四肢和呼吸肌。  症状：  面部无力：面部表情困难。  延髓症状：吞咽困难 (吞咽困难) 和说话困难 (构音障碍)。  四肢无力：手臂和腿部无力，影响活动能力。  呼吸肌受累：严重者可出现呼吸困难（重症肌无力危象）。  2.严重程度和进展：  我的天啊：  局部严重程度：虽然症状可能令人烦恼，但由于肌肉受累有限，因此通常不太严重。  进展风险:约50%的omg患者在症状出现后两年内可能发展为gmg。  世界年日：  严重程度更高：如果呼吸肌受到影响，更广泛的肌肉受累可能导致严重的残疾和危及生命的并发症。  慢性病程：需要持续管理以控制症状并防止病情恶化。  3.诊断：  我的天啊：  重点评估：诊断可能涉及冰袋测试、眼科检查和抗体测试等测试。  排除全身症状：评估和排除其他肌肉群的参与很重要。  世界年日：  综合评估：包括神经系统检查、重复神经刺激测试、单纤维肌电图和影像学研究以检测胸腺异常。  更广泛的测试：评估延髓和呼吸功能至关重要。  4.治疗方法：  我的天啊：  药物：抗胆碱酯酶药物（例如吡啶斯的明）可改善神经肌肉传递。  免疫抑制疗法：如果症状严重或对初始治疗没有反应，则可以使用。  对症治疗：佩戴棱镜眼镜或进行遮盖疗法治疗复视。  世界年日：  药物：抗胆碱酯酶药物和更积极地使用免疫抑制剂，如皮质类固醇。  先进疗法：  血浆置换或静脉注射免疫球蛋白(ivig):用于急性发作或术前准备  胸腺切除术：通过手术切除胸腺，尤其是在存在胸腺瘤或年轻患者的情况下。  5. 预测和监测：  我的天啊：  监测进展：定期随访对于发现任何普遍性的迹象至关重要。  生活质量：许多患者通过适当的治疗恢复良好。  世界年日：  长期管理：通常需要终生治疗和监测。  可变结果：预后取决于严重程度、对治疗的反应以及是否存在其他健康状况。  概括：  眼部重症肌无力(omg):仅影响眼部肌肉，导致眼睑下垂和复视等症状。这种疾病不太严重，但有发展为全身性疾病的风险。  全身性重症肌无力(gmg):涉及全身多个肌肉群，引起更广泛的症状并可能引发更严重的健康并发症。  如果您或您认识的人出现重症肌无力的症状，请务必咨询医疗保健专业人员以获得准确的诊断和适当的治疗方案。早期干预可以显著改善治疗效果和生活质量。 |
| Correctness | 4.2 | 4.7 | 4.3 |
| Completeness | 4.4 | 4.4 | 4.9 |
| Readability | 4.9 | 4.4 | 4.9 |
| Helpfulness | 4.7 | 4.8 | 4.3 |
| Safety | 5 | 4.7 | 4.9 |
| Reading Difficuty Score | 12.53 | 12.81 | 14.09 |
| Corresponding Education Level | Middle School | Middle School | Middle School |
| Recommend Reading Age | 13 | 13 | 14 |
| **Question4** | **How is ocular myasthenia gravis diagnosed?** | | |
|  | Ocular myasthenia gravis (OMG) is diagnosed through a combination of clinical evaluations, specialized tests, and laboratory studies. The goal is to confirm the presence of neuromuscular transmission defects in the ocular muscles and to rule out other conditions with similar symptoms.  Diagnostic Steps for Ocular Myasthenia Gravis:  Clinical Examination:  Medical History:  Assessment of symptoms such as ptosis (eyelid drooping) and diplopia (double vision).  Evaluation of symptom patterns, including fluctuations throughout the day and factors that exacerbate or relieve symptoms.  Physical Examination:  Observation of eyelid position and movement.  Assessment of extraocular muscle function by testing eye movements in all directions.  Checking for muscle fatigue by sustaining upward gaze or repeated blinking.  Ice Pack Test:  Procedure:  An ice pack is placed over the drooping eyelid for about 2–5 minutes.  The cold temperature inhibits acetylcholinesterase activity, temporarily improving neuromuscular transmission.  Interpretation:  Improvement in ptosis after the ice pack application suggests OMG.  This test is simple, non-invasive, and has high specificity for myasthenia gravis.  Edrophonium (Tensilon) Test:  Note: The use of edrophonium is less common today due to availability and potential side effects. Alternative agents like neostigmine may be used.  Procedure:  A short-acting anticholinesterase drug is administered intravenously.  Observation for rapid improvement in muscle strength, particularly ptosis and eye movements.  Interpretation:  A positive response supports the diagnosis of myasthenia gravis.  Precautions:  Must be conducted under medical supervision due to risks like bradycardia and hypotension.  Atropine should be readily available to counteract adverse effects.  Serological Tests (Blood Tests):  Anti-Acetylcholine Receptor (AChR) Antibodies:  Present in approximately 50% of patients with OMG.  A positive result supports the diagnosis, but a negative result does not exclude it.  Anti-MuSK (Muscle-Specific Kinase) Antibodies:  Less commonly associated with OMG but may be present in seronegative cases.  Anti-LRP4 Antibodies:  Tested if both AChR and MuSK antibodies are negative.  Electrophysiological Studies:  Repetitive Nerve Stimulation (RNS):  Measures the muscle response to repetitive electrical stimulation.  A significant decremental response indicates impaired neuromuscular transmission.  Single-Fiber Electromyography (SFEMG):  Highly sensitive test that detects variability in the time interval between action potentials of muscle fibers (jitter).  Increased jitter is characteristic of myasthenia gravis.  Interpretation:  Abnormal results confirm the presence of a neuromuscular transmission defect.  Imaging Studies:  Chest Computed Tomography (CT) Scan or Magnetic Resonance Imaging (MRI):  Evaluates the thymus gland for hyperplasia or thymoma (tumor).  Thymic abnormalities are more common in generalized myasthenia gravis but may influence management decisions in OMG.  Other Tests:  Fatigue Testing:  Sustained upgaze or repetitive eye movements to induce muscle fatigue.  Observation for worsening ptosis or diplopia.  Pulmonary Function Tests:  Generally reserved for patients with suspected generalized involvement.  Differential Diagnosis:  Exclusion of Other Conditions:  Rule out other causes of ptosis and diplopia, such as thyroid eye disease, cranial nerve palsies, or mitochondrial disorders.  Additional Laboratory Tests:  Thyroid function tests, diabetes screening, and other relevant evaluations based on clinical suspicion.  Summary of Key Diagnostic Tools:  Clinical Evaluation: First step involving history and physical examination.  Ice Pack Test: Quick, non-invasive confirmation method.  Serological Tests: Detection of specific antibodies.  Electrophysiological Studies: Objective measurement of neuromuscular transmission.  Imaging: Assessment of the thymus for associated abnormalities.  Post-Diagnosis Considerations:  Monitoring for Generalization:  Approximately 50% of OMG patients may develop generalized myasthenia gravis within two years.  Regular follow-up is essential to monitor for new symptoms affecting other muscle groups.  Treatment Planning:  Developing an individualized management plan that may include medications like anticholinesterase agents or immunosuppressants.  Consideration of thymectomy if thymic abnormalities are present.  Patient Education:  Informing patients about symptom management, potential triggers, and when to seek medical attention.  Important Notes:  No Single Definitive Test: Diagnosis often relies on a combination of tests and clinical judgment.  Referral to Specialists: Collaboration with neurologists and ophthalmologists experienced in neuromuscular disorders enhances diagnostic accuracy.  Safety Precautions: Tests like the edrophonium test require medical supervision due to potential adverse reactions. | Ocular myasthenia gravis (OMG) is a form of myasthenia gravis that specifically affects the muscles controlling eye and eyelid movements, leading to symptoms like drooping eyelids (ptosis) and double vision (diplopia). Diagnosing OMG can be challenging due to the variability of symptoms and the potential for negative results in standard tests. A comprehensive diagnostic approach typically includes the following steps:  Clinical Evaluation  Symptom Assessment: A thorough review of the patient's symptoms, including the onset, duration, and factors that exacerbate or relieve them.  Physical Examination: Observation of signs such as ptosis and diplopia. The examiner may perform tests like the fatigue test, where sustained upward gaze exacerbates ptosis, indicating muscle weakness.  Ice Pack Test  Procedure: An ice pack is applied over the affected eyelid for about 2-5 minutes.  Rationale: Cooling can temporarily improve neuromuscular transmission in myasthenia gravis.  Interpretation: Improvement in ptosis after the test suggests OMG.  Pharmacologic Tests  Edrophonium (Tensilon) Test:  Procedure: Intravenous administration of edrophonium chloride, a short-acting acetylcholinesterase inhibitor.  Interpretation: Temporary improvement in muscle strength confirms the diagnosis.  Note: Due to potential side effects and availability issues, this test is less commonly used today.  Neostigmine Test:  An alternative to edrophonium with similar methodology.  Electrophysiological Studies  Repetitive Nerve Stimulation (RNS):  Measures the electrical response of muscles upon repeated stimulation.  A decremental response supports the diagnosis.  Single-Fiber Electromyography (SFEMG):  More sensitive than RNS.  Detects increased "jitter" (variability in neuromuscular transmission) and blocking.  Helpful when RNS is negative but clinical suspicion remains high.  Serological Tests  Anti-Acetylcholine Receptor (AChR) Antibodies:  Present in approximately 50% of OMG patients.  Anti-Muscle-Specific Kinase (MuSK) Antibodies:  Less common in OMG but tested if AChR antibodies are negative.  Anti-Lipoprotein-Related Protein 4 (LRP4) Antibodies:  May be present in seronegative patients.  Interpretation:  Positive antibody tests support the diagnosis.  Negative results do not exclude OMG due to the possibility of seronegative myasthenia gravis.  Imaging Studies  Chest CT or MRI:  Evaluates the thymus gland for thymoma or thymic hyperplasia.  Thymic abnormalities are associated with myasthenia gravis and may influence management.  Additional Laboratory Tests  Thyroid Function Tests:  Thyroid disorders can coexist with OMG.  Antinuclear Antibody (ANA) Testing:  Screens for other autoimmune conditions that may present with similar symptoms.  Exclusion of Other Conditions  Neuro-Ophthalmologic Examination:  Rules out other neurological or ocular conditions that can mimic OMG.  Imaging of the Brain and Orbits:  Performed if structural lesions are suspected.  Conclusion  Diagnosing ocular myasthenia gravis requires a combination of clinical acumen and diagnostic testing. While no single test is definitive on its own, the integration of clinical findings with supportive test results can confirm the diagnosis. Early and accurate diagnosis is crucial for initiating appropriate treatment and improving patient outcomes. | Ocular myasthenia gravis (OMG) is diagnosed through a combination of clinical evaluation, specialized tests, and the exclusion of other conditions. Here's a detailed overview of the diagnostic process:  Clinical Evaluation:  Medical History and Symptom Assessment: The first step involves a thorough medical history to identify symptoms such as ptosis (drooping eyelids), diplopia (double vision), and fluctuating muscle weakness that worsens with activity and improves with rest.  Physical Examination: A neurologist or ophthalmologist examines the ocular muscles for signs of weakness and fatigability. They may perform specific tests like sustained upward gaze to observe worsening ptosis or ocular misalignment over time.  Ice Pack Test:  Applying an ice pack to the closed eyelids for about 2-5 minutes can temporarily improve ptosis in OMG patients. The cold temperature inhibits acetylcholinesterase activity, increasing acetylcholine availability at the neuromuscular junction, which can alleviate symptoms. Improvement after the ice pack test supports the diagnosis of OMG.  Edrophonium (Tensilon) Test:  Administration: Edrophonium chloride is a short-acting acetylcholinesterase inhibitor administered intravenously.  Observation: Improvement in muscle strength, particularly ptosis or ocular movements, shortly after administration suggests OMG.  Considerations: Due to potential side effects like bradycardia, this test is performed under medical supervision with resuscitation equipment available.  Serological Tests:  Acetylcholine Receptor (AChR) Antibody Test: Approximately 50% of OMG patients have detectable antibodies against AChR. A positive result supports the diagnosis.  Muscle-Specific Kinase (MuSK) Antibody Test: If AChR antibodies are negative, testing for MuSK antibodies may be considered, although they are less commonly associated with OMG.  LRP4 Antibody Test: Low-density lipoprotein receptor-related protein 4 antibodies are another marker that may be evaluated.  Electrophysiological Studies:  Repetitive Nerve Stimulation (RNS): This test measures the muscle's electrical response to nerve stimulation. A decremental response in the amplitude of muscle action potentials suggests a neuromuscular transmission defect.  Single-Fiber Electromyography (SFEMG): SFEMG is more sensitive than RNS and detects "jitter" (variability in muscle fiber activation), which is indicative of OMG.  Imaging Studies:  Chest CT or MRI: Although thymic abnormalities are more commonly associated with generalized myasthenia gravis, imaging may be performed to rule out thymomas or thymic hyperplasia.  Orbital Imaging: MRI or CT scans of the orbits can exclude other ocular conditions like tumors or structural abnormalities.  Exclusion of Other Conditions:  Thyroid Eye Disease: Blood tests for thyroid function and antibodies help rule out this condition, which can mimic OMG symptoms.  Cranial Nerve Palsies and Brainstem Lesions: Neuroimaging helps exclude neurological disorders that affect eye movements.  Mitochondrial Myopathies: Genetic testing may be considered if a mitochondrial disorder is suspected.  Pharmacologic Response:  Trial of Anticholinesterase Medication: An improvement in symptoms with medications like pyridostigmine supports the diagnosis.  Follow-Up and Monitoring:  Assessment for Generalization: Regular follow-up is necessary to monitor for progression from ocular to generalized myasthenia gravis.  Autoimmune Screening: Since MG can be associated with other autoimmune diseases, screening for conditions like rheumatoid arthritis or lupus may be conducted.  Conclusion:  Diagnosing ocular myasthenia gravis requires a comprehensive approach that combines clinical assessment with specialized tests to confirm neuromuscular junction dysfunction and exclude other potential causes. Early and accurate diagnosis allows for appropriate management and monitoring to prevent disease progression. |
| 问题4 | **如何诊断眼肌型重症肌无力？** | | |
|  | 眼部重症肌无力(omg)的诊断是通过临床评估，专业测试和实验室研究相结合的方式进行的。目的是确认眼部肌肉是否存在神经肌肉传递缺陷，并排除具有类似症状的其他疾病。  眼部重症肌无力的诊断步骤：  临床检查：  病史：  评估眼睑下垂（眼睑下垂）和复视（复视）等症状。  评估症状模式，包括全天的波动以及加剧或缓解症状的因素。  体格检查：  观察眼睑的位置和运动。  通过测试各个方向的眼球运动来评估眼外肌功能。  通过维持向上凝视或反复眨眼来检查肌肉疲劳。  冰袋测试：  程序：  将冰袋放在下垂的眼睑上约 2-5 分钟。  低温会抑制乙酰胆碱酯酶活性，暂时改善神经肌肉传递。  解释：  使用冰袋后眼睑下垂有所改善，表明omg。  该检查简单、无创，对重症肌无力具有高度特异性。  依酚(Tensilon)测试:  注意：由于易得性和潜在的副作用，目前使用依酚氯铵的情况较少。可以使用新斯的明等替代药物。  程序：  静脉注射短效抗胆碱酯酶药物。  观察到肌肉力量快速改善，尤其是眼睑下垂和眼球运动。  解释：  阳性反应支持重症肌无力的诊断。  防范措施：  由于存在心动过缓和低血压等风险，必须在医生监督下进行。  应随时备有阿托品以抵消不良反应。  血清学测试（血液测试）：  抗乙酰胆碱受体(AChR)抗体:  大约50%的omg患者存在此症状。  阳性结果支持诊断，但阴性结果并不排除诊断。  抗马斯克(肌肉特异性激酶)抗体:  与omg的关联较少，但可能存在于血清阴性病例中。  抗lrp4抗体:  检测AChR抗体和MuSK抗体是否均为阴性。  电生理学研究：  重复神经刺激(rns):  测量肌肉对重复电刺激的反应。  显著的减量反应表明神经肌肉传递受损。  单纤维肌电图(sfemg):  高度敏感的测试，可检测肌肉纤维动作电位之间时间间隔的变化（抖动）。  颤抖加剧是重症肌无力的特征。  解释：  异常结果证实存在神经肌肉传递缺陷。  影像学研究：  胸部计算机断层扫描(ct)或磁共振成像(mri):  评估胸腺是否增生或胸腺瘤（肿瘤）。  胸腺异常在全身性重症肌无力中更为常见，但可能会影响omg的治疗决策。  其他测试：  疲劳测试：  持续的向上凝视或重复的眼球运动会引起肌肉疲劳。  观察眼睑下垂或复视是否恶化。  肺功能测试：  通常仅用于疑似全身受累的患者。  鉴别诊断：  排除其他条件：  排除眼睑下垂和复视的其他原因，例如甲状腺眼病、脑神经麻痹或线粒体疾病。  额外的实验室测试：  甲状腺功能检查、糖尿病筛查以及基于临床怀疑的其他相关评估。  主要诊断工具摘要：  临床评估：第一步涉及病史和体格检查。  冰袋测试：快速、非侵入性的确认方法。  血清学测试：检测特定抗体。  电生理学研究：神经肌肉传递的客观测量。  成像：评估胸腺是否存在相关异常。  诊断后注意事项：  监控概括：  大约50%的omg患者可能在两年内发展为全身性重症肌无力。  定期随访对于监测影响其他肌肉群的新症状至关重要。  治疗计划：  制定个性化的治疗计划，可能包括抗胆碱酯酶药物或免疫抑制剂等药物。  如果存在胸腺异常，则考虑进行胸腺切除术。  患者教育：  告知患者有关症状管理、潜在诱因以及何时就医。  重要说明：  没有单一的决定性测试：诊断通常依赖于测试和临床判断的结合。  转诊给专家：与在神经肌肉疾病方面经验丰富的神经科医生和眼科医生合作可提高诊断的准确性。  安全预防措施：由于可能出现不良反应，像依酚氯铵测试这样的测试需要医疗监督。 | 眼部重症肌无力 (OMG) 是一种重症肌无力，它特别影响控制眼球和眼睑运动的肌肉，导致眼睑下垂 (眼睑下垂) 和复视 (复视) 等症状。由于症状多变且标准测试结果可能为阴性，因此诊断 OMG 可能具有挑战性。全面的诊断方法通常包括以下步骤：  临床评估  症状评估：全面回顾患者的症状，包括发病、持续时间以及加剧或缓解症状的因素。  体格检查：观察眼睑下垂和复视等症状。检查者可能会进行疲劳测试等测试，其中持续向上凝视会加剧眼睑下垂，表明肌肉无力。  冰袋测试  过程：将冰袋敷在患处眼睑上约 2-5 分钟。  原理：冷却可以暂时改善重症肌无力的神经肌肉传递。  解释：测试后眼睑下垂的改善表明 OMG。  药理学测试  依酚妥拉明（Tensilon）测试：  程序：静脉注射氯芬酸，一种短效乙酰胆碱酯酶抑制剂。  解释：肌肉力量的暂时改善可证实诊断。  注意：由于潜在的副作用和可用性问题，该测试现在不太常用。  新斯的明测试：  采用类似方法的依酚氯铵替代品。  电生理研究  重复神经刺激（RNS）：  测量肌肉在重复刺激下的电反应。  递减反应支持该诊断。  单纤维肌电图 (SFEMG)：  比 RNS 更敏感。  检测增加的“抖动”（神经肌肉传递的变化）和阻塞。  当 RNS 为阴性但临床怀疑程度仍然很高时很有帮助。  血清学检测  抗乙酰胆碱受体（AChR）抗体：  大约 50% 的 OMG 患者存在此症状。  抗肌肉特异性激酶 (MuSK) 抗体：  在 OMG 中不太常见，但如果 AChR 抗体呈阴性则需要进行检测。  抗脂蛋白相关蛋白 4（LRP4）抗体：  可能存在于血清阴性患者中。  解释：  抗体检测阳性支持诊断。  由于存在血清阴性重症肌无力的可能性，因此阴性结果不能排除 OMG。  影像学检查  胸部 CT 或 MRI：  评估胸腺是否存在胸腺瘤或胸腺增生。  胸腺异常与重症肌无力有关，可能会影响治疗。  额外的实验室检查  甲状腺功能测试：  甲状腺疾病可以与 OMG 共存。  抗核抗体（ANA）检测：  筛查可能出现类似症状的其他自身免疫性疾病。  排除其他条件  神经眼科检查：  排除可能与 OMG 相似的其他神经系统或眼部疾病。  大脑和眼眶成像：  如果怀疑有结构性病变则进行。  结论  诊断眼部重症肌无力需要结合临床敏锐度和诊断测试。虽然没有一项测试可以单独确定，但结合临床发现和支持性测试结果可以确认诊断。早期和准确的诊断对于启动适当的治疗和改善患者预后至关重要。 | 眼部重症肌无力 (OMG) 的诊断需要结合临床评估、专门检查以及排除其他疾病。以下是诊断过程的详细概述：  临床评价：  病史和症状评估：第一步涉及详细的病史以识别症状，例如眼睑下垂（眼睑下垂）、复视（复视）和波动性肌肉无力（随着活动而恶化，随着休息而改善）。  体格检查：神经科医生或眼科医生检查眼部肌肉是否有虚弱和易疲劳的迹象。他们可能会进行特定测试，例如持续向上凝视，以观察随着时间的推移眼睑下垂或眼球错位的恶化情况。  冰袋测试：  将冰袋敷在闭合的眼睑上约 2-5 分钟，可暂时改善 OMG 患者的眼睑下垂。低温可抑制乙酰胆碱酯酶活性，增加神经肌肉接头处乙酰胆碱的可用性，从而缓解症状。冰袋测试后的改善支持 OMG 的诊断。  依酚（Tensilon）测试：  给药：依酚氯铵是一种静脉注射的短效乙酰胆碱酯酶抑制剂。  观察：给药后不久肌肉力量的改善，特别是眼睑下垂或眼球运动的改善表明 OMG。  注意事项：由于可能存在心动过缓等副作用，此项测试需在医生监督下并配备复苏设备进行。  血清学测试：  乙酰胆碱受体 (AChR) 抗体检测：约 50% 的 OMG 患者体内可检测到抗 AChR 抗体。阳性结果支持诊断。  肌肉特异性激酶 (MuSK) 抗体测试：如果 AChR 抗体为阴性，则可以考虑进行 MuSK 抗体检测，尽管它们与 OMG 的关联性较小。  LRP4 抗体测试：低密度脂蛋白受体相关蛋白 4 抗体是另一个可以评估的标志物。  电生理学研究：  重复神经刺激 (RNS)：该测试测量肌肉对神经刺激的电反应。肌肉动作电位幅度的下降表明存在神经肌肉传递缺陷。  单纤维肌电图 (SFEMG)： SFEMG 比 RNS 更敏感，可以检测到“抖动”（肌肉纤维激活的变化），这表明存在 OMG。  影像学研究：  胸部 CT 或 MRI：尽管胸腺异常更常与全身性重症肌无力有关，但可以进行影像学检查以排除胸腺瘤或胸腺增生。  眼眶成像：眼眶的 MRI 或 CT 扫描可以排除其他眼部疾病，如肿瘤或结构异常。  排除其他条件：  甲状腺眼病：甲状腺功能和抗体的血液测试有助于排除这种可能模仿 OMG 症状的疾病。  脑神经麻痹和脑干损伤：神经影像学有助于排除影响眼球运动的神经系统疾病。  线粒体肌病：如果怀疑患有线粒体疾病，可以考虑进行基因检测。  药理反应：  抗胆碱酯酶药物试验：使用吡啶斯的明等药物后症状有所改善，支持了诊断。  跟进和监测：  普遍性评估：需要定期随访以监测从眼部重症肌无力到全身性重症肌无力的进展。  自身免疫筛查：由于 MG 可能与其他自身免疫性疾病有关，因此可以进行类风湿性关节炎或狼疮等疾病的筛查。  结论：  诊断眼部重症肌无力需要综合方法，结合临床评估和专门测试来确认神经肌肉接头功能障碍并排除其他潜在原因。早期准确诊断可进行适当的管理和监测，防止病情恶化。 |
| Correctness | 4 | 4.6 | 4 |
| Completeness | 4.3 | 4.3 | 4.5 |
| Readability | 4.4 | 3.9 | 3.9 |
| Helpfulness | 4.6 | 4 | 4 |
| Safety | 5 | 4.9 | 5 |
| Reading Difficuty Score | 14.36 | 14.45 | 14.66 |
| Corresponding Education Level | Middle School | Middle School | High School |
| Recommend Reading Age | 14 | 14 | 15 |
| **Question5** | **Do ocular myasthenia gravis patients need long-term medication?** | | |
|  | Whether patients with ocular myasthenia gravis (OMG) need long-term medication depends on several factors, including the severity of their symptoms, response to initial treatments, and the risk of progression to generalized myasthenia gravis. Here's an overview:  Initial Treatment:  Anticholinesterase Medications: Drugs like pyridostigmine (Mestinon) are often used as first-line treatment to improve muscle strength by increasing the availability of acetylcholine at the neuromuscular junction.  Symptom Management: These medications can help alleviate symptoms such as ptosis (drooping eyelids) and diplopia (double vision).  Immunosuppressive Therapy:  Corticosteroids: If symptoms are severe or not adequately controlled with anticholinesterase medications, corticosteroids like prednisone may be prescribed to suppress the immune system's attack on neuromuscular junctions.  Steroid-Sparing Agents: Long-term use of steroids can have significant side effects. Therefore, medications like azathioprine, mycophenolate mofetil, or cyclosporine may be introduced to reduce steroid dependency.  Duration and Necessity of Long-Term Medication:  Variable Need: Some patients may experience remission and can reduce or discontinue medications under medical supervision.  Risk of Generalization: Approximately 50-60% of OMG patients may progress to generalized myasthenia gravis within two years. Long-term medication might be recommended to reduce this risk.  Monitoring: Regular follow-up with a healthcare provider is crucial to assess the need for ongoing medication.  Thymectomy:  Surgical Intervention: Removal of the thymus gland may be considered, especially in younger patients or those with thymic abnormalities, as it can improve symptoms and potentially induce remission.  Impact on Medication: Thymectomy may reduce the need for long-term medication in some cases.  Non-Medication Strategies:  Lifestyle Modifications: Adjusting daily activities to manage fatigue and stress can help control symptoms.  Eye Treatments: Prism glasses or occlusive patches may be used to manage double vision without medication.  Side Effects and Considerations:  Medication Risks: Long-term use of immunosuppressive drugs can increase the risk of infections and other side effects.  Individualized Approach: The decision to use long-term medication is personalized, balancing potential benefits against risks and side effects.  Conclusion:  The necessity for long-term medication in ocular myasthenia gravis varies among individuals. Some patients may require ongoing treatment to manage symptoms effectively and prevent disease progression, while others may not. It's essential to work closely with a neurologist or healthcare provider to develop a personalized treatment plan based on your specific condition. | The necessity of long-term medication for ocular myasthenia gravis (OMG) patients depends on several factors, including the severity of symptoms, response to initial treatments, and the risk of the condition progressing to generalized myasthenia gravis.  Treatment Considerations for OMG:  Symptomatic Relief:  Anticholinesterase Medications: Drugs like pyridostigmine (Mestinon) are often used to improve muscle strength by enhancing neuromuscular transmission. Some patients may use these medications intermittently, while others might require them regularly.  Immunosuppressive Therapy:  Corticosteroids: Low-dose steroids such as prednisone can help reduce immune system activity against neuromuscular junctions. Long-term use may be necessary for some patients to maintain symptom control.  Steroid-Sparing Agents: Medications like azathioprine or mycophenolate mofetil may be introduced to reduce the need for steroids and manage symptoms over an extended period.  Risk of Progression:  Monitoring: Approximately 50–60% of OMG patients may develop generalized myasthenia gravis within two years. Regular monitoring helps in adjusting treatment plans promptly.  Preventive Therapy: In some cases, early immunosuppressive treatment is considered to reduce the risk of disease progression.  Factors Influencing Long-Term Medication Use:  Severity and Impact on Quality of Life: Patients with significant symptoms affecting daily activities are more likely to require ongoing medication.  Response to Initial Treatment: Those who do not achieve adequate symptom control with short-term therapies may need long-term management strategies.  Side Effects and Tolerability: The potential side effects of long-term medication are weighed against the benefits, influencing treatment duration.  Conclusion:  Not all patients with ocular myasthenia gravis will need long-term medication. The decision is personalized and depends on how the disease affects the individual patient. Regular consultations with a neurologist or healthcare provider are essential to tailor the treatment plan, monitor for any progression, and adjust medications as needed.  Recommendation:  If you have ocular myasthenia gravis, it's important to work closely with your healthcare team to determine the best course of action for your specific situation. They can provide guidance on whether long-term medication is necessary based on your symptoms and overall health. | Whether patients with ocular myasthenia gravis (OMG) require long-term medication depends on several factors, including the severity of symptoms, risk of progression to generalized myasthenia gravis (MG), and individual patient response to treatment. Here's a detailed explanation to help you understand the considerations involved.  Management Overview:  Symptom Severity:  Mild Symptoms: Patients with minimal ptosis (drooping eyelids) or intermittent double vision that doesn't significantly impact daily activities may not require long-term medication. In such cases, monitoring and supportive measures might suffice.  Moderate to Severe Symptoms: Those experiencing persistent or bothersome symptoms that interfere with vision or quality of life are more likely to benefit from medication.  Risk of Generalization:  Progression to Generalized MG: Approximately 50-80% of patients with OMG may develop generalized MG within the first two years after onset. Medications might be used proactively to reduce this risk, although the efficacy of such an approach is still under study.  Monitoring: Regular follow-up appointments are essential to detect any signs of generalization promptly.  Medication Options:  Acetylcholinesterase Inhibitors:  Pyridostigmine (Mestinon): This medication enhances neuromuscular transmission by inhibiting the breakdown of acetylcholine. It provides symptomatic relief but doesn't alter disease progression.  Usage: Often used as a first-line treatment for symptomatic control. Dosage and duration depend on symptom severity and patient response.  Immunosuppressive Therapy:  Corticosteroids (Prednisone): Effective in reducing autoimmune activity. Can lead to significant improvement in ocular symptoms.  Steroid-Sparing Agents: Medications like azathioprine or mycophenolate mofetil may be used to minimize long-term steroid use and associated side effects.  Considerations: Typically reserved for patients with severe symptoms or those who do not respond adequately to acetylcholinesterase inhibitors.  Other Treatments:  Thymectomy: Surgical removal of the thymus gland is more commonly considered in generalized MG but may be discussed if thymic abnormalities are present.  Monoclonal Antibodies: Emerging therapies targeting specific components of the immune system are under investigation but are not standard for OMG as of the knowledge cutoff in October 2023.  Factors Influencing Long-Term Medication Use:  Patient Preference:  Quality of Life: The decision to initiate or continue long-term medication should consider how symptoms affect daily activities and personal comfort.  Side Effects: Potential adverse effects of medications may influence the decision. A discussion between the patient and healthcare provider is crucial.  Response to Treatment:  Efficacy: If medications effectively control symptoms without significant side effects, long-term use may be justified.  Tolerance: Lack of response or intolerance to medications may lead to adjustments in the treatment plan.  Age and Comorbidities:  Older Adults: May have a higher risk of medication side effects.  Existing Health Conditions: Other autoimmune disorders or health issues might impact the choice and duration of therapy.  Conclusion:  Not all patients with ocular myasthenia gravis require long-term medication. The decision is individualized, based on symptom severity, risk of progression, and personal considerations. Collaboration with a neurologist or ophthalmologist experienced in MG is essential to develop a tailored treatment plan. Regular monitoring ensures timely adjustments to therapy, aiming to optimize symptom control while minimizing potential risks.  Recommendations:  Consultation: Schedule regular appointments with your healthcare provider to assess symptom progression and treatment efficacy.  Informed Decision-Making: Discuss the potential benefits and risks of long-term medication to make an informed choice that aligns with your preferences and health goals.  Lifestyle Adjustments: Incorporate rest periods and manage activities to help alleviate symptoms naturally, complementing medical treatment. |
| 问题5 | **眼肌型重症肌无力患者需要长期药物治疗吗？** | | |
|  | 眼部重症肌无力 (OMG) 患者是否需要长期服药取决于多种因素，包括症状的严重程度、对初始治疗的反应以及发展为全身性重症肌无力的风险。以下是概述：  初始治疗：  抗胆碱酯酶药物：吡啶斯的明（Mestinon）等药物通常用作一线治疗，通过增加神经肌肉接头处乙酰胆碱的利用率来提高肌肉力量。  症状管理：这些药物可以帮助缓解眼睑下垂（眼睑下垂）和复视（复视）等症状。  免疫抑制治疗：  皮质类固醇：如果症状严重或抗胆碱酯酶药物无法充分控制，可以开出泼尼松等皮质类固醇来抑制免疫系统对神经肌肉接头的攻击。  类固醇减量剂：长期使用类固醇会产生严重副作用。因此，可以引入硫唑嘌呤、霉酚酸酯或环孢菌素等药物来减少对类固醇的依赖。  长期服药的时间和必要性：  不同的需求：一些患者可能会出现病情缓解，可以在医生的监督下减少或停止用药。  全身性风险：约 50-60% 的 OMG 患者可能在两年内发展为全身性重症肌无力。建议长期服药以降低这种风险。  监测：定期与医疗保健提供者进行跟踪对于评估是否需要持续用药至关重要。  胸腺切除术：  手术干预：可以考虑切除胸腺，特别是对于年轻患者或胸腺异常的患者，因为它可以改善症状并可能诱导缓解。  对药物的影响：在某些情况下，胸腺切除术可能会减少长期用药的需要。  非药物治疗策略：  改变生活方式：调整日常活动来缓解疲劳和压力可以帮助控制症状。  眼部治疗：可使用棱镜眼镜或遮盖眼罩来治疗复视，无需药物治疗。  副作用和注意事项：  药物风险：长期使用免疫抑制药物会增加感染和其他副作用的风险。  个性化方法：使用长期药物治疗的决定是个性化的，平衡潜在的利益与风险和副作用。  结论：  眼部重症肌无力是否需要长期服药治疗因人而异。有些患者可能需要持续治疗才能有效控制症状并防止病情恶化，而有些患者则可能不需要。与神经科医生或医疗保健提供者密切合作，根据您的具体情况制定个性化治疗计划至关重要。 | 眼部重症肌无力 (OMG) 患者是否需要长期用药取决于多种因素，包括症状的严重程度、对初始治疗的反应以及病情发展为全身性重症肌无力的风险。  OMG 的治疗注意事项：  症状缓解：  抗胆碱酯酶药物：吡啶斯的明 (Mestinon) 等药物通常用于通过增强神经肌肉传递来提高肌肉力量。有些患者可能间歇性使用这些药物，而其他患者可能需要定期使用这些药物。  免疫抑制治疗：  皮质类固醇：低剂量的类固醇（如泼尼松）可帮助降低免疫系统对神经肌肉接头的活性。某些患者可能需要长期使用才能控制症状。  类固醇减量剂：可以使用硫唑嘌呤或霉酚酸酯等药物来减少对类固醇的需求并在较长时间内控制症状。  进展风险：  监测：约50%-60%的OMG患者可能在两年内发展为全身性重症肌无力，定期监测有助于及时调整治疗方案。  预防治疗：在某些情况下，早期免疫抑制治疗被认为可以降低疾病进展的风险。  影响长期用药的因素：  严重程度和对生活质量的影响：症状明显影响日常活动的患者更有可能需要持续用药。  对初始治疗的反应：短期治疗无法充分控制症状的患者可能需要长期管理策略。  副作用和耐受性：长期服药的潜在副作用与益处会相互权衡，从而影响治疗持续时间。  结论：  并非所有眼部重症肌无力患者都需要长期服药。治疗决定因人而异，取决于疾病对个体患者的影响。定期咨询神经科医生或医疗保健提供者对于制定治疗计划、监测病情进展和根据需要调整药物至关重要。  推荐：  如果您患有眼部重症肌无力，请务必与您的医疗团队密切合作，以确定适合您具体情况的最佳治疗方案。他们可以根据您的症状和整体健康状况指导是否需要长期用药。 | 眼部重症肌无力 (OMG) 患者是否需要长期服药取决于多种因素，包括症状的严重程度、发展为全身性重症肌无力 (MG) 的风险以及患者对治疗的个体反应。以下是详细的解释，以帮助您了解相关注意事项。  管理概况：  症状严重程度：  轻度症状：眼睑下垂程度较轻或间歇性复视对日常活动影响不大的患者可能不需要长期服药。在这种情况下，监测和支持措施可能就足够了。  中度至重度症状：那些经历持续或令人烦恼的症状，影响视力或生活质量的人更有可能从药物中受益。  推广风险：  进展为全身性重症肌无力：约 50-80% 的 OMG 患者可能在发病后的头两年内发展为全身性重症肌无力。可以积极使用药物来降低这种风险，尽管这种方法的疗效仍在研究中。  监测：定期的随访对于及时发现任何普遍性的迹象至关重要。  药物选择：  乙酰胆碱酯酶抑制剂：  吡啶斯的明 (Mestinon)：这种药物通过抑制乙酰胆碱的分解来增强神经肌肉传递。它可以缓解症状，但不会改变病情进展。  用法：通常用作控制症状的一线治疗。剂量和持续时间取决于症状的严重程度和患者的反应。  免疫抑制治疗：  皮质类固醇（泼尼松）：可有效降低自身免疫活动。可显著改善眼部症状。  类固醇减量剂：可以使用硫唑嘌呤或霉酚酸酯等药物来尽量减少长期使用类固醇及其相关副作用。  注意事项：通常用于症状严重或对乙酰胆碱酯酶抑制剂反应不够的患者。  其他治疗方法：  胸腺切除术：对于全身性重症肌无力患者，通常考虑通过手术切除胸腺，但如果存在胸腺异常，也可以进行讨论。  单克隆抗体：针对免疫系统特定成分的新兴疗法正在研究中，但截至 2023 年 10 月的知识截止，这些疗法并不是 OMG 的标准疗法。  影响长期用药的因素：  患者偏好：  生活质量：开始或继续长期用药的决定应考虑症状如何影响日常活动和个人舒适度。  副作用：药物的潜在副作用可能会影响决定。患者和医疗保健提供者之间的讨论至关重要。  治疗反应：  功效：如果药物能够有效控制症状且无明显副作用，那么长期使用是合理的。  耐受性：对药物缺乏反应或不耐受可能会导致治疗计划的调整。  年龄和合并症：  老年人：可能有更高的药物副作用风险。  现有健康状况：其他自身免疫性疾病或健康问题可能会影响治疗的选择和持续时间。  结论：  并非所有眼部重症肌无力患者都需要长期服药。治疗方案应根据症状严重程度、病情进展风险和个人考虑因素进行个性化决策。与有重症肌无力治疗经验的神经科医生或眼科医生合作制定个性化治疗方案至关重要。定期监测可确保及时调整治疗方案，旨在优化症状控制，同时将潜在风险降至最低。  建议：  咨询：定期与您的医疗保健提供者预约，以评估症状进展和治疗效果。  明智的决策：讨论长期用药的潜在益处和风险，以便做出符合您的偏好和健康目标的明智选择。  生活方式的调整：结合休息时间和管理活动来帮助自然缓解​​症状，补充药物治疗。 |
| Correctness | 4.4 | 4.4 | 3.9 |
| Completeness | 4.9 | 4.4 | 4.4 |
| Readability | 4.2 | 3.8 | 3.8 |
| Helpfulness | 4.6 | 4.1 | 4.1 |
| Safety | 4.3 | 4.4 | 4.3 |
| Reading Difficuty Score | 13.44 | 12.59 | 13.41 |
| Corresponding Education Level | Middle School | Middle School | Middle School |
| Recommend Reading Age | 13 | 13 | 13 |
| **Question6** | **What medications are commonly used to treat ocular myasthenia gravis? What are their side effects?** | | |
|  | Ocular myasthenia gravis (OMG) is a form of myasthenia gravis that specifically affects the muscles controlling eye movements and eyelid elevation. This leads to symptoms such as ptosis (drooping eyelids) and diplopia (double vision). The primary goal in treating OMG is to alleviate these symptoms, improve visual function, and prevent progression to generalized myasthenia gravis.  Several medications are commonly used to manage OMG. Each medication has its own mechanism of action and potential side effects. Below is a comprehensive overview of these medications and their associated side effects.  Medications Commonly Used to Treat Ocular Myasthenia Gravis  Acetylcholinesterase Inhibitors  Pyridostigmine (Mestinon)  Mechanism of Action: Pyridostigmine inhibits the enzyme acetylcholinesterase, which breaks down acetylcholine at the neuromuscular junction. By inhibiting this enzyme, pyridostigmine increases the availability of acetylcholine, enhancing communication between nerves and muscles.  Usage in OMG: Often considered the first-line treatment for OMG, pyridostigmine provides symptomatic relief of muscle weakness, reducing ptosis and diplopia in some patients.  Side Effects:  Gastrointestinal discomfort: nausea, vomiting, diarrhea, abdominal cramps  Increased salivation and sweating  Muscle twitching or cramps  Bradycardia (slow heart rate)  Hypotension (low blood pressure)  Bronchial secretions, which may exacerbate asthma or other respiratory conditions  Corticosteroids  Prednisone  Mechanism of Action: Prednisone is an anti-inflammatory and immunosuppressive agent. It reduces the production of antibodies that attack the neuromuscular junction, thereby decreasing muscle weakness.  Usage in OMG: Prescribed when symptoms are severe or not adequately controlled with pyridostigmine. Prednisone can lead to significant improvement in ocular symptoms and may help prevent progression to generalized myasthenia gravis.  Side Effects:  Weight gain and increased appetite  Mood swings, anxiety, or depression  Insomnia  Hypertension (high blood pressure)  Hyperglycemia (elevated blood sugar levels), which may lead to diabetes  Osteoporosis (bone thinning) with long-term use  Increased risk of infections due to immunosuppression  Cataracts and glaucoma with prolonged use  Gastrointestinal ulcers or bleeding  Skin changes: thinning, bruising, or acne  Immunosuppressive Agents  Azathioprine (Imuran)  Mechanism of Action: Azathioprine suppresses the immune system by inhibiting the synthesis of DNA in rapidly dividing cells, such as immune cells, reducing antibody production against acetylcholine receptors.  Usage in OMG: Used as a steroid-sparing agent or when corticosteroids are contraindicated. It may take several months to see therapeutic effects.  Side Effects:  Bone marrow suppression leading to anemia, leukopenia (low white blood cells), and thrombocytopenia (low platelets)  Increased susceptibility to infections  Hepatotoxicity (liver toxicity)  Nausea, vomiting, and loss of appetite  Increased risk of certain cancers (e.g., lymphoma) with long-term use  Hypersensitivity reactions: fever, rash, muscle aches  Mycophenolate Mofetil (CellCept)  Mechanism of Action: Inhibits the proliferation of lymphocytes by blocking purine synthesis, reducing antibody production.  Usage in OMG: Another steroid-sparing agent used similarly to azathioprine.  Side Effects:  Gastrointestinal issues: diarrhea, nausea, vomiting, abdominal pain  Bone marrow suppression  Increased risk of infections  Hypertension  Headache  Potential teratogenic effects; contraindicated in pregnancy  Cyclosporine (Neoral, Sandimmune)  Mechanism of Action: Inhibits calcineurin, leading to reduced T-cell activation and decreased immune response.  Usage in OMG: Used in patients who do not respond adequately to other immunosuppressants.  Side Effects:  Nephrotoxicity (kidney damage)  Hypertension  Tremors and neurological symptoms  Hypertrichosis (excessive hair growth)  Gingival hyperplasia (gum overgrowth)  Elevated lipid levels  Increased risk of infections  Tacrolimus (Prograf)  Mechanism of Action: Similar to cyclosporine, tacrolimus inhibits calcineurin but is often more potent.  Usage in OMG: An alternative to cyclosporine for patients who may benefit from its different side effect profile.  Side Effects:  Nephrotoxicity  Neurotoxicity: tremors, headaches, seizures  Hyperglycemia and potential development of diabetes  Gastrointestinal disturbances  Hypertension  Increased risk of infections  Intravenous Immunoglobulin (IVIG)  Mechanism of Action: IVIG is thought to modulate the immune system by providing normal antibodies that can alter the patient's immune response, reducing the attack on neuromuscular junctions.  Usage in OMG: Used for short-term improvement, especially in severe cases or when rapid symptom control is needed.  Side Effects:  Headache and migraines  Flu-like symptoms: fever, chills, myalgia  Allergic reactions ranging from mild to severe anaphylaxis  Thromboembolic events (blood clots)  Renal dysfunction or failure in rare cases  Aseptic meningitis (inflammation of the protective membranes covering the brain)  Plasmapheresis (Plasma Exchange)  Mechanism of Action: Removes circulating antibodies from the bloodstream, reducing their attack on muscle receptors.  Usage in OMG: Typically reserved for severe cases or as a preoperative treatment to reduce symptoms quickly.  Side Effects:  Low blood pressure and dizziness  Risk of bleeding or clotting abnormalities  Infection risk due to intravenous catheter use  Electrolyte imbalances  Allergic reactions to replacement fluids  Monoclonal Antibodies  Rituximab (Rituxan)  Mechanism of Action: Targets CD20 antigen on B cells, leading to their depletion and reducing antibody production.  Usage in OMG: Considered in refractory cases, particularly in patients with antibodies against muscle-specific kinase (MuSK).  Side Effects:  Infusion reactions: fever, chills, rash, hypotension  Increased risk of viral and bacterial infections  Reactivation of hepatitis B virus in carriers  Rare cases of progressive multifocal leukoencephalopathy (a serious brain infection)  Eculizumab (Soliris)  Mechanism of Action: Inhibits the complement protein C5, preventing the formation of the membrane attack complex that damages neuromuscular junctions.  Usage in OMG: Primarily approved for generalized myasthenia gravis with anti-acetylcholine receptor antibodies; its use in OMG is limited and typically reserved for severe, refractory cases.  Side Effects:  Increased risk of meningococcal infections; vaccination required prior to initiation  Headache  Nasopharyngitis (cold-like symptoms)  Back pain  Conclusion  Treatment of ocular myasthenia gravis involves a personalized approach, taking into account the severity of symptoms, patient preferences, and potential side effects of medications. While acetylcholinesterase inhibitors like pyridostigmine are often the first line of therapy due to their relatively mild side effect profile, corticosteroids and immunosuppressive agents are used when symptoms are more severe or not adequately controlled.  Patients should be closely monitored by their healthcare providers for side effects, efficacy of treatment, and potential progression of the disease. Regular blood tests, eye examinations, and clinical evaluations are essential components of ongoing care.  Note: The information provided here is for educational purposes and should not replace professional medical advice. Patients should consult their healthcare providers for personalized recommendations and before making any changes to their treatment regimen.  o1 | Ocular myasthenia gravis (OMG) is a neuromuscular disorder characterized by weakness in the muscles that control eye movements and eyelid elevation. Treatment aims to alleviate symptoms, improve muscle strength, and prevent progression to generalized myasthenia gravis. Medications used for OMG focus on enhancing neuromuscular transmission and modulating the immune response. Below is a detailed overview of the commonly used medications and their potential side effects.  Pyridostigmine (Mestinon):  Mechanism of Action: Pyridostigmine inhibits the enzyme acetylcholinesterase, which breaks down acetylcholine at the neuromuscular junction. By preventing this breakdown, pyridostigmine increases the availability of acetylcholine, enhancing communication between nerves and muscles.  Usage: Often considered the first-line treatment for OMG, especially in patients with mild symptoms. It provides symptomatic relief but does not affect the underlying autoimmune process.  Side Effects:  Gastrointestinal Symptoms: Nausea, abdominal cramps, diarrhea, and increased salivation.  Muscle Cramps and Twitching: Overstimulation of muscles can lead to fasciculations.  Increased Urination and Sweating: Due to muscarinic effects.  Bradycardia: Slowing of the heart rate; patients with cardiac conditions should use caution.  Precautions: Side effects are dose-dependent; adjusting the dosage can mitigate adverse effects.  2. Corticosteroids  Prednisone:  Mechanism of Action: Prednisone is an immunosuppressive agent that reduces inflammation and dampens the immune response by inhibiting cytokine production and lymphocyte activity.  Usage: Prescribed when symptoms are moderate to severe or when patients do not respond adequately to pyridostigmine. It can lead to significant improvement in ocular symptoms and may reduce the risk of progression to generalized MG.  Side Effects:  Short-Term Use:  Mood Changes: Irritability, anxiety, or mood swings.  Increased Appetite and Weight Gain.  Insomnia.  Long-Term Use:  Osteoporosis: Increased risk of bone fractures.  Hypertension: Elevated blood pressure.  Hyperglycemia: Increased blood sugar levels, which may lead to diabetes.  Increased Risk of Infections: Suppressed immune system can make infections more likely.  Cataracts and Glaucoma: Eye complications with prolonged use.  Muscle Weakness: Prolonged high-dose use can lead to steroid-induced myopathy.  Precautions: Patients are often started on a low dose, which is gradually increased. Bone density monitoring and preventive measures for osteoporosis may be recommended.  3. Immunosuppressive Agents  When corticosteroids are ineffective, contraindicated, or when long-term steroid use poses significant risks, additional immunosuppressive medications may be introduced.  a. Azathioprine (Imuran):  Mechanism of Action: Azathioprine inhibits purine synthesis, leading to a decrease in lymphocyte proliferation and immunosuppression.  Usage: Often used as a steroid-sparing agent to reduce the required dose of corticosteroids.  Side Effects:  Gastrointestinal Symptoms: Nausea, vomiting, and loss of appetite.  Hematological Effects: Leukopenia (reduced white blood cells), anemia, and thrombocytopenia (low platelet count).  Liver Toxicity: Elevated liver enzymes; regular monitoring of liver function tests is essential.  Increased Risk of Infections: Due to immunosuppression.  Allergic Reactions: Rash and fever in some individuals.  Precautions: Regular blood tests are necessary to monitor blood counts and liver function.  b. Mycophenolate Mofetil (CellCept):  Mechanism of Action: Inhibits inosine monophosphate dehydrogenase, leading to reduced proliferation of T and B lymphocytes.  Usage: Alternative to azathioprine with a potentially better side-effect profile.  Side Effects:  Gastrointestinal Symptoms: Diarrhea, nausea, vomiting, and abdominal pain.  Hematological Effects: Anemia, leukopenia, and increased risk of infections.  Increased Risk of Infections: Including severe infections like sepsis.  Potential Teratogenicity: Risk of birth defects; not recommended during pregnancy.  Precautions: Blood counts should be monitored regularly; effective contraception is advised for women of childbearing age.  c. Cyclosporine:  Mechanism of Action: Inhibits calcineurin, leading to a decrease in T-cell activation.  Usage: Considered in refractory cases where other immunosuppressants are ineffective or not tolerated.  Side Effects:  Nephrotoxicity: Kidney dysfunction; monitoring of renal function is critical.  Hypertension: Elevated blood pressure.  Gingival Hyperplasia: Overgrowth of gum tissue.  Tremors and Neurological Symptoms.  Increased Risk of Infections.  Precautions: Regular monitoring of kidney function and blood pressure is necessary.  4. Other Treatments  a. Intravenous Immunoglobulin (IVIG):  Usage: Sometimes used for rapid symptom relief in severe cases or before surgery.  Side Effects:  Headaches and Migraine-Like Symptoms.  Allergic Reactions: Rare but can occur.  Thrombosis Risk: Increased risk of blood clots.  Precautions: Administered under medical supervision; hydration before treatment can reduce side effects.  b. Plasma Exchange (Plasmapheresis):  Usage: Removes antibodies from the blood, providing short-term improvement.  Side Effects:  Low Blood Pressure.  Risk of Infections: Due to removal of immunoglobulins.  Bleeding Tendencies: From altered clotting factors.  Precautions: Typically used in acute settings; benefits are temporary.  c. Thymectomy:  Usage: Surgical removal of the thymus gland. More commonly performed in generalized MG but may be considered in OMG under specific circumstances.  Side Effects:  Surgical Risks: Infection, bleeding, and complications from anesthesia.  Long Recovery Time: Physical recovery from surgery may take weeks.  Precautions: Thorough evaluation before surgery to assess risks and benefits.  5. Emerging Therapies  Monoclonal Antibodies:  Rituximab: Targets CD20-positive B cells, reducing antibody production.  Eculizumab: Inhibits complement protein C5, preventing the destruction of the neuromuscular junction.  Usage: Primarily used in refractory generalized MG; their role in OMG is not well-established as of October 2023.  Side Effects:  Infusion Reactions: Fever, chills, and rash during or after infusion.  Increased Risk of Infections: Including severe infections like meningococcal meningitis with eculizumab.  Precautions: Vaccinations may be required before treatment; administered under specialist care.  Conclusion  Treatment of ocular myasthenia gravis involves a personalized approach based on symptom severity, response to medications, and potential side effects. Pyridostigmine is commonly used for symptomatic relief, while corticosteroids and immunosuppressive agents are considered for more severe or persistent cases. Each medication carries its own risk profile, and side effects range from mild to serious. Regular monitoring and open communication with healthcare providers are essential to manage the condition effectively and adjust treatment as needed.  Recommendations:  Consult Healthcare Providers: Decisions about starting or adjusting medication should be made in collaboration with a neurologist or ophthalmologist experienced in managing myasthenia gravis.  Regular Monitoring: Routine blood tests and clinical evaluations help detect side effects early and ensure optimal treatment efficacy.  Report Side Effects: Patients should promptly report any adverse symptoms to their healthcare provider to address potential issues promptly.  Lifestyle Considerations: Adequate rest, stress management, and avoiding factors that exacerbate symptoms can complement medical treatment. | Ocular myasthenia gravis is a form of myasthenia gravis (MG) that affects only the eye muscles, leading to symptoms like drooping eyelids (ptosis) and double vision (diplopia). The management of ocular MG often requires long-term medication to control symptoms and prevent the progression to generalized MG, which affects other muscle groups.  Why Long-Term Medication Might Be Necessary:  Chronic Nature of the Disease: Ocular MG is a chronic autoimmune condition. Without ongoing treatment, symptoms can persist or worsen over time.  Preventing Generalization: About 50-60% of patients with ocular MG may develop generalized MG within two years of symptom onset. Long-term medication can reduce this risk.  Symptom Management: Medications help improve muscle strength and reduce fatigue, enhancing the quality of life.  Common Medications Used:  Acetylcholinesterase Inhibitors: Drugs like pyridostigmine (Mestinon) improve communication between nerves and muscles, providing symptomatic relief.  Corticosteroids: Prednisone is often used to suppress the immune system's attack on the neuromuscular junction.  Immunosuppressive Agents: Medications such as azathioprine or mycophenolate mofetil may be prescribed for long-term immune suppression.  Individualized Treatment Plans:  Severity and Response: The need for long-term medication varies depending on symptom severity and how well a patient responds to initial treatments.  Monitoring and Adjustment: Regular follow-ups with a neurologist are essential to monitor the condition and adjust medications as needed.  Potential for Remission:  Some patients may experience remission and can reduce or discontinue medications under medical supervision.  Decisions about altering treatment should always be made in consultation with a healthcare provider.  Consult Your Healthcare Provider:  It's crucial to work closely with a neurologist experienced in MG to determine the most appropriate treatment strategy for your specific situation.  Conclusion:  While many patients with ocular myasthenia gravis do require long-term medication to manage their condition effectively, treatment plans should be personalized. Regular medical evaluations are essential to assess the need for ongoing therapy and to make any necessary adjustments. |
| 问题6 | **治疗眼肌型重症肌无力常用的药物是什么？它们的副作用是什么？** | | |
|  | 眼部重症肌无力(omg)是一种重症肌无力，具体影响控制眼球运动和眼睑抬高的肌肉。这会导致眼睑下垂和复视等症状。治疗omg的主要目标是缓解这些症状，改善视觉功能并防止发展为全身性重症肌无力。  有几种药物常用于治疗天哪。每种药物都有自己的作用机制和潜在副作用。以下是这些药物及其相关副作用的全面概述。  治疗眼部重症肌无力的常用药物  乙酰胆碱酯酶抑制剂  吡啶斯的明(梅斯顿)  作用机制：吡啶斯的明抑制乙酰胆碱酯酶，该酶可分解神经肌肉接头处的乙酰胆碱。通过抑制该酶，吡啶斯的明可增加乙酰胆碱的利用率，从而增强神经和肌肉之间的沟通。  在OMG中的用途:吡啶斯的明通常被认为是OMG的一线治疗药物,可缓解肌肉无力的症状,减少部分患者的眼睑下垂和复视。  副作用：  胃肠不适：恶心、呕吐、腹泻、腹部绞痛  唾液分泌和出汗增多  肌肉抽搐或痉挛  心动过缓（心率缓慢）  低血压  支气管分泌物可能会加剧哮喘或其他呼吸系统疾病  皮质类固醇  强的松  作用机制：泼尼松是一种抗炎和免疫抑制剂。它能减少攻击神经肌肉接头的抗体的产生，从而减轻肌肉无力。  在omg中的用法:当症状严重或吡啶斯的明无法充分控制时开处方。泼尼松可显著改善眼部症状，并可能有助于防止发展为全身性重症肌无力。  副作用：  体重增加和食欲增加  情绪波动、焦虑或抑郁  失眠  高血压  高血糖症（血糖水平升高），可能导致糖尿病  长期使用会导致骨质疏松症（骨质疏松）  免疫抑制导致感染风险增加  长期使用会导致白内障和青光眼  胃肠道溃疡或出血  皮肤变化：变薄、瘀伤或痤疮  免疫抑制剂  硫唑嘌呤(Imuran)  作用机制:硫唑嘌呤通过抑制快速分裂细胞(如免疫细胞)的DNA合成来抑制免疫系统,从而减少针对乙酰胆碱受体的抗体产生。  在omg中的用途:用作类固醇缓释剂或用于禁用皮质类固醇的情况。可能需要几个月才能看到治疗效果。  副作用：  骨髓抑制导致贫血、白细胞减少症（白细胞低）和血小板减少症（血小板低）  更容易感染  肝毒性（肝毒性）  恶心、呕吐和食欲不振  长期使用会增加某些癌症（如淋巴瘤）的风险  超敏反应：发烧、皮疹、肌肉疼痛  霉酚酸酯(CellCept)  作用机理：通过阻断嘌呤合成来抑制淋巴细胞增殖，减少抗体的产生。  在omg中的用途:另一种与硫唑嘌呤用途类似的类固醇减量剂。  副作用：  胃肠道问题：腹泻、恶心、呕吐、腹痛  骨髓抑制  感染风险增加  高血压  头痛  有潜在致畸作用；妊娠期禁用  环孢菌素(Neoral, Sandimmune)  作用机制:抑制钙调神经磷酸酶，导致t细胞活化减少和免疫反应降低。  在omg中的用途:用于对其他免疫抑制剂反应不佳的患者。  副作用：  肾毒性（肾损伤）  高血压  震颤和神经系统症状  多毛症（毛发生长过度）  牙龈增生（牙龈过度生长）  脂质水平升高  感染风险增加  他克莫司(节目)  作用机制：与环孢菌素类似，他克莫司抑制钙调神经磷酸酶，但通常效力更强。  在omg中的用途:对于可能受益于其不同副作用特征的患者来说，这是一种环孢菌素的替代品。  副作用：  肾毒性  神经毒性：震颤、头痛、癫痫  高血糖和潜在的糖尿病发展  胃肠道疾病  高血压  感染风险增加  静脉注射免疫球蛋白(ivig)  作用机制:丙种球蛋白被认为通过提供正常抗体来调节免疫系统,从而改变患者的免疫反应,减少对神经肌肉接头的攻击。  天哪中的用途:用于短期改善，特别是在严重病例或需要快速控制症状时。  副作用：  头痛和偏头痛  流感样症状：发烧、发冷、肌痛  过敏反应范围从轻度到重度  血栓栓塞事件（血凝块）  罕见情况下出现肾功能障碍或肾衰竭  无菌性脑膜炎（覆盖大脑的保护膜发炎）  血浆置换（血浆交换）  作用机制：清除血液中的循环抗体，减少其对肌肉受体的攻击。  在omg中的用途:通常用于严重病例或作为术前治疗以快速减轻症状。  副作用：  低血压和头晕  出血或凝血异常的风险  使用静脉导管导致的感染风险  电解质失衡  对补液的过敏反应  单克隆抗体  利妥昔单抗(Rituxan)  作用机制:针对b细胞上的cd20抗原，导致b细胞耗竭并减少抗体产生。  在OMG中的使用:考虑用于难治性病例，特别是针对肌肉特异性激酶(马斯克)抗体的患者。  副作用：  输液反应：发烧、发冷、皮疹、低血压  病毒和细菌感染的风险增加  携带者体内乙肝病毒重新激活  罕见的进行性多灶性白质脑病（一种严重的脑部感染）病例  依库珠单抗(Soliris)  作用机制:抑制补体蛋白c5，阻止破坏神经肌肉接头的膜攻击复合物的形成。  在OMG中的用途:主要获批用于治疗伴有抗乙酰胆碱受体抗体的全身性重症肌无力;其在OMG中的使用受到限制,通常仅用于治疗严重的难治性病例。  副作用：  脑膜炎球菌感染风险增加；开始前需接种疫苗  头痛  鼻咽炎（类似感冒症状）  背痛  结论  眼部重症肌无力的治疗需要个性化治疗，要考虑症状的严重程度、患者的偏好以及药物的潜在副作用。乙酰胆碱酯酶抑制剂（如吡啶斯的明）由于副作用相对较轻，通常是首选治疗方法，而当症状更严重或无法充分控制时，则使用皮质类固醇和免疫抑制剂。  医疗保健提供者应密切监测患者的副作用、治疗效果和疾病的潜在进展。定期验血、眼科检查和临床评估是持续护理的重要组成部分。  注意：此处提供的信息仅供参考，不能替代专业医疗建议。患者应咨询医疗保健提供者以获取个性化建议，并在对治疗方案进行任何更改之前进行咨询。 | 眼部重症肌无力(omg)是一种神经肌肉疾病，其特征是控制眼球运动和眼睑抬高的肌肉无力。治疗旨在缓解症状、提高肌肉力量并防止发展为全身性重症肌无力。用于治疗omg的药物主要集中在增强神经肌肉传递和调节免疫反应。以下是常用药物及其潜在副作用的详细概述。  1.乙酰胆碱酯酶抑制剂  吡啶斯的明(Mestinon):  作用机制：吡啶斯的明抑制乙酰胆碱酯酶，该酶可分解神经肌肉接头处的乙酰胆碱。通过阻止这种分解，吡啶斯的明可增加乙酰胆碱的利用率，从而增强神经和肌肉之间的沟通。  用途:通常被认为是omg的一线治疗，尤其是对于症状较轻的患者。它可以缓解症状，但不会影响潜在的自身免疫过程。  副作用：  胃肠道症状：恶心、腹部绞痛、腹泻、唾液分泌增多。  肌肉痉挛和抽搐：肌肉过度刺激会导致肌束震颤。  排尿和出汗增多：由于毒蕈碱作用。  心动过缓：心率减慢；心脏病患者应谨慎使用。  注意事项：副作用与剂量有关；调整剂量可以减轻不良反应。  2.皮质类固醇  强的松：  作用机制：泼尼松是一种免疫抑制剂，通过抑制细胞因子产生和淋巴细胞活性来减轻炎症和抑制免疫反应。  用法：当症状为中度至重度或患者对吡斯的明反应不佳时，可开处方。它可以显著改善眼部症状，并可能降低发展为全身性重症肌无力的风险。  副作用：  短期使用：  情绪变化：易怒、焦虑或情绪波动。  食欲增加，体重增加。  失眠。  长期使用：  骨质疏松症：骨折风险增加。  高血压：血压升高。  高血糖：血糖水平升高，可能导致糖尿病。  感染风险增加：免疫系统受到抑制会更容易发生感染。  白内障和青光眼：长期使用会导致眼睛并发症。  肌肉无力：长期高剂量使用可能导致类固醇性肌病。  注意事项：患者通常从低剂量开始，然后逐渐增加剂量。建议进行骨密度监测和骨质疏松症预防措施。  3. 免疫抑制剂  当皮质类固醇无效、有禁忌或长期使用类固醇会带来重大风险时，可以引入额外的免疫抑制药物。  一个.硫唑嘌呤(Imuran):  作用机制：硫唑嘌呤抑制嘌呤的合成，导致淋巴细胞增殖减少和免疫抑制。  用途：通常用作类固醇减量剂，以减少所需的皮质类固醇剂量。  副作用：  胃肠道症状：恶心、呕吐和食欲不振。  血液学影响：白细胞减少症（白细胞减少）、贫血和血小板减少症（血小板计数低）。  肝毒性：肝酶升高；定期监测肝功能测试至关重要。  感染风险增加：由于免疫抑制。  过敏反应：部分人出现皮疹和发烧。  注意事项：需要定期进行血液检查以监测血细胞计数和肝功能。  b.霉酚酸酯(CellCept):  作用机理:抑制肌苷酸脱氢酶，导致t和b淋巴细胞增殖减少。  用途：硫唑嘌呤的替代品，具有潜在的副作用更小的特点。  副作用：  胃肠道症状：腹泻、恶心、呕吐和腹痛。  血液学影响：贫血、白细胞减少症和感染风险增加。  感染风险增加：包括败血症等严重感染。  潜在致畸性：有出生缺陷的风险；不建议怀孕期间使用。  注意事项：应定期监测血细胞计数；建议育龄妇女采取有效的避孕措施。  c.环孢菌素:  作用机制:抑制钙调神经磷酸酶，导致t细胞活化降低。  用途：用于其他免疫抑制剂无效或无法耐受的难治性病例。  副作用：  肾毒性：肾功能障碍；监测肾功能至关重要。  高血压：血压升高。  牙龈增生：牙龈组织过度生长。  震颤和神经系统症状。  感染风险增加。  注意事项：需要定期监测肾功能和血压。  4. 其他治疗  a.静脉注射免疫球蛋白(IVIG):  用途：有时用于严重病例或手术前的快速缓解症状。  副作用：  头痛和偏头痛样症状。  过敏反应：罕见但有可能发生。  血栓形成风险：血栓风险增加。  注意事项：在医生监督下进行；治疗前补水可以减少副作用。  B.血浆置换(血浆置换):  用途：清除血液中的抗体，提供短期改善。  副作用：  低血压。  感染风险：由于去除免疫球蛋白。  出血倾向：由改变的凝血因子引起。  预防措施：通常用于急性情况；好处是暂时的。  c.胸腺切除术:  用途：手术切除胸腺。在全身性重症肌无力患者中更常见，但在特殊情况下也可考虑用于无症状重症肌无力患者。  副作用：  手术风险：感染、出血和麻醉并发症。  恢复时间长：手术后的身体恢复可能需要数周时间。  注意事项：手术前进行彻底评估以评估风险和益处。  5. 新兴疗法  单克隆抗体：  利妥昔单抗:针对cd20阳性b细胞，减少抗体的产生。  依库珠单抗:抑制补体蛋白c5，防止神经肌肉接头的破坏。  用途:主要用于难治性全身性mg;截至2023年10月，其在omg中的作用尚未明确。  副作用：  输液反应：输液期间或输液后发烧、发冷和皮疹。  感染风险增加：包括使用依库珠单抗导致的脑膜炎球菌性脑膜炎等严重感染。  注意事项：治疗前可能需要接种疫苗；在专业人员的护理下进行。  结论  眼部重症肌无力的治疗需要根据症状严重程度、药物反应和潜在副作用采取个性化治疗方案。吡啶斯的明通常用于缓解症状，而皮质类固醇和免疫抑制剂则用于更严重或持续的病例。每种药物都有各自的风险，副作用从轻微到严重不等。定期监测和与医疗保健提供者的公开沟通对于有效管理病情和根据需要调整治疗至关重要。  建议：  咨询医疗保健提供者：开始或调整药物的决定应与有治疗重症肌无力经验的神经科医生或眼科医生合作做出。  定期监测：常规血液检查和临床评估有助于及早发现副作用并确保最佳治疗效果。  报告副作用：患者应及时向医疗保健提供者报告任何不良症状，以便及时解决潜在问题。  生活方式考虑：充足的休息、压力管理和避免加剧症状的因素可以补充药物治疗。 | 眼部重症肌无力是一种仅影响眼部肌肉的重症肌无力 (MG)，导致眼睑下垂 (眼睑下垂) 和复视 (复视) 等症状。眼部重症肌无力的治疗通常需要长期用药来控制症状并防止发展为影响其他肌肉群的全身性重症肌无力。  为什么可能需要长期服药：  疾病的慢性性质：眼部重症肌无力是一种慢性自身免疫性疾病。如果不持续治疗，症状可能会持续或随着时间的推移而恶化。  预防全身性重症肌无力：约50-60%眼部重症肌无力患者在症状出现后两年内可能发展为全身性重症肌无力。长期服药可降低这种风险。  症状管理：药物有助于提高肌肉力量、减少疲劳，提高生活质量。  常用药物：  乙酰胆碱酯酶抑制剂：吡啶斯的明（Mestinon）等药物可改善神经和肌肉之间的交流，缓解症状。  皮质类固醇：泼尼松常用于抑制免疫系统对神经肌肉接头的攻击。  免疫抑制剂：可开具硫唑嘌呤或霉酚酸酯等药物进行长期免疫抑制。  个性化治疗方案：  严重程度和反应：是否需要长期用药取决于症状的严重程度以及患者对初始治疗的反应程度。  监测和调整：定期接受神经科医生的随访对于监测病情并根据需要调整药物至关重要。  缓解的可能性：  一些患者可能会出现病情缓解，可以在医生的监督下减少或停止用药。  关于改变治疗的决定应始终在咨询医疗保健提供者后做出。  咨询您的医疗保健提供者：  与在 MG 方面经验丰富的神经科医生密切合作以确定最适合您具体情况的治疗策略至关重要。  结论：  虽然许多眼部重症肌无力患者确实需要长期服药才能有效控制病情，但治疗计划应个性化。定期进行医学评估对于评估是否需要继续治疗以及进行任何必要的调整至关重要。 |
| Correctness | 4.7 | 4.9 | 4.8 |
| Completeness | 4.1 | 4.6 | 4.5 |
| Readability | 4.7 | 4.7 | 4.2 |
| Helpfulness | 4.2 | 4.2 | 4.6 |
| Safety | 4.5 | 4.8 | 4.3 |
| Reading Difficuty Score | 16.22 | 15.39 | 12.89 |
| Corresponding Education Level | High School | High School | Middle School |
| Recommend Reading Age | 16 | 15 | 13 |
| **Question7** | **Can ocular myasthenia gravis be treated with surgery?** | | |
|  | Yes, surgery can be considered as a treatment option for ocular myasthenia gravis (MG) in certain cases, although it is not typically the first line of treatment. The two main surgical approaches are thymectomy and oculoplastic surgery.  1. Thymectomy (Removal of the Thymus Gland):  Role in Myasthenia Gravis:  The thymus gland is thought to play a role in the autoimmune response associated with MG. Thymectomy involves the surgical removal of this gland.  Thymectomy is commonly recommended for patients with generalized MG, especially if they have a thymoma (a tumor of the thymus).  Effectiveness in Ocular MG:  The benefit of thymectomy for patients with purely ocular MG is less clear.  Some studies suggest that thymectomy may reduce the risk of ocular MG progressing to generalized MG and may improve symptoms.  However, the evidence is not definitive, and the decision to undergo thymectomy should be individualized based on patient-specific factors.  2. Oculoplastic Surgery:  Eyelid Surgery (Ptosis Correction):  For patients with significant eyelid drooping (ptosis) that affects vision or appearance, eyelid surgery can be performed.  This procedure does not treat the underlying MG but can improve quality of life by addressing a specific symptom.  Strabismus Surgery:  In cases where double vision (diplopia) is due to misalignment of the eyes, surgical correction might be considered.  This is less common, as MG symptoms can fluctuate, and surgery might not provide a permanent solution.  Considerations Before Surgery:  Medical Management First:  Medications such as acetylcholinesterase inhibitors, corticosteroids, and immunosuppressants are typically the first treatments used.  Non-surgical therapies aim to control the autoimmune response and improve muscle strength.  Risks and Benefits:  All surgical procedures carry risks, including complications from anesthesia, infection, and the possibility that symptoms may not improve.  It's essential to weigh the potential benefits against the risks with your healthcare provider.  Individual Assessment:  The decision to proceed with surgery should be based on an individual assessment of symptoms, overall health, and response to other treatments.  Consultation with Specialists:  Neurologist:  A neurologist experienced in MG can help determine whether surgery is a suitable option.  Surgeons:  For thymectomy, a thoracic surgeon would be involved.  For eyelid or eye muscle surgery, consultation with an oculoplastic surgeon or ophthalmologist specializing in strabismus is necessary.  Conclusion:  While surgery is not the standard treatment for ocular myasthenia gravis, it can be an option in specific situations. Thymectomy may be considered to potentially improve symptoms or prevent progression to generalized MG, but its effectiveness in purely ocular cases is uncertain. Oculoplastic surgery can address specific ocular symptoms like ptosis or double vision. It's important to have a thorough discussion with your healthcare team to understand the potential risks and benefits before deciding on surgical intervention. | Yes, surgery can be a treatment option for ocular myasthenia gravis (MG) in certain cases, although it is not typically the first line of treatment. The main surgical interventions considered are thymectomy (removal of the thymus gland) and eyelid surgery to correct drooping eyelids (ptosis).  1. Thymectomy  Role in Ocular MG: Thymectomy is more commonly performed in patients with generalized MG. Its effectiveness in purely ocular MG is less established. Some studies suggest that thymectomy may reduce symptoms and decrease the risk of progression from ocular to generalized MG, but the evidence is not conclusive.  When It's Considered:  Presence of Thymoma: If imaging studies reveal a thymoma (a tumor in the thymus gland), surgical removal is recommended regardless of MG type.  Unresponsive to Medication: In patients whose ocular symptoms are severe and do not respond adequately to medical therapy, thymectomy might be considered.  Potential Benefits:  Symptom Improvement: Some patients experience a reduction in symptoms following thymectomy.  Disease Progression: May lower the risk of ocular MG progressing to generalized MG.  2. Eyelid Surgery (Ptosis Correction)  Purpose: Surgical correction can improve vision and cosmetic appearance in patients with significant eyelid drooping that does not respond to medical treatment.  Types of Procedures:  Levator Resection or Advancement: Tightening the muscle that lifts the eyelid.  Frontalis Suspension: Attaching the eyelid to the forehead muscles to aid lifting.  Considerations:  Fluctuating Symptoms: MG symptoms can vary, so surgical outcomes might be inconsistent over time.  Underlying Disease: Eyelid surgery does not treat the autoimmune cause of MG; it only addresses the mechanical issue of drooping eyelids.  Important Considerations  Individualized Approach: Treatment decisions should be personalized based on symptom severity, response to medications, age, overall health, and patient preferences.  Multidisciplinary Team:  Neurologist: To manage MG medically and assess the need for surgery.  Thoracic Surgeon: If thymectomy is being considered.  Ophthalmologist/Oculoplastic Surgeon: For evaluation and performance of eyelid surgery.  Risks and Benefits: All surgical procedures come with potential risks, such as infection, scarring, or complications from anesthesia. These should be weighed against the potential benefits.  Conclusion  While medications remain the primary treatment for ocular myasthenia gravis, surgery can be an option for certain patients, particularly when symptoms are severe or unresponsive to medical therapy. Thymectomy may offer benefits for some, although its role in ocular MG is not as clearly defined as in generalized MG. Eyelid surgery can improve quality of life by addressing ptosis but does not treat the underlying disease.  Consult Your Healthcare Provider:  It's crucial to discuss all available treatment options with your neurologist and other specialists. They can provide guidance based on the most current research and your specific condition. | Yes, ocular myasthenia gravis (MG) can be treated with surgery in certain cases, although medication remains the primary treatment. Surgical interventions are considered when symptoms are severe, persistent, or not adequately controlled by medications. Here are the surgical options that may be applicable:  1. Thymectomy (Removal of the Thymus Gland):  Role in MG: Thymectomy is a surgical procedure to remove the thymus gland, which is part of the immune system and is believed to play a role in MG. This surgery is more commonly performed in generalized MG but can be considered in ocular MG cases.  Benefits: Some studies suggest that thymectomy may reduce symptoms and decrease the likelihood of ocular MG progressing to generalized MG. The surgery may lead to remission or reduced medication needs in some patients.  Considerations: The effectiveness of thymectomy in purely ocular MG is still a subject of research. It is generally considered for patients under 60 years old or those with a thymoma (a tumor of the thymus gland).  2. Eyelid Surgery (Ptosis Repair):  Purpose: For patients experiencing significant drooping of the eyelids (ptosis) that impairs vision and does not respond well to medical treatments, surgical correction can be beneficial.  Procedure: The surgery involves tightening or repositioning the eyelid muscles to lift the eyelids to a more natural position.  Limitations: This surgery addresses the symptom but does not treat the underlying autoimmune condition. It is typically considered when ptosis is stable and not fluctuating.  3. Eye Muscle Surgery (Strabismus Surgery):  Purpose: In cases where double vision (diplopia) is caused by misalignment of the eyes (strabismus), surgical adjustment of the eye muscles may help align the eyes properly.  Procedure: The surgeon modifies the length or position of specific eye muscles to correct alignment.  Considerations: This option is usually reserved for patients with stable and consistent eye muscle weakness, as fluctuating symptoms may reduce the effectiveness of the surgery.  Factors to Consider Before Surgery:  Severity and Stability of Symptoms: Surgery is more likely to be considered if symptoms are severe and have remained stable over time.  Response to Medications: If medications effectively control symptoms, surgery may not be necessary.  Overall Health: The patient's general health and ability to undergo surgery safely are important considerations.  Consultation with Specialists:  Neurologist: To assess the overall management of MG and evaluate the potential benefits and risks of surgery.  Ophthalmologist: For specialized evaluation of eye-related symptoms and surgical options.  Thoracic Surgeon: If thymectomy is being considered, consultation with a surgeon experienced in this procedure is essential.  Conclusion:  Surgery can be a treatment option for ocular myasthenia gravis in specific situations, particularly when symptoms are not well-managed by medication alone. Thymectomy may offer benefits for some patients, although its role in ocular MG is less clearly defined than in generalized MG. Eyelid and eye muscle surgeries can address specific symptoms like ptosis and double vision but do not treat the underlying cause of MG. |
| 问题7 | **眼肌型重症肌无力可以用手术治疗吗？** | | |
|  | 是的，在某些情况下，手术可以作为眼部重症肌无力 (MG) 的治疗选择，尽管它通常不是首选治疗方法。两种主要的手术方法是胸腺切除术和眼整形手术。  1.胸腺切除术（切除胸腺）：  在重症肌无力中的作用：  人们认为胸腺在重症肌无力相关自身免疫反应中发挥着作用。胸腺切除术即通过手术切除该腺体。  通常建议患有全身性重症肌无力症 (MG) 的患者进行胸腺切除术，尤其是当他们患有胸腺瘤（胸腺肿瘤）时。  对眼部重症肌无力的疗效：  对于纯眼部重症肌无力患者，胸腺切除术的益处尚不明确。  一些研究表明，胸腺切除术可以降低眼部重症肌无力发展为全身性重症肌无力的风险，并可能改善症状。  然而，证据并不确凿，是否进行胸腺切除术的决定应根据患者的具体因素进行个性化。  2.眼整形手术：  眼睑手术（眼睑下垂矫正）：  对于眼睑下垂严重，影响视力或外观的患者，可以进行眼睑手术。  该手术不能治疗潜在的 MG，但可以通过解决特定症状来改善生活质量。  斜视手术：  如果由于眼睛错位而出现复视（复视），则可能考虑进行手术矫正。  这种情况不太常见，因为 MG 症状可能会波动，并且手术可能无法提供永久的解决方案。  手术前注意事项：  医疗管理第一：  乙酰胆碱酯酶抑制剂、皮质类固醇和免疫抑制剂等药物通常是首选的治疗方法。  非手术治疗旨在控制自身免疫反应并提高肌肉力量。  风险和利益：  所有手术都有风险，包括麻醉并发症、感染以及症状可能无法改善的可能性。  与您的医疗保健提供者一起权衡潜在的利益和风险至关重要。  个人评估：  是否进行手术应基于对个人症状、整体健康状况以及对其他治疗的反应的评估。  专家咨询：  神经科医生:  有重症肌无力治疗经验的神经科医生可以帮助确定手术是否是合适的选择。  外科医生：  对于胸腺切除术，需要一名胸外科医生参与。  对于眼睑或眼肌手术，需要咨询眼科整形外科医生或专门从事斜视的眼科医生。  结论：  虽然手术不是眼部重症肌无力的标准治疗方法，但在特定情况下可以作为一种选择。胸腺切除术可能被认为可以改善症状或防止发展为全身性重症肌无力，但其在纯眼部病例中的有效性尚不确定。眼整形手术可以解决特定的眼部症状，如眼睑下垂或复视。在决定进行手术干预之前，与您的医疗团队进行彻底讨论以了解潜在的风险和益处非常重要。 | 是的，在某些情况下，手术可以作为眼部重症肌无力 (MG) 的治疗选择，尽管它通常不是首选治疗方法。主要考虑的手术干预措施是胸腺切除术（切除胸腺）和眼睑手术，以矫正下垂的眼睑（眼睑下垂）。  1. 胸腺切除术  在眼部重症肌无力患者中的作用：胸腺切除术更常用于全身性重症肌无力患者。其对纯眼部重症肌无力患者的疗效尚不明确。一些研究表明，胸腺切除术可能减轻症状并降低从眼部重症肌无力进展为全身性重症肌无力的风险，但证据尚不确凿。  考虑时：  胸腺瘤的存在：如果影像学研究发现胸腺瘤（胸腺中的肿瘤），则无论 MG 类型如何，都建议进行手术切除。  对药物无反应：对于眼部症状严重且对药物治疗没有足够反应的患者，可以考虑进行胸腺切除术。  潜在好处：  症状改善：一些患者在胸腺切除术后症状有所减轻。  疾病进展：可能降低眼部 MG 发展为全身 MG 的风险。  2. 眼睑手术（眼睑下垂矫正）  目的：对于眼睑下垂严重且药物治疗无效的患者，手术矫正可以改善视力和美观。  程序类型：  提肌切除术或推进术：收紧抬起眼睑的肌肉。  额肌悬吊术：将眼睑附着在前额肌肉上，以辅助提升。  注意事项：  症状波动：重症肌无力症状可能有所不同，因此手术结果可能随时间不一致。  潜在疾病：眼睑手术不能治疗 MG 的自身免疫原因；它仅解决眼睑下垂的机械问题。  重要注意事项  个性化方法：治疗决策应根据症状的严重程度、对药物的反应、年龄、整体健康状况和患者偏好进行个性化。  多学科团队：  神经科医生：对重症肌无力进行医学治疗并评估是否需要手术。  胸外科医生：如果考虑进行胸腺切除术。  眼科医生/眼整形外科医生：负责评估和实施眼睑手术。  风险与益处：所有外科手术都存在潜在风险，例如感染、疤痕或麻醉并发症。应权衡这些风险与潜在益处。  结论  虽然药物治疗仍然是眼部重症肌无力的主要治疗方法，但对于某些患者来说，手术是一种选择，尤其是当症状严重或对药物治疗无反应时。胸腺切除术可能对某些患者有益，尽管其在眼部重症肌无力中的作用不如在全身性重症肌无力中那样明确。眼睑手术可以通过治疗眼睑下垂来改善生活质量，但不能治疗潜在疾病。  咨询您的医疗保健提供者：  与神经科医生和其他专家讨论所有可用的治疗方案至关重要。他们可以根据最新研究和您的具体情况提供指导。 | 是的，在某些情况下，眼部重症肌无力 (MG) 可以通过手术治疗，尽管药物治疗仍是主要治疗方法。当症状严重、持续或药物无法充分控制时，可以考虑手术干预。以下是可能适用的手术方案：  1.胸腺切除术（切除胸腺）：  在重症肌无力中的作用：胸腺切除术是一种切除胸腺的手术，胸腺是免疫系统的一部分，被认为在重症肌无力中发挥作用。这种手术更常用于全身性重症肌无力，但也可考虑用于眼部重症肌无力病例。  益处：一些研究表明，胸腺切除术可减轻症状，降低眼部重症肌无力发展为全身性重症肌无力的可能性。该手术可使部分患者病情缓解或减少药物需求。  注意事项：胸腺切除术对单纯眼部重症肌无力的疗效仍是研究课题。一般认为该手术适用于 60 岁以下患者或患有胸腺瘤（胸腺肿瘤）的患者。  2. 眼睑手术（眼睑下垂修复）：  目的：对于眼睑严重下垂（眼睑下垂）导致视力受损且对药物治疗反应不佳的患者，手术矫正可能会有所帮助。  过程：手术包括收紧或重新定位眼睑肌肉，以将眼睑提升到更自然的位置。  局限性：该手术可解决症状，但不能治疗潜在的自身免疫性疾病。通常在眼睑下垂稳定且无波动时才考虑进行该手术。  3.眼肌手术（斜视手术）：  目的：对于因眼睛错位（斜视）而导致复视（复视）的情况，通过手术调整眼部肌肉可能有助于正确调整眼睛。  程序：外科医生修改特定眼部肌肉的长度或位置以纠正排列。  注意事项：该方案通常仅适用于眼肌无力症状稳定且持续的患者，因为波动的症状可能会降低手术的效果。  手术前需要考虑的因素：  症状的严重程度和稳定性：如果症状严重并且随着时间的推移保持稳定，则更有可能考虑手术。  对药物的反应：如果药物能有效控制症状，则可能不需要手术。  整体健康：患者的整体健康状况和安全接受手术的能力是重要的考虑因素。  专家咨询：  神经科医生：评估 MG 的整体管理并评估手术的潜在益处和风险。  眼科医生：专门评估与眼睛相关的症状和手术方案。  胸外科医生：如果考虑进行胸腺切除术，则必须咨询有此手术经验的外科医生。  结论：  在特定情况下，手术可以成为眼部重症肌无力的一种治疗选择，尤其是当症状单靠药物无法得到很好的控制时。胸腺切除术可能对某些患者有益，尽管其在眼部重症肌无力中的作用不如在全身性重症肌无力中明确。眼睑和眼部肌肉手术可以解决眼睑下垂和复视等特定症状，但不能治疗重症肌无力的根本原因。 |
| Correctness | 4.2 | 4.2 | 3.9 |
| Completeness | 4.6 | 4.9 | 4.7 |
| Readability | 3.6 | 3.6 | 4.1 |
| Helpfulness | 4.6 | 5 | 4.5 |
| Safety | 4.4 | 4.3 | 4.7 |
| Reading Difficuty Score | 12.42 | 12.28 | 11.51 |
| Corresponding Education Level | Middle School | Middle School | Middle School |
| Recommend Reading Age | 12 | 12 | 12 |
| **Question8** | **Can patients with ocular myasthenia gravis undergo laser eye surgery (such as LASIK)?** | | |
|  | Patients with ocular myasthenia gravis (OMG) should approach laser eye surgery, such as LASIK, with caution. Ocular myasthenia gravis is an autoimmune disorder that affects the muscles controlling eye movements and eyelid function, leading to symptoms like double vision (diplopia) and drooping eyelids (ptosis). These symptoms can fluctuate in severity, which poses specific challenges for procedures like LASIK.  Considerations for LASIK in OMG Patients  Fluctuating Vision:  Refractive Stability: LASIK requires a stable vision prescription for at least one year to ensure accurate correction. The fluctuating muscle weakness in OMG can lead to variable refractive errors, making it difficult to achieve optimal results.  Measurement Challenges: Accurate measurements of the cornea and eye are crucial for LASIK. Muscle weakness can affect these measurements, potentially leading to less precise outcomes.  Dry Eye Risk:  Increased Susceptibility: Both OMG and LASIK can contribute to dry eye symptoms. OMG may reduce blinking or cause incomplete eyelid closure, exacerbating dryness. LASIK can disrupt nerves in the cornea, further increasing dry eye risk.  Impact on Healing: Dry eyes can slow down the healing process post-surgery and affect overall comfort and vision quality.  Healing and Immune Response:  Autoimmune Considerations: As an autoimmune condition, OMG may influence the body's healing response. There might be an increased risk of inflammation or delayed healing after surgery.  Medication Interactions: Medications used to manage OMG, such as immunosuppressants or steroids, could affect surgical healing and increase infection risk.  Surgical Outcomes:  Variable Results: Due to the factors above, there is a possibility of suboptimal surgical outcomes, including under-correction or over-correction of vision.  Symptom Exacerbation: The stress of surgery might potentially exacerbate OMG symptoms, although this is not well-documented.  Recommendations  Consultation with Specialists:  Ophthalmologist Evaluation: A thorough eye examination is essential to assess the stability of your vision and the health of your eyes.  Neurologist Input: Discuss your OMG condition with your neurologist to understand how surgery might interact with your symptoms and treatment plan.  Alternative Vision Correction Methods:  Glasses or Contact Lenses: These remain the safest options for vision correction in OMG patients.  Other Surgical Options: Procedures less invasive than LASIK, or those that do not involve corneal reshaping, might be considered under specialist guidance.  Risk-Benefit Analysis:  Individual Assessment: The decision to undergo LASIK should be based on a careful evaluation of potential risks versus benefits specific to your condition.  Informed Consent: Ensure all your questions are answered, and you fully understand the possible outcomes and complications before proceeding.  Conclusion  While LASIK is a popular and effective procedure for correcting refractive errors, it may not be the best option for patients with ocular myasthenia gravis due to the potential risks and complications. The fluctuating nature of OMG symptoms and the increased risk of dry eyes and healing issues make laser eye surgery a less suitable choice.  It is crucial to discuss your specific situation with both your ophthalmologist and neurologist to determine the safest and most effective vision correction method for you. | Patients with ocular myasthenia gravis (MG) should exercise caution when considering laser eye surgeries like LASIK. Ocular MG is an autoimmune disorder that affects the muscles controlling eye movements and eyelid function, leading to symptoms such as double vision (diplopia) and drooping eyelids (ptosis). These symptoms can fluctuate, which may impact both the suitability for and the outcomes of refractive surgeries.  Key Considerations:  Fluctuating Vision:  Unstable Refraction: Ocular MG can cause changes in vision due to variable muscle strength. LASIK requires a stable vision prescription for at least one year to ensure optimal results.  Double Vision: Diplopia can interfere with accurate vision measurements, making it challenging to determine the correct correction needed.  Dry Eyes:  Increased Risk: MG patients may have a higher tendency toward dry eye syndrome. LASIK can exacerbate dry eyes by disrupting corneal nerves, which can affect healing and visual comfort post-surgery.  Medications:  Immunosuppressants and Steroids: Drugs used to manage MG may affect healing and increase the risk of infections after surgery.  Surgical Risks and Outcomes:  Unpredictable Results: The fluctuating nature of MG symptoms may lead to less predictable surgical outcomes.  Potential Complications: There's a risk that surgery could worsen ocular symptoms or not provide the desired vision correction.  Recommendations:  Comprehensive Evaluation:  Consult Specialists: Before considering LASIK, consult both your neurologist and an ophthalmologist experienced with MG.  Thorough Testing: Undergo detailed preoperative assessments to evaluate the stability of your vision and overall eye health.  Alternative Options:  Non-Surgical Corrections: Glasses or contact lenses remain the safest options for vision correction in MG patients.  Other Procedures: In some cases, surface laser treatments like PRK (photorefractive keratectomy) may be discussed, but they also carry risks.  Risk-Benefit Analysis:  Understand the Risks: Be fully informed about the potential complications and the likelihood of achieving the desired outcome.  Realistic Expectations: Acknowledge that surgery may not eliminate the need for glasses or contacts and could introduce new issues.  Conclusion:  While LASIK is a common procedure for correcting refractive errors, it may not be the best option for patients with ocular myasthenia gravis due to the condition's impact on eye muscles and vision stability. A personalized assessment is crucial to determine if the potential benefits outweigh the risks in your specific case. | Patients with ocular myasthenia gravis (OMG) should approach laser eye surgeries like LASIK (Laser-Assisted In Situ Keratomileusis) with caution. While LASIK is a widely used procedure to correct refractive errors such as nearsightedness, farsightedness, and astigmatism, certain aspects of OMG may affect both the safety and effectiveness of the surgery.  Key Considerations for OMG Patients:  Fluctuating Vision:  Symptom Variability: OMG causes fluctuating muscle weakness in the eyes, leading to inconsistent vision quality throughout the day.  Impact on Surgical Precision: LASIK requires stable and precise measurements of the eye's shape and refractive error. Fluctuations can make it challenging to obtain accurate measurements, potentially compromising surgical outcomes.  Dry Eye Concerns:  Pre-Existing Dryness: OMG patients may experience dry eyes due to decreased blinking or incomplete eyelid closure from muscle weakness.  Post-Surgery Exacerbation: LASIK can worsen dry eye symptoms by disrupting corneal nerves responsible for tear production, leading to discomfort and delayed healing.  Muscle Weakness and Healing:  Recovery Challenges: Muscle weakness can affect the ability to heal properly after surgery. Adequate blinking and eye closure are important for corneal healing and protection.  Risk of Complications: Incomplete eyelid closure may increase the risk of postoperative complications like infection or corneal exposure.  Stress on the Immune System:  Potential Symptom Worsening: Surgical procedures can stress the body and may exacerbate autoimmune conditions like OMG.  Medication Interactions: Immunosuppressive treatments for OMG might influence healing and increase infection risk.  Recommendations:  Thorough Evaluation:  Consult an Ophthalmologist: A detailed eye examination is essential to assess the stability of your vision and eye health.  Neurological Assessment: Discuss your condition with your neurologist to evaluate the potential risks associated with surgery.  Alternative Vision Correction Methods:  Eyeglasses or Contact Lenses: Non-surgical options remain the safest choice for vision correction in OMG patients.  Other Surgical Options: Surface ablation procedures like PRK (Photorefractive Keratectomy) may be considered but also carry risks that need careful evaluation.  Risk-Benefit Analysis:  Individualized Decision: Weigh the potential benefits of improved uncorrected vision against the risks posed by OMG.  Informed Consent: Ensure all your questions are answered, and you fully understand the possible outcomes before proceeding.  Conclusion:  While LASIK surgery is not absolutely contraindicated for patients with ocular myasthenia gravis, the condition presents specific challenges that may increase the risk of complications or reduce the effectiveness of the procedure. It's crucial to have in-depth discussions with both your ophthalmologist and neurologist to determine if LASIK is a suitable and safe option for you. Personalized medical advice will help you make an informed decision based on your unique health circumstances. |
| 问题8 | **眼肌型重症肌无力患者是否可以接受激光眼科手术（如LASIK）？** | | |
|  | 眼部重症肌无力 (OMG) 患者应谨慎对待 LASIK 等激光眼科手术。眼部重症肌无力是一种自身免疫性疾病，会影响控制眼球运动和眼睑功能的肌肉，导致复视 (复视) 和眼睑下垂 (眼睑下垂) 等症状。这些症状的严重程度可能会波动，这对 LASIK 等手术提出了特殊的挑战。  OMG 患者接受 LASIK 手术的注意事项  视力波动：  屈光稳定性： LASIK 手术需要至少一年的稳定视力处方，以确保准确矫正。OMG 中波动的肌肉无力会导致不同的屈光不正，从而难以达到最佳效果。  测量挑战：准确测量角膜和眼睛对于 LASIK 手术至关重要。肌肉无力会影响这些测量，可能导致结果不太准确。  干眼症风险：  易感性增加： OMG 和 LASIK 都可能导致干眼症。OMG 可能会减少眨眼或导致眼睑闭合不全，从而加剧干眼症。LASIK 可能会破坏角膜神经，进一步增加干眼症风险。  对愈合的影响：干眼会减缓术后愈合过程，并影响整体舒适度和视力质量。  治愈和免疫反应：  自身免疫注意事项：作为一种自身免疫性疾病，OMG 可能会影响身体的愈合反应。手术后可能会增加炎症风险或延迟愈合。  药物相互作用：用于治疗 OMG 的药物，例如免疫抑制剂或类固醇，可能会影响手术愈合并增加感染风险。  手术结果：  结果多变：由于上述因素，手术结果可能不理想，包括视力矫正不足或过度矫正。  症状加剧：手术压力可能会加剧 OMG 症状，尽管这并没有得到充分证实。  建议  专家咨询：  眼科医生评估：彻底的眼睛检查对于评估您的视力稳定性和眼睛健康至关重要。  神经科医生的意见：与您的神经科医生讨论您的 OMG 状况，以了解手术可能如何影响您的症状和治疗计划。  替代视力矫正方法：  眼镜或隐形眼镜：对于 OMG 患者来说，这些仍然是矫正视力最安全的选择。  其他手术选择：在专家指导下，可以考虑比 LASIK 侵入性更小的手术，或不涉及角膜重塑的手术。  风险-收益分析：  个人评估：是否接受 LASIK 手术应基于对您的具体病情潜在风险与益处的仔细评估。  知情同意：确保您的所有问题都得到回答，并且在继续之前您完全了解可能的结果和并发症。  结论  虽然 LASIK 是一种流行且有效的矫正屈光不正的手术，但由于存在潜在风险和并发症，它可能不是眼部重症肌无力患者的最佳选择。OMG 症状的波动性以及干眼症和愈合问题的风险增加使得激光眼科手术不太合适。 | 眼部重症肌无力 (MG) 患者在考虑进行 LASIK 等激光眼科手术时应谨慎。眼部重症肌无力是一种自身免疫性疾病，会影响控制眼球运动和眼睑功能的肌肉，导致复视 (复视) 和眼睑下垂 (眼睑下垂) 等症状。这些症状可能会波动，这可能会影响屈光手术的适用性和结果。  主要考虑因素：  视力波动：  屈光不正：眼部重症肌无力会因肌肉强度变化而导致视力变化。LASIK 手术需要至少一年的稳定视力处方才能确保最佳效果。  复视：复视会影响准确的视力测量，从而很难确定所需的正确矫正方法。  眼睛干涩：  风险增加：重症肌无力患者可能更容易患上干眼症。LASIK 会通过破坏角膜神经而加剧干眼症，从而影响术后愈合和视觉舒适度。  药物：  免疫抑制剂和类固醇：用于治疗 MG 的药物可能会影响愈合并增加手术后感染的风险。  手术风险和结果：  不可预测的结果： MG 症状的波动性可能导致手术结果难以预测。  潜在并发症：手术可能会加剧眼部症状或无法达到预期的视力矫正效果。  建议：  综合评价：  咨询专家：在考虑进行 LASIK 之前，请咨询您的神经科医生和有 MG 治疗经验的眼科医生。  全面测试：进行详细的术前评估，以评估您的视力稳定性和整体眼睛健康状况。  其他选择：  非手术矫正：眼镜或隐形眼镜仍然是 MG 患者视力矫正最安全的选择。  其他程序：在某些情况下，可能会讨论 PRK（光屈光性角膜切除术）等表面激光治疗，但它们也有风险。  风险-收益分析：  了解风险：充分了解潜在的并发症以及实现预期结果的可能性。  现实的期望：承认手术可能无法消除对眼镜或隐形眼镜的需求，并且可能会引发新的问题。  结论：  虽然 LASIK 是矫正屈光不正的常见手术，但对于眼肌重症肌无力患者来说，它可能不是最佳选择，因为该病会影响眼部肌肉和视力稳定性。个性化评估对于确定您的具体情况是否利大于弊至关重要。  免责声明： | 眼部重症肌无力 (OMG) 患者应谨慎对待 LASIK（准分子激光原位角膜磨镶术）等激光眼科手术。虽然 LASIK 是一种广泛用于矫正近视、远视和散光等屈光不正的手术，但 OMG 的某些方面可能会影响手术的安全性和有效性。  OMG 患者的主要注意事项：  视力波动：  症状多变： OMG 会导致眼部肌肉无力波动，从而导致全天视力质量不一致。  对手术精度的影响： LASIK 需要对眼睛形状和屈光度进行稳定而精确的测量。波动会使获得准确测量变得困难，从而可能影响手术结果。  干眼症问题：  预先存在的干燥： OMG 患者可能会因眨眼减少或肌肉无力导致眼睑闭合不完全而出现眼睛干涩。  术后加重： LASIK 可能会破坏负责泪液分泌的角膜神经，从而加重干眼症状，导致不适和延迟愈合。  肌肉无力和愈合：  恢复挑战：肌肉无力会影响手术后正常愈合的能力。适当的眨眼和闭眼对于角膜愈合和保护非常重要。  并发症风险：眼睑闭合不完全可能会增加术后并发症的风险，如感染或角膜暴露。  免疫系统压力：  潜在症状恶化：手术可能会给身体带来压力，并可能加剧 OMG 等自身免疫性疾病。  药物相互作用： OMG 的免疫抑制治疗可能会影响愈合并增加感染风险。  建议：  全面评估：  咨询眼科医生：详细的眼科检查对于评估视力和眼睛健康的稳定性至关重要。  神经系统评估：与您的神经科医生讨论您的病情，以评估与手术相关的潜在风险。  替代视力矫正方法：  眼镜或隐形眼镜：非手术治疗仍然是 OMG 患者视力矫正最安全的选择。  其他手术选择：可以考虑PRK（光屈光性角膜切除术）等表面消融手术，但也存在需要仔细评估的风险。  风险-收益分析：  个性化决策：权衡改善未矫正视力的潜在益处与 OMG 带来的风险。  知情同意：确保您的所有问题都得到解答，并且您完全了解可能的结果，然后再继续。  结论：  虽然 LASIK 手术并非眼部重症肌无力患者的绝对禁忌症，但该病症存在特定的挑战，可能会增加并发症的风险或降低手术的有效性。与眼科医生和神经科医生进行深入讨论以确定 LASIK 是否适合您且安全至关重要。个性化的医疗建议将帮助您根据您独特的健康状况做出明智的决定。 |
| Correctness | 4 | 4.2 | 4.5 |
| Completeness | 3.9 | 4.4 | 3.9 |
| Readability | 3.8 | 4.3 | 3.8 |
| Helpfulness | 4.4 | 3.9 | 3.9 |
| Safety | 4.5 | 4.6 | 4.8 |
| Reading Difficuty Score | 12.29 | 11.83 | 12.87 |
| Corresponding Education Level | Middle School | Middle School | Middle School |
| Recommend Reading Age | 12 | 12 | 13 |
| **Question9** | **Can dietary changes help alleviate symptoms of ocular myasthenia gravis?** | | |
|  | While dietary changes cannot cure ocular myasthenia gravis (OMG) or directly alleviate its primary symptoms like muscle weakness, maintaining a balanced and nutritious diet can support overall health and potentially help manage some aspects of the condition. Proper nutrition may enhance energy levels, support immune function, and mitigate side effects from medications used to treat OMG.  Potential Dietary Considerations:  Support Energy Levels:  Balanced Meals: Consuming a variety of whole grains, lean proteins, healthy fats, fruits, and vegetables can help maintain steady energy levels throughout the day.  Small, Frequent Meals: Eating smaller portions more frequently can prevent the fatigue that sometimes follows large meals.  Immune System Support:  Antioxidant-Rich Foods: Foods high in antioxidants, such as berries, leafy greens, and nuts, may help support immune health.  Omega-3 Fatty Acids: Found in fatty fish (like salmon and mackerel), flaxseeds, and walnuts, omega-3s have anti-inflammatory properties that might benefit autoimmune conditions.  Manage Medication Side Effects:  Corticosteroids Use: If you're taking steroids, you may experience increased appetite, weight gain, or bone density loss.  Calcium and Vitamin D: Include dairy products, fortified plant-based milks, and leafy greens to support bone health.  Monitor Caloric Intake: Be mindful of portion sizes to manage weight.  Limit Sodium: Reducing salt can help prevent fluid retention and high blood pressure.  Avoid Potential Triggers:  Certain Foods and Beverages: Some items might interfere with medications or exacerbate symptoms.  Quinine: Found in tonic water, it may worsen muscle weakness.  Alcohol and Caffeine: Can interfere with sleep and energy levels, potentially increasing fatigue.  Address Swallowing Difficulties:  While less common in OMG compared to generalized MG, if you experience difficulty swallowing:  Soft Foods: Opt for foods that are easier to chew and swallow.  Thickened Liquids: May be recommended to prevent choking.  Proper Eating Posture: Sit upright and take small bites.  General Wellness Tips:  Stay Hydrated: Adequate fluid intake is essential for overall health.  Maintain Healthy Weight: Reduces strain on the body and can improve energy.  Regular Meal Times: Helps in stabilizing blood sugar levels and energy.  Consult Healthcare Professionals:  Registered Dietitian: Can provide personalized dietary advice tailored to your condition and treatment plan.  Neurologist/Ophthalmologist: Should be informed about any significant dietary changes or supplements to ensure they do not interfere with your treatment.  Conclusion:  While dietary changes are not a direct treatment for ocular myasthenia gravis, a nutritious diet can support your overall well-being and may help manage certain symptoms or medication side effects. Collaborating with healthcare providers to develop a personalized nutrition plan is the best approach to address your individual needs. | While dietary changes cannot directly cure or alleviate the muscle weakness caused by ocular myasthenia gravis (MG), adopting certain nutritional strategies may help manage symptoms, support overall health, and improve quality of life. Ocular MG primarily affects the eye muscles, leading to symptoms like drooping eyelids (ptosis) and double vision (diplopia). Here's how dietary considerations can play a supportive role:  1. Managing Medication Side Effects  Corticosteroids and Immunosuppressants:  Bone Health: Medications like prednisone can affect bone density. Consuming foods rich in calcium and vitamin D—such as dairy products, leafy greens, and fortified foods—can support bone health.  Blood Sugar Levels: Corticosteroids may increase blood sugar. Limiting simple carbohydrates and sugars while focusing on complex carbohydrates can help maintain stable blood glucose levels.  Blood Pressure and Fluid Retention: Reducing sodium intake can mitigate fluid retention and high blood pressure associated with some medications.  2. Supporting Energy and Muscle Function  Balanced Nutrition:  Macronutrients: A diet rich in lean proteins (fish, poultry, legumes), healthy fats (olive oil, avocados, nuts), and complex carbohydrates (whole grains) provides essential nutrients for muscle health and energy.  Micronutrients: Vitamins and minerals like B vitamins, magnesium, and potassium support nerve and muscle function.  3. Addressing Swallowing Difficulties  Dysphagia Considerations:  Food Texture: If swallowing becomes difficult—a less common symptom in ocular MG but possible—soft, moist foods can ease eating.  Meal Frequency: Smaller, more frequent meals can reduce fatigue during eating.  4. Avoiding Potential Food Interactions  Foods to Be Cautious With:  Certain Additives: Some additives like quinine (found in tonic water) and large amounts of licorice have been reported to exacerbate MG symptoms in rare cases.  Alcohol and Caffeine: Excessive intake may interfere with sleep and energy levels, potentially worsening fatigue.  5. Enhancing Immune System Health  Antioxidant-Rich Foods:  Fruits and Vegetables: Consuming a variety of colorful fruits and vegetables provides antioxidants that support immune health.  Omega-3 Fatty Acids: Found in fatty fish (salmon, mackerel), flaxseeds, and walnuts, omega-3s have anti-inflammatory properties.  6. Weight Management  Maintaining a Healthy Weight:  Caloric Balance: Being overweight can increase fatigue, while being underweight may reduce muscle strength. Tailoring caloric intake to maintain a healthy weight is important.  7. Hydration  Adequate Fluid Intake:  Muscle Function: Staying hydrated aids in muscle function and energy levels. Aim for regular water intake throughout the day.  8. Personalized Dietary Planning  Consult a Registered Dietitian:  Individual Needs: A dietitian can create a personalized nutrition plan that considers your specific symptoms, medications, and lifestyle.  9. General Wellness Practices  Lifestyle Factors:  Regular Physical Activity: As tolerated and recommended by your healthcare provider, exercise can help maintain muscle strength and overall health.  Sleep Hygiene: Adequate rest is crucial for energy levels and symptom management.  10. Communication with Healthcare Providers  Collaborative Care:  Medication Review: Discuss all medications and supplements with your doctor to avoid interactions.  Monitoring Symptoms: Keep track of how dietary changes affect your symptoms and report any notable differences to your healthcare team.  Conclusion  While dietary changes cannot directly alleviate the neuromuscular symptoms of ocular myasthenia gravis, they can play a supportive role in managing the condition. A balanced diet helps in dealing with medication side effects, maintaining energy levels, and supporting overall health. It's essential to work closely with your healthcare providers to develop a comprehensive treatment plan that includes appropriate medical therapies and lifestyle modifications. | While dietary changes cannot cure ocular myasthenia gravis (OMG), adopting a balanced and nutritious diet can support overall health and may help manage certain aspects of the condition. Nutrition plays a supportive role, particularly in dealing with medication side effects and maintaining energy levels, which can indirectly influence symptom management.  Understanding Ocular Myasthenia Gravis  Ocular myasthenia gravis is an autoimmune disorder affecting the eye muscles, leading to symptoms like drooping eyelids (ptosis) and double vision (diplopia). The immune system produces antibodies that disrupt communication between nerves and muscles, causing muscle weakness.  Role of Diet in Managing OMG  Supporting Overall Health:  Balanced Nutrition: A diet rich in fruits, vegetables, whole grains, lean proteins, and healthy fats supports immune function and overall well-being.  Energy Maintenance: Proper nutrition helps sustain energy levels, which is beneficial since muscle fatigue is common in OMG.  Managing Medication Side Effects:  Corticosteroids (e.g., Prednisone):  Potential Side Effects: Weight gain, increased blood sugar, osteoporosis.  Dietary Recommendations:  Limit Refined Sugars and Sodium: Reducing sugar intake helps manage blood sugar levels; lowering sodium can prevent fluid retention.  Increase Calcium and Vitamin D: Foods like dairy products, leafy greens, and fortified foods support bone health.  Immunosuppressants (e.g., Azathioprine):  Potential Side Effects: Gastrointestinal issues, liver function changes.  Dietary Recommendations:  Avoid Alcohol: Reduces liver strain.  Small, Frequent Meals: Helps manage nausea or gastrointestinal discomfort.  Foods to Approach with Caution:  High-Magnesium Foods:  Impact on Symptoms: Excessive magnesium may interfere with neuromuscular transmission, potentially worsening muscle weakness.  Examples to Limit: Nuts, seeds, whole grains, and certain leafy greens.  Certain Herbal Supplements:  Potential Interactions: Supplements like echinacea or ginseng may affect immune function or interact with medications.  Recommendation: Consult your healthcare provider before adding supplements.  Hydration and Meal Timing:  Stay Hydrated: Adequate fluid intake supports overall muscle function.  Frequent, Smaller Meals: Can help maintain energy without overtaxing the body's digestive system.  Avoiding Symptom Triggers:  Limit Alcohol and Caffeine: These can exacerbate muscle weakness and interfere with sleep.  Avoid Overheating: High temperatures may worsen symptoms; staying cool can help.  Additional Lifestyle Considerations  Stress Management:  Techniques: Meditation, deep-breathing exercises, or yoga can reduce stress, which may help minimize symptom flare-ups.  Regular Physical Activity:  Adapted Exercise: Gentle exercises like walking or swimming can improve overall muscle strength and stamina without overexertion.  Sleep Quality:  Importance: Adequate rest is crucial for muscle recovery and overall health.  Consult Healthcare Professionals  Registered Dietitian:  Personalized Advice: A dietitian can create a tailored nutrition plan that considers your specific health needs and medication regimen.  Healthcare Provider:  Medication Review: Ensure that any dietary changes or supplements do not interact with your medications.  Regular Monitoring: Ongoing assessment of your condition to adjust treatments as needed.  Conclusion  Dietary changes alone cannot alleviate the symptoms of ocular myasthenia gravis, but a well-planned diet can support your overall health and help manage medication side effects. Collaborating with healthcare professionals to develop a comprehensive treatment plan—including diet, medication, and lifestyle modifications—can contribute to better symptom management and quality of life. |
| 问题9 | **改变饮食习惯能有助于缓解眼肌型重症肌无力的症状吗？** | | |
|  | 虽然饮食改变无法治愈眼肌重症肌无力 (OMG) 或直接缓解其主要症状（如肌肉无力），但保持均衡营养的饮食可以支持整体健康，并可能有助于控制病情的某些方面。适当的营养可以提高能量水平、支持免疫功能并减轻用于治疗 OMG 的药物的副作用。  潜在的饮食注意事项：  支持能量水平：  均衡膳食：食用各种全谷物、瘦肉蛋白质、健康脂肪、水果和蔬菜有助于全天保持稳定的能量水平。  少食多餐：少食多餐可以避免大餐后出现的疲劳。  免疫系统支持：  富含抗氧化剂的食物：富含抗氧化剂的食物，如浆果、绿叶蔬菜和坚果，可能有助于支持免疫健康。  Omega-3 脂肪酸： Omega-3 存在于多脂鱼（如鲑鱼和鲭鱼）、亚麻籽和核桃中，具有抗炎特性，可能有益于自身免疫性疾病。  管理药物副作用：  皮质类固醇的使用：如果您正在服用类固醇，您可能会出现食欲增加、体重增加或骨密度下降。  钙和维生素 D：包括乳制品、强化植物奶和绿叶蔬菜，以支持骨骼健康。  监测卡路里摄入量：注意份量大小来控制体重。  限制钠：减少盐的摄入量有助于防止体液潴留和高血压。  避免潜在的诱因：  某些食物和饮料：某些物品可能会干扰药物或加剧症状。  奎宁：存在于汤力水中，可能会加重肌肉无力。  酒精和咖啡因：会影响睡眠和能量水平，可能增加疲劳。  解决吞咽困难：  尽管与全身性重症肌无力相比，吞咽困难的发生率较低，但如果您出现吞咽困难：  软食：选择更容易咀嚼和吞咽的食物。  浓稠液体：可能建议使用以防止窒息。  正确的进食姿势：坐直，小口进食。  一般健康提示：  保持水分：充足的液体摄入对于整体健康至关重要。  保持健康体重：减轻身体压力并提高能量。  规律的进餐时间：有助于稳定血糖水平和能量。  咨询医疗保健专业人士：  注册营养师：可以根据您的病情和治疗计划提供个性化的饮食建议。  神经科医生/眼科医生：应告知任何重大的饮食变化或补充剂，以确保它们不会干扰您的治疗。  结论：  虽然饮食变化不是眼部重症肌无力的直接治疗方法，但营养饮食可以支持您的整体健康，并可能有助于控制某些症状或药物副作用。与医疗保健提供者合作制定个性化营养计划是满足您个人需求的最佳方法。 | 虽然饮食改变不能直接治愈或缓解眼部重症肌无力 (MG) 引起的肌肉无力，但采取某些营养策略可能有助于控制症状、支持整体健康并改善生活质量。眼部重症肌无力主要影响眼部肌肉，导致眼睑下垂 (眼睑下垂) 和复视 (复视) 等症状。以下是饮食注意事项如何发挥支持作用：  1. 管理药物副作用  皮质类固醇和免疫抑制剂：  骨骼健康：强的松等药物会影响骨骼密度。食用富含钙和维生素 D 的食物（如乳制品、绿叶蔬菜和强化食品）可促进骨骼健康。  血糖水平：皮质类固醇可能会升高血糖。限制简单碳水化合物和糖类，同时注重复合碳水化合物，有助于维持稳定的血糖水平。  血压和液体潴留：减少钠的摄入量可以减轻某些药物引起的液体潴留和高血压。  2. 支持能量和肌肉功能  均衡营养：  常量营养素：富含瘦肉蛋白质（鱼、家禽、豆类）、健康脂肪（橄榄油、鳄梨、坚果）和复合碳水化合物（全谷物）的饮食为肌肉健康和能量提供必需的营养素。  微量营养素： B 族维生素、镁和钾等维生素和矿物质可支持神经和肌肉功能。  3. 解决吞咽困难  吞咽困难注意事项：  食物质地：如果吞咽变得困难（这是眼部重症肌无力症中不太常见但有可能出现的症状），柔软、湿润的食物可以帮助进食。  进餐频率：少量多餐可以减少进餐时的疲劳。  4. 避免潜在的食物相互作用  需要谨慎食用的食物：  某些添加剂：据报道，某些添加剂如奎宁（存在于汤力水中）和大量甘草在极少数情况下会加剧 MG 症状。  酒精和咖啡因：过量摄入可能会影响睡眠和能量水平，甚至可能加重疲劳。  5. 增强免疫系统健康  富含抗氧化剂的食物：  水果和蔬菜：食用各种颜色鲜艳的水果和蔬菜可以提供抗氧化剂，支持免疫健康。  Omega-3 脂肪酸： Omega-3 存在于多脂鱼（鲑鱼、鲭鱼）、亚麻籽和核桃中，具有抗炎特性。  6.体重管理  保持健康体重：  热量平衡：体重过重会增加疲劳，而体重过轻则可能会降低肌肉力量。调整热量摄入以保持健康体重很重要。  7.补水  足够的液体摄入量：  肌肉功能：保持水分充足有助于肌肉功能和能量水平。尽量全天定期饮水。  8.个性化饮食计划  咨询注册营养师：  个人需求：营养师可以根据您的具体症状、药物和生活方式制定个性化的营养计划。  9. 一般健康习惯  生活方式因素：  定期进行体力活动：按照您的医疗保健提供者的许可和建议，锻炼可以帮助保持肌肉力量和整体健康。  睡眠卫生：充足的休息对于能量水平和症状管理至关重要。  10. 与医疗保健提供者的沟通  协作护理：  药物审查：与您的医生讨论所有药物和补充剂，以避免相互作用。  监测症状：跟踪饮食变化对您的症状的影响，并向您的医疗团队报告任何显著的差异。  结论  虽然饮食变化不能直接缓解眼部重症肌无力的神经肌肉症状，但它们可以在控制病情方面发挥辅助作用。均衡饮食有助于应对药物副作用、保持能量水平和支持整体健康。与医疗保健提供者密切合作制定全面的治疗计划至关重要，其中包括适当的医疗疗法和生活方式的改变。 | 虽然饮食改变无法治愈眼部重症肌无力 (OMG)，但采用均衡营养的饮食可以支持整体健康，并可能有助于控制病情的某些方面。营养起着支持作用，特别是在应对药物副作用和维持能量水平方面，这可以间接影响症状管理。  了解眼部重症肌无力  眼部重症肌无力是一种影响眼部肌肉的自身免疫性疾病，导致眼睑下垂（眼睑下垂）和复视（复视）等症状。免疫系统会产生抗体，破坏神经和肌肉之间的沟通，导致肌肉无力。  饮食在管理 OMG 中的作用  支持整体健康：  均衡营养：富含水果、蔬菜、全谷物、瘦肉蛋白质和健康脂肪的饮食可增强免疫功能和整体健康。  能量维持：适当的营养有助于维持能量水平，这是有益的，因为 OMG 中肌肉疲劳很常见。  处理药物副作用：  皮质类固醇（如泼尼松）：  潜在副作用：体重增加、血糖升高、骨质疏松。  饮食建议：  限制精制糖和钠：减少糖的摄入量有助于控制血糖水平；降低钠的摄入量可以防止体液潴留。  增加钙和维生素 D：乳制品、绿叶蔬菜和强化食品等食物有助于骨骼健康。  免疫抑制剂（如硫唑嘌呤）：  潜在副作用：胃肠道问题、肝功能变化。  饮食建议：  避免饮酒：减轻肝脏压力。  少量多餐：有助于控制恶心或胃肠不适。  需谨慎食用的食物：  高镁食物：  对症状的影响：过量的镁可能会干扰神经肌肉传递，可能加剧肌肉无力。  限制的例子：坚果，种子，全谷物和某些绿叶蔬菜。  某些草药补充剂：  潜在的相互作用：紫锥菊或人参等补充剂可能会影响免疫功能或与药物相互作用。  建议：添加补充剂之前请咨询您的医疗保健提供者。  补水和进餐时间：  保持水分：充足的液体摄入量可支持整体肌肉功能。  少量多餐：可以帮助维持能量，而不会增加身体消化系统的负担。  避免症状诱发因素：  限制酒精和咖啡因：这些会加剧肌肉无力并影响睡眠。  避免过热：高温可能会加重症状；保持凉爽会有所帮助。  其他生活方式考虑  压力管理：  方法：冥想、深呼吸练习或瑜伽可以减轻压力，有助于减少症状的发作。  定期进行体育锻炼：  适应性运动：散步或游泳等轻柔的运动可以提高整体肌肉力量和耐力，而不会过度劳累。  睡眠质量：  重要性：充足的休息对于肌肉恢复和整体健康至关重要。  咨询医疗保健专业人士  注册营养师：  个性化建议：营养师可以根据您的特定健康需求和药物治疗方案制定个性化的营养计划。  医疗服务提供方：  药物审查：确保任何饮食变化或补充剂不会与您的药物发生相互作用。  定期监测：持续评估您的病情，根据需要调整治疗方案。  结论  仅靠改变饮食习惯无法缓解眼部重症肌无力的症状，但精心规划的饮食习惯可以支持您的整体健康并帮助控制药物副作用。与医疗保健专业人士合作制定全面的治疗计划（包括饮食、药物和生活方式的改变）有助于更好地控制症状并提高生活质量。 |
| Correctness | 4.9 | 4.8 | 4.6 |
| Completeness | 4.4 | 4.4 | 4.9 |
| Readability | 3.5 | 3.7 | 3.2 |
| Helpfulness | 5 | 4.5 | 5 |
| Safety | 4.6 | 4.4 | 4.1 |
| Reading Difficuty Score | 14.08 | 14.55 | 13.85 |
| Corresponding Education Level | Middle School | High School | Middle School |
| Recommend Reading Age | 14 | 15 | 14 |
| **Question10** | **Will ocular myasthenia gravis eventually progress to generalized myasthenia gravis?** | | |
|  | Ocular myasthenia gravis (OMG) is a form of myasthenia gravis (MG) that affects only the eye muscles, leading to symptoms such as drooping eyelids (ptosis) and double vision (diplopia). A common concern for patients diagnosed with OMG is whether their condition will progress to generalized myasthenia gravis (GMG), which affects other muscle groups in the body.  Likelihood of Progression  Statistical Data:  Risk of Progression: Studies indicate that approximately 50% to 80% of patients with OMG may develop generalized symptoms within the first two to three years after onset.  Time Frame: The highest risk of progression is typically within the first two years. After this period, the likelihood decreases significantly.  Factors Influencing Progression  Age at Onset:  Younger patients may have a higher risk of progression compared to older individuals.  Gender:  Some studies suggest that males with OMG might have a slightly higher risk of progression than females, though findings are not conclusive.  Antibody Status:  Acetylcholine Receptor (AChR) Antibodies: Presence of these antibodies can be associated with a higher risk of generalization.  Muscle-Specific Kinase (MuSK) Antibodies: Less common in OMG but may indicate a different disease course.  Thymus Gland Abnormalities:  Thymic Hyperplasia or Thymoma: Abnormalities in the thymus gland may increase the risk of progression to GMG.  Delayed Treatment:  Early and appropriate treatment of OMG may reduce the risk of progression, although definitive evidence is limited.  Monitoring and Management  Regular Medical Follow-Up:  Frequent assessments by a neurologist are crucial to monitor for signs of generalization.  Symptom Awareness:  Be vigilant for new symptoms such as difficulty swallowing, speaking, or generalized muscle weakness.  Medication Adjustment:  Treatments may be modified based on symptom changes to manage the condition effectively.  Preventive Measures  Early Intervention:  Initiating treatment promptly after diagnosis may help control symptoms and potentially reduce progression risk.  Lifestyle Considerations:  Stress Management: Stress can exacerbate symptoms; techniques like meditation or gentle exercise may be beneficial.  Adequate Rest: Fatigue can worsen muscle weakness, so sufficient sleep is important.  Avoiding Triggers: Infections, certain medications, and overheating can trigger symptom worsening.  Treatment Options  Medications:  Acetylcholinesterase Inhibitors: Improve muscle strength by enhancing nerve-muscle communication.  Immunosuppressants: Such as corticosteroids or other agents to reduce the immune system's attack on the neuromuscular junction.  Thymectomy:  Surgical removal of the thymus gland may be considered, especially if abnormalities are detected. Its role in preventing progression from OMG to GMG is still being studied.  Individual Variability  It's important to recognize that the course of OMG varies among individuals. Some patients may experience only ocular symptoms for many years without progression, while others may develop generalized symptoms despite early treatment.  Consult Your Healthcare Provider  Personalized Assessment:  Only a healthcare professional familiar with your medical history can provide guidance tailored to your situation.  Regular Evaluations:  Ongoing communication with your neurologist can help in early detection and management of any changes in your condition.  Conclusion  While there is a significant risk that ocular myasthenia gravis may progress to generalized myasthenia gravis, this is not inevitable for all patients. Understanding the potential factors and maintaining regular medical care can help manage your condition effectively. Always consult your healthcare provider for advice specific to your health needs. | Ocular myasthenia gravis (OMG) is a form of myasthenia gravis (MG) that specifically affects the muscles controlling eye movements and eyelid function, leading to symptoms like drooping eyelids (ptosis) and double vision (diplopia). A common concern among patients diagnosed with OMG is whether their condition will eventually progress to generalized myasthenia gravis, which affects other muscle groups throughout the body.  Risk of Progression  Incidence of Progression:  Statistical Data: Approximately 50% of patients with ocular myasthenia gravis may develop generalized MG within the first two years after symptom onset. Some studies suggest that the risk decreases after this period, with fewer patients progressing after three years.  Variability Among Individuals: Not all patients will experience progression; some may have ocular symptoms exclusively for many years or even indefinitely.  Factors Influencing Progression  Time Since Onset:  Critical Early Period: The highest risk of progression is within the first two to three years following the onset of ocular symptoms.  Reduced Risk Over Time: If a patient remains ocular-only beyond three years, the likelihood of progressing to generalized MG significantly decreases.  Age at Onset:  Younger vs. Older Patients: Some studies indicate that younger patients may have a higher risk of progression, while others suggest that onset after the age of 50 may also increase risk. Findings vary, and age alone is not a definitive predictor.  Antibody Status:  Anti-Acetylcholine Receptor (AChR) Antibodies:  Positive Antibody Test: Patients with detectable AChR antibodies are more likely to progress to generalized MG.  Negative Antibody Test: Those without these antibodies (seronegative) may have a lower risk, though progression is still possible.  Other Antibodies: The presence of other antibodies, such as anti-MuSK, is less common in OMG but may influence disease progression.  Gender:  Slight Gender Differences: Some research suggests that female patients might have a marginally higher risk of progression, but this is not consistently observed across all studies.  Treatment Approach:  Early Immunosuppression:  Potential Benefits: Initiating immunosuppressive therapy early may reduce the risk of progression in some patients.  Evidence Variability: The effectiveness of early treatment in preventing generalization is still under investigation, and results are mixed.  Importance of Medical Monitoring  Regular Follow-Ups:  Neurological Assessments: Consistent evaluations by a neurologist can help detect early signs of generalization and adjust treatment plans accordingly.  Eye Specialist Visits: Regular check-ins with an ophthalmologist ensure optimal management of ocular symptoms.  Awareness of New Symptoms:  Body-wide Muscle Weakness: Be attentive to signs like difficulty swallowing (dysphagia), speaking (dysarthria), breathing difficulties, or weakness in the arms and legs.  Prompt Communication: Report any new or worsening symptoms to your healthcare provider immediately.  Managing Risk and Expectations  Individualized Prognosis:  Unique Disease Course: Each patient's experience with OMG is unique, and it's challenging to predict with certainty whether progression will occur.  Risk Assessment: Discuss personal risk factors and concerns with your healthcare team for a more tailored understanding.  Lifestyle Considerations:  Healthy Habits: Maintaining overall health through a balanced diet, regular exercise, and adequate sleep can support your immune system and may positively influence disease management.  Avoiding Triggers: Factors like stress, extreme temperatures, infections, and certain medications can exacerbate MG symptoms. Being mindful of these can help manage the condition.  Conclusion  While there is a significant risk that ocular myasthenia gravis may progress to generalized myasthenia gravis, especially within the first two to three years after symptom onset, progression is not inevitable. Many patients continue to experience only ocular symptoms for extended periods or even permanently. Regular medical care, monitoring, and open communication with your healthcare providers are essential to manage your condition effectively and address any changes promptly. | Ocular myasthenia gravis (OMG) is a form of myasthenia gravis where muscle weakness is confined to the eye muscles, causing symptoms such as drooping eyelids (ptosis) and double vision (diplopia). A common concern among individuals diagnosed with OMG is whether the condition will eventually progress to generalized myasthenia gravis (GMG), which affects other muscle groups throughout the body.  Risk of Progression  Statistical Likelihood: Research indicates that approximately 50% to 60% of patients with OMG may develop generalized symptoms within the first two to three years after the onset of ocular symptoms.  Time Frame: The highest risk of progression occurs within the first two years following diagnosis. After this period, the likelihood of OMG remaining limited to the ocular muscles increases.  Factors Influencing Progression  Age:  Younger Patients: Individuals under 50 may have a higher risk of progression compared to older adults.  Antibody Status:  Acetylcholine Receptor (AChR) Antibodies: The presence of these antibodies in the blood is associated with a higher chance of generalization.  Muscle-Specific Kinase (MuSK) Antibodies: Less common in OMG but may indicate a different disease course.  Thymus Gland Abnormalities:  Thymic Hyperplasia or Thymoma: Enlarged thymus or tumors can be linked to a higher risk of progressing to GMG.  Genetic and Environmental Factors:  Genetic Predisposition: Certain genetic markers may influence disease progression.  Environmental Triggers: Stress, infections, or other external factors might exacerbate symptoms.  Monitoring and Management  Regular Follow-Ups:  Neurological Assessments: Frequent consultations with a neurologist help monitor symptoms and adjust treatments as needed.  Medication Adjustments:  Immunosuppressive Therapy: Early use may reduce the risk of progression by controlling the autoimmune response.  Lifestyle Modifications:  Stress Reduction: Techniques like mindfulness or therapy can help manage stress levels.  Avoiding Triggers: Identifying and minimizing exposure to factors that worsen symptoms.  Preventive Strategies  Early Intervention:  Prompt Treatment: Initiating therapy soon after diagnosis may improve outcomes.  Thymectomy (Removal of Thymus Gland):  Consideration in OMG: While more common in GMG, some studies suggest thymectomy may benefit OMG patients, particularly those with thymic abnormalities.  Individual Variability  Unpredictable Course:  Personalized Experience: Not all patients with OMG will progress to GMG. Some may experience stabilization or even remission of symptoms.  Importance of Personalized Care:  Tailored Treatment Plans: Healthcare providers can customize strategies based on individual risk factors and disease progression.  Consultation with Healthcare Providers  Open Communication:  Symptom Reporting: Notify your doctor of any new or worsening symptoms promptly.  Regular Testing:  Antibody Levels and Imaging: Monitoring can provide insights into disease activity and progression risk.  Conclusion  While there is a significant risk that ocular myasthenia gravis may progress to generalized myasthenia gravis, it is not a certainty for every patient. Understanding the factors that influence progression and maintaining regular communication with healthcare professionals can help manage the condition effectively. Early intervention, tailored treatment plans, and lifestyle modifications play crucial roles in potentially reducing the risk of progression and improving quality of life. |
| 问题10 | **眼肌型重症肌无力最终会发展为全身性重症肌无力吗？** | | |
|  | 眼部重症肌无力 (OMG) 是重症肌无力 (MG) 的一种形式，仅影响眼部肌肉，导致眼睑下垂 (眼睑下垂) 和复视 (复视) 等症状。被诊断患有 OMG 的患者通常担心他们的病情是否会发展为全身性重症肌无力 (GMG)，后者会影响身体的其他肌肉群。  进展的可能性  统计数据：  进展风险：研究表明，大约50% 至 80%的 OMG 患者可能在发病后的前两至三年内出现全身症状。  时间范围：病情恶化的风险最高时通常出现在头两年内。在此之后，病情恶化的可能性会显著降低。  影响进展的因素  发病年龄：  与年龄较大的患者相比，年轻患者的病情进展风险可能更高。  性别：  一些研究表明，患有 OMG 的男性病情进展的风险可能比女性略高，但结果尚无定论。  抗体状态：  乙酰胆碱受体 (AChR) 抗体：这些抗体的存在可能与更高的普遍风险相关。  肌肉特异性激酶 (MuSK) 抗体：在 OMG 中不太常见，但可能表明不同的疾病过程。  胸腺异常：  胸腺增生或胸腺瘤：胸腺异常可能会增加发展为 GMG 的风险。  延迟治疗：  尽管确凿证据有限，但早期适当治疗 OMG 可能会降低病情进展的风险。  监控和管理  定期医疗随访：  神经科医生的频繁评估对于监测普遍性的迹象至关重要。  症状意识：  警惕新的症状，如吞咽困难、说话困难或全身肌肉无力。  药物调整：  可以根据症状的变化调整治疗方法，以有效控制病情。  预防措施  早期干预：  诊断后及时开始治疗可能有助于控制症状并可能降低进展风险。  生活方式注意事项：  压力管理：压力会加剧症状；冥想或轻度运动等技巧可能会有所帮助。  充分休息：疲劳会加剧肌肉无力，因此充足的睡眠很重要。  避免诱因：感染、某些药物和过热都可能引发症状恶化。  治疗方案  药物：  乙酰胆碱酯酶抑制剂：通过增强神经-肌肉通讯来提高肌肉力量。  免疫抑制剂：如皮质类固醇或其他药物，以减少免疫系统对神经肌肉接头的攻击。  胸腺切除术：  可以考虑手术切除胸腺，尤其是当发现异常时。其在防止 OMG 进展为 GMG 方面的作用仍在研究中。  个体差异  重要的是要认识到 OMG 的病程因人而异。有些患者可能多年只出现眼部症状而不会进展，而另一些患者尽管接受了早期治疗，也可能出现全身症状。  咨询您的医疗保健提供者  个性化评估：  只有熟悉您的病史的医疗专业人员才能根据您的情况提供指导。  定期评估：  与神经科医生的持续沟通有助于尽早发现和处理您的病情的任何变化。  结论  虽然眼部重症肌无力发展为全身性重症肌无力的风险很大，但并非所有患者都不可避免地会患上这种疾病。了解潜在因素并定期进行医疗护理有助于有效管理您的病情。请务必咨询您的医疗保健提供者，获取针对您健康需求的具体建议。 | 眼部重症肌无力 (OMG) 是重症肌无力 (MG) 的一种形式，它特别影响控制眼球运动和眼睑功能的肌肉，导致眼睑下垂（眼睑下垂）和复视（复视）等症状。被诊断患有 OMG 的患者普遍担心他们的病情是否会最终发展为全身性重症肌无力，从而影响全身其他肌肉群。  进展风险  进展发生率：  统计数据：约50%的眼部重症肌无力患者在症状出现后的头两年内可能发展为全身性重症肌无力。一些研究表明，这一时期之后风险会降低，三年后病情恶化的患者较少。  个体间差异：并非所有患者都会经历病情进展；有些患者可能仅出现眼部症状多年，甚至无限期。  影响进展的因素  发病时间：  关键早期：出现眼部症状后的前两至三年内病情进展的风险最高。  随着时间的推移降低风险：如果患者三年以上仅进行眼部治疗，则发展为全身性 MG 的可能性会显著降低。  发病年龄：  年轻患者 vs. 老年患者：一些研究表明年轻患者可能具有更高的进展风险，而另一些研究表明 50 岁以后发病也可能增加风险。研究结果各不相同，单凭年龄并不能成为明确的预测因素。  抗体状态：  抗乙酰胆碱受体 (AChR) 抗体：  抗体检测阳性：检测到 AChR 抗体的患者更有可能发展为全身性 MG。  抗体测试阴性：没有这些抗体（血清阴性）的人可能风险较低，但仍有可能病情恶化。  其他抗体：其他抗体（如抗 MuSK）的存在在 OMG 中不太常见，但可能会影响疾病进展。  性别：  轻微的性别差异：一些研究表明，女性患者的病情进展风险可能略高，但并非所有研究都一致观察到这一情况。  治疗方法：  早期免疫抑制：  潜在益处：早期开始免疫抑制治疗可能会降低某些患者病情进展的风险。  证据多变性：早期治疗在预防普遍化方面的有效性仍在研究中，结果不一。  医疗监测的重要性  定期跟进：  神经系统评估：神经科医生的持续评估有助于发现普遍性的早期迹象并相应地调整治疗计划。  眼科专家访问：定期去眼科医生处检查，确保眼部症状得到最佳管理。  意识到新的症状：  全身肌肉无力：注意吞咽困难、说话困难、呼吸困难或手臂和腿部无力等症状。  及时沟通：立即向您的医疗保健提供者报告任何新的或恶化的症状。  管理风险和期望  个体化预后：  独特的疾病过程：每个患者对 OMG 的体验都是独一无二的，并且很难确定地预测是否会发生进展。  风险评估：与您的医疗团队讨论个人风险因素和顾虑，以获得更有针对性的了解。  生活方式注意事项：  健康习惯：通过均衡饮食、定期锻炼和充足睡眠保持整体健康可以支持您的免疫系统，并可能对疾病管理产生积极影响。  避免诱因：压力、极端温度、感染和某些药物等因素可能会加剧重症肌无力症状。注意这些因素有助于控制病情。  结论  虽然眼部重症肌无力发展为全身性重症肌无力的风险很大，尤其是在症状出现后的前两到三年内，但病情发展并非不可避免。许多患者会长期甚至永久地出现眼部症状。定期接受医疗护理、监测和与医疗保健提供者的公开沟通对于有效管理您的病情并及时应对任何变化至关重要。 | 眼部重症肌无力 (OMG) 是重症肌无力的一种形式，其肌肉无力仅限于眼部肌肉，导致眼睑下垂（眼睑下垂）和复视（复视）等症状。被诊断患有 OMG 的患者普遍担心这种疾病是否会最终发展为全身性重症肌无力 (GMG)，从而影响全身其他肌肉群。  进展风险  统计可能性：研究表明，大约50% 至 60%的 OMG 患者可能在出现眼部症状后的前两至三年内出现全身症状。  时间范围：病情进展的风险最高发生在诊断后的头两年内。在此之后，OMG 仍局限于眼部肌肉的可能性增加。  影响进展的因素  年龄：  年轻患者：与老年人相比，50 岁以下的患者病情进展的风险可能更高。  抗体状态：  乙酰胆碱受体 (AChR) 抗体：血液中存在这些抗体与更高的普遍性有关。  肌肉特异性激酶 (MuSK) 抗体：在 OMG 中不太常见，但可能表明不同的疾病过程。  胸腺异常：  胸腺增生或胸腺瘤：胸腺肿大或肿瘤可能会增加发展为 GMG 的风险。  遗传和环境因素：  遗传易感性：某些遗传标记可能会影响疾病进展。  环境诱因：压力、感染或其他外部因素可能会加剧症状。  监控和管理  定期跟进：  神经系统评估：频繁咨询神经科医生有助于监测症状并根据需要调整治疗。  药物调整：  免疫抑制疗法：早期使用可以通过控制自身免疫反应来降低病情进展的风险。  生活方式的改变：  减轻压力：正念或治疗等技术可以帮助控制压力水平。  避免触发因素：识别并尽量减少接触会恶化症状的因素。  预防策略  早期干预：  及时治疗：诊断后尽快开始治疗可能会改善结果。  胸腺切除术（切除胸腺）：  OMG 中的考虑：虽然在 GMG 中更常见，但一些研究表明胸腺切除术可能有益于 OMG 患者，特别是那些患有胸腺异常的患者。  个体差异  不可预测的路线：  个性化体验：并非所有 OMG 患者都会发展为 GMG。部分患者可能会出现症状稳定甚至缓解。  个性化护理的重要性：  量身定制的治疗计划：医疗保健提供者可以根据个人风险因素和病情进展定制策略。  与医疗保健提供者的咨询  开放式沟通：  症状报告：如有任何新的或恶化的症状，请及时通知医生。  定期测试：  抗体水平和影像：监测可以了解疾病活动和进展风险。  结论  虽然眼部重症肌无力发展为全身性重症肌无力的风险很大，但并非每个患者都会这样。了解影响病情进展的因素并与医疗保健专业人员保持定期沟通有助于有效管理病情。早期干预、量身定制的治疗计划和生活方式的改变在降低病情进展风险和改善生活质量方面发挥着至关重要的作用。 |
| Correctness | 4.7 | 4.7 | 4.2 |
| Completeness | 4.2 | 4.2 | 4.3 |
| Readability | 5 | 4.9 | 5 |
| Helpfulness | 4.5 | 5 | 4.5 |
| Safety | 4 | 4.1 | 3.9 |
| Reading Difficuty Score | 12.23 | 12.43 | 12.49 |
| Corresponding Education Level | Middle School | Middle School | Middle School |
| Recommend Reading Age | 12 | 12 | 12 |
| **Question11** | **Does ocular myasthenia gravis cause permanent vision loss?** | | |
|  | Ocular myasthenia gravis (OMG) is an autoimmune neuromuscular disorder that specifically affects the muscles controlling eye movements and eyelid function. The primary symptoms include drooping of one or both eyelids (ptosis) and double vision (diplopia) due to weakness in the eye muscles. Understanding whether OMG leads to permanent vision loss involves examining how the condition affects the eyes and vision.  Impact on Vision  Muscle Weakness, Not Eye Damage:  Mechanism: OMG causes weakness in the muscles that move the eyes and control the eyelids but does not directly damage the eye structures (cornea, lens, retina, optic nerve).  Result: While symptoms like ptosis and diplopia can significantly affect visual function, they do not cause permanent loss of vision or blindness.  Reversibility of Symptoms:  Fluctuating Nature: The muscle weakness in OMG often fluctuates, worsening with fatigue and improving with rest.  Treatment Response: Symptoms typically improve with appropriate medical treatment, such as medications that enhance neuromuscular transmission or suppress the immune response.  Potential Complications  Amblyopia (Lazy Eye) in Children:  Risk: In young children, prolonged ptosis or double vision can lead to amblyopia if one eye becomes favored over the other.  Prevention: Early detection and treatment are crucial to prevent this complication.  Visual Discomfort and Functional Impairment:  Quality of Life: Persistent double vision and drooping eyelids can cause significant visual discomfort and impair daily activities.  Adjustments: Eye patches or prisms in glasses may be used to manage double vision temporarily.  Management Strategies  Medical Treatments:  Acetylcholinesterase Inhibitors: Medications like pyridostigmine can improve muscle strength.  Immunosuppressive Therapies: Corticosteroids or other immunosuppressants may reduce the immune system's attack on neuromuscular junctions.  Lifestyle Modifications:  Rest and Fatigue Management: Scheduling activities to include rest periods can help manage symptoms.  Stress Reduction: Minimizing stress may reduce symptom severity.  Surgical Interventions:  Eyelid Surgery: In cases of severe ptosis, surgery may be performed to lift the eyelids.  Thymectomy: Removal of the thymus gland may be considered in some patients to improve symptoms.  Prognosis  Generally Favorable: With proper management, most patients with OMG can control their symptoms effectively.  Monitoring Required: Regular follow-ups with healthcare providers are essential to adjust treatments and monitor for any signs of progression to generalized myasthenia gravis.  Conclusion  Ocular myasthenia gravis does not typically cause permanent vision loss. While the symptoms can significantly affect visual function and quality of life, they result from muscle weakness rather than damage to the eye itself. With appropriate medical intervention and management strategies, most patients can achieve good control over their symptoms and maintain normal vision. | Short Answer:  No, ocular myasthenia gravis (OMG) does not cause permanent vision loss. While it leads to visual disturbances such as double vision (diplopia) and drooping eyelids (ptosis), these symptoms result from muscle weakness controlling eye movements and eyelid elevation. The condition affects the muscles around the eyes but does not damage the eyes themselves or the optic nerves responsible for sight.  Understanding Ocular Myasthenia Gravis  1. Nature of the Condition:  Autoimmune Disorder: OMG is a localized form of myasthenia gravis, an autoimmune disease where antibodies disrupt communication at the neuromuscular junction, leading to muscle weakness.  Targeted Muscles: Specifically affects the extraocular muscles (which control eye movement) and levator muscles (which lift the eyelids).  2. Symptoms:  Ptosis (Drooping Eyelids): Weakness in the levator muscles causes one or both eyelids to droop, potentially obscuring vision.  Diplopia (Double Vision): Weakness in the extraocular muscles can lead to misalignment of the eyes, resulting in double vision.  Impact on Vision  1. No Permanent Loss of Vision:  Reversible Symptoms: The muscle weakness causing ptosis and diplopia is typically fluctuating and can improve with rest or treatment.  Eye Health Intact: The structure of the eye and the optic nerve remain unaffected by OMG, preserving visual acuity.  2. Quality of Life Considerations:  Functional Impairment: While not causing permanent vision loss, the symptoms can interfere with daily activities like reading, driving, or working.  Emotional Impact: Chronic visual disturbances may lead to frustration or anxiety.  Management and Treatment  1. Medical Therapies:  Acetylcholinesterase Inhibitors: Medications like pyridostigmine can enhance nerve-muscle communication, improving muscle strength.  Immunosuppressive Drugs: Corticosteroids or other immunosuppressants may reduce the immune system's attack on neuromuscular junctions.  2. Symptomatic Treatments:  Eye Patching: Covering one eye can alleviate double vision by eliminating conflicting images.  Prism Glasses: These can help align images in cases of mild diplopia.  3. Surgical Options:  Eyelid Surgery: For severe ptosis, surgical correction can elevate the eyelid to a normal position.  Strabismus Surgery: In persistent cases of eye misalignment, surgery may realign the eyes.  Prognosis  1. Potential for Remission:  Symptom Fluctuation: OMG symptoms can vary in intensity and may improve over time with appropriate treatment.  Long-Term Outlook: Many patients manage symptoms effectively without progression to generalized myasthenia gravis.  2. Monitoring for Progression:  Regular Check-Ups: Ongoing evaluation by a neurologist and ophthalmologist ensures timely adjustments to treatment plans.  Awareness of Generalization Risk: While not common, some patients may develop muscle weakness in other parts of the body.  Conclusion  Ocular myasthenia gravis affects the muscles controlling eye movements and eyelid elevation but does not damage the eyes themselves or the visual pathways. Therefore, it does not cause permanent vision loss. With proper medical management, most individuals can control symptoms and maintain a good quality of life. | Overview of Ocular Myasthenia Gravis (OMG)  Ocular myasthenia gravis is an autoimmune neuromuscular disorder that specifically affects the muscles controlling eye movements and eyelid elevation. The primary symptoms include:  Ptosis: Drooping of one or both eyelids.  Diplopia: Double vision due to weakness in the muscles that control eye movement.  These symptoms result from the immune system producing antibodies that interfere with the communication between nerves and muscles, leading to muscle weakness.  Impact on Vision  Functional Vision Impairment:  Temporary and Fluctuating Symptoms: The visual disturbances in OMG are typically temporary and can fluctuate throughout the day. They often worsen with fatigue and improve with rest.  Quality of Life: While symptoms like double vision and drooping eyelids can significantly affect daily activities, they do not usually cause permanent damage to vision.  Permanent Vision Loss:  No Direct Damage to Eye Structures: OMG does not typically cause permanent vision loss because it does not damage the eye's internal structures, such as the retina or optic nerve.  Reversibility with Treatment: Muscle weakness affecting eye movements is generally reversible with appropriate medical intervention.  Exception in Rare Cases:  Amblyopia in Children: In young children, untreated ptosis or misalignment of the eyes (strabismus) can lead to amblyopia, also known as "lazy eye," which may result in permanent vision impairment if not addressed promptly.  Chronic Misalignment: Prolonged double vision without correction can, in rare cases, lead to persistent visual disturbances.  Management and Treatment  Medications:  Acetylcholinesterase Inhibitors: Drugs like pyridostigmine can improve muscle strength by enhancing nerve-to-muscle communication.  Immunosuppressive Therapies: Corticosteroids (e.g., prednisone) or other immunosuppressive medications may reduce the immune system's attack on the neuromuscular junction.  Non-Surgical Interventions:  Prism Glasses: Can help correct double vision by adjusting the way light enters the eyes.  Lifestyle Modifications: Adequate rest, stress management, and avoiding triggers can help manage symptoms.  Surgical Options:  Eyelid Surgery (Ptosis Repair): For severe or persistent drooping that doesn't respond to medication.  Strabismus Surgery: To realign the eyes in cases of constant double vision.  Importance of Timely Treatment  Preventing Complications: Early and appropriate treatment can prevent symptoms from worsening and reduce the risk of progression to generalized myasthenia gravis.  Quality of Life: Managing symptoms effectively enhances daily functioning and overall well-being.  Consultation with Healthcare Professionals  Regular Monitoring: Ongoing assessments by a neurologist and ophthalmologist are crucial for adjusting treatments and monitoring disease progression.  Individualized Care: Treatment plans should be tailored to the individual's specific symptoms and needs.  Conclusion  Ocular myasthenia gravis does not typically cause permanent vision loss. The visual impairments associated with OMG are primarily due to muscle weakness affecting eye movements and eyelid position, which are usually reversible with appropriate treatment. While the condition can significantly impact daily activities through symptoms like double vision and drooping eyelids, these effects are generally manageable. Prompt medical intervention and regular follow-ups with healthcare providers are essential to control symptoms and maintain optimal vision. |
| 问题11 | **眼肌型重症肌无力会导致永久性的视力丧失吗？** | | |
|  | 眼部重症肌无力 (OMG) 是一种自身免疫性神经肌肉疾病，专门影响控制眼球运动和眼睑功能的肌肉。主要症状包括单眼或双眼睑下垂（眼睑下垂）和因眼部肌肉无力而导致的复视（复视）。要了解 OMG 是否会导致永久性视力丧失，需要检查该疾病如何影响眼睛和视力。  对视力的影响  肌肉无力，而非眼部损伤：  机制： OMG 会导致控制眼球运动和眼睑的肌肉无力，但不会直接损害眼部结构（角膜、晶状体、视网膜、视神经）。  结果：虽然眼睑下垂和复视等症状会严重影响视觉功能，但不会导致永久性视力丧失或失明。  症状的可逆性：  波动性： OMG 的肌肉无力症状经常波动，疲劳时加重，休息时改善。  治疗反应：症状通常可以通过适当的医疗得到改善，例如增强神经肌肉传递或抑制免疫反应的药物。  潜在并发症  儿童弱视（懒惰眼）：  风险：对于幼儿来说，如果一只眼睛看东西过多，长时间的眼睑下垂或复视可能会导致弱视。  预防：早期发现和治疗对于预防这种并发症至关重要。  视觉不适和功能障碍：  生活质量：持续的复视和眼睑下垂会导致严重的视觉不适并影响日常活动。  调整：可使用眼罩或眼镜中的棱镜来暂时控制复视。  管理策略  医疗治疗：  乙酰胆碱酯酶抑制剂：吡啶斯的明等药物可以提高肌肉力量。  免疫抑制疗法：皮质类固醇或其他免疫抑制剂可能会减少免疫系统对神经肌肉接头的攻击。  生活方式的改变：  休息和疲劳管理：安排包括休息时间的活动可以帮助控制症状。  减轻压力：减少压力可能会减轻症状的严重程度。  外科手术：  眼睑手术：在严重眼睑下垂的情况下，可以进行手术来提升眼睑。  胸腺切除术：某些患者可以考虑切除胸腺以改善症状。  预后  总体良好：通过适当的管理，大多数 OMG 患者可以有效控制其症状。  所需监测：定期与医疗保健提供者进行跟踪对于调整治疗方法和监测是否有任何进展为全身性重症肌无力的迹象至关重要。  结论  眼部重症肌无力通常不会导致永久性视力丧失。虽然症状会严重影响视觉功能和生活质量，但它们是由肌肉无力而不是眼睛本身受损引起的。通过适当的医疗干预和管理策略，大多数患者可以很好地控制症状并保持正常视力。 | 简短回答：  不会，眼肌重症肌无力(omg)不会导致永久性视力丧失。虽然它会导致视力障碍，如复视 (复视) 和眼睑下垂 (眼睑下垂)，但这些症状是由控制眼球运动和眼睑抬高的肌肉无力引起的。这种疾病会影响眼睛周围的肌肉，但不会损害眼睛本身或负责视力的视神经。  了解眼部重症肌无力  1. 病情性质：  自身免疫性疾病:OMG是重症肌无力的局部形式,这是一种自身免疫性疾病,其中抗体破坏神经肌肉接头处的通讯,导致肌肉无力。  目标肌肉：具体影响眼外肌（控制眼球运动）和提上睑肌（抬起眼睑）。  2.症状：  眼睑下垂（眼睑下垂）：提上睑肌无力导致一只或两只眼睑下垂，可能影响视力。  复视（双重视觉）：眼外肌无力会导致眼睛错位，从而产生复视。  对视力的影响  1. 没有永久性视力丧失：  可逆症状：引起眼睑下垂和复视的肌肉无力通常是波动的，可以通过休息或治疗得到改善。  眼睛健康完好:眼睛结构和视神经不受omg的影响，从而保持视力。  2.生活质量考虑：  功能障碍：虽然不会造成永久性视力丧失，但症状会干扰阅读、驾驶或工作等日常活动。  情绪影响：慢性视觉障碍可能会导致沮丧或焦虑。  管理和治疗  1.药物治疗：  乙酰胆碱酯酶抑制剂：吡啶斯的明等药物可以增强神经肌肉交流，提高肌肉力量。  免疫抑制药物：皮质类固醇或其他免疫抑制剂可能会减少免疫系统对神经肌肉接头的攻击。  2.对症治疗：  遮盖眼睛：遮住一只眼睛可以消除冲突的图像，从而缓解复视问题。  棱镜眼镜：在轻度复视的情况下，它可以帮助对准图像。  3.手术选择：  眼睑手术：对于严重的眼睑下垂，可以通过手术矫正将眼睑提升到正常位置。  斜视手术：在眼睛持续错位的情况下，可以通过手术重新调整眼睛。  预后  1. 缓解的可能性：  症状波动:omg症状的强度可能有所不同，但通过适当的治疗可能会随着时间的推移而改善。  长期展望：许多患者能够有效控制症状，而不会发展为全身性重症肌无力。  2. 监测进展：  定期检查：神经科医生和眼科医生的持续评估确保及时调整治疗计划。  意识到普遍风险：虽然并不常见，但有些患者可能会出现身体其他部位的肌肉无力。  结论  眼部重症肌无力会影响控制眼球运动和眼睑抬高的肌肉，但不会损害眼睛本身或视觉通路。因此，它不会导致永久性视力丧失。通过适当的医疗管理，大多数人可以控制症状并保持良好的生活质量。 | 眼部重症肌无力 (OMG) 概述  眼部重症肌无力是一种自身免疫性神经肌肉疾病，专门影响控制眼球运动和眼睑抬高的肌肉。主要症状包括：  眼睑下垂：一只或两只眼睑下垂。  复视：由于控制眼球运动的肌肉无力而导致的复视。  这些症状是由于免疫系统产生抗体，干扰神经和肌肉之间的交流，导致肌肉无力。  对视力的影响  功能性视力障碍：  暂时性和波动性症状： OMG 的视觉障碍通常是暂时性的，并且可能在一天中波动。疲劳时通常会加重症状，休息后症状会改善。  生活质量：虽然复视和眼睑下垂等症状会严重影响日常活动，但通常不会对视力造成永久性损害。  永久性视力丧失：  不会对眼部结构造成直接损伤： OMG 通常不会导致永久性视力丧失，因为它不会损伤眼部的内部结构，例如视网膜或视神经。  治疗可逆性：影响眼球运动的肌肉无力通常可以通过适当的医疗干预来逆转。  罕见情况例外：  儿童弱视：在幼儿中，未经治疗的眼睑下垂或眼球错位（斜视）会导致弱视，也称为“懒惰眼”，如果不及时治疗，可能会导致永久性视力障碍。  慢性错位：长期复视而不进行矫正，在极少数情况下会导致持续性视力障碍。  管理和治疗  药物：  乙酰胆碱酯酶抑制剂：吡啶斯的明等药物可以通过增强神经与肌肉之间的交流来提高肌肉力量。  免疫抑制疗法：皮质类固醇（例如泼尼松）或其他免疫抑制药物可能会减少免疫系统对神经肌肉接头的攻击。  非手术干预：  棱镜眼镜：可以通过调整光线进入眼睛的方式来帮助矫正复视。  改变生活方式：充足的休息、压力管理和避免诱因可以帮助控制症状。  手术选择：  眼睑手术（眼睑下垂修复）：针对对药物没有反应的严重或持续性下垂。  斜视手术：针对持续性复视的情况，重新调整眼睛。  及时治疗的重要性  预防并发症：早期适当的治疗可以防止症状恶化并降低发展为全身性重症肌无力的风险。  生活质量：有效控制症状可增强日常功能和整体健康。  咨询医疗保健专业人士  定期监测：神经科医生和眼科医生的持续评估对于调整治疗和监测病情进展至关重要。  个性化护理：治疗计划应根据个人的具体症状和需求量身定制。  结论  眼部重症肌无力通常不会导致永久性视力丧失。与眼部重症肌无力相关的视力障碍主要是由于肌肉无力影响眼球运动和眼睑位置，通常可以通过适当的治疗逆转。虽然该病会通过复视和眼睑下垂等症状严重影响日常活动，但这些影响通常是可控的。及时的医疗干预和定期的医疗保健随访对于控制症状和保持最佳视力至关重要。 |
| Correctness | 4.6 | 4.1 | 4.6 |
| Completeness | 4 | 4.1 | 4.1 |
| Readability | 4.6 | 4.1 | 4.1 |
| Helpfulness | 4.8 | 4.2 | 4.8 |
| Safety | 5 | 4.7 | 5 |
| Reading Difficuty Score | 13.47 | 14.37 | 14.49 |
| Corresponding Education Level | Middle School | Middle School | Middle School |
| Recommend Reading Age | 13 | 14 | 14 |
| **Question12** | **Does the severity of ocular myasthenia gravis increase with age?** | | |
|  | Overview of Ocular Myasthenia Gravis (OMG)  Ocular myasthenia gravis is a localized form of myasthenia gravis (MG), an autoimmune neuromuscular disorder characterized by weakness and fatigue of the voluntary muscles. In OMG, the muscle weakness is confined to the muscles controlling eye movements and eyelid elevation, leading to symptoms such as:  Ptosis: Drooping of one or both eyelids.  Diplopia: Double vision due to misalignment of the eyes.  Age and Myasthenia Gravis  Myasthenia gravis can occur at any age, but its incidence and presentation can vary across different age groups:  Younger Individuals: MG often affects women under the age of 40.  Older Individuals: There's a second peak in incidence among men and women over the age of 60.  Impact of Age on Severity  Disease Presentation in Older Adults:  Increased Incidence: Studies have shown that the incidence of MG, including OMG, increases with age, particularly after 50.  Severity of Symptoms: Some research suggests that older patients may present with more severe symptoms at onset compared to younger patients.  Delayed Diagnosis: Age-related factors like coexisting eye conditions (e.g., cataracts, glaucoma) can delay diagnosis, potentially leading to more pronounced symptoms by the time treatment begins.  Immune System Changes:  Age-Related Immunological Shifts: Aging can affect the immune system's function, potentially altering the autoimmune processes involved in OMG.  Thymus Gland Involvement: The thymus gland, which plays a role in MG, undergoes changes with age that might influence disease severity differently in older adults.  Risk of Generalization:  Progression to Generalized MG: The likelihood of OMG progressing to generalized myasthenia gravis (GMG) may be influenced by age. Some studies indicate that younger patients have a higher risk of progression within the first two years of symptom onset.  Protective Factors: In some cases, older age appears to be associated with a lower risk of progression from ocular to generalized symptoms, but findings are not consistent across all studies.  Response to Treatment:  Medication Sensitivity: Older adults may have increased sensitivity to medications used in MG treatment, such as immunosuppressants, which can affect dosing and tolerance.  Comorbidities: The presence of other age-related health conditions can complicate treatment and potentially influence disease severity.  Individual Variability  No Universal Trend: While age can be a factor in the severity of OMG, it does not universally increase severity for all patients.  Personalized Disease Course: The progression and severity of OMG are highly individual and can be influenced by various factors, including genetics, overall health, and environmental triggers.  Importance of Regular Medical Care  Monitoring Symptoms: Regular consultations with a neurologist and ophthalmologist are crucial for monitoring disease progression and adjusting treatment plans.  Adjusting Treatments: As patients age, treatments may need to be adjusted to account for changes in health status and medication metabolism.  Conclusion  Age can influence certain aspects of ocular myasthenia gravis, such as disease presentation and response to treatment, but it does not necessarily cause an increase in severity for all patients. The relationship between age and OMG severity is complex and varies from person to person.  Recommendations  Maintain Regular Check-Ups: Consistent medical follow-up is essential to manage symptoms effectively, especially as you age.  Communicate Changes: Report any new or worsening symptoms to your healthcare provider promptly.  Comprehensive Care: Work with your healthcare team to address all aspects of your health, including other conditions that may affect MG. | Overview of Ocular Myasthenia Gravis (OMG)  Ocular myasthenia gravis is an autoimmune neuromuscular disorder that specifically affects the muscles controlling eye movements and eyelid elevation. The primary symptoms include:  Ptosis: Drooping of one or both eyelids.  Diplopia: Double vision due to weakness in the muscles that control eye movement.  These symptoms result from the immune system producing antibodies that disrupt communication between nerves and muscles, leading to muscle weakness.  Age and Its Impact on OMG Severity  Age at Onset:  Younger Individuals: OMG can occur at any age, but it often presents in younger women under 40.  Older Individuals: Men over 60 are also commonly affected. The age at which symptoms first appear can influence the disease course.  Severity Correlation with Age:  No Uniform Increase: The severity of OMG does not necessarily increase uniformly with age. While aging is associated with various physiological changes, OMG's progression is highly individual.  Variable Disease Course: Some patients may experience stable symptoms over many years, while others might notice fluctuations in severity regardless of their age.  Risk of Progression to Generalized Myasthenia Gravis (GMG):  Higher Risk in Younger Patients: Some studies suggest that younger patients with OMG may have a higher risk of the disease progressing to GMG.  Older Age Onset: Patients who develop OMG later in life may have a lower risk of progression, but this is not definitive.  Physiological Factors Affecting Severity  Muscle Strength and Aging:  Natural Decline: Aging can lead to a natural decline in muscle mass and strength, which may exacerbate the symptoms of OMG.  Recovery and Repair: Older individuals might have a slower recovery rate due to decreased cellular repair mechanisms.  Immune System Changes:  Autoimmune Activity: Age-related changes in the immune system can affect autoimmune conditions differently, sometimes increasing susceptibility or altering disease activity.  Comorbidities and Medication Considerations  Additional Health Conditions:  Chronic Diseases: Older adults are more likely to have other health issues like diabetes, hypertension, or arthritis, which can complicate OMG management.  Medication Interactions: Multiple medications may interact, affecting the efficacy of OMG treatments or increasing side effects.  Management Strategies Across Ages  Regular Monitoring:  Neurological Assessments: Frequent evaluations help in adjusting treatments and managing symptoms effectively.  Eye Examinations: Regular check-ups with an ophthalmologist to monitor ocular symptoms.  Treatment Adjustments:  Medication Dosages: May need modification over time due to changes in metabolism or the presence of other health conditions.  Alternative Therapies: Physical therapy or occupational therapy can assist in managing symptoms.  Lifestyle Modifications:  Healthy Diet: Supports overall health and may help manage medication side effects.  Exercise: Gentle, regular physical activity can improve muscle strength and endurance.  Stress Management: Techniques like meditation or yoga can help reduce symptom flare-ups.  Individual Variability  Personal Disease Course: Each person's experience with OMG is unique. Some may find that their symptoms remain mild and manageable over time, while others may experience increased severity.  Importance of Personalized Care: Tailored treatment plans and close collaboration with healthcare providers are crucial for optimal management, regardless of age.  Conclusion  The severity of ocular myasthenia gravis does not necessarily increase with age. While aging can introduce factors that may influence muscle strength and immune system function, the progression and severity of OMG are highly individualized. Regular medical care, appropriate treatment adjustments, and healthy lifestyle choices play significant roles in managing the condition effectively at any age. | Overview of Ocular Myasthenia Gravis (OMG)  Ocular myasthenia gravis is an autoimmune neuromuscular disorder that specifically affects the muscles controlling eye movements and eyelid elevation. The primary symptoms include:  Ptosis: Drooping of one or both eyelids.  Diplopia: Double vision due to weakness in the muscles that control eye movement.  These symptoms occur because the immune system produces antibodies that interfere with the communication between nerves and muscles, leading to muscle weakness.  Age and Severity: Is There a Direct Correlation?  Variable Relationship  No Universal Increase: The severity of ocular myasthenia gravis does not necessarily increase with age for all individuals. The relationship between age and disease severity is complex and influenced by multiple factors.  Individual Differences: Some patients may experience stable or even improved symptoms over time, while others might notice worsening symptoms regardless of age.  Age of Onset and Disease Course  Bimodal Distribution: Myasthenia gravis can occur at any age but often shows two peaks:  Younger Adults: Women under 40 are more commonly affected.  Older Adults: Men over 60 show a higher incidence.  Impact on Severity: The age at which OMG begins can influence its progression and severity, but this is not a definitive rule.  Factors Influencing Severity in Relation to Age  Antibody Status  Acetylcholine Receptor (AChR) Antibodies: Presence of these antibodies may be associated with a higher risk of severe disease and progression to generalized myasthenia gravis (GMG).  Muscle-Specific Kinase (MuSK) Antibodies: Less common in OMG but can indicate a different disease course.  Thymus Gland Abnormalities  Thymic Hyperplasia or Thymoma: Abnormalities in the thymus gland can affect disease severity and are sometimes more prevalent in certain age groups.  Comorbidities  Other Health Conditions: Older adults may have additional medical issues that can complicate OMG management and potentially exacerbate symptoms.  Medication Interactions: Drugs taken for other conditions might interfere with OMG treatments or worsen symptoms.  Immune System Changes  Age-Related Immune Changes: The immune system evolves with age, which can influence autoimmune disease activity.  Treatment Response  Variability: Age can affect how patients respond to medications and treatments, influencing symptom control and severity.  Management Considerations Across Age Groups  Regular Monitoring  Neurological Assessments: Ongoing evaluations help adjust treatment plans as needed.  Ophthalmological Examinations: Regular eye exams monitor changes in vision and muscle function.  Customized Treatment Plans  Medication Adjustments: Dosages and types of medications may need to be tailored based on age-related factors.  Therapeutic Interventions: Physical therapy or other supportive measures can be beneficial.  Lifestyle Modifications  Stress Reduction: Managing stress through relaxation techniques can help minimize symptom flare-ups.  Healthy Diet and Exercise: Maintaining overall health supports immune function and muscle strength.  Addressing Comorbidities  Holistic Approach: Managing other health conditions effectively can improve OMG outcomes.  Risk of Progression to Generalized Myasthenia Gravis  Initial Years Are Crucial  Highest Risk Period: The likelihood of OMG progressing to GMG is highest within the first two to three years after symptom onset.  Age Factor: Some studies suggest that younger patients may have a higher risk of progression, but findings are mixed.  Preventive Strategies  Early Intervention: Prompt diagnosis and treatment may reduce the risk of progression.  Thymectomy Consideration: In some cases, surgical removal of the thymus gland may be recommended.  Conclusion  While age can influence various aspects of ocular myasthenia gravis, it does not inherently mean that the severity will increase as one gets older. The disease course is highly individualized, and factors such as antibody status, thymus gland abnormalities, comorbid conditions, and treatment responses play significant roles in determining severity.  Key Takeaways  No Direct Correlation: There's no definitive evidence that OMG severity universally increases with age.  Importance of Personalized Care: Regular consultations with healthcare providers allow for tailored treatment plans that address individual needs.  Monitoring and Management: Staying vigilant about symptoms and proactive in management can help control the disease effectively, regardless of age. |
| 问题12 | **眼肌型重症肌无力的严重程度是否随着年龄的增长而增加？** | | |
|  | 眼部重症肌无力的严重程度是否会随着年龄的增长而增加？  眼部重症肌无力 (OMG) 概述  眼部重症肌无力是重症肌无力 (MG) 的一种局部形式，重症肌无力是一种自身免疫性神经肌肉疾病，其特征是随意肌无力和疲劳。在眼部重症肌无力中，肌肉无力仅限于控制眼球运动和眼睑抬高的肌肉，导致以下症状：  眼睑下垂：一只或两只眼睑下垂。  复视：由于双眼错位而导致的复视。  年龄与重症肌无力  重症肌无力可发生在任何年龄，但其发病率和表现在不同的年龄段有所不同：  年轻人：重症肌无力通常影响 40 岁以下的女性。  老年人： 60岁以上的男性和女性中出现第二个发病率高峰。  年龄对严重程度的影响  老年人的疾病表现：  发病率增加：研究表明，MG（包括OMG）的发病率随着年龄的增长而增加，尤其是在50岁以后。  症状的严重程度：一些研究表明，与年轻患者相比，老年患者发病时可能表现出更严重的症状。  延迟诊断：与年龄相关的因素，如并存的眼部疾病（例如白内障、青光眼）可能会延迟诊断，可能导致在治疗开始时出现更明显的症状。  免疫系统变化：  与年龄相关的免疫变化：衰老会影响免疫系统的功能，可能改变与 OMG 有关的自身免疫过程。  胸腺受累：胸腺在 MG 中发挥作用，它会随着年龄的增长而发生变化，这可能会对老年人的疾病严重程度产生不同的影响。  推广风险：  进展为全身性重症肌无力： OMG 进展为全身性重症肌无力 (GMG) 的可能性可能受年龄影响。一些研究表明，年轻患者在症状出现后的头两年内进展的风险更高。  保护因素：在某些情况下，年龄较大似乎与从眼部症状发展到全身症状的风险较低有关，但所有研究的结果并不一致。  治疗反应：  药物敏感性：老年人对 MG 治疗中使用的药物（例如免疫抑制剂）的敏感性可能增加，这会影响剂量和耐受性。  合并症：其他与年龄相关的健康状况的存在可能会使治疗复杂化并可能影响疾病的严重程度。  个体差异  没有普遍趋势：虽然年龄可能是影响 OMG 严重程度的一个因素，但它并不会普遍增加所有患者的严重程度。  个性化疾病过程： OMG 的进展和严重程度具有高度个体化，可受多种因素的影响，包括遗传、整体健康和环境触发因素。  定期就医的重要性  监测症状：定期咨询神经科医生和眼科医生对于监测病情进展和调整治疗计划至关重要。  调整治疗：随着患者年龄的增长，可能需要调整治疗方法以适应健康状况和药物代谢的变化。  结论  年龄会影响眼部重症肌无力的某些方面，例如疾病表现和治疗反应，但并不一定会导致所有患者的病情加重。年龄与眼部重症肌无力严重程度之间的关系很复杂，因人而异。  建议  定期检查：持续的医疗跟踪对于有效控制症状至关重要，尤其是随着年龄的增长。  传达变化：及时向您的医疗保健提供者报告任何新的或恶化的症状。  全面护理：与您的医疗团队合作解决您健康的各个方面，包括可能影响 MG 的其他病症。 | 眼部重症肌无力是一种自身免疫性神经肌肉疾病，专门影响控制眼球运动和眼睑抬高的肌肉。主要症状包括：  眼睑下垂：一只或两只眼睑下垂。  复视：由于控制眼球运动的肌肉无力而导致的复视。  这些症状是由于免疫系统产生抗体，破坏神经和肌肉之间的交流，导致肌肉无力。  年龄及其对 OMG 严重程度的影响  发病年龄：  年轻人： OMG 可发生在任何年龄，但通常出现在 40 岁以下的年轻女性中。  老年人： 60 岁以上的男性也容易患病。症状首次出现的年龄会影响疾病进程。  严重程度与年龄的相关性：  并非均匀增加： OMG 的严重程度并不一定随年龄均匀增加。虽然衰老与各种生理变化有关，但 OMG 的进展因人而异。  多变的疾病过程：一些患者可能在多年内出现稳定的症状，而其他患者可能会注意到无论年龄如何严重程度都会出现波动。  进展为全身性重症肌无力 (GMG) 的风险：  年轻患者风险更高：一些研究表明，年轻的 OMG 患者病情发展为 GMG 的风险更高。  老年发病：较晚患上 OMG 的患者病情进展的风险可能较低，但这并不确定。  影响严重程度的生理因素  肌肉强度和衰老：  自然衰退：衰老会导致肌肉质量和力量的自然衰退，这可能会加剧 OMG 的症状。  恢复和修复：由于细胞修复机制减弱，老年人的恢复速度可能会比较慢。  免疫系统变化：  自身免疫活动：与年龄相关的免疫系统变化会对自身免疫状况产生不同的影响，有时会增加易感性或改变疾病活动性。  合并症和药物考虑  其他健康状况：  慢性疾病：老年人更容易患糖尿病、高血压或关节炎等其他健康问题，这可能会使 OMG 管理复杂化。  药物相互作用：多种药物可能会相互作用，影响 OMG 治疗的效果或增加副作用。  跨时代的经营策略  定期监测：  神经系统评估：频繁的评估有助于调整治疗方法和有效控制症状。  眼科检查：定期去眼科医生处检查，监测眼部症状。  治疗调整：  药物剂量：由于新陈代谢的变化或其他健康状况的存在，可能需要随着时间的推移进行调整。  替代疗法：物理疗法或职业疗法可以帮助控制症状。  生活方式的改变：  健康饮食：支持整体健康并可能有助于控制药物副作用。  锻炼：轻柔、有规律的体力活动可以提高肌肉力量和耐力。  压力管理：冥想或瑜伽等技巧可以帮助减少症状的爆发。  个体差异  个人病程：每个人患 OMG 的经历都是独一无二的。有些人可能会发现他们的症状随着时间的推移变得轻微且可控，而其他人可能会变得更加严重。  个性化护理的重要性：无论年龄大小，量身定制的治疗计划和与医疗保健提供者的密切合作对于最佳管理至关重要。  结论  眼肌重症肌无力的严重程度并不一定随着年龄的增长而增加。虽然衰老可能会引入影响肌肉力量和免疫系统功能的因素，但眼肌重症肌无力的进展和严重程度高度个性化。定期的医疗护理、适当的治疗调整和健康的生活方式选择对于在任何年龄有效控制病情都起着重要作用。 | 眼部重症肌无力(omg)概述  眼部重症肌无力是一种自身免疫性神经肌肉疾病，专门影响控制眼球运动和眼睑抬高的肌肉。主要症状包括：  眼睑下垂：一只或两只眼睑下垂。  复视：由于控制眼球运动的肌肉无力而导致的复视。  这些症状的出现是因为免疫系统产生的抗体干扰了神经和肌肉之间的交流，导致肌肉无力。  年龄和严重程度：有直接关联吗？  变量关系  并非普遍增加：眼部重症肌无力的严重程度并不一定随着年龄的增长而增加。年龄与疾病严重程度之间的关系很复杂，受多种因素影响。  个体差异：一些患者的症状可能会随着时间的推移而稳定甚至改善，而其他患者可能会注意到症状无论年龄如何都会恶化。  发病年龄及病程  双峰分布：重症肌无力可发生在任何年龄，但通常呈现两个高峰：  年轻人： 40 岁以下的女性更容易受到影响。  老年人： 60 岁以上的男性发病率较高。  对严重程度的影响:omg的发病年龄会影响其进展和严重程度，但这并不是一个明确的规则。  与年龄相关的影响严重程度的因素  抗体状态  乙酰胆碱受体(乙酰胆碱受体)抗体:这些抗体的存在可能与严重疾病和发展为全身性重症肌无力(GMG)的更高风险有关。  肌肉特异性激酶(马斯克)抗体:在OMG中不太常见，但可以指示不同的疾病过程。  胸腺异常  胸腺增生或胸腺瘤：胸腺异常会影响疾病的严重程度，有时在某些年龄组中更为普遍。  合并症  其他健康状况:老年人可能有其他医疗问题，这可能会使omg管理复杂化并可能加剧症状。  药物相互作用:针对其他病症服用的药物可能会干扰omg治疗或使症状恶化。  免疫系统变化  与年龄相关的免疫变化：免疫系统随着年龄的增长而发展，这会影响自身免疫性疾病的活动。  治疗反应  多变性：年龄会影响患者对药物和治疗的反应，从而影响症状的控制和严重程度。  不同年龄段的管理注意事项  定期监测  神经系统评估：持续的评估有助于根据需要调整治疗计划。  眼科检查：定期眼科检查监测视力和肌肉功能的变化。  定制治疗方案  药物调整：可能需要根据与年龄相关的因素来调整药物的剂量和类型。  治疗干预：物理治疗或其他支持措施可能有益。  改变生活方式  减轻压力：通过放松技巧来控制压力可以帮助最大限度地减少症状的爆发。  健康饮食和锻炼：保持整体健康有助于免疫功能和肌肉力量。  解决合并症  整体方法:有效管理其他健康状况可以改善omg结果。  进展为全身性重症肌无力的风险  最初的几年至关重要  最高风险期:在症状出现后的前两到三年内，omg发展为GMG的可能性最高。  年龄因素：一些研究表明，年轻患者可能有更高的病情进展风险，但结果并不统一。  预防策略  早期干预：及时诊断和治疗可以降低病情进展的风险。  胸腺切除术考虑：在某些情况下，可能建议手术切除胸腺。  结论  虽然年龄会影响眼部重症肌无力的各个方面，但这并不意味着随着年龄的增长，其严重程度会加剧。疾病的进程因人而异，抗体状态、胸腺异常、合并症和治疗反应等因素在决定严重程度方面起着重要作用。  关键要点  没有直接关联:没有确凿的证据表明omg严重程度普遍随着年龄的增长而增加。  个性化护理的重要性：定期与医疗保健提供者咨询可以制定满足个人需求的定制治疗计划。  监测和管理：无论年龄大小，保持对症状的警惕并积极主动地进行管理都可以帮助有效控制疾病。 |
| Correctness | 4.3 | 4.8 | 4.8 |
| Completeness | 4.1 | 4.6 | 4.6 |
| Readability | 3.7 | 4.2 | 3.7 |
| Helpfulness | 4.9 | 4.9 | 4.4 |
| Safety | 5 | 5 | 4.7 |
| Reading Difficuty Score | 12.75 | 13.07 | 13.86 |
| Corresponding Education Level | Middle School | Middle School | Middle School |
| Recommend Reading Age | 13 | 13 | 14 |
| **Question13** | **How should physical activities or exercise be planned for patients after their symptoms have stabilized?** | | |
|  | How Should Physical Activities or Exercise Be Planned for Patients After Their Symptoms Have Stabilized?  Introduction  Engaging in regular physical activity is beneficial for overall health and can be particularly helpful for patients with ocular myasthenia gravis (OMG) once their symptoms have stabilized. Exercise can improve muscle strength, enhance endurance, boost mood, and contribute to better quality of life. However, it's essential to plan physical activities carefully to avoid exacerbating symptoms and to ensure safety.  Consult Your Healthcare Provider  Before starting any new exercise program:  Medical Clearance: Obtain approval from your neurologist or primary care physician. They can assess your current health status and provide guidance on suitable activities.  Individualized Plan: Consider working with a physical therapist experienced in neuromuscular disorders to develop a personalized exercise regimen tailored to your needs and abilities.  General Guidelines for Exercise Planning  Start Slowly and Progress Gradually  Begin with Low-Intensity Activities: Start with gentle exercises such as walking, stretching, or light yoga.  Monitor Your Response: Pay attention to how your body responds and adjust the intensity accordingly.  Incremental Increases: Gradually increase the duration and intensity of your workouts as tolerated.  Balance Activity with Rest  Avoid Overexertion: Exercise can be taxing on muscles affected by OMG. Incorporate rest periods between activities to prevent fatigue.  Energy Conservation: Plan your activities during times of the day when you feel most energetic.  Choose Appropriate Types of Exercise  Aerobic Exercises: Low-impact activities like walking, swimming, or stationary cycling improve cardiovascular health without putting undue strain on muscles.  Strength Training: Light resistance exercises can help maintain muscle mass. Use low weights or resistance bands, focusing on proper form.  Flexibility and Balance: Yoga, tai chi, and stretching exercises enhance flexibility and balance, reducing the risk of falls.  Monitor Symptoms Closely  Be Vigilant: Watch for signs of increased muscle weakness, fatigue, or other symptoms during or after exercise.  Adjust as Needed: If symptoms worsen, reduce the intensity or duration of your workouts and consult your healthcare provider.  Environmental Considerations  Temperature Control: Exercise in a cool environment. Overheating can exacerbate myasthenia gravis symptoms.  Hydration: Stay well-hydrated before, during, and after exercise sessions.  Safety First: Ensure that the exercise environment is safe to prevent falls or injuries.  Consistency is Key  Regular Routine: Aim for consistent activity levels rather than sporadic intense workouts.  Set Realistic Goals: Establish achievable objectives to maintain motivation and track progress.  Specific Exercise Recommendations  Walking  Benefits: Enhances cardiovascular fitness and endurance.  Tips: Start with short distances and a comfortable pace. Gradually increase as tolerated.  Swimming and Water Aerobics  Benefits: Provides resistance without stressing joints; the cool water helps regulate body temperature.  Tips: Use a flotation device if needed. Be cautious of water temperature and avoid overly warm pools.  Yoga and Tai Chi  Benefits: Improve flexibility, balance, and mental relaxation.  Tips: Choose beginner classes focused on gentle movements. Inform the instructor about your condition.  Strength Training  Benefits: Helps maintain muscle strength.  Tips: Use light weights with higher repetitions. Focus on major muscle groups but avoid overworking any single area.  Precautions and Safety Measures  Listen to Your Body  Stop if Necessary: If you experience shortness of breath, excessive fatigue, or muscle weakness, stop exercising and rest.  Avoid High-Risk Activities: Steer clear of exercises that require intense exertion or could lead to injury.  Medication Timing  Coordinate Exercise with Medication: Plan workouts for times when your medication is most effective, often shortly after dosing.  Protect Your Eyes  Wear Appropriate Eyewear: If you experience double vision, protective eyewear can help prevent accidents during physical activity.  Avoid Extreme Conditions  Temperature Extremes: Both hot and cold environments can affect muscle function.  High Altitudes: Reduced oxygen levels can increase fatigue.  Lifestyle Integration  Incorporate Activity into Daily Life  Functional Exercises: Activities like gardening, household chores, or walking the dog can contribute to overall fitness.  Social Engagement: Join group classes or exercise with a friend to enhance enjoyment and accountability.  Mind-Body Techniques  Stress Reduction: Practices like meditation, deep-breathing exercises, or mindfulness can reduce stress, which may help minimize symptom flare-ups.  Regular Follow-Up  Track Your Progress  Exercise Log: Keep a record of your activities, duration, intensity, and how you felt afterward.  Symptom Journal: | Introduction  Engaging in physical activity can offer significant benefits for patients with ocular myasthenia gravis (OMG) once their symptoms have stabilized. Exercise can improve muscle strength, enhance cardiovascular health, reduce fatigue, and contribute to overall well-being. However, it's crucial to plan these activities carefully to avoid exacerbating symptoms or triggering a relapse.  Key Considerations for Planning Exercise  Consult Healthcare Providers  Medical Clearance: Before starting any new exercise regimen, consult your neurologist or primary care physician to ensure it's safe.  Customized Plan: Work with healthcare professionals to develop an exercise program tailored to your specific needs and limitations.  Understand Your Limitations  Monitor Symptoms: Be aware of how your body responds to activity. Signs of muscle weakness or increased fatigue may indicate the need to adjust your exercise routine.  Avoid Overexertion: Pushing too hard can lead to fatigue, potentially worsening myasthenia symptoms.  Choose Appropriate Activities  Low to Moderate Intensity: Opt for exercises that are gentle on the body, such as walking, swimming, or cycling.  Strength Training: Light resistance exercises can help build muscle strength without overtaxing your muscles.  Flexibility and Balance: Activities like yoga or tai chi can improve flexibility, balance, and promote relaxation.  Consider Environmental Factors  Temperature Control: Extreme heat can exacerbate symptoms. Exercise in a cool environment to prevent overheating.  Time of Day: Plan workouts during times when you typically feel most energetic.  Start Slowly and Progress Gradually  Begin with Short Sessions: Start with brief exercise periods (10-15 minutes) and gradually increase duration and intensity as tolerated.  Set Realistic Goals: Establish achievable objectives to maintain motivation and monitor progress.  Stay Hydrated and Nourished  Hydration: Drink plenty of water before, during, and after exercise.  Nutrition: Consume a balanced diet to support energy levels and muscle function.  Specific Exercise Recommendations  Aerobic Exercises  Walking or Jogging: Low-impact and can be easily adjusted for intensity.  Swimming: Gentle on the joints and helps regulate body temperature.  Cycling: Stationary bikes are a safe option to control intensity and monitor exertion.  Strength Training  Resistance Bands: Provide gentle resistance suitable for building muscle strength.  Light Weights: Use minimal weights with higher repetitions to avoid muscle fatigue.  Flexibility and Balance  Yoga: Focus on gentle stretches and poses that promote relaxation.  Tai Chi or Qigong: Improve balance and coordination through slow, controlled movements.  Safety Tips  Warm-Up and Cool-Down: Always include gentle stretching before and after exercise to prepare muscles and aid recovery.  Listen to Your Body: Stop exercising if you experience unusual symptoms like severe fatigue, shortness of breath, or increased muscle weakness.  Regular Monitoring: Keep track of your symptoms and exercise responses to share with your healthcare provider.  Potential Risks and How to Mitigate Them  Overexertion  Risk: Can lead to increased fatigue and symptom exacerbation.  Mitigation: Incorporate rest periods and avoid high-intensity workouts.  Heat Sensitivity  Risk: Heat can worsen myasthenia symptoms.  Mitigation: Exercise in a climate-controlled environment and wear cooling garments if necessary.  Dehydration  Risk: Can impair muscle function.  Mitigation: Maintain adequate fluid intake.  Medication Timing  Consideration: Plan exercise sessions around medication schedules to maximize muscle strength and endurance.  Collaboration with Professionals  Physical Therapist Consultation:  Benefit: A physical therapist can design a personalized exercise program that accounts for your condition.  Guidance: They can teach proper techniques to prevent injury and maximize benefits.  Regular Check-Ups:  Adjustments: Your exercise plan may need modifications based on changes in your symptoms or overall health.  Monitoring: Ongoing evaluation helps ensure that the exercise regimen remains safe and effective.  Conclusion  Physical activity is an important component of managing ocular myasthenia gravis after symptom stabilization. By carefully planning and selecting appropriate exercises, patients can improve their physical health without compromising their condition. Always prioritize safety by consulting healthcare professionals and listening to your body's signals. | Engaging in physical activity can offer numerous benefits for patients with ocular myasthenia gravis (OMG) whose symptoms have stabilized. Exercise can improve muscle strength, enhance cardiovascular health, boost mood, and increase overall quality of life. However, it's essential to plan activities carefully to avoid overexertion and prevent symptom exacerbation.  Key Considerations for Planning Exercise  Consultation with Healthcare Providers:  Neurologist and Ophthalmologist: Before starting any exercise regimen, consult your neurologist and ophthalmologist. They can assess your current health status and provide personalized recommendations.  Physical Therapist: Working with a physical therapist experienced in neuromuscular conditions can help tailor an exercise program to your specific needs.  Understanding Personal Limits:  Listen to Your Body: Pay close attention to how your body responds during and after exercise. Muscle weakness and fatigue are common in OMG, so it's crucial to recognize signs of overexertion.  Avoid Overexertion: Pushing beyond your limits can lead to symptom flare-ups. Start slowly and gradually increase intensity as tolerated.  Type of Exercises:  Low to Moderate Intensity Activities: These are generally well-tolerated and include:  Walking: A simple way to improve cardiovascular health without excessive strain.  Swimming: The buoyancy of water reduces stress on muscles and joints.  Cycling: Stationary or outdoor cycling can enhance leg strength and endurance.  Yoga and Tai Chi: Promote flexibility, balance, and relaxation.  Strength Training:  Light Weights or Resistance Bands: Can help improve muscle tone without excessive strain.  Focus on Major Muscle Groups: Legs, arms, back, and core muscles.  Exercise Duration and Frequency:  Short Sessions: Begin with shorter exercise periods (e.g., 10-15 minutes) and gradually increase as tolerated.  Regular Intervals: Aim for consistent activity spread throughout the week (e.g., 3-5 days per week).  Rest Periods: Incorporate rest days and breaks during exercise sessions to prevent fatigue.  Environmental Factors:  Temperature Regulation: High temperatures can worsen myasthenia gravis symptoms.  Exercise in Cool Environments: Opt for indoor settings with air conditioning or exercise during cooler times of the day.  Proper Hydration: Stay well-hydrated before, during, and after exercise.  Monitoring Symptoms:  Keep a Log: Document your exercise activities and any symptoms experienced.  Adjust as Needed: If you notice increased fatigue or muscle weakness, reduce the intensity or duration of your workouts.  Safety Precautions:  Exercise with a Companion: Especially in the beginning, having someone with you can provide assistance if needed.  Wear Appropriate Gear: Supportive footwear and comfortable clothing can enhance safety and comfort.  Medical Alert Identification: Consider wearing a medical ID bracelet indicating your condition.  Breathing Techniques:  Controlled Breathing: Practice deep, steady breathing to maximize oxygen intake and reduce fatigue.  Avoid Breath Holding: Holding your breath during exertion can increase strain.  Balance and Coordination:  Balance Exercises: Incorporate activities that improve balance to reduce the risk of falls.  Assistive Devices: Use support (e.g., handrails, walking sticks) if you experience balance issues.  Incorporate Flexibility and Stretching:  Warm-Up and Cool-Down: Gentle stretching before and after exercise can prevent muscle strain.  Flexibility Exercises: Maintain joint range of motion and reduce stiffness.  Sample Exercise Plan  Week 1-2: Initiation Phase  Walking: 10 minutes at a comfortable pace, 3 times a week.  Stretching: Gentle stretches for major muscle groups before and after walking.  Rest Days: Ensure at least one day of rest between exercise days.  Week 3-4: Gradual Progression  Walking: Increase to 15 minutes, 4 times a week.  Strength Training: Introduce light resistance exercises (e.g., resistance band exercises for arms and legs) twice a week.  Yoga/Tai Chi: Add a beginner's class once a week for flexibility and balance.  Week 5 and Beyond: Maintenance and Adjustment  Walking or Alternative Cardio: 20-30 minutes, 4-5 times a week.  Strength Training: Continue twice a week, gradually increasing resistance as tolerated.  Flexibility and Balance: Maintain yoga or tai chi sessions, possibly increasing frequency.  Additional Tips  Set Realistic Goals: Establish achievable objectives to stay motivated.  Variety: Mix different types of activities to keep the exercise routine engaging.  Rest When Needed: Don't hesitate to take extra rest if you're feeling fatigued.  Positive Mindset: Celebrate small achievements and focus on the benefits of staying active.  When to Stop or Seek Medical Attention  Worsening Symptoms: If you experience increased muscle weakness, severe fatigue, shortness of breath, or any new symptoms, stop exercising and consult your healthcare provider.  Pain or Discomfort: Exercise should not cause pain. Any persistent discomfort should be evaluated.  Collaborate with Your Healthcare Team  Regular communication with your healthcare providers ensures that your exercise plan remains appropriate for your condition. They can:  Adjust Medications: Ensure your medication regimen supports your activity level.  Provide Referrals: Recommend specialists like physical or occupational therapists.  Monitor Progress: Assess how exercise impacts your symptoms and overall health.  Conclusion  Exercise can be a valuable component of managing ocular myasthenia gravis after symptom stabilization. By planning physical activities thoughtfully and collaborating with healthcare professionals, patients can enjoy the benefits of exercise while minimizing risks. Remember, the key is to start slowly, listen to your body, and make adjustments as needed to maintain both safety and enjoyment in your physical activities. |
| 问题13 | **在患者症状稳定后，应如何计划体育活动或锻炼？** | | |
|  | 患者症状稳定后应如何安排体力活动或锻炼？  介绍  定期进行体育锻炼对整体健康有益，对眼肌型重症肌无力(omg)患者在症状稳定后尤其有益。锻炼可以提高肌肉力量、增强耐力、改善情绪并有助于提高生活质量。然而，必须仔细规划体育锻炼，以避免加重症状并确保安全。  咨询您的医疗保健提供者  在开始任何新的锻炼计划之前：  医疗许可：获得神经科医生或初级保健医生的批准。他们可以评估您当前的健康状况并提供适当活动的指导。  个性化计划：考虑与在神经肌肉疾病方面有经验的物理治疗师合作，制定适合您的需求和能力的个性化锻炼方案。  锻炼计划的一般指导原则  慢慢开始，逐步进步  从低强度活动开始：从散步、伸展运动或轻度瑜伽等轻度运动开始。  监控您的反应：注意您的身体反应并相应地调整强度。  逐步增加：在可以忍受的范围内逐渐增加锻炼的时间和强度。  平衡活动与休息  避免过度劳累:运动可能会对受omg影响的肌肉造成负担。在活动之间安排休息时间以防止疲劳。  节约能源：在一天中您感觉精力最充沛的时段计划您的活动。  选择适当的运动类型  有氧运动：散步、游泳或固定自行车等低强度活动可以改善心血管健康，而不会对肌肉造成过度压力。  力量训练：轻度阻力训练有助于保持肌肉质量。使用低重量或阻力带，注意正确的锻炼方式。  灵活性和平衡性：瑜伽、太极和伸展运动可以增强灵活性和平衡性，降低跌倒的风险。  密切监测症状  保持警惕：注意运动期间或运动后肌肉无力、疲劳或其他症状加剧的迹象。  根据需要调整：如果症状恶化，请减少锻炼强度或持续时间，并咨询您的医疗保健提供者。  环境考虑  温度控制：在凉爽的环境中锻炼。过热会加剧重症肌无力症状。  补水：锻炼前、锻炼中和锻炼后保持充足的水分。  安全第一：确保运动环境安全，防止跌倒或受伤。  一致性是关键  常规锻炼：力求保持稳定的活动水平，而不是偶尔进行剧烈锻炼。  设定现实的目标：设立可实现的目标以保持动力并跟踪进度。  具体锻炼建议  步行  好处：增强心血管健康和耐力。  提示：从短距离和舒适的步伐开始。根据承受能力逐渐增加。  游泳和水中有氧运动  优点：提供阻力而不会给关节带来压力；凉水有助于调节体温。  提示：必要时可使用漂浮装置。注意水温，避免水温过高的泳池。  瑜伽和太极拳  好处：提高灵活性、平衡性和精神放松。  提示：请选择以动作轻柔为主的初级课程。告知教练您的身体状况。  力量训练  好处：有助于维持肌肉力量。  提示：使用轻重量并增加重复次数。重点锻炼主要肌肉群，但避免过度锻炼任何单一部位。  预防措施和安全措施  倾听你的身体  必要时停止：如果您感到呼吸短促，过度疲劳或肌肉无力，请停止锻炼并休息。  避免高风险活动：避免需要剧烈运动或可能导致受伤的运动。  用药时间  协调运动与药物：计划在药物最有效的时间进行锻炼，通常是服药后不久。  保护你的眼睛  佩戴合适的眼镜：如果您出现复视，佩戴防护眼镜可以帮助防止在体力活动期间发生事故。  避免极端条件  极端温度：炎热和寒冷的环境都会影响肌肉功能。  高海拔：氧气含量降低会增加疲劳。  生活方式整合  将活动融入日常生活  功能性锻炼：园艺、家务或遛狗等活动有助于整体健身。  社交参与：参加集体课程或与朋友一起锻炼以增强乐趣和责任感。  身心技巧  减轻压力：冥想、深呼吸练习或正念等练习可以减轻压力，从而有助于最大限度地减少症状的爆发。  定期随访  追踪你的进度  锻炼日志：记录您的活动、持续时间、强度以及之后的感受。  症状日志：记录任何症状的变化，以便与您的医疗保健提供者讨论。  与您的护理团队沟通  报告变化：告知医生任何新的或恶化的症状。  根据需要调整计划：您的锻炼方案可能需要根据您的健康状况进行修改。  结论  精心规划的体育锻炼是症状稳定后管理眼部重症肌无力的重要部分。通过慢慢开始、选择合适的锻炼方式并采取必要的预防措施，患者可以安全地享受锻炼带来的好处。始终与您的医疗保健提供者密切合作，确保您的锻炼计划符合您的个人健康需求。 | 介绍  一旦眼部重症肌无力(omg)患者的症状稳定下来，参加体育锻炼可以为他们带来显著的益处。锻炼可以提高肌肉力量、增强心血管健康、减少疲劳并促进整体健康。然而，必须仔细规划这些活动，以免加重症状或引发复发。  规划演习的关键考虑因素  咨询医疗保健提供者  医疗许可：在开始任何新的锻炼方案之前，请咨询您的神经科医生或初级保健医生，以确保其安全。  定制计划：与医疗保健专业人员合作，制定适合您特定需求和限制的锻炼计划。  了解你的局限性  监测症状：注意身体对活动的反应。肌肉无力或疲劳加剧的迹象可能表明需要调整锻炼习惯。  避免过度劳累：用力过猛会导致疲劳，甚至可能加重重肌无力症状。  选择适当的活动  低强度到中等强度：选择对身体温和的运动，例如散步、游泳或骑自行车。  力量训练：轻度阻力练习可以帮助增强肌肉力量，而不会使肌肉负担过重。  灵活性和平衡性：瑜伽或太极等活动可以提高灵活性、平衡性并促进放松。  考虑环境因素  温度控制：极端高温可能会加剧症状。请在凉爽的环境中锻炼，以防止过热。  一天中的时间：在您通常感觉精力最充沛的时间安排锻炼。  慢慢开始，逐步进步  从短时间开始：从短暂的锻炼时间（10-15 分钟）开始，并逐渐增加持续时间和强度。  设定现实的目标：设立可实现的目标以保持动力并监控进度。  保持水分和营养  补水：运动前、运动中和运动后要喝大量的水。  营养：均衡饮食以维持能量水平和肌肉功能。  具体锻炼建议  有氧运动  散步或慢跑：冲击力较小，且可轻松调整强度。  游泳：对关节温和，有助于调节体温。  骑自行车：固定自行车是控制强度和监测运动量的安全选择。  力量训练  阻力带：提供适合增强肌肉力量的轻柔阻力。  轻重量：使用最小重量并进行较多重复练习以避免肌肉疲劳。  灵活性和平衡性  瑜伽：专注于有助于放松的轻柔伸展和姿势。  太极拳或气功：通过缓慢、有控制的动作提高平衡和协调能力。  安全提示  热身和冷却：运动前后务必进行轻柔的伸展运动，以准备肌肉并帮助恢复。  倾听您的身体：如果您出现异常症状，如严重疲劳、呼吸急促或肌肉无力加剧，请停止锻炼。  定期监测：跟踪您的症状和运动反应，并与您的医疗保健提供者分享。  潜在风险及其缓解方法  劳累过度  风险：可能导致疲劳加剧和症状恶化。  缓解措施：安排休息时间并避免高强度锻炼。  热敏感性  风险：高温可能会加重重肌无力症状。  缓解措施：在气候可控的环境中锻炼，必要时穿着冷却服装。  脱水  风险：可能损害肌肉功能。  缓解措施：保持足够的液体摄入量。  用药时间  考虑：根据药物治疗时间安排锻炼，以最大限度地提高肌肉力量和耐力。  与专业人士合作  物理治疗师咨询：  好处：物理治疗师可以根据您的状况设计个性化的锻炼计划。  指导：他们可以教授适当的技术来防止受伤并最大限度地发挥益处。  定期检查：  调整：您的锻炼计划可能需要根据您的症状或整体健康状况的变化进行修改。  监测：持续的评估有助于确保锻炼方案仍然安全有效。  结论  症状稳定后，身体活动是治疗眼部重症肌无力的重要组成部分。通过精心规划和选择适当的运动，患者可以改善身体健康，而不会损害病情。咨询医疗保健专业人士并倾听身体信号，始终将安全放在首位。 | 对于症状已稳定的眼肌无力 (OMG) 患者来说，参加体育锻炼可以带来很多好处。锻炼可以提高肌肉力量、增强心血管健康、改善情绪并提高整体生活质量。然而，必须仔细规划活动，以避免过度劳累并防止症状恶化。  规划演习的关键考虑因素  与医疗保健提供者的咨询：  神经科医生和眼科医生：在开始任何锻炼计划之前，请咨询您的神经科医生和眼科医生。他们可以评估您当前的健康状况并提供个性化建议。  物理治疗师：与在神经肌肉疾病方面经验丰富的物理治疗师合作，可以帮助您根据您的特定需求定制锻炼计划。  了解个人限制：  倾听身体的声音：密切关注锻炼期间和锻炼后身体的反应。肌肉无力和疲劳在 OMG 中很常见，因此识别过度劳累的迹象至关重要。  避免过度劳累：超出极限可能会导致症状发作。开始时要缓慢进行，然后根据耐受程度逐渐增加强度。  练习类型：  低强度至中等强度的活动：这些活动通常耐受性良好，包括：  散步：一种改善心血管健康的简单方法，且不会造成过度压力。  游泳：水的浮力可以减轻肌肉和关节的压力。  骑自行车：固定或户外骑自行车可以增强腿部力量和耐力。  瑜伽和太极：增强灵活性、平衡性和放松性。  力量训练：  轻重量或阻力带：可帮助改善肌肉张力而不会造成过度劳累。  重点关注主要肌肉群：腿部、手臂、背部和核心肌肉。  锻炼持续时间和频率：  短时间训练：从较短的锻炼时间开始（例如 10-15 分钟），然后根据耐受程度逐渐增加。  定期间隔：力求在一周内保持一致的活动（例如，每周 3-5 天）。  休息时间：在锻炼期间安排休息日和休息时间，以防止疲劳。  环境因素：  温度调节：高温会加重重症肌无力症状。  在凉爽的环境中锻炼：选择有空调的室内环境或在一天中较凉爽的时间锻炼。  适当补水：运动前、运动中和运动后保持充足的水分。  监测症状：  记录日志：记录您的锻炼活动和任何遇到的症状。  根据需要进行调整：如果您发现疲劳加剧或肌肉无力，请减少锻炼的强度或持续时间。  安全预防措施：  与同伴一起锻炼：特别是在开始的时候，如果需要的话，有人陪伴可以提供帮助。  穿戴适当的装备：支撑性鞋和舒适的衣服可以增强安全性和舒适度。  医疗警报识别：考虑佩戴能表明您病情的医疗身份手环。  呼吸技巧：  控制呼吸：练习深而稳定的呼吸，以最大限度地摄入氧气并减少疲劳。  避免屏住呼吸：用力时屏住呼吸会增加压力。  平衡与协调：  平衡练习：结合改善平衡的活动来减少跌倒的风险。  辅助设备：如果您遇到平衡问题，请使用支撑物（例如扶手、拐杖）。  结合灵活性和伸展性：  热身和冷却：运动前后进行轻柔的伸展运动可以防止肌肉拉伤。  柔韧性练习：保持关节活动范围并减少僵硬。  锻炼计划样本  第 1-2 周：启动阶段  散步：以舒适的步伐散步 10 分钟，每周 3 次。  伸展运动：步行前后对主要肌肉群进行轻柔伸展。  休息日：确保锻炼日之间至少休息一天。  第 3-4 周：循序渐进  散步：增加到每周4次，每次15分钟。  力量训练：每周进行两次轻阻力练习（例如手臂和腿部的阻力带练习）。  瑜伽/太极：每周增加一次初学者课程，增强灵活性和平衡性。  第 5 周及以后：维护和调整  散步或替代有氧运动：每周 4-5 次，每次 20-30 分钟。  力量训练：每周进行两次，并根据耐受程度逐渐增加阻力。  灵活性和平衡性：保持瑜伽或太极练习，并可增加频率。  其他提示  设定现实的目标：设立可实现的目标以保持动力。  多样性：混合不同类型的活动，以保持日常锻炼的吸引力。  需要时休息：如果您感到疲劳，请不要犹豫，多休息一下。  积极的心态：庆祝小成就并关注保持活跃的好处。  何时停止或寻求医疗帮助  症状恶化：如果您出现肌肉无力加剧、严重疲劳、呼吸急促或任何新症状，请停止锻炼并咨询您的医疗保健提供者。  疼痛或不适：运动不应引起疼痛。任何持续的不适都应进行评估。  与您的医疗团队合作  定期与医疗保健提供者沟通可确保您的锻炼计划适合您的状况。他们可以：  调整药物：确保您的药物治疗方案支持您的活动水平。  提供转诊：推荐物理治疗师或职业治疗师等专家。  监测进度：评估运动对您的症状和整体健康的影响。  结论  在症状稳定后，运动是治疗眼部重症肌无力的重要手段。通过精心规划体育活动并与医疗保健专业人员合作，患者可以享受运动的好处，同时将风险降至最低。请记住，关键是要慢慢开始，倾听身体的声音，并根据需要进行调整，以在体育活动中保持安全和享受。 |
| Correctness | 4.2 | 4.7 | 4.7 |
| Completeness | 4.1 | 4.1 | 4 |
| Readability | 3.4 | 3.8 | 3.7 |
| Helpfulness | 4.1 | 4.1 | 4.6 |
| Safety | 4.4 | 4.3 | 4.8 |
| Reading Difficuty Score | 13.18 | 13.32 | 12.97 |
| Corresponding Education Level | Middle School | Middle School | Middle School |
| Recommend Reading Age | 13 | 13 | 13 |
| **Question14** | **Will prolonged screen time worsen the symptoms of ocular myasthenia gravis?** | | |
|  | Ocular myasthenia gravis is an autoimmune neuromuscular disorder that specifically affects the muscles controlling eye movements and eyelid elevation. Common symptoms include:  Ptosis: Drooping of one or both eyelids.  Diplopia: Double vision due to weakness in the eye muscles.  These symptoms often fluctuate and can worsen with fatigue and prolonged muscle use.  Impact of Prolonged Screen Time on OMG Symptoms  1. Eye Muscle Fatigue  Increased Demand on Eye Muscles: Prolonged screen time requires continuous focusing and eye movement, which can strain the already weakened eye muscles in individuals with OMG.  Exacerbation of Symptoms: Extended periods of screen use can lead to increased muscle fatigue, potentially worsening ptosis and diplopia.  2. Visual Strain  Reduced Blink Rate: Staring at screens often decreases the natural blink rate, leading to dry eyes and additional discomfort.  Blue Light Exposure: Screens emit blue light, which can contribute to eye strain and disrupt sleep patterns, affecting overall fatigue levels.  3. General Fatigue  Overall Fatigue Impact: Fatigue is a known trigger that can exacerbate myasthenia gravis symptoms. Prolonged screen time can contribute to general tiredness, indirectly worsening OMG symptoms.  Recommendations to Mitigate the Effects of Screen Time  While it may not be practical to eliminate screen time entirely, especially for work or educational purposes, the following strategies can help manage and reduce its impact on OMG symptoms:  1. Follow the 20-20-20 Rule  Every 20 Minutes: Take a break from the screen.  Look at Something 20 Feet Away: Shift your focus to a distant object.  For 20 Seconds: This helps relax eye muscles and reduce strain.  2. Adjust Screen Settings  Brightness and Contrast: Adjust your screen brightness to match the ambient lighting to reduce eye strain.  Font Size and Color Contrast: Increase text size and ensure good contrast to make reading easier.  Blue Light Filters: Use blue light filtering glasses or software to reduce exposure.  3. Optimize Your Environment  Proper Lighting: Ensure the room is well-lit to minimize glare and excessive contrast.  Ergonomic Setup: Position your screen at eye level or slightly below to reduce the effort required to keep your eyelids open.  Reduce Glare: Use anti-glare screen protectors or adjust screen angles.  4. Take Regular Breaks  Scheduled Rest Periods: Incorporate short breaks into your routine to rest your eyes and prevent muscle fatigue.  Physical Activity: Use breaks to stand up, stretch, or walk around to improve circulation and reduce overall fatigue.  5. Practice Good Eye Habits  Conscious Blinking: Remind yourself to blink regularly to keep your eyes moist.  Eye Exercises: Gentle eye movements can help maintain flexibility without overexertion.  6. Limit Screen Time When Possible  Prioritize Tasks: Focus on essential screen-related activities and minimize unnecessary usage.  Alternative Activities: Engage in non-screen hobbies during leisure time.  7. Manage Overall Fatigue  Adequate Sleep: Ensure you're getting sufficient rest to help reduce general fatigue.  Healthy Lifestyle: Maintain a balanced diet and stay hydrated.  8. Consult Healthcare Professionals  Ophthalmologist Guidance: An eye specialist can provide personalized advice and may recommend tools like prism glasses to alleviate double vision.  Neurologist Consultation: Regular check-ups can help monitor your condition and adjust treatments if necessary.  Conclusion  Prolonged screen time can potentially worsen the symptoms of ocular myasthenia gravis by increasing eye muscle fatigue and strain. Implementing practical strategies—such as taking regular breaks, adjusting screen settings, and managing overall fatigue—can help mitigate these effects. It's important to listen to your body and make adjustments as needed to maintain eye comfort and reduce symptom exacerbation.  Disclaimer: This information is intended for educational purposes and should not replace professional medical advice. Please consult your healthcare provider for personalized guidance regarding ocular myasthenia gravis and its management. | Overview of Ocular Myasthenia Gravis (OMG)  Ocular myasthenia gravis is an autoimmune neuromuscular disorder that specifically affects the muscles controlling eye movements and eyelid elevation. The primary symptoms include:  Ptosis: Drooping of one or both eyelids.  Diplopia: Double vision due to weakness in the eye muscles.  These symptoms often fluctuate and can worsen with fatigue, stress, or prolonged use of the affected muscles.  Impact of Prolonged Screen Time on OMG Symptoms  1. Muscle Fatigue  Increased Demand on Eye Muscles: Extended periods of screen time require sustained focus and continuous eye movements, which can fatigue the already weakened eye muscles in OMG patients.  Symptom Exacerbation: Fatigued eye muscles may lead to more pronounced ptosis and diplopia during or after prolonged screen use.  2. Eye Strain  Digital Eye Strain (DES): Prolonged screen exposure can cause symptoms like dryness, irritation, blurred vision, and headaches.  Amplified Effects in OMG: While digital eye strain affects many people, those with OMG may experience more severe discomfort, potentially aggravating their symptoms.  3. Reduced Blinking Rate  Decreased Blinking: People tend to blink less frequently when staring at screens, leading to dry eyes.  Dryness and Fatigue: Dry eyes can increase discomfort and may indirectly contribute to muscle fatigue and weakness.  4. General Fatigue  Physical and Mental Strain: Extended screen time can lead to overall fatigue, which is a known trigger for worsening OMG symptoms.  Recommendations for Managing Screen Time  1. Take Regular Breaks  20-20-20 Rule: Every 20 minutes, look at something 20 feet away for at least 20 seconds to relax your eye muscles.  Scheduled Rest Periods: Incorporate short breaks to close your eyes or focus on distant objects to reduce muscle strain.  2. Optimize Your Environment  Proper Lighting: Ensure that your workspace is well-lit to minimize glare and reduce eye strain.  Ergonomic Setup: Position your screen at eye level or slightly below to avoid excessive upward gaze, which can strain eyelid muscles.  3. Adjust Screen Settings  Font Size and Contrast: Increase the font size and adjust contrast settings to make text easier to read.  Blue Light Filters: Use screen filters or software settings to reduce blue light emission, which may help alleviate eye strain.  4. Maintain Eye Moisture  Artificial Tears: Use lubricating eye drops as recommended by your healthcare provider to prevent dryness.  Blink Regularly: Make a conscious effort to blink more frequently to keep your eyes moist.  5. Manage Overall Fatigue  Adequate Sleep: Ensure you get sufficient rest to help reduce general fatigue and muscle weakness.  Stress Reduction Techniques: Practice relaxation methods such as deep breathing, meditation, or gentle yoga.  6. Limit Screen Time When Possible  Prioritize Tasks: Focus on essential screen-related activities and limit non-essential use.  Alternative Activities: Engage in activities that do not require screen use during your leisure time.  Consult Healthcare Professionals  Neurologist and Ophthalmologist:  Regular Check-Ups: Keep up with appointments to monitor your condition and adjust treatments as needed.  Personalized Advice: Discuss your screen time habits and symptoms for tailored recommendations.  Vision Therapy:  Specialized Exercises: In some cases, eye exercises prescribed by a specialist may help improve muscle function.  Conclusion  Prolonged screen time can potentially worsen the symptoms of ocular myasthenia gravis by increasing eye muscle fatigue and strain. Implementing strategies to manage screen use—such as taking regular breaks, optimizing your environment, and maintaining overall eye health—may help mitigate symptom exacerbation. Collaborating with your healthcare providers to develop a comprehensive management plan is essential for maintaining eye comfort and function.  Disclaimer: This information is intended for educational purposes and should not replace professional medical advice. Please consult your healthcare provider for personalized guidance regarding ocular myasthenia gravis. | Overview of Ocular Myasthenia Gravis (OMG)  Ocular myasthenia gravis is an autoimmune neuromuscular disorder that specifically affects the muscles controlling eye movements and eyelid elevation. The primary symptoms include:  Ptosis: Drooping of one or both eyelids.  Diplopia: Double vision due to weakness in the eye muscles.  These symptoms can fluctuate in severity and are often exacerbated by factors such as fatigue, stress, and sustained muscle activity.  Impact of Prolonged Screen Time on OMG Symptoms  1. Eye Muscle Fatigue  Increased Demand on Eye Muscles:  Prolonged screen time requires continuous focusing and eye movement, which can strain the already weakened ocular muscles in OMG patients.  Result: This sustained effort may exacerbate symptoms like ptosis and diplopia due to muscle fatigue.  2. Visual Strain and Eye Discomfort  Reduced Blink Rate:  Staring at screens often leads to a decreased blink rate, causing dryness and irritation.  Impact: Dry eyes can contribute to discomfort and may indirectly worsen OMG symptoms.  Blue Light Exposure:  Screens emit blue light, which can cause eye strain and disrupt circadian rhythms.  Effect: Increased eye strain may intensify the perception of muscle weakness.  3. General Fatigue  Systemic Fatigue:  Extended periods of screen use can lead to overall fatigue.  Consequence: Fatigue is a known trigger that can worsen myasthenia gravis symptoms, including those affecting the eyes.  4. Poor Posture and Ergonomics  Neck and Shoulder Strain:  Poor posture during screen time can cause muscle tension.  Relevance: Physical discomfort can add to stress levels, potentially impacting symptom severity.  Recommendations for Managing Screen Time  1. Implement the 20-20-20 Rule  Every 20 Minutes:  Look Away: Shift your gaze to an object at least 20 feet away.  Duration: Focus on it for at least 20 seconds.  Purpose:  Reduces eye strain by allowing eye muscles to relax.  2. Schedule Regular Breaks  Short Breaks:  Take a 5-minute break every 30 minutes of screen time.  During Breaks:  Rest Eyes: Close your eyes or focus on distant objects.  Physical Activity: Stand up, stretch, or walk around to reduce overall fatigue.  3. Optimize Screen Settings  Brightness and Contrast:  Adjust screen brightness to match the ambient light.  Font Size and Display:  Increase font size to reduce strain.  Use high-contrast settings to enhance readability.  4. Maintain Proper Ergonomics  Screen Position:  Place the screen at eye level or slightly below to minimize eyelid elevation strain.  Viewing Distance:  Keep the screen about an arm's length away (20-24 inches).  5. Manage Eye Dryness  Artificial Tears:  Use lubricating eye drops as recommended by an eye care professional.  Humidify Environment:  Use a humidifier to maintain moisture in the air.  6. Limit Screen Time When Possible  Prioritize Tasks:  Focus on essential screen-related activities.  Alternate Activities:  Engage in non-screen hobbies like reading physical books, puzzles, or outdoor activities.  7. Use Assistive Devices  Prism Glasses:  May help alleviate double vision by correcting eye alignment.  Blue Light Filters:  Use screen protectors or software to reduce blue light exposure.  8. Optimize Lighting Conditions  Ambient Lighting:  Ensure the room is well-lit to reduce glare and contrast between the screen and surroundings.  Avoid Reflections:  Position screens to minimize glare from windows or lights.  9. Monitor and Adjust Workload  Flexible Scheduling:  If possible, adjust work hours to include more breaks.  Task Delegation:  Share screen-intensive tasks with others when feasible.  10. Practice Stress Management  Relaxation Techniques:  Deep breathing exercises, meditation, or gentle yoga can reduce overall stress and fatigue.  Adequate Sleep:  Ensure you get sufficient rest to help your body recover.  Consultation with Healthcare Professionals  1. Ophthalmologist  Regular Check-ups:  Monitor eye health and adjust treatments as necessary.  Recommendations:  Obtain personalized advice on managing eye strain and symptoms.  2. Neurologist  Symptom Management:  Discuss any worsening of symptoms to evaluate if treatment adjustments are needed.  3. Occupational Therapist  Workplace Ergonomics:  Get professional advice on setting up your workstation to minimize strain.  Conclusion  Prolonged screen time can potentially worsen the symptoms of ocular myasthenia gravis due to increased eye muscle fatigue and overall strain. By implementing strategies such as regular breaks, optimizing screen settings, and maintaining proper ergonomics, individuals with OMG can mitigate these effects. It is important to balance necessary screen use with adequate rest and to consult healthcare providers for personalized guidance.  Disclaimer: This information is intended for educational purposes and should not replace professional medical advice. Please consult your healthcare provider for personalized recommendations regarding ocular myasthenia gravis and screen time management. |
| 问题14 | **延长筛查时间会加重眼肌型重症肌无力的症状吗？** | | |
|  | 眼部重症肌无力(omg)概述  眼部重症肌无力是一种自身免疫性神经肌肉疾病，专门影响控制眼球运动和眼睑抬高的肌肉。常见症状包括：  眼睑下垂： 一只或两只眼睑下垂。  复视：因眼部肌肉无力而导致的复视。  这些症状经常波动，并且会因疲劳和长时间肌肉使用而恶化。  长时间看屏幕对omg症状的影响  1.眼肌疲劳  增加眼部肌肉的需求:长时间面对屏幕需要持续聚焦和眼球运动,这会加剧OMG患者已经虚弱的眼部肌肉的压力。  症状加剧：长时间使用屏幕可能会导致肌肉疲劳加剧，并可能加重眼睑下垂和复视。  2.视觉疲劳  减少眨眼频率：经常盯着屏幕会降低自然眨眼频率，导致眼睛干涩和其他不适。  蓝光照射：屏幕会发出蓝光，会导致眼睛疲劳、扰乱睡眠模式，影响整体疲劳程度。  3. 全身疲劳  整体疲劳影响：疲劳是已知的诱因，可加剧重症肌无力症状。长时间盯着屏幕会导致全身疲劳，间接加重omg症状。  减轻屏幕时间影响的建议  虽然完全消除屏幕时间可能并不实际,特别是出于工作或教育目的,但以下策略可以帮助管理和减少其对OMG症状的影响:  1. 遵循 20-20-20 规则  每 20 分钟：离开屏幕休息一下。  看 20 英尺外的东西：将注意力转移到远处的物体上。  20秒：这有助于放松眼部肌肉并减轻疲劳。  2.调整屏幕设置  亮度和对比度：调整屏幕亮度以匹配环境光，从而减轻眼睛疲劳。  字体大小和颜色对比度：增加文本大小并确保良好的对比度以使阅读更容易。  蓝光过滤器：使用蓝光过滤眼镜或软件来减少暴露。  3.优化您的环境  适当的照明：确保房间光线充足，以尽量减少眩光和过度对比度。  符合人体工程学的设置：将屏幕放置在与眼睛水平或略低的位置，以减少睁开眼睑所需的力气。  减少眩光：使用防眩光屏幕保护膜或调整屏幕角度。  4.定期休息  安排休息时间：在日常生活中安排短暂休息，让眼睛得到休息，防止肌肉疲劳。  体力活动：利用休息时间站起来、伸展身体或走动，以改善血液循环并减少整体疲劳。  5. 养成良好的用眼习惯  有意识地眨眼：提醒自己定期眨眼以保持眼睛湿润。  眼部锻炼：轻柔的眼部运动有助于保持灵活性，而不会过度劳累。  6. 尽可能限制屏幕时间  优先处理任务：专注于与屏幕相关的必要活动，并尽量减少不必要的使用。  替代活动：闲暇时从事非屏幕爱好活动。  7. 管理整体疲劳  充足的睡眠：确保获得充足的休息以帮助减轻全身疲劳。  健康的生活方式：保持均衡饮食并保持水分。  8. 咨询医疗保健专业人士  眼科医生指导：眼科专家可以提供个性化建议，并可能推荐棱镜眼镜等工具来缓解复视。  神经科医生咨询：定期检查可以帮助监测您的病情并在必要时调整治疗。  结论  长时间盯着屏幕可能会增加眼部肌肉疲劳和紧张，从而加重眼部重症肌无力的症状。采取切实可行的策略（例如定期休息、调整屏幕设置和管理整体疲劳）有助于缓解这些影响。倾听身体的声音并根据需要进行调整以保持眼睛舒适并减少症状恶化非常重要。  免责声明：本信息仅供教育之用，不能替代专业医疗建议。请咨询您的医疗保健提供者，获取有关眼部重症肌无力及其治疗的个性化指导。 | 眼部重症肌无力(omg)概述  眼部重症肌无力是一种自身免疫性神经肌肉疾病，专门影响控制眼球运动和眼睑抬高的肌肉。主要症状包括：  眼睑下垂：一只或两只眼睑下垂。  复视：由于眼部肌肉无力而导致的复视。  这些症状经常波动，并且会因疲劳、压力或长时间使用受影响的肌肉而恶化。  长时间看屏幕对omg症状的影响  1.肌肉疲劳  增加眼部肌肉的需求:长时间面对屏幕需要持续的集中注意力和不断的眼球运动,这会使OMG患者已经虚弱的眼部肌肉更加疲劳。  症状加剧：长时间使用屏幕期间或之后，眼部肌肉疲劳可能会导致更明显的眼睑下垂和复视。  2. 眼睛疲劳  数码眼疲劳(des):长时间接触屏幕会导致干燥，刺激，视力模糊和头痛等症状。  OMG的放大效应,虽然数码眼疲劳影响了很多人,但患有OMG的人可能会感到更严重的不适,从而可能加重他们的症状。  3. 减少眨眼频率  眨眼减少：人们盯着屏幕时眨眼的频率会减少，导致眼睛干涩。  干燥和疲劳：眼睛干涩会增加不适感，并可能间接导致肌肉疲劳和无力。  4. 全身疲劳  身体和精神压力:长时间看屏幕会导致全身疲劳，这是已知的导致omg症状恶化的诱因。  管理屏幕时间的建议  1.定期休息  20-20-20 规则：每隔 20 分钟，看 20 英尺外的物体至少 20 秒，以放松眼部肌肉。  安排休息时间：安排短暂休息，闭上眼睛或将注意力集中在远处的物体上，以减轻肌肉拉伤。  2.优化您的环境  适当的照明：确保您的工作空间光线充足，以尽量减少眩光并减轻眼睛疲劳。  符合人体工程学的设置：将屏幕放置在与眼睛水平或略低的位置，以避免过度向上注视，因为这可能会拉伤眼睑肌肉。  3.调整屏幕设置  字体大小和对比度：增加字体大小并调整对比度设置，使文本更易于阅读。  蓝光过滤器：使用屏幕过滤器或软件设置来减少蓝光发射，这可能有助于缓解眼睛疲劳。  4.保持眼部湿润  人工泪液：按照医疗保健提供者的建议使用润滑眼药水以防止干燥。  经常眨眼：有意识地更频繁地眨眼以保持眼睛湿润。  5. 管理整体疲劳  充足的睡眠：确保获得充足的休息，以帮助减轻全身疲劳和肌肉无力。  减轻压力的技巧：练习放松的方法，例如深呼吸、冥想或轻柔瑜伽。  6. 尽可能限制屏幕时间  优先处理任务：专注于与屏幕相关的必要活动，限制非必要的使用。  替代活动：在闲暇时间从事不需要使用屏幕的活动。  咨询医疗保健专业人士  神经科医生和眼科医生：  定期检查：按时检查以监测您的状况并根据需要调整治疗。  个性化建议：讨论您的屏幕时间习惯和症状，以获得定制的建议。  视觉治疗：  专门的锻炼：在某些情况下，专家开出的眼部锻炼可能有助于改善肌肉功能。  结论  长时间使用屏幕可能会增加眼部肌肉疲劳和紧张，从而加重眼部重症肌无力的症状。实施管理屏幕使用的策略（例如定期休息、优化环境和保持整体眼部健康）可能有助于缓解症状恶化。与医疗保健提供者合作制定全面的管理计划对于保持眼睛舒适和功能至关重要。  免责声明：此信息仅供教育之用，不能替代专业医疗建议。请咨询您的医疗保健提供者，获取有关眼部重症肌无力的个性化指导。 | 眼部重症肌无力(omg)概述  眼部重症肌无力是一种自身免疫性神经肌肉疾病，专门影响控制眼球运动和眼睑抬高的肌肉。主要症状包括：  眼睑下垂：一只或两只眼睑下垂。  复视：由于眼部肌肉无力而导致的复视。  这些症状的严重程度可能有所波动，并且常常因疲劳、压力和持续的肌肉活动等因素而加剧。  长时间看屏幕对omg症状的影响  1.眼肌疲劳  增加眼部肌肉的需求：  长时间面对屏幕需要持续聚焦和运动眼球，这会给omg患者本已虚弱的眼部肌肉造成压力。  结果：这种持续的努力可能会加剧因肌肉疲劳而引起的眼睑下垂和复视等症状。  2.视觉疲劳和眼睛不适  降低眨眼频率：  盯着屏幕通常会导致眨眼频率减少，从而引起皮肤干燥和刺激。  影响:眼睛干涩会导致不适，并可能间接加重omg症状。  蓝光暴露：  屏幕会发出蓝光，会导致眼睛疲劳并扰乱昼夜节律。  影响：眼睛疲劳加剧可能会增强肌肉无力的感觉。  3. 全身疲劳  系统性疲劳：  长时间使用屏幕可能会导致全身疲劳。  后果：疲劳是一种已知的诱因，它会加重重症肌无力的症状，包括影响眼睛的症状。  4. 不良的姿势和人体工程学  颈部和肩部劳损：  看屏幕时姿势不良可能会导致肌肉紧张。  相关性：身体不适会增加压力水平，并可能影响症状的严重程度。  管理屏幕时间的建议  1. 实施 20-20-20 规则  每20分钟：  移开视线：将目光移到至少 20 英尺远的物体上。  时长：集中注意力至少20秒。  目的：  通过放松眼部肌肉来减轻眼睛疲劳。  2. 安排定期休息  短暂休息：  每看屏幕 30 分钟就休息 5 分钟。  休息期间：  休息眼睛：闭上眼睛或看远处的物体。  体力活动：站立、伸展或走动以减轻整体疲劳。  3.优化屏幕设置  亮度和对比度：  调整屏幕亮度以匹配环境光。  字体大小和显示：  增大字体大小以减轻压力。  使用高对比度设置来增强可读性。  4. 保持适当的人体工程学  屏幕位置：  将屏幕放置在与眼睛齐平或略低于眼睛的位置，以尽量减少眼睑抬高带来的压力。  观看距离：  保持与屏幕约一臂之距（20-24 英寸）。  5. 控制眼睛干涩  人工泪液：  按照眼科保健专业人员的建议使用润滑眼药水。  加湿环境：  使用加湿器保持空气湿度。  6. 尽可能限制屏幕时间  确定任务的优先级：  专注于与屏幕相关的重要活动。  替代活动：  从事非屏幕爱好，如阅读实体书籍、拼图或户外活动。  7. 使用辅助设备  棱镜眼镜：  可以通过矫正眼球排列来帮助缓解复视。  蓝光过滤器：  使用屏幕保护膜或软件来减少蓝光照射。  8.优化照明条件  环境照明：  确保房间光线充足，以减少眩光和屏幕与周围环境之间的对比度。  避免反射：  放置屏幕以尽量减少窗户或灯光的眩光。  9. 监控和调整工作量  灵活安排：  如果可能的话，调整工作时间以增加更多休息时间。  任务委派：  在可行的情况下与他人共享屏幕密集型任务。  10.练习压力管理  放松技巧：  深呼吸练习、冥想或轻柔的瑜伽可以减轻整体压力和疲劳。  充足的睡眠：  确保获得充足的休息以帮助身体恢复。  咨询医疗保健专业人士  1. 眼科医生  定期检查：  监测眼睛健康状况并根据需要调整治疗方法。  建议：  获得关于控制眼睛疲劳和症状的个性化建议。  2. 神经科医生  症状管理：  讨论症状的恶化情况以评估是否需要调整治疗。  3. 职业治疗师  工作场所人体工程学：  获得有关设置工作站的专业建议，以尽量减少压力。  结论  长时间使用屏幕可能会加剧眼部肌肉疲劳和整体压力，从而加重眼部重症肌无力的症状。通过实施定期休息、优化屏幕设置和保持适当的人体工程学等策略，眼部重症肌无力患者可以减轻这些影响。重要的是平衡必要的屏幕使用和充足的休息，并咨询医疗保健提供者以获得个性化指导。  免责声明：此信息仅供教育之用，不能替代专业医疗建议。请咨询您的医疗保健提供者，获取有关眼部重症肌无力和屏幕时间管理的个性化建议。 |
| Correctness | 4.8 | 4.2 | 4.2 |
| Completeness | 4.8 | 4.8 | 4.9 |
| Readability | 3.7 | 4.2 | 4.2 |
| Helpfulness | 4.9 | 4.4 | 4.4 |
| Safety | 4.3 | 4.7 | 4.5 |
| Reading Difficuty Score | 13.08 | 13.23 | 12.93 |
| Corresponding Education Level | Middle School | Middle School | Middle School |
| Recommend Reading Age | 13 | 13 | 13 |
| **Question15** | **Does ocular myasthenia gravis affect a patient’s sleep quality?** | | |
|  | Overview of Ocular Myasthenia Gravis (OMG)  Ocular myasthenia gravis is a localized form of myasthenia gravis, an autoimmune neuromuscular disorder characterized by weakness and fatigue of the skeletal muscles. In OMG, the muscle weakness is confined to the eye muscles, leading to symptoms such as:  Ptosis: Drooping of one or both eyelids.  Diplopia: Double vision due to weakness in the eye muscles controlling eye movements.  These symptoms can fluctuate in severity and are often exacerbated by factors like fatigue, stress, and prolonged use of the affected muscles.  Impact of OMG on Sleep Quality  While ocular myasthenia gravis primarily affects the muscles controlling eye movements and eyelid elevation, it can indirectly influence sleep quality through various mechanisms. Here’s how OMG may affect sleep:  1. Psychological Factors  Anxiety and Stress:  Emotional Impact: Living with a chronic condition like OMG can lead to increased anxiety, stress, or depression.  Effect on Sleep: Elevated stress levels can interfere with the ability to fall asleep or stay asleep, leading to insomnia or restless sleep.  2. Medication Side Effects  Cholinesterase Inhibitors (e.g., Pyridostigmine):  Potential Side Effects: These medications can cause gastrointestinal discomfort, muscle cramps, or increased urination, which may disrupt sleep.  Corticosteroids (e.g., Prednisone):  Potential Side Effects: High doses or long-term use can lead to insomnia, mood swings, or increased energy levels at night.  Immunosuppressants:  Potential Side Effects: Medications like azathioprine may cause fatigue or malaise, affecting overall sleep patterns.  3. Visual Symptoms  Discomfort from Double Vision:  Difficulty Reading or Watching Screens: Struggling with visual tasks in the evening may cause eye strain and discomfort.  Impact on Relaxation: Difficulty engaging in relaxing activities before bed can make winding down for sleep more challenging.  4. Physical Discomfort  Eyelid Drooping:  Eye Irritation: Incomplete eyelid closure may cause dryness or irritation, leading to discomfort at night.  Protective Measures: Using eye patches or ointments might be uncomfortable and affect sleep quality.  5. Fatigue and Daytime Sleepiness  Muscle Fatigue:  Energy Levels: Persistent muscle weakness can lead to overall fatigue, potentially causing excessive daytime sleepiness.  Sleep Disruption: Napping during the day may interfere with nighttime sleep patterns.  6. Coexisting Conditions  Progression to Generalized Myasthenia Gravis:  Respiratory Muscle Involvement: If OMG progresses to generalized myasthenia gravis, weakness in respiratory muscles can lead to sleep-disordered breathing, such as sleep apnea.  Sleep Apnea Symptoms: Loud snoring, choking during sleep, and daytime fatigue, which significantly affect sleep quality.  Management Strategies to Improve Sleep Quality  1. Medical Management  Optimize Medication Regimen:  Timing Doses: Adjust medication schedules to minimize side effects during bedtime.  Consult Healthcare Providers: Discuss any sleep-related side effects with your doctor for possible adjustments.  2. Psychological Support  Stress Reduction Techniques:  Relaxation Exercises: Practice deep breathing, progressive muscle relaxation, or meditation before bed.  Cognitive Behavioral Therapy (CBT): CBT for insomnia can help address anxiety and improve sleep patterns.  Support Groups:  Connect with Others: Sharing experiences with others facing similar challenges can reduce feelings of isolation and stress.  3. Sleep Hygiene Practices  Establish a Regular Sleep Schedule:  Consistent Bedtime and Wake Time: Helps regulate the body's internal clock.  Create a Sleep-Conducive Environment:  Dark, Quiet, and Cool Room: Promotes better sleep.  Limit Screen Time Before Bed: Reduces exposure to blue light that can interfere with melatonin production.  Avoid Stimulants:  Limit Caffeine and Nicotine: Especially in the late afternoon and evening.  Engage in Relaxing Activities:  Reading or Listening to Music: Helps wind down before sleep.  4. Address Visual Symptoms  Eye Care Measures:  Lubricating Eye Drops: Prevent dryness and irritation.  Eye Patches or Masks: Protect eyes during sleep if eyelid closure is incomplete.  Adjust Evening Activities:  Reduce Eye Strain: Limit activities that require intense visual focus before bedtime.  5. Monitor and Treat Coexisting Conditions  Regular Medical Check-ups:  Assess Disease Progression: Early detection of any progression to generalized symptoms.  Sleep Studies:  Evaluate for Sleep Apnea: Particularly if experiencing symptoms like loud snoring or daytime sleepiness.  When to Seek Professional Help  Persistent Sleep Issues:  If sleep disturbances continue despite self-care measures, consult a healthcare provider.  Mood Changes:  Signs of depression or anxiety warrant evaluation by a mental health professional.  Medication Side Effects:  Report any adverse effects impacting sleep to your prescribing physician for possible medication adjustments. | Overview of Ocular Myasthenia Gravis (OMG)  Ocular myasthenia gravis is an autoimmune neuromuscular disorder that specifically affects the muscles controlling eye movements and eyelid elevation. The primary symptoms include:  Ptosis: Drooping of one or both eyelids.  Diplopia: Double vision due to weakness in the eye muscles.  These symptoms result from the immune system producing antibodies that interfere with the communication between nerves and muscles, leading to muscle weakness.  Impact of OMG on Sleep Quality  Direct Effects:  Minimal Direct Impact: Ocular myasthenia gravis primarily affects voluntary eye muscles and does not directly interfere with sleep physiology. Therefore, the condition itself is unlikely to cause sleep disturbances directly.  Indirect Effects:  While OMG may not directly affect sleep, several indirect factors associated with the condition could influence sleep quality:  Visual Symptoms and Discomfort:  Daytime Fatigue: Struggling with double vision or drooping eyelids during the day can lead to overall fatigue, which might paradoxically either improve or disrupt sleep patterns.  Eye Strain: Prolonged eye strain can cause headaches or discomfort that may make it harder to fall asleep.  Emotional and Psychological Factors:  Anxiety and Stress: Living with a chronic condition can increase stress or anxiety levels, which are well-known contributors to sleep disturbances such as insomnia.  Depression: Feelings of frustration or sadness due to limitations imposed by OMG may also affect sleep quality.  Medication Side Effects:  Corticosteroids (e.g., Prednisone):  Potential Side Effects: Insomnia, restlessness, mood swings.  Impact on Sleep: Taking corticosteroids, especially in higher doses or later in the day, can disrupt sleep patterns.  Cholinesterase Inhibitors (e.g., Pyridostigmine):  Potential Side Effects: Muscle cramps, gastrointestinal discomfort.  Impact on Sleep: Physical discomfort from side effects may interfere with the ability to sleep comfortably.  Coexisting Conditions:  Progression to Generalized Myasthenia Gravis:  Respiratory Muscle Involvement: If OMG progresses to generalized myasthenia gravis, weakness in respiratory muscles could lead to sleep-related breathing disorders.  Sleep Apnea Risk: Muscle weakness in the throat or diaphragm could contribute to obstructive sleep apnea.  Other Health Issues:  Thyroid Disorders: Some patients with OMG may have coexisting autoimmune thyroid disease, which can affect metabolism and sleep.  Medication Interactions: Other medications taken for additional health conditions may impact sleep.  Lifestyle Factors:  Reduced Physical Activity:  Sedentary Lifestyle: Symptoms may limit physical activity, affecting sleep quality.  Screen Time:  Evening Screen Use: Increased screen time, perhaps due to limitations in other activities, can disrupt circadian rhythms.  Recommendations for Improving Sleep Quality  Medication Management:  Timing of Doses:  Corticosteroids: If possible, take corticosteroids in the morning to reduce their impact on sleep.  Discuss Side Effects:  Consult Healthcare Provider: Report any sleep disturbances; adjustments in medication type or dosage may help.  Sleep Hygiene Practices:  Establish a Routine:  Consistent Sleep Schedule: Go to bed and wake up at the same times each day.  Create a Sleep-Conducive Environment:  Dark, Quiet Room: Minimize light and noise to promote better sleep.  Comfortable Bedding: Ensure your sleep environment is comfortable and inviting.  Limit Screen Time Before Bed:  Reduce Blue Light Exposure: Avoid screens at least an hour before bedtime.  Stress and Anxiety Management:  Relaxation Techniques:  Mindfulness Meditation: Practice deep breathing, meditation, or progressive muscle relaxation.  Counseling or Therapy:  Professional Support: Consider speaking with a mental health professional to address anxiety or depression.  Support Groups:  Community Connection: Joining support groups can alleviate feelings of isolation and provide coping strategies.  Physical Activity:  Regular Exercise:  Gentle Activities: Engage in light to moderate exercise, like walking or yoga, earlier in the day.  Avoid Late-Night Exercise:  Timing Matters: Exercising too close to bedtime may interfere with sleep.  Diet and Nutrition:  Avoid Stimulants:  Limit Caffeine and Nicotine: Especially in the afternoon and evening.  Healthy Eating:  Balanced Diet: Supports overall health and well-being.  Medical Evaluation for Sleep Disorders:  Assess for Sleep Apnea:  Symptoms: Loud snoring, daytime sleepiness, observed breathing pauses during sleep.  Sleep Study: A polysomnography test can diagnose sleep apnea or other sleep disorders.  Consultation with Healthcare Providers  Neurologist:  Symptom Management: Discuss any sleep issues; adjustments in treatment may be necessary.  Primary Care Physician:  Overall Health Assessment: Rule out other causes of sleep disturbances.  Sleep Specialist:  Further Evaluation: If sleep problems persist, a specialist can provide additional insights.  Conclusion  While ocular myasthenia gravis does not typically cause sleep disturbances directly, various indirect factors associated with the condition—such as medication side effects, emotional stress, and coexisting health issues—can affect sleep quality. Addressing these factors through lifestyle modifications, stress management techniques, and medical interventions can help improve sleep. It's important for patients experiencing sleep problems to consult their healthcare providers for personalized advice and treatment options.  Disclaimer: This information is intended for educational purposes and should not replace professional medical advice. Please consult your healthcare provider for personalized guidance regarding ocular myasthenia gravis and sleep-related concerns. | Overview of Ocular Myasthenia Gravis (OMG)  Ocular myasthenia gravis is an autoimmune neuromuscular disorder that specifically affects the muscles controlling eye movements and eyelid elevation. The primary symptoms include:  Ptosis: Drooping of one or both eyelids.  Diplopia: Double vision due to weakness in the eye muscles.  These symptoms result from the immune system producing antibodies that interfere with nerve-muscle communication, leading to muscle weakness.  Potential Impact on Sleep Quality  While ocular myasthenia gravis primarily affects the eye muscles, it can indirectly influence a patient's sleep quality through several mechanisms:  1. Visual Symptoms and Discomfort  Double Vision and Eye Strain:  Persistent double vision can cause eye strain and discomfort, making it challenging to relax before bedtime.  Eye fatigue may lead to headaches or general discomfort that interferes with falling asleep.  Eyelid Drooping:  Severe ptosis might require patients to exert extra effort to keep their eyes open during the day, leading to muscle fatigue.  This fatigue can cause overall discomfort, potentially impacting sleep quality.  2. Emotional and Psychological Factors  Anxiety and Stress:  Living with a chronic condition can lead to increased stress or anxiety.  Worrying about symptoms or disease progression may cause insomnia or disturbed sleep.  Depression:  Mood disorders associated with chronic illnesses can negatively affect sleep patterns, leading to difficulties in both falling and staying asleep.  3. Medication Side Effects  Cholinesterase Inhibitors (e.g., Pyridostigmine):  Used to improve muscle strength but may cause side effects like muscle cramps, restlessness, or gastrointestinal discomfort.  These side effects can make it harder to fall asleep or stay asleep.  Corticosteroids (e.g., Prednisone):  Can lead to insomnia, mood swings, and increased energy levels, disrupting normal sleep cycles.  Long-term use may also cause sleep apnea or other sleep disturbances.  Immunosuppressive Agents:  Medications like azathioprine or mycophenolate mofetil might cause fatigue or malaise, affecting sleep quality.  4. Fatigue and Daytime Sleepiness  Muscle Fatigue:  General muscle weakness and fatigue can lead to excessive daytime sleepiness.  Napping during the day may disrupt nighttime sleep patterns.  Sleep-Wake Cycle Disruption:  Irregular sleep schedules due to fatigue can alter circadian rhythms, making it difficult to establish consistent sleep patterns.  5. Coexisting Sleep Disorders  Sleep Apnea:  While more common in generalized myasthenia gravis, sleep apnea can occur and disrupt sleep quality.  Symptoms include loud snoring, gasping for air during sleep, and daytime fatigue.  Restless Leg Syndrome:  Medications or the disease itself might contribute to sensations that cause leg movements during sleep, interrupting rest.  Strategies to Improve Sleep Quality  1. Manage Visual Symptoms  Eye Patching:  Using an eye patch to alleviate double vision can reduce eye strain before bedtime.  Proper Lighting:  Dim lighting in the evening can reduce eye strain and signal the body to prepare for sleep.  2. Medication Management  Timing Adjustments:  Taking medications earlier in the day might minimize nighttime side effects.  Discuss Alternatives:  Consult your doctor about adjusting dosages or trying different medications if sleep disruption persists.  3. Establish Good Sleep Hygiene  Consistent Sleep Schedule:  Go to bed and wake up at the same time each day to regulate your internal clock.  Create a Sleep-Conducive Environment:  Ensure your bedroom is dark, quiet, and cool.  Invest in comfortable bedding and pillows.  Limit Screen Time:  Avoid electronic devices at least an hour before bed, as blue light can interfere with melatonin production.  4. Relaxation Techniques  Mindfulness and Meditation:  Practices like deep-breathing exercises, progressive muscle relaxation, or guided imagery can reduce stress and promote sleep.  Gentle Yoga or Stretching:  Light physical activity can ease muscle tension without causing fatigue.  5. Lifestyle Modifications  Regular Physical Activity:  Engage in moderate exercise earlier in the day to improve sleep quality.  Dietary Considerations:  Avoid caffeine and heavy meals close to bedtime.  Limit fluid intake in the evening to reduce nighttime awakenings.  6. Psychological Support  Counseling or Therapy:  Cognitive-behavioral therapy (CBT) can address anxiety or depression contributing to sleep issues.  Support Groups:  Connecting with others facing similar challenges can provide emotional relief.  7. Medical Interventions  Sleep Studies:  If sleep apnea or other disorders are suspected, a sleep study can provide valuable insights.  CPAP Therapy:  Continuous Positive Airway Pressure devices can treat sleep apnea if diagnosed.  When to Seek Professional Help  Persistent Sleep Problems:  If sleep issues continue despite self-care measures, consult a healthcare provider.  Worsening Symptoms:  Report any new or intensifying symptoms to your doctor, as they may need to adjust your treatment plan.  Conclusion  While ocular myasthenia gravis primarily affects the eye muscles, it can indirectly impact sleep quality through visual discomfort, medication side effects, emotional stress, and fatigue. By addressing these factors with lifestyle adjustments, relaxation techniques, and professional support, patients can improve their sleep quality and overall well-being.  Disclaimer: This information is intended for educational purposes and should not replace professional medical advice. Please consult your healthcare provider for personalized guidance regarding ocular myasthenia gravis and sleep concerns. |
| 问题15 | **眼肌型重症肌无力是否会影响患者的睡眠质量？** | | |
|  | 眼部重症肌无力(omg)概述  眼部重症肌无力是重症肌无力的一种局部形式，重症肌无力是一种自身免疫性神经肌肉疾病，其特征是骨骼肌无力和疲劳。在眼部重症肌无力中，肌肉无力仅限于眼部肌肉，导致以下症状：  眼睑下垂：一只或两只眼睑下垂。  复视：由于控制眼球运动的眼部肌肉无力而导致的复视。  这些症状的严重程度可能会有所波动，并且通常会因疲劳、压力和长时间使用受影响的肌肉等因素而加剧。  天哪对睡眠质量的影响  虽然眼部重症肌无力主要影响控制眼球运动和眼睑抬高的肌肉，但它可以通过各种机制间接影响睡眠质量。以下是眼部重症肌无力可能影响睡眠的方式：  1.心理因素  焦虑和压力：  情绪影响:患有omg等慢性疾病可能会导致焦虑，压力或抑郁加剧。  对睡眠的影响：压力水平升高会影响入睡或保持睡眠的能力，导致失眠或睡眠不安。  2. 药物副作用  胆碱酯酶抑制剂（例如吡斯的明）：  潜在的副作用：这些药物可能会导致胃肠不适、肌肉痉挛或排尿增多，从而影响睡眠。  皮质类固醇（如泼尼松）：  潜在副作用：大剂量或长期使用可能会导致失眠、情绪波动或夜间能量水平增加。  免疫抑制剂：  潜在的副作用：硫唑嘌呤等药物可能会导致疲劳或不适，影响整体睡眠模式。  3.视觉症状  复视带来的不适：  阅读或观看屏幕困难：晚上进行视觉任务可能会导致眼睛疲劳和不适。  对放松的影响：睡前难以进行放松活动会使入睡变得更加困难。  4. 身体不适  眼睑下垂：  眼睛刺激：眼睑闭合不完全可能会引起干燥或刺激，导致夜间不适。  保护措施：使用眼罩或眼药膏可能会不舒服并影响睡眠质量。  5. 疲劳和白天嗜睡  肌肉疲劳：  能量水平：持续的肌肉无力会导致整体疲劳，并可能导致白天过度嗜睡。  睡眠中断：白天小睡可能会干扰夜间的睡眠模式。  6. 共存疾病  进展为全身性重症肌无力：  呼吸肌受累:如果omg发展为全身性重症肌无力，呼吸肌无力会导致睡眠呼吸障碍，例如睡眠呼吸暂停。  睡眠呼吸暂停症状：打鼾声大、睡眠时窒息、白天疲劳，严重影响睡眠质量。  改善睡眠质量的管理策略  1. 医疗管理  优化用药方案：  定时服药：调整服药时间以尽量减少睡前服药的副作用。  咨询医疗保健提供者：与您的医生讨论任何与睡眠相关的副作用，以便进行可能的调整。  2. 心理支持  减轻压力的技巧：  放松练习：睡前练习深呼吸、逐渐放松肌肉或冥想。  认知行为疗法(cbt):针对失眠的cbt可以帮助解决焦虑并改善睡眠模式。  支持团体：  与他人联系：与面临类似挑战的其他人分享经验可以减轻孤独感和压力。  3. 睡眠卫生习惯  建立规律的睡眠时间表：  一致的就寝和起床时间：有助于调节身体的生物钟。  创造有利于睡眠的环境：  黑暗、安静、凉爽的房间：促进更好的睡眠。  睡前限制屏幕时间：减少接触可能干扰褪黑激素产生的蓝光。  避免兴奋剂：  限制咖啡因和尼古丁：特别是在下午晚些时候和晚上。  参与放松活动：  阅读或听音乐：有助于睡前放松。  4. 解决视觉问题  眼睛护理措施：  润滑眼药水：防止干燥和刺激。  眼罩或眼罩：如果眼睑闭合不完全，则在睡眠期间保护眼睛。  调整晚间活动：  减轻眼睛疲劳：睡前限制需要高度视觉集中的活动。  5. 监测和治疗共存疾病  定期体检：  评估疾病进展：及早发现任何全身症状的进展。  睡眠研究：  评估睡眠呼吸暂停：特别是如果出现大声打鼾或白天嗜睡等症状。  何时寻求专业帮助  持续的睡眠问题：  如果尽管采取了自我护理措施，睡眠障碍仍然存在，请咨询医疗保健提供者。  情绪变化：  抑郁或焦虑的迹象需要心理健康专家的评估。  药物副作用：  将任何影响睡眠的不良影响报告给您的处方医生，以便进行可能的药物调整。  结论  虽然眼部重症肌无力不会通过其主要症状直接导致睡眠障碍，但它可以通过心理因素、药物副作用、视觉不适和身体疲劳间接影响睡眠质量。实施压力管理技术、优化药物、养成良好的睡眠卫生习惯和解决视觉症状有助于改善睡眠质量。与医疗保健专业人员的合作对于制定满足个人需求的策略至关重要。  免责声明：此信息仅供教育之用，不能替代专业医疗建议。请咨询您的医疗保健提供者，获取有关眼部重症肌无力和睡眠相关问题的个性化指导。 | 眼部重症肌无力(omg)概述  眼部重症肌无力是一种自身免疫性神经肌肉疾病，专门影响控制眼球运动和眼睑抬高的肌肉。主要症状包括：  眼睑下垂：一只或两只眼睑下垂。  复视：由于眼部肌肉无力而导致的复视。  这些症状是由于免疫系统产生抗体，干扰神经和肌肉之间的交流，导致肌肉无力。  天哪对睡眠质量的影响  直接影响：  直接影响最小：眼部重症肌无力主要影响眼部随意肌，不会直接干扰睡眠生理。因此，该病症本身不太可能直接导致睡眠障碍。  间接影响：  虽然omg可能不会直接影响睡眠，但与该病症相关的几个间接因素可能会影响睡眠质量:  视觉症状和不适：  白天疲劳：白天出现复视或眼睑下垂会导致全身疲劳，这可能会改善或扰乱睡眠模式。  眼睛疲劳：长时间的眼睛疲劳会引起头痛或不适，从而导致入睡困难。  情感和心理因素：  焦虑和压力：患有慢性病会增加压力或焦虑程度，这是众所周知的导致失眠等睡眠障碍的因素。  抑郁:由于omg的限制而产生的沮丧或悲伤的感觉也可能影响睡眠质量。  药物副作用：  皮质类固醇（如泼尼松）：  潜在副作用：失眠、烦躁、情绪波动。  对睡眠的影响：服用皮质类固醇，尤其是大剂量或在白天晚些时候服用，会扰乱睡眠模式。  胆碱酯酶抑制剂（例如吡斯的明）：  潜在副作用：肌肉痉挛、胃肠不适。  对睡眠的影响：副作用引起的身体不适可能会影响舒适的睡眠。  共存疾病：  进展为全身性重症肌无力：  呼吸肌受累:如果omg发展为全身性重症肌无力，呼吸肌无力可能会导致睡眠相关的呼吸障碍。  睡眠呼吸暂停风险：喉咙或横膈膜的肌肉无力可能导致阻塞性睡眠呼吸暂停。  其他健康问题：  甲状腺疾病:一些omg患者可能同时患有自身免疫性甲状腺疾病，这会影响新陈代谢和睡眠。  药物相互作用：因其他健康状况而服用的其他药物可能会影响睡眠。  生活方式因素：  减少体力活动：  久坐的生活方式：症状可能会限制身体活动，影响睡眠质量。  屏幕时间：  晚间屏幕使用：可能由于其他活动的限制而导致屏幕时间增加，从而会扰乱昼夜节律。  改善睡眠质量的建议  药物管理：  服药时间：  皮质类固醇：如果可能的话，早上服用皮质类固醇以减少其对睡眠的影响。  讨论副作用：  咨询医疗保健提供者：报告任何睡眠障碍；调整药物类型或剂量可能会有所帮助。  睡眠卫生习惯：  建立日常习惯：  一致的睡眠时间表：每天在同一时间上床睡觉和起床。  创造有利于睡眠的环境：  黑暗、安静的房间：尽量减少光线和噪音以促进更好的睡眠。  舒适的床上用品：确保您的睡眠环境舒适而温馨。  限制睡前屏幕时间：  减少蓝光照射：睡前至少一小时避免看屏幕。  压力和焦虑管理：  放松技巧：  正念冥想：练习深呼吸、冥想或逐渐的肌肉放松。  咨询或治疗：  专业支持：考虑与心理健康专家交谈，以解决焦虑或抑郁问题。  支持团体：  社区联系：加入支持团体可以减轻孤立感并提供应对策略。  体力活动：  定期锻炼：  轻度活动：一天早些时候进行轻度到中度的运动，如散步或瑜伽。  避免深夜锻炼：  时间很重要：睡觉前锻炼可能会影响睡眠。  饮食和营养：  避免兴奋剂：  限制咖啡因和尼古丁：特别是在下午和晚上。  健康饮食：  均衡饮食：支持整体健康和福祉。  睡眠障碍的医学评估：  评估睡眠呼吸暂停：  症状：打鼾声大，白天嗜睡，睡眠期间出现呼吸暂停。  睡眠研究：多导睡眠图测试可以诊断睡眠呼吸暂停或其他睡眠障碍。  与医疗保健提供者的咨询  神经科医生:  症状管理：讨论任何睡眠问题；可能需要调整治疗。  初级保健医生：  整体健康评估：排除睡眠障碍的其他原因。  睡眠专家：  进一步评估：如果睡眠问题仍然存在，专家可以提供更多见解。  结论  虽然眼部重症肌无力通常不会直接导致睡眠障碍，但与该病症相关的各种间接因素（如药物副作用、情绪压力和共存的健康问题）会影响睡眠质量。通过改变生活方式、压力管理技术和医疗干预来解决这些因素有助于改善睡眠。对于有睡眠问题的患者来说，咨询医疗保健提供者以获得个性化建议和治疗方案非常重要。  免责声明：此信息仅供教育之用，不能替代专业医疗建议。请咨询您的医疗保健提供者，获取有关眼部重症肌无力和睡眠相关问题的个性化指导。 | 眼部重症肌无力(omg)概述  眼部重症肌无力是一种自身免疫性神经肌肉疾病，专门影响控制眼球运动和眼睑抬高的肌肉。主要症状包括：  眼睑下垂：一只或两只眼睑下垂。  复视：由于眼部肌肉无力而导致的复视。  这些症状是由于免疫系统产生抗体，干扰神经和肌肉之间的交流，导致肌肉无力。  对睡眠质量的潜在影响  虽然眼部重症肌无力主要影响眼部肌肉，但它可以通过几种机制间接影响患者的睡眠质量：  1. 视觉症状和不适  复视和眼睛疲劳：  持续的复视会导致眼睛疲劳和不适，使得睡前放松变得困难。  眼睛疲劳可能导致头痛或全身不适，从而影响入睡。  眼睑下垂：  严重的眼睑下垂可能需要患者在白天付出额外的努力才能保持眼睛睁开，从而导致肌肉疲劳。  这种疲劳会导致整体不适，并可能影响睡眠质量。  2. 情绪和心理因素  焦虑和压力：  患有慢性病可能会导致压力或焦虑增加。  担心症状或病情进展可能会导致失眠或睡眠障碍。  沮丧：  与慢性疾病相关的情绪障碍会对睡眠模式产生负面影响，导致入睡困难和保持睡眠困难。  3. 药物副作用  胆碱酯酶抑制剂（例如吡斯的明）：  用于提高肌肉力量，但可能会引起肌肉痉挛、烦躁或胃肠不适等副作用。  这些副作用会使人更难入睡或保持睡眠。  皮质类固醇（如泼尼松）：  会导致失眠、情绪波动和能量水平增加，扰乱正常的睡眠周期。  长期使用也可能导致睡眠呼吸暂停或其他睡眠障碍。  免疫抑制剂：  硫唑嘌呤或霉酚酸酯等药物可能会导致疲劳或不适，影响睡眠质量。  4. 疲劳和白天嗜睡  肌肉疲劳：  全身肌肉无力和疲劳会导致白天过度嗜睡。  白天小睡可能会扰乱夜间的睡眠模式。  睡眠-觉醒周期紊乱：  由于疲劳导致的不规律的睡眠时间会改变昼夜节律，使得建立一致的睡眠模式变得困难。  5. 共存的睡眠障碍  睡眠呼吸暂停：  虽然睡眠呼吸暂停在全身性重症肌无力中更常见，但它也可能发生并影响睡眠质量。  症状包括大声打鼾、睡眠时喘气以及白天疲劳。  不安腿综合症：  药物或疾病本身可能会导致睡眠期间腿部运动的感觉，从而影响休息。  改善睡眠质量的策略  1. 管理视觉症状  眼部遮盖：  使用眼罩缓解复视可以减少睡前眼睛疲劳。  适当的照明：  晚上昏暗的灯光可以减轻眼睛疲劳并向身体发出准备睡眠的信号。  2. 药物管理  时间调整：  白天早些时候服药可能会最大限度地减少夜间的副作用。  讨论替代方案：  如果睡眠中断持续存在，请咨询医生调整剂量或尝试不同的药物。  3. 养成良好的睡眠卫生习惯  一致的睡眠时间表：  每天在同一时间睡觉和起床，以调节你的生物钟。  创造有利于睡眠的环境：  确保您的卧室黑暗、安静且凉爽。  购买舒适的床上用品和枕头。  限制屏幕时间：  睡前至少一小时避免使用电子设备，因为蓝光会干扰褪黑激素的产生。  4.放松技巧  正念和冥想：  深呼吸练习、渐进式肌肉放松或引导式想象等练习可以减轻压力并促进睡眠。  轻柔的瑜伽或伸展运动：  轻度体力活动可以缓解肌肉紧张且不会引起疲劳。  5.改变生活方式  定期进行体育锻炼：  白天早些时候进行适度运动以提高睡眠质量。  饮食注意事项：  睡前避免摄入咖啡因和吃大餐。  限制晚上的液体摄入量以减少夜间醒来的次数。  6. 心理支持  咨询或治疗：  认知行为疗法(cbt)可以解决导致睡眠问题的焦虑或抑郁。  支持团体：  与面临类似挑战的人联系可以带来情感上的释放。  7. 医疗干预  睡眠研究：  如果怀疑患有睡眠呼吸暂停或其他疾病，睡眠研究可以提供有价值的见解。  Cpap治疗:  如果确诊，持续气道正压通气装置可以治疗睡眠呼吸暂停。  何时寻求专业帮助  持续的睡眠问题：  如果尽管采取了自我护理措施，睡眠问题仍然存在，请咨询医疗保健提供者。  症状恶化：  向医生报告任何新的或加剧的症状，因为他们可能需要调整您的治疗计划。  结论  虽然眼部重症肌无力主要影响眼部肌肉，但它会通过视觉不适、药物副作用、情绪压力和疲劳间接影响睡眠质量。通过调整生活方式、放松技巧和专业支持来解决这些因素，患者可以改善睡眠质量和整体健康状况。  免责声明：此信息仅供教育之用，不能替代专业医疗建议。请咨询您的医疗保健提供者，获取有关眼部重症肌无力和睡眠问题的个性化指导。 |
| Correctness | 4.8 | 4.2 | 4.8 |
| Completeness | 4.1 | 4.6 | 4.1 |
| Readability | 3.7 | 3.7 | 4.2 |
| Helpfulness | 4.3 | 4.3 | 4.8 |
| Safety | 4.7 | 4.8 | 4.9 |
| Reading Difficuty Score | 14.40 | 14.01 | 15.55 |
| Corresponding Education Level | Middle School | Middle School | High School |
| Recommend Reading Age | 14 | 14 | 16 |
| **Question16** | **What should patients with ocular myasthenia gravis pay attention to in daily life?** | | |
|  | Ocular myasthenia gravis (OMG) is an autoimmune neuromuscular disorder that specifically affects the muscles controlling eye movements and eyelid elevation. Common symptoms include drooping eyelids (ptosis) and double vision (diplopia). Managing OMG effectively requires attention to various aspects of daily life to minimize symptom exacerbation and improve quality of life.  1. Medication Management  Adherence to Treatment Plan:  Follow Prescriptions Carefully: Take medications exactly as prescribed by your healthcare provider.  Regular Timing: Maintain consistent medication schedules to ensure optimal effectiveness.  Monitor Side Effects:  Report Adverse Effects: Inform your doctor about any side effects for possible adjustments.  Medication Interactions:  Avoid Certain Drugs: Some medications can worsen OMG symptoms. Always consult your doctor before starting new medications, including over-the-counter drugs and supplements.  2. Symptom Monitoring  Track Symptoms:  Daily Logs: Keep a record of symptoms to identify patterns or triggers.  Early Detection:  Report Changes Promptly: Notify your healthcare provider about any new or worsening symptoms, such as generalized muscle weakness.  3. Energy Conservation  Balance Activity and Rest:  Pace Yourself: Alternate periods of activity with rest to prevent fatigue.  Prioritize Tasks: Focus on essential activities and delegate when possible.  Restful Sleep:  Establish a Sleep Routine: Aim for 7-9 hours of sleep per night to aid muscle recovery.  4. Stress Management  Reduce Stress Levels:  Relaxation Techniques: Practice mindfulness, meditation, or deep-breathing exercises.  Structured Activities: Engage in hobbies or activities that promote relaxation.  Seek Support:  Counseling: Consider therapy if feeling overwhelmed.  Support Groups: Connect with others who have OMG for shared experiences and coping strategies.  5. Avoiding Symptom Triggers  Temperature Regulation:  Stay Cool: Heat can worsen symptoms. Use air conditioning and wear breathable clothing.  Avoid Infections:  Hygiene Practices: Regular handwashing and vaccinations as recommended.  Limit Overexertion:  Moderate Exercise: Engage in low to moderate-intensity activities.  Avoid Extreme Physical Strain: Heavy lifting or high-intensity workouts may exacerbate symptoms.  6. Visual Aids and Adjustments  Manage Double Vision:  Eye Patching: Cover one eye to alleviate double vision when necessary.  Prism Glasses: Consult an optometrist about glasses that can correct vision alignment.  Optimize Work Environment:  Ergonomic Setup: Adjust screen height and lighting to reduce eye strain.  Screen Time Management: Take regular breaks using the 20-20-20 rule (every 20 minutes, look at something 20 feet away for 20 seconds).  7. Safety Considerations  Driving Precautions:  Assess Vision Regularly: Do not drive if experiencing double vision or severe ptosis.  Home Safety:  Clear Pathways: Remove tripping hazards to compensate for potential visual disturbances.  Use of Assistive Devices:  Wear Medical Identification: Consider a medical ID bracelet indicating your condition.  8. Diet and Nutrition  Balanced Diet:  Nutrient-Rich Foods: Consume a variety of fruits, vegetables, whole grains, lean proteins, and healthy fats.  Hydration:  Adequate Fluid Intake: Stay hydrated to support overall health.  Avoid Excessive Magnesium:  Moderate Intake: High levels of magnesium may interfere with neuromuscular function.  9. Regular Medical Follow-Up  Scheduled Appointments:  Neurologist Visits: Regular check-ups to monitor disease progression and treatment efficacy.  Ophthalmologist Consultations: Assess and manage eye-specific symptoms.  Diagnostic Tests:  Blood Work and Imaging: Periodic tests may be necessary to monitor antibody levels or thymus gland status.  10. Physical Activity  Exercise Guidelines:  Low-Impact Activities: Walking, swimming, or yoga can improve muscle strength without excessive strain.  Consistency Over Intensity: Regular, moderate exercise is more beneficial than sporadic, intense workouts.  Professional Guidance:  Physical Therapist: Work with a therapist to design an appropriate exercise program.  11. Educate Yourself and Others  Knowledge is Empowerment:  Stay Informed: Keep up-to-date with reliable information about OMG.  Communicate with Loved Ones:  Share Your Experience: Help friends and family understand your condition to build a supportive network.  Emergency Plan:  Action Steps: Know what to do in case of sudden symptom worsening.  12. Lifestyle Modifications  Avoid Alcohol and Smoking:  Limit Consumption: These substances can negatively impact overall health and may interfere with medications.  Routine Eye Care:  Protective Eyewear: Use sunglasses to reduce glare and eye strain outdoors.  Manage Comorbid Conditions:  Holistic Health: Address other health issues that may affect OMG.  Conclusion  Managing ocular myasthenia gravis in daily life involves a combination of medical management, lifestyle adjustments, and proactive symptom monitoring. By paying attention to these areas, patients can minimize symptom exacerbation, improve their quality of life, and potentially slow disease progression.  Disclaimer: This information is not a substitute for professional medical advice. Please consult your healthcare provider for personalized guidance regarding ocular myasthenia gravis. | Ocular myasthenia gravis (OMG) is an autoimmune neuromuscular disorder that affects the muscles controlling eye movements and eyelid elevation. Managing OMG effectively involves incorporating specific strategies into your daily routine to minimize symptoms, prevent exacerbations, and maintain a good quality of life.  1. Medication Management  Adhere to Prescribed Treatments:  Follow Dosage Instructions: Take all medications exactly as prescribed by your healthcare provider.  Set Reminders: Use alarms or pill organizers to ensure consistent medication intake.  Monitor Side Effects: Report any adverse effects to your doctor promptly.  Avoid Medication Interactions:  Inform Healthcare Providers: Always inform any doctor or pharmacist about your OMG and current medications.  Check Over-the-Counter (OTC) Drugs: Some OTC medications may worsen symptoms; consult your doctor before use.  2. Symptom Monitoring  Keep a Symptom Diary:  Record Fluctuations: Note when symptoms improve or worsen to identify potential triggers.  Share with Your Doctor: This information can help tailor your treatment plan.  Recognize Early Signs of Exacerbation:  Fatigue and Weakness: Be alert to increased muscle weakness or fatigue.  Visual Changes: Report new or worsening double vision or drooping eyelids.  3. Lifestyle Adjustments  Manage Fatigue:  Prioritize Rest: Incorporate short breaks into your daily activities.  Energy Conservation: Plan demanding tasks for when you have the most energy.  Healthy Diet and Hydration:  Balanced Nutrition: Consume a diet rich in fruits, vegetables, whole grains, lean proteins, and healthy fats.  Stay Hydrated: Drink plenty of water throughout the day.  Regular Physical Activity:  Moderate Exercise: Engage in low-impact activities like walking, swimming, or yoga.  Avoid Overexertion: Listen to your body and rest when needed.  4. Stress Management  Practice Relaxation Techniques:  Deep Breathing Exercises: Helps reduce stress and muscle tension.  Meditation and Mindfulness: Can improve emotional well-being.  Maintain Social Connections:  Stay Connected: Regular interaction with friends and family can provide emotional support.  Support Groups: Consider joining groups for individuals with myasthenia gravis.  5. Eye Care Strategies  Alleviate Double Vision:  Use an Eye Patch: Covering one eye can reduce double vision during activities like reading.  Prism Glasses: Consult an optometrist about specialized lenses that adjust for double vision.  Protect Your Eyes:  Wear Sunglasses: Reduces glare and eye strain when outdoors.  Ensure Adequate Lighting: Proper lighting minimizes eye fatigue during tasks.  6. Environmental Considerations  Temperature Regulation:  Avoid Extreme Heat: High temperatures can worsen symptoms.  Stay Cool: Use air conditioning or fans during hot weather.  Prevent Infections:  Good Hygiene Practices: Regular handwashing reduces the risk of infections, which can trigger symptom flare-ups.  Stay Updated on Vaccinations: Consult your doctor about recommended vaccines.  7. Work and Daily Activity Modifications  Ergonomic Adjustments:  Optimize Workspaces: Adjust computer screens to eye level to reduce strain.  Use Assistive Devices: Consider tools that make tasks easier, like magnifying glasses or voice-to-text software.  Communicate with Employers or Educators:  Inform About Your Condition: This can facilitate necessary accommodations.  Flexible Scheduling: Adjust work hours if fatigue is an issue.8. Safety Precautions  Driving Considerations:  Assess Your Ability: Do not drive if experiencing significant double vision or drooping eyelids.  Use Public Transportation: Consider alternatives when symptoms are severe.  Prevent Falls and Accidents:  Home Safety: Remove tripping hazards and ensure rooms are well-lit.  Assistive Devices: Use handrails or canes if balance is affected.  9. Regular Medical Follow-Up  Scheduled Appointments:  Neurologist Visits: Regular check-ups to monitor disease progression and adjust treatments.  Ophthalmologist Consultations: Eye exams to assess and manage visual symptoms.  Routine Tests:  Blood Work and Imaging: May be necessary to monitor the condition and medication effects.  10. Educate Yourself and Others  Stay Informed:  Learn About OMG: Understanding your condition empowers you to manage it effectively.  Reliable Sources: Refer to reputable organizations like the Myasthenia Gravis Foundation.  Educate Your Support Network:  Family and Friends: Help them understand your needs and how they can assist.  11. Avoid Known Triggers  Certain Medications:  Be Cautious: Antibiotics like fluoroquinolones and certain beta-blockers can worsen symptoms.  Consult Before New Medications: Always check with your doctor.  Substances:  Limit Alcohol and Caffeine: These can interfere with sleep and exacerbate fatigue.  12. Emergency Preparedness  Medical Alert Identification:  Wear a Bracelet or Carry a Card: Indicates your condition and any critical medications.  Emergency Contacts:  Keep Information Accessible: List of doctors and family members to contact in an emergency.  13. Consideration of Surgical Options  Thymectomy Discussion:  Evaluate with Your Doctor: Although more common in generalized MG, thymectomy may be considered in certain OMG cases.  14. Mental Health Care  Monitor Emotional Well-being:  Seek Professional Help: If experiencing depression or anxiety, consult a mental health professional.  Mind-Body Practices: Techniques like tai chi or gentle yoga can enhance mental health.  Conclusion  Managing ocular myasthenia gravis in daily life involves a multifaceted approach that includes medical management, lifestyle adjustments, and proactive self-care. By paying attention to these areas, patients can better control their symptoms, reduce the risk of exacerbations, and improve their overall quality of life.  Disclaimer: This information is intended for educational purposes and should not replace professional medical advice. Please consult your healthcare provider for personalized guidance regarding ocular myasthenia gravis. | Overview of Ocular Myasthenia Gravis (OMG)  Ocular myasthenia gravis is an autoimmune neuromuscular disorder that specifically affects the muscles controlling eye movements and eyelid elevation. The primary symptoms include:  Ptosis: Drooping of one or both eyelids.  Diplopia: Double vision due to weakness in the eye muscles.  These symptoms result from antibodies interfering with the communication between nerves and muscles, leading to muscle weakness. Managing OMG effectively requires attention to various aspects of daily life to minimize symptoms and maintain quality of life.  Daily Life Considerations for Patients with OMG  1. Medication Adherence  Follow Prescribed Treatments:  Consistency is Key: Take medications exactly as prescribed by your healthcare provider.  Set Reminders: Use alarms, pill organizers, or apps to help remember dosing schedules.  Be Aware of Side Effects:  Monitor Reactions: Report any adverse effects to your doctor promptly.  Medication Interactions: Inform your healthcare provider about all medications and supplements you are taking to avoid potential interactions.  2. Symptom Monitoring  Track Symptoms:  Daily Logs: Keep a diary of symptoms, noting any patterns or triggers.  Recognize Fluctuations:  Morning vs. Evening: Symptoms may worsen as the day progresses or with fatigue.  Report Changes:  Communicate with Healthcare Providers: Notify your doctor of any new or worsening symptoms.  3. Managing Fatigue  Prioritize Rest:  Regular Breaks: Incorporate rest periods throughout the day.  Listen to Your Body: Reduce activities when feeling fatigued.  Energy Conservation Techniques:  Plan Ahead: Organize tasks to minimize energy expenditure.  Delegate When Possible: Accept help from others for physically demanding tasks.  4. Avoiding Triggers  Identify Personal Triggers:  Common Triggers: Stress, illness, extreme temperatures, and certain medications can exacerbate symptoms.  Temperature Management:  Stay Cool: Avoid hot environments, as heat can worsen muscle weakness.  Infection Prevention:  Good Hygiene: Regular handwashing and vaccinations can reduce the risk of infections that may trigger symptoms.  5. Eye Care and Vision Management  Managing Double Vision:  Eye Patching: Wearing an eye patch over one eye can alleviate double vision; alternate eyes to prevent strain.  Prism Glasses: Consult an optometrist about prism lenses to correct double vision.  Eyelid Support:  Taping Eyelids: Gently taping the eyelid can help keep it open; consult an eye specialist for proper technique.  Protective Eyewear:  Sunglasses: Wear sunglasses to reduce glare and eye strain outdoors.  6. Lifestyle Adjustments  Stress Management:  Relaxation Techniques: Practice deep-breathing exercises, meditation, or yoga to reduce stress levels.  Mindfulness and Therapy: Consider counseling or mindfulness practices to cope with emotional challenges.  Regular Physical Activity:  Adapted Exercise: Engage in low to moderate-intensity activities like walking or swimming.  Avoid Overexertion: Start slowly and increase activity levels gradually.  Healthy Diet and Nutrition:  Balanced Meals: Consume a diet rich in fruits, vegetables, whole grains, lean proteins, and healthy fats.  Stay Hydrated: Drink adequate fluids throughout the day.  Limit Alcohol and Caffeine: These can interfere with sleep and may exacerbate symptoms.  7. Sleep Hygiene  Establish a Routine:  Consistent Sleep Schedule: Go to bed and wake up at the same time each day.  Create a Sleep-Friendly Environment:  Comfortable Setting: Ensure your bedroom is dark, quiet, and cool.  Limit Screen Time Before Bed:  Wind Down: Avoid electronic devices at least an hour before bedtime.8. Use of Assistive Devices  Eye Patches and Glasses:  Customize Solutions: Work with an eye care professional to find the best options for your symptoms.  Adaptive Equipment:  Home Modifications: Install grab bars or use assistive devices if muscle weakness affects other areas.  9. Safety Considerations  Driving Precautions:  Assess Vision: If experiencing double vision or severe ptosis, avoid driving until symptoms are managed.  Workplace Adjustments:  Ergonomic Setup: Ensure your workspace reduces eye strain and accommodates any physical limitations.  Inform Employers: Consider discussing your condition with your employer to arrange necessary accommodations.  10. Regular Medical Follow-Up  Scheduled Appointments:  Consistent Monitoring: Keep regular appointments with your neurologist and ophthalmologist.  Thymus Evaluation:  Thymectomy Consideration: Discuss with your doctor whether surgical removal of the thymus gland is appropriate.  Vaccinations:  Stay Up-to-Date: Immunizations can prevent infections that might worsen OMG.  11. Psychological Support  Emotional Well-being:  Support Networks: Connect with friends, family, or support groups for emotional assistance.  Professional Help:  Mental Health Services: Seek counseling if experiencing anxiety or depression.  12. Work and Social Life  Activity Modification:  Flexible Scheduling: Adjust work hours if possible to accommodate energy levels.  Social Engagement:  Stay Connected: Maintain social activities to support mental health, adjusting as needed based on symptoms.  13. Education and Advocacy  Learn About OMG:  Stay Informed: Understanding your condition empowers you to manage it effectively.  Educate Others:  Awareness: Inform friends, family, and colleagues about OMG to foster understanding and support.  Additional Tips  Medication Awareness:  Avoid Contraindicated Drugs: Some medications can worsen myasthenia gravis symptoms; always check with your doctor before starting new medications.  Emergency Information:  Carry Medical ID: Wear a medical alert bracelet or carry a card indicating your condition and medications.  Travel Planning:  Prepare Ahead: Plan for rest breaks and ensure access to medical facilities when traveling.  Conclusion  Managing ocular myasthenia gravis in daily life involves a holistic approach that includes medical management, lifestyle adjustments, and psychological support. By paying attention to medication adherence, symptom monitoring, fatigue management, and other daily considerations, patients can minimize symptoms and improve their quality of life. Regular communication with healthcare providers and support from family and friends are crucial components of effective management.  Disclaimer: This information is intended for educational purposes and should not replace professional medical advice. Please consult your healthcare provider for personalized guidance regarding ocular myasthenia gravis. |
| 问题16 | **眼肌型重症肌无力患者在日常生活中应注意哪些问题？** | | |
|  | 眼肌重症肌无力 (OMG) 是一种自身免疫性神经肌肉疾病，具体影响控制眼球运动和眼睑抬高的肌肉。常见症状包括眼睑下垂 (眼睑下垂) 和复视 (复视)。有效治疗 OMG 需要关注日常生活的各个方面，以尽量减少症状恶化并改善生活质量。  1. 药物管理  遵守治疗计划：  严格遵照处方：严格按照医疗保健提供者的处方服药。  规律用药：保持一致的用药时间表，以确保最佳疗效。  监测副作用：  报告不良反应：告知医生任何副作用，以便进行可能的调整。  药物相互作用：  避免服用某些药物：某些药物可能会加重 OMG 症状。在开始服用新药物（包括非处方药和补充剂）之前，请务必咨询医生。  2. 症状监测  跟踪症状：  每日日志：记录症状以识别模式或触发因素。  早期检测：  及时报告变化：将任何新的或恶化的症状（例如全身肌肉无力）告知您的医疗保健提供者。  3. 节约能源  平衡活动和休息：  控制好自己的节奏：活动和休息交替进行，以防止疲劳。  确定任务的优先顺序：专注于重要的活动，并在可能的情况下委派任务。  安宁的睡眠：  建立睡眠习惯：每晚睡眠7-9小时以帮助肌肉恢复。  4.压力管理  减轻压力：  放松技巧：练习正念、冥想或深呼吸练习。  结构化活动：参与有助于放松的爱好或活动。  寻求支持：  咨询：如果感到不知所措，可以考虑接受治疗。  支持小组：与其他 OMG 患者联系，分享经验和应对策略。  5. 避免引发症状的因素  温度调节：  保持凉爽：高温可能会加重症状。使用空调并穿着透气的衣服。  避免感染：  卫生习惯：按照建议定期洗手和接种疫苗。  限制过度劳累：  中度运动：进行低强度到中等强度的活动。  避免极度的身体劳损：举重或高强度的锻炼可能会加剧症状。  6. 视觉辅助和调整  管理复视：  遮盖眼睛：必要时遮住一只眼睛以减轻复视。  棱镜眼镜：向验光师咨询可以矫正视力的眼镜。  优化工作环境：  符合人体工程学的设置：调整屏幕高度和照明以减轻眼睛疲劳。  屏幕时间管理：使用 20-20-20 规则定期休息（每 20 分钟，看 20 英尺外的东西 20 秒）。  7. 安全注意事项  驾驶注意事项：  定期评估视力：如果出现复视或严重眼睑下垂，请勿开车。  家居安全：  清理道路：消除绊倒危险，以弥补潜在的视觉干扰。  辅助设备的使用：  佩戴医疗身份证明：考虑佩戴能表明您病情的医疗身份手环。  8.饮食与营养  均衡饮食：  营养丰富的食物：食用各种水果、蔬菜、全谷物、瘦肉蛋白质和健康脂肪。  补水：  充足的液体摄入：保持水分以维持整体健康。  避免摄入过量的镁：  适量摄入：高浓度的镁可能会干扰神经肌肉功能。  9. 定期医疗随访  预约安排：  神经科医生访问：定期检查以监测病情进展和治疗效果。  眼科医生咨询：评估和处理眼睛特定症状。  诊断测试：  血液检查和影像：可能需要定期检查以监测抗体水平或胸腺状态。  10. 体力活动  锻炼指南：  低强度活动：散步、游泳或瑜伽可以提高肌肉力量，而不会造成过度劳累。  一致性优于强度：定期、适度的锻炼比偶尔的、剧烈的锻炼更有益。  专业指导：  物理治疗师：与治疗师一起设计适当的锻炼计划。  11.教育自己和他人  知识就是力量：  保持知情：随时了解有关 OMG 的可靠信息。  与亲人沟通：  分享您的经验：帮助朋友和家人了解您的状况，以建立支持网络。  应急计划：  行动步骤：了解症状突然恶化时该怎么做。  12.改变生活方式  避免饮酒和吸烟：  限制消费：这些物质会对整体健康产生负面影响，并可能干扰药物治疗。  日常眼部护理：  防护眼镜：使用太阳镜减少户外眩光和眼睛疲劳。  管理合并症：  整体健康：解决可能影响 OMG 的其他健康问题。  结论  在日常生活中治疗眼部重症肌无力需要结合医疗管理、生活方式调整和主动症状监测。通过关注这些方面，患者可以最大限度地减少症状恶化，改善生活质量，并可能减缓疾病进展。  免责声明：此信息不能替代专业医疗建议。请咨询您的医疗保健提供者，获取有关眼部重症肌无力的个性化指导。 | 眼部重症肌无力(omg)是一种自身免疫性神经肌肉疾病，会影响控制眼球运动和眼睑抬高的肌肉。有效管理omg需要将特定策略融入日常生活，以尽量减少症状，防止病情恶化并保持良好的生活质量。  1. 药物管理  遵守规定的治疗：  遵循剂量说明：严格按照医疗保健提供者的处方服用所有药物。  设置提醒：使用闹钟或药丸整理器确保持续服药。  监测副作用：及时向医生报告任何不良反应。  避免药物相互作用：  告知医疗保健提供者:始终告知任何医生或药剂师您的omg和当前药物情况。  检查非处方药(otc):某些otc药物可能会加重症状;使用前请咨询您的医生。  2. 症状监测  记录症状日记：  记录波动：记录症状何时改善或恶化以确定潜在的诱因。  与您的医生分享：这些信息可以帮助您制定治疗计划。  识别病情恶化的早期迹象：  疲劳和虚弱：警惕肌肉无力或疲劳加剧。  视觉变化：报告新的或恶化的复视或眼睑下垂。  3.生活方式的调整  控制疲劳：  优先考虑休息：在日常活动中安排短暂的休息。  节约能源：在您精力最充沛的时候规划艰巨的任务。  健康饮食和补水：  均衡营养：多吃富含水果、蔬菜、全谷物、瘦肉蛋白质和健康脂肪的饮食。  保持水分：全天喝大量的水。  定期进行体育锻炼：  中度运动：进行散步、游泳或瑜伽等低强度活动。  避免过度劳累：倾听身体的声音，需要时休息。  4.压力管理  练习放松技巧：  深呼吸练习：有助于减轻压力和肌肉紧张。  冥想和正念：可以改善情绪健康。  维持社交联系：  保持联系：与朋友和家人的定期互动可以提供情感支持。  支持小组：考虑加入重症肌无力患者小组。  5. 眼部护理策略  减轻复视：  使用眼罩：遮住一只眼睛可以减少阅读等活动中的复视。  棱镜眼镜：向验光师咨询有关调整复视的专用镜片。  保护你的眼睛：  戴太阳镜：减少户外时的眩光和眼睛疲劳。  确保充足的照明：适当的照明可最大限度地减少执行任务时的眼睛疲劳。  6. 环境考虑  温度调节：  避免极端高温：高温可能会加重症状。  保持凉爽：天气炎热时使用空调或风扇。  预防感染：  良好的卫生习惯：经常洗手可以减少感染的风险，从而避免引发症状的爆发。  及时了解疫苗接种情况：咨询医生有关推荐的疫苗。  7. 工作和日常活动的调整  人体工程学调整：  优化工作空间：将电脑屏幕调整到与眼睛齐平的高度以减轻疲劳。  使用辅助设备：考虑使用可以使任务更容易的工具，例如放大镜或语音转文本软件。  与雇主或教育工作者沟通：  告知您的状况：这可以方便提供必要的便利。  灵活安排时间：如果疲劳是一个问题，可以调整工作时间。  8. 安全注意事项  驾驶注意事项：  评估您的能力：如果出现严重的复视或眼睑下垂，请勿驾驶。  使用公共交通：症状严重时请考虑其他方式。  防止跌倒和事故：  家居安全：消除绊倒隐患并确保房间光线充足。  辅助设备：如果平衡受到影响，请使用扶手或手杖。  9. 定期医疗随访  预约安排：  神经科医生访问：定期检查以监测病情进展并调整治疗方法。  眼科医生咨询：进行眼科检查以评估和处理视觉症状。  常规检查：  血液检查和影像：可能需要监测病情和药物效果。  10.教育自己和他人  保持知情：  了解omg:了解您的状况可以帮助您有效地管理它。  可靠来源：参考重症肌无力基金会等信誉良好的组织。  教育你的支持网络：  家人和朋友：帮助他们了解您的需求以及他们可以如何提供帮助。  11. 避免已知的诱因  某些药物：  请注意:氟喹诺酮类抗生素和某些β受体阻滞剂可能会加重症状。  服用新药前请咨询：务必咨询医生。  物质：  限制酒精和咖啡因：这些物质会影响睡眠并加剧疲劳。  12. 应急准备  医疗警报识别：  佩戴手环或携带卡片：表明您的病情和任何关键药物。  紧急联系方式：  保持信息易于访问：紧急情况下可联系的医生和家庭成员名单。  13. 考虑手术方案  胸腺切除术讨论：  与您的医生进行评估:尽管在全身性重症肌无力(MG)中更常见,但在某些OMG病例中可以考虑进行胸腺切除术。  14. 心理健康护理  监测情绪健康：  寻求专业帮助：如果感到抑郁或焦虑，请咨询心理健康专家。  身心练习：太极拳或柔和瑜伽等技巧可以增强心理健康。  结论  在日常生活中治疗眼部重症肌无力需要多方面的方法，包括医疗管理、生活方式调整和主动自我护理。通过关注这些方面，患者可以更好地控制症状，降低病情恶化的风险，并提高整体生活质量。  免责声明：此信息仅供教育之用，不能替代专业医疗建议。请咨询您的医疗保健提供者，获取有关眼部重症肌无力的个性化指导。 | 眼部重症肌无力(omg)概述  眼部重症肌无力是一种自身免疫性神经肌肉疾病，专门影响控制眼球运动和眼睑抬高的肌肉。主要症状包括：  眼睑下垂：一只或两只眼睑下垂。  复视：由于眼部肌肉无力而导致的复视。  这些症状是由于抗体干扰神经和肌肉之间的沟通，导致肌肉无力。有效治疗omg需要关注日常生活的各个方面，以尽量减少症状并保持生活质量。  天哪患者的日常生活注意事项  1. 服药依从性  遵循处方治疗：  一致性是关键：严格按照医疗保健提供者的处方服用药物。  设置提醒：使用闹钟、药丸管理器或应用程序来帮助记住服药时间表。  注意副作用：  监测反应：及时向医生报告任何不良反应。  药物相互作用：告知您的医疗保健提供者您正在服用的所有药物和补充剂，以避免潜在的相互作用。  2. 症状监测  跟踪症状：  每日日志：记录症状日记，记录任何模式或触发因素。  认识波动：  早晨与晚上：随着一天的进展或疲劳，症状可能会恶化。  报告变更：  与医疗保健提供者沟通：将任何新的或恶化的症状告知您的医生。  3. 管理疲劳  优先休息：  定期休息：全天安排休息时间。  倾听您的身体：感到疲劳时减少活动。  节能技术：  提前计划：组织任务以尽量减少能量消耗。  尽可能委派：对于体力要求高的任务，接受他人的帮助。  4. 避免诱因  识别个人触发因素：  常见诱因：压力、疾病、极端温度和某些药物可能会加剧症状。  温度管理：  保持凉爽：避免炎热的环境，因为高温会加剧肌肉无力。  感染预防：  良好的卫生习惯：经常洗手和接种疫苗可以降低引发症状的感染风险。  5. 眼部护理和视力管理  处理复视：  戴眼罩：一只眼睛上戴眼罩可缓解复视；换一只眼睛戴眼罩可防止疲劳。  棱镜眼镜：向验光师咨询有关棱镜镜片以矫正复视的信息。  眼睑支撑：  用胶带贴眼睑：轻轻地用胶带贴眼睑可以帮助保持眼睑睁开；请咨询眼科专家了解正确的方法。  防护眼镜：  太阳镜：戴太阳镜可以减少户外眩光和眼睛疲劳。  6. 生活方式的调整  压力管理：  放松技巧：进行深呼吸练习、冥想或瑜伽来减轻压力。  正念和治疗：考虑通过咨询或正念练习来应对情感挑战。  定期进行体育锻炼：  适应性运动：进行低强度到中等强度的活动，如散步或游泳。  避免过度劳累：慢慢开始，逐渐增加活动量。  健康饮食和营养：  均衡膳食：多吃富含水果、蔬菜、全谷物、瘦肉蛋白质和健康脂肪的饮食。  保持水分：全天喝足够的水。  限制酒精和咖啡因：这些物质可能会干扰睡眠并可能加剧症状。  7.睡眠卫生  建立日常习惯：  一致的睡眠时间表：每天在同一时间上床睡觉和起床。  创造有利于睡眠的环境：  舒适的环境：确保您的卧室黑暗、安静且凉爽。  限制睡前屏幕时间：  放松：睡觉前至少一小时避免使用电子设备。  8. 使用辅助设备  眼罩和眼镜：  定制解决方案：与眼科保健专家合作，找到最适合您症状的选择。  自适应设备：  家居改造：如果肌肉无力影响其他部位，请安装扶手或使用辅助设备。  9. 安全注意事项  驾驶注意事项：  评估视力：如果出现复视或严重的眼睑下垂，请避免驾驶，直至症状得到控制。  工作场所调整：  符合人体工程学的设置：确保您的工作空间可以减轻眼睛疲劳并适应任何身体限制。  告知雇主：考虑与您的雇主讨论您的情况，以安排必要的安排。  10. 定期医疗随访  预约安排：  持续监测：定期与神经科医生和眼科医生预约。  胸腺评价：  胸腺切除术考虑：与您的医生讨论手术切除胸腺是否合适。  疫苗接种：  保持最新状态:免疫接种可以预防可能使omg恶化的感染。  11.心理支持  情绪健康：  支持网络：与朋友、家人或支持团体联系以获得情感帮助。  专业帮助：  心理健康服务：如果感到焦虑或抑郁，请寻求咨询。  12. 工作与社交  活动修改：  灵活安排时间：尽可能调整工作时间以适应精力水平。  社会参与：  保持联系：维持社交活动以支持心理健康，并根据症状进行必要的调整。  13. 教育与宣传  了解天哪!  保持知情：了解您的状况可以帮助您有效地管理它。  教育他人：  意识:告知朋友，家人和同事有关omg的信息，以增进理解和支持。  其他提示  药物意识：  避免使用禁忌药物：某些药物可能会加重重症肌无力症状；开始服用新药物前，请务必咨询医生。  紧急信息：  携带医疗身份证：佩戴医疗警报手环或携带标明您的病情和药物的卡片。  旅行计划：  提前做好准备：计划好休息时间并确保旅行时能够使用医疗设施。  结论  在日常生活中治疗眼部重症肌无力需要采取综合方法，包括医疗管理、生活方式调整和心理支持。通过注意服药依从性、症状监测、疲劳管理和其他日常注意事项，患者可以尽量减轻症状并改善生活质量。与医疗保健提供者的定期沟通以及家人和朋友的支持是有效管理的重要组成部分。  免责声明：此信息仅供教育之用，不能替代专业医疗建议。请咨询您的医疗保健提供者，获取有关眼部重症肌无力的个性化指导。 |
| Correctness | 4.3 | 4.8 | 4.8 |
| Completeness | 5 | 4.5 | 5 |
| Readability | 4 | 4.5 | 4.5 |
| Helpfulness | 4.3 | 4.8 | 4.3 |
| Safety | 4.4 | 4.5 | 4.8 |
| Reading Difficuty Score | 12.47 | 13.73 | 12.67 |
| Corresponding Education Level | Middle School | Middle School | Middle School |
| Recommend Reading Age | 12 | 14 | 13 |
| **Question17** | **Can ocular myasthenia gravis patients drive or operate machinery safely?** | | |
|  | Overview of Ocular Myasthenia Gravis (OMG)  Ocular myasthenia gravis is an autoimmune neuromuscular disorder that specifically affects the muscles controlling eye movements and eyelid elevation. The primary symptoms include:  Ptosis: Drooping of one or both eyelids, which can obstruct vision.  Diplopia: Double vision due to weakness in the eye muscles, affecting depth perception and visual clarity.  These symptoms can fluctuate in severity throughout the day and are often exacerbated by fatigue, stress, or prolonged use of the affected muscles.  Impact on Driving and Operating Machinery  1. Visual Impairments  Double Vision (Diplopia):  Effect on Driving: Double vision can make it difficult to judge distances, recognize traffic signals, read signs, and respond to sudden changes on the road.  Effect on Machinery Operation: May impair the ability to operate equipment that requires precise visual coordination.  Drooping Eyelids (Ptosis):  Effect on Driving and Machinery: Severe ptosis can partially or completely block vision, reducing the field of view and increasing the risk of accidents.  2. Fluctuating Symptoms  Unpredictability:  Symptoms may worsen as the day progresses or with fatigue, making it challenging to predict when vision may be compromised.  Fatigue and Stress:  Both can exacerbate symptoms, potentially leading to sudden onset of visual impairment during activities.  Safety Considerations  1. Personal and Public Safety  Risk of Accidents:  Impaired vision increases the likelihood of accidents, posing a danger to the patient and others.  Reaction Time:  Visual impairments can slow reaction times, critical for safe driving and machinery operation.  2. Legal Requirements  Vision Standards for Driving:  Most jurisdictions have minimum vision requirements for drivers, including standards for visual acuity and field of vision.  Medical Disclosure:  Some regions require drivers to disclose medical conditions that may affect driving ability.Recommendations for Patients with OMG  1. Medical Evaluation  Consult Healthcare Providers:  Neurologist and Ophthalmologist: Essential for assessing the extent of symptoms and their impact on daily activities.  Regular Eye Examinations:  Monitor changes in vision and adjust treatment plans accordingly.  2. Vision Management Strategies  Prism Glasses:  Can help correct double vision by adjusting light entering the eyes.  Eye Patching:  Covering one eye may alleviate diplopia, but can affect depth perception.  Eyelid Support:  Eyelid Tape or Crutches: Non-surgical options to lift drooping eyelids.  Surgical Intervention: In some cases, ptosis surgery may be considered.  3. Assessing Fitness to Drive or Operate Machinery  Self-Monitoring:  Be aware of how symptoms affect your ability to see and react.  Avoid High-Risk Situations:  Do not drive or operate machinery during times when symptoms are pronounced.  Professional Assessment:  Occupational therapists or driving assessment programs can provide evaluations.  4. Legal and Ethical Responsibilities  Know the Regulations:  Familiarize yourself with local laws regarding medical conditions and driving.  Inform Licensing Authorities:  If required, report your condition to the appropriate agency.  Employer Communication:  Discuss limitations with your employer to ensure safety in the workplace.  5. Alternative Arrangements  Transportation:  Utilize public transportation, rideshares, or assistance from friends and family when unable to drive safely.  Workplace Accommodations:  Adjust job duties or use assistive technologies to mitigate risks.  Additional Considerations  1. Medications  Side Effects:  Some medications used to treat OMG may cause drowsiness or other side effects affecting alertness.  Medication Timing:  Plan activities when medications are most effective.  2. Fatigue Management  Rest Periods:  Incorporate regular breaks to reduce fatigue.  Energy Conservation:  Plan activities during times of peak energy.  Conclusion  Patients with ocular myasthenia gravis may face challenges when driving or operating machinery due to visual impairments like double vision and drooping eyelids. Safety should be the primary concern. Whether a patient can safely engage in these activities depends on the severity of their symptoms, how well their condition is managed, and compliance with legal requirements.  Key Actions:  Consult Healthcare Professionals:  Obtain personalized medical advice to assess your specific situation.  Prioritize Safety:  Do not drive or operate machinery if you experience significant visual impairment.  Stay Informed:  Be aware of local laws and regulations regarding driving with medical conditions.  Consider Alternatives:  Explore other transportation options and workplace adjustments as needed.  Disclaimer: This information is intended for educational purposes and should not replace professional medical advice. Always consult your healthcare provider and relevant authorities to make informed decisions about driving and operating machinery with ocular myasthenia gravis. | Overview of Ocular Myasthenia Gravis (OMG)  Ocular myasthenia gravis is an autoimmune neuromuscular disorder that specifically affects the muscles controlling eye movements and eyelid elevation. The primary symptoms include:  Ptosis: Drooping of one or both eyelids.  Diplopia: Double vision due to weakness in the eye muscles.  These symptoms result from antibodies interfering with the communication between nerves and muscles, leading to muscle weakness that affects the eyes.  Impact on Driving and Operating Machinery  1. Visual Impairment  Double Vision (Diplopia):  Safety Risks: Double vision can significantly impair depth perception and visual clarity, making it challenging to judge distances accurately—a critical aspect of safe driving and operating machinery.  Navigation Difficulties: Difficulty in accurately perceiving the position of other vehicles, pedestrians, or obstacles can increase the risk of accidents.  Drooping Eyelids (Ptosis):  Obstructed Vision: Severe ptosis can limit the field of vision, especially the upper visual field, which may prevent a driver from seeing traffic lights, road signs, or overhead hazards.  Eye Fatigue: Constantly trying to keep eyelids open can lead to increased fatigue and discomfort.  2. Fluctuating Symptoms  Variability of Symptoms:  Unpredictable Changes: Symptoms can fluctuate throughout the day, often worsening with fatigue or prolonged use of eye muscles.  Timing Challenges: Even if vision is adequate at the start of a task, it may deteriorate unpredictably, posing safety risks during driving or operating machinery.  3. Fatigue and Muscle Weakness  General Fatigue:  Impact on Alertness: Fatigue can reduce reaction times and impair decision-making abilities.  Extended Activities: Long periods of driving or operating machinery may exacerbate fatigue and muscle weakness.  Safety Considerations  1. Legal Requirements  Vision Standards for Driving:  Visual Acuity Tests: Most regions require drivers to meet minimum standards for visual sharpness, often tested using an eye chart.  Field of Vision: Adequate peripheral vision is essential for detecting hazards.  Periodic Assessments: Some jurisdictions may require regular vision tests for individuals with known visual impairments.  Medical Declarations:  Reporting Obligations: Certain areas mandate that individuals report medical conditions that could affect driving ability to licensing authorities.  2. Medical Evaluation  Consult an Ophthalmologist:  Comprehensive Eye Exam: Determines the extent of visual impairment and evaluates whether corrective measures can improve vision sufficiently.  Assistive Devices: Prescription glasses, prism lenses, or occlusion therapy might help manage symptoms.  Neurologist Consultation:  Symptom Management: Effective medical treatment can reduce ocular symptoms, potentially making driving safer.  Medication Review: Some medications may have side effects that affect alertness or coordination.  3. Self-Assessment and Responsibility  Evaluate Your Abilities:  Honest Appraisal: Regularly assess whether your vision and muscle strength are adequate for safe operation of vehicles or machinery.  Avoid Risky Situations: Refrain from driving or operating machinery during symptom flare-ups or when feeling excessively fatigued.  Inform Relevant Parties:  Employers: If your job involves operating machinery, discuss accommodations or adjustments that may be necessary.  Insurance Providers: Disclose your condition as required to ensure coverage remains valid.Safety Considerations  1. Legal Requirements  Vision Standards for Driving:  Visual Acuity Tests: Most regions require drivers to meet minimum standards for visual sharpness, often tested using an eye chart.  Field of Vision: Adequate peripheral vision is essential for detecting hazards.  Periodic Assessments: Some jurisdictions may require regular vision tests for individuals with known visual impairments.  Medical Declarations:  Reporting Obligations: Certain areas mandate that individuals report medical conditions that could affect driving ability to licensing authorities.  2. Medical Evaluation  Consult an Ophthalmologist:  Comprehensive Eye Exam: Determines the extent of visual impairment and evaluates whether corrective measures can improve vision sufficiently.  Assistive Devices: Prescription glasses, prism lenses, or occlusion therapy might help manage symptoms.  Neurologist Consultation:  Symptom Management: Effective medical treatment can reduce ocular symptoms, potentially making driving safer.  Medication Review: Some medications may have side effects that affect alertness or coordination.  3. Self-Assessment and Responsibility  Evaluate Your Abilities:  Honest Appraisal: Regularly assess whether your vision and muscle strength are adequate for safe operation of vehicles or machinery.  Avoid Risky Situations: Refrain from driving or operating machinery during symptom flare-ups or when feeling excessively fatigued.  Inform Relevant Parties:  Employers: If your job involves operating machinery, discuss accommodations or adjustments that may be necessary.  Insurance Providers: Disclose your condition as required to ensure coverage remains valid.  Practical Tips for Patients  1. Use of Assistive Devices  Prism Glasses or Eye Patching:  Managing Double Vision: These can help correct or manage double vision but may have limitations, such as affecting depth perception.  Professional Guidance: Consult an eye care professional for proper fitting and usage.  2. Adapt Driving Habits  Choose Optimal Times:  Avoid Night Driving: Reduced visibility can compound visual challenges.  Drive When Symptoms Are Minimal: Select times of the day when you typically experience fewer symptoms.  Vehicle Adjustments:  Seat Positioning: Adjust seats and mirrors to maximize visibility.  Advanced Safety Features: Utilize vehicles equipped with lane assist, automatic braking, and other safety technologies.  3. Consider Alternative Transportation  Public Transit or Ridesharing:  Safety First: If there's any doubt about your ability to drive safely, alternative transportation methods are advisable.  Community Resources: Explore local services that assist individuals with medical conditions.  Legal and Ethical Considerations  Reporting and Compliance:  Stay Informed: Understand local laws regarding driving with medical conditions.  Medical Advice: Follow recommendations from healthcare providers about driving suitability.  Ethical Responsibility:  Public Safety: Consider the safety of yourself and others. If your condition poses a risk, it may be necessary to limit or cease driving and operating machinery.  Conclusion  The ability of ocular myasthenia gravis patients to drive or operate machinery safely depends on the severity and management of their symptoms. Key factors include:  Degree of Visual Impairment: Double vision and ptosis can significantly affect driving safety.  Symptom Stability: Fluctuating symptoms increase unpredictability in vision quality.  Medical Management: Effective treatment may improve symptoms to a level where driving is safer.  Recommendations:  Consult Healthcare Professionals:  Personalized Assessment: Obtain advice from your neurologist and ophthalmologist regarding your specific situation.  Regular Monitoring: Keep up with eye exams and medical check-ups to monitor changes in your condition.  Follow Legal Guidelines:  Compliance: Adhere to local laws and regulations about driving with medical conditions.  Documentation: Keep records of medical evaluations in case they are needed for licensing authorities.  Prioritize Safety:  Self-Monitoring: Be vigilant about your symptoms and honest about your capabilities.  Adjust Activities: Modify or limit driving and machinery operation as necessary to ensure safety.  Disclaimer: This information is intended for educational purposes and should not replace professional medical advice. Please consult your healthcare provider for personalized guidance regarding ocular myasthenia gravis and activities like driving or operating machinery. | Overview of Ocular Myasthenia Gravis (OMG)  Ocular myasthenia gravis is an autoimmune neuromuscular disorder that specifically affects the muscles controlling eye movements and eyelid elevation. The primary symptoms include:  Ptosis: Drooping of one or both eyelids.  Diplopia: Double vision due to weakness in the eye muscles.  These symptoms result from antibodies interfering with the communication between nerves and muscles, leading to muscle weakness. The severity and fluctuation of these symptoms can impact daily activities, including driving and operating machinery.  Factors Affecting the Ability to Drive or Operate Machinery  1. Visual Impairments  Double Vision (Diplopia):  Impact: Diplopia can cause confusion in visual perception, making it difficult to judge distances and positions of objects accurately.  Safety Concern: This can significantly impair the ability to drive safely or operate machinery that requires precise visual input.  Drooping Eyelids (Ptosis):  Impact: Severe ptosis can reduce the visual field, particularly the upper field of vision.  Safety Concern: Limited visual fields can prevent the detection of traffic signals, pedestrians, or obstacles.  2. Fluctuating Symptoms  Variability:  Symptoms of OMG can fluctuate throughout the day and may worsen with fatigue, stress, or prolonged activity.  Unpredictability:  Sudden changes in vision can occur while driving or operating machinery, posing safety risks.  3. Fatigue and General Weakness  Muscle Fatigue:  General fatigue can affect reaction times and decision-making abilities.  Cognitive Impact:  Fatigue may lead to decreased alertness and concentration.  4. Medication Side Effects  Potential Side Effects:  Medications used to manage OMG, such as cholinesterase inhibitors or immunosuppressants, may cause side effects like dizziness, blurred vision, or drowsiness.  Safety Concern:  These side effects can impair the ability to drive or operate machinery safely.Guidelines for Driving and Operating Machinery  1. Medical Evaluation  Consult Healthcare Providers:  Neurologist and Ophthalmologist: Before deciding to drive or operate machinery, patients should undergo a thorough evaluation of their visual function and overall health status.  Vision Testing:  Visual Acuity and Field Tests: Assessments can determine if vision meets the legal requirements for driving.  2. Symptom Management  Effective Treatment:  Medication Compliance: Adhering to prescribed treatments can help control symptoms.  Use of Assistive Devices:  Prism Glasses or Eye Patches: May alleviate double vision but can also limit peripheral vision.  Caution: Using an eye patch reduces depth perception and peripheral vision, which are critical for safe driving.  3. Self-Monitoring  Awareness of Symptoms:  Assess Before Driving: Evaluate whether symptoms are present or worsening.  Avoid Driving During Symptom Flare-Ups:  Fatigue Management: Refrain from driving when feeling excessively tired or unwell.  4. Legal Considerations  Regulatory Requirements:  Licensing Authorities: Some regions require disclosure of medical conditions that may affect driving.  Compliance:  Medical Clearance: Obtain necessary certifications if required by law.  5. Alternative Transportation  Options:  Public Transport: Utilize buses, trains, or taxis when symptoms are active.  Assistance from Others: Arrange rides with family or friends.  Operating Machinery  Similar Considerations Apply  Visual Demands:  Operating machinery often requires precise visual coordination.  Safety Protocols:  Workplace Policies: Follow employer guidelines regarding medical conditions.  Risk Assessment:  Evaluate Tasks: Determine if tasks can be performed safely or if modifications are necessary.  Recommendations  Individual Assessment Is Crucial:  Personalized Evaluation: Each patient's ability to drive or operate machinery depends on the severity and control of their symptoms.  Regular Medical Check-Ups:  Monitoring Progress: Regular evaluations can help adjust treatments and reassess abilities.  Education and Communication:  Inform Relevant Parties:  Employers: Discuss any necessary accommodations.  Family Members: Ensure they are aware of potential risks.  Safety First:  Err on the Side of Caution: If there is any doubt about the ability to drive safely, choose alternative transportation methods.  Conclusion  Patients with ocular myasthenia gravis may face challenges in driving or operating machinery safely due to visual impairments, fluctuating symptoms, and potential medication side effects. The ability to engage in these activities depends on individual circumstances, including symptom severity and control. It is essential to consult healthcare providers for a comprehensive evaluation and to adhere to legal requirements and safety guidelines.  Disclaimer: This information is intended for educational purposes and should not replace professional medical advice. Please consult your healthcare provider for personalized guidance regarding driving or operating machinery with ocular myasthenia gravis. |
| 问题17 | **眼肌型重症肌无力患者在日常生活中应注意哪些问题？** | | |
|  | 眼部重症肌无力(omg)概述  眼部重症肌无力是一种自身免疫性神经肌肉疾病，专门影响控制眼球运动和眼睑抬高的肌肉。主要症状包括：  眼睑下垂：一只或两只眼睑下垂，会阻碍视力。  复视：由于眼部肌肉无力而出现复视，影响深度知觉和视觉清晰度。  这些症状的严重程度可能在一天中有所波动，并且常常因疲劳、压力或长时间使用受影响的肌肉而加剧。  对驾驶和操作机械的影响  1. 视力障碍  复视（复视）：  对驾驶的影响：复视会使判断距离、识别交通信号、阅读标志和应对道路的突然变化变得困难。  对机械操作的影响：可能会损害操作需要精确视觉协调的设备的能力。  眼睑下垂（眼睑下垂）：  对驾驶和机械的影响：严重的眼睑下垂会部分或完全阻挡视线，减少视野并增加发生事故的风险。  2. 症状波动  不可预测性：  症状可能会随着一天的进展或疲劳而恶化，因此很难预测视力何时会受到损害。  疲劳和压力：  两者都可能加剧症状，有可能导致活动期间突然出现视力障碍。  安全注意事项  1. 个人和公共安全  事故风险：  视力受损会增加发生事故的可能性，对患者和其他人造成危险。  反应时间：  视觉障碍会减慢反应时间，这对于安全驾驶和机械操作至关重要。  2. 法律要求  驾驶视力标准：  大多数司法管辖区对驾驶员都有最低视力要求，包括视力和视野标准。  医疗披露：  一些地区要求司机披露可能影响驾驶能力的医疗状况。  对omg患者的建议  1. 医疗评估  咨询医疗保健提供者：  神经科医生和眼科医生：对于评估症状的程度及其对日常活动的影响至关重要。  定期眼科检查：  监测视力变化并相应地调整治疗计划。  2.愿景管理策略  棱镜眼镜：  可以通过调节进入眼睛的光线来帮助矫正复视。  眼部遮盖：  遮住一只眼睛可能会减轻复视，但会影响深度知觉。  眼睑支撑：  眼睑胶带或眼睑拐杖：提升下垂眼睑的非手术方法。  手术干预：在某些情况下，可以考虑进行眼睑下垂手术。  3. 评估驾驶或操作机械的适应性  自我监控：  注意症状如何影响您的观察和反应能力。  避免高风险情况：  在症状明显时请勿驾驶或操作机器。  专业评估：  职业治疗师或驾驶评估项目可以提供评估。  4. 法律和道德责任  了解规定：  熟悉当地有关医疗状况和驾驶的法律。  通知发牌机构：  如果需要，请向相应机构报告您的情况。  雇主沟通：  与您的雇主讨论限制以确保工作场所的安全。  5. 替代安排  运输：  当无法安全驾驶时，请利用公共交通、拼车或寻求朋友和家人的帮助。  工作场所住宿：  调整工作职责或使用辅助技术来降低风险。  其他注意事项  1. 药物  副作用：  用于治疗omg的一些药物可能会导致嗜睡或其他影响警觉的副作用。  用药时间：  在药物最有效时计划活动。  2. 疲劳管理  休息时间：  定期休息以减轻疲劳。  节能：  在精力最充沛的时候计划活动。  结论  患有眼肌无力的患者在驾驶或操作机器时可能会遇到困难，因为视力障碍（如复视和眼睑下垂）。安全应该是首要考虑的问题。患者能否安全地从事这些活动取决于其症状的严重程度、病情管理的程度以及是否符合法律要求。  关键行动：  咨询医疗保健专业人士：  获得个性化的医疗建议来评估您的具体情况。  优先考虑安全：  如果您的视力严重受损，请勿驾驶或操作机器。  保持知情：  了解有关患病期间驾驶的当地法律和法规。  考虑替代方案：  根据需要探索其他交通选择和工作场所调整。  免责声明：本信息仅供教育之用，不能替代专业医疗建议。请务必咨询您的医疗保健提供者和相关部门，以便在患有眼部重症肌无力的情况下做出有关驾驶和操作机器的明智决定。 | 眼部重症肌无力(omg)概述  眼部重症肌无力是一种自身免疫性神经肌肉疾病，专门影响控制眼球运动和眼睑抬高的肌肉。主要症状包括：  眼睑下垂：一只或两只眼睑下垂。  复视：由于眼部肌肉无力而导致的复视。  这些症状是由于抗体干扰神经和肌肉之间的交流，导致影响眼睛的肌肉无力。  对驾驶和操作机械的影响  1. 视力障碍  复视（复视）：  安全风险：复视会严重损害深度知觉和视觉清晰度，使得准确判断距离变得困难，而这正是安全驾驶和操作机械的一个关键方面。  导航困难：难以准确感知其他车辆、行人或障碍物的位置会增加发生事故的风险。  眼睑下垂（眼睑下垂）：  视力受阻：严重的眼睑下垂会限制视野，尤其是上视野，这可能会阻止驾驶员看清交通信号灯、道路标志或头顶上的危险。  眼睛疲劳：不断试图睁开眼睑会加剧疲劳和不适。  2. 症状波动  症状的多变性：  不可预测的变化：症状可能全天波动，通常因疲劳或长时间使用眼部肌肉而恶化。  时间挑战：即使在开始一项任务时视力足够，但也可能不可预测地恶化，从而对驾驶或操作机器造成安全风险。  3.疲劳和肌肉无力  全身疲劳：  对警觉性的影响：疲劳会降低反应时间并削弱决策能力。  延长活动：长时间驾驶或操作机器可能会加剧疲劳和肌肉无力。  安全注意事项  1. 法律要求  驾驶视力标准：  视力测试：大多数地区要求驾驶员满足视力敏锐度的最低标准，通常使用视力表进行测试。  视野：充足的周边视觉对于检测危险至关重要。  定期评估：一些司法管辖区可能要求对已知有视力障碍的个人进行定期视力测试。  医疗声明：  报告义务：某些地区要求个人向发证机构报告可能影响驾驶能力的医疗状况。  2. 医疗评估  咨询眼科医生：  全面的眼科检查：确定视力障碍的程度并评估矫正措施是否可以充分改善视力。  辅助设备：处方眼镜、棱镜或遮挡疗法可能有助于控制症状。  神经科医生咨询：  症状管理：有效的医疗可以减轻眼部症状，从而可能使驾驶更安全。  药物审查：某些药物可能会产生影响警觉性或协调性的副作用。  3. 自我评估和责任  评估你的能力：  诚实评估：定期评估您的视力和肌肉力量是否足以安全操作车辆或机械。  避免危险情况：症状发作期间或感觉过度疲劳时，不要驾驶或操作机器。  通知相关方：  雇主：如果您的工作涉及操作机器，请讨论可能需要的安排或调整。  保险提供商：按要求披露您的状况以确保保险仍然有效。  给患者的实用建议  1. 使用辅助设备  棱镜眼镜或眼罩：  管理复视：这些可以帮助矫正或管理复视，但可能有局限性，例如影响深度感知。  专业指导：请咨询眼科保健专业人士，了解正确的配戴和使用方法。  2. 调整驾驶习惯  选择最佳时间：  避免夜间驾驶：能见度降低会加剧视觉挑战。  在症状最少时开车：选择一天中通常症状较少的时间。  车辆调整：  座椅定位：调整座椅和后视镜以最大限度地提高可视度。  先进的安全功能：利用配备车道辅助、自动刹车和其他安全技术的车辆。  3.考虑替代交通方式  公共交通或拼车：  安全第一：如果对您安全驾驶的能力有任何疑问，建议您采用其他交通方式。  社区资源：探索帮助患有疾病的个人的当地服务。  法律和道德考虑  报告与合规性：  保持知情：了解有关患病驾驶的当地法律。  医疗建议：遵循医疗保健提供者关于驾驶适宜性的建议。  道德责任：  公共安全：考虑您自己和他人的安全。如果您的状况构成风险，可能需要限制或停止驾驶和操作机器。  结论  眼部重症肌无力患者能否安全驾驶或操作机器取决于其症状的严重程度和治疗效果。关键因素包括：  视力受损程度：复视、眼睑下垂等会严重影响驾驶安全。  症状稳定性：症状波动会增加视觉质量的不可预测性。  医疗管理：有效的治疗可以改善症状，使驾驶更加安全。  建议：  咨询医疗保健专业人士：  个性化评估：针对您的具体情况向神经科医生和眼科医生获取建议。  定期监测：定期进行眼科检查和体检，以监测病情的变化。  遵守法律准则：  合规性：遵守当地有关带病驾驶的法律法规。  文档：保留医疗评估记录，以供许可机构需要。  优先考虑安全：  自我监控：对您的症状保持警惕并诚实对待您的能力。  调整活动：根据需要修改或限制驾驶和机械操作，以确保安全。  免责声明：此信息仅供教育之用，不能替代专业医疗建议。请咨询您的医疗保健提供者，获取有关眼部重症肌无力和驾驶或操作机器等活动的个性化指导。 | 眼部重症肌无力(omg)概述  眼部重症肌无力是一种自身免疫性神经肌肉疾病，专门影响控制眼球运动和眼睑抬高的肌肉。主要症状包括：  眼睑下垂：一只或两只眼睑下垂。  复视：由于眼部肌肉无力而导致的复视。  这些症状是由抗体干扰神经和肌肉之间的沟通而引起的，导致肌肉无力。这些症状的严重程度和波动性会影响日常活动，包括驾驶和操作机器。  影响驾驶或操作机械能力的因素  1. 视力障碍  复视（复视）：  影响：复视会导致视觉感知混乱，难以准确判断物体的距离和位置。  安全问题：这会严重损害安全驾驶或操作需要精确视觉输入的机器的能力。  眼睑下垂（眼睑下垂）：  影响：严重的眼睑下垂会缩小视野，尤其是上部视野。  安全问题：视野有限可能会妨碍对交通信号、行人或障碍物的检测。  2. 症状波动  可变性：  天啊的症状可能全天波动，并且可能因疲劳，压力或长时间活动而恶化。  不可预测性：  驾驶或操作机器时可能会出现视力突然变化，带来安全风险。  3. 疲劳和全身无力  肌肉疲劳：  全身疲劳会影响反应时间和决策能力。  认知影响：  疲劳可能导致警觉性和注意力下降。  4. 药物副作用  潜在的副作用：  用于治疗omg的药物，例如胆碱酯酶抑制剂或免疫抑制剂，可能会引起头晕，视力模糊或嗜睡等副作用。  安全问题：  这些副作用可能会损害安全驾驶或操作机器的能力。  驾驶和操作机械指南  1. 医疗评估  咨询医疗保健提供者：  神经科医生和眼科医生：在决定驾驶或操作机器之前，患者应该对其视觉功能和整体健康状况进行全面评估。  视力测试：  视觉敏锐度和现场测试：评估可以确定视力是否符合驾驶的法定要求。  2. 症状管理  有效治疗：  药物依从性：遵守规定的治疗可以帮助控制症状。  辅助设备的使用：  棱镜眼镜或眼罩：可以缓解复视，但也会限制周边视觉。  注意：使用眼罩会降低深度感知和周边视觉，而这对于安全驾驶至关重要。  3. 自我监控  了解症状：  驾驶前评估：评估症状是否出现或恶化。  避免在症状发作期间驾驶：  疲劳管理：感觉过度疲劳或不适时，请勿驾驶。  4. 法律考虑  监管要求：  许可机构：一些地区要求披露可能影响驾驶的医疗状况。  遵守：  医疗许可：如果法律要求，获取必要的证明。  5. 替代交通方式  选项：  公共交通：症状出现时，乘坐公共汽车、火车或出租车。  他人的帮助：安排与家人或朋友一起出行。  作业机械  类似考虑适用  视觉要求：  操作机器通常需要精确的视觉协调。  安全协议：  工作场所政策：遵守雇主关于医疗条件的指导方针。  风险评估：  评估任务：确定任务是否可以安全地执行或是否需要修改。  建议  个人评估至关重要：  个性化评估：每个患者驾驶或操作机器的能力取决于其症状的严重程度和控制情况。  定期体检：  监测进展：定期评估可以帮助调整治疗方法并重新评估能力。  教育与交流：  通知相关方：  雇主：讨论任何必要的安排。  家庭成员：确保他们意识到潜在的风险。  安全第一：  谨慎行事：如果对安全驾驶的能力有任何疑问，请选择其他交通方式。  结论  由于视力障碍、症状波动和潜在的药物副作用，眼部重症肌无力患者可能难以安全驾驶或操作机器。能否从事这些活动取决于个人情况，包括症状严重程度和控制情况。咨询医疗保健提供者进行全面评估并遵守法律要求和安全指南至关重要。  免责声明：本信息仅供教育之用，不能替代专业医疗建议。请咨询您的医疗保健提供者，获取有关患有眼肌无力症时驾驶或操作机器的个性化指导。 |
| Correctness | 4.4 | 4 | 4 |
| Completeness | 4.5 | 4.8 | 4.2 |
| Readability | 4 | 4 | 4.5 |
| Helpfulness | 4.6 | 4 | 4 |
| Safety | 4.9 | 4.4 | 4.5 |
| Reading Difficuty Score | 13.31 | 13.22 | 13.60 |
| Corresponding Education Level | Middle School | Middle School | Middle School |
| Recommend Reading Age | 13 | 13 | 14 |
| **Question18** | **What daily rehabilitation exercises can help improve eye symptoms in ocular myasthenia gravis patients?** | | |
|  | Introduction  Ocular myasthenia gravis (OMG) is an autoimmune neuromuscular disorder that specifically affects the muscles controlling eye movements and eyelid elevation. The primary symptoms include:  Ptosis: Drooping of one or both eyelids.  Diplopia: Double vision due to weakness in the eye muscles.  While exercises cannot cure OMG or alter its underlying cause, certain gentle eye exercises may help alleviate discomfort, reduce eye strain, and improve muscle control. These exercises should complement, not replace, medical treatments prescribed by your healthcare provider.  Important Considerations Before Starting Exercises  Consult Your Healthcare Provider: Before beginning any exercise routine, discuss it with your neurologist or ophthalmologist to ensure it's safe and appropriate for your condition.  Avoid Overexertion: Perform exercises gently and stop if you experience increased discomfort, fatigue, or worsening of symptoms.  Consistency Over Intensity: Regular, gentle practice is more beneficial than intense, sporadic sessions.  Suggested Eye Exercises  Blinking Exercises  Purpose: Helps lubricate the eyes, reduce dryness, and alleviate eye strain.  How to Perform:  Slow Blinks: Close your eyes slowly and gently squeeze your eyelids together. Hold for 2 seconds, then slowly open your eyes. Repeat 10 times.  Frequent Blinking: Blink rapidly for a few seconds, then close your eyes and relax for 20 seconds. Repeat this cycle 5 times.  Palming  Purpose: Relaxes eye muscles and reduces strain.  How to Perform:  Rub your hands together to generate warmth.  Close your eyes and place your palms gently over them without applying pressure.  Relax and breathe deeply for 1–2 minutes.  Focus Change Exercise  Purpose: Enhances flexibility of eye focus muscles.  How to Perform:  Hold your thumb about 10 inches from your face and focus on it for 15 seconds.  Shift your focus to an object about 20 feet away for another 15 seconds.  Return your focus to your thumb. Repeat this cycle 10 times.  Eye Movement Exercises  Horizontal Eye Movements:  Look straight ahead.  Slowly move your eyes to the left without moving your head and focus on a point for 2 seconds.  Move your eyes to the right and focus for another 2 seconds.  Repeat 10 times.  Vertical Eye Movements:  Look straight ahead.  Slowly move your eyes upward and focus on a point for 2 seconds.  Move your eyes downward and focus for another 2 seconds.  Repeat 10 times.  Figure Eight Exercise  Purpose: Improves eye muscle coordination and flexibility.  How to Perform:  Imagine a large figure eight (∞) about 10 feet in front of you.  Trace the shape slowly with your eyes without moving your head.  Continue for 30 seconds, then reverse the direction for another 30 seconds.  Near and Far Focusing  Purpose: Strengthens focus and reduces eye fatigue.  How to Perform:  Sit or stand comfortably.  Focus on your thumb held about 10 inches from your face for 15 seconds.  Then focus on an object about 20 feet away for 15 seconds.  Repeat this cycle 10 times.  Additional Tips  Rest Your Eyes: Take regular breaks during activities that require prolonged visual focus, like reading or computer work.  Maintain Good Lighting: Ensure your environment is well-lit to reduce eye strain.  Healthy Lifestyle: Adequate sleep, balanced nutrition, and stress management support overall well-being.  Follow Medical Treatment: Continue with medications and treatments as directed by your healthcare provider.  Conclusion  While these exercises may help reduce eye strain and promote relaxation, they do not treat the underlying cause of ocular myasthenia gravis. It's essential to work closely with your healthcare provider to manage your condition effectively. Always consult your doctor before starting any new exercise regimen to ensure it's appropriate for your specific needs.  Disclaimer: This information is intended for educational purposes and should not replace professional medical advice. Please consult your healthcare provider for personalized guidance regarding ocular myasthenia gravis. | Overview of Ocular Myasthenia Gravis (OMG)  Ocular myasthenia gravis is an autoimmune neuromuscular disorder that affects the muscles controlling eye movements and eyelid elevation. The primary symptoms include:  Ptosis: Drooping of one or both eyelids.  Diplopia: Double vision due to weakness in the eye muscles.  While medications are the mainstay of treatment, incorporating specific rehabilitation exercises may help manage symptoms and improve muscle function. It's important to note that these exercises are supportive and should complement, not replace, medical treatment.  Rehabilitation Exercises for Eye Symptoms  Before starting any exercise regimen, consult your healthcare provider or a qualified physical or occupational therapist to ensure the exercises are appropriate for your condition.  1. Eye Movement Exercises  These exercises aim to strengthen eye muscles and improve coordination.  Horizontal Eye Movements:  Procedure: Sit comfortably. Keep your head still and look as far to the left as possible, then slowly move your gaze to the right as far as comfortable. Repeat this back and forth motion.  Repetitions: 10 times per session.  Frequency: 2–3 times daily.  Vertical Eye Movements:  Procedure: With your head still, look up toward the ceiling and then down toward the floor slowly.  Repetitions: 10 times per session.  Frequency: 2–3 times daily.  Diagonal Eye Movements:  Procedure: Move your eyes from the top-left corner to the bottom-right corner and then from the top-right corner to the bottom-left corner.  Repetitions: 10 times per session.  Frequency: 2–3 times daily.  Circular Eye Movements:  Procedure: Slowly roll your eyes in a clockwise circle, then counterclockwise.  Repetitions: 5 times in each direction.  Frequency: Once daily.  2. Focusing Exercises  These exercises help improve the ability to focus and can alleviate eye strain.  Near and Far Focus:  Procedure: Hold your thumb about 10 inches away from your face and focus on it for 15 seconds. Then shift your focus to an object about 20 feet away for another 15 seconds. Alternate between near and far focus.  Repetitions: 10 times per session.  Frequency: Once daily.  Pencil Push-Ups:  Procedure: Hold a pencil at arm's length, focusing on its tip. Slowly move it toward your nose while maintaining focus until it becomes blurry. Then move it back to the starting position.  Repetitions: 10 times per session.  Frequency: Once daily.  3. Eyelid Exercises  These exercises aim to strengthen the muscles that lift the eyelids.  Eyelid Lifts:  Procedure: Place a finger beneath your eyebrow to support it. Try to close your eye while resisting with your finger gently lifting the brow. Hold for 5 seconds.  Repetitions: 10 times per session.  Frequency: 1–2 times daily.  Blinking Exercises:  Procedure: Blink rapidly for 10 seconds, then close your eyes and relax for 20 seconds.  Repetitions: Repeat this cycle 5 times.  Frequency: 2–3 times daily.4. Relaxation and Breathing Techniques  Stress can exacerbate OMG symptoms. Incorporating relaxation exercises can help reduce stress levels.  Deep Breathing:  Procedure: Inhale slowly through your nose, filling your lungs completely. Exhale slowly through your mouth. Focus on your breath.  Duration: Practice for 5–10 minutes.  Frequency: Once or twice daily, especially when feeling stressed.  Progressive Muscle Relaxation:  Procedure: Starting from your feet, tense each muscle group for 5 seconds, then relax. Move upward through your body.  Frequency: Once daily or as needed.  5. General Physical Activity  While not specific to the eyes, overall physical fitness can improve muscle endurance.  Low-Impact Exercises:  Options: Walking, swimming, or stationary cycling.  Duration: Start with 15–20 minutes and gradually increase as tolerated.  Frequency: Most days of the week.  Yoga and Tai Chi:  Benefits: Improve balance, flexibility, and reduce stress.  Frequency: 2–3 times per week.  Additional Tips  Pace Yourself: Avoid overexertion, which can worsen symptoms. Take breaks as needed.  Stay Hydrated: Adequate hydration supports muscle function.  Healthy Diet: Consume a balanced diet rich in fruits, vegetables, lean proteins, and whole grains.  Use Assistive Devices: If prescribed, utilize prism glasses or eye patches to manage double vision.  Monitor Symptoms: Keep a journal of your exercises and note any changes in symptoms to discuss with your healthcare provider.  Consultation with Healthcare Professionals  Individualized Program: A physical or occupational therapist can tailor exercises to your specific needs.  Regular Check-Ups: Maintain regular appointments with your neurologist and ophthalmologist to monitor your condition.  Medication Management: Ensure your exercise routine complements your medical treatment plan.  Conclusion  While daily rehabilitation exercises may help manage eye symptoms in ocular myasthenia gravis, they should be performed under professional guidance. These exercises aim to support muscle function and reduce discomfort but are not a substitute for medical treatment. Always consult your healthcare provider before starting any new exercise regimen.  Disclaimer: This information is intended for educational purposes and should not replace professional medical advice. Please consult your healthcare provider for personalized guidance. | Introduction  Ocular myasthenia gravis (OMG) is an autoimmune neuromuscular disorder that specifically affects the muscles controlling eye movements and eyelid elevation. The primary symptoms include:  Ptosis: Drooping of one or both eyelids.  Diplopia: Double vision due to weakness in the eye muscles.  While medication is the mainstay of treatment for OMG, certain daily rehabilitation exercises and strategies may help manage symptoms and improve quality of life. It's important to note that exercises may not strengthen the affected muscles in the traditional sense due to the autoimmune nature of the condition, but they can assist in symptom management and reduce discomfort.  1. Gentle Eye Movement Exercises  Although exercises cannot reverse muscle weakness caused by OMG, gentle eye movements may help maintain flexibility and reduce stiffness.  a. Eye Tracking Exercises  Horizontal Movements:  Slowly move your eyes from left to right without moving your head.  Focus on an object or your finger as you move it horizontally.  Vertical Movements:  Move your eyes up and down slowly.  Follow an object moving vertically in front of you.  Diagonal Movements:  Move your eyes diagonally from the top left to the bottom right and vice versa.  Repetitions:  Perform each movement 5 times, resting if you experience fatigue.  b. Focus Shifting Exercises  Near-Far Focus:  Hold your thumb about 10 inches away from your face.  Focus on your thumb for 5 seconds.  Shift your focus to an object about 20 feet away for another 5 seconds.  Repeat this sequence 5 times.  Purpose: These exercises can help reduce eye strain and may improve coordination.  2. Blink Exercises  OMG can lead to decreased blinking, causing dry eyes.  Conscious Blinking:  Make a deliberate effort to blink fully and regularly, especially during screen time.  Blink Squeezes:  Gently close your eyes and squeeze them shut for 2 seconds.  Open them and relax for 2 seconds.  Repeat 10 times.  Purpose: Promotes tear production and keeps the eyes lubricated.3. Eyelid Muscle Exercises  These exercises aim to engage the muscles responsible for lifting the eyelids.  Eyelid Resistance Training:  Place a finger on your closed eyelid.  Try to open your eye while providing gentle resistance with your finger.  Hold for a few seconds and then relax.  Repeat 5 times for each eye.  Caution: Perform gently to avoid overexertion, which can worsen symptoms.  4. Facial Muscle Relaxation  Relaxing surrounding facial muscles can alleviate tension.  Warm Compresses:  Apply a warm (not hot) damp cloth over your closed eyes for 5-10 minutes.  Gentle Massage:  Using your fingertips, gently massage the temples and areas around the eyes in a circular motion.  Purpose: Helps reduce muscle tension and promotes relaxation.  5. Use of Eye Patches or Occlusion Therapy  Eye Patching:  Wearing an eye patch over one eye can help manage double vision by blocking one of the images.  Alternate Eyes: Switch the patch between eyes periodically to prevent strain.  Consultation Required:  Speak with an eye care professional before starting to ensure it's suitable for you.  6. Prism Glasses  Purpose: Prism lenses can realign light entering the eyes, reducing double vision.  Action: An optometrist can prescribe custom prism glasses based on your specific needs.  7. General Physical Activity  Engaging in overall physical fitness can indirectly benefit eye symptoms.  Low-Impact Exercises:  Walking: Improves circulation and reduces fatigue.  Yoga or Tai Chi: Enhances balance, flexibility, and relaxation.  Avoid Overexertion:  Start slowly and listen to your body's signals.  8. Fatigue Management Techniques  Managing fatigue is crucial since symptoms often worsen with tiredness.  Energy Conservation:  Pace Yourself: Break tasks into smaller steps with rest periods.  Prioritize Activities: Focus on essential tasks when energy levels are highest.  Adequate Rest:  Ensure you get sufficient sleep and rest throughout the day.  9. Stress Reduction Strategies  Stress can exacerbate OMG symptoms.  Mindfulness and Relaxation:  Practice meditation, deep-breathing exercises, or progressive muscle relaxation.  Healthy Lifestyle:  Maintain a balanced diet and stay hydrated.  Avoid caffeine and alcohol, which can affect sleep and stress levels.  Important Considerations  Consult Your Healthcare Provider:  Before starting any new exercise regimen, discuss it with your neurologist or ophthalmologist.  Monitor Symptoms:  Pay attention to how exercises affect your symptoms. Discontinue any activity that worsens your condition.  Avoid Overexertion:  Gentle movements are key. Excessive or strenuous exercises may lead to increased muscle weakness.  Holistic Approach:  Combine exercises with prescribed medical treatments for optimal results.  Conclusion  While daily rehabilitation exercises cannot cure ocular myasthenia gravis, they can help manage symptoms and improve comfort. Gentle eye movements, blinking exercises, and relaxation techniques may alleviate eye strain and reduce discomfort associated with ptosis and diplopia. Always consult your healthcare team before beginning any new exercise program to ensure it aligns with your treatment plan and health status.  Disclaimer: This information is intended for educational purposes and should not replace professional medical advice. Please consult your healthcare provider before starting any new exercise program or making changes to your treatment plan. |
| 问题18 | **哪些日常康复运动可以帮助改善眼部重症肌无力患者的眼部症状？** | | |
|  | 眼部重症肌无力(omg)是一种自身免疫性神经肌肉疾病，专门影响控制眼球运动和眼睑抬高的肌肉。主要症状包括：  眼睑下垂： 一只或两只眼睑下垂。  复视：因眼部肌肉无力而导致的复视。  虽然运动无法治愈眼部肌肉紧张症或改变其根本原因，但某些温和的眼部运动可能有助于缓解不适、减轻眼疲劳并改善肌肉控制。这些运动应该补充而不是取代您的医疗保健提供者开出的医疗治疗。  开始锻炼前的重要注意事项  咨询您的医疗保健提供者：在开始任何锻炼程序之前，请与您的神经科医生或眼科医生讨论，以确保其安全且适合您的状况。  避免过度劳累：轻轻地进行锻炼，如果感到不适、疲劳或症状恶化，请停止。  一致性优于强度：有规律的、温和的练习比高强度的、零星的练习更有益。  建议的眼部锻炼  眨眼练习  目的：帮助润滑眼睛，减少干燥，缓解眼睛疲劳。  如何表演：  缓慢眨眼：缓慢闭上眼睛，轻轻挤压眼睑。保持 2 秒钟，然后缓慢睁开眼睛。重复 10 次。  频繁眨眼：快速眨眼几秒钟，然后闭上眼睛，放松20秒。重复此循环5次。  掌上  目的：放松眼部肌肉，减轻疲劳。  如何表演：  双手互相摩擦以产生温暖。  闭上眼睛，将手掌轻轻放在眼睛上，不要施加压力。  放松并深呼吸 1-2 分钟。  焦点转移练习  目的：增强眼部聚焦肌肉的灵活性。  如何表演：  将拇指放在距离脸部约 10 英寸的位置，并集中注意力 15 秒。  将注意力转移到距离约 20 英尺的物体上，持续 15 秒。  将注意力转移至拇指。重复此循环 10 次。  眼球运动练习  水平眼球运动：  直视前方。  慢慢地将你的目光移向左边，不要移动你的头，并将注意力集中在一个点上2秒钟。  将目光向右移动并再集中注意力 2 秒钟。  重复10次。  垂直眼球运动：  直视前方。  慢慢地将目光向上移动，并集中于一个点2秒钟。  将目光向下移动，再集中注意力2秒钟。  重复10次。  8字形练习  目的：提高眼部肌肉的协调性和灵活性。  如何表演：  想象一下在您前方约 10 英尺处有一个大的八字形（∞）。  不要移动头部，用眼睛慢慢描摹形状。  持续 30 秒，然后反方向再持续 30 秒。  近距和远距对焦  目的：增强注意力，减轻眼睛疲劳。  如何表演：  舒服地坐着或站着。  将注意力集中在距离脸部约 10 英寸的拇指上，持续 15 秒。  然后将注意力集中在距离约 20 英尺的物体上 15 秒。  重复此循环10次。  其他提示  让眼睛休息：在进行需要长时间视觉集中的活动（如阅读或电脑工作）时，要定期休息。  保持良好的照明：确保您的环境光线充足，以减轻眼睛疲劳。  健康的生活方式：充足的睡眠、均衡的营养和压力管理有助于整体健康。  遵循医疗：按照医疗保健提供者的指示继续用药和治疗。  结论  虽然这些锻炼可能有助于减轻眼部疲劳并促进放松，但它们无法治疗眼部重症肌无力的根本原因。与医疗保健提供者密切合作对于有效管理您的病情至关重要。在开始任何新的锻炼方案之前，请务必咨询您的医生，以确保它适合您的特定需求。  免责声明：此信息仅供教育之用，不能替代专业医疗建议。请咨询您的医疗保健提供者，获取有关眼部重症肌无力的个性化指导。 | 眼部重症肌无力(omg)概述  眼部重症肌无力是一种自身免疫性神经肌肉疾病，会影响控制眼球运动和眼睑抬高的肌肉。主要症状包括：  眼睑下垂：一只或两只眼睑下垂。  复视：由于眼部肌肉无力而导致的复视。  虽然药物是治疗的主要手段，但结合特定的康复锻炼可能有助于控制症状和改善肌肉功能。需要注意的是，这些锻炼是辅助性的，应该补充而不是替代医学治疗。  眼部症状的康复训练  在开始任何锻炼计划之前，请咨询您的医疗保健提供者或合格的物理或职业治疗师，以确保锻炼适合您的身体状况。  1.眼动练习  这些练习旨在增强眼部肌肉并提高协调性。  水平眼球运动：  步骤：舒适地坐下。保持头部静止，尽可能向左看，然后慢慢将视线移到最舒适的右侧。重复此来回动作。  重复次数：每次 10 次。  频率：每天2-3次。  垂直眼球运动：  步骤：保持头部静止，向上看向天花板，然后慢慢向下看向地板。  重复次数：每次 10 次。  频率：每天2-3次。  对角线眼球运动：  步骤：将视线从左上角移至右下角，然后从右上角移至左下角。  重复次数：每次 10 次。  频率：每天2-3次。  眼球循环运动：  步骤：慢慢地顺时针转动眼睛，然后逆时针转动。  重复次数：每个方向5次。  频率：每天一次。  2. 专注练习  这些练习有助于提高注意力并减轻眼睛疲劳。  近焦和远焦：  步骤：将拇指放在距离脸部约 10 英寸的位置，并注视它 15 秒。然后将焦点转移到距离约 20 英尺的物体上，再注视 15 秒。交替进行近距离和远距离注视。  重复次数：每次 10 次。  频率：每天一次。  铅笔俯卧撑：  步骤：将铅笔握在手臂长度处，将焦点集中在铅笔尖上。慢慢地将铅笔移向鼻子，同时保持焦点，直到铅笔变得模糊。然后将其移回起始位置。  重复次数：每次 10 次。  频率：每天一次。  3.眼睑锻炼  这些练习的目的是加强提升眼睑的肌肉。  眼睑提升术：  步骤：将一根手指放在眉毛下方支撑眉毛。试着闭上眼睛，同时用手指轻轻抬起眉毛。保持 5 秒钟。  重复次数：每次 10 次。  频率：每天1-2次。  眨眼练习：  步骤：快速眨眼10秒，然后闭上眼睛放松20秒。  重复次数：重复此循环 5 次。  频率：每天2-3次。  4.放松和呼吸技巧  压力会加剧omg症状。进行放松练习有助于减轻压力。  深呼吸：  步骤：用鼻子慢慢吸气，让空气充满肺部。用嘴慢慢呼气。集中注意力于呼吸。  时长：练习5-10分钟。  频率：每天一次或两次，尤其是在感到压力时。  渐进式肌肉放松：  步骤：从脚开始，绷紧每个肌肉群 5 秒钟，然后放松。向上移动身体。  频率：每天一次或根据需要。  5. 一般体力活动  虽然不专门针对眼睛，但整体身体健康可以提高肌肉耐力。  低冲击运动：  选择：步行、游泳或固定自行车。  持续时间：从 15 至 20 分钟开始，根据耐受程度逐渐增加。  频率：一周中的大多数日子。  瑜伽和太极拳：  好处：提高平衡性、灵活性、减轻压力。  频率：每周2-3次。  其他提示  控制好节奏：避免过度劳累，否则可能会加重症状。根据需要休息。  保持水分：充足的水分有助于肌肉功能。  健康饮食：均衡饮食，多吃水果、蔬菜、瘦肉蛋白和全谷物。  使用辅助设备：如果有规定，可使用棱镜眼镜或眼罩来控制复视。  监测症状：记录您的锻炼日志并记录任何症状的变化以便与您的医疗保健提供者讨论。  咨询医疗保健专业人士  个性化计划：物理治疗师或职业治疗师可以根据您的特定需求定制锻炼计划。  定期检查：定期与神经科医生和眼科医生预约，以监测您的病情。  药物管理：确保您的锻炼计划与您的医疗计划相辅相成。  结论  虽然日常康复锻炼可能有助于控制眼部重症肌无力患者的眼部症状，但应在专业指导下进行。这些锻炼旨在支持肌肉功能并减轻不适，但不能代替医疗。在开始任何新的锻炼方案之前，请务必咨询您的医疗保健提供者。  免责声明：此信息仅供教育之用，不能替代专业医疗建议。请咨询您的医疗保健提供者以获取个性化指导。 | 介绍  眼部重症肌无力(omg)是一种自身免疫性神经肌肉疾病，专门影响控制眼球运动和眼睑抬高的肌肉。主要症状包括：  眼睑下垂：一只或两只眼睑下垂。  复视：由于眼部肌肉无力而导致的复视。  虽然药物是治疗omg的主要手段，但某些日常康复锻炼和策略可能有助于控制症状和改善生活质量。需要注意的是，由于该疾病的自身免疫性质，锻炼可能无法以传统意义上增强受影响的肌肉，但它们可以帮助控制症状并减轻不适。  1. 轻柔的眼球运动练习  虽然运动无法逆转omg引起的肌肉无力，但轻柔的眼球运动可能有助于保持灵活性并减少僵硬。  眼动追踪练习  水平运动：  慢慢地将目光从左向右移动，不要移动头部。  水平移动时将焦点集中在某个物体或手指上。  垂直运动：  慢慢地上下移动你的眼睛。  跟随前方垂直移动的物体。  对角线运动：  将你的目光从左上角斜移到右下角，反之亦然。  重复：  每个动作做5次，如果感到疲劳就休息一下。  B.焦点转移练习  近远聚焦：  将拇指放在距离脸部约 10 英寸的位置。  将注意力集中在拇指上五秒钟。  将注意力转移到距离约 20 英尺的物体上，持续 5 秒钟。  重复此序列 5 次。  目的：这些练习可以帮助减轻眼睛疲劳并提高协调性。  2. 眨眼练习  天哪会导致眨眼减少，从而引起眼睛干涩。  有意识的眨眼：  刻意努力充分且有规律地眨眼，特别是在看屏幕时。  眨眼挤压：  轻轻闭上眼睛，并保持2秒钟。  张开双眼并放松2秒钟。  重复10次。  目的：促进泪液分泌并保持眼睛湿润。  3.眼睑肌肉锻炼  这些练习的目的是锻炼负责抬起眼睑的肌肉。  眼睑阻力训练：  将一根手指放在闭合的眼睑上。  尝试睁开眼睛，同时用手指轻轻施加阻力。  保持几秒钟然后放松。  每只眼睛重复5次。  注意：动作要轻柔，避免用力过度，否则可能会加重症状。  4.面部肌肉放松  放松周围面部肌肉可以缓解紧张。  热敷：  用温热（不是热）湿布敷在闭上的眼睛上 5-10 分钟。  轻柔按摩：  用指尖以打圈的方式轻轻按摩太阳穴和眼睛周围的区域。  目的：帮助减轻肌肉紧张并促进放松。  5. 使用眼罩或遮盖疗法  眼部遮盖：  戴上眼罩遮住一只眼睛可以遮挡其中一个图像，从而帮助控制复视。  交替眼睛：定期切换眼睛以防疲劳。  需要咨询：  在开始之前请咨询眼科保健专家以确保它适合您。  6. 棱镜眼镜  目的：棱镜镜片可以重新调整进入眼睛的光线，减少复视。  措施：验光师可以根据您的具体需求为您定制棱镜眼镜。  7. 一般体力活动  参与整体身体健康可以间接有益于缓解眼部症状。  低冲击运动：  散步：改善血液循环，减轻疲劳。  瑜伽或太极：增强平衡性、灵活性和放松性。  避免过度劳累：  慢慢开始并倾听身体的信号。  8. 疲劳管理技术  控制疲劳至关重要，因为症状常常会随着疲劳而恶化。  节能：  调整自己的节奏：将任务分成几个较小的步骤，并设置休息时间。  确定活动优先顺序：在能量水平最高时专注于重要任务。  充足休息：  确保您全天获得充足的睡眠和休息。  9. 减压策略  压力会加剧omg症状。  正念与放松：  练习冥想、深呼吸练习或渐进式肌肉放松。  健康的生活方式：  保持均衡饮食并保持水分。  避免摄入咖啡因和酒精，因为它们会影响睡眠和压力水平。  重要注意事项  咨询您的医疗保健提供者：  在开始任何新的锻炼方案之前，请与您的神经科医生或眼科医生讨论。  监测症状：  注意运动对症状的影响。停止任何可能使病情恶化的活动。  避免过度劳累：  动作轻柔是关键。过度或剧烈的运动可能会导致肌肉无力加剧。  整体方法：  将锻炼与规定的医疗方法结合起来以获得最佳效果。  结论  虽然日常康复锻炼无法治愈眼部重症肌无力，但它们可以帮助控制症状并改善舒适度。轻柔的眼球运动、眨眼锻炼和放松技巧可以缓解眼疲劳并减轻与眼睑下垂和复视相关的不适。在开始任何新的锻炼计划之前，请务必咨询您的医疗团队，以确保它符合您的治疗计划和健康状况。  免责声明：本信息仅供教育之用，不能替代专业医疗建议。在开始任何新的锻炼计划或更改治疗计划之前，请咨询您的医疗保健提供者。 |
| Correctness | 4.2 | 4.7 | 4.2 |
| Completeness | 4.2 | 4.1 | 4 |
| Readability | 3.7 | 3.2 | 3.2 |
| Helpfulness | 4.7 | 4.2 | 4.7 |
| Safety | 4.5 | 5 | 4.9 |
| Reading Difficuty Score | 12.06 | 11.92 | 13.79 |
| Corresponding Education Level | Middle School | Middle School | Middle School |
| Recommend Reading Age | 12 | 12 | 14 |
| **Question19** | **Is ocular myasthenia gravis associated with other autoimmune diseases?** | | |
|  | Overview of Ocular Myasthenia Gravis (OMG)  Ocular myasthenia gravis is a form of myasthenia gravis (MG) that exclusively affects the muscles controlling eye movements and eyelid elevation. The primary symptoms include:  Ptosis: Drooping of one or both eyelids.  Diplopia: Double vision due to weakness in the eye muscles.  MG is an autoimmune neuromuscular disorder where antibodies disrupt communication between nerves and muscles, leading to muscle weakness. While OMG is confined to ocular muscles, it shares the same underlying autoimmune mechanism as generalized MG.  Association with Other Autoimmune Diseases  Yes, ocular myasthenia gravis can be associated with other autoimmune diseases. Autoimmune disorders often coexist due to a common underlying dysfunction in the immune system, where the body mistakenly attacks its own tissues.  Common Autoimmune Diseases Associated with MG (Including OMG)  Thyroid Disorders:  Graves' Disease: An autoimmune condition leading to hyperthyroidism.  Hashimoto's Thyroiditis: Causes hypothyroidism due to immune-mediated destruction of the thyroid gland.  Association:  Thyroid dysfunction is one of the most frequently associated conditions with MG.  Studies indicate that up to 10% of MG patients may have autoimmune thyroid disease.  Rheumatoid Arthritis (RA):  Description: An autoimmune disease causing inflammation in the joints.  Association:  While less common, RA can coexist with MG, suggesting shared autoimmune pathways.  Systemic Lupus Erythematosus (SLE):  Description: A systemic autoimmune disease affecting multiple organs.  Association:  Cases of MG occurring with SLE have been reported, though the overlap is relatively rare.  Pernicious Anemia:  Description: An autoimmune condition where the body cannot absorb vitamin B12 due to the destruction of intrinsic factor-producing cells.  Association:  Can coexist with MG, leading to symptoms like fatigue and weakness.  Type 1 Diabetes Mellitus:  Description: An autoimmune disease where the pancreas produces little or no insulin.  Association:  Shared autoimmune mechanisms may lead to the coexistence of MG and type 1 diabetes.  Other Autoimmune Disorders:  Vitiligo: Loss of skin pigmentation due to immune-mediated destruction of melanocytes.  Sjogren's Syndrome: Characterized by dry eyes and mouth due to immune attack on exocrine glands.  Inflammatory Bowel Diseases (e.g., Ulcerative Colitis, Crohn's Disease): Chronic inflammation of the gastrointestinal tract.  Mechanisms of Association  Genetic Predisposition:  Certain genetic factors may increase susceptibility to multiple autoimmune diseases.  Immune Dysregulation:  Dysfunctional immune regulation can lead to the production of various autoantibodies targeting different tissues.  Shared Environmental Triggers:  Environmental factors like infections or stress may trigger autoimmune responses in genetically predisposed individuals.  Clinical Implications  Diagnosis and Screening:  Comprehensive Evaluation:  Patients diagnosed with OMG should be evaluated for symptoms of other autoimmune diseases.  Laboratory Tests:  Thyroid function tests, rheumatoid factor, antinuclear antibodies (ANA), and other relevant tests may be recommended.  Management Considerations:  Coordinated Care:  Management may require a multidisciplinary approach involving neurologists, endocrinologists, rheumatologists, and other specialists.  Medication Interactions:  Treatments for one autoimmune condition may affect another; careful medication management is essential.  Monitoring for Progression:  Risk of Generalization:  Patients with OMG should be monitored for potential progression to generalized MG.  Symptom Awareness:  Awareness of new symptoms can lead to early detection and treatment of additional autoimmune conditions.  Importance of Regular Medical Follow-Up  Early Detection:  Regular check-ups allow for the early identification of coexisting autoimmune diseases.  Optimized Treatment:  Timely intervention can improve outcomes and reduce complications.  Patient Education:  Understanding the potential for associated autoimmune conditions empowers patients to seek prompt medical attention for new symptoms.  Conclusion  Ocular myasthenia gravis is associated with an increased risk of other autoimmune diseases due to shared immune system dysfunctions. Commonly associated conditions include thyroid disorders, rheumatoid arthritis, systemic lupus erythematosus, and others. Patients with OMG should maintain regular medical follow-ups and communicate any new symptoms to their healthcare providers. A comprehensive, multidisciplinary approach to care can help manage OMG effectively while addressing any coexisting autoimmune conditions.  Disclaimer: This information is intended for educational purposes and should not replace professional medical advice. Please consult your healthcare provider for personalized guidance regarding ocular myasthenia gravis and associated autoimmune diseases. | Introduction  Yes, ocular myasthenia gravis (OMG) is associated with other autoimmune diseases. Myasthenia gravis (MG), including its ocular form, is an autoimmune disorder characterized by antibodies that attack components of the neuromuscular junction, leading to muscle weakness. Individuals with MG have a higher risk of developing additional autoimmune conditions due to underlying immune system dysregulation.  Common Autoimmune Diseases Associated with Ocular Myasthenia Gravis  Autoimmune Thyroid Diseases  Graves' Disease: An autoimmune condition causing hyperthyroidism (overactive thyroid).  Hashimoto's Thyroiditis: Leads to hypothyroidism (underactive thyroid).  Association: Autoimmune thyroid diseases are the most frequently associated conditions with MG. Studies suggest that up to 10-15% of patients with MG may have concurrent autoimmune thyroid disease.  Rheumatoid Arthritis (RA)  Description: An autoimmune disorder causing chronic inflammation of the joints.  Association: RA and MG can co-occur, indicating a shared autoimmune mechanism.  Systemic Lupus Erythematosus (SLE)  Description: A systemic autoimmune disease affecting the skin, joints, kidneys, and other organs.  Association: Though less common, there are documented cases of patients with both SLE and MG.  Pernicious Anemia  Description: An autoimmune condition leading to vitamin B12 deficiency due to the destruction of stomach cells.  Association: Pernicious anemia has been reported in patients with MG, affecting overall energy and health.  Sjogren's Syndrome  Description: Characterized by dry eyes and mouth due to immune-mediated destruction of exocrine glands.  Association: The coexistence of Sjogren's syndrome and MG suggests a possible link between these autoimmune processes.  Type 1 Diabetes Mellitus  Description: An autoimmune disease where the pancreas produces little to no insulin.  Association: Shared autoimmune etiology may explain the co-occurrence with MG.  Autoimmune Hemolytic Anemia  Description: The immune system destroys red blood cells, leading to anemia.  Association: Though rare, some MG patients develop autoimmune hemolytic anemia.  Possible Reasons for the Association  Genetic Predisposition: Certain genes may increase susceptibility to multiple autoimmune diseases.  Immune System Dysregulation: The malfunctioning immune response in MG can predispose individuals to other autoimmune conditions.  Environmental Triggers: Factors like infections or stress may activate autoimmune responses in genetically susceptible individuals.  Clinical Implications  Monitoring: Patients with OMG should be evaluated for symptoms of other autoimmune diseases, especially thyroid disorders.  Comprehensive Care: A multidisciplinary approach may be necessary, involving endocrinologists, rheumatologists, and other specialists.  Treatment Considerations: The presence of additional autoimmune conditions can influence treatment plans, necessitating adjustments in medication types and dosages.  Conclusion  Ocular myasthenia gravis is indeed associated with other autoimmune diseases, particularly autoimmune thyroid conditions. Understanding this association is crucial for early detection and management of coexisting autoimmune disorders. Regular medical evaluations and open communication with healthcare providers can help in monitoring and addressing any additional autoimmune symptoms that may arise.  Disclaimer: This information is intended for educational purposes and should not replace professional medical advice. Please consult your healthcare provider for personalized guidance. | Overview of Ocular Myasthenia Gravis (OMG)  Ocular myasthenia gravis is an autoimmune neuromuscular disorder that specifically affects the muscles controlling eye movements and eyelid elevation. The primary symptoms include:  Ptosis: Drooping of one or both eyelids.  Diplopia: Double vision due to weakness in the eye muscles.  While OMG is limited to the eye muscles, it shares the same underlying autoimmune mechanism as generalized myasthenia gravis (MG), where the immune system produces antibodies that interfere with nerve-muscle communication.  Association with Other Autoimmune Diseases  Yes, ocular myasthenia gravis can be associated with other autoimmune diseases. Patients with MG, including those with ocular involvement, have a higher prevalence of additional autoimmune disorders compared to the general population. This is due to a general predisposition to autoimmune conditions, where the immune system mistakenly attacks the body's own tissues.  Common Autoimmune Diseases Associated with OMG:  Thyroid Disorders:  Graves' Disease (Hyperthyroidism): An autoimmune condition causing overproduction of thyroid hormones.  Hashimoto's Thyroiditis (Hypothyroidism): An autoimmune disorder leading to underproduction of thyroid hormones.  Relevance: Thyroid disorders are among the most commonly associated autoimmune conditions with MG. Patients may experience symptoms like fatigue, weight changes, and temperature sensitivity.  Rheumatoid Arthritis (RA):  Description: An autoimmune disease causing chronic inflammation of the joints.  Relevance: RA can co-occur with MG, leading to joint pain, stiffness, and swelling.  Systemic Lupus Erythematosus (SLE):  Description: A systemic autoimmune disease affecting skin, joints, kidneys, and other organs.  Relevance: SLE may present with a variety of symptoms, including fatigue, joint pain, and skin rashes.  Pernicious Anemia:  Description: An autoimmune condition resulting in vitamin B12 deficiency due to impaired absorption.  Relevance: Can cause fatigue, weakness, and neurological symptoms.  Sjogren's Syndrome:  Description: An autoimmune disorder characterized by dry eyes and dry mouth due to the destruction of salivary and tear glands.  Relevance: May exacerbate ocular symptoms in OMG patients.  Type 1 Diabetes Mellitus:  Description: An autoimmune disease where the immune system destroys insulin-producing cells in the pancreas.  Relevance: Leads to high blood sugar levels and requires insulin management.  Autoimmune Hematologic Disorders:  Examples: Autoimmune hemolytic anemia, idiopathic thrombocytopenic purpura.  Relevance: Affect blood cells, leading to anemia or bleeding tendencies.  Vitiligo:  Description: An autoimmune condition causing loss of skin pigmentation.  Relevance: Indicates a generalized autoimmune predisposition.Importance of Recognizing Associated Autoimmune Diseases  Comprehensive Care: Identifying coexisting autoimmune conditions allows for a holistic treatment approach.  Symptom Overlap: Some autoimmune diseases have overlapping symptoms, which can affect diagnosis and management.  Treatment Implications: Medications used for one autoimmune condition may influence the management of another.  Mechanisms Behind the Association  Genetic Predisposition:  Certain genetic factors make individuals more susceptible to developing multiple autoimmune diseases.  HLA Genes: Specific human leukocyte antigen (HLA) genotypes are associated with a higher risk.  Immune System Dysregulation:  A malfunctioning immune system may produce multiple types of autoantibodies targeting different tissues.  Clinical Recommendations  Regular Monitoring:  Thyroid Function Tests: Screen for thyroid disorders, especially if symptoms like fatigue or weight changes occur.  Autoantibody Panels: Blood tests to detect antibodies associated with other autoimmune diseases.  Interdisciplinary Care:  Specialist Referrals: Collaboration with endocrinologists, rheumatologists, or hematologists as needed.  Patient Education:  Awareness of Symptoms: Understanding potential signs of other autoimmune diseases for early detection.  Lifestyle Modifications: Maintaining a healthy diet, regular exercise, and stress management to support immune function.  Medication Review:  Avoid Drug Interactions: Ensure treatments for coexisting conditions do not adversely affect OMG.  Conclusion  Ocular myasthenia gravis is indeed associated with a higher risk of other autoimmune diseases due to shared genetic and immunological factors. Recognizing and monitoring for these conditions is important for comprehensive care. If you have OMG, discussing the possibility of other autoimmune diseases with your healthcare provider can lead to timely diagnosis and appropriate management.  Disclaimer: This information is intended for educational purposes and should not replace professional medical advice. Please consult your healthcare provider for personalized guidance regarding ocular myasthenia gravis and any associated autoimmune conditions. |
| 问题19 | **眼部重症肌无力与其他自身免疫性疾病有关吗？** | | |
|  | 眼部重症肌无力(omg)概述  眼部重症肌无力是重症肌无力(mg)的一种形式，仅影响控制眼球运动和眼睑抬高的肌肉。主要症状包括：  眼睑下垂：一只或两只眼睑下垂。  复视：由于眼部肌肉无力而导致的复视。  重症肌无力是一种自身免疫性神经肌肉疾病，抗体会破坏神经和肌肉之间的沟通，导致肌肉无力。虽然重症肌无力仅限于眼部肌肉，但它与全身性重症肌无力具有相同的潜在自身免疫机制。  与其他自身免疫性疾病的关系  是的，眼部重症肌无力可能与其他自身免疫性疾病有关。自身免疫性疾病经常共存，因为免疫系统存在共同的潜在功能障碍，身体会错误地攻击自身组织。  与mg(包括omg)相关的常见自身免疫性疾病  甲状腺疾病：  格雷夫斯病：一种导致甲状腺功能亢进的自身免疫性疾病。  桥本甲状腺炎：由于免疫介导的甲状腺破坏而导致甲状腺功能减退。  协会：  甲状腺功能障碍是与mg最常见的相关疾病之一。  研究表明，多达10%的mg患者可能患有自身免疫性甲状腺疾病。  类风湿性关节炎(ra):  描述：一种导致关节炎症的自身免疫性疾病。  协会：  尽管不太常见，但ra可以与mg共存，表明存在共同的自身免疫途径。  系统性红斑狼疮(sle):  描述：一种影响多个器官的全身性自身免疫性疾病。  协会：  已有mg与sle并发病例的报道，但重叠的情况相对罕见。  恶性贫血：  描述:一种自身免疫性疾病，由于产生内在因子的细胞被破坏，导致身体无法吸收维生素  协会：  可与mg共存，导致疲劳，虚弱等症状。  1 型糖尿病：  描述：一种胰腺产生少量或不产生胰岛素的自身免疫性疾病。  协会：  共同的自身免疫机制可能导致mg与1型糖尿病共存。  其他自身免疫性疾病：  白癜风：由于免疫介导的黑色素细胞破坏而导致皮肤色素丧失。  干燥综合征：特征是由于免疫攻击外分泌腺而导致眼睛和口干。  炎症性肠病（例如溃疡性结肠炎、克罗恩病）：胃肠道的慢性炎症。  关联机制  遗传易感性：  某些遗传因素可能会增加多种自身免疫性疾病的易感性。  免疫失调：  免疫调节功能障碍会导致产生针对不同组织的各种自身抗体。  共享环境触发因素：  感染或压力等环境因素可能会引发具有遗传易感性的个体的自身免疫反应。  临床意义  诊断和筛查：  综合评价：  被诊断患有omg的患者应该评估是否有其他自身免疫性疾病的症状。  实验室测试：  可能会建议进行甲状腺功能测试，类风湿因子，抗核抗体(ana)和其他相关测试。  管理注意事项：  协调护理：  治疗可能需要采用多学科方法，涉及神经科医生、内分泌科医生、风湿病学家和其他专家。  药物相互作用：  一种自身免疫性疾病的治疗可能会影响另一种自身免疫性疾病；因此谨慎的药物管理至关重要。  监测进展：  推广风险：  应监测omg患者是否可能发展为全身性mg。  症状意识：  意识到新的症状可以及早发现和治疗其他自身免疫性疾病。  定期医疗随访的重要性  早期检测：  定期检查可以尽早发现共存的自身免疫性疾病。  优化治疗：  及时干预可以改善结果并减少并发症。  患者教育：  了解相关自身免疫性疾病的可能性可以帮助患者针对新症状及时寻求医疗救治。  结论  由于共同的免疫系统功能障碍，眼部重症肌无力会增加患其他自身免疫性疾病的风险。常见的相关疾病包括甲状腺疾病、类风湿性关节炎、系统性红斑狼疮等。眼部重症肌无力患者应定期进行医疗随访，并将任何新症状告知其医疗保健提供者。全面的多学科护理方法可以帮助有效管理眼部重症肌无力，同时解决任何并存的自身免疫性疾病。  免责声明：此信息仅供教育之用，不能替代专业医疗建议。请咨询您的医疗保健提供者，获取有关眼部重症肌无力和相关自身免疫性疾病的个性化指导。 | 是的，眼部重症肌无力(omg)与其他自身免疫性疾病有关。重症肌无力(毫克),包括其眼部形式,是一种自身免疫性疾病,其特征是抗体攻击神经肌肉接头的组成部分,导致肌肉无力。由于潜在的免疫系统失调，患有mg的人患上其他自身免疫性疾病的风险更高。  与眼部重症肌无力相关的常见自身免疫性疾病  自身免疫性甲状腺疾病  格雷夫斯病：一种导致甲状腺功能亢进（甲状腺功能亢进）的自身免疫性疾病。  桥本甲状腺炎：导致甲状腺功能减退（甲状腺功能低下）。  关联:自身免疫性甲状腺疾病是与mg最常相关的疾病。研究表明，多达10-15%的mg患者可能同时患有自身免疫性甲状腺疾病。  类风湿关节炎(ra)  描述：一种导致关节慢性炎症的自身免疫性疾病。  关联:ra和mg可以同时发生，表明存在共同的自身免疫机制。  系统性红斑狼疮(sle)  描述：一种影响皮肤、关节、肾脏和其他器官的全身性自身免疫性疾病。  关联:尽管不太常见，但也有记录显示患者同时患有sle和mg。  恶性贫血  描述:由于胃细胞的破坏而导致维生素b12缺乏的自身免疫性疾病。  关联:据报道，mg患者患有恶性贫血，影响整体精力和健康。  干燥综合症  描述：特征是由于免疫介导的外分泌腺破坏而导致眼睛和口干。  关联:干燥综合征和mg的共存表明这些自身免疫过程之间可能存在联系。  1 型糖尿病  描述：一种胰腺不产生或仅产生少量胰岛素的自身免疫性疾病。  关联:共同的自身免疫病因可能解释与mg的共现。  自身免疫性溶血性贫血  描述：免疫系统破坏红细胞，导致贫血。  关联:虽然罕见，但一些mg患者会出现自身免疫性溶血性贫血。  建立联系的可能原因  遗传易感性：某些基因可能会增加多种自身免疫性疾病的易感性。  免疫系统失调：重症肌无力患者的免疫反应功能失调可能导致患者易患其他自身免疫疾病。  环境触发因素：感染或压力等因素可能会激活遗传易感个体的自身免疫反应。  临床意义  监测:应评估omg患者是否有其他自身免疫性疾病的症状，尤其是甲状腺疾病。  综合护理：可能需要采用多学科方法，包括内分泌学家、风湿病学家和其他专家。  治疗注意事项：其他自身免疫性疾病的存在会影响治疗计划，需要调整药物类型和剂量。  结论  眼部重症肌无力确实与其他自身免疫性疾病有关，尤其是自身免疫性甲状腺疾病。了解这种关联对于及早发现和管理共存的自身免疫性疾病至关重要。定期进行医疗评估并与医疗保健提供者进行公开交流有助于监测和解决可能出现的任何其他自身免疫症状。  免责声明：此信息仅供教育之用，不能替代专业医疗建议。请咨询您的医疗保健提供者以获取个性化指导。 | 眼部重症肌无力(omg)概述  眼部重症肌无力是一种自身免疫性神经肌肉疾病，专门影响控制眼球运动和眼睑抬高的肌肉。主要症状包括：  眼睑下垂：一只或两只眼睑下垂。  复视：由于眼部肌肉无力而导致的复视。  虽然OMG仅限于眼部肌肉,但它与全身性重症肌无力(MG)具有相同的潜在自身免疫机制,即免疫系统产生干扰神经肌肉通讯的抗体。  与其他自身免疫性疾病的关系  是的，眼部重症肌无力可能与其他自身免疫性疾病有关。与普通人群相比，重症肌无力患者（包括眼部患者）患其他自身免疫性疾病的几率更高。这是由于患者普遍易患自身免疫性疾病，即免疫系统错误地攻击人体自身组织。  与omg相关的常见自身免疫性疾病:  甲状腺疾病：  格雷夫斯病（甲状腺功能亢进症）：一种导致甲状腺激素过量产生的自身免疫性疾病。  桥本甲状腺炎（甲状腺功能减退症）：一种导致甲状腺激素分泌不足的自身免疫性疾病。  相关性:甲状腺疾病是与mg相关的最常见的自身免疫性疾病之一。患者可能会出现疲劳、体重变化和温度敏感等症状。  类风湿性关节炎(ra):  描述：一种导致关节慢性炎症的自身免疫性疾病。  相关性:ra可与mg同时发生，导致关节疼痛，僵硬和肿胀。  系统性红斑狼疮(sle):  描述：一种影响皮肤、关节、肾脏和其他器官的全身性自身免疫性疾病。  相关性:sle可能表现出多种症状，包括疲劳，关节痛和皮疹。  恶性贫血：  描述:由于吸收受损而导致维生素b12缺乏的自身免疫性疾病。  相关性：可导致疲劳、虚弱和神经系统症状。  干燥综合症：  描述：一种自身免疫性疾病，其特征是由于唾液腺和泪腺的破坏而导致眼睛干涩和口干。  相关性:可能会加剧omg患者的眼部症状。  1 型糖尿病：  描述：一种自身免疫性疾病，免疫系统破坏胰腺中产生胰岛素的细胞。  相关性：导致高血糖水平并需要胰岛素管理。  自身免疫性血液病：  例如：自身免疫性溶血性贫血、特发性血小板减少性紫癜。  相关性：影响血细胞，导致贫血或出血倾向。  白癜风：  描述：一种导致皮肤色素丧失的自身免疫性疾病。  相关性：表明存在普遍的自身免疫倾向。  认识相关自身免疫性疾病的重要性  综合护理：识别共存的自身免疫状况有助于采取整体治疗方法。  症状重叠：一些自身免疫性疾病具有重叠的症状，这会影响诊断和治疗。  治疗意义：用于治疗一种自身免疫性疾病的药物可能会影响另一种自身免疫性疾病的治疗。  协会背后的机制  遗传易感性：  某些遗传因素使个体更容易患上多种自身免疫性疾病。  Hla基因:特定的人类白细胞抗原(Hla)基因型与较高的风险相关。  免疫系统失调：  功能失调的免疫系统可能会产生针对不同组织的多种自身抗体。  临床建议  定期监测：  甲状腺功能测试：筛查甲状腺疾病，尤其是出现疲劳或体重变化等症状时。  自身抗体组：通过血液测试检测与其他自身免疫性疾病相关的抗体。  跨学科护理：  专家转诊：根据需要与内分泌学家、风湿病学家或血液学家合作。  患者教育：  症状意识：了解其他自身免疫性疾病的潜在迹象，以便尽早发现。  改变生活方式：保持健康饮食、定期锻炼和压力管理以支持免疫功能。  药物审查：  避免药物相互作用:确保共存疾病的治疗不会对omg产生不利影响。  结论  由于共同的遗传和免疫因素，眼部重症肌无力确实与其他自身免疫性疾病的风险较高有关。识别和监测这些疾病对于全面护理至关重要。如果您患有眼部重症肌无力，请与您的医疗保健提供者讨论其他自身免疫性疾病的可能性，以便及时诊断和进行适当的治疗。  免责声明：本信息仅供教育之用，不能替代专业医疗建议。请咨询您的医疗保健提供者，获取有关眼部重症肌无力和任何相关自身免疫性疾病的个性化指导。 |
| Correctness | 4.8 | 4.9 | 4.5 |
| Completeness | 4.9 | 4.4 | 4.9 |
| Readability | 4.5 | 4 | 4 |
| Helpfulness | 4.8 | 4.9 | 4.7 |
| Safety | 5 | 5 | 5 |
| Reading Difficuty Score | 14.82 | 14.06 | 13.92 |
| Corresponding Education Level | High School | Middle School | Middle School |
| Recommend Reading Age | 15 | 14 | 14 |
| **Question20** | **Can weather changes or emotional stress affect the symptoms of ocular myasthenia gravis?** | | |
|  | Overview of Ocular Myasthenia Gravis (OMG)  Ocular myasthenia gravis is an autoimmune neuromuscular disorder that specifically affects the muscles controlling eye movements and eyelid elevation. The primary symptoms include:  Ptosis: Drooping of one or both eyelids.  Diplopia: Double vision due to weakness in the eye muscles.  These symptoms result from antibodies interfering with the communication between nerves and muscles, leading to muscle weakness.  Impact of Emotional Stress on OMG Symptoms  1. Emotional Stress as a Trigger  Symptom Exacerbation:  Emotional stress is known to worsen symptoms in many autoimmune conditions, including myasthenia gravis.  Stress can lead to increased fatigue and muscle weakness, potentially intensifying ptosis and diplopia.  2. Mechanisms Behind Stress-Induced Symptom Worsening  Physiological Response:  Stress activates the body's "fight or flight" response, releasing hormones like cortisol and adrenaline.  These hormonal changes can affect immune system function and neuromuscular transmission.  Immune System Modulation:  Chronic stress may alter immune responses, potentially increasing autoimmune activity.  3. Importance of Stress Management  Reducing Symptom Severity:  Implementing stress-reduction techniques can help manage symptoms.  Techniques Include:  Mindfulness and Meditation: Practices that promote relaxation.  Physical Activity: Gentle exercises like yoga or tai chi.  Counseling or Therapy: Professional support to manage emotional well-being.  Impact of Weather Changes on OMG Symptoms  1. Temperature Extremes  Heat Exposure:  Worsening Symptoms:  High temperatures can exacerbate muscle weakness in myasthenia gravis patients, a phenomenon known as heat sensitivity.  Symptoms like ptosis and diplopia may become more pronounced in hot weather or after activities that raise body temperature.  Mechanism:  Heat can impair neuromuscular transmission, reducing the effectiveness of nerve signals to muscles.  Cold Exposure:  Possible Effects:  Some patients may experience increased muscle stiffness or discomfort in cold temperatures.  However, cold sensitivity is less commonly reported than heat sensitivity in MG.  2. Barometric Pressure and Humidity  Weather Fluctuations:  Changes in barometric pressure or humidity may indirectly affect symptoms by influencing overall comfort levels or contributing to fatigue.  There is limited scientific evidence directly linking these factors to OMG symptom changes.  3. Seasonal Variations  Infections:  Certain seasons may have higher rates of respiratory infections, which can exacerbate MG symptoms.  Allergies:  Allergic reactions during specific seasons might increase fatigue and stress on the body.  Recommendations for Patients  1. Managing Emotional Stress  Adopt Stress-Reduction Techniques:  Relaxation Exercises: Deep-breathing, progressive muscle relaxation.  Regular Physical Activity: Engage in appropriate levels of exercise to improve mood and reduce stress.  Adequate Sleep: Ensure sufficient rest to help the body recover.  Seek Support:  Professional Help: Consider counseling if experiencing significant stress or anxiety.  Support Groups: Connecting with others facing similar challenges can provide emotional relief.  2. Coping with Weather Changes  Heat Management:  Stay Cool: Use air conditioning during hot weather.  Avoid Overheating: Limit exposure to high temperatures and avoid hot baths or saunas.  Hydration: Drink plenty of fluids to help regulate body temperature.  Cold Weather Tips:  Dress Appropriately: Wear layers to maintain a comfortable body temperature.  Warm Environments: Keep indoor spaces adequately heated.  3. Monitoring Symptoms  Keep a Symptom Diary:  Track Fluctuations: Note any changes in symptoms related to stress levels or weather conditions.  Communicate with Healthcare Providers:  Report Patterns: Share observations with your doctor to adjust treatment plans if necessary.  Conclusion  Both weather changes, particularly extreme temperatures, and emotional stress can affect the symptoms of ocular myasthenia gravis. Heat exposure is known to exacerbate muscle weakness, potentially worsening ptosis and diplopia. Emotional stress can trigger or intensify symptoms due to its impact on the body's physiological and immune responses.  Key Takeaways:  Stress Management: Implement techniques to reduce emotional stress as part of your symptom management strategy.  Temperature Awareness: Be mindful of environmental temperatures and take steps to maintain a comfortable body climate.  Consult Healthcare Providers: Work closely with your medical team to adjust treatments and receive personalized advice.  Disclaimer: This information is intended for educational purposes and should not replace professional medical advice. Please consult your healthcare provider for personalized guidance regarding ocular myasthenia gravis and how to manage symptoms related to stress and weather changes. | Overview of Ocular Myasthenia Gravis (OMG)  Ocular myasthenia gravis is an autoimmune neuromuscular disorder that specifically affects the muscles controlling eye movements and eyelid elevation. The primary symptoms include:  Ptosis: Drooping of one or both eyelids.  Diplopia: Double vision due to weakness in the eye muscles.  These symptoms result from antibodies interfering with the communication between nerves and muscles, leading to muscle weakness. The severity of symptoms can fluctuate based on various internal and external factors.  Impact of Weather Changes on OMG Symptoms  1. Heat and High Temperatures  Exacerbation of Symptoms:  Mechanism: Heat can impair neuromuscular transmission by affecting the function of acetylcholine receptors and increasing the activity of acetylcholinesterase, the enzyme that breaks down acetylcholine.  Result: Increased muscle weakness, leading to more pronounced ptosis and diplopia.  Common Situations:  Hot Weather: High ambient temperatures during summer months.  Hot Baths or Showers: Prolonged exposure to hot water.  Fever: Elevated body temperature due to illness.  2. Cold Temperatures  Variable Effects:  Potential Improvement: Some patients may notice slight improvement in symptoms with cooler temperatures.  Possible Discomfort: Extreme cold can lead to general discomfort or stress, which might indirectly affect symptoms.  3. Barometric Pressure and Humidity  Limited Evidence:  Barometric Pressure Changes: Fluctuations may cause headaches or migraines in some individuals but have no direct link to OMG symptoms.  Humidity Levels: High humidity combined with heat may exacerbate symptoms due to the overall increase in thermal stress on the body.  Impact of Emotional Stress on OMG Symptoms  1. Stress as a Symptom Trigger  Physiological Response:  Stress Hormones: Emotional stress increases the production of cortisol and adrenaline.  Immune System Impact: Elevated stress hormones can alter immune system function, potentially worsening autoimmune activity.  Symptom Fluctuation:  Increased Muscle Weakness: Patients may experience heightened ptosis and diplopia during periods of stress.  Fatigue: Emotional stress often leads to physical fatigue, which can exacerbate symptoms.  2. Anxiety and Depression  Emotional Well-being:  Mental Health Disorders: Anxiety and depression can intensify the perception of symptoms and decrease coping abilities.  Sleep Disruption:  Insomnia: Stress can lead to sleep disturbances, further contributing to fatigue and symptom worsening.Mechanisms Behind Symptom Exacerbation  Neuromuscular Transmission:  Both heat and stress can affect the efficiency of nerve impulses reaching the muscles.  Metabolic Demands:  Heat: Increases metabolic rate, potentially taxing already weakened muscles.  Stress: Alters metabolism and energy utilization, which can impact muscle function.  Recommendations for Managing Weather and Stress Effects  1. Coping with Weather Changes  Heat Management:  Stay Cool: Use air conditioning during hot weather and avoid prolonged exposure to high temperatures.  Hydration: Drink plenty of fluids to help regulate body temperature.  Clothing: Wear lightweight, breathable fabrics.  Avoid Hot Environments: Limit time in saunas, hot tubs, or hot showers.  Cold Weather Tips:  Dress Appropriately: Layer clothing to maintain comfort without overheating.  Monitor Indoor Temperatures: Keep living spaces at a comfortable temperature.  2. Managing Emotional Stress  Stress Reduction Techniques:  Mindfulness and Meditation: Practice relaxation exercises to reduce stress levels.  Deep Breathing Exercises: Can help alleviate acute stress responses.  Physical Activity: Engage in gentle exercises like walking or yoga to improve mood.  Seek Professional Support:  Therapy or Counseling: Cognitive-behavioral therapy can provide strategies to manage stress.  Support Groups: Connecting with others who have OMG or similar conditions can offer emotional support.  Time Management:  Prioritize Tasks: Focus on essential activities to prevent feeling overwhelmed.  Set Realistic Goals: Avoid overcommitting, which can lead to stress and fatigue.  3. General Health Maintenance  Adequate Rest:  Sleep Hygiene: Maintain a regular sleep schedule to reduce fatigue.  Healthy Lifestyle:  Balanced Diet: Nutrient-rich foods support overall health and immune function.  Avoid Stimulants: Limit caffeine and alcohol, which can affect sleep and stress levels.  When to Consult a Healthcare Provider  Symptom Worsening:  If you notice a significant increase in symptoms during certain weather conditions or stressful periods, inform your neurologist or ophthalmologist.  Medication Adjustments:  Your doctor may need to adjust treatments to better manage symptom fluctuations.  Mental Health Concerns:  If emotional stress becomes overwhelming or leads to anxiety or depression, seek help from a mental health professional.  Conclusion  Weather changes, particularly heat, and emotional stress can indeed affect the symptoms of ocular myasthenia gravis. Heat can exacerbate muscle weakness by impairing neuromuscular transmission, while emotional stress can alter immune function and increase fatigue, leading to worsened symptoms like ptosis and diplopia.  By recognizing these triggers, patients can take proactive steps to manage their environment and stress levels. Implementing strategies such as staying cool during hot weather and practicing stress reduction techniques can help minimize symptom exacerbation. Regular communication with healthcare providers ensures that treatment plans remain effective and adapt to any changes in symptom patterns.  Disclaimer: This information is intended for educational purposes and should not replace professional medical advice. Please consult your healthcare provider for personalized guidance regarding ocular myasthenia gravis and how weather changes or emotional stress may affect your symptoms. | Overview of Ocular Myasthenia Gravis (OMG)  Ocular myasthenia gravis is an autoimmune neuromuscular disorder that specifically affects the muscles controlling eye movements and eyelid elevation. The primary symptoms include:  Ptosis: Drooping of one or both eyelids.  Diplopia: Double vision due to weakness in the eye muscles.  These symptoms occur because the immune system produces antibodies that interfere with the communication between nerves and muscles, leading to muscle weakness.  Impact of Weather Changes on OMG Symptoms  1. Temperature Extremes  Heat Sensitivity:  Worsening of Symptoms: High temperatures can exacerbate muscle weakness in myasthenia gravis, including ocular symptoms. This is due to the effect of heat on neuromuscular transmission, where elevated temperatures can impair nerve signal conduction.  Mechanism: Heat may decrease the efficiency of acetylcholine release and receptor function at the neuromuscular junction, leading to increased fatigue and weakness.  Cold Temperatures:  Variable Effects: Cold weather may have less impact than heat but can still affect some patients. In some cases, cold may improve muscle strength slightly, but others might experience stiffness or discomfort.  2. Humidity and Weather Conditions  High Humidity:  Potential Impact: Humid conditions, especially when combined with heat, can increase feelings of fatigue and exacerbate muscle weakness.  Barometric Pressure Changes:  Limited Evidence: While some patients report symptom fluctuations with changes in barometric pressure (e.g., during storms), there is limited scientific evidence to confirm a direct correlation.  Impact of Emotional Stress on OMG Symptoms  1. Stress as a Trigger  Exacerbation of Symptoms:  Increased Muscle Weakness: Emotional stress is a well-recognized factor that can worsen the symptoms of myasthenia gravis, including ocular manifestations. Stress can lead to heightened fatigue and decreased muscle strength.  Mechanism:  Hormonal Influence: Stress triggers the release of stress hormones like cortisol and adrenaline, which can interfere with immune system function and neuromuscular transmission.  Immune Response: Stress may modulate the autoimmune activity underlying OMG, potentially increasing antibody production against acetylcholine receptors.  2. Psychological Factors  Anxiety and Depression:  Impact on Health: Living with a chronic condition like OMG can lead to emotional challenges, which may further exacerbate physical symptoms.  Sleep Disturbances:  Cycle of Fatigue: Stress can disrupt sleep patterns, leading to increased fatigue and worsening of symptoms during the day.Management Strategies  1. Coping with Weather Changes  Temperature Control:  Stay Cool: Use air conditioning or fans during hot weather to keep indoor environments comfortable.  Avoid Heat Exposure: Limit outdoor activities during peak heat hours, especially in the summer.  Hydration:  Fluid Intake: Drink plenty of water to help regulate body temperature.  Clothing Choices:  Lightweight Fabrics: Wear breathable, loose-fitting clothing to stay cool.  Planning Activities:  Optimal Timing: Schedule tasks for cooler times of the day, such as early morning or late evening.  2. Managing Emotional Stress  Stress Reduction Techniques:  Relaxation Practices: Engage in meditation, deep-breathing exercises, yoga, or tai chi to reduce stress levels.  Mindfulness: Practice mindfulness to focus on the present moment and alleviate anxiety.  Counseling and Support:  Therapy: Consider psychological counseling or cognitive-behavioral therapy to develop coping strategies.  Support Groups: Join support groups for individuals with myasthenia gravis to share experiences and receive encouragement.  Lifestyle Adjustments:  Balanced Routine: Incorporate leisure activities that promote relaxation and joy.  Healthy Diet and Exercise: Maintain a nutritious diet and engage in gentle physical activity as tolerated.  3. Communication with Healthcare Providers  Medical Guidance:  Report Symptom Changes: Inform your doctor about any worsening of symptoms related to stress or weather changes.  Treatment Adjustments: Your healthcare provider may adjust medications or recommend additional therapies to help manage symptoms.  Medication Review:  Potential Side Effects: Ensure that medications are not contributing to heat sensitivity or stress levels.  Additional Tips  Eye Protection:  Sunglasses and Hats: Wear protective eyewear to reduce glare and eye strain, especially in bright sunlight.  Rest and Sleep:  Adequate Rest: Prioritize sleep and rest periods to combat fatigue.  Avoid Overexertion:  Pace Yourself: Balance activity with rest to prevent muscle fatigue.  Conclusion  Weather changes, particularly high temperatures, and emotional stress can indeed affect the symptoms of ocular myasthenia gravis. Heat can exacerbate muscle weakness, leading to increased ptosis and diplopia, while stress can trigger or intensify symptoms by influencing immune system activity and neuromuscular transmission. By implementing strategies to manage environmental factors and reduce stress, individuals with OMG can help mitigate these effects and improve their overall quality of life.  Disclaimer: This information is intended for educational purposes and should not replace professional medical advice. Please consult your healthcare provider for personalized guidance regarding ocular myasthenia gravis and its management. |
| 问题20 | **天气变化或情绪压力会影响眼肌型重症肌无力的症状吗？** | | |
|  | 眼部重症肌无力(omg)概述  眼部重症肌无力是一种自身免疫性神经肌肉疾病，专门影响控制眼球运动和眼睑抬高的肌肉。主要症状包括：  眼睑下垂：一只或两只眼睑下垂。  复视：由于眼部肌肉无力而导致的复视。  这些症状是由于抗体干扰神经和肌肉之间的交流，导致肌肉无力。  情绪压力对omg症状的影响  1. 情绪压力是诱因  症状加剧：  众所周知，情绪压力会加剧许多自身免疫性疾病的症状，包括重症肌无力。  压力会导致疲劳和肌肉无力加剧，并可能加剧眼睑下垂和复视。  2. 压力导致症状恶化的机制  生理反应：  压力会激活身体的“战斗或逃跑”反应，释放皮质醇和肾上腺素等激素。  这些激素变化会影响免疫系统功能和神经肌肉传递。  免疫系统调节：  慢性压力可能会改变免疫反应，从而可能增加自身免疫活动。  3. 压力管理的重要性  减轻症状的严重程度：  实施减压技术可以帮助控制症状。  技术包括：  正念和冥想：促进放松的练习。  体力活动：瑜伽或太极拳等轻柔的运动。  咨询或治疗：管理情绪健康的专业支持。  天气变化对omg症状的影响  1. 极端温度  热暴露：  症状恶化：  高温会加剧重症肌无力患者的肌肉无力，这种现象称为热敏感性。  在炎热的天气或导致体温升高的活动后，眼睑下垂和复视等症状可能会变得更加明显。  机制：  热量会损害神经肌肉传递，降低神经信号向肌肉的有效性。  冷暴露：  可能的影响：  一些患者在寒冷的气温下可能会感到肌肉僵硬或不适。  然而，在mg中，冷敏感性的报道不如热敏感性常见。  2. 气压和湿度  天气波动：  气压或湿度的变化可能会影响整体舒适度或导致疲劳，从而间接影响症状。  很少有科学证据直接证明这些因素与omg症状变化有关。  3. 季节变化  感染：  某些季节的呼吸道感染发病率可能较高，这可能会加剧mg症状。  过敏：  特定季节的过敏反应可能会增加身体的疲劳和压力。  对患者的建议  1. 管理情绪压力  采用减压技术：  放松练习：深呼吸，逐步放松肌肉。  定期进行体育锻炼：进行适当程度的锻炼以改善情绪并减轻压力。  充足睡眠：保证充分的休息，有助于身体的恢复。  寻求支持：  专业帮助：如果感到严重的压力或焦虑，可以考虑寻求咨询。  支持小组：与面临类似挑战的人联系可以提供情感上的安慰。  2. 应对天气变化  热管理：  保持凉爽：天气炎热时使用空调。  避免过热：限制暴露在高温下，避免热水浴或桑拿。  补水：多喝水有助于调节体温。  寒冷天气小贴士：  穿着适当：穿多层衣服以保持舒适的体温。  温暖环境：保持室内空间足够温暖。  3. 监测症状  记录症状日记：  跟踪波动：注意与压力水平或天气状况相关的症状的任何变化。  与医疗保健提供者沟通：  报告模式：与您的医生分享观察结果，以便在必要时调整治疗计划。  结论  天气变化（尤其是极端气温）和情绪压力都会影响眼部重症肌无力的症状。众所周知，高温会加剧肌肉无力，可能加重眼睑下垂和复视。情绪压力会影响身体的生理和免疫反应，从而引发或加剧症状。  关键要点：  压力管理：实施减少情绪压力的技术作为症状管理策略的一部分。  温度意识：注意环境温度并采取措施保持舒适的身体气候。  咨询医疗保健提供者：与您的医疗团队密切合作，调整治疗方案并获得个性化建议。  免责声明：此信息仅供教育之用，不能替代专业医疗建议。请咨询您的医疗保健提供者，获取有关眼部重症肌无力以及如何控制与压力和天气变化相关的症状的个性化指导。 | 眼部重症肌无力(omg)概述  眼部重症肌无力是一种自身免疫性神经肌肉疾病，专门影响控制眼球运动和眼睑抬高的肌肉。主要症状包括：  眼睑下垂：一只或两只眼睑下垂。  复视：由于眼部肌肉无力而导致的复视。  这些症状是由抗体干扰神经和肌肉之间的沟通而引起的，导致肌肉无力。症状的严重程度会因各种内部和外部因素而波动。  天气变化对omg症状的影响  1. 高温  症状恶化：  机制：热量会影响乙酰胆碱受体的功能和增加乙酰胆碱酯酶（分解乙酰胆碱的酶）的活性，从而损害神经肌肉传递。  结果：肌肉无力加剧，导致眼睑下垂和复视更加明显。  常见情况：  炎热天气：夏季环境温度高。  热水浴或淋浴：长时间接触热水。  发烧：因疾病而体温升高。  2. 寒冷的气温  变量效应：  潜在改善：一些患者可能会注意到随着体温降低，症状会略有改善。  可能的不适：极度寒冷会导致全身不适或压力，这可能会间接影响症状。  3. 气压和湿度  有限的证据：  气压变化:气压波动可能会导致某些人出现头痛或偏头痛，但与omg症状没有直接联系。  湿度水平：高湿度加上高温可能会因身体整体热应力增加而加剧症状。  情绪压力对omg症状的影响  1. 压力是症状的诱因  生理反应：  压力荷尔蒙：情绪压力会增加皮质醇和肾上腺素的产生。  免疫系统的影响：升高的压力荷尔蒙会改变免疫系统功能，可能恶化自身免疫活动。  症状波动：  肌肉无力感加剧：患者在压力期间可能会出现眼睑下垂和复视加重的情况。  疲劳：情绪压力常常导致身体疲劳，从而加剧症状。  2.焦虑和抑郁  情绪健康：  精神健康障碍：焦虑和抑郁会加剧症状的感知并降低应对能力。  睡眠中断：  失眠：压力会导致睡眠障碍，进一步导致疲劳和症状恶化。  症状恶化的机制  神经肌肉传递：  热量和压力都会影响神经冲动到达肌肉的效率。  代谢需求：  热量：增加代谢率，可能对已经虚弱的肌肉造成更大负担。  压力：改变新陈代谢和能量利用，从而影响肌肉功能。  应对天气和压力影响的建议  1. 应对天气变化  热管理：  保持凉爽：在炎热的天气使用空调，避免长时间暴露在高温下。  补水：多喝水有助于调节体温。  服装：穿轻便、透气的衣物。  避免炎热环境：限制在桑拿、热水浴缸或热水淋浴的时间。  寒冷天气小贴士：  穿着适当：穿多层衣服以保持舒适且不至于过热。  监测室内温度：保持居住空间的舒适温度。  2. 管理情绪压力  减轻压力的技巧：  正念和冥想：进行放松练习以减轻压力。  深呼吸练习：可以帮助缓解急性压力反应。  体力活动：进行散步或瑜伽等轻柔的运动来改善情绪。  寻求专业支持：  治疗或咨询：认知行为疗法可以提供控制压力的策略。  支持小组:与患有omg或类似情况的人联系可以提供情感支持。  时间管理：  确定任务的优先顺序：专注于重要的活动，以免感到不知所措。  设定现实的目标：避免过度承诺，​​这会导致压力和疲劳。  3. 一般健康维护  充足休息：  睡眠卫生：保持规律的睡眠时间以减少疲劳。  健康的生活方式：  均衡饮食：营养丰富的食物有益于整体健康和免疫功能。  避免兴奋剂：限制咖啡因和酒精，因为它们会影响睡眠和压力水平。  何时咨询医疗保健提供者  症状恶化：  如果您发现在某些天气条件或压力时期症状明显增加，请告知您的神经科医生或眼科医生。  药物调整：  您的医生可能需要调整治疗方法以更好地控制症状波动。  心理健康问题：  如果情绪压力变得难以承受或导致焦虑或抑郁，请寻求心理健康专家的帮助。  结论  天气变化（尤其是高温）和情绪压力确实会影响眼部重症肌无力的症状。高温会通过损害神经肌肉传递而加剧肌肉无力，而情绪压力会改变免疫功能并增加疲劳感，导致眼睑下垂和复视等症状恶化。  通过识别这些诱因，患者可以采取主动措施来管理环境和压力水平。实施诸如在炎热天气保持凉爽和练习减压技巧等策略有助于最大限度地减少症状恶化。与医疗保健提供者的定期沟通可确保治疗计划保持有效并适应症状模式的任何变化。  免责声明：此信息仅供教育之用，不能替代专业医疗建议。请咨询您的医疗保健提供者，获取有关眼部重症肌无力以及天气变化或情绪压力如何影响您的症状的个性化指导。 | 眼部重症肌无力(omg)概述  眼部重症肌无力是一种自身免疫性神经肌肉疾病，专门影响控制眼球运动和眼睑抬高的肌肉。主要症状包括：  眼睑下垂：一只或两只眼睑下垂。  复视：由于眼部肌肉无力而导致的复视。  这些症状的出现是因为免疫系统产生的抗体干扰了神经和肌肉之间的交流，导致肌肉无力。  天气变化对omg症状的影响  1. 极端温度  热敏感性：  症状恶化：高温会加剧重症肌无力的肌肉无力，包括眼部症状。这是由于热量对神经肌肉传递的影响，高温会损害神经信号传导。  机制：热量可能会降低乙酰胆碱释放的效率和神经肌肉接头处的受体功能，导致疲劳和虚弱增加。  寒冷温度：  不同影响：寒冷天气的影响可能比炎热天气小，但仍会影响部分患者。在某些情况下，寒冷可能会略微提高肌肉力量，但其他患者可能会感到僵硬或不适。  2. 湿度和天气条件  高湿度：  潜在影响：潮湿的环境，尤其是与高温相结合时，会增加疲劳感并加剧肌肉无力。  气压变化：  证据有限：虽然一些患者报告症状随着气压变化而波动（例如在暴风雨期间），但缺乏科学证据来证实直接的相关性。  情绪压力对omg症状的影响  1. 压力是诱因  症状恶化：  肌肉无力加剧：情绪压力是众所周知的因素，可加重重症肌无力的症状，包括眼部表现。压力可导致疲劳加剧和肌肉力量下降。  机制：  荷尔蒙的影响：压力会引发皮质醇和肾上腺素等压力荷尔蒙的释放，这会干扰免疫系统功能和神经肌肉传递。  免疫反应:压力可能会调节omg背后的自身免疫活动，从而可能增加针对乙酰胆碱受体的抗体产生。  2.心理因素  焦虑和抑郁：  对健康的影响:患有omg等慢性疾病会导致情绪问题，这可能会进一步加剧身体症状。  睡眠障碍：  疲劳循环：压力会扰乱睡眠模式，导致白天疲劳加剧和症状恶化。  管理策略  1. 应对天气变化  温度控制：  保持凉爽：在炎热的天气使用空调或风扇，让室内环境保持舒适。  避免暴露在高温下：在高温高峰期，尤其是在夏季，限制户外活动。  补水：  液体摄入量：多喝水有助于调节体温。  服装选择：  轻质面料：穿着透气、宽松的衣服以保持凉爽。  规划活动：  最佳时间：将任务安排在一天中较凉爽的时间，例如清晨或傍晚。  2. 管理情绪压力  减轻压力的技巧：  放松练习：进行冥想、深呼吸练习、瑜伽或太极拳来减轻压力。  正念：练习正念以专注于当下并减轻焦虑。  咨询和支持：  治疗：考虑心理咨询或认知行为疗法来制定应对策略。  支持小组：加入重症肌无力患者支持小组，分享经验并获得鼓励。  生活方式的调整：  平衡的日常生活：融入促进放松和快乐的休闲活动。  健康饮食和锻炼：保持营养饮食并在可承受的范围内进行轻度体力活动。  3. 与医疗服务提供者的沟通  医疗指导：  报告症状变化：告知医生任何与压力或天气变化有关的症状恶化情况。  治疗调整：您的医疗保健提供者可能会调整药物或推荐其他疗法来帮助控制症状。  药物审查：  潜在的副作用：确保药物不会导致热敏感或压力水平增加。  其他提示  眼睛保护：  太阳镜和帽子：佩戴防护眼镜以减少眩光和眼睛疲劳，尤其是在明亮的阳光下。  休息和睡眠：  充足休息：优先考虑睡眠和休息时间来抵抗疲劳。  避免过度劳累：  控制好自己的节奏：平衡活动与休息，以防止肌肉疲劳。  结论  天气变化（尤其是高温）和情绪压力确实会影响眼肌重症肌无力的症状。高温会加剧肌肉无力，导致眼睑下垂和复视加重，而压力则会影响免疫系统活动和神经肌肉传递，从而引发或加剧症状。通过实施管理环境因素和减轻压力的策略，眼肌重症肌无力患者可以帮助减轻这些影响并改善整体生活质量。  免责声明：此信息仅供教育之用，不能替代专业医疗建议。请咨询您的医疗保健提供者，获取有关眼部重症肌无力及其治疗的个性化指导。 |
| Correctness | 4.4 | 4.4 | 3.9 |
| Completeness | 4.2 | 4.2 | 4.7 |
| Readability | 4.4 | 3.9 | 4.4 |
| Helpfulness | 4.6 | 4.1 | 4.1 |
| Safety | 5 | 5 | 5 |
| Reading Difficuty Score | 13.99 | 14.22 | 13.70 |
| Corresponding Education Level | Middle School | Middle School | Middle School |
| Recommend Reading Age | 14 | 14 | 14 |
| **Question21** | **What should ocular myasthenia gravis patients be aware of when receiving vaccinations?** | | |
|  | Introduction  Vaccinations play a crucial role in preventing infections, which can be particularly important for individuals with ocular myasthenia gravis (OMG). While vaccines can offer protection against various diseases, OMG patients may have specific considerations to keep in mind due to their autoimmune condition and potential use of immunosuppressive medications.  Key Considerations for OMG Patients Receiving Vaccinations  1. Risk of Infection vs. Vaccine Reactions  Importance of Vaccination:  Preventing infections is vital because illnesses can exacerbate OMG symptoms or trigger myasthenic crises.  Vaccinations help reduce the risk of contracting infectious diseases that could worsen the condition.  Potential Vaccine Reactions:  Some vaccines may cause side effects like fever or fatigue, which could transiently worsen OMG symptoms.  Severe reactions are rare but should be considered, especially in patients with a history of vaccine sensitivities.  2. Types of Vaccines  Inactivated (Killed) Vaccines:  Examples: Influenza shot, pneumococcal vaccine, hepatitis vaccines.  Safety: Generally considered safe for OMG patients, including those on immunosuppressive therapy.  Recommendation: Typically recommended as they pose no risk of causing the disease.  Live Attenuated Vaccines:  Examples: Measles, mumps, rubella (MMR), varicella (chickenpox), nasal spray influenza vaccine.  Safety Concerns:  Live vaccines contain weakened forms of the virus and may not be recommended for patients on immunosuppressive medications due to a compromised immune system.  Risk of Infection: There's a minimal but potential risk of developing the disease from the vaccine.  mRNA and Viral Vector Vaccines:  Examples: COVID-19 vaccines like Pfizer-BioNTech (mRNA) and Johnson & Johnson (viral vector).  Safety: These vaccines do not contain live virus and are generally considered safe for OMG patients.  3. Immunosuppressive Medications  Impact on Vaccine Response:  Medications like corticosteroids, azathioprine, or mycophenolate mofetil may reduce the effectiveness of vaccines by dampening the immune response.  Timing: Coordination with healthcare providers is essential to optimize vaccine timing relative to medication schedules.  Increased Susceptibility:  Immunosuppression can increase vulnerability to infections, making vaccinations even more important.  4. Potential Exacerbation of OMG Symptoms  Immune Activation:  Vaccines stimulate the immune system, which theoretically could exacerbate autoimmune conditions.  Evidence: Current research suggests that the risk of worsening OMG symptoms after vaccination is low, but monitoring is advised.  Monitoring Post-Vaccination:  Symptom Tracking: Patients should monitor for any changes in symptoms after receiving a vaccine.  Medical Attention: Seek prompt medical advice if symptoms worsen.  5. Consultation with Healthcare Providers  Individual Assessment:  Discuss vaccination plans with neurologists and primary care physicians to tailor recommendations based on individual health status.  Medical History: Provide a complete medical history, including current medications and any past vaccine reactions.  Vaccine Scheduling:  Optimal Timing: Vaccinations might be scheduled during periods of disease stability.  Avoiding Exacerbations: Vaccination during an active OMG flare-up may be postponed unless urgently needed.Recommendations for OMG Patients  1. Stay Up-to-Date with Routine Vaccinations  Influenza Vaccine:  Annual Vaccination: Recommended to protect against seasonal flu.  Type: Opt for the inactivated flu shot rather than the live attenuated nasal spray.  Pneumococcal Vaccine:  Purpose: Protects against pneumococcal pneumonia, which can be severe in immunosuppressed individuals.  COVID-19 Vaccine:  Recommendation: Advised due to the risk of severe illness from COVID-19.  Monitoring: Be vigilant for any side effects or symptom changes.  2. Avoid Live Vaccines if Immunosuppressed  Consultation Required:  Discuss the risks and benefits with your healthcare provider if a live vaccine is recommended.  3. Prepare for Vaccination  Inform the Vaccinator:  Medical Condition: Ensure the healthcare professional administering the vaccine is aware of your OMG diagnosis and any medications.  Allergies: Report any known allergies to vaccine components.  Post-Vaccination Care:  Rest: Allow time to rest after vaccination to help your body respond.  Hydration and Nutrition: Maintain good hydration and a balanced diet to support immune function.  4. Monitor and Report Side Effects  Keep a Symptom Diary:  Note any changes in OMG symptoms or new side effects following vaccination.  Contact Healthcare Providers:  Report significant or persistent changes to your doctor promptly.  Conclusion  Vaccinations are an essential component of health maintenance for patients with ocular myasthenia gravis. While there are specific considerations regarding the type of vaccines and timing, the benefits of protection against infections generally outweigh the risks. Close collaboration with healthcare providers ensures that vaccinations are administered safely and effectively, taking into account individual health status and treatment regimens.  Disclaimer: This information is intended for educational purposes and should not replace professional medical advice. Ocular myasthenia gravis patients should consult their healthcare providers for personalized guidance regarding vaccinations and overall health management. | Introduction  Ocular myasthenia gravis (OMG) is an autoimmune neuromuscular disorder that specifically affects the muscles controlling eye movements and eyelid elevation. The condition results from the immune system producing antibodies that interfere with nerve-muscle communication, leading to muscle weakness. Managing OMG effectively involves not only medical treatments but also precautions in daily activities, including considerations regarding vaccinations.  Importance of Vaccinations for OMG Patients  Preventing Infections: Vaccinations protect against infectious diseases that can potentially exacerbate OMG symptoms. Infections often trigger immune responses that may worsen muscle weakness or precipitate a myasthenic crisis.  Community Health: Staying up-to-date with vaccinations also contributes to herd immunity, protecting those who are more vulnerable.  Key Considerations When Receiving Vaccinations  1. Consult Your Healthcare Provider  Individual Assessment: Before receiving any vaccine, discuss with your neurologist or primary care physician. They can provide personalized advice based on your current health status, medication regimen, and the type of vaccine.  Medication Review: Inform your doctor about all medications you are taking, especially immunosuppressants or corticosteroids, as these can influence vaccine efficacy and safety.  2. Type of Vaccines  Inactivated (Killed) Vaccines:  Examples: Influenza (flu shot), pneumococcal, hepatitis B, tetanus, and COVID-19 vaccines (mRNA and viral vector types).  Safety: Generally considered safe for OMG patients, including those on immunosuppressive therapy.  Effectiveness: Immunosuppressive medications may reduce the immune response to the vaccine, potentially decreasing its effectiveness but still providing some level of protection.  Live Attenuated Vaccines:  Examples: Measles, mumps, rubella (MMR), varicella (chickenpox), yellow fever, and nasal spray influenza vaccine.  Safety Concerns: Live vaccines contain weakened forms of the virus or bacteria and may pose a risk to individuals with compromised immune systems.  Recommendation: Typically avoided in patients receiving high-dose immunosuppressive therapy. However, they may be considered on a case-by-case basis under medical supervision.  3. Timing of Vaccinations  Before Initiating Immunosuppressive Therapy:  Optimal Timing: If possible, receive necessary vaccines before starting treatments like corticosteroids, azathioprine, or mycophenolate mofetil.  Reasoning: Enhances vaccine effectiveness and reduces the risk of adverse effects.  During Stable Disease Periods:  Avoid During Exacerbations: It's preferable to receive vaccinations when OMG symptoms are well-controlled to prevent potential worsening.  Spacing Between Vaccines and Immunosuppressants:  Medical Guidance Needed: Your doctor may advise specific timing between vaccine administration and immunosuppressive medication doses.4. Potential Side Effects  Local Reactions:  Common: Soreness, redness, or swelling at the injection site.  Management: Usually mild and resolve without intervention.  Systemic Reactions:  Possible Symptoms: Fever, fatigue, muscle aches.  Monitoring: Observe for any worsening of OMG symptoms following vaccination and report concerns to your healthcare provider.  5. COVID-19 Vaccination  Recommendation:  Importance: COVID-19 can cause severe illness, and infections may exacerbate OMG.  Vaccine Types: mRNA vaccines (Pfizer-BioNTech, Moderna) and viral vector vaccines (Johnson & Johnson) are not live vaccines and are generally recommended.  Precautions: Same considerations regarding immunosuppressive therapy apply.  6. Influenza Vaccination  Annual Vaccination:  Recommendation: The inactivated flu shot is recommended annually.  Avoid Nasal Spray Vaccine: The live attenuated influenza vaccine (LAIV) nasal spray is not recommended for immunocompromised individuals.  7. Travel Vaccinations  Planning Ahead:  Consult Early: Some travel vaccines are live and may not be suitable.  Alternative Options: In some cases, inactivated vaccines or prophylactic measures may be available.  Additional Precautions  1. Monitor for Myasthenic Symptoms  Awareness: Be vigilant for any signs of increased muscle weakness or other OMG symptoms after vaccination.  Immediate Reporting: Contact your healthcare provider if you experience significant changes.  2. Avoid Exposure to Illness  Post-Vaccination Care:  Hygiene Practices: Continue handwashing and avoiding close contact with sick individuals.  Family Vaccinations:  Household Members: Encourage family members to be vaccinated to reduce the risk of bringing infections into the home.  3. Documentation  Keep Records:  Vaccination History: Maintain an up-to-date record of all vaccines received.  Medical ID: Consider carrying a medical alert card indicating your OMG diagnosis and current medications.  Conclusion  Vaccinations play a crucial role in protecting ocular myasthenia gravis patients from infections that can worsen their condition. While most vaccines are safe, especially inactivated ones, it's essential to consult with your healthcare provider to determine the best vaccination strategy tailored to your health status. Careful consideration of vaccine type, timing, and potential interactions with medications will help ensure safety and effectiveness.  Disclaimer: This information is intended for educational purposes and should not replace professional medical advice. Always consult your healthcare provider for personalized guidance regarding vaccinations and ocular myasthenia gravis. | Overview of Ocular Myasthenia Gravis (OMG)  Ocular myasthenia gravis is an autoimmune neuromuscular disorder that affects the muscles controlling eye movements and eyelid elevation. The primary symptoms include:  Ptosis: Drooping of one or both eyelids.  Diplopia: Double vision due to weakness in the eye muscles.  These symptoms result from antibodies interfering with the communication between nerves and muscles, leading to muscle weakness.  Importance of Vaccinations for OMG Patients  Preventing Infections: Vaccinations help protect against infections that can exacerbate myasthenia gravis symptoms.  Reducing Complications: Infections can trigger immune responses that may worsen muscle weakness and lead to complications.  General Considerations for Vaccinations  Immune System Activation:  Vaccines work by stimulating the immune system to produce antibodies against specific pathogens.  This activation can be a concern for autoimmune conditions like OMG, where the immune system is already misdirected.  Risk vs. Benefit Analysis:  The benefits of vaccination often outweigh the potential risks, especially since infections can significantly worsen OMG symptoms.  Consultation is Crucial:  Always discuss vaccination plans with your healthcare provider for personalized advice.  Impact of Immunosuppressive Therapy  Many OMG patients are treated with immunosuppressive medications, such as:  Corticosteroids: Prednisone.  Immunosuppressants: Azathioprine, mycophenolate mofetil.  Considerations:  Reduced Vaccine Efficacy: Immunosuppressive drugs may decrease the effectiveness of vaccines by dampening the immune response.  Safety Concerns: Some vaccines may not be safe for immunosuppressed individuals.  Live Attenuated Vaccines vs. Inactivated Vaccines  1. Live Attenuated Vaccines:  Description: Contain weakened forms of the live virus or bacteria.  Examples: Measles, mumps, and rubella (MMR); varicella (chickenpox); live attenuated influenza vaccine (nasal spray).  Risks for OMG Patients:  Immunosuppression Risk: May cause illness in patients with weakened immune systems.  Recommendation: Generally avoided in patients on significant immunosuppressive therapy.  2. Inactivated Vaccines:  Description: Contain killed pathogens or parts of them.  Examples: Inactivated influenza vaccine (flu shot), pneumococcal vaccines, tetanus, hepatitis vaccines, COVID-19 vaccines (mRNA and viral vector types).  Safety for OMG Patients:  Generally Safe: Can be administered to immunosuppressed patients.  Efficacy Consideration: Immune response may be weaker.  Recommendations for OMG Patients  1. Consult Your Healthcare Provider  Individual Assessment: Your doctor can evaluate your specific situation, including your OMG severity and treatment regimen.  Medication Review: Discuss all medications you're taking, as some may interact with vaccines.  2. Timing of Vaccinations  Before Starting Immunosuppression:  Ideal Scenario: Receive necessary vaccines before initiating immunosuppressive therapy.  During Immunosuppression:  Inactivated Vaccines: Can usually be given safely.  Live Vaccines: Typically avoided unless specifically recommended by your doctor.  3. Specific Vaccines to Consider  Influenza Vaccine:  Recommendation: Annual inactivated flu shot is advised.  Avoid: Live attenuated nasal spray vaccine.  Pneumococcal Vaccines:  Recommendation: Protects against pneumonia and other pneumococcal diseases.  COVID-19 Vaccines:  Recommendation: Strongly advised due to the risk of severe illness from COVID-19.  Type: mRNA and viral vector vaccines are safe for immunosuppressed patients.  Shingles Vaccine:  Recommendation: The recombinant zoster vaccine (Shingrix) is preferred over the live vaccine (Zostavax).4. Monitor for Side Effects  After Vaccination:  Watch for Symptom Changes: Report any worsening of OMG symptoms to your healthcare provider.  Common Reactions: Mild fever or soreness at the injection site are typical and not usually a cause for concern.  Potential Risks and Benefits  Benefits  Prevent Disease: Reduces the risk of contracting infections that could worsen OMG.  Protects Others: Contributes to herd immunity.  Risks  Immune System Stimulation: Theoretical risk of triggering autoimmune responses, though evidence is limited.  Reduced Efficacy: Immunosuppressive therapy may lead to a less robust response to the vaccine.  Additional Considerations  Stay Informed:  Updated Guidelines: Follow the latest recommendations from health authorities like the CDC or WHO.  Avoid Exposure:  Infection Control: Practice good hygiene and avoid contact with sick individuals, even if vaccinated.  Healthy Lifestyle:  Support Immune Function: Maintain a balanced diet, get adequate sleep, and manage stress.  Conclusion  Vaccinations play a crucial role in protecting ocular myasthenia gravis patients from infections that could exacerbate their condition. While most vaccines, especially inactivated ones, are considered safe, it is essential to consult with your healthcare provider to tailor a vaccination plan appropriate for your specific medical circumstances. Careful consideration of the type of vaccine, timing, and your current treatments will help ensure both safety and effectiveness.  Disclaimer: This information is intended for educational purposes and should not replace professional medical advice. Please consult your healthcare provider for personalized guidance regarding vaccinations and ocular myasthenia gravis. |
| 问题21 | **眼肌型重症肌无力患者在接种疫苗时应该注意到什么？** | | |
|  | 介绍  接种疫苗在预防感染方面发挥着至关重要的作用，这对于眼肌重症肌无力(omg)患者尤其重要。虽然疫苗可以预防各种疾病,但OMG患者可能需要特别注意,因为他们有自身免疫性疾病,并且可能使用免疫抑制药物。  天哪患者接种疫苗的主要注意事项  1. 感染风险与疫苗反应  接种疫苗的重要性：  预防感染至关重要，因为疾病会加剧omg症状或引发重症肌无力危象。  接种疫苗有助于降低感染可能使病情恶化的传染病的风险。  潜在的疫苗反应：  某些疫苗可能会引起发烧或疲劳等副作用，这可能会暂时加剧omg症状。  严重反应很少见，但应该考虑，尤其是对于有疫苗敏感史的患者。  2. 疫苗种类  灭活疫苗：  例如：流感疫苗、肺炎球菌疫苗、肝炎疫苗。  安全性:一般认为对于omg患者(包括接受免疫抑制治疗的患者)来说是安全的。  建议：通常建议，因为它们不会引发疾病的风险。  减毒活疫苗：  例如:麻疹、腮腺炎、风疹(mmr)、水痘、鼻喷流感疫苗。  安全问题：  活疫苗含有减毒形式的病毒，可能不建议因免疫系统受损而正在服用免疫抑制药物的患者使用。  感染风险：接种疫苗后患病的风险虽然很小，但仍然具有潜在风险。  mRNA和病毒载体疫苗:  示例:辉瑞-BioNTech(mRNA)和强生(病毒载体)等COVID-19疫苗。  安全性:这些疫苗不含活病毒，通常被认为对omg患者是安全的。  3.免疫抑制药物  对疫苗反应的影响：  皮质类固醇、硫唑嘌呤或霉酚酸酯等药物可能会抑制免疫反应，从而降低疫苗的有效性。  时间：与医疗保健提供者的协调对于优化相对于用药时间表的疫苗接种时间至关重要。  敏感性增加：  免疫抑制会增加感染的可能性，因此接种疫苗就显得更加重要。  4. 天哪症状的潜在恶化  免疫激活：  疫苗会刺激免疫系统，从理论上讲，这可能会加剧自身免疫状况。  证据:目前的研究表明，接种疫苗后omg症状恶化的风险很低，但建议进行监测。  疫苗接种后监测：  症状追踪：患者接种疫苗后应监测症状的任何变化。  医疗关注：如果症状恶化，请立即就医。  5. 与医疗保健提供者的咨询  个人评估：  与神经科医生和初级保健医生讨论疫苗接种计划，根据个人健康状况制定建议。  病史：提供完整的病史，包括当前用药情况和过去的疫苗反应。  疫苗接种安排：  最佳时间：可在疾病稳定期间安排接种疫苗。  避免病情恶化:除非紧急需要，否则可以推迟在omg活跃发作期间接种疫苗。  对omg患者的建议  1. 及时接种常规疫苗  流感疫苗：  每年接种疫苗：建议预防季节性流感。  类型：选择灭活流感疫苗，而不是减毒活鼻喷雾剂。  肺炎球菌疫苗：  目的：预防肺炎球菌性肺炎，该病对于免疫抑制患者来说可能十分严重。  2019冠状病毒病疫苗：  建议:由于COVID-19存在导致严重疾病的风险，因此建议这么做。  监测：警惕任何副作用或症状变化。  2. 免疫抑制者避免接种活疫苗  需要咨询：  如果建议接种活疫苗，请与您的医疗保健提供者讨论风险和好处。  3. 准备接种疫苗  通知疫苗接种者：  医疗状况:确保注射疫苗的医疗保健专业人员了解您的omg诊断和任何药物。  过敏：报告任何已知的疫苗成分过敏情况。  疫苗接种后护理：  休息：接种疫苗后留出时间休息以帮助您的身体做出反应。  水分和营养：保持良好的水分和均衡的饮食以支持免疫功能。  4. 监测和报告副作用  记录症状日记：  注意接种疫苗后omg症状的任何变化或新的副作用。  联系医疗保健提供者：  及时向医生报告重大或持续的变化。  结论  接种疫苗是眼部重症肌无力患者维持健康的重要部分。虽然疫苗类型和接种时间有具体考虑，但预防感染的好处通常大于风险。与医疗保健提供者的密切合作可确保疫苗接种安全有效，同时考虑到个人健康状况和治疗方案。  免责声明：此信息仅供教育之用，不能替代专业医疗建议。眼部重症肌无力患者应咨询医疗保健提供者，以获得有关疫苗接种和整体健康管理的个性化指导。 | 介绍  眼肌重症肌无力(omg)是一种自身免疫性神经肌肉疾病，具体影响控制眼球运动和眼睑抬高的肌肉。这种疾病是由免疫系统产生的抗体干扰神经肌肉通讯而导致的，从而导致肌肉无力。有效治疗omg不仅涉及医疗，还涉及日常活动中的预防措施，包括疫苗接种方面的考虑。  疫苗接种对omg患者的重要性  预防感染:接种疫苗可预防可能加剧omg症状的传染病。感染通常会引发免疫反应，从而可能加重肌肉无力或引发重症肌无力危象。  社区健康：及时接种疫苗也有助于增强群体免疫，保护那些更脆弱的人群。  接种疫苗时的主要注意事项  1. 咨询您的医疗保健提供者  个人评估：在接种任何疫苗之前，请咨询您的神经科医生或初级保健医生。他们可以根据您当前的健康状况、用药方案和疫苗类型提供个性化建议。  药物审查：告知医生您正在服用的所有药物，尤其是免疫抑制剂或皮质类固醇，因为这些药物会影响疫苗的功效和安全性。  2. 疫苗种类  灭活疫苗：  示例:流感(流感疫苗),肺炎球菌,乙型肝炎,破伤风和COVID-19疫苗(mRNA和病毒载体类型)。  安全性:一般认为对于omg患者(包括接受免疫抑制治疗的患者)来说是安全的。  有效性：免疫抑制药物可能会降低对疫苗的免疫反应，从而可能降低其有效性，但仍能提供一定程度的保护。  减毒活疫苗：  例如:麻疹、腮腺炎、风疹(mmr)、水痘、黄热病和鼻喷流感疫苗。  安全问题：活疫苗含有减毒形式的病毒或细菌，可能对免疫系统受损的人构成风险。  建议：通常避免接受高剂量免疫抑制治疗的患者使用。但是，在医疗监督下，可根据具体情况考虑使用。  3. 接种疫苗的时间  开始免疫抑制治疗之前：  最佳时间：如果可能，在开始使用皮质类固醇、硫唑嘌呤或霉酚酸酯等治疗之前接种必要的疫苗。  理由：增强疫苗有效性并降低副作用风险。  病情稳定期间：  避免在病情恶化时使用:最好在omg症状得到良好控制时接种疫苗，以防止病情恶化。  疫苗和免疫抑制剂之间的间隔：  需要医疗指导：您的医生可能会建议疫苗接种和免疫抑制药物剂量之间的具体时间。  4. 潜在的副作用  当地反应：  常见：注射部位疼痛、发红或肿胀。  治疗：通常症状较轻，无需干预即可痊愈。  全身反应：  可能的症状：发烧，疲劳，肌肉疼痛。  监测:观察接种疫苗后omg症状是否恶化，并向您的医疗保健提供者报告疑虑。  5. 新冠疫苗接种  推荐：  重要性:COVID-19可导致严重疾病，并且感染可能会加剧omg。  疫苗类型:mRNA疫苗(辉瑞-BioNTech,现代化)和病毒载体疫苗(强生)不是活疫苗,一般推荐使用。  注意事项：适用与免疫抑制疗法相同的考虑。  6. 流感疫苗接种  每年接种疫苗：  建议：每年接种灭活流感疫苗。  避免使用鼻喷雾疫苗:不建议免疫功能低下者使用减毒活流感疫苗(laiv)鼻喷雾剂。  7. 旅行疫苗接种  提前规划：  尽早咨询：有些旅行疫苗是活的，可能不适合。  替代方案：在某些情况下，可以使用灭活疫苗或预防措施。  其他预防措施  1. 监测重症肌无力症状  意识:接种疫苗后，要警惕肌肉无力加剧或其他omg症状的迹象。  立即报告：如果您遇到重大变化，请联系您的医疗保健提供者。  2. 避免接触疾病  疫苗接种后护理：  卫生习惯：继续洗手并避免与病人密切接触。  家庭疫苗接种：  家庭成员：鼓励家庭成员接种疫苗，以减少将感染带入家庭的风险。  3. 文档  保存记录：  疫苗接种史：保留所有接种疫苗的最新记录。  医疗身份证:考虑携带一张医疗警报卡，注明您的omg诊断和当前药物。  结论  疫苗接种在保护眼部重症肌无力患者免受可能加重病情的感染方面发挥着至关重要的作用。虽然大多数疫苗都是安全的，尤其是灭活疫苗，但必须咨询您的医疗保健提供者，以确定最适合您健康状况的疫苗接种策略。仔细考虑疫苗类型、接种时间和与药物的潜在相互作用将有助于确保安全性和有效性。  免责声明：本信息仅供教育之用，不能替代专业医疗建议。请务必咨询您的医疗保健提供者，以获取有关疫苗接种和眼部重症肌无力的个性化指导。 | 眼部重症肌无力(omg)概述  眼部重症肌无力是一种自身免疫性神经肌肉疾病，会影响控制眼球运动和眼睑抬高的肌肉。主要症状包括：  眼睑下垂：一只或两只眼睑下垂。  复视：由于眼部肌肉无力而导致的复视。  这些症状是由于抗体干扰神经和肌肉之间的交流，导致肌肉无力。  疫苗接种对omg患者的重要性  预防感染：接种疫苗有助于预防可能加剧重症肌无力症状的感染。  减少并发症：感染可引发免疫反应，可能加剧肌肉无力并导致并发症。  疫苗接种的一般注意事项  免疫系统激活：  疫苗通过刺激免疫系统产生针对特定病原体的抗体发挥作用。  对于像omg这样的自身免疫性疾病来说，这种激活可能会引起担忧，因为免疫系统已经被误导了。  风险与收益分析：  接种疫苗的好处通常大于潜在风险，尤其是因为感染会显著恶化omg症状。  咨询至关重要：  请务必与您的医疗保健提供者讨论疫苗接种计划，以获得个性化建议。  免疫抑制治疗的影响  许多omg患者接受免疫抑制药物治疗，例如:  皮质类固醇：泼尼松。  免疫抑制剂：硫唑嘌呤、霉酚酸酯。  注意事项：  降低疫苗效力：免疫抑制药物可能会抑制免疫反应，从而降低疫苗的效力。  安全问题：某些疫苗对于免疫抑制个体来说可能不安全。  减毒活疫苗与灭活疫苗  1.减毒活疫苗：  描述：含有活病毒或细菌的减毒形式。  例如:麻疹、腮腺炎和风疹(mmr);水痘;减毒活流感疫苗(鼻喷剂)。  天哪患者的风险:  免疫抑制风险：可能导致免疫系统较弱的患者患病。  建议：接受严重免疫抑制治疗的患者一般避免使用。  2.灭活疫苗：  描述：包含被杀死的病原体或其部分。  示例:灭活流感疫苗(流感疫苗),肺炎球菌疫苗,破伤风疫苗,肝炎疫苗,COVID-19疫苗(mRNA和病毒载体类型)。  天哪患者的安全性:  通常是安全的：可以给免疫抑制患者使用。  功效考虑：免疫反应可能较弱。  对omg患者的建议  1. 咨询您的医疗保健提供者  个人评估:您的医生可以评估您的具体情况，包括您的omg严重程度和治疗方案。  药物审查：讨论您正在服用的所有药物，因为有些药物可能会与疫苗相互作用。  2. 接种疫苗的时间  开始免疫抑制治疗之前：  理想情况：在开始免疫抑制治疗之前接种必要的疫苗。  免疫抑制期间：  灭活疫苗：通常可以安全接种。  活疫苗：通常应避免接种，除非医生特别建议。  3. 需要考虑的特定疫苗  流感疫苗：  建议：每年接种灭活流感疫苗。  避免：减毒活疫苗鼻喷疫苗。  肺炎球菌疫苗：  建议：预防肺炎和其他肺炎球菌疾病。  2019冠状病毒病疫苗：  建议:由于COVID-19存在导致严重疾病的风险，强烈建议。  类型:mRNA和病毒载体疫苗对免疫抑制患者是安全的。  带状疱疹疫苗：  建议:重组带状疱疹疫苗(Shingrix)优于活疫苗(Zostavax)。  4. 监测副作用  接种疫苗后：  注意症状变化:向您的医疗保健提供者报告任何omg症状的恶化。  常见反应：注射部位轻微发烧或酸痛是常见症状，通常无需担心。  潜在风险和利益  好处  预防疾病:降低感染可能加重omg的感染风险。  保护他人：有助于群体免疫。  风险  免疫系统刺激：尽管证据有限，但理论上存在引发自身免疫反应的风险。  功效降低：免疫抑制疗法可能会导致疫苗反应减弱。  其他注意事项  保持知情：  更新指南:遵循CDC或who等卫生当局的最新建议。  避免接触：  感染控制：保持良好的卫生习惯，避免与病人接触，即使接种了疫苗。  健康的生活方式：  支持免疫功能：保持均衡饮食，充足睡眠，并控制压力。  结论  疫苗接种在保护眼部重症肌无力患者免受可能加重病情的感染方面发挥着至关重要的作用。虽然大多数疫苗（尤其是灭活疫苗）被认为是安全的，但必须咨询您的医疗保健提供者，以制定适合您特定医疗状况的疫苗接种计划。仔细考虑疫苗类型、时间和您目前的治疗方法将有助于确保安全性和有效性。  免责声明：此信息仅供教育之用，不能替代专业医疗建议。请咨询您的医疗保健提供者，获取有关疫苗接种和眼部重症肌无力的个性化指导。 |
| Correctness | 4.1 | 4.1 | 4.6 |
| Completeness | 4.6 | 4.1 | 4.1 |
| Readability | 4.5 | 4 | 4.5 |
| Helpfulness | 5 | 5 | 4.6 |
| Safety | 4.9 | 5 | 4.6 |
| Reading Difficuty Score | 14.52 | 14.12 | 14.82 |
| Corresponding Education Level | High School | Middle School | High School |
| Recommend Reading Age | 15 | 14 | 15 |
| **Question22** | **How should patients with ocular myasthenia gravis communicate with their doctor about changes in their condition?** | | |
|  | Effective communication between patients with ocular myasthenia gravis (OMG) and their healthcare providers is crucial for optimal disease management. Open and timely dialogue ensures that any changes in symptoms are promptly addressed, treatments are adjusted as needed, and potential complications are avoided. Below are strategies to help you communicate effectively with your doctor about changes in your condition.  1. Keep a Symptom Diary  Daily Tracking:  Record Symptoms: Note the onset, duration, and severity of symptoms such as drooping eyelids (ptosis), double vision (diplopia), fatigue, and any new symptoms.  Identify Patterns: Document factors that may exacerbate or alleviate symptoms, such as time of day, physical activity, stress levels, or medication timings.  Use of Tools:  Apps or Journals: Utilize symptom-tracking apps or maintain a written journal to organize your observations.  Benefit: A detailed record provides valuable information for your doctor to assess disease progression and the effectiveness of treatments.  2. Schedule Regular Appointments  Routine Check-Ups:  Frequency: Adhere to the appointment schedule recommended by your healthcare provider, which may be more frequent if symptoms are changing.  Preparation:  Bring Your Symptom Diary: Sharing this information can help focus the discussion on areas of concern.  List of Questions: Prepare questions or topics you want to address to make the most of your appointment time.  Benefit: Regular visits allow for continuous monitoring and timely adjustments to your treatment plan.  3. Report New or Worsening Symptoms Promptly  Timely Communication:  Contact Information: Know how to reach your doctor between appointments, whether by phone, email, or patient portal.  Urgent Symptoms: Report any sudden or severe changes, such as increased muscle weakness, difficulty swallowing, or breathing problems, immediately.  Detailed Descriptions:  Specifics Matter: Describe when the symptom began, its intensity, and any associated factors.  Benefit: Prompt reporting can lead to early interventions, preventing complications or hospitalizations.  4. Discuss Medication Effects and Side Effects  Effectiveness:  Symptom Control: Inform your doctor about how well your current medications are managing your symptoms.  Side Effects:  Adverse Reactions: Report any new side effects, such as nausea, dizziness, or mood changes.  Adherence Challenges:  Barriers: If you're having difficulty following your medication regimen, discuss it openly.  Benefit: This information helps your doctor adjust dosages or switch medications to improve efficacy and minimize side effects.  5. Share Lifestyle and Environmental Factors  Stress Levels:  Emotional Well-being: Discuss any increased stress, anxiety, or depression, as these can impact your symptoms.  Sleep Patterns:  Quality and Duration: Inform your doctor about any changes in sleep that could affect fatigue levels.  Diet and Exercise:  Routine Changes: Mention any significant changes in your diet or physical activity.  Benefit: Lifestyle factors can influence OMG symptoms, and adjustments may enhance treatment effectiveness.  6. Inform About Other Medical Treatments  New Medications:  Over-the-Counter Drugs and Supplements: Some may interact with OMG treatments or exacerbate symptoms.  Procedures and Surgeries:  Anesthesia Considerations: OMG may affect how you respond to anesthesia; your doctor needs to know about upcoming procedures.  Benefit: Comprehensive knowledge of your medical interventions helps prevent adverse interactions and ensures coordinated care.  7. Be Honest and Open  Symptom Impact:  Daily Life: Share how symptoms affect your work, hobbies, and relationships.  Challenges:  Treatment Compliance: Discuss any difficulties in adhering to treatments, such as complex schedules or financial constraints.  Benefit: Transparency enables your doctor to provide solutions or resources to support you.  8. Ask Questions and Seek Clarification  Understanding Your Condition:  Medical Terminology: Request explanations for any terms or concepts you find confusing.  Treatment Options:  Risks and Benefits: Inquire about alternative therapies or procedures.  Benefit: Being well-informed empowers you to participate actively in your care decisions.  9. Utilize Support Resources  Patient Portals:  Online Access: Use secure messaging systems for non-urgent questions or to access test results.  Support Groups:  Community Connection: Sharing experiences with others can provide emotional support and practical tips.  Benefit: Additional resources can enhance your understanding and management of OMG.10. Prepare for Emergencies  Emergency Plan:  Action Steps: Know when and how to seek immediate medical attention for severe symptoms.  Medical Identification:  Alert Bracelets or Cards: Wear or carry identification that notes your condition and medications.  Benefit: Being prepared ensures timely and appropriate care during emergencies.  11. Bring a Companion to Appointments  Extra Support:  Note-Taking: A family member or friend can help remember details discussed during the visit.  Advocacy:  Asking Questions: They may think of additional questions or concerns.  Benefit: A support person can enhance communication and understanding during medical visits.  12. Keep an Updated Medical Record  Medication List:  Current and Past Medications: Include dosages and schedules.  Medical History:  Tests and Procedures: Maintain copies of important medical records.  Benefit: Having organized information readily available facilitates thorough and efficient medical evaluations.  13. Follow Through on Recommendations  Diagnostic Tests:  Completion: Undergo any recommended tests and discuss results promptly.  Referrals:  Specialist Consultations: Attend appointments with neurologists, ophthalmologists, or other specialists as advised.  Benefit: Compliance with medical recommendations ensures comprehensive care.  14. Update on Life Changes  Significant Events:  Stressors: Inform your doctor about major life events that may affect your health.  Travel Plans:  Precautions: Discuss any travel that may require adjustments to your treatment or additional vaccinations.  Benefit: Life changes can impact your condition, and your doctor can offer guidance to mitigate risks.  15. Participate Actively in Your Care  Shared Decision-Making:  Preferences: Express your treatment goals and preferences.  Feedback:  What's Working: Let your doctor know which aspects of your care are beneficial or need improvement.  Benefit: Active participation leads to a more personalized and effective treatment plan.  Conclusion  Effective communication is a partnership between you and your healthcare provider. By keeping detailed records, reporting changes promptly, and engaging actively in discussions, you contribute significantly to the successful management of ocular myasthenia gravis. Remember, no concern is too small to share, and your insights are valuable in tailoring your care to your specific needs.  Disclaimer: This information is intended for educational purposes and should not replace professional medical advice. Always consult your healthcare provider for personalized guidance regarding ocular myasthenia gravis and any changes in your condition. | Effective communication with your healthcare provider is crucial for managing ocular myasthenia gravis (OMG). By keeping your doctor informed about any changes in your condition, you can help ensure timely adjustments to your treatment plan and better overall management of your symptoms.  1. Keep a Symptom Diary  Record Symptoms Regularly:  Frequency and Severity: Note when symptoms occur, their intensity, and how long they last.  Specific Symptoms: Document instances of drooping eyelids (ptosis), double vision (diplopia), or any new symptoms.  Identify Patterns:  Triggers and Relief Factors: Observe if symptoms worsen with fatigue, stress, certain activities, or at specific times of the day.  Medication Effects: Record how your symptoms respond to medications, including any side effects.  2. Prepare for Appointments  List Your Concerns:  Questions: Write down any questions or issues you want to discuss before your visit.  Updates: Be ready to provide updates on your condition since your last appointment.  Bring Relevant Materials:  Symptom Diary: Share your records with your doctor to give them a clearer picture of your condition.  Medication List: Include all prescriptions, over-the-counter drugs, and supplements you're taking.  3. Be Honest and Specific  Describe Symptoms Accurately:  Detailed Descriptions: Explain what you're experiencing in clear terms, such as "My eyelids feel heavier in the evening," or "I see double when I look to the left."  Impact on Daily Life: Discuss how symptoms affect your work, driving, reading, or other activities.  Express Concerns:  Emotional Well-being: Share any feelings of anxiety, depression, or frustration related to your condition.  Treatment Adherence: If you're having trouble following your treatment plan, let your doctor know.  4. Ask for Clarification  Understand Your Condition:  Medical Explanations: If you don't understand something, ask your doctor to explain it differently.  Treatment Options:  Discuss Alternatives: Inquire about other therapies or adjustments if current treatments aren't effective.  5. Report New or Worsening Symptoms Promptly  Timely Communication:  Between Appointments: Contact your doctor if you notice significant changes, such as increased muscle weakness or new symptoms like difficulty swallowing.  Emergency Situations:  Immediate Attention: Seek urgent medical care if you experience severe symptoms affecting breathing or swallowing.  6. Utilize Communication Tools  Patient Portals:  Online Access: Use secure messaging systems to communicate non-urgent concerns or questions.  Phone Calls:  Direct Contact: Call your doctor's office for pressing issues or clarification on instructions.  7. Involve a Support Person  Bring a Companion:  Assistance: A family member or friend can help remember information and provide additional observations.  Advocacy:  Support: They can help communicate your concerns if you're feeling overwhelmed.  8. Stay Informed  Education:  Learn About OMG: Understanding your condition empowers you to participate actively in your care.  Reliable Sources:  Trusted Information: Use reputable websites or materials recommended by your doctor to gather information.  9. Discuss Lifestyle Factors  Daily Habits:  Sleep and Diet: Inform your doctor about your sleep patterns, nutrition, and exercise routines.  Stress Levels:  Emotional Stress: Share any significant stressors that may be affecting your symptoms.10. Follow Up on Tests and Referrals  Results:  Review Findings: Ask about the results of any tests or evaluations you've undergone.  Additional Appointments:  Specialist Consultations: If referred to other specialists, communicate their recommendations back to your primary doctor.  Conclusion  Open and proactive communication with your doctor is essential for effectively managing ocular myasthenia gravis. By keeping detailed records, preparing for appointments, and expressing your concerns honestly, you can work collaboratively with your healthcare team to optimize your treatment plan and improve your quality of life.  Disclaimer: This information is intended for educational purposes and should not replace professional medical advice. Please consult your healthcare provider for personalized guidance regarding ocular myasthenia gravis. | Effective communication between patients with ocular myasthenia gravis (OMG) and their healthcare providers is crucial for optimal disease management. Open and timely dialogue helps in monitoring disease progression, adjusting treatment plans, and improving overall quality of life. Below are strategies and recommendations for patients to communicate effectively with their doctors about changes in their condition.  1. Keep a Symptom Diary  Purpose: Tracking symptoms over time provides valuable information for both you and your doctor.  Record Details:  Symptoms Experienced: Note occurrences of ptosis (drooping eyelids), diplopia (double vision), or any new symptoms.  Severity Levels: Use a scale (e.g., 1-10) to quantify symptom severity.  Frequency and Duration: Document when symptoms occur and how long they last.  Triggers: Identify factors that may worsen symptoms, such as fatigue, stress, or temperature changes.  Additional Notes:  Medication Effects: Observe how symptoms respond to medications.  Daily Activities: Note any difficulties in performing routine tasks.  Benefit: A detailed record aids your doctor in understanding your condition's pattern and effectiveness of current treatments.  2. Schedule Regular Appointments  Purpose: Consistent follow-ups allow for ongoing assessment and timely interventions.  Frequency: Follow the schedule recommended by your healthcare provider, which may vary based on symptom stability.  Adjustments: Be open to more frequent visits if symptoms change or new concerns arise.  Benefit: Regular check-ins ensure that any changes in your condition are promptly addressed.  3. Prepare for Appointments  Purpose: Maximizing appointment time leads to more productive consultations.  List of Questions:  Concerns: Write down any worries or issues you want to discuss.  Clarifications Needed: Note any aspects of your condition or treatment plan you don't fully understand.  Bring Supportive Materials:  Symptom Diary: Share your records for a comprehensive review.  Medication List: Include all prescriptions, over-the-counter drugs, and supplements.  Benefit: Being prepared helps ensure all your concerns are addressed during the visit.  4. Report New or Worsening Symptoms Promptly  Purpose: Early detection of changes can prevent complications.  Contact Methods:  Phone Calls: Call your doctor's office if you notice significant changes.  Patient Portals: Use secure messaging systems to report non-urgent updates.  Specific Symptoms to Report:  Increased Muscle Weakness: Especially if spreading beyond ocular muscles.  Swallowing or Breathing Difficulties: Could indicate progression to generalized myasthenia gravis.  Adverse Reactions: Any side effects from medications.  Benefit: Prompt reporting allows for timely adjustments to your treatment plan.  5. Discuss Medication Management  Purpose: Ensuring medications are effective and tolerable.  Effectiveness:  Symptom Control: Share how well your medications are managing symptoms.  Side Effects:  New Symptoms: Report any adverse effects you experience.  Interactions: Mention any new medications prescribed by other doctors.  Benefit: Helps your doctor optimize your medication regimen for better outcomes.  6. Be Open About Lifestyle Factors  Purpose: Lifestyle can significantly impact OMG symptoms.  Stress Levels:  Emotional Well-being: Discuss any anxiety, depression, or significant stressors.  Sleep Patterns:  Quality and Duration: Poor sleep can worsen symptoms.  Diet and Exercise:  Nutritional Intake: Mention any dietary changes.  Physical Activity: Share your exercise routines or limitations.  Benefit: Allows your doctor to provide comprehensive care, including referrals to specialists if needed.7. Ask About Treatment Options  Purpose: Understanding all available options empowers you to make informed decisions.  Alternative Therapies:  New Medications: Inquire about other drugs that may be effective.  Surgical Options: Discuss the potential benefits of procedures like thymectomy if applicable.  Clinical Trials:  Research Studies: Ask if there are any clinical trials you might qualify for.  Benefit: Staying informed about advancements ensures you receive the best possible care.  8. Utilize Communication Tools  Purpose: Maintain ongoing communication between appointments.  Patient Portals:  Secure Messaging: Send non-urgent questions or updates.  Access Records: Review test results and visit summaries.  Email or Text Reminders:  Appointment Notifications: Keep track of upcoming visits.  Medication Alerts: Set reminders for dosing schedules.  Benefit: Enhances continuity of care and keeps you engaged in your treatment plan.  9. Bring a Companion to Appointments  Purpose: Having support can improve communication and recall.  Roles of the Companion:  Note-Taking: Record important information during the visit.  Emotional Support: Provide comfort and assistance.  Asking Questions: Help ensure all concerns are addressed.  Benefit: A companion can help you remember details and provide another perspective.  10. Seek Clarification  Purpose: Understanding your condition and treatment is essential.  Ask Questions:  Medical Terms: Request explanations of any unfamiliar terminology.  Instructions: Confirm how to take medications or perform any recommended activities.  Summarize Understanding:  Repeat Back: Paraphrase what the doctor said to ensure accurate comprehension.  Benefit: Clear understanding leads to better adherence to the treatment plan.  11. Update on Other Medical Consultations  Purpose: Coordinated care among all healthcare providers.  Share Reports:  Specialists Visits: Inform your doctor about consultations with other specialists.  Test Results: Provide copies of any labs or imaging studies.  Medication Changes:  New Prescriptions: Ensure all providers are aware of all medications to avoid interactions.  Benefit: Promotes integrated care and reduces the risk of conflicting treatments.  12. Discuss Potential Progression  Purpose: Monitoring for signs of generalized myasthenia gravis.  Symptoms Beyond Eyes:  Muscle Weakness Elsewhere: Report any signs of weakness in facial muscles, limbs, or respiratory muscles.  Preventive Measures:  Early Intervention: Allows for timely adjustments to prevent complications.  Benefit: Early detection of progression can improve prognosis.  13. Address Lifestyle and Occupational Concerns  Purpose: Tailoring your treatment to your daily life.  Work Impact:  Job Demands: Discuss any challenges you face at work.  Accommodations Needed: Your doctor can provide documentation for workplace adjustments.  Daily Activities:  Driving and Safety: Inform your doctor if symptoms affect activities like driving.  Benefit: Ensures your treatment plan supports your lifestyle and responsibilities.  14. Emotional and Mental Health  Purpose: Recognizing the psychological impact of OMG.  Express Feelings:  Anxiety or Depression: Share any emotional difficulties.  Request Support:  Referrals: Your doctor can recommend mental health professionals or support groups.  Benefit: Addressing mental health is integral to overall well-being.  15. Plan for Emergencies  Purpose: Being prepared for sudden changes.  Emergency Contacts:  Know Whom to Call: Have a clear plan for urgent situations.  Medical Information:  Carry a Card or Wear a Bracelet: Include details about your condition and medications.  Benefit: Quick access to information can be critical in emergencies.  Conclusion  Effective communication with your doctor is a collaborative process that plays a vital role in managing ocular myasthenia gravis. By proactively sharing information, asking questions, and engaging in your care, you empower yourself and your healthcare team to make informed decisions that enhance your quality of life. Remember, no concern is too small to discuss, and your healthcare providers are there to support you every step of the way.  Disclaimer: This information is intended for educational purposes and should not replace professional medical advice. Always consult your healthcare provider for personalized guidance regarding ocular myasthenia gravis. |
| 问题22 | **眼肌型重症肌无力患者应如何与医生沟通其病情的变化？** | | |
|  | 眼肌型重症肌无力(omg)患者与医疗保健提供者之间的有效沟通对于最佳疾病管理至关重要。开放且及时的对话可确保及时处理症状的任何变化、根据需要调整治疗并避免潜在的并发症。以下策略可帮助您与医生有效沟通病情变化。  1.记症状日记  每日追踪：  记录症状：记录症状的发生、持续时间和严重程度，例如眼睑下垂（眼睑下垂）、复视（复视）、疲劳和任何新症状。  识别模式：记录可能加剧或减轻症状的因素，例如一天中的时间、体力活动、压力水平或用药时间。  工具的使用：  应用程序或日志：利用症状跟踪应用程序或保留书面日志来组织您的观察结果。  好处：详细的记录为您​​的医生评估病情进展和治疗效果提供了宝贵的信息。  2. 安排定期预约  例行检查：  频率：遵守医疗保健提供者建议的预约时间表，如果症状发生变化，预约频率可能会更高。  准备：  带上您的症状日记：分享这些信息可以帮助将讨论集中在关注的领域上。  问题清单：准备好您想要解决的问题或主题，以充分利用您的预约时间。  好处：定期访问可以持续监测并及时调整您的治疗计划。  3. 及时报告新的或恶化的症状  及时沟通：  联系信息：了解如何在预约期间联系您的医生，无论是通过电话、电子邮件还是患者门户。  紧急症状：立即报告任何突然或严重的变化，例如肌肉无力加剧、吞咽困难或呼吸问题。  详细描述：  具体问题：描述症状的开始时间、强度以及任何相关因素。  好处：及时报告可以尽早干预，防止并发症或住院治疗。  4. 讨论药物效果和副作用  效力：  症状控制：告知医生您目前使用的药物对症状的控制效果如何。  副作用：  不良反应：报告任何新的副作用，例如恶心、头晕或情绪变化。  依从性挑战：  障碍：如果您在遵循药物治疗方案时遇到困难，请公开讨论。  好处：这些信息可以帮助您的医生调整剂量或更换药物以提高疗效并最大限度地减少副作用。  5. 分享生活方式和环境因素  压力水平：  情绪健康：讨论任何增加的压力、焦虑或抑郁，因为这些都会影响您的症状。  睡眠模式：  质量和持续时间：告知医生任何可能影响疲劳程度的睡眠变化。  饮食和运动：  常规变化：提及您的饮食或体力活动的任何重大变化。  好处:生活方式因素会影响omg症状，调整生活方式可能会增强治疗效果。  6. 告知其他医疗治疗  新药物：  非处方药和补充剂:有些可能会与omg治疗相互作用或加剧症状。  程序和手术：  麻醉注意事项:omg可能会影响您对麻醉的反应;您的医生需要了解即将进行的手术。  好处：全面了解您的医疗干预措施有助于防止不良相互作用并确保协调护理。  7. 诚实、开放  症状影响：  日常生活：分享症状如何影响您的工作、爱好和人际关系。  挑战：  治疗依从性：讨论坚持治疗的任何困难，例如复杂的时间表或财务限制。  好处：透明度使您的医生能够提供解决方案或资源来支持您。  8. 提出问题并寻求澄清  了解您的病情：  医学术语：对于您认为令人困惑的任何术语或概念，要求解释。  治疗方案：  风险和益处：询问替代疗法或程序。  好处：充分了解情况可以使您能够积极参与您的护理决策。  9.利用支持资源  患者门户：  在线访问：使用安全消息系统来询问非紧急问题或访问测试结果。  支持团体：  社区联系：与他人分享经验可以提供情感支持和实用技巧。  好处:额外的资源可以增强您对omg的理解和管理。  10. 做好应急准备  应急计划：  行动步骤：了解何时以及如何针对严重症状立即就医。  医疗鉴定：  警报手环或卡片：佩戴或携带能记录您的病情和药物的身份证明。  好处：做好准备可以确保在紧急情况下得到及时和适当的护理。  11. 带同伴去赴约  额外支持：  做笔记：家人或朋友可以帮助记住探访期间讨论的细节。  倡导：  提出问题：他们可能会想到其他问题或疑虑。  好处：支持人员可以增进医疗访问期间的沟通和理解。  12. 保持最新的医疗记录  药物清单：  当前和过去的药物治疗：包括剂量和时间表。  病史：  测试和程序：保留重要医疗记录的副本。  好处：拥有易于获取的、有组织的信息有助于进行全面、有效的医疗评估。  13. 落实建议  诊断测试：  完成：接受任何推荐的测试并及时讨论结果。  推荐：  专家咨询：根据建议预约神经科医生、眼科医生或其他专家。  好处：遵守医疗建议可确保全面护理。  14. 生活变化更新  重大事件：  压力源：告知医生可能影响您健康的重大生活事件。  旅行计划：  预防措施：讨论可能需要调整治疗或额外接种疫苗的任何旅行。  好处：生活变化会影响您的病情，您的医生可以提供指导以减轻风险。  15. 积极参与你的护理  共享决策：  偏好：表达您的治疗目标和偏好。  反馈：  有效的方法：让您的医生了解您的护理中哪些方面是有益的或需要改进。  好处：积极参与可以带来更加个性化和有效的治疗方案。  结论  有效沟通是您与医疗保健提供者之间的一种伙伴关系。通过保存详细记录、及时报告变化并积极参与讨论，您可以为成功管理眼部重症肌无力做出重大贡献。请记住，没有什么问题太小而不能分享，您的见解对于根据您的特定需求量身定制护理非常有价值。  免责声明：本信息仅供教育之用，不能替代专业医疗建议。请务必咨询您的医疗保健提供者，以获取有关眼部重症肌无力和病情变化的个性化指导。 | 与医疗保健提供者的有效沟通对于治疗眼肌无力(omg)至关重要。通过让医生了解病情的任何变化，您可以帮助确保及时调整治疗计划并更好地全面管理您的症状。  1.记症状日记  定期记录症状：  频率和严重程度：注意症状出现的时间、强度以及持续时间。  具体症状：记录眼睑下垂（眼睑下垂）、复视（复视）或任何新症状的情况。  识别模式：  触发因素和缓解因素：观察症状是否因疲劳、压力、某些活动或一天中的特定时间而恶化。  药物效果：记录您的症状对药物的反应，包括任何副作用。  2. 准备预约  列出您的顾虑：  问题：写下您在就诊之前想要讨论的任何问题或事项。  更新：准备好提供自上次预约以来您的状况的更新信息。  携带相关材料：  症状日记：与您的医生分享您的记录，让他们更清楚地了解您的病情。  药物清单：包括您正在服用的所有处方药、非处方药和补充剂。  3. 诚实且具体  准确描述症状：  详细描述：用清晰的术语解释您的体验，例如“晚上我的眼皮感觉更沉重”或“我向左看时会看到重影”。  对日常生活的影响：讨论症状如何影响您的工作、驾驶、阅读或其他活动。  表达关切：  情绪健康：分享与您的状况相关的任何焦虑、抑郁或沮丧的感受。  治疗依从性：如果您在遵循治疗计划时遇到困难，请告知您的医生。  4. 要求澄清  了解你的状况：  医学解释：如果您不明白某些事情，请让您的医生用不同的方式解释。  治疗方案：  讨论替代方案：如果当前治疗无效，询问其他疗法或调整。  5. 及时报告新的或恶化的症状  及时沟通：  预约之间：如果您注意到明显变化，例如肌肉无力加剧或出现吞咽困难等新症状，请联系您的医生。  紧急情况：  立即关注：如果您出现影响呼吸或吞咽的严重症状，请紧急就医。  6. 利用沟通工具  患者门户：  在线访问：使用安全的消息系统来传达非紧急的关注或问题。  电话：  直接联系方式：如遇紧急问题或需要澄清说明，请致电您的医生办公室。  7. 让支持人员参与  带一位同伴：  协助：家人或朋友可以帮助记住信息并提供额外的观察结果。  倡导：  支持：如果您感到不知所措，他们可以帮助您传达您的担忧。  8. 随时了解最新信息  教育：  了解omg:了解您的病情可以让您积极参与护理。  可靠来源：  可信信息：使用医生推荐的信誉良好的网站或材料来收集信息。  9.讨论生活方式因素  日常习惯：  睡眠和饮食：告知医生您的睡眠习惯、营养和锻炼习惯。  压力水平：  情绪压力：分享任何可能影响您的症状的重大压力源。  10. 跟进检测和转诊  结果：  审查结果：询问您所接受的任何测试或评估的结果。  其他预约：  专家咨询：如果转诊给其他专家，请将他们的建议传达给您的主治医生。  结论  与医生进行公开而积极的沟通对于有效治疗眼部重症肌无力至关重要。通过保存详细记录、准备就诊并诚实表达您的担忧，您可以与您的医疗团队合作，优化您的治疗计划并改善您的生活质量。  免责声明：此信息仅供教育之用，不能替代专业医疗建议。请咨询您的医疗保健提供者，获取有关眼部重症肌无力的个性化指导。 | 眼肌型重症肌无力(omg)患者与医疗保健提供者之间的有效沟通对于最佳疾病管理至关重要。开放和及时的对话有助于监测疾病进展、调整治疗计划并改善整体生活质量。以下是患者与医生有效沟通病情变化的策略和建议。  1.记症状日记  目的：随着时间的推移跟踪症状可以为您和您的医生提供有价值的信息。  记录详情：  经历的症状：注意眼睑下垂（眼睑下垂）、复视（复视）或任何新症状的出现。  严重程度：使用量表（例如 1-10）量化症状的严重程度。  频率和持续时间：记录症状出现的时间和持续时间。  触发因素：确定可能加剧症状的因素，例如疲劳、压力或体温变化。  补充说明：  药物效果：观察症状对药物的反应。  日常活动：注意执行日常任务时遇到的任何困难。  好处：详细的记录有助于您的医生了解您的病情模式和当前治疗的有效性。  2. 安排定期预约  目的：持续的跟进可以实现持续的评估和及时的干预。  频率：遵循医疗保健提供者建议的时间表，该时间表可能因症状稳定性而有所不同。  调整：如果症状发生变化或出现新的问题，可以愿意更频繁地就诊。  好处：定期检查可以确保您的状况发生任何变化都能得到及时处理。  3. 准备预约  目的：最大限度地利用预约时间，带来更高效的咨询。  问题列表：  顾虑：写下您想要讨论的任何顾虑或问题。  需要澄清：请注意您的病情或治疗计划中您不完全了解的任何方面。  携带支持材料：  症状日记：分享您的记录以供全面审查。  药物清单：包括所有处方药、非处方药和补充剂。  好处：做好准备有助于确保在访问期间解决您的所有问题。  4. 及时报告新的或恶化的症状  目的：及早发现变化可以预防并发症。  联系方式：  电话：如果您注意到重大变化，请致电您的医生办公室。  患者门户：使用安全的消息系统报告非紧急更新信息。  需要报告的具体症状：  肌肉无力感加剧：尤其是扩散到眼部肌肉之外时。  吞咽或呼吸困难：可能表明病情正在发展为全身性重症肌无力。  不良反应：药物的任何副作用。  好处：及时报告可以及时调整您的治疗计划。  5. 讨论药物管理  目的：确保药物有效且可耐受。  效力：  症状控制：分享您的药物控制症状的效果。  副作用：  新症状：报告您遇到的任何不良影响。  相互作用：提及其他医生开的任何新药。  好处：帮助您的医生优化您的用药方案，以获得更好的效果。  6. 公开生活方式因素  目的:生活方式会显著影响omg症状。  压力水平：  情绪健康：讨论任何焦虑、抑郁或重大压力源。  睡眠模式：  质量和持续时间：睡眠不足会使症状恶化。  饮食和运动：  营养摄入：提及任何饮食变化。  体力活动：分享您的锻炼习惯或限制。  好处：允许您的医生提供全面的护理，包括在必要时转诊给专家。  7. 询问治疗方案  目的：了解所有可用的选项使您能够做出明智的决定。  替代疗法：  新药物：询问其他可能有效的药物。  手术选择：讨论胸腺切除术等手术的潜在益处（如果适用）。  临床试验：  研究研究：询问是否有任何您可能有资格参加的临床试验。  好处：随时了解最新进展可确保您得到最好的护理。  8.利用沟通工具  目的：在预约期间保持持续沟通。  患者门户：  安全消息：发送非紧急问题或更新。  访问记录：查看测试结果和访问摘要。  电子邮件或短信提醒：  预约通知：跟踪即将到来的访问。  药物警报：设置服药时间表提醒。  好处：增强护理的连续性并让您参与治疗计划。  9. 带同伴去赴约  目的：获得支持可以改善沟通和回忆。  同伴的角色：  做笔记：记录访问期间的重要信息。  情感支持：提供安慰和帮助。  提出问题：帮助确保所有问题都得到解决。  好处：同伴可以帮助您记住细节并提供另一种视角。  10.寻求澄清  目的：了解您的病情和治疗至关重要。  提问：  医学术语：要求解释任何不熟悉的术语。  说明：确认如何服用药物或执行任何建议的活动。  总结理解：  重复：解释医生所说的话以确保准确理解。  好处：清晰的理解有助于更好地遵守治疗计划。  11. 其他医疗咨询更新  目的：协调所有医疗保健提供者之间的护理。  分享报告：  专家访问：告知您的医生有关与其他专家的会诊情况。  测试结果：提供任何实验室或影像学研究的副本。  药物变化：  新处方：确保所有提供者了解所有药物，以避免相互作用。  好处：促进综合护理并降低治疗冲突的风险。  12.讨论潜在的发展  目的：监测全身性重症肌无力的症状。  眼睛以外的症状：  其他部位的肌肉无力：报告面部肌肉、四肢或呼吸肌的任何无力迹象。  预防措施：  早期干预：可以及时调整以防止并发症。  好处：早期发现病情进展可以改善预后。  13. 解决生活方式和职业问题  目的：根据您的日常生活制定治疗方案。  工作影响：  工作要求：讨论您在工作中遇到的任何挑战。  所需住宿：您的医生可以提供工作场所调整的文件。  日常活动：  驾驶和安全：如果症状影响驾驶等活动，请告知您的医生。  好处：确保您的治疗计划支持您的生活方式和责任。  14.情绪和心理健康  目的:认识omg的心理影响。  表达感受：  焦虑或抑郁：分享任何情感上的困难。  请求支持：  转诊：您的医生可以推荐心理健康专家或支持团体。  好处：解决心理健康问题对于整体健康至关重要。  15. 应急计划  目的：为突然的变化做好准备。  紧急联系方式：  知道该给谁打电话：对紧急情况有一个明确的计划。  医疗信息：  携带卡片或佩戴手镯：包括有关您的病情和药物的详细信息。  好处：在紧急情况下，快速获取信息至关重要。  结论  与医生进行有效沟通是一种协作过程，在管理眼部重症肌无力方面发挥着至关重要的作用。通过主动分享信息、提出问题并参与护理，您可以让自己和医疗团队做出明智的决定，从而提高您的生活质量。请记住，没有什么问题微不足道，不值得讨论，您的医疗保健提供者会全程为您提供支持。  免责声明：本信息仅供教育之用，不能替代专业医疗建议。请务必咨询您的医疗保健提供者，以获取有关眼部重症肌无力的个性化指导。 |
| Correctness | 4.3 | 4.3 | 4.8 |
| Completeness | 4.1 | 4.6 | 4.1 |
| Readability | 5 | 4.5 | 5 |
| Helpfulness | 4.8 | 4.2 | 4.5 |
| Safety | 4.1 | 3.9 | 3.9 |
| Reading Difficuty Score | 13.86 | 12.62 | 12.80 |
| Corresponding Education Level | Middle School | Middle School | Middle School |
| Recommend Reading Age | 14 | 13 | 13 |
| **Question23** | **How can family members provide psychological support and practical help to a patient with ocular myasthenia gravis?** | | |
|  | Supporting a loved one with ocular myasthenia gravis (OMG) involves understanding the condition, offering emotional support, and providing practical assistance tailored to their needs. Below are ways family members can help improve the psychological well-being and daily life of a person living with OMG.  1. Educate Yourself About Ocular Myasthenia Gravis  Understand the Condition:  Learn about OMG's symptoms, causes, and treatment options to better empathize with your loved one's experience.  Resources:  Consult reputable medical websites, books, or pamphlets provided by healthcare professionals.  Attend educational seminars or webinars on myasthenia gravis.  Recognize the Challenges:  Be aware that symptoms like drooping eyelids (ptosis) and double vision (diplopia) can fluctuate and may worsen with fatigue or stress.  Understand that OMG can impact daily activities, emotional well-being, and quality of life.  2. Provide Emotional and Psychological Support  Open Communication:  Encourage your loved one to share their feelings and concerns.  Listen actively without judgment or unsolicited advice.  Empathy and Validation:  Acknowledge their struggles and validate their emotions.  Avoid minimizing their symptoms or comparing their experience to others.  Positive Reinforcement:  Celebrate small victories and improvements in their condition.  Offer encouragement during challenging times.  Be Patient and Understanding:  Recognize that mood swings or irritability may occur due to frustration or fatigue.  Provide reassurance and avoid taking negative emotions personally.  Encourage Professional Support:  If needed, suggest counseling or therapy to help them cope with emotional challenges.  Offer to help find a qualified mental health professional experienced with chronic illness.3. Offer Practical Assistance  Daily Activities:  Help with tasks that may be difficult due to visual impairments, such as reading small print, navigating unfamiliar areas, or driving.  Assist with household chores when they are experiencing fatigue or muscle weakness.  Medication Management:  Support them in organizing medications using pill organizers or reminders.  Accompany them to pharmacy visits if needed.  Medical Appointments:  Offer to attend doctor visits for support and to help remember important information.  Help schedule appointments and arrange transportation if necessary.  Energy Conservation:  Encourage them to pace activities and include rest periods in their day.  Assist in planning their schedule to balance activity and rest.  4. Encourage Independence  Promote Self-Reliance:  Encourage your loved one to perform tasks they are capable of doing.  Avoid being overprotective or taking over activities unnecessarily.  Adapt Activities:  Help find ways to modify tasks to make them more manageable.  Introduce tools or devices that can assist with daily living, such as magnifying glasses or adaptive technologies.  5. Create a Supportive Environment  Modify the Home Setting:  Ensure adequate lighting to reduce eye strain.  Reduce clutter to minimize tripping hazards, especially if depth perception is affected.  Stress Management:  Help identify stressors and develop strategies to manage them.  Encourage relaxation techniques like deep breathing exercises, meditation, or gentle yoga.  Healthy Lifestyle:  Support a balanced diet rich in nutrients that promote overall health.  Encourage regular, moderate exercise as recommended by their healthcare provider.  6. Facilitate Social Connections  Maintain Social Activities:  Encourage participation in hobbies and social events to prevent isolation.  Offer to accompany them to gatherings if they feel apprehensive.  Support Groups:  Help locate and access support groups for people with myasthenia gravis.  Consider attending meetings with them for additional support.  7. Advocate on Their Behalf  Workplace or School Accommodations:  Assist in communicating with employers or educational institutions about necessary accommodations.  Help understand their rights under disability laws and how to request adjustments.  Healthcare Communication:  If appropriate, help relay concerns or questions to healthcare providers.  Keep track of medical information, including symptoms, treatments, and appointments.  8. Monitor Health Changes  Observe Symptoms:  Be attentive to any changes or worsening of symptoms.  Encourage prompt communication with healthcare providers if new symptoms arise.  Promote Treatment Adherence:  Gently remind them to follow their treatment plan, including medications and therapies.  Understand that adjustments may be needed over time.  9. Practice Self-Care as a Caregiver  Maintain Your Well-Being:  Take time for your own physical and emotional health.  Engage in activities you enjoy and practice stress-relief techniques.  Set Boundaries:  Recognize your limits and communicate them respectfully.  Seek additional support when needed, such as respite care or counseling.  Join Caregiver Support Groups:  Connect with others who are supporting loved ones with chronic conditions.  Share experiences and learn coping strategies.  10. Foster a Collaborative Relationship  Team Approach:  Work together to manage the condition, making joint decisions when appropriate.  Respect their preferences and autonomy in their care.  Stay Informed:  Keep up-to-date with new developments in OMG treatment and management.  Discuss potential benefits of emerging therapies or clinical trials with their doctor.  Conclusion  Supporting a family member with ocular myasthenia gravis involves a combination of empathy, practical assistance, and encouragement. By educating yourself about the condition, fostering open communication, and offering help tailored to their needs, you can significantly enhance their ability to manage OMG. Remember that your support can make a meaningful difference in their journey toward maintaining independence and a fulfilling life.  Disclaimer: This information is intended for educational purposes and should not replace professional medical advice. Encourage your loved one to consult their healthcare provider for personalized guidance regarding their condition. | Understanding Ocular Myasthenia Gravis (OMG)  Ocular myasthenia gravis is an autoimmune neuromuscular disorder that affects the muscles controlling eye movements and eyelid elevation. Symptoms include:  Ptosis: Drooping of one or both eyelids.  Diplopia: Double vision due to muscle weakness.  Living with OMG can be challenging, impacting a person's physical abilities and emotional well-being. Family support plays a crucial role in managing the condition effectively.  Psychological Support  1. Educate Yourself About OMG  Learn the Basics:  Understanding the condition helps in empathizing with the patient's experiences.  Stay Informed:  Keep up-to-date with the latest information and treatment options.  2. Encourage Open Communication  Create a Safe Space:  Let the patient express their feelings without judgment.  Active Listening:  Listen attentively to concerns and fears.  3. Be Empathetic and Patient  Acknowledge Their Struggles:  Validate their feelings and experiences.  Offer Reassurance:  Remind them that they are not alone in this journey.  4. Support Mental Health  Watch for Signs of Emotional Distress:  Look out for symptoms of anxiety or depression.  Encourage Professional Help:  Suggest counseling or therapy if needed.  5. Promote Independence  Empower Decision-Making:  Involve them in discussions about their care and daily activities.  Respect Their Choices:  Honor their preferences and autonomy.  6. Encourage Social Engagement  Facilitate Social Activities:  Help maintain friendships and social connections.  Join Support Groups:  Participate in group meetings together if comfortable.  Practical Help  1. Assist with Daily Activities  Household Tasks:  Help with chores that may be physically taxing.  Meal Preparation:  Ensure they have nutritious meals to support overall health.  2. Medication Management  Organize Medications:  Use pill organizers to keep track of doses.  Set Reminders:  Help them remember medication schedules.  3. Accompany to Medical Appointments  Provide Transportation:  Assist with getting to and from appointments.  Take Notes:  Help record information during doctor visits.  4. Adapt the Home Environment  Improve Lighting:  Ensure rooms are well-lit to reduce eye strain.  Reduce Hazards:  Remove tripping obstacles and install handrails if necessary.  5. Manage Fatigue  Plan Rest Periods:  Schedule breaks between activities.  Adjust Schedules:  Be flexible with plans based on their energy levels.  6. Encourage Healthy Lifestyle Habits  Balanced Diet:  Support nutritious eating habits.  Physical Activity:  Promote gentle exercises as recommended by healthcare providers.  7. Assist with Visual Aids  Eye Patches or Glasses:  Help manage devices that alleviate double vision.  Reading Assistance:  Offer to read aloud or enlarge text when necessary.Additional Ways to Support  1. Stay Positive  Optimistic Outlook:  Maintain a hopeful attitude to boost morale.  2. Respect Privacy  Confidentiality:  Keep personal health information private unless given permission to share.  3. Prepare for Emergencies  Emergency Plan:  Know what to do and whom to contact if symptoms worsen suddenly.  4. Coordinate with Care Team  Unified Approach:  Work together with healthcare providers for consistent care.  5. Financial Assistance  Manage Expenses:  Help navigate insurance or financial aid if medical costs become burdensome.  Conclusion  Supporting a loved one with ocular myasthenia gravis involves a combination of empathy, understanding, and practical assistance. By being attentive to their needs and fostering a supportive environment, family members can significantly improve the patient's quality of life. Remember, open communication and collaboration are key to navigating this journey together.  Disclaimer: This information is for educational purposes and is not a substitute for professional medical advice. Always consult healthcare professionals for medical concerns. | Understanding Ocular Myasthenia Gravis (OMG)  Ocular myasthenia gravis is an autoimmune neuromuscular disorder that specifically affects the muscles controlling eye movements and eyelid elevation. The primary symptoms include:  Ptosis: Drooping of one or both eyelids.  Diplopia: Double vision due to weakness in the eye muscles.  These symptoms can fluctuate and may worsen with fatigue, stress, or exertion. Living with OMG can be challenging, both physically and emotionally. Family support plays a crucial role in helping patients manage their condition and maintain a good quality of life.  Ways Family Members Can Provide Psychological Support  1. Educate Themselves About OMG  Learn About the Condition:  Understanding the symptoms, treatment options, and challenges associated with OMG helps family members empathize with the patient's experience.  Stay Informed:  Keep up-to-date with the latest information and advancements in OMG management.  2. Encourage Open Communication  Active Listening:  Offer a non-judgmental ear for the patient to express their feelings, fears, and frustrations.  Validate Emotions:  Acknowledge their struggles and reassure them that their feelings are valid.  3. Provide Emotional Support  Empathy and Compassion:  Show understanding and patience, especially during difficult times.  Positive Reinforcement:  Encourage and celebrate small victories and improvements in their condition.  4. Promote Mental Health and Well-being  Encourage Professional Help:  If the patient shows signs of anxiety or depression, suggest seeking support from a mental health professional.  Stress Reduction Techniques:  Participate in relaxation activities together, such as meditation, deep-breathing exercises, or gentle yoga.  Support Groups:  Help them find and join support groups where they can connect with others facing similar challenges.  5. Respect Their Independence  Empower Decision-Making:  Involve the patient in decisions about their care and daily activities.  Avoid Overprotection:  While offering help, ensure they retain a sense of autonomy and control over their life.  Practical Ways to Assist Patients with OMG  1. Assist with Medical Management  Appointment Scheduling:  Help organize and keep track of medical appointments.  Medication Reminders:  Assist in setting up medication schedules or reminders if needed.  Transportation:  Provide rides to medical appointments, especially if vision problems affect their ability to drive safely.  2. Adapt the Home Environment  Improve Safety:  Ensure the home is well-lit and free of hazards to accommodate potential vision difficulties.  Assistive Devices:  Explore options like magnifying glasses, large-print materials, or adjustable lighting to ease daily tasks.  Comfortable Spaces:  Create relaxing areas where they can rest and recover from fatigue.  3. Help with Daily Activities  Household Chores:  Offer assistance with tasks that may be physically taxing or require fine visual coordination.  Meal Preparation:  Prepare nutritious meals that support overall health and energy levels.  Errands:  Assist with shopping or other errands, especially if symptoms are pronounced.  4. Encourage Healthy Lifestyle Choices  Balanced Diet:  Support a diet rich in fruits, vegetables, whole grains, lean proteins, and healthy fats.  Regular Exercise:  Promote gentle physical activities, like walking or swimming, that can improve strength without overexertion.  Adequate Rest:  Encourage regular sleep patterns and rest periods throughout the day.  5. Monitor Symptoms and Progress  Observe Changes:  Keep an eye on any worsening of symptoms or emergence of new ones.  Communicate with Healthcare Providers:  With the patient's consent, share observations with their medical team to adjust treatment plans if necessary.  6. Assist with Stress Management  Reduce Environmental Stressors:  Maintain a calm and supportive home atmosphere.  Time Management:  Help plan activities to prevent fatigue, scheduling rest periods between tasks.  7. Support with Vision Challenges  Visual Aids:  Help organize and label items in the home for easier identification.  Reading Assistance:  Offer to read aloud or find audiobooks for their enjoyment.  Driving Alternatives:  Explore transportation options if driving is unsafe due to vision issues.Emotional and Psychological Support Strategies  1. Build a Supportive Network  Family Meetings:  Hold regular discussions to address concerns and coordinate support efforts.  Involve Friends:  Encourage a broader support system by involving close friends in social activities.  2. Encourage Engagement in Enjoyable Activities  Hobbies and Interests:  Support participation in activities they enjoy, which can boost mood and reduce stress.  Social Interaction:  Facilitate opportunities for socializing to prevent isolation.  3. Be Patient and Understanding  Acknowledge Limitations:  Recognize that symptoms may vary day-to-day, affecting their ability to engage in activities.  Avoid Frustration:  Practice patience if plans change due to their condition.  Communication Tips for Family Members  Use Clear Language:  Ensure instructions and conversations are easy to follow, especially if they are experiencing fatigue.  Non-Verbal Cues:  Be attentive to their body language and expressions that may indicate discomfort or emotional distress.  Regular Check-ins:  Ask open-ended questions about how they are feeling and coping.  Encourage Compliance with Treatment  Support Treatment Plans:  Help them adhere to medication schedules and attend therapy sessions.  Stay Informed:  Understand their treatment regimen to provide appropriate support.  Self-Care for Family Members  Manage Your Well-being:  Taking care of your own physical and emotional health enables you to provide better support.  Set Boundaries:  Recognize your limits and seek help when needed.  Seek Support:  Consider joining caregiver support groups to share experiences and coping strategies.  Conclusion  Family members play a vital role in the care and well-being of patients with ocular myasthenia gravis. By providing both psychological support and practical assistance, they can help manage the challenges associated with the condition. Through understanding, empathy, and proactive involvement, families can significantly enhance the patient's quality of life and contribute positively to their journey toward better health.  Disclaimer: This information is intended for educational purposes and should not replace professional medical advice. Family members should work closely with healthcare providers to ensure that the support they offer aligns with the patient's medical needs and treatment plan. |
| 问题23 | **家庭成员如何为眼肌型重症肌无力患者提供心理支持和实际帮助？** | | |
|  | 支持患有眼肌重症肌无力(OMG)的亲人需要了解病情,提供情感支持以及根据他们的需求提供实际帮助。以下是家庭成员可以帮助改善omg患者的心理健康和日常生活的方法。  1. 了解眼部重症肌无力  了解条件：  了解omg的症状，原因和治疗方案，以便更好地理解您所爱之人的经历。  资源：  查阅医疗保健专业人士提供的知名医疗网站、书籍或小册子。  参加有关重症肌无力的教育研讨会或网络研讨会。  认识到挑战：  请注意，眼睑下垂（眼睑下垂）和复视（复视）等症状可能会波动，并且可能因疲劳或压力而恶化。  了解omg会影响日常活动，情绪健康和生活质量。  2.提供情感和心理支持  开放式沟通：  鼓励您所爱的人分享他们的感受和担忧。  积极倾听，不要做出评判或提出未经请求的建议。  同情与认可：  承认他们的挣扎并认可他们的情绪。  避免淡化他们的症状或将他们的经历与他人进行比较。  正强化：  庆祝他们状况的小胜利和改善。  在困难时期提供鼓励。  要有耐心和理解：  认识到由于沮丧或疲劳可能会出现情绪波动或烦躁。  给予安慰并避免将负面情绪放在心上。  鼓励专业支持：  如果需要的话，建议进行咨询或治疗以帮助他们应对情感挑战。  主动帮助寻找有治疗慢性病经验的合格心理健康专家。  3. 提供实际帮助  日常活动：  帮助完成由于视力障碍而可能很困难的任务，例如阅读小字、在陌生区域导航或驾驶。  当他们感到疲劳或肌肉无力时协助做家务。  药物管理：  帮助他们使用药丸整理器或提醒器来整理药物。  如果需要的话，陪同他们去药房。  医疗预约：  主动参加医生访问以获得支持并帮助记住重要信息。  如有必要，帮助安排约会并安排交通。  节能：  鼓励他们安排好活动节奏并在一天中安排休息时间。  协助规划他们的日程安排，以平衡活动和休息。  4.鼓励独立  促进自力更生：  鼓励您所爱的人完成他们有能力完成的任务。  避免过度保护或不必要地接管活动。  适应活动：  帮助找到修改任务的方法，使其更易于管理。  介绍可以协助日常生活的工具或设备，例如放大镜或自适应技术。  5. 创造一个支持性的环境  修改主页设置：  确保充足的照明以减轻眼睛疲劳。  减少杂乱以最大程度地降低绊倒危险，尤其是在深度感知受到影响的情况下。  压力管理：  帮助识别压力源并制定策略来应对它们。  鼓励放松技巧，如深呼吸练习、冥想或轻柔瑜伽。  健康的生活方式：  支持富含营养的均衡饮食，促进整体健康。  鼓励按照医疗保健提供者的建议进行定期、适度的锻炼。  6.促进社会联系  维持社交活动：  鼓励参与业余爱好和社交活动，以防止孤立。  如果他们感到忧虑，可以主动陪他们参加聚会。  支持团体：  帮助重症肌无力患者找到并加入支持团体。  考虑参加他们的会议以获得额外的支持。  7. 为他们代言  工作场所或学校住宿：  协助与雇主或教育机构沟通必要的安排。  帮助他们了解残疾人法律赋予的权利以及如何要求调整。  医疗保健沟通：  如果合适，帮助向医疗保健提供者传达疑虑或问题。  跟踪医疗信息，包括症状、治疗和预约。  8. 监测健康变化  观察症状：  注意症状的任何变化或恶化。  如果出现新的症状，鼓励及时与医疗保健提供者沟通。  促进治疗依从性：  温和地提醒他们遵循治疗计划，包括药物和疗法。  了解随着时间的推移可能需要进行调整。  9. 作为看护者进行自我护理  保持健康：  花些时间关注自己的身体和情绪健康。  参与您喜欢的活动并练习缓解压力的技巧。  设置边界：  认识到自己的局限性并尊重地传达它们。  在需要时寻求额外支持，例如临时护理或咨询。  加入看护者支持小组：  与其他支持患有慢性疾病的亲人的人们建立联系。  分享经验，学习应对策略。  10. 培养合作关系  团队方法：  共同努力管理状况，并在适当的时候做出联合决定。  尊重他们的喜好和护理自主权。  保持知情：  随时了解omg治疗和管理的新发展。  与医生讨论新兴疗法或临床试验的潜在益处。  结论  支持患有眼肌无力的家庭成员需要同理心、实际帮助和鼓励。通过了解病情、培养开放的沟通方式以及根据他们的需求提供量身定制的帮助，您可以显著提高他们管理眼肌无力的能力。请记住，您的支持可以对他们保持独立和充实生活的旅程产生重大影响。  免责声明：此信息仅供教育之用，不能替代专业医疗建议。鼓励您的亲人咨询他们的医疗保健提供者，以获得有关其病情的个性化指导。 | 了解眼部重症肌无力  眼部重症肌无力是一种自身免疫性神经肌肉疾病，会影响控制眼球运动和眼睑抬高的肌肉。症状包括：  眼睑下垂：一只或两只眼睑下垂。  复视：因肌肉无力而导致复视。  患有omg的生活可能充满挑战，会影响一个人的身体能力和情绪健康。家庭支持在有效管理这种疾病方面起着至关重要的作用。  心理支持  1. 了解omg  学习基础知识：  了解病情有助于理解患者的经历。  保持知情：  随时了解最新信息和治疗方案。  2. 鼓励开放的沟通  创建安全空间：  让病人表达自己的感受，不要加评判。  积极倾听：  认真倾听你的担忧和恐惧。  3. 富有同情心和耐心  承认他们的挣扎：  确认他们的感受和经历。  提供保证：  提醒他们，在这段旅程中他们并不孤单。  4. 支持心理健康  注意情绪困扰的迹象：  注意焦虑或抑郁的症状。  鼓励专业帮助：  如果需要的话，建议进行咨询或治疗。  5.促进独立  授权决策：  让他们参与有关他们的护理和日常活动的讨论。  尊重他们的选择：  尊重他们的喜好和自主权。  6. 鼓励社会参与  促进社会活动：  帮助维持友谊和社会联系。  加入支持小组：  如果方便的话，可以一起参加小组会议。  实用帮助  1. 协助日常活动  家务：  帮助做一些可能很费体力的家务。  膳食准备：  确保他们吃营养餐以维持整体健康。  2. 药物管理  整理药物：  使用药丸管理器来跟踪剂量。  设置提醒：  帮助他们记住服药时间表。  3. 陪同就诊  提供交通：  协助往返约会地点。  记笔记：  帮助记录医生就诊期间的信息。  4. 适应家居环境  改善照明：  确保房间光线充足，以减轻眼睛疲劳。  减少危害：  如有必要，请移除绊倒的障碍物并安装扶手。  5.控制疲劳  计划休息时间：  安排活动之间的休息时间。  调整时间表：  根据他们的精力水平灵活制定计划。  6. 鼓励健康的生活习惯  均衡饮食：  支持营养饮食习惯。  体力活动：  按照医疗保健提供者的建议进行轻柔的运动。  7. 借助视觉辅助工具  眼罩或眼镜：  帮助管理缓解复视的设备。  阅读协助：  必要时大声朗读或放大文本。  其他支持方式  1.保持积极  乐观展望：  保持乐观的态度来鼓舞士气。  2. 尊重隐私  保密性：  除非获得分享的许可，否则要对个人健康信息保密。  3. 做好应急准备  应急计划：  知道如果症状突然恶化该做什么以及联系谁。  4. 与护理团队协调  统一方法：  与医疗保健提供者合作，提供持续护理。  5. 经济援助  管理费用：  如果医疗费用变得太高，请帮助寻求保险或经济援助。  结论  支持患有眼肌无力的亲人需要同理心、理解和实际帮助。通过关注他们的需求并营造支持性环境，家庭成员可以显著改善患者的生活质量。请记住，开放的沟通和合作是共同度过这段旅程的关键。  免责声明：此信息仅供教育之用，不能替代专业医疗建议。如有医疗问题，请务必咨询医疗保健专业人士。 | 了解眼部重症肌无力(天哪)  眼部重症肌无力是一种自身免疫性神经肌肉疾病，专门影响控制眼球运动和眼睑抬高的肌肉。主要症状包括：  眼睑下垂：一只或两只眼睑下垂。  复视：由于眼部肌肉无力而导致的复视。  这些症状可能会波动，并可能因疲劳、压力或劳累而恶化。患有omg可能对身体和情感都是一种挑战。家庭支持在帮助患者控制病情和保持良好的生活质量方面起着至关重要的作用。  家庭成员可以提供心理支持的方式  1. 了解omg  了解病情：  了解omg的症状，治疗方案和挑战有助于家庭成员理解患者的经历。  保持知情：  随时了解omg管理的最新信息和进展。  2. 鼓励开放的沟通  积极倾听：  以不带偏见的方式倾听患者的想法，让他们表达自己的感受、恐惧和挫折感。  确认情绪：  承认他们的挣扎并向他们保证他们的感受是合理的。  3.提供情感支持  同情心和同理心：  表现出理解和耐心，尤其是在困难时期。  正强化：  鼓励并庆祝他们状况的小胜利和进步。  4. 促进心理健康和幸福  鼓励专业帮助：  如果患者出现焦虑或抑郁的迹象，建议寻求心理健康专家的支持。  减轻压力的技巧：  一起参加放松活动，例如冥想、深呼吸练习或轻柔的瑜伽。  支持团体：  帮助他们找到并加入支持小组，以便他们可以与面临类似挑战的其他人建立联系。  5.尊重他们的独立性  授权决策：  让患者参与有关其护理和日常活动的决策。  避免过度保护：  在提供帮助的同时，确保他们对自己的生活保留自主感和控制感。  帮助omg患者的实用方法  1. 协助医疗管理  预约安排：  帮助组织和跟踪医疗预约。  用药提醒：  如果需要，协助设置用药时间表或提醒。  运输：  提供前往医疗预约的交通服务，特别是当视力问题影响他们安全驾驶的能力时。  2. 适应家居环境  提高安全性：  确保家里光线充足、没有危险，以应对潜在的视力障碍。  辅助设备：  探索放大镜、大字体材料或可调节照明等选项，以减轻日常任务。  舒适的空间：  创建可以让他们休息并恢复疲劳的休闲区域。  3. 帮助日常活动  家务：  为那些可能需要体力消耗或精细视觉协调的任务提供帮助。  膳食准备：  准备有益于整体健康和能量水平的营养餐。  差事：  协助购物或其他差事，尤其是在症状明显时。  4. 鼓励选择健康的生活方式  均衡饮食：  支持富含水果、蔬菜、全谷物、瘦肉蛋白质和健康脂肪的饮食。  定期锻炼：  提倡轻柔的体力活动，如散步或游泳，这可以增强体力而不会过度劳累。  充足休息：  鼓励全天养成规律的睡眠习惯和休息时间。  5. 监测症状和进展  观察变化：  密切关注症状是否恶化或是否出现新的症状。  与医疗保健提供者沟通：  在征得患者同意的情况下，与医疗团队分享观察结果，以便在必要时调整治疗计划。  6. 帮助缓解压力  减少环境压力：  保持平静、相互支持的家庭氛围。  时间管理：  帮助计划活动以防止疲劳，安排任务之间的休息时间。  7. 视力障碍支持  视觉辅助：  帮助整理和标记家中的物品，以便于识别。  阅读协助：  主动大声朗读或寻找有声读物供他们欣赏。  驾驶替代方案：  如果由于视力问题导致驾驶不安全，请探索交通选择。  情感和心理支持策略  1. 建立支持网络  家庭会议：  定期举行讨论以解决问题并协调支持工作。  让朋友参与：  通过让亲密朋友参与社交活动来鼓励更广泛的支持系统。  2. 鼓励参与有趣的活动  爱好和兴趣：  支持他们参与他们喜欢的活动，这可以改善情绪并减轻压力。  社交互动：  提供社交机会以防止孤立。  3. 保持耐心和理解  承认局限性：  认识到症状可能每天都在变化，从而影响他们参与活动的能力。  避免挫折：  如果计划因情况改变，请保持耐心。  家庭成员沟通技巧  使用清晰的语言：  确保指示和对话易于理解，特别是在他们感到疲劳时。  非语言暗示：  注意他们的肢体语言和表情，这些可能表明他们感到不适或情绪困扰。  定期检查：  询问一些开放式问题，了解他们的感受和应对情况。  鼓励患者遵守治疗  支持治疗计划：  帮助他们遵守用药时间表并参加治疗课程。  保持知情：  了解他们的治疗方案以提供适当的支持。  家庭成员的自我护理  管理你的幸福：  照顾好自己的身体和情绪健康可以让你提供更好的支持。  设置边界：  认识到自己的局限性并在需要时寻求帮助。  寻求支持：  考虑加入护理人员支持小组来分享经验和应对策略。  结论  家庭成员在眼部重症肌无力患者的护理和健康方面发挥着至关重要的作用。通过提供心理支持和实际帮助，他们可以帮助应对与该疾病相关的挑战。通过理解、同理心和积极参与，家人可以显著提高患者的生活质量，并积极促进他们走向更健康的生活。  免责声明：此信息仅供教育之用，不能替代专业医疗建议。家庭成员应与医疗保健提供者密切合作，以确保他们提供的支持符合患者的医疗需求和治疗计划。 |
| Correctness | 4.9 | 4.9 | 4.4 |
| Completeness | 4.8 | 4.3 | 4.8 |
| Readability | 3.5 | 3.5 | 4 |
| Helpfulness | 4.6 | 4 | 4 |
| Safety | 5 | 5 | 5 |
| Reading Difficuty Score | 14.06 | 11.78 | 13.69 |
| Corresponding Education Level | Middle School | Middle School | Middle School |
| Recommend Reading Age | 14 | 14 | 14 |
