# Supplementary material for: Evaluating the Effectiveness of Large Language Models in Providing Patient Education for Chinese Patients With Ocular Myasthenia Gravis: Mixed Methods Study
Source: J Med Internet Res. 2025 Apr 10;27:e67883. doi: 10.2196/67883 (PMC12022522; doi:10.2196/67883)
Supplement: Multimedia Appendix 6 [file jmir_v27i1e67883_app6.docx]

| **Table S6: Detailed Information of Real-World Study** | | |
| --- | --- | --- |
| Group | **ChatGPT o1-preview** | |
| Sex | F | |
| Age | 42 | |
|  | Ocular Myasthenia Gravis Patient Education Brochure  Dear Patient:  Greetings! To help you better understand and manage oculomuscular myasthenia gravis, we have prepared the following information for you.  What is Ocular Myasthenia Gravis?  Ocular myasthenia gravis is an autoimmune disease that primarily affects the muscles that control eye and eyelid movements. This disease causes muscle weakness and common symptoms include droopy eyelids (ptosis) and double vision (double vision).  Symptoms  Ptosis: Drooping of the upper eyelid that may obscure part or all of the field of vision.  Diplopia: double vision when seeing objects, which may interfere with daily activities.  Ocular dyskinesia: inability to rotate the eyeballs properly, resulting in a restricted field of vision.  Analysis of the cause of the disease  The disease is caused by the immune system mistakenly attacking receptors at the neuromuscular junction, resulting in muscle weakness as nerve signals are not properly transmitted to the muscles.  Diagnosis  Physical Examination: Your doctor will evaluate your eye muscle function.  Antibody testing: Blood tests to detect specific autoantibodies.  Electrophysiologic tests: Such as repeated nerve stimulation tests to assess nerve conduction function.  Imaging tests: To rule out other possible causes, such as brain or orbital lesions.  Treatment Options  Medication:  Acetylcholinesterase inhibitors: e.g. pyridostigmine, to enhance neuromuscular conduction.  Immunosuppressants: e.g. corticosteroids, attenuate immune system overreaction.  Surgical treatment:  Thymectomy: For some patients, removal of the thymus may improve symptoms.  Other treatments:  Plasma exchange or immunoglobulin therapy: for severe or acute exacerbations.  Recommendations for daily management  Regular follow-ups: see your doctor on time to monitor changes in your condition.  Lifestyle modifications:  Maintain adequate rest and sleep.  Avoid excessive fatigue and stress.  Eat a balanced diet to enhance immunity.  Eye care:  Use eye shields or special glasses to correct diplopia.  Avoid prolonged eye use and take proper rest.  Prognosis and Support  Most patients can effectively control their symptoms and maintain a good quality of life through active treatment. It is recommended to join a patient support group to share experiences and get emotional support from others.  Contact Us  Please feel free to contact your ophthalmologist or medical team if you have any questions or need further assistance.  We wish you a speedy recovery!  Translated with DeepL.com (free version) | 眼肌型重症肌无力患者教育手册  亲爱的患者：  您好！为了帮助您更好地了解和管理眼肌型重症肌无力，我们为您准备了以下信息。  什么是眼肌型重症肌无力？  眼肌型重症肌无力是一种自身免疫性疾病，主要影响控制眼球和眼睑运动的肌肉。这种疾病会导致肌肉无力，常见症状包括眼睑下垂（上睑下垂）和复视（重影）。  症状表现  眼睑下垂：上眼皮下垂，可能遮挡部分或全部视野。  复视：视物时出现双重影像，可能影响日常活动。  眼球运动障碍：眼球无法正常转动，导致视野受限。  病因分析  该疾病是由于免疫系统错误地攻击了神经肌肉接头处的受体，导致神经信号无法正常传递给肌肉，造成肌肉无力。  诊断方法  体格检查：医生会评估您的眼部肌肉功能。  抗体检测：血液测试检测特定的自身抗体。  电生理测试：如重复神经刺激试验，评估神经传导功能。  影像学检查：排除其他可能的病因，如脑部或眼眶病变。  治疗方案  药物治疗：  乙酰胆碱酯酶抑制剂：如吡啶斯的明，增强神经肌肉传导。  免疫抑制剂：如皮质类固醇，减弱免疫系统的过度反应。  手术治疗：  胸腺切除术：对于某些患者，移除胸腺可能改善症状。  其他治疗：  血浆置换或免疫球蛋白疗法：用于重症或急性发作期。  日常管理建议  定期复诊：按时就医，监测病情变化。  生活方式调整：  保持充足的休息和睡眠。  避免过度疲劳和压力。  均衡饮食，增强免疫力。  眼部护理：  使用眼罩或特殊眼镜矫正复视。  避免长时间用眼，适当休息。  预后与支持  大多数患者通过积极治疗可以有效控制症状，维持良好的生活质量。建议加入患者支持团体，与他人分享经验和获得情感支持。  联系我们  如有任何疑问或需要进一步的帮助，请随时联系您的眼科医生或医疗团队。  祝您早日康复！ |
| Q1 | Will my symptoms get worse over time? | 我的症状是否会进一步加重？ |
|  | Ocular myasthenia gravis affects the muscles that control eye movements and eyelids, leading to symptoms like double vision and drooping eyelids. The progression of symptoms can vary widely among individuals.  In some cases, the condition remains limited to the eye muscles, and symptoms may stabilize or even improve with appropriate treatment. In other instances, it can progress to generalized myasthenia gravis, affecting other muscle groups in the body.  It's important to work closely with your healthcare provider to monitor your condition and adjust your treatment plan as needed. They can offer personalized advice based on your specific situation and help manage any changes in your symptoms over time. | 眼部重症肌无力会影响控制眼球运动和眼睑的肌肉，导致复视和眼睑下垂等症状。症状的进展因人而异。  在某些情况下，病情仅局限于眼部肌肉，经过适当的治疗，症状可能会稳定甚至改善。在其他情况下，病情可能会发展为全身性重症肌无力，影响身体的其他肌肉群。  与您的医疗保健提供者密切合作以监测您的病情并根据需要调整您的治疗计划非常重要。他们可以根据您的具体情况提供个性化建议，并帮助您管理症状随时间的变化。 |
| Q2 | Is this condition curable, or will I always have it? | 这种病能完全治愈吗？ |
|  | Ocular myasthenia gravis is a chronic autoimmune disorder affecting the muscles that control eye movements and eyelids. While there is currently no cure for this condition, treatments are available that can help manage symptoms effectively. Medications, such as acetylcholinesterase inhibitors and immunosuppressants, can improve muscle strength and reduce symptoms. Some individuals may experience periods of remission where symptoms decrease significantly, but ongoing management is usually necessary.  It's important to work closely with your healthcare provider to develop a treatment plan tailored to your needs. They can offer the most accurate information regarding your prognosis and help you navigate the management of your condition over time. | 眼部重症肌无力是一种慢性自身免疫性疾病，会影响控制眼球运动和眼睑的肌肉。虽然目前尚无治愈该病的方法，但可以通过治疗有效控制症状。乙酰胆碱酯酶抑制剂和免疫抑制剂等药物可以提高肌肉力量并减轻症状。有些人可能会经历缓解期，症状会显著减轻，但通常需要持续治疗。  与医疗保健提供者密切合作，制定符合您需求的治疗计划非常重要。他们可以提供有关您的预后的最准确信息，并帮助您长期管理您的病情。 |
| Q3 | Will ocular myasthenia gravis affect my vision permanently? | 眼肌型重症肌无力会影响视力吗？ |
|  | Ocular myasthenia gravis affects the muscles controlling eye movements and eyelids, leading to symptoms like double vision and drooping eyelids. These symptoms are due to muscle weakness, not damage to the eyes themselves. In most cases, the condition does not cause permanent vision loss or structural eye damage.  With appropriate treatment and management, many people experience significant improvement in their symptoms. However, symptoms can fluctuate over time, and some individuals may have persistent issues. It's important to work closely with your healthcare provider to monitor your condition and adjust your treatment plan as needed. They can offer personalized guidance on what to expect and how best to manage your symptoms. | 眼部重症肌无力会影响控制眼球运动和眼睑的肌肉，导致复视和眼睑下垂等症状。这些症状是由于肌肉无力引起的，而不是眼睛本身受损。在大多数情况下，这种疾病不会导致永久性视力丧失或眼部结构损伤。  通过适当的治疗和管理，许多人的症状会得到显著改善。然而，症状可能会随着时间的推移而波动，有些人可能会有持续的问题。与你的医疗保健提供者密切合作以监测你的病情并根据需要调整你的治疗计划是很重要的。他们可以提供个性化的指导，告诉你会发生什么以及如何最好地控制你的症状。 |
| Patient Satisfaction | 4.8 | |
| Patient Readability | 4.7 | |
| Correctness | 5 | |
| Completeness | 4.5 | |
| Readability | 4.6 | |
| Helpfulness | 4.5 | |
| Safety | 5 | |
| Group | **ChatGPT o1-preview** | |
| Sex | M | |
| Age | 42 | |
|  | Patient Education Guide: Understanding Ocular Myasthenia Gravis  Dear Patient,  We recognize that learning about a new medical condition can be overwhelming. This guide is designed to help you understand ocular myasthenia gravis and provide you with the information you need to manage your health effectively.  What is Ocular Myasthenia Gravis?  Ocular myasthenia gravis is a chronic autoimmune disorder that specifically affects the muscles controlling eye movements and eyelid function. It leads to muscle weakness, resulting in symptoms like drooping eyelids (ptosis) and double vision (diplopia). Unlike generalized myasthenia gravis, this form is limited to the ocular muscles.  Symptoms  Drooping Eyelids (Ptosis): One or both eyelids may droop, which can worsen throughout the day or with fatigue.  Double Vision (Diplopia): Seeing two images of a single object due to misalignment of the eyes.  Difficulty in Eye Movements: Challenges in moving the eyes in certain directions.  Causes  Ocular myasthenia gravis occurs when the immune system mistakenly attacks the neuromuscular junction—the connection between nerves and muscles. This disrupts the transmission of nerve signals to the muscles, leading to muscle weakness. The exact cause of this autoimmune response is not fully understood.  Diagnosis  Medical History and Physical Examination: Assessment of symptoms and eye muscle function.  Blood Tests: Detecting antibodies associated with myasthenia gravis.  Edrophonium Test: A short-acting drug is administered to see if muscle strength temporarily improves.  Electrodiagnostic Tests: Repetitive nerve stimulation to evaluate nerve-to-muscle signal transmission.  Imaging Studies: CT or MRI scans to examine the thymus gland and rule out other conditions.  Treatment Options  Medications  Acetylcholinesterase Inhibitors:  Example: Pyridostigmine (Mestinon)  Function: Enhances communication between nerves and muscles to improve muscle strength.  Immunosuppressants:  Examples: Prednisone, azathioprine  Function: Suppress the immune system's abnormal response.  Surgical Intervention  Thymectomy:  Description: Surgical removal of the thymus gland.  Indication: May be considered if a thymoma (tumor of the thymus) is present or to improve symptoms.  Other Therapies  Plasmapheresis:  Function: Removes harmful antibodies from the blood.  Usage: Typically used in severe cases or before surgery.  Intravenous Immunoglobulin (IVIG):  Function: Provides normal antibodies that alter the immune response.  Usage: For short-term relief of symptoms.  Managing Your Condition  Follow Your Treatment Plan  Take medications exactly as prescribed.  Attend all scheduled medical appointments.  Report any changes in symptoms to your healthcare provider promptly.  Lifestyle Adjustments  Rest and Sleep: Ensure adequate rest to help reduce muscle fatigue.  Stress Management: Practice relaxation techniques like deep breathing, meditation, or gentle yoga.  Balanced Diet: Maintain a nutritious diet to support overall health.  Eye Care Strategies  Eye Patching:  Cover one eye to alleviate double vision (alternate eyes periodically).  Prism Glasses:  Special lenses that help correct double vision.  Protective Eyewear:  Wear sunglasses to reduce glare and light sensitivity.  Artificial Tears:  Use lubricating eye drops to prevent dryness if eyelid closure is incomplete.  Frequently Asked Questions  Q: Will my symptoms get worse over time?  A: The progression of ocular myasthenia gravis varies among individuals. Some people may experience stable symptoms, while others might see progression to generalized myasthenia gravis. Regular follow-ups with your healthcare provider are essential to monitor your condition.  Q: Is this condition curable?  A: While there is currently no cure for ocular myasthenia gravis, treatments are available that effectively manage symptoms. Some patients may achieve remission or significant symptom improvement.  Q: Will it affect my vision permanently?  A: Ocular myasthenia gravis affects muscle function but does not typically cause permanent vision loss or damage to the eyes themselves. With proper management, symptoms can often be controlled.  Support and Resources  Educational Materials: Request brochures or reliable websites for more information.  Support Groups: Connecting with others facing similar challenges can provide emotional support.  Professional Counseling: Consider counseling if you're feeling anxious or depressed about your condition.  When to Seek Immediate Medical Attention  Sudden worsening of symptoms.  Difficulty breathing or swallowing (seek emergency care immediately).  New or unexplained symptoms. | 患者教育指南：了解眼部重症肌无力  亲爱的患者，  我们认识到，了解一种新的疾病可能会让人不知所措。本指南旨在帮助您了解眼部重症肌无力，并为您提供有效管理健康所需的信息。  什么是眼部重症肌无力？  眼部重症肌无力是一种慢性自身免疫性疾病，专门影响控制眼球运动和眼睑功能的肌肉。它会导致肌肉无力，从而导致眼睑下垂（眼睑下垂）和复视（复视）等症状。与全身性重症肌无力不同，这种形式仅限于眼部肌肉。  症状  眼睑下垂（眼睑下垂）：一只或两只眼睑可能下垂，并且可能在一天中或因疲劳而恶化。  复视（复视）：由于双眼错位而看到一个物体的两个图像。  眼球运动困难：向某个方向移动眼睛有困难。  原因  眼部重症肌无力是指免疫系统错误地攻击神经肌肉接头（神经和肌肉之间的连接）时发生的。这会破坏神经信号向肌肉的传输，导致肌肉无力。这种自身免疫反应的确切原因尚不完全清楚。  诊断  病史和体格检查：症状和眼肌功能的评估。  血液测试：检测与重症肌无力相关的抗体。  依酚氯铵试验：使用一种短效药物来观察肌肉力量是否暂时改善。  电诊断测试：重复神经刺激以评估神经至肌肉的信号传输。  影像学研究:ct或mri扫描检查胸腺并排除其他疾病。  治疗方案  药物  乙酰胆碱酯酶抑制剂：  例如:吡啶斯的明(Mestinon)  功能：增强神经和肌肉之间的沟通，提高肌肉力量。  免疫抑制剂：  例如：泼尼松、硫唑嘌呤  功能：抑制免疫系统的异常反应。  手术干预  胸腺切除术：  描述：手术切除胸腺。  适应症：如果存在胸腺瘤（胸腺肿瘤）或为了改善症状，可能需要考虑。  其他疗法  血浆置换：  功能：清除血液中的有害抗体。  用途：通常用于严重病例或手术前。  静脉注射免疫球蛋白(英文):  功能：提供改变免疫反应的正常抗体。  用途：用于短期缓解症状。  管理您的病情  遵循治疗计划  严格按照医嘱服药。  参加所有预定的医疗预约。  如有任何症状变化，请及时向您的医疗保健提供者报告。  生活方式的调整  休息和睡眠：确保充足的休息，有助于减轻肌肉疲劳。  压力管理：练习放松技巧，如深呼吸、冥想或轻柔瑜伽。  均衡饮食：保持营养饮食以支持整体健康。  眼部护理策略  眼部遮盖：  遮住一只眼睛以减轻复视（定期交替眼睛）。  棱镜眼镜：  有助于矫正复视的特殊镜片。  防护眼镜：  戴上太阳镜以减少眩光和光敏感度。  人工泪液：  如果眼睑闭合不完全，请使用润滑眼药水防止干燥。  常见问题  问：我的症状会随着时间的推移而恶化吗？  答：眼部重症肌无力的进展因人而异。有些人的症状可能比较稳定，而另一些人则可能发展为全身性重症肌无力。定期去看医生对于监测病情至关重要。  问： 这种病能治愈吗？  答：目前眼部重症肌无力尚无治愈方法，但有治疗方法可以有效控制症状。部分患者可能获得缓解或症状显著改善。  问：这会对我的视力造成永久性的影响吗？  答：眼部重症肌无力会影响肌肉功能，但通常不会导致永久性视力丧失或眼睛本身受损。通过适当的治疗，症状通常可以得到控制。  支持和资源  教育材料：索取小册子或可靠的网站以获取更多信息。  支持小组：与面临类似挑战的人联系可以提供情感支持。  专业咨询：如果您对自己的状况感到焦虑或沮丧，请考虑接受咨询。  何时应立即就医  症状突然恶化。  呼吸困难或吞咽困难（立即寻求紧急救治）。  新的或无法解释的症状。 |
| Q1 | How can I prevent relapses or flare-ups? | 有什么方法可以预防复发？ |
|  | Preventing relapses or flare-ups of ocular myasthenia gravis involves a combination of medical management and lifestyle adjustments. Here are some strategies that may help:  1. Adhere to Your Treatment Plan  Take Medications as Prescribed:  Consistency is Key: Ensure you take all medications exactly as directed by your healthcare provider to maintain stable muscle function.  Regular Medical Check-Ups:  Monitor Progress: Keep all scheduled appointments to assess your condition and adjust treatments if necessary.  2. Manage Stress Effectively  Stress Reduction Techniques:  Relaxation Practices: Engage in activities like deep breathing exercises, meditation, yoga, or tai chi.  Balanced Lifestyle:  Work-Life Balance: Avoid overexertion and allocate time for leisure and relaxation.  3. Ensure Adequate Rest  Sleep Hygiene:  Quality Sleep: Aim for 7-9 hours of uninterrupted sleep each night.  Rest Periods:  Take Breaks: Incorporate short rest periods during the day, especially when performing tasks that strain your eyes.  4. Avoid Known Triggers  Infections:  Prevent Illness: Practice good hygiene and stay updated on vaccinations as recommended by your doctor.  Medications:  Drug Interactions: Some medications can worsen symptoms. Always consult your healthcare provider before starting new medications.  Extreme Temperatures:  Climate Control: Avoid exposure to very hot or cold environments, as they can exacerbate symptoms.  5. Maintain a Healthy Lifestyle  Balanced Diet:  Nutrient-Rich Foods: Consume a diet rich in fruits, vegetables, whole grains, and lean proteins.  Regular Physical Activity:  Moderate Exercise: Engage in light to moderate activities like walking or swimming, after consulting your doctor.  Avoid Harmful Substances:  Limit Alcohol and Tobacco: These can negatively impact your overall health and immune system.  6. Practice Good Eye Care  Reduce Eye Strain:  Adjust Lighting: Ensure adequate lighting when reading or working.  Screen Time: Limit time spent on digital devices and take regular breaks.  Use Assistive Devices:  Prism Glasses or Eye Patches: These can help manage double vision.  Protect Your Eyes:  Wear Sunglasses: Shield your eyes from bright light and glare.  7. Stay Informed and Connected  Educate Yourself:  Knowledge Empowerment: Understand your condition to make informed decisions about your health.  Support Networks:  Community Support: Join support groups or online forums to share experiences and coping strategies.  8. Communicate with Your Healthcare Team  Report Changes Promptly:  Symptom Tracking: Keep a diary of your symptoms to discuss during medical visits.  Collaborative Care:  Ask Questions: Don't hesitate to seek clarification or express concerns to your healthcare providers.  Remember: Each person's experience with ocular myasthenia gravis is unique. While these strategies can help reduce the likelihood of relapses or flare-ups, it's important to work closely with your healthcare provider to develop a personalized plan that's right for you. | 预防眼部重症肌无力复发或发作需要结合医疗管理和生活方式调整。以下是一些可能有帮助的策略：  1. 遵守治疗计划  按医嘱服药：  一致性是关键：确保您严格按照医疗保健提供者的指示服用所有药物，以保持稳定的肌肉功能。  定期体检：  监测进展：遵守所有预约，评估您的状况并在必要时调整治疗。  2.有效管理压力  减轻压力的技巧：  放松练习：进行深呼吸练习、冥想、瑜伽或太极等活动。  平衡的生活方式：  工作与生活的平衡：避免过度劳累，分配时间用于休闲和放松。  3. 确保充足休息  睡眠卫生：  优质睡眠：每晚保证 7-9 小时不间断的睡眠。  休息时间：  休息：在一天中安排短暂的休息时间，特别是在执行使眼睛疲劳的任务时。  4. 避免已知的诱因  感染：  预防疾病：保持良好的卫生习惯并按照医生的建议及时接种疫苗。  药物：  药物相互作用：某些药物可能会加重症状。开始服用新药物前，请务必咨询您的医疗保健提供者。  极端温度：  气候控制：避免暴露在极热或极冷的环境中，因为它们会加剧症状。  5.保持健康的生活方式  均衡饮食：  营养丰富的食物：多吃富含水果、蔬菜、全谷物和瘦肉蛋白的饮食。  定期进行体育锻炼：  中度运动：咨询医生后，进行轻度到中度的活动，如散步或游泳。  避免有害物质：  限制酒精和烟草：这些可能会对您的整体健康和免疫系统产生负面影响。  6. 做好眼部护理  减轻眼睛疲劳：  调整照明：阅读或工作时确保充足的照明。  屏幕时间：限制使用数码设备的时间并定期休息。  使用辅助设备：  棱镜眼镜或眼罩：可以帮助控制复视。  保护你的眼睛：  戴太阳镜：保护眼睛免受强光和眩光的伤害。  7. 保持信息畅通和联系  自我教育：  知识赋权：了解您的状况，以便对您的健康做出明智的决定。  支持网络：  社区支持：加入支持小组或在线论坛，分享经验和应对策略。  8. 与您的医疗团队沟通  及时报告变化：  症状跟踪：记录您的症状日记，以便在就诊时讨论。  协作护理：  提出问题：不要犹豫，向您的医疗保健提供者寻求澄清或表达担忧。  请记住：每个人患眼部重症肌无力的经历都是独一无二的。虽然这些策略可以帮助降低复发或发作的可能性，但与您的医疗保健提供者密切合作制定适合您的个性化计划非常重要。 |
| Q2 | What are the side effects of the medications? Is long-term medication safe? | 药物治疗的副作用是什么？长期用药会有问题吗？ |
|  | Understanding Medication Side Effects and Long-Term Safety in Ocular Myasthenia Gravis  Managing ocular myasthenia gravis often involves medications that help improve muscle strength and reduce immune system activity. It's important to be aware of the potential side effects of these medications and understand the considerations regarding long-term use.  Common Medications and Their Side Effects  1. Acetylcholinesterase Inhibitors  Example: Pyridostigmine (Mestinon)  Function: Improves communication between nerves and muscles by preventing the breakdown of acetylcholine, a neurotransmitter.  Possible Side Effects:  Gastrointestinal Issues: Nausea, vomiting, diarrhea, abdominal cramps.  Increased Salivation and Sweating  Muscle Cramps or Twitching  Low Blood Pressure  Bradycardia (Slow Heart Rate)  Long-Term Use Considerations:  Generally well-tolerated for long-term use.  Side effects are often dose-related and may be managed by adjusting the dosage.  Regular monitoring is advisable to ensure optimal dosing.  2. Corticosteroids  Example: Prednisone  Function: Suppresses the immune system to reduce antibody production that interferes with nerve-muscle communication.  Possible Side Effects:  Weight Gain and Increased Appetite  Mood Changes: Anxiety, irritability, depression.  High Blood Pressure  Elevated Blood Sugar Levels: May lead to diabetes in some cases.  Osteoporosis (Bone Thinning)  Increased Risk of Infections  Cataracts and Glaucoma  Muscle Weakness (with prolonged use)  Long-Term Use Considerations:  Long-term use can lead to significant side effects; doctors aim to use the lowest effective dose.  May require additional medications to manage side effects (e.g., calcium and vitamin D supplements for bone health).  Regular check-ups are essential to monitor blood pressure, blood sugar, bone density, and eye health.  3. Immunosuppressants  Examples: Azathioprine (Imuran), Mycophenolate Mofetil (CellCept), Cyclosporine, Methotrexate  Function: Reduce the immune system's activity to decrease the production of harmful antibodies.  Possible Side Effects:  Increased Risk of Infections  Liver Toxicity: Elevated liver enzymes.  Kidney Dysfunction (especially with Cyclosporine)  Gastrointestinal Symptoms: Nausea, vomiting, diarrhea.  Blood Cell Count Changes: Anemia, leukopenia (low white blood cells), thrombocytopenia (low platelets).  Hair Loss  Increased Risk of Certain Cancers: With prolonged use.  Long-Term Use Considerations:  Requires regular blood tests to monitor liver and kidney function and blood cell counts.  Dose adjustments may be necessary based on test results and side effects.  Patients should promptly report any signs of infection or unusual symptoms.  4. Intravenous Immunoglobulin (IVIG) and Plasmapheresis  Function: Short-term treatments that temporarily modify the immune system.  Possible Side Effects of IVIG:  Headaches and Migraine  Flu-like Symptoms: Fever, chills, muscle aches.  Allergic Reactions: Rare but can include rash or anaphylaxis.  Blood Clots and Kidney Problems: Very rare.  Possible Side Effects of Plasmapheresis:  Low Blood Pressure  Dizziness or Fainting  Risk of Infection or Bleeding at the Insertion Site  Electrolyte Imbalances  Long-Term Use Considerations:  Typically used for acute symptom management rather than long-term therapy.  Side effects are generally manageable with proper medical supervision.  Is Long-Term Medication Safe?  Key Points:  Benefit vs. Risk: Long-term medication use is often necessary to control symptoms effectively. The benefits usually outweigh the risks when medications are used appropriately.  Monitoring: Regular follow-up appointments and monitoring tests are crucial to detect and manage potential side effects early.  Individualized Treatment: Dosages and medication choices are tailored to each patient's needs and health status.  Lifestyle Factors: Healthy habits can help mitigate some risks associated with long-term medication use (e.g., diet and exercise to support bone health while on corticosteroids).  Safety Measures:  Open Communication: Keep your healthcare provider informed about any new symptoms or concerns.  Adherence to Treatment Plan: Take medications exactly as prescribed to maintain optimal control of your condition.  Preventive Care: Stay up-to-date with vaccinations and routine health screenings.  Conclusion  While the medications used to treat ocular myasthenia gravis can have side effects, many people use them safely over the long term under medical supervision. Close collaboration with your healthcare team can help manage side effects and ensure that your treatment plan remains safe and effective. Regular monitoring and open communication are essential components of long-term medication safety.  Disclaimer: This information is intended to provide a general overview and should not replace professional medical advice. Please consult your healthcare provider for personalized guidance regarding your medications and health management. | 了解眼部重症肌无力的药物副作用和长期安全性  治疗眼部重症肌无力通常需要使用有助于提高肌肉力量和降低免疫系统活性的药物。重要的是要意识到这些药物的潜在副作用，并了解长期使用的注意事项。  常见药物及其副作用  1.乙酰胆碱酯酶抑制剂  例如:吡啶斯的明(Mestinon)  功能：通过防止神经递质乙酰胆碱的分解来改善神经和肌肉之间的交流。  可能的副作用：  胃肠道问题：恶心、呕吐、腹泻、腹部绞痛。  唾液分泌和出汗增多  肌肉痉挛或抽搐  低血压  心动过缓（心率缓慢）  长期使用注意事项：  一般而言，长期使用耐受性良好。  副作用通常与剂量有关，可以通过调整剂量来控制。  建议定期监测以确保最佳剂量。  2.皮质类固醇  例如：泼尼松  功能：抑制免疫系统，减少干扰神经肌肉通讯的抗体产生。  可能的副作用：  体重增加和食欲增加  情绪变化：焦虑、易怒、抑郁。  高血压  血糖升高：在某些情况下可能导致糖尿病。  骨质疏松症（骨质疏松）  感染风险增加  白内障和青光眼  肌肉无力（长期使用）  长期使用注意事项：  长期使用会导致严重的副作用；医生尽量使用最低有效剂量。  可能需要额外的药物来控制副作用(例如，用于骨骼健康的钙和维生素d补充剂)。  定期检查对于监测血压、血糖、骨密度和眼睛健康至关重要。  3.免疫抑制剂  例如:硫唑嘌呤(Imuran)、霉酚酸酯(CellCept)、环孢菌素、甲氨蝶呤  功能：降低免疫系统的活性，减少有害抗体的产生。  可能的副作用：  感染风险增加  肝毒性：肝酶升高。  肾功能障碍（尤其是使用环孢菌素时）  胃肠道症状：恶心、呕吐、腹泻。  血细胞计数变化：贫血、白细胞减少症（白细胞低）、血小板减少症（血小板低）。  脱发  某些癌症的风险增加：长期使用。  长期使用注意事项：  需要定期进行血液检查以监测肝肾功能和血细胞计数。  根据测试结果和副作用可能需要调整剂量。  患者应及时报告任何感染迹象或异常症状。  4. 静脉注射免疫球蛋白(ivig)和血浆置换  功能：暂时改变免疫系统的短期治疗。  Ivig的可能副作用:  头痛和偏头痛  流感样症状：发烧，发冷，肌肉疼痛。  过敏反应：罕见，但可能出现皮疹或过敏反应。  血栓和肾脏问题：非常罕见。  血浆置换的可能副作用：  低血压  头晕或昏厥  插入部位感染或出血的风险  电解质失衡  长期使用注意事项：  通常用于急性症状管理而不是长期治疗。  在适当的医疗监督下，副作用通常可以控制。  长期服药安全吗？  要点：  益处与风险：长期用药往往是有效控制症状的必要条件。如果用药得当，益处通常大于风险。  监测：定期的随访预约和监测测试对于及早发现和处理潜在的副作用至关重要。  个性化治疗：根据每个患者的需求和健康状况定制剂量和药物选择。  生活方式因素：健康的习惯有助于减轻长期用药带来的一些风险（例如，服用皮质类固醇期间，通过饮食和运动来维持骨骼健康）。  安全措施：  开放的沟通：让您的医疗保健提供者了解任何新的症状或疑虑。  遵守治疗计划：严格按照处方服药，以保持对病情的最佳控制。  预防保健：及时接种疫苗并进行常规健康检查。  结论  虽然用于治疗眼部重症肌无力的药物可能会产生副作用，但许多人在医生监督下长期使用这些药物都是安全的。与医疗团队密切合作可以帮助控制副作用并确保您的治疗计划保持安全有效。定期监测和公开交流是长期用药安全的重要组成部分。  免责声明：此信息仅供参考，不能替代专业医疗建议。请咨询您的医疗保健提供者，获取有关您的药物和健康管理的个性化指导。 |
| Q3 | Will lifestyle or dietary changes help manage my condition? | 生活方式或饮食习惯会影响病情吗？ |
|  | Yes, lifestyle and dietary changes can support the management of ocular myasthenia gravis and may help reduce symptoms or prevent flare-ups. While these changes are not a substitute for medical treatment, they can complement your overall care plan. Here are some recommendations:  Lifestyle Changes  1. Manage Stress  Stress Reduction Techniques:  Relaxation Methods: Practice deep breathing exercises, meditation, yoga, or tai chi to help reduce stress, which can exacerbate symptoms.  Mindfulness: Engage in mindfulness practices to stay present and reduce anxiety related to your condition.  Adequate Rest:  Sleep Hygiene: Aim for 7-9 hours of quality sleep each night.  Rest Periods: Take short breaks during the day to prevent muscle fatigue, especially during activities that require eye strain.  2. Moderate Physical Activity  Regular Exercise:  Low-Impact Activities: Engage in gentle exercises like walking, swimming, or stationary cycling.  Energy Conservation: Balance activity with rest periods to avoid overexertion.  Physical Therapy:  Customized Programs: A physical therapist can design an exercise plan tailored to your needs and limitations.  3. Eye Care Strategies  Reduce Eye Strain:  Limit Screen Time: Take frequent breaks when using computers, smartphones, or watching TV.  Adjust Lighting: Ensure adequate and comfortable lighting to reduce squinting and eye fatigue.  Use Assistive Devices:  Prism Glasses or Eye Patches: These can help manage double vision.  Protective Eyewear:  Sunglasses: Wear sunglasses to shield your eyes from bright light and glare.  4. Avoid Known Triggers  Temperature Extremes:  Climate Control: Avoid exposure to very hot or cold environments, as they may worsen symptoms.  Infections:  Prevent Illness: Practice good hygiene and avoid close contact with people who are sick.  Medication Awareness:  Drug Interactions: Consult your doctor before starting new medications, including over-the-counter drugs and supplements.  Dietary Changes  1. Balanced Nutrition  Whole Foods Diet:  Fruits and Vegetables: Consume a variety of colorful fruits and vegetables rich in antioxidants.  Lean Proteins: Include sources like fish, poultry, beans, and legumes.  Whole Grains: Choose whole-grain bread, rice, and pasta over refined grains.  Healthy Fats:  Omega-3 Fatty Acids: Found in fatty fish like salmon and flaxseeds, they may help reduce inflammation.  2. Maintain Healthy Weight  Portion Control:  Caloric Balance: Ensure you're consuming the right amount of calories to maintain a healthy weight.  Regular Meals:  Steady Energy Levels: Eat regular, balanced meals to prevent fatigue.  3. Hydration  Adequate Fluid Intake:  Water Consumption: Aim for at least 8 glasses of water daily unless otherwise advised by your doctor.  Limit Caffeinated Beverages:  Moderation: Excess caffeine can interfere with sleep and may increase anxiety.  4. Limit Potentially Harmful Substances  Alcohol:  Moderation: Alcohol can interact with medications and worsen muscle weakness.  Avoid Smoking:  Quit Tobacco: Smoking can impair circulation and overall health, potentially exacerbating symptoms.  5. Monitor Nutrient Intake  Calcium and Vitamin D:  Bone Health: Especially important if you're taking corticosteroids, which can affect bone density.  Folate and Vitamin B12:  Nervous System Support: Ensure adequate intake to support nerve health.  Additional Recommendations  1. Consult Professionals  Dietitian Services:  Personalized Plan: A registered dietitian can help tailor a diet specific to your needs and medical conditions.  Regular Medical Follow-Up:  Monitor Health: Keep up with appointments to adjust treatments as necessary.  2. Stay Informed  Education:  Reliable Sources: Stay updated on new research and recommendations regarding myasthenia gravis.  3. Community Support  Support Groups:  Shared Experiences: Connecting with others can provide emotional support and practical tips.  Remember, while lifestyle and dietary adjustments can aid in managing your condition, they should complement your medical treatment plan, not replace it. Always discuss any significant changes with your healthcare provider to ensure they're appropriate for your specific situation. Working collaboratively with your medical team will help you manage ocular myasthenia gravis more effectively and improve your overall quality of life. | 是的，生活方式和饮食习惯的改变有助于控制眼部重症肌无力，并可能有助于减轻症状或防止病情发作。虽然这些改变不能代替医疗，但它们可以补充您的整体护理计划。以下是一些建议：  生活方式的改变  1. 管理压力  减轻压力的技巧：  放松方法：进行深呼吸练习、冥想、瑜伽或太极拳有助于减轻压力，压力可能会加剧症状。  正念：进行正念练习以保持现状并减少与您的状况相关的焦虑。  充足休息：  睡眠卫生：每晚保证 7-9 小时的优质睡眠。  休息时间：白天短暂休息以防止肌肉疲劳，特别是在需要眼睛疲劳的活动中。  2. 适度的体力活动  定期锻炼：  低强度活动：进行轻柔的运动，例如散步、游泳或骑固定自行车。  节约能源：平衡活动与休息时间，避免过度劳累。  物理治疗：  定制计划：物理治疗师可以根据您的需求和限制设计锻炼计划。  3. 眼部护理策略  减轻眼睛疲劳：  限制屏幕时间：使用电脑、智能手机或看电视时要经常休息。  调整照明：确保充足舒适的照明，以减少斜视和眼睛疲劳。  使用辅助设备：  棱镜眼镜或眼罩：可以帮助控制复视。  防护眼镜：  太阳镜：戴上太阳镜可以保护眼睛免受强光和眩光的伤害。  4. 避免已知的诱因  极端温度：  气候控制：避免暴露在极热或极冷的环境中，因为它们可能会加重症状。  感染：  预防疾病：保持良好的卫生习惯，避免与病人密切接触。  药物意识：  药物相互作用：开始服用新药物（包括非处方药和补充剂）之前，请咨询医生。  饮食变化  1.均衡营养  全食饮食：  水果和蔬菜：吃各种富含抗氧化剂的彩色水果和蔬菜。  瘦肉蛋白质：包括鱼、家禽、豆类和豆科植物等来源。  全谷物：选择全麦面包、米饭和面食，而不是精制谷物。  健康脂肪：  Omega-3脂肪酸:存在于鲑鱼和亚麻籽等脂肪鱼中，可能有助于减轻炎症。  2.保持健康体重  份量控制：  热量平衡：确保摄入适量的热量以维持健康的体重。  常规餐食：  稳定的能量水平：规律饮食，均衡饮食，以防止疲劳。  3.补水  足够的液体摄入量：  饮水量：除非医生另有建议，否则每天至少喝 8 杯水。  限制含咖啡因的饮料：  适量：过量咖啡因会影响睡眠，并可能增加焦虑。  4. 限制潜在有害物质  酒精：  适量：酒精会与药物相互作用，并加剧肌肉无力。  避免吸烟：  戒烟：吸烟会损害血液循环和整体健康，并可能加剧症状。  5. 监测营养摄入量  钙和维生素d:  骨骼健康：如果您正在服用皮质类固醇，这一点尤其重要，因为它会影响骨密度。  叶酸和维生素b12:  神经系统支持：确保足够的摄入量以支持神经健康。  其他建议  1. 咨询专业人士  营养师服务：  个性化计划：注册营养师可以帮助您根据您的需求和医疗状况定制饮食。  定期医疗随访：  监测健康状况：按时预约，以便根据需要调整治疗。  2. 随时了解最新信息  教育：  可靠来源：了解有关重症肌无力的新研究和建议。  3.社区支持  支持团体：  分享经历：与他人联系可以提供情感支持和实用建议。  请记住，虽然生活方式和饮食调整有助于控制病情，但它们应该作为医疗计划的补充，而不是替代计划。务必与医疗保健提供者讨论任何重大变化，以确保这些变化适合您的具体情况。与医疗团队合作将帮助您更有效地管理眼部重症肌无力并改善您的整体生活质量。 |
| Patient Satisfaction | 4.6 | |
| Patient Readability | 3.9 | |
| Correctness | 4.6 | |
| Completeness | 5 | |
| Readability | 4 | |
| Helpfulness | 4.7 | |  |
| Safety | 4.9 | |  |
| Group | **ChatGPT o1-preview** | |
| Sex | M | |
| Age | 20 | |
|  | Patient Education Guide: Understanding Ocular Myasthenia Gravis  Dear Patient,  We understand that being diagnosed with a new medical condition can be challenging. This guide aims to provide you with comprehensive information about ocular myasthenia gravis to help you understand and manage your condition effectively.  What is Ocular Myasthenia Gravis?  Ocular myasthenia gravis is a chronic autoimmune disorder that specifically affects the muscles controlling eye movements and eyelid function. It causes muscle weakness, leading to symptoms such as drooping eyelids (ptosis) and double vision (diplopia). Unlike generalized myasthenia gravis, this form is limited to the eye muscles.  Symptoms  Drooping Eyelids (Ptosis): One or both eyelids may sag, which can worsen throughout the day or with fatigue.  Double Vision (Diplopia): Seeing two images of a single object due to misalignment of the eyes.  Difficulty in Eye Movements: Challenges in moving the eyes in certain directions, leading to limited eye mobility.  Eye Fatigue: Increased tiredness in the eyes, especially after prolonged use.  Causes  Ocular myasthenia gravis occurs when the immune system mistakenly attacks the neuromuscular junction—the connection between nerves and muscles responsible for muscle contraction. This disrupts the transmission of nerve signals to the muscles, leading to muscle weakness. The exact cause of this autoimmune response is not fully understood, but factors may include genetics and environmental triggers.  Diagnosis  Medical History and Physical Examination: Assessment of symptoms and evaluation of eye muscle function.  Blood Tests: Detecting antibodies associated with myasthenia gravis, such as anti-acetylcholine receptor (AChR) antibodies.  Edrophonium Test (Tensilon Test): A short-acting drug is administered to see if muscle strength temporarily improves.  Electrodiagnostic Tests: Repetitive nerve stimulation or single-fiber electromyography to evaluate nerve-to-muscle signal transmission.  Imaging Studies: CT or MRI scans to examine the thymus gland and rule out other conditions.  Treatment Options  1. Medications  Acetylcholinesterase Inhibitors:  Example: Pyridostigmine (Mestinon)  Function: Enhances communication between nerves and muscles to improve muscle strength.  Usage: Taken orally multiple times a day as prescribed.  Immunosuppressants:  Examples: Prednisone, Azathioprine, Mycophenolate Mofetil  Function: Suppress the immune system's abnormal response.  Usage: Dosage and type depend on severity and patient response.  2. Surgical Intervention  Thymectomy:  Description: Surgical removal of the thymus gland.  Indication: May be considered if a thymoma (tumor of the thymus) is present or to improve symptoms even without a tumor.  3. Other Therapies  Plasmapheresis:  Function: Removes harmful antibodies from the blood.  Usage: Typically used in severe cases or before surgery.  Intravenous Immunoglobulin (IVIG):  Function: Provides normal antibodies that alter the immune system's response.  Usage: For short-term relief of symptoms.  Managing Your Condition  Follow Your Treatment Plan  Medication Adherence: Take medications exactly as prescribed to maintain muscle strength.  Regular Medical Appointments: Keep all scheduled visits to monitor progress and adjust treatments.  Report Changes: Inform your doctor of any new or worsening symptoms promptly.  Lifestyle Adjustments  Rest and Sleep:  Adequate Rest: Ensure you get sufficient sleep to reduce fatigue.  Rest Periods: Take short breaks during activities that strain your eyes.  Stress Management:  Relaxation Techniques: Practice deep breathing, meditation, or gentle yoga.  Balanced Schedule: Avoid overexertion and allow time for leisure activities.  Eye Care Strategies  Reduce Eye Strain:  Limit Screen Time: Take frequent breaks when using digital devices.  Proper Lighting: Ensure good lighting to prevent squinting.  Use Assistive Devices:  Prism Glasses: Help correct double vision.  Eye Patches: Cover one eye to alleviate double vision (alternate eyes periodically).  Protective Eyewear:  Sunglasses: Wear sunglasses outdoors to reduce glare and light sensitivity.  Frequently Asked Questions  Q: Will my symptoms get worse over time?  A: The progression varies among individuals. Some people experience stable symptoms limited to the eyes, while others may develop generalized myasthenia gravis affecting other muscles. Regular monitoring and treatment can help manage symptoms effectively.  Q: Is this condition curable, or will I always have it?  A: While there is currently no cure for ocular myasthenia gravis, treatments can effectively manage symptoms. Some patients may experience periods of remission or significant improvement.  Q: Will it affect my vision permanently?  A: Ocular myasthenia gravis affects muscle function but typically does not cause permanent vision loss or damage to the eye structures. With proper management, symptoms can often be controlled.  Q: How can I prevent relapses or flare-ups?  A: Adhering to your treatment plan, managing stress, getting adequate rest, avoiding known triggers, and maintaining a healthy lifestyle can help reduce the likelihood of relapses.  Q: What are the side effects of the medications? Is long-term medication safe?  A: Medications can have side effects, such as gastrointestinal issues with acetylcholinesterase inhibitors or increased infection risk with immunosuppressants. Regular monitoring and communication with your healthcare provider can help manage side effects. Long-term use is generally considered safe under medical supervision.  Q: Will lifestyle or dietary changes help manage my condition?  A: Yes, lifestyle and dietary changes can support your treatment. Managing stress, getting adequate rest, engaging in moderate exercise, and maintaining a balanced diet can complement medical therapies.  Support and Resources  Educational Materials: Request brochures or visit reputable websites for more information.  Support Groups: Connecting with others can provide emotional support and practical advice.  Professional Counseling: Seek counseling if you're experiencing anxiety or depression related to your condition.  When to Seek Immediate Medical Attention  Sudden Worsening of Symptoms: Significant increase in muscle weakness.  Difficulty Breathing or Swallowing: These could indicate a medical emergency.  Signs of Infection: Fever, sore throat, or other signs, especially if you're on immunosuppressants. | 患者教育指南：了解眼部重症肌无力  亲爱的患者，  我们理解，被诊断出患有一种新疾病是一件非常困难的事情。本指南旨在为您提供有关眼部重症肌无力的全面信息，帮助您了解和有效管理您的病情。  什么是眼部重症肌无力？  眼部重症肌无力是一种慢性自身免疫性疾病，专门影响控制眼球运动和眼睑功能的肌肉。它会导致肌肉无力，从而导致眼睑下垂（眼睑下垂）和复视（复视）等症状。与全身性重症肌无力不同，这种重症肌无力仅限于眼部肌肉。  症状  眼睑下垂（眼睑下垂）：一只或两只眼睑可能下垂，并且这种情况可能在一天之中或因疲劳而恶化。  复视（复视）：由于双眼错位而看到一个物体的两个图像。  眼球运动困难：眼球在某些方向上移动时遇到困难，导致眼球活动受限。  眼睛疲劳：眼睛疲劳加剧，尤其是长时间使用后。  原因  眼部重症肌无力是指免疫系统错误地攻击神经肌肉接头（负责肌肉收缩的神经和肌肉之间的连接）时发生的。这会破坏神经信号向肌肉的传输，导致肌肉无力。这种自身免疫反应的确切原因尚不完全清楚，但因素可能包括遗传和环境触发因素。  诊断  病史和体格检查：症状评估和眼肌功能评估。  血液检查:检测与重症肌无力相关的抗体，例如抗乙酰胆碱受体(AChR)抗体。  依酚氯铵试验(Tensilon试验):使用一种短效药物来观察肌肉力量是否暂时改善。  电诊断测试：重复神经刺激或单纤维肌电图以评估神经到肌肉的信号传输。  影像学研究:ct或mri扫描检查胸腺并排除其他疾病。  治疗方案  1. 药物  乙酰胆碱酯酶抑制剂：  例如:吡啶斯的明(Mestinon)  功能：增强神经和肌肉之间的沟通，提高肌肉力量。  用法：按规定每天口服多次。  免疫抑制剂：  例如：泼尼松、硫唑嘌呤、霉酚酸酯  功能：抑制免疫系统的异常反应。  用法：剂量和类型取决于严重程度和患者的反应。  2. 手术干预  胸腺切除术：  描述：手术切除胸腺。  适应症：如果存在胸腺瘤（胸腺肿瘤），或即使没有肿瘤也需要改善症状，则可能需要考虑。  3. 其他疗法  血浆置换：  功能：清除血液中的有害抗体。  用途：通常用于严重病例或手术前。  静脉注射免疫球蛋白(英文):  功能：提供改变免疫系统反应的正常抗体。  用途：用于短期缓解症状。  管理您的病情  遵循治疗计划  药物依从性：严格按照处方服药以保持肌肉力量。  定期就医：遵守所有预约就诊安排，以监测病情进展并调整治疗方案。  报告变化：及时告知医生任何新的或恶化的症状。  生活方式的调整  休息和睡眠：  充足休息：保证充足的睡眠以减少疲劳。  休息时间：在使眼睛疲劳的活动中短暂休息一下。  压力管理：  放松技巧：练习深呼吸，冥想，或柔和的瑜伽。  平衡时间表：避免过度劳累，留出时间进行休闲活动。  眼部护理策略  减轻眼睛疲劳：  限制屏幕时间：使用数码设备时要经常休息。  适当的照明：确保良好的照明以防止眯眼。  使用辅助设备：  棱镜眼镜：有助于矫正复视。  眼罩：遮住一只眼睛可缓解复视（定期交替眼睛）。  防护眼镜：  太阳镜：在户外佩戴太阳镜可以减少眩光和光敏感度。  常见问题  问：我的症状会随着时间的推移而恶化吗？  答：病情发展因人而异。有些人的症状稳定，仅限于眼睛，而另一些人则可能发展为全身性重症肌无力，影响其他肌肉。定期监测和治疗有助于有效控制症状。  问：这种病能治愈吗？还是我会一直患有这种病？  答：虽然目前眼部重症肌无力尚无治愈方法，但治疗可以有效控制症状。部分患者可能会经历一段时间的缓解或显著改善。  问：这会对我的视力造成永久性的影响吗？  答：眼部重症肌无力会影响肌肉功能，但通常不会导致永久性视力丧失或眼部结构损伤。通过适当的治疗，症状通常可以得到控制。  问：如何预防复发或发作？  答：遵守治疗计划、控制压力、充分休息、避免已知诱因和保持健康的生活方式可以帮助减少复发的可能性。  问：药物的副作用有哪些？长期服药安全吗？  答：药物可能会产生副作用，例如乙酰胆碱酯酶抑制剂会引起胃肠道问题，免疫抑制剂会增加感染风险。定期监测并与医疗保健提供者沟通有助于控制副作用。在医生监督下，长期使用通常被认为是安全的。  问：改变生活方式或饮食习惯有助于控制我的病情吗？  答：是的，生活方式和饮食习惯的改变可以支持您的治疗。控制压力、获得充分的休息、进行适度运动和保持均衡饮食可以补充医学疗法。  支持和资源  教育材料：索取小册子或访问信誉良好的网站以获取更多信息。  支持小组：与他人联系可以提供情感支持和实用建议。  专业咨询：如果您因自己的状况而感到焦虑或抑郁，请寻求咨询。  何时应立即就医  症状突然恶化：肌肉无力明显增加。  呼吸困难或吞咽困难：这可能表明有医疗紧急情况。  感染迹象：发烧、喉咙痛或其他迹象，尤其是在服用免疫抑制剂时。 |
| Q1 | Can I continue working and living normally? What precautions should I take? | 我可以正常工作和生活吗？需要注意什么？ |
|  | Yes, many people with ocular myasthenia gravis are able to continue working and maintain a normal lifestyle. However, certain precautions can help you manage your symptoms more effectively and reduce the impact on your daily activities.  Continuing Work and Daily Activities  1. Open Communication at Work  Inform Your Employer (If Comfortable):  Discuss Your Condition: If you fee  Reasonable Adjustments:  Flexible Scheduling: Arrange for flexible  Modify Tasks: Request adjust  2. Manage Fatigue  Energy Conservation:  Pace Yourself: Break  Prioritize Activities: Fo  3. Adapt Your Environment  Optimize Lighting:  Reduce Glare: Use  Ergonomic Setup:  Comfortable Workstation: Ensure your workspace is set up to reduce physical strain, especially on your eyes and neck.  Precautions to Take  1. Eye Care  Reduce Eye Strain:  Regular Breaks: Follow the 20-20-20 rule—every 20 minutes, look at something 20 feet away for 20 seconds.  Use Assistive Devices:  Prism Glasses or Eye Patch: These can help manage double vision during work or daily activities.  Protective Eyewear:  Sunglasses: Wear sunglasses outdoors to reduce light sensitivity.  2. Health Management  Medication Adherence:  Follow Prescriptions: Take your medications exactly as prescribed to maintain symptom control.  Regular Medical Check-Ups:  Monitor Progress: Keep up with appointments to adjust treatments if necessary.  3. Lifestyle Adjustments  Stress Management:  Relaxation Techniques: Practice meditation, deep breathing exercises, or yoga to reduce stress, which can exacerbate symptoms.  Adequate Rest:  Sleep Hygiene: Aim for 7-9 hours of quality sleep each night.  Balanced Diet and Hydration:  Nutrient-Rich Foods: Maintain a healthy diet to support overall well-being.  Stay Hydrated: Drink sufficient water throughout the day.  4. Avoid Known Triggers  Temperature Extremes:  Climate Control: Avoid very hot or cold environments that may worsen symptoms.  Illness Prevention:  Hygiene Practices: Wash hands regularly and avoid close contact with sick individuals to prevent infections.  5. Physical Activity  Moderate Exercise:  Low-Impact Activities: Engage in gentle exercises like walking or swimming after consulting your doctor.  Listen to Your Body:  Avoid Overexertion: Stop activities before reaching the point of significant fatigue.  Additional Tips  1. Plan Ahead  Schedule Wisely:  Optimal Timing: Plan demanding tasks for times when you typically feel most energetic.  Assistive Technology:  Use Tools: Voice-to-text software or screen readers can reduce the need for prolonged reading or typing.  2. Support System  Connect with Others:  Support Groups: Join groups for people with myasthenia gravis to share experiences and coping strategies.  Family and Friends:  Communicate Needs: Let your loved ones know how they can support you.  3. Safety Considerations  Driving:  Assess Vision: Be cautious if experiencing double vision or drooping eyelids; consider alternative transportation if necessary.  Workplace Safety:  Avoid Hazardous Tasks: If your job involves operating machinery or working at heights, take extra precautions.  Consult Your Healthcare Provider  It's important to discuss your specific situation with your healthcare provider. They can offer personalized advice based on the severity of your symptoms and your job requirements. They may also provide documentation if workplace accommodations are needed.  Remember, with proper management and support, many individuals with ocular myasthenia gravis lead full and active lives. Taking proactive steps can help you continue your normal activities while effectively managing your condition. | 是的，许多患有眼部重症肌无力的人能够继续工作并维持正常生活方式。但是，某些预防措施可以帮助您更有效地控制症状并减少对日常活动的影响。  继续工作和日常活动  1. 工作中开放沟通  通知您的雇主（如果愿意的话）：  讨论你的病情：如果你感觉  合理调整：  灵活安排：灵活安排  修改任务：请求调整  2. 控制疲劳  节能：  调整自己的节奏：休息  确定活动优先次序：  3. 调整你的环境  优化照明：  减少眩光：使用  人体工程学设置：  舒适的工作站：确保您的工作空间设置得当，可以减轻身体压力，尤其是眼睛和颈部的压力。  注意事项  1. 眼部护理  减轻眼睛疲劳：  定期休息：遵循 20-20-20 规则 - 每 20 分钟，看 20 英尺外的某个物体 20 秒。  使用辅助设备：  棱镜眼镜或眼罩：可以帮助控制工作或日常活动中的复视。  防护眼镜：  太阳镜：在户外佩戴太阳镜可以减少光敏感度。  2. 健康管理  服药依从性：  遵照处方：严格按照处方服用药物以控制症状。  定期体检：  监测进展：如有必要，按时预约以调整治疗。  3.生活方式的调整  压力管理：  放松技巧：练习冥想、深呼吸练习或瑜伽来减轻压力，因为压力可能会加剧症状。  充足休息：  睡眠卫生：每晚保证 7-9 小时的优质睡眠。  均衡饮食和补充水分：  营养丰富的食物：保持健康的饮食以支持整体健康。  保持水分：全天喝足够的水。  4. 避免已知的诱因  极端温度：  气候控制：避免过热或过冷的环境，因为这可能会加重症状。  疾病预防：  卫生习惯：经常洗手并避免与病人密切接触以防止感染。  5. 身体活动  适度运动：  低强度活动：咨询医生后，进行散步或游泳等轻柔运动。  倾听你的身体：  避免过度劳累：在达到严重疲劳的程度之前停止活动。  其他提示  1.提前计划  合理安排时间：  最佳时间：在您通常感觉精力最充沛的时候规划艰巨的任务。  辅助技术：  使用工具：语音转文本软件或屏幕阅读器可以减少长时间阅读或打字的需要。  2. 支持系统  与他人联系：  支持小组：加入重症肌无力患者小组，分享经验和应对策略。  家人和朋友：  传达需求：让您所爱的人知道他们可以如何支持您。  3. 安全考虑  驾驶：  评估视力：如果出现复视或眼睑下垂，请小心；必要时考虑其他交通方式。  工作场所安全：  避免危险任务：如果您的工作涉及操作机器或高空作业，请格外小心。  咨询您的医疗保健提供者  与您的医疗保健提供者讨论您的具体情况非常重要。他们可以根据您的症状严重程度和工作要求提供个性化建议。如果需要工作场所便利，他们也可能提供文件。  请记住，通过适当的管理和支持，许多眼肌型重症肌无力患者可以过上充实而积极的生活。采取积极措施可以帮助您继续正常活动，同时有效控制病情。 |
| Q2 | Is there a risk of my ocular myasthenia gravis progressing to generalized myasthenia gravis? | 眼肌型重症肌无力会发展成全身型吗？ |
|  | Yes, there is a risk that ocular myasthenia gravis can progress to generalized myasthenia gravis.  Understanding the Risk  Progression Statistics:  Studies indicate that approximately 50% to 80% of individuals diagnosed with ocular myasthenia gravis may develop generalized myasthenia gravis within the first two to three years after symptom onset.  The risk of progression is highest during the initial two years following diagnosis.  Factors Influencing Progression:  Age at Onset:  Younger patients, especially those under 50, may have a higher risk of progression.  Antibody Status:  The presence of certain antibodies, such as anti-acetylcholine receptor (AChR) antibodies, may increase the likelihood of generalization.  Thymus Abnormalities:  Enlargement or tumors of the thymus gland (thymoma) are associated with a higher risk.  Gender:  Some studies suggest that males may have a slightly higher risk of progression compared to females, but findings are not conclusive.  Monitoring and Management  Regular Medical Follow-Up:  Neurological Assessments: Regular check-ups with your neurologist or ophthalmologist can help detect early signs of generalization.  Symptom Tracking: Keep a detailed record of any new symptoms or changes in existing ones.  Early Intervention:  Prompt Reporting: Notify your healthcare provider immediately if you experience symptoms like generalized muscle weakness, difficulty swallowing, or breathing problems.  Treatment Adjustments: Early detection may allow for timely adjustments in your treatment plan to manage symptoms effectively.  What You Can Do  Stay Informed:  Educate Yourself: Understanding your condition can help you recognize potential signs of progression early.  Ask Questions: Don't hesitate to ask your healthcare provider for clarification or additional information.  Maintain a Healthy Lifestyle:  Stress Management: Practice relaxation techniques such as deep breathing exercises, meditation, or yoga.  Adequate Rest: Ensure you get sufficient sleep to reduce fatigue.  Balanced Diet: Consume a nutritious diet to support overall health.  Avoid Potential Triggers:  Medications: Some drugs can exacerbate myasthenia gravis symptoms. Always consult your doctor before starting new medications.  Infections: Prevent infections through good hygiene practices, as illnesses can trigger symptom worsening.  Consult Your Healthcare Provider  While statistical data can provide general insights, individual experiences may vary. It's important to discuss your specific situation with your healthcare provider, who can offer personalized guidance based on:  Your Medical History  Current Symptoms  Diagnostic Test Results  They can also provide recommendations on:  Frequency of Monitoring  Preventive Strategies  Potential Treatment Options  Remember: Early detection and proactive management are key to addressing any progression of the condition. Regular communication with your healthcare team will help ensure the best possible outcomes. | 是的，眼部重症肌无力有发展为全身性重症肌无力的风险。  了解风险  进度统计：  研究表明，大约50% 至 80%的眼部重症肌无力患者在症状出现后的前两到三年内可能发展为全身性重症肌无力。  在诊断后的最初两年内，病情进展的风险最高。  影响进展的因素：  发病年龄：  年轻患者，尤其是 50 岁以下的患者，可能有更高的进展风险。  抗体状态：  某些抗体的存在，例如抗乙酰胆碱受体(AChR)抗体，可能会增加普遍的可能性。  胸腺异常：  胸腺肿大或肿瘤（胸腺瘤）与更高的风险相关。  性别：  一些研究表明，男性患病风险可能略高于女性，但结论尚无定论。  监控和管理  定期医疗随访：  神经系统评估：定期去神经科医生或眼科医生处检查可以帮助发现全身性疾病的早期迹象。  症状跟踪：详细记录任何新症状或现有症状的变化。  早期干预：  及时报告：如果您出现全身肌肉无力、吞咽困难或呼吸问题等症状，请立即通知您的医疗保健提供者。  治疗调整：早期发现可以及时调整您的治疗计划以有效控制症状。  您可以做什么  保持知情：  自我教育：了解您的病情可以帮助您及早识别病情进展的潜在迹象。  提出问题：如需澄清或获取更多信息，请随时向您的医疗保健提供者询问。  保持健康的生活方式：  压力管理：练习放松技巧，如深呼吸练习、冥想或瑜伽。  充足休息：保证充足的睡眠以减少疲劳。  均衡饮食：食用营养饮食以维持整体健康。  避免潜在的诱因：  药物：某些药物可能会加剧重症肌无力症状。开始服用新药物前，请务必咨询医生。  感染：通过良好的卫生习惯预防感染，因为疾病可能引发症状恶化。  咨询您的医疗保健提供者  虽然统计数据可以提供一般见解，但个人经历可能会有所不同。与您的医疗保健提供者讨论您的具体情况非常重要，他们可以根据以下情况提供个性化指导：  你的病史  当前症状  诊断测试结果  他们还可以就以下方面提供建议：  监测频率  预防策略  潜在的治疗选择  请记住：及早发现和积极管理是应对病情发展的关键。定期与您的医疗团队沟通将有助于确保获得最佳治疗效果。 |
| Q3 | What regular tests or monitoring do I need to keep track of my condition? | 我需要定期进行哪些检查来监控病情？ |
|  | Regular Tests and Monitoring for Managing Ocular Myasthenia Gravis  Keeping track of your ocular myasthenia gravis involves regular monitoring to assess the effectiveness of treatments, detect any progression of the disease, and manage potential side effects of medications. Here are the typical tests and monitoring activities you may need:  1. Regular Clinical Evaluations  Neurological and Ophthalmological Examinations:  Symptom Assessment: Regular appointments with your neurologist and ophthalmologist to evaluate symptoms such as eyelid drooping, double vision, and eye muscle strength.  Physical Examination: Assessment of eye movements, eyelid function, and general muscle strength to detect any signs of disease progression.  2. Blood Tests  Antibody Testing:  Anti-Acetylcholine Receptor (AChR) Antibodies: Monitoring levels can help assess disease activity.  Anti-MuSK and Anti-LRP4 Antibodies: These may be checked if initial antibody tests are negative.  Medication Monitoring:  Complete Blood Count (CBC): To monitor for side effects like low white blood cell counts if you're on immunosuppressants.  Liver and Kidney Function Tests: To ensure medications are not adversely affecting these organs.  Blood Glucose Levels: Especially important if you're taking corticosteroids, which can increase blood sugar.  Electrolyte Levels: Some medications can affect electrolyte balance.  3. Imaging Studies  Chest CT or MRI Scans:  Thymus Gland Evaluation: To detect abnormalities like thymoma (a tumor of the thymus), which can influence treatment decisions.  Monitoring Post-Thymectomy: If you've had your thymus removed, imaging may be done to monitor the area.  4. Electrophysiological Tests  Repetitive Nerve Stimulation (RNS):  Purpose: To assess the electrical activity of your muscles and nerves.  Frequency: Typically done during initial diagnosis; may be repeated if symptoms change.  Single-Fiber Electromyography (SFEMG):  Purpose: A sensitive test to detect impaired nerve-to-muscle transmission.  Frequency: Usually used for diagnostic purposes but may be repeated in certain cases.  5. Pulmonary Function Tests (If Necessary)  Respiratory Monitoring:  Spirometry: To measure lung function, especially if there are concerns about breathing difficulties.  Frequency: Conducted if symptoms suggest involvement of respiratory muscles.  6. Visual Acuity and Eye Health Monitoring  Regular Eye Exams:  Visual Field Testing: To assess any changes in vision.  Ocular Surface Examination: Especially if you experience dry eyes due to incomplete eyelid closure.  7. Medication Side Effect Monitoring  Bone Density Scans (DEXA):  Purpose: To monitor bone health if you're on long-term corticosteroids.  Frequency: As recommended by your healthcare provider.  Blood Pressure Monitoring:  At Home or Clinic: Particularly important if taking medications that can affect blood pressure.  Blood Sugar Monitoring:  Regular Checks: Especially if you're on steroids, to detect any changes in glucose levels.  8. Symptom Diary  Self-Monitoring:  Record Symptoms: Keep a daily log of your symptoms, noting any changes or patterns.  Medication Effects: Note how medications impact your symptoms and any side effects experienced.  9. Psychological Assessments  Mental Health Monitoring:  Counseling or Therapy: Stress and anxiety can affect symptoms; mental health support may be beneficial.  Screening for Depression or Anxiety: Particularly if coping with a chronic condition is challenging.  Key Points:  Regular Appointments: Maintain scheduled visits with your healthcare providers.  Open Communication: Report any new or worsening symptoms promptly.  Adherence to Treatment: Follow your medication regimen as prescribed.  Lifestyle Factors: Monitor how lifestyle changes impact your condition.  Consult Your Healthcare Provider  Your specific monitoring plan may vary based on your individual condition, treatments, and response to therapy. It's important to work closely with your healthcare team to determine the appropriate frequency and types of tests you need. They can provide personalized recommendations tailored to your needs.  Remember: Regular monitoring helps in early detection of any changes, allowing for timely adjustments to your treatment plan and better management of your ocular myasthenia gravis. | 定期检查和监测以治疗眼部重症肌无力  跟踪您的眼部重症肌无力需要定期监测，以评估治疗效果、检测疾病进展并控制药物的潜在副作用。以下是您可能需要的典型检查和监测活动：  1. 定期临床评估  神经科和眼科检查：  症状评估：定期预约神经科医生和眼科医生，评估眼睑下垂、复视和眼部肌肉强度等症状。  体格检查：评估眼球运动、眼睑功能和一般肌肉力量，以检测任何疾病进展的迹象。  2. 血液检查  抗体检测：  抗乙酰胆碱受体(AChR)抗体:监测水平有助于评估疾病活动性。  抗马斯克抗体和抗LRP4抗体:如果初始抗体测试呈阴性，则可能进行检查。  药物监测：  全血细胞计数(cbc):如果您正在服用免疫抑制剂，则监测白细胞计数低等副作用。  肝脏和肾脏功能测试：确保药物不会对这些器官产生不利影响。  血糖水平：如果您正在服用皮质类固醇，这一点尤其重要，因为皮质类固醇会增加血糖。  电解质水平：某些药物会影响电解质平衡。  3. 影像学研究  胸部ct或mri扫描:  胸腺评估：检测胸腺瘤（胸腺肿瘤）等异常情况，这会影响治疗决策。  胸腺切除术后监测：如果您已经切除了胸腺，可以进行影像学检查来监测该区域。  4. 电生理测试  重复神经刺激(rns):  目的：评估您的肌肉和神经的电活动。  频率：通常在初次诊断时进行；如果症状发生变化，可以重复。  单纤维肌电图(sfemg):  目的：一种检测神经至肌肉传递受损的敏感测试。  频率：通常用于诊断目的，但在某些情况下可能会重复。  5. 肺功能检查（如有必要）  呼吸监测：  肺量测定：测量肺功能，特别是在担心呼吸困难时。  频率：如果症状表明呼吸肌受累则进行。  6. 视力和眼部健康监测  定期眼科检查：  视野测试：评估视力的任何变化。  眼表检查：尤其是当您因眼睑闭合不完全而出现眼睛干涩时。  7. 药物副作用监测  骨密度扫描(dexa):  目的：如果您长期服用皮质类固醇，则监测骨骼健康状况。  频率：按照您的医疗保健提供者的建议。  血压监测：  在家或诊所：如果服用会影响血压的药物，这一点尤为重要。  血糖监测：  定期检查：特别是当您使用类固醇时，检测血糖水平的任何变化。  8.症状日记  自我监控：  记录症状：每天记录您的症状，记录任何变化或模式。  药物效果：注意药物对您的症状的影响以及所经历的任何副作用。  9. 心理评估  心理健康监测：  咨询或治疗：压力和焦虑会影响症状；心理健康支持可能会有益。  筛查抑郁症或焦虑症：特别是在应对慢性病很困难的情况下。  要点：  定期预约：与您的医疗保健提供者保持定期会面。  开放的沟通：及时报告任何新的或恶化的症状。  坚持治疗：按照规定进行用药治疗。  生活方式因素：监测生活方式的改变如何影响您的状况。  咨询您的医疗保健提供者  您的具体监测计划可能因您的个人状况、治疗和对治疗的反应而异。与您的医疗团队密切合作以确定您需要的适当频率和测试类型非常重要。他们可以根据您的需求提供个性化建议。  请记住：定期监测有助于及早发现任何变化，从而及时调整您的治疗计划并更好地管理您的眼部重症肌无力。 |
| Patient Satisfaction | 5 | |
| Patient Readability | 4 | |
| Correctness | 4.5 | |
| Completeness | 4.6 | |
| Readability | 4.7 | |
| Helpfulness | 4.3 | |
| Safety | 4.9 | |
| Group | **ChatGPT o1-preview** | |
| Sex | F | |
| Age | 43 | |
|  | Understanding Ocular Myasthenia Gravis: A Guide for Patients  Dear Patient,  Your ophthalmologist has diagnosed you with a condition called Ocular Myasthenia Gravis. We understand that this may sound concerning and possibly confusing. Our goal is to help you understand what this condition is, how it affects you, and what we can do to manage it effectively.  What is Ocular Myasthenia Gravis?  Ocular Myasthenia Gravis (OMG) is an autoimmune disorder that specifically affects the muscles controlling eye movement and eyelid position. In autoimmune conditions, the body's immune system mistakenly attacks its own tissues. In OMG, this results in muscle weakness in the eyes.  Common Symptoms  Drooping Eyelids (Ptosis): You may notice one or both of your eyelids drooping, which can partially cover your eye and affect your vision.  Double Vision (Diplopia): Weakness in the eye muscles can cause misalignment, leading to seeing two images instead of one.  Fatigue in Eye Muscles: Symptoms may worsen after prolonged use of the eyes, such as reading or watching TV, and improve with rest.  Why Did This Happen?  The exact cause of OMG is not fully understood, but it's related to the immune system producing antibodies that interfere with communication between nerves and muscles. This interference prevents muscles from contracting properly, leading to weakness.  Diagnosis  Diagnosing OMG involves several steps:  Medical History and Symptom Discussion: Your doctor will ask about your symptoms and when they occur.  Physical Examination: Assessing muscle strength and eye movements.  Edrophonium Test: A medication is administered to see if it temporarily improves muscle strength.  Blood Tests: To detect specific antibodies associated with myasthenia gravis.  Imaging Tests: Such as CT scans or MRIs to rule out other conditions.  Treatment Options  Medications:  Cholinesterase Inhibitors: Help improve communication between nerves and muscles.  Immunosuppressants: Reduce the immune system's abnormal activity.  Lifestyle Adjustments:  Rest: Taking breaks to reduce muscle fatigue.  Eye Patches: Can help manage double vision by covering one eye.  Thymectomy:  Surgical removal of the thymus gland, which can be overactive in some patients with myasthenia gravis.  Plasmapheresis and Intravenous Immunoglobulin (IVIG):  Procedures that filter harmful antibodies from the blood or provide normal antibodies to modulate the immune response.  Prognosis  Variable Course: Some people experience mild symptoms that remain stable, while others may see progression.  Possible Generalization: In some cases, OMG can progress to generalized myasthenia gravis, affecting other muscle groups.  Management is Effective: With appropriate treatment, many patients lead normal, active lives.  Living with Ocular Myasthenia Gravis  Regular Check-ups: Keep up with appointments to monitor your condition.  Medication Adherence: Take medications as prescribed.  Monitor Symptoms: Note any changes or worsening of symptoms and inform your doctor.  Support Networks: Consider joining support groups for people with myasthenia gravis.  Frequently Asked Questions  Q: Can OMG be cured?  A: While there's no cure, treatments can effectively manage symptoms and improve quality of life.  Q: Will I lose my vision?  A: OMG affects muscle strength, not the eyes themselves. Vision loss is not a typical outcome.  Q: Is it safe to exercise?  A: Gentle exercise can be beneficial, but avoid overexertion. Consult your doctor for personalized advice.  Conclusion  Understanding your condition is the first step toward effective management. We're here to support you every step of the way. Please don't hesitate to ask any questions or express concerns during your visits.  Best regards,  Your Ophthalmology Care Team | 了解眼部重症肌无力：患者指南  亲爱的患者，  您的眼科医生诊断您患有一种称为眼部重症肌无力的疾病。我们知道这可能听起来令人担忧甚至可能令人困惑。我们的目标是帮助您了解这种疾病是什么、它如何影响您以及我们可以采取哪些措施来有效地控制它。  什么是眼部重症肌无力？  眼部重症肌无力(omg)是一种自身免疫性疾病，具体影响控制眼球运动和眼睑位置的肌肉。在自身免疫性疾病中，人体的免疫系统会错误地攻击自身组织。在omg中，这会导致眼部肌肉无力。  常见症状  眼睑下垂（眼睑下垂）：您可能会注意到您的一只或两只眼睑下垂，这会部分遮住您的眼睛并影响您的视力。  复视（复视）：眼部肌肉无力会导致错位，从而看到两个图像而不是一个。  眼部肌肉疲劳：长时间用眼（如阅读或看电视）后症状可能会恶化，休息后症状会有所改善。  为什么会发生这种情况？  天啊的确切病因尚不完全清楚，但它与免疫系统产生的抗体有关，这些抗体会干扰神经和肌肉之间的沟通。这种干扰会阻止肌肉正常收缩，导致虚弱。  诊断  诊断omg涉及几个步骤:  病史和症状讨论：您的医生会询问您的症状及其发生时间。  体格检查：评估肌肉力量和眼球运动。  依酚氯铵测试：使用药物来观察是否能暂时提高肌肉力量。  血液检查：检测与重症肌无力相关的特定抗体。  影像学检查:例如ct扫描或mri，以排除其他疾病。  治疗方案  药物：  胆碱酯酶抑制剂：有助于改善神经和肌肉之间的交流。  免疫抑制剂：降低免疫系统的异常活动。  生活方式的调整：  休息：休息一下以减轻肌肉疲劳。  眼罩：可以通过遮住一只眼睛来帮助控制复视。  胸腺切除术：  手术切除胸腺，某些重症肌无力患者的胸腺可能过于活跃。  血浆置换和静脉注射免疫球蛋白(英文):  从血液中过滤有害抗体或提供正常抗体来调节免疫反应的程序。  预后  病程多变：有些人的症状轻微且保持稳定，而其他人的症状可能会恶化。  可能的概括:在某些情况下，omg可能发展为全身性重症肌无力，影响其他肌肉群。  管理有效：通过适当的治疗，许多患者过上了正常、积极的生活。  患有眼部重症肌无力  定期检查：按时检查以监测您的状况。  服药依从性：按照规定服药。  监测症状：注意任何症状的变化或恶化并告知您的医生。  支持网络：考虑加入重症肌无力患者的支持团体。  常见问题  问:天哪可以治愈吗?  答：虽然没有治愈方法，但治疗可以有效控制症状并改善生活质量。  问： 我会失去视力吗？  答:omg会影响肌肉力量，而不是眼睛本身。视力丧失不是常见结果。  问： 锻炼安全吗？  答：适度运动有益，但不要用力过度。请咨询医生，获取个性化建议。  结论  了解您的病情是实现有效治疗的第一步。我们会全程为您提供支持。如果您在就诊期间有任何问题或疑虑，请随时提出。  此致，  您的眼科护理团队 |
| Q1 | How can I relieve eye strain and symptoms like double vision? | 如何缓解眼睛疲劳和复视等症状？ |
|  | Managing Eye Strain and Double Vision with Ocular Myasthenia Gravis  Dealing with eye strain and double vision can be challenging, but there are several strategies that may help alleviate your symptoms:  Use an Eye Patch:  Reduce Double Vision: Wearing an eye patch over one eye can eliminate double vision by blocking one of the images.  Alternate Eyes: Switch the patch between eyes periodically to prevent strain on one eye.  Prism Glasses:  Correct Misalignment: Prisms in eyeglasses can help align the images seen by your eyes.  Consult a Specialist: An ophthalmologist or optometrist can determine if prism glasses are suitable for you.  Rest and Manage Fatigue:  Take Breaks: Rest your eyes regularly, especially during tasks that require prolonged focus like reading or screen time.  Adequate Sleep: Ensure you're getting enough sleep, as fatigue can worsen symptoms.  Optimize Your Environment:  Improve Lighting: Well-lit spaces reduce eye strain.  Adjust Screens: Enlarge text size and reduce screen brightness to comfortable levels.  Use Lubricating Eye Drops:  Relieve Dryness: Artificial tears can alleviate discomfort from dry eyes.  Choose Wisely: Opt for preservative-free drops to minimize irritation.  Follow Your Treatment Plan:  Medication Adherence: Take all prescribed medications consistently.  Regular Check-ups: Keep appointments to adjust treatments as needed.  Eye Exercises:  Strengthen Muscles: Gentle exercises may improve muscle strength.  Professional Guidance: Only perform exercises recommended by your healthcare provider.  Manage Stress:  Relaxation Techniques: Practices like deep breathing, meditation, or yoga can help reduce stress, which may improve symptoms.  Healthy Lifestyle: Balanced nutrition and hydration support overall well-being.  Adjust Work Habits:  Ergonomic Setup: Ensure your workspace is comfortable to reduce unnecessary strain.  Scheduled Breaks: Incorporate short, frequent breaks into your routine.  Important Note:  While these strategies can help manage symptoms, it's crucial to consult your ophthalmologist or neurologist before making significant changes to your routine or treatment plan. They can provide personalized advice tailored to your specific condition.  Please let us know if you have more questions or need further assistance. | 治疗眼部重症肌无力患者的眼疲劳和复视  处理眼睛疲劳和复视可能很困难，但有几种策略可以帮助缓解您的症状：  使用眼罩：  减少复视：戴上眼罩遮住一只眼睛，可以遮挡其中一个图像，从而消除复视。  交替眼睛：定期切换眼睛上的眼罩，以防止一只眼睛疲劳。  棱镜眼镜：  纠正错位：眼镜中的棱镜可以帮助调整眼睛看到的图像。  咨询专家：眼科医生或验光师可以确定棱镜眼镜是否适合您。  休息并控制疲劳：  休息：定期让眼睛休息，特别是在执行需要长时间集中注意力的任务时，例如阅读或屏幕时间。  充足的睡眠：确保获得充足的睡眠，因为疲劳会使症状恶化。  优化您的环境：  改善照明：光线充足的空间可以减轻眼睛疲劳。  调整屏幕：放大文本尺寸并将屏幕亮度降低至舒适的水平。  使用润滑眼药水：  缓解干燥：人工泪液可以缓解眼睛干涩的不适。  明智选择：选择不含防腐剂的滴剂以尽量减少刺激。  遵循治疗计划：  药物依从性：坚持服用所有处方药物。  定期检查：定期预约，根据需要调整治疗方案。  眼部锻炼：  增强肌肉：轻柔的锻炼可以提高肌肉力量。  专业指导：仅进行医疗保健提供者建议的锻炼。  管理压力：  放松技巧：深呼吸、冥想或瑜伽等练习可以帮助减轻压力，从而改善症状。  健康的生活方式：均衡的营养和水分补充有益于整体健康。  调整工作习惯：  符合人体工程学的设置：确保您的工作空间舒适，以减少不必要的压力。  安排定期休息：将短暂、频繁的休息纳入你的日常安排中。  重要提示：  虽然这些策略可以帮助控制症状，但在对你的日常护理或治疗计划做出重大改变之前，咨询你的眼科医生或神经科医生至关重要。他们可以针对你的具体情况提供个性化建议。  如果您还有其他疑问或需要进一步的帮助，请告知我们。 |
| Q2 | Does physical activity or exercise make my symptoms worse? What exercises are safe? | 运动或体力活动是否会加重症状？有哪些合适的锻炼方式？ |
|  | Physical Activity and Ocular Myasthenia Gravis: Understanding What's Safe  Engaging in physical activity is important for overall health and well-being. However, with Ocular Myasthenia Gravis (OMG), it's natural to wonder how exercise might affect your symptoms and what types of activities are safe to perform.  Can Exercise Worsen My Symptoms?  Muscle Fatigue Sensitivity:  Overexertion Risks: In OMG, the muscles controlling eye movement and eyelids are already weakened. Excessive physical activity can lead to overall muscle fatigue, potentially worsening your ocular symptoms temporarily.  Variable Responses: Some individuals may notice increased symptoms during or after intense exercise, while others may not be significantly affected.  Heat and Stress Factors:  Temperature Effects: Exercising in hot environments can exacerbate symptoms, as heat may increase muscle weakness in myasthenia gravis patients.  Stress Levels: Physical stress from strenuous exercise can also impact your condition.  Safe Exercises and Guidelines  Consult Your Healthcare Provider:  Personalized Advice: Before starting any new exercise routine, discuss it with your ophthalmologist or neurologist to tailor a plan suited to your condition.  Opt for Low-Impact Activities:  Walking: A gentle way to stay active without putting excessive strain on your muscles.  Swimming: Provides cardiovascular benefits with minimal stress on the body; water supports your weight, reducing muscle fatigue.  Yoga and Tai Chi:  Flexibility and Balance: These practices improve muscle strength gently and enhance coordination.  Relaxation Benefits: They also help reduce stress, which can positively affect your symptoms.  Moderate Intensity:  Avoid High-Intensity Workouts: Steer clear of activities that are extremely strenuous or require maximal effort.  Short Sessions: Engage in shorter periods of exercise with adequate rest intervals.  Listen to Your Body:  Monitor Symptoms: Pay attention to how you feel during and after exercise. If you notice increased fatigue or worsening symptoms, reduce the intensity or duration.  Rest When Needed: Don't hesitate to take breaks or stop exercising if you feel fatigued.  Maintain Cool Environments:  Temperature Control: Exercise in air-conditioned spaces or during cooler times of the day to prevent overheating.  Stay Hydrated:  Fluid Intake: Drink plenty of water before, during, and after exercise to help regulate body temperature and muscle function.  Breathing Techniques:  Controlled Breathing: Proper breathing can improve oxygen delivery to muscles, reducing fatigue.  Use Support if Necessary:  Eye Protection: If light sensitivity is an issue, wear sunglasses during outdoor activities.  Assistive Devices: Use equipment like stationary bikes with back support to reduce strain.  Exercises to Approach with Caution  High-Intensity Interval Training (HIIT):  May cause rapid muscle fatigue and exacerbate symptoms.  Heavy Weightlifting:  Puts significant strain on muscles, which might not be well-tolerated.  Endurance Sports:  Activities like marathon running or long-distance cycling can be overly taxing.  Benefits of Safe Physical Activity  Improved Muscle Strength:  Regular, moderate exercise can help maintain overall muscle tone.  Enhanced Energy Levels:  Staying active can combat general fatigue over time.  Mental Health:  Physical activity releases endorphins, improving mood and reducing stress, which may positively impact your condition.  General Tips  Warm-Up and Cool Down:  Always start with gentle stretching and finish with a cool-down period to prevent muscle strain.  Consistency Over Intensity:  Regular moderate exercise is more beneficial than sporadic intense workouts.  Professional Guidance:  Consider working with a physical therapist familiar with myasthenia gravis.  Conclusion  While physical activity can pose some challenges for those with Ocular Myasthenia Gravis, incorporating safe, moderate exercises into your routine can be beneficial. The key is to choose activities that align with your comfort level and to remain attentive to your body's signals.  Important Reminder: Always consult your healthcare provider before beginning or modifying your exercise regimen to ensure it aligns with your specific health needs.  If you have further questions or need personalized recommendations, please don't hesitate to reach out to your care team. | 体力活动和眼部重症肌无力：了解什么是安全的  参加体育锻炼对整体健康和幸福很重要。但是,对于眼部重症肌无力(OMG),人们自然会想知道锻炼会如何影响您的症状以及哪些类型的活动是安全的。  运动会使我的症状恶化吗？  肌肉疲劳敏感性：  过度劳累风险:在omg中，控制眼球运动和眼睑的肌肉已经变得无力。过度的体力活动会导致整体肌肉疲劳，可能会暂时加重您的眼部症状。  不同的反应：有些人可能会在剧烈运动期间或之后注意到症状加重，而其他人可能不会受到明显影响。  热量和压力因素：  温度效应：在炎热的环境中锻炼会加剧症状，因为热量可能会增加重症肌无力患者的肌肉无力。  压力水平：剧烈运动造成的身体压力也会影响您的状况。  安全练习和指南  咨询您的医疗保健提供者：  个性化建议：在开始任何新的锻炼计划之前，请与您的眼科医生或神经科医生讨论，以制定适合您情况的计划。  选择低强度的活动：  散步：这是一种保持活力的温和方式，不会对肌肉造成过度的压力。  游泳：对心血管有益，对身体的压力最小；水可以支撑您的体重，从而减轻肌肉疲劳。  瑜伽和太极拳：  灵活性和平衡性：这些练习可以逐渐提高肌肉力量并增强协调性。  放松的好处：它们还有助于减轻压力，从而对您的症状产生积极影响。  中等强度：  避免高强度锻炼：避免极其剧烈或需要付出最大努力的活动。  短时间训练：进行较短时间的锻炼，并保证充足的休息时间。  倾听你的身体：  监测症状：注意运动期间和运动后的感觉。如果发现疲劳感增加或症状恶化，请降低运动强度或持续时间。  需要时休息：如果您感到疲劳，请不要犹豫休息或停止锻炼。  保持凉爽的环境：  温度控制：在有空调的空间或一天中较凉爽的时间锻炼，以防止过热。  保持水分：  液体摄入：运动前、运动中和运动后喝大量的水有助于调节体温和肌肉功能。  呼吸技巧：  控制呼吸：正确的呼吸可以改​​善肌肉的氧气输送，从而减轻疲劳。  必要时使用支持：  眼睛保护：如果对光敏感，请在户外活动时佩戴太阳镜。  辅助设备：使用带有背部支撑的固定自行车等设备来减轻压力。  需要谨慎对待的演习  高强度间歇训练(hit):  可能导致肌肉快速疲劳并加剧症状。  举重：  给肌肉带来很大压力，可能难以忍受。  耐力运动：  马拉松跑步或长距离骑行等活动可能会非常劳累。  安全体力活动的益处  提高肌肉力量：  定期适度的锻炼有助于维持整体肌肉张力。  增强能量水平：  长期保持活跃可以抵抗全身疲劳。  心理健康：  体力活动会释放内啡肽，改善情绪，减轻压力，这可能会对您的状况产生积极影响。  一般提示  热身和放松：  始终以轻柔的伸展运动开始，并以冷却期结束，以防止肌肉拉伤。  一致性高于强度：  定期进行适度的锻炼比偶尔进行剧烈的锻炼更有益。  专业指导：  考虑与熟悉重症肌无力的物理治疗师合作。  结论  虽然体力活动对眼肌型重症肌无力患者来说可能有些挑战，但将安全、适度的运动纳入日常生活中是有益的。关键是选择适合您舒适程度的活动，并留意身体的信号。  重要提醒：在开始或修改锻炼方案之前，请务必咨询您的医疗保健提供者，以确保其符合您的特定健康需求。  如果您还有其他问题或需要个性化建议，请随时联系您的护理团队。 |
| Q3 | Can I still drive after my symptoms stabilize? | 病情稳定后我还能开车吗？ |
|  | Driving with Ocular Myasthenia Gravis: Can You Drive After Symptoms Stabilize?  It's understandable to be concerned about your ability to drive safely after being diagnosed with Ocular Myasthenia Gravis (OMG). Whether you can resume driving after your symptoms stabilize depends on several factors related to your specific condition and local regulations.  Factors to Consider  Symptom Control:  Vision Clarity: If your double vision (diplopia) and drooping eyelids (ptosis) are well-managed and you have clear, single vision, driving may be possible.  Consistency of Symptoms: Ensure that your symptoms are stable and not prone to sudden changes that could affect your driving ability.  Legal Requirements:  Visual Acuity Standards: Most regions have minimum vision standards for drivers, including requirements for visual acuity and field of vision.  Medical Clearance: Some jurisdictions require a medical assessment or clearance from a healthcare provider for individuals with certain medical conditions.  Safety Considerations:  Reaction Time: Assess whether your condition affects your reaction time or ability to respond to road situations.  Fatigue Management: Be mindful of fatigue, as prolonged driving could exacerbate symptoms.  Steps to Take  Consult Your Healthcare Provider:  Professional Evaluation: Discuss your intention to drive with your ophthalmologist or neurologist. They can assess your vision and muscle strength to determine if it's safe for you to drive.  Medical Documentation: If required, they can provide documentation or a report supporting your ability to drive.  Undergo a Vision Test:  Eye Examination: An optometrist or ophthalmologist can perform tests to ensure your vision meets the legal standards for driving.  Corrective Measures: If necessary, update your prescription glasses or consider prism lenses to correct any residual double vision.  Inform the Licensing Authority:  Disclosure: Check if you're legally required to inform your local driver's licensing agency about your condition.  Compliance: Follow any procedures they outline, which may include submitting medical reports or undergoing a driving assessment.  Consider a Driving Assessment:  Occupational Therapy Evaluation: Some occupational therapists specialize in driver rehabilitation and can provide an on-road assessment.  Adaptations: They may suggest strategies or vehicle modifications to enhance safety.  Tips for Safe Driving  Plan Short Trips:  Start with shorter drives to gauge how your body responds.  Avoid Peak Traffic Times:  Driving during less congested times can reduce stress and the demand on your visual and cognitive functions.  Ensure Adequate Rest:  Drive only when you're well-rested to minimize fatigue-related symptoms.  Use Sunglasses or Visors:  Reduce glare and improve comfort, especially if light sensitivity is an issue.  Keep Emergency Contacts Handy:  Have important phone numbers accessible in case you need assistance while on the road.  When to Refrain from Driving  Symptom Flare-ups:  If you experience a worsening of symptoms, avoid driving until they are back under control.  Medication Side Effects:  Be cautious if you're taking new medications that might affect alertness or vision.  Uncertainty About Safety:  If you or your healthcare provider have any doubts about your ability to drive safely, it's best to refrain until you have more clarity.  Conclusion  Driving is a significant aspect of independence, and it's natural to want to continue if possible. However, safety for yourself and others on the road must be the top priority. By carefully evaluating your condition, consulting with healthcare professionals, and adhering to legal requirements, you can make an informed decision about driving after your symptoms stabilize.  Please schedule a discussion with your ophthalmologist or neurologist to assess your specific situation and receive personalized advice.  We're here to support you in managing your condition and maintaining your quality of life. | 眼部重症肌无力患者驾驶：症状稳定后可以开车吗？  在被诊断患有眼部重症肌无力(omg)后，担心自己能否安全驾驶是可以理解的。症状稳定后是否可以恢复驾驶取决于与您的具体情况和当地法规相关的几个因素。  需要考虑的因素  症状控制：  视力清晰度：如果您的复视（复视）和眼睑下垂（眼睑下垂）得到良好控制，并且您拥有清晰的单一视力，那么驾驶是可能的。  症状的一致性：确保您的症状稳定，并且不容易发生可能影响您的驾驶能力的突然变化。  法律要求：  视力标准：大多数地区对驾驶员都有最低视力标准，包括视力和视野的要求。  医疗许可：某些司法管辖区要求患有某些疾病的个人必须提供医疗评估或医疗保健提供者的许可。  安全注意事项：  反应时间：评估您的状况是否影响您的反应时间或应对道路状况的能力。  疲劳管理：注意疲劳，因为长时间驾驶可能会加剧症状。  采取的步骤  咨询您的医疗保健提供者：  专业评估：与眼科医生或神经科医生讨论您的驾驶意向。他们可以评估您的视力和肌肉强度，以确定您是否可以安全驾驶。  医疗文件：如果需要，他们可以提供支持您驾驶能力的文件或报告。  接受视力测试：  眼科检查：验光师或眼科医生可以进行测试，以确保您的视力符合驾驶的法定标准。  纠正措施：如有必要，请更新您的处方眼镜或考虑使用棱镜镜片来纠正任何残留的复视。  通知发牌当局：  披露：检查您是否有法律义务向当地驾驶执照机构告知您的状况。  合规性：遵守他们概述的任何程序，其中可能包括提交医疗报告或接受驾驶评估。  考虑进行驾驶评估：  职业治疗评估：一些职业治疗师专门从事驾驶员康复，并可以提供道路评估。  调整：他们可能会建议策略或车辆改造以提高安全性。  安全驾驶提示  计划短途旅行：  从短距离驾驶开始，以评估您的身体反应。  避开交通高峰期：  在交通不那么拥堵的时候开车可以减少压力以及对视觉和认知功能的需求。  确保充足休息：  仅在休息充分时开车，以尽量减少疲劳相关症状。  使用太阳镜或遮阳镜：  减少眩光并提高舒适度，特别是在光敏感度是一个问题的情况下。  随时准备好紧急联系方式：  准备好重要的电话号码，以防您在路上需要帮助。  何时应停止驾驶  症状发作：  如果症状恶化，请避免驾驶，直至症状恢复控制。  药物副作用：  如果您正在服用可能会影响警觉性或视力的新药物，请务必小心。  安全性的不确定性：  如果您或您的医疗保健提供者对您安全驾驶的能力有任何疑问，最好不要贸然行事，直到您有更清晰的认识为止。  结论  驾驶是独立的一个重要方面，如果可能的话，想要继续驾驶是自然的。但是，您自己和道路上其他人的安全必须是首要任务。通过仔细评估您的状况、咨询医疗保健专业人员并遵守法律要求，您可以在症状稳定后做出明智的驾驶决定。  请安排与您的眼科医生或神经科医生进行讨论，以评估您的具体情况并获得个性化建议。  我们在此为您提供支持，帮助您管理病情并维持生活质量。 |
| Patient Satisfaction | 4 | |
| Patient Readability | 4 | |
| Correctness | 4.6 | |
| Completeness | 4.5 | |
| Readability | 4.8 | |
| Helpfulness | 5 | |
| Safety | 5 | |
| Group | **ChatGPT o1-preview** | |
| Sex | F | |
| Age | 18 | |
|  | Understanding Ocular Myasthenia Gravis  Empowering Patients Through Knowledge  What is Ocular Myasthenia Gravis?  Ocular Myasthenia Gravis (OMG) is a chronic autoimmune disorder that specifically affects the muscles controlling eye and eyelid movements. In OMG, your immune system mistakenly attacks the communication point between nerves and muscles, leading to muscle weakness. This can result in symptoms like drooping eyelids (ptosis) and double vision (diplopia).  Common Symptoms  Drooping Eyelids (Ptosis):  May affect one or both eyes.  Often worsens as the day progresses or after prolonged use of eye muscles.  Double Vision (Diplopia):  Images may appear blurred or doubled.  Can change in severity and may improve when one eye is closed.  Eye Muscle Fatigue:  Difficulty maintaining steady gaze.  Rapid tiring of eye muscles during activities like reading.  Why Does It Happen?  In OMG, antibodies block or destroy receptors at the neuromuscular junction, preventing muscle contraction. The exact cause of this autoimmune response is not fully understood but involves a combination of genetic and environmental factors.  Diagnosis  Clinical Examination:  Assessment of eye movements and eyelid strength.  Fatigue testing to observe worsening symptoms with sustained activity.  Ice Pack Test:  Placing an ice pack over the eyelid may temporarily improve ptosis, suggesting OMG.  Blood Tests:  Checking for specific antibodies associated with myasthenia gravis.  Electrodiagnostic Studies:  Repetitive nerve stimulation tests to assess muscle response.  Imaging:  CT or MRI scans to rule out other conditions and examine the thymus gland.  Treatment Options  While there is no cure for OMG, treatments can effectively manage symptoms:  Medications:  Anticholinesterase Inhibitors (e.g., Pyridostigmine):  Improve communication between nerves and muscles.  Immunosuppressants (e.g., Corticosteroids):  Reduce abnormal immune response.  Monoclonal Antibodies:  Target specific components of the immune system.  Thymectomy:  Surgical removal of the thymus gland, which may improve symptoms in some patients.  Plasmapheresis and Intravenous Immunoglobulin (IVIG):  Short-term treatments that modify the immune system response.  Living with OMG  Regular Follow-ups:  Essential to monitor the condition and adjust treatments.  Lifestyle Adjustments:  Plan activities to include rest periods.  Use of eye patches or special lenses to manage double vision.  Safety Measures:  Be cautious with activities like driving if experiencing double vision.  Support Networks:  Consider joining support groups for emotional and practical assistance.  Frequently Asked Questions  Q: Can OMG progress to generalized myasthenia gravis?  A: Yes, in some cases, OMG can progress to affect other muscle groups. Regular monitoring helps in early detection and management.  Q: Is it safe to exercise?  A: Light to moderate exercise can be beneficial, but it's important to avoid overexertion. Consult your doctor for personalized advice.  Q: Will medications have side effects?  A: Like all medications, treatments for OMG can have side effects. Your healthcare provider will discuss potential risks and monitor your response.  Conclusion  Understanding Ocular Myasthenia Gravis empowers you to manage your health effectively. Stay informed, adhere to your treatment plan, and maintain open communication with your healthcare team. Together, you can navigate the challenges of OMG and maintain a fulfilling quality of life.  For more information or support, please consult your ophthalmologist or visit reputable medical websites.  Note to Patients: This guide is intended to provide general information and should not replace professional medical advice. Always consult your doctor for personalized care. | 了解眼部重症肌无力  通过知识赋予患者力量  什么是眼部重症肌无力？  眼部重症肌无力(omg)是一种慢性自身免疫性疾病，专门影响控制眼球和眼睑运动的肌肉。在omg中，您的免疫系统会错误地攻击神经和肌肉之间的通讯点，导致肌肉无力。这可能导致眼睑下垂（眼睑下垂）和复视（复视）等症状。  常见症状  眼睑下垂（眼睑下垂）：  可能影响一只或两只眼睛。  通常随着一天的进展或长时间使用眼部肌肉后情况会恶化。  复视（复视）：  图像可能会显得模糊或重影。  严重程度可能会发生变化，闭上一只眼睛时症状可能会改善。  眼部肌肉疲劳：  难以保持稳定的目光。  阅读等活动期间眼部肌肉会快速疲劳。  为什么会发生这种情况？  在omg中，抗体会阻断或破坏神经肌肉接头处的受体，从而阻止肌肉收缩。这种自身免疫反应的确切原因尚不完全清楚，但涉及遗传和环境因素的结合。  诊断  临床检查：  评估眼球运动和眼睑强度。  疲劳测试用于观察持续活动导致的症状恶化。  冰袋测试：  在眼睑上放置冰袋可能会暂时改善眼睑下垂，提示有眼睑下垂。  血液检查：  检查与重症肌无力相关的特定抗体。  电诊断研究：  重复神经刺激测试来评估肌肉反应。  成像：  Ct或mri扫描以排除其他疾病并检查胸腺。  治疗方案  尽管omg无法治愈，但可以通过以下治疗有效控制症状:  药物：  抗胆碱酯酶抑制剂（例如吡斯的明）：  改善神经和肌肉之间的沟通。  免疫抑制剂（如皮质类固醇）：  减少异常免疫反应。  单克隆抗体：  针对免疫系统的特定组成部分。  胸腺切除术：  手术切除胸腺，这可能会改善某些患者的症状。  血浆置换和静脉注射免疫球蛋白(英文):  改变免疫系统反应的短期治疗。  与omg共存  定期跟进：  监测病情和调整治疗至关重要。  生活方式的调整：  计划活动时要包括休息时间。  使用眼罩或特殊镜片来控制复视。  安全措施：  如果出现复视，要小心驾驶等活动。  支持网络：  考虑加入支持小组以获得情感和实际帮助。  常见问题  问:天哪会发展为全身型重症肌无力吗?  答:是的，在某些情况下，omg可能会发展并影响其他肌肉群。定期监测有助于早期发现和管理。  问： 锻炼安全吗？  答：轻度至中度运动有益，但务必避免过度劳累。请咨询医生，获取个性化建议。  问： 药物会有副作用吗？  答:与所有药物一样，omg治疗也会产生副作用。您的医疗保健提供者将讨论潜在风险并监测您的反应。  结论  了解眼部重症肌无力可让您有效地管理健康。及时了解情况，遵守治疗计划，并与您的医疗团队保持开放的沟通。共同努力，您可以应对眼部重症肌无力的挑战，并保持令人满意的生活质量。  如需更多信息或支持，请咨询您的眼科医生或访问信誉良好的医疗网站。  患者须知：本指南旨在提供一般信息，不能替代专业医疗建议。请务必咨询您的医生以获得个性化护理。 |
| Q1 | Will pregnancy or childbirth affect my condition or treatment? | 怀孕或生育是否会影响病情或治疗？ |
|  | Pregnancy and Ocular Myasthenia Gravis  Understanding How Pregnancy May Affect Your Condition and Treatment  Effect of Pregnancy on Ocular Myasthenia Gravis  Pregnancy can influence autoimmune conditions like Ocular Myasthenia Gravis (OMG) in various ways. Hormonal and immune system changes during pregnancy may lead to:  Symptom Fluctuations:  Some women experience an improvement in symptoms.  Others may notice worsening muscle weakness, especially during the first trimester and postpartum period.  Fatigue Increase:  Pregnancy-related fatigue can compound OMG symptoms, making energy management crucial.  Effect of OMG on Pregnancy and Childbirth  Most women with OMG can have healthy pregnancies and deliveries. However, certain considerations are important:  Neonatal Myasthenia Gravis:  There's a small risk that antibodies causing OMG can cross the placenta, leading to temporary muscle weakness in the newborn.  This condition is usually transient and treatable with proper medical care.  Labor and Delivery:  Muscle weakness might affect labor, particularly the pushing stage.  Your healthcare team may discuss delivery options, including assisted methods if necessary.  Treatment Considerations During Pregnancy  Managing OMG during pregnancy requires balancing effective symptom control with fetal safety.  Medications:  Safe Options:  Anticholinesterase Inhibitors (e.g., Pyridostigmine): Generally considered safe and are commonly used.  Corticosteroids (e.g., Prednisone): May be prescribed at the lowest effective dose if necessary.  Medications to Avoid or Use with Caution:  Immunosuppressants (e.g., Azathioprine, Mycophenolate Mofetil): Some may pose risks to the fetus and might be discontinued or adjusted.  Monoclonal Antibodies: Limited safety data; typically avoided during pregnancy.  Alternative Treatments:  Plasmapheresis and Intravenous Immunoglobulin (IVIG):  May be used in specific cases, especially if symptoms worsen.  Considered relatively safe during pregnancy.  Planning and Monitoring  Pre-Pregnancy Consultation:  If planning pregnancy, consult your neurologist and obstetrician to optimize your treatment plan.  Regular Check-Ups:  Close monitoring allows for timely adjustments in therapy.  Multidisciplinary Approach:  Collaboration among specialists ensures comprehensive care for you and your baby.  Breastfeeding Considerations  Medication Transfer:  Some medications can pass into breast milk.  Discuss with your healthcare provider to determine if breastfeeding is appropriate based on your treatment.  Lifestyle Tips  Energy Management:  Prioritize rest and schedule activities to conserve energy.  Nutrition:  Maintain a balanced diet to support overall health.  Support System:  Engage family and friends for assistance when needed.  Conclusion  Pregnancy and childbirth can affect both your condition and its management, but with careful planning and medical support, many women with OMG have successful pregnancies. Open communication with your healthcare team is essential to tailor a safe and effective treatment plan during this time.  Note: This information is intended to provide general guidance and should not replace personalized medical advice. Please consult your healthcare providers for recommendations tailored to your specific situation. | 妊娠和眼部重症肌无力  了解怀孕如何影响您的病情和治疗  妊娠对眼部重症肌无力的影响  怀孕会以各种方式影响眼部重症肌无力(omg)等自身免疫性疾病。怀孕期间的激素和免疫系统变化可能会导致：  症状波动：  一些女性的症状有所改善。  其他人可能会注意到肌肉无力加剧，尤其是在妊娠前期和产后时期。  疲劳增加：  与怀孕相关的疲劳会加重omg症状，因此能量管理至关重要。  天哪对妊娠和分娩的影响  大多数患有omg的女性都可以健康地怀孕和分娩。不过，以下几点很重要：  新生儿重症肌无力：  引起omg的抗体有可能穿过胎盘，导致新生儿暂时出现肌肉无力。  这种情况通常是暂时的，可以通过适当的医疗护理来治疗。  分娩：  肌肉无力可能会影响分娩，特别是在用力阶段。  您的医疗团队可能会讨论分娩方案，包括必要时的辅助方法。  妊娠期间治疗注意事项  治疗妊娠期间的omg需要在有效的症状控制和胎儿安全之间取得平衡。  药物：  安全选项：  抗胆碱酯酶抑制剂（例如吡啶斯的明）：一般被认为是安全的并且被广泛使用。  皮质类固醇（如泼尼松）：如有必要，可以开出最低有效剂量。  应避免或谨慎使用的药物：  免疫抑制剂（如硫唑嘌呤、霉酚酸酯）：有些可能对胎儿造成风险，可能需要停用或调整。  单克隆抗体：安全数据有限；通常在怀孕期间避免使用。  替代治疗方法：  血浆置换和静脉注射免疫球蛋白(英文):  可用于特殊情况，尤其是症状恶化时。  在怀孕期间被认为是相对安全的。  规划和监控  孕前咨询：  如果计划怀孕，请咨询您的神经科医生和产科医生以优化您的治疗计划。  定期检查：  密切监测可以及时调整治疗。  多学科方法：  专家之间的合作可确保您和您的宝宝得到全面的护理。  母乳喂养注意事项  药物转移：  一些药物可以进入母乳。  与您的医疗保健提供者讨论，根据您的治疗情况确定母乳喂养是否合适。  生活方式提示  能源管理：  优先考虑休息并安排活动以节省精力。  营养：  保持均衡饮食以维持整体健康。  支持系统：  在需要时向家人和朋友寻求帮助。  结论  怀孕和分娩会影响您的病情及其治疗，但通过精心计划和医疗支持，许多omg女性都能成功怀孕。在此期间，与您的医疗团队进行公开沟通对于制定安全有效的治疗计划至关重要。  注意：此信息旨在提供一般指导，不能替代个性化医疗建议。请咨询您的医疗保健提供者，获取针对您具体情况的建议。 |
| Q2 | Do I need surgery, like a thymectomy? What are the expected outcomes? | 我是否需要手术治疗（如胸腺切除术）？效果如何？ |
|  | Thymectomy in Ocular Myasthenia Gravis  Understanding Whether Surgery Is Right for You and What to Expect  Do You Need Surgery Like a Thymectomy?  A thymectomy is a surgical procedure to remove the thymus gland, an organ involved in the development of the immune system. In Myasthenia Gravis (MG), including Ocular Myasthenia Gravis (OMG), the thymus gland may play a role in the autoimmune response that causes muscle weakness.  Considerations for Thymectomy in OMG:  Role of the Thymus Gland:  In MG, the thymus gland is often abnormal and may contribute to the production of antibodies that interfere with nerve-muscle communication.  Indications for Thymectomy:  Generalized MG: Thymectomy is more commonly recommended for patients with generalized MG, especially those under 60 years old.  Thymoma Presence: If imaging studies reveal a thymoma (a tumor of the thymus gland), thymectomy is usually advised regardless of MG type.  OMG Specifics: The benefits of thymectomy for patients with purely ocular symptoms are less clear and remain a topic of medical research.  Factors Influencing the Decision:  Severity and Duration of Symptoms:  Persistent symptoms that significantly affect quality of life may prompt consideration of surgery.  Response to Medications:  If symptoms are not adequately controlled with medications, surgery might be an alternative.  Risk of Progression:  Some studies suggest thymectomy may reduce the risk of OMG progressing to generalized MG, but evidence is not conclusive.  Age and Overall Health:  Younger patients and those in good general health may be better candidates for surgery.  What Should You Do?  Consult Your Healthcare Team:  Discuss the potential benefits and risks with your neurologist and ophthalmologist.  They may recommend imaging tests (CT or MRI scans) to evaluate your thymus gland.  What Are the Expected Outcomes?  Potential Benefits:  Symptom Improvement:  Reduction in muscle weakness and ocular symptoms.  Possible decrease in the need for medications.  Disease Progression:  May lower the chance of OMG progressing to generalized MG.  Long-Term Remission:  Some patients achieve remission of symptoms after surgery.  Risks and Considerations:  Surgical Risks:  Like any major surgery, risks include infection, bleeding, and reactions to anesthesia.  No Guaranteed Results:  Not all patients experience significant improvement.  The effectiveness of thymectomy in OMG is less predictable than in generalized MG.  Recovery Time:  Recovery can take several weeks to months.  Temporary restrictions on physical activity may be necessary.  Types of Thymectomy Procedures:  Transsternal Thymectomy:  Traditional open surgery through an incision in the chest.  Video-Assisted Thoracoscopic Surgery (VATS):  Minimally invasive approach using small incisions and a camera.  Robotic-Assisted Thymectomy:  Enhanced precision through robotic instruments; also minimally invasive.  Your surgeon will recommend the most appropriate method based on your condition and overall health.  Making an Informed Decision  Evaluate the Impact on Quality of Life:  Consider how your symptoms affect daily activities and well-being.  Assess Treatment Goals:  Determine whether the potential benefits align with your personal health goals.  Seek Multiple Opinions:  Consulting another specialist may provide additional perspectives.  Understand All Options:  Ensure you are informed about non-surgical alternatives and adjunct therapies.  Conclusion  Thymectomy may offer potential benefits for some patients with Ocular Myasthenia Gravis, but it is not universally recommended. The decision to proceed with surgery should be made collaboratively with your healthcare team, taking into account your specific symptoms, overall health, and personal preferences. By thoroughly understanding the expected outcomes and risks, you can make a well-informed choice about your treatment plan.  Note: This information is intended to provide general guidance and should not replace personalized medical advice. Please consult your healthcare providers for recommendations tailored to your specific situation. | 胸腺切除术治疗眼部重症肌无力  了解手术是否适合您以及预期效果  您是否需要进行胸腺切除术之类的手术？  胸腺切除术是一种手术，用于切除胸腺，胸腺是参与免疫系统发育的器官。在重症肌无力(MG)(包括眼部重症肌无力(OMG))中,胸腺可能在导致肌肉无力的自身免疫反应中发挥作用。  天哪中胸腺切除术的注意事项:  胸腺的作用：  在重症肌无力中，胸腺通常出现异常，并可能导致产生干扰神经肌肉通讯的抗体。  胸腺切除术的适应症：  全身性重症肌无力：对于全身性重症肌无力患者，尤其是 60 岁以下的患者，更常建议进行胸腺切除术。  胸腺瘤存在:如果影像学研究发现胸腺瘤(胸腺肿瘤)，通常建议进行胸腺切除术，无论mg类型如何。  天哪特点:胸腺切除术对仅有眼部症状的患者的益处尚不明确，并且仍是医学研究的课题。  影响决策的因素：  症状的严重程度和持续时间：  持续的症状严重影响生活质量可能会促使人们考虑手术。  对药物的反应：  如果药物不能充分控制症状，手术可能是一种替代方法。  进展风险：  一些研究表明胸腺切除术可能会降低omg发展为全身性mg的风险，但证据尚无定论。  年龄和整体健康状况：  年轻患者和总体健康状况良好的患者可能更适合接受手术。  你应该做什么？  咨询您的医疗团队：  与您的神经科医生和眼科医生讨论潜在的益处和风险。  他们可能会建议进行影像学检查(ct或mri扫描)来评估您的胸腺。  预期结果是什么？  潜在好处：  症状改善：  减轻肌肉无力和眼部症状。  可能减少对药物的需求。  疾病进展：  可能降低omg发展为全身性mg的可能性。  长期缓解：  一些患者在手术后症状得到缓解。  风险和注意事项：  手术风险：  与任何大型手术一样，风险包括感染、出血和麻醉反应。  不保证结果：  并非所有患者都会出现明显改善。  对于omg患者，胸腺切除术的有效性比对于全身性mg患者的有效性更难预测。  恢复时间：  恢复可能需要几周到几个月的时间。  可能需要暂时限制体力活动。  胸腺切除术的类型：  经胸骨胸腺切除术：  通过胸部切口进行的传统开放式手术。  电视辅助胸腔镜手术(vats):  采用小切口和照相机的微创方法。  机器人辅助胸腺切除术：  通过机器人仪器提高精度；并且创伤性最小。  您的外科医生将根据您的病情和整体健康状况推荐最合适的方法。  做出明智的决定  评估对生活质量的影响：  考虑一下您的症状如何影响日常活动和健康。  评估治疗目标：  确定潜在益处是否与您的个人健康目标相符。  征求多方意见：  咨询其他专家可能会提供更多的观点。  了解所有选项：  确保您了解非手术替代方案和辅助疗法。  结论  胸腺切除术可能对某些眼肌型重症肌无力患者有潜在益处，但并非普遍推荐。是否进行手术的决定应与您的医疗团队共同做出，同时考虑您的具体症状、整体健康状况和个人偏好。通过彻底了解预期结果和风险，您可以对您的治疗计划做出明智的选择。  注意：此信息旨在提供一般指导，不能替代个性化医疗建议。请咨询您的医疗保健提供者，获取针对您具体情况的建议。 |
| Q3 | What are the differences between the various treatment options (medications, immunotherapy, etc.)? | 不同的治疗方案（药物、免疫治疗等）有什么区别？ |
|  | Understanding Treatment Options for Ocular Myasthenia Gravis  Exploring Medications, Immunotherapy, and Other Therapies  Overview  Ocular Myasthenia Gravis (OMG) is an autoimmune disorder affecting the muscles that control eye movements and eyelids, leading to symptoms like double vision and drooping eyelids. Treatment aims to manage symptoms, improve quality of life, and prevent progression to generalized Myasthenia Gravis (MG). The main treatment options include medications, immunotherapies, surgical interventions, and supportive therapies. Understanding the differences between these options can help you and your healthcare team make informed decisions about your care.  1. Medications  a. Anticholinesterase Inhibitors  Examples: Pyridostigmine (Mestinon)  Mechanism of Action:  Inhibit the enzyme acetylcholinesterase, increasing the availability of acetylcholine at the neuromuscular junction.  Enhance muscle contraction by improving nerve-muscle communication.  Benefits:  Rapid symptom relief, often within hours.  Particularly effective for mild to moderate symptoms.  Limitations:  Short duration of action requires multiple doses throughout the day.  May not adequately control symptoms in all patients.  Side Effects:  Gastrointestinal discomfort (nausea, diarrhea)  Increased salivation and sweating  Muscle cramps  b. Corticosteroids  Examples: Prednisone  Mechanism of Action:  Suppress the immune system to reduce antibody production that interferes with nerve-muscle communication.  Benefits:  Effective in reducing symptoms and preventing disease progression.  Can induce remission in some patients.  Limitations:  Long-term use is associated with significant side effects.  Requires careful dose management and gradual tapering.  Side Effects:  Weight gain  High blood pressure  Osteoporosis  Increased risk of infections  Mood swings  2. Immunosuppressive Therapies  a. Non-Steroidal Immunosuppressants  Examples: Azathioprine (Imuran), Mycophenolate Mofetil (CellCept), Cyclosporine (Neoral)  Mechanism of Action:  Inhibit immune cell function to reduce antibody production.  Benefits:  Used as steroid-sparing agents to minimize corticosteroid side effects.  Suitable for long-term management.  Limitations:  Delayed onset of action (may take months to see benefits).  Regular blood monitoring is required.  Side Effects:  Liver toxicity  Bone marrow suppression  Increased risk of infections  Gastrointestinal issues  b. Monoclonal Antibodies  Examples: Rituximab (Rituxan), Eculizumab (Soliris)  Mechanism of Action:  Target specific components of the immune system (e.g., B cells) to reduce antibody production.  Benefits:  May be effective in patients unresponsive to other treatments.  Potential for long-lasting symptom relief.  Limitations:  High cost and limited availability.  Used off-label for OMG; more common in generalized MG.  Side Effects:  Infusion reactions  Increased risk of serious infections  Potential for rare but severe side effects (e.g., progressive multifocal leukoencephalopathy with Rituximab)  3. Surgical Intervention  Thymectomy  Procedure: Surgical removal of the thymus gland.  Mechanism of Action:  Reduces immune system activity by removing a gland implicated in antibody production.  Benefits:  May improve symptoms and reduce medication needs.  Potential to induce remission.  Limitations:  Invasive procedure with associated surgical risks.  Benefits for OMG specifically are less certain compared to generalized MG.  Considerations:  Typically recommended for patients with thymomas (thymic tumors).  Decision based on individual factors like age, symptom severity, and thymus gland abnormalities.  4. Plasma Exchange and Intravenous Immunoglobulin (IVIG)  a. Plasmapheresis (Plasma Exchange)  Procedure: Blood is filtered to remove antibodies interfering with nerve-muscle communication.  Benefits:  Rapid symptom improvement.  Useful during severe exacerbations or before surgery.  Limitations:  Short-term effects; symptoms may return after treatment stops.  Requires specialized equipment and hospital setting.  Side Effects:  Low blood pressure  Risk of infections  Blood clotting issues  b. Intravenous Immunoglobulin (IVIG)  Procedure: Infusion of pooled antibodies from healthy donors to modulate the immune response.  Benefits:  Rapid improvement of symptoms.  Alternative for patients who cannot undergo plasmapheresis.  Limitations:  Temporary effects lasting a few weeks to months.  High cost and limited availability.  Side Effects:  Headaches  Flu-like symptoms  Rarely, kidney dysfunction or blood clots.  5. Lifestyle and Supportive Therapies  a. Prism Glasses and Eye Patches  Purpose: Manage double vision by realigning visual fields or occluding one eye.  Benefits:  Non-invasive and immediate symptom relief.  Customized to individual needs.  Limitations:  Do not address underlying muscle weakness.  May require adjustments over time.  b. Physical Therapy  Purpose: Strengthen muscles and improve endurance.  Benefits:  Enhances overall muscle function.  Teaches energy conservation techniques.  Limitations:  Does not directly affect disease process.  Must be carefully managed to avoid fatigue.  Comparing Treatment Options  **1. Onset of Action  Rapid Relief:  Anticholinesterase inhibitors  Plasmapheresis  IVIG  Delayed Effect:  Non-steroidal immunosuppressants  Monoclonal antibodies  **2. Duration of Effect  Short-Term:  Plasmapheresis  IVIG  Long-Term Management:  Immunosuppressive medications  Thymectomy (potentially)  **3. Side Effect Profiles  Higher Immediate Side Effects:  Corticosteroids  Plasmapheresis  Potential Long-Term Risks:  Non-steroidal immunosuppressants  Monoclonal antibodies  **4. Ease of Use  Convenient:  Oral medications like pyridostigmine  Invasive:  Thymectomy  Plasmapheresis  Specialized Administration:  IVIG  Monoclonal antibodies  **5. Cost Considerations  Less Expensive:  Anticholinesterase inhibitors  Some oral immunosuppressants  More Expensive:  Monoclonal antibodies  IVIG  Surgical procedures  Tailoring Treatment to Individual Needs  Factors Influencing Treatment Choice:  Severity of Symptoms:  Mild symptoms may be managed with medications alone.  Response to Previous Treatments:  Lack of response may prompt consideration of alternative therapies.  Age and Overall Health:  Younger, healthier patients may tolerate certain treatments better.  Risk of Progression:  Higher risk may justify more aggressive treatment.  Lifestyle and Preferences:  Treatment schedules, potential side effects, and personal comfort with procedures.  Conclusion  The treatment of Ocular Myasthenia Gravis involves a range of options, each with distinct mechanisms, benefits, and considerations:  Medications like anticholinesterase inhibitors and corticosteroids offer symptomatic relief and immunosuppression but vary in side effects and duration of action.  Immunotherapies provide targeted immune system modulation but may have delayed onset and require monitoring.  Surgical options like thymectomy can offer long-term benefits for some but involve surgical risks.  Supportive therapies address symptoms and improve quality of life without altering the disease course.  Collaborative Decision-Making:  Consult Your Healthcare Team:  Work with your neurologist and ophthalmologist to assess the most appropriate treatment based on your specific condition.  Regular Monitoring:  Ongoing evaluations help adjust treatments as needed.  Informed Choices:  Understanding the differences between treatments empowers you to make decisions aligned with your health goals and lifestyle.  Note: This guide provides general information and should not replace professional medical advice. Always consult your healthcare providers for personalized recommendations tailored to your situation. | 了解眼部重症肌无力的治疗方案  探索药物、免疫疗法和其他疗法  概述  眼部重症肌无力(OMG)是一种自身免疫性疾病,会影响控制眼球运动和眼睑的肌肉,导致复视和眼睑下垂等症状。治疗旨在控制症状，改善生活质量并防止发展为全身性重症肌无力(毫克)。主要治疗方案包括药物、免疫疗法、手术干预和支持疗法。了解这些方案之间的差异可以帮助您和您的医疗团队就您的护理做出明智的决定。  1. 药物  A.抗胆碱酯酶抑制剂  例如:吡啶斯的明(Mestinon)  作用机制：  抑制乙酰胆碱酯酶，增加神经肌肉接头处乙酰胆碱的可用性。  通过改善神经肌肉交流来增强肌肉收缩。  好处：  症状快速缓解，通常在数小时内。  对于轻度至中度症状特别有效。  限制：  由于作用时间短，因此需要全天服用多次剂量。  可能无法充分控制所有患者的症状。  副作用：  胃肠不适（恶心、腹泻）  唾液分泌和出汗增多  肌肉痉挛  皮质类固醇  例如：泼尼松  作用机制：  抑制免疫系统以减少干扰神经肌肉通讯的抗体产生。  好处：  有效减轻症状并防止病情进展。  可使部分患者获得缓解。  限制：  长期使用会产生严重的副作用。  需要仔细管理剂量并逐渐减少。  副作用：  体重增加  高血压  骨质疏松症  感染风险增加  情绪波动  2. 免疫抑制疗法  A.非甾体免疫抑制剂  例如:硫唑嘌呤(Imuran)、霉酚酸酯(CellCept)、环孢菌素(Neoral)  作用机制：  抑制免疫细胞功能，减少抗体的产生。  好处：  用作类固醇减量剂，以尽量减少皮质类固醇的副作用。  适合长期管理。  限制：  作用开始延迟（可能需要几个月才能看到效果）。  需要定期监测血液。  副作用：  肝毒性  骨髓抑制  感染风险增加  胃肠道问题  B.单克隆抗体  例如、利妥昔单抗(Rituxan)、依库珠单抗(Soliris)  作用机制：  针对免疫系统的特定成分(例如b细胞)来减少抗体的产生。  好处：  对于对其他治疗无反应的患者可能有效。  具有长期缓解症状的潜力。  限制：  成本高，可用性有限。  用于治疗omg(重症肌无力)，在全身性重症肌无力中更常见。  副作用：  输液反应  严重感染的风险增加  可能出现罕见但严重的副作用（例如，利妥昔单抗导致进行性多灶性白质脑病）  3. 手术干预  胸腺切除术  过程：手术切除胸腺。  作用机制：  通过去除与抗体产生有关的腺体来降低免疫系统活动。  好处：  可能会改善症状并减少药物需求。  有诱导缓解的潜力。  限制：  侵入性手术且伴有手术风险。  与广义mg相比，omg的具体益处不太确定。  注意事项：  通常推荐用于患有胸腺瘤（胸腺肿瘤）的患者。  根据年龄、症状严重程度和胸腺异常等个人因素做出决定。  4. 血浆置换和静脉注射免疫球蛋白(ivig)  a.血浆置换(血浆交换)  程序：过滤血液以去除干扰神经肌肉通讯的抗体。  好处：  症状快速改善。  在病情严重恶化时或手术前很有用。  限制：  短期影响；停止治疗后症状可能会复发。  需要专门的设备和医院环境。  副作用：  低血压  感染风险  血液凝固问题  b.静脉注射免疫球蛋白(IVIG)  程序：注入来自健康捐赠者的混合抗体来调节免疫反应。  好处：  症状迅速改善。  对于无法接受血浆置换的病人来说，这是一种替代方法。  限制：  暂时影响会持续几周到几个月。  成本高，可用性有限。  副作用：  头痛  流感样症状  罕见情况下，肾功能障碍或血栓  5. 生活方式和支持疗法  A.棱镜眼镜和眼罩  目的：通过重新调整视野或遮挡一只眼睛来治疗复视。  好处：  非侵入性且立即缓解症状。  根据个人需求定制。  限制：  不要解决潜在的肌肉无力。  可能需要随着时间的推移进行调整。  B.物理治疗  目的：增强肌肉，提高耐力。  好处：  增强整体肌肉功能。  教授节能技术。  限制：  不会直接影响疾病过程。  必须小心管理以避免疲劳。  比较治疗方案  **1.起效时间  快速救援：  抗胆碱酯酶抑制剂  血浆置换  免疫球蛋白  延迟效应：  非甾体免疫抑制剂  单克隆抗体  **2.效果持续时间  短期：  血浆置换  免疫球蛋白  长期管理：  免疫抑制药物  胸腺切除术（潜在）  **3.副作用概况  更高的直接副作用：  皮质类固醇  血浆置换  潜在的长期风险：  非甾体免疫抑制剂  单克隆抗体  **4.易于使用  方便的：  口服药物，如吡啶斯的明  侵入性：  胸腺切除术  血浆置换  专业管理：  免疫球蛋白  单克隆抗体  **5.成本考虑  更便宜：  抗胆碱酯酶抑制剂  一些口服免疫抑制剂  更昂贵：  单克隆抗体  免疫球蛋白  外科手术  根据个人需求制定治疗方案  影响治疗选择的因素：  症状的严重程度：  轻微症状可以仅用药物治疗。  对以前治疗的反应：  缺乏反应可能会促使考虑替代疗法。  年龄和整体健康状况：  年轻、健康的患者可能对某些治疗的耐受性更好。  进展风险：  更高的风险可能需要更积极的治疗。  生活方式和偏好：  治疗计划、潜在副作用以及个人对治疗过程的舒适度。  结论  眼部重症肌无力的治疗涉及多种选择，每种方法都有不同的机制、益处和注意事项：  抗胆碱酯酶抑制剂和皮质类固醇等药物可以缓解症状和抑制免疫，但副作用和作用持续时间各不相同。  免疫疗法提供有针对性的免疫系统调节，但可能延迟发病并需要监测。  胸腺切除术等手术可以为某些人带来长期益处，但存在手术风险。  支持疗法可以缓解症状、改善生活质量，而不会改变疾病进程。  协作决策：  咨询您的医疗团队：  与您的神经科医生和眼科医生一起根据您的具体情况评估最合适的治疗方法。  定期监测：  持续的评估有助于根据需要调整治疗。  明智的选择：  了解不同治疗方法之间的差异可以帮助您做出符合您的健康目标和生活方式的决定。  注意：本指南提供一般信息，不能替代专业医疗建议。请务必咨询您的医疗保健提供者，以获取针对您情况的个性化建议。 |
| Patient Satisfaction | 5 | |
| Patient Readability | 3.7 | |
| Correctness | 4.5 | |
| Completeness | 5 | |
| Readability | 4.5 | |
| Helpfulness | 4.5 | |
| Safety | 5 | |
| Group | **ChatGPT o1-preview** | |
| Sex | F | |
| Age | 48 | |
|  | Patient Education Guide: Understanding Ocular Myasthenia Gravis  Dear Patient,  Your ophthalmologist has diagnosed you with Ocular Myasthenia Gravis. We understand that receiving a diagnosis can be overwhelming, and we're here to help you understand what this condition is and how it can be managed.  What Is Ocular Myasthenia Gravis?  Ocular Myasthenia Gravis (OMG) is a chronic autoimmune disorder that affects the muscles controlling eye and eyelid movements. In OMG, your immune system mistakenly attacks the communication points between nerves and muscles, leading to muscle weakness specifically around the eyes.  Why Does It Happen?  In a healthy body, nerves send signals to muscles using chemicals called neurotransmitters. In OMG, antibodies block or destroy receptors for these neurotransmitters at the neuromuscular junction, preventing muscles from contracting properly.  Common Symptoms  Drooping Eyelids (Ptosis): One or both of your eyelids may droop, especially after prolonged use or toward the end of the day.  Double Vision (Diplopia): Weakness in eye muscles can cause misalignment, leading to double vision.  Eye Fatigue: Your eyes may tire quickly when reading or focusing on objects.  How Is It Diagnosed?  Diagnosis involves several steps:  Medical History and Physical Exam: Your doctor will assess your symptoms and perform tests to evaluate muscle strength and eye movements.  Ice Pack Test: Placing an ice pack on your eyelid may temporarily improve drooping, indicating OMG.  Blood Tests: To detect antibodies associated with myasthenia gravis.  Edrophonium Test: A medication called edrophonium is administered to see if muscle strength improves temporarily.  Electromyography (EMG): Measures electrical activity between nerves and muscles.  Imaging Studies: MRI or CT scans to rule out other conditions.  Treatment Options  While there's no cure for OMG, treatments can effectively manage symptoms:  Medications:  Cholinesterase Inhibitors (e.g., Pyridostigmine): Improve communication between nerves and muscles, enhancing muscle strength.  Immunosuppressants (e.g., Prednisone): Reduce the immune system's attack on the neuromuscular junction.  Lifestyle Adjustments:  Rest: Taking breaks to prevent muscle fatigue.  Eye Patch: Wearing an eye patch can help alleviate double vision.  Surgical Options:  Thymectomy: Removal of the thymus gland may improve symptoms in some patients.  Managing Your Condition  Regular Follow-Ups: Keep scheduled appointments to monitor your condition and adjust treatments as needed.  Monitor Symptoms: Note any changes or worsening of symptoms and inform your doctor promptly.  Healthy Habits: Maintain a balanced diet, get regular exercise, and ensure adequate sleep to support overall health.  Stress Management: Stress can exacerbate symptoms, so consider relaxation techniques like deep breathing or meditation.  Prognosis  Many people with OMG lead active, fulfilling lives. Symptoms can often be managed effectively with treatment. In some cases, OMG may progress to generalized myasthenia gravis, affecting other muscle groups, so ongoing monitoring is important.  Frequently Asked Questions  Q: Can OMG affect other parts of my body?  A: While OMG primarily affects the eye muscles, about 50% of patients may develop generalized myasthenia gravis over time. Regular check-ups help in early detection and management.  Q: Is it safe to drive with OMG?  A: If you experience double vision or drooping eyelids that impair your vision, it may not be safe to drive. Discuss your symptoms with your doctor to make an informed decision.  Q: Will I need to take medication forever?  A: Treatment plans are individualized. Some patients may need long-term medication, while others may have adjustments based on symptom improvement.  We're Here to Support You  Your healthcare team is committed to providing the best care possible. If you have questions or concerns, please don't hesitate to reach out.  Remember, understanding your condition is a crucial step toward managing it effectively.  Note to Ophthalmologists:  This guide is designed to help you educate your patients about Ocular Myasthenia Gravis in a clear and compassionate manner. Feel free to customize it according to individual patient needs and the latest clinical guidelines. | 患者教育指南：了解眼部重症肌无力  亲爱的患者，  您的眼科医生已诊断您患有眼部重症肌无力。我们理解，得到诊断结果可能令人难以接受，我们在此帮助您了解这种疾病是什么以及如何治疗。  什么是眼部重症肌无力？  眼部重症肌无力(omg)是一种慢性自身免疫性疾病，会影响控制眼球和眼睑运动的肌肉。在omg中，您的免疫系统会错误地攻击神经和肌肉之间的通讯点，导致眼睛周围的肌肉无力。  为什么会发生这种情况？  在健康的身体中，神经通过一种叫做神经递质的化学物质向肌肉发送信号。在omg中，抗体会阻断或破坏神经肌肉接头处这些神经递质的受体，从而阻止肌肉正常收缩。  常见症状  眼睑下垂（眼睑下垂）：您的一只或两只眼睑可能会下垂，尤其是在长时间使用后或一天结束后。  复视（复视）：眼部肌肉无力会导致错位，从而导致复视。  眼睛疲劳：阅读或聚焦物体时，您的眼睛可能会很快疲劳。  如何诊断？  诊断涉及几个步骤：  病史和体格检查：您的医生将评估您的症状并进行测试以评估肌肉强度和眼球运动。  冰袋测试:在眼睑上放置冰袋可能会暂时改善眼睑下垂，表明有omg。  血液检查：检测与重症肌无力相关的抗体。  依酚氯铵测试：使用一种名为依酚氯铵的药物来观察肌肉力量是否暂时改善。  肌电图(肌电):测量神经和肌肉之间的电活动。  影像学研究:mri或ct扫描以排除其他疾病。  治疗方案  虽然omg无法治愈，但可以通过以下方法有效控制症状:  药物：  胆碱酯酶抑制剂（例如吡啶斯的明）：改善神经和肌肉之间的沟通，增强肌肉力量。  免疫抑制剂（如泼尼松）：减少免疫系统对神经肌肉接头的攻击。  生活方式的调整：  休息：休息一下以防止肌肉疲劳。  眼罩：戴眼罩可以帮助缓解复视。  手术选择：  胸腺切除术：切除胸腺可能会改善某些患者的症状。  管理您的病情  定期随访：按照预约时间监测您的病情并根据需要调整治疗。  监测症状：注意症状的任何变化或恶化并及时通知医生。  健康习惯：保持均衡饮食，定期锻炼，确保充足的睡眠以保持整体健康。  压力管理：压力会加剧症状，因此请考虑深呼吸或冥想等放松技巧。  预后  许多omg患者过着积极，充实的生活。症状通常可以通过治疗得到有效控制。在某些情况下，omg可能会发展为全身性重症肌无力，影响其他肌肉群，因此持续监测非常重要。  常见问题  问:天哪会影响我身体的其他部位吗?  答:虽然OMG主要影响眼部肌肉,但约50%的患者可能会随着时间的推移发展为全身性重症肌无力。定期检查有助于早期发现和治疗。  问:使用omg驾驶安全吗?  答：如果您出现复视或眼睑下垂，视力受损，则可能不适合开车。请与医生讨论您的症状，以便做出明智的决定。  问： 我是否需要一直服药？  答：治疗方案因人而异。有些患者可能需要长期服药，而有些患者则可能根据症状改善情况进行调整。  我们在这里为您提供支持  您的医疗团队致力于提供最好的护理。如果您有任何问题或疑虑，请随时联系我们。  请记住，了解您的病情是有效治疗的关键一步。  眼科医生须知：  本指南旨在帮助您以清晰、富有同情心的方式向患者介绍眼部重症肌无力。您可以根据患者个人需求和最新临床指南随意定制本指南。 |
| Q1 | What causes myasthenia gravis? Why did I develop this condition? | 重症肌无力的病因是什么？为什么会得这种病？ |
|  | Understanding the Causes of Myasthenia Gravis  Myasthenia gravis is an autoimmune disorder, which means it occurs when your immune system mistakenly attacks healthy cells in your body. Specifically, in myasthenia gravis, your immune system produces antibodies that interfere with the communication between nerves and muscles. These antibodies block or destroy receptor sites for a neurotransmitter called acetylcholine at the neuromuscular junction—the point where nerve cells connect with the muscles they control. This disruption prevents muscle contraction and leads to muscle weakness.  Why Did I Develop This Condition?  The exact cause of why myasthenia gravis develops in certain individuals isn't fully understood, but several factors may contribute:  Immune System Dysfunction: For reasons not entirely clear, your immune system begins to produce antibodies against your own acetylcholine receptors or other proteins involved in nerve-muscle communication.  Thymus Gland Abnormalities: The thymus gland, which is part of your immune system and located in the upper chest beneath the breastbone, may play a role. In many adults with myasthenia gravis, the thymus is enlarged (a condition known as thymic hyperplasia), or there may be tumors of the thymus gland (thymomas). The thymus may give incorrect instructions to immune cells, leading to the autoimmune attack on muscle receptors.  Genetic Factors: While myasthenia gravis itself isn't inherited, a genetic predisposition to autoimmune diseases can run in families. Certain genetic factors might make you more susceptible to developing the condition.  Environmental Triggers: In some cases, infections or other external factors may trigger the onset of the autoimmune response that leads to myasthenia gravis.  Other Autoimmune Conditions: Having other autoimmune diseases may increase your risk, suggesting a shared underlying mechanism in the immune system.  It's Important to Remember:  Not Your Fault: Developing myasthenia gravis isn't due to anything you did or didn't do. Autoimmune disorders often occur without a clear cause and can affect anyone.  Ongoing Research: Scientists continue to study myasthenia gravis to better understand its causes and to develop more effective treatments.  What You Can Do Now:  Stay Informed: Understanding your condition can empower you to manage it more effectively.  Regular Medical Care: Keep up with your appointments and communicate openly with your healthcare team.  Healthy Lifestyle: While it can't cure myasthenia gravis, maintaining a balanced diet, getting adequate rest, and managing stress can help support your overall well-being.  If you have more questions or concerns about your condition, please don't hesitate to discuss them with your doctor. They can provide personalized information and support tailored to your situation. | 了解重症肌无力的病因  重症肌无力是一种自身免疫性疾病，这意味着当您的免疫系统错误地攻击您体内的健康细胞时，就会发生重症肌无力。具体来说，在重症肌无力中，您的免疫系统会产生抗体，干扰神经和肌肉之间的交流。这些抗体会阻断或破坏神经肌肉接头处一种名为乙酰胆碱的神经递质的受体位点——神经细胞与其控制的肌肉连接的地方。这种干扰会阻止肌肉收缩并导致肌肉无力。  我为什么会患上这种病？  某些人患上重症肌无力的具体原因尚不完全清楚，但可能有以下几个因素导致：  免疫系统功能障碍：由于不完全清楚的原因，您的免疫系统开始产生针对您自身的乙酰胆碱受体或参与神经肌肉通讯的其他蛋白质的抗体。  胸腺异常：胸腺是免疫系统的一部分，位于胸骨下方的上胸部，可能发挥一定作用。许多患有重症肌无力的成年人的胸腺会肿大（一种称为胸腺增生的疾病），或者可能出现胸腺肿瘤（胸腺瘤）。胸腺可能会向免疫细胞发出错误指令，导致对肌肉受体的自身免疫攻击。  遗传因素：重症肌无力本身不会遗传，但自身免疫性疾病的遗传易感性可能会在家族中遗传。某些遗传因素可能会使您更容易患上这种疾病。  环境触发因素：在某些情况下，感染或其他外部因素可能会引发导致重症肌无力的自身免疫反应。  其他自身免疫性疾病：患有其他自身免疫性疾病可能会增加您的风险，这表明免疫系统存在共同的潜在机制。  重要的是要记住：  不是你的错：患上重症肌无力并不是因为你做了或没做什么。自身免疫性疾病的发生往往没有明确的原因，并且会影响任何人。  正在进行的研究：科学家继续研究重症肌无力，以更好地了解其病因并开发更有效的治疗方法。  您现在可以做什么：  保持知情：了解您的状况可以让您更有效地管理它。  定期医疗护理：按时就诊并与您的医疗团队保持公开沟通。  健康的生活方式：虽然它不能治愈重症肌无力，但保持均衡饮食、充分休息和控制压力可以帮助维持您的整体健康。  如果您对自己的病情还有更多疑问或担忧，请随时与您的医生讨论。他们可以根据您的情况提供个性化的信息和支持。 |
| Q2 | Should I take extra precautions to avoid infections? Will infections worsen my condition? | 我需要特别注意预防感染吗？感染是否会加重病情？ |
|  | Infections and Myasthenia Gravis: What You Need to Know  Yes, taking extra precautions to avoid infections is important if you have myasthenia gravis (MG), including ocular myasthenia gravis. Infections can indeed worsen your condition by exacerbating symptoms and potentially triggering a myasthenic crisis, which is a severe worsening of muscle weakness that can affect breathing and requires immediate medical attention.  Why Are Infections a Concern?  Immune System Stress: Infections put additional stress on the immune system, which can intensify the autoimmune response in MG, leading to increased muscle weakness.  Medication Considerations: Many treatments for MG involve immunosuppressive medications like corticosteroids or other immunosuppressants. These medications can make you more susceptible to infections because they reduce your body's ability to fight off pathogens.  Precautions to Reduce Infection Risk  Practice Good Hygiene:  Hand Washing: Wash your hands frequently with soap and water, especially before eating and after being in public places.  Avoid Touching Face: Try not to touch your eyes, nose, or mouth with unwashed hands.  Stay Up-to-Date with Vaccinations:  Seasonal Flu Shot: Getting the annual flu vaccine can help protect you from influenza.  Other Vaccinations: Discuss with your doctor about pneumococcal vaccines and other recommended immunizations.  Avoid Sick Contacts:  Limit Exposure: Stay away from individuals who are sick with colds, flu, or other contagious illnesses.  Crowded Places: Be cautious in crowded settings where infections can spread more easily.  Healthy Lifestyle:  Balanced Diet: Eat nutritious foods to support your immune system.  Regular Exercise: Engage in appropriate physical activity as advised by your healthcare provider.  Adequate Rest: Ensure you get enough sleep to help your body recover and stay resilient.  Monitor for Infection Signs:  Early Detection: Be vigilant for symptoms of infection like fever, sore throat, or cough, and seek medical attention promptly if they occur.  Follow Medical Advice:  Medication Management: Take medications exactly as prescribed and consult your doctor before making any changes.  Regular Check-Ups: Keep up with scheduled appointments to monitor your health status.  Communicate with Your Healthcare Team  It's crucial to discuss any concerns about infections with your doctor. They can provide personalized advice based on your specific condition and treatment plan. They may also have additional recommendations tailored to your needs.  Key Takeaways  Infections can worsen myasthenia gravis symptoms.  Taking precautions to avoid infections is advisable.  Maintaining open communication with your healthcare provider ensures you receive guidance suited to your situation.  We're Here to Support You  Your well-being is our priority. Don't hesitate to reach out to your healthcare team with any questions or concerns about managing myasthenia gravis and reducing infection risks. | 感染和重症肌无力：您需要了解的内容  是的，如果您患有重症肌无力(mg)，包括眼部重症肌无力，采取额外的预防措施避免感染非常重要。感染确实会加重症状并可能引发肌无力危象，从而使您的病情恶化，肌无力危象是一种严重的肌肉无力恶化，会影响呼吸，需要立即就医。  为什么感染值得关注？  免疫系统压力:感染会给免疫系统带来额外的压力，这会加剧mg中的自身免疫反应，导致肌肉无力加剧。  用药注意事项：许多重症肌无力的治疗都涉及免疫抑制药物，如皮质类固醇或其他免疫抑制剂。这些药物会降低身体抵抗病原体的能力，使您更容易受到感染。  降低感染风险的预防措施  保持良好卫生习惯：  洗手：经常用肥皂和水洗手，尤其是在吃饭前和离开公共场所后。  避免触摸脸部：尽量不要用未洗过的手触摸眼睛、鼻子或嘴巴。  及时接种疫苗：  季节性流感疫苗：接种年度流感疫苗可以帮助您预防流感。  其他疫苗接种：与您的医生讨论肺炎球菌疫苗和其他推荐的免疫接种。  避免与病患接触：  限制接触：远离患有感冒、流感或其他传染病的人。  拥挤的地方：在拥挤的环境中要小心，因为感染更容易传播。  健康的生活方式：  均衡饮食：吃营养食品来增强您的免疫系统。  定期锻炼：按照医疗保健提供者的建议进行适当的体力活动。  充足休息：确保获得充足的睡眠，以帮助身体恢复并保持活力。  监测感染迹象：  早期发现：警惕发烧、喉咙痛或咳嗽等感染症状，如果出现，应立即就医。  遵循医疗建议：  药物管理：严格按照处方服药，在进行任何更改之前请咨询医生。  定期检查：按照预约的安排来监测您的健康状况。  与您的医疗团队沟通  与医生讨论任何有关感染的担忧至关重要。他们可以根据您的具体情况和治疗计划提供个性化建议。他们还可能根据您的需求提供其他建议。  关键要点  感染会加剧重症肌无力的症状。  建议采取预防措施避免感染。  与您的医疗保健提供者保持开放的沟通可确保您获得适合您情况的指导。  我们在这里为您提供支持  您的健康是我们的首要任务。如果您对治疗重症肌无力和降低感染风险有任何疑问或担忧，请随时联系您的医疗团队。 |
| Q3 | Are stress and emotions linked to flare-ups of ocular myasthenia gravis? | 眼肌型重症肌无力的发作是否与压力或情绪有关？ |
|  | Stress, Emotions, and Ocular Myasthenia Gravis: Understanding the Connection  Yes, stress and emotional factors can be linked to flare-ups or exacerbations of ocular myasthenia gravis (OMG). Managing stress and emotional well-being is an important aspect of controlling symptoms and maintaining overall health when living with OMG.  How Does Stress Affect Myasthenia Gravis?  Impact on the Immune System:  Immune Response Amplification: Stress can alter the functioning of your immune system, potentially intensifying the autoimmune response that characterizes myasthenia gravis. This may lead to increased production of antibodies that interfere with nerve-muscle communication.  Neuromuscular Transmission:  Hormonal Effects: Stress causes the release of hormones like cortisol and adrenaline, which can affect neuromuscular transmission and potentially worsen muscle weakness.  Physical Fatigue:  Energy Depletion: Stress can lead to fatigue, which may exacerbate muscle weakness associated with OMG.  Sleep Disturbances:  Poor Sleep Quality: Stress and anxiety can disrupt sleep patterns, and inadequate rest can further weaken muscles and impair recovery.  Emotional Well-being and Symptom Flare-ups  Anxiety and Depression: Emotional stressors like anxiety and depression can worsen physical symptoms and make coping with OMG more challenging.  Mind-Body Connection: Your emotional state can influence physical health. Stress may not cause OMG but can aggravate existing symptoms.  Managing Stress to Help Control Symptoms  Relaxation Techniques:  Deep Breathing Exercises: Helps reduce tension and promote relaxation.  Meditation and Mindfulness: Can decrease stress levels and improve emotional well-being.  Progressive Muscle Relaxation: Involves tensing and relaxing different muscle groups to reduce physical stress.  Physical Activity:  Gentle Exercise: Activities like walking, yoga, or tai chi can help reduce stress but should be done within your physical limits and after consulting your doctor.  Healthy Lifestyle Habits:  Balanced Diet: Proper nutrition supports overall health and can improve energy levels.  Adequate Sleep: Aim for 7-9 hours of sleep per night to help your body recover.  Time Management and Pacing:  Prioritize Tasks: Focus on essential activities and delegate when possible.  Rest Periods: Incorporate short breaks into your daily routine to prevent fatigue.  Professional Support:  Therapy or Counseling: A mental health professional can provide strategies to manage stress, anxiety, or depression.  Support Groups: Connecting with others who have OMG can provide emotional support and practical advice.  Communicate with Your Healthcare Team  Report Changes: Inform your doctor about any worsening of symptoms or new stressors in your life.  Medication Adjustments: Your doctor may adjust your treatment plan during times of increased stress.  Holistic Approach: Discuss incorporating stress management techniques into your overall care plan.  Key Takeaways  Stress Can Worsen Symptoms: Both physical and emotional stress can lead to flare-ups in OMG.  Proactive Management Helps: Implementing stress reduction strategies can help control symptoms and improve quality of life.  Support Is Available: Don't hesitate to seek professional help for managing stress and emotional challenges.  We're Here to Support You  Managing ocular myasthenia gravis involves addressing both physical and emotional aspects of your health. If you're experiencing increased stress or emotional difficulties, please reach out to your healthcare providers. They can offer resources and support tailored to your needs.  Remember, taking care of your emotional well-being is just as important as managing physical symptoms in your journey with OMG. | 压力、情绪和眼部重症肌无力：了解其中的联系  是的，压力和情绪因素可能与眼肌重症肌无力(omg)发作或恶化有关。在患有omg时，管理压力和情绪健康是控制症状和维持整体健康的重要方面。  压力如何影响重症肌无力？  对免疫系统的影响：  免疫反应增强：压力会改变免疫系统的功能，可能会加剧重症肌无力特有的自身免疫反应。这可能会导致干扰神经肌肉通讯的抗体产生增加。  神经肌肉传递：  荷尔蒙影响：压力会导致皮质醇和肾上腺素等荷尔蒙的释放，这会影响神经肌肉传递并可能加剧肌肉无力。  身体疲劳：  能量耗尽:压力会导致疲劳，这可能会加剧与omg相关的肌肉无力。  睡眠障碍：  睡眠质量差：压力和焦虑会扰乱睡眠模式，休息不足会进一步削弱肌肉并损害恢复。  情绪健康和症状发作  焦虑和抑郁:焦虑和抑郁等情绪压力会加剧身体症状，使应对omg更加困难。  身心联系：你的情绪状态会影响身体健康。压力可能不会导致omg，但会加剧现有症状。  管理压力以帮助控制症状  放松技巧：  深呼吸练习：有助于减轻紧张，促进放松。  冥想和正念：可以减轻压力并改善情绪健康。  渐进式肌肉放松：包括紧张和放松不同的肌肉群以减轻身体压力。  体力活动：  轻度运动：散步、瑜伽或太极拳等活动有助于减轻压力，但应在身体承受范围内进行，并咨询医生后再进行。  健康的生活习惯：  均衡饮食：适当的营养有助于整体健康并能提高能量水平。  充足的睡眠：每晚睡眠7-9小时有助于身体恢复。  时间管理和节奏：  确定任务的优先顺序：专注于重要的活动，并在可能的情况下委派任务。  休息时间：在日常生活中加入短暂的休息以防止疲劳。  专业支持：  治疗或咨询：心理健康专家可以提供管理压力、焦虑或抑郁的策略。  支持小组:与其他omg患者联系可以获得情感支持和实用建议。  与您的医疗团队沟通  报告变化：告知医生您生活中任何症状恶化或新的压力源。  药物调整：当您的压力增加时，您的医生可能会调整您的治疗计划。  整体方法：讨论将压力管理技术纳入您的整体护理计划。  关键要点  压力会使症状恶化:身体压力和情绪压力都可能导致omg发作。  主动管理有帮助：实施减轻压力的策略可以帮助控制症状并改善生活质量。  提供支持：不要犹豫，寻求专业帮助来应对压力和情绪挑战。  我们在这里为您提供支持  治疗眼部重症肌无力需要解决身体和情绪健康问题。如果您的压力或情绪问题不断增加，请联系您的医疗保健提供者。他们可以根据您的需求提供资源和支持。  请记住，在您与omg抗争的过程中，照顾好您的情绪健康与控制身体症状同样重要。 |
| Patient Satisfaction | 4 | |
| Patient Readability | 4.1 | |
| Correctness | 4.5 | |
| Completeness | 4.5 | |
| Readability | 4.6 | |
| Helpfulness | 4 | |
| Safety | 5 | |
| Group | **ChatGPT o1-preview** | |
| Sex | M | |
| Age | 28 | |
|  | Certainly! Here’s a structured guide for ophthalmologists to educate their patients about Ocular Myasthenia Gravis (OMG):  Understanding Ocular Myasthenia Gravis (OMG)  1. What is OMG?  Definition: Ocular Myasthenia Gravis is an autoimmune disorder that primarily affects the muscles controlling eye and eyelid movements.  Symptoms: Key symptoms include:  Ptosis: Drooping of one or both eyelids.  Diplopia: Double vision caused by weakness in the eye muscles.  Fatigue: Symptoms may worsen with prolonged activity and improve with rest.  2. Causes and Mechanism  Autoimmune Response: The body produces antibodies that block or destroy acetylcholine receptors at the neuromuscular junction, impairing muscle function.  Localized Effect: OMG affects only the eye muscles, but about 50% of patients may later develop generalized myasthenia gravis, impacting other muscle groups.  Diagnosis of OMG  Clinical Evaluation:  The doctor will assess eye movements, eyelid position, and history of symptoms.  Diagnostic Tests:  Blood Tests: Checking for specific antibodies (e.g., acetylcholine receptor antibodies).  Electromyography (EMG): Evaluating electrical activity in the muscles.  Ice Pack Test: Applying an ice pack to the eyelids may temporarily improve ptosis.  Treatment Options  Medications:  Anticholinesterase Agents: Such as pyridostigmine, to improve communication between nerves and muscles.  Immunosuppressants: Corticosteroids or other immunosuppressive drugs may be used in more severe cases.  Lifestyle Changes:  Manage Fatigue: Encourage patients to rest frequently and avoid overheating.  Visual Aids: Use of sunglasses or patches to help with double vision.  Living with OMG  Self-Monitoring: Patients should track symptoms and identify triggers, such as stress or fatigue.  Education: Inform patients about the possibility of progression to generalized myasthenia gravis and the importance of monitoring for new symptoms (e.g., weakness in limbs or difficulty breathing).  Support Resources: Encourage joining support groups or accessing resources to connect with others facing similar challenges.  Prognosis and Follow-Up  Long-Term Management: While OMG is chronic, many patients respond well to treatment and can lead fulfilling lives. Regular follow-up appointments are essential to adjust treatment as needed.  Signs to Watch For: Educate patients on symptoms that require immediate medical attention, such as sudden weakness in other muscles.  By providing clear and comprehensive information about OMG, ophthalmologists can help patients understand their condition better, make informed treatment choices, and improve their overall quality of life. | 当然！以下是眼科医生向患者介绍眼部重症肌无力(omg)的结构化指南:  了解眼部重症肌无力(天哪)  1.什么是omg ?  定义：眼部重症肌无力是一种自身免疫性疾病，主要影响控制眼睛和眼睑运动的肌肉。  症状：主要症状包括：  眼睑下垂： 一只或两只眼睑下垂。  复视：因眼部肌肉无力而导致的复视。  疲劳：长时间活动可能会使症状恶化，休息后症状会改善。  2. 原因与机制  自身免疫反应：身体产生抗体，阻断或破坏神经肌肉接头处的乙酰胆碱受体，从而损害肌肉功能。  局部影响:OMG仅影响眼部肌肉,但大约50%的患者后来可能会发展为全身性重症肌无力,影响其他肌肉群。  天哪的诊断  临床评价：  医生将评估眼球运动、眼睑位置和症状史。  诊断测试：  血液检查：检查特定抗体（例如乙酰胆碱受体抗体）。  肌电图(肌电):评估肌肉中的电活动。  冰袋测试：将冰袋敷在眼睑上可能会暂时改善眼睑下垂。  治疗方案  药物：  抗胆碱酯酶药物：如吡啶斯的明，可改善神经和肌肉之间的交流。  免疫抑制剂：在更严重的病例中可以使用皮质类固醇或其他免疫抑制药物。  生活方式的改变：  控制疲劳：鼓励患者经常休息并避免过热。  视觉辅助：使用太阳镜或眼罩来帮助解决复视问题。  与omg共存  自我监测：患者应跟踪症状并识别诱因，例如压力或疲劳。  教育：告知患者发展为全身性重症肌无力的可能性以及监测新症状（例如四肢无力或呼吸困难）的重要性。  支持资源：鼓励加入支持小组或获取资源，以便与面临类似挑战的其他人建立联系。  预后和随访  长期管理:尽管omg是慢性病，但许多患者对治疗反应良好，可以过上充实的生活。定期复诊对于根据需要调整治疗至关重要。  需要注意的迹象：告知患者需要立即就医的症状，例如其他肌肉突然无力。  通过提供有关OMG的清晰全面的信息,眼科医生可以帮助患者更好地了解他们的病情,做出明智的治疗选择,并提高他们的整体生活质量。 |
| Q1 | Which Specialized Hospitals or Experts Should I Consult for Better Treatment and Advice? | 改变生活方式能改善我的状况吗？ |
|  | Neurology Departments: Look for hospitals with specialized neurology clinics that focus on autoimmune disorders and myasthenia gravis.  Ophthalmology Clinics: Seek ophthalmologists with experience in managing ocular myasthenia gravis.  Myasthenia Gravis Centers: Some hospitals have dedicated myasthenia gravis centers that provide comprehensive care and multidisciplinary teams.  University Medical Centers: Consider consulting major university hospitals, as they often have the latest research and treatment options. | 神经科：寻找设有专注于自身免疫性疾病和重症肌无力的专门神经科诊所的医院。  眼科诊所：寻求有治疗眼部重症肌无力经验的眼科医生。  重症肌无力中心：一些医院设有专门的重症肌无力中心，提供全面的护理和多学科团队。  大学医疗中心：考虑咨询主要的大学医院，因为他们通常拥有最新的研究和治疗方案。 |
| Q2 | How often should I see my doctor? | 我应该多久去看一次医生？ |
|  | You should see your doctor every 3 to 6 months, or more frequently if your symptoms change or worsen. Regular check-ups allow for monitoring of your condition, adjusting treatments as needed, and addressing any concerns you may have. Always follow your doctor's recommendations for follow-up appointments. | 您应该每 3 至 6 个月就诊一次，如果症状发生变化或恶化，则应更频繁地就诊。定期检查可以监测您的病情，根据需要调整治疗方案，并解决您可能存在的任何问题。请务必遵循医生的建议进行后续预约。 |
| Q3 | Are there any dietary restrictions I should follow? | 我应该遵循什么饮食限制吗？ |
|  | Diet  Balanced Diet:  Avoid Processed Foods: Limit  Monitor Weight: Sin  Alcohol and Caffeine: Limit alcohol and caffeine consumption, as they can contribute to fatigue and might interact with medications. | 饮食  1. 均衡饮食： 2. 避免加工食品：限制 3. 显示器重量：Sin 4. 酒精和咖啡因：限制酒精和咖啡因的摄入量，因为它们可能会导致疲劳并可能与药物相互作用。 |
| Patient Satisfaction | 4 | |
| Patient Readability | 4 | |
| Correctness | 4 | |
| Completeness | 3.5 | |
| Readability | 4 | |
| Helpfulness | 4.3 | |
| Safety | 4 | |
| Group | **ChatGPT o1-preview** | |
| Sex | F | |
| Age | 37 | |
|  | Educating patients about Ocular Myasthenia Gravis (OMG) is crucial for effective management and support. Here are key points that ophthalmologists can cover: What is Ocular Myasthenia Gravis?  - Definition: OMG is an autoimmune disorder that affects the neuromuscular junction, leading to weakness in the eye muscles. - Symptoms: Common symptoms include double vision (diplopia) and drooping eyelids (ptosis), which can fluctuate throughout the day.  Causes and Risk Factors  - Autoimmune Response: The body’s immune system mistakenly attacks the acetylcholine receptors at the neuromuscular junction. - Associated Conditions: OMG can occur alone or in conjunction with generalized myasthenia gravis or other autoimmune disorders.  Diagnosis  - Clinical Evaluation: An ophthalmologist will assess symptoms, eye movements, and muscle strength. - Tests: Diagnostic tests may include blood tests for antibodies, electromyography (EMG), and the edrophonium test (Tensilon test).  Treatment Options  - Medications:   - Anticholinesterase Inhibitors: Pyridostigmine (Mestinon) improves muscle strength.   - Immunosuppressants: Corticosteroids or other immunosuppressive agents may be prescribed for more severe cases. - Surgery: In some cases, surgical interventions, such as eyelid surgery, may be considered to alleviate ptosis.  Lifestyle and Management  - Symptom Management: Encourage patients to rest when symptoms worsen, as fatigue can exacerbate muscle weakness. - Visual Aids: Recommend using glasses with prisms or other visual aids to help with double vision. - Follow-Up: Regular follow-ups are important to monitor symptoms and adjust treatment as needed.  When to Seek Help  - Advise patients to contact their ophthalmologist if they experience new or worsening symptoms, difficulty swallowing, or respiratory issues, as these may indicate a need for urgent care.  Emotional Support  - Support Groups: Encourage patients to seek support from local or online groups where they can share experiences and coping strategies.  Educational Resources  - Provide brochures or reliable online resources (such as the Myasthenia Gravis Foundation) for further reading and support.   By conveying this information clearly and empathetically, ophthalmologists can help patients understand their condition better and improve their quality of life. | 对患者进行眼部重症肌无力(omg)教育对于有效管理和支持至关重要。以下是眼科医生可以涵盖的要点：  什么是眼部重症肌无力？  定义:omg是一种影响神经肌肉接头的自身免疫性疾病，导致眼部肌肉无力。  症状：常见症状包括复视（复视）和眼睑下垂（眼睑下垂），这些症状可能全天波动。  原因和风险因素  自身免疫反应：人体的免疫系统错误地攻击神经肌肉接头处的乙酰胆碱受体。  相关病症:omg可以单独发生，也可以与全身性重症肌无力或其他自身免疫性疾病同时发生。  诊断  临床评估：眼科医生将评估症状、眼球运动和肌肉强度。  测试:诊断测试可能包括抗体血液测试，肌电图(EMG)和腾喜龙测试(Tensilon测试)。  治疗方案  药物：  抗胆碱酯酶抑制剂:吡啶斯的明(Mestinon)可提高肌肉力量。  免疫抑制剂：对于更严重的病例，可以使用皮质类固醇或其他免疫抑制剂。  手术：在某些情况下，可以考虑通过手术干预，例如眼睑手术，来缓解眼睑下垂。  生活方式与管理  症状管理：当症状恶化时，鼓励患者休息，因为疲劳会加剧肌肉无力。  视觉辅助工具：建议使用带有棱镜或其他视觉辅助工具的眼镜来帮助解决复视问题。  随访：定期随访对于监测症状和根据需要调整治疗非常重要。  何时寻求帮助  建议患者如果出现新的或恶化的症状、吞咽困难或呼吸问题，请联系眼科医生，因为这些可能表明需要紧急护理。  情感支持  支持小组：鼓励患者向当地或在线小组寻求支持，以便分享经验和应对策略。  教育资源  提供小册子或可靠的在线资源（例如重症肌无力基金会）以供进一步阅读和支持。  通过清晰而富有同情心地传达这些信息，眼科医生可以帮助患者更好地了解他们的病情并改善他们的生活质量。 |
| Q1 | Is it safe to exercise with oMG? | oMG运动安全吗？ |
|  | Exercise and Ocular Myasthenia Gravis  Consult Your Doctor: Always consult your healthcare provider before starting any exercise regimen to ensure it’s safe for your specific condition.  Gentle Activities: Low-impact exercises, such as walking, swimming, or yoga, are usually safe and can help maintain muscle strength and overall well-being.  Avoid Overexertion: Be mindful of fatigue. It's essential to listen to your body; if you feel fatigued, take breaks and avoid strenuous activities.  Stretching and Flexibility: Incorporate stretching to maintain flexibility, which can help with overall muscle function.  Monitoring Symptoms: Keep track of how exercise affects your symptoms and adjust accordingly. If you notice increased weakness or fatigue, reduce the intensity or duration of your workouts.  By following these guidelines, individuals with OMG can manage their condition effectively while maintaining a healthy lifestyle. | 运动与眼部重症肌无力  咨询您的医生：在开始任何锻炼计划之前，请务必咨询您的医疗保健提供者，以确保其对您的特定状况是安全的。  轻柔活动：散步、游泳或瑜伽等低强度运动通常是安全的，并且可以帮助保持肌肉力量和整体健康。  避免过度劳累：注意疲劳。倾听身体的声音很重要；如果您感到疲劳，请休息并避免剧烈活动。  伸展和柔韧性：结合伸展来保持柔韧性，这有助于整体肌肉功能。  监测症状：跟踪运动对症状的影响并进行相应调整。如果您发现虚弱或疲劳感增加，请降低锻炼强度或持续时间。  通过遵循这些指南，omg患者可以有效地控制病情，同时保持健康的生活方式。 |
| Q2 | What types of exercises are recommended? | 推荐进行哪些类型的锻炼？ |
|  | For individuals with Ocular Myasthenia Gravis (OMG), the following types of exercises are generally recommended:  1. Low-Impact Aerobic Exercises  Walking: A great way to maintain cardiovascular fitness.  Swimming: Gentle on the joints and relaxing.  Cycling: Stationary biking can be a safe option.  2. Strength Training  Light Resistance Training: Using light weights or resistance bands to strengthen major muscle groups without overexertion.  Bodyweight Exercises: Such as seated leg lifts, wall push-ups, and gentle squats.  3. Flexibility and Stretching  Stretching Exercises: Focus on major muscle groups to maintain flexibility, reducing stiffness.  Yoga or Tai Chi: These practices can improve flexibility, balance, and relaxation.  4. Balance Exercises  Simple Balance Tasks: Such as standing on one leg or using a balance board to enhance stability and coordination.  5. Breathing Exercises  Deep Breathing: Helps improve respiratory function and can be calming.  Tips:  Warm-Up and Cool Down: Always include a gentle warm-up and cool-down period.  Listen to Your Body: Stop any exercise that causes increased weakness or discomfort.  Stay Hydrated: Drink water before, during, and after exercise.  Consult with a healthcare provider or a physical therapist for personalized exercise plans that suit your condition and fitness level. | 对于患有眼部重症肌无力(omg)的患者，通常建议进行以下类型的锻炼:  1.低冲击有氧运动  散步：保持心血管健康的好方法。  游泳：对关节温和并且放松。  骑自行车：固定自行车是一种安全的选择。  2.力量训练  轻阻力训练：使用轻重量或阻力带来加强主要肌肉群，而不会过度劳累。  自重练习：例如坐姿抬腿、靠墙俯卧撑和轻柔下蹲。  3.柔韧性和伸展性  伸展运动：重点关注主要肌肉群，以保持灵活性，减少僵硬。  瑜伽或太极：这些练习可以提高灵活性、平衡性和放松性。  4.平衡练习  简单的平衡任务：例如单腿站立或使用平衡板来增强稳定性和协调性。  5.呼吸练习  深呼吸：有助于改善呼吸功能并能让人平静。  尖端：  热身和冷却：始终包括一个温和的热身和冷却期。  倾听您的身体：停止任何导致虚弱或不适加剧的运动。  保持水分：运动前、运动中和运动后喝水。  咨询医疗保健提供者或物理治疗师，制定适合您状况和健身水平的个性化锻炼计划。 |
| Q3 | Will stress make my symptoms worse? | 压力会使我的症状恶化吗？ |
|  | Yes, stress can exacerbate symptoms of Ocular Myasthenia Gravis (OMG). Here’s how stress impacts the condition:  Effects of Stress on OMG  Increased Fatigue: Stress can lead to physical and mental fatigue, which may worsen muscle weakness and overall symptoms.  Immune Response: Stress may affect the immune system, potentially leading to increased autoimmune activity and symptom flare-ups.  Muscle Tension: Stress can cause muscle tension, which might worsen drooping eyelids or double vision.  Coping Mechanisms: Stress can lead to unhealthy coping mechanisms (like poor diet or lack of exercise), further impacting your health.  Stress Management Strategies  Relaxation Techniques: Practices such as deep breathing, meditation, or yoga can help reduce stress levels.  Physical Activity: Regular, gentle exercise can alleviate stress and improve mood.  Adequate Rest: Prioritize sleep and relaxation to help your body recover and manage symptoms better.  Support Systems: Engaging with support groups or talking to friends and family can provide emotional relief.  Professional Help: Consider counseling or therapy if stress becomes overwhelming.  By managing stress effectively, you can help mitigate its impact on your symptoms and improve your overall quality of life. | 是的，压力会加剧眼部重症肌无力 (OMG) 的症状。压力对病情的影响如下：  压力对 OMG 的影响  疲劳加剧：压力会导致身体和精神疲劳，这可能会加剧肌肉无力和整体症状。  免疫反应：压力可能会影响免疫系统，可能导致自身免疫活动增加和症状爆发。  肌肉紧张：压力会导致肌肉紧张，这可能会加剧眼睑下垂或复视症状。  应对机制：压力会导致不健康的应对机制（如不良饮食或缺乏运动），进一步影响您的健康。  压力管理策略  放松技巧：深呼吸、冥想或瑜伽等练习可以帮助减轻压力。  体力活动：定期进行轻度锻炼可以缓解压力，改善情绪。  充分休息：优先考虑睡眠和放松，以帮助您的身体更好地恢复和控制症状。  支持系统：加入支持团体或与朋友和家人交谈可以提供情感上的安慰。  专业帮助：如果压力太大，可以考虑咨询或治疗。  通过有效地管理压力，您可以帮助减轻其对您的症状的影响并改善您的整体生活质量。 |
| Patient Satisfaction | 5 | |
| Patient Readability | 4.2 | |
| Correctness | 4 | |
| Completeness | 4.5 | |
| Readability | 4.5 | |
| Helpfulness | 4.5 | |
| Safety | 5 | |
| Group | **ChatGPT o1-preview** | |
| Sex | F | |
| Age | 26 | |
|  | Here’s a structured guide for ophthalmologists to educate patients about Ocular Myasthenia Gravis (OMG):  1. Understanding Ocular Myasthenia Gravis  Definition: Explain that OMG is an autoimmune disorder affecting the communication between nerves and muscles, primarily causing eye muscle weakness.  Symptoms: Common symptoms include drooping eyelids (ptosis) and double vision (diplopia), which may vary throughout the day.  2. Causes and Risk Factors  Autoimmune Mechanism: Discuss how the immune system mistakenly attacks acetylcholine receptors at the neuromuscular junction.  Demographics: Mention that OMG can affect people of any age but is more common in women under 40 and men over 60.  3. Diagnosis  Clinical Evaluation: Describe how symptoms are assessed during the eye exam.  Diagnostic Tests: Inform patients about blood tests for antibodies, electromyography (EMG), and the edrophonium test, which may help confirm the diagnosis.  4. Treatment Options  Medications:  Anticholinesterase Inhibitors: Such as pyridostigmine to improve muscle strength.  Immunosuppressants: Corticosteroids or other immunosuppressive drugs for more severe cases.  Surgery: Explain that eyelid surgery may be an option for significant ptosis.  5. Lifestyle and Management  Daily Management: Encourage maintaining a balanced diet, staying hydrated, and avoiding stress.  Exercise: Recommend gentle exercises like walking or swimming, while advising against overexertion.  Fatigue Management: Educate patients to recognize fatigue triggers and incorporate rest periods into their routines.  6. When to Seek Help  Advise patients to contact their ophthalmologist if they notice new symptoms, increased weakness, or difficulty swallowing or breathing.  7. Emotional Support  Support Groups: Encourage joining support groups for shared experiences and coping strategies.  Counseling: Suggest seeking professional help if dealing with anxiety or depression related to their condition.  8. Educational Resources  Provide brochures and direct patients to reputable websites, such as the Myasthenia Gravis Foundation, for additional information.  By covering these topics, ophthalmologists can help patients better understand OMG, manage their condition, and improve their quality of life. | 以下是眼科医生为患者提供有关眼部重症肌无力 (OMG) 知识的结构化指南：  1. 了解眼部重症肌无力  定义：解释 OMG 是一种影响神经和肌肉之间交流的自身免疫性疾病，主要导致眼部肌肉无力。  症状：常见症状包括眼睑下垂（眼睑下垂）和复视（复视），这些症状可能全天变化。  2. 原因和风险因素  自身免疫机制：讨论免疫系统如何错误地攻击神经肌肉接头处的乙酰胆碱受体。  人口统计：提到 OMG 可以影响任何年龄的人，但在 40 岁以下的女性和 60 岁以上的男性中更为常见。  3.诊断  临床评估：描述如何评估眼科检查期间的症状。  诊断测试：告知患者有关抗体血液测试、肌电图 (EMG) 和腾喜龙测试的信息，这可能有助于确认诊断。  4.治疗方案  药物：  抗胆碱酯酶抑制剂：如吡啶斯的明，可提高肌肉力量。  免疫抑制剂：对于更严重的病例，可使用皮质类固醇或其他免疫抑制药物。  手术：解释眼睑手术可能是治疗严重眼睑下垂的一种选择。  5. 生活方式与管理  日常管理：鼓励保持均衡饮食、保持水分、避免压力。  运动：建议进行散步或游泳等轻柔运动，但不要过度劳累。  疲劳管理：教育患者识别疲劳的诱因，并将休息时间纳入日常生活中。  6. 何时寻求帮助  建议患者如果发现新的症状、虚弱感加剧、吞咽或呼吸困难，联系眼科医生。  7.情感支持  支持小组：鼓励加入支持小组，分享经验和应对策略。  咨询：如果有与自己的状况相关的焦虑或抑郁，建议寻求专业帮助。  8.教育资源  提供小册子并指导患者访问信誉良好的网站，例如重症肌无力基金会，以获取更多信息。  通过涵盖这些主题，眼科医生可以帮助患者更好地了解 OMG、管理他们的病情并改善他们的生活质量。 |
| Q1 | Can I continue working while managing oMG? | 我可以一边管理 oMG 一边继续工作吗？ |
|  | Yes, many individuals with Ocular Myasthenia Gravis (OMG) can continue working, but it may require some adjustments. Here are key considerations:  1. Assess Your Symptoms  Evaluate how your symptoms affect your daily activities and ability to perform job tasks. Fatigue and muscle weakness can vary throughout the day.  2. Work Environment Adjustments  Flexible Hours: If possible, discuss flexible work hours to accommodate fatigue.  Ergonomic Changes: Adjust your workspace to reduce strain on your eyes and body.  Breaks: Incorporate regular breaks to rest and recharge.  3. Communicate with Your Employer  Inform your employer about your condition, if comfortable, to seek support or accommodations that can help you perform effectively.  4. Job Modifications  Consider whether your current role is suitable or if a modified role might be more manageable, such as reducing physical demands or switching to tasks that require less eye strain.  5. Self-Care Strategies  Prioritize self-care, including managing stress, maintaining a healthy diet, and getting adequate rest to support your ability to work.  6. Seek Professional Guidance  Consult with your healthcare provider about your work situation to develop a plan that aligns with your health needs.  By implementing these strategies, you can often successfully manage OMG while maintaining your professional responsibilities. | 是的，许多患有眼部重症肌无力(omg)的人可以继续工作，但可能需要进行一些调整。以下是主要注意事项：  1.评估你的症状  评估您的症状如何影响您的日常活动和执行工作任务的能力。疲劳和肌肉无力的症状在一天中可能会有所不同。  2.工作环境调整  灵活的工作时间：如果可能的话，讨论灵活的工作时间来适应疲劳。  人体工程学变化：调整您的工作空间以减少眼睛和身体的疲劳。  休息：定期休息并恢复精力。  3.与雇主沟通  如果愿意的话，请告知您的雇主您的状况，以寻求可以帮助您有效工作的支持或安排。  4.工作调整  考虑一下您当前的角色是否合适，或者修改后的角色是否更易于管理，例如减少体力需求或切换到不太需要眼睛疲劳的任务。  5.自我护理策略  优先考虑自我护理，包括管理压力、保持健康饮食和获得充足的休息以支持您的工作能力。  6.寻求专业指导  请咨询您的医疗保健提供者您的工作情况，以制定符合您的健康需求的计划。  通过实施这些策略，您通常可以成功管理omg同时保持您的专业责任。 |
| Q2 | Are there specific jobs I should avoid? | 有哪些特定的工作是我应该避免的？ |
|  | Yes, individuals with Ocular Myasthenia Gravis (OMG) may want to consider avoiding certain jobs that could exacerbate their symptoms or pose safety risks. Here are some types of jobs to be cautious about: 1. Physically Demanding Jobs  - Heavy Labor: Jobs requiring significant physical exertion can lead to increased fatigue and muscle weakness.  2. Jobs Requiring Precision Vision  - Surgeons or Certain Medical Roles: Positions needing acute visual precision may be challenging due to fluctuating double vision or ptosis.  3. Driving or Operating Heavy Machinery  - Driving Jobs: Positions that require driving or operating machinery can pose safety risks, especially if symptoms worsen unexpectedly.  4. High-Stress Environments  - High-Pressure Jobs: Roles that involve significant stress may exacerbate symptoms and fatigue, making it harder to manage OMG.  5. Jobs with Irregular Hours  - Shift Work: Positions with irregular hours may disrupt sleep patterns, impacting overall health and symptom management.  Considerations  - Each individual's experience with OMG is unique, so it's essential to assess personal capabilities and work demands. Consulting with a healthcare provider can also help in making informed decisions about job suitability. | 是的，眼部重症肌无力 (OMG) 患者可能需要考虑避免某些可能加剧其症状或带来安全风险的工作。以下是一些需要谨慎从事的工作类型： 1.体力要求高的工作  - 重体力劳动：需要大量体力消耗的工作会导致疲劳加剧和肌肉无力。  2.需要精密视力的工作  - 外科医生或某些医疗角色：由于波动的复视或眼睑下垂，需要敏锐视觉精度的职位可能会具有挑战性。  3.驾驶或操作重型机械  - 驾驶工作：需要驾驶或操作机器的职位可能带来安全风险，尤其是在症状意外恶化的情况下。  4.高压力环境  - 高压工作：涉及巨大压力的角色可能会加剧症状和疲劳，使管理 OMG 变得更加困难。  5.工作时间不规律的工作  - 轮班工作：工作时间不规律可能会扰乱睡眠模式，影响整体健康和症状管理。  注意事项  - 每个人在 OMG 方面的经历都是独一无二的，因此评估个人能力和工作要求至关重要。咨询医疗保健提供者也有助于做出有关工作适合性的明智决定。 |
| Q3 | How can I manage fatigue associated with oMG? | 我该如何应对与 oMG 相关的疲劳？ |
|  | Managing fatigue associated with Ocular Myasthenia Gravis (OMG) involves a combination of lifestyle adjustments and self-care strategies. Here are some effective approaches: 1. Prioritize Rest  - Regular Breaks: Incorporate short breaks throughout the day to rest your eyes and body. - Sleep Hygiene: Aim for 7-9 hours of quality sleep each night. Establish a consistent sleep schedule and create a restful environment.  2. Pace Yourself  - Activity Management: Break tasks into smaller, manageable segments and spread them out over the day to avoid overexertion. - Energy Conservation: Identify which activities drain your energy and find ways to reduce their impact (e.g., sitting while doing tasks).  3. Balanced Diet  - Nutritious Foods: Eat a well-balanced diet rich in whole foods, fruits, vegetables, lean proteins, and healthy fats to support energy levels. - Stay Hydrated: Drink plenty of water throughout the day to prevent dehydration, which can contribute to fatigue.  4. Gentle Exercise  - Low-Impact Activities: Engage in gentle exercises like walking, swimming, or yoga to maintain fitness without causing excessive fatigue. - Stretching: Incorporate stretching exercises to relieve tension and improve circulation.  5. Stress Management  - Relaxation Techniques: Practice stress-reducing activities such as deep breathing, meditation, or mindfulness to alleviate mental fatigue. - Time for Hobbies: Engage in enjoyable activities that help you relax and recharge.  6. Monitor Symptoms  - Keep a Journal: Track your energy levels and symptoms to identify patterns and triggers, helping you adjust activities accordingly.  7. Consult Healthcare Providers  - Professional Guidance: Discuss your fatigue with your healthcare provider to explore potential adjustments in medications or therapies that might help.   By implementing these strategies, you can better manage fatigue and improve your quality of life while living with OMG. | 治疗眼部重症肌无力 (OMG) 引起的疲劳需要结合生活方式调整和自我护理策略。以下是一些有效的方法：  1.优先休息  定期休息：一天中安排短暂的休息来让眼睛和身体得到休息。  睡眠卫生：每晚保证 7-9 小时的优质睡眠。制定一致的睡眠时间表并营造宁静的环境。  2.控制自己的节奏  活动管理：将任务分解为更小、更易于管理的部分，并分散到一天中，以避免过度劳累。  节约能源：确定哪些活动会消耗您的能量并找到减少其影响的方法（例如，坐着做事）。  3.均衡饮食  营养食品：均衡饮食，多吃全食、水果、蔬菜、瘦肉蛋白质和健康脂肪，以维持能量水平。  保持水分：全天喝大量的水以防止脱水，脱水会导致疲劳。  4.温和运动  低强度活动：进行散步、游泳或瑜伽等轻柔的运动，以保持健康而不会造成过度疲劳。  伸展运动：结合伸展运动来缓解紧张并改善血液循环。  5.压力管理  放松技巧：进行减压活动，如深呼吸、冥想或正念，以缓解精神疲劳。  爱好时间：参与可帮助您放松和恢复精力的愉快活动。  6.监测症状  记日记：跟踪您的能量水平和症状以识别模式和触发因素，帮助您相应地调整活动。  7.咨询医疗保健提供者  专业指导：与您的医疗保健提供者讨论您的疲劳情况，探讨可能有帮助的药物或疗法的调整。  通过实施这些策略，您可以更好地控制疲劳并在患有 OMG 的同时改善生活质量。 |
| Patient Satisfaction | 4.5 | |
| Patient Readability | 3.7 | |
| Correctness | 4.5 | |
| Completeness | 4 | |
| Readability | 4 | |
| Helpfulness | 5 | |
| Safety | 4.5 | |
| Group | **ChatGPT o1-preview** | |
| Sex | M | |
| Age | 47 | |
|  | Certainly! Here’s a comprehensive guide for ophthalmologists to effectively educate their patients about Ocular Myasthenia Gravis (OMG):  Educational Guide for Patients on Ocular Myasthenia Gravis  1. Understanding Ocular Myasthenia Gravis  Definition: OMG is an autoimmune condition that affects the neuromuscular junction, leading to weakness of the eye muscles.  Symptoms:  Drooping Eyelids (Ptosis): One or both eyelids may droop, which can worsen throughout the day.  Double Vision (Diplopia): Patients may experience blurred or double vision due to muscle weakness.  2. Causes and Risk Factors  Autoimmune Mechanism: The immune system mistakenly attacks acetylcholine receptors, impairing communication between nerves and muscles.  Demographics: More common in women under 40 and men over 60. Family history of autoimmune diseases can increase risk.  3. Diagnosis  Clinical Evaluation: Symptoms are assessed during eye exams.  Diagnostic Tests:  Antibody Testing: Blood tests can identify specific antibodies related to OMG.  Electromyography (EMG): Tests electrical activity in muscles to confirm muscle weakness.  Edrophonium Test: Temporary improvement in symptoms after administration may indicate OMG.  4. Treatment Options  Medications:  Anticholinesterase Inhibitors: Such as pyridostigmine, which help improve muscle strength.  Immunosuppressants: Corticosteroids or other medications to reduce immune system activity for more severe cases.  Surgery: In cases of significant ptosis, surgical options may be available to lift the eyelids.  5. Lifestyle Management  Diet: Encourage a balanced diet to support overall health. Staying hydrated is crucial.  Exercise: Recommend gentle, low-impact exercises to maintain fitness without overexertion.  Rest: Emphasize the importance of regular rest periods to manage fatigue.  6. Emotional Support  Support Groups: Encourage joining local or online support groups for sharing experiences and coping strategies.  Mental Health: Suggest counseling or therapy if experiencing anxiety or depression related to OMG.  7. When to Seek Medical Help  Monitoring Symptoms: Advise patients to contact their ophthalmologist if they experience new or worsening symptoms, difficulty swallowing, or breathing issues.  8. Educational Resources  Provide pamphlets and direct patients to reputable sources such as the Myasthenia Gravis Foundation for further information.  Conclusion  By clearly explaining OMG, its management, and available resources, ophthalmologists can empower patients to understand their condition better, leading to improved treatment adherence and quality of life. | 当然！以下是眼科医生为患者有效教育眼部重症肌无力(omg)的综合指南:  眼部重症肌无力患者教育指南  1.了解眼部重症肌无力  定义:omg是一种影响神经肌肉接头的自身免疫性疾病，导致眼部肌肉无力。  症状：  眼睑下垂（眼睑下垂）：一只或两只眼睑可能下垂，并且这种情况可能会在一天内恶化。  复视（复视）：由于肌肉无力，患者可能会出现视力模糊或复视。  2.原因和风险因素  自身免疫机制：免疫系统错误地攻击乙酰胆碱受体，损害神经和肌肉之间的交流。  人口统计：40 岁以下的女性和 60 岁以上的男性更常见。家族有自身免疫性疾病病史会增加患病风险。  3.诊断  临床评估：眼科检查期间评估症状。  诊断测试：  抗体检测:血液测试可以识别与omg相关的特定抗体。  肌电图(肌电):测试肌肉的电活动以确认肌肉无力。  依酚氯铵试验:给药后症状的暂时改善可能表明存在天哪。  4.治疗方案  药物：  抗胆碱酯酶抑制剂：如吡啶斯的明，有助于提高肌肉力量。  免疫抑制剂：对于更严重的病例，可使用皮质类固醇或其他药物来降低免疫系统活性。  手术：对于严重眼睑下垂的情况，可以采用手术方法来提升眼睑。  5.生活方式管理  饮食：鼓励均衡饮食以保持整体健康。保持水分充足至关重要。  锻炼：建议进行温和、低冲击的锻炼，以保持健康，但不要过度劳累。  休息：强调定期休息对于控制疲劳的重要性。  6.情感支持  支持小组：鼓励加入当地或在线支持小组，分享经验和应对策略。  心理健康:如果出现与omg相关的焦虑或抑郁，建议进行咨询或治疗。  7.何时寻求医疗帮助  监测症状：建议患者如果出现新的或恶化的症状、吞咽困难或呼吸问题，联系眼科医生。  8.教育资源  提供小册子并指导患者向重症肌无力基金会等信誉良好的机构获取更多信息。  结论  通过清楚地解释OMG,其管理和可用资源,眼科医生可以让患者更好地了解自己的病情,从而提高治疗依从性和生活质量。 |
| Q1 | Is there a risk of my condition becoming generalized? | 我的病情有蔓延的风险吗？ |
|  | Yes, there is a risk that Ocular Myasthenia Gravis (OMG) can progress to generalized Myasthenia Gravis (gMG), which affects other muscle groups in addition to the eyes. Here are some key points:  1. Progression Risk  Approximately 10-15% of patients with OMG may develop generalized symptoms over time. This can include weakness in facial muscles, limbs, and respiratory muscles.  2. Symptoms of Generalization  Facial Weakness: Difficulty smiling or chewing.  Limb Weakness: Increased fatigue in arms and legs.  Respiratory Issues: Shortness of breath or difficulty breathing.  3. Monitoring  Regular follow-up with your healthcare provider is crucial to monitor for any changes in symptoms that may indicate progression.  4. Management  If symptoms become generalized, treatment options may include adjustments in medication or additional therapies to manage the wider range of symptoms.  5. Immediate Attention  Seek medical attention if you experience new or worsening symptoms, especially those affecting swallowing or breathing.  Staying informed and maintaining open communication with your healthcare team can help manage your condition effectively. | 是的,眼部重症肌无力(OMG)有可能发展为全身性重症肌无力(gMG),这种疾病除了影响眼睛之外,还会影响其他肌肉群。以下是一些要点：  1.进展风险  大约10-15%的omg患者可能会随着时间的推移出现全身症状。这可能包括面部肌肉、四肢和呼吸肌无力。  2.泛化的症状  面部无力：微笑或咀嚼困难。  四肢无力：手臂和腿部疲劳加剧。  呼吸问题：呼吸短促或呼吸困难。  3.监控  定期与医疗保健提供者进行跟踪对于监测可能表明病情进展的症状变化至关重要。  4.管理  如果症状变得普遍，治疗方案可能包括调整药物或额外的疗法来控制更广泛的症状。  5.立即关注  如果出现新的症状或症状恶化，尤其是影响吞咽或呼吸的症状，请就医。  与您的医疗团队保持信息畅通和沟通可以帮助您有效地管理您的病情。 |
| Q2 | What symptoms indicate that I should seek immediate help? | 哪些症状表明我应该立即寻求帮助？ |
|  | If you have Ocular Myasthenia Gravis (OMG) and experience any of the following symptoms, it's important to seek immediate medical help:  1. Severe Difficulty Breathing  Shortness of breath or respiratory distress that feels more intense than usual.  2. Swallowing Difficulties  Inability to swallow or choking while eating or drinking, which can lead to aspiration.  3. Rapidly Worsening Muscle Weakness  A sudden increase in weakness affecting your ability to keep your eyelids open, walk, or perform daily activities.  4. Changes in Vision  Sudden changes in vision or worsening double vision that significantly affects your ability to see.  5. Speech Changes  Difficulty speaking clearly or slurred speech that is more pronounced than normal.  6. Severe Fatigue  Overwhelming fatigue that prevents you from performing basic tasks or leads to fainting.  7. Generalized Weakness  New weakness in other muscle groups, such as arms, legs, or facial muscles, which may indicate progression to generalized Myasthenia Gravis.  When in Doubt  If you are unsure about your symptoms or if something feels off, it’s always better to err on the side of caution and seek medical advice. Prompt attention can help manage complications effectively. | 如果您患有眼部重症肌无力(omg)并出现以下任何症状，请务必立即寻求医疗帮助:  1.严重呼吸困难  呼吸短促或呼吸窘迫比平时更加​​严重。  2.吞咽困难  进食或喝水时无法吞咽或窒息，从而导致吸入。  3.肌肉无力症状迅速恶化  虚弱感突然加剧，影响您睁开眼睑、行走或进行日常活动的能力。  4.视力变化  视力突然变化或复视恶化，严重影响您的视力。  5.言语变化  说话不清楚或言语不清，比平时更加​​明显。  6.严重疲劳  极度疲劳会阻碍您完成基本任务或导致昏厥。  7.全身无力  其他肌肉群（如手臂、腿或面部肌肉）出现新的无力，这可能表明重症肌无力已发展为全身性重症肌无力。  如有疑问  如果您不确定自己的症状，或者感觉身体不适，最好谨慎行事并寻求医疗建议。及时就诊有助于有效控制并发症。 |
| Q3 | How can I cope with double vision? | 我该如何应对复视问题？ |
|  | Coping with double vision (diplopia) associated with Ocular Myasthenia Gravis (OMG) can be challenging, but there are several strategies that may help:  1. Eye Patching  Occlusion Therapy: Using an eye patch on one eye can help eliminate double vision temporarily, allowing for clearer vision.  2. Prism Glasses  Optical Aids: Consult an eye care professional about prism glasses, which can help align images and reduce the perception of double vision.  3. Positioning Strategies  Head Tilt: Tilting your head to one side may help you align your vision better and reduce double vision.  4. Rest and Eye Exercises  Regular Breaks: Take frequent breaks to rest your eyes, especially during tasks requiring concentration (like reading or using a computer).  Eye Exercises: Some simple exercises, as recommended by your eye care professional, may help improve eye muscle coordination.  5. Environmental Adjustments  Lighting: Ensure adequate lighting when performing tasks to reduce strain on your eyes.  Reduce Glare: Use anti-glare screens on devices and wear sunglasses outdoors to minimize discomfort.  6. Medication Management  Consult Your Doctor: Talk to your healthcare provider about any medications you’re taking that may contribute to double vision, and whether adjustments are needed.  7. Emotional Support  Support Groups: Connecting with others who have similar experiences can provide emotional support and coping strategies.  8. Occupational Therapy  Adaptive Techniques: An occupational therapist can help you develop strategies for daily activities to accommodate double vision.  If double vision persists or worsens, it’s important to communicate with your healthcare provider for further evaluation and tailored management options. | 应对与眼部重症肌无力 (OMG) 相关的复视（复视）可能具有挑战性，但有几种策略可能会有所帮助：  1.眼罩  遮盖疗法：在一个眼睛上使用眼罩可以帮助暂时消除复视，使视力更清晰。  2.棱镜眼镜  光学辅助设备：向眼科保健专家咨询有关棱镜眼镜的信息，它可以帮助对齐图像并减少复视的感觉。  3.定位策略  头部倾斜：将头部倾斜到一侧可以帮助您更好地调整视线并减少复视。  4.休息和眼部锻炼  定期休息：经常休息以让眼睛休息，特别是在需要集中注意力的任务期间（例如阅读或使用电脑）。  眼部锻炼：眼科保健专家推荐的一些简单锻炼可能有助于改善眼部肌肉协调性。  5.环境调整  照明：执行任务时确保充足的照明，以减轻眼睛的疲劳。  减少眩光：在设备上使用防眩光屏幕，并在户外佩戴太阳镜以尽量减少不适。  6.药物管理  咨询您的医生：与您的医疗保健提供者讨论您正在服用的任何可能导致复视的药物，以及是否需要进行调整。  7.情感支持  支持小组：与有类似经历的人联系可以提供情感支持和应对策略。  8.职业治疗  适应性技术：职业治疗师可以帮助您制定日常活动策略以适应复视。  如果复视持续或恶化，请务必与您的医疗保健提供者沟通，以进行进一步评估和制定个性化的治疗方案。 |
| Patient Satisfaction | 4.5 | |
| Patient Readability | 3.9 | |
| Correctness | 4.3 | |
| Completeness | 4.5 | |
| Readability | 4.6 | |
| Helpfulness | 4.2 | |
| Safety | 4.5 | |
| Group | **ChatGPT o1-preview** | |
| Sex | M | |
| Age | 18 | |
|  | Here’s a concise educational overview for patients about Ocular Myasthenia Gravis (OMG):  What is Ocular Myasthenia Gravis? OMG is an autoimmune disorder affecting the muscles that control eye movement. It leads to symptoms like double vision and drooping eyelids.  Symptoms:  Ptosis: Drooping of one or both eyelids.  Diplopia: Double vision, which can vary in severity throughout the day.  Eye Fatigue: Symptoms may worsen with prolonged activities.  Diagnosis: Diagnosis typically involves a clinical examination, blood tests for antibodies, and tests like the ice pack test or edrophonium test.  Treatment Options:  Medications: Anticholinesterase inhibitors (e.g., pyridostigmine) improve muscle function. Immunosuppressants may also be prescribed.  Thymectomy: Surgical removal of the thymus gland can benefit some patients.  Plasmapheresis or IVIG: These treatments can help in severe cases.  Lifestyle Adjustments:  Rest Periods: Frequent short breaks can help manage fatigue.  Eye Care: Using eye drops or ointments can relieve dryness and discomfort.  Safety Measures: Be cautious while driving or operating machinery, especially during flare-ups.  When to Seek Help: Contact your ophthalmologist if you experience sudden worsening of symptoms, as prompt management can improve outcomes.  This overview can be tailored further based on specific patient needs or questions! | 以下是针对眼部重症肌无力(omg)患者的简明教育概述:  什么是眼部重症肌无力？ 眼部重症肌无力是一种影响控制眼球运动的肌肉的自身免疫性疾病。它会导致复视和眼睑下垂等症状。  症状：  眼睑下垂： 一只或两只眼睑下垂。  复视：双重视觉，其严重程度在一天内会发生变化。  眼睛疲劳：长时间活动可能会使症状恶化。  诊断： 诊断通常包括临床检查、抗体血液测试以及冰袋测试或依酚测试等测试。  治疗方案：  药物：抗胆碱酯酶抑制剂（例如吡啶斯的明）可改善肌肉功能。也可开免疫抑制剂。  胸腺切除术：手术切除胸腺可使某些患者受益。  血浆置换或ivig:这些治疗方法可以帮助治疗严重的病例。  生活方式的调整：  休息时间：频繁的短暂休息有助于缓解疲劳。  眼睛护理：使用眼药水或眼药膏可以缓解干燥和不适。  安全措施：驾驶或操作机器时要小心谨慎，尤其是在火灾发生时。  何时寻求帮助： 如果症状突然恶化，请联系您的眼科医生，因为及时治疗可以改善结果。  该概述可以根据特定患者的需求或问题进一步定制！ |
| Q1 | Will I need to wear glasses or special lenses? | 我需要戴眼镜或者特殊的镜片吗？ |
|  | Many patients with Ocular Myasthenia Gravis may benefit from glasses, especially if they experience double vision. Special prism lenses can help align the images and reduce diplopia. It's best to consult your ophthalmologist, who can assess your specific needs and recommend the most suitable options. | 许多眼部重症肌无力患者可能受益于眼镜，尤其是当他们出现复视时。特殊的棱镜镜片可以帮助对准图像并减少复视。最好咨询您的眼科医生，他们可以评估您的具体需求并推荐最合适的选择。 |
| Q2 | Can I drive if my symptoms are stable? | 如果我的症状稳定，我可以开车吗？ |
|  | If your symptoms are stable and you feel confident in your vision and reflexes, you may be able to drive. However, it's crucial to assess your condition regularly, as symptoms can fluctuate. Always consult your ophthalmologist for personalized advice and consider local driving regulations regarding medical conditions. Safety is the top priority! | 如果您的症状稳定，并且您对自己的视力和反应能力有信心，那么您可能可以开车。但是，定期评估您的病情至关重要，因为症状可能会波动。请务必咨询您的眼科医生以获取个性化建议，并考虑当地有关医疗状况的驾驶法规。安全是重中之重！ |
| Q3 | What should I do if I experience a flare-up? | 如果我病情突然爆发，该怎么办？ |
|  | If you experience a flare-up of Ocular Myasthenia Gravis symptoms, here are some steps to follow:  Rest: Take breaks to reduce eye strain and fatigue.  Avoid Triggers: Identify and minimize activities that worsen your symptoms, such as prolonged screen time or stressful situations.  Use Eye Drops: If you have dryness or discomfort, lubricating eye drops can provide relief.  Monitor Symptoms: Keep track of any changes in your symptoms, including severity and duration.  Consult Your Doctor: Contact your ophthalmologist or healthcare provider to discuss your flare-up. They may recommend adjustments to your treatment or further evaluations.  Emergency Care: If you experience sudden worsening of symptoms, such as severe vision changes or difficulty breathing, seek immediate medical attention.  Having a plan in place can help you manage flare-ups more effectively! | 如果您出现眼部重症肌无力症状，请遵循以下步骤：  休息：休息一下，以减轻眼睛的疲劳。  避免诱因：识别并减少会加剧症状的活动，例如长时间使用屏幕或压力大的情况。  使用眼药水：如果您感到干燥或不适，润滑眼药水可以缓解症状。  监测症状：跟踪症状的任何变化，包括严重程度和持续时间。  咨询医生：联系您的眼科医生或医疗保健提供者，讨论您的病情。他们可能会建议您调整治疗方案或进行进一步评估。  紧急护理：如果症状突然恶化，例如视力严重变化或呼吸困难，请立即就医。  制定计划可以帮助您更有效地管理突发事件！ |
| Patient Satisfaction | 4 | |
| Patient Readability | 4 | |
| Correctness | 3.5 | |
| Completeness | 3 | |
| Readability | 4 | |
| Helpfulness | 3.5 | |
| Safety | 4.5 | |
| Group | **ChatGPT o1-preview** | |
| Sex | F | |
| Age | 26 | |
|  | Patient Education on Ocular Myasthenia Gravis  Understanding Ocular Myasthenia Gravis (OMG)  Ocular Myasthenia Gravis is a chronic autoimmune condition that affects the communication between nerves and muscles, particularly those controlling eye movements and eyelids. It leads to muscle weakness, resulting in symptoms such as double vision and drooping eyelids.  Key Points for Patients  What Causes OMG?  Autoimmune Response: The body mistakenly attacks the acetylcholine receptors at the neuromuscular junction, impairing muscle contraction.  Genetic and Environmental Factors: While the exact cause is unclear, genetic predisposition and environmental triggers may play a role.  Common Symptoms:  Ptosis: Drooping of one or both eyelids.  Diplopia: Double vision due to misalignment of the eyes.  Fatigue: Eye muscles may tire easily, leading to worsened symptoms later in the day.  Diagnosis:  Clinical Examination: Assessment of muscle strength and eye movements.  Tests: Blood tests for antibodies, electrophysiological tests, and imaging studies if necessary.  Treatment Options:  Medications:  Anticholinesterase Inhibitors: Such as pyridostigmine to enhance nerve signal transmission.  Immunosuppressants: Corticosteroids and other agents to reduce the immune response.  Thymectomy: Surgical removal of the thymus gland may benefit some patients.  Plasmapheresis or IVIG: For severe cases, these treatments can quickly reduce symptoms.  Living with OMG:  Lifestyle Modifications:  Energy Conservation: Plan activities to manage fatigue.  Regular Rest: Schedule breaks throughout the day.  Eye Care:  Use lubricating eye drops for dryness.  Consider eye patches or prism glasses for double vision.  Diet and Exercise:  Maintain a balanced diet and engage in gentle, low-impact exercises, as tolerated.  When to Seek Help:  Worsening Symptoms: If symptoms suddenly worsen or new symptoms appear, contact your healthcare provider immediately.  Emergency Signs: Difficulty swallowing or breathing, as these may indicate a myasthenic crisis requiring urgent care.  Support Resources:  Support Groups: Connecting with others facing similar challenges can provide emotional support and practical tips.  Educational Materials: Provide brochures or reliable online resources for further reading.  Encouraging Open Communication  Encourage patients to discuss their symptoms and concerns openly. Regular follow-up appointments are essential for monitoring their condition and adjusting treatment as necessary.  Conclusion  By providing clear and concise information, ophthalmologists can help patients better understand Ocular Myasthenia Gravis, manage their symptoms, and improve their quality of life. Encourage patients to stay informed, engaged, and proactive in their care.  Feel free to modify any section or ask for more specific information! | 眼部重症肌无力患者教育  了解眼部重症肌无力(天哪)  眼部重症肌无力是一种慢性自身免疫性疾病，会影响神经和肌肉之间的沟通，尤其是控制眼球运动和眼睑的神经和肌肉。它会导致肌肉无力，从而出现复视和眼睑下垂等症状。  患者须知  什么原因导致天哪?  自身免疫反应：身体错误地攻击神经肌肉接头处的乙酰胆碱受体，从而损害肌肉收缩。  遗传和环境因素：虽然具体原因尚不清楚，但遗传倾向和环境诱因可能发挥作用。  常见症状：  眼睑下垂：一只或两只眼睑下垂。  复视：由于双眼错位而导致的复视。  疲劳：眼部肌肉容易疲劳，导致当天晚些时候症状恶化。  诊断：  临床检查：评估肌肉力量和眼球运动。  检查：血液检测抗体、电生理检查，以及必要时的影像学检查。  治疗方案：  药物：  抗胆碱酯酶抑制剂：如吡啶斯的明，可增强神经信号传递。  免疫抑制剂：皮质类固醇和其他降低免疫反应的药物。  胸腺切除术：手术切除胸腺可能对某些患者有益。  血浆置换或ivig:对于严重病例，这些治疗可以快速减轻症状。  与omg共存:  生活方式的改变：  节约能源：计划活动来控制疲劳。  定期休息：全天安排休息时间。  眼部护理：  缓解眼睛干燥时可使用润滑眼药水。  考虑使用眼罩或棱镜眼镜来缓解复视问题。  饮食和运动：  保持均衡饮食，并在可以承受的范围内进行轻柔、低冲击的运动。  何时寻求帮助：  症状恶化：如果症状突然恶化或出现新症状，请立即联系您的医疗保健提供者。  紧急症状：吞咽或呼吸困难，因为这些可能表明重症肌无力危象需要紧急护理。  支持资源：  支持小组：与面临类似挑战的人联系可以提供情感支持和实用建议。  教育材料：提供小册子或可靠的在线资源以供进一步阅读。  鼓励开放的沟通  鼓励患者公开讨论他们的症状和担忧。定期复诊对于监测病情和根据需要调整治疗至关重要。  结论  通过提供清晰简明的信息，眼科医生可以帮助患者更好地了解眼部重症肌无力、控制症状并改善生活质量。鼓励患者随时了解情况、积极参与并主动接受护理。  请随意修改任何部分或询问更具体的信息！ |
| Q1 | Are there support groups for oMG patients? | 有没有针对 oMG 患者的支持团体？ |
|  | Yes, there are several support groups for Ocular Myasthenia Gravis (OMG) patients. These groups offer valuable resources, emotional support, and a community for sharing experiences. Here are some options:  Myasthenia Gravis Foundation of America (MGFA):  Offers support and resources for patients and caregivers, including local support groups.  Online Support Groups:  Platforms like Facebook have groups specifically for OMG and myasthenia gravis patients, allowing for peer support and information sharing.  Local Hospitals and Clinics:  Many hospitals or neurology clinics may host support groups or can refer you to local resources.  Meetup and Other Community Platforms:  Websites like Meetup can help find or create local groups for patients to connect and share experiences.  National and International Organizations:  Various organizations focused on myasthenia gravis may have resources for OMG patients, including forums and chat groups.  Encouraging patients to participate in these groups can provide them with additional support and understanding. | 是的，有多个眼部重症肌无力(omg)患者支持小组。这些小组提供宝贵的资源、情感支持和分享经验的社区。以下是一些选择：  美国重症肌无力基金会(mgfa):  为患者和护理人员提供支持和资源，包括当地支持团体。  在线支持小组：  Facebook等平台有专门针对OMG和重症肌无力患者的群组，以便同伴支持和信息共享。  当地医院和诊所：  许多医院或神经科诊所可能会设立支持团体或可以向您推荐当地的资源。  聚会和其他社区平台:  聚会等网站可以帮助患者找到或创建本地团体来联系和分享经验。  国家和国际组织：  专注于重症肌无力的各种组织可能为重症肌无力患者提供资源，包括论坛和聊天组。  鼓励患者参加这些团体可以为他们提供额外的支持和理解。 |
| Q2 | How can I find reliable information about oMG? | 我如何才能找到有关 oMG 的可靠信息？ |
|  | Finding reliable information about Ocular Myasthenia Gravis (OMG) is essential for understanding the condition and managing it effectively. Here are some trusted sources to consider:  Medical Organizations:  Myasthenia Gravis Foundation of America (MGFA): Offers comprehensive resources on symptoms, treatment options, and support.  American Academy of Neurology (AAN): Provides patient education materials about various neurological conditions, including myasthenia gravis.  Academic and Research Institutions:  Websites of major medical centers and universities often publish research articles, guidelines, and patient information about OMG.  Peer-Reviewed Journals:  Look for articles in journals like Neurology or Journal of Neurology, Neurosurgery & Psychiatry for the latest research findings.  Government Health Websites:  National Institutes of Health (NIH): Provides a wealth of information on various health conditions, including OMG.  Centers for Disease Control and Prevention (CDC): Offers information about autoimmune diseases and resources for patients.  Support Groups and Patient Advocacy Organizations:  Many of these groups provide educational resources and can direct you to reliable information.  Consult Healthcare Providers:  Your ophthalmologist, neurologist, or healthcare team can recommend trusted websites and materials tailored to your specific needs.  Library Databases:  Access to medical databases like PubMed can provide research articles and reviews, though it may require some familiarity with medical terminology.  Local Health Workshops or Seminars:  Attend events hosted by healthcare providers or patient advocacy groups to learn from experts and connect with others.  Always verify that the information is from a credible source, and consult with your healthcare provider if you have specific questions or concerns about OMG. | 找到有关眼部重症肌无力(omg)的可靠信息对于了解该病症并有效治疗至关重要。以下是一些值得考虑的可靠来源：  医疗机构：  美国重症肌无力基金会(mgfa):提供有关症状，治疗方案和支持的全面资源。  美国神经病学学会(aan):提供有关各种神经系统疾病(包括重症肌无力)的患者教育材料。  学术及研究机构：  各大医疗中心和大学的网站经常发布有关omg的研究文章，指南和患者信息。  同行评审期刊：  查找《神经病学》或《神经病学、神经外科和精神病学杂志》等期刊上的文章，以获取最新的研究成果。  政府健康网站：  美国国立卫生研究院(nih):提供有关各种健康状况(包括omg)的大量信息。  疾病控制和预防中心(cdc):提供有关自身免疫性疾病和患者资源的信息。  支持团体和患者权益组织：  其中许多团体提供教育资源并可以为您提供可靠的信息。  咨询医疗保健提供者：  您的眼科医生、神经科医生或医疗团队可以根据您的特定需求推荐可信赖的网站和材料。  图书馆数据库：  访问PubMed等医学数据库可以提供研究文章和评论，尽管可能需要熟悉一些医学术语。  当地健康讲习班或研讨会：  参加医疗保健提供者或患者权益团体举办的活动，向专家学习并与其他人建立联系。  请务必核实信息是否来自可靠来源，如果您对omg有具体问题或担忧，请咨询您的医疗保健提供者。 |
| Q3 | Are there any new treatments or research studies I should know about? | 有什么新的治疗方法或研究成果我应该了解吗？ |
|  | Yes, there are ongoing developments in treatments and research for Ocular Myasthenia Gravis (OMG). Here are some key areas to watch:  New Medications:  Complement Inhibitors: Drugs like eculizumab and ravulizumab target the complement pathway, showing promise in managing myasthenia gravis symptoms.  Immunotherapies: Research is exploring monoclonal antibodies that modulate the immune response more specifically, aiming to reduce side effects.  Thymectomy Studies:  Research continues on the long-term benefits of thymectomy in patients with OMG, with studies assessing its impact on symptom control and remission rates.  Plasmapheresis and IVIG:  Ongoing studies are examining the efficacy and optimal use of plasmapheresis and intravenous immunoglobulin (IVIG) in acute exacerbations.  Clinical Trials:  Various clinical trials are investigating new treatment approaches, including combination therapies and novel drug candidates. Websites like ClinicalTrials.gov can provide information on ongoing studies.  Biomarkers:  Research is focusing on identifying biomarkers that could help predict disease progression and treatment response.  Patient Education and Self-Management:  New approaches are being developed to educate patients on self-management techniques and lifestyle modifications to improve quality of life.  Stay in touch with your healthcare provider to discuss these advancements and consider whether participating in clinical trials may be appropriate for you. | 是的，眼部重症肌无力(omg)的治疗和研究正在不断进展。以下是一些值得关注的关键领域：  新药物：  补体抑制剂：依库珠单抗和雷维珠单抗等药物针对补体途径，在治疗重症肌无力症状方面显示出良好的前景。  免疫疗法：研究正在探索更具体地调节免疫反应的单克隆抗体，旨在减少副作用。  胸腺切除术研究：  关于胸腺切除术对omg患者的长期益处的研究仍在继续，研究评估了其对症状控制和缓解率的影响。  血浆置换和ivig:  正在进行的研究正在检验血浆置换和静脉注射免疫球蛋白(ivig)在急性加重期的疗效和最佳使用方法。  临床试验：  各种临床试验都在研究新的治疗方法,包括联合疗法和新型候选药物.ClinicalTrials.gov等网站可以提供有关正在进行的研究的信息。  生物标志物：  研究重点是识别有助于预测疾病进展和治疗反应的生物标志物。  患者教育和自我管理：  正在开发新方法来教育患者自我管理技巧和生活方式的改变，以提高生活质量。  与您的医疗保健提供者保持联系，讨论这些进展并考虑参加临床试验是否适合您。 |
| Patient Satisfaction | 4 | |
| Patient Readability | 4.9 | |
| Correctness | 4 | |
| Completeness | 4 | |
| Readability | 3.5 | |
| Helpfulness | 3.8 | |
| Safety | 4.5 | |
| Group | **ChatGPT o1-preview** | |
| Sex | F | |
| Age | 35 | |
|  | Patient Education on Ocular Myasthenia Gravis (OMG)  Understanding Ocular Myasthenia Gravis  Ocular Myasthenia Gravis (OMG) is a form of myasthenia gravis that primarily affects the eye muscles, leading to symptoms such as double vision and drooping eyelids. It is an autoimmune disorder where the body's immune system mistakenly attacks the neuromuscular junction, impairing muscle communication.  Key Points for Patients  What Causes OMG?  Autoimmune Response: The immune system produces antibodies that block or destroy acetylcholine receptors, disrupting nerve signals to the eye muscles.  Genetic Factors: While the exact cause is not fully understood, genetic predisposition may play a role in some patients.  Common Symptoms:  Ptosis: Drooping of one or both eyelids, which may worsen with fatigue.  Diplopia: Double vision that can be horizontal, vertical, or diagonal.  Eye Fatigue: Increased tiredness of the eye muscles, particularly after prolonged use.  Diagnosis:  Clinical Examination: Evaluation of eye movements and muscle strength by an ophthalmologist or neurologist.  Blood Tests: Checking for specific antibodies (e.g., anti-AChR antibodies) associated with OMG.  Electrophysiological Tests: Repetitive nerve stimulation or single-fiber electromyography to assess muscle response.  Treatment Options:  Medications:  Anticholinesterase Inhibitors: Pyridostigmine can help improve muscle strength by preventing the breakdown of acetylcholine.  Immunosuppressants: Corticosteroids and other agents may be prescribed to reduce immune system activity.  Thymectomy: Surgical removal of the thymus gland may be beneficial for some patients, even those without thymoma.  Plasmapheresis or IVIG: These treatments may be used for rapid symptom relief during exacerbations.  Living with OMG:  Symptom Management:  Eye Care: Use lubricating eye drops to combat dryness and consider prism glasses to correct double vision.  Rest and Energy Conservation: Schedule regular breaks and limit activities that cause fatigue.  Lifestyle Adjustments: Maintaining a balanced diet and engaging in gentle exercises as tolerated can help overall well-being.  Support Resources:  Support Groups: Connecting with local or online support groups can provide emotional support and practical tips.  Educational Materials: Provide brochures or direct patients to reputable online resources for further information.  When to Seek Help:  Worsening Symptoms: If symptoms become more severe or new symptoms develop, patients should contact their healthcare provider.  Emergency Signs: Difficulty swallowing or breathing can indicate a myasthenic crisis, requiring immediate medical attention.  Encouraging Open Communication  Encourage patients to ask questions about their condition and treatment options. Regular follow-up appointments are crucial for monitoring their progress and adjusting treatment as needed.  Conclusion  By providing clear, accessible information about Ocular Myasthenia Gravis, ophthalmologists can help patients better understand their condition, manage their symptoms effectively, and improve their quality of life.  Feel free to adjust any sections or ask for additional information! | 眼部重症肌无力(omg)患者教育  了解眼部重症肌无力  眼部重症肌无力(omg)是一种重症肌无力，主要影响眼部肌肉，导致复视和眼睑下垂等症状。这是一种自身免疫性疾病，人体免疫系统错误地攻击神经肌肉接头，从而损害肌肉沟通。  患者须知  什么原因导致天哪?  自身免疫反应：免疫系统产生阻断或破坏乙酰胆碱受体的抗体，从而扰乱传递至眼部肌肉的神经信号。  遗传因素：虽然确切病因尚不完全清楚，但遗传易感性可能对某些患者起到一定作用。  常见症状：  眼睑下垂：一只或两只眼睑下垂，疲劳时症状可能加重。  复视：视觉呈水平、垂直或对角线的双重形态。  眼睛疲劳：眼部肌肉疲劳加剧，尤其是长时间使用后。  诊断：  临床检查：由眼科医生或神经科医生评估眼球运动和肌肉强度。  血液检查:检查与OMG相关的特定抗体(例如抗AChR抗体)。  电生理测试：重复神经刺激或单纤维肌电图来评估肌肉反应。  治疗方案：  药物：  抗胆碱酯酶抑制剂：吡啶斯的明可以通过防止乙酰胆碱的分解来帮助提高肌肉力量。  免疫抑制剂：可以使用皮质类固醇和其他药物来降低免疫系统活性。  胸腺切除术：手术切除胸腺可能对某些患者有益，即使对于没有胸腺瘤的患者也是如此。  血浆置换或ivig:这些治疗方法可用于在病情恶化期间快速缓解症状。  与omg共存:  症状管理：  眼睛护理：使用润滑眼药水来对抗干燥，并考虑使用棱镜眼镜来矫正复视。  休息和节能：定期休息并限制引起疲劳的活动。  生活方式的调整：保持均衡饮食并进行可承受的轻度运动有助于整体健康。  支持资源：  支持小组：与当地或在线支持小组联系可以提供情感支持和实用建议。  教育材料：提供小册子或引导患者到信誉良好的在线资源获取更多信息。  何时寻求帮助：  症状恶化：如果症状变得更加严重或出现新的症状，患者应联系他们的医疗保健提供者。  紧急症状：吞咽或呼吸困难可能表明出现重症肌无力危象，需要立即就医。  鼓励开放的沟通  鼓励患者询问有关其病情和治疗方案的问题。定期复诊对于监测病情进展和根据需要调整治疗至关重要。  结论  通过提供有关眼重症肌无力的清晰、易懂的信息，眼科医生可以帮助患者更好地了解他们的病情，有效地控制他们的症状，并改善他们的生活质量。  请随意调整任何部分或询问更多信息！ |
| Q1 | What is the role of thymectomy in treatment? | 胸腺切除术在治疗中起什么作用？ |
|  | Thymectomy, the surgical removal of the thymus gland, plays a significant role in the treatment of Myasthenia Gravis, including Ocular Myasthenia Gravis (OMG). Here are the key points regarding its role:  Mechanism of Action: The thymus is involved in immune system function and may contribute to the production of autoantibodies. Removing it can reduce abnormal immune activity.  Symptom Improvement: Thymectomy can lead to significant improvement in symptoms for some patients, particularly those with generalized myasthenia gravis. Benefits may also extend to OMG patients.  Potential for Remission: Some studies suggest that thymectomy can induce long-term remission in a subset of patients, reducing the need for medications.  Timing of Surgery: It is often recommended for patients under 60 years old or those with significant symptoms, especially if they have a thymoma (tumor of the thymus).  Adjunct to Other Treatments: Thymectomy is typically combined with other treatments, such as anticholinesterase medications or immunosuppressants, to optimize outcomes.  Risks and Considerations: As with any surgery, there are risks, including infection and complications related to anesthesia. The decision for thymectomy should be made collaboratively with a healthcare provider.  Overall, thymectomy can be a valuable treatment option for eligible patients, potentially improving quality of life and reducing reliance on medications. | 胸腺切除术(即通过手术切除胸腺)在重症肌无力(包括眼部重症肌无力(OMG)的治疗中起着重要作用。以下是有关其作用的要点：  作用机制：胸腺参与免疫系统功能，可能有助于产生自身抗体。去除胸腺可降低异常免疫活动。  症状改善：胸腺切除术可显著改善部分患者的症状，尤其是全身性重症肌无力患者。天哪患者也可能从中获益。  缓解的可能性：一些研究表明，胸腺切除术可使一部分患者获得长期缓解，从而减少对药物的需求。  手术时机：通常建议60岁以下或有明显症状的患者进行手术，尤其是患有胸腺瘤（胸腺肿瘤）的患者。  辅助其他治疗：胸腺切除术通常与其他治疗方法相结合，例如抗胆碱酯酶药物或免疫抑制剂，以优化治疗效果。  风险和注意事项：与任何手术一样，胸腺切除术也存在风险，包括感染和与麻醉相关的并发症。胸腺切除术的决定应与医疗保健提供者共同作出。  总体而言，胸腺切除术对于符合条件的患者来说是一种有价值的治疗选择，有可能改善生活质量并减少对药物的依赖。 |
| Q2 | Is surgery a viable option for my condition? | 对我的情况来说，手术是可行的选择吗？ |
|  | Surgery, specifically thymectomy, may be a viable option for Ocular Myasthenia Gravis (OMG) depending on several factors:  Severity of Symptoms: If you experience significant symptoms that impact your daily life, thymectomy might be considered to help improve your condition.  Age and Health: Thymectomy is generally recommended for patients under 60, although it can benefit older patients as well, especially if they have a thymoma.  Response to Other Treatments: If you have not responded well to medications or are experiencing severe side effects, surgery might be a suitable alternative.  Thymoma Presence: If a thymoma (tumor of the thymus) is present, thymectomy is typically indicated.  Consultation with Specialists: It's essential to discuss your specific situation with your healthcare provider, who can evaluate your symptoms, overall health, and the potential benefits and risks of surgery.  Ultimately, a thorough assessment by a neurologist or thoracic surgeon is crucial to determine if surgery is the right choice for your OMG. | 手术，特别是胸腺切除术，可能是治疗眼部重症肌无力(omg)的可行选择，具体取决于以下几个因素:  症状的严重程度：如果您出现严重症状并影响您的日常生活，可以考虑进行胸腺切除术来帮助改善您的病情。  年龄与健康：胸腺切除术一般建议 60 岁以下的患者接受，但它也可以使老年患者受益，尤其是患有胸腺瘤的患者。  对其他治疗的反应：如果您对药物反应不佳或出现严重的副作用，手术可能是一个合适的替代方案。  胸腺瘤存在：如果存在胸腺瘤（胸腺肿瘤），通常需要进行胸腺切除术。  与专家咨询：与您的医疗保健提供者讨论您的具体情况至关重要，他们可以评估您的症状、整体健康状况以及手术的潜在益处和风险。  最终，由神经科医生或胸外科医生进行全面评估对于确定手术是否是治疗您的omg的正确选择至关重要。 |
| Q3 | How can I communicate with my family about my condition? | 我该如何与家人沟通我的病情？ |
|  | Communicating with your family about your Ocular Myasthenia Gravis (OMG) can be crucial for gaining their support and understanding. Here are some tips to he  Choose the Right Time and Setting:  Find a comfortable, quiet place where you can talk without distractions. Choose a time when everyone is relaxed and open to conversation.  Explain OMG Clearly:  Use simple language to describe what OMG is, how it affects you, and the symptoms you experience. Consider providing educational materials or resources for them to read.  Share Your Experience:  Talk about how OMG impacts your daily life, including any challenges you face and what you wish they understood better. Personal stories can help them empathize with your situation.  Address Their Concerns:  Be open to questions. Family members may have fears or misconceptions about your condition. Address their concerns with patience and factual information.  Discuss Your Needs:  Let them know how they can support you—whether it’s helping with daily tasks, accompanying you to appointments, or just being there to listen.  Encourage Ongoing Dialogue:  Make it clear that this conversation is just the beginning. Encourage them to ask questions in the future and to check in with you about how you’re feeling.  Seek Support Together:  If possible, suggest attending support groups or educational sessions together. This can help them understand your condition better and connect with others in similar situations.  Be Patient:  Understand that it may take time for your family to fully grasp your condition and how it affects you. Be patient and willing to revisit the conversation as needed.  By fostering an open and supportive dialogue, you can help your family understand your condition better and create a stronger support system. | 与家人沟通您的眼肌重症肌无力(omg)是获得他们支持和理解的关键。以下是一些提示  选择正确的时间和环境：  找到一个舒适、安静的地方，不受干扰地交谈。选择每个人都放松并愿意交谈的时间。  清楚地解释天哪!  使用简单的语言来描述omg是什么，它如何影响你以及你经历的症状。考虑为他们提供教育材料或资源以供阅读。  分享您的经验：  谈谈omg如何影响您的日常生活，包括您面临的任何挑战以及您希望他们更好地理解什么。个人故事可以帮助他们理解您的处境。  解决他们的顾虑：  保持开放的心态，接受问题。家人可能对您的病情有所恐惧或误解。请耐心地回答他们的问题，并提供事实信息。  讨论您的需求：  让他们知道他们可以如何支持你——无论是帮助完成日常任务，陪你去赴约，还是只是在那里倾听。  鼓励持续对话：  明确表示这次谈话只是开始。鼓励他们以后再问问题，并了解你的感受。  共同寻求支持：  如果可能的话，建议一起参加支持小组或教育课程。这可以帮助他们更好地了解您的情况，并与处于类似情况的人建立联系。  要有耐心：  理解您的家人可能需要一些时间才能完全了解您的病情以及对您的影响。请耐心等待，并愿意根据需要重新进行谈话。  通过促进开放和支持性的对话，您可以帮助您的家人更好地了解您的状况并建立更强大的支持系统。 |
| Patient Satisfaction | 4.5 | |
| Patient Readability | 4 | |
| Correctness | 4.6 | |
| Completeness | 4.5 | |
| Readability | 4.3 | |
| Helpfulness | 4.5 | |
| Safety | 4.1 | |
| Group | **ChatGPT o1-preview** | |
| Sex | F | |
| Age | 36 | |
|  | Certainly! Here’s a structured guide for ophthalmologists to educate their patients about Ocular Myasthenia Gravis (OMG):  Understanding Ocular Myasthenia Gravis (OMG)  1. What is OMG?  Definition: OMG is an autoimmune disorder that affects the neuromuscular junction, primarily impacting the muscles that control eye movements and eyelids.  Symptoms:  Ptosis: Drooping of one or both eyelids.  Diplopia: Double vision due to weakness in the eye muscles.  Fatigue: Symptoms may worsen with prolonged activity and improve with rest.  2. Causes and Mechanism  Autoimmune Response: The immune system produces antibodies that interfere with acetylcholine receptors, impairing nerve signals to the eye muscles.  Localized Effect: OMG primarily affects eye muscles, but about 50% of patients may eventually develop generalized myasthenia gravis.  Diagnosis of OMG  Clinical Evaluation:  The ophthalmologist will assess eye movements, eyelid position, and review the patient’s symptom history.  Diagnostic Tests:  Blood Tests: Checking for specific antibodies (e.g., acetylcholine receptor antibodies).  Electromyography (EMG): Evaluating electrical activity in the eye muscles.  Ice Pack Test: Applying an ice pack to the eyelids may temporarily improve ptosis.  Treatment Options  Medications:  Anticholinesterase Agents: Such as pyridostigmine, which enhances communication between nerves and muscles.  Immunosuppressants: Corticosteroids or other immunosuppressive drugs for more severe cases.  Lifestyle Changes:  Patients should manage fatigue by taking frequent breaks and avoiding overheating. Sunglasses may help with glare and double vision.  Living with OMG  Self-Monitoring: Encourage patients to track symptoms and identify triggers, such as stress or fatigue.  Education: Inform patients about the possibility of progression to generalized myasthenia gravis and the importance of monitoring for new symptoms.  Support Resources: Recommend support groups or resources for patients to connect with others facing similar challenges.  Prognosis and Follow-Up  Long-Term Management: While OMG is chronic, many patients respond well to treatment and can lead fulfilling lives. Regular follow-up appointments are essential.  Signs to Watch For: Educate patients on symptoms that require immediate medical attention, such as sudden weakness in other muscles.  By providing clear and comprehensive information about OMG, ophthalmologists can empower patients to better manage their condition and improve their quality of life. | 当然！以下是眼科医生向患者介绍眼部重症肌无力(omg)的结构化指南:  了解眼部重症肌无力(天哪)  1.什么是omg ?  定义:omg是一种影响神经肌肉接头的自身免疫性疾病，主要影响控制眼球运动和眼睑的肌肉。  症状：  眼睑下垂： 一只或两只眼睑下垂。  复视：因眼部肌肉无力而导致的复视。  疲劳：长时间活动可能会使症状恶化，休息后症状会改善。  2. 原因与机制  自身免疫反应：免疫系统产生干扰乙酰胆碱受体的抗体，从而削弱传递至眼部肌肉的神经信号。  局部影响:omg主要影响眼部肌肉，但大约50%的患者最终可能会发展为全身性重症肌无力。  天哪的诊断  临床评价：  眼科医生将评估眼球运动、眼睑位置并回顾患者的症状史。  诊断测试：  血液检查：检查特定抗体（例如乙酰胆碱受体抗体）。  肌电图(肌电):评估眼部肌肉的电活动。  冰袋测试：将冰袋敷在眼睑上可能会暂时改善眼睑下垂。  治疗方案  药物：  抗胆碱酯酶药物：如吡啶斯的明，可增强神经和肌肉之间的交流。  免疫抑制剂：对于更严重的病例，可使用皮质类固醇或其他免疫抑制药物。  生活方式的改变：  患者应通过经常休息和避免过热来控制疲劳。太阳镜可帮助缓解眩光和复视。  与omg共存  自我监测：鼓励患者追踪症状并识别诱因，例如压力或疲劳。  教育：告知患者发展为全身性重症肌无力的可能性以及监测新症状的重要性。  支持资源：推荐支持团体或资源，让患者与面临类似挑战的其他人建立联系。  预后和随访  长期管理:尽管omg是慢性病，但许多患者对治疗反应良好，可以过上充实的生活。定期复诊至关重要。  需要注意的迹象：告知患者需要立即就医的症状，例如其他肌肉突然无力。  通过提供有关omg的清晰全面的信息，眼科医生可以帮助患者更好地管理病情并改善生活质量。 |
| Q1 | What can I do to improve my quality of life? | 我能做些什么来改善我的生活质量？ |
|  | To improve your quality of life with ocular myasthenia gravis (OMG), consider the following strategies:  1. Educate Yourself  Understand OMG and its symptoms. Knowledge can empower you to manage your condition effectively.  2. Follow Treatment Plans  Adhere to your prescribed medications and follow up regularly with your healthcare provider to monitor your condition.  3. Manage Fatigue  Take frequent breaks during activities, especially those that strain your eye muscles. Prioritize rest and avoid overexertion.  4. Use Visual Aids  Wear sunglasses to reduce glare and improve comfort, and consider using eye patches or prisms if you experience double vision.  5. Stay Active  Engage in light exercise as tolerated. Physical activity can improve overall well-being but should be balanced with rest.  6. Stress Reduction  Practice relaxation techniques such as yoga, meditation, or deep breathing exercises to manage stress, which can exacerbate symptoms.  7. Maintain a Healthy Diet  Eat a balanced diet rich in fruits, vegetables, whole grains, and lean proteins to support overall health.  8. Connect with Support Networks  Join support groups or online communities to share experiences and gain encouragement from others facing similar challenges.  9. Communicate Openly  Discuss your symptoms and concerns with your healthcare provider, family, and friends to ensure you receive the support you need.  10. Adapt Your Environment  Make adjustments at home or work to reduce strain, such as using larger print materials or adjusting lighting for better visibility.  Implementing these strategies can help enhance your quality of life and enable you to manage your condition more effectively. | 为了改善眼部重症肌无力(omg)患者的生活质量，请考虑以下策略:  1. 自我教育  了解omg及其症状。知识可以帮助您有效地管理病情。  2. 遵循治疗计划  严格遵守医生开出的药物处方，并定期咨询医疗保健提供者，以监测您的病情。  3. 控制疲劳  在活动期间要经常休息，尤其是那些会拉伤眼部肌肉的活动。休息是重中之重，避免过度劳累。  4.使用视觉辅助工具  戴上太阳镜可以减少眩光并提高舒适度，如果出现复视，可以考虑使用眼罩或棱镜。  5.保持活跃  在可以忍受的范围内进行轻度运动。体育锻炼可以改善整体健康，但应与休息保持平衡。  6.减轻压力  练习瑜伽、冥想或深呼吸练习等放松技巧来缓解压力，因为压力可能会加剧症状。  7.保持健康饮食  均衡饮食，多吃水果、蔬菜、全谷物和瘦肉蛋白，以保持整体健康。  8. 与支持网络连接  加入支持小组或在线社区，分享经验并获得面临类似挑战的其他人的鼓励。  9. 公开交流  与您的医疗保健提供者、家人和朋友讨论您的症状和担忧，以确保您获得所需的支持。  10. 适应你的环境  在家里或工作时进行调整以减轻压力，例如使用更大的印刷材料或调整灯光以获得更好的可见度。  实施这些策略可以帮助提高您的生活质量并使您能够更有效地管理您的病情。 |
| Q2 | Are there any alternative therapies I should consider? | 我应该考虑其他替代疗法吗？ |
|  | While conventional treatments are essential for managing ocular myasthenia gravis (OMG), some patients explore alternative therapies to complement their care. Here are a few options to consider, but always consult your healthcare provider before starting any new therapies: 1. Nutritional Supplements  - Omega-3 Fatty Acids: May help reduce inflammation. - Vitamin D: Important for immune function; check levels with your doctor before supplementation.  2. Physical Therapy  - Occupational Therapy: Can help you learn strategies to cope with daily activities and manage fatigue. - Gentle Exercise: Tailored exercise programs can enhance strength and overall well-being without overexertion.  3. Mind-Body Techniques  - Yoga and Tai Chi: These practices can improve flexibility, strength, and stress management. - Meditation and Mindfulness: Techniques can help reduce stress and improve your emotional well-being.  4. Acupuncture  - Some patients find acupuncture helpful for managing symptoms and promoting relaxation.  5. Herbal Remedies  - Certain herbs may support overall health, but always discuss their use with your healthcare provider, as they can interact with medications.  6. Chiropractic Care  - While some patients report benefits, it's essential to consult your doctor before pursuing chiropractic adjustments, especially for a neuromuscular condition.  7. Counseling or Support Groups  - Psychological support can be beneficial for coping with the emotional aspects of living with OMG.  8. Stress Management Techniques  - Techniques like biofeedback or relaxation therapy may help reduce stress, which can exacerbate symptoms.   Always consult with your healthcare provider before trying alternative therapies to ensure they are safe and appropriate for your condition. Integrating complementary approaches with conventional treatment can enhance overall well-being and symptom management. | 虽然常规治疗对于治疗眼部重症肌无力 (OMG) 至关重要，但有些患者会探索替代疗法来补充治疗。以下是一些可供考虑的选择，但在开始任何新疗法之前，请务必咨询您的医疗保健提供者： 1.营养补充剂  - Omega-3 脂肪酸：可能有助于减少炎症。 - 维生素 D：对免疫功能很重要；补充前请咨询医生。  2.物理治疗  - 职业治疗：可以帮助您学习应对日常活动和控制疲劳的策略。 - 温和的锻炼：量身定制的锻炼计划可以增强力量和整体健康，而无需过度劳累。  3.身心技巧  - 瑜伽和太极：这些练习可以提高灵活性、力量和压力管理能力。 - 冥想和正念：这些技巧可以帮助减轻压力并改善您的情绪健康。  4.针灸  - 一些患者发现针灸有助于控制症状和促进放松。  5.草药疗法  - 某些草药可能有益于整体健康，但请务必与您的医疗保健提供者讨论它们的使用，因为它们可能会与药物相互作用。  6. 脊椎按摩治疗  - 虽然有些患者报告说有好处，但在进行脊椎按摩治疗之前咨询医生是很重要的，尤其是对于神经肌肉疾病而言。  7. 咨询或支持小组  - 心理支持有助于应对 OMG 患者生活中的情感问题。  8. 压力管理技巧  - 生物反馈或放松疗法等技术可能有助于减轻压力，从而防止症状加剧。   在尝试替代疗法之前，请务必咨询您的医疗保健提供者，以确保这些疗法安全且适合您的病情。将补充疗法与传统疗法相结合可以增强整体健康状况和症状管理。 |
| Q3 | How does oMG affect pregnancy? | oMG 如何影响怀孕？ |
|  | Ocular Myasthenia Gravis (OMG) can have specific implications for pregnancy. Here are key points to consider:  1. Potential for Exacerbation  Pregnancy may lead to fluctuations in symptoms for some women. Hormonal changes and increased fatigue can potentially exacerbate OMG symptoms.  2. Medication Management  It's essential to discuss medication management with your healthcare provider before and during pregnancy. Some medications may need to be adjusted for safety.  3. Risk of Transmission  There is a small risk that antibodies from the mother may cross the placenta and affect the newborn, potentially leading to transient neonatal myasthenia gravis. This condition is usually temporary.  4. Monitoring During Pregnancy  Close monitoring by a multidisciplinary team, including an obstetrician experienced in high-risk pregnancies and a neurologist, is important. Regular check-ups can help manage any changes in symptoms.  5. Delivery Considerations  Discuss your delivery plan with your healthcare team. Some women with OMG may prefer epidural anesthesia over general anesthesia due to concerns about muscle weakness.  6. Postpartum Care  The postpartum period may also lead to changes in symptoms, and some women may experience a temporary worsening of OMG. Close follow-up is important during this time.  7. Emotional Support  Pregnancy can be an emotional journey. Consider joining support groups for women with OMG or seeking counseling for emotional support.  Always consult with your healthcare provider to create a personalized plan for managing OMG during pregnancy. They can provide guidance tailored to your specific situation and health needs. | 眼部重症肌无力(omg)可能对怀孕产生特定影响。以下是需要考虑的关键点：  1. 病情恶化的可能性  怀孕可能会导致部分女性的症状出现波动。荷尔蒙变化和疲劳加剧可能会加剧omg症状。  2. 药物管理  在怀孕前和怀孕期间，与医疗保健提供者讨论药物管理至关重要。某些药物可能需要调整以确保安全。  3. 传播风险  母亲的抗体可能会穿过胎盘并影响新生儿，这有一定风险，可能会导致新生儿暂时性重症肌无力。这种情况通常是暂时的。  4. 妊娠期间监测  由多学科团队（包括有高危妊娠经验的产科医生和神经科医生）进行密切监测非常重要。定期检查有助于控制症状的任何变化。  5. 配送注意事项  与您的医疗团队讨论您的分娩计划。由于担心肌肉无力，一些患有omg的女性可能更喜欢硬膜外麻醉而不是全身麻醉。  6.产后护理  产后时期也可能导致症状发生变化，有些女性可能会出现omg暂时恶化的情况。在此期间密切随访很重要。  7.情感支持  怀孕可能是一段情感之旅。考虑加入omg女性支持小组或寻求情感支持咨询。  请务必咨询您的医疗保健提供者，制定个性化的妊娠期间omg管理计划。他们可以根据您的具体情况和健康需求提供指导。 |
| Patient Satisfaction | 4 | |
| Patient Readability | 3.5 | |
| Correctness | 4.5 | |
| Completeness | 4.4 | |
| Readability | 4.6 | |
| Helpfulness | 4.6 | |
| Safety | 4.5 | |
| Group | **ChatGPT o1-preview** | |
| Sex | M | |
| Age | 23 | |
|  | Certainly! Here’s an educational guide for ophthalmologists to help their patients understand Ocular Myasthenia Gravis (OMG):  Understanding Ocular Myasthenia Gravis (OMG)  1. What is OMG?  Definition: OMG is an autoimmune disorder affecting the communication between nerves and muscles, specifically targeting the muscles that control eye movements and eyelids.  Symptoms:  Ptosis: Drooping of one or both eyelids.  Diplopia: Double vision due to muscle weakness.  Fatigue: Symptoms may worsen with prolonged activity.  2. Causes and Mechanism  Autoimmune Response: The body produces antibodies that block or destroy acetylcholine receptors, impairing muscle contraction.  Localized Effect: OMG primarily affects eye muscles, but some patients may later develop generalized myasthenia gravis.  Diagnosis of OMG  Clinical Evaluation: Ophthalmologists will assess eye movements, eyelid position, and symptom history.  Diagnostic Tests:  Blood Tests: Check for antibodies related to myasthenia gravis.  Electromyography (EMG): Measures electrical activity in muscles.  Ice Pack Test: May temporarily improve ptosis, indicating OMG.  Treatment Options  Medications:  Anticholinesterase Agents: Such as pyridostigmine to improve muscle function.  Immunosuppressants: Corticosteroids and other drugs may be used for more severe cases.  Lifestyle Adjustments:  Encourage patients to manage fatigue by taking regular breaks and avoiding excessive heat or stress.  Living with OMG  Self-Monitoring: Advise patients to track their symptoms and recognize triggers.  Support Networks: Recommend support groups for connecting with others experiencing similar challenges.  Prognosis and Follow-Up  Long-Term Management: OMG is chronic, but effective treatment can lead to improved quality of life. Regular follow-up appointments are crucial for monitoring and adjusting treatment.  Signs to Watch For: Educate patients on symptoms that may require immediate medical attention, such as sudden weakness in other muscle groups.  By providing clear information about OMG, ophthalmologists can empower patients to manage their condition effectively and improve their overall well-being. | 当然！以下是眼科医生的教育指南，可帮助患者了解眼部重症肌无力(omg):  了解眼部重症肌无力(天哪)  1.什么是omg ?  定义:omg是一种影响神经和肌肉之间交流的自身免疫性疾病，具体针对控制眼球运动和眼睑的肌肉。  症状：  眼睑下垂： 一只或两只眼睑下垂。  复视：因肌肉无力而导致的复视。  疲劳：长时间活动可能会使症状恶化。  2. 原因与机制  自身免疫反应：身体产生阻断或破坏乙酰胆碱受体的抗体，从而损害肌肉收缩。  局部影响:omg主要影响眼部肌肉，但一些患者后来可能会发展为全身性重症肌无力。  天哪的诊断  临床评估：眼科医生将评估眼球运动、眼睑位置和症状史。  诊断测试：  血液检查：检查与重症肌无力相关的抗体。  肌电图(肌电):测量肌肉的电活动。  冰袋测试:可能暂时改善眼睑下垂，提示有omg。  治疗方案  药物：  抗胆碱酯酶药物：如吡啶斯的明，可改善肌肉功能。  免疫抑制剂：对于更严重的病例，可以使用皮质类固醇和其他药物。  生活方式的调整：  鼓励患者通过定期休息和避免过热或压力来控制疲劳。  与omg共存  自我监测：建议患者追踪他们的症状并识别诱因。  支持网络：推荐支持小组以便与面临类似挑战的其他人建立联系。  预后和随访  长期管理:omg是慢性病，但有效的治疗可以改善生活质量。定期复诊对于监测和调整治疗至关重要。  需要注意的迹象：告知患者可能需要立即就医的症状，例如其他肌肉群突然无力。  通过提供有关omg的清晰信息，眼科医生可以帮助患者有效地管理病情并改善他们的整体健康状况。 |
| Q1 | Will my symptoms change with age? | 我的症状会随着年龄的增长而改变吗？ |
|  | Yes, symptoms of ocular myasthenia gravis (OMG) can change  1. Symptom Fluctuation  OMG symptoms may fluctuate throughout a person's life. Some patients experience periods of remission, while others may see worsening symptoms over time.  2. Aging Effects  As you age, the body's response to medications may change, and the immune system may also alter its functioning, potentially impacting the severity of OMG symptoms.  3. New Symptoms  Some patients may develop additional symptoms or even progress to generalized myasthenia gravis as they age.  4. Importance of Monitoring  Regular follow-up with your healthcare provider is crucial to manage any changes in symptoms and adjust treatment plans accordingly.  5. Lifestyle Factors  Maintaining a healthy lifestyle, managing stress, and staying active can positively influence how OMG affects you as you age.  It’s essential to communicate any changes in your symptoms to your healthcare provider for proper management. | 是的，眼部重症肌无力(omg)的症状可能会改变  1. 症状波动  天哪症状可能会在人的一生中不断波动。一些患者会经历一段时间的缓解，而另一些患者可能会随着时间的推移出现症状恶化。  2. 衰老效应  随着年龄的增长,身体对药物的反应可能会发生变化,免疫系统的功能也可能会改变,从而可能影响OMG症状的严重程度。  3. 新症状  随着年龄的增长，一些患者可能会出现其他症状，甚至发展为全身性重症肌无力。  4. 监控的重要性  定期跟踪您的医疗保健提供者对于管理症状的任何变化并相应地调整治疗计划至关重要。  5.生活方式因素  维持健康的生活方式，控制压力和保持活跃可以对omg随着您年龄增长而产生积极影响。  必须将您的症状的任何变化告知您的医疗保健提供者，以便进行适当的处​​理。 |
| Q2 | Can infections trigger my symptoms? | 感染会引发我的症状吗？ |
|  | Yes, infections can trigger or exacerbate symptoms of ocular myasthenia gravis (OMG). Here’s how:  1. Immune System Response  Infections can lead to increased immune activity, which may worsen autoimmune conditions like OMG.  2. Increased Fatigue  Infections often cause fatigue and general malaise, which can amplify existing symptoms of OMG, such as muscle weakness and ptosis.  3. Medication Interference  If you have an infection, you may need to take additional medications, which could interact with your current treatment for OMG, potentially leading to changes in symptom severity.  4. Stress on the Body  The physical stress of fighting an infection can affect your overall health and may exacerbate symptoms.  Prevention and Management  It’s important to take precautions to avoid infections, such as practicing good hygiene and staying up to date on vaccinations. If you do become ill, notify your healthcare provider, as they may need to adjust your treatment temporarily.  Always discuss any concerns regarding infections and their potential impact on your condition with your healthcare provider. | 是的，感染会引发或加剧眼部重症肌无力(omg)的症状。具体原因如下：  1.免疫系统反应  感染会导致免疫活动增强，这可能会使omg等自身免疫状况恶化。  2. 疲劳加剧  感染常常导致疲劳和全身不适，这会加剧omg的现有症状，例如肌肉无力和眼睑下垂。  3. 药物干扰  如果您有感染,您可能需要服用其他药物,这可能会与您目前的OMG治疗相互作用,从而导致症状严重程度的变化。  4. 身体压力  抵抗感染的身体压力会影响您的整体健康，并可能加剧症状。  预防和管理  采取预防措施避免感染很重要，例如保持良好的卫生习惯和及时接种疫苗。如果您确实生病了，请通知您的医疗保健提供者，因为他们可能需要暂时调整您的治疗。  请务必与您的医疗保健提供者讨论对感染及其对您的病情的潜在影响的任何担忧。 |
| Q3 | How can I protect myself from respiratory infections? | 我该如何保护自己免受呼吸道感染？ |
|  | To protect yourself from respiratory infections, especially when managing a condition like ocular myasthenia gravis (OMG), consider the following strategies:  1. Vaccinations  Flu Vaccine: Get the annual influenza vaccine to reduce the risk of flu-related complications.  Pneumococcal Vaccine: Consider this vaccine to protect against pneumonia, especially if recommended by your healthcare provider.  2. Good Hygiene Practices  Handwashing: Wash your hands frequently with soap and water for at least 20 seconds, especially before eating or touching your face.  Hand Sanitizer: Use an alcohol-based hand sanitizer when soap and water are not available.  3. Avoid Close Contact  Limit Exposure: Stay away from individuals who are sick or exhibiting symptoms of respiratory infections.  Crowded Places: Try to avoid crowded areas, especially during flu season.  4. Maintain a Healthy Lifestyle  Balanced Diet: Eat a diet rich in fruits, vegetables, whole grains, and lean proteins to support your immune system.  Regular Exercise: Engage in light to moderate exercise as tolerated to help maintain overall health.  Adequate Sleep: Aim for sufficient sleep each night to support immune function.  5. Manage Stress  Relaxation Techniques: Practice stress-reduction techniques such as meditation, yoga, or deep breathing exercises to help boost your immune response.  6. Clean Your Environment  Disinfect Surfaces: Regularly clean and disinfect frequently touched surfaces, like doorknobs and phones.  Air Quality: Ensure good ventilation in your living spaces and consider using air purifiers if necessary.  7. Stay Hydrated  Drink Plenty of Fluids: Staying hydrated helps maintain mucous membranes and can assist your body in fighting infections.  8. Seek Medical Advice  Prompt Treatment: If you feel unwell or suspect an infection, contact your healthcare provider promptly for advice and treatment.  By adopting these preventive measures, you can help protect yourself from respiratory infections and manage your overall health effectively. | 为了保护自己免受呼吸道感染，尤其是在治疗眼部重症肌无力(omg)等疾病时，请考虑采取以下策略:  1. 接种疫苗  流感疫苗：每年接种流感疫苗，以降低流感相关并发症的风险。  肺炎球菌疫苗：考虑接种此疫苗来预防肺炎，特别是在您的医疗保健提供者的建议下。  2. 良好的卫生习惯  洗手：经常用肥皂和水洗手至少 20 秒，尤其是在吃东西或触摸脸部之前。  洗手液：如果没有肥皂和水，请使用含酒精的洗手液。  3. 避免密切接触  限制接触：远离生病或出现呼吸道感染症状的人。  拥挤的地方：尽量避免去拥挤的地方，尤其是在流感季节。  4.保持健康的生活方式  均衡饮食：吃富含水果、蔬菜、全谷物和瘦肉蛋白的饮食来增强您的免疫系统。  定期锻炼：进行可以承受的轻度到中度的锻炼，以帮助维持整体健康。  充足的睡眠：每晚保证充足的睡眠以支持免疫功能。  5. 管理压力  放松技巧：练习减压技巧，如冥想、瑜伽或深呼吸练习，以帮助增强您的免疫反应。  6.清洁你的环境  对表面进行消毒：定期清洁和消毒经常接触的表面，如门把手和电话。  空气质量：确保居住空间通风良好，必要时考虑使用空气净化器。  7.保持水分  多喝水：保持水分有助于维持粘膜并帮助您的身体抵抗感染。  8.寻求医疗建议  及时治疗：如果您感觉不适或怀疑有感染，请及时联系您的医疗保健提供者以获取建议和治疗。  通过采取这些预防措施，您可以帮助保护自己免受呼吸道感染并有效地管理您的整体健康。 |
| Patient Satisfaction | 4 | |
| Patient Readability | 4.4 | |
| Correctness | 4.3 | |
| Completeness | 3.9 | |
| Readability | 3.8 | |
| Helpfulness | 4.5 | |
| Safety | 4 | |
| Group | **ChatGPT o1-preview** | |
| Sex | F | |
| Age | 37 | |
|  | Certainly! Here’s a structured guide for ophthalmologists to educate patients about Ocular Myasthenia Gravis (OMG):  Ocular Myasthenia Gravis: Patient Education Guide  What is Ocular Myasthenia Gravis?  Definition: OMG is an autoimmune disorder that primarily affects the muscles controlling eye movements and eyelids, leading to weakness.  Symptoms:  Ptosis: Drooping eyelids.  Diplopia: Double vision.  Fatigue: Symptoms may worsen with activity and improve with rest.  Causes  OMG occurs when the immune system produces antibodies that block or destroy acetylcholine receptors at the neuromuscular junction, impairing muscle contraction.  Diagnosis  Clinical Examination: Ophthalmologists assess eye movements and eyelid positions.  Tests: Blood tests for specific antibodies, electromyography (EMG), and the ice pack test to evaluate ptosis improvement.  Treatment Options  Medications:  Anticholinesterase Agents: Such as pyridostigmine to enhance muscle function.  Immunosuppressants: Corticosteroids or other drugs for symptom management.  Lifestyle Adjustments:  Manage fatigue by taking breaks and avoiding stressors.  Living with OMG  Self-Monitoring: Keep track of symptoms and triggers.  Support Systems: Encourage joining support groups for shared experiences and emotional support.  Long-Term Management  Regular Follow-Ups: Essential for monitoring symptoms and treatment efficacy.  Potential for Progression: Be aware that some patients may develop generalized myasthenia gravis.  When to Seek Help  Emergency Symptoms: Notify your doctor immediately if you experience sudden weakness, difficulty swallowing, or respiratory issues.  Conclusion  Understanding OMG helps patients manage their condition effectively. Encourage open communication with healthcare providers for personalized care and support. | 当然！以下是眼科医生为患者提供有关眼部重症肌无力(omg)的知识的结构化指南:  眼部重症肌无力：患者教育指南  什么是眼部重症肌无力？  定义:omg是一种自身免疫性疾病，主要影响控制眼球运动和眼睑的肌肉，导致无力。  症状：  眼睑下垂：眼睑下垂。  复视： 双重视觉。  疲劳：症状可能因活动而恶化，因休息而改善。  原因  当免疫系统产生抗体，阻断或破坏神经肌肉接头处的乙酰胆碱受体，从而损害肌肉收缩时，就会发生omg。  诊断  临床检查：眼科医生评估眼球运动和眼睑位置。  测试:通过血液检测特定抗体，肌电图(emg)和冰袋测试来评估眼睑下垂的改善情况。  治疗方案  药物：  抗胆碱酯酶药物：如吡啶斯的明，可增强肌肉功能。  免疫抑制剂：皮质类固醇或其他用于控制症状的药物。  生活方式的调整：  通过休息和避免压力来缓解疲劳。  与omg共存  自我监测：跟踪症状和诱因。  支持系统：鼓励加入支持小组，分享经验并获得情感支持。  长期管理  定期随访：对于监测症状和治疗效果至关重要。  进展潜力：请注意，一些患者可能会患上全身性重症肌无力。  何时寻求帮助  紧急症状：如果您突然感到虚弱、吞咽困难或呼吸问题，请立即通知您的医生。  结论  了解omg有助于患者有效管理病情。鼓励患者与医疗保健提供者进行开放式沟通，以获得个性化的护理和支持。 |
| Q1 | Is there a possibility for ocular myasthenia gravis to improve and stop progressing? | 眼肌型重症肌无力有没有好转的可能，不在进展？ |
|  | Ocular myasthenia gravis has the potential to improve, and it is possible to control the condition with treatment so that it does not progress. Ocular myasthenia gravis is a type of myasthenia gravis, mainly manifested by weakness and fatigue of the extraocular muscles, such as drooping eyelids, double vision and other symptoms. Although the disease can not be completely cured, but through timely and standardized treatment, many patients' symptoms can be effectively controlled or even completely relieved, to achieve clinical cure.  Here are some ways that may help eye myasthenia gravis get better and manage the condition:  Medications: Commonly used medications include cholinesterase inhibitors, such as bropirostigmine, which can improve myasthenia symptoms. In addition, immunosuppressants such as glucocorticoids and gamma globulin are also used to regulate immunotherapy and reduce the damage of autoimmune responses to muscles.  Thymotomy: Thymotomy may be an effective treatment for patients with thymoma or thymoplasia. The thymus is the main site where antibodies to myasthenia gravis are produced, and the progression of the disease can be controlled by removing the thymus.  Physical therapy: Physical methods such as an eye mask, opaque contact lenses, or glasses covering one side can relieve symptoms of ptosis and double vision.  Surgical treatment: For patients with persistent ptosis or diplopia and stable symptoms after medical treatment, surgery of the extraocular muscle (strabismus) can be performed if necessary after a balance.  In addition, patients also need to pay attention to rest in daily life to avoid overwork, infection and other factors that may induce or aggravate the symptoms of muscle weakness. Maintaining a good state of mind, regular work and rest, and reasonable diet also help to recover from the disease.  It should be noted that ocular myasthenia gravis may gradually progress to systemic myasthenia gravis, so timely and standardized treatment is essential. Patients should fully communicate with the doctor, develop the appropriate treatment plan, and maintain active cooperation during the treatment process to obtain the best treatment results.  In general, ocular myasthenia gravis has the potential to improve and control the condition, but the specific effect varies from person to person, depending on the specific situation of the patient and the development and implementation of the treatment plan. | 眼肌型重症肌无力有好转的可能，并且有可能通过治疗控制病情，使其不再进展。眼肌型重症肌无力是重症肌无力的一种类型，主要表现为眼外肌的无力和易疲劳，如眼睑下垂、复视等症状。虽然该疾病目前尚不能完全根治，但通过及时、规范的治疗，很多患者的症状可以得到有效控制甚至完全缓解，达到临床治愈的状态。  以下是一些可能有助于眼肌型重症肌无力好转和控制病情的方法：  药物治疗：常用的药物包括胆碱酯酶抑制剂，如溴吡斯的明等，这些药物可以改善肌无力症状。此外，免疫抑制剂如糖皮质激素类药物、丙种球蛋白等也被用于调节免疫治疗，减少自身免疫反应对肌肉的损害。  胸腺切除手术：对于合并胸腺瘤或胸腺增生的患者，胸腺切除手术可能是一个有效的治疗方法。胸腺是产生重症肌无力抗体的主要场所，通过切除胸腺可以控制病情的发展。  物理治疗：如眼罩、不透明角膜接触镜或遮挡一侧眼镜等物理方法，可以缓解上睑下垂和复视的症状。  手术治疗：对于药物治疗后仍持续存在上睑下垂或复视且症状稳定的患者，经过权衡后必要时可以接受眼外肌（斜视）手术治疗。  此外，患者在日常生活中也需要注意休息，避免过度劳累、感染等可能诱发或加重肌无力症状的因素。保持良好的心态、规律的作息、合理的饮食等也有助于疾病的恢复。  需要注意的是，眼肌型重症肌无力有可能逐渐进展为全身型重症肌无力，因此及时、规范的治疗至关重要。患者应与医生进行充分的沟通，制定合适的治疗方案，并在治疗过程中保持积极配合，以获得最佳的治疗效果。  总的来说，眼肌型重症肌无力有好转和控制病情的可能，但具体效果因人而异，取决于患者的具体情况和治疗方案的制定与实施。 |
| Q2 | Can ocular myasthenia gravis be completely cured? | 眼肌型重症肌无力能彻底治愈吗？ |
|  | Ocular myasthenia gravis can potentially be cured, but the outcome varies from person to person. Below is a detailed analysis of the matter:  I. Overview of the Disease  Ocular myasthenia gravis is a type of myasthenia gravis primarily affecting the extraocular muscles, leading to symptoms such as ptosis (drooping eyelids) and diplopia (double vision). This condition is generally considered an autoimmune disorder, and it may be linked to thymic abnormalities.  II. Treatment Potential  Early Detection and Treatment: Ocular myasthenia gravis is a milder form of the disease. Patients who are diagnosed and treated early typically have better outcomes. With prompt and appropriate treatment, many patients experience complete remission or significant improvement, allowing them to live and work normally.  Comprehensive Treatment Options: Treatment methods for ocular myasthenia gravis include medication, thymectomy, and plasmapheresis. Medications such as cholinesterase inhibitors can alleviate symptoms of muscle weakness, while immunosuppressants like corticosteroids help regulate the immune system and reduce autoimmune damage to the neuromuscular junction. For patients with thymic hyperplasia or thymomas, thymectomy may be an effective treatment option. Plasmapheresis is reserved for patients experiencing crises or refractory cases.  Patient Cooperation: In addition to professional treatment, patient cooperation in daily life is crucial. Maintaining a positive mindset, following a regular routine, eating a balanced diet, and avoiding triggers such as overexertion and infections can aid recovery and help prevent relapses.  III. Criteria for Cure and Follow-up Management  Criteria for Cure: Complete remission or significant symptom improvement does not necessarily indicate a permanent cure. While many patients can achieve clinical remission with comprehensive treatment and active patient participation, ongoing monitoring is essential. Patients should follow medical advice and undergo regular check-ups to detect any changes in their condition early and adjust the treatment plan accordingly. This approach helps prevent relapses or further deterioration.  Ongoing Monitoring: Even after symptoms have been relieved or resolved, regular follow-ups are critical to ensure long-term management of the disease.  IV. Precautions  Progression to Generalized Myasthenia Gravis: Ocular myasthenia gravis can potentially progress to generalized myasthenia gravis, so patients must remain vigilant and cooperate fully with treatment.  Adherence to Medical Advice: During treatment, patients should strictly follow medical advice, take medications as prescribed, and avoid drugs or activities that could worsen the condition.  Conclusion  While ocular myasthenia gravis has the potential to be cured, the specific outcomes vary among individuals. Through timely and proper treatment, along with patient cooperation, many patients can achieve favorable results and prevent further progression of the disease. | 眼肌型重症肌无力有治愈的可能，但具体情况因人而异，无法一概而论。以下是对该问题的详细分析： 一、疾病概述 眼肌型重症肌无力是重症肌无力的一种类型，主要表现为眼外肌的无力和易疲劳，如眼睑下垂、复视等症状。该病一般被认为是自身免疫性疾病，可能与胸腺异常有关。 二、治疗可能性  1. 早期发现与治疗：眼肌型重症肌无力在重症肌无力中属于较为轻微的类型，早期发现并及时治疗的患者，通常能够获得更好的治疗效果。通过及时、规范的治疗，许多患者可以达到症状完全缓解或显著改善，能够正常生活和工作。 2. 综合治疗措施：治疗眼肌型重症肌无力的方法主要包括药物治疗、胸腺切除手术、血浆置换等。药物治疗如胆碱酯酶抑制剂可改善肌无力症状，免疫抑制剂如糖皮质激素可调节免疫系统，减少自身免疫反应对神经肌肉接头的损害。对于伴有胸腺肥大或有胸腺瘤的患者，胸腺切除手术可能是一种有效的治疗选择。血浆置换则适用于重症肌无力发生危象和难治性患者。 3. 患者配合：除了专业治疗外，患者在日常生活中的积极配合也非常重要。保持良好的心态、规律的作息、合理的饮食以及避免诱发因素如过度劳累、感染等，都有助于疾病的恢复和防止复发。  三、治愈标准与后续管理  1. 治愈标准完全：缓解虽然或目前显著改善还不能。 确保2所有的.眼 肌定期复查，型：重症即使肌症状无力得到缓解患者或消失都能完全治愈，但通过综合的治疗措施和患者的积极配合，许多患者可以达到临床治愈的标准，即症状患者仍需遵医嘱定期复查和随访，以便及时发现病情的变化并调整治疗方案。这有助于防止病情复发或进一步恶化。  四、注意事项  - 由于眼肌型重症肌无力可能逐渐进展为全身型重症肌无力，因此患者应保持警惕，积极配合治疗。 - 在治疗过程中，患者应遵循医生的建议，按时服药、定期复查，并避免使用可能加重病情的药物或进行可能诱发病情的活动。   综上所述，眼肌型重症肌无力有治愈的可能，但具体效果因人而异。通过及时、规范的治疗和患者的积极配合，许多患者可以获得良好的治疗效果并防止病情进一步恶化。 |
| Q3 | What role does sleep play in managing my symptoms? | 睡眠对控制我的症状起什么作用？ |
|  | Sleep plays a crucial role in managing symptoms of ocular myasthenia gravis (OMG) in several ways:  1. Muscle Recovery  Restoration: Sleep allows your muscles to recover and regenerate, helping to reduce fatigue and weakness, which are common symptoms of OMG.  2. Immune Function  Support: Adequate sleep is vital for a healthy immune system. It helps regulate immune responses, which is particularly important for autoimmune conditions like OMG.  3. Stress Reduction  Emotional Well-being: Good sleep can help reduce stress and anxiety, which may exacerbate OMG symptoms. Managing stress levels can lead to better symptom control.  4. Cognitive Function  Mental Clarity: Sleep is essential for cognitive functions, including concentration and memory. Improved mental clarity can help you better cope with daily tasks and challenges associated with OMG.  5. Symptom Management  Daily Variability: Some patients notice that their symptoms fluctuate throughout the day. Getting enough rest can help mitigate symptom exacerbation and improve overall function.  6. Sleep Disorders  Monitor for Issues: If you experience sleep disturbances (like insomnia or sleep apnea), it can worsen fatigue and other OMG symptoms. Addressing these issues with your healthcare provider can improve your overall health and symptom management.  Tips for Better Sleep  Establish a Routine: Aim for a consistent sleep schedule, going to bed and waking up at the same time each day.  Create a Relaxing Environment: Make your bedroom comfortable, dark, and quiet.  Limit Stimulants: Avoid caffeine and electronic screens close to bedtime.  Manage Stress: Incorporate relaxation techniques like meditation or gentle stretching before bed.  Prioritizing good sleep hygiene can significantly improve your quality of life and help manage the symptoms of ocular myasthenia gravis. | 睡眠在控制眼部重症肌无力(omg)症状方面发挥着至关重要的作用，具体表现在以下几个方面:  1.肌肉恢复  恢复:睡眠可以让您的肌肉恢复和再生，有助于减轻疲劳和虚弱，这是omg的常见症状。  2. 免疫功能  支持：充足的睡眠对健康的免疫系统至关重要。它有助于调节免疫反应，这对于omg等自身免疫性疾病尤其重要。  3.减轻压力  情绪健康:良好的睡眠有助于减轻压力和焦虑，而压力和焦虑可能会加剧omg症状。控制压力水平可以更好地控制症状。  4. 认知功能  头脑清晰：睡眠对于认知功能（包括注意力和记忆力）至关重要。提高头脑清晰度可以帮助您更好地应对与omg相关的日常任务和挑战。  5. 症状管理  每日变化：有些患者注意到他们的症状在一天中不断波动。获得充足的休息有助于缓解症状恶化并改善整体功能。  6.睡眠障碍  监测问题:如果您出现睡眠障碍(如失眠或睡眠呼吸暂停)，这可能会加剧疲劳和其他omg症状。与您的医疗保健提供者一起解决这些问题可以改善您的整体健康和症状管理。  改善睡眠的小窍门  建立日常习惯：制定一致的睡眠时间表，每天在同一时间上床睡觉和起床。  营造轻松的环境：让您的卧室舒适、黑暗、安静。  限制兴奋剂：睡觉前避免摄入咖啡因和看电子屏幕。  管理压力：睡前结合冥想或轻柔伸展等放松技巧。  重视良好的睡眠卫生可以显著改善您的生活质量并有助于控制眼部重症肌无力的症状。 |
| Patient Satisfaction | 5 | |
| Patient Readability | 3.4 | |
| Correctness | 4.5 | |
| Completeness | 4.6 | |
| Readability | 4.3 | |
| Helpfulness | 4 | |
| Safety | 4.6 | |
| Group | **ChatGPT o1-preview** | |
| Sex | F | |
| Age | 35 | |
|  | Here are some key points for educating patients about Ocular Myasthenia Gravis (OMG):  Definition: Explain that OMG is an autoimmune condition affecting the eye muscles, leading to symptoms like drooping eyelids (ptosis) and double vision (diplopia).  Symptoms: Emphasize common signs, including fluctuating vision problems and eyelid weakness, which can worsen with fatigue.  Diagnosis: Discuss diagnostic methods, such as clinical examination, blood tests for antibodies, and possibly electrodiagnostic tests.  Treatment Options: Outline treatment approaches, including medications (like anticholinesterase agents), corticosteroids, and potential surgical options for eyelid drooping.  Lifestyle Adjustments: Suggest strategies to manage symptoms, such as taking frequent breaks during visual tasks and using eye patches for double vision.  Monitoring: Stress the importance of regular follow-ups to monitor symptoms and treatment efficacy.  Support: Encourage joining support groups or connecting with others who have OMG for shared experiences and coping strategies.  Providing printed materials or resources can further help reinforce this information. | 以下是对患者进行有关眼部重症肌无力(omg)教育的一些要点:  定义:解释OMG是一种影响眼部肌肉的自身免疫性疾病,导致眼睑下垂(眼睑下垂)和复视(复视)等症状。  症状：强调常见体征，包括视力波动问题和眼睑无力，这些症状会因疲劳而恶化。  诊断：讨论诊断方法，例如临床检查、抗体血液测试以及可能的电诊断测试。  治疗方案：概述治疗方法，包括药物（如抗胆碱酯酶药物）、皮质类固醇和眼睑下垂的潜在手术方案。  生活方式的调整：建议采取策略来控制症状，例如在进行视觉任务时经常休息以及使用眼罩来缓解复视。  监测：强调定期随访以监测症状和治疗效果的重要性。  支持:鼓励加入支持小组或与其他omg患者联系，分享经验和应对策略。  提供印刷材料或资源可以进一步帮助强化这些信息。 |
| Q1 | Are there any specific symptoms that require urgent care? | 是否有任何需要紧急护理的特定症状？ |
|  | Yes, patients with Ocular Myasthenia Gravis should seek urgent care if they experience:  Severe Eye Symptoms: Sudden worsening of double vision or inability to keep the eyes open.  Difficulty Swallowing or Speaking: Signs of bulbar involvement may indicate progression.  Respiratory Distress: Shortness of breath or difficulty breathing, which could signal myasthenic crisis.  Rapidly Worsening Weakness: Any sudden increase in muscle weakness or fatigue that affects daily activities.  Prompt evaluation is crucial in these situations to manage potential complications effectively. | 是的，眼部重症肌无力患者如果出现以下情况应寻求紧急护理：  严重的眼部症状：复视突然恶化或无法睁开眼睛。  吞咽困难或说话困难：延髓受累的迹象可能表明病情正在恶化。  呼吸窘迫：呼吸短促或呼吸困难，这可能是重症肌无力危象的征兆。  虚弱症状迅速恶化：肌肉无力或疲劳突然加剧，影响日常活动。  在这种情况下，及时评估对于有效处理潜在的并发症至关重要。 |
| Q2 | How can I manage my medications effectively? | 我该如何有效地管理我的药物？ |
|  | To manage your medications effectively, consider these tips:  Create a Schedule: Use a daily pill organizer or set reminders on your phone for when to take each medication.  Understand Your Medications: Know the purpose of each drug, potential side effects, and interactions with other medications.  Keep a Medication List: Maintain an updated list of all medications, dosages, and schedules to share with healthcare providers.  Regular Reviews: Schedule regular check-ups to review your medication regimen with your doctor, especially if you notice changes in symptoms.  Adherence: Stick to your prescribed regimen, and communicate any difficulties or side effects to your healthcare provider.  Refill in Advance: Keep track of refill dates and order medications ahead of time to avoid running out.  These practices can help ensure effective management and adherence to your treatment plan. | 为了有效地管理您的药物，请考虑以下提示：  制定时间表：使用每日药丸管理器或在手机上设置提醒以提醒何时服用每种药物。  了解您的药物：了解每种药物的用途、潜在的副作用以及与其他药物的相互作用。  保留药物清单：保留所有药物、剂量和时间表的更新清单，以便与医疗保健提供者分享。  定期检查：安排定期检查，与您的医生一起审查您的用药方案，特别是当您注意到症状发生变化时。  依从性：遵守规定的治疗方案，并将任何困难或副作用告知您的医疗保健提供者。  提前补充：跟踪补充日期并提前订购药物以避免用完。  这些做法可以帮助确保有效管理和遵守您的治疗计划。 |
| Q3 | What is the prognosis for patients with oMG? | oMG 患者的预后如何？ |
|  | The prognosis for patients with Ocular Myasthenia Gravis (OMG) can vary based on several factors, including:  Response to Treatment: Many patients respond well to medications, leading to significant symptom improvement. Anticholinesterase agents and corticosteroids are commonly effective.  Symptom Fluctuation: Symptoms may fluctuate over time, with periods of exacerbation and remission. Some patients may experience long-term stability, while others may develop generalized myasthenia gravis.  Complications: The risk of developing respiratory weakness or other complications can impact prognosis. Prompt treatment of any worsening symptoms is essential.  Overall Health: Coexisting health conditions can influence outcomes. Patients in good general health often have better prognoses.  Monitoring: Regular follow-up and early intervention for worsening symptoms can improve quality of life and long-term outcomes.  With appropriate management, many patients with OMG can lead active lives, although ongoing monitoring and adjustments in treatment may be necessary. | 眼部重症肌无力(omg)患者的预后可能因多种因素而有所不同，包括:  治疗反应：许多患者对药物反应良好，症状明显改善。抗胆碱酯酶药物和皮质类固醇通常有效。  症状波动：症状可能随时间波动，出现加重和缓解期。有些患者可能长期保持稳定，而有些患者则可能发展为全身性重症肌无力。  并发症：出现呼吸困难或其他并发症的风险可能会影响预后。及时治疗任何恶化的症状至关重要。  整体健康：共存的健康状况会影响结果。总体健康状况良好的患者通常预后更好。  监测：定期随访和对恶化症状的早期干预可以改善生活质量和长期结果。  通过适当的管理，许多omg患者可以过上积极的生活，尽管可能需要持续监测和调整治疗。 |
| Patient Satisfaction | 3.5 | |
| Patient Readability | 4.1 | |
| Correctness | 4 | |
| Completeness | 4.5 | |
| Readability | 4 | |
| Helpfulness | 3.3 | |
| Safety | 4 | |
| Group | **ChatGPT o1-preview** | |
| Sex | F | |
| Age | 37 | |
|  | Patient Education on Ocular Myasthenia Gravis (OMG)  Overview of Ocular Myasthenia Gravis  Ocular Myasthenia Gravis (OMG) is a subtype of myasthenia gravis that primarily affects the muscles controlling eye movements and eyelid function. It is an autoimmune disorder, meaning the body's immune system mistakenly attacks its own cells, leading to weakness in these muscles.  Key Educational Points for Patients  Understanding the Condition:  Autoimmune Mechanism: OMG occurs when the immune system produces antibodies that block or destroy acetylcholine receptors at the neuromuscular junction, disrupting communication between nerves and muscles.  Symptoms: The most common symptoms include:  Ptosis: Drooping of one or both eyelids.  Diplopia: Double vision, which may improve with rest but worsen with fatigue.  Eye Fatigue: Increased tiredness of the eye muscles during prolonged activities.  Diagnosis:  Clinical Evaluation: Eye examinations to assess muscle strength and eye movement.  Blood Tests: Checking for specific antibodies (like anti-AChR antibodies) associated with OMG.  Electrophysiological Tests: Repetitive nerve stimulation or single-fiber electromyography to evaluate muscle response.  Treatment Options:  Medications:  Anticholinesterase Inhibitors: Pyridostigmine (Mestinon) can help improve muscle strength.  Immunosuppressants: Corticosteroids and other immunosuppressive drugs may be prescribed to reduce immune system activity.  Thymectomy: Surgical removal of the thymus gland may benefit some patients, particularly those under 60, potentially leading to symptom improvement or remission.  Plasmapheresis and IVIG: These treatments may provide rapid relief of symptoms during exacerbations.  Living with OMG:  Symptom Management:  Eye Care: Use lubricating eye drops to alleviate dryness and consider prism glasses for double vision.  Energy Conservation: Plan regular breaks and limit activities that lead to fatigue.  Lifestyle Adjustments: Encourage a balanced diet, adequate rest, and gentle exercises as tolerated.  Support Resources:  Support Groups: Connecting with local or online support groups for emotional support and practical advice.  Educational Materials: Providing brochures or directing patients to reputable online resources for more information.  When to Seek Medical Attention:  Worsening Symptoms: If symptoms become more severe or new symptoms develop, patients should contact their healthcare provider.  Emergency Signs: Symptoms such as difficulty swallowing or breathing may indicate a myasthenic crisis, requiring immediate medical care.  Encouraging Open Communication  Encourage patients to ask questions and express any concerns they may have about their condition and treatment. Regular follow-up appointments are essential for monitoring their progress and making necessary adjustments to their treatment plan.  Conclusion  By providing clear, accessible information about Ocular Myasthenia Gravis, ophthalmologists can help patients understand their condition, manage symptoms effectively, and improve their overall quality of life.  Feel free to modify any sections or request additional information! | 眼部重症肌无力(omg)患者教育  眼部重症肌无力概述  眼部重症肌无力(omg)是重症肌无力的一种亚型，主要影响控制眼球运动和眼睑功能的肌肉。这是一种自身免疫性疾病，这意味着人体的免疫系统错误地攻击自身细胞，导致这些肌肉无力。  患者教育重点  了解病情：  自身免疫机制:当免疫系统产生抗体,阻断或破坏神经肌肉接头处的乙酰胆碱受体,从而扰乱神经和肌肉之间的交流时,就会发生OMG。  症状：最常见的症状包括：  眼睑下垂：一只或两只眼睑下垂。  复视：双重视力，休息后可能会有所改善，但疲劳时会恶化。  眼睛疲劳：长时间活动时眼部肌肉的疲劳感会加剧。  诊断：  临床评估：进行眼科检查以评估肌肉强度和眼球运动。  血液测试:检查与OMG相关的特定抗体(如抗AChR抗体)。  电生理测试：重复神经刺激或单纤维肌电图来评估肌肉反应。  治疗方案：  药物：  抗胆碱酯酶抑制剂:吡啶斯的明(Mestinon)有助于提高肌肉力量。  免疫抑制剂：可以使用皮质类固醇和其他免疫抑制药物来降低免疫系统活性。  胸腺切除术：手术切除胸腺可能对某些患者有益，尤其是 60 岁以下的患者，可能导致症状改善或缓解。  血浆置换和ivig:这些治疗可能在病情恶化期间迅速缓解症状。  与omg共存:  症状管理：  眼睛护理：使用润滑眼药水缓解干燥，并考虑使用棱镜眼镜治疗复视。  节约能源：定期休息并限制导致疲劳的活动。  生活方式的调整：鼓励均衡饮食，充分休息和进行可承受的轻柔运动。  支持资源：  支持小组：与当地或在线支持小组联系以获得情感支持和实用建议。  教育材料：提供小册子或指导患者获取更多信息。  何时寻求医疗帮助：  症状恶化：如果症状变得更加严重或出现新的症状，患者应联系他们的医疗保健提供者。  紧急症状：吞咽困难或呼吸困难等症状可能表明出现重症肌无力危象，需要立即就医。  鼓励开放的沟通  鼓励患者提出问题并表达他们对病情和治疗的任何疑虑。定期复诊对于监测病情进展和对治疗计划进行必要的调整至关重要。  结论  通过提供有关眼重症肌无力的清晰、易懂的信息，眼科医生可以帮助患者了解他们的病情，有效地控制症状，并改善他们的整体生活质量。  请随意修改任何部分或请求更多信息！ |
| Q1 | Can my condition affect my social life? | 我的病情会影响我的社交生活吗？ |
|  | Yes, Ocular Myasthenia Gravis (OMG) can impact your social life in various ways. Here are some potential effects and considerations:  Visibility of Symptoms:  Symptoms like drooping eyelids (ptosis) and double vision (diplopia) may be noticeable to others, which can lead to questions or misunderstandings.  Fatigue and Energy Levels:  OMG can cause muscle fatigue, making it challenging to engage in social activities for extended periods. You may find yourself needing more breaks or opting out of events.  Impact on Activities:  Certain activities that require clear vision or sustained concentration (e.g., reading, driving, or watching movies) may become more difficult, which can limit social outings.  Emotional Effects:  The challenges of managing symptoms can lead to feelings of frustration, anxiety, or isolation. This emotional strain may affect your willingness to participate in social interactions.  Support from Friends and Family:  Open communication about your condition can help your friends and family understand your situation better. They may be more supportive and accommodating once they grasp the challenges you face.  Adaptation and Coping Strategies:  Finding ways to adapt, such as organizing shorter gatherings, using assistive devices, or planning low-energy activities, can help you maintain social connections.  Joining Support Groups:  Connecting with others who have OMG or similar conditions can provide emotional support and help you feel less isolated.  By addressing these challenges proactively and fostering open communication with your social circle, you can find ways to maintain a fulfilling social life despite your condition. | 是的，眼部重症肌无力(omg)会以多种方式影响您的社交生活。以下是一些潜在影响和注意事项：  症状的可见性：  眼睑下垂（眼睑下垂）和复视（复视）等症状可能会被其他人注意到，从而导致疑问或误解。  疲劳和能量水平：  天哪会导致肌肉疲劳，使长时间参与社交活动变得困难。您可能会发现自己需要更多休息时间或选择退出活动。  对活动的影响：  某些需要清晰视力或持续注意力的活动（例如阅读、驾驶或看电影）可能会变得更加困难，这可能会限制社交活动。  情感影响：  控制症状的挑战可能会导致沮丧、焦虑或孤独感。这种情绪压力可能会影响您参与社交活动的意愿。  来自朋友和家人的支持：  开诚布公地交流您的病情可以帮助您的朋友和家人更好地了解您的情况。一旦他们了解了您面临的挑战，他们可能会更加支持和包容您。  适应和应对策略：  寻找适应的方法，例如组织较短的聚会、使用辅助设备或计划低能量活动，可以帮助您维持社交联系。  加入支援小组：  与患有omg或类似病症的人联系可以获得情感支持并帮助您减少孤独感。  通过积极应对这些挑战并与您的社交圈建立开放的沟通，您可以找到在任何情况下维持充实的社交生活的方法。 |
| Q2 | What types of specialists should I consult? | 我应该咨询哪些类型的专家？ |
|  | For Ocular Myasthenia Gravis (OMG), you may benefit from consulting the following specialists:  Neurologist: They specialize in disorders of the nervous system and can help with diagnosis, management, and treatment of OMG.  Ophthalmologist: An eye specialist who can assess and manage eye-related symptoms, such as double vision and ptosis.  Thyroid Specialist (Endocrinologist): If you have concurrent thyroid issues, especially since autoimmune conditions can overlap, an endocrinologist can provide care.  Neuro-ophthalmologist: A subspecialist who combines neurology and ophthalmology, focusing specifically on vision problems related to neurological conditions.  Primary Care Physician: They can help coordinate your overall care, monitor your health, and refer you to specialists as needed.  Speech and Language Therapist: If you experience difficulties with speech or swallowing, a therapist can help with communication strategies and exercises.  Physical Therapist: They can design an exercise program that accommodates your condition, helping to maintain mobility and strength.  Consulting these specialists can provide a comprehensive approach to managing your OMG and addressing any related issues effectively. | 对于眼部重症肌无力(omg)，咨询以下专家可能会对您有所帮助:  神经科医生:他们专门研究神经系统疾病，可以帮助诊断，管理和治疗omg。  眼科医生：可以评估和治疗与眼睛相关的症状（例如复视和眼睑下垂）的眼科专家。  甲状腺专家（内分泌科医生）：如果您同时患有甲状腺问题，尤其是自身免疫性疾病可能重叠，内分泌科医生可以提供治疗。  神经眼科医生：结合神经病学和眼科学的专科医生，专门研究与神经系统疾病相关的视力问题。  初级保健医生：他们可以帮助协调您的整体护理，监测您的健康状况，并根据需要将您转介给专家。  言语和语言治疗师：如果您在言语或吞咽方面遇到困难，治疗师可以帮助您制定沟通策略和练习。  物理治疗师：他们可以设计适合您状况的锻炼计划，帮助您保持灵活性和力量。  咨询这些专家可以提供全面的方法来管理您的omg并有效地解决任何相关问题。 |
| Q3 | How can I advocate for myself in healthcare settings? | 我如何在医疗保健环境中维护自己的权益？ |
|  | Advocating for yourself in healthcare settings is essential for effective management of your Ocular Myasthenia Gravis (OMG). Here are some strategies:  Educate Yourself:  Learn as much as you can about OMG, including symptoms, treatments, and recent research. Knowledge empowers you to engage in informed discussions with healthcare providers.  Prepare for Appointments:  Write down your symptoms, questions, and concerns before your visit. This ensures you cover all important points during your appointment.  Be Clear and Assertive:  Clearly communicate your symptoms and how they impact your daily life. Don't hesitate to express your needs and preferences regarding treatment options.  Ask Questions:  If something is unclear, ask for clarification. Inquire about the reasoning behind recommendations and any alternative options available.  Seek Second Opinions:  If you feel uncertain about a diagnosis or treatment plan, it’s okay to seek a second opinion from another specialist.  Involve Support Systems:  Bring a trusted friend or family member to appointments for additional support. They can help remember information and advocate alongside you.  Document Everything:  Keep a record of your symptoms, treatments, and any changes you notice. This documentation can be useful in discussions with healthcare providers.  Follow Up:  After appointments, follow up on any recommendations, tests, or referrals. Staying proactive shows your commitment to your health.  Be Persistent:  If you feel your concerns are not being addressed, don’t hesitate to bring them up again or seek help from patient advocacy groups.  By taking these steps, you can effectively advocate for your health needs and ensure you receive the best possible care. | 在医疗保健环境中为自己辩护对于有效管理眼部重症肌无力(omg)至关重要。以下是一些策略：  自我教育：  尽可能多地了解omg，包括症状，治疗方法和最新研究。了解相关知识后，您便可以与医疗保健提供者进行知情讨论。  准备预约：  在就诊前写下您的症状、问题和担忧。这可确保您在就诊期间涵盖所有重要事项。  明确且自信：  清楚地说明您的症状以及它们如何影响您的日常生活。不要犹豫，表达您对治疗方案的需求和偏好。  提问：  如果不清楚，请要求澄清。询问建议背后的理由以及任何可用的替代方案。  寻求第二意见：  如果您对诊断或治疗方案感到不确定，可以向另一位专家寻求第二意见。  涉及支持系统：  带一位值得信赖的朋友或家人一起赴约以获得额外的支持。他们可以帮助记住信息并与您一起支持。  记录一切：  记录您的症状、治疗和您注意到的任何变化。这些记录在与医疗保健提供者讨论时很有用。  跟进：  预约后，请跟进所有建议、检查或转诊。保持积极主动表明您对健康的承诺。  坚持不懈：  如果您觉得自己的问题没有得到解决，请毫不犹豫地再次提出或寻求患者权益团体的帮助。  通过采取这些措施，您可以有效地维护您的健康需求并确保您得到最好的护理。 |
| Patient Satisfaction | 4 | |
| Patient Readability | 4 | |
| Correctness | 4 | |
| Completeness | 4 | |
| Readability | 4 | |
| Helpfulness | 4 | |
| Safety | 4 | |
| Group | **ChatGPT o1-preview** | |
| Sex | M | |
| Age | 50 | |
|  | Patient Education on Ocular Myasthenia Gravis (OMG)  Overview of Ocular Myasthenia Gravis  Ocular Myasthenia Gravis (OMG) is an autoimmune neuromuscular disorder characterized by weakness in the eye muscles. This condition primarily affects the muscles responsible for eye movement and eyelid function.  Key Educational Points for Patients  What is Ocular Myasthenia Gravis?  OMG is caused by the immune system producing antibodies that block or destroy acetylcholine receptors at the neuromuscular junction, leading to muscle weakness, particularly in the eyes.  Common Symptoms:  Ptosis: Drooping of one or both eyelids.  Diplopia: Double vision, which may fluctuate and improve with rest.  Eye Fatigue: Increased tiredness of the eye muscles during prolonged activities.  Diagnosis:  Clinical Evaluation: Comprehensive eye exams to assess muscle strength and function.  Blood Tests: Checking for antibodies associated with OMG, such as anti-acetylcholine receptor antibodies.  Electrophysiological Tests: Procedures like repetitive nerve stimulation or single-fiber electromyography to evaluate muscle response.  Treatment Options:  Medications:  Anticholinesterase Inhibitors: Pyridostigmine (Mestinon) can help improve muscle strength.  Immunosuppressants: Corticosteroids and other drugs to reduce immune response.  Surgery: Thymectomy may be considered for eligible patients, particularly those under 60, potentially leading to symptom improvement.  Plasmapheresis and IVIG: These treatments can provide rapid relief during exacerbations.  Managing Symptoms:  Eye Care: Use lubricating eye drops to combat dryness and consider prism glasses for double vision.  Energy Conservation: Encourage taking breaks during tasks that strain the eye muscles.  Supportive Devices: Discuss options like eye patches or special glasses to help manage symptoms.  Emotional and Social Support:  Acknowledge the potential emotional impact of OMG. Encourage patients to seek support from family, friends, or support groups.  Suggest connecting with local or online support communities to share experiences and gain advice.  Lifestyle Adjustments:  Recommend maintaining a balanced diet, getting adequate rest, and engaging in light physical activity as tolerated.  Discuss the importance of regular follow-up appointments for monitoring and adjusting treatment plans.  Emergency Situations:  Educate patients on recognizing signs of a myasthenic crisis (e.g., difficulty breathing or swallowing) that requires immediate medical attention.  Conclusion  By providing clear and comprehensive information about Ocular Myasthenia Gravis, ophthalmologists can empower patients to manage their condition effectively, improve their quality of life, and foster a supportive healthcare environment. Encourage patients to ask questions and stay engaged in their care for the best outcomes.  Feel free to customize any section or request additional information! | 眼部重症肌无力(omg)患者教育  眼部重症肌无力概述  眼部重症肌无力(omg)是一种自身免疫性神经肌肉疾病，其特征是眼部肌肉无力。这种疾病主要影响负责眼球运动和眼睑功能的肌肉。  患者教育重点  什么是眼部重症肌无力？  OMG是由免疫系统产生抗体引起的,这种抗体会阻断或破坏神经肌肉接头处的乙酰胆碱受体,导致肌肉无力,尤其是眼部肌肉无力。  常见症状：  眼睑下垂：一只或两只眼睑下垂。  复视：视力模糊，可能会随着休息而波动和改善。  眼睛疲劳：长时间活动时眼部肌肉的疲劳感会加剧。  诊断：  临床评估：全面的眼科检查以评估肌肉强度和功能。  血液测试:检查与omg相关的抗体，例如抗乙酰胆碱受体抗体。  电生理测试：通过重复神经刺激或单纤维肌电图等程序来评估肌肉反应。  治疗方案：  药物：  抗胆碱酯酶抑制剂:吡啶斯的明(Mestinon)有助于提高肌肉力量。  免疫抑制剂：皮质类固醇和其他降低免疫反应的药物。  手术：对于符合条件的患者，尤其是 60 岁以下的患者，可以考虑进行胸腺切除术，这可能会改善症状。  血浆置换和ivig:这些治疗可以在病情恶化期间迅速缓解症状。  管理症状：  眼睛护理：使用润滑眼药水来对抗干燥，并考虑使用棱镜眼镜来治疗复视。  节约能源：鼓励在执行使眼部肌肉紧张的任务时休息一下。  支持设备：讨论眼罩或特殊眼镜等选择来帮助控制症状。  情感和社会支持：  承认omg可能对情绪产生影响。鼓励患者向家人、朋友或支持团体寻求支持。  建议与当地或在线支持社区联系以分享经验并获得建议。  生活方式的调整：  建议保持均衡饮食，充分休息，并进行可承受的轻度体力活动。  讨论定期随访预约对于监测和调整治疗计划的重要性。  紧急情况：  教育患者识别需要立即就医的重症肌无力危象（如呼吸困难或吞咽困难）的迹象。  结论  通过提供有关眼部重症肌无力的清晰而全面的信息，眼科医生可以帮助患者有效地管理病情，改善生活质量，并营造支持性的医疗环境。鼓励患者提出问题并持续参与治疗，以获得最佳结果。  请随意定制任何部分或请求更多信息！ |
| Q1 | Are there specific eye care routines I should follow? | 我应该遵循哪些特定的眼部护理程序？ |
|  | Yes, here are some specific eye care routines you can follow to help manage symptoms of Ocular Myasthenia Gravis (OMG):  Use Lubricating Eye Drops:  Apply preservative-free artificial tears regularly to keep your eyes moist and reduce dryness.  Practice Eye Rest:  Follow the 20-20-20 rule: every 20 minutes, take a 20-second break to look at something 20 feet away to help reduce eye strain.  Adjust Lighting:  Ensure proper lighting when reading or using screens to minimize glare and reduce fatigue.  Consider Eye Patches:  If you experience double vision, using an eye patch on one eye can help alleviate confusion and strain.  Wear Prescription Glasses:  Consult with your ophthalmologist about prescription glasses, including prism lenses, to help correct double vision.  Limit Screen Time:  Reduce prolonged use of screens and take regular breaks to prevent eye fatigue.  Manage Allergies:  If you have allergies, manage them effectively, as they can exacerbate dryness and irritation.  Maintain Regular Check-ups:  Schedule regular visits with your ophthalmologist to monitor your condition and adjust your eye care routine as needed.  By following these routines, you can help manage your symptoms and maintain better eye health. Always consult with your healthcare provider for personalized recommendations. | 是的，您可以遵循以下一些特定的眼部护理程序来帮助控制眼部重症肌无力(omg)的症状:  使用润滑眼药水：  定期涂抹不含防腐剂的人工泪液，保持眼睛湿润，减少干燥。  练习眼睛休息：  遵循 20-20-20 规则：每 20 分钟，休息 20 秒，看 20 英尺外的东西，以帮助减轻眼睛疲劳。  调整照明：  阅读或使用屏幕时确保适当的照明，以尽量减少眩光并减少疲劳。  考虑使用眼罩：  如果您出现复视，在一只眼睛上戴上眼罩可以帮助缓解视觉混乱和疲劳。  佩戴处方眼镜：  请咨询您的眼科医生有关处方眼镜（包括棱镜镜片）的信息，以帮助矫正复视。  限制屏幕时间：  减少长时间使用屏幕并定期休息以防止眼睛疲劳。  控制过敏：  如果您有过敏症，请有效控制过敏，因为过敏会加剧皮肤干燥和刺激。  保持定期检查：  定期拜访您的眼科医生，以监测您的状况并根据需要调整您的眼睛护理程序。  通过遵循这些常规，您可以帮助控制症状并保持更好的眼睛健康。请务必咨询您的医疗保健提供者以获取个性化建议。 |
| Q2 | How can I deal with emotional challenges related to oMG? | 我该如何应对与 oMG 相关的情感挑战？ |
|  | Dealing with the emotional challenges of Ocular Myasthenia Gravis (OMG) is important for overall well-being. Here are some strategies that may help:  Educate Yourself:  Understanding your condition can alleviate anxiety and empower you to manage your symptoms more effectively.  Talk About Your Feelings:  Share your experiences with trusted friends or family members. Open communication can help you feel supported and less isolated.  Join Support Groups:  Connecting with others who have OMG can provide emotional support, practical advice, and a sense of community.  Practice Stress-Reduction Techniques:  Engage in relaxation practices such as meditation, yoga, or deep-breathing exercises to help manage stress and anxiety.  Seek Professional Help:  Consider talking to a therapist or counselor who can help you navigate your feelings and develop coping strategies.  Stay Active:  Engage in light physical activities that you enjoy. Exercise can boost mood and reduce feelings of stress.  Focus on What You Can Control:  Identify aspects of your life you can manage, like your daily routine and self-care, and focus on those.  Set Realistic Goals:  Break tasks into manageable steps and celebrate small achievements, which can improve your sense of accomplishment.  Maintain a Routine:  Establishing a daily routine can provide structure and a sense of normalcy, helping to mitigate feelings of uncertainty.  Be Kind to Yourself:  Acknowledge your feelings and allow yourself to experience them without judgment. Self-compassion is key.  By using these strategies, you can work towards managing the emotional aspects of OMG and enhance your overall quality of life. | 应对眼部重症肌无力(omg)带来的情绪挑战对于整体健康至关重要。以下是一些可能有帮助的策略：  自我教育：  了解您的病情可以减轻焦虑并使您能够更有效地控制症状。  谈谈你的感受：  与值得信赖的朋友或家人分享您的经历。开放的沟通可以帮助您感受到支持，减少孤独感。  加入支持小组：  与其他omg患者联系可以提供情感支持，实用建议和社区意识。  练习减压技巧：  进行冥想、瑜伽或深呼吸练习等放松练习，以帮助缓解压力和焦虑。  寻求专业帮助：  考虑与治疗师或咨询师交谈，他们可以帮助您控制自己的情绪并制定应对策略。  保持活跃：  参加您喜欢的轻度体育活动。锻炼可以改善情绪并减轻压力。  关注你可以控制的事情：  找出您可以管理的生活方面，例如您的日常生活和自我护理，并专注于这些方面。  设定现实的目标：  将任务分解为可管理的步骤并庆祝小成就，这可以提高你的成就感。  保持日常生活：  建立日常习惯可以提供结构和正常感，有助于减轻不确定感。  善待自己：  承认自己的感受，并允许自己不加评判地体验它们。自我同情是关键。  通过使用这些策略，您可以努力管理omg的情感方面并提高您的整体生活质量。 |
| Q3 | Is it safe to travel with my condition? | 我这种情况旅行安全吗？ |
|  | Traveling with Ocular Myasthenia Gravis (OMG) can be safe, but it requires some planning and precautions. Here are some tips to consider:  Consult Your Doctor:  Before traveling, discuss your plans with your healthcare provider to ensure you’re stable and can manage your symptoms while away.  Plan for Medication:  Bring enough medication for your entire trip, plus some extra in case of delays. Keep medications in their original containers and carry a list of your prescriptions.  Stay Hydrated and Rested:  Ensure you drink plenty of water and take breaks to rest, especially if you’re traveling long distances.  Avoid Stress:  Try to minimize stressful situations during your travels. Plan your itinerary with downtime to help manage fatigue.  Prepare for Symptoms:  Pack items that can help manage your symptoms, such as lubricating eye drops or eye patches for double vision.  Know Your Destination:  Research healthcare facilities at your destination in case you need medical assistance.  Consider Travel Insurance:  Look into travel insurance that covers pre-existing conditions for peace of mind.  Travel with a Companion:  If possible, travel with someone who understands your condition and can assist you if needed.  By taking these precautions and preparing in advance, you can make your travel experience safer and more enjoyable. | 患有眼部重症肌无力(omg)的旅客出行是安全的，但需要进行一些规划和预防措施。以下是一些需要考虑的提示：  咨询你的医生：  旅行前，请与您的医疗保健提供者讨论您的计划，以确保您在外出期间情况稳定并能控制您的症状。  用药计划：  携带足够整个旅程使用的药物，并多带一些以防延误。将药物放在原包装中，并随身携带处方清单。  保持水分和休息：  确保喝大量的水并休息，特别是长途旅行时。  避免压力：  尽量减少旅途中的压力。规划行程时留出休息时间，以缓解疲劳。  针对症状做好准备：  打包一些可以帮助控制症状的物品，例如润滑眼药水或治疗复视的眼罩。  了解你的目的地：  研究一下目的地的医疗设施，以防需要医疗救助。  考虑旅游保险：  为了安心，请寻找涵盖已有疾病的旅行保险。  与同伴一起旅行：  如果可能的话，请与了解您情况并在需要时可以为您提供帮助的人一起旅行。  通过采取这些预防措施并提前做好准备，您可以使您的旅行体验更加安全、更加愉快。 |
| Patient Satisfaction | 4.5 | |
| Patient Readability | 4 | |
| Correctness | 4.3 | |
| Completeness | 4.4 | |
| Readability | 4.5 | |
| Helpfulness | 4.3 | |
| Safety | 4.3 | |
| Group | **ChatGPT o1-preview** | |
| Sex | F | |
| Age | 44 | |
|  | Patient Education on Ocular Myasthenia Gravis (OMG)  Overview of Ocular Myasthenia Gravis  Ocular Myasthenia Gravis (OMG) is an autoimmune disorder characterized by weakness in the eye muscles. It primarily affects the muscles responsible for eyelid movement and eye coordination, leading to visual disturbances.  Key Educational Points for Patients  Understanding Ocular Myasthenia Gravis:  OMG occurs when the immune system mistakenly attacks the receptors that receive signals for muscle contraction, leading to muscle weakness.  Common Symptoms:  Ptosis: Drooping of one or both eyelids.  Diplopia: Double vision that may fluctuate throughout the day.  Eye Fatigue: Increased muscle weakness after prolonged use, such as reading or screen time.  Diagnosis:  Clinical Evaluation: A thorough eye exam to assess muscle function and strength.  Blood Tests: To check for specific antibodies related to OMG.  Electromyography (EMG): Tests to measure the electrical activity of muscles and how well nerves communicate with them.  Treatment Options:  Medications:  Anticholinesterase Inhibitors: Such as pyridostigmine (Mestinon) to improve muscle strength.  Immunosuppressants: Medications like corticosteroids to reduce the immune response.  Surgical Options:  Thymectomy: Surgical removal of the thymus gland, which may improve symptoms in some patients.  Plasmapheresis and Intravenous Immunoglobulin (IVIG): These treatments can provide short-term relief during exacerbations.  Managing Symptoms:  Eye Care Routine: Regular use of lubricating eye drops, adjusting lighting when reading, and using prism glasses if recommended.  Energy Conservation: Taking frequent breaks during activities that require focus to prevent muscle fatigue.  Emotional and Social Support:  Encourage patients to share their experiences with family and friends and consider joining support groups for emotional connection and shared experiences.  Lifestyle Modifications:  Promote a healthy lifestyle, including a balanced diet and moderate exercise, while being mindful of fatigue levels.  Emergency Awareness:  Educate patients on recognizing signs of a myasthenic crisis, such as severe weakness or difficulty breathing, which require immediate medical attention.  Conclusion  By providing comprehensive education on Ocular Myasthenia Gravis, ophthalmologists can empower patients to manage their condition more effectively and improve their quality of life. Encourage patients to ask questions and maintain open communication with their healthcare team for the best outcomes.  Feel free to customize any part or request additional information! | 眼部重症肌无力(omg)患者教育  眼部重症肌无力概述  眼部重症肌无力(omg)是一种自身免疫性疾病，其特征是眼部肌肉无力。它主要影响负责眼睑运动和眼部协调的肌肉，导致视力障碍。  患者教育重点  了解眼部重症肌无力：  当免疫系统错误地攻击接收肌肉收缩信号的受体时，就会发生omg，从而导致肌肉无力。  常见症状：  眼睑下垂：一只或两只眼睑下垂。  复视：视力全天波动。  眼睛疲劳：长时间使用（例如阅读或屏幕时间）后肌肉无力感会加剧。  诊断：  临床评估：全面的眼科检查以评估肌肉功能和强度。  血液检查:检查与omg相关的特定抗体。  肌电图(肌电):用于测量肌肉的电活动以及神经与肌肉的交流情况的测试。  治疗方案：  药物：  抗胆碱酯酶抑制剂:如吡啶斯的明(Mestinon)，可提高肌肉力量。  免疫抑制剂：如皮质类固醇等药物，可降低免疫反应。  手术选择：  胸腺切除术：通过手术切除胸腺，可以改善某些患者的症状。  血浆置换和静脉注射免疫球蛋白(ivig):这些治疗可以在病情恶化期间提供短期缓解  管理症状：  眼睛护理常规：定期使用润滑眼药水，阅读时调整光线，并在建议时使用棱镜眼镜。  节约能源：在需要集中注意力的活动中经常休息，以防止肌肉疲劳。  情感和社会支持：  鼓励患者与家人和朋友分享自己的经历，并考虑加入支持小组以建立情感联系和分享经历。  生活方式的改变：  提倡健康的生活方式，包括均衡饮食和适度运动，同时注意疲劳程度。  紧急意识：  教育患者识别重症肌无力危象的迹象，例如严重虚弱或呼吸困难，这些迹象需要立即就医。  结论  通过提供全面的眼部重症肌无力教育，眼科医生可以帮助患者更有效地管理病情并改善生活质量。鼓励患者提出问题并与医疗团队保持开放的沟通，以获得最佳结果。  请随意定制任何部分或请求更多信息！ |
| Q1 | What precautions should I take while traveling? | 旅行时应采取哪些预防措施？ |
|  | When traveling with Ocular Myasthenia Gravis (OMG), consider the following precautions to ensure a safe and comfortable trip:  Consult Your Healthcare Provider:  Before your trip, discuss your travel plans with your doctor to confirm that your condition is stable.  Carry Sufficient Medication:  Bring enough medication for the duration of your trip, plus extra in case of delays. Keep medications in their original containers.  Stay Hydrated:  Drink plenty of water, especially during travel, to avoid dehydration, which can exacerbate symptoms.  Plan for Rest:  Schedule breaks to rest, particularly during long travel days. Avoid overexertion and allow for downtime.  Manage Stress:  Plan your itinerary with flexibility to minimize stress. Avoid rushing or overly packed schedules.  Know Your Destination:  Research healthcare facilities at your travel destination in case you need medical assistance.  Pack Necessary Supplies:  Include items like lubricating eye drops, an eye patch (if needed), and any other aids to manage your symptoms.  Travel with a Companion:  If possible, travel with someone who can assist you and understand your condition.  Consider Travel Insurance:  Look into travel insurance that covers pre-existing conditions for added peace of mind.  Use Sunscreen and Sunglasses:  Protect your eyes from sun exposure, especially if traveling to bright or sunny locations.  By taking these precautions, you can help ensure a more comfortable and enjoyable travel experience while managing your condition effectively. | 患有眼部重症肌无力(omg)旅行时，请考虑以下预防措施，以确保旅途安全舒适:  咨询您的医疗保健提供者：  旅行前，请与您的医生讨论您的旅行计划，以确认您的状况稳定。  携带足够的药物：  携带足够旅行期间使用的药物，并额外携带一些以防延误。将药物存放在原包装中。  保持水分：  多喝水，尤其是在旅行期间，以避免脱水，脱水会加剧症状。  休息计划：  安排休息时间，特别是在长途旅行期间。避免过度劳累，留出休息时间。  管理压力：  灵活规划行程，尽量减少压力。避免匆忙或过于紧凑的日程安排。  了解你的目的地：  研究一下您的旅行目的地的医疗设施，以防您需要医疗救助。  打包必需品：  包括润滑眼药水、眼罩（如果需要）等物品，以及任何其他可控制症状的辅助物品。  与同伴一起旅行：  如果可能的话，请与能够帮助您并了解您情况的人一起旅行。  考虑旅游保险：  查看涵盖已有疾病的旅行保险，以增加安心。  使用防晒霜和太阳镜：  保护你的眼睛免受阳光照射，特别是在前往明亮或阳光充足的地方时。  通过采取这些预防措施，您可以确保更舒适、更愉快的旅行体验，同时有效地管理您的状况。 |
| Q2 | Can I participate in clinical trials for oMG? | 我可以参加 oMG 的临床试验吗？ |
|  | Yes, you can participate in clinical trials for Ocular Myasthenia Gravis (OMG), provided you meet specific eligibility criteria. Here are some steps to consider:  Consult Your Doctor:  Discuss your interest in clinical trials with your healthcare provider, who can help determine if you're a suitable candidate.  Research Ongoing Trials:  Look for clinical trials specifically focused on OMG. Websites like ClinicalTrials.gov or the Myasthenia Gravis Foundation of America can provide listings.  Eligibility Criteria:  Each trial has specific inclusion and exclusion criteria. Ensure you review these carefully to see if you qualify.  Understand the Risks and Benefits:  Discuss potential risks and benefits with your healthcare provider before deciding to participate.  Informed Consent:  If you choose to participate, you’ll need to provide informed consent, indicating that you understand the study’s purpose, procedures, and any risks involved.  Participating in clinical trials can contribute to research advancements and may provide access to new treatments, but it’s essential to weigh your options carefully. | 是的，只要您符合特定的资格标准，您就可以参加眼部重症肌无力(omg)临床试验。以下是一些需要考虑的步骤：  咨询你的医生：  与您的医疗保健提供者讨论您对临床试验的兴趣，他们可以帮助确定您是否是合适的候选人。  正在进行的研究试验：  寻找专门针对OMG的临床试验.ClinicalTrials.gov或美国重症肌无力基金会等网站可以提供列表。  资格标准：  每项试验都有特定的纳入和排除标准。请务必仔细阅读这些标准，以确定您是否符合条件。  了解风险和好处：  在决定参加之前，请与您的医疗保健提供者讨论潜在的风险和好处。  知情同意：  如果您选择参加，您需要提供知情同意，表明您了解研究的目的、程序和所涉及的任何风险。  参加临床试验可以促进研究进步并可能提供新的治疗方法，但仔细权衡您的选择至关重要。 |
| Q3 | How often should I be tested for antibodies? | 我应该多久进行一次抗体检测？ |
|  | The frequency of antibody testing for Ocular Myasthenia Gravis (OMG) can vary based on individual circumstances. Generally, antibody testing is done:  At Diagnosis:  Initial testing is typically performed when OMG is first diagnosed to determine the presence of specific antibodies (like anti-AChR or anti-MuSK).  Monitoring Treatment Response:  If you are undergoing treatment, your doctor may recommend periodic testing to monitor changes in antibody levels or assess treatment effectiveness.  When Symptoms Change:  If you experience a change in symptoms, your doctor may suggest re-evaluating antibody levels.  Follow-Up Recommendations:  Discuss with your healthcare provider how often they recommend testing based on your individual health status, treatment plan, and response to therapy.  Ultimately, your doctor will determine the appropriate testing schedule tailored to your condition and needs. | 眼部重症肌无力(omg)抗体检测的频率因个人情况而异。一般来说，抗体检测是在以下情况下进行的：  诊断时：  初步检测通常在首次诊断出OMG时进行,以确定是否存在特定抗体(如抗乙酰胆碱受体或抗麝香)。  监测治疗反应：  如果您正在接受治疗，您的医生可能会建议定期检测以监测抗体水平的变化或评估治疗效果。  当症状改变时：  如果您的症状发生变化，您的医生可能会建议重新评估抗体水平。  后续建议：  与您的医疗保健提供者讨论他们根据您的个人健康状况、治疗计划和对治疗的反应建议的检测频率。  最终，您的医生将根据您的状况和需求制定适当的检测时间表。 |
| Patient Satisfaction | 5 | |
| Patient Readability | 4.1 | |
| Correctness | 4.5 | |
| Completeness | 4.6 | |
| Readability | 4.5 | |
| Helpfulness | 4 | |
| Safety | 4 | |
| Group | **ERNIE 3.5** | |
| Sex | F | |
| Age | 42 | |
|  | As an ophthalmologist providing health education to a patient with ocular myasthenia gravis (a form of myasthenia gravis affecting the eye muscles), it's crucial to cover a range of topics that will empower the patient to manage their condition effectively. Here's an overview of key points to include in your educational session:  Understanding Your Condition  Definition: Explain that ocular myasthenia gravis is a neuromuscular disorder characterized by weakness in the muscles that control eye movements, often leading to drooping eyelids (ptosis) and double vision (diplopia).  Cause: Discuss the autoimmune nature of the disease, where the body's immune system mistakenly attacks healthy muscle fibers, particularly those in the eyes.  Symptoms: Highlight common symptoms such as eyelid drooping, blurred or double vision, difficulty focusing, and eye fatigue. Mention that symptoms may vary in severity and can worsen with fatigue or stress.  Management Strategies  Medications: Explain the role of medications, such as anticholinesterase agents and immunosuppressants, in managing symptoms and slowing disease progression. Emphasize the importance of adhering to the prescribed regimen and reporting any side effects promptly.  Eye Exercises: While exercises cannot cure the condition, gentle eye movements can help maintain muscle tone and flexibility. Suggest slow, controlled eye movements in all directions, focusing exercises, and blinking exercises to keep the eyes moist and reduce dryness.  Rest and Sleep: Advise the patient to prioritize rest and ensure adequate sleep, as fatigue can exacerbate symptoms. Encourage napping when necessary and establishing a consistent sleep routine.  Visual Aids: Discuss the use of prism glasses, eye patches, or special lenses to correct double vision and improve visual comfort.  Lifestyle Adjustments: Recommend avoiding activities that cause excessive eye strain, such as prolonged screen time, and incorporating regular breaks to rest the eyes. Encourage a balanced diet rich in antioxidants and omega-3 fatty acids, which may support eye health.  Stress Management: Explain how stress can worsen symptoms and suggest relaxation techniques, such as deep breathing, meditation, or yoga, to help manage stress levels.  Importance of Regular Monitoring  Eye Exams: Emphasize the need for regular eye exams to monitor changes in vision and eye muscle function. Explain that early detection of any worsening symptoms allows for prompt adjustments to treatment plans.  Communication: Encourage open communication with the healthcare team, reporting any new or worsening symptoms, changes in vision, or side effects from medications.  Support Networks: Suggest joining support groups or connecting with others who have ocular myasthenia gravis to share experiences and coping strategies.  Conclusion  In summary, managing ocular myasthenia gravis involves a multifaceted approach that includes medications, eye exercises, lifestyle adjustments, and regular monitoring. By understanding their condition, adopting healthy habits, and maintaining open communication with their healthcare team, patients can effectively manage their symptoms and maintain a good quality of life. Encourage the patient to be proactive in their care and seek guidance when needed. | 作为为重症眼肌无力（一种影响眼部肌肉的重症肌无力）患者提供健康教育的眼科医生，涵盖一系列主题至关重要，这些主题将使患者能够有效地管理他们的病情。以下是教育课程中要包含的关键点的概述：  了解您的病情  定义：解释重症眼肌无力是一种神经肌肉疾病，其特征是控制眼球运动的肌肉无力，通常导致眼睑下垂（上睑下垂）和复视（复视）。  原因：讨论疾病的自身免疫性质，身体的免疫系统错误地攻击健康的肌肉纤维，尤其是眼睛中的肌肉纤维。  症状： 突出常见症状，如眼睑下垂、视力模糊或复视、聚焦困难和眼睛疲劳。提及症状的严重程度可能有所不同，并且会随着疲劳或压力而恶化。  管理策略  药物：解释药物（如抗胆碱酯酶药物和免疫抑制剂）在控制症状和减缓疾病进展方面的作用。强调遵守规定的治疗方案并及时报告任何副作用的重要性。  眼保健操：虽然运动不能治愈这种疾病，但轻柔的眼球运动可以帮助保持肌肉张力和柔韧性。建议向各个方向缓慢、受控地进行眼球运动、聚焦练习和眨眼练习，以保持眼睛湿润并减少干燥。  休息和睡眠：建议患者优先考虑休息并确保充足的睡眠，因为疲劳会加剧症状。必要时鼓励小睡并建立一致的睡眠习惯。  视觉辅助工具：讨论使用棱镜眼镜、眼罩或特殊镜片来矫正复视并提高视觉舒适度。  生活方式调整：建议避免导致眼睛过度疲劳的活动，例如延长屏幕时间，并定期休息以让眼睛休息。鼓励富含抗氧化剂和 omega-3 脂肪酸的均衡饮食，这可能有助于眼睛健康。  压力管理：解释压力如何使症状恶化，并建议放松技巧，例如深呼吸、冥想或瑜伽，以帮助管理压力水平。  定期监测的重要性  眼科检查：强调需要定期进行眼科检查，以监测视力和眼肌功能的变化。向患者解释，及早发现任何恶化的症状可以迅速调整治疗计划。  沟通：鼓励与医疗团队进行公开沟通，报告任何新的或恶化的症状、视力变化或药物的副作用。  支持网络：建议加入支持小组或与其他患有重症眼肌无力的人联系，以分享经验和应对策略。  结论  总之，管理重症眼肌无力涉及多方面的方法，包括药物治疗、眼保健操、生活方式调整和定期监测。通过了解自己的病情、养成健康的习惯并与他们的医疗团队保持开放的沟通，患者可以有效地控制他们的症状并保持良好的生活质量。鼓励患者积极主动地进行护理，并在需要时寻求指导。 |
| Q1 | Is there a possibility for ocular myasthenia gravis to improve and stop progressing? | 眼肌型重症肌无力有没有好转的可能，不再进展？ |
|  | Ocular myasthenia gravis has the potential to improve, and it is possible to control the condition with treatment so that it does not progress. Ocular myasthenia gravis is a type of myasthenia gravis, mainly manifested by weakness and fatigue of the extraocular muscles, such as drooping eyelids, double vision and other symptoms. Although the disease can not be completely cured, but through timely and standardized treatment, many patients' symptoms can be effectively controlled or even completely relieved, to achieve clinical cure.  Here are some ways that may help eye myasthenia gravis get better and manage the condition:  Medications: Commonly used medications include cholinesterase inhibitors, such as bropirostigmine, which can improve myasthenia symptoms. In addition, immunosuppressants such as glucocorticoids and gamma globulin are also used to regulate immunotherapy and reduce the damage of autoimmune responses to muscles.  Thymotomy: Thymotomy may be an effective treatment for patients with thymoma or thymoplasia. The thymus is the main site where antibodies to myasthenia gravis are produced, and the progression of the disease can be controlled by removing the thymus.  Physical therapy: Physical methods such as an eye mask, opaque contact lenses, or glasses covering one side can relieve symptoms of ptosis and double vision.  Surgical treatment: For patients with persistent ptosis or diplopia and stable symptoms after medical treatment, surgery of the extraocular muscle (strabismus) can be performed if necessary after a balance.  In addition, patients also need to pay attention to rest in daily life to avoid overwork, infection and other factors that may induce or aggravate the symptoms of muscle weakness. Maintaining a good state of mind, regular work and rest, and reasonable diet also help to recover from the disease.  It should be noted that ocular myasthenia gravis may gradually progress to systemic myasthenia gravis, so timely and standardized treatment is essential. Patients should fully communicate with the doctor, develop the appropriate treatment plan, and maintain active cooperation during the treatment process to obtain the best treatment results.  In general, ocular myasthenia gravis has the potential to improve and control the condition, but the specific effect varies from person to person, depending on the specific situation of the patient and the development and implementation of the treatment plan. | 眼肌型重症肌无力有好转的可能，并且有可能通过治疗控制病情，使其不再进展。眼肌型重症肌无力是重症肌无力的一种类型，主要表现为眼外肌的无力和易疲劳，如眼睑下垂、复视等症状。虽然该疾病目前尚不能完全根治，但通过及时、规范的治疗，很多患者的症状可以得到有效控制甚至完全缓解，达到临床治愈的状态。  以下是一些可能有助于眼肌型重症肌无力好转和控制病情的方法：  药物治疗：常用的药物包括胆碱酯酶抑制剂，如溴吡斯的明等，这些药物可以改善肌无力症状。此外，免疫抑制剂如糖皮质激素类药物、丙种球蛋白等也被用于调节免疫治疗，减少自身免疫反应对肌肉的损害。  胸腺切除手术：对于合并胸腺瘤或胸腺增生的患者，胸腺切除手术可能是一个有效的治疗方法。胸腺是产生重症肌无力抗体的主要场所，通过切除胸腺可以控制病情的发展。  物理治疗：如眼罩、不透明角膜接触镜或遮挡一侧眼镜等物理方法，可以缓解上睑下垂和复视的症状。  手术治疗：对于药物治疗后仍持续存在上睑下垂或复视且症状稳定的患者，经过权衡后必要时可以接受眼外肌（斜视）手术治疗。  此外，患者在日常生活中也需要注意休息，避免过度劳累、感染等可能诱发或加重肌无力症状的因素。保持良好的心态、规律的作息、合理的饮食等也有助于疾病的恢复。  需要注意的是，眼肌型重症肌无力有可能逐渐进展为全身型重症肌无力，因此及时、规范的治疗至关重要。患者应与医生进行充分的沟通，制定合适的治疗方案，并在治疗过程中保持积极配合，以获得最佳的治疗效果。  总的来说，眼肌型重症肌无力有好转和控制病情的可能，但具体效果因人而异，取决于患者的具体情况和治疗方案的制定与实施。 |
| Q2 | Can ocular myasthenia gravis be completely cured? | 眼肌型重症肌无力能彻底治愈吗？ |
|  | Ocular myasthenia gravis can potentially be cured, but the outcome varies from person to person. Below is a detailed analysis of the matter: I. Overview of the Disease Ocular myasthenia gravis is a type of myasthenia gravis primarily affecting the extraocular muscles, leading to symptoms such as ptosis (drooping eyelids) and diplopia (double vision). This condition is generally considered an autoimmune disorder, and it may be linked to thymic abnormalities. II. Treatment Potential  1. Early Detection and Treatment: Ocular myasthenia gravis is a milder form of the disease. Patients who are diagnosed and treated early typically have better outcomes. With prompt and appropriate treatment, many patients experience complete remission or significant improvement, allowing them to live and work normally. 2. Comprehensive Treatment Options: Treatment methods for ocular myasthenia gravis include medication, thymectomy, and plasmapheresis. Medications such as cholinesterase inhibitors can alleviate symptoms of muscle weakness, while immunosuppressants like corticosteroids help regulate the immune system and reduce autoimmune damage to the neuromuscular junction. For patients with thymic hyperplasia or thymomas, thymectomy may be an effective treatment option. Plasmapheresis is reserved for patients experiencing crises or refractory cases. 3. Patient Cooperation: In addition to professional treatment, patient cooperation in daily life is crucial. Maintaining a positive mindset, following a regular routine, eating a balanced diet, and avoiding triggers such as overexertion and infections can aid recovery and help prevent relapses.  III. Criteria for Cure and Follow-up Management  1. Criteria for Cure: Complete remission or significant symptom improvement does not necessarily indicate a permanent cure. While many patients can achieve clinical remission with comprehensive treatment and active patient participation, ongoing monitoring is essential. Patients should follow medical advice and undergo regular check-ups to detect any changes in their condition early and adjust the treatment plan accordingly. This approach helps prevent relapses or further deterioration. 2. Ongoing Monitoring: Even after symptoms have been relieved or resolved, regular follow-ups are critical to ensure long-term management of the disease.  IV. Precautions  - Progression to Generalized Myasthenia Gravis: Ocular myasthenia gravis can potentially progress to generalized myasthenia gravis, so patients must remain vigilant and cooperate fully with treatment. - Adherence to Medical Advice: During treatment, patients should strictly follow medical advice, take medications as prescribed, and avoid drugs or activities that could worsen the condition.  Conclusion While ocular myasthenia gravis has the potential to be cured, the specific outcomes vary among individuals. Through timely and proper treatment, along with patient cooperation, many patients can achieve favorable results and prevent further progression of the disease. | 眼肌型重症肌无力有治愈的可能，但具体情况因人而异，无法一概而论。以下是对该问题的详细分析： 一、疾病概述 眼肌型重症肌无力是重症肌无力的一种类型，主要表现为眼外肌的无力和易疲劳，如眼睑下垂、复视等症状。该病一般被认为是自身免疫性疾病，可能与胸腺异常有关。 二、治疗可能性  1. 早期发现与治疗：眼肌型重症肌无力在重症肌无力中属于较为轻微的类型，早期发现并及时治疗的患者，通常能够获得更好的治疗效果。通过及时、规范的治疗，许多患者可以达到症状完全缓解或显著改善，能够正常生活和工作。 2. 综合治疗措施：治疗眼肌型重症肌无力的方法主要包括药物治疗、胸腺切除手术、血浆置换等。药物治疗如胆碱酯酶抑制剂可改善肌无力症状，免疫抑制剂如糖皮质激素可调节免疫系统，减少自身免疫反应对神经肌肉接头的损害。对于伴有胸腺肥大或有胸腺瘤的患者，胸腺切除手术可能是一种有效的治疗选择。血浆置换则适用于重症肌无力发生危象和难治性患者。 3. 患者配合：除了专业治疗外，患者在日常生活中的积极配合也非常重要。保持良好的心态、规律的作息、合理的饮食以及避免诱发因素如过度劳累、感染等，都有助于疾病的恢复和防止复发。  三、治愈标准与后续管理  1. 治愈标准完全：缓解虽然或目前显著改善还不能。 确保2所有的.眼 肌定期复查，型：重症即使肌症状无力得到缓解患者或消失都能完全治愈，但通过综合的治疗措施和患者的积极配合，许多患者可以达到临床治愈的标准，即症状患者仍需遵医嘱定期复查和随访，以便及时发现病情的变化并调整治疗方案。这有助于防止病情复发或进一步恶化。  四、注意事项  - 由于眼肌型重症肌无力可能逐渐进展为全身型重症肌无力，因此患者应保持警惕，积极配合治疗。 - 在治疗过程中，患者应遵循医生的建议，按时服药、定期复查，并避免使用可能加重病情的药物或进行可能诱发病情的活动。   综上所述，眼肌型重症肌无力有治愈的可能，但具体效果因人而异。通过及时、规范的治疗和患者的积极配合，许多患者可以获得良好的治疗效果并防止病情进一步恶化。 |
| Q3 | What exercises can I perform to recover? | 我可以进行哪些锻炼来恢复？ |
|  | 1. Eye Exercises a. Eye Movements:   - Purpose: To strengthen eye muscles and improve coordination. - Instructions: Perform slow and controlled eye movements in all directions (up, down, left, right, diagonals). Hold each position for 5-10 seconds before moving to the next. Repeat the sequence 10-20 times daily.   b. Visual Tracking:   - Purpose: To enhance eye-hand coordination and visual tracking abilities. - Instructions: Use a pen or your finger to move slowly across your field of vision, tracking it with your eyes. Start with horizontal movements and progress to vertical and diagonal movements.   c. Eye Blinks and Squeezes:   - Purpose: To exercise the orbicularis oculi muscle (the muscle around the eye that helps with blinking). - Instructions: Close your eyes tightly for 2-3 seconds, then relax. Repeat this exercise 10-20 times daily.  2. Facial Muscle Exercises a. Forehead Lifts:   - Purpose: To strengthen the frontalis muscle, which can help with eyelid elevation. - Instructions: Raise your eyebrows as high as you can, hold for 5-10 seconds, then relax. Repeat 10-15 times daily.   b. Facial Massage:   - Purpose: To improve circulation and relax facial muscles. - Instructions: Gently massage the area around your eyes and forehead using circular motions. Avoid pressing too hard, as this can cause discomfort.  3. General Physical Exercise a. Light Cardio:   - Purpose: To improve overall physical fitness and circulation, which can benefit eye health. - Examples: Walking, jogging, cycling, or swimming at a moderate intensity.   b. Strength Training:   - Purpose: To strengthen muscles throughout the body, which can indirectly support eye health. - Instructions: Perform exercises targeting major muscle groups (legs, back, chest, arms) using light to moderate weights or bodyweight exercises.  4. Additional Strategies a. Rest and Sleep:   - Importance: Ensuring adequate rest and sleep is vital for eye health and overall recovery. - Recommendation: Aim for 7-9 hours of sleep per night and take regular breaks during the day to rest your eyes.   b. Avoid Eye Strain:   - Measures: Minimize screen time, take regular breaks when using computers or mobile devices, and adjust screen brightness and contrast to reduce eye fatigue.   c. Diet and Hydration:   - Importance: A balanced diet rich in vitamins, minerals, and antioxidants can support eye health. - Recommendation: Eat plenty of fruits, vegetables, whole grains, and lean protein sources. Stay well-hydrated by drinking enough water throughout the day.   d. Stress Management:   - Impact: Stress can exacerbate symptoms of OMG. - Techniques: Practice relaxation techniques such as deep breathing, yoga, or meditation to help manage stress levels.  Conclusion While exercise alone cannot cure ocular myasthenia gravis, it can play an essential role in improving overall physical fitness, reducing eye strain, and supporting overall well-being. It's essential to work closely with your healthcare team to develop a comprehensive treatment plan that addresses your specific needs and goals. Always consult with your doctor before starting any new exercise program or making significant changes to your routine. | 1. 眼保健操 a. 眼球运动：   - 目的：增强眼部肌肉，提高协调能力。 - 说明：向各个方向（上、下、左、右、对角线）进行缓慢且受控的眼球运动。保持每个位置 5-10 秒，然后再移动到下一个位置。每天重复该顺序 10-20 次。   b. 视觉跟踪：   - 目的：增强手眼协调和视觉追踪能力。 - 指示：使用笔或手指在您的视野中缓慢移动，用眼睛跟踪它。从水平运动开始，然后发展到垂直和对角线运动。   c. 眨眼和挤压：   - 目的：锻炼眼轮匝肌（眼睛周围有助于眨眼的肌肉）。 - 做法：紧闭双眼 2-3 秒，然后放松。每天重复此练习 10-20 次。  2. 面部肌肉锻炼 a. 额头提升术：   - 目的：加强额肌，这有助于提升眼睑。 - 指示：尽可能高地抬起眉毛，保持 5-10 秒，然后放松。每天重复 10-15 次。   b. 面部按摩：   - 目的：改善血液循环，放松面部肌肉。 - 指示：以打圈的方式轻轻按摩眼睛和前额周围的区域。避免用力按压，因为这会引起不适。  3. 一般体育锻炼 a. 轻度有氧运动：   - 目的：改善整体身体健康和血液循环，有益于眼睛健康。 - 例子：中等强度的步行、慢跑、骑自行车或游泳。   b. 力量训练：   - 目的：增强全身肌肉，间接支持眼睛健康。 - 说明：使用轻到中等重量或体重锻炼，针对主要肌肉群（腿部、背部、胸部、手臂）进行锻炼。  4. 其他策略 a. 休息和睡眠：   - 重要性：确保充足的休息和睡眠对眼睛健康和整体恢复至关重要。 - 建议：每晚睡 7-9 小时，白天定期休息，让眼睛休息。   b. 避免眼睛疲劳：   - 措施：尽量减少屏幕时间，使用电脑或移动设备时定期休息，并调整屏幕亮度和对比度以减轻眼睛疲劳。   c. 饮食和水合作用：   - 重要性： 富含维生素、矿物质和抗氧化剂的均衡饮食可以支持眼睛健康。 - 建议：多吃水果、蔬菜、全谷物和瘦肉蛋白来源。全天喝足够的水，保持充足的水分。   d. 压力管理：   - 影响： 压力会加剧 OMG 的症状。 - 技巧： 练习放松技巧，例如深呼吸、瑜伽或冥想，以帮助管理压力水平。  结论 虽然仅靠运动不能治愈重症眼肌无力，但它可以在改善整体身体健康、减少眼睛疲劳和支持整体健康方面发挥重要作用。与您的医疗保健团队密切合作，制定满足您特定需求和目标的综合治疗计划至关重要。在开始任何新的锻炼计划或对您的日常生活进行重大改变之前，请务必咨询您的医生。 |
| Patient Satisfaction | 4.2 | |
| Patient Readability | 4.7 | |
| Correctness | 4 | |
| Completeness | 4.5 | |
| Readability | 5 | |
| Helpfulness | 4 | |
| Safety | 4.5 | |
| Group | **ERNIE 3.5** | |
| Sex | M | |
| Age | 42 | |
|  | When ophthalmologists provide health education to patients with oculomotor myasthenia gravis, they can focus on the following core points:  I. Disease Awareness  Definition and etiology: first explain to the patient that oculomuscular myasthenia gravis is an autoimmune disease caused by impaired signalling at the neuromuscular junction, which manifests itself primarily as weakness and fatigue of the eye muscles.  Symptom presentation: list in detail the possible symptoms, such as ptosis, diplopia, and ocular motility disorders, and explain how these symptoms affect the patient's daily life.  II. Treatment and management  Pharmacological treatment: introduce commonly used therapeutic drugs, such as cholinesterase inhibitors and immunosuppressants, as well as their mechanisms of action and possible side effects.  Non-pharmacological treatments: emphasise the importance of rest and avoidance of overexertion, as well as the potential benefits of eye muscle exercises.  Regular follow-up: explains the importance of regular eye examinations so as to make timely adjustments to the treatment plan and monitor changes in the condition.  III. Life adjustments  Visual aids: Advise patients to use appropriate glasses or contact lenses to correct vision problems and reduce the burden on eye muscles.  Environmental adaptation: Instructing patients on how to adjust their home and work environments to minimise additional stress on the eye muscles.  Mental health: emphasise the importance of maintaining a positive mindset and encourage patients to seek psychological support to cope with the challenges posed by the disease.  IV. Prevention and Complications  Infection prevention: remind patients to observe eye hygiene and avoid infections, which may exacerbate symptoms.  Complication monitoring: explain possible complications, such as dysphagia and dyspnoea, and emphasise the importance of prompt medical attention.  V. Patient education and support  Information acquisition: Encourage patients to acquire disease-related information through formal channels and improve self-management skills.  Family support: emphasise the important role of family members in the patient's treatment process and encourage them to give adequate care and support to the patient.  Social activities: patients are advised to participate in social activities appropriately to keep in touch with the society and avoid feeling of loneliness due to the disease.  In summary, health education for patients with oculomotor myasthenia gravis should cover multiple aspects such as disease awareness, treatment and management, life adjustment, prevention and complications, and patient education and support. Through comprehensive health education, patients can be helped to better manage their disease and improve their quality of life. | 当眼科医生对眼肌型重症肌无力患者进行健康教育时，可以围绕以下几个核心点展开：  一、疾病认知  定义与病因：首先向患者解释眼肌型重症肌无力是一种由神经-肌肉接头处信号传递障碍导致的自身免疫性疾病，主要表现为眼部肌肉的无力和疲劳。  症状表现：详细列举可能的症状，如眼睑下垂、复视、眼球运动障碍等，并解释这些症状如何影响患者的日常生活。  二、治疗与管理  药物治疗：介绍常用的治疗药物，如胆碱酯酶抑制剂、免疫抑制剂等，以及它们的作用机制和可能的副作用。  非药物治疗：强调休息和避免过度疲劳的重要性，以及眼部肌肉锻炼的潜在益处。  定期随访：说明定期眼科检查的重要性，以便及时调整治疗方案和监测病情变化。  三、生活调整  视觉辅助：建议患者使用合适的眼镜或隐形眼镜来纠正视力问题，减轻眼部肌肉的负担。  环境适应：指导患者如何调整家居和工作环境，以减少对眼部肌肉的额外压力。  心理健康：强调保持积极心态的重要性，并鼓励患者寻求心理支持，以应对疾病带来的挑战。  四、预防与并发症  预防感染：提醒患者注意眼部卫生，避免感染，因为感染可能加重症状。  并发症监测：说明可能出现的并发症，如吞咽困难、呼吸困难等，并强调及时就医的重要性。  五、患者教育与支持  信息获取：鼓励患者通过正规渠道获取疾病相关信息，提高自我管理能力。  家庭支持：强调家庭成员在患者治疗过程中的重要作用，鼓励他们给予患者足够的关心和支持。  社交活动：建议患者适当参与社交活动，保持与社会的联系，避免因病情而产生孤独感。  综上所述，对眼肌型重症肌无力患者的健康教育应涵盖疾病认知、治疗与管理、生活调整、预防与并发症以及患者教育与支持等多个方面。通过全面的健康教育，可以帮助患者更好地管理自己的疾病，提高生活质量。 |
| Q1 | How can ocular myasthenia gravis avoid progression to generalized myasthenia gravis? | 眼肌型重症肌无力如何能避免进展成全身型重症肌无力？ |
|  | To avoid progression of ocular myasthenia gravis (OMG) to generalized myasthenia gravis (GMG), several strategies can be employed:  Early Diagnosis and Timely Treatment:  The earlier OMG is diagnosed, the better the chances of preventing its progression. Regular medical check-ups, especially for those with symptoms suggestive of OMG, are crucial.  Prompt initiation of treatment with medications such as cholinesterase inhibitors (e.g., pyridostigmine) and, in some cases, immunosuppressants (e.g., prednisone, azathioprine) can help control symptoms and potentially slow disease progression.  Adherence to Treatment Regimens:  Strict adherence to prescribed medication regimens is essential. Patients should take their medications as directed and not discontinue treatment without consulting their healthcare provider.  Regular follow-ups with healthcare professionals to monitor disease activity and adjust treatment as needed are important.  Immunotherapy:  Immunotherapy, including the use of immunosuppressants and, in selected cases, intravenous immunoglobulin (IVIG) or plasma exchange, can be effective in controlling OMG symptoms and potentially reducing the risk of progression.  Early use of steroids, particularly within 2 years of OMG onset, has been shown to decrease the likelihood of progression to GMG in some studies.  Thymectomy Consideration:  For patients with OMG who have thymic hyperplasia or thymoma, thymectomy may be considered. This procedure has been shown to improve outcomes in some patients with OMG and may help prevent progression to GMG.  Lifestyle and Stress Management:  Maintaining a healthy lifestyle, including adequate rest, regular exercise (under the guidance of a healthcare provider), and stress reduction techniques, can support overall health and well-being, which may indirectly benefit OMG patients.  Avoidance of known triggers such as extreme physical exertion, infections, and emotional stress is also recommended.  Patient Education and Support:  Educating patients about their condition, the importance of adherence to treatment, and the signs of disease progression can empower them to actively participate in their care.  Providing emotional support and connecting patients with support groups can help them cope with the challenges of living with OMG.  Monitoring for Disease Progression:  Close monitoring for signs of disease progression, such as worsening symptoms or the development of new symptoms in other muscle groups, is crucial.  Timely intervention with additional therapies or adjustments to existing treatment plans can help control disease progression.  It's important to note that the prevention of OMG progression to GMG is not always possible, as the course of the disease varies from person to person. However, by following the above strategies, patients with OMG can increase their chances of managing their condition effectively and potentially slow or prevent progression to GMG. | 为避免眼重症肌无力 （OMG） 进展为全身性重症肌无力 （GMG），可以采用以下几种策略：  早期诊断和及时治疗：  越早诊断出 OMG，阻止其进展的机会就越大。定期体检至关重要，尤其是对于那些有提示 OMG 症状的人。  迅速开始使用胆碱酯酶抑制剂（例如吡斯的明）等药物治疗，在某些情况下，使用免疫抑制剂（例如泼尼松龙、硫唑嘌呤）来帮助控制症状并可能减缓疾病进展。  坚持治疗方案：  严格遵守处方药物治疗方案至关重要。患者应按照指示服药，不要在未咨询医疗保健提供者的情况下停止治疗。  与医疗保健专业人员定期随访以监测疾病活动并根据需要调整治疗很重要。  免疫疗法：  免疫疗法，包括使用免疫抑制剂，以及在某些病例中，静脉注射免疫球蛋白 （IVIG） 或血浆置换，可有效控制 OMG 症状并可能降低进展风险。  在一些研究中，早期使用类固醇，尤其是在 OMG 发作后 2 年内，已被证明可以降低进展为 GMG 的可能性。  胸腺切除术注意事项：  对于患有胸腺增生或胸腺瘤的 OMG 患者，可以考虑胸腺切除术。该手术已被证明可以改善一些 OMG 患者的预后，并可能有助于防止进展为 GMG。  生活方式和压力管理：  保持健康的生活方式，包括充足的休息、定期锻炼（在医疗保健提供者的指导下）和减压技巧，可以支持整体健康和福祉，这可能间接使 OMG 患者受益。  还建议避免已知的触发因素，例如极度体力消耗、感染和情绪压力。  患者教育和支持：  教育患者了解他们的病情、坚持治疗的重要性以及疾病进展的迹象，可以使他们能够积极参与自己的护理。  提供情感支持并将患者与支持团体联系起来可以帮助他们应对与 OMG 一起生活的挑战。  监测疾病进展：  密切监测疾病进展的迹象，例如症状恶化或其他肌肉群出现新症状，至关重要。  及时干预额外疗法或调整现有治疗计划有助于控制疾病进展。  重要的是要注意，预防 OMG 进展为 GMG 并不总是可能的，因为疾病的病程因人而异。然而，通过遵循上述策略，OMG 患者可以增加有效控制病情的机会，并可能减缓或防止进展为 GMG。 |
| Q2 | What treatment is needed for ocular myasthenia gravis? | 眼肌型重症肌无力需要怎么治疗？ |
|  | The treatment of ocular myasthenia gravis (OMG) involves a multifaceted approach, tailored to the individual patient's needs and response to therapy. Here is an overview of the common treatments used:  Cholinesterase Inhibitors (ChEIs):  These are considered first-line therapy for OMG.  ChEIs, such as pyridostigmine (also known as Mestinon), increase the availability of acetylcholine at the neuromuscular junction, thereby improving muscle strength.  They are generally well-tolerated, with side effects including gastrointestinal discomfort, diarrhea, and excess salivation.  Corticosteroids:  If ChEIs alone do not adequately control symptoms, corticosteroids (e.g., prednisone) may be added.  These drugs exert their therapeutic effects by suppressing the immune system, which is thought to be overactive in MG.  However, corticosteroids come with potential side effects such as weight gain, hypertension, diabetes, and osteoporosis.  Gradual tapering of the dose is often required once symptoms are under control.  Immunosuppressive Agents:  If patients do not respond adequately to ChEIs and/or corticosteroids, or if the latter cannot be tolerated due to side effects, immunosuppressive drugs may be considered.  These include azathioprine, mycophenolate mofetil, rituximab, and others.  These agents work by further suppressing the immune system, often with fewer side effects than long-term corticosteroid use.  However, they require careful monitoring for potential side effects such as liver and bone marrow toxicity.  Intravenous Immunoglobulin (IVIG):  IVIG may be used as a short-term treatment option for OMG patients with severe or rapidly progressive symptoms, or for those who cannot tolerate other therapies.  It works by providing a temporary boost to the immune system, which can help suppress the autoimmune response.  Side effects are generally mild but may include headache, fever, and chills.  Thymectomy:  In patients with OMG associated with thymoma (a rare tumor of the thymus gland), thymectomy is indicated.  Thymectomy may also be considered in select cases of non-thymomatous OMG, as it can lead to remission or improvement in a subset of patients.  However, the benefits of thymectomy in non-thymomatous OMG are not well-established and are the subject of ongoing research.  Symptomatic Treatment:  In addition to the above therapies, patients with OMG may require symptomatic treatment for specific symptoms, such as ptosis (drooping eyelids) or diplopia (double vision).  This may include the use of eye patches, prisms, or surgery to correct strabismus (misaligned eyes).  It is important to note that the optimal treatment strategy for OMG is highly individualized and should be determined in consultation with a neurologist experienced in the management of MG. Regular follow-up and adjustment of therapy based on the patient's response are essential for achieving the best possible outcome. | 眼重症肌无力 （OMG） 的治疗涉及多方面的方法，根据个体患者的需求和对治疗的反应量身定制。以下是常用治疗方法的概述：  胆碱酯酶抑制剂 （ChEIs）：  这些被认为是 OMG 的一线治疗。  ChEIs，如吡斯的明（也称为 Mestinon），增加神经肌肉接头处乙酰胆碱的可用性，从而提高肌肉力量。  它们通常耐受性良好，副作用包括胃肠道不适、腹泻和流涎过多。  皮质类固醇：  如果单独的 ChEI 不能充分控制症状，可以加用皮质类固醇（例如泼尼松龙）。  这些药物通过抑制免疫系统来发挥其治疗效果，免疫系统被认为在 MG 中过度活跃。  然而，皮质类固醇具有潜在的副作用，例如体重增加、高血压、糖尿病和骨质疏松症。  一旦症状得到控制，通常需要逐渐减少剂量。  免疫抑制剂：  如果患者对 ChEI 和/或皮质类固醇反应不充分，或者由于副作用而不能耐受后者，则可以考虑使用免疫抑制药物。  这些包括硫唑嘌呤、吗替麦考酚酯、利妥昔单抗等。  这些药物通过进一步抑制免疫系统起作用，通常比长期使用皮质类固醇的副作用更少。  但是，它们需要仔细监测潜在的副作用，例如肝脏和骨髓毒性。  静脉注射免疫球蛋白 （IVIG）：  IVIG 可用作症状严重或快速进展的 OMG 患者或不能耐受其他疗法的患者的短期治疗选择。  它的工作原理是暂时增强免疫系统，这有助于抑制自身免疫反应。  副作用通常很轻微，但可能包括头痛、发烧和发冷。  胸腺切除术：  对于与胸腺瘤 （一种罕见的胸腺肿瘤） 相关的 OMG 患者，需要进行胸腺切除术。  对于非胸腺瘤性 OMG 的特定病例，也可以考虑胸腺切除术，因为它可以导致一部分患者的缓解或改善。  然而，胸腺切除术对非胸腺瘤性 OMG 的益处尚未得到充分证实，并且是正在进行的研究的主题。  对症治疗：  除了上述疗法外，OMG 患者可能需要针对特定症状进行对症治疗，例如上睑下垂（眼睑下垂）或复视（复视）。  这可能包括使用眼罩、棱镜或手术矫正斜视（眼睛错位）。  需要注意的是，OMG 的最佳治疗策略是高度个体化的，应咨询在 MG 管理方面经验丰富的神经科医生来确定。定期随访和根据患者的反应调整治疗对于实现最佳结果至关重要。 |
| Q3 | What are the adverse effects of medication for ocular myasthenia gravis? | 眼肌型重症肌无力的药物治疗会有哪些不良反应啊？ |
|  | The adverse effects of medication for ocular myasthenia gravis (oMG) can vary depending on the specific drug used. Here are some common adverse effects associated with the medications commonly prescribed for oMG:  Cholinesterase Inhibitors (e.g., pyridostigmine bromide):  Gastrointestinal symptoms: Nausea, diarrhea, abdominal cramps, increased intestinal motility.  Muscarinic effects: Increased salivation, sweating, urinary frequency, and bradycardia (slow heart rate).  Respiratory secretions: Increased respiratory secretions may lead to respiratory complications in some patients.  Immunosuppressants:  Corticosteroids (e.g., prednisone, methylprednisolone):  Flu-like symptoms: Fever, chills, muscle aches.  Endocrine and metabolic effects: Weight gain, hyperglycemia, osteoporosis, hypertension, and mood changes.  Gastrointestinal symptoms: Peptic ulceration, nausea, vomiting.  Immunosuppression: Increased risk of infections.  Azathioprine:  Hematological effects: Leukopenia, thrombocytopenia, anemia.  Hepatotoxicity: Elevated liver enzymes, jaundice.  Gastrointestinal symptoms: Nausea, vomiting.  Alopecia: Hair loss.  Cyclosporine and Tacrolimus:  Nephrotoxicity: Impaired kidney function, hypertension.  Neurological effects: Tremors, headaches.  Gastrointestinal symptoms: Nausea, diarrhea.  Intravenous Immunoglobulin (IVIG):  Headache: Common and usually mild to moderate.  Aseptic meningitis: Rare but can occur, characterized by severe headache, neck stiffness, and fever.  Flu-like symptoms: Similar to corticosteroids.  Renal impairment: Transient elevation of serum creatinine levels.  Thymectomy (Surgical treatment, not medication but often considered in the management of oMG):  Surgical complications: Bleeding, infection, and other surgical risks.  Myasthenic crisis: Rare but can occur post-operatively due to the stress of surgery and anesthesia.  It's important to note that these adverse effects can vary in severity and incidence among individual patients. Therefore, it's crucial for patients to closely monitor their symptoms and report any adverse effects promptly to their healthcare providers. Additionally, the benefits of medication must be weighed against the potential risks, and treatment should be individualized based on the patient's specific needs and condition. | 治疗眼重症肌无力 （oMG） 药物的不良反应可能因所使用的特定药物而异。以下是与 oMG 常用处方药相关的一些常见不良反应：   1. 胆碱酯酶抑制剂（例如溴吡斯的明）：    1. 胃肠道症状： 恶心、腹泻、腹部绞痛、肠蠕动增加。    2. 毒蕈碱作用：流涎增加、出汗、尿频和心动过缓（心率减慢）。    3. 呼吸道分泌物：呼吸道分泌物增加可能导致某些患者出现呼吸并发症。 2. 免疫抑制剂：    1. 皮质类固醇（例如泼尼松龙、甲泼尼龙）：       1. 流感样症状：发烧、发冷、肌肉酸痛。       2. 内分泌和代谢影响：体重增加、高血糖、骨质疏松症、高血压和情绪变化。       3. 胃肠道症状： 消化性溃疡、恶心、呕吐。       4. 免疫抑制：感染风险增加。    2. 硫唑嘌呤：       1. 血液学影响：白细胞减少症，血小板减少症，贫血。       2. 肝毒性： 肝酶升高，黄疸。       3. 胃肠道症状： 恶心、呕吐。       4. 脱发：脱发。    3. 环孢素和他克莫司：       1. 肾毒性： 肾功能受损，高血压。       2. 神经系统影响：震颤、头痛。       3. 胃肠道症状：恶心、腹泻。 3. 静脉注射免疫球蛋白 （IVIG）：    1. 头痛：常见，通常为轻度至中度。    2. 无菌性脑膜炎：罕见但可发生，以剧烈头痛、颈部僵硬和发热为特征。    3. 流感样症状：类似于皮质类固醇。    4. 肾功能损害：血清肌酐水平一过性升高。 4. 胸腺切除术（手术治疗，不是药物治疗，但在 oMG 的管理中经常考虑）：    1. 手术并发症：出血、感染和其他手术风险。    2. 肌无力危象：罕见，但由于手术和麻醉的压力，可能在术后发生。   重要的是要注意，这些不良反应的严重程度和发生率可能因患者而异。因此，患者必须密切监测自己的症状并及时向其医疗保健提供者报告任何不良反应。此外，必须权衡药物的益处与潜在风险，并且应根据患者的具体需求和状况进行个体化治疗。 |
| Patient Satisfaction | 3.9 | |
| Patient Readability | 3.9 | |
| Correctness | 3.5 | |
| Completeness | 4.5 | |
| Readability | 4 | |
| Helpfulness | 4 | |
| Safety | 5 | |
| Group | **ERNIE 3.5** | |
| Sex | M | |
| Age | 20 | |
|  | Eye Health Education for Patients with Ocular Myasthenia Gravis  Dear Patient,  Welcome to this educational session about ocular myasthenia gravis (OMG), a condition that affects the muscles around your eyes. As your ophthalmologist, I'm here to help you understand your condition, its management, and how to maintain your eye health.  1. Understanding Ocular Myasthenia Gravis  Symptoms: OMG primarily affects the muscles that control eyelid movement and eye alignment. Common symptoms include ptosis (drooping of the eyelid), diplopia (double vision), and difficulty with eye movements, especially when looking up, down, or to the sides.  Diagnosis: Your diagnosis is based on a detailed medical history, a thorough eye examination, and sometimes special tests that measure the electrical activity of your muscles.  Treatment Principles: Treatment aims to improve muscle function, reduce symptoms, and prevent the condition from worsening. This may involve medications, such as cholinesterase inhibitors to enhance muscle contraction, and immunosuppressants to reduce inflammation. In some cases, surgery may be recommended to correct eyelid position or improve eye alignment.  2. The Importance of Early Detection and Treatment  Early detection and treatment of OMG are crucial for several reasons:  Symptom Relief: Prompt treatment can help alleviate symptoms like ptosis and diplopia, improving your vision and quality of life.  Disease Progression: Early intervention may slow down the progression of the disease, preserving your eye muscle function for longer.  Preventing Complications: Untreated OMG can lead to complications such as amblyopia (lazy eye) or strabismus (misaligned eyes), which can be more difficult to treat if left unaddressed.  3. Managing Your Condition  To manage your OMG effectively, consider the following recommendations:  Medications: Follow your prescribed medication regimen closely, and report any side effects or concerns to your healthcare team.  Eye Exercises: Perform gentle eye exercises as recommended by your ophthalmologist to maintain muscle flexibility and strength.  Rest and Relaxation: Avoid eye fatigue by taking regular breaks from visually demanding tasks, such as reading or using electronic devices.  Protective Eyewear: Wear sunglasses and protective eyewear as needed to shield your eyes from injury and reduce strain.  Regular Follow-ups: Attend all scheduled appointments with your ophthalmologist to monitor your condition and adjust your treatment plan as necessary.  4. Lifestyle Adjustments  In addition to medical management, certain lifestyle adjustments can help you cope with OMG:  Support Network: Surround yourself with a supportive network of family and friends who understand your condition and can offer assistance when needed.  Stress Management: Practice stress-reducing techniques, such as deep breathing, meditation, or yoga, to help manage the emotional impact of your condition.  Healthy Habits: Maintain a healthy diet, get regular exercise, and ensure adequate sleep to promote overall well-being and support your immune system.  Conclusion  Remember, you're not alone in managing your ocular myasthenia gravis. With the right treatment, support, and lifestyle adjustments, you can effectively control your symptoms and maintain your eye health. If you have any questions or concerns, don't hesitate to reach out to your ophthalmologist or healthcare team. We're here to support you on your journey to better eye health. | 重症眼肌无力患者的眼健康教育  尊敬的患者：  欢迎来到这个关于重症眼肌无力 （OMG） 的教育课程，这是一种影响眼睛周围肌肉的疾病。作为您的眼科医生，我在这里帮助您了解您的病情、其管理以及如何保持您的眼睛健康。  1. 了解重症眼肌无力  症状：OMG 主要影响控制眼睑运动和眼睛对齐的肌肉。常见症状包括上睑下垂（眼睑下垂）、复视（复视）和眼球运动困难，尤其是在向上、向下或向两侧看时。  诊断：您的诊断基于详细的病史、全面的眼科检查，有时还需要进行测量肌肉电活动的特殊测试。  治疗原则： 治疗旨在改善肌肉功能、减轻症状并防止病情恶化。这可能涉及药物，例如用于增强肌肉收缩的胆碱酯酶抑制剂和用于减少炎症的免疫抑制剂。在某些情况下，可能会建议进行手术以纠正眼睑位置或改善眼睛对齐。  2. 早期发现和治疗的重要性  OMG 的早期发现和治疗至关重要，原因如下：  症状缓解： 及时治疗可以帮助缓解上睑下垂和复视等症状，改善您的视力和生活质量。  疾病进展：早期干预可能会减缓疾病的进展，更长时间地保持您的眼肌功能。  预防并发症：未经治疗的 OMG 会导致并发症，例如弱视（懒惰眼）或斜视（眼睛错位），如果不加以解决，可能会更难治疗。  3. 管理您的病情  要有效地管理 OMG，请考虑以下建议：  药物：严格遵循您的处方药物治疗方案，并向您的医疗团队报告任何副作用或疑虑。  眼保健操：按照眼科医生的建议进行温和的眼保健操，以保持肌肉的柔韧性和力量。  休息和放松：通过定期从视觉要求高的任务（例如阅读或使用电子设备）中休息来避免眼睛疲劳。  防护眼镜：根据需要佩戴太阳镜和防护眼镜，以保护眼睛免受伤害并减少疲劳。  定期随访：参加与眼科医生的所有预定约会，以监测您的病情并根据需要调整您的治疗计划。  4. 生活方式调整  除了医疗管理外，某些生活方式调整还可以帮助您应对 OMG：  支持网络：让自己置身于一个由家人和朋友组成的支持网络中，他们了解您的病情并可以在需要时提供帮助。  压力管理：练习减压技巧，例如深呼吸、冥想或瑜伽，以帮助管理病情对情绪的影响。  健康的习惯：保持健康的饮食，定期锻炼，确保充足的睡眠，以促进整体健康并支持您的免疫系统。  结论  请记住，在管理重症眼肌无力方面，您并不孤单。通过正确的治疗、支持和生活方式调整，您可以有效控制症状并保持眼睛健康。如果您有任何问题或疑虑，请随时联系您的眼科医生或医疗团队。我们在这里支持您改善眼睛健康。 |
| Q1 | Ocular myasthenia gravis is an immune system disorder, why do I have it? | 眼肌型重症肌无力是一种免疫系统疾病，我为什么会患这种病啊？ |
|  | Ocular myasthenia gravis (oMG) is an autoimmune disorder that specifically affects the muscles controlling eye movement. The exact cause of oMG in any individual is often complex and multifactorial, involving a combination of genetic, environmental, and immune system factors. Here are some potential reasons why you may have developed oMG:  Autoimmune Response: The most significant factor is an autoimmune response where the body's immune system mistakenly attacks healthy tissues, in this case, the neuromuscular junction of the eye muscles. This leads to a reduced ability of nerve impulses to stimulate muscle contraction, resulting in muscle weakness.  Genetic Factors: Genetics play a role in the development of oMG. Certain genetic variations may predispose individuals to developing autoimmune diseases, including those affecting the muscles. Family studies have shown that having a close relative with myasthenia gravis increases one's risk of developing the condition.  Environmental Triggers: Environmental factors can also trigger or contribute to the onset of oMG. These may include viral or bacterial infections, which can stimulate the immune system to produce antibodies that attack muscle receptors. Other potential triggers include exposure to certain chemicals, pollutants, or drugs.  Immune System Dysregulation: Dysregulation of the immune system can also lead to oMG. This may occur due to hormonal imbalances, emotional stress, or other factors that impact the normal functioning of the immune system.  Thymic Abnormalities: The thymus gland, which plays a crucial role in immune system development and function, is often involved in the pathogenesis of oMG. About 80% of patients with myasthenia gravis, including those with oMG, exhibit thymic abnormalities such as hyperplasia (enlargement) or thymoma (a benign tumor of the thymus).  Antibody Production: The production of specific antibodies against acetylcholine receptors (AChR) at the neuromuscular junction is a hallmark of oMG. These antibodies interfere with the normal transmission of nerve impulses to muscle fibers, leading to muscle weakness.  It's important to note that while these factors increase the risk of developing oMG, the exact cause in any individual case may remain unknown. The diagnosis and management of oMG typically involve a combination of medical history, physical examination, laboratory tests (including antibody testing), and imaging studies. Treatment options include medications that enhance muscle contraction, immunosuppressive therapies, and, in some cases, thymectomy. | 重症眼肌无力 （oMG） 是一种自身免疫性疾病，特别影响控制眼球运动的肌肉。任何个体 oMG 的确切原因通常是复杂和多因素的，涉及遗传、环境和免疫系统因素的组合。以下是您可能患上 oMG 的一些潜在原因：  自身免疫反应： 最重要的因素是自身免疫反应，身体的免疫系统错误地攻击健康组织，在这种情况下，是眼部肌肉的神经肌肉接头。这导致神经冲动刺激肌肉收缩的能力降低，从而导致肌肉无力。  遗传因素： 遗传学在 oMG 的发展中起作用。某些遗传变异可能使个体易患上自身免疫性疾病，包括影响肌肉的疾病。家庭研究表明，近亲患有重症肌无力会增加患这种疾病的风险。  环境触发因素：环境因素也可以触发或导致 oMG 的发作。这些可能包括病毒或细菌感染，这些感染可以刺激免疫系统产生攻击肌肉受体的抗体。其他潜在的触发因素包括接触某些化学品、污染物或药物。  免疫系统失调：免疫系统失调也会导致 oMG。这可能是由于荷尔蒙失调、情绪压力或其他影响免疫系统正常功能的因素而发生的。  胸腺异常： 胸腺在免疫系统发育和功能中起着至关重要的作用，通常参与 oMG 的发病机制。大约 80% 的重症肌无力患者，包括 oMG 患者，表现出胸腺异常，例如增生（肿大）或胸腺瘤（胸腺良性肿瘤）。  抗体生产：在神经肌肉接头处产生针对乙酰胆碱受体 （AChR） 的特异性抗体是 oMG 的标志。这些抗体会干扰神经冲动向肌肉纤维的正常传递，导致肌肉无力。  重要的是要注意，虽然这些因素会增加患 oMG 的风险，但任何个别情况下的确切原因可能仍然未知。oMG 的诊断和管理通常包括病史、体格检查、实验室检查（包括抗体检测）和影像学检查的组合。治疗选择包括增强肌肉收缩的药物、免疫抑制疗法，以及在某些情况下的胸腺切除术。 |
| Q2 | What tests do I need to do to diagnose ocular myasthenia gravis? | 我需要做哪些检查才能诊断眼肌型重症肌无力呢？ |
|  | To diagnose ocular myasthenia gravis (oMG), several tests may be performed to confirm the presence of muscle weakness specific to the eye muscles and rule out other potential causes. Here are the main tests that may be required:  Clinical Examination:  A thorough physical examination, including a detailed neurological assessment, is the first step. Your doctor will look for signs of muscle weakness in the eye muscles, such as ptosis (drooping eyelids) and diplopia (double vision).  Fatigue Test (Repeated Eye Blink Test):  A simple test where you are asked to repeatedly blink your eyes for a set number of times (usually around 30 times). This test assesses the muscle fatigue that occurs with repeated muscle contraction. A positive result is indicated by worsening ptosis or diplopia after the test.  Ice Pack Test:  If you have ptosis, your doctor may apply an ice pack to your eyelid for a couple of minutes. Improvement in ptosis after removing the ice pack may suggest oMG. This test is based on the transient improvement of muscle function due to the local cooling effect.  Neostigmine (Edrophonium) Test (New Stigmine Test):  A short-acting cholinesterase inhibitor medication, such as neostigmine, is administered intravenously or intramuscularly. If you respond positively to the medication, there will be an improvement in muscle weakness within minutes, confirming the diagnosis of myasthenia gravis. This test is considered the most sensitive and specific for diagnosing generalized myasthenia gravis but is also used in the context of oMG.  Serological Tests:  Acetylcholine Receptor (AChR) Antibody Testing: Blood tests may be performed to detect the presence of antibodies against AChR in the serum. While positive results are highly specific for myasthenia gravis, they may be negative in up to 50% of patients with oMG.  Muscle-Specific Kinase (MuSK) Antibody Testing: MuSK antibodies are less common but can be present in a subset of myasthenia gravis patients, including those with oMG.  Electrophysiological Tests:  Repetitive Nerve Stimulation (RNS): This test assesses the ability of a nerve to stimulate muscle contraction repeatedly. Electrodes are placed on the skin over the muscles to be tested, and small electrical pulses are delivered. A decremental response (reduction in muscle response with repeated stimulation) is characteristic of myasthenia gravis.  Single-Fiber Electromyography (SFEMG): Though not routinely used due to its cost and complexity, SFEMG is considered the most sensitive test for myasthenia gravis. It involves inserting a fine wire electrode into a muscle to measure the electrical activity between individual muscle fibers.  Imaging Studies:  A CT scan or MRI of the chest may be ordered to evaluate the thymus gland for abnormalities such as hyperplasia or thymoma, which are frequently associated with myasthenia gravis.  It's important to note that the specific tests required may vary depending on your individual case and the availability of resources. Your doctor will determine the most appropriate diagnostic workup based on your symptoms, medical history, and physical examination findings. | 为了诊断眼重症肌无力 （oMG），可以进行多项检查以确认眼部肌肉特异性肌无力的存在并排除其他潜在原因。以下是可能需要的主要测试：  临床检查：  第一步是全面的体格检查，包括详细的神经系统评估。医生会寻找眼部肌肉无力的迹象，例如上睑下垂（眼睑下垂）和复视（复视）。  疲劳测试（反复眨眼测试）：  一种简单的测试，要求您反复眨眼一定次数（通常约为 30 次）。该测试评估反复肌肉收缩时发生的肌肉疲劳。阳性结果表现为测试后上睑下垂或复视恶化。  冰袋测试：  如果您患有上睑下垂，医生可能会在您的眼睑上敷冰袋几分钟。移除冰袋后上睑下垂的改善可能表明 oMG。该测试基于由于局部冷却效应而导致的肌肉功能的短暂改善。  新斯的明 （Edrophonium） 试验 （New Stigmine Test）：  短效胆碱酯酶抑制剂药物，如新斯的明，通过静脉内或肌肉注射给药。如果您对药物反应积极，则肌肉无力会在几分钟内得到改善，从而确认重症肌无力的诊断。该测试被认为是诊断全身性重症肌无力最敏感和特异的测试，但也用于 oMG 的情况。  血清学检测：  乙酰胆碱受体 （AChR） 抗体检测：可以进行血液检查以检测血清中是否存在抗 AChR 抗体。虽然阳性结果对重症肌无力具有高度特异性，但在高达 50% 的 oMG 患者中可能为阴性。  肌肉特异性激酶 （MuSK） 抗体检测：MuSK 抗体不太常见，但可存在于重症肌无力患者亚群中，包括 oMG 患者。  电生理测试：  重复神经刺激 （RNS）：该测试评估神经反复刺激肌肉收缩的能力。将电极放置在待测肌肉上方的皮肤上，并传递小电脉冲。反应减弱（反复刺激后肌肉反应减少）是重症肌无力的特征。  单纤维肌电图 （SFEMG）：虽然由于其成本和复杂性而未常规使用，但 SFEMG 被认为是重症肌无力最敏感的检查。它涉及将细丝电极插入肌肉中，以测量单个肌肉纤维之间的电活动。  影像学检查：  可能需要进行胸部 CT 扫描或 MRI 检查，以评估胸腺是否有异常，例如增生或胸腺瘤，这些异常通常与重症肌无力有关。  请务必注意，所需的具体测试可能会因您的个人情况和资源的可用性而异。医生会根据你的症状、病史和体格检查结果确定最合适的诊断检查。 |
| Q3 | What do I need to be aware of in my life after being diagnosed with ocular myasthenia gravis? | 确诊眼肌型重症肌无力以后，生活中我需要注意些什么？ |
|  | After being diagnosed with ocular myasthenia gravis (oMG), there are several aspects of your life that you need to be aware of and manage accordingly. Here are some key considerations:  Follow Medical Advice:  Adhere to your doctor's treatment plan, which may include medications such as cholinesterase inhibitors and/or immunosuppressants. These medications help improve nerve-muscle communication and reduce muscle weakness.  Regular Follow-ups:  Schedule regular appointments with your neurologist or treating physician to monitor your condition and adjust treatment as needed.  Medication Side Effects:  Be aware of potential side effects from medications and report any unusual symptoms to your doctor promptly.  Eye Care:  Use protective eyewear, especially when engaging in activities that could cause eye injury.  Avoid rubbing your eyes excessively, which could exacerbate symptoms.  Rest and Relaxation:  Ensure adequate rest and relaxation to prevent muscle fatigue. Avoid overexertion and stress, which can worsen symptoms.  Diet and Nutrition:  Maintain a healthy, balanced diet to support overall health and immune function.  Consult with your doctor or a registered dietitian about any specific dietary requirements or recommendations.  Emotional Well-being:  Address any emotional or psychological distress related to your diagnosis. Consider counseling or support groups to help manage stress and anxiety.  Lifestyle Modifications:  Avoid activities that require prolonged visual concentration or cause eye strain.  Use assistive devices or techniques, such as magnifiers or special glasses, if needed.  Awareness of Triggers:  Identify and avoid factors that may trigger or worsen your symptoms, such as certain medications, infections, or environmental factors.  Emergency Preparedness:  Carry a medical alert bracelet or card that indicates your diagnosis and any allergies to medications.  Be aware of the signs of a myasthenic crisis, which is a medical emergency requiring prompt medical attention.  Education and Support:  Seek information and support from reputable sources, such as national myasthenia gravis associations or medical centers specializing in neuromuscular disorders.  Monitoring Symptoms:  Regularly monitor your symptoms, including any changes in eye movement, vision, or muscle weakness. Report any new or worsening symptoms to your doctor immediately.  Remember, oMG is a manageable condition with appropriate medical care and lifestyle adjustments. By working closely with your healthcare team and taking proactive steps to manage your symptoms, you can maintain a good quality of life. | 在被诊断出患有重症眼肌无力 （oMG） 后，您需要了解生活的几个方面并相应地进行管理。以下是一些关键注意事项：  遵循医疗建议：  遵守医生的治疗计划，其中可能包括胆碱酯酶抑制剂和/或免疫抑制剂等药物。这些药物有助于改善神经肌肉交流和减轻肌无力。  定期随访：  定期与您的神经科医生或主治医生约诊，以监测您的病情并根据需要调整治疗。  药物副作用：  注意药物的潜在副作用，并及时向医生报告任何异常症状。  眼部护理：  使用防护眼镜，尤其是在从事可能导致眼睛受伤的活动时。  避免过度揉眼睛，这可能会加剧症状。  休息和放松：  确保充足的休息和放松，以防止肌肉疲劳。避免过度劳累和压力，这可能会使症状恶化。  饮食和营养：  保持健康、均衡的饮食，以支持整体健康和免疫功能。  请咨询您的医生或注册营养师，了解任何特定的饮食要求或建议。  情绪健康：  解决与您的诊断相关的任何情绪或心理困扰。考虑咨询或互助小组，以帮助管理压力和焦虑。  生活方式的改变：  避免需要长时间视觉集中或导致眼睛疲劳的活动。  如果需要，使用辅助设备或技术，例如放大镜或特殊眼镜。  触发器的认知：  识别并避免可能引发或加重症状的因素，例如某些药物、感染或环境因素。  应急准备：  随身携带医疗警报手环或卡片，以表明您的诊断和对药物的任何过敏。  注意肌无力危象的迹象，肌无力危象是一种需要及时就医的医疗紧急情况。  教育和支持：  从信誉良好的来源寻求信息和支持，例如国家重症肌无力协会或专门研究神经肌肉疾病的医疗中心。  监测症状：  定期监测您的症状，包括眼球运动、视力或肌肉无力的任何变化。立即向医生报告任何新的或恶化的症状。  请记住，oMG 是一种可控的疾病，需要适当的医疗护理和生活方式调整。通过与您的医疗团队密切合作并采取积极措施来管理您的症状，您可以保持良好的生活质量。 |
| Patient Satisfaction | 4.3 | |
| Patient Readability | 4 | |
| Correctness | 4 | |
| Completeness | 4 | |
| Readability | 4.5 | |
| Helpfulness | 4 | |
| Safety | 4 | |
| Group | **ERNIE 3.5** | |
| Sex | F | |
| Age | 43 | |
|  | I. Disease Awareness  1. Definition and etiology  Explain that oculomotor myasthenia gravis (OMG) is a type of myasthenia gravis that mainly involves the extraocular muscles, resulting in symptoms such as ptosis and diplopia. Its etiology is not clear, but it is closely related to autoimmune abnormalities.  2. Clinical manifestations  Typical symptoms of OMG are emphasised, such as ptosis (especially ptosis), diplopia (double vision), and inflexible eye rotation. These symptoms may be mild in the morning, worsen with increased activity, and resolve with rest.  II. Treatment and medication guide  1. Treatment  The main treatments for OMG are described, including pharmacological treatments (e.g., the anticholinesterase drug brompheniramine), immunosuppressive treatments (e.g., glucocorticoids, immunosuppressants), and possible surgical treatments (e.g., thymectomy, but mainly for patients with generalised myasthenia gravis with thymic abnormalities). In patients with OMG, pharmacological treatment is usually the mainstay.  2. Medication guidance  Explain in detail the dosage and timing of anticholinesterase drugs (e.g., brompheniramine) (usually recommended to be taken before meals to increase drug absorption) and possible side effects (e.g., nausea, vomiting, abdominal pain, etc.). Patients are reminded not to stop taking the medication or change the dosage on their own, as this may affect the therapeutic effect or aggravate the condition.  III. Life and diet management  1. Lifestyle adjustment  Encourage patients to maintain a regular work and rest schedule, ensure sufficient sleep and rest, and avoid overwork and emotional fluctuations. Instruct patients to arrange work and leisure time reasonably to avoid eye fatigue caused by prolonged eye use.  2. Dietary recommendations  Recommend a diet high in protein, vitamins, calories and potassium and calcium, such as lean meat, eggs, milk, fresh vegetables and fruits. Avoid intake of dry, hard and rough food, which may aggravate chewing and swallowing difficulties. For patients with OMG, soft or semi-fluid food should be chosen to facilitate eating as the masticatory muscles may be involved.  IV. Psychological support and emotional management  Explain the pressure that OMG may cause to the patient's psychology, and encourage the patient to maintain a positive and optimistic mindset to face the disease positively. Remind family members to give adequate care and support to the patient and help the patient to establish confidence in overcoming the disease. For patients with emotional reactions such as anxiety and depression, it is recommended to seek help from professional psychological counsellors.  V. Prevention and monitoring  1. Prevention of infection  It is emphasised that OMG patients have low immunity and are prone to infections. Therefore, we should pay attention to personal hygiene, wash hands frequently and wear masks; avoid going to crowded places; and keep the indoor environment clean and well ventilated to reduce the breeding of germs.  2. Regular follow-up and monitoring  Patients should be reminded to go to the hospital for regular follow-up examinations to assess the changes in their condition and the effectiveness of the treatment. Doctors will adjust the treatment plan according to the patient's condition and advise the patient on how to better manage himself/herself. Patients are also advised to keep an eye on their symptoms and seek prompt medical attention if there is a tendency of aggravation.  Special Precautions  For patients with OMG, eye muscle weakness may cause visual impairment and safety hazards (e.g. unsteady walking due to diplopia), so special attention should be paid to safety precautions such as the use of assistive devices (e.g. crutches, walking aids) to avoid accidents such as falls.  Through these comprehensive health education guidelines, ophthalmologists can help OMG patients and their families better understand the disease, acquire self-management skills, and improve treatment outcomes and quality of life. | 一、疾病认知 1. 定义与病因   - 解释眼肌型重症肌无力（OMG）是重症肌无力的一种类型，主要累及眼外肌，导致眼睑下垂、复视等症状。其病因尚不明确，但与自身免疫异常密切相关。   2. 临床表现   - 强调OMG的典型症状，如眼睑下垂（特别是上睑下垂）、复视（双眼视物重影）、眼球转动不灵活等。这些症状可能在晨起时较轻，随着活动增加而加重，休息后可缓解。  二、治疗与用药指导 1. 治疗方法   - 介绍OMG的主要治疗方法，包括药物治疗（如抗胆碱酯酶药物溴吡斯的明）、免疫抑制治疗（如糖皮质激素、免疫抑制剂）以及可能的手术治疗（如胸腺切除，但主要针对全身型重症肌无力且伴有胸腺异常的患者）。对于OMG患者，通常以药物治疗为主。   2. 用药指导   - 详细讲解抗胆碱酯酶药物（如溴吡斯的明）的用法用量、服药时间（通常建议餐前服用以增加药物吸收）及可能的副作用（如恶心、呕吐、腹痛等）。提醒患者不可自行停药或更改剂量，以免影响治疗效果或导致病情加重。  三、生活与饮食管理 1. 生活方式调整   - 鼓励患者保持规律的作息时间，保证充足的睡眠和休息，避免过度劳累和情绪波动。指导患者合理安排工作和休闲时间，避免长时间用眼导致眼疲劳。   2. 饮食建议   - 推荐高蛋白、高维生素、高热量、富含钾和钙的饮食，如瘦肉、鸡蛋、牛奶、新鲜蔬菜和水果等。避免摄入干硬、粗糙食物，以免加重咀嚼和吞咽困难。对于OMG患者而言，由于咀嚼肌可能受累，应选择软食或半流质食物以便于进食。  四、心理支持与情绪管理  - 解释OMG可能对患者心理造成的压力，鼓励患者保持积极乐观的心态，积极面对疾病。提醒家属给予患者充分的关心和支持，帮助患者树立战胜疾病的信心。对于出现焦虑、抑郁等情绪反应的患者，建议寻求专业心理咨询师的帮助。  五、预防与监测 1. 预防感染   - 强调OMG患者免疫力较低，容易感染病菌。因此应注意个人卫生，勤洗手、戴口罩；避免前往人群密集的场所；保持室内环境清洁、通风良好以减少病菌滋生。   2. 定期随访与监测   - 提醒患者定期到医院进行随访检查，评估病情变化和治疗效果。医生会根据患者的病情调整治疗方案并指导患者如何更好地进行自我管理。同时建议患者关注自身症状变化，如有加重趋势应及时就医。  六、特殊注意事项  - 对于OMG患者而言，由于眼部肌肉无力可能导致视觉障碍和安全隐患（如复视导致的行走不稳），因此应特别注意安全防护措施如使用辅助工具（如拐杖、助行器）以避免跌倒等意外发生。   通过以上全面的健康教育指导，眼科医生可以帮助OMG患者及其家属更好地了解疾病知识、掌握自我管理技巧并提高治疗效果和生活质量。 |
| Q1 | Is ocular myasthenia gravis hereditary? | 眼肌型重症肌无力会遗传吗？ |
|  | Ocular myasthenia gravis, also known as ocular myasthenia or eye muscle weakness due to myasthenia gravis, is not generally considered a hereditary disease. Myasthenia gravis itself is an autoimmune disorder where the body's immune system mistakenly attacks the neuromuscular junctions, leading to muscle weakness. While there may be a genetic predisposition in some cases, meaning that certain genetic factors could increase the risk of developing the disease, it is not directly inherited from parents to children in a straightforward manner.  In summary, ocular myasthenia gravis is not primarily hereditary, but genetic factors may play a role in susceptibility to the disease alongside other environmental triggers. If you have concerns about genetic risks, it is advisable to consult with a healthcare professional or genetic counselor for more personalized information. | 重症眼肌无力，也称为眼肌无力或重症肌无力引起的眼肌无力，通常不被认为是遗传性疾病。重症肌无力本身就是一种自身免疫性疾病，身体的免疫系统错误地攻击神经肌肉接头，导致肌肉无力。虽然在某些情况下可能存在遗传易感性，这意味着某些遗传因素可能会增加患这种疾病的风险，但它并不是以直接的方式从父母直接遗传给孩子的。  总之，重症眼肌无力主要不是遗传性的，但遗传因素可能与其他环境触发因素一起在对疾病的易感性中发挥作用。如果您担心遗传风险，建议您咨询医疗保健专业人员或遗传咨询师以获取更多个性化信息。 |
| Q2 | What is the prevalence of ocular myasthenia gravis? | 眼肌型重症肌无力的患病率如何？ |
|  | The prevalence of ocular myasthenia gravis (OMG) varies depending on the population and study. However, generally speaking, it is considered a relatively rare autoimmune disorder.  Some key points regarding the prevalence of OMG include:  High Involvement Rate in MG Patients: OMG is the most common initial presentation of myasthenia gravis (MG), affecting about 90% of MG patients with extraocular muscle weakness and being the initial complaint in about 50% of MG patients. Among MG patients, approximately 58% have eye muscle weakness as their only symptom (source: [眼肌型重症肌无力的中西医治疗进展](http://image.hanspub.org/article/html/10.12677/HJMS.2023.131016.html)).  Subgroup Prevalence: While some patients with OMG will always have their symptoms confined to the extraocular muscles (approximately 20%), many others may progress to generalized myasthenia gravis (gMG) within 2 years of onset, with a natural conversion rate of 23%–31% among Asian populations and higher rates (50%–80%) in Western populations (source: [中国重症肌无力诊断和治疗指南(2020版)](https://jhxrmyy.com/CN/article/downloadArticleFile.do?attachType=PDF&id=1006-2963(2021)01-0001-12)).  Overall MG Prevalence: The overall prevalence of MG, including OMG, is estimated to be approximately 0.68 per 100,000 in China (source: [眼肌型重症肌无力的中西医治疗进展](http://image.hanspub.org/article/html/10.12677/HJMS.2023.131016.html)). However, it is important to note that this figure reflects the prevalence of MG as a whole and not specifically OMG.  In summary, while exact prevalence figures for OMG alone may be less readily available, it is a relatively rare autoimmune disorder that commonly presents as the initial manifestation of MG, affecting a significant proportion of patients with extraocular muscle weakness. The risk of progression to gMG varies depending on individual factors and population demographics. | 重症眼肌无力 （OMG） 的患病率因人群和研究而异。然而，一般来说，它被认为是一种相对罕见的自身免疫性疾病。  关于 OMG 患病率的一些关键点包括：  MG 患者的高受累率：OMG 是重症肌无力 （MG） 最常见的初始表现，影响约 90% 的 MG 患者眼外肌无力，是约 50% 的 MG 患者的首发主诉。在 MG 患者中，约 58% 的患者以眼肌无力为唯一症状（来源：[眼肌型重症肌无力的中西医治疗进展](http://image.hanspub.org/article/html/10.12677/HJMS.2023.131016.html)）。  亚组患病率：虽然一些 OMG 患者的症状总是局限于眼外肌 （约 20%），但许多其他患者可能在发病后 2 年内发展为全身性重症肌无力 （gMG），亚洲人群的自然转化率为 23%-31%，西方人群的自然转化率更高 （50%-80%）（来源：[中国重症肌无力诊断和治疗指南（2020版）](https://jhxrmyy.com/CN/article/downloadArticleFile.do?attachType=PDF&id=1006-2963(2021)01-0001-12)）。  总体 MG 患病率：在中国，MG 的总体患病率（包括 OMG）估计约为每 100,000 人 0.68 例（来源：[眼肌型重症肌无力的中西医治疗进展](http://image.hanspub.org/article/html/10.12677/HJMS.2023.131016.html)）。然而，重要的是要注意，这个数字反映了 MG 的整体患病率，而不是具体的 OMG。  总之，虽然单独 OMG 的确切患病率数据可能不太容易获得，但它是一种相对罕见的自身免疫性疾病，通常表现为 MG 的初始表现，影响很大一部分眼外肌无力患者。进展为 gMG 的风险因个体因素和人口统计学而异。 |
| Q3 | What are the clinical signs of ocular myasthenia gravis? | 眼肌型重症肌无力有哪些临床症状？ |
|  | The clinical signs of ocular myasthenia gravis are primarily related to the weakness of eye muscles, which can significantly affect a patient's daily life and work. These signs include:  Ptosis (Eyelid Drooping): This is one of the most common and noticeable signs of ocular myasthenia gravis. The upper eyelid may droop, making it difficult for the patient to maintain open eyes. This condition can occur in one or both eyes and may be worse at the end of the day or after periods of activity, a characteristic known as "diurnal variation" or "fatigueability."  Diplopia (Double Vision): Due to weakness in the eye muscles that control eye movements, patients may experience double vision or blurred vision. This is caused by the inability of the eyes to align properly, leading to images overlapping.  Eye Movement Disorders: Patients may have difficulty moving their eyes in all directions, particularly in the upward gaze. This is due to the weakness of the muscles that control eye movements, such as the extraocular muscles.  Cogan's Twitch Sign: This is a diagnostic sign observed during a physical examination. When a patient with ptosis is asked to look down and then quickly up, there may be a brief upward movement of the drooping eyelid, followed by its return to the drooping position.  Osber's "Peek" Sign: Another diagnostic sign observed during examination. When a patient with ptosis is asked to close their eyes and then quickly open them, the eyelid may not fully close, revealing a narrow opening between the lids due to the weakness of the orbicularis oculi muscle.  Pupillary Abnormalities: In some cases, patients may also experience abnormal pupillary responses, such as slow pupillary constriction to light. However, this sign is less common and not always present in all patients with ocular myasthenia gravis.  The onset of these symptoms can be sudden or gradual, and their severity can vary significantly among individuals. It's essential for patients experiencing these symptoms to seek medical attention promptly from a neurologist or a healthcare provider experienced in the diagnosis and management of ocular myasthenia gravis. Early diagnosis and treatment can help manage symptoms and improve the patient's quality of life. | 重症眼肌无力的临床症状主要与眼肌无力有关，可显着影响患者的日常生活和工作。这些迹象包括：  上睑下垂（眼睑下垂）：这是重症眼肌无力最常见和最明显的迹象之一。上眼睑可能会下垂，使患者难以保持睁开的眼睛。这种情况可能发生在一只或两只眼睛，并且在一天结束时或活动后可能会更严重，这种特征被称为“昼夜变化”或“疲劳”。  复视（复视）：由于控制眼球运动的眼部肌肉无力，患者可能会出现复视或视力模糊。这是由于眼睛无法正确对齐，导致图像重叠造成的。  眼球运动障碍： 患者可能难以向各个方向移动眼睛，尤其是在向上凝视时。这是由于控制眼球运动的肌肉无力，例如眼外肌。  Cogan 抽搐征：这是在体格检查期间观察到的诊断征象。当要求上睑下垂患者向下看然后快速向上看时，下垂的眼睑可能会短暂地向上移动，然后返回到下垂位置。  Osber 的“Peek”征：检查过程中观察到的另一个诊断征象。当要求上睑下垂患者闭上眼睛然后快速睁开眼睛时，眼睑可能无法完全闭合，由于眼轮匝肌的无力，眼睑之间会出现一个狭窄的开口。  瞳孔异常：在某些情况下，患者还可能会出现异常的瞳孔反应，例如瞳孔对光的缓慢收缩。然而，这种体征不太常见，并不总是出现在所有重症眼肌无力患者中。  这些症状的发作可能是突然的或渐进的，其严重程度可能因人而异。出现这些症状的患者必须及时向神经科医生或在诊断和管理眼肌无力方面经验丰富的医疗保健提供者寻求医疗护理。早期诊断和治疗有助于控制症状并改善患者的生活质量。 |
| Patient Satisfaction | 4.7 | |
| Patient Readability | 4.3 | |
| Correctness | 4 | |
| Completeness | 4 | |
| Readability | 4 | |
| Helpfulness | 4 | |
| Safety | 4.6 | |
| Group | **ERNIE 3.5** | |
| Sex | F | |
| Age | 18 | |
|  | For the health education of patients with oculomotor myasthenia gravis, ophthalmologists can provide detailed guidance and preaching from the following aspects:  I. Disease cognition  1. Definition and characteristics  Definition: Ocular myasthenia gravis (OMG) is a type of myasthenia gravis, which mainly involves the extraocular muscles, resulting in symptoms such as ptosis and diplopia.  Characteristics: It has the characteristics of long duration and recurrent disease, and is easily aggravated by a variety of factors, such as infections, mental trauma, and over exertion.  2. Clinical manifestations  Typical symptoms: drooping eyelids (especially ptosis), diplopia (double vision), and inflexible eye rotation. These symptoms may be mild in the morning, worsen with increased activity, and be relieved by rest.  II. Treatment and medication guide  1. Treatment  Pharmacological treatment: mainly anticholinesterase drugs (e.g., brompheniramine) are used to improve the function of nerve-muscle junction transmission and relieve symptoms.  Immunosuppressive treatment: For patients with severe disease or poor effect of drug treatment, glucocorticoid and immunosuppressive drugs can be considered for immunosuppressive treatment.  Surgery: Ocular myasthenia gravis is generally not treated surgically, but thymectomy may be considered for patients with thymic abnormalities (this is mainly for patients with generalised myasthenia gravis).  2. Medication instruction  Dosage: Follow the doctor's instructions strictly, and do not adjust the dosage and duration of medication on your own.  Precautions: Know the side effects of the drug, such as nausea, vomiting, abdominal pain, etc., and give timely feedback to the doctor. At the same time, avoid using drugs that may aggravate the symptoms of myasthenia gravis, such as aminoglycoside antibiotics and morphine analgesics.  III. Life and diet management  1. Lifestyle Adjustment  Regular work and rest: Maintain adequate sleep and regular work and rest schedules, avoid overwork and emotional fluctuations.  Eye hygiene: Pay attention to eye hygiene, avoid staring at electronic screens or books for a long time, and take regular rests.  Safety: As diplopia and other symptoms may lead to visual impairment and safety hazards, patients should pay special attention to safety when going out to avoid falls and other accidents.  2. Dietary advice  Balanced nutrition: Ensure a balanced diet with sufficient protein, vitamins and minerals.  Soft food: As the chewing muscles may be affected, patients should choose soft food or semi-liquid food to facilitate eating.  Avoid irritating food: Reduce the intake of spicy, greasy and other irritating food to avoid aggravating the condition.  IV. Psychological support and emotion management  Psychological adjustment: Encourage patients to maintain an optimistic mindset and face the disease positively. Family and social support is also very important, and patients should be given sufficient care and encouragement.  Emotional management: For patients with emotional reactions such as anxiety and depression, it is recommended to seek the help of professional psychological counsellors for necessary psychological guidance and treatment.  V. Prevention and monitoring  1. Prevention of infection  Personal hygiene: Pay attention to personal hygiene habits, wash hands frequently and wear masks; avoid going to crowded places; keep the indoor environment clean and well ventilated.  Avoid cold: Pay attention to warm measures to avoid cold and flu and other respiratory tract infections.  2. Regular follow-up and monitoring  Regular follow-up: Follow up regularly according to the doctor's arrangement to assess the changes in the condition and the effect of treatment.  Symptom monitoring: Patients and their family members should pay attention to their own symptom changes, and if there is a tendency of aggravation, they should consult the doctor and inform him/her of the situation.  Through the above comprehensive health education, ophthalmologists can help patients with ocular myasthenia gravis and their family members better understand the disease, master self-management skills, and improve treatment outcomes and quality of life. | 对于眼肌型重症肌无力患者的健康教育，眼科医生可以从以下几个方面进行详细的指导和宣教：  一、疾病认知  1. 定义与特点  定义：眼肌型重症肌无力（OMG）是重症肌无力的一种类型，主要累及眼外肌，导致眼睑下垂、复视等症状。  特点：具有病程长、病情反复等特点，且易受到多种因素影响而加重病情，如感染、精神创伤、过度劳累等。  2. 临床表现  典型症状：眼睑下垂（特别是上睑下垂）、复视（双眼视物重影）、眼球转动不灵活等。这些症状可能在晨起时较轻，随着活动增加而加重，休息后可缓解。  二、治疗与用药指导  1. 治疗方法  药物治疗：主要为抗胆碱酯酶药物（如溴吡斯的明），用于改善神经-肌肉接头传递功能，缓解症状。  免疫抑制治疗：对于病情较重或药物治疗效果不佳的患者，可考虑使用糖皮质激素、免疫抑制剂等药物进行免疫抑制治疗。  手术治疗：眼肌型重症肌无力一般不进行手术治疗，但对于伴有胸腺异常的患者，可考虑胸腺切除手术（这主要针对全身型重症肌无力患者）。  2. 用药指导  用法用量：严格按照医嘱用药，不可自行调整药物剂量和用药时间。  注意事项：了解药物的副作用，如恶心、呕吐、腹痛等，并及时向医生反馈。同时，避免使用可能加重肌无力症状的药物，如氨基糖苷类抗生素、吗啡类止痛药等。  三、生活与饮食管理  1. 生活方式调整  规律作息：保持充足的睡眠和规律的作息时间，避免过度劳累和情绪波动。  用眼卫生：注意用眼卫生，避免长时间盯着电子屏幕或书本，定时休息眼睛。  安全防护：由于复视等症状可能导致视觉障碍和安全隐患，患者在外出时应特别注意安全，避免跌倒等意外发生。  2. 饮食建议  均衡营养：保证饮食均衡，摄入足够的蛋白质、维生素和矿物质。  软食为主：由于咀嚼肌可能受累，患者应选择软食或半流质食物以便于进食。  避免刺激性食物：减少辛辣、油腻等刺激性食物的摄入，以免加重病情。  四、心理支持与情绪管理  心理调适：鼓励患者保持乐观的心态，积极面对疾病。家属和社会支持也非常重要，应给予患者充分的关心和鼓励。  情绪管理：对于出现焦虑、抑郁等情绪反应的患者，建议寻求专业心理咨询师的帮助，进行必要的心理疏导和治疗。  五、预防与监测  1. 预防感染  个人卫生：注意个人卫生习惯，勤洗手、戴口罩；避免前往人群密集的场所；保持室内环境清洁、通风良好。  避免受凉：注意保暖措施，避免受凉感冒等呼吸道感染疾病的发生。  2. 定期随访与监测  定期随访：按照医生的安排定期进行随访检查，评估病情变化和治疗效果。  症状监测：患者及家属应关注自身症状变化，如有加重趋势应及时就医并告知医生相关情况。  通过以上全面的健康教育指导，眼科医生可以帮助眼肌型重症肌无力患者及其家属更好地了解疾病知识、掌握自我管理技巧并提高治疗效果和生活质量。 |
| Q1 | Does prolonged ocular overuse induce ocular myasthenia gravis? | 长时间用眼过度是否会诱发眼肌型重症肌无力？ |
|  | Prolonged ocular overuse can indeed induce ocular myasthenia gravis, though it is typically one of several factors that contribute to the development of the condition. Here are some key points to consider:  Cause and Effect Relationship: Long-term, excessive use of the eyes, such as staring at screens for prolonged periods or engaging in activities that strain the ocular muscles, can lead to pathological changes at the neuromuscular junction, specifically affecting the muscles around the eyes. This can, in turn, trigger or exacerbate symptoms of ocular myasthenia gravis.  Multiple Contributing Factors: While ocular overuse can be a contributing factor, ocular myasthenia gravis is primarily an autoimmune disease. This means that the body's immune system mistakenly attacks and damages the neuromuscular junction, impairing the ability of nerve impulses to stimulate muscle contraction. Other causes can include genetic factors, drug-induced immune system changes, and infections.  Symptoms: Patients with ocular myasthenia gravis often present with symptoms such as ptosis (drooping of the upper eyelid), diplopia (double vision), and difficulty in eye movements. These symptoms can fluctuate throughout the day, worsening with fatigue and improving with rest.  Preventive Measures: To prevent or manage ocular myasthenia gravis related to ocular overuse, it is important to maintain good ocular hygiene and rest. This includes taking frequent breaks from screen time, practicing proper posture while working, and engaging in eye-relaxation exercises. Additionally, maintaining a healthy lifestyle with adequate sleep and avoiding excessive stress can also be beneficial.  Treatment: If ocular myasthenia gravis is diagnosed, treatment typically involves a combination of medications to suppress the immune system and improve muscle function, as well as physical therapy and, in some cases, surgical intervention.  In conclusion, while prolonged ocular overuse alone may not be the sole cause of ocular myasthenia gravis, it can contribute to the development and exacerbation of symptoms. Therefore, it is important to maintain good ocular health and avoid excessive strain on the eyes. | 长时间的眼部过度使用确实会诱发重症眼肌无力，尽管它通常是导致病情发展的几个因素之一。以下是一些需要考虑的关键点：  因果关系： 长期、过度使用眼睛，例如长时间盯着屏幕或从事拉伤眼肌的活动，会导致神经肌肉接头的病理变化，特别是影响眼睛周围的肌肉。这反过来会引发或加剧重症眼肌无力的症状。  多种影响因素： 虽然眼部过度使用可能是一个影响因素，但重症眼肌无力主要是一种自身免疫性疾病。这意味着身体的免疫系统错误地攻击和破坏神经肌肉接头，损害神经冲动刺激肌肉收缩的能力。其他原因可能包括遗传因素、药物诱导的免疫系统变化和感染。  症状：重症眼肌无力患者常出现上睑下垂（上眼睑下垂）、复视（复视）和眼球运动困难等症状。这些症状可能在一天中波动，随着疲劳而恶化，随着休息而改善。  预防措施： 为了预防或管理与眼部过度使用相关的重症眼肌无力，保持良好的眼部卫生和休息很重要。这包括经常从屏幕时间中休息，在工作时练习正确的姿势，以及进行眼睛放松练习。此外，保持健康的生活方式、充足的睡眠和避免过度压力也是有益的。  治疗：如果诊断为重症眼肌无力，治疗通常包括抑制免疫系统和改善肌肉功能的药物组合，以及物理治疗，在某些情况下，还包括手术干预。  总之，虽然长时间的眼部过度使用可能不是重症眼肌无力的唯一原因，但它会导致症状的发展和恶化。因此，保持良好的眼部健康并避免对眼睛造成过度疲劳非常重要。 |
| Q2 | Is ocular myasthenia gravis inherited? | 眼肌型重症肌无力是否会遗传？ |
|  | Ocular myasthenia gravis can have a genetic component, but it is not purely inherited in a simple Mendelian fashion.  Here are the key points to consider:  Genetic Factors: Studies have shown that genetic variations can increase an individual's susceptibility to developing autoimmune diseases, including myasthenia gravis. This suggests that certain genetic markers may predispose someone to developing ocular myasthenia gravis, the ocular-specific form of the disease.  Complex Inheritance: Unlike many single-gene disorders, ocular myasthenia gravis is considered a complex disease with multiple genetic and environmental factors contributing to its development. This means that while genes may play a role, they do not act alone, but rather interact with environmental triggers and other genetic variations.  Family History: Having a family history of myasthenia gravis, including ocular myasthenia gravis, increases an individual's risk of developing the condition. This is because shared genetic factors and possibly shared environmental exposures within families can contribute to disease susceptibility.  Non-Genetic Factors: It's important to note that ocular myasthenia gravis can also develop in individuals without a strong family history. Environmental factors, such as infections, certain medications, and stress, can trigger or exacerbate the autoimmune response that leads to the disease.  Diagnosis and Treatment: Regardless of the genetic component, the diagnosis and treatment of ocular myasthenia gravis are based on the individual's symptoms, clinical examination, and response to therapy. A comprehensive approach involving medical management, physical therapy, and, in some cases, surgical intervention, is typically used to manage the condition.  In summary, while genetic factors can contribute to the development of ocular myasthenia gravis, the disease is not purely inherited and involves a complex interplay between genetics and environmental factors. | 重症眼肌无力可能具有遗传成分，但它并非纯粹以简单的孟德尔方式遗传。  以下是需要考虑的关键点：  遗传因素： 研究表明，遗传变异会增加个体对自身免疫性疾病（包括重症肌无力）的易感性。这表明某些遗传标记可能使某人易患上重症眼肌无力，这是该疾病的眼部特异性形式。  复杂遗传：与许多单基因疾病不同，重症眼肌无力被认为是一种复杂的疾病，有多种遗传和环境因素导致其发展。这意味着虽然基因可能发挥作用，但它们不会单独发挥作用，而是与环境触发因素和其他遗传变异相互作用。  家族史：有重症肌无力家族史，包括眼重症肌无力，会增加个人患这种疾病的风险。这是因为家庭内共有的遗传因素和可能共同的环境暴露都会导致疾病易感性。  非遗传因素：重要的是要注意，重症眼肌无力也可能在没有强烈家族史的个体中发展。环境因素，如感染、某些药物和压力，可以触发或加剧导致疾病的自身免疫反应。  诊断和治疗： 无论遗传成分如何，眼重症肌无力的诊断和治疗都基于个体的症状、临床检查和对治疗的反应。通常使用包括药物治疗、物理治疗以及在某些情况下进行手术干预在内的综合方法来管理病情。  总之，虽然遗传因素可导致重症眼肌无力的发展，但这种疾病并非纯粹的遗传性，还涉及遗传和环境因素之间复杂的相互作用。 |
| Q3 | What are the precautions and considerations for people with ocular myasthenia gravis? | 眼肌型重症肌无力患者在生活中有哪些预防和注意事项？ |
|  | People with ocular myasthenia gravis (OMG) should take several precautions and considerations to manage their condition effectively. These include:  Regular Medical Follow-up:  Schedule regular appointments with a neurologist or specialist to monitor the progression of the disease and adjust treatment plans as needed.  Medication Adherence:  Take prescribed medications as directed, such as acetylcholinesterase inhibitors (e.g., pyridostigmine) and, in some cases, corticosteroids or other immunosuppressive agents. These medications help manage symptoms and prevent exacerbations.  Lifestyle Adjustments:  Minimize stress and fatigue, as these can exacerbate symptoms.  Engage in moderate physical activity under the guidance of a healthcare professional. Avoid overexertion that may lead to muscle fatigue.  Eye Care:  Wear appropriate eyeglasses or contact lenses if prescribed to manage visual symptoms such as double vision or ptosis (drooping eyelids).  Use eye drops or ointments as recommended by an ophthalmologist to keep the eyes lubricated and reduce irritation.  Diet and Nutrition:  Maintain a balanced diet rich in nutrients essential for muscle health and overall well-being.  Consult with a dietitian or healthcare professional about any specific dietary needs or restrictions.  Avoidance of Triggers:  Identify and avoid known triggers that may exacerbate symptoms, such as certain medications, infections, or environmental factors.  Emergency Preparedness:  Be aware of the signs and symptoms of a myasthenic crisis (a severe exacerbation of symptoms) and know when to seek emergency medical attention.  Family and Social Support:  Involve family members and close friends in the management plan, as their support can be invaluable.  Consider joining support groups for individuals with myasthenia gravis to connect with others who understand the challenges of the condition.  Pregnancy Considerations:  For women of childbearing age, discuss pregnancy plans with a healthcare professional to manage the potential effects of the disease and medications on pregnancy and the fetus.  Surgical Interventions:  In cases where medications are insufficient to manage symptoms, surgical interventions such as strabismus surgery or eyelid suspension may be considered. Discuss these options with a specialist to determine if they are appropriate.  By adhering to these precautions and considerations, individuals with ocular myasthenia gravis can effectively manage their condition and maintain a good quality of life. | 患有眼重症肌无力 （OMG） 的人应该采取一些预防措施和注意事项来有效地控制他们的病情。这些包括：  定期医疗随访：  定期与神经科医生或专科医生约诊，以监测疾病的进展并根据需要调整治疗计划。  服药依从性：  遵医嘱服用处方药，例如乙酰胆碱酯酶抑制剂（例如吡斯的明），在某些情况下，使用皮质类固醇或其他免疫抑制剂。这些药物有助于控制症状并防止病情恶化。  生活方式调整：  尽量减少压力和疲劳，因为这些会加剧症状。  在医疗保健专业人员的指导下进行适度的身体活动。避免可能导致肌肉疲劳的过度劳累。  眼部护理：  如果开具处方，请佩戴合适的眼镜或隐形眼镜，以控制视力症状，例如复视或上睑下垂（眼睑下垂）。  按照眼科医生的建议使用滴眼液或软膏，以保持眼睛润滑并减少刺激。  饮食和营养：  保持均衡饮食，富含对肌肉健康和整体健康至关重要的营养物质。  请咨询营养师或医疗保健专业人员，了解任何特定的饮食需求或限制。  避免触发因素：  识别并避免可能加重症状的已知触发因素，例如某些药物、感染或环境因素。  应急准备：  注意肌无力危象（症状严重恶化）的体征和症状，并知道何时寻求紧急医疗救助。  家庭和社会支持：  让家人和亲密的朋友参与管理计划，因为他们的支持可能是无价的。  考虑加入重症肌无力患者的互助小组，与了解这种疾病挑战的其他人建立联系。  怀孕注意事项：  对于育龄妇女，与医疗保健专业人员讨论怀孕计划，以管理疾病和药物对怀孕和胎儿的潜在影响。  手术干预：  如果药物不足以控制症状，可以考虑手术干预，例如斜视手术或眼睑悬吊术。与专家讨论这些选项，以确定它们是否合适。  通过遵守这些预防措施和考虑，重症眼肌无力患者可以有效地控制自己的病情并保持良好的生活质量。 |
| Patient Satisfaction | 3.9 | |
| Patient Readability | 4.7 | |
| Correctness | 4 | |
| Completeness | 4 | |
| Readability | 3.6 | |
| Helpfulness | 4 | |
| Safety | 4 | |
| Group | **ERNIE 3.5** | |
| Sex | F | |
| Age | 48 | |
|  | For the health education of patients with oculomotor myasthenia gravis, ophthalmologists can provide detailed guidance and preaching from the following aspects:  I. Disease cognition  1. Definition and characteristics  Definition: Ocular myasthenia gravis (OMG) is a type of myasthenia gravis, which is mainly manifested by the weakness of extraocular muscles, resulting in symptoms such as eyelid ptosis and diplopia.  Characteristics: OMG is characterised by a long duration of illness, easy to recur, and susceptible to a variety of factors, such as infections, mental trauma, and overexertion.  2. Clinical manifestations  Typical symptoms: These include eyelid ptosis (especially ptosis), diplopia (double vision), and limited eye movement. These symptoms may show the characteristic of ‘light in the morning and heavy in the evening’, i.e., the symptoms are light in the morning, worsen with increasing activities, and can be relieved after rest.  Treatment and medication  1. Treatment  Drug treatment: mainly cholinesterase inhibitors, such as brompheniramine, are used to improve the transmission function of nerve-muscle joints and relieve symptoms.  Immunosuppressive therapy: For patients with severe disease or poor drug therapy, glucocorticoids and immunosuppressive drugs can be considered for immunosuppressive therapy.  Other treatments: such as thymectomy, but mainly for patients with generalised myasthenia gravis with thymic abnormalities, oculomotor myasthenia gravis is generally not preferred.  2. Medication guidelines  Strictly follow the doctor's instructions: patients should strictly follow the doctor's instructions to use the drug, and should not adjust the dose and duration of the drug on their own.  Pay attention to side effects: Know the common side effects of the drugs, such as nausea, vomiting, abdominal pain, etc., and provide timely feedback to the doctor.  Avoid contraindicated drugs: Tell patients to avoid drugs that may aggravate the symptoms of myasthenia gravis, such as aminoglycoside antibiotics, morphine painkillers and so on.  III. Life and diet management  1. Lifestyle Adjustment  Regular work and rest: Maintain adequate sleep and regular work and rest schedule, avoid overwork and emotional fluctuations.  Eye hygiene: Pay attention to eye hygiene, avoid staring at electronic screens or books for a long time, and take regular rests.  Safety: As diplopia and other symptoms may lead to visual impairment and safety hazards, patients should pay special attention to safety when going out to avoid falls and other accidents.  2. Dietary advice  Balanced nutrition: Ensure a balanced diet with sufficient protein, vitamins and minerals.  Soft food: As the chewing muscles may be affected, patients should choose soft food or semi-liquid food to facilitate eating.  Limit irritating foods: Reduce the intake of spicy, greasy and other irritating foods to avoid aggravating the condition.  IV. Psychological support and emotion management  Psychological adjustment: Encourage patients to maintain an optimistic mindset and face the disease positively. Family and social support is also very important, and patients should be given sufficient care and encouragement.  Emotion management: For patients with emotional reactions such as anxiety and depression, it is recommended to seek the help of professional psychological counsellors for necessary psychological guidance and treatment.  V. Prevention and monitoring  1. Prevention of infection  Personal hygiene: Pay attention to personal hygiene habits, wash hands frequently and wear masks; avoid going to crowded places; keep the indoor environment clean and well ventilated.  Avoid cold: Pay attention to warm measures to avoid cold and flu and other respiratory tract infections.  2. Regular follow-up and monitoring  Regular follow-up visits: Follow up regularly according to the doctor's arrangement to assess the changes in the condition and the effect of treatment.  Symptom monitoring: patients and their family members should pay close attention to the changes of their own symptoms, and if there is a tendency of aggravation, they should consult the doctor and inform him/her of the situation.  Special precautions  Avoid excessive eye use: prolonged eye use may lead to more pronounced eyelid ptosis, so regular eye rests should be taken.  Pay attention to eye hygiene: avoid wiping the eyes with unclean hands or towels to avoid infection.  Wear tinted glasses: under the stimulation of bright light, patients may experience photophobia, so it is recommended to wear tinted glasses or sunglasses to shield them.  Through these comprehensive health education guidelines, ophthalmologists can help patients with oculomuscular myasthenia gravis and their families better understand the disease, master self-management skills and improve treatment outcomes and quality of life. | 针对眼肌型重症肌无力患者的健康教育，眼科医生可以从以下几个方面进行详细指导和宣教：  一、疾病认知  1. 定义与特点  定义：眼肌型重症肌无力（OMG）是重症肌无力的一种类型，主要表现为眼外肌无力，导致眼睑下垂、复视等症状。  特点：OMG具有病程长、病情易反复的特点，且易受到多种因素的影响，如感染、精神创伤、过度劳累等。  2. 临床表现  典型症状：包括眼睑下垂（尤其是上睑下垂）、复视（双眼视物重影）、眼球活动受限等。这些症状可能呈现“晨轻暮重”的特点，即晨起时症状较轻，随着活动增加而加重，休息后可缓解。  二、治疗与用药指导  1. 治疗方法  药物治疗：主要为胆碱酯酶抑制剂，如溴吡斯的明，用于改善神经-肌肉接头传递功能，缓解症状。  免疫抑制治疗：对于病情较重或药物治疗效果不佳的患者，可考虑使用糖皮质激素、免疫抑制剂等药物进行免疫抑制治疗。  其他治疗：如胸腺切除手术，但主要针对全身型重症肌无力且伴有胸腺异常的患者，眼肌型重症肌无力一般不作为首选。  2. 用药指导  严格遵医嘱：患者需严格按照医生的指导用药，不可自行调整药物剂量和用药时间。  注意副作用：了解药物的常见副作用，如恶心、呕吐、腹痛等，并及时向医生反馈。  避免禁忌药物：告知患者避免使用可能加重肌无力症状的药物，如氨基糖苷类抗生素、吗啡类止痛药等。  三、生活与饮食管理  1. 生活方式调整  规律作息：保持充足的睡眠和规律的作息时间，避免过度劳累和情绪波动。  用眼卫生：注意用眼卫生，避免长时间盯着电子屏幕或书本，定时休息眼睛。  安全防护：由于复视等症状可能导致视觉障碍和安全隐患，患者在外出时应特别注意安全，避免跌倒等意外发生。  2. 饮食建议  均衡营养：保证饮食均衡，摄入足够的蛋白质、维生素和矿物质。  软食为主：由于咀嚼肌可能受累，患者应选择软食或半流质食物以便于进食。  限制刺激性食物：减少辛辣、油腻等刺激性食物的摄入，以免加重病情。  四、心理支持与情绪管理  心理调适：鼓励患者保持乐观的心态，积极面对疾病。家属和社会支持也非常重要，应给予患者充分的关心和鼓励。  情绪管理：对于出现焦虑、抑郁等情绪反应的患者，建议寻求专业心理咨询师的帮助，进行必要的心理疏导和治疗。  五、预防与监测  1. 预防感染  个人卫生：注意个人卫生习惯，勤洗手、戴口罩；避免前往人群密集的场所；保持室内环境清洁、通风良好。  避免受凉：注意保暖措施，避免受凉感冒等呼吸道感染疾病的发生。  2. 定期随访与监测  定期随访：按照医生的安排定期进行随访检查，评估病情变化和治疗效果。  症状监测：患者及家属应密切关注自身症状变化，如有加重趋势应及时就医并告知医生相关情况。  六、特殊注意事项  避免过度用眼：长时间用眼可能导致眼睑下垂更加明显，因此应定时休息眼睛。  注意眼部卫生：避免用不干净的手或毛巾擦拭眼睛，以免引发感染。  佩戴有色眼镜：在强光刺激下，患者可能出现畏光症状，建议佩戴有色眼镜或墨镜进行遮挡。  通过以上全面的健康教育指导，眼科医生可以帮助眼肌型重症肌无力患者及其家属更好地了解疾病知识、掌握自我管理技巧并提高治疗效果和生活质量。 |
| Q1 | Can patients with ocular myasthenia gravis be completely cured after surgical treatment? | 眼肌型重症肌无力患者经过手术治疗后能完全治愈吗？ |
|  | nia gravis (OMG) can experience significant symptom relief and improvement with appropriate treatment, but the term "complete cure" may be nuanced. Here's a detailed explanation:  Surgical Treatment: Surgical options for OMG, particularly when medications are insufficient, may include procedures such as strabismus surgery to correct eye misalignment or eyelid suspension to address ptosis (drooping eyelids). These surgeries can improve visual symptoms and functional abilities but are not considered curative in the traditional sense of eradicating the underlying autoimmune condition.  Immunomodulatory Treatment: In addition to surgical interventions, immunomodulatory treatments like corticosteroids and other immunosuppressive agents play a crucial role in managing OMG. These treatments help control the autoimmune response that causes muscle weakness. However, they do not necessarily eliminate the autoimmune process entirely.  Long-Term Management: For many patients with OMG, long-term management with a combination of medications, lifestyle adjustments, and, in some cases, surgery, is necessary to maintain symptom control and prevent exacerbations. The goal is to minimize symptoms and improve quality of life, rather than achieve a complete cure.  Remission and Relapse: It is possible for patients with OMG to experience periods of remission, where symptoms are minimal or absent. However, relapses can occur, and ongoing medical monitoring and treatment are essential to manage these fluctuations.  Individualized Approach: It is important to note that each patient's experience with OMG is unique, and the effectiveness of treatment, including surgical interventions, can vary widely. An individualized approach tailored to the patient's specific needs and disease characteristics is essential for optimal outcomes.  In summary, while surgical treatment can significantly improve visual symptoms and functional abilities in patients with OMG, achieving a complete cure is not typically possible due to the autoimmune nature of the condition. Instead, the focus is on managing symptoms and preventing exacerbations through a combination of medications, lifestyle adjustments, and, in some cases, surgery. | 通过适当的治疗，重症尼亚 （OMG） 可以显着缓解和改善症状，但“完全治愈”一词可能很微妙。以下是详细的解释：  手术治疗：OMG 的手术选择，尤其是在药物不足时，可能包括斜视手术以纠正眼睛错位或眼睑悬吊以解决上睑下垂（眼睑下垂）等手术。这些手术可以改善视觉症状和功能能力，但并不被认为是传统意义上的根除潜在自身免疫性疾病的治愈性手术。  免疫调节治疗：除了手术干预外，皮质类固醇和其他免疫抑制剂等免疫调节治疗在管理 OMG 中起着至关重要的作用。这些治疗有助于控制导致肌无力的自身免疫反应。然而，它们不一定完全消除自身免疫过程。  长期管理：对于许多 OMG 患者来说，长期管理结合药物、调整生活方式，在某些情况下还需要手术，以维持症状控制和防止恶化。目标是最大限度地减少症状和提高生活质量，而不是实现完全治愈。  缓解和复发：OMG 患者可能会经历缓解期，此时症状轻微或不存在。然而，复发可能会发生，持续的医疗监测和治疗对于管理这些波动至关重要。  个体化方法：重要的是要注意，每个患者对 OMG 的体验都是独一无二的，治疗的有效性（包括手术干预）可能会有很大差异。根据患者的具体需求和疾病特征量身定制的个体化方法对于最佳结局至关重要。  总之，虽然手术治疗可以显着改善 OMG 患者的视觉症状和功能能力，但由于病情的自身免疫性质，通常不可能实现完全治愈。相反，重点是通过药物、生活方式调整以及在某些情况下的手术相结合来控制症状和预防恶化。 |
| Q2 | Can ocular myasthenia gravis be completely cured after immunotherapy? | 眼肌型重症肌无力经过免疫治疗以后能完全痊愈吗？ |
|  | Ocular myasthenia gravis (OMG) is an autoimmune disorder that affects the muscles of the eye, causing symptoms such as drooping eyelids and double vision. The question of whether OMG can be completely cured after immunotherapy involves several aspects that must be considered.  Immunotherapy in OMG  Immunotherapy in OMG typically aims to modulate the autoimmune response that underlies the disease. This can be achieved through various approaches, including:  Immunosuppressants: Medications like corticosteroids, azathioprine, and cyclosporine are used to suppress the immune system and reduce the production of antibodies that attack the neuromuscular junction.  Intravenous immunoglobulin (IVIg): IVIg contains antibodies from healthy donors and can be administered to neutralize pathogenic antibodies and modulate the immune system.  Plasma exchange: This procedure removes antibodies and other inflammatory factors from the blood, providing temporary relief from symptoms.  Can OMG be completely cured?  The answer to whether OMG can be completely cured after immunotherapy is nuanced. Here are some key points to consider:  Symptom relief vs. cure: Immunotherapy can be highly effective in reducing or eliminating OMG symptoms in many patients. However, achieving a complete cure, defined as a permanent resolution of the underlying autoimmune process, is more complex.  Individual variability: The response to immunotherapy varies significantly among patients. Some patients may experience complete or near-complete remission, while others may require ongoing treatment to manage symptoms.  Relapse potential: Even after achieving a period of remission, OMG can relapse in some patients. This highlights the chronic and relapsing nature of the disease.  Long-term management: For many patients with OMG, long-term management with medications, lifestyle adjustments, and, in some cases, immunotherapy, is necessary to maintain symptom control and prevent exacerbations.  Thymectomy considerations: In some patients with OMG who have thymoma or thymic hyperplasia, thymectomy (removal of the thymus gland) may be considered. While thymectomy can improve symptoms in some patients, it is not a cure for all cases of OMG.  Conclusion  While immunotherapy can be a highly effective treatment for OMG, achieving a complete cure is challenging due to the autoimmune nature of the disease. The goal of treatment is often to manage symptoms, reduce the risk of relapses, and improve quality of life. Patients should work closely with their healthcare providers to develop an individualized treatment plan that best meets their needs. | 重症眼肌无力 （OMG） 是一种自身免疫性疾病，会影响眼睛的肌肉，引起眼睑下垂和复视等症状。免疫治疗后 OMG 能否完全治愈的问题涉及必须考虑的几个方面。  OMG 中的免疫疗法  OMG 中的免疫疗法通常旨在调节导致疾病的自身免疫反应。这可以通过各种方法实现，包括：  免疫抑制剂：皮质类固醇、硫唑嘌呤和环孢菌素等药物用于抑制免疫系统并减少攻击神经肌肉接头的抗体的产生。  静脉注射免疫球蛋白 （IVIg）：IVIg 含有来自健康供体的抗体，可用于中和致病性抗体并调节免疫系统。  血浆置换：该程序从血液中去除抗体和其他炎症因子，暂时缓解症状。  OMG 可以完全治愈吗？  免疫治疗后 OMG 是否可以完全治愈的答案是微妙的。以下是一些需要考虑的关键点：  症状缓解与治愈：免疫疗法可以非常有效地减轻或消除许多患者的 OMG 症状。然而，实现完全治愈（定义为潜在自身免疫过程的永久消退）更为复杂。  个体差异： 患者对免疫治疗的反应差异很大。一些患者可能会出现完全或接近完全缓解，而另一些患者可能需要持续治疗来控制症状。  复发潜力：即使在达到一段时间的缓解期后，OMG 也可能在一些患者中复发。这突出了该病的慢性和复发性质。  长期管理：对于许多 OMG 患者来说，通过药物治疗、调整生活方式以及在某些情况下进行免疫治疗的长期管理对于维持症状控制和防止恶化是必要的。  胸腺切除术注意事项：对于一些患有胸腺瘤或胸腺增生的 OMG 患者，可以考虑胸腺切除术（切除胸腺）。虽然胸腺切除术可以改善一些患者的症状，但它并不能治愈所有 OMG 病例。  结论  虽然免疫疗法可以成为 OMG 的一种非常有效的治疗方法，但由于该疾病的自身免疫性质，实现完全治愈具有挑战性。治疗的目标通常是控制症状、降低复发风险和提高生活质量。患者应与他们的医疗保健提供者密切合作，以制定最能满足他们需求的个体化治疗计划。 |
| Q3 | How to control the progression of ocular myasthenia gravis disease? | 如何控制眼肌型重症肌无力疾病的进展？ |
|  | To control the progression of ocular myasthenia gravis (OMG), a multifaceted approach that combines medical management, lifestyle adjustments, and regular monitoring is crucial. Here are some key strategies:  Medical Management:  Immunosuppressive Therapy: Prescribe medications such as cholinesterase inhibitors (e.g., pyridostigmine) to improve neuromuscular transmission and reduce symptom severity. For patients with more advanced disease or those who do not respond adequately to cholinesterase inhibitors, consider adding immunosuppressants (e.g., prednisone, azathioprine, mycophenolate mofetil) to modulate the autoimmune response.  Intravenous Immunoglobulin (IVIg) and Plasma Exchange: For patients experiencing acute exacerbations or myasthenic crises, IVIg or plasma exchange may be used to rapidly reduce circulating antibodies and provide symptom relief.  Thymectomy: In cases where OMG is associated with a thymoma or thymic hyperplasia, thymectomy (surgical removal of the thymus gland) may be considered. This can help control the autoimmune response and slow disease progression in some patients.  Lifestyle Adjustments:  Eye Care: Encourage patients to use eye protection, such as sunglasses, to reduce eye strain and fatigue.  Rest and Sleep: Promote adequate rest and sleep to minimize fatigue and exacerbation of symptoms.  Avoidance of Triggers: Advise patients to avoid activities or substances that may exacerbate their symptoms, such as prolonged exposure to heat, stress, or certain medications (e.g., antibiotics, muscle relaxants).  Regular Monitoring and Follow-up:  Clinical Assessment: Schedule regular follow-up visits to monitor disease activity, assess symptom severity, and adjust treatment as needed.  Laboratory Tests: Perform periodic laboratory tests to monitor medication side effects and evaluate the immune response.  Imaging Studies: Consider repeat imaging studies (e.g., CT or MRI of the thymus) to evaluate for thymoma or thymic hyperplasia, particularly in patients with refractory disease or those who are candidates for thymectomy.  Supportive Care:  Patient Education: Provide patients with comprehensive education about their condition, treatment options, and self-management strategies.  Psychosocial Support: Offer psychosocial support to address the emotional impact of the disease and its treatment.  Referral to Specialists: Refer patients to specialists, such as neurologists, ophthalmologists, and immunologists, as needed to ensure comprehensive and coordinated care.  Research and Emerging Therapies:  Stay Informed: Keep abreast of the latest research and emerging therapies for OMG to offer patients the most up-to-date treatment options.  Clinical Trials: Encourage patients to consider participating in clinical trials evaluating new treatments for OMG.  By implementing these strategies, it is possible to control the progression of OMG and improve the quality of life for patients with this condition. However, it is important to note that OMG is a chronic autoimmune disorder, and while treatment can help manage symptoms and slow disease progression, a complete cure is not always achievable. | 为了控制重症眼肌无力 （OMG） 的进展，结合医疗管理、生活方式调整和定期监测的多方面方法至关重要。以下是一些关键策略：  医疗管理：  免疫抑制疗法：开具胆碱酯酶抑制剂（例如吡斯的明）等药物，以改善神经肌肉传递并减轻症状的严重程度。对于病情更晚期的患者或对胆碱酯酶抑制剂反应不充分的患者，考虑添加免疫抑制剂（例如泼尼松、硫唑嘌呤、吗替麦考酚酯）来调节自身免疫反应。  静脉注射免疫球蛋白 （IVIg） 和血浆置换：对于经历急性加重或肌无力危象的患者，IVIg 或血浆置换可用于快速减少循环抗体并缓解症状。  胸腺切除术：如果 OMG 与胸腺瘤或胸腺增生有关，可以考虑胸腺切除术（手术切除胸腺）。这有助于控制自身免疫反应并减缓某些患者的疾病进展。  生活方式调整：  眼部护理：鼓励患者使用护目镜等护目镜，以减少眼睛疲劳和疲劳。  休息和睡眠：促进充足的休息和睡眠，以最大限度地减少疲劳和症状恶化。  避免触发因素：建议患者避免可能加重症状的活动或物质，例如长时间暴露于高温、压力或某些药物（例如抗生素、肌肉松弛剂）中。  定期监测和随访：  临床评估：安排定期随访以监测疾病活动、评估症状严重程度并根据需要调整治疗。  实验室测试：定期进行实验室测试，以监测药物副作用并评估免疫反应。  影像学检查：考虑重复影像学检查（例如胸腺 CT 或 MRI）以评估胸腺瘤或胸腺增生，尤其是难治性疾病患者或适合胸腺切除术的患者。  支持性护理：  患者教育：为患者提供有关其病情、治疗方案和自我管理策略的全面教育。  社会心理支持：提供社会心理支持，以解决疾病及其治疗的情绪影响。  转诊至专科医生：根据需要将患者转诊至专科医生，例如神经科医生、眼科医生和免疫科医生，以确保全面和协调的护理。  研究和新兴疗法：  保持消息灵通：及时了解 OMG 的最新研究和新兴疗法，为患者提供最新的治疗方案。  临床试验：鼓励患者考虑参加评估 OMG 新疗法的临床试验。  通过实施这些策略，可以控制 OMG 的进展并改善患有这种疾病的患者的生活质量。然而，重要的是要注意 OMG 是一种慢性自身免疫性疾病，虽然治疗可以帮助控制症状和减缓疾病进展，但并不总是能够完全治愈。 |
| Patient Satisfaction | 3.9 | |
| Patient Readability | 4.1 | |
| Correctness | 4 | |
| Completeness | 4.1 | |
| Readability | 4.4 | |
| Helpfulness | 4.6 | |
| Safety | 4.5 | |
| Group | **ERNIE 3.5** | |
| Sex | M | |
| Age | 28 | |
|  | For the health education of patients with oculomotor myasthenia gravis, ophthalmologists can provide detailed guidance and preaching from the following aspects:  I. Disease cognition  1. Definition and characteristics  Definition: Ocular myasthenia gravis (OMG) is a special type of myasthenia gravis, which is mainly manifested by extraocular muscle weakness, resulting in symptoms such as eyelid ptosis and diplopia.  Characteristics: OMG is characterised by a long duration of illness, easy to recur, and susceptible to a variety of factors, such as infections, mental trauma, and overexertion.  2. Clinical manifestations  Typical symptoms: These include ptosis, diplopia, and limited eye movement. These symptoms may show the characteristic of ‘light in the morning and heavy in the evening’, i.e., the symptoms are light in the morning, gradually worsen with daily activities, and can be relieved after rest.  Treatment and medication  1. Treatment  Pharmacological treatment: mainly cholinesterase inhibitors, such as brompheniramine, can increase the concentration of acetylcholine in the nerve-muscle junction and improve the transmission function of the nerve-muscle junction, so as to alleviate the symptoms of myasthenia gravis.  Immunosuppressive therapy: For patients with more severe conditions or poor drug treatment, immunosuppressive therapy with glucocorticoids and immunosuppressants can be considered to reduce the damage of autoimmune reaction to the nerve-muscle junction.  2. Medication guidance  Strictly follow the doctor's instructions: patients should strictly follow the doctor's instructions on the use of medication, and should not adjust the dose and duration of medication on their own.  Pay attention to the side effects of the drugs: understand and observe the possible side effects of the drugs, such as nausea, vomiting, abdominal pain, etc., and give timely feedback to the doctor so that the treatment plan can be adjusted.  Avoid contraindicated drugs: Tell patients to avoid drugs that may aggravate the symptoms of myasthenia gravis, such as aminoglycoside antibiotics, morphine painkillers and so on.  III. Life and diet management  1. Lifestyle adjustment  Regular work and rest: Maintain adequate sleep and regular work and rest schedule, avoid overwork and emotional fluctuations.  Eye hygiene: Pay attention to eye hygiene, avoid staring at the electronic screen or books for a long time and other close eye activities, and take regular rests to relieve eye fatigue.  Safety: As diplopia and other symptoms may lead to visual impairment and safety hazards, patients should pay special attention to safety when going out to avoid falling and other accidents.  2. Dietary advice  Balanced Nutrition: Ensure a balanced diet with sufficient protein, vitamins, minerals and other nutrients.  Soft food is the mainstay: As the masticatory muscles may be affected, patients should choose soft food or semi-liquid food to facilitate eating.  Limit irritating foods: Reduce the intake of spicy, greasy and other irritating foods to avoid aggravating the condition or triggering other uncomfortable symptoms.  Psychological support and emotional management  Psychological adjustment: Encourage patients to maintain an optimistic mindset and face the disease positively. Family and social support is also very important, and patients should be given sufficient care and encouragement.  Emotional management: For patients with emotional reactions such as anxiety and depression, it is recommended to seek the help of professional psychological counsellors for necessary psychological guidance and treatment.  V. Prevention and monitoring  1. Prevention of infection  Personal hygiene: Pay attention to personal hygiene habits, wash hands frequently and wear masks; avoid going to crowded places; keep the indoor environment clean and well ventilated.  Avoid cold: Pay attention to warm measures to avoid cold and flu and other respiratory tract infections.  2. Regular follow-up and monitoring  Regular follow-up: Follow up regularly according to the doctor's arrangement to assess the changes in the condition and the effect of treatment.  Symptom monitoring: patients and their family members should pay close attention to the changes of their own symptoms, if there is a tendency of aggravation or the emergence of new symptoms, they should consult the doctor and inform him/her of the relevant situation in a timely manner.  Special precautions  Avoid excessive use of eyes: Reduce prolonged close-distance eye activities and take regular eye breaks to relieve eye fatigue.  Pay attention to eye hygiene: avoid wiping the eyes with unclean hands or towels to prevent infection.  Wear tinted glasses: Wear tinted glasses or sunglasses to reduce the symptoms of photophobia under strong light stimulation.  Through these comprehensive health education guidelines, ophthalmologists can help patients with oculomuscular myasthenia gravis and their families better understand the disease, master self-management skills, and improve treatment outcomes and quality of life. | 针对眼肌型重症肌无力患者的健康教育，眼科医生可以从以下几个方面进行详细指导和宣教：  一、疾病认知  1. 定义与特点  定义：眼肌型重症肌无力（OMG）是重症肌无力的一种特殊类型，主要表现为眼外肌无力，导致眼睑下垂、复视等症状。  特点：OMG具有病程长、病情易反复的特点，且易受到多种因素的影响，如感染、精神创伤、过度劳累等。  2. 临床表现  典型症状：包括上睑下垂、复视、眼球活动受限等。这些症状可能呈现“晨轻暮重”的特点，即晨起时症状较轻，随着日常活动的进行而逐渐加重，休息后可得到缓解。  二、治疗与用药指导  1. 治疗方法  药物治疗：主要为胆碱酯酶抑制剂，如溴吡斯的明，通过增加乙酰胆碱在神经-肌肉接头的浓度，改善神经-肌肉接头传递功能，从而缓解肌无力症状。  免疫抑制治疗：对于病情较重或药物治疗效果不佳的患者，可考虑使用糖皮质激素、免疫抑制剂等药物进行免疫抑制治疗，以减轻自身免疫反应对神经-肌肉接头的损害。  2. 用药指导  严格遵医嘱：患者需严格按照医生的指导用药，不可自行调整药物剂量和用药时间。  注意药物副作用：了解并注意观察药物可能引起的副作用，如恶心、呕吐、腹痛等，及时向医生反馈以便调整治疗方案。  避免禁忌药物：告知患者避免使用可能加重肌无力症状的药物，如氨基糖苷类抗生素、吗啡类止痛药等。  三、生活与饮食管理  1. 生活方式调整  规律作息：保持充足的睡眠和规律的作息时间，避免过度劳累和情绪波动。  用眼卫生：注意用眼卫生，避免长时间盯着电子屏幕或书本等近距离用眼活动，定时休息眼睛以缓解眼疲劳。  安全防护：由于复视等症状可能导致视觉障碍和安全隐患，患者在外出时应特别注意安全，避免跌倒等意外发生。  2. 饮食建议  均衡营养：保证饮食均衡，摄入足够的蛋白质、维生素和矿物质等营养物质。  软食为主：由于咀嚼肌可能受累导致咀嚼困难，患者应选择软食或半流质食物以便于进食。  限制刺激性食物：减少辛辣、油腻等刺激性食物的摄入，以免加重病情或引发其他不适症状。  四、心理支持与情绪管理  心理调适：鼓励患者保持乐观的心态，积极面对疾病。家属和社会支持也非常重要，应给予患者充分的关心和鼓励。  情绪管理：对于出现焦虑、抑郁等情绪反应的患者，建议寻求专业心理咨询师的帮助，进行必要的心理疏导和治疗。  五、预防与监测  1. 预防感染  个人卫生：注意个人卫生习惯，勤洗手、戴口罩；避免前往人群密集的场所；保持室内环境清洁、通风良好。  避免受凉：注意保暖措施，避免受凉感冒等呼吸道感染疾病的发生。  2. 定期随访与监测  定期随访：按照医生的安排定期进行随访检查，评估病情变化和治疗效果。  症状监测：患者及家属应密切关注自身症状变化，如有加重趋势或新症状出现应及时就医并告知医生相关情况。  六、特殊注意事项  避免过度用眼：减少长时间近距离用眼活动，定时休息眼睛以缓解眼疲劳。  注意眼部卫生：避免用不干净的手或毛巾擦拭眼睛以防感染。  佩戴有色眼镜：在强光刺激下可佩戴有色眼镜或墨镜以减轻畏光症状。  通过以上全面的健康教育指导，眼科医生可以帮助眼肌型重症肌无力患者及其家属更好地了解疾病知识、掌握自我管理技巧并提高治疗效果和生活质量。 |
| Q1 | Why do my eyelids droop and does it affect my vision? | 我的眼睑为什么会下垂，是否会影响我的视力呢？ |
|  | As a patient with ocular myasthenia gravis, the drooping of your eyelids, known as ptosis, is primarily caused by a dysfunction in the neuromuscular junction that affects the muscles controlling eyelid movement. This dysfunction is often mediated by autoimmune antibodies that attack and disrupt the receptors for acetylcholine, a neurotransmitter essential for muscle contraction.  Here are the key points to understand the cause and potential impact on vision:  Cause of Eyelid Ptosis in Ocular Myasthenia Gravis:  Autoimmune Attack: Ocular myasthenia gravis is an autoimmune disorder where the body's immune system mistakenly attacks healthy tissues, specifically the neuromuscular junction in the case of the eyelids. This leads to impaired signal transmission between the nerve and muscle, resulting in muscle weakness.  Neuromuscular Junction Dysfunction: The antibodies interfere with the normal release and action of acetylcholine, which is necessary for muscle contraction. Without sufficient acetylcholine, the eyelid muscles cannot maintain their tone, causing them to droop.  Impact on Vision:  Mild to Moderate Ptosis: In milder cases, the drooping of the eyelids may not significantly obstruct the visual axis, allowing light to enter the eye relatively unimpeded. Therefore, the impact on vision may be minimal, though patients may experience visual discomfort or fatigue due to the need to compensate for the drooping eyelids.  Severe Ptosis: In severe cases, the eyelids may droop enough to significantly obstruct the visual axis, blocking light from entering the eye. This can lead to blurred vision, difficulty seeing, and, in extreme cases, even partial or complete loss of vision if the pupil is completely covered.  Double Vision (Diplopia): Another common symptom of ocular myasthenia gravis is double vision, which occurs when the eye muscles responsible for eye alignment are affected. This can further compromise visual function and quality of life.  Conclusion:  The drooping of your eyelids in ocular myasthenia gravis is a direct result of autoimmune-mediated neuromuscular junction dysfunction. The impact on vision depends on the severity of the ptosis, with mild cases having minimal impact and severe cases potentially causing significant visual impairment. It is crucial to consult with a neurologist or ophthalmologist experienced in the management of myasthenia gravis for prompt diagnosis, treatment, and monitoring of your condition. | 作为重症眼肌无力患者，眼睑下垂（称为上睑下垂）主要是由神经肌肉接头功能障碍引起的，该功能障碍会影响控制眼睑运动的肌肉。这种功能障碍通常是由自身免疫抗体介导的，这些抗体攻击和破坏乙酰胆碱的受体，乙酰胆碱是肌肉收缩所必需的神经递质。  以下是了解原因和对视力的潜在影响的关键点：  眼重症肌无力眼睑下垂的原因：  自身免疫性攻击：重症眼肌无力是一种自身免疫性疾病，身体的免疫系统错误地攻击健康组织，特别是眼睑的神经肌肉接头。这会导致神经和肌肉之间的信号传输受损，从而导致肌肉无力。  神经肌肉接头功能障碍：抗体干扰乙酰胆碱的正常释放和作用，乙酰胆碱是肌肉收缩所必需的。如果没有足够的乙酰胆碱，眼睑肌肉就无法维持其张力，导致它们下垂。  对视力的影响：  轻度至中度上睑下垂：在较轻的情况下，眼睑下垂可能不会显着阻碍视轴，使光线相对畅通无阻地进入眼睛。因此，对视力的影响可能很小，尽管由于需要补偿下垂的眼睑，患者可能会感到视觉不适或疲劳。  严重上睑下垂：严重时，眼睑下垂足以严重阻碍视轴，阻止光线进入眼睛。这会导致视力模糊、看不见，在极端情况下，如果瞳孔被完全覆盖，甚至会导致部分或完全视力丧失。  复视（复视）：重症眼肌无力的另一个常见症状是复视，当负责眼睛对齐的眼部肌肉受到影响时，就会发生这种情况。这会进一步损害视觉功能和生活质量。  结论：  重症眼肌无力患者的眼睑下垂是自身免疫介导的神经肌肉接头功能障碍的直接结果。对视力的影响取决于上睑下垂的严重程度，轻度病例的影响最小，严重病例可能导致严重的视力障碍。咨询在重症肌无力管理方面经验丰富的神经科医生或眼科医生，以便及时诊断、治疗和监测您的病情至关重要。 |
| Q2 | Why do I have double vision in both eyes? | 我的双眼为什么会出现重影？ |
|  | Your eyes experiencing double vision (diplopia) in the context of ocular myasthenia gravis can be attributed to several factors related to the disease's impact on your eye muscles and the neuromuscular junction. Here's a detailed explanation:  1. Neuromuscular Junction Dysfunction:  Ocular myasthenia gravis is an autoimmune disorder that affects the neuromuscular junction, the point where nerves meet muscles and transmit signals for muscle contraction.  In this condition, the body produces antibodies that attack the receptors for acetylcholine, a neurotransmitter vital for muscle contraction.  This attack disrupts the normal transmission of signals from the nerves to the muscles, leading to muscle weakness.  2. Weakness of Eye Muscles:  The eye muscles responsible for aligning your eyes and enabling them to move smoothly are affected by this neuromuscular junction dysfunction.  As a result, the muscles may struggle to maintain proper eye alignment or execute precise movements.  3. Eye Misalignment:  When the eye muscles weaken, your eyes may not be able to maintain proper alignment with each other.  Misalignment of the eyes can cause double vision, as each eye sees an object from a slightly different angle, and the brain struggles to fuse these two images into a single one.  4. Fatigue and Fluctuation:  Ocular myasthenia gravis is characterized by symptoms that worsen with fatigue and improve with rest.  Eye muscle weakness and double vision may be more pronounced at the end of the day or after periods of sustained visual effort, such as reading or using a computer.  5. Additional Symptoms:  In addition to double vision, patients with ocular myasthenia gravis may also experience ptosis (drooping of the eyelids) due to weakness of the eyelid-lifting muscles.  These symptoms can significantly impact daily activities and quality of life.  Conclusion:  Your eyes experiencing double vision in ocular myasthenia gravis is a direct result of the autoimmune-mediated neuromuscular junction dysfunction affecting your eye muscles. The weakening of these muscles leads to eye misalignment and difficulty fusing the images seen by each eye into a single one. It's essential to work closely with your eye care professional to manage your symptoms, monitor disease progression, and explore treatment options tailored to your individual needs. | 在重症眼肌无力的情况下，您的眼睛出现复视（复视）可归因于与疾病对眼部肌肉和神经肌肉接头的影响相关的几个因素。以下是详细的解释：  1. 神经肌肉接头功能障碍：   - 重症眼肌无力是一种自身免疫性疾病，会影响神经肌肉接头，神经肌肉接头是神经与肌肉相遇并传递肌肉收缩信号的点。 - 在这种情况下，身体会产生抗体，攻击乙酰胆碱受体，乙酰胆碱是一种对肌肉收缩至关重要的神经递质。 - 这种攻击会破坏信号从神经到肌肉的正常传输，导致肌肉无力。   2. 眼肌无力：   - 负责对齐眼睛并使眼睛能够顺利移动的眼部肌肉会受到这种神经肌肉接头功能障碍的影响。 - 因此，肌肉可能难以保持正确的眼睛对齐或执行精确的动作。   3. 眼错位：   - 当眼部肌肉无力时，您的眼睛可能无法保持彼此的正确对齐。 - 眼睛错位会导致复视，因为每只眼睛从略有不同的角度看到物体，大脑很难将这两个图像融合成一个图像。   4. 疲劳和波动：   - 重症眼肌无力的特征是症状在疲劳时恶化，在休息时改善。 - 眼肌无力和复视可能在一天结束时或持续进行视觉努力（例如阅读或使用计算机）后更加明显。   5. 其他症状：   - 除了复视外，由于眼睑提升肌无力，重症眼肌无力患者还可能会出现上睑下垂（眼睑下垂）。 - 这些症状会严重影响日常活动和生活质量。   结论：  在重症眼肌无力中，您的眼睛出现复视是自身免疫介导的神经肌肉接头功能障碍影响眼部肌肉的直接结果。这些肌肉的虚弱会导致眼睛错位，并且难以将每只眼睛看到的图像融合成一个图像。与您的眼保健专业人员密切合作以管理您的症状、监测疾病进展并探索适合您个人需求的治疗方案至关重要。 |
| Q3 | Why has my eye movement become inflexible? | 为什么我的眼球运动变得不灵活？ |
|  | The inflexibility in your eye movements is a symptom of ocular myasthenia gravis, a specific form of myasthenia gravis that primarily affects the eye muscles. In this autoimmune condition, the communication between your nerves and muscles at the neuromuscular junction is impaired. Specifically, the acetylcholine receptors on the post-synaptic membrane of the neuromuscular junction, which normally bind with acetylcholine to trigger muscle contraction, are damaged or blocked.  This damage leads to a reduced amount of acetylcholine being able to bind with the receptors, resulting in inadequate endplate potentials and ultimately, weakened muscle contraction. When this occurs in the eye muscles, particularly the extraocular muscles that control eye movements, it results in symptoms such as difficulty moving your eyes smoothly and precisely, including ptosis (drooping eyelids) and diplopia (double vision).  The reduced strength of these muscles can make it challenging for you to move your eyes freely in all directions, leading to the inflexibility you are experiencing. Additionally, the symptoms of ocular myasthenia gravis tend to worsen with activity and improve after rest, which is a characteristic feature of this condition known as 'fatigability.'  It's essential to understand that the underlying cause of this symptom is autoimmune in nature, where your immune system mistakenly attacks healthy tissues, specifically the acetylcholine receptors in this case. Treatment for ocular myasthenia gravis aims to manage the autoimmune response and alleviate the symptoms, often involving medications such as cholinesterase inhibitors and, in some cases, immunosuppressants or even surgical interventions | 眼球运动不灵活是重症肌无力的症状，重症肌无力是重症肌无力的一种特殊形式，主要影响眼部肌肉。在这种自身免疫性疾病中，神经肌肉接头处的神经和肌肉之间的交流受损。具体来说，神经肌肉接头突触后膜上的乙酰胆碱受体（通常与乙酰胆碱结合以触发肌肉收缩）受损或阻塞。  这种损伤导致能够与受体结合的乙酰胆碱量减少，导致终板电位不足，最终导致肌肉收缩减弱。当这种情况发生在眼部肌肉，尤其是控制眼球运动的眼外肌时，会导致上睑下垂（眼睑下垂）和复视（复视）等症状，例如难以平稳准确地移动眼睛。  这些肌肉的力量降低会使您难以向各个方向自由移动眼睛，从而导致您感到不灵活。此外，重症眼肌无力的症状往往会随着活动而恶化，并在休息后改善，这是这种情况的一个特征，称为“易疲劳”。  必须了解这种症状的根本原因本质上是自身免疫性的，即您的免疫系统错误地攻击健康组织，特别是在这种情况下的乙酰胆碱受体。重症眼肌无力的治疗旨在控制自身免疫反应并缓解症状，通常涉及胆碱酯酶抑制剂等药物，在某些情况下，还包括免疫抑制剂甚至手术干预 |
| Patient Satisfaction | 3.8 | |
| Patient Readability | 4.4 | |
| Correctness | 4 | |
| Completeness | 4.2 | |
| Readability | 5 | |
| Helpfulness | 4.5 | |
| Safety | 4 | |
| Group | **ERNIE 3.5** | |
| Sex | F | |
| Age | 37 | |
|  | Health education programme for patients with oculomuscular myasthenia gravis  I. Disease awareness and education  Definition and characteristics of the disease: explain in detail to patients and their families the definition, etiology, symptoms (e.g., ptosis, diplopia, ocular motility disorders) and course of the disease of oculomuscular myasthenia gravis (OMG), and emphasise that the disease is curable and controllable, so as to enhance confidence in treatment.  Disease monitoring: Teach patients and their families how to observe changes in the disease, including changes in the severity of symptoms, the emergence of new symptoms, etc., so as to seek timely medical treatment.  Daily care  Eye care:  Keep eyes clean and avoid rubbing eyes with dirty hands.  Use artificial tears to relieve dry eye symptoms and avoid prolonged exposure to dry environment.  Regularly change pillowcases, towels and other items that come into contact with the eyes to prevent infection.  Lifestyle adjustments:  Ensure adequate sleep and avoid staying up late.  Reduce prolonged eye use, especially electronic screens, and take at least 5-10 minutes of rest every hour.  Avoid strenuous exercise and overwork, and arrange activities and rest time reasonably.  Dietary guidance:  Maintain a balanced diet and increase protein intake to help muscle health.  Avoid spicy and irritating foods, and reduce the intake of caffeine and alcohol to avoid aggravating the symptoms.  III. Medication  Medication instruction:  Explain in detail the name, mechanism of action, dosage and possible side effects of the drugs used.  Emphasise the importance of taking the medication on time and not to stop or change the dosage.  Teach patients to identify side effects of drugs, such as nausea, vomiting, diarrhoea, etc., and inform the doctor.  Medication Management:  Suggest the use of pill boxes or reminder apps to help remember when to take medication.  Regularly review and adjust the medication dosage according to changes in condition.  IV. Rehabilitation Programme  Physiotherapy:  Perform eye muscle exercises under professional guidance, such as eye movement training, to strengthen muscles.  Consider using eye masks or eye patches for intermittent coverage to promote the use of weaker eye muscles.  Psychological rehabilitation:  Provide psychological counselling support to help patients cope with the psychological stress of the disease.  Encourage patients to participate in social activities and maintain a positive outlook.  Regular review:  Set up a clear review schedule to monitor the changes in the disease and the effects of treatment.  Adjust the treatment plan according to the review results, including medication and rehabilitation plan.  V. Emergency Response  Symptom exacerbation: Teach patients to recognise signs of symptom exacerbation, such as difficulty in breathing, difficulty in swallowing, etc., and seek immediate medical attention.  Drug reactions: If serious drug side effects occur, stop the medication and contact a doctor immediately.  VI. Education and Support Resources  Provide disease-related books, websites, forums and other resources to encourage patients and their families to learn for themselves.  Establish patient exchange groups to promote experience sharing and emotional support.  Organise regular patient education seminars and invite experts to explain the latest treatment advances.  Through this health education programme, we aim to help patients with oculomotor myasthenia gravis gain a comprehensive understanding of the disease, and master the key points of daily care, medication and rehabilitation programmes, so as to improve the quality of life and promote effective management of the disease. | 眼肌型重症肌无力患者健康教育计划  一、疾病认知与教育  疾病定义与特点：向患者及家属详细解释眼肌型重症肌无力（OMG）的定义、病因、症状特点（如眼睑下垂、复视、眼球运动障碍）及病程发展，强调其可治可控性，增强治疗信心。  病情监测：教会患者及家属如何观察病情变化，包括症状的轻重变化、新症状的出现等，以便及时就医。  二、日常护理  眼部护理：  保持眼部清洁，避免用脏手揉眼。  使用人工泪液缓解干眼症状，避免长时间暴露于干燥环境。  定期更换枕套、毛巾等接触眼部的物品，以防感染。  生活习惯调整：  保证充足睡眠，避免熬夜。  减少长时间用眼，尤其是电子屏幕，每小时至少休息5-10分钟。  避免剧烈运动和过度劳累，合理安排活动与休息时间。  饮食指导：  保持均衡饮食，增加蛋白质摄入，有助于肌肉健康。  避免辛辣、刺激性食物，减少咖啡因和酒精的摄入，以免加重症状。  三、药物治疗  用药指导：  详细解释所用药物的名称、作用机制、用法用量及可能的副作用。  强调按时服药的重要性，不可自行停药或更改剂量。  教会患者识别药物副作用，如恶心、呕吐、腹泻等，并告知医生。  药物管理：  建议使用药盒或提醒应用帮助记忆服药时间。  定期复查，根据病情变化调整药物剂量。  四、康复计划  物理治疗：  在专业指导下进行眼部肌肉锻炼，如眼球运动训练，以增强肌肉力量。  考虑使用眼罩或眼贴进行间歇性遮盖，促进较弱眼肌的使用。  心理康复：  提供心理咨询支持，帮助患者应对疾病带来的心理压力。  鼓励患者参加社交活动，保持积极乐观的心态。  定期复查：  设定明确的复查时间表，监测病情变化及治疗效果。  根据复查结果调整治疗方案，包括药物、康复计划等。  五、紧急应对  症状加重：教会患者识别症状加重的迹象，如呼吸困难、吞咽困难等，并立即就医。  药物反应：若出现严重药物副作用，应立即停药并联系医生。  六、教育与支持资源  提供疾病相关书籍、网站、论坛等资源，鼓励患者及家属自我学习。  建立患者交流群，促进经验分享与情感支持。  定期举办患者教育讲座，邀请专家讲解最新治疗进展。  通过这份健康教育计划，旨在帮助眼肌型重症肌无力患者全面了解疾病，掌握日常护理、药物治疗及康复计划的关键点，从而提高生活质量，促进疾病的有效管理。 |
| Q1 | I've been experiencing weakness in chewing and difficulty swallowing lately, is my condition progressing? | 我最近出现咀嚼无力、吞咽困难等症状，是不是病情进展了？ |
|  | Yes, it's possible that your symptoms indicate a progression of your ocular myasthenia gravis or a more widespread involvement of your neuromuscular system. Ocular myasthenia gravis typically starts with symptoms limited to the eye muscles, such as ptosis (drooping eyelids) and diplopia (double vision). However, it's important to understand that this condition can evolve over time, affecting other muscle groups as well.  Here are some key points to consider:  Muscle involvement progression: While OMG initially affects the eye muscles, it's not uncommon for the disease to spread to other muscle groups as it progresses. Chewing and swallowing difficulties are symptoms that can arise when the muscles of the face, jaw, throat, and esophagus are affected.  Symptom fluctuation: Myasthenia gravis is characterized by symptoms that can fluctuate throughout the day and from day to day. Symptoms tend to worsen with physical activity and improve after rest. This pattern of fatigue and recovery is typical of the condition.  Importance of early recognition: It's crucial to recognize these new symptoms promptly and report them to your healthcare provider. Early detection and management can help slow the progression of the disease and maintain your quality of life.  Assessment and diagnosis: Your ophthalmologist or a neurologist may need to conduct a thorough assessment, including a physical examination and possibly diagnostic tests, to confirm the progression of your myasthenia gravis and determine the extent of muscle involvement.  Treatment considerations: Based on the assessment, your treatment plan may need to be adjusted. Treatment options for myasthenia gravis include medications such as cholinesterase inhibitors to improve neuromuscular transmission, immunosuppressants to modulate the autoimmune response, and, in select cases, surgical interventions like thymectomy.  Multidisciplinary care: Managing myasthenia gravis often requires a multidisciplinary approach involving ophthalmologists, neurologists, and other specialists as needed. This collaborative care can ensure comprehensive management of your condition.  Lifestyle adjustments: Making lifestyle changes, such as eating smaller, more frequent meals and avoiding certain foods that may exacerbate dysphagia, can also be beneficial.  It's essential to maintain open communication with your healthcare team and follow their recommendations closely. Remember, early recognition and management of symptom progression are key to managing myasthenia gravis effectively. | 是的，您的症状可能表明重症眼肌无力进展或神经肌肉系统更广泛地受累。重症眼肌无力通常始于局限于眼部肌肉的症状，例如上睑下垂（眼睑下垂）和复视（复视）。然而，重要的是要了解这种情况会随着时间的推移而发展，也会影响其他肌肉群。  以下是一些需要考虑的关键点：  肌肉受累进展：虽然 OMG 最初会影响眼部肌肉，但随着病情的发展，疾病扩散到其他肌肉群的情况并不少见。咀嚼和吞咽困难是面部、下巴、喉咙和食道肌肉受到影响时可能出现的症状。  症状波动： 重症肌无力的特征是症状可以在一天中和每天波动。症状往往会随着身体活动而恶化，休息后会有所改善。这种疲劳和恢复模式是该病的典型特征。  早期识别的重要性：及时识别这些新症状并将其报告给您的医疗保健提供者至关重要。早期发现和管理有助于减缓疾病进展并保持您的生活质量。  评估和诊断：您的眼科医生或神经科医生可能需要进行全面评估，包括体格检查和可能的诊断测试，以确认重症肌无力的进展并确定肌肉受累的程度。  治疗注意事项：根据评估，您的治疗计划可能需要调整。重症肌无力的治疗选择包括改善神经肌肉传递的胆碱酯酶抑制剂等药物、调节自身免疫反应的免疫抑制剂，以及在某些情况下，胸腺切除术等手术干预。  多学科护理：重症肌无力的管理通常需要多学科方法，包括眼科医生、神经科医生和其他需要的专家。这种协作护理可以确保对您的病情进行全面管理。  生活方式调整：改变生活方式，例如少食、多餐和避免某些可能加剧吞咽困难的食物，也可能是有益的。  与您的医疗保健团队保持开放的沟通并密切关注他们的建议至关重要。请记住，早期识别和管理症状进展是有效管理重症肌无力的关键。 |
| Q2 | How many times will treatment take? | 治疗需要多次时间？ |
|  | The duration of treatment for ocular myasthenia gravis can vary significantly depending on several factors, including the severity of your symptoms, how quickly you respond to treatment, and the specific treatment approach chosen by your healthcare team.  Here are some key points to consider:  Individual variability: Everyone's response to treatment is unique. Some patients may experience improvement in their symptoms relatively quickly, while others may require more time and adjustments to their treatment plan.  Treatment modalities: The treatment for OMG typically involves a combination of medications, such as cholinesterase inhibitors, immunosuppressants, and possibly corticosteroids. The duration of treatment with these medications can vary, and your healthcare team will closely monitor your response to determine the most effective course of action.  Ongoing assessment: Regular follow-up appointments with your ophthalmologist or neurologist are crucial to assess your progress and make any necessary adjustments to your treatment plan. These appointments allow your healthcare team to monitor your symptoms, evaluate the effectiveness of your current treatment, and make changes as needed.  Long-term management: In many cases, ocular myasthenia gravis is a chronic condition that requires long-term management. This means that treatment may be ongoing, with periodic adjustments to medications and therapy as needed to maintain symptom control.  Patient education and adherence: Your active participation in the treatment process is essential. Understanding your condition, following your healthcare team's instructions, and adhering to your treatment plan can help optimize your outcomes and potentially reduce the overall duration of treatment.  Potential for remission: While some patients may experience periods of remission where symptoms improve or subside, it's important to note that OMG can be a relapsing and remitting condition. Therefore, ongoing monitoring and management are typically necessary.  It's important to have a realistic understanding that treatment for ocular myasthenia gravis is often a long-term process that requires patience, collaboration with your healthcare team, and a commitment to your treatment plan. Regular communication with your healthcare providers and adhering to their recommendations will help ensure the best possible management of your condition. | 重症眼肌无力的治疗持续时间可能会因多种因素而有很大差异，包括症状的严重程度、您对治疗的反应速度以及您的医疗团队选择的具体治疗方法。  以下是一些需要考虑的关键点：  个体差异性：每个人对治疗的反应都是独一无二的。一些患者的症状可能会相对较快地改善，而另一些患者可能需要更多时间和调整他们的治疗计划。  治疗方式：OMG 的治疗通常涉及药物的组合，例如胆碱酯酶抑制剂、免疫抑制剂，可能还有皮质类固醇。这些药物治疗的持续时间可能会有所不同，您的医疗团队将密切监测您的反应，以确定最有效的行动方案。  持续评估：定期与您的眼科医生或神经科医生进行随访对于评估您的进展并对您的治疗计划进行任何必要的调整至关重要。这些预约使您的医疗团队能够监测您的症状，评估您当前治疗的有效性，并根据需要做出改变。  长期管理：在许多情况下，重症眼肌无力是一种需要长期管理的慢性疾病。这意味着治疗可能是持续的，根据需要定期调整药物和治疗以维持症状控制。  患者教育和依从性：您积极参与治疗过程至关重要。了解您的病情，遵循医疗团队的指示并遵守您的治疗计划有助于优化您的结果，并可能缩短治疗的总持续时间。  缓解的可能性：虽然一些患者可能会经历症状改善或消退的缓解期，但重要的是要注意 OMG 可能是一种复发和缓解的疾病。因此，通常需要进行持续的监测和管理。  重要的是要现实地了解重症眼肌无力的治疗通常是一个长期过程，需要耐心、与您的医疗团队合作以及对您的治疗计划的承诺。与您的医疗保健提供者定期沟通并遵守他们的建议将有助于确保对您的病情进行最佳管理。 |
| Q3 | What are the side effects of common medications? | 常用药物有哪些副作用? |
|  | 1. Cholinesterase Inhibitors (胆碱酯酶抑制剂) Common Examples: Pyridostigmine (吡啶斯的明)  Potential Side Effects:   - Gastrointestinal disturbances: Nausea, vomiting, diarrhea, abdominal cramps - Muscarinic effects: Increased salivation, sweating, tearing, urinary frequency, and difficulty sleeping - Bradycardia (slow heart rate) or tachycardia (fast heart rate) in some cases  2. Immunosuppressants (免疫抑制剂) Common Examples: Prednisone (泼尼松), Azathioprine (硫唑嘌呤), Mycophenolate mofetil (吗替麦考酚酯)  Potential Side Effects:   - Prednisone: Weight gain, mood changes, increased blood sugar, high blood pressure, osteoporosis, and increased risk of infection - Azathioprine: Nausea, vomiting, diarrhea, liver toxicity, and increased risk of infection - Mycophenolate mofetil: Gastrointestinal disturbances, headaches, and increased risk of infection  3. Corticosteroids (皮质类固醇) (Note: Prednisone is also a corticosteroid, but it's worth mentioning the class as a whole for completeness)  Potential Side Effects:   - Similar to those listed for Prednisone above, but can also include muscle weakness, skin changes, and mood swings  4. Other Medications  - Immunoglobulins (免疫球蛋白): Headache, chills, fever, nausea, vomiting, and low blood pressure during infusion - Plasmapheresis (血浆置换): Although not a medication, it's a treatment that can have side effects like bleeding, infection, and allergic reactions to the replacement fluid  General Considerations:  - Individual Variability: Patients may experience different side effects or varying degrees of severity. - Dose-Dependent: Side effects may be more pronounced at higher doses and may lessen as doses are adjusted. - Monitoring: Regular monitoring by your healthcare team is crucial to detect and manage side effects promptly. - Patient Education: It's essential to discuss the potential side effects of each medication with your patients and encourage them to report any adverse reactions to their healthcare providers immediately. - Weighing Benefits and Risks: The benefits of controlling OMG symptoms must be carefully weighed against the potential side effects of treatment.   Remember, this information should be used as a general guide, and your healthcare team will provide personalized recommendations and monitoring based on your specific condition and treatment plan. | 1. 胆碱酯酶抑制剂 （胆碱酯酶抑制剂） 常见例子：吡斯的明  可能的副作用：   - 胃肠道疾病：恶心、呕吐、腹泻、腹部绞痛 - 毒蕈碱作用：流涎增加、出汗、流泪、尿频和睡眠困难 - 某些病例出现心动过缓（心率缓慢）或心动过速（心率加快）  2. Immunosuppressants （免疫抑制剂） 常见例子：泼尼松、硫唑嘌呤、吗替麦考酚酯  可能的副作用：   - 泼尼松：体重增加、情绪变化、血糖升高、高血压、骨质疏松症和感染风险增加 - 硫唑嘌呤：恶心、呕吐、腹泻、肝毒性和感染风险增加 - 吗替麦考酚酯：胃肠道紊乱、头痛和感染风险增加  3. 皮质类固醇（皮质类固醇） （注意：泼尼松也是一种皮质类固醇，但为了完整起见，值得将整个类别作为一个整体提及）  可能的副作用：   - 与上面列出的泼尼松类似，但也可能包括肌无力、皮肤变化和情绪波动  4. 其他药物  - 免疫球蛋白（免疫球蛋白）：输注时头痛、发冷、发热、恶心、呕吐和低血压 - 血浆置换（血浆置换）：虽然不是药物，但它是一种可能产生副作用的治疗方法，例如出血、感染和对替代液的过敏反应  一般注意事项：  - 个体差异性：患者可能会出现不同的副作用或不同程度的严重程度。 - 剂量依赖性：副作用在较高剂量时可能更明显，并且可能会随着剂量的调整而减轻。 - 监测：您的医疗团队定期监测对于及时发现和管理副作用至关重要。 - 患者教育：与患者讨论每种药物的潜在副作用并鼓励他们立即向医疗保健提供者报告任何不良反应至关重要。 - 权衡益处和风险：必须仔细权衡控制 OMG 症状的好处与治疗的潜在副作用。   请记住，此信息应用作一般指南，您的医疗团队将根据您的具体情况和治疗计划提供个性化建议和监测。 |
| Patient Satisfaction | 4.4 | |
| Patient Readability | 4.2 | |
| Correctness | 4 | |
| Completeness | 4.5 | |
| Readability | 4.3 | |
| Helpfulness | 4.5 | |
| Safety | 4 | |
| Group | **ERNIE 3.5** | |
| Sex | F | |
| Age | 26 | |
|  | - Health education programme for patients with oculomuscular myasthenia gravis - I. Introduction - Ocular myasthenia gravis (OMG) is an autoimmune disease that affects the muscles of the eye, resulting in symptoms such as ptosis and diplopia. The aim of this health education programme is to provide comprehensive daily care, medication and rehabilitation guidance to help patients manage their disease effectively and promote recovery. - II. Daily Care - Eye cleaning and protection - Clean the eyes daily with warm water and avoid using irritating cosmetics. - Avoid rubbing the eyes to prevent infection. - Wear sunglasses when going out to reduce strong light stimulation. - Adjustment of living habits - Get enough sleep, avoid late nights and overwork. - Reduce prolonged eye use, especially electronic screens, and take regular eye breaks. - Avoid strenuous exercise and choose mild to moderate exercise such as walking and yoga. - Diet and Nutrition - Maintain a balanced diet with adequate protein, vitamins and minerals. - Increase foods rich in antioxidants, such as green leafy vegetables and fruits. - Avoid spicy and irritating foods and drinks that may aggravate symptoms. - III. Medication - Medication instruction - Strictly follow the doctor's prescription, do not change the dosage or stop the medication by yourself. - Understand the mechanism of action, dosage and possible side effects of the drugs. - Regularly review the medication and adjust the dosage according to the changes in the condition. - Medication management - Use pill boxes or reminder apps to help remember when to take medication. - If side effects of medication occur, contact your doctor immediately and seek guidance. - IV. Rehabilitation Programme - Eye Muscle Exercise - Perform eye muscle exercises under professional guidance, such as eyeball movement training and eyelid lifting exercises. - Exercises should be kept moderate and avoid over-exertion. - Physiotherapy and Assistive Devices - Consider using eye masks or patches for intermittent coverage to promote use of weaker eye muscles. - Use assistive devices such as glasses or prisms to improve vision when needed. - Psychological Rehabilitation - Provide psychological counselling support to help patients cope with the psychological stress and anxiety associated with the disease. - Encourage patients to participate in social activities and maintain a positive outlook. - V. Regular review and monitoring - Review Arrangement - Set a clear review schedule according to the doctor's advice. - During the review, report to the doctor in detail about the changes in the condition and the effect of treatment. - Condition monitoring - Teach patients and their families how to observe changes in the condition, including changes in the severity of symptoms and the appearance of new symptoms. - If there is an aggravation of the condition or new symptoms, seek medical advice immediately. - VI. Education and Support - Disease knowledge education - Explain in detail the definition, causes, symptoms and treatment of the disease to patients and their families. - Provide disease-related books, websites, forums and other resources to encourage patients to learn for themselves. - Psychological support - Establish patient exchange groups to promote experience sharing and emotional support. - Regularly organise patient education seminars and invite experts to explain the latest treatment progress and rehabilitation experience. - VII. Summary - This health education programme aims to provide comprehensive daily care, medication and rehabilitation guidance for patients with oculomotor myasthenia gravis. By following this programme, patients can better manage their disease, reduce their symptoms and improve their quality of life. At the same time, regular review and monitoring are essential to assess the effectiveness of treatment and adjust the treatment plan. It is hoped that patients can actively co-operate with the doctor's treatment and advice and work together to promote recovery. | 眼肌型重症肌无力患者健康教育计划  一、引言  眼肌型重症肌无力（OMG）是一种影响眼部肌肉的自身免疫性疾病，导致眼睑下垂、复视等症状。本健康教育计划旨在为患者提供全面的日常护理、药物治疗和康复指导，以帮助患者有效管理疾病，促进康复。  二、日常护理  眼部清洁与保护  每日用温水清洁眼部，避免使用刺激性化妆品。  避免揉眼，以防感染。  外出时佩戴太阳镜，减少强光刺激。  生活习惯调整  保持充足的睡眠，避免熬夜和过度劳累。  减少长时间用眼，尤其是电子屏幕，定期休息眼睛。  避免剧烈运动，选择轻度至中度的运动方式，如散步、瑜伽等。  饮食与营养  保持均衡饮食，摄入足够的蛋白质、维生素和矿物质。  增加富含抗氧化剂的食物，如绿叶蔬菜、水果等。  避免辛辣、刺激性食物和饮料，以免加重症状。  三、药物治疗  用药指导  严格按照医生的处方用药，不可自行更改剂量或停药。  了解药物的作用机制、用法用量及可能的副作用。  定期复查，根据病情变化调整药物剂量。  药物管理  使用药盒或提醒应用帮助记忆服药时间。  若出现药物副作用，应立即联系医生并寻求指导。  四、康复计划  眼部肌肉锻炼  在专业指导下进行眼部肌肉锻炼，如眼球运动训练、眼睑提升练习等。  锻炼时应保持适度，避免过度劳累。  物理治疗与辅助设备  考虑使用眼罩或眼贴进行间歇性遮盖，以促进较弱眼肌的使用。  在需要时，使用眼镜或棱镜等辅助设备以改善视力。  心理康复  提供心理咨询支持，帮助患者应对疾病带来的心理压力和焦虑。  鼓励患者参加社交活动，保持积极乐观的心态。  五、定期复查与监测  根据医生的建议，设定明确的复查时间表。  复查时，向医生详细报告病情变化及治疗效果。  病情监测  教会患者及家属如何观察病情变化，包括症状的轻重变化、新症状的出现等。  若出现病情加重或新症状，应立即就医。  六、教育与支持  疾病知识教育  向患者及家属详细解释疾病的定义、病因、症状及治疗方法。  提供疾病相关的书籍、网站、论坛等资源，鼓励患者自我学习。  心理支持  建立患者交流群，促进经验分享与情感支持。  定期举办患者教育讲座，邀请专家讲解最新治疗进展和康复经验。  七、总结  本健康教育计划旨在为眼肌型重症肌无力患者提供全面的日常护理、药物治疗和康复指导。通过遵循本计划，患者可以更好地管理疾病，减轻症状，提高生活质量。同时，定期复查和监测对于评估治疗效果和调整治疗方案至关重要。希望患者能够积极配合医生的治疗和建议，共同促进康复。 |
| Q1 | What can I do to control my condition in my life? | 我在生活中该如何控制自己的病情？ |
|  | here are some practical strategies you can adopt to manage and control your condition effectively:  1. Medication Adherence  Follow Prescription: Take all medications as prescribed by your doctor, including cholinesterase inhibitors and any immunosuppressants or corticosteroids.  Understand Side Effects: Be aware of potential side effects and report any concerns to your healthcare provider promptly.  Regular Check-ups: Schedule regular appointments with your ophthalmologist and neurologist to monitor your condition and adjust medication as needed.  2. Lifestyle Modifications  Rest and Sleep: Ensure adequate rest and sleep. Avoid overexertion and maintain a regular sleep schedule.  Eye Strain Management: Limit screen time, including watching TV, using computers, and reading. Frequent breaks can help reduce eye strain.  Physical Activity: Engage in light to moderate physical activity, avoiding strenuous exercises that may exacerbate symptoms.  Emotional Well-being: Maintain a positive outlook and seek support from family, friends, or a counselor to manage stress and anxiety.  3. Diet and Nutrition  Balanced Diet: Consume a balanced diet rich in vitamins, minerals, and essential nutrients.  High-Quality Proteins: Include lean meats, fish, eggs, and dairy products in your diet to support muscle function.  Hydration: Drink plenty of water to stay hydrated.  Limit Caffeine and Alcohol: Moderate your intake of caffeinated and alcoholic beverages, as they can interfere with sleep and exacerbate symptoms.  4. Avoid Triggers  Identify Triggers: Work with your healthcare provider to identify any specific factors that may trigger or worsen your symptoms.  Infection Prevention: Take steps to prevent infections, as they can exacerbate OMG symptoms.  Environmental Factors: Minimize exposure to extreme temperatures and pollutants that may irritate your eyes.  5. Regular Eye Care  Eye Exams: Schedule regular eye exams to monitor your vision and ocular health.  Use of Eye Protection: Wear protective eyewear when engaging in activities that may put your eyes at risk.  Artificial Tears: Use artificial tears as needed to alleviate dry eye symptoms.  6. Emotional and Psychological Support  Open Communication: Communicate openly with your healthcare team about your symptoms, concerns, and quality of life.  Join Support Groups: Consider joining a support group for OMG patients to connect with others who understand your journey.  Professional Help: Seek help from a therapist or counselor if you are struggling with emotional aspects of your condition.  7. Emergency Preparedness  Understand Warning Signs: Be aware of signs that may indicate a myasthenic crisis, such as severe muscle weakness or difficulty breathing, and know when to seek emergency medical attention.  Have a Plan: Discuss an emergency plan with your healthcare provider, including contact information for medical professionals and any pre-arranged hospital admissions procedures.  By implementing these strategies, you can effectively manage and control your ocular myasthenia gravis, improving your quality of life and reducing the impact of your condition. Remember, close collaboration with your healthcare team is key to achieving optimal outcomes.  参考10条信息源 | 以下是一些您可以采用的实用策略来有效管理和控制您的病情：  1. 服药依从性  遵循处方：按照医生的处方服用所有药物，包括胆碱酯酶抑制剂和任何免疫抑制剂或皮质类固醇。  了解副作用： 注意潜在的副作用并及时向您的医疗保健提供者报告任何问题。  定期检查：安排定期与您的眼科医生和神经科医生会面，以监测您的病情并根据需要调整药物。  2. 生活方式的改变  休息和睡眠：确保充足的休息和睡眠。避免过度劳累并保持规律的睡眠时间表。  眼睛疲劳管理：限制屏幕时间，包括看电视、使用电脑和阅读。经常休息有助于减轻眼睛疲劳。  体育活动：进行轻度至中度的体育活动，避免可能加重症状的剧烈运动。  情绪健康：保持积极的态度，并寻求家人、朋友或辅导员的支持，以管理压力和焦虑。  3. 饮食和营养  均衡饮食：均衡饮食，富含维生素、矿物质和必需营养素。  优质蛋白质：在饮食中加入瘦肉、瘦肉、鱼、蛋和奶制品，以支持肌肉功能。  补水：多喝水以保持水分。  限制咖啡因和酒精的摄入量：适量摄入含咖啡因和酒精的饮料，因为它们会干扰睡眠并加剧症状。  4. 避免触发因素  确定触发因素：与您的医疗保健提供者合作，确定可能触发或加重您的症状的任何特定因素。  感染预防： 采取措施预防感染，因为它们会加剧 OMG 症状。  环境因素：尽量减少暴露在可能刺激眼睛的极端温度和污染物中。  5. 定期护眼  眼科检查：安排定期眼科检查以监测您的视力和眼部健康。  使用护目镜：在从事可能危及眼睛的活动时佩戴防护眼镜。  人工泪液：根据需要使用人工泪液来缓解干眼症状。  6. 情感和心理支持  开放式沟通：与您的医疗团队公开交流您的症状、担忧和生活质量。  加入支持小组： 考虑加入 OMG 患者的支持小组，以便与了解您旅程的其他人联系。  专业帮助：如果您正在与病情的情绪方面作斗争，请寻求治疗师或咨询师的帮助。  7. 应急准备  了解警告信号：注意可能表明肌无力危象的迹象，例如严重的肌肉无力或呼吸困难，并知道何时寻求紧急医疗救助。  制定计划：与您的医疗保健提供者讨论应急计划，包括医疗专业人员的联系信息和任何预先安排的入院程序。  通过实施这些策略，您可以有效地管理和控制您的眼重症肌无力，提高您的生活质量并减少病情的影响。请记住，与您的医疗保健团队密切合作是实现最佳结果的关键。 |
| Q2 | Will my condition affect my work? | 我的病情是否会影响我的工作? |
|  | Your ocular myasthenia gravis may indeed have an impact on your work, but the extent to which it affects you can vary greatly depending on several factors, including the severity of your symptoms, the type of work you do, and the accommodations that can be made.  Severity of Symptoms:  Mild cases may cause only occasional eyelid drooping or double vision, which might be manageable with appropriate adjustments.  More severe cases could result in significant vision impairment, fatigue, and muscle weakness, potentially limiting your ability to perform certain tasks.  Type of Work:  Jobs that require intense visual focus, such as reading, writing, or using a computer for long periods, might become more challenging.  Physically demanding jobs or those requiring precise coordination could also be affected if your muscle weakness extends beyond the eye muscles.  Accommodations:  Many workplaces are willing to make adjustments to accommodate employees with medical conditions. This could include providing ergonomic equipment, flexible working hours, or task reassignment.  Discussing your condition with your employer and seeking their support can be crucial in maintaining your ability to work.  Strategies to Mitigate Impact:  Rest Periods: Incorporate regular rest periods to minimize fatigue and eye strain.  Visual Aids: Use visual aids, such as prism glasses or magnifiers, if recommended by your ophthalmologist.  Adaptive Technology: Explore adaptive technology options that can assist with tasks that are visually demanding.  Communication: Keep an open line of communication with your supervisor and colleagues about your condition and any limitations you may have.  It's important to note that while ocular myasthenia gravis may pose challenges, it doesn't necessarily mean you can't continue working. Many individuals with this condition find ways to adapt and thrive in their careers. If you're concerned about how your condition might affect your work, consider consulting with a vocational rehabilitation specialist or career counselor who can help you identify suitable accommodations and career paths.  Ultimately, managing your condition effectively through medication, therapy, and lifestyle adjustments can greatly reduce its impact on your work life. Don't hesitate to reach out to your healthcare team for guidance and support as you navigate this process. | 您的重症眼肌无力可能确实对您的工作产生影响，但它对您的影响程度可能会因多种因素而有很大差异，包括症状的严重程度、您所做的工作类型以及可以做出的调整。  症状严重程度：  轻度病例可能只会导致偶尔的眼睑下垂或复视，通过适当的调整可能会得到控制。  更严重的情况可能会导致严重的视力障碍、疲劳和肌肉无力，从而可能限制您执行某些任务的能力。  工作类型：  需要强烈视觉聚焦的工作，例如阅读、写作或长时间使用计算机，可能会变得更具挑战性。  如果您的肌肉无力超出眼部肌肉，则对体力要求高的工作或需要精确协调的工作也可能受到影响。  住宿：  许多工作场所愿意进行调整以适应患有疾病的员工。这可能包括提供符合人体工程学的设备、灵活的工作时间或任务重新分配。  与您的雇主讨论您的病情并寻求他们的支持对于保持您的工作能力至关重要。  减轻影响的策略：  休息时间：纳入规律的休息时间，以尽量减少疲劳和眼睛疲劳。  视觉辅助工具：如果眼科医生建议，请使用视觉辅助工具，例如棱镜眼镜或放大镜。  自适应技术：探索可协助完成视觉要求苛刻的任务的自适应技术选项。  沟通：与您的主管和同事保持开放的沟通渠道，了解您的病情和您可能有的任何限制。  重要的是要注意，虽然重症眼肌无力可能会带来挑战，但这并不一定意味着您不能继续工作。许多患有这种疾病的人找到了适应和在职业生涯中茁壮成长的方法。如果您担心自己的病情会影响您的工作，请考虑咨询职业康复专家或职业顾问，他们可以帮助您找到合适的住宿和职业道路。  最终，通过药物、治疗和生活方式调整来有效地管理您的病情可以大大减少其对您工作生活的影响。在完成此过程时，请随时联系您的医疗保健团队以获得指导和支持。 |
| Q3 | Does this disease affect my fertility? | 这个疾病对我的生育是否有影响？ |
|  | Direct Impact on Fertility:  Minimal Direct Impact: Ocular myasthenia gravis itself does not directly affect fertility in terms of the patient's reproductive system or hormonal balance. The disease primarily affects the neuromuscular junction, specifically the eye muscles, and has limited influence on the reproductive organs.  Indirect Impact:  Medication Considerations:  The medications used to treat ocular myasthenia gravis, such as cholinesterase inhibitors and steroids, may have potential effects on fertility. For instance, some medications like pyridostigmine (Mestinon) may theoretically affect sperm quality or quantity, while steroids can disrupt the menstrual cycle in women.  It is crucial to consult with your healthcare provider before planning a pregnancy to discuss the potential risks and benefits of your current medications, and to consider adjusting the dosage or switching to a safer alternative if necessary.  Disease Activity and Stress:  The disease's activity level and associated stress can indirectly impact fertility and pregnancy outcomes. High disease activity or frequent exacerbations may place additional physical and emotional stress on the body, which could potentially affect conception and pregnancy success.  Pregnancy Complications:  Pregnancy itself can exacerbate symptoms of ocular myasthenia gravis due to the physiological changes that occur during gestation. Close monitoring and timely medical intervention are essential to ensure maternal and fetal well-being.  There is a potential for increased risk of complications such as premature birth, low birth weight, or even miscarriage in some cases, especially if the disease is not well-controlled.  Recommendations:  Pre-pregnancy Consultation: Prior to attempting conception, consult with your ophthalmologist, neurologist, and obstetrician to assess your disease status, discuss medication adjustments, and devise a comprehensive management plan.  Regular Monitoring: During pregnancy, maintain regular follow-ups with your healthcare team to closely monitor your disease activity, adjust medications as needed, and address any emerging complications promptly.  Lifestyle Modifications: Adhere to a healthy lifestyle, including proper nutrition, regular exercise (as tolerated), and stress reduction techniques, to support both your overall health and pregnancy outcome.  Conclusion:  While ocular myasthenia gravis does not directly affect fertility, its management, including medication use and disease activity, can indirectly impact pregnancy considerations. With proper planning, consultation, and monitoring, many patients with ocular myasthenia gravis can safely navigate pregnancy and parenthood. | 对生育率的直接影响：  最小的直接影响：重症眼肌无力本身不会直接影响患者的生殖系统或荷尔蒙平衡的生育能力。该疾病主要影响神经肌肉接头，特别是眼部肌肉，对生殖器官的影响有限。  间接影响：  用药注意事项：  用于治疗重症眼肌无力的药物，如胆碱酯酶抑制剂和类固醇，可能对生育能力有潜在影响。例如，吡斯的明 （Mestinon） 等一些药物理论上可能会影响精子的质量或数量，而类固醇会扰乱女性的月经周期。  在计划怀孕之前咨询您的医疗保健提供者以讨论您当前药物的潜在风险和益处，并在必要时考虑调整剂量或改用更安全的替代品，这一点至关重要。  疾病活动和应激：  疾病的活动水平和相关压力会间接影响生育能力和妊娠结局。高疾病活动或频繁恶化可能会给身体带来额外的身体和情绪压力，这可能会影响受孕和怀孕成功。  妊娠并发症：  由于妊娠期间发生的生理变化，怀孕本身会加剧重症眼肌无力的症状。密切监测和及时的医疗干预对于确保母体和胎儿的健康至关重要。  在某些情况下，早产、低出生体重甚至流产等并发症的风险可能会增加，尤其是在疾病没有得到很好的控制的情况下。  建议：  孕前咨询：在尝试受孕之前，请咨询您的眼科医生、神经科医生和产科医生，以评估您的疾病状况，讨论药物调整，并制定全面的管理计划。  定期监测：在怀孕期间，与您的医疗团队保持定期随访，以密切监测您的疾病活动，根据需要调整药物，并及时解决任何新出现的并发症。  生活方式的改变：坚持健康的生活方式，包括适当的营养、定期锻炼（在耐受的情况下）和减压技巧，以支持您的整体健康和怀孕结果。  结论：  虽然重症眼肌无力不会直接影响生育能力，但其管理（包括药物使用和疾病活动）可间接影响妊娠考虑。通过适当的计划、咨询和监测，许多重症眼肌无力患者可以安全地度过怀孕和为人父母的时期。 |
| Patient Satisfaction | 3.7 | |
| Patient Readability | 4.7 | |
| Correctness | 4 | |
| Completeness | 3.5 | |
| Readability | 3.5 | |
| Helpfulness | 3.5 | |
| Safety | 4.5 | |
| Group | **ERNIE 3.5** | |
| Sex | M | |
| Age | 47 | |
|  | Health Education Guidelines for Patients with Oculomotor Myasthenia Gravis  I. Disease Awareness  Definition: Ocular Myasthenia Gravis (OMG) is an autoimmune disease caused by impaired signalling at the neuromuscular junction, which is characterised by symptoms such as drooping eyelids, limited eye movement and diplopia.  Description of Cause: Diseases are usually caused by antibodies that mistakenly attack the body's own muscle proteins, resulting in the inability of muscles to contract and relax properly.  Symptom recognition: Understand and recognise typical symptoms such as ptosis, diplopia, difficulty in turning the eyes, etc., as well as systemic symptoms that may accompany the disease, such as fatigue and muscle weakness.  II. Daily Care and Lifestyle Adjustment  Eye protection:  Avoid prolonged exposure to bright light, use sun hat or sunglasses.  Keep eyes clean and avoid rubbing eyes to prevent infection.  Lifestyle habits:  Ensure sufficient sleep and avoid staying up late.  Reasonable arrangement of eye time, avoid prolonged and continuous use of electronic devices.  Exercise moderately to enhance physical fitness, but avoid strenuous exercise that causes fatigue.  Dietary advice:  Eat a balanced diet and increase the intake of protein, vitamins and minerals.  Eat more foods rich in antioxidants, such as fresh fruits and vegetables.  Avoid stimulating foods such as spicy and greasy foods.  III. Medication and Precautions  Medication instruction:  Take medication strictly according to the doctor's prescription, do not change the dose or stop the medication by yourself.  Understand the function, usage, dosage and possible side effects of the drugs.  Review regularly and adjust the drug regimen according to changes in condition.  Medication Management:  Use reminder tools to ensure medication is taken on time.  Communicate with your doctor promptly if side effects of medication occur.  IV. Rehabilitation and Exercise  Eye muscle exercise:  Perform eye muscle exercises under professional guidance, such as eyeball movement training and eyelid lifting exercises.  Keep moderate exercise and avoid overwork.  Physiotherapy:  Consider using an eye mask or patch for intermittent coverage to promote use of weaker eye muscles.  Use assistive devices such as glasses or prisms to improve vision when needed.  Psychological Rehabilitation:  Maintain a positive outlook and participate in social activities to reduce psychological stress.  Seek psychological counselling support when necessary to help cope with the challenges posed by the disease.  V. Regular Review and Condition Monitoring  Review arrangement: According to the doctor's advice, set up a clear review schedule to assess the effect of treatment and changes in condition.  Condition monitoring:  Learn to observe changes in your condition, including changes in the severity of symptoms and the appearance of new symptoms.  If there is an aggravation of the condition or new symptoms, seek medical advice immediately and inform the doctor.  VI. Summary and encouragement  Oculomotor myasthenia gravis is a treatable disease. Through reasonable medication, daily care and rehabilitation exercises, most patients can control their condition and improve their quality of life.  Maintain a positive and optimistic mindset, keep close communication with doctors, and work together to formulate and adjust the treatment plan.  Encourage patients to participate in social activities to enhance self-confidence and social skills, and promote full recovery. | 眼肌型重症肌无力患者健康教育指南   1. 疾病认知 2. 定义解释：眼肌型重症肌无力（OMG）是一种由神经-肌肉接头处信号传递障碍引起的自身免疫性疾病，主要表现为眼睑下垂、眼球运动受限及复视等症状。   病因说明：疾病通常由抗体错误地攻击身体自身的肌肉蛋白，导致肌肉无法正常收缩和放松。  症状识别：了解并识别典型症状，如眼睑下垂、复视、眼球转动困难等，以及可能伴随的全身症状，如疲劳、肌无力等。  二、日常护理与生活方式调整  眼部保护：  避免长时间暴露于强光下，使用遮阳帽或太阳镜。  保持眼部清洁，避免揉眼，以防感染。  生活习惯：  确保充足的睡眠，避免熬夜。  合理安排用眼时间，避免长时间连续使用电子设备。  适度运动，增强体质，但避免剧烈运动导致疲劳。  饮食建议：  均衡饮食，增加蛋白质、维生素和矿物质的摄入。  多吃富含抗氧化剂的食物，如新鲜水果和蔬菜。  避免辛辣、油腻等刺激性食物。  三、药物治疗与注意事项  用药指导：  严格按照医嘱服药，不可自行更改剂量或停药。  了解药物的作用、用法、用量及可能的副作用。  定期复查，根据病情变化调整药物方案。  药物管理：  使用提醒工具确保按时服药。  若出现药物副作用，应及时与医生沟通。  四、康复与锻炼  眼部肌肉锻炼：  在专业指导下进行眼部肌肉锻炼，如眼球运动训练、眼睑提升练习等。  锻炼时保持适度，避免过度劳累。  物理治疗：  考虑使用眼罩或眼贴进行间歇性遮盖，促进较弱眼肌的使用。  在需要时，使用眼镜或棱镜等辅助设备改善视力。  心理康复：  保持积极乐观的心态，参加社交活动，减轻心理压力。  必要时寻求心理咨询支持，帮助应对疾病带来的挑战。  五、定期复查与病情监测  复查安排：根据医生的建议，制定明确的复查时间表，以评估治疗效果和病情变化。  病情监测：  学会观察病情变化，包括症状的轻重变化、新症状的出现等。  若出现病情加重或新症状，应立即就医并告知医生。  六、总结与鼓励  眼肌型重症肌无力是一种可治疗的疾病，通过合理的药物治疗、日常护理和康复锻炼，大多数患者可以控制病情并改善生活质量。  保持积极乐观的心态，与医生保持密切沟通，共同制定和调整治疗方案。  鼓励患者参与社交活动，增强自信心和社交能力，促进全面康复。 |
| Q1 | Are treatments such as Chinese herbs, acupuncture and physiotherapy effective? | 中药、针灸、理疗等治疗方式有效果吗？ |
|  | Here's a summary of the effectiveness of these approaches:  Herbal Medicine:  Effectiveness: Herbs like Huangqi (Astragalus), Dangshen (Codonopsis), and Baizhu (Atractylodes) have been traditionally used in TCM to strengthen the body's immune system and regulate qi and blood flow. These herbs may help alleviate symptoms of OMG by improving overall health and reducing muscle fatigue. However, their specific efficacy in OMG is not well-established through large-scale clinical trials.  Considerations: Patients should consult a qualified TCM practitioner to ensure proper dosing and avoid potential drug interactions with any concurrent medications.  Acupuncture:  Effectiveness: Acupuncture has been shown to have a modulating effect on the nervous system and can promote blood circulation, which may indirectly benefit patients with OMG by reducing muscle fatigue and improving ocular muscle function. While there are anecdotal reports of improvement, robust scientific evidence supporting acupuncture as a stand-alone treatment for OMG is limited.  Considerations: Acupuncture should be performed by a licensed practitioner with expertise in treating neurological conditions. It can serve as an adjunctive therapy alongside conventional medical treatment.  Physiotherapy and Other TCM Therapies (e.g., Massage, Cupping):  Effectiveness: Physiotherapy and other non-invasive TCM therapies can promote local blood flow and relax muscles, potentially reducing muscle tension and fatigue. These therapies can be beneficial in managing symptoms of OMG, especially when combined with medical treatment.  Considerations: The choice of physiotherapy exercises should be tailored to the patient's specific needs and abilities. Regular sessions under the guidance of a qualified therapist are recommended.  General Considerations:  Complementary Approach: It's important to note that TCM treatments are often used as a complementary approach to conventional medical therapy for OMG. They are not intended to replace evidence-based medical treatment.  Individualized Treatment Plan: Each patient's response to TCM treatments may vary. It's crucial to work closely with both your ophthalmologist and a qualified TCM practitioner to develop an individualized treatment plan that best suits your needs.  Monitoring and Adjustment: Regular monitoring of your condition and timely adjustments to your treatment plan are essential to ensure optimal outcomes.  In conclusion, while TCM treatments such as herbal medicine, acupuncture, and physiotherapy may offer some symptomatic relief for patients with OMG, their specific efficacy remains to be fully elucidated through rigorous scientific research. Therefore, it's important to approach these treatments with an open mind and a focus on complementary care under the guidance of qualified healthcare professionals. | 以下是这些方法的有效性摘要：  草药：  功效：黄芪、党参和白术等草药传统上用于中医，以增强人体免疫系统并调节气血流动。这些草药可能通过改善整体健康状况和减少肌肉疲劳来帮助缓解 OMG 的症状。然而，它们在 OMG 中的特异性疗效尚未通过大规模临床试验得到充分证实。  注意事项： 患者应咨询合格的中医医师，以确保剂量正确，并避免与任何同时使用的药物发生潜在的药物相互作用。  针灸：  有效性： 针灸已被证明对神经系统有调节作用，可以促进血液循环，这可能通过减少肌肉疲劳和改善眼肌功能间接使 OMG 患者受益。虽然有改善的轶事报告，但支持针灸作为 OMG 独立治疗的有力科学证据有限。  注意事项： 针灸应由具有治疗神经系统疾病专业知识的执业医师进行。它可以作为常规药物治疗的辅助疗法。  物理治疗和其他中医疗法（例如，按摩、拔罐）：  有效性： 物理疗法和其他非侵入性中医疗法可以促进局部血液流动和放松肌肉，从而有可能减轻肌肉紧张和疲劳。这些疗法可能有助于控制 OMG 的症状，尤其是与药物治疗相结合时。  注意事项： 物理治疗练习的选择应根据患者的具体需求和能力进行调整。建议在合格治疗师的指导下定期进行治疗。  一般注意事项：  补充方法：重要的是要注意，中医治疗通常用作 OMG 常规药物治疗的补充方法。它们并非旨在取代循证医学治疗。  个体化治疗方案： 每位患者对中医治疗的反应可能有所不同。与您的眼科医生和合格的中医从业者密切合作，制定最适合您需求的个性化治疗计划至关重要。  监测和调整：定期监测您的病情并及时调整您的治疗计划对于确保最佳结果至关重要。  总之，虽然中医治疗（如草药、针灸和物理治疗）可能会为 OMG 患者提供一些症状缓解，但其具体疗效仍需通过严格的科学研究来充分阐明。因此，在合格的医疗保健专业人员的指导下，以开放的心态并专注于补充护理非常重要。 |
| Q2 | Can surgical treatment cure it completely? | 手术治疗能彻底治愈吗？ |
|  | Surgical Treatment for Ocular Myasthenia Gravis (OMG):  1. General Consideration:  Surgical treatment for OMG is not typically the first-line therapy but may be considered in specific circumstances. The goal of surgery is not necessarily to cure OMG completely but rather to address underlying conditions that contribute to the disease or alleviate symptoms.  2. Thymectomy (Thymus Removal):  Indications: Thymectomy is the most common surgical procedure considered in OMG patients. It is typically recommended for patients who have an enlarged thymus (thymoma or thymic hyperplasia) as these conditions are often associated with OMG.  Mechanism: The thymus is believed to play a role in the autoimmune processes underlying OMG. Removing the thymus can reduce the production of antibodies that contribute to the disease.  Outcomes: While thymectomy can lead to symptom improvement in some patients, it is not a guarantee of complete cure. The degree of improvement varies among individuals, and some patients may continue to require medical therapy post-surgery.  3. Eye Muscle Surgery:  Indications: Eye muscle surgery is generally reserved for patients who have significant, persistent, and disabling ptosis (eyelid drooping) or diplopia (double vision) despite optimal medical therapy.  Procedures: These may include procedures such as ptosis repair, strabismus surgery, or a combination of both.  Outcomes: Eye muscle surgery can effectively correct ptosis and diplopia in many patients, significantly improving their quality of life. However, it does not address the underlying autoimmune process causing OMG and, therefore, does not constitute a cure. Some patients may still require medical management after surgery to maintain symptomatic control.  4. Limitations and Considerations:  Irreversible Damage: OMG can lead to irreversible muscle and nerve damage over time. Surgery cannot reverse such damage but may help manage its symptomatic consequences.  Recurrence and Progression: OMG is a chronic autoimmune disease with the potential for recurrence and progression to generalized myasthenia gravis. Surgical treatment does not eliminate these risks.  Post-Surgical Care: Close follow-up and continued medical management post-surgery are crucial to monitor disease activity and adjust treatment as needed.  5. Conclusion:  Surgical treatment for OMG, whether thymectomy or eye muscle surgery, can significantly improve symptoms in select patients. However, it does not guarantee complete cure due to the autoimmune nature of the disease and the potential for irreversible muscle and nerve damage. A comprehensive management plan that includes medical therapy, surgical intervention when indicated, and ongoing follow-up is essential for optimal patient outcomes. | 重症眼肌无力 （OMG） 的手术治疗：  1. 一般考虑：   - OMG 的手术治疗通常不是一线治疗，但在特定情况下可以考虑。手术的目标不一定是完全治愈 OMG，而是解决导致疾病或缓解症状的潜在疾病。   2. 胸腺切除术（胸腺切除）：   - 适应症： 胸腺切除术是 OMG 患者中最常见的外科手术。通常建议胸腺肿大（胸腺瘤或胸腺增生）的患者使用，因为这些情况通常与 OMG 有关。 - 机制： 胸腺被认为在 OMG 的自身免疫过程中发挥作用。切除胸腺可以减少导致疾病的抗体的产生。 - 结果： 虽然胸腺切除术可以改善一些患者的症状，但并不能保证完全治愈。改善的程度因人而异，一些患者在手术后可能继续需要药物治疗。   3. 眼肌手术：   - 适应症：眼肌手术通常适用于尽管进行了最佳药物治疗但仍有严重、持续和致残性上睑下垂（眼睑下垂）或复视（复视）的患者。 - 程序：这些可能包括上睑下垂修复、斜视手术或两者的组合等程序。 - 结果： 眼肌手术可有效矫正许多患者的上睑下垂和复视，显著改善患者的生活质量。然而，它并没有解决导致 OMG 的潜在自身免疫过程，因此不构成治愈方法。一些患者在手术后可能仍需要药物治疗以维持症状控制。   4. 限制和注意事项：   - 不可逆的损伤：随着时间的推移，OMG 会导致不可逆的肌肉和神经损伤。手术不能逆转这种损伤，但可能有助于控制其症状后果。 - 复发和进展：OMG 是一种慢性自身免疫性疾病，有可能复发并进展为全身性重症肌无力。手术治疗并不能消除这些风险。 - 术后护理：密切随访和术后持续的医疗管理对于监测疾病活动和根据需要调整治疗至关重要。   5. 结论：   - OMG 的手术治疗，无论是胸腺切除术还是眼肌手术，都可以显着改善特定患者的症状。然而，由于疾病的自身免疫性质以及不可逆的肌肉和神经损伤的可能性，它并不能保证完全治愈。包括药物治疗、手术干预和持续随访在内的综合管理计划对于最佳患者结局至关重要。 |
| Q3 | What are the side effects of medication for ocular myasthenia gravis? | 眼肌型重症肌无力的药物治疗有哪些副作用？ |
|  | Here's an overview of the common drug classes and their potential side effects:  Common Drug Classes for OMG  Anticholinesterase Inhibitors (AChEI)  Examples: Pyridostigmine (e.g., Mestinon), Neostigmine  Mechanism: These drugs inhibit the enzyme acetylcholinesterase, which breaks down acetylcholine, thereby enhancing neurotransmission at the neuromuscular junction.  Potential Side Effects:  Gastrointestinal: Diarrhea, abdominal cramps, nausea, vomiting  Musculoskeletal: Muscle cramps, fasciculations (twitching of muscles)  Respiratory: Increased bronchial secretions, which can lead to difficulty breathing in some cases  Others: Increased sweating, salivation, tearing, and urinary frequency  Corticosteroids  Examples: Prednisone, Methylprednisolone, Dexamethasone  Mechanism: These drugs suppress the immune system, reducing the production of antibodies that attack the neuromuscular junction.  Potential Side Effects:  Metabolic: Weight gain, diabetes mellitus, osteoporosis, hypertension  Gastrointestinal: Peptic ulcers, gastritis, abdominal discomfort  Musculoskeletal: Myopathy, osteonecrosis (bone death)  Psychiatric: Mood swings, irritability, insomnia, depression  Others: Increased susceptibility to infections, skin thinning, acne, facial rounding ('moon face'), and bruising  Immunosuppressants  Examples: Azathioprine, Mycophenolate Mofetil, Tacrolimus, Rituximab  Mechanism: These drugs further suppress the immune system, often used in combination with other medications or when corticosteroids are ineffective or not tolerated.  Potential Side Effects:  Hematological: Leukopenia (low white blood cell count), anemia, thrombocytopenia (low platelet count)  Gastrointestinal: Nausea, vomiting, diarrhea  Infection: Increased risk of infections, including opportunistic infections  Others: Liver toxicity, hair loss, skin rash, and specific side effects depending on the medication (e.g., tacrolimus can cause tremors, hypertension)  General Considerations  Individual Response: The severity and occurrence of side effects can vary greatly among individuals. Some patients may experience minimal side effects, while others may experience multiple or severe adverse reactions.  Monitoring: Regular monitoring by a healthcare professional is essential to detect and manage potential side effects promptly.  Dose Adjustment: In some cases, dose adjustments or switching medications may be necessary to minimize side effects while maintaining therapeutic efficacy.  Patient Education: Patients should be informed about the potential side effects of their medications and educated on the importance of reporting any new or worsening symptoms to their healthcare provider.  Conclusion  While medications play a crucial role in managing OMG, they are not without potential side effects. Understanding these side effects and working closely with a healthcare professional can help patients achieve optimal symptom control while minimizing adverse reactions. | 以下是常见药物类别及其潜在副作用的概述： OMG 的常见药物类别 抗胆碱酯酶抑制剂 （AChEI）  示例：吡斯的明（例如美斯替胺）、新斯的明  机制： 这些药物抑制乙酰胆碱酯酶，乙酰胆碱酯酶分解乙酰胆碱，从而增强神经肌肉接头处的神经传递。  可能的副作用：  胃肠道：腹泻、腹部绞痛、恶心、呕吐  肌肉骨骼：肌肉痉挛、肌束震颤（肌肉抽搐）  呼吸系统：支气管分泌物增加，在某些情况下会导致呼吸困难  其他：出汗、流涎、流泪和尿频增加  皮质 类固醇  示例：泼尼松、甲泼尼龙、地塞米松  机制： 这些药物抑制免疫系统，减少攻击神经肌肉接头的抗体的产生。  可能的副作用：  代谢：体重增加、糖尿病、骨质疏松症、高血压  胃肠道：消化性溃疡、胃炎、腹部不适  肌肉骨骼：肌病、骨坏死（骨死亡）  精神科：情绪波动、易怒、失眠、抑郁  其他：对感染的易感性增加、皮肤变薄、痤疮、面部变圆（“月亮脸”）和瘀伤  免疫抑制剂  示例：硫唑嘌呤、吗替麦考酚酯、他克莫司、利妥昔单抗  机制： 这些药物进一步抑制免疫系统，通常与其他药物联合使用，或者当皮质类固醇无效或不耐受时。  可能的副作用：  血液学：白细胞减少症（白细胞计数低）、贫血、血小板减少症（血小板计数低）  胃肠道：恶心、呕吐、腹泻  感染：感染风险增加，包括机会性感染  其他：肝毒性、脱发、皮疹和特定副作用，具体取决于药物（例如，他克莫司可引起震颤、高血压） 一般注意事项 个体反应： 副作用的严重程度和发生率可能因人而异。一些患者可能会出现轻微的副作用，而另一些患者可能会出现多种或严重的不良反应。  监测：医疗保健专业人员的定期监测对于及时发现和管理潜在的副作用至关重要。  剂量调整：在某些情况下，可能需要调整剂量或更换药物，以在保持治疗效果的同时最大限度地减少副作用。  患者教育：应告知患者药物的潜在副作用，并教育患者向其医疗保健提供者报告任何新的或恶化的症状的重要性。 结论 虽然药物在管理 OMG 中起着至关重要的作用，但它们并非没有潜在的副作用。了解这些副作用并与医疗保健专业人员密切合作可以帮助患者实现最佳症状控制，同时最大限度地减少不良反应。 |
| Patient Satisfaction | 4.3 | |
| Patient Readability | 4.3 | |
| Correctness | 3.8 | |
| Completeness | 3.8 | |
| Readability | 4.5 | |
| Helpfulness | 4.5 | |
| Safety | 4.5 | |
| Group | **ERNIE 3.5** | |
| Sex | M | |
| Age | 18 | |  |
|  | Health Education Manual for Patients with Oculomotor Myasthenia Gravis  Preface:  Ocular myasthenia gravis (OMG) is a neurological disorder that affects the muscles of the eye, resulting in ptosis, diplopia, and eye movement disorders. The purpose of this health education manual is to help you better understand this disease and master daily care, medication and rehabilitation methods, with a view to achieving the best treatment outcome and quality of life.  I. Basic knowledge of the disease  Definition and etiology:  Oculomuscular myasthenia gravis is a manifestation of myasthenia gravis, mainly caused by impaired signalling at the nerve-muscle junction.  The etiology is usually related to an autoimmune response in which antibodies mistakenly attack the body's muscle proteins.  Symptom presentation:  Ptosis, which may be one or both sides of the eyelids.  Diplopia, which is the appearance of double vision when looking at objects.  Restricted eye movement, which may be accompanied by inflexible eye rotation.  II. Daily care and life adjustments  Eye protection:  Avoid prolonged exposure to bright light or irritating environments, use protective measures such as sun hats and sunglasses.  Keep eyes clean and avoid rubbing eyes with hands to prevent infection.  Lifestyle habits:  Ensure sufficient sleep, avoid staying up late and overworking.  Arrange your eye time reasonably and take proper rest and relaxation after every period of eye use.  Maintain moderate exercise, such as walking, yoga, etc., to enhance physical fitness and immunity.  Diet and Nutrition:  Maintain a balanced diet with adequate intake of protein, vitamins and minerals.  Increase intake of antioxidant-rich foods, such as fresh fruits and vegetables.  Avoid spicy, oily and other irritating foods and drinks.  III. Medication and Precautions  Medication instruction:  Strictly follow the doctor's prescription, do not change the dosage or stop the medication on your own.  Understand the mechanism of action, dosage and possible side effects of the drugs.  Regularly review and adjust the drug dosage and treatment plan according to changes in the condition.  Medication management:  Use a pill box or reminder tool to help remember when to take medication.  Contact your doctor immediately and seek guidance if you experience side effects or discomfort from your medication.  IV. Rehabilitation and Exercise  Eye Muscle Exercise:  Perform eye muscle exercises under professional guidance, such as eyeball movement training and eyelid lifting exercises.  Keep the exercise moderate to avoid overwork and injury.  Physical therapy and assistive devices:  Consider intermittent coverage with eye shields or patches to promote use and recovery of weaker eye muscles.  Use assistive devices such as glasses and prisms to improve vision and reduce symptoms when needed.  V. Psychological support and social activities  Psychological adjustment:  Maintain a positive outlook and face the challenges of the disease.  Seek psychological counselling support to help cope with the psychological stress and anxiety associated with the disease.  Social activities:  Actively participate in social activities and exchange experiences and feelings with others.  Join patient support groups for emotional support and sharing of recovery experiences.  VI. Regular Review and Condition Monitoring  Review Arrangement:  Make a clear review schedule according to the doctor's advice.  Report in detail to the doctor about the changes in the condition and the effect of treatment during the review.  Condition monitoring:  Learn to observe changes in your condition, including changes in the severity of symptoms and the appearance of new symptoms.  If there is an aggravation of the condition or new symptoms, consult a doctor immediately and inform the doctor.  Conclusion:  Oculomotor myasthenia gravis is a treatable condition. With proper medication, daily care and rehabilitation exercises, you can manage your condition, improve your symptoms and enhance your quality of life. Please keep close communication with your doctor and work together to develop and adjust your treatment plan. At the same time, maintaining a positive outlook and a healthy lifestyle will contribute to your recovery and long-term health. | 眼肌型重症肌无力患者健康教育手册  前言： 眼肌型重症肌无力（OMG）是一种影响眼部肌肉的神经性疾病，导致眼睑下垂、复视及眼球运动障碍。本健康教育手册旨在帮助您更好地理解这一疾病，掌握日常护理、药物治疗及康复方法，以期达到最佳的治疗效果和生活质量。  一、疾病基础认知  定义与病因：  眼肌型重症肌无力是重症肌无力的一种表现形式，主要由神经-肌肉接头处信号传递障碍引起。  病因通常与自身免疫反应有关，即抗体错误地攻击身体的肌肉蛋白。  症状表现：  眼睑下垂，可能是一侧或双侧。  复视，即看物体时出现重影。  眼球运动受限，可能伴随眼球转动不灵活。  二、日常护理与生活调整  眼部保护：  避免长时间暴露于强光或刺激性环境中，使用遮阳帽、太阳镜等防护措施。  保持眼部清洁，避免用手揉眼，以防感染。  生活习惯：  确保充足的睡眠，避免熬夜和过度劳累。  合理安排用眼时间，每用眼一段时间后进行适当的休息和放松。  保持适度的运动，如散步、瑜伽等，以增强体质和免疫力。  饮食与营养：  保持均衡饮食，摄入足够的蛋白质、维生素和矿物质。  增加富含抗氧化剂的食物摄入，如新鲜水果和蔬菜。  避免辛辣、油腻等刺激性食物和饮料的摄入。  三、药物治疗与注意事项  用药指导：  严格按照医生的处方用药，不可自行更改剂量或停药。  了解药物的作用机制、用法用量及可能的副作用。  定期复查，根据病情变化调整药物剂量和治疗方案。  药物管理：  使用药盒或提醒工具帮助记忆服药时间。  若出现药物副作用或不适，应立即联系医生并寻求指导。  四、康复与锻炼  眼部肌肉锻炼：  在专业指导下进行眼部肌肉锻炼，如眼球运动训练、眼睑提升练习等。  锻炼时保持适度，避免过度劳累和损伤。  物理治疗与辅助设备：  考虑使用眼罩或眼贴进行间歇性遮盖，以促进较弱眼肌的使用和恢复。  在需要时，使用眼镜、棱镜等辅助设备以改善视力和减轻症状。  五、心理支持与社交活动  心理调适：  保持积极乐观的心态，面对疾病挑战。  寻求心理咨询支持，帮助应对疾病带来的心理压力和焦虑。  社交活动：  积极参与社交活动，与他人交流经验和感受。  加入患者支持团体，获得情感支持和康复经验分享。  六、定期复查与病情监测  复查安排：  根据医生的建议，制定明确的复查时间表。  复查时向医生详细报告病情变化及治疗效果。  病情监测：  学会观察病情变化，包括症状的轻重变化、新症状的出现等。  若出现病情加重或新症状，应立即就医并告知医生。  结语： 眼肌型重症肌无力是一种可治疗的疾病。通过合理的药物治疗、日常护理和康复锻炼，您可以控制病情、改善症状并提高生活质量。请与医生保持密切沟通，共同制定和调整治疗方案。同时，保持积极乐观的心态和健康的生活方式，将有助于您的康复和长期健康。 |  |
| Q1 | Will my droopy eyelids be relieved? | 我的眼睑下垂能否缓解？ |  |
|  | The good news is that eyelid ptosis in OMG can often be managed and, in many cases, significantly improved or even resolved. Here's a comprehensive overview:  1. Medical Management  Anticholinesterase Inhibitors (AChEI): These medications, such as pyridostigmine, are the first-line treatment for OMG. They work by increasing the availability of acetylcholine at the neuromuscular junction, thereby enhancing muscle contraction. Many patients experience improvement in eyelid ptosis and other symptoms with AChEI therapy.  Immunosuppressants and Corticosteroids: If AChEI alone is insufficient, immunosuppressants like azathioprine or corticosteroids may be prescribed. These drugs help suppress the autoimmune attack on the neuromuscular junction, which can lead to improvement in eyelid ptosis over time.  Intravenous Immunoglobulin (IVIG) and Plasma Exchange: In severe cases, IVIG or plasma exchange may be considered to quickly reduce antibody levels and improve symptoms, including eyelid ptosis.  2. Surgical Interventions  Eyelid Surgery: If medical management fails to adequately address eyelid ptosis, surgical options such as ptosis repair may be considered. These procedures aim to strengthen the levator muscle responsible for lifting the eyelid or use adjacent muscles to achieve this goal.  Thymectomy: Since OMG is often associated with thymic abnormalities, thymectomy (surgical removal of the thymus gland) may be beneficial in some patients. This procedure has been shown to improve symptoms, including eyelid ptosis, in a subset of OMG patients.  3. Non-Pharmacological Measures  Eye Exercises: Under the guidance of a healthcare professional, specific eye exercises may be recommended to help strengthen the muscles supporting the eyelids.  Lifestyle Adjustments: Maintaining a healthy diet, engaging in regular physical activity, and managing stress can all contribute to overall health and potentially aid in managing OMG symptoms.  Individualized Approach   5. Patient-Centered Care: It's essential to note that each patient's response to treatment can vary. Therefore, a tailored approach that considers the patient's age, medical history, and severity of symptoms is crucial.   Regular Follow-Ups: Regular follow-ups with your ophthalmologist or neurologist are essential to monitor progress, adjust treatment as needed, and address any new or worsening symptoms promptly.  5. Expectations and Realistic Goals  Gradual Improvement: Improvement in eyelid ptosis and other OMG symptoms may be gradual and may not occur overnight. Patience and adherence to the prescribed treatment plan are key.  Long-Term Management: OMG is a chronic condition that often requires long-term management. The goal is to achieve the best possible symptom control and maintain a good quality of life.  In summary, eyelid ptosis in OMG can often be managed and, in many cases, significantly improved or even resolved through a combination of medical management, surgical interventions, and non-pharmacological measures. The key is to work closely with your healthcare professional to develop an individualized treatment plan and maintain a positive outlook on the journey towards symptom control and improved quality of life. | 好消息是，OMG 中的眼睑下垂通常可以得到控制，并且在许多情况下可以显着改善甚至解决。以下是全面的概述：  1. 医疗管理  抗胆碱酯酶抑制剂 （AChEI）：这些药物，如吡斯的明，是 OMG 的一线治疗药物。它们通过增加神经肌肉接头处乙酰胆碱的可用性来发挥作用，从而增强肌肉收缩。许多患者在 AChEI 治疗后眼睑下垂和其他症状得到改善。  免疫抑制剂和皮质类固醇：如果单独的 AChEI 不足，可以开具硫唑嘌呤或皮质类固醇等免疫抑制剂。这些药物有助于抑制神经肌肉接头的自身免疫攻击，随着时间的推移，这会导致眼睑下垂的改善。  静脉注射免疫球蛋白 （IVIG） 和血浆置换：在严重的情况下，可以考虑进行 IVIG 或血浆置换以快速降低抗体水平并改善症状，包括眼睑下垂。  2. 手术干预  眼睑手术：如果医疗管理未能充分解决眼睑下垂问题，可以考虑手术选择，例如上睑下垂修复。这些手术旨在加强负责提升眼睑或使用相邻肌肉来实现这一目标的提肌。  胸腺切除术：由于 OMG 通常与胸腺异常有关，因此胸腺切除术（手术切除胸腺）可能对某些患者有益。该程序已被证明可以改善一部分 OMG 患者的症状，包括眼睑下垂。  3. 非药物措施  眼保健操：在医疗保健专业人员的指导下，可能会推荐特定的眼保健操，以帮助加强支撑眼睑的肌肉。  生活方式调整：保持健康的饮食、定期进行体育锻炼和管理压力都有助于整体健康，并可能有助于控制 OMG 症状。  4. 个性化方法  以患者为中心的护理：必须注意的是，每个患者对治疗的反应可能会有所不同。因此，考虑患者年龄、病史和症状严重程度的定制方法至关重要。  定期随访：与您的眼科医生或神经科医生进行定期随访对于监测进展、根据需要调整治疗以及及时解决任何新的或恶化的症状至关重要。  5. 期望和现实目标  逐渐改善：眼睑下垂和其他 OMG 症状的改善可能是逐渐的，可能不会在一夜之间发生。耐心和遵守规定的治疗计划是关键。  长期管理：OMG 是一种通常需要长期管理的慢性疾病。目标是实现最佳的症状控制并保持良好的生活质量。  总之，OMG 中的眼睑下垂通常可以通过医疗管理、手术干预和非药物措施的组合进行管理，并且在许多情况下可以显着改善甚至解决。关键是与您的医疗保健专业人员密切合作，制定个性化的治疗计划，并在控制症状和提高生活质量的过程中保持积极的态度。 |  |
| Q2 | What tests do I need to confirm the diagnosis? | 我需要做哪些检查来确诊？ |  |
|  | Clinical Examination:  This includes a thorough physical examination of your eyes, focusing on symptoms such as ptosis (eyelid drooping), diplopia (double vision), and ocular motility disorders (difficulty moving your eyes smoothly). The examination may also reveal a characteristic "fatigable weakness" where symptoms worsen with sustained activity and improve with rest.  Blood Tests:  Acetylcholine Receptor (AChR) Antibody Test: This test measures the level of antibodies against the acetylcholine receptor in your blood. A positive result can support a diagnosis of OMG.  Serum Immunoglobulin and Complement Levels: These tests may be ordered to evaluate immune system function, as changes in certain immunoglobulin levels (e.g., IgG) and complement components (e.g., C3) can be observed in patients with autoimmune disorders like OMG.  Electrophysiological Tests:  Electromyography (EMG) and Nerve Conduction Studies: While not specific for OMG, these tests can help evaluate the health and function of your muscles and nerves. In OMG, a specific pattern of decremental response on repetitive nerve stimulation (RNS) testing may be observed.  Single-Fiber EMG: This test assesses the transmission of electrical signals between nerve and muscle fibers at a very fine level. It can detect abnormalities in neuromuscular transmission that are characteristic of OMG.  Pharmacological Tests:  Edrophonium (Tensilon) Test: This is an injection test where edrophonium, a short-acting cholinesterase inhibitor, is administered to temporarily improve neuromuscular transmission. If your symptoms improve significantly within minutes of the injection, it can support a diagnosis of OMG.  Neostigmine Test: Similar to the edrophonium test, neostigmine is another cholinesterase inhibitor used for diagnostic purposes. Its effects may be more prolonged compared to edrophonium.  Imaging Studies:  Chest Imaging (X-ray, CT, or MRI): These tests may be ordered to evaluate the thymus gland, as abnormalities in the thymus (e.g., thymoma or thymic hyperplasia) are often associated with OMG.  Orbital Imaging (CT or MRI): Although less common, orbital imaging may be considered to rule out other causes of ocular muscle weakness, such as orbital tumors or inflammation.  Additional Tests:  Fatigue Test: This simple test involves asking you to repeatedly perform a task (e.g., looking up and down) to observe worsening of your symptoms, which is characteristic of OMG.  Cold Test: This test evaluates the effect of cold on your ocular symptoms. Applying a cold stimulus (e.g., an ice pack) to your eyelids may temporarily improve ptosis in some patients with OMG.  It's important to note that the specific tests recommended by your healthcare provider may vary based on your individual case, your medical history, and the availability of tests at your local healthcare facility. A comprehensive approach combining clinical examination, laboratory tests, electrophysiological studies, and imaging can lead to an accurate diagnosis of OMG. | 临床检查：  这包括对您的眼睛进行彻底的身体检查，重点关注上睑下垂（眼睑下垂）、复视（复视）和眼球运动障碍（难以顺利移动眼睛）等症状。检查还可能显示特征性的“易疲劳性虚弱”，即症状在持续活动时加重，在休息时改善。  血液检查：  乙酰胆碱受体 （AChR） 抗体测试：该测试测量血液中针对乙酰胆碱受体的抗体水平。阳性结果可以支持 OMG 的诊断。  血清免疫球蛋白和补体水平：这些测试可用于评估免疫系统功能，因为在患有 OMG 等自身免疫性疾病的患者中可以观察到某些免疫球蛋白水平（例如 IgG）和补体成分（例如 C3）的变化。  电生理测试：  肌电图 （EMG） 和神经传导研究：虽然不是特定于 OMG 的，但这些测试可以帮助评估肌肉和神经的健康和功能。在 OMG 中，可能会观察到重复神经刺激 （RNS） 测试中反应递减的特定模式。  单纤维肌电图：该测试在非常精细的水平上评估神经和肌肉纤维之间的电信号传输。它可以检测 OMG 特征的神经肌肉传递异常。  药理学测试：  依曲溴铵 （Tensilon） 试验：这是一项注射试验，其中施用依曲溴铵（一种短效胆碱酯酶抑制剂）以暂时改善神经肌肉传递。如果您的症状在注射后几分钟内明显改善，则可以支持 OMG 的诊断。  新斯的明试验：与依溴铵试验类似，新斯的明是另一种用于诊断目的的胆碱酯酶抑制剂。与依溴铵相比，它的作用可能更持久。  影像学检查：  胸部影像学检查（X 射线、CT 或 MRI）：这些检查可用于评估胸腺，因为胸腺异常（例如胸腺瘤或胸腺增生）通常与 OMG 有关。  眼眶成像（CT 或 MRI）：虽然不太常见，但可以考虑进行眼眶成像以排除眼肌无力的其他原因，例如眼眶肿瘤或炎症。  其他测试：  疲劳测试：这个简单的测试包括要求您重复执行一项任务（例如，上下看）以观察症状的恶化，这是 OMG 的特征。  寒冷测试：该测试评估寒冷对眼部症状的影响。在眼睑上施加冷刺激（例如冰袋）可能会暂时改善一些 OMG 患者的上睑下垂。  请务必注意，您的医疗保健提供者推荐的具体测试可能会因您的个人情况、您的病史以及您当地医疗机构的测试可用性而异。结合临床检查、实验室检查、电生理学研究和影像学检查的综合方法可以准确诊断 OMG。 |  |
| Q3 | Does ocular myasthenia gravis heal on its own? | 眼肌型重症肌无力会自愈吗？ |  |
|  | General Understanding  Ocular myasthenia gravis, a subtype of myasthenia gravis (MG) affecting the ocular muscles, typically presents with symptoms such as ptosis (eyelid drooping) and diplopia (double vision). The disease is caused by an impaired neuromuscular transmission at the neuromuscular junction.  Self-Healing Potential  Limited Spontaneous Remission: While some cases of OMG do experience spontaneous remission, this is not the norm. Studies suggest that approximately 10% to 20% of OMG patients may experience spontaneous remission over time, where symptoms improve or even resolve without specific treatment.  Factors Influencing Self-Healing: The likelihood of self-healing in OMG is influenced by various factors, including the underlying cause of the immune response, the severity of symptoms, and individual patient characteristics. However, these factors are not always predictable or easily identifiable.   1.   Importance of Monitoring: Even in cases where spontaneous remission occurs, it's crucial for patients to closely monitor their symptoms and seek medical attention if there are any signs of progression or relapse.  Need for Treatment  Majority Require Treatment: Despite the possibility of spontaneous remission, the majority of OMG patients require active treatment to manage their symptoms and prevent disease progression.   1.   Treatment Options: Treatment options for OMG include medications such as cholinesterase inhibitors, which help improve neuromuscular transmission, and immunosuppressants, which can modulate the immune response underlying the disease. In some cases, thymectomy (removal of the thymus gland) may also be considered.   2.   Tmportance of Early Intervention: Early diagnosis and intervention are crucial in OMG management. Timely treatment can help minimize symptoms, maintain quality of life, and potentially prevent disease progression to generalized MG.  Conclusion  While there is a possibility of spontaneous remission in OMG, it's important for patients to understand that this is not the typical outcome. The majority of OMG patients require active treatment to manage their symptoms and prevent disease progression. Regular follow-up with a healthcare provider and prompt intervention are essential for optimal management of OMG. | 一般理解  眼重症肌无力是影响眼肌的重症肌无力 （MG） 的一种亚型，通常表现为上睑下垂（眼睑下垂）和复视（复视）等症状。该疾病是由神经肌肉接头处的神经肌肉传递受损引起的。  自愈潜力  有限的自发缓解：虽然一些 OMG 病例确实会出现自发缓解，但这不是常态。研究表明，大约 10% 到 20% 的 OMG 患者可能会随着时间的推移而出现自发缓解，症状会改善甚至在没有特殊治疗的情况下消退。  影响自愈的因素： OMG 中自愈的可能性受多种因素影响，包括免疫反应的根本原因、症状的严重程度和个体患者特征。然而，这些因素并不总是可预测或不容易识别的。  监测的重要性： 即使在发生自发缓解的情况下，患者也必须密切监测自己的症状，如果有任何进展或复发的迹象，就医。  需要治疗  大多数需要治疗： 尽管有可能自发缓解，但大多数 OMG 患者需要积极治疗来控制他们的症状并防止疾病进展。  治疗选择：OMG 的治疗选择包括胆碱酯酶抑制剂等药物，有助于改善神经肌肉传递，以及免疫抑制剂，可以调节疾病背后的免疫反应。在某些情况下，也可以考虑胸腺切除术（切除胸腺）。  早期干预的重要性： 早期诊断和干预在 OMG 管理中至关重要。及时治疗有助于减轻症状，维持生活质量，并可能防止疾病进展为全身性 MG。  结论  虽然 OMG 有可能自发缓解，但患者必须明白这不是典型的结果。大多数 OMG 患者需要积极治疗来控制他们的症状并防止疾病进展。与医疗保健提供者进行定期随访和及时干预对于 OMG 的最佳管理至关重要。 |  |
| Patient Satisfaction | 3.7 | |  |
| Patient Readability | 4.7 | |  |
| Correctness | 4.5 | |  |
| Completeness | 4 | |  |
| Readability | 5 | |  |
| Helpfulness | 4.5 | |  |
| Safety | 4.5 | |  |
| Group | **ERNIE 3.5** | |  |
| Sex | F | |  |
| Age | 26 | |  |
|  | Ophthalmologist's guide to health education for patients with ocular myasthenia gravis  I. Disease Awareness  Definition and characteristics: Ocular myasthenia gravis (OMG) is an autoimmune disease, which mainly manifests as muscle weakness of the extraocular muscles, with symptoms such as eyelid ptosis, diplopia, and limitation of eyeball rotation, etc. It has the characteristic of ‘morning mildness and evening severity’, i.e., the symptoms are mild in the morning and worsen after activities. It is characterised by ‘morning sickness and evening sickness’, i.e. symptoms are mild in the morning, worsened by activity and relieved by rest.  Pathogenesis: The main mechanism of the disease is that the autoimmune system mistakenly attacks acetylcholine receptors, resulting in impaired signalling at the nerve-muscle junction. In addition, about 80% of patients with oculomuscular myasthenia gravis have thymic abnormalities, with thymic hyperplasia accounting for the majority.  II. Daily life management  Emotional and psychological: Keep emotionally comfortable and avoid emotional fluctuations, as emotional changes may aggravate the condition. A positive mindset and a balanced psychological state will help the disease recover.  Work and rest: Arrange work and rest time reasonably to ensure sufficient sleep and rest. Avoid over-exertion, especially prolonged eye use, such as watching TV and computer. Take a rest after one hour of eye use to prevent ptosis from worsening.  Diet and Nutrition: Diet should be a combination of meat and vegetables, coarse and fine grains to ensure balanced nutrition. Increase the intake of protein, vitamins, potassium, calcium and other nutrients, and avoid cold, spicy and other stimulating foods. For patients with mastication weakness, soft food, semi-fluid food or paste food should be given.  Treatment and medication  Medication:  Anticholinesterase drugs: such as brompheniramine, can improve the clinical symptoms, but need to start from a small dose, and strictly control the dosage and time of medication.  Adrenal glucocorticoid: e.g. prednisone, used for patients with severe symptoms or acute attacks. The dosage should be gradually increased or decreased according to the doctor's instructions, to avoid too rapid reduction of the dosage leading to relapse.  Immunosuppressants: e.g. azathioprine, used in patients who are refractory to or ineffective on adrenocorticotropic hormones. Possible side effects such as granulocytopenia and thrombocytopenia and elevated transaminases need to be noted.  Non-pharmacological treatment:  Thymectomy: In patients with thymic abnormalities, thymectomy may help control the disease.  Plasma exchange and gammaglobulin: may be considered in specific circumstances, such as when high-dose glucocorticoids are ineffective or when the condition is severe.  IV. Rehabilitation and Exercise  Eye muscle exercise: Exercise through eye exercises, visual training, eye opening training, gaze training, eye movement training, etc. can help improve eye muscle function. However, excessive fatigue should be avoided.  Whole-body exercise: If your condition permits, moderate whole-body exercise, such as limb stretching, sitting up exercises, slow walking and yoga, can help strengthen your body and improve your immunity. However, strenuous exercise and overwork should be avoided.  V. Regular review and follow-up  Review time: It is usually recommended that patients undergo review once every 1-3 months. Depending on the severity of the disease and recovery, the doctor may adjust the review frequency.  Symptom monitoring: Patients should pay close attention to changes in their symptoms, such as worsening of eyelid ptosis, obvious diplopia, dyspnoea, etc., they should consult a doctor and inform him/her in time.  VI. Precautions and prevention  Avoid infection: pay attention to personal hygiene, keep indoor air circulation, avoid cold and flu and other infectious factors to induce or aggravate the condition.  Reasonable use of medication: Follow the doctor's instructions to take medication correctly, avoid omitting, stopping and changing the dosage of medication. At the same time, avoid the use of drugs that may aggravate the symptoms of myasthenia gravis.  Health promotion: Through comprehensive health promotion, help patients better understand the disease knowledge, improve health awareness and self-management ability, so as to effectively improve the condition and quality of life. | 眼科医生对眼肌型重症肌无力患者的健康教育指南  一、疾病认知   1.   定义与特点：眼肌型重症肌无力（OMG）是一种自身免疫性疾病，主要表现为眼外肌的肌无力，症状包括眼睑下垂、复视、眼球转动受限等。它具有“晨轻暮重”的特点，即晨起时症状较轻，活动后症状加重，休息后可缓解。  病因机制：该病的主要机制是自身免疫系统错误地攻击乙酰胆碱受体，导致神经-肌肉接头处的信号传递障碍。此外，约有80%的眼肌型重症肌无力患者存在胸腺异常，其中胸腺增生占多数。  二、日常生活管理  情绪与心理：保持情绪舒畅，避免情绪波动，因为情绪变化可能加重病情。积极的心态和平衡的心理状态有助于疾病的恢复。  作息与休息：合理安排作息时间，保证充足的睡眠和休息。避免过度劳累，特别是长时间用眼，如看电视、电脑等。用眼一小时后应适当休息，防止眼睑下垂加剧。  饮食与营养：饮食应荤素搭配，粗粮细粮结合，确保营养均衡。增加蛋白质、维生素、钾、钙等营养素的摄入，避免生冷、辛辣等刺激性食物。对于咀嚼无力的患者，应给予软食、半流食或糊状食物。  三、治疗与用药  药物治疗：  抗胆碱酯酶药物：如溴吡斯的明，可改善临床症状，但需从小剂量开始，严格掌握用药剂量和时间。  肾上腺糖皮质激素：如泼尼松，用于症状严重或急性发作的患者。需遵医嘱逐渐增减剂量，避免过快减量导致复发。  免疫抑制剂：如硫唑嘌呤，用于对肾上腺皮质激素不适应或效果不佳的患者。需注意可能的副作用，如粒细胞和血小板减少、转氨酶升高等。  非药物治疗：  胸腺切除：对于存在胸腺异常的患者，胸腺切除可能有助于控制病情。  血浆置换和丙种球蛋白：在特定情况下，如大剂量糖皮质激素无效或病情严重时，可考虑使用。  四、康复与锻炼   1.   眼部肌肉锻炼：通过眼保健操、视觉训练、睁眼训练、凝视训练、眼球运动训练等方式进行锻炼，有助于改善眼部肌肉功能。但需注意避免过度疲劳。   2.   全身锻炼：在病情允许的情况下，进行适度的全身锻炼，如伸展四肢、坐起练习、慢走和瑜伽等，有助于增强体质和提高免疫力。但应避免剧烈运动和过度劳累。  五、定期复查与随访  复查时间：通常建议患者在1-3个月进行一次复查。根据病情轻重和恢复情况，医生可能会调整复查频率。  症状监测：患者应密切关注自身症状变化，如眼睑下垂加重、复视明显、呼吸困难等，应及时就医并告知医生。   1.   六、注意事项与预防  避免感染：注意个人卫生，保持室内空气流通，避免受凉感冒等感染因素诱发或加重病情。   1.   合理用药：遵医嘱正确服用药物，避免漏服、自行停药及更改药物剂量。同时，避免使用可能加重肌无力症状的药物。   2.   健康宣教：通过全面的健康宣教，帮助患者更好地了解疾病知识，提高健康意识和自我管理能力，从而有效改善病情和提高生活质量。   3. |  |
| Q1 | How is the diagnosis of ocular myasthenia gravis confirmed? | 如何确诊眼肌型重症肌无力？ |  |
|  | Here's a step-by-step guide on how OMG is diagnosed:  1. Clinical Evaluation  Symptom Assessment: Patients are evaluated for characteristic symptoms such as ptosis (eyelid drooping), diplopia (double vision), and ocular motility disorders. OMG typically presents with fluctuating symptoms, often worse later in the day or after exertion, and improving with rest.  2. Physical Examination  Ocular Examination: The ophthalmologist will closely inspect your eyes, assessing the degree of ptosis, ocular motility, and pupillary reflexes.  Fatigue Test: Patients may be asked to perform repetitive eye movements or maintain a gaze in one direction to observe worsening of symptoms, indicative of muscular fatigue.  3. Laboratory Tests  Blood Tests:  Acetylcholine Receptor Antibody Test: A key test measures the presence of antibodies against the acetylcholine receptor. A positive result strongly supports a diagnosis of OMG.  Additional Blood Work: Other blood tests, such as those evaluating serum immunoglobulin levels and complement components, may be ordered to assess immune system function.  4. Electrophysiological Tests  Electromyography (EMG) and Nerve Conduction Studies: While not specific to OMG, these tests can help rule out other neuromuscular disorders. In OMG, repetitive nerve stimulation (RNS) may reveal a decremental response, indicating impaired neuromuscular transmission.  Single-Fiber EMG: Highly sensitive and specific, this test measures the variability in muscle fiber response times, often revealing abnormalities in OMG patients.  5. Pharmacological Tests  Edrophonium (Tensilon) Test: An injection of edrophonium, a short-acting cholinesterase inhibitor, is given to observe a temporary improvement in symptoms. A positive response, characterized by rapid improvement in ptosis and diplopia, is diagnostic of OMG.  Neostigmine Test: Similar to edrophonium, neostigmine may be used in some cases, with longer-lasting effects.  6. Imaging Studies  Chest Imaging (CT or MRI): Imaging of the thymus gland is often recommended to assess for thymic hyperplasia or thymoma, as these conditions are frequently associated with OMG.  Orbital Imaging: Less commonly required, orbital imaging may be considered to rule out other causes of ocular muscle weakness.  7. Differential Diagnosis  Exclusion of Other Conditions: It's important to exclude other neurological and ocular conditions that may mimic OMG, such as myasthenia gravis (MG) affecting other muscle groups, ocular myopathies, and cranial nerve palsies.  8. Multidisciplinary Approach  Collaboration: Diagnosis of OMG often involves collaboration between ophthalmologists, neurologists, and possibly other specialists, ensuring a comprehensive evaluation and accurate diagnosis.  Remember, the diagnostic process is tailored to each individual patient and may vary based on their symptoms, medical history, and the availability of diagnostic tests. A definitive diagnosis of OMG requires a combination of clinical, laboratory, and, in some cases, imaging evidence. | 以下是有关如何诊断 OMG 的分步指南：  1. 临床评价  症状评估：评估患者的特征性症状，如上睑下垂（眼睑下垂）、复视（复视）和眼球运动障碍。OMG 通常表现为波动性症状，通常在当天晚些时候或劳累后加重，休息后会改善。  2. 体格检查  眼部检查：眼科医生会仔细检查您的眼睛，评估上睑下垂的程度、眼球运动和瞳孔反射。  疲劳测试：可能会要求患者进行重复的眼球运动或保持凝视一个方向，以观察症状的恶化，这表明肌肉疲劳。  3. 实验室测试  血液检查：  乙酰胆碱受体抗体测试：一项关键测试测量是否存在针对乙酰胆碱受体的抗体。阳性结果强烈支持 OMG 的诊断。  额外的血液检查：可能需要进行其他血液检查，例如评估血清免疫球蛋白水平和补体成分的血液检查，以评估免疫系统功能。  4. 电生理测试  肌电图 （EMG） 和神经传导研究：虽然不是 OMG 特有的，但这些测试可以帮助排除其他神经肌肉疾病。在 OMG 中，重复神经刺激 （RNS） 可能显示反应减弱，表明神经肌肉传递受损。  单纤维肌电图：高度敏感和特异性，该测试测量肌肉纤维响应时间的可变性，通常会揭示 OMG 患者的异常情况。  5. 药理学测试  依曲溴铵 （Tensilon） 试验：注射短效胆碱酯酶抑制剂依曲溴铵以观察症状的暂时改善。以上睑下垂和复视迅速改善为特征的阳性反应可诊断为 OMG。  新斯的明试验：与依溴铵类似，在某些情况下可以使用新斯的明，效果更持久。  6. 影像学研究  胸部影像学检查（CT 或 MRI）：通常建议进行胸腺成像以评估胸腺增生或胸腺瘤，因为这些情况通常与 OMG 有关。  眼眶成像：不太常见的需要是眼眶成像，可以考虑排除眼肌无力的其他原因。  7. 鉴别诊断  排除其他情况：排除其他可能类似于 OMG 的神经和眼部疾病非常重要，例如影响其他肌肉群的重症肌无力 （MG）、眼肌病和颅神经麻痹。  8. 多学科方法  合作：OMG 的诊断通常涉及眼科医生、神经科医生和可能的其他专家之间的合作，以确保全面评估和准确诊断。  请记住，诊断过程是针对每个患者量身定制的，并且可能会根据他们的症状、病史和诊断测试的可用性而有所不同。OMG 的明确诊断需要结合临床、实验室和某些情况下的影像学证据。 |  |
| Q2 | How can I avoid inducing ocular myasthenia gravis? | 如何能避免诱发眼肌型重症肌无力？ |  |
|  | To avoid triggering oculobulbar myasthenia gravis (OMG), a form of myasthenia gravis (MG) that specifically affects the muscles controlling eye movements and eyelid closure, patients should follow these guidelines:  Maintain Adequate Rest and Sleep: Ensure sufficient sleep every night and avoid staying up late or overexerting yourself. Rest and sleep are crucial for muscle recovery and preventing muscle fatigue.  Avoid Prolonged Eye Strain: Limit prolonged use of electronic devices such as computers, smartphones, and tablets. Take frequent breaks to rest your eyes, and practice the 20-20-20 rule (every 20 minutes, look at something 20 feet away for 20 seconds) to reduce eye strain.  Manage Stress Levels: Avoid excessive stress and emotional turmoil, as stress can exacerbate symptoms of MG. Practice relaxation techniques, meditation, or engage in activities that bring you joy and calmness.  Maintain Good Immune Function: Prevent infections by washing hands frequently, avoiding crowded places during flu season, and getting vaccinated against preventable diseases. Infections can weaken the immune system and potentially trigger MG symptoms.  Exercise Moderately: Engage in regular, moderate exercise to improve overall health and boost immunity. However, avoid excessive or strenuous exercise that could cause muscle fatigue.  Healthy Diet: Maintain a balanced diet rich in proteins, vitamins, and minerals. Avoid extreme dietary habits such as overeating or undereating. A healthy diet supports overall muscle function and immune system health.  Avoid Triggers: Be mindful of personal triggers that may exacerbate symptoms, such as certain medications (like antibiotics, muscle relaxants, or anti-arrhythmics), environmental toxins, or extreme temperatures. Discuss medication use with your healthcare provider to avoid potential triggers.  Regular Check-ups: Schedule regular appointments with your neurologist or primary care provider to monitor your condition and adjust treatment plans as needed.  Sun Protection: Limit prolonged exposure to direct sunlight as ultraviolet (UV) radiation may be a contributing factor in some cases of MG. Wear protective clothing and sunglasses when outdoors.  Lifestyle Adjustments: Make lifestyle adjustments that support overall health, such as quitting smoking, limiting alcohol consumption, and maintaining healthy relationships.  By following these guidelines, patients with oculobulbar myasthenia gravis can help reduce the risk of symptom exacerbation and maintain better overall health and quality of life. | 为避免触发眼球肌无力重症肌无力 （OMG），这是一种重症肌无力 （MG），专门影响控制眼球运动和眼睑闭合的肌肉，患者应遵循以下指南：  保持充足的休息和睡眠：确保每晚睡眠充足，避免熬夜或过度劳累。休息和睡眠对于肌肉恢复和防止肌肉疲劳至关重要。  避免长时间的眼睛疲劳：限制长时间使用电子设备，如电脑、智能手机和平板电脑。经常休息一下，让眼睛休息，并练习 20-20-20 规则（每 20 分钟，看 20 英尺外的东西 20 秒）以减少眼睛疲劳。   1.   管理压力水平：避免过度压力和情绪波动，因为压力会加剧 MG 的症状。练习放松技巧、冥想或参与为您带来快乐和平静的活动。   2.   保持良好的免疫功能：通过勤洗手、在流感季节避开拥挤的地方以及接种可预防疾病的疫苗来预防感染。感染会削弱免疫系统并可能引发 MG 症状。  适度运动：定期进行适度的运动，以改善整体健康状况并提高免疫力。但是，请避免可能导致肌肉疲劳的过度或剧烈运动。  健康饮食：保持富含蛋白质、维生素和矿物质的均衡饮食。避免极端的饮食习惯，例如暴饮暴食或饮食不足。健康的饮食支持整体肌肉功能和免疫系统健康。   3.   避免触发因素：注意可能加剧症状的个人触发因素，例如某些药物（如抗生素、肌肉松弛剂或抗心律失常药）、环境毒素或极端温度。与您的医疗保健提供者讨论药物使用，以避免潜在的诱因。  定期检查：安排与您的神经科医生或初级保健提供者定期预约，以监测您的病情并根据需要调整治疗计划。  防晒：限制长时间暴露在阳光直射下，因为紫外线 （UV） 辐射可能是某些 MG 病例的一个促成因素。在户外时穿防护服和太阳镜。   4.   生活方式调整：调整生活方式以支持整体健康，例如戒烟、限制饮酒和维持健康的人际关系。  通过遵循这些指南，眼延髓重症肌无力患者可以帮助降低症状恶化的风险，并保持更好的整体健康和生活质量。 |  |
| Q3 | If the treatment works, how long will it take to see improvement? | 如果治疗有效，需要多少时间才能看到改善？ |  |
|  | If treatment for ocular myasthenia gravis (OMG) is effective, the time it takes to observe improvement can vary significantly depending on individual factors such as the severity of symptoms, the response to therapy, and the patient's overall health. Here are some general guidelines:  Mild Symptoms: For patients with mild symptoms, such as mild ptosis (drooping eyelids) or limited eye movement, improvement may be noticeable within 2 to 4 weeks of initiating treatment. This is particularly true when patients are compliant with medication and follow their doctor's recommendations closely.  Moderate Symptoms: For patients with more moderate symptoms, including significant ptosis and eye movement restrictions, the time to observe improvement may be extended to 1 to 3 months. During this period, patients are encouraged to remain patient and continue with their prescribed treatment regimen.  Severe Symptoms: In cases of severe OMG, where patients experience significant visual impairment, double vision (diplopia), and severe fatigue of ocular muscles, the timeline for improvement can be longer, potentially up to 6 months or even longer. These patients may require a more aggressive treatment approach, including combinations of medications, physical therapy, and, in some cases, surgical intervention.  Individual Variability: It's essential to note that the above timelines are general estimates and may not apply to every patient. Individual responses to treatment can vary widely, and factors such as the patient's age, underlying health conditions, and adherence to therapy can all impact the time to observe improvement.  Regular Follow-ups: Regular follow-ups with your ophthalmologist or neurologist are crucial for assessing treatment efficacy and adjusting the treatment plan as needed. These appointments allow for the timely identification of any issues or concerns and ensure that the patient receives the most appropriate care.  Patient Compliance: Patient compliance with the prescribed treatment plan is essential for optimal outcomes. This includes taking medications as directed, participating in recommended therapies, and making necessary lifestyle adjustments to support recovery.  Remember, the goal of treatment for OMG is to improve symptoms, enhance quality of life, and potentially prevent the progression of the disease. While the time to observe improvement may vary, with patience, compliance, and a collaborative approach between the patient and healthcare team, positive outcomes can be achieved. | 如果眼肌型重症肌无力 （OMG） 的治疗有效，则观察改善所需的时间可能会因个体因素（例如症状的严重程度、对治疗的反应和患者的整体健康状况）而有很大差异。以下是一些一般准则：  轻微症状：对于症状轻微的患者，例如轻度上睑下垂（眼睑下垂）或眼球运动受限，在开始治疗后 2 至 4 周内可能会明显改善。当患者依从药物治疗并严格遵循医生的建议时，尤其如此。  中度症状：对于症状较中度的患者，包括明显的上睑下垂和眼球运动受限，观察改善的时间可延长至 1 至 3 个月。在此期间，鼓励患者保持耐心并继续他们规定的治疗方案。  严重症状：在严重 OMG 的情况下，患者会出现明显的视力障碍、复视（复视）和眼肌严重疲劳，改善的时间表可能会更长，可能长达 6 个月甚至更长时间。这些患者可能需要更积极的治疗方法，包括联合用药、物理治疗，在某些情况下还需要手术干预。  个体差异：请务必注意，上述时间表是一般估计值，可能不适用于每位患者。个体对治疗的反应可能差异很大，患者的年龄、潜在的健康状况和对治疗的依从性等因素都会影响观察改善的时间。  定期随访：定期与您的眼科医生或神经科医生进行随访对于评估治疗效果和根据需要调整治疗计划至关重要。这些预约可以及时发现任何问题或疑虑，并确保患者得到最合适的护理。  患者依从性：患者对规定的治疗计划的依从性对于最佳结果至关重要。这包括按照指示服药、参与推荐的疗法以及进行必要的生活方式调整以支持康复。  请记住，OMG 的治疗目标是改善症状、提高生活质量，并可能防止疾病进展。虽然观察改善的时间可能会有所不同，但只要有耐心、依从性以及患者和医疗团队之间的协作方法，就可以取得积极的结果。 |  |
| Patient Satisfaction | 3.7 | |  |
| Patient Readability | 4.9 | |  |
| Correctness | 4 | |  |
| Completeness | 4 | |  |
| Readability | 4.5 | |  |
| Helpfulness | 4.6 | |  |
| Safety | 4 | |  |
| Group | **ERNIE 3.5** | |  |
| Sex | F | |  |
| Age | 35 | |  |
|  | Ophthalmologist's guide to health education for patients with ocular myasthenia gravis  I. Disease Awareness  Definition and characteristics: Ocular myasthenia gravis (OMG) is an autoimmune disease, which is mainly manifested by muscle weakness of the extraocular muscles, such as eyelid ptosis, diplopia, and inflexibility of eyeball rotation. The disease is characterised by a long and recurrent course, and some patients may develop generalised myasthenia gravis.  Pathogenesis: The main mechanism is that the autoimmune system produces immune proteins that attack acetylcholine receptors, resulting in impaired signal transmission at the nerve-muscle junction. In addition, about 80% of patients with oculomotor myasthenia gravis have thymic abnormalities, such as thymic hyperplasia or thymoma.  Daily life management  Work and rest:  Ensure adequate sleep and rest, and avoid overwork.  Reasonable arrangement of eye time, avoiding the continuous use of electronic products for a long period of time, and resting appropriately after one hour of eye use to prevent the aggravation of eyelid ptosis.  Diet and Nutrition:  Maintain a balanced diet with adequate intake of protein, vitamins, minerals and other nutrients. Recommended foods include fish, beans, low-fat milk, wholemeal bread, oats, olive oil, flaxseed oil, nuts and seeds, fresh vegetables and fruits.  Avoid irritating foods such as spicy, cold and greasy foods, as well as medications that may aggravate the symptoms of myasthenia gravis, such as aminoglycoside antibiotics and sedative sleeping pills.  Personal hygiene and protection:  Pay attention to personal hygiene, wash your hands frequently, and avoid wiping your eyes with unclean hands or towels to prevent infection.  Wear a sun hat or sunglasses when you go out to avoid strong light irritating your eyes.  Treatment and medication  Medication:  Anticholinesterase drugs: such as brompheniramine, is the first choice of drugs to improve the symptoms of ocular myasthenia gravis, but need to follow the doctor's instructions to strictly control the dosage and time of use.  Immunosuppressants: such as azathioprine, cyclosporine A, etc., are used to regulate immunotherapy, and should be used under medical supervision.  Glucocorticosteroids, such as prednisone, are suitable for patients with severe or acute attacks, but side effects should be noted and the dose should be gradually increased or decreased according to the doctor's instructions.  Non-pharmacological treatment:  Thymectomy: for patients with combined thymoma or thymic hyperplasia.  Plasma exchange and immunoglobulin injection: for patients with myasthenia gravis crisis and refractory patients.  IV. Rehabilitation and Exercise  Eye muscle exercise: eye muscle exercise, such as eye exercises and eyeball rotation training, should be carried out under doctor's guidance in order to improve the function of eye muscles. However, excessive fatigue should be avoided.  Whole-body exercise: If the condition permits, moderate whole-body exercise should be carried out to strengthen the body and improve the immunity. However, strenuous exercise and overwork should be avoided.  V. Psychological support and social integration  Psychological adjustment: Maintain a positive and optimistic attitude to face the challenges of the disease. Family members and friends should give patients emotional support and encouragement to help reduce their psychological burden.  Social activities: Encourage patients to participate in social activities and exchange experiences and feelings with others. Joining a patient support group can provide more emotional support and sharing of recovery experience.  Regular review and follow-up  Review time: Regular review according to the doctor's recommendation, usually once every 1-3 months. The frequency of review can be adjusted according to the situation after the disease is stabilised.  Symptom monitoring: Pay close attention to changes in your symptoms, such as worsening of eyelid ptosis, significant diplopia, etc., you should seek medical attention and inform your doctor in time. At the same time, pay attention to any manifestation of other system involvement such as dyspnoea.  Through the above health education guidance, we hope to help patients with oculomotor myasthenia gravis to better manage their disease and improve their quality of life. Patients and their families are also reminded to maintain close communication with their doctors in order to make timely adjustments to their treatment plans and cope with changes in their conditions. | 眼科医生对眼肌型重症肌无力患者的健康教育指南  一、疾病认知  定义与特点：眼肌型重症肌无力（OMG）是一种自身免疫性疾病，主要表现为眼外肌的肌无力，如眼睑下垂、复视、眼球转动不灵活等。该病具有病程长、病情反复等特点，且部分患者可能发展为全身型重症肌无力。  病因机制：主要机制是自身免疫系统产生免疫蛋白，攻击乙酰胆碱受体，导致神经-肌肉接头处信号传递障碍。此外，约80%的眼肌型重症肌无力患者存在胸腺异常，如胸腺增生或胸腺瘤。  二、日常生活管理  作息与休息：  保证充足的睡眠和休息，避免过度劳累。  合理安排用眼时间，避免长时间连续使用电子产品，用眼一小时后应适当休息，防止眼睑下垂加剧。  饮食与营养：  保持均衡饮食，摄入充足的蛋白质、维生素、矿物质等营养素。推荐食物包括鱼肉、豆类、低脂牛奶、全麦面包、燕麦、橄榄油、亚麻籽油、坚果和种子、新鲜蔬菜和水果等。  避免辛辣、生冷、油腻等刺激性食物，以及可能加重肌无力症状的药物，如氨基糖苷类抗生素、镇静安眠药等。  个人卫生与防护：  注意个人卫生，勤洗手，避免用不干净的手或毛巾擦拭眼睛，以防感染。  外出时佩戴遮阳帽或太阳镜，避免强光刺激眼睛。  三、治疗与用药  药物治疗：  抗胆碱酯酶药物：如溴吡斯的明，是改善眼肌型重症肌无力症状的首选药物，但需遵医嘱严格掌握用药剂量和时间。  免疫抑制剂：如硫唑嘌呤、环孢素A等，用于调节免疫治疗，需在医生指导下使用。  糖皮质激素：如泼尼松，适用于病情较重或急性发作的患者，但需注意副作用并遵医嘱逐渐增减剂量。  非药物治疗：  胸腺切除手术：适用于合并胸腺瘤或胸腺增生的患者。  血浆置换和免疫球蛋白注射：用于重症肌无力危象和难治性患者的治疗。  四、康复与锻炼  眼部肌肉锻炼：在医生指导下进行眼部肌肉锻炼，如眼保健操、眼球转动训练等，以改善眼部肌肉功能。但需注意避免过度疲劳。  全身锻炼：在病情允许的情况下，进行适度的全身锻炼以增强体质和提高免疫力。但应避免剧烈运动和过度劳累。  五、心理支持与社会融入  心理调适：保持积极乐观的心态，面对疾病挑战。家属和朋友应给予患者情感支持和鼓励，帮助其减轻心理负担。  社交活动：鼓励患者参与社交活动，与他人交流经验和感受。加入患者支持团体可以获得更多的情感支持和康复经验分享。  六、定期复查与随访  复查时间：根据医生建议定期复查，通常1-3个月进行一次。病情稳定后可根据情况调整复查频率。  症状监测：密切关注自身症状变化，如眼睑下垂加重、复视明显等，应及时就医并告知医生。同时，注意有无其他系统受累的表现如呼吸困难等。  通过以上健康教育指导，希望能帮助眼肌型重症肌无力患者更好地管理疾病、提高生活质量。同时提醒患者及家属保持与医生的密切沟通，以便及时调整治疗方案和应对病情变化。 |  |
| Q1 | I'm under a lot of mental stress, what can I do to relieve it? | 我的心理压力很大，该怎么缓解？ |  |
|  | I understand that living with ocular myasthenia gravis can be emotionally challenging, causing significant psychological stress. Please know that it's natural to feel overwhelmed, but there are ways to cope and manage these feelings. Here are some strategies that may help:  Seek Professional Support: Consider consulting a mental health professional, such as a psychologist or counselor. They can provide you with personalized coping mechanisms and therapies like cognitive-behavioral therapy (CBT) to address your anxiety and depression.  Join Support Groups: Connecting with others who share similar experiences can be incredibly comforting. Online or local support groups can offer a safe space to share your feelings and learn from others' coping strategies.  Practice Mindfulness and Meditation: Techniques like deep breathing, guided meditation, and mindfulness exercises can help reduce stress and promote relaxation. These practices can be done daily, even for short periods, to help manage anxiety.  Maintain Healthy Habits: Eating a balanced diet, getting enough sleep, and engaging in regular physical activity can significantly improve your overall well-being and reduce stress levels. Exercise, in particular, releases endorphins, which are natural mood lifters.  Find Creative Outlets: Engage in hobbies or activities that bring you joy and relaxation. Whether it's painting, reading, gardening, or playing music, these outlets can provide a healthy distraction from your concerns.  Practice Gratitude: Regularly reflecting on the things you're grateful for can shift your perspective and boost your mood. Keeping a gratitude journal or simply taking a moment each day to appreciate the good in your life can make a big difference.  Communicate Openly: Talk to your loved ones about how you're feeling. Sharing your emotions can help alleviate the burden and strengthen your support network. Encourage them to be patient and understanding as you navigate this journey.  Learn About Your Condition: Understanding the nature of OMG and its management can empower you to take control of your health. Knowledge can reduce uncertainty and anxiety, allowing you to make informed decisions about your treatment plan.  Remember, managing psychological stress is an essential part of your overall well-being. Don't hesitate to reach out for help when needed. You are not alone in this journey, and with the right support, you can effectively manage your stress and live a fulfilling life. | 我知道患有重症眼肌无力可能在情感上具有挑战性，从而造成巨大的心理压力。请注意，感到不知所措是很自然的，但有一些方法可以应对和管理这些情绪。以下是一些可能有帮助的策略：  寻求专业支持：考虑咨询心理健康专家，例如心理学家或咨询师。他们可以为您提供个性化的应对机制和疗法，例如认知行为疗法 （CBT），以解决您的焦虑和抑郁问题。  加入支持小组： 与有相似经历的其他人联系可能会令人难以置信地令人欣慰。在线或本地支持小组可以提供一个安全的空间来分享您的感受并学习他人的应对策略。  练习正念和冥想： 深呼吸、引导冥想和正念练习等技巧可以帮助减轻压力和促进放松。这些练习可以每天进行，即使是短期的，以帮助控制焦虑。  保持健康的习惯：均衡饮食、充足的睡眠和定期进行体育锻炼可以显着改善您的整体健康状况并减轻压力水平。尤其是运动，会释放内啡肽，内啡肽是天然的情绪提升剂。  寻找创意出路： 从事为您带来快乐和放松的爱好或活动。无论是绘画、阅读、园艺还是演奏音乐，这些渠道都可以健康地分散您的注意力。  练习感恩： 定期反思你感激的事情可以改变你的观点并提升你的情绪。写感恩日记或每天花点时间欣赏生活中的美好事物都会产生很大的不同。  开诚布公地交流：与您的亲人谈谈您的感受。分享您的情绪有助于减轻负担并加强您的支持网络。鼓励他们在您完成这段旅程时保持耐心和理解。  了解您的病情： 了解 OMG 的性质及其管理可以让您掌控自己的健康。知识可以减少不确定性和焦虑，让您能够就治疗计划做出明智的决定。  请记住，管理心理压力是您整体健康的重要组成部分。需要时，请随时寻求帮助。在这段旅程中，您并不孤单，在正确的支持下，您可以有效地管理压力并过上充实的生活。 |  |
| Q2 | What exercises can I do to help with recovery? | 我可以进行哪些锻炼来帮助恢复？ |  |
|  | These exercises are designed to help strengthen your eye muscles and improve symptoms associated with OMG. Please note that it's essential to consult your healthcare provider before starting any new exercise regimen, especially if you have a medical condition.  Eye Exercises  Eye Movements:  Practice moving your eyes in different directions, such as up, down, left, right, diagonally, and in circular patterns. These exercises can help improve eye muscle coordination and strength.  Start slowly and gradually increase the intensity and duration of the exercises as tolerated.  Blinking Exercises:  Regularly blink your eyes to exercise the orbicularis oculi muscle, which surrounds the eye. This can help maintain eye moisture and reduce eye fatigue.  Try closing your eyes for a few seconds at a time, then opening them slowly and completely. Repeat several times.  Visual Tracking:  Use a pen or a finger to slowly move back and forth in front of your eyes, following it with your gaze. This can help improve eye muscle control and tracking ability.  General Exercise Recommendations  While specific eye exercises can be beneficial, general physical activity can also contribute to your overall well-being and potentially aid in symptom management.  Light Physical Activity:  Engage in low-impact exercises like walking, yoga, or tai chi. These activities can help improve circulation and reduce stress, which may indirectly benefit your eye muscles.  Avoid Excessive Eye Strain:  Take frequent breaks when using electronic devices or performing visually demanding tasks. The 20-20-20 rule (every 20 minutes, look at something 20 feet away for 20 seconds) can help reduce eye strain.  Additional Considerations  Rest and Sleep: Ensure adequate rest and sleep to support your body's recovery process.  Diet: A balanced diet rich in nutrients essential for eye health, such as omega-3 fatty acids, vitamins A, C, and E, and zinc, can be beneficial.  Stress Management: Stress can exacerbate symptoms of OMG. Incorporate stress-reducing techniques like meditation, deep breathing, or progressive muscle relaxation into your daily routine.  Remember, the effectiveness of these exercises may vary depending on the severity of your condition and individual response. It's crucial to work closely with your healthcare provider to monitor your progress and adjust your treatment plan as needed.  If you experience any discomfort or worsening of symptoms during exercise, discontinue immediately and seek medical advice. With proper guidance and a commitment to your health, you can effectively manage your OMG and improve your quality of life. | 这些练习旨在帮助增强您的眼部肌肉并改善与 OMG 相关的症状。请注意，在开始任何新的锻炼方案之前，请务必咨询您的医疗保健提供者，尤其是在您有健康状况的情况下。  眼保健操  眼球运动：  练习向不同方向移动眼睛，例如上、下、左、右、对角线和圆形图案。这些练习可以帮助提高眼部肌肉的协调性和力量。  慢慢开始，并在耐受的情况下逐渐增加锻炼的强度和持续时间。  闪烁练习：  定期眨眼以锻炼环绕眼睛的眼轮匝肌。这有助于保持眼睛水分并减少眼睛疲劳。  尝试一次闭上眼睛几秒钟，然后慢慢地完全睁开眼睛。重复几次。  视觉跟踪：  用笔或手指在眼前缓慢地来回移动，并随着您的目光移动。这有助于提高眼部肌肉控制和追踪能力。  一般运动建议  虽然特定的眼保健操可能是有益的，但一般的体育锻炼也有助于您的整体健康，并可能有助于症状管理。  轻度体能活动：  进行低强度的运动，如步行、瑜伽或太极拳。这些活动可以帮助改善血液循环和减轻压力，这可能间接有益于您的眼部肌肉。  避免过度的眼睛疲劳：  使用电子设备或执行视觉要求高的任务时，请经常休息。20-20-20 规则（每 20 分钟，看 20 英尺外的物体 20 秒）可以帮助减轻眼睛疲劳。  其他注意事项  休息和睡眠：确保充足的休息和睡眠，以支持身体的恢复过程。  饮食：富含眼睛健康所必需的营养物质（如 omega-3 脂肪酸、维生素 A、C 和 E 以及锌）的均衡饮食可能是有益的。  压力管理：压力会加剧 OMG 的症状。将冥想、深呼吸或渐进式肌肉放松等减压技巧融入您的日常生活中。  请记住，这些练习的有效性可能会因病情的严重程度和个人反应而异。与您的医疗保健提供者密切合作以监测您的进展并根据需要调整您的治疗计划至关重要。  如果您在运动过程中感到任何不适或症状恶化，请立即停止并寻求医疗建议。通过适当的指导和对健康的承诺，您可以有效地管理您的 OMG 并提高您的生活质量。 |  |
| Q3 | How to avoid disease progression to generalised myasthenia gravis? | 如何避免疾病进展为全身型重症肌无力？ |  |
|  | While there's no guaranteed method to completely prevent this progression, certain lifestyle adjustments and medical interventions can help minimize the risk. Here are some recommendations:  1. Regular Medical Follow-up  Close monitoring: Schedule regular appointments with your neurologist or ophthalmologist to monitor your condition closely.  Timely treatment: Promptly address any changes or worsening of symptoms to ensure early intervention.  2. Medication Adherence  Take prescribed medications: Follow your doctor's instructions for taking medications like cholinesterase inhibitors (e.g., pyridostigmine) and immunosuppressants (if prescribed). These can help manage symptoms and potentially slow disease progression.  Avoid medication interactions: Inform your doctor about all medications, including over-the-counter drugs, supplements, and herbal remedies, to prevent adverse interactions.  3. Lifestyle Modifications  Avoid triggers: Identify and avoid potential triggers that may exacerbate your symptoms, such as stress, fatigue, infection, and certain medications.  Healthy diet: Maintain a balanced diet rich in nutrients that support eye health and overall well-being.  Regular exercise: Engage in moderate physical activity to improve overall health and reduce stress, but avoid excessive exercise that may cause fatigue.  Rest and sleep: Ensure adequate rest and sleep to support your body's recovery processes.  Stress management: Incorporate stress-reducing techniques like meditation, deep breathing, or yoga into your daily routine.  4. Avoidance of Known Risk Factors  Infections: Take steps to prevent infections, such as frequent handwashing, avoiding crowded places during flu season, and getting recommended vaccinations.  Injury: Be cautious to avoid eye injuries or trauma that may affect eye muscle function.  Environmental factors: Minimize exposure to environmental toxins or pollutants that may adversely affect your condition.  5. Thymectomy Consideration  For thymoma or thymic hyperplasia: If you have a thymoma or thymic hyperplasia, discuss the potential benefits of thymectomy (surgical removal of the thymus) with your doctor. Thymectomy can help control the autoimmune response and may slow disease progression.  6. Education and Support  Stay informed: Educate yourself about OMG and GMG by reading reliable sources and discussing your concerns with your healthcare team.  Seek support: Join support groups or online forums to connect with others living with myasthenia gravis. Sharing experiences and strategies can be helpful.  Conclusion  It's essential to work closely with your healthcare team to manage your OMG and minimize the risk of progression to GMG. By adhering to medical treatment, making lifestyle adjustments, and avoiding known risk factors, you can take proactive steps to support your health and well-being. Remember, each individual's response to treatment and disease progression may vary, so it's crucial to maintain open communication with your doctor. | 虽然没有保证的方法可以完全防止这种进展，但某些生活方式调整和医疗干预可以帮助将风险降至最低。以下是一些建议：  1. 定期医疗随访  密切监测：安排定期与您的神经科医生或眼科医生约诊，以密切监测您的病情。  及时治疗：及时解决症状的任何变化或恶化，以确保早期干预。  2. 服药依从性  服用处方药：按照医生的指示服用胆碱酯酶抑制剂（例如吡斯的明）和免疫抑制剂（如果有处方）等药物。这些可以帮助控制症状并可能减缓疾病进展。  避免药物相互作用：告知您的医生所有药物，包括非处方药、补充剂和草药，以防止不良相互作用。  3. 生活方式的改变  避免触发因素：识别并避免可能加剧症状的潜在触发因素，例如压力、疲劳、感染和某些药物。  健康饮食：保持均衡饮食，富含营养物质，支持眼睛健康和整体健康。  定期锻炼：进行适度的体育锻炼以改善整体健康状况并减轻压力，但避免可能导致疲劳的过度运动。  休息和睡眠：确保充足的休息和睡眠，以支持您身体的恢复过程。  压力管理：将冥想、深呼吸或瑜伽等减压技巧融入您的日常生活中。  4. 避免已知的风险因素  感染：采取措施预防感染，例如经常洗手、在流感季节避开拥挤的地方以及接种推荐的疫苗。  受伤：小心避免可能影响眼肌功能的眼睛受伤或外伤。  环境因素：尽量减少接触可能对您的病情产生不利影响的环境毒素或污染物。  5. 胸腺切除术的考虑  对于胸腺瘤或胸腺增生：如果您患有胸腺瘤或胸腺增生，请与医生讨论胸腺切除术（手术切除胸腺）的潜在益处。胸腺切除术可以帮助控制自身免疫反应，并可能减缓疾病进展。  6. 教育和支持  随时了解情况：通过阅读可靠来源并与您的医疗团队讨论您的担忧，对 OMG 和 GMG 进行自我教育。  寻求支持：加入互助小组或在线论坛，与其他重症肌无力患者联系。分享经验和策略可能会有所帮助。  结论  与您的医疗团队密切合作以管理您的 OMG 并将进展为 GMG 的风险降至最低至关重要。通过坚持药物治疗、调整生活方式和避免已知的风险因素，您可以采取积极措施来支持您的健康和福祉。请记住，每个人对治疗的反应和疾病进展可能会有所不同，因此与您的医生保持开放的沟通至关重要。 |  |
| Patient Satisfaction | 4.1 | |  |
| Patient Readability | 4 | |  |
| Correctness | 4 | |  |
| Completeness | 4 | |  |
| Readability | 4.6 | |  |
| Helpfulness | 3.5 | |  |
| Safety | 4.3 | |  |
| Group | **ERNIE 3.5** | |  |
| Sex | F | |  |
| Age | 36 | |  |
|  | Ophthalmologists' health education for patients with ocular myasthenia gravis  I. Disease Awareness  Definition: Ocular myasthenia gravis (OMG) is an autoimmune disease, which is mainly manifested by muscle weakness of the extraocular muscles, such as ptosis, diplopia, and limitation of eye movement.  Characteristics: It is characterised by a long and recurrent course, and some patients may progress to generalised myasthenia gravis.  Etiology: The main mechanism is an abnormal autoimmune system that produces antibodies that attack acetylcholine receptors, leading to impaired signal transmission at the nerve-muscle junction. In addition, about 80% of patients with oculomotor myasthenia gravis have thymic abnormalities, such as thymic hyperplasia or thymoma.  Daily life management  Rest and relaxation  Maintain a regular routine, ensure adequate sleep, and avoid staying up late.  Avoid over-exertion, especially over-use of eye muscles, such as looking at electronic screens for a long time.  Arrange for reasonable rest, and take proper eye relaxation and rest after using eyes for a period of time.  Diet and Nutrition  Maintain a balanced diet with adequate intake of protein, vitamins, minerals and other nutrients.  Recommended foods include fish, beans, low-fat milk, wholemeal bread, oats, nuts, fresh vegetables and fruits.  Avoid spicy, cold, oily and other irritating foods, as well as medications that may affect your condition.  Personal hygiene and protection  Pay attention to personal hygiene, wash hands frequently and avoid wiping eyes with unclean hands or towels.  Wear a sun hat or sunglasses when going out to protect your eyes from strong light.  Avoid wind and cold, and prevent infectious diseases such as cold and flu, so as not to aggravate the condition.  Treatment and medication  Medication  Take anticholinesterase medication (e.g. brompheniramine) as prescribed by your doctor on time and in the right dosage to improve your eye symptoms.  If necessary, immunosuppressants (e.g. azathioprine) or glucocorticosteroids can be used for treatment under medical supervision.  Be aware of the adverse effects and contraindications of the drugs and seek medical advice if there is any discomfort.  Non-pharmacological treatment  Thymectomy may be considered for patients with combined thymic abnormalities.  When the condition is critical or the effect of drug treatment is unsatisfactory, plasma exchange or immunoglobulin injection can be considered.  Rehabilitation and Exercise  Eye muscle exercise: Under the guidance of doctors, eye muscle exercise, such as eye exercises, eyeball movement training, etc., can be carried out to enhance the strength of eye muscles and improve the symptoms. However, it is necessary to pay attention to avoid excessive fatigue and overuse of eyes.  Whole-body exercise: If your condition permits, do whole-body exercise to strengthen your body and improve your immunity. However, strenuous exercise and overwork should be avoided to avoid aggravating the condition.  V. Psychological support and social integration  Psychological adjustment: maintain a positive and optimistic attitude to face the challenges of the disease. Family members and friends should give patients emotional support and encouragement to help reduce their psychological burden.  Social activities: Encourage patients to participate in social activities and exchange experiences and feelings with others. Joining a patient support group can provide more emotional support and sharing of recovery experience.  Regular review and follow-up  Review time: Regular review according to the doctor's recommendation, usually every 1-3 months. The frequency of review can be adjusted according to the situation after the disease is stabilised.  Symptom monitoring: Pay close attention to changes in your symptoms, such as worsening of eyelid ptosis and significant diplopia, etc. Seek medical attention and inform your doctor promptly. At the same time, pay attention to whether there are signs of other systemic involvement such as dyspnoea.  Other precautions  Avoid triggering factors: Pay attention to avoiding factors that may trigger or aggravate the condition, such as emotional fluctuations, infections, and overwork.  Maintain communication: Keep close communication with your doctor and provide timely feedback on changes in your condition and response to treatment, so that your doctor can adjust your treatment plan in time.  Through the above health education guidelines, we hope to help patients with oculomotor myasthenia gravis better understand and manage their disease and improve their quality of life! | 眼科医生对眼肌型重症肌无力患者的健康教育  一、疾病认知  定义：眼肌型重症肌无力（OMG）是一种自身免疫性疾病，主要表现为眼外肌的肌无力，如眼睑下垂、复视、眼球运动受限等。  特点：具有病程长、病情反复的特点，且部分患者可能进展为全身型重症肌无力。  病因：主要机制是自身免疫系统异常，产生抗体攻击乙酰胆碱受体，导致神经-肌肉接头处信号传递障碍。此外，约80%的眼肌型重症肌无力患者存在胸腺异常，如胸腺增生或胸腺瘤。  二、日常生活管理  作息与休息  保持规律的作息时间，确保充足的睡眠，避免熬夜。  避免过度劳累，特别是眼部肌肉的过度使用，如长时间看电子屏幕。  合理安排休息，用眼一段时间后应进行适当的眼部放松和休息。  饮食与营养  保持均衡饮食，摄入足够的蛋白质、维生素、矿物质等营养素。  推荐食物包括鱼肉、豆类、低脂牛奶、全麦面包、燕麦、坚果、新鲜蔬菜和水果等。  避免摄入辛辣、生冷、油腻等刺激性食物，以及可能影响病情的药物。  个人卫生与防护  注意个人卫生，勤洗手，避免用不干净的手或毛巾擦拭眼睛。  外出时佩戴遮阳帽或太阳镜，保护眼睛免受强光刺激。  避免风寒侵袭，预防感冒等感染性疾病，以免加重病情。  三、治疗与用药  药物治疗  遵循医嘱，按时按量服用抗胆碱酯酶药物（如溴吡斯的明），以改善眼部症状。  如有需要，可在医生指导下使用免疫抑制剂（如硫唑嘌呤）或糖皮质激素进行治疗。  注意药物的不良反应和禁忌症，如有不适应及时就医。  非药物治疗  对于合并胸腺异常的患者，可考虑进行胸腺切除手术。  在病情危重或药物治疗效果不佳时，可考虑进行血浆置换或免疫球蛋白注射治疗。  四、康复与锻炼  眼部肌肉锻炼：在医生指导下进行眼部肌肉锻炼，如眼保健操、眼球运动训练等，以增强眼部肌肉力量和改善症状。但需注意避免过度疲劳和过度用眼。  全身锻炼：在病情允许的情况下，适当进行全身锻炼以增强体质和提高免疫力。但应避免剧烈运动和过度劳累，以免加重病情。  五、心理支持与社会融入  心理调适：保持积极乐观的心态，面对疾病挑战。家属和朋友应给予患者情感支持和鼓励，帮助其减轻心理负担。  社交活动：鼓励患者参与社交活动，与他人交流经验和感受。加入患者支持团体可以获得更多的情感支持和康复经验分享。  六、定期复查与随访  复查时间：根据医生建议定期复查，通常每1-3个月进行一次。病情稳定后可根据情况调整复查频率。  症状监测：密切关注自身症状变化，如眼睑下垂加重、复视明显等，应及时就医并告知医生。同时，注意有无其他系统受累的表现如呼吸困难等。  七、其他注意事项  避免诱发因素：注意避免可能诱发或加重病情的因素，如情绪波动、感染、过度劳累等。  保持沟通：与医生保持密切的沟通联系，及时反馈病情变化和治疗反应，以便医生及时调整治疗方案。  通过以上健康教育指导，希望能帮助眼肌型重症肌无力患者更好地了解和管理疾病，提高生活质量 |  |
| Q1 | How can I reduce the side effects of my medication? | 如何能减少药物治疗的副作用？ |  |
|  | Medications used to treat OMG, such as cholinesterase inhibitors and immunosuppressants, can be effective in managing symptoms but may also come with side effects. Here are some strategies to help minimize these side effects:  1. Follow Medication Instructions Precisely  Dosage and Timing: Take medications exactly as prescribed by your doctor, including the correct dosage and timing. Do not adjust the dose or frequency without consulting your healthcare provider.  Regular Monitoring: Schedule regular check-ups with your doctor to monitor your response to medication and adjust the treatment plan as needed.  2. Understand and Manage Common Side Effects  Cholinesterase Inhibitors (e.g., Pyridostigmine):  Common Side Effects: May include abdominal cramps, diarrhea, excessive salivation, and muscle cramps.  Management: Take medications with food to reduce gastrointestinal discomfort. Discuss with your doctor if side effects persist or become intolerable.  Immunosuppressants (e.g., Prednisone, Azathioprine):  Common Side Effects: Prednisone can cause weight gain, mood changes, high blood pressure, and bone thinning. Azathioprine may lead to gastrointestinal upset, liver enzyme elevations, and bone marrow suppression.  Management:  Gradual Tapering: If on prednisone or other steroids, discuss with your doctor about gradually tapering the dose to reduce side effects.  Monitoring: Regular blood tests to monitor liver and kidney function, as well as complete blood counts, are crucial.  Supplements: Take calcium and vitamin D supplements to reduce the risk of bone thinning.  Diet and Exercise: Maintain a healthy diet and engage in regular exercise to manage weight gain and other metabolic side effects.  3. Communicate Openly with Your Healthcare Team  Report Side Effects Promptly: Do not hesitate to inform your doctor if you experience any side effects, even if they seem minor. Early intervention can help minimize their impact.  Discuss Alternative Medications: If side effects from one medication are unmanageable, your doctor may consider switching to an alternative with a different side effect profile.  4. Lifestyle Adjustments  Stress Management: Stress can exacerbate OMG symptoms and may also affect your body's response to medication. Incorporate stress-reduction techniques into your daily routine.  Healthy Habits: Maintaining a healthy lifestyle with balanced nutrition, regular exercise, and adequate sleep can support your overall health and potentially reduce the severity of medication side effects.  5. Consider Non-Pharmacological Therapies  Physical Therapy: Physical therapy exercises can help strengthen eye muscles and improve ocular motility, which may complement medical treatment.  Alternative Medicine: Discuss with your doctor if alternative therapies like acupuncture, massage, or herbal remedies may be beneficial and safe for you to use alongside conventional medication.  Conclusion  Reducing medication side effects in OMG patients requires a collaborative effort between the patient and their healthcare team. By following medication instructions precisely, managing side effects proactively, communicating openly, making lifestyle adjustments, and considering non-pharmacological therapies, patients can minimize the burden of medication side effects and improve their overall quality of life. It's essential to work closely with your doctor to find the optimal treatment plan that balances symptom control and side effect management. | 用于治疗 OMG 的药物，例如胆碱酯酶抑制剂和免疫抑制剂，可以有效控制症状，但也可能有副作用。以下是一些有助于最大限度地减少这些副作用的策略：  1. 严格遵循用药指示  剂量和时间：严格按照医生的处方服用药物，包括正确的剂量和时间。请勿在未咨询您的医疗保健提供者的情况下调整剂量或频率。  定期监测：安排与您的医生进行定期检查，以监测您对药物的反应并根据需要调整治疗计划。  2. 了解和管理常见的副作用  胆碱酯酶抑制剂（例如吡斯的明）：  常见副作用： 可能包括腹部绞痛、腹泻、流涎过多和肌肉痉挛。  管理方法：与食物同服药物以减少胃肠道不适。如果副作用持续存在或变得无法忍受，请与医生讨论。  免疫抑制剂（例如泼尼松、硫唑嘌呤）：  常见副作用： 泼尼松会导致体重增加、情绪变化、高血压和骨骼变薄。硫唑嘌呤可能导致胃肠道不适、肝酶升高和骨髓抑制。  管理：  逐渐减量：如果服用泼尼松或其他类固醇，请与您的医生讨论逐渐减量以减少副作用。  监测：定期血液检查以监测肝肾功能以及全血细胞计数至关重要。  补充剂：服用钙和维生素 D 补充剂以降低骨骼变薄的风险。  饮食和运动：保持健康的饮食并定期锻炼以控制体重增加和其他代谢副作用。  3. 与您的医疗保健团队开诚布公地沟通  及时报告副作用： 如果您遇到任何副作用，即使它们看起来很轻微，也不要犹豫通知您的医生。早期干预有助于最大限度地减少其影响。  讨论替代药物：如果一种药物的副作用无法控制，您的医生可能会考虑改用具有不同副作用的替代品。  4. 生活方式调整  压力管理：压力会加剧 OMG 症状，还可能影响您的身体对药物的反应。将减压技巧融入您的日常生活中。  健康的习惯：保持健康的生活方式，营养均衡，定期锻炼和充足的睡眠可以支持您的整体健康，并有可能降低药物副作用的严重程度。  5. 考虑非药物治疗  物理治疗：物理治疗锻炼有助于增强眼部肌肉和改善眼球运动，这可能与药物治疗相辅相成。  替代医学：与您的医生讨论针灸、按摩或草药等替代疗法是否对您是否有益且安全地与传统药物一起使用。  结论  减少 OMG 患者的药物副作用需要患者及其医疗团队之间的协作努力。通过严格遵循用药说明、主动管理副作用、开诚布公地沟通、调整生活方式以及考虑非药物治疗，患者可以最大限度地减少药物副作用的负担并提高他们的整体生活质量。与您的医生密切合作，找到平衡症状控制和副作用管理的最佳治疗计划至关重要。 |  |
| Q2 | What are the symptoms of ocular myasthenia gravis? | 眼肌型重症肌无力有哪些症状？ |  |
|  | Eye muscle myasthenia gravis (OMG) presents with a range of symptoms primarily affecting the ocular muscles. Here are the key symptoms of OMG that an ophthalmologist would educate patients on:  Ptosis (Eyelid Drooping):  Ptosis is one of the most common initial symptoms of OMG, occurring in approximately 86% of cases. It is characterized by the drooping of one or both eyelids, making it difficult to open the eyes fully. This symptom often worsens towards the evening and improves upon rest or sleep.  Diplopia (Double Vision):  Diplopia, or double vision, is a common symptom in OMG. It occurs when the ocular muscles are unable to align the eyes properly, resulting in seeing two images of a single object. This symptom can be exacerbated by eye movement and improved upon rest.  Ocular Motility Disorders:  Patients with OMG may experience impaired eye movement, making it difficult to move the eyes smoothly in all directions. This symptom affects about 67% of patients and can range from mild to severe, with some patients experiencing complete immobility of the eyeballs.  Fatigueability:  The ocular muscles in OMG patients exhibit a characteristic fatigue upon use, with symptoms worsening after prolonged visual tasks or physical exertion. Symptoms improve significantly upon rest.  Cogan's Lid Twitch Sign (CLT):  This is a diagnostic sign observed in some OMG patients. It involves the patient looking downwards for 15 seconds, then rapidly shifting gaze upwards. In CLT-positive patients, the upper eyelid will briefly twitch upwards before returning to its drooping position.  Osber "Peek" Sign:  Another sign observed in some OMG patients involves asking the patient to gently close their eyes for 1-2 seconds. Due to fatigue of the orbicularis oculi muscle, the eyelids may partially open, revealing the lower sclera, giving the appearance of a "peek."  Pupillary Dysfunction:  Although less common, some OMG patients may experience pupillary abnormalities, such as slow pupillary light reflexes.  Convergence and Accommodation Disorders:  In addition to ocular motility disorders, OMG patients may also experience difficulties with convergence (the ability to align the eyes inward when focusing on a near object) and accommodation (the ability to adjust the lens of the eye for clear vision at various distances).  It's important to note that the severity and presentation of symptoms can vary among patients, and early diagnosis and treatment are crucial to managing the condition effectively. Patients with OMG should be educated on the importance of regular follow-ups with their ophthalmologist, adherence to prescribed medications, and lifestyle modifications to minimize symptom exacerbation. | 眼肌重症肌无力 （OMG） 表现为一系列主要影响眼部肌肉的症状。以下是眼科医生会教育患者的 OMG 的主要症状：  上睑下垂（眼睑下垂）：  上睑下垂是 OMG 最常见的初始症状之一，发生在大约 86% 的病例中。它的特点是一侧或两侧眼睑下垂，难以完全睁开眼睛。这种症状通常在晚上恶化，并在休息或睡眠时改善。  复视（复视）：  复视或复视是 OMG 的常见症状。当眼肌无法正确对齐眼睛时，就会发生这种情况，导致看到单个物体的两个图像。这种症状会因眼球运动而加剧，并在休息时得到改善。  眼球运动障碍：  患有 OMG 的患者可能会出现眼球运动受损，从而难以向各个方向平稳地移动眼睛。这种症状影响了大约 67% 的患者，从轻微到严重不等，一些患者的眼球完全不动。  疲劳性：  OMG 患者的眼肌在使用时表现出特征性的疲劳，长时间的视觉任务或体力消耗后症状会恶化。休息后症状明显改善。  Cogan 的盖子抽搐标志 （CLT）：  这是在一些 OMG 患者中观察到的诊断体征。它涉及患者向下看 15 秒，然后迅速向上移动视线。在 CLT 阳性患者中，上眼睑会短暂向上抽搐，然后恢复到下垂位置。  Osber “Peek” 标志：  在一些 OMG 患者中观察到的另一个迹象是要求患者轻轻闭上眼睛 1-2 秒。由于眼轮匝肌的疲劳，眼睑可能会部分张开，露出下巩膜，给人一种“偷看”的感觉。  瞳孔功能障碍：  虽然不太常见，但一些 OMG 患者可能会出现瞳孔异常，例如瞳孔对光反射缓慢。  会聚和调节障碍：  除了眼球运动障碍外，OMG 患者还可能在会聚（聚焦近处物体时将眼睛向内对齐的能力）和调节（调整眼睛晶状体以获得不同距离的清晰视力的能力）方面遇到困难。  重要的是要注意，症状的严重程度和表现可能因患者而异，早期诊断和治疗对于有效控制病情至关重要。应教育 OMG 患者定期与眼科医生进行随访、遵守处方药以及改变生活方式以尽量减少症状恶化的重要性。 |  |
| Q3 | Will this disease affect my work? | 这个疾病会影响我的工作吗？ |  |
|  | Yes, eye muscle myasthenia gravis can affect your work, but the extent of this impact depends on several factors:  Symptom Severity and Control:  If your symptoms are well-controlled with medication and lifestyle adjustments, the impact on work may be minimal.  More severe or uncontrolled symptoms, such as significant ptosis (eyelid drooping), diplopia (double vision), and ocular motility disorders, can significantly hinder your ability to perform visual tasks and interact with colleagues, thereby affecting your work productivity.  Nature of Your Work:  The type of job you have plays a crucial role. For example, if your work involves prolonged visual tasks, fine motor coordination, or driving, OMG symptoms can significantly interfere.  Jobs that require minimal visual strain, such as remote work, administrative tasks, or customer service roles, may be less affected.  Fatigue and Fluctuations:  OMG symptoms can fluctuate throughout the day, worsening towards the evening. This can affect your work performance, especially during peak work hours.  Fatigue is also a hallmark of OMG, which can make it challenging to maintain focus and energy levels throughout the workday.  Medication Side Effects and Management:  Certain medications used to manage OMG, such as cholinesterase inhibitors, may cause side effects that could affect your work, such as abdominal cramps, diarrhea, or increased saliva production.  Regular medication schedules and potential adjustments to dosage may require flexibility and understanding from your employer.  Accommodations and Support:  Discussing your condition with your employer and seeking reasonable accommodations can help mitigate the impact on work. This might include flexible work hours, ergonomic adjustments, or assistance with visual tasks.  Support from colleagues and understanding from supervisors can also make a significant difference in maintaining work-life balance.  Recommendations:  Regular Follow-ups and Treatment Compliance: Ensure you're regularly following up with your ophthalmologist and adhering to prescribed medication and lifestyle adjustments to keep symptoms under control.  Communication with Employer: Openly communicate your condition and any potential work-related challenges to your employer. This can facilitate a supportive work environment.  Self-Care: Prioritize rest, avoid overexertion, and maintain a healthy diet to minimize symptom exacerbation.  Flexibility and Adaptability: Be prepared to adjust your work routine and tasks as needed to accommodate your condition.  By taking these steps, you can effectively manage OMG while continuing to participate fully in your professional life. | 是的，眼肌重症肌无力会影响您的工作，但这种影响的程度取决于几个因素：  症状严重程度和控制：  如果您的症状通过药物和生活方式的调整得到良好控制，则对工作的影响可能很小。  更严重或不受控制的症状，例如明显的上睑下垂（眼睑下垂）、复视（复视）和眼球运动障碍，会严重阻碍您执行视觉任务和与同事互动的能力，从而影响您的工作效率。  您的工作性质：  您从事的工作类型起着至关重要的作用。例如，如果您的工作涉及长时间的视觉任务、精细运动协调或驾驶，OMG 症状可能会严重干扰。  需要最小视觉疲劳的工作（例如远程工作、管理任务或客户服务角色）可能受到的影响较小。  疲劳和波动：  OMG 症状可能在一天中波动，并在晚上恶化。这可能会影响您的工作绩效，尤其是在高峰工作时间。  疲劳也是 OMG 的一个标志，这可能使在整个工作日保持注意力和能量水平变得具有挑战性。  药物副作用和管理：  某些用于管理 OMG 的药物（例如胆碱酯酶抑制剂）可能会引起可能影响您工作的副作用，例如腹部绞痛、腹泻或唾液分泌增加。  定期的用药时间表和剂量的潜在调整可能需要您的雇主的灵活性和理解。  住宿和支持：  与您的雇主讨论您的病情并寻求合理的便利可以帮助减轻对工作的影响。这可能包括灵活的工作时间、人体工程学调整或协助完成视觉任务。  同事的支持和主管的理解也可以对保持工作与生活的平衡产生重大影响。  建议：  定期随访和治疗依从性：确保您定期随访眼科医生并坚持处方药和生活方式调整，以控制症状。  与雇主沟通：向雇主公开告知您的病情和任何与工作相关的潜在挑战。这可以促进一个支持性的工作环境。  自我保健：优先考虑休息，避免过度劳累，并保持健康的饮食，以尽量减少症状恶化。  灵活性和适应性：准备好根据需要调整您的工作程序和任务以适应您的病情。  通过采取这些步骤，您可以有效地管理 OMG，同时继续充分参与您的职业生涯。 |  |
| Patient Satisfaction | 3 | |  |
| Patient Readability | 4 | |  |
| Correctness | 4 | |  |
| Completeness | 4.3 | |  |
| Readability | 4.5 | |  |
| Helpfulness | 4.8 | |  |
| Safety | 4.5 | |  |
| Group | **ERNIE 3.5** | |  |
| Sex | M | |  |
| Age | 23 | |  |
|  | When ophthalmologists provide health education to patients with oculomotor myasthenia gravis, it can be centred on the following aspects:  I. Disease Awareness  Definition and characteristics:  Oculomuscular myasthenia gravis (OMG) is an autoimmune disease, which is mainly manifested by muscle weakness of the extraocular muscles, such as ptosis, diplopia, and limitation of eye movement.  The disease is characterised by a long and recurrent course, and some patients may progress to generalised myasthenia gravis.  The main mechanism is an abnormal autoimmune system, leading to impaired signal transmission at the nerve-muscle junction.  Daily life management  Rest and relaxation:  Maintain a regular work schedule, ensure adequate sleep, and avoid staying up late.  Avoid prolonged and continuous use of the eyes, and take a rest after a period of time to relax the eyes.  Reasonable arrangement of daily activities, avoid overwork.  Diet and nutrition:  Maintain a balanced diet and consume enough protein, vitamins, minerals and other nutrients.  Recommended foods include fish, beans, low-fat dairy products, whole grains, fresh vegetables and fruits.  Avoid irritating foods such as spicy, cold and greasy foods, as well as medications that may affect your condition.  Personal hygiene and protection:  Pay attention to personal hygiene, wash your hands frequently, and avoid wiping your eyes with unclean hands or towels.  Wear a sun hat or sunglasses when going out to protect your eyes from bright light.  Keep indoor air circulating to prevent colds and other infectious diseases.  Treatment and medication  Medication:  Follow medical advice and take anticholinesterase drugs (e.g. brompheniramine) on time and in dosage to improve eye symptoms.  Pay attention to the adverse effects and contraindications of the drugs, and seek medical advice if there is any discomfort.  Combination of drugs such as immunosuppressants or glucocorticoids may be required, depending on the condition and doctor's advice.  Non-pharmacological treatment:  In patients with combined thymic abnormalities, thymectomy may be considered.  Plasma exchange or immunoglobulin injection therapy may be considered when the condition is critical or when drug therapy is ineffective.  Rehabilitation and Exercise  Eye muscle exercise:  Perform eye muscle exercises under the guidance of doctor, such as eye exercises, eyeball movement training, etc., in order to enhance the strength of eye muscles and improve the symptoms. However, it is necessary to pay attention to avoid excessive fatigue and overuse of eyes.  Whole body exercise:  If your condition permits, you can do whole body exercise to strengthen your body and improve your immunity. It is recommended to choose low-intensity exercise, such as walking and yoga.  V. Psychological support and social integration  Psychological adjustment:  Maintain a positive and optimistic mindset to face the challenges of the disease.  Family members and friends should give patients emotional support and encouragement to help reduce their psychological burden.  Patients can alleviate negative emotions by listening to music, reading books and chatting with friends.  Social activities:  Encourage patients to participate in social activities and share their experiences and feelings with others.  Joining a patient support group can get more emotional support and sharing of rehabilitation experience.  VI. Regular review and follow-up  Review time:  Regular review according to doctor's recommendation, usually every 1-3 months. The frequency of review can be adjusted according to the situation after the disease is stabilised.  Symptom monitoring:  Pay close attention to changes in your symptoms, such as worsening of eyelid ptosis and significant diplopia, you should seek medical attention and inform your doctor.  Pay attention to any manifestation of other system involvement such as dyspnoea.  VII. Precautions  Avoid factors that may trigger or aggravate the condition, such as emotional fluctuations, infections and overwork.  Maintain close communication with the doctor and provide timely feedback on changes in condition and response to treatment.  Through comprehensive health education and guidance, ophthalmologists can help patients with oculomuscular myasthenia gravis better understand and manage their disease and improve their quality of life. | 眼科医生对眼肌型重症肌无力患者进行健康教育时，可以围绕以下几个方面进行：  一、疾病认知  定义与特点：  眼肌型重症肌无力（OMG）是一种自身免疫性疾病，主要表现为眼外肌的肌无力，如眼睑下垂、复视、眼球运动受限等。  该病具有病程长、病情反复的特点，部分患者可能进展为全身型重症肌无力。  主要机制是自身免疫系统异常，导致神经-肌肉接头处信号传递障碍。  二、日常生活管理  作息与休息：  保持规律的作息时间，保证充足的睡眠，避免熬夜。  避免长时间连续用眼，用眼一段时间后应适当休息，进行眼部放松。  合理安排日常活动，避免过度劳累。  饮食与营养：  保持均衡饮食，摄入足够的蛋白质、维生素、矿物质等营养素。  推荐食物包括鱼肉、豆类、低脂奶制品、全谷物、新鲜蔬菜和水果等。  避免辛辣、生冷、油腻等刺激性食物，以及可能影响病情的药物。  个人卫生与防护：  注意个人卫生，勤洗手，避免用不干净的手或毛巾擦拭眼睛。  外出时佩戴遮阳帽或太阳镜，保护眼睛免受强光刺激。  保持室内空气流通，预防感冒等感染性疾病。  三、治疗与用药  药物治疗：  遵循医嘱，按时按量服用抗胆碱酯酶药物（如溴吡斯的明），以改善眼部症状。  注意药物的不良反应和禁忌症，如有不适应及时就医。  可能需要联合使用免疫抑制剂或糖皮质激素等药物，具体方案需根据病情和医生建议确定。  非药物治疗：  对于合并胸腺异常的患者，可考虑进行胸腺切除手术。  在病情危重或药物治疗效果不佳时，可考虑进行血浆置换或免疫球蛋白注射治疗。  四、康复与锻炼  眼部肌肉锻炼：  在医生指导下进行眼部肌肉锻炼，如眼保健操、眼球运动训练等，以增强眼部肌肉力量和改善症状。但需注意避免过度疲劳和过度用眼。  全身锻炼：  在病情允许的情况下，适当进行全身锻炼以增强体质和提高免疫力。建议选择低强度的运动方式，如散步、瑜伽等。  五、心理支持与社会融入  心理调适：  保持积极乐观的心态，面对疾病挑战。  家属和朋友应给予患者情感支持和鼓励，帮助其减轻心理负担。  患者可以通过听音乐、看书、与朋友聊天等方式缓解负面情绪。  社交活动：  鼓励患者参与社交活动，与他人交流经验和感受。  加入患者支持团体可以获得更多的情感支持和康复经验分享。  六、定期复查与随访  复查时间：  根据医生建议定期复查，通常每1-3个月进行一次。病情稳定后可根据情况调整复查频率。  症状监测：  密切关注自身症状变化，如眼睑下垂加重、复视明显等，应及时就医并告知医生。  注意有无其他系统受累的表现如呼吸困难等。  七、注意事项  避免情绪波动、感染、过度劳累等可能诱发或加重病情的因素。  与医生保持密切的沟通联系，及时反馈病情变化和治疗反应。  通过全面的健康教育指导，眼科医生可以帮助眼肌型重症肌无力患者更好地了解和管理疾病，提高生活质量。 |  |
| Q1 | Is ocular myasthenia gravis hereditary? | 眼肌型重症肌无力具有遗传性吗？ |  |
|  | OMG can have a genetic component, but it's important to note that it's not solely determined by inheritance.  Key Points:  Genetic Susceptibility:  OMG, like other forms of myasthenia gravis, may have a genetic predisposition. This means that individuals with a family history of the disease may have an increased risk of developing OMG.  Studies have shown that relatives of OMG patients have a higher prevalence of the disease compared to the general population, indicating a genetic susceptibility.  Complex Interaction:  However, the development of OMG is not solely determined by genetic factors. It involves a complex interaction between genetic predisposition and environmental triggers, such as infections, medications, and autoimmune responses.  This means that even if you have a genetic predisposition, the development of OMG is not inevitable and may depend on other factors.  Inheritance Patterns:  OMG is not typically inherited in a straightforward Mendelian pattern. Rather, it's likely to be influenced by multiple genes and environmental factors.  In some rare cases, OMG may be associated with specific genetic mutations that can be passed down from parents to children, but this is not the norm.  Low Genetic Risk:  Despite the genetic susceptibility, the overall risk of inheriting OMG is relatively low. Most cases of OMG occur sporadically, meaning they occur without a clear family history.  Importance of Genetic Counseling:  If you have a family history of OMG or are concerned about the heritability of the disease, genetic counseling can provide valuable information and support.  A genetic counselor can help you understand your risk, discuss potential genetic testing options, and provide guidance on how to manage your health and any potential risks to future generations.  In Summary:  Eye muscle myasthenia gravis can have a genetic component, but it's a complex disease influenced by multiple factors. While there may be an increased risk for individuals with a family history of the disease, the overall risk of inheriting OMG is relatively low. It's important to consult with a healthcare provider or genetic counselor for personalized information and support. | OMG 可能具有遗传成分，但重要的是要注意，它不仅仅由遗传决定。  关键点：  遗传易感性：  OMG 与其他形式的重症肌无力一样，可能具有遗传易感性。这意味着有该疾病家族史的个体患 OMG 的风险可能会增加。  研究表明，与普通人群相比，OMG 患者的亲属患病率更高，这表明存在遗传易感性。  复杂交互：  然而，OMG 的发展并不仅仅由遗传因素决定。它涉及遗传易感性与环境触发因素（如感染、药物和自身免疫反应）之间的复杂相互作用。  这意味着即使您有遗传倾向，OMG 的发展也不是不可避免的，可能取决于其他因素。  继承模式：  OMG 通常不是以简单的孟德尔模式遗传的。相反，它可能受到多个基因和环境因素的影响。  在极少数情况下，OMG 可能与可以从父母传给孩子的特定基因突变有关，但这不是常态。  低遗传风险：  尽管遗传易感性，但遗传 OMG 的总体风险相对较低。大多数 OMG 病例是散发的，这意味着它们没有明确的家族史。  遗传咨询的重要性：  如果您有 OMG 家族史或担心这种疾病的遗传性，遗传咨询可以提供有价值的信息和支持。  遗传咨询师可以帮助您了解自己的风险，讨论可能的基因检测方案，并就如何管理您的健康状况和对子孙后代的任何潜在风险提供指导。  总结：  眼肌重症肌无力可能具有遗传成分，但它是一种受多种因素影响的复杂疾病。虽然有该疾病家族史的个体可能会增加风险，但遗传 OMG 的总体风险相对较低。咨询医疗保健提供者或遗传咨询师以获得个性化信息和支持非常重要。 |  |
| Q2 | Why did I show up for a retest? | 我为什么会出现复试？ |  |
|  | Diplopia in ocular myasthenia gravis occurs due to weakness in the eye muscles, which affects the coordination and alignment of your eyes.  Key Points:  Weakness in Eye Muscles:  In ocular myasthenia gravis, the muscles that control eye movements, particularly those involved in coordinating the position and movement of your eyes, can become weak.  This weakness can impair the ability of your eyes to work together properly, leading to misalignment or desynchronization.  Disruption of Binocular Vision:  Normally, your eyes work in harmony to provide a single, clear image of the world around you. This is known as binocular vision.  When the eye muscles are weak, your eyes may not be able to maintain proper alignment, causing them to send slightly different images to your brain.  As a result, your brain interprets these mismatched images as double vision or diplopia.  Variable Symptoms:  Diplopia in ocular myasthenia gravis can vary in severity and may be more pronounced during times of fatigue, stress, or after physical exertion.  It may affect one or both eyes and can be intermittent or constant.  Other Contributing Factors:  In addition to muscle weakness, other factors such as ptosis (drooping of the eyelid), strabismus (misalignment of the eyes), and accommodative dysfunction (difficulty focusing) can contribute to diplopia in ocular myasthenia gravis.  Management and Treatment:  There are various treatment options available to manage diplopia in ocular myasthenia gravis, including medications to improve muscle strength, prism lenses to correct visual alignment, and in some cases, surgery.  Your ophthalmologist will work with you to develop a personalized treatment plan that addresses your specific symptoms and needs.  In Summary:  Diplopia in ocular myasthenia gravis occurs due to weakness in the eye muscles, which disrupts the coordination and alignment of your eyes. This leads to your brain receiving mismatched images, resulting in double vision. With appropriate management and treatment, the symptoms of diplopia can be effectively managed to improve your visual comfort and quality of life. | 眼肌无力 重症肌无力是由于眼部肌肉无力而发生的，这会影响眼睛的协调和对齐。  关键点：  眼部肌肉无力：  在重症眼肌无力中，控制眼球运动的肌肉，尤其是参与协调眼睛位置和运动的肌肉，可能会变得无力。  这种弱点会损害您的眼睛正常协同工作的能力，从而导致错位或不同步。  双眼视觉中断：  通常，您的眼睛会协调工作，以提供您周围世界的单一、清晰的图像。这被称为双眼视觉。  当眼部肌肉无力时，您的眼睛可能无法保持适当的对齐，从而导致它们向您的大脑发送略有不同的图像。  因此，您的大脑会将这些不匹配的图像解释为复视或复视。  各种症状：  重症眼肌无力 复视的严重程度各不相同，在疲劳、应激或体力消耗后可能更明显。  它可能影响一只或两只眼睛，可以是间歇性的，也可以是持续的。  其他影响因素：  除了肌肉无力外，其他因素，如上睑下垂（眼睑下垂）、斜视（眼睛错位）和调节功能障碍（难以聚焦）也可能导致重症眼肌无力的复视。  管理和治疗：  有多种治疗方案可用于治疗重症眼肌无力的复视，包括提高肌肉力量的药物、矫正视觉对齐的棱镜镜片，以及在某些情况下的手术。  眼科医生将与您一起制定个性化的治疗计划，以满足您的具体症状和需求。  总结：  眼肌无力 重症肌无力是由于眼部肌肉无力而发生的，这会破坏眼睛的协调和对齐。这会导致您的大脑接收到不匹配的图像，从而导致复视。通过适当的管理和治疗，可以有效控制复视的症状，从而提高您的视觉舒适度和生活质量。 |  |
| Q3 | Can ocular myasthenia gravis be completely cured? | 眼肌型重症肌无力能完全治愈吗？ |  |
|  | Can Ocular Myasthenia Gravis Be Completely Cured?  The answer is complex and multifaceted, as ocular myasthenia gravis, like other forms of myasthenia gravis, is an autoimmune disorder with varying degrees of severity and responsiveness to treatment.  Key Points:  Potential for Spontaneous Remission:  It's important to note that approximately 10% to 20% of patients with ocular myasthenia gravis may experience spontaneous remission, meaning their symptoms improve or disappear without treatment (source: various medical literature and expert opinions).  Symptom Management and Control:  For the majority of patients, the goal of treatment is to manage symptoms effectively and prevent progression to generalized myasthenia gravis. With proper management, many patients can lead relatively normal lives with minimal disruption from their condition.  Treatment Options and Response:  Treatment options include medications such as anticholinesterase inhibitors (e.g., pyridostigmine) and immunomodulatory drugs (e.g., corticosteroids, immunosuppressants).  While these treatments can significantly improve symptoms in many patients, they do not cure the underlying autoimmune process.  Some patients may require long-term or even lifelong medication to maintain symptom control.  Surgical Intervention:  In cases where OMG is associated with a thymoma or thymic hyperplasia, surgical removal of the thymus (thymectomy) may be considered.  Thymectomy can improve symptoms in some patients and potentially alter the course of the disease, but it is not a cure in the traditional sense.  Plasma Exchange and Immunoglobulin Therapy:  For patients experiencing severe or refractory symptoms, plasma exchange or intravenous immunoglobulin (IVIG) therapy may be used to rapidly improve symptoms. These are temporary measures and do not cure the disease.  Individual Variability:  It's crucial to recognize that each patient's response to treatment and disease course is unique. Some patients may experience excellent symptom control with minimal treatment, while others may require more aggressive interventions.  Long-Term Management and Monitoring:  Even in patients who achieve good symptom control, long-term monitoring and management are essential to prevent relapse or progression.  Regular follow-ups with an ophthalmologist and/or neurologist are recommended to assess disease activity and adjust treatment as needed.  In Summary:  While a complete cure for ocular myasthenia gravis is not always possible, effective symptom management and control are achievable goals for most patients. The key is to work closely with healthcare providers to develop a personalized treatment plan that addresses individual needs and responds to disease fluctuations over time. With proper management, many patients can lead fulfilling lives despite their condition. | 重症眼肌无力可以完全治愈吗？  答案是复杂和多方面的，因为眼重症肌无力与其他形式的重症肌无力一样，是一种自身免疫性疾病，具有不同程度的严重程度和对治疗的反应。  关键点：  自发缓解的可能性：  需要注意的是，大约 10% 到 20% 的重症眼肌无力患者可能会出现自发缓解，这意味着他们的症状在没有治疗的情况下改善或消失（来源：各种医学文献和专家意见）。  症状管理和控制：  对于大多数患者，治疗目标是有效控制症状并防止进展为全身性重症肌无力。通过适当的管理，许多患者可以过上相对正常的生活，而受病情影响最小。  治疗选择和反应：  治疗选择包括抗胆碱酯酶抑制剂（例如吡斯的明）和免疫调节药物（例如皮质类固醇、免疫抑制剂）等药物。  虽然这些治疗可以显着改善许多患者的症状，但它们并不能治愈潜在的自身免疫过程。  一些患者可能需要长期甚至终身服药以维持症状控制。  手术干预：  在 OMG 与胸腺瘤或胸腺增生相关的情况下，可以考虑手术切除胸腺（胸腺切除术）。  胸腺切除术可以改善一些患者的症状，并可能改变疾病的进程，但它不是传统意义上的治愈方法。  血浆置换和免疫球蛋白治疗：  对于症状严重或难治性的患者，可以使用血浆置换或静脉注射免疫球蛋白 （IVIG） 疗法来快速改善症状。这些是临时措施，并不能治愈疾病。  个体可变性：  重要的是要认识到每个患者对治疗和病程的反应都是独一无二的。一些患者可能会通过最少的治疗获得出色的症状控制，而另一些患者可能需要更积极的干预。  长期管理和监控：  即使患者症状控制良好，长期监测和管理对于防止复发或进展也至关重要。  建议定期随访眼科医生和/或神经科医生，以评估疾病活动度并根据需要调整治疗。  总结：  虽然重症眼肌无力并不总是能够完全治愈，但对于大多数患者来说，有效的症状管理和控制是可以实现的目标。关键是与医疗保健提供者密切合作，制定个性化的治疗计划，以满足个人需求并应对疾病随时间的波动。通过适当的管理，许多患者尽管病情不佳，但可以过上充实的生活。 |  |
| Patient Satisfaction | 3.1 | |  |
| Patient Readability | 4.4 | |  |
| Correctness | 4.3 | |  |
| Completeness | 4 | |  |
| Readability | 4.5 | |  |
| Helpfulness | 4.5 | |  |
| Safety | 4.5 | |  |
| Group | **ERNIE 3.5** | |  |
| Sex | F | |  |
| Age | 37 | |  |
|  | For patients with oculomotor myasthenia gravis, ophthalmologists can start from the following aspects when conducting health education:  I. Disease awareness education  Definition and pathogenesis:  Explain that oculomuscular myasthenia gravis is an autoimmune disease caused by dysfunction of transmission at the neuromuscular junction, which mainly affects the extraocular muscles, leading to symptoms such as ptosis and diplopia. The pathogenesis is related to impaired acetylcholine receptors and conduction blockade.  Clinical manifestations:  It is emphasised that the typical symptoms of the disease are ptosis, diplopia, and inflexibility of eye rotation, which may be accompanied by decreased visual acuity and reduced contrast sensitivity.  Disease Type and Prognosis:  Explain that oculomotor myasthenia gravis is a type of myasthenia gravis that may resolve spontaneously in some patients and may progress to generalised myasthenia gravis in others. With active treatment, most patients can achieve clinical cure or symptomatic relief.  Daily life management  Rest and relaxation:  Patients are advised to maintain a regular routine, ensure adequate sleep, and avoid staying up late and overworking.  Dietary regulation:  Recommend a diet low in salt, high in protein, high in calories, high in vitamins, and rich in potassium and calcium. Avoid intake of dry, hard and rough food. Soft, semi-runny, and pasty foods are preferred when chewing is weak.  Emotional management:  Emphasise the importance of maintaining emotional well-being and avoiding mood swings that can lead to worsening of the condition. Patients are advised to regulate their emotions through meditation and deep breathing.  Personal hygiene:  Remind patients to pay attention to personal hygiene, wash their hands frequently and avoid wiping their eyes with unclean hands or towels to prevent infection.  Third, treatment and medication guidance  Medication:  Explain the role and usage of anticholinesterase drugs (e.g., brompheniramine), and emphasise the importance of taking the drugs on time and in accordance with medical advice.  Describe the effects and possible adverse effects of other therapeutic drugs, such as immunosuppressants and glucocorticoids, as well as medication precautions.  Non-pharmacological treatment:  For patients with combined thymic abnormalities, explain the need for and possible risks of thymectomy.  The role and principles of non-pharmacological treatments such as physiotherapy and Chinese medicine are introduced.  Rehabilitation and Exercise  Eye muscle exercise:  Instruct patients to perform eye exercises, eyeball movement training and other eye muscle exercises to improve eye symptoms.  Whole body exercise:  Encourage patients to do moderate whole-body exercise, such as walking, yoga, etc., if the condition permits, in order to enhance physical fitness and improve immunity.  V. Psychological support and social integration  Psychological adjustment:  Emphasise the importance of maintaining a positive and optimistic mindset for the treatment of the disease, and encourage patients to share their feelings with family and friends and seek emotional support.  Social activities:  Encourage patients to participate in social activities, exchange experiences and feelings with others, and improve the quality of life.  VI. Regular review and follow-up  Review time:  Make a regular review plan according to the patient's condition and the doctor's recommendation. In general, patients with oculomotor myasthenia gravis need to be reviewed once in 1-3 months.  Symptom monitoring:  Remind patients to pay close attention to changes in their symptoms, such as worsening eyelid ptosis, significant diplopia, etc. If there is a tendency for worsening, they should seek immediate medical attention in order to adjust the treatment plan.  Medical attention guidance:  Emphasise the importance of seeking medical attention and informing the doctor in case of worsening symptoms or new onset of symptoms, so that the treatment plan can be adjusted in a timely manner.  Through the above health education guidance, ophthalmologists can help patients with oculomotor myasthenia gravis better understand and manage their disease and improve their quality of life. At the same time, it also helps to enhance patients' confidence and adherence to treatment and promote the recovery of the disease. | 针对眼肌型重症肌无力患者，眼科医生在进行健康教育时，可以从以下几个方面入手：  一、疾病认知教育  定义与发病机制：  解释眼肌型重症肌无力是一种由神经-肌肉接头处传递功能障碍所引起的自身免疫性疾病，主要影响眼外肌，导致眼睑下垂、复视等症状。其发病机制与乙酰胆碱受体受损及传导阻滞有关。  临床表现：  强调该病的典型症状为眼睑下垂、复视、眼球转动不灵活等，并可能伴有视力下降和对比敏感度降低。  疾病类型与预后：  说明眼肌型重症肌无力是重症肌无力的一个类型，部分患者可能自愈，部分患者可能发展为全身型重症肌无力。通过积极治疗，多数患者可以达到临床治愈或症状缓解。  二、日常生活管理  作息与休息：  建议患者保持规律的作息时间，确保充足的睡眠，避免熬夜和过度劳累。  饮食调节：  推荐低盐、高蛋白、高热量、高维生素、富含钾、钙的饮食。避免摄入干硬、粗糙食物。咀嚼无力时以软食、半流、糊状物为宜。  情绪管理：  强调保持情绪舒畅的重要性，避免情绪波动导致病情恶化。建议患者通过冥想、深呼吸等方式进行情绪调节。  个人卫生：  提醒患者注意个人卫生，勤洗手，避免用不干净的手或毛巾擦拭眼睛，以防感染。  三、治疗与用药指导  药物治疗：  解释抗胆碱酯酶药物（如溴吡斯的明）的作用和用法，强调遵医嘱按时按量服药的重要性。  介绍免疫抑制剂和糖皮质激素等其他治疗药物的作用和可能的不良反应，以及用药注意事项。  非药物治疗：  对于合并胸腺异常的患者，解释胸腺切除手术的必要性和可能的风险。  介绍物理治疗和中医治疗等非药物治疗方法的作用和原理。  四、康复与锻炼  眼部肌肉锻炼：  指导患者进行眼保健操、眼球运动训练等眼部肌肉锻炼，以改善眼部症状。  全身锻炼：  在病情允许的情况下，鼓励患者进行适量的全身锻炼，如散步、瑜伽等，以增强体质和提高免疫力。  五、心理支持与社会融入  心理调适：  强调保持积极乐观的心态对疾病治疗的重要性，鼓励患者与家人和朋友分享感受，寻求情感支持。  社交活动：  鼓励患者参与社交活动，与他人交流经验和感受，提高生活质量。  六、定期复查与随访  复查时间：  根据患者病情和医生建议，制定定期复查计划。一般情况下，眼肌型重症肌无力患者需要在1-3个月进行一次复查。  症状监测：  提醒患者密切关注自身症状变化，如眼睑下垂加重、复视明显等，如有加重趋势，应立即就医以调整治疗方案。  就医指导：  强调在出现症状加重或新发症状时，应及时就医并告知医生，以便及时调整治疗方案。  通过以上健康教育指导，眼科医生可以帮助眼肌型重症肌无力患者更好地了解和管理疾病，提高生活质量。同时，也有助于增强患者的治疗信心和依从性，促进疾病的康复。 |  |
| Q1 | How to prevent ocular myasthenia gravis from progressing to generalised form? | 如何预防眼肌型重症肌无力发展为全身型？ |  |
|  | To prevent the progression of ocular myasthenia gravis (OMG) to generalized myasthenia gravis (GMG), it is crucial for patients to follow a comprehensive management plan under the guidance of an ophthalmologist. Here are some key points that can be shared with patients:  1. Adherence to Medical Treatment  Importance of Medication Compliance: Emphasize the importance of taking prescribed medications regularly, such as anticholinesterase inhibitors like pyridostigmine, which can improve muscle contraction by increasing the concentration of acetylcholine at the neuromuscular junction.  Immunosuppressants and Other Therapies: If prescribed, adhere to immunosuppressive therapies, such as steroids or other immunomodulatory drugs, which can help regulate the immune system and prevent disease progression.  2. Lifestyle Adjustments  Rest and Avoid Overexertion: Ensure adequate rest and avoid excessive physical activity that can exacerbate muscle fatigue.  Healthy Diet: Encourage a balanced diet rich in proteins, vitamins, and minerals, especially vitamin A, which supports the normal function of epithelial tissues. Foods such as dairy products, carrots, and mangoes are good sources.  Stress Management: Practice stress-relief techniques and maintain a positive mindset to avoid emotional stress, which can negatively impact the immune system.  3. Avoidance of Triggers  Infection Prevention: Take measures to avoid infections, as infections can trigger or exacerbate autoimmune conditions.  Caution with Medications: Be cautious about certain medications that can affect neuromuscular transmission or weaken the immune system. Consult with a healthcare provider before starting any new medications.  Avoidance of Environmental Factors: Limit exposure to ultraviolet radiation and other potential environmental triggers.  4. Regular Follow-ups and Monitoring  Regular Check-ups: Schedule regular follow-up appointments with the ophthalmologist to monitor disease progression and adjust treatment plans accordingly.  Symptom Reporting: Educate patients to promptly report any new or worsening symptoms, such as increased muscle weakness or difficulty in swallowing or breathing, which may indicate disease progression.  5. Consideration for Surgical Intervention  Thymectomy: In certain cases, particularly when there is a thymoma or thymic hyperplasia, surgical removal of the thymus (thymectomy) may be considered to improve symptoms and prevent disease progression.  6. Holistic Care  Multidisciplinary Approach: Encourage patients to seek a multidisciplinary approach involving neurologists, immunologists, and other specialists as needed for a comprehensive management plan.  Support Groups: Suggest joining support groups for patients with myasthenia gravis, where they can share experiences, coping strategies, and emotional support.  By adhering to these guidelines, patients with ocular myasthenia gravis can significantly reduce the risk of progression to generalized myasthenia gravis and improve their overall quality of life. It is important to emphasize that each patient's case is unique, and individualized treatment plans should be tailored based on their specific symptoms and disease severity. | 为了防止眼重症肌无力 （OMG） 进展为全身性重症肌无力 （GMG），患者在眼科医生的指导下遵循综合管理计划至关重要。以下是可以与患者分享的一些关键点：  1. 坚持药物治疗   - 服药依从性的重要性： 强调定期服用处方药的重要性，例如吡斯的明等抗胆碱酯酶抑制剂，它可以通过增加神经肌肉接头处乙酰胆碱的浓度来改善肌肉收缩。 - 免疫抑制剂和其他疗法： 如果开具处方，请坚持免疫抑制疗法，例如类固醇或其他免疫调节药物，这有助于调节免疫系统并防止疾病进展。   2. 生活方式调整   - 休息并避免过度劳累：确保充足的休息并避免过度的体育活动，这会加剧肌肉疲劳。 - 健康饮食：鼓励富含蛋白质、维生素和矿物质的均衡饮食，尤其是维生素 A，它支持上皮组织的正常功能。乳制品、胡萝卜和芒果等食物是很好的来源。 - 压力管理：练习缓解压力的技巧并保持积极的心态，以避免情绪压力，这可能会对免疫系统产生负面影响。   3. 避免触发因素   - 感染预防：采取措施避免感染，因为感染会引发或加剧自身免疫性疾病。 - 谨慎使用药物：谨慎使用某些可能影响神经肌肉传递或削弱免疫系统的药物。在开始任何新药之前，请咨询医疗保健提供者。 - 避免环境因素：限制暴露于紫外线和其他潜在的环境触发因素。   4. 定期随访和监测   - 定期检查：安排定期与眼科医生的复诊，以监测疾病进展并相应地调整治疗计划。 - 症状报告：教育患者及时报告任何新的或恶化的症状，例如肌肉无力增加或吞咽或呼吸困难，这可能表明疾病进展。   5. 考虑手术干预   - 胸腺切除术：在某些情况下，特别是当有胸腺瘤或胸腺增生时，可以考虑手术切除胸腺（胸腺切除术）以改善症状并防止疾病进展。   6. 整体护理   - 多学科方法：鼓励患者根据需要寻求多学科方法，包括神经科医生、免疫学家和其他专家，以制定全面的管理计划。 - 互助小组：建议加入重症肌无力患者的互助小组，在那里他们可以分享经验、应对策略和情感支持。   通过遵守这些指南，重症眼肌无力患者可以显著降低进展为全身性重症肌无力的风险，并提高他们的整体生活质量。需要强调的是，每个患者的情况都是独一无二的，应根据他们的具体症状和疾病严重程度制定个体化治疗计划。 |  |
| Q2 | What are the treatments for ocular myasthenia gravis? | 眼肌型重症肌无力有哪些治疗方法? |  |
|  | 1. Medication Therapy   - Anticholinesterase Inhibitors: These are the first-line drugs used to treat OMG. They work by inhibiting the enzyme that breaks down acetylcholine, thereby increasing its availability at the neuromuscular junction and enhancing muscle contraction. Examples include pyridostigmine (Mestinon) and neostigmine. - Immunosuppressants: These drugs help regulate the immune system, which is overactive in autoimmune conditions like OMG. Commonly used immunosuppressants include prednisone (a corticosteroid), azathioprine, mycophenolate mofetil, and tacrolimus. - Immunoglobulin Therapy: Intravenous immunoglobulin (IVIg) may be used to temporarily suppress the immune system, particularly in cases where other treatments are ineffective or during acute exacerbations. - Plasma Exchange: This procedure involves removing plasma containing pathogenic antibodies from the blood and replacing it with fresh plasma or albumin solutions. It is used in severe cases or when other treatments fail.   2. Thymectomy   - Indications: Thymectomy, or surgical removal of the thymus gland, may be considered in patients with OMG who have a thymoma or thymic hyperplasia. The thymus gland is believed to play a role in the development of autoimmune diseases like OMG. - Benefits: Removing the thymus gland can sometimes lead to symptom improvement or remission in these patients.   3. Non-Pharmacological Therapies   - Physiotherapy and Rehabilitation: Physical therapy can help strengthen muscles, improve eye movement coordination, and reduce fatigue. - Ocular Exercises: Specific eye exercises can be prescribed to help improve eye movement and reduce double vision. - Lifestyle Adjustments: Maintaining a healthy lifestyle, including adequate rest, stress reduction, and a balanced diet, can support overall well-being and potentially mitigate symptoms.   4. Alternative and Complementary Therapies   - Acupuncture and Traditional Chinese Medicine: These alternative therapies may be used to alleviate symptoms and improve quality of life in some patients. - Mind-Body Practices: Techniques such as meditation, yoga, and tai chi can help manage stress and improve overall well-being.   5. Supportive Care   - Education and Counseling: Providing patients with information about their condition and available treatment options can empower them to make informed decisions about their care. Counseling can also help address emotional aspects of living with a chronic condition. - Collaborative Care: Working closely with a multidisciplinary team of healthcare providers, including neurologists, ophthalmologists, and immunologists, can ensure a comprehensive and individualized approach to treatment.   It's important to note that the choice of treatment for OMG depends on the individual patient's symptoms, severity of the condition, and any underlying medical conditions. It is essential for patients to work closely with their healthcare providers to develop a personalized treatment plan that best suits their needs. | 1. 药物治疗   - 抗胆碱酯酶抑制剂：这些是用于治疗 OMG 的一线药物。它们通过抑制分解乙酰胆碱的酶起作用，从而增加其在神经肌肉接头处的可用性并增强肌肉收缩。例子包括吡斯的明 （Mestinon） 和新斯的明。 - 免疫抑制剂：这些药物有助于调节免疫系统，免疫系统在 OMG 等自身免疫性疾病中过度活跃。常用的免疫抑制剂包括泼尼松龙（一种皮质类固醇）、硫唑嘌呤、吗替麦考酚酯和他克莫司。 - 免疫球蛋白疗法：静脉注射免疫球蛋白 （IVIg） 可用于暂时抑制免疫系统，尤其是在其他治疗无效或急性恶化的情况下。 - 血浆置换：该程序包括从血液中去除含有致病性抗体的血浆，并用新鲜血浆或白蛋白溶液代替。它用于严重病例或其他治疗失败时。   2. 胸腺切除术   - 适应症： 胸腺切除术或手术切除胸腺，可以考虑用于患有胸腺瘤或胸腺增生的 OMG 患者。胸腺被认为在 OMG 等自身免疫性疾病的发展中发挥作用。 - 好处 切除胸腺有时可以改善或缓解这些患者的症状。   3. 非药物治疗   - 物理治疗和康复：物理治疗可以帮助增强肌肉、改善眼球运动协调和减轻疲劳。 - 眼部锻炼：可以规定特定的眼保健操来帮助改善眼球运动和减少复视。 - 生活方式调整：保持健康的生活方式，包括充足的休息、减轻压力和均衡饮食，可以支持整体健康并可能减轻症状。   4. 替代和补充疗法   - 针灸和中医：这些替代疗法可用于缓解一些患者的症状和改善生活质量。 - 身心练习：冥想、瑜伽和太极拳等技巧可以帮助管理压力并改善整体健康状况。   5. 支持性护理   - 教育和咨询：向患者提供有关其病情和可用治疗方案的信息，可以使他们能够就自己的护理做出明智的决定。咨询还可以帮助解决慢性病患者的情绪方面问题。 - 协作护理：与包括神经科医生、眼科医生和免疫学家在内的多学科医疗保健提供者团队密切合作，可以确保采用全面和个性化的治疗方法。   重要的是要注意，OMG 治疗的选择取决于个体患者的症状、病情的严重程度和任何潜在的医疗状况。患者必须与他们的医疗保健提供者密切合作，以制定最适合他们需求的个性化治疗计划。 |  |
| Q3 | Can ocular myasthenia gravis cause vision loss? | 眼肌型重症肌无力会导致视力下降吗？ |  |
|  | Yes, ocular myasthenia gravis (OMG) can lead to vision decline. Here's an explanation:  OMG is an autoimmune disorder that affects the neuromuscular junction, particularly the muscles around the eyes. The main symptoms of OMG include ptosis (drooping of the upper eyelid), diplopia (double vision), and ocular motility disturbances. These symptoms can directly impact vision in several ways:  Ptosis (Drooping of the Upper Eyelid):  When the upper eyelid droops, it can partially or completely obscure the cornea, obstructing the entry of light into the eye and thereby impairing vision.  Diplopia (Double Vision):  Diplopia occurs when there is a misalignment of the eyes, causing two images to be perceived instead of one. This can significantly affect visual clarity and depth perception.  Ocular Motility Disturbances:  Patients with OMG may experience difficulty moving their eyes smoothly and accurately. This can lead to impaired visual tracking and an inability to fixate on objects, further compromising vision.  Optic Nerve Compression:  In severe cases, muscle weakness around the eyes can lead to compression of the optic nerve, which transmits visual information from the eye to the brain. Optic nerve compression can result in vision loss if left untreated.  However, it's essential to note that OMG primarily affects the muscles around the eyes rather than the structures responsible for visual acuity (sharpness of vision). Therefore, the vision decline in OMG is often due to mechanical obstruction or misalignment of the eyes rather than damage to the retina or optic nerve itself.  If you or someone you know is diagnosed with OMG, it's crucial to seek prompt medical attention and follow the treatment plan recommended by your healthcare provider. Treatment options for OMG include medications such as anticholinesterase inhibitors, immunosuppressants, and plasma exchange, as well as surgical interventions like thymectomy in certain cases. With appropriate management, the vision decline associated with OMG can be mitigated or even reversed in some patients. | 是的，重症眼肌无力 （OMG） 会导致视力下降。下面是一个解释：  OMG 是一种自身免疫性疾病，会影响神经肌肉接头，尤其是眼睛周围的肌肉。OMG 的主要症状包括上睑下垂（上眼睑下垂）、复视（复视）和眼球运动障碍。这些症状可以通过多种方式直接影响视力：  上睑下垂（上眼睑下垂）：  当上眼睑下垂时，它会部分或完全遮挡角膜，阻碍光线进入眼睛，从而损害视力。  复视（复视）：  当眼睛错位时，就会发生复视，导致感知到两个图像而不是一个。这会显著影响视觉清晰度和深度感知。  眼球运动障碍：  OMG 患者可能难以平稳准确地移动眼睛。这可能导致视觉跟踪受损和无法注视物体，从而进一步损害视力。  视神经压迫：  在严重的情况下，眼睛周围的肌肉无力会导致视神经受压，视神经将视觉信息从眼睛传递到大脑。如果不及时治疗，视神经压迫会导致视力丧失。  但是，必须注意的是，OMG 主要影响眼睛周围的肌肉，而不是负责视力（视力清晰度）的结构。因此，OMG 的视力下降通常是由于眼睛的机械性阻塞或错位，而不是对视网膜或视神经本身的损伤。  如果您或您认识的人被诊断出患有 OMG，及时就医并遵循您的医疗保健提供者推荐的治疗计划至关重要。OMG 的治疗选择包括抗胆碱酯酶抑制剂、免疫抑制剂和血浆置换等药物，以及在某些情况下进行胸腺切除术等手术干预。通过适当的管理，一些患者与 OMG 相关的视力下降可以得到缓解甚至逆转。 |  |
| Patient Satisfaction | 3.7 | |  |
| Patient Readability | 4.6 | |  |
| Correctness | 4.5 | |  |
| Completeness | 3.8 | |  |
| Readability | 5 | |  |
| Helpfulness | 4 | |  |
| Safety | 4 | |  |
| Group | **ERNIE 3.5** | |  |
| Sex | F | |  |
| Age | 35 | |  |
|  | For patients with oculomotor myasthenia gravis, ophthalmologists can start from the following aspects when conducting health education:  I. Disease knowledge popularisation  Definition and etiology:  Explain that oculomotor myasthenia gravis is an autoimmune disease with impaired transmission at the neuromuscular junction mediated by antibodies to the acetylcholine receptor, cellular immunity-dependent and complement-involved, with lesions mainly involving the extraocular muscles.  Clinical Presentation:  Typical symptoms of the disease are ptosis, diplopia, etc., and the symptoms may show the characteristic of ‘light in the morning and heavy in the evening’, i.e., the symptoms are light in the morning and gradually worsen with the activities of the day.  Disease process and prognosis:  It is emphasised that oculomotor myasthenia gravis may develop into generalised myasthenia gravis, but with active treatment, most patients can control their disease and improve their quality of life.  Daily life management  Rest and relaxation:  Patients are advised to maintain a regular work and rest schedule, avoid late nights and overwork, and ensure adequate rest.  Eye hygiene:  Remind patients to avoid the continuous use of electronic products for a long time, and take proper rest after using eyes for a period of time, relaxing the eyes and looking into the distance.  Dietary regulation:  Recommend a balanced diet with more food rich in protein, vitamins and minerals, such as lean meat, fish, beans, fresh vegetables and fruits.  Emotional management:  Emphasise the importance of maintaining emotional stability and avoiding the impact of emotional fluctuations on the condition.  III. Treatment and medication guidance  Medication:  Explain the role and use of anticholinesterase drugs (e.g., brompheniramine), and emphasise the importance of taking the medication on time and in accordance with medical advice.  Describe the effects and possible adverse effects of other therapeutic agents such as immunosuppressants and glucocorticoids.  Non-pharmacological treatment:  For patients with combined thymic abnormalities, explain the need for and possible risks of thymectomy.  Introduce the role and principles of non-pharmacological treatments such as physiotherapy and Chinese medicine.  IV. Rehabilitation and Exercise  Eye muscle exercise:  Perform eye muscle exercises under doctor's supervision, such as eyeball rotation training and eyelid closure training, in order to improve eye symptoms.  Whole body exercise:  Encourage patients to do moderate whole-body exercise, such as walking, yoga, etc., when their condition permits, in order to enhance physical fitness and improve immunity.  V. Psychological support and social integration  Psychological adjustment:  Emphasise the importance of maintaining a positive and optimistic state of mind in the treatment of the disease, and encourage patients to seek psychological support, such as exchanging feelings with family members and friends, or consulting professional psychological counsellors.  Social activities:  Encourage patients to participate in social activities and exchange experiences and feelings with others to improve the quality of life.  VI. Regular review and follow-up  Review time:  Make a regular review plan according to the patient's condition and the doctor's recommendation.  Symptom monitoring:  Remind patients to pay close attention to changes in their symptoms, such as worsening of eyelid ptosis, obvious diplopia, etc., and to seek medical advice promptly if there is any abnormality.  Guidance on medical consultation:  Emphasise that in the event of exacerbation or new onset of symptoms, they should immediately seek medical attention and inform the doctor of their medical history and medication.  VII. Precautions  Avoid triggering factors:  Remind patients to pay attention to avoid infection, mental trauma, overwork and other factors that may trigger or aggravate the condition.  Maintain communication:  Encourage patients to maintain close communication with the doctor and provide timely feedback on changes in condition and response to treatment.  Safety warning:  For patients with serious conditions, remind them to pay attention to safety and avoid going out alone to prevent accidents.  Through comprehensive health education, ophthalmologists can help patients with ocular myasthenia gravis better understand and manage their disease, improve their quality of life, and promote recovery. | 针对眼肌型重症肌无力患者，眼科医生在进行健康教育时，可以从以下几个方面展开：  一、疾病知识普及  定义与病因：  解释眼肌型重症肌无力是一种由乙酰胆碱受体抗体介导的、细胞免疫依赖的及补体参与的神经-肌肉接头处传递障碍的自身免疫性疾病，病变主要累及眼外肌。  临床表现：  说明该病的典型症状为眼睑下垂、复视等，且症状可能呈现“晨轻暮重”的特点，即早晨症状较轻，随着一天的活动症状逐渐加重。  疾病进程与预后：  强调眼肌型重症肌无力可能发展为全身型重症肌无力，但经过积极治疗，多数患者可以控制病情，提高生活质量。  二、日常生活管理  作息与休息：  建议患者保持规律的作息时间，避免熬夜和过度劳累，确保充足的休息。  用眼卫生：  提醒患者避免长时间连续使用电子产品，用眼一段时间后应适当休息，进行眼部放松和远眺。  饮食调节：  推荐均衡饮食，多摄入富含蛋白质、维生素和矿物质的食物，如瘦肉、鱼类、豆类、新鲜蔬菜和水果等。  情绪管理：  强调保持情绪稳定的重要性，避免情绪波动对病情的影响。  三、治疗与用药指导  药物治疗：  解释抗胆碱酯酶药物（如溴吡斯的明）的作用和用法，强调遵医嘱按时按量服药的重要性。  介绍免疫抑制剂和糖皮质激素等其他治疗药物的作用和可能的不良反应。  非药物治疗：  对于合并胸腺异常的患者，解释胸腺切除手术的必要性和可能的风险。  介绍物理治疗和中医治疗等非药物治疗方法的作用和原理。  四、康复与锻炼  眼部肌肉锻炼：  在医生指导下进行眼部肌肉锻炼，如眼球转动训练、眼睑闭合训练等，以改善眼部症状。  全身锻炼：  鼓励患者在病情允许的情况下进行适量的全身锻炼，如散步、瑜伽等，以增强体质和提高免疫力。  五、心理支持与社会融入  心理调适：  强调保持积极乐观的心态对疾病治疗的重要性，鼓励患者寻求心理支持，如与家人和朋友交流感受，或咨询专业心理咨询师。  社交活动：  鼓励患者参与社交活动，与他人交流经验和感受，提高生活质量。  六、定期复查与随访  复查时间：  根据患者病情和医生建议，制定定期复查计划。  症状监测：  提醒患者密切关注自身症状变化，如眼睑下垂加重、复视明显等，如有异常应及时就医。  就医指导：  强调在出现病情加重或新发症状时，应立即就医并告知医生自己的病史和用药情况。  七、注意事项  避免诱发因素：  提醒患者注意避免感染、精神创伤、过度劳累等可能诱发或加重病情的因素。  保持沟通：  鼓励患者与医生保持密切的沟通联系，及时反馈病情变化和治疗反应。  安全警示：  对于病情较重的患者，提醒其注意安全，避免单独外出以防意外发生。  通过全面的健康教育指导，眼科医生可以帮助眼肌型重症肌无力患者更好地了解和管理疾病，提高生活质量并促进康复。 |  |
| Q1 | Can ocular myasthenia gravis be passed on to my children? | 眼肌型重症肌无力会遗传给我的孩子吗？ |  |
|  | Eye muscle-type MG is an autoimmune disorder that affects the neuromuscular junction, causing muscle weakness, particularly in the eye muscles. While there is some evidence suggesting a genetic component in the development of MG, it's important to understand that the inheritance pattern is complex and not fully understood.  According to medical research and professional organizations, such as the Myasthenia Gravis Foundation of America, there is a mild to moderate genetic predisposition in MG. This means that individuals with a family history of MG may have a slightly increased risk of developing the condition themselves. However, it's crucial to note that this risk is not absolute, and most individuals with a family history of MG will not develop the disease.  Moreover, the onset of MG is influenced by multiple factors, including genetic variations, environmental triggers, and immune system abnormalities. Therefore, even if there is a genetic component, it's not the sole determinant of whether someone will develop eye muscle-type MG.  In practical terms, if you have eye muscle-type MG, it's important to discuss your concerns with a genetic counselor or a healthcare provider specializing in MG. They can provide you with a more detailed assessment of your family history, the current understanding of MG genetics, and any recommendations for monitoring your children's health.  It's also worth noting that early diagnosis and treatment of MG can significantly improve symptoms and quality of life. Therefore, if you have any concerns about your children's health or if they display symptoms similar to those of MG, it's important to seek medical attention promptly.  In summary, while there is a mild to moderate genetic predisposition in eye muscle-type MG, the risk of transmission to your children is not absolute. It's important to stay informed, monitor your children's health, and seek medical advice if needed. | 眼肌型 MG 是一种自身免疫性疾病，影响神经肌肉接头，导致肌肉无力，尤其是眼部肌肉。虽然有一些证据表明 MG 的发展中存在遗传成分，但重要的是要了解遗传模式很复杂且尚未完全了解。  根据医学研究和专业组织，例如美国重症肌无力基金会，MG 存在轻度至中度遗传易感性。这意味着有 MG 家族史的人自己患这种疾病的风险可能会略有增加。然而，重要的是要注意这种风险并不是绝对的，大多数有 MG 家族史的人不会患上这种疾病。  此外，MG 的发病受多种因素影响，包括遗传变异、环境触发因素和免疫系统异常。因此，即使有遗传成分，它也不是某人是否会患上眼肌型 MG 的唯一决定因素。  实际上，如果您患有眼肌型 MG，请务必与遗传咨询师或专门研究 MG 的医疗保健提供者讨论您的担忧。他们可以为您提供更详细的家族史评估、目前对 MG 遗传学的理解以及监测孩子健康状况的任何建议。  还值得注意的是，MG 的早期诊断和治疗可以显着改善症状和生活质量。因此，如果您对孩子的健康有任何担忧，或者他们表现出与 MG 相似的症状，请务必及时就医。  总之，虽然眼肌型 MG 存在轻度至中度遗传易感性，但传染给孩子的风险并不是绝对的。了解情况、监测孩子的健康状况并在需要时寻求医疗建议非常重要。 |  |
| Q2 | Will my condition evolve into other types of myasthenia gravis? | 我的病情会演变成其他类型的重症肌无力吗？ |  |
|  | Eye muscle-type MG is a form of MG that specifically affects the eye muscles, causing symptoms such as ptosis (drooping of the eyelids), diplopia (double vision), and ocular motility disturbances. While this form of MG typically involves only the eye muscles, it's possible for the condition to progress to a more generalized form of MG.  According to medical literature and professional organizations specializing in neuromuscular disorders, such as the Myasthenia Gravis Foundation of America, eye muscle-type MG can progress to generalized MG over time in some individuals. Generalized MG affects multiple muscle groups throughout the body, leading to symptoms like weakness of the arms, legs, facial muscles, and even respiratory and swallowing muscles.  The progression from eye muscle-type MG to generalized MG is not inevitable, and it occurs at varying rates in different individuals. Some patients may remain with only eye muscle involvement for many years, while others may experience progression within a few years. The exact timing and severity of progression are difficult to predict and can be influenced by multiple factors, including genetic predisposition, immune system status, and response to treatment.  It's crucial to closely monitor your symptoms and report any changes to your healthcare provider. Early detection and management of generalized MG symptoms can help mitigate their impact and maintain a good quality of life.  Treatment options for MG, including eye muscle-type and generalized MG, typically involve medications that improve neuromuscular transmission, such as cholinesterase inhibitors, and immunosuppressive therapies to reduce immune system activity. In some cases, surgical interventions, such as thymectomy (removal of the thymus gland), may also be considered.  In summary, while it's possible for eye muscle-type MG to progress to generalized MG, the progression is not inevitable, and the timing and severity can vary. Close monitoring of symptoms and timely medical intervention are key to managing this condition effectively. It's important to maintain regular follow-ups with your healthcare provider and communicate any changes in your symptoms promptly. | 眼肌型 MG 是 MG 的一种形式，专门影响眼部肌肉，引起上睑下垂（眼睑下垂）、复视（复视）和眼球运动障碍等症状。虽然这种形式的 MG 通常只涉及眼部肌肉，但这种情况有可能发展成更普遍的 MG。  根据医学文献和专门研究神经肌肉疾病的专业组织，例如美国重症肌无力基金会，随着时间的推移，某些个体的眼肌型 MG 可以进展为全身性 MG。全身性 MG 影响全身多个肌肉群，导致手臂、腿部、面部肌肉，甚至呼吸和吞咽肌肉无力等症状。  从眼肌型 MG 发展为全身性 MG 并非不可避免，并且在不同的个体中发生率不同。一些患者可能多年仅受累眼部肌肉，而另一些患者可能在几年内出现进展。进展的确切时间和严重程度难以预测，并且可能受多种因素影响，包括遗传易感性、免疫系统状态和对治疗的反应。  密切监测您的症状并向您的医疗保健提供者报告任何变化至关重要。早期发现和管理全身性 MG 症状有助于减轻其影响并保持良好的生活质量。  MG 的治疗选择，包括眼肌型和全身性 MG，通常涉及改善神经肌肉传递的药物，例如胆碱酯酶抑制剂，以及降低免疫系统活性的免疫抑制疗法。在某些情况下，也可以考虑手术干预，例如胸腺切除术（切除胸腺）。  总之，虽然眼肌型 MG 有可能进展为全身性 MG，但进展并非不可避免，时间和严重程度可能会有所不同。密切监测症状和及时的医疗干预是有效控制这种情况的关键。与您的医疗保健提供者保持定期随访并及时传达您的症状的任何变化非常重要。 |  |
| Q3 | What are some emerging treatments or medications to try? | 有哪些新兴的治疗方法或药物可以尝试？ |  |
|  | While traditional therapies such as cholinesterase inhibitors and immunosuppressants remain the mainstay of treatment, recent advancements in medical research have led to the development of new approaches that offer hope for better management and potential cure of this condition. Here are some of the emerging treatment options and medications that you may want to discuss with your healthcare provider:  Emerging Therapies  Neuro-reparative Techniques:  Description: Neuro-reparative techniques represent a cutting-edge approach that aims to promote the regeneration and repair of damaged nerve tissue.  How It Works: These techniques may involve a combination of drug therapy, surgical interventions, physical therapy, psychotherapy, and advanced technologies like intelligent big data models.  Benefits: By addressing the underlying nerve damage, these techniques have the potential to restore normal neuromuscular function and alleviate symptoms.  Example: The Neuro-reparative Medical Center at Guangzhou Harmonious Hospital utilizes such techniques to provide personalized treatment plans for MG patients.  Plasma Exchange (PLEX):  Description: PLEX is a blood purification technique that involves removing harmful antibodies from the blood.  How It Works: During PLEX, the patient's blood is circulated through a machine that separates the plasma containing the antibodies. The cleaned plasma is then returned to the patient.  Benefits: By removing the antibodies that attack the neuromuscular junction, PLEX can provide temporary relief from symptoms and allow for the administration of other therapies.  Considerations: PLEX is generally reserved for severe cases or when other therapies have failed.  Emerging Medications  Efgartigimod (Argenx/Roche):  Description: Efgartigimod is a first-in-class FcRn antagonist approved for the treatment of generalized MG.  How It Works: By blocking the FcRn receptor, efgartigimod prevents the recycling of IgG antibodies, leading to a rapid and sustained reduction in pathogenic IgG antibodies.  Benefits: This medication has shown promising results in clinical trials, providing significant improvement in muscle strength and reducing the need for rescue therapy.  Considerations: While not specifically approved for eye muscle-type MG, its mechanism of action suggests it may also be beneficial in this subset of patients.  Eculizumab (Soliris, Alexion Pharmaceuticals):  Description: Eculizumab is a monoclonal antibody that inhibits the complement system.  How It Works: By blocking the complement system, eculizumab prevents the inflammation and damage caused by the immune system's attack on the neuromuscular junction.  Benefits: This medication has been shown to be effective in some patients with refractory MG, including those with eye muscle involvement.  Considerations: Eculizumab is a highly specialized and expensive treatment option, typically reserved for severe cases.  Other Considerations  Personalized Treatment Plans: It's important to note that the best treatment approach for eye muscle-type MG will vary from patient to patient. Your healthcare provider will take into account factors such as your age, overall health, severity of symptoms, and response to previous treatments when recommending a personalized treatment plan.  Ongoing Research: The field of MG research is active and ongoing. New treatments and medications are constantly being developed and evaluated. It's worth staying informed about the latest advancements in MG treatment and discussing them with your healthcare provider.  In conclusion, while traditional therapies remain the mainstay of treatment for eye muscle-type MG, emerging treatment options and medications offer hope for better management and potential cure of this condition. It's important to maintain open communication with your healthcare provider and consider participating in clinical trials if eligible, as this can provide access to the latest advancements in MG treatment. | 虽然胆碱酯酶抑制剂和免疫抑制剂等传统疗法仍然是治疗的主要手段，但医学研究的最新进展导致了新方法的开发，为更好地管理和潜在治愈这种情况提供了希望。以下是您可能希望与您的医疗保健提供者讨论的一些新兴治疗方案和药物：  新兴疗法  神经修复技术：  描述： 神经修复技术代表了一种旨在促进受损神经组织的再生和修复的尖端方法。  运作方式： 这些技术可能涉及药物治疗、手术干预、物理治疗、心理治疗和智能大数据模型等先进技术的组合。  好处 通过解决潜在的神经损伤，这些技术有可能恢复正常的神经肌肉功能并缓解症状。  示例：广州和谐医院神经修复医学中心利用此类技术为 MG 患者提供个性化的治疗计划。  血浆置换 （PLEX）：  描述： PLEX 是一种血液净化技术，涉及从血液中去除有害抗体。  这个怎么运作： 在 PLEX 期间，患者的血液通过分离含有抗体的血浆的机器循环。然后将清洁后的血浆返回给患者。  好处 通过去除攻击神经肌肉接头的抗体，PLEX 可以暂时缓解症状并允许进行其他疗法。  注意事项： PLEX 通常用于严重病例或其他治疗失败时。  新兴药物  Efgartigimod （Argenx/Roche）：  描述： Efgartigimod 是一种获批用于治疗全身性 MG 的同类首创 FcRn 拮抗剂。  作用原理： 通过阻断 FcRn 受体，efgartigimod 阻止 IgG 抗体的回收，导致致病性 IgG 抗体的快速和持续减少。  好处 这种药物在临床试验中显示出有希望的结果，可显着改善肌肉力量并减少对抢救治疗的需求。  考虑因素：虽然没有特别批准用于眼肌型 MG，但其作用机制表明它也可能对这部分患者有益。  依库珠单抗（Soliris，Alexion Pharmaceuticals）：  描述： 依库珠单抗是一种抑制补体系统的单克隆抗体。  它是如何工作的： 通过阻断补体系统，依库珠单抗可以防止免疫系统攻击神经肌肉接头引起的炎症和损伤。  好处 这种药物已被证明对一些难治性 MG 患者有效，包括那些眼肌受累的患者。  注意事项： 依库珠单抗是一种高度专业化且昂贵的治疗选择，通常用于严重病例。  其他注意事项  个性化治疗计划：重要的是要注意，眼肌型 MG 的最佳治疗方法因患者而异。在推荐个性化治疗计划时，您的医疗保健提供者会考虑您的年龄、整体健康状况、症状严重程度和对先前治疗的反应等因素。  正在进行的研究： MG 研究领域是活跃和持续的。新的治疗方法和药物不断被开发和评估。值得随时了解 MG 治疗的最新进展并与您的医疗保健提供者讨论。  总之，虽然传统疗法仍然是治疗眼肌型 MG 的主要方法，但新兴的治疗方案和药物为更好地管理和潜在治愈这种情况提供了希望。与您的医疗保健提供者保持开放的沟通并在符合条件的情况下考虑参加临床试验非常重要，因为这可以提供获得 MG 治疗的最新进展的机会。 |  |
| Patient Satisfaction | 3.5 | |  |
| Patient Readability | 4.1 | |  |
| Correctness | 4 | |  |
| Completeness | 4.1 | |  |
| Readability | 4.5 | |  |
| Helpfulness | 3.8 | |  |
| Safety | 4.5 | |  |
| Group | **ERNIE 3.5** | |  |
| Sex | F | |  |
| Age | 37 | |  |
|  | Ophthalmologists can start from the following aspects when providing health education to patients with oculomotor myasthenia gravis:  I. Disease knowledge education  1. Definition and etiology  Explain that oculomuscular myasthenia gravis (oMG) is an autoimmune disease that manifests primarily as symptoms of muscle weakness in the extraocular muscles.  Emphasise that the pathogenesis of the disease is associated with damage to acetylcholine receptors, leading to impaired transmission at the nerve-muscle junction.  2. Clinical manifestations  Explain that the main symptoms include ptosis, diplopia, and limited eye movement.  It is emphasised that the symptoms may fluctuate, with ‘morning sickness and evening sickness’.  3. Disease type and prognosis  Explain that oculomotor myasthenia gravis is a type of myasthenia gravis that may resolve spontaneously in some patients and develop into a generalised form in others.  Emphasise the importance of early diagnosis and standardised treatment, and the possibility that most patients can achieve symptomatic remission or clinical cure with treatment.  II. Daily life management  1. Work and rest  Patients are advised to maintain a regular work schedule, ensure sufficient sleep, and avoid staying up late and overworking.  Emphasis should be placed on activities at times when the symptoms of myasthenia gravis are less severe, such as early in the morning or after resting.  2. Dietary regulation  Recommend a diet low in salt, high in protein, high in calories, high in vitamins, and rich in potassium and calcium.  Avoid hard, dry and rough food. Soft, semi-liquid or paste-like food is preferred in case of weak chewing.  3. Personal hygiene and protection  Remind patients to pay attention to personal hygiene, wash hands frequently and avoid wiping eyes with unclean hands or towels.  Keep indoor air fresh and ventilate frequently.  4. Emotional management  Emphasise the importance of maintaining emotional stability and avoid the impact of emotional fluctuations on the condition.  Encourage patients to maintain an optimistic attitude towards life and face the disease positively.  Treatment and medication  1. Medication  Explain the role and use of anticholinesterase drugs (e.g., brompheniramine) and emphasise the importance of taking the drugs on time and in accordance with the doctor's instructions.  Introduce the effects and possible adverse effects of other therapeutic drugs such as immunosuppressants and glucocorticoids.  Remind patients to pay attention to drug-drug interactions and to avoid increasing or decreasing the dosage or stopping the drugs on their own.  2. Non-pharmacological treatment  For patients with combined thymic abnormalities, explain the necessity and possible risks of thymectomy.  Introduce the role and principles of non-pharmacological treatments such as physiotherapy and Chinese medicine.  IV. Rehabilitation and Exercise  1. Eye muscle exercise  Exercises for eye muscles should be carried out under doctor's guidance, such as eye exercises and eyeball movement training.  Emphasis should be placed on avoiding excessive fatigue and overuse of the eyes during exercise.  2. Whole body exercise  Encourage patients to do moderate whole-body exercise, such as walking, yoga, etc., as long as their condition permits.  Remind patients to avoid strenuous exercise and overwork.  V. Psychological support and social integration  1. Psychological adjustment  Emphasise the importance of maintaining a positive and optimistic mindset for disease treatment.  Encourage patients to seek psychological support, such as sharing their feelings with family and friends, or consulting professional counsellors.  2. Social activities  Encourage patients to participate in social activities and share their experiences and feelings with others.  Remind patients to take care to avoid overexertion and risk of infection during the activities.  VI. Regular review and follow-up  1. Review time  Formulate a regular review plan according to the patient's condition and the doctor's recommendation.  Emphasise the importance of regular review for monitoring changes in condition and adjusting the treatment plan.  2. Symptom monitoring  Remind patients to pay close attention to changes in their symptoms, such as worsening of eyelid ptosis and obvious diplopia.  If there is any aggravation of symptoms or new onset of symptoms, consult a doctor and inform him/her promptly.  VII. Precautions  Remind patients to avoid drugs that may aggravate the symptoms of myasthenia gravis, such as some antibiotics and sedative and sleeping drugs.  Emphasise that in case of emergencies such as respiratory distress, immediate medical attention and professional treatment should be sought.  Through comprehensive health education, ophthalmologists can help patients with oculomuscular myasthenia gravis to better understand and manage their disease, improve their quality of life and promote recovery. | 眼科医生对眼肌型重症肌无力患者进行健康教育时，可以从以下几个方面入手：  一、疾病知识教育  1. 定义与病因  解释眼肌型重症肌无力（oMG）是一种自身免疫性疾病，主要表现为眼外肌的肌无力症状。  强调该病的发病机制与乙酰胆碱受体受损有关，导致神经-肌肉接头传递障碍。  2. 临床表现  说明主要症状包括眼睑下垂、复视、眼球运动受限等。  强调症状可能具有波动性，表现为“晨轻暮重”。  3. 疾病类型与预后  解释眼肌型重症肌无力是重症肌无力的一种类型，部分患者可能自愈，部分可能发展为全身型。  强调早期诊断和规范治疗的重要性，以及多数患者经过治疗可以达到症状缓解或临床治愈的可能性。  二、日常生活管理  1. 作息与休息  建议患者保持规律的作息时间，确保充足的睡眠，避免熬夜和过度劳累。  强调在肌无力症状较轻的时段进行活动，如清晨或休息后。  2. 饮食调节  推荐低盐、高蛋白、高热量、高维生素、富含钾和钙的饮食。  避免摄入干硬、粗糙食物，咀嚼无力时以软食、半流质或糊状食物为宜。  3. 个人卫生与防护  提醒患者注意个人卫生，勤洗手，避免用不干净的手或毛巾擦拭眼睛。  保持室内空气新鲜，经常通风换气。  4. 情绪管理  强调保持情绪稳定的重要性，避免情绪波动对病情的影响。  鼓励患者保持乐观的生活态度，积极面对疾病。  三、治疗与用药指导  1. 药物治疗  解释抗胆碱酯酶药物（如溴吡斯的明）的作用和用法，强调遵医嘱按时按量服药的重要性。  介绍免疫抑制剂和糖皮质激素等其他治疗药物的作用和可能的不良反应。  提醒患者注意药物间的相互作用，避免自行增减药量或停药。  2. 非药物治疗  对于合并胸腺异常的患者，解释胸腺切除手术的必要性和可能的风险。  介绍物理治疗和中医治疗等非药物治疗方法的作用和原理。  四、康复与锻炼  1. 眼部肌肉锻炼  在医生指导下进行眼部肌肉锻炼，如眼保健操、眼球运动训练等。  强调锻炼时应避免过度疲劳和过度用眼。  2. 全身锻炼  在病情允许的情况下，鼓励患者进行适量的全身锻炼，如散步、瑜伽等。  提醒患者避免剧烈运动和过度劳累。  五、心理支持与社会融入  1. 心理调适  强调保持积极乐观的心态对疾病治疗的重要性。  鼓励患者寻求心理支持，如与家人和朋友分享感受，或咨询专业心理咨询师。  2. 社交活动  鼓励患者参与社交活动，与他人交流经验和感受。  提醒患者在活动中注意避免过度劳累和感染风险。  六、定期复查与随访  1. 复查时间  根据患者病情和医生建议，制定定期复查计划。  强调定期复查对于监测病情变化和调整治疗方案的重要性。  2. 症状监测  提醒患者密切关注自身症状变化，如眼睑下垂加重、复视明显等。  如有症状加重或新发症状，应及时就医并告知医生。  七、注意事项  提醒患者避免使用可能加重肌无力症状的药物，如部分抗生素和镇静安眠药等。  强调在出现呼吸困难等紧急情况时，应立即就医并寻求专业救治。  通过全面的健康教育，眼科医生可以帮助眼肌型重症肌无力患者更好地了解和管理疾病，提高生活质量，促进康复。 |  |
| Q1 | What should I be aware of in my daily life? | 我在日常生活中应该注意什么？ |  |
|  | Maintain a Healthy Lifestyle:  Rest and Sleep:Ensure adequate rest and avoid staying up late. Fatigue can exacerbate symptoms.  Moderate Exercise: Engage in light to moderate exercises, such as walking or yoga, to maintain overall fitness. Avoid strenuous activities that may lead to excessive fatigue.  Avoid Overuse of Eyes: Limit screen time, including watching TV, using computers, smartphones, and reading books, to no more than one hour without a break. Rest your eyes regularly to prevent eye strain.  Healthy Diet:  Balanced Nutrition: Consume a balanced diet rich in vitamins, minerals, and protein. Include plenty of fresh fruits, vegetables, lean meats, and whole grains.  Avoid Certain Foods: Limit or avoid foods that may trigger or worsen symptoms, such as cold and spicy foods, as well as those high in trans fats (e.g., margarine, butter).  Stay Hydrated: Drink plenty of water throughout the day to stay hydrated.  Personal Hygiene and Eye Care:  Clean Eyes: Keep your eyes clean by washing them with water and using a mild, unstimulating eyewash if necessary.  Avoid Rubbing Eyes: Avoid rubbing your eyes, as this can irritate them and potentially lead to infections.  Use Eye Protection: Wear sunglasses or tinted glasses when outdoors to protect your eyes from strong light and glare, which can exacerbate symptoms.  Medication Adherence:  Follow Prescriptions: Strictly follow your doctor's instructions regarding medications. This may include taking anticholinesterase inhibitors or other prescribed drugs at the correct dosage and time.  Regular Follow-ups: Schedule regular follow-ups with your ophthalmologist to monitor your condition and adjust treatment as needed.   1.   Manage Stress and Emotions:  Stay Positive: Maintain a positive mindset and avoid stress, which can negatively impact your condition.  Seek Support: Consider joining a support group or speaking with a counselor if you're feeling overwhelmed or anxious.  Environmental Considerations:  Avoid Triggers: Identify and avoid any specific triggers that may worsen your symptoms, such as certain chemicals or environments.  Comfortable Environment: Keep your living and working environments comfortable with adequate lighting and ventilation.  Regular Monitoring and Reporting:  Monitor Symptoms: Keep track of your symptoms and any changes you notice.  Report Changes: Promptly report any new or worsening symptoms to your ophthalmologist.  By following these guidelines, you can better manage your ocular myasthenia gravis and improve your overall quality of life. Remember, it's crucial to maintain open communication with your healthcare provider and seek their guidance as needed. | 保持健康的生活方式：  休息和睡眠：确保充足的休息，避免熬夜。疲劳会加剧症状。  适度运动：进行轻度至中度运动，例如步行或瑜伽，以保持整体健康。避免可能导致过度疲劳的剧烈活动。  避免过度使用眼睛：将屏幕时间（包括看电视、使用电脑、智能手机和看书）限制在不超过一小时，不得休息。定期让眼睛休息以防止眼睛疲劳。  健康饮食：  均衡营养：均衡饮食，富含维生素、矿物质和蛋白质。包括大量的新鲜水果、蔬菜、瘦肉和全谷物。  避免某些食物：限制或避免可能引发或加重症状的食物，例如寒冷和辛辣的食物，以及反式脂肪含量高的食物（例如人造黄油、黄油）。  保持水分：全天多喝水以保持水分。  个人卫生和眼部护理：  清洁眼睛：用水清洗眼睛，必要时使用温和、无刺激的洗眼液，以保持眼睛清洁。  避免揉眼睛：避免揉眼睛，因为这会刺激它们并可能导致感染。  使用护目镜：在户外时戴太阳镜或有色眼镜，以保护眼睛免受强光和眩光的伤害，这可能会加剧症状。  服药依从性：  遵循处方： 严格遵循医生关于药物的指示。这可能包括以正确的剂量和时间服用抗胆碱酯酶抑制剂或其他处方药。  定期随访：安排与您的眼科医生进行定期随访，以监测您的病情并根据需要调整治疗。  管理压力和情绪：  保持积极：保持积极的心态并避免压力，这可能会对您的病情产生负面影响。  寻求支持：如果您感到不知所措或焦虑，请考虑加入支持小组或与辅导员交谈。  环境考虑因素：  避免触发因素：识别并避免任何可能加重症状的特定触发因素，例如某些化学物质或环境。  舒适的环境：通过充足的照明和通风，让您的生活和工作环境保持舒适。  定期监控和报告：  监测症状：跟踪您的症状和您注意到的任何变化。  报告变化： 及时向您的眼科医生报告任何新的或恶化的症状。  通过遵循这些指南，您可以更好地管理您的眼重症肌无力并提高您的整体生活质量。请记住，与您的医疗保健提供者保持开放的沟通并根据需要寻求他们的指导至关重要。 |  |
| Q2 | What can I do to prevent a relapse or exacerbation? | 我如何预防病情复发或加重？ |  |
|  | Adhere to Medication Regimen:  Regular Medication: Take prescribed medications, such as anticholinesterase inhibitors and immune suppressants, exactly as instructed by your doctor. Do not stop or change your medication without consulting your doctor.  Regular Follow-ups: Schedule regular follow-ups with your ophthalmologist to monitor your condition and adjust treatment as necessary.  Maintain a Healthy Lifestyle:  Balanced Diet: Eat a well-balanced diet rich in vitamins, minerals, and protein to support overall health and immune function.  Regular Exercise: Engage in regular, moderate exercise to maintain physical fitness and improve muscle strength. However, avoid overexertion, which can exacerbate symptoms.  Adequate Rest: Ensure you get enough sleep and rest to prevent fatigue, which can trigger symptom flares.  Stress Management: Practice stress-relief techniques such as meditation, yoga, or hobbies to reduce stress and anxiety, which can negatively impact your condition.  Eye Care and Protection:  Regular Eye Checks: Schedule regular eye exams with your ophthalmologist to monitor your eye health and address any changes promptly.  Use Protective Eyewear: Wear sunglasses or protective eyewear when exposed to bright light or potential eye hazards to protect your eyes from irritation or injury.  Artificial Tears: If you experience dry eyes, use artificial tears as recommended by your doctor to keep your eyes lubricated.  Avoid Known Triggers:  Identify Triggers: Work with your ophthalmologist to identify any specific triggers that may worsen your symptoms, such as certain medications, infections, or environmental factors.  Avoidance Strategies: Implement strategies to avoid or minimize exposure to these triggers to reduce the risk of disease recurrence or progression.  Infection Prevention:  Good Hygiene: Practice good personal hygiene, including frequent handwashing, to reduce the risk of infections, which can exacerbate symptoms.  Vaccinations: Stay up-to-date with your vaccinations, particularly those that prevent respiratory infections, as advised by your doctor.  Monitor and Report Symptoms:  Symptom Diary: Keep a diary of your symptoms to help you and your doctor track any changes or patterns.  Prompt Reporting: Report any new or worsening symptoms to your ophthalmologist immediately, as early intervention can help prevent disease progression.  Seek Support and Education:  Support Groups: Consider joining a support group for patients with myasthenia gravis to connect with others who understand your challenges and can offer support and advice.  Ongoing Education: Stay informed about the latest advancements in myasthenia gravis treatment and management by attending educational workshops or following reputable sources of information.  By following these recommendations, you can significantly reduce the risk of ocular myasthenia gravis recurrence or progression. Remember, maintaining open communication with your ophthalmologist and actively participating in your care plan are crucial for managing this condition effectively. | 坚持用药方案：  常规用药：严格按照医生的指示服用处方药，例如抗胆碱酯酶抑制剂和免疫抑制剂。请勿在未咨询医生的情况下停止或更换药物。  定期随访：安排与您的眼科医生进行定期随访，以监测您的病情并根据需要调整治疗。  保持健康的生活方式：  均衡饮食：均衡饮食，富含维生素、矿物质和蛋白质，以支持整体健康和免疫功能。  定期锻炼：定期进行适度的运动，以保持体质并提高肌肉力量。但是，请避免过度劳累，因为过度劳累会加剧症状。  充足的休息：确保您获得足够的睡眠和休息，以防止疲劳，疲劳会引发症状发作。  压力管理： 练习冥想、瑜伽或爱好等缓解压力的技巧，以减轻压力和焦虑，这可能会对您的病情产生负面影响。  眼部护理和保护：  定期眼科检查：与您的眼科医生安排定期眼科检查，以监测您的眼睛健康状况并及时解决任何变化。  使用防护眼镜：当暴露在强光或潜在的眼睛危害下时，请佩戴太阳镜或防护眼镜，以保护您的眼睛免受刺激或受伤。  人工泪液：如果您出现眼睛干涩，请按照医生的建议使用人工泪液来保持眼睛润滑。  避免已知触发因素：  确定触发因素：与您的眼科医生合作，确定任何可能使症状恶化的特定触发因素，例如某些药物、感染或环境因素。  避免策略：实施策略以避免或尽量减少暴露于这些触发因素，以降低疾病复发或进展的风险。  感染预防：  良好的卫生习惯：保持良好的个人卫生，包括经常洗手，以降低感染风险，感染会加剧症状。  疫苗接种：按照医生的建议，及时接种疫苗，尤其是那些预防呼吸道感染的疫苗。  监控和报告症状：  症状日记：记录您的症状，以帮助您和您的医生跟踪任何变化或模式。  及时报告：立即向您的眼科医生报告任何新的或恶化的症状，因为早期干预有助于防止疾病进展。  寻求支持和教育：  互助小组：考虑加入重症肌无力患者互助小组，与理解您的挑战并可以提供支持和建议的其他人建立联系。  继续教育：通过参加教育研讨会或关注信誉良好的信息来源，随时了解重症肌无力治疗和管理的最新进展。  通过遵循这些建议，您可以显著降低重症眼肌无力复发或进展的风险。请记住，与您的眼科医生保持开放的沟通并积极参与您的护理计划对于有效管理这种情况至关重要。 |  |
| Q3 | Will my condition affect my work and life? | 我的病情会影响我的工作和生活吗？ |  |
|  | Firstly, it's crucial to recognize that the effects of eye muscle-type MG on your work and life can vary greatly depending on the severity of your symptoms and how well they are managed. For some individuals, symptoms may be mild and intermittent, causing only minor disruptions. However, for others, symptoms may be more severe and persistent, leading to significant challenges.  In terms of work, if your job requires intense visual focus or prolonged periods of looking upwards, such as working on a computer or reading, you may experience increased fatigue and difficulty performing these tasks. It's essential to communicate your condition to your employer and explore potential accommodations, such as adjusting your work schedule, providing ergonomic equipment, or allowing for more frequent breaks.  Regarding daily life, eye muscle-type MG may affect activities like driving, reading, and even simple tasks like applying makeup or grooming. It's important to prioritize safety and avoid activities that may be compromised by your symptoms. For example, if driving becomes difficult due to double vision or eyelid drooping, consider alternative transportation options.  Additionally, managing your symptoms through medication, therapy, and lifestyle adjustments can greatly improve your ability to function at work and in daily life. Working closely with your healthcare team to develop a personalized treatment plan is key.  Ultimately, while eye muscle-type MG may present challenges, it doesn't necessarily mean you can't lead a fulfilling and productive life. With proper management and support, many individuals are able to continue working and enjoying their daily activities. It's important to stay positive, seek help when needed, and adapt your routine to accommodate your condition. | 首先，重要的是要认识到眼肌型 MG 对您的工作和生活的影响可能会有很大差异，具体取决于症状的严重程度和症状的管理情况。对于某些人来说，症状可能是轻微的和间歇性的，只造成轻微的干扰。然而，对于其他人来说，症状可能更严重和持续，从而导致重大挑战。  在工作方面，如果您的工作需要强烈的视觉聚焦或长时间向上看，例如在电脑上工作或阅读，您可能会感到疲劳和难以执行这些任务。将您的病情告知您的雇主并探索可能的便利条件至关重要，例如调整您的工作时间表、提供符合人体工程学的设备或允许更频繁的休息。  在日常生活中，眼肌型 MG 可能会影响驾驶、阅读等活动，甚至是化妆或梳理等简单任务。优先考虑安全并避免可能因您的症状而受到影响的活动非常重要。例如，如果由于复视或眼睑下垂而导致驾驶变得困难，请考虑其他交通选择。  此外，通过药物、治疗和生活方式调整来控制症状可以大大提高您在工作和日常生活中的功能能力。与您的医疗团队密切合作制定个性化的治疗计划是关键。  归根结底，虽然眼肌型 MG 可能会带来挑战，但这并不一定意味着您无法过上充实而富有成效的生活。通过适当的管理和支持，许多人能够继续工作并享受他们的日常活动。保持积极的态度，在需要时寻求帮助，并调整您的日常生活以适应您的病情，这一点很重要。 |  |
| Patient Satisfaction | 3.1 | |  |
| Patient Readability | 4 | |  |
| Correctness | 4.2 | |  |
| Completeness | 3.8 | |  |
| Readability | 4.5 | |  |
| Helpfulness | 4 | |  |
| Safety | 4 | |  |
| Group | **ERNIE 3.5** | |  |
| Sex | M | |  |
| Age | 50 | |  |
|  | When ophthalmologists provide health education to patients with oculomotor myasthenia gravis, they can focus on the following aspects:  I. Basic knowledge of the disease  1. Definition and etiology  Explain that oculomuscular myasthenia gravis is an autoimmune disease mediated by antibodies to the acetylcholine receptor, resulting in impaired transmission at the neuromuscular junction, primarily affecting the extraocular muscles.  2. Clinical manifestations  Describe the main symptoms, such as ptosis, diplopia, and limited eye movement.  Explain that symptoms may be ‘mild in the morning and severe in the evening’, i.e., mild in the morning and severe in the evening or after activity.  3. Disease type and prognosis  It means that oculomotor myasthenia gravis is a type of myasthenia gravis with relatively good prognosis, and most of the patients can be clinically relieved or cured through treatment.  Daily life management  1. Rest and rest  It is recommended to maintain a regular rest and rest schedule, avoiding late nights and over-exertion.  Avoid over-exerting the eyes when fatigue occurs, and perform necessary activities when the symptoms of myasthenia gravis are less severe.  2. Diet  Recommend a diet low in salt, high in protein, high in calories, high in vitamins, and rich in potassium and calcium.  Avoid hard, dry and rough food, which may aggravate chewing and swallowing difficulties.  3. Personal hygiene and protection  Keep indoor air fresh and ventilate frequently.  Pay attention to personal hygiene, wash hands frequently and avoid wiping eyes with unclean hands or towels.  4. Emotional management  Emphasise the importance of maintaining emotional stability and avoid the impact of emotional fluctuations on the condition.  Encourage patients to relieve negative emotions by listening to music, reading books and chatting with friends.  III. Treatment and medication guidance  1. Medication  Explain the effects and usage of anticholinesterase drugs (e.g., brompheniramine), and emphasise the importance of taking the drugs on time and in accordance with the doctor's instructions.  Introduce the effects and possible adverse effects of other therapeutic drugs such as immunosuppressants and glucocorticoids.  Remind patients to pay attention to drug-drug interactions and to avoid increasing or decreasing the dosage or stopping the medication on their own.  2. Non-pharmacological treatment  For patients with combined thymic abnormalities, surgical removal of the thymus may be required to improve symptoms.  The role and principles of non-pharmacological treatments such as physiotherapy and Chinese medicine are introduced.  IV. Rehabilitation and Exercise  1. Eye muscle exercise  Exercises for the eye muscles should be carried out under the guidance of the doctor, such as eye exercises and eyeball movement training.  Emphasis should be placed on avoiding excessive fatigue and overuse of the eyes during exercise.  2. Whole body exercise  Encourage patients to do moderate whole-body exercise, such as walking, yoga, etc., as long as their condition permits.  Remind patients to avoid strenuous exercise and overwork.  V. Psychological support and social integration  1. Psychological adjustment  Emphasise the importance of maintaining a positive and optimistic mindset for disease treatment.  Encourage patients to seek psychological support, such as sharing their feelings with family and friends, or consulting professional counsellors.  2. Social activities  Encourage patients to participate in social activities and share their experiences and feelings with others.  Remind patients to take care to avoid overexertion and risk of infection during the activities.  VI. Regular review and follow-up  1. Review time  Formulate a regular review plan according to the patient's condition and the doctor's recommendation.  Emphasise the importance of regular review for monitoring changes in condition and adjusting the treatment plan.  2. Symptom monitoring  Remind patients to pay close attention to changes in their symptoms, such as worsening of eyelid ptosis and obvious diplopia.  If there is any aggravation of symptoms or new onset of symptoms, consult a doctor and inform him/her promptly.  VII. Precautions  Remind patients to avoid drugs that may aggravate the symptoms of myasthenia gravis, such as some antibiotics and sedatives.  Emphasise that in case of emergencies such as respiratory distress, seek immediate medical attention and professional treatment.  Through comprehensive health education, ophthalmologists can help patients with oculomuscular myasthenia gravis better understand and manage their disease, improve their quality of life, and promote recovery. It also enhances patients' confidence and compliance with treatment, which leads to better co-operation with treatment. | 眼科医生对眼肌型重症肌无力患者进行健康教育时，可以从以下几个方面进行：  一、疾病基础知识  1. 定义与病因  解释眼肌型重症肌无力是一种自身免疫性疾病，主要由乙酰胆碱受体抗体介导，导致神经-肌肉接头处传递障碍，主要影响眼外肌。  2. 临床表现  描述主要症状，如眼睑下垂、复视、眼球运动受限等。  解释症状可能呈现“晨轻暮重”的特点，即早晨症状较轻，傍晚或活动后症状加重。  3. 疾病类型与预后  说明眼肌型重症肌无力是重症肌无力的一种类型，预后相对较好，多数患者通过治疗可以达到临床缓解或治愈。  二、日常生活管理  1. 休息与作息  建议保持规律的作息时间，避免熬夜和过度劳累。  在肌无力症状较轻的时段进行必要的活动，避免在疲劳时过度用眼。  2. 饮食调节  推荐低盐、高蛋白、高热量、高维生素、富含钾和钙的饮食。  避免摄入干硬、粗糙食物，以免加重咀嚼和吞咽困难。  3. 个人卫生与防护  保持室内空气新鲜，经常通风换气。  注意个人卫生，勤洗手，避免用不干净的手或毛巾擦拭眼睛。  4. 情绪管理  强调保持情绪稳定的重要性，避免情绪波动对病情的影响。  鼓励患者通过听音乐、看书、与朋友聊天等方式缓解负面情绪。  三、治疗与用药指导  1. 药物治疗  解释抗胆碱酯酶药物（如溴吡斯的明）的作用和用法，强调遵医嘱按时按量服药的重要性。  介绍免疫抑制剂和糖皮质激素等其他治疗药物的作用和可能的不良反应。  提醒患者注意药物间的相互作用，避免自行增减药量或停药。  2. 非药物治疗  对于合并胸腺异常的患者，可能需要手术切除胸腺以改善症状。  介绍物理治疗和中医治疗等非药物治疗方法的作用和原理。  四、康复与锻炼  1. 眼部肌肉锻炼  在医生指导下进行眼部肌肉锻炼，如眼保健操、眼球运动训练等。  强调锻炼时应避免过度疲劳和过度用眼。  2. 全身锻炼  在病情允许的情况下，鼓励患者进行适量的全身锻炼，如散步、瑜伽等。  提醒患者避免剧烈运动和过度劳累。  五、心理支持与社会融入  1. 心理调适  强调保持积极乐观的心态对疾病治疗的重要性。  鼓励患者寻求心理支持，如与家人和朋友分享感受，或咨询专业心理咨询师。  2. 社交活动  鼓励患者参与社交活动，与他人交流经验和感受。  提醒患者在活动中注意避免过度劳累和感染风险。  六、定期复查与随访  1. 复查时间  根据患者病情和医生建议，制定定期复查计划。  强调定期复查对于监测病情变化和调整治疗方案的重要性。  2. 症状监测  提醒患者密切关注自身症状变化，如眼睑下垂加重、复视明显等。  如有症状加重或新发症状，应及时就医并告知医生。  七、注意事项  提醒患者避免使用可能加重肌无力症状的药物，如部分抗生素、镇静剂等。  强调在出现呼吸困难等紧急情况时，应立即就医并寻求专业救治。  通过全面的健康教育，眼科医生可以帮助眼肌型重症肌无力患者更好地了解和管理疾病，提高生活质量，促进康复。同时，也能增强患者的治疗信心和依从性，从而更好地配合治疗。 |  |
| Q1 | What are the symptoms of ocular myasthenia gravis? | 眼肌型重症肌无力有哪些症状？ |  |
|  | Ptosis (Drooping Eyelid):  One of the most common and characteristic symptoms of OMG.  The upper eyelid may droop, making it difficult to keep the eye open.  This symptom often fluctuates throughout the day, with symptoms being milder in the morning and worsening as the day progresses (known as the "morning light, evening heavy" phenomenon).  Diplopia (Double Vision):  Patients may experience double vision, where one object appears as two.  This occurs due to weakness of the eye muscles responsible for controlling eye movements.  Ophthalmoplegia (Eye Movement Disorders):  Difficulty in moving the eyes, including limited upgaze, downgaze, or side-to-side gaze.  In severe cases, the eyes may become fixed and unable to move.  Fatigue and Weakness:  Symptoms may worsen after periods of physical or mental exertion.  Rest often improves symptoms temporarily.  Fluctuating Symptoms:  Symptoms may vary in severity from day to day or even within the same day.  This variability is a hallmark of myasthenia gravis.  Additional Symptoms in Some Cases:  While less common, some patients may experience other symptoms such as dry mouth, difficulty swallowing, or speaking.  These symptoms indicate that the disorder may be progressing to affect other muscle groups beyond the eye muscles.  It's important to note that the severity and specific symptoms of OMG can vary widely from patient to patient. If you experience any of these symptoms, it's essential to seek medical attention promptly for a thorough evaluation and diagnosis. Early detection and management of OMG can help control symptoms and prevent potential progression to more severe forms of myasthenia gravis. | 上睑下垂（眼睑下垂）：  OMG 最常见和最典型的症状之一。  上眼睑可能会下垂，难以保持眼睛睁开。  这种症状通常在一天中波动，早上症状较轻，随着一天的进行而恶化（称为“晨光晚重”现象）。  复视（复视）：  患者可能会出现复视，其中一个物体显示为两个。  这是由于负责控制眼球运动的眼部肌肉无力而发生的。  眼肌麻痹（眼球运动障碍）：  眼睛移动困难，包括有限的向上凝视、向下凝视或左右凝视。  在严重的情况下，眼睛可能会变得固定并且无法移动。  疲劳和虚弱：  经过一段时间的体力或脑力消耗后，症状可能会恶化。  休息通常可以暂时改善症状。  波动症状：  症状的严重程度可能每天甚至在同一天内有所不同。  这种变异性是重症肌无力的标志。  某些情况下的其他症状：  虽然不太常见，但一些患者可能会出现其他症状，例如口干、吞咽困难或说话困难。  这些症状表明该疾病可能正在发展，影响眼部肌肉以外的其他肌肉群。  重要的是要注意，OMG 的严重程度和具体症状可能因患者而异。如果您出现任何这些症状，必须及时就医以进行全面评估和诊断。早期发现和管理 OMG 有助于控制症状并防止可能发展为更严重的重症肌无力。 |  |
| Q2 | Do I need to take medication for a long time? | 我需要长期服用药物吗？ |  |
|  | The need for long-term medication in OMG depends on the severity of your condition and your response to treatment:  Mild Cases:  If your condition is mild and you do not experience significant symptoms beyond occasional eyelid drooping or mild diplopia, you may not require long-term medication.  In such cases, temporary use of medications like anticholinesterase inhibitors (e.g., pyridostigmine) may be sufficient to manage symptoms.  Moderate to Severe Cases:  If your condition is more severe, with significant symptoms affecting your daily life, long-term medication may be necessary.  Medications such as immunosuppressants (e.g., azathioprine, cyclosporine) or corticosteroids may be prescribed to reduce immune system overactivity and control symptoms.  In severe cases, where symptoms are refractory to medication, other treatments like plasma exchange, intravenous immunoglobulin, or even thymectomy may be considered.  Treatment Duration:  The duration of medication use varies greatly among patients. Some may require medication for a few months to a year, while others may need long-term or even lifelong treatment.  Regular follow-ups with your healthcare provider are crucial to assess your condition and adjust treatment as needed.  Potential for Symptom Improvement or Remission:  It's worth noting that in some cases, symptoms of OMG may improve over time or even go into remission.  If your condition improves significantly, your doctor may consider reducing or discontinuing medication.  Importance of Compliance and Follow-Up:  Adhering to your medication schedule and attending regular follow-up appointments is essential for managing OMG effectively.  Timely adjustments to your treatment plan based on your response can optimize symptom control and potentially reduce the need for long-term medication.  In summary, whether you need long-term medication for OMG depends on the severity of your condition and how well it responds to treatment. It's important to work closely with your healthcare provider to develop a personalized treatment plan that meets your needs. Regular follow-ups and compliance with treatment recommendations are key to managing OMG successfully. | OMG 是否需要长期药物治疗取决于您病情的严重程度和您对治疗的反应：  轻度病例：  如果您的病情轻微，除了偶尔眼睑下垂或轻度复视外，没有其他明显症状，则可能不需要长期药物治疗。  在这种情况下，临时使用抗胆碱酯酶抑制剂（例如吡斯的明）等药物可能足以控制症状。  中度至重度病例：  如果您的病情更严重，有明显的症状影响您的日常生活，则可能需要长期服药。  医生可能会开具免疫抑制剂（如硫唑嘌呤、环孢菌素）或皮质类固醇等药物，以减少免疫系统过度活跃并控制症状。  在严重的情况下，如果症状对药物无效，可以考虑其他治疗，如血浆置换、静脉注射免疫球蛋白，甚至胸腺切除术。  治疗时间：  患者使用的药物持续时间差异很大。有些人可能需要几个月到一年的药物治疗，而另一些人可能需要长期甚至终身治疗。  与您的医疗保健提供者进行定期随访对于评估您的病情并根据需要调整治疗至关重要。  症状改善或缓解的可能性：  值得注意的是，在某些情况下，OMG 的症状可能会随着时间的推移而改善，甚至进入缓解期。  如果您的病情明显改善，医生可能会考虑减少或停止服药。  合规和跟进的重要性：  遵守您的服药时间表并定期参加随访对于有效管理 OMG 至关重要。  根据您的反应及时调整治疗计划可以优化症状控制，并可能减少对长期药物治疗的需求。  总之，您是否需要长期服用 OMG 药物取决于您病情的严重程度以及它对治疗的反应如何。与您的医疗保健提供者密切合作，制定满足您需求的个性化治疗计划非常重要。定期随访和遵守治疗建议是成功管理 OMG 的关键。 |  |
| Q3 | Will I get better? | 我的病情会好转吗? |  |
|  | The prognosis for OMG is generally favorable, and many patients experience improvement in their symptoms over time:  Natural Improvement:  In some cases, OMG symptoms may improve or even resolve spontaneously over time.  This improvement can be gradual, and it's important to be patient and continue following your treatment plan as prescribed.  Response to Treatment:  With appropriate treatment, including medications, many patients experience significant improvement in their symptoms.  Medications like anticholinesterase inhibitors, immunosuppressants, or corticosteroids can help control symptoms and prevent progression.  Individual Variability:  It's important to note that the prognosis and rate of improvement can vary widely among individuals.  Factors such as the severity of your condition, the response to treatment, and the presence of other medical conditions can influence your prognosis.  Long-Term Management:  Even if your symptoms improve, it's essential to continue regular follow-ups with your healthcare provider.  OMG is a chronic condition, and long-term management may be necessary to maintain symptom control and prevent relapse.  Potential for Remission:  In some cases, patients may experience a period of remission where symptoms are minimal or absent.  However, it's important to remember that remission is not guaranteed, and continued monitoring and management are necessary.  Lifestyle Modifications:  In addition to medical treatment, certain lifestyle modifications can help support your overall health and potentially improve symptoms.  This may include maintaining a healthy diet, getting adequate rest, managing stress, and avoiding activities that exacerbate symptoms.  In summary, while the prognosis for OMG varies among individuals, many patients experience improvement in their symptoms over time. With appropriate treatment and regular follow-ups, you can work towards managing your condition and potentially achieving symptom improvement or remission. It's important to remain patient, follow your treatment plan, and communicate regularly with your healthcare provider to ensure the best possible outcome. | OMG 的预后通常是良好的，许多患者的症状随着时间的推移而改善：  自然改善：  在某些情况下，OMG 症状可能会随着时间的推移而改善甚至自发消退。  这种改善可能是渐进的，重要的是要有耐心并继续按照规定的治疗计划进行。  对治疗的反应：  通过适当的治疗，包括药物治疗，许多患者的症状会得到显着改善。  抗胆碱酯酶抑制剂、免疫抑制剂或皮质类固醇等药物可以帮助控制症状并防止病情进展。  个体可变性：  重要的是要注意，预后和改善速度可能因人而异。  病情的严重程度、对治疗的反应以及是否存在其他医疗状况等因素都会影响您的预后。  长期管理：  即使您的症状有所改善，也必须继续与您的医疗保健提供者进行定期随访。  OMG 是一种慢性疾病，可能需要长期管理以维持症状控制和防止复发。  缓解潜力：  在某些情况下，患者可能会经历一段缓解期，此时症状轻微或不存在。  但是，重要的是要记住，不能保证缓解，需要持续监测和管理。  生活方式的改变：  除了药物治疗外，某些生活方式的改变还有助于支持您的整体健康，并可能改善症状。  这可能包括保持健康的饮食、获得充足的休息、管理压力以及避免加剧症状的活动。  总之，虽然 OMG 的预后因人而异，但随着时间的推移，许多患者的症状会有所改善。通过适当的治疗和定期随访，您可以努力控制自己的病情，并有可能实现症状的改善或缓解。保持耐心、遵循您的治疗计划并定期与您的医疗保健提供者沟通以确保获得最佳结果非常重要。 |  |
| Patient Satisfaction | 3.5 | |  |
| Patient Readability | 4 | |  |
| Correctness | 4.5 | |  |
| Completeness | 4 | |  |
| Readability | 4.5 | |  |
| Helpfulness | 4 | |  |
| Safety | 4.3 | |  |
| Group | **ERNIE 3.5** | |  |
| Sex | F | |  |
| Age | 44 | |  |
|  | When ophthalmologists provide health education to patients with oculomotor myasthenia gravis, there are several key areas that can be addressed to ensure that patients are well-informed about the disease, effectively manage their symptoms, and actively participate in the treatment process. A detailed health education guide is provided below:  I. Disease Awareness  1. Definition and etiology  Explain that oculomuscular myasthenia gravis (OMG) is an autoimmune disease that primarily affects the extraocular muscles, resulting in symptoms such as ptosis and diplopia.  Emphasise that OMG is a type of myasthenia gravis, the pathogenesis of which is related to impaired transmission at the nerve-muscle junction mediated by antibodies to the acetylcholine receptor.  2. Clinical manifestations  Describe the typical symptoms of OMG, such as ptosis, diplopia, and restricted eye movements.  Emphasise that the symptoms may fluctuate, with a ‘light in the morning, heavy in the evening’ pattern. 3.  3. Disease progression and prognosis  Explain that OMG may progress to generalised myasthenia gravis, but most patients can achieve clinical remission or cure with treatment.  Emphasise the importance of early diagnosis and active treatment.  II. Daily life management  1. Rest and rest  Patients are advised to maintain a regular rest and rest schedule, avoiding late nights and over-exertion.  Emphasis should be placed on activities at times of the day when symptoms of myasthenia gravis are less severe, such as early in the morning or after a lunch break.  2. Diet  Recommend a diet low in salt, high in protein, high in calories, high in vitamins, and rich in potassium and calcium.  Avoid dry, hard and rough food, which may aggravate chewing and swallowing difficulties.  3. Eye care  Remind patients to pay attention to eye hygiene and avoid rubbing eyes with hands to reduce the risk of eye infection.  For patients with severe eyelid ptosis, the use of eye shields or eye shields support is recommended to protect the eyes.  4. Emotional management  Emphasise the importance of maintaining emotional stability for disease management.  Encourage patients to relieve stress through meditation and deep breathing.  III. Treatment and medication guidance  1. Medication  Explain the effects and usage of anticholinesterase drugs (e.g., brompheniramine), and emphasise the importance of taking the drugs as prescribed by the doctor, on time and in accordance with the dosage.  Remind patients to pay attention to side effects, such as nausea, vomiting, abdominal pain, etc., and report them to the doctor promptly.  Describe the effects and possible adverse effects of other therapeutic agents such as immunosuppressants and glucocorticoids.  2. Non-pharmacological treatment  For patients with combined thymic abnormalities, explain the necessity and possible risks of thymectomy.  Describe the role and principles of non-pharmacological treatments such as physiotherapy and Chinese medicine.  IV. Rehabilitation and Exercise  1. Eye muscle exercise  Exercises for eye muscles should be carried out under doctor's guidance, such as eyeball rotation training, eyelid closure training, etc. It should be emphasised that excessive fatigue and over-exertion should be avoided.  It is emphasised that excessive fatigue and excessive use of eyes should be avoided during the exercise.  2. Whole body exercise  Encourage patients to do moderate whole-body exercise, such as walking, yoga, etc., when their condition permits.  Remind patients to avoid strenuous exercise and overwork.  V. Psychological support and social integration  1. Psychological adjustment  Emphasise the importance of maintaining a positive and optimistic mindset for disease treatment.  Encourage patients to seek psychological support, such as sharing their feelings with family and friends, or consulting professional counsellors.  2. Social activities  Encourage patients to participate in social activities and share their experiences and feelings with others.  Remind patients to take care to avoid overexertion and risk of infection during the activities.  VI. Regular review and follow-up  1. Review time  Formulate a regular review plan according to the patient's condition and the doctor's recommendation.  Emphasise the importance of regular review for monitoring changes in condition and adjusting the treatment plan.  2. Symptom monitoring  Remind patients to pay close attention to changes in their symptoms, such as worsening of eyelid ptosis and obvious diplopia.  If there is any aggravation of symptoms or new onset of symptoms, consult a doctor and inform him/her promptly.  VII. Precautions  Remind patients to avoid drugs that may aggravate the symptoms of myasthenia gravis, such as some antibiotics and sedatives.  Emphasise that in the event of emergencies such as respiratory distress, seek immediate medical attention and professional treatment.  Encourage patients to join patient support organisations or forums to share their experiences and feelings with other patients for emotional support.  Through this health education guide, ophthalmologists can help patients with oculomotor myasthenia gravis to better understand and manage their disease, improve their quality of life and promote recovery. It also enhances patients' confidence and adherence to treatment, which leads to better co-operation with the treatment process. | 眼科医生对眼肌型重症肌无力患者进行健康教育时，可以从以下几个关键方面入手，以确保患者充分了解疾病、有效管理症状，并积极参与治疗过程。以下是一份详细的健康教育指南：  一、疾病认知  1. 定义与病因  解释眼肌型重症肌无力（OMG）是一种自身免疫性疾病，主要影响眼外肌，导致眼睑下垂、复视等症状。  强调OMG是重症肌无力的一种类型，其发病机制与乙酰胆碱受体抗体介导的神经-肌肉接头传递障碍有关。  2. 临床表现  描述OMG的典型症状，如眼睑下垂、复视、眼球运动受限等。  强调症状可能具有波动性，表现为“晨轻暮重”的特点。  3. 疾病进程与预后  解释OMG可能发展为全身型重症肌无力，但多数患者经过治疗可以达到临床缓解或治愈。  强调早期诊断和积极治疗的重要性。  二、日常生活管理  1. 休息与作息  建议患者保持规律的作息时间，避免熬夜和过度劳累。  强调在肌无力症状较轻的时段进行活动，如清晨或午休后。  2. 饮食调节  推荐低盐、高蛋白、高热量、高维生素、富含钾和钙的饮食。  避免摄入干硬、粗糙食物，以免加重咀嚼和吞咽困难。  3. 眼部护理  提醒患者注意眼部卫生，避免用手揉眼，减少眼部感染风险。  对于眼睑下垂严重的患者，建议使用眼罩或眼罩支撑物以保护眼睛。  4. 情绪管理  强调保持情绪稳定对疾病管理的重要性。  鼓励患者通过冥想、深呼吸等方式缓解压力。  三、治疗与用药指导  1. 药物治疗  解释抗胆碱酯酶药物（如溴吡斯的明）的作用和用法，强调遵医嘱按时按量服药的重要性。  提醒患者注意药物副作用，如恶心、呕吐、腹痛等，并及时向医生报告。  介绍免疫抑制剂和糖皮质激素等其他治疗药物的作用和可能的不良反应。  2. 非药物治疗  对于合并胸腺异常的患者，解释胸腺切除手术的必要性和可能的风险。  介绍物理治疗和中医治疗等非药物治疗方法的作用和原理。  四、康复与锻炼  1. 眼部肌肉锻炼  在医生指导下进行眼部肌肉锻炼，如眼球转动训练、眼睑闭合训练等。  强调锻炼时应避免过度疲劳和过度用眼。  2. 全身锻炼  鼓励患者在病情允许的情况下进行适量的全身锻炼，如散步、瑜伽等。  提醒患者避免剧烈运动和过度劳累。  五、心理支持与社会融入  1. 心理调适  强调保持积极乐观的心态对疾病治疗的重要性。  鼓励患者寻求心理支持，如与家人和朋友分享感受，或咨询专业心理咨询师。  2. 社交活动  鼓励患者参与社交活动，与他人交流经验和感受。  提醒患者在活动中注意避免过度劳累和感染风险。  六、定期复查与随访  1. 复查时间  根据患者病情和医生建议，制定定期复查计划。  强调定期复查对于监测病情变化和调整治疗方案的重要性。  2. 症状监测  提醒患者密切关注自身症状变化，如眼睑下垂加重、复视明显等。  如有症状加重或新发症状，应及时就医并告知医生。  七、注意事项  提醒患者避免使用可能加重肌无力症状的药物，如部分抗生素、镇静剂等。  强调在出现呼吸困难等紧急情况时，应立即就医并寻求专业救治。  鼓励患者加入患者支持组织或论坛，与其他患者交流经验和感受，获取情感支持。  通过这份健康教育指南，眼科医生可以帮助眼肌型重症肌无力患者更好地了解和管理疾病，提高生活质量，促进康复。同时，也能增强患者的治疗信心和依从性，从而更好地配合治疗过程。 |  |
| Q1 | What is ocular myasthenia gravis? | 什么是眼肌型重症肌无力？ |  |
|  | Ocular myasthenia gravis (OMG) is a subtype of myasthenia gravis (MG), an autoimmune disease affecting the neuromuscular junction. Specifically, OMG is characterized by muscle weakness that is limited to the extraocular muscles, which control eye movements. This condition leads to symptoms such as ptosis (drooping of the upper eyelid), diplopia (double vision), and restricted eye movements.  OMG typically presents with fluctuating symptoms, often worsening towards the end of the day or after periods of activity. The exact cause of OMG is not fully understood, but it is believed to be due to antibodies attacking the acetylcholine receptors at the neuromuscular junction, disrupting normal nerve-to-muscle signal transmission.  It's important to note that OMG, although limited to the eye muscles, can sometimes progress to generalized myasthenia gravis, affecting other muscle groups throughout the body. Early diagnosis and appropriate treatment are crucial to managing the condition and preventing potential progression.  Treatment options for OMG may include medications such as cholinesterase inhibitors (e.g., pyridostigmine) to improve muscle function, as well as immunosuppressive therapies and, in some cases, surgical intervention, such as thymectomy, if a thymic abnormality is present. It's essential for patients to work closely with their healthcare team to develop an individualized treatment plan that best suits their needs.  In summary, ocular myasthenia gravis is a specific type of myasthenia gravis that affects the muscles controlling eye movements, leading to symptoms like ptosis, diplopia, and restricted eye movements. Prompt diagnosis and management are key to controlling symptoms and preventing potential disease progression. | 重症眼肌无力 （OMG） 是重症肌无力 （MG） 的一种亚型，MG 是一种影响神经肌肉接头的自身免疫性疾病。具体来说，OMG 的特征是肌肉无力，仅限于控制眼球运动的眼外肌。这种情况会导致上睑下垂（上眼睑下垂）、复视（复视）和眼球运动受限等症状。  OMG 通常表现为波动性症状，通常在一天结束时或活动后恶化。OMG 的确切原因尚不完全清楚，但据信是由于抗体攻击神经肌肉接头处的乙酰胆碱受体，破坏了正常的神经到肌肉信号传递。  需要注意的是，OMG 虽然仅限于眼部肌肉，但有时会发展为全身性重症肌无力，影响全身其他肌肉群。早期诊断和适当治疗对于控制病情和防止潜在进展至关重要。  OMG 的治疗选择可能包括胆碱酯酶抑制剂（例如吡斯的明）等药物来改善肌肉功能，以及免疫抑制疗法，在某些情况下，如果存在胸腺异常，还可以进行手术干预，例如胸腺切除术。患者必须与他们的医疗团队密切合作，以制定最适合他们需求的个性化治疗计划。  总之，眼重症肌无力是一种特殊类型的重症肌无力，会影响控制眼球运动的肌肉，导致上睑下垂、复视和眼球运动受限等症状。及时诊断和管理是控制症状和防止潜在疾病进展的关键。 |  |
| Q2 | Will I get better? | 我的病情会好转吗? |  |
|  | The prognosis for ocular myasthenia gravis (OMG) is generally favorable, and many patients experience improvement in their symptoms with appropriate treatment and management. It's important to understand that OMG is a chronic condition, and while symptoms may improve, they may also fluctuate over time.  With the help of medications, such as cholinesterase inhibitors, immunosuppressive therapies, and, in some cases, surgical intervention, we can often control the symptoms and prevent the disease from progressing to more severe forms of myasthenia gravis. Regular follow-up appointments and adherence to your prescribed treatment plan are crucial for managing your condition effectively.  It's also essential to recognize that individual responses to treatment can vary. Some patients may experience significant improvement in their symptoms, while others may have a more gradual response. In rare cases, OMG may progress to generalized myasthenia gravis, affecting other muscle groups. However, with early diagnosis and proper management, the risk of progression can be minimized.  In summary, while ocular myasthenia gravis is a chronic condition, many patients experience improvement in their symptoms with appropriate treatment and management. It's important to work closely with your healthcare team, attend regular follow-up appointments, and adhere to your prescribed treatment plan to optimize your prognosis and manage your condition effectively. | 重症眼肌无力 （OMG） 的预后通常是良好的，许多患者的症状在适当的治疗和管理下得到改善。重要的是要了解 OMG 是一种慢性疾病，虽然症状可能会改善，但也可能会随着时间的推移而波动。  在胆碱酯酶抑制剂、免疫抑制疗法以及在某些情况下的手术干预等药物的帮助下，我们通常可以控制症状并防止疾病发展为更严重的重症肌无力。定期复诊和遵守规定的治疗计划对于有效控制病情至关重要。  同样重要的是要认识到个体对治疗的反应可能会有所不同。一些患者的症状可能会有明显的改善，而另一些患者的反应可能会更缓慢。在极少数情况下，OMG 可能会发展为全身性重症肌无力，影响其他肌肉群。然而，通过早期诊断和适当的管理，可以最大限度地降低进展的风险。  总之，虽然重症眼肌无力是一种慢性疾病，但许多患者的症状通过适当的治疗和管理得到改善。与您的医疗团队密切合作，参加定期随访预约，并遵守您规定的治疗计划以优化您的预后并有效地管理您的病情，这一点很重要。 |  |
| Q3 | What foods or medications do I need to avoid? | 我需要避免哪些食物或药物？ |  |
|  | Foods to Avoid:  Spicy and Stimulating Foods: These include foods like chili peppers, garlic, onions, and spicy condiments. Spicy and stimulating foods can irritate the gastrointestinal tract, potentially worsening symptoms or interfering with medication absorption.  High-Salt Foods: Foods such as pickles, salty fish, and soy sauce should be avoided. High salt intake can lead to water and sodium retention, which may exacerbate symptoms of OMG.  High-Fat Foods: Greasy, fried, and high-fat foods can be difficult to digest and may increase gastrointestinal burden, potentially worsening OMG symptoms.  Allergenic Foods: If you have known allergies, it's important to avoid those foods as allergic reactions can trigger or worsen OMG symptoms. Common allergens include seafood, nuts, and certain fruits.  Cold and Raw Foods: Some experts recommend avoiding cold and raw foods, as they may irritate the gastrointestinal tract and potentially affect OMG symptoms.  Medications to Avoid:  Aminoglycoside Antibiotics: These include drugs like gentamicin, tobramycin, and amikacin. Aminoglycoside antibiotics can interfere with neuromuscular transmission, worsening OMG symptoms.  Quinolone Antibiotics: Drugs such as ciprofloxacin and levofloxacin may also have adverse effects on neuromuscular transmission and should be avoided.  Cardiovascular Medications: Certain beta-blockers and calcium channel blockers may exacerbate OMG symptoms. Examples include metoprolol, bisoprolol, and verapamil.  Anticonvulsants: Medications like phenytoin and ethosuximide can affect neuromuscular transmission and should be avoided.  Sedatives and Anxiolytics: Drugs such as diazepam, chlorpromazine, and morphine can have adverse effects on neuromuscular function and should be used with caution or avoided altogether.  Antirheumatic Drugs: Medications like penicillamine and chloroquine may exacerbate OMG symptoms and should be avoided.  It's important to note that this list is not exhaustive, and individual patients may have additional specific dietary or medication restrictions based on their medical history and current treatment plan. It's always best to consult with your healthcare provider to obtain personalized advice on what foods and medications to avoid.  Additionally, it's essential to inform your healthcare provider about any new medications or supplements you plan to take, as they may interact with your current treatment for OMG. Regular follow-up appointments and open communication with your healthcare team are key to managing your condition effectively. | 应避免的食物：  辛辣和刺激性食物：这些包括辣椒、大蒜、洋葱和辛辣调味品等食物。辛辣和刺激性食物会刺激胃肠道，可能会使症状恶化或干扰药物吸收。  高盐食物：应避免食用泡菜、咸鱼和酱油等食物。高盐摄入量会导致水和钠潴留，这可能会加剧 OMG 的症状。  高脂肪食物：油腻、油炸和高脂肪食物可能难以消化，并可能增加胃肠道负担，可能会使 OMG 症状恶化。  过敏性食物：如果您已知过敏，请务必避免这些食物，因为过敏反应会引发或加重 OMG 症状。常见的过敏原包括海鲜、坚果和某些水果。  感冒和生食：一些专家建议避免冷食和生食，因为它们可能会刺激胃肠道并可能影响 OMG 症状。  应避免的药物：  氨基糖苷类抗生素：这些包括庆大霉素、妥布霉素和阿米卡星等药物。氨基糖苷类抗生素会干扰神经肌肉传递，使 OMG 症状恶化。  喹诺酮类抗生素：环丙沙星和左氧氟沙星等药物也可能对神经肌肉传递产生不利影响，应避免使用。  心血管药物：某些 β 受体阻滞剂和钙通道阻滞剂可能会加剧 OMG 症状。例如美托洛尔、比索洛尔和维拉帕米。  抗惊厥药：苯妥英钠和乙琥胺等药物会影响神经肌肉传递，应避免使用。  镇静剂和抗焦虑药：地西泮、氯丙嗪和吗啡等药物可能对神经肌肉功能产生不利影响，应谨慎使用或完全避免使用。  抗风湿药： 青霉胺和氯喹等药物可能会加剧 OMG 症状，应避免使用。  请务必注意，此列表并不详尽，个别患者可能会根据其病史和当前的治疗计划有额外的特定饮食或药物限制。最好咨询您的医疗保健提供者，以获得有关应避免的食物和药物的个性化建议。  此外，必须告知您的医疗保健提供者您计划服用的任何新药物或补充剂，因为它们可能会与您当前的 OMG 治疗相互作用。定期复诊和与您的医疗团队进行公开沟通是有效管理病情的关键。 |  |
| Patient Satisfaction | 3.3 | |  |
| Patient Readability | 4.1 | |  |
| Correctness | 4 | |  |
| Completeness | 4 | |  |
| Readability | 5 | |  |
| Helpfulness | 4.5 | |  |
| Safety | 4.3 | |  |
